# Supplementary material for: Health of mothers of children with a life-limiting condition: a protocol for comparative cohort study using the Clinical Practice Research Datalink
Source: BMJ Open. 2020 Jul 13;10(7):e034024. doi: 10.1136/bmjopen-2019-034024 (PMC7359378; doi:10.1136/bmjopen-2019-034024)
Supplement: Author's manuscript [file bmjopen-2019-034024.draft_revisions.pdf]

BMJ Open is committed to open peer review. As part of this commitment we make the peer review history of every article we publish publicly available.

When an article is published we post the peer reviewers' comments and the authors' responses online. We also post the versions of the paper that were used during peer review. These are the versions that the peer review comments apply to.

The versions of the paper that follow are the versions that were submitted during the peer review process. They are not the versions of record or the final published versions. They should not be cited or distributed as the published version of this manuscript.

BMJ Open is an open access journal and the full, final, typeset and author-corrected version of record of the manuscript is available on our site with no access controls, subscription charges or pay-per-view fees (http://bmjopen.bmj.com).

If you have any questions on BMJ Open's open peer review process please email info.bmjopen@bmj.com

BMJ Open

The health of mothers of children with a life-limiting condition; a protocol for comparative cohort study using the CPRD database

Journal:	*BMJ Open*
Manuscript ID	bmjopen-2019-034024
Article Type:	Protocol
Date Submitted by the Author:	02-Sep-2019
Complete List of Authors:	Fraser, Lorna; University of York,<br>Murtagh, Fliss; Wolfson Palliative Care Research Centre, Hull York Medical School, University of Hull, UK,<br>Sheldon, Trevor; University of York, health Sciences<br>Gilbody, Simon; The University of York, Department of Health Sciences<br>Hewitt, Catherine; The University of York, Health Sciences
Keywords:	Developmental neurology & neurodisability < PAEDIATRICS, Life-limiting, maternal health

SCHOLARONE™
Manuscripts

I, the Submitting Author has the right to grant and does grant on behalf of all authors of the Work (as defined in the below author licence), an exclusive licence and/or a non-exclusive licence for contributions from authors who are: i) UK Crown employees; ii) where BMJ has agreed a CC-BY licence shall apply, and/or iii) in accordance with the terms applicable for US Federal Government officers or employees acting as part of their official duties; on a worldwide, perpetual, irrevocable, royalty-free basis to BMJ Publishing Group Ltd ("BMJ") its licensees and where the relevant Journal is co-owned by BMJ to the co-owners of the Journal, to publish the Work in this journal and any other BMJ products and to exploit all rights, as set out in our licence.

The Submitting Author accepts and understands that any supply made under these terms is made by BMJ to the Submitting Author unless you are acting as an employee on behalf of your employer or a postgraduate student of an affiliated institution which is paying any applicable article publishing charge ("APC") for Open Access articles. Where the Submitting Author wishes to make the Work available on an Open Access basis (and intends to pay the relevant APC), the terms of reuse of such Open Access shall be governed by a Creative Commons licence – details of these licences and which Creative Commons licence will apply to this Work are set out in our licence referred to above.

Other than as permitted in any relevant BMJ Author's Self Archiving Policies, I confirm this Work has not been accepted for publication elsewhere, is not being considered for publication elsewhere and does not duplicate material already published. I confirm all authors consent to publication of this Work and authorise the granting of this licence.

For peer review only

TITLE: The health of mothers of children with a life-limiting condition; a protocol for comparative cohort study using the CPRD database

Fraser LK[1,2], Murtagh FEM[3], Sheldon TA[1], Gilbody S[1], Hewitt CE[1]

[1] Department of Health Sciences, University of York, UK

[2]Martin House Research Centre, University of York, UK

[3] Wolfson Palliative Care Centre, Hull York Medical School, Hull, UK

Corresponding Author:

Dr Lorna Fraser

Department of Health Sciences

Area 2 Seebohm Rowntree Building

Heslington

YORK

YO10 5DD

lorna.fraser@york.ac.uk

01904 321889

Word Count: 2489 words.

Tables :1

For peer review only

For peer review only - http://bmjopen.bmj.com/site/about/guidelines.xhtml

ABSTRACT

Introduction

There are now nearly 50,000 children with a life-limiting or life-threatening conditions in the UK. These include conditions where there is no reasonable hope of cure and from which they will die, as well as conditions for which curative treatment may be feasible but can fail, e.g. cancer or heart failure. Having a child with a life-limiting condition involves being a coordinator and provider of healthcare in addition to the responsibilities and pressures of parenting a child who is expected to die young. This adversely affects the health and wellbeing of these mothers and affects their ability to care for their child, but the extent of the impact is poorly understood.

This study aims to quantify the incidence and nature of mental and physical morbidity in mothers of children with a life-limiting condition, their healthcare use and to assess whether there is a relationship between the health of the mother and the child's condition.

Methods and analysis

A comparative cohort study using data from the Clinical Practice Research Datalink and linked hospital data will include three groups of children and their mothers (those with a life-limiting condition, those with a chronic conditions and those with no long term health condition total =20,000 mother-child dyads). Incidence rates and incidence rate ratios will be used to quantify and compare the outcomes between groups with multivariable regression modelling used to assess the relationship between the child's disease trajectory and mother's health.

Ethics and dissemination

This study protocol has approval from the Independent Scientific Advisory Committee for the UK Medicines and Healthcare products Regulatory Agency Database Research. The results of this study will be reported according to the STROBE and RECORD guidelines. There will also be a lay summary for parents which will be available to download from the Martin House Research Centre website (www.york.ac.uk/mhrc).

For peer review only

ARTICLE SUMMARY

Strengths and Limitations of this study

This study is utilising a large, longitudinal, nationally representative primary care data source.

The Clinical Practice Research Datalink data enables the identification of mother and child dyads and linkage to secondary care data.

This study is reliant on identification of the cohort of interest by diagnostic coding therefore on the quality of diagnostic coding within the CPRD and HES datasets.

INTRODUCTION

The number of children with life-limiting or life-threatening conditions has been rising, with latest figures estimating 49,000 children and young people with a life-limiting condition in the UK.[1] These include conditions for which there is no reasonable hope of cure and from which children or young people will die, as well as conditions for which curative treatment may be feasible but can fail, such as cancer or heart failure. In children and young people, more than 300 diagnoses are life-limiting or life-threatening[2] , including duchenne muscular dystrophy, severe cerebral palsy, neurodegenerative conditions, and severe congenital anomalies.

Although many of the individual diagnoses are rare, collectively life-limiting conditions affect more children and young people than more common single long term conditions, such as diabetes mellitus[3] . Many of these children are living longer due to the use of medical technologies, e.g. ventilation and gastrostomy feeding, and more aggressive treatment of complications and they are often high users of healthcare services[4] . The parents of these children have an added layer of 'pressure' from having a child with a chronic condition who also has a shortened life expectancy[5] . Having a child with a life-limiting condition often involves being a coordinator and provider of healthcare as well as being a parent, and this responsibility exists 24 hours a day, 7 days a week, especially for mothers. The health of these mothers is important, both for their own well-being, and also in terms of their ability to care

For peer review only - http://bmjopen.bmj.com/site/about/guidelines.xhtml

for their child. Currently the NHS and statutory services for these children rarely extend to providing care for the parents. The focus is on mothers rather than fathers in this study as mothers are the main carer for the majority of these children.

The need for further research on the emotional and psychological support and interventions for parents or carers of children with a life-limiting condition was highlighted recently in NICE guidelines on end of life care in children and young people[6] . This stated that research was needed on 'What emotional support do children and young people with life-limiting conditions and their parents or carers need, and how would they like these needs to be addressed? The NICE guidelines[6] also noted that no studies had quantified the psychological/ mental health of mothers of children with life-limiting condition. There have been some attempts to quantify these in children with special needs[7] or specific disabilities[8 9] which have shown higher levels of parental distress or emotional problems than parents of healthy children (36% cf 20%). These however, do not address the specific needs of those with life-limiting conditions or the added burden that parents of children with a life-limiting condition face, knowing their child is likely to die.

Evidence about the physical health of mothers with life-limiting condition is also sparse, although two studies in mothers of children with disabilities found higher prevalence of physical conditions compared to mothers of healthy children. For example, prevalence of back pain and hypertension was 35.2% and 24.7% respectively in mothers of children with disabilities, compared to 26.7% and 19.1% in mothers of healthy children. However, these studies were cross sectional, self-reported and were not explicitly for mothers of children with a life-limiting condition [9 10].. To date, no study using a nationally representative sample has assessed the incidence or prevalence of mental and physical health conditions in mothers of children with a life-limiting condition. Quantifying the extent and nature of both physical and mental health conditions in these mothers is important when trying to develop and target appropriate preventative or treatment interventions for this population.

This study aims to quantify the incidence and nature of mental and physical morbidity in mothers of children with a life-limiting condition, their healthcare use and to assess whether there is a relationship between the health of the mother and the child's condition.

For peer review only - http://bmjopen.bmj.com/site/about/guidelines.xhtml

METHODS AND ANALYSIS

Research Questions

1. What is the nature and incidence of mental and physical morbidity in mothers of children with a life-limiting condition?

2. What is the relationship between the health of the mother and the child's condition?

3. How many primary care visits do these mothers have compared to other mothers?

4. How many hospital admissions do these mothers have compared to other mothers?

5. What are the resource costs of healthcare for these mothers?

Data sources and sample selection

This will be an observational comparative cohort study design using data from the Clinical Practice Research Datalink (CPRD). The CPRD dataset contains anonymized, longitudinal records of primary care from a representative sample of GP practices across the UK (covering approximately 8.5% of the UK population)[11]. Individuals within the primary care CPRD dataset are also linked to secondary care data (inpatient, outpatient, A & E Hospital Episodes Statistics (HES) and the Mental Health Minimum Dataset)[12 13] and Office for National Statistics death certificate data. The Clinical Practice Research Datalink provides a unique opportunity to assess the relationship between maternal morbidity and their child health due to the longitudinal nature of data collection (since 1987) and the ability to link mothers and children via their mother-baby link algorithm[14].

The index children will be extracted from the CPRD mother baby linkage, where the mother has at least 1 years registration and where they eligible for HES linkage (resident in England). Children will be grouped in to those with a life-limiting condition (n~5000) or chronic condition that is not life-limiting e.g. diabetes, asthma (n~5000) and they will be identified within the CPRD dataset between 2007-2017, using READ codes in the CPRD data and ICD10 codes in the HES data[1] (see Table 1) . The development of these ICD-10 code list have been

For peer review only - http://bmjopen.bmj.com/site/about/guidelines.xhtml

described previously for life-limiting[1] and chronic conditions[15]. Subjects will be matched by year of birth, sex and region to the control group of healthy children (n~10,000) with no long term conditions.

All primary and the linked secondary care data will be extracted for each mother and child dyad.

Table 1 Cohort identification processes

Groups	Inclusion criteria	Exclusion Criteria
1. Life-limiting or life-threatening condition	• Children from the source population who have prevalent diagnosis event records for life-limiting conditions, either in the Clinical or Referral files in CPRD GOLD based on the Read codes detailed in Supplementary material, or in HES APC based on the ICD-10 codes in Supplementary material.<br>• Children have above events before the end of the study period (31/12/2017).<br>• Children have above events within their UTS follow-up period.<br>• Children are aged 18 years or less on the diagnosis event date.	None
2. Chronic condition that is not life-limiting	One matching control will be provided for each case in Group 1. The controls will comprise of patients from the source population who fulfil the following criteria:<br>• Children from the source population who have diagnosis event records for other chronic conditions in the Clinical or Referral files in CPRD GOLD based	•Children with a diagnosis on or before 31/12/2017 of life-limiting conditions, either in the Clinical or Referral files in CPRD GOLD based on the Read codes detailed in Appendix 1, or in HES APC based on the ICD-10 codes in Appendix 2.

For peer review only - http://bmjopen.bmj.com/site/about/guidelines.xhtml

	on the Read codes detailed in Supplementary material, or in HES APC based on the ICD-10 codes in Supplementary material.<br>• Children have above events before the end of the study period of (31/12/2017).<br>• Children have above events within their UTS follow-up period.<br>• Children are aged 18 years or less on the diagnosis date.	• Children who have deregistered from the CPRD on or before 01/04/2007<br><br>• Children who are siblings of children in the case population (Group 1), based on having a link to the same mother in the Mother Baby Link
3. No long term conditions	Up to two matching controls will be provided for each case in Group 1. The controls will comprise of patients from the source population who fulfil the following criteria:<br><br>• Children from the source population who have at least one day of registration during follow-up start and end.	• Children with a diagnosis of life-limiting conditions on or before 31/12/2017, either in the Clinical or Referral files in CPRD GOLD based on the Read codes detailed in Appendix 1, or in HES APC based on the ICD-10 codes in Appendix 2, will be excluded<br>• Children with a diagnosis of other chronic conditions on or before 31/12/2017, in the Clinical or Referral files in CPRD GOLD based on the Read codes detailed in Appendix 3, or in HES APC based on the ICD-10 codes in Appendix 4, will be excluded.<br>• Children who have deregistered from the CPRD on or before 01/04/2007<br>• Children who are siblings of children in the case population (Group 1), based on

For peer review only

		having a link to the same mother in the Mother Baby Link

Sample size

A sample size calculation based on 80% power, 5% significance to detect a 5% difference in prevalence in maternal anxiety/depression between the groups (from 15-20% prevalence) indicates a minimum of 1,131 participants required in each group. Given the heterogeneity in the life-limiting condition group, and to facilitate analyses within life-limiting condition subgroups, we will aim to have 5,000 participants in each of the life-limiting condition and chronic disease groups, with 10,000 in the control (no long-term condition) group. The total sample size will be 40,000 (20,000 children and 20,000 mothers).

Exposures

The key exposure is the disease status of the child (life-limiting condition/chronic disease (asthma or diabetes)/ no long-term condition). These groups will be identified using previously developed READ and ICD 10 diagnostic code lists (see supplementary material). The use of both READ and ICD 10 codes will enable identification of relevant diagnoses from either the primary care records (READ) or hospital admission data (ICD 10).

Outcomes

The outcomes for the mothers will be recorded in either the primary care data or the linked secondary care datasets. The dates of these outcomes will be assessed in relation to the date of the child's diagnoses.

Incidence of mental health and physical health conditions

- Maternal mental health diagnoses, including both common mental illness and severe mental illness. These will be identified using diagnostic READ codes and relevant

For peer review only - http://bmjopen.bmj.com/site/about/guidelines.xhtml

prescription data. A previously developed READ code algorithm[16] will be utilised. The linked MHMDS will indicate the presence of a more serious mental health conditions.

- Maternal physical diagnoses, including e.g. obesity, hypertension, musculoskeletal problems, and cardiovascular disease. These will be identified using diagnostic READ codes, relevant prescription data, HES data, and related biometric data (including blood pressure, BMI, and total cholesterol).

The association of maternal health with the child's disease trajectory

- The number of A&E attendances, hospital admissions and length of stay for the children (HES data).

Healthcare use and resources of the mother

- Referrals to other services, especially secondary services for mothers (CPRD and HES data).
- Uptake of cervical screening by mothers (CPRD data).
- The number of primary care attendances (GP, practice nurse etc.) per year by mothers (CPRD data)
- The number and nature of prescribed medication for mothers (Prescription data).
- The number of A&E attendances, hospital admissions and length of stay for mothers (HES data).

Confounding

The following clinical and demographic confounders will be considered in the analyses:

- Ethnicity, this will be classified according to the census 2011 categories[17] using data from CPRD and HES data.
- Pre-existing comorbidities of the mother
- Child's diagnoses: grouped into disease categories[1]
- Socioeconomic data is based on the index of multiple deprivation category based on the local superoutput area of residence[18] .

For peer review only

Statistical methods

All analyses will be undertaken using STATA version 15[19]. All analyses will adjust for known clinical and demographic confounders of importance (age, co-morbidities, socioeconomic status, previous use of primary care services, etc.). The left and right censored nature of these data will be taken into account when undertaking these analyses.

Incidence of mental health and physical health conditions

Incidence rates of the physical and mental health conditions will be calculated in each group of mothers by dividing the number of cases in each group by the person-time at risk in each group. A comparison with the incidence of these conditions in the three groups of mothers will be undertaken using incidence rate ratios.

The association of maternal health with the child's disease trajectory

The presence of physical or mental health diagnoses in the mother will be the outcome variable of interest in a multivariable model with a key covariate being the presence of prolonged or repeated hospital admission for the child. A prolonged admission is one that lasts > 14 days and repeated admissions will be defined as more than 2 admissions in a 6 month time period.

Healthcare use and resources of the mother

Using multivariable Poisson regression modelling for the count of GP visits, referrals and hospital admissions with the key exposure of disease status of the child (life-limiting condition /Chronic disease/ no long-term condition). Multilevel modelling will be used to account for repeated measures within individuals. For the analyses of healthcare costs, we will use information from Unit Costs of Health and Social Care[20] , the NHS national tariffs[21] and Department of Health Reference Costs[22] .

Missing data

For peer review only - http://bmjopen.bmj.com/site/about/guidelines.xhtml

We expect there to be missing data for some of the key covariates in the data e.g. ethnicity. We will assess the level of missingness across all sources of these variable (HES and CPRD). If appropriate then we will use multiple imputation techniques [23] to address this in order to avoid the potential bias from only undertaking complete case analyses.

PATIENT AND PUBLIC INVOLVEMENT

The views of parents and carers of children with a life-limiting condition have informed the development of this study. Initial ideas about the topic area and planned study were discussed with a group of 10 parents of children with a life-limiting condition at Martin House Children's Hospice. They helped to refine the research question and future stages of this programme of work. The Family Advisory Board of the Martin House Research Centre (www.york.ac.uk/mhrc) will have involvement in this study with an emphasis on the preparation of the lay summaries and dissemination to parents and children.

ETHICS AND DISSEMINATION

This study protocol has approval from the Independent Scientific Advisory Committee for the UK Medicines and Healthcare products Regulatory Agency Database Research (protocol 18_313). They have NREC approval for release of pseudonymised data for observational research.

The results of this study will be reported according to the STROBE and RECORD guidelines (ref). There were also be a lay summary for parents which will be available to download from the Martin House Research Centre website (www.york.ac.uk/mhrc).

Authors' contributions: LF had the original idea for this study and wrote the first draft of this manuscript. FM, SG, TS and CH contributed to the development of this idea, study design and revised the manuscript. All authors approved the final submitted version of this manuscript.

Funding statement: This paper is independent research arising from a Career Development Fellowship held by Lorna Fraser (CDF-2018-11-ST2-002) supported by the National Institute

For peer review only - http://bmjopen.bmj.com/site/about/guidelines.xhtml

for Health Research. The views expressed in this publication are those of the author(s) and not necessarily those of the NHS, the National Institute for Health Research or the Department of Health.

Competing interests statement: none declared

Ethics approval: This study protocol has approval from the Independent Scientific Advisory Committee for the UK Medicines and Healthcare products Regulatory Agency Database Research (protocol 18_313).

Data sharing statement: the clinical codes used for this study are provided as supplementary material.

For peer review only

For peer review only - http://bmjopen.bmj.com/site/about/guidelines.xhtml

REFERENCES

1. Fraser LK, Miller M, Hain R, et al. Rising National Prevalence of Life-Limiting Conditions in Children in England. *Pediatrics* 2012;129(4):E923-E29. doi: 10.1542/peds.2011-2846

2. Hain R, Devins M, Hastings R, et al. Paediatric palliative care: development and pilot study of a 'Directory' of life-limiting conditions. *Bmc Palliative Care* 2013;12 doi: 10.1186/1472-684x-12-43

3. Royal College of Paediatrics and Child Health. National Paediatric Diabetes Audit, 2014.

4. Feudtner C, DiGiuseppe DL, Neff JM. Hospital care for children and young adults with complex chronic conditions in the last year of life. *Pediatric Research* 2003;53(4):266A-66A.

5. Fraser LK, Jarvis SW, Moran N, et al. Children in Scotland requiring Palliative Care: identifying numbers and needs (The ChiSP Study). York, UK: University of York, 2015.

6. National Institute for Health and Crae Excellence (NICE). End of life care for infants, children and young people with life-limiting conditions: planning and management, 2016.

7. Thurston S, Paul L, Loney P, et al. Associations and costs of parental symptoms of psychiatric distress in a multi-diagnosis group of children with special needs. *Journal of Intellectual Disability Research* 2011;55:263-80. doi: 10.1111/j.1365-2788.2010.01356.x

8. Brehaut JC, Kohen DE, Raina P, et al. The Health of Primary Caregivers of Children With Cerebral Palsy: How Does It Compare With That of Other Canadian Caregivers? *Pediatrics* 2004;114(2):e182-e91. doi: 10.1542/peds.114.2.e182

9. Lach LM, Kohen DE, Garner RE, et al. The health and psychosocial functioning of caregivers of children with neurodevelopmental disorders. *Disability and Rehabilitation* 2009;31(8):607-18. doi: 10.1080/09638280802242163

10. Lee MH, Park C, Matthews AK, et al. Differences in physical health, and health behaviors between family caregivers of children with and without disabilities. *Disability and Health Journal* 2017;10(4):565-70. doi: 10.1016/j.dhjo.2017.03.007

11. Herrett E, Gallagher AM, Bhaskaran K, et al. Data Resource Profile: Clinical Practice Research Datalink (CPRD). *International Journal of Epidemiology* 2015;44(3):827-36. doi: 10.1093/ije/dyv098

12. Clinical Practice Research Datalink. Hospital Episode Statistics (HES) Admitted Patient Care and GOLD Documentation (Set 14): Medicines & Healthcare products Regulatory Agency, 2017.

For peer review only

For peer review only - http://bmjopen.bmj.com/site/about/guidelines.xhtml

13. Health & Social Care Information Centre. Methodology for creation of the HES Patient ID (HESID), 2014.

14. CPRD. Clinical Practice Research Datalink 2014 [Available from: http://www.cprd.com/intro.asp accessed 29/01/2014.

15. Hardelid P, Dattani N, Gilbert R. Estimating the prevalence of chronic conditions in children who die in England, Scotland and Wales: a data linkage cohort study. *BMJ Open* 2014;4(8):e005331. doi: 10.1136/bmjopen-2014-005331

16. Prady SL, Pickett KE, Petherick ES, et al. Evaluation of ethnic disparities in detection of depression and anxiety in primary care during the maternal period: combined analysis of routine and cohort data. *British Journal of Psychiatry* 2016;208(5):453-61. doi: 10.1192/bjp.bp.114.158832

17. NOMIS. Census 2011 - Ethnic group by sex by age: NOMIS, 2013.

18. Government DfCaL. The English Indices of Deprivation 2015, 2015.

19. StataCorp. Stata Statistical Software: Release 14. College Station, TX: StataCorp LP, 2017.

20. Personal Social Services Research Unit. Unit Costs of Health and Social Care 2014, 2014.

21. Department of Health. Payment by Results Guidance for 2013-14, 2013.

22. Department of health. Reference costs 2013-14, 2014.

23. Sterne JAC, White IR, Carlin JB, et al. Multiple imputation for missing data in epidemiological and clinical research: potential and pitfalls. *BMJ* 2009;338:b2393. doi: 10.1136/bmj.b2393

For peer review only

Supplementary Material

Appendix 1 Read Codes to identify a child with a Life-limiting condition

medcode	clinicalevents	referralevents	testevents	immunisationevents	readcode	readterm	databasebuild
241	217428	9975	65	0	G30..00	Acute myocardial infarction	Feb-09
245	7800	168	4	0	G410.00	Primary pulmonary hypertension	Feb-09
318	411	23	0	0	B210.00	Malignant neoplasm of glottis	Feb-09
319	4215	149	0	0	B21..00	Malignant neoplasm of larynx	Feb-09
348	54604	2944	8	0	B34..11	Ca female breast	Feb-09
512	61437	4071	14	0	K05..00	Chronic renal failure	Feb-09
579	9051	803	14	0	BBE1.00	[M]Malignant melanoma NOS	Feb-09
684	86980	7892	6	0	F20..00	Multiple sclerosis	Feb-09
765	3287	381	1	0	BBe1.00	[M]Neurofibromatosis NOS	Feb-09
780	123877	3483	21	0	B46..00	Malignant neoplasm of prostate	Feb-09
865	30087	2172	10	0	B32..00	Malignant melanoma of skin	Feb-09
1056	8129	525	4	0	B5z..00	Malignant neoplasm of other and unspecified site NOS	Feb-09
1062	16003	663	2	0	B10..00	Malignant neoplasm of oesophagus	Feb-09
1204	4721	258	1	0	G30..14	Heart attack	Feb-09
1220	35966	1430	2	0	B13..00	Malignant neoplasm of colon	Feb-09
1391	312	6	0	0	C327100	Gaucher's disease	Feb-09
1481	48	1	0	0	B600.00	Reticulosarcoma	Feb-09
1483	12690	675	2	0	BBg1.11	[M]Lymphoma NOS	Feb-09
1599	4047	172	1	0	B4A0.00	Malignant neoplasm of kidney parenchyma	Feb-09
1624	38351	2463	10	0	BB2A.00	[M]Squamous cell carcinoma NOS	Feb-09
1800	21118	946	2	0	B141.00	Malignant neoplasm of rectum	Feb-09
1950	9157	203	0	0	BB4..00	[M]Transitional cell papillomas and carcinomas	Feb-09
1952	166	3	0	0	B580.00	Secondary malignant neoplasm of kidney	Feb-09
1986	11636	589	3	0	B440.11	Cancer of ovary	Feb-09
2123	944	54	1	0	BBc1.00	[M]Neuroblastoma NOS	Feb-09

For peer review only - http://bmjopen.bmj.com/site/about/guidelines.xhtml

2272	28720	722	23	0	BB5..11	[M]Adenocarcinomas	Feb-09
2298	2427	120	0	0	F203.00	Exacerbation of multiple sclerosis	Feb-09
2462	10288	433	13	0	B61..00	Hodgkin's disease	Feb-09
2492	9864	1312	66	0	B33z.00	Malignant neoplasm of skin NOS	Feb-09
2587	35784	1469	5	0	B22z.11	Lung cancer	Feb-09
2744	3591	129	2	0	B40..00	"Malignant neoplasm of uterus, part unspecified"	Feb-09
2747	7108	210	1	0	B41..00	Malignant neoplasm of cervix uteri	Feb-09
2755	19616	1231	106	0	B....11	Cancers	Feb-09
2815	6116	113	1	0	B133.00	Malignant neoplasm of sigmoid colon	Feb-09
2816	833	34	0	0	P51..00	Transposition of great vessels	Feb-09
2835	6829	291	5393	0	43C3.11	HIV positive	Feb-09
2890	7205	174	2	0	B430200	Malignant neoplasm of endometrium of corpus uteri	Feb-09
2961	1999	106	0	0	B47z.11	Seminoma of testis	Feb-09
3152	13083	265	0	0	BB13.00	"[M]Carcinoma, metastatic, NOS"	Feb-09
3197	22344	1073	2	0	BB03.00	"[M]Neoplasm, metastatic"	Feb-09
3204	20442	926	6	0	G55..00	Cardiomyopathy	Feb-09
3213	294	19	0	0	B430.00	"Malignant neoplasm of corpus uteri, excluding isthmus"	Feb-09
3230	4027	195	0	0	B41..11	Cervical carcinoma (uterus)	Feb-09
3357	1876	129	0	0	B1...11	Carcinoma of digestive organs and peritoneum	Feb-09
3371	3101	115	0	0	BBg2.11	[M]Non Hodgkins lymphoma	Feb-09
3541	536	56	0	0	B48..00	Malignant neoplasm of penis and other male genital organs	Feb-09
3591	4247	308	0	0	F134.00	Huntington's chorea	Feb-09
3604	14600	525	0	0	B627.00	Non - Hodgkin's lymphoma	Feb-09
3710	407	34	0	0	BBB1.00	[M]Adenolymphoma	Feb-09
3811	6639	194	0	0	B134.00	Malignant neoplasm of caecum	Feb-09
3862	244	16	0	0	P601.00	Congenital atresia of the pulmonary valve	Feb-09

For peer review only

3903	34821	1333	3	0	B22z.00	Malignant neoplasm of bronchus or lung NOS	Feb-09
3947	6445	288	10	0	P1...00	Spina bifida	Feb-09
3968	128623	3421	8	0	B34..00	Malignant neoplasm of female breast	Feb-09
3969	21	0	0	0	BB9M.00	[M]Intracystic carcinoma NOS	Feb-09
4072	544	43	0	0	B680.00	Acute leukaemia NOS	Feb-09
4118	360	14	0	0	BBV9.00	[M]Myxoid chondrosarcoma	Feb-09
4137	5072	154	1	0	B570.00	Secondary malignant neoplasm of lung	Feb-09
4158	1619	149	0	0	PG51.00	Osteogenesis imperfecta	Feb-09
4165	641	70	0	0	F140.00	Friedreich's ataxia	Feb-09
4217	770	26	0	0	BB5c.00	[M]Parathyroid adenomas and adenocarcinomas	Feb-09
4218	48	0	0	0	B541.00	Malignant neoplasm of parathyroid gland	Feb-09
4222	2158	123	0	0	B64..11	Lymphatic leukaemia	Feb-09
4250	3215	381	0	0	B68z.00	Leukaemia NOS	Feb-09
4251	2752	114	0	0	B640.00	Acute lymphoid leukaemia	Feb-09
4272	983	58	0	0	PH3y200	Epidermolysis bullosa	Feb-09
4388	897	21	0	0	B020.00	Malignant neoplasm of parotid gland	Feb-09
4403	13552	429	0	0	B577.11	Liver metastases	Feb-09
4413	6444	194	0	0	B650.00	Acute myeloid leukaemia	Feb-09
4473	590	13	0	0	BBY0.00	[M]Ewing's sarcoma	Feb-09
4478	1446	87	0	0	F256.00	Infantile spasms	Feb-09
4479	1238	93	0	0	PK5..00	Tuberous sclerosis	Feb-09
4554	1713	44	0	0	B454.00	Malignant neoplasm of vulva unspecified	Feb-09
4632	14309	947	1	0	B33..00	Other malignant neoplasm of skin	Feb-09
4637	722	14	0	0	BBr..00	[M]Leukaemias	Feb-09
4673	P20..00	254	0	8	0	Encephalocele	Feb-09
4796	F152.00	10361	0	689	0	Motor neurone disease	Feb-09
4852	642	90	0	0	BB24.12	[M]Verrucous squamous cell carcinoma	Feb-09
4865	7627	274	1	0	B10z.11	Oesophageal cancer	Feb-09
4944	14705	788	3	0	B630.00	Multiple myeloma	Feb-09
5052	250	13	0	0	BBV1.13	[M]Osteogenic sarcoma NOS	Feb-09

For peer review only - http://bmjopen.bmj.com/site/about/guidelines.xhtml

5136	80	10	1	0	B911013	Choriocarcinoma	Feb-09
5137	302	81	1	0	B624.11	Leukaemic reticuloendotheliosis	Feb-09
5179	232	11	1	0	B620.00	Nodular lymphoma (Brill - Symmers disease)	Feb-09
5198	3093	125	0	0	B583000	Secondary malignant neoplasm of brain	Feb-09
5199	5722	87	0	0	B583200	Cerebral metastasis	Feb-09
5306	235	15	0	0	P100000	"Spina bifida with hydrocephalus, unspecified"	Feb-09
5393	672	51	0	0	F391000	Duchenne muscular dystrophy	Feb-09
5395	2491	73	0	0	A540.00	Eczema herpeticum - Kaposi's varicelliform eruption	Feb-09
5431	P11..00	87	0	1	0	Spina bifida without mention of hydrocephalus	Feb-09
5443	305	6	0	0	F122.00	Malignant neuroleptic syndrome	Feb-09
5455	2673	45	0	0	BB53.00	"[M]Adenocarcinoma, metastatic, NOS"	Feb-09
5637	5356	129	2	0	B53..00	Malignant neoplasm of thyroid gland	Feb-09
5712	2991	84	0	0	P21..00	Microcephalus	Feb-09
5842	6910	159	0	0	B58..00	Secondary malignant neoplasm of other specified sites	Feb-09
5901	9334	321	0	0	B141.12	Rectal carcinoma	Feb-09
5915	536	12	0	0	BBrA400	[M]Hairy cell leukaemia	Feb-09
5932	493	19	0	0	BB06.00	"[M]Tumour cells, uncertain whether benign or malignant"	Feb-09
5964	1625	125	2	0	F39B.00	Muscular dystrophy	Feb-09
6170	10811	117	0	0	B590.11	Carcinomatosis	Feb-09
6220	13732	778	263	0	C370.00	Cystic fibrosis	Feb-09
6316	245	4	0	0	BBr0100	[M]Acute leukaemia NOS	Feb-09
6436	3624	40	0	0	BB43.00	[M]Transitional cell carcinoma NOS	Feb-09
6471	1998	50	0	0	B57..11	Metastases of respiratory and/or digestive systems	Feb-09
6515	P211.00	231	0	2	0	Micrencephaly	Feb-09
6712	3881	65	1	0	K050.00	End stage renal failure	Feb-09

For peer review only - http://bmjopen.bmj.com/site/about/guidelines.xhtml

6746	2004	25	1	0	BB5M.00	[M]Tubular adenomas and adenocarcinomas	Feb-09
6806	187	6	0	0	B12..00	Malignant neoplasm of small intestine and duodenum	Feb-09
6883	951	15	0	0	Q317000	Perinatal bronchopulmonary dysplasia	Feb-09
6920	640	22	0	0	BB5U.00	[M]Villous adenomas and adenocarcinomas	Feb-09
6935	745	5	0	0	B131.00	Malignant neoplasm of transverse colon	Feb-09
6966	267	1	0	0	BB1D.00	[M]Spindle cell carcinoma	Feb-09
7046	2915	13	0	0	B43..00	Malignant neoplasm of body of uterus	Feb-09
7176	2417	106	0	0	B65..00	Myeloid leukaemia	Feb-09
7219	2385	46	0	0	B141.11	Carcinoma of rectum	Feb-09
7319	240	0	0	0	BB9G.00	[M]Infiltrating ductular carcinoma	Feb-09
7476	942	17	0	0	BBQ1.00	[M]Seminomas	Feb-09
7484	4152	107	0	0	B226.00	Mesothelioma	Feb-09
7535	3403	77	0	0	G554400	Primary dilated cardiomyopathy	Feb-09
7593	3116	266	0	0	H51y700	Malignant pleural effusion	Feb-09
7654	7216	308	2	0	B585.00	Secondary malignant neoplasm of bone and bone marrow	Feb-09
7740	236	10	0	0	B470200	Seminoma of undescended testis	Feb-09
7805	9705	177	0	0	B440.00	Malignant neoplasm of ovary	Feb-09
7830	2203	37	0	0	B56..11	Lymph node metastases	Feb-09
7856	362	15	0	0	BBJH.00	[M]Dedifferentiated liposarcoma	Feb-09
7940	453	8	0	0	ByuDF11	[X]Non-Hodgkin's lymphoma NOS	Feb-09
7941	183	2	0	0	BBW4.00	[M]Chondrosarcoma NOS	Feb-09
7945	134	0	0	0	F256000	Hypsarrhythmia	Feb-09
7982	224	2	0	0	B161200	Malignant neoplasm of common bile duct	Feb-09
8010	3501	202	0	0	G551.00	Hypertrophic obstructive cardiomyopathy	Feb-09
8032	1365	37	0	0	BB5B.00	[M]Pancreatic adenomas and carcinomas	Feb-09
8085	2979	102	0	0	BBF1.00	[M]Sarcoma NOS	Feb-09
8088	18	1	0	0	BBG3.00	[M]Fibromyxosarcoma	Feb-09
8101	2854	120	0	0	BB5a.00	[M]Renal adenoma and carcinoma	Feb-09

For peer review only - http://bmjopen.bmj.com/site/about/guidelines.xhtml

8154	800	49	0	0	B576200	Malignant ascites	Feb-09
8166	14157	485	1	0	B17..00	Malignant neoplasm of pancreas	Feb-09
8267	29	1	0	0	F363.00	Refsum's disease	Feb-09
8281	11	0	0	0	A789A00	HIV disease resulting in wasting syndrome	Feb-09
8328	326	7	0	0	BBbC.00	"[M]Astrocytoma, anaplastic type"	Feb-09
8351	907	12	0	0	BB91.00	[M]Infiltrating duct carcinoma	Feb-09
8386	10022	372	1	0	B11..00	Malignant neoplasm of stomach	Feb-09
8523	3902	212	0	0	BBb0.11	[M]Glioma NOS	Feb-09
8547	3113	135	1	0	BBbB.00	[M]Astrocytoma NOS	Feb-09
8550	98	2	0	0	B542000	Malignant neoplasm of pituitary gland	Feb-09
8600	2847	117	0	0	1D18.00	Pain from metastases	Feb-09
8606	2207	45	0	0	BB5j.00	[M]Endometrioid adenomas and carcinomas	Feb-09
8625	16007	1249	4	0	B641.00	Chronic lymphoid leukaemia	Feb-09
8627	492	5	1	0	BB07.00	"[M]Tumour cells, malignant"	Feb-09
8649	455	11	0	0	ByuDF00	"[X]Non-Hodgkin's lymphoma, unspecified type"	Feb-09
8660	139	2	0	0	BBV1.00	[M]Osteosarcoma NOS	Feb-09
8695	6720	163	2	0	BB12.00	[M]Carcinoma NOS	Feb-09
8711	3485	112	3	0	BB5D100	[M]Cholangiocarcinoma	Feb-09
8771	1081	5	0	0	B170.00	Malignant neoplasm of head of pancreas	Feb-09
8918	229	4	0	0	B15..00	Malignant neoplasm of liver and intrahepatic bile ducts	Feb-09
8930	4784	25	0	0	BB52.00	[M]Adenocarcinoma NOS	Feb-09
9011	368	9	0	0	P543.00	Eisenmenger's complex	Feb-09
9030	179	4	0	0	B55..00	Malignant neoplasm of other and ill-defined sites	Feb-09
9088	480	10	1	0	B130.00	Malignant neoplasm of hepatic flexure of colon	Feb-09
9118	6778	234	0	0	B13z.11	Colonic cancer	Feb-09
9156	201	4	0	0	BB1K.00	[M]Oat cell carcinoma	Feb-09
9179	703	69	0	0	F151.00	Spinal muscular atrophy	Feb-09

For peer review only - http://bmjopen.bmj.com/site/about/guidelines.xhtml

9237	578	4	0	0	B21z.00	Malignant neoplasm of larynx NOS	Feb-09
9271	2219	87	0	0	F240.00	Quadriplegia	Feb-09
9291	2384	46	0	0	BB1J.00	[M]Small cell carcinoma NOS	Feb-09
9366	297	7	0	0	BB13.11	[M]Secondary carcinoma	Feb-09
9402	734	15	0	0	G55y.11	Secondary dilated cardiomyopathy	Feb-09
9444	142	7	0	0	ZV10400	[V]Personal history of malignant neoplasm of genital organ	Feb-09
9447	969	3	0	0	BB5j200	[M]Endometrioid carcinoma	Feb-09
9470	10661	57	3	0	B34z.00	Malignant neoplasm of female breast NOS	Feb-09
9476	729	18	0	0	B471100	Teratoma of descended testis	Feb-09
9491	632	23	0	0	B142.11	Anal carcinoma	Feb-09
9505	71	2	0	0	B582600	Secondary malignant neoplasm of skin of breast	Feb-09
9575	3391	54	1	0	BBbL.11	[M]Glioblastoma multiforme	Feb-09
9600	1246	24	0	0	B232.00	Mesothelioma of pleura	Feb-09
9618	801	46	0	0	B56..00	Secondary and unspecified malignant neoplasm of lymph nodes	Feb-09
9622	41	4	0	0	B525.00	Malignant neoplasm of cauda equina	Feb-09
9712	820	9	0	0	BB4A.00	[M]Papillary transitional cell carcinoma	Feb-09
9722	P114.00	268	0	3	0	Meningomyelocele	Feb-09
9859	261	1	0	0	BBQ1z00	[M]Seminoma NOS	Feb-09
9902	1265	48	0	0	B3...11	"Carcinoma of bone, connective tissue, skin and breast"	Feb-09
9984	168	6	0	0	B00..11	Carcinoma of lip	Feb-09
10166	1986	62	8	0	C307000	Hyperglycinaemia	Feb-09
10283	3195	152	0	0	B01..00	Malignant neoplasm of tongue	Feb-09
10358	1844	16	0	0	B222.00	"Malignant neoplasm of upper lobe, bronchus or lung"	Feb-09
10368	972	21	0	0	B11..11	Gastric neoplasm	Feb-09
10439	120	7	0	0	ZV76.00	[V]Screening for malignant neoplasm	Feb-09
10541	264	2	0	0	BB22.00	[M]Papillary carcinoma NOS	Feb-09

For peer review only - http://bmjopen.bmj.com/site/about/guidelines.xhtml

10588	1685	50	0	0	BBK0200	[M]Leiomyosarcoma NOS	Feb-09
10628	882	38	0	0	PJyy200	Fragile X chromosome	Feb-09
10668	2130	21	0	0	BB5a000	[M]Renal cell carcinoma	Feb-09
10698	76	2	0	0	B450100	Malignant neoplasm of vaginal vault	Feb-09
10726	3296	148	0	0	B651.00	Chronic myeloid leukaemia	Feb-09
10851	779	26	0	0	B51..11	Cerebral tumour - malignant	Feb-09
10864	565	6	0	0	B132.00	Malignant neoplasm of descending colon	Feb-09
10913	72	0	0	0	BBD0.00	[M]Paraganglioma NOS	Feb-09
10946	1278	22	0	0	B136.00	Malignant neoplasm of ascending colon	Feb-09
10949	433	11	0	0	B162.00	Malignant neoplasm of ampulla of Vater	Feb-09
10955	407	17	0	0	C391100	Di George syndrome	Feb-09
10956	487	23	0	0	PKy9300	Prader - Willi syndrome	Feb-09
10995	2057	31	0	0	B5...00	Malignant neoplasm of other and unspecified sites	Feb-09
11035	475	4	0	0	B593.00	Primary malignant neoplasm of unknown site	Feb-09
11513	297	15	0	0	PH30.00	Congenital ectodermal dysplasia	Feb-09
11531	431	15	0	0	P00..00	Anencephalus	Feb-09
11628	5160	168	0	0	B1z0.11	Cancer of bowel	Feb-09
11773	3006	40	0	0	7L1A.11	Dialysis for renal failure	Feb-09
11991	58	0	0	0	B454.11	Primary vulval cancer	Feb-09
12067	227	20	0	0	B927.11	Von Recklinghausen's disease	Feb-09
12106	966	14	0	0	ZV10.00	[V]Personal history of malignant neoplasm	Feb-09
12146	17	4	0	0	BBr2000	[M]Lymphoid leukaemia NOS	Feb-09
12204	220	10	0	0	C354A00	Metastatic calcification	Feb-09
12309	524	15	0	0	BBb..00	[M]Gliomas	Feb-09
12323	1428	3	0	0	B6...00	Malignant neoplasm of lymphatic and haemopoietic tissue	Feb-09
12335	6535	469	2	0	B62y.00	Malignant lymphoma NOS	Feb-09
12388	249	3	0	0	BB43.11	[M]Urothelial carcinoma	Feb-09
12389	514	9	0	0	B4A1.00	Malignant neoplasm of renal pelvis	Feb-09

For peer review only - http://bmjopen.bmj.com/site/about/guidelines.xhtml

12427	293	8	0	0	BB9F.00	[M]Lobular carcinoma NOS	Feb-09
12464	142	1	0	0	B62x200	Peripheral T-cell lymphoma	Feb-09
12480	321	39	0	0	BB9K000	[M]Paget's disease and intraductal carcinoma of breast	Feb-09
12490	74	2	0	0	B550200	Malignant neoplasm of nose NOS	Feb-09
12497	143	0	0	0	BB82100	[M]Mucinous adenocarcinoma	Feb-09
12499	1207	3	0	0	Byu6.00	[X]Malignant neoplasm of breast	Feb-09
12539	1040	61	0	0	B3...12	Sarcoma of bone and connective tissue	Feb-09
12580	173	0	0	0	BBB0.00	[M]Adenosquamous carcinoma	Feb-09
12582	1332	11	0	0	B224100	Malignant neoplasm of lower lobe of lung	Feb-09
12609	220	3	0	0	BB19.00	"[M]Carcinoma, anaplastic type, NOS"	Feb-09
12870	2717	45	0	0	B221.00	Malignant neoplasm of main bronchus	Feb-09
13243	10271	325	1	0	B22..00	"Malignant neoplasm of trachea, bronchus and lung"	Feb-09
13252	1710	16	0	0	B4...00	Malignant neoplasm of genitourinary organ	Feb-09
13569	20097	639	1	0	B590.00	Disseminated malignancy NOS	Feb-09
13574	10	0	0	0	BB36.00	[M]Metatypical carcinoma	Feb-09
13575	32	1	31	0	4F32.00	Ascitic fluid: malignant cells	Feb-09
13581	149	0	0	0	R210.00	[D]Sudden infant death syndrome	Feb-09
14242	159	13	240	0	42D4.00	RBC's - sickle cells present	Feb-09
14712	565	42	0	0	B00..00	Malignant neoplasm of lip	Feb-09
14792	674	42	0	0	B05..00	Malignant neoplasm of other and unspecified parts of mouth	Feb-09
14800	5086	263	1	0	B11z.00	Malignant neoplasm of stomach NOS	Feb-09
15027	844	45	0	0	B62yz00	Malignant lymphoma NOS	Feb-09
15036	49	2	0	0	B626.00	Malignant mast cell tumours	Feb-09
15103	4814	199	3	0	B577.00	Secondary malignant neoplasm of liver	Feb-09
15148	2504	66	1	0	B47..00	Malignant neoplasm of testis	Feb-09
15182	411	35	6	0	B31z.00	"Malignant neoplasm of connective and soft tissue, site NOS"	Feb-09
15221	187	8	0	0	B220.00	Malignant neoplasm of trachea	Feb-09

For peer review only - http://bmjopen.bmj.com/site/about/guidelines.xhtml

15223	633	16	0	0	B4A2.00	Malignant neoplasm of ureter	Feb-09
15343	1275	95	1	0	PG52.00	Osteopetrosis	Feb-09
15422	2316	216	3	0	D20..00	Aplastic anaemia	Feb-09
15504	150	5	0	0	B62y800	Malignant lymphoma NOS of lymph nodes of multiple sites	Feb-09
15543	104	9	0	0	BB01.00	"[M]Neoplasm, uncertain whether benign or malignant"	Feb-09
15644	173	6	0	0	B4A3.00	Malignant neoplasm of urethra	Feb-09
15658	87	10	0	0	D201.00	Acquired aplastic anaemia	Feb-09
15684	33	4	1	0	B204.00	Malignant neoplasm of frontal sinus	Feb-09
15709	1697	725	0	0	B1...00	Malignant neoplasm of digestive organs and peritoneum	Feb-09
15711	137	2	0	0	B510.00	Malignant neoplasm cerebrum (excluding lobes and ventricles)	Feb-09
15868	95	12	0	0	B335z00	"Malignant neoplasm of skin of trunk, excluding scrotum, NOS"	Feb-09
15907	224	20	5	0	B16z.00	Malignant neoplasm gallbladder/extrahepatic bile ducts NOS	Feb-09
15976	240	19	0	0	B552.00	Malignant neoplasm of abdomen	Feb-09
15989	1174	80	0	0	B47z.12	Teratoma of testis	Feb-09
15991	424	15	0	0	B506.00	Malignant neoplasm of choroid	Feb-09
16038	69	1	0	0	C303300	Maple syrup urine disease	Feb-09
16075	394	22	0	0	B30z.00	Malignant neoplasm of bone and articular cartilage NOS	Feb-09
16087	423	12	0	0	PKy4.00	William syndrome	Feb-09
16105	842	12	0	0	B160.00	Malignant neoplasm of gallbladder	Feb-09
16117	1296	88	0	0	F240.11	Tetraplegia	Feb-09
16118	F392000	1938	0	149	1	Dystrophia myotonica (Steinert's disease)	Feb-09
16126	357	12	0	0	B150000	Primary carcinoma of liver	Feb-09
16146	42	4	0	0	BBB2.00	[M]Adenocarcinoma with squamous metaplasia	Feb-09

For peer review only - http://bmjopen.bmj.com/site/about/guidelines.xhtml

16202	652	24	0	0	B333400	Malignant neoplasm of skin of nose (external)	Feb-09
16213	341	33	0	0	B572.00	Secondary malignant neoplasm of pleura	Feb-09
16241	1580	43	0	0	B060.00	Malignant neoplasm of tonsil	Feb-09
16280	158	1	0	0	B550400	Malignant neoplasm of neck NOS	Feb-09
16297	654	30	0	0	B0z0.00	Malignant neoplasm of pharynx unspecified	Feb-09
16298	48	0	0	0	B18z.00	Malignant neoplasm of retroperitoneum and peritoneum NOS	Feb-09
16416	400	56	1	0	B681.00	Chronic leukaemia NOS	Feb-09
16443	45	3	0	0	C375211	Hunter's syndrome	Feb-09
16460	554	30	0	0	BBg2.00	"[M]Malignant lymphoma, non Hodgkin's type"	Feb-09
16500	809	32	0	0	B58z.00	Secondary malignant neoplasm of other specified site NOS	Feb-09
16677	143	3	0	0	BB9B.00	[M]Medullary carcinoma NOS	Feb-09
16692	1029	9	0	0	BB14.00	[M]Carcinomatosis	Feb-09
16704	73	1	0	0	B302.00	Malignant neoplasm of vertebral column	Feb-09
16711	519	34	0	0	PKy7B00	Stickler syndrome	Feb-09
16723	678	27	0	0	BB5S212	[M]Bronchiolar carcinoma	Feb-09
16760	371	4	0	0	B58y000	Secondary malignant neoplasm of breast	Feb-09
16774	412	9	0	0	BBmD.00	[M] Cutaneous lymphoma	Feb-09
16854	2691	189	0	0	B927.00	Neurofibromatosis - Von Recklinghausen's disease	Feb-09
16915	96	1	0	0	B151.00	Malignant neoplasm of intrahepatic bile ducts	Feb-09
16967	32	0	0	0	B432.00	Malignant neoplasm of overlapping lesion of corpus uteri	Feb-09
17151	1215	68	0	0	BB81.11	[M]Ovarian cystadenoma or carcinoma	Feb-09
17178	525	12	0	0	BBg..00	"[M]Lymphomas, NOS or diffuse"	Feb-09
17182	1343	8	0	0	B627C11	Follicular lymphoma NOS	Feb-09
17212	143	1	0	0	BBLH.00	[M]Rhabdoid sarcoma	Feb-09

For peer review only

17314	758	81	0	0	BBL7112	[M]Wilms' tumour	Feb-09
17366	296	24	0	0	BBF..00	[M]Soft tissue tumours and sarcomas NOS	Feb-09
17391	142	4	0	0	B221000	Malignant neoplasm of carina of bronchus	Feb-09
17460	93	3	0	0	B627700	Diffuse non-Hodgkin's lymphoblastic (diffuse) lymphoma	Feb-09
17468	289	5	0	0	BBQ..00	[M]Germ cell neoplasms	Feb-09
17475	128	6	0	0	B300A00	Malignant neoplasm of maxilla	Feb-09
17559	550	22	0	0	B1z0.00	"Malignant neoplasm of intestinal tract, part unspecified"	Feb-09
17841	147	0	0	0	B481.00	Malignant neoplasm of glans penis	Feb-09
17874	184	7	0	0	B181.00	Mesothelioma of peritoneum	Feb-09
17887	982	16	0	0	B62x.00	Malignant lymphoma otherwise specified	Feb-09
17912	24	10	0	0	B042.00	"Malignant neoplasm, overlapping lesion of floor of mouth"	Feb-09
18245	152	8	0	0	B330.00	Malignant neoplasm of skin of lip	Feb-09
18255	141	19	1	0	BB5L.00	[M]Adenomatous and adenocarcinomatous polyps	Feb-09
18270	1315	40	0	0	7G03K00	Excision malignant skin tumour	Feb-09
18314	255	5	0	0	B30..00	Malignant neoplasm of bone and articular cartilage	Feb-09
18321	20	1	0	0	PG56012	Conradi - Hunermann syndrome	Feb-09
18354	86	2	0	0	B33y.00	Malignant neoplasm of other specified skin sites	Feb-09
18383	603	7	0	0	BBmH.00	[M] Large cell lymphoma	Feb-09
18537	224	8	0	0	P233.11	Dandy - Walker syndrome	Feb-09
18613	383	57	1	0	B120.00	Malignant neoplasm of duodenum	Feb-09
18616	313	0	0	0	B58y.00	Secondary malignant neoplasm of other specified sites	Feb-09
18617	3533	53	0	0	B51..00	Malignant neoplasm of brain	Feb-09

18618	31	1	0	0	B335300	Malignant neoplasm of skin of abdominal wall	Feb-09
18619	351	2	0	0	B137.00	Malignant neoplasm of splenic flexure of colon	Feb-09
18632	305	2	0	0	B135.00	Malignant neoplasm of appendix	Feb-09
18676	1030	33	0	0	B585000	Pathological fracture due to metastatic bone disease	Feb-09
18678	226	0	0	0	B224000	Malignant neoplasm of lower lobe bronchus	Feb-09
18712	2030	46	0	0	B4A..11	Renal malignant neoplasm	Feb-09
18744	465	6	0	0	BBn0.11	[M]Multiple myeloma	Feb-09
18771	72	0	0	0	BBLJ.00	[M]Clear cell sarcoma of kidney	Feb-09
18882	26	16	1	0	B006.00	Malignant neoplasm of overlapping lesion of lip	Feb-09
18905	8	0	0	0	C370300	Cystic fibrosis with intestinal manifestations	Feb-09
18914	187	13	0	0	C370200	Cystic fibrosis with pulmonary manifestations	Feb-09
19007	218	0	0	0	Q48E.00	Periventricular leucomalacia	Feb-09
19038	32	0	0	0	PJ1z.11	Trisomy 13 NOS	Feb-09
19041	947	23	0	0	BB29.12	[M]Intraepidermal carcinoma NOS	Feb-09
19083	1453	209	0	0	1J04.00	Suspected lymphoma	Feb-09
19091	1049	154	0	0	BB5..00	[M]Adenomas and adenocarcinomas	Feb-09
19140	3	0	0	0	B614800	Hodgkin's nodular sclerosis of lymph nodes of multiple sites	Feb-09
19141	439	7	0	0	B44..00	Malignant neoplasm of ovary and other uterine adnexa	Feb-09
19144	222	10	0	0	Byu4.00	[X]Melanoma and other malignant neoplasms of skin	Feb-09
19162	41	0	0	0	B493.00	Malignant neoplasm of anterior wall of urinary bladder	Feb-09
19226	142	3	0	0	B513.00	Malignant neoplasm of parietal lobe	Feb-09

For peer review only - http://bmjopen.bmj.com/site/about/guidelines.xhtml

19263	417	21	0	0	BB5f.00	[M]Thyroid adenoma and adenocarcinoma	Feb-09
19318	116	3	0	0	B112.00	Malignant neoplasm of pyloric antrum of stomach	Feb-09
19321	25	1	0	0	B311300	Malignant neoplasm of connective and soft tissue of hand	Feb-09
19334	231	3	0	0	BBL9.00	[M]Carcinosarcoma NOS	Feb-09
19372	1294	108	0	0	B64..00	Lymphoid leukaemia	Feb-09
19415	732	23	0	0	B0...00	"Malignant neoplasm of lip, oral cavity and pharynx"	Feb-09
19423	663	14	0	0	B35..00	Malignant neoplasm of male breast	Feb-09
19437	481	12	0	0	B30z000	Osteosarcoma	Feb-09
19444	520	7	0	0	Byu4100	"[X]Malignant melanoma of skin, unspecified"	Feb-09
19475	54	1	0	0	B471.00	Malignant neoplasm of descended testis	Feb-09
19584	413	3	1	0	Q401.00	Congenital cytomegalovirus infection	Feb-09
19657	158	6	0	0	B911000	Malignant hydatidiform mole	Feb-09
19678	996	118	0	0	BB29.13	[M]Intraepithelial squamous cell carcinoma	Feb-09
19692	286	85	0	0	1J02.00	Suspected leukaemia	Feb-09
19731	26	1	0	0	BB5N.11	[M]Adenoma or or adenocarcinoma in polyposis coli	Feb-09
19945	340	41	27	0	B582.00	Secondary malignant neoplasm of skin	Feb-09
19974	42	6	0	0	B660.00	Acute monocytic leukaemia	Feb-09
20092	562	18	0	0	B04..00	Malignant neoplasm of floor of mouth	Feb-09
20151	371	18	9	0	Q400.00	Congenital rubella	Feb-09
20160	382	13	0	0	B50..00	Malignant neoplasm of eye	Feb-09
20166	41	1	0	0	B45z.00	Malignant neoplasm of female genital organ NOS	Feb-09
20292	299	7	0	0	B02..00	Malignant neoplasm of major salivary glands	Feb-09
20437	350	9	0	0	BBk..00	"[M]Lymphomas, nodular or follicular"	Feb-09

For peer review only - http://bmjopen.bmj.com/site/about/guidelines.xhtml

20440	221	9	0	0	B69..00	Myelomonocytic leukaemia	Feb-09
20493	1686	19	0	0	F20z.00	Multiple sclerosis NOS	Feb-09
20635	32	1	0	0	BBr2011	[M]Lymphatic leukaemia	Feb-09
20685	91	1	0	0	B346.00	Malignant neoplasm of axillary tail of female breast	Feb-09
20699	135	1	0	0	7H19500	Closure of gastrochisis	Feb-09
20710	843	30	0	0	BBj..00	[M]Hodgkin's disease	Feb-09
20772	281	7	0	0	P67..00	Hypoplastic left heart syndrome	Feb-09
20807	184	18	0	0	BB26.00	[M]Papillary squamous cell carcinoma	Feb-09
20822	132	3	0	0	Q48y100	Congenital cardiac failure	Feb-09
21217	64	2	0	0	BB1N.00	[M]Small cell-large cell carcinoma	Feb-09
21249	196	2	0	0	F232.00	Congenital quadriplegia	Feb-09
21327	310	7	0	0	B333500	Malignant neoplasm of skin of temple	Feb-09
21330	128	2	0	0	B180.00	Malignant neoplasm of retroperitoneum	Feb-09
21402	404	6	0	0	B602.00	Burkitt's lymphoma	Feb-09
21418	140	6	0	0	PKy6011	Cornelia de Lange syndrome	Feb-09
21447	14	0	0	0	BBV3.00	[M]Fibroblastic osteosarcoma	Feb-09
21463	272	2	0	0	BBgC.11	[M]Lymphocytic lymphoma NOS	Feb-09
21549	739	16	0	0	B627C00	Follicular non-Hodgkin's lymphoma	Feb-09
21590	352	11	0	0	B58y500	Secondary malignant neoplasm of prostate	Feb-09
21609	131	3	0	0	BB18.00	"[M]Carcinoma, undifferentiated type, NOS"	Feb-09
21620	86	0	0	0	B111.00	Malignant neoplasm of pylorus of stomach	Feb-09
21659	269	4	0	0	BB5Bz00	[M]Pancreatic adenoma or carcinoma NOS	Feb-09
21682	24	0	0	0	BBQ7500	"[M]Malignant teratoma, intermediate type"	Feb-09
21698	631	6	0	0	B221z00	Malignant neoplasm of main bronchus NOS	Feb-09
21715	297	5	0	0	Byu5011	[X]Mesothelioma of lung	Feb-09

For peer review only - http://bmjopen.bmj.com/site/about/guidelines.xhtml

21723	55	4	0	0	D201z00	Acquired aplastic anaemia NOS	Feb-09
21732	40	0	0	0	BBH1.00	[M]Myxosarcoma	Feb-09
21741	64	1	0	0	BB5f100	[M]Follicular adenocarcinoma NOS	Feb-09
21769	458	12	0	0	J625.11	[X] Liver failure	Feb-09
21770	836	71	0	0	BBPX.00	"[M]Mesothelioma, unspecified"	Feb-09
21786	1506	23	0	0	B471000	Seminoma of descended testis	Feb-09
21802	238	1	0	0	P1z..00	Spina bifida NOS	Feb-09
21833	854	4	0	0	BB91.11	[M]Duct carcinoma NOS	Feb-09
21847	356	2	0	0	BB5f111	[M]Follicular carcinoma	Feb-09
21852	184	34	0	0	G554200	Familial cardiomyopathy	Feb-09
21868	2570	18	0	0	BB02.00	"[M]Neoplasm, malignant"	Feb-09
21914	262	7	0	0	BB11.11	[M]Intraepithelial carcinoma NOS	Feb-09
22050	507	8	0	0	B691.00	Chronic myelomonocytic leukaemia	Feb-09
22071	8	0	0	0	BBr0111	[M]Blast cell leukaemia	Feb-09
22146	91	0	0	0	B581100	Secondary malignant neoplasm of bladder	Feb-09
22156	208	2	0	0	BB08.00	"[M]Malignant tumour, small cell type"	Feb-09
22158	88	1	0	0	B630000	"Malignant plasma cell neoplasm, extramedullary plasmacytoma"	Feb-09
22163	673	13	0	0	B134.11	Carcinoma of caecum	Feb-09
22174	10	1	0	0	F390z00	Congenital hereditary muscular dystrophy NOS	Feb-09
22187	2226	46	0	0	B150300	Hepatocellular carcinoma	Feb-09
22267	215	19	0	0	BB04.00	"[M]Neoplasm, malig, uncertain whether primary or metastatic"	Feb-09
22290	27	3	0	0	B313.00	Malignant neoplasm of connective and soft tissue of thorax	Feb-09
22441	28	1	0	0	B212.00	Malignant neoplasm of subglottis	Feb-09
22524	318	0	0	0	B58yz00	Secondary malignant neoplasm of other specified site NOS	Feb-09
22561	187	13	0	0	BBV4.00	[M]Telangiectatic osteosarcoma	Feb-09
22650	307	10	0	0	BBT1.11	[M]Angiosarcoma	Feb-09

For peer review only - http://bmjopen.bmj.com/site/about/guidelines.xhtml

22692	124	6	0	0	BBEG000	"[M]Acral lentiginous melanoma, malignant"	Feb-09
22728	168	1	0	0	SP08700	Acute graft-versus-host disease	Feb-09
22851	173	11	1	0	C375.00	Mucopolysaccharidosis	Feb-09
22893	485	8	0	0	B06..00	Malignant neoplasm of oropharynx	Feb-09
22894	150	3	0	0	B110100	Malignant neoplasm of cardio-oesophageal junction of stomach	Feb-09
22993	1078	20	0	0	G55z.00	Cardiomyopathy NOS	Feb-09
23083	888	11	0	0	BBbL.00	[M]Glioblastoma NOS	Feb-09
23320	53	3	0	0	N233011	Arthrogryposis multiplex congenita.	Feb-09
23380	162	19	0	0	B340000	Malignant neoplasm of nipple of female breast	Feb-09
23389	286	14	0	0	B200.00	Malignant neoplasm of nasal cavities	Feb-09
23399	919	7	0	0	B344.00	Malignant neoplasm of upper-outer quadrant of female breast	Feb-09
23415	97	9	0	0	F256100	Salaam attacks	Feb-09
23433	109	0	0	0	B161.00	Malignant neoplasm of extrahepatic bile ducts	Feb-09
23455	74	5	0	0	BBe1.12	[M]Von Recklinghausen's disease	Feb-09
23480	145	16	0	0	B335900	Malignant neoplasm of perianal skin	Feb-09
23566	40	1	0	0	Q490.00	Neonatal cardiac failure	Feb-09
23711	127	5	0	0	BBg1000	"[M]Malignant lymphoma, diffuse NOS"	Feb-09
23730	99	16	0	0	F202.00	Generalised multiple sclerosis	Feb-09
23775	223	20	0	0	J62y.12	Liver failure NOS	Feb-09
23861	57	5	0	0	B551100	Malignant neoplasm of chest wall NOS	Feb-09
23936	36	2	0	0	ZV10411	[V]Personal history of malignant neoplasm of cervix uteri	Feb-09
23945	544	22	0	0	PK80.00	Fetal alcohol syndrome	Feb-09
23951	43	0	0	0	A789200	HIV disease resulting in candidiasis	Feb-09
23960	17	1	0	0	PD03011	Potter's syndrome	Feb-09
24048	13	0	0	0	B180200	Malignant neoplasm of retrocaecal tissue	Feb-09

For peer review only - http://bmjopen.bmj.com/site/about/guidelines.xhtml

24293	1136	14	0	0	BB2B.00	"[M]Squamous cell carcinoma, metastatic NOS"	Feb-09
24301	46	1	0	0	B57..12	Secondary carcinoma of respiratory and/or digestive systems	Feb-09
24312	29	0	0	0	BB62.00	[M]Apocrine adenoma and adenocarcinomas	Feb-09
24370	983	54	1	0	B142.00	Malignant neoplasm of anal canal	Feb-09
24374	478	23	0	0	B0...11	"Carcinoma of lip, oral cavity and pharynx"	Feb-09
24375	693	37	0	0	B339.00	Dermatofibrosarcoma protuberans	Feb-09
24397	140	4	0	0	B061.00	Malignant neoplasm of tonsillar fossa	Feb-09
24437	830	29	0	0	PB5z.12	Short bowel syndrome	Feb-09
24511	89	6	0	0	BB09.00	"[M]Malignant tumour, giant cell type"	Feb-09
24539	55	2	0	0	BBV2.00	[M]Chondroblastic osteosarcoma	Feb-09
24551	49	2	0	0	BBE1.11	[M]Melanocarcinoma	Feb-09
24675	446	5	0	0	B07..00	Malignant neoplasm of nasopharynx	Feb-09
24852	100	0	0	0	B016.00	Malignant neoplasm of lingual tonsil	Feb-09
24924	59	2	0	0	BBD..00	[M]Paragangliomas and glomus tumours	Feb-09
25191	518	39	0	0	B68..00	Leukaemia of unspecified cell type	Feb-09
25245	13	2	0	0	B336400	Malignant neoplasm of skin of finger	Feb-09
25282	P203.11	68	0	3	0	Meningocele - cranial	Feb-09
25304	140	5	0	0	Q402300	Congenital toxoplasmosis	Feb-09
25306	196	6	0	0	PKyz511	Angelman syndrome	Feb-09
25429	37	0	0	0	F390300	Myotubular myopathy	Feb-09
25535	566	20	1	0	B150.00	Primary malignant neoplasm of liver	Feb-09
25597	237	2	0	0	PB61.00	Biliary atresia	Feb-09
25602	13	2	0	0	B326400	Malignant melanoma of finger	Feb-09
25641	149	7	0	0	BB5D513	[M]Liver cell carcinoma	Feb-09
25657	29	0	0	0	A752.00	Pfeiffer's disease	Feb-09
25695	171	3	0	0	SP08800	Chronic graft-versus-host disease	Feb-09
25835	45573	21	1	0	ZV76200	[V]Screening for malignant neoplasm of cervix	Feb-09

For peer review only - http://bmjopen.bmj.com/site/about/guidelines.xhtml

For peer review only

25886	2578	12	0	0	B222100	Malignant neoplasm of upper lobe of lung	Feb-09
25961	165	3	0	0	BB17.00	[M]Large cell carcinoma NOS	Feb-09
26034	125	0	0	0	B591.00	Other malignant neoplasm NOS	Feb-09
26111	237	4	0	0	M162800	Lymphomatoid papulosis	Feb-09
26120	186	6	0	0	BB5V.00	[M]Pituitary adenomas and carcinomas	Feb-09
26134	10	0	0	0	B064000	"Malignant neoplasm of epiglottis, free border"	Feb-09
26165	408	8	0	0	B211.00	Malignant neoplasm of supraglottis	Feb-09
26253	940	8	0	0	BB5R900	[M]Neuroendocrine carcinoma	Feb-09
26393	865	24	0	0	B152.00	Malignant neoplasm of liver unspecified	Feb-09
26413	100	3	0	0	BB1A.00	[M]Pleomorphic carcinoma	Feb-09
26448	4	0	0	0	B060000	Malignant neoplasm of faucial tonsil	Feb-09
26454	13	0	0	0	B45X.00	Malignant neoplasm/overlapping lesion/feml genital organs	Feb-09
26478	260	7	0	0	SN5y000	Malignant hyperpyrexia due to anaesthetic	Feb-09
26813	74	0	0	0	B21y.00	"Malignant neoplasm of larynx, other specified site"	Feb-09
26814	207	4	0	0	BB5D512	"[M]Hepatoma, malignant"	Feb-09
26848	20	1	0	0	BB5S.00	[M]Respiratory tract adenomas and adenocarcinomas	Feb-09
26853	342	14	0	0	B340.00	Malignant neoplasm of nipple and areola of female breast	Feb-09
26858	137	5	0	0	BB5C.00	[M]Gastrinoma and carcinomas	Feb-09
27280	45	4	0	0	PKy0.12	Prader-Willi syndrome	Feb-09
27330	61	7	0	0	B624.00	Leukaemic reticuloendotheliosis	Feb-09
27363	19	0	0	0	BBd2.00	"[M]Meningioma, malignant"	Feb-09
27370	172	5	0	0	B333.00	Malignant neoplasm skin of other and unspecified parts face	Feb-09
27377	494	62	0	0	F152200	Progressive bulbar palsy	Feb-09
27391	311	20	0	0	B576100	Secondary malignant neoplasm of peritoneum	Feb-09

For peer review only - http://bmjopen.bmj.com/site/about/guidelines.xhtml

27416	197	8	0	0	B601.00	Lymphosarcoma	Feb-09
27439	335	23	0	0	BBTA.00	[M]Kaposi's sarcoma	Feb-09
27449	48	2	0	0	B554.00	Malignant neoplasm of upper limb NOS	Feb-09
27458	129	18	0	0	B661.00	Chronic monocytic leukaemia	Feb-09
27475	195	0	0	0	13M3.00	Sudden infant death	Feb-09
27483	149	6	0	0	B240.00	Malignant neoplasm of thymus	Feb-09
27509	95	0	0	0	BBP1.00	"[M]Mesothelioma, malignant"	Feb-09
27520	65	0	0	0	B651z00	Chronic myeloid leukaemia NOS	Feb-09
27528	12	0	0	0	B303.00	"Malignant neoplasm of ribs, sternum and clavicle"	Feb-09
27540	46	0	0	0	B4A1000	Malignant neoplasm of renal calyces	Feb-09
27562	5	0	0	0	BBk0.12	[M]Follicular lymphosarcoma NOS	Feb-09
27617	7	0	0	0	B45y000	Malignant neoplasm of overlapping lesion of vulva	Feb-09
27641	215	1	0	0	A789300	HIV disease resulting in Pneumocystis carinii pneumonia	Feb-09
27651	183	2	0	0	B58..11	Secondary carcinoma of other specified sites	Feb-09
27653	149	1	0	0	BBbz.00	[M]Glioma NOS	Feb-09
27664	253	2	0	0	B65y100	Acute promyelocytic leukaemia	Feb-09
27683	112	11	0	0	G558100	Cardiomyopathy in myotonic dystrophy	Feb-09
27686	P203.00	89	0	0	0	Meningocele - cerebral	Feb-09
27715	15	0	0	0	B242.00	Malignant neoplasm of anterior mediastinum	Feb-09
27728	202	3	0	0	BB90.00	"[M]Intraductal carcinoma, noninfiltrating NOS"	Feb-09
27744	969	36	0	0	BBbQ.00	[M]Oligodendroglioma NOS	Feb-09
27748	131	4	0	0	BBbB.11	[M]Astrocytic glioma	Feb-09
27790	865	59	0	0	B641.11	Chronic lymphatic leukaemia	Feb-09
27846	46	0	0	0	BBbF.00	[M]Fibrillary astrocytoma	Feb-09
27849	12	0	0	0	BB5U200	[M]Villous adenocarcinoma	Feb-09
27853	45	0	0	0	A789500	HIV disease resulting in Kaposi's sarcoma	Feb-09

For peer review only - http://bmjopen.bmj.com/site/about/guidelines.xhtml

27855	1087	13	1	0	B140.00	Malignant neoplasm of rectosigmoid junction	Feb-09
27897	267	2	0	0	B143.00	Malignant neoplasm of anus unspecified	Feb-09
27931	66	4	0	0	B33z000	Kaposi's sarcoma of skin	Feb-09
27965	23	1	0	0	BBv2.00	[M]AngiocentricT-cell lymphoma	Feb-09
28003	144	2	0	0	B420.00	Choriocarcinoma	Feb-09
28069	43	2	0	0	B505.00	Malignant neoplasm of retina	Feb-09
28148	204	0	0	0	B540.00	Malignant neoplasm of adrenal gland	Feb-09
28163	2504	28	0	0	B13z.00	Malignant neoplasm of colon NOS	Feb-09
28178	863	71	0	0	BBa0.00	[M]Craniopharyngioma	Feb-09
28210	33	2	0	0	F391300	Other limb-girdle muscular dystrophy	Feb-09
28241	155	0	0	0	B496.00	Malignant neoplasm of ureteric orifice	Feb-09
28272	472	14	0	0	BB57.00	"[M]Adenocarcinoma, intestinal type"	Feb-09
28291	165	19	0	0	BB69.00	[M]Sebaceous adenoma and adenocarcinoma	Feb-09
28311	1295	7	0	0	B41z.00	Malignant neoplasm of cervix uteri NOS	Feb-09
28344	60	1	0	0	BBb3.12	[M]Subependymal astrocytoma NOS	Feb-09
28355	222	4	0	0	7904	Other correction of transposition of great vessels	Feb-09
28388	189	1	0	0	BB5jz00	[M]Endometrioid adenoma or carcinoma NOS	Feb-09
28451	28	0	0	0	B08z.00	Malignant neoplasm of hypopharynx NOS	Feb-09
28556	815	48	0	0	B32z.00	Malignant melanoma of skin NOS	Feb-09
28559	18	0	0	0	B055z00	Malignant neoplasm of palate NOS	Feb-09
28599	484	21	0	0	BBJ1.00	[M]Liposarcoma NOS	Feb-09
28625	72	0	0	0	BB71.00	[M]Mucoepidermoid carcinoma	Feb-09
28628	72	1	0	0	BBJ3.00	"[M]Liposarcoma, well differentiated type"	Feb-09
28639	119	1	0	0	B627000	Follicular non-Hodgkin's small cleaved cell lymphoma	Feb-09
28663	26	3	0	0	PKy9200	Menke's syndrome	Feb-09
28665	42	0	0	0	B07z.00	Malignant neoplasm of nasopharynx NOS	Feb-09

For peer review only - http://bmjopen.bmj.com/site/about/guidelines.xhtml

28727	138	1	0	0	B575000	Secondary malignant neoplasm of colon	Feb-09
28824	95	1	0	0	P240200	Schizencephaly	Feb-09
28836	523	37	0	0	BBc9.00	[M]Retinoblastomas	Feb-09
28881	8	1	0	0	ZV10513	[V]Personal history of malignant neoplasm of kidney	Feb-09
28919	44	2	0	0	B521.00	Malignant neoplasm of cerebral meninges	Feb-09
28941	88	0	0	0	BBQ3.00	[M]Embryonal carcinoma NOS	Feb-09
28955	F392100	319	0	23	0	Myotonia congenita (Thomsen's disease)	Feb-09
29008	51	0	0	0	BB5W111	[M]Hurthle cell adenocarcinoma	Feb-09
29160	30	2	0	0	B313000	Malignant neoplasm of connective and soft tissue of axilla	Feb-09
29178	515	1	0	0	B614.00	"Hodgkin's disease, nodular sclerosis"	Feb-09
29283	11	0	0	0	B2zy.00	Malignant neoplasm of other site of respiratory tract	Feb-09
29284	82	9	0	0	ZV10112	[V]Personal history of malignant neoplasm of lung	Feb-09
29335	117	2	0	0	BBr2700	[M]Adult T-cell leukaemia/lymphoma	Feb-09
29337	80	2	0	0	BBVA.00	[M] Small cell osteosarcoma	Feb-09
29462	1773	8	0	0	B4Az.00	Malignant neoplasm of kidney or urinary organs NOS	Feb-09
29580	16	1	0	0	BBCA.00	[M]Sertoli cell carcinoma	Feb-09
29787	244	19	0	0	BB2C.00	"[M]Squamous cell carcinoma, keratinising type NOS"	Feb-09
29826	266	1	0	0	B342.00	Malignant neoplasm of upper-inner quadrant of female breast	Feb-09
29876	7	0	0	0	B613z00	"Hodgkin's, lymphocytic-histiocytic predominance NOS"	Feb-09
29945	93	0	0	0	BBR4.00	"[M]Malignant teratoma, trophoblastic"	Feb-09
30119	8	0	0	0	C1zy100	Progeria	Feb-09
30165	3	1	0	0	B18y200	Malignant neoplasm of mesorectum	Feb-09

For peer review only - http://bmjopen.bmj.com/site/about/guidelines.xhtml

30189	81	0	0	0	BB91000	[M]Intraductal papillary adenocarcinoma with invasion	Feb-09
30273	381	13	0	0	BBbG.00	[M]Pilocytic astrocytoma	Feb-09
30283	1419	10	0	0	7B2C700	Intravesical install chemotherapeutic agent for malignancy	Feb-09
30322	73	9	0	0	ZV10500	[V]Personal history of malignant neoplasm of urinary organ	Feb-09
30402	77	6	0	0	B050.11	Malignant neoplasm of buccal mucosa	Feb-09
30416	12	0	0	0	BB82111	[M]Colloid adenocarcinoma	Feb-09
30526	47	0	0	0	Byu5100	"[X]Mesothelioma, unspecified"	Feb-09
30537	15	0	0	0	F373.00	Polyneuropathy in malignant disease	Feb-09
30543	120	1	0	0	B335200	Malignant neoplasm of skin of breast	Feb-09
30576	395	6	0	0	B333300	Malignant neoplasm of skin of forehead	Feb-09
30577	80	2	0	0	B336200	Malignant neoplasm of skin of fore-arm	Feb-09
30632	44	1	0	0	B67z.00	Other specified leukaemia NOS	Feb-09
30645	431	7	0	0	B333000	"Malignant neoplasm of skin of cheek, external"	Feb-09
30646	27	0	0	0	B6y..00	Malignant neoplasm lymphatic or haematopoietic tissue OS	Feb-09
30700	2796	53	0	0	B10z.00	Malignant neoplasm of oesophagus NOS	Feb-09
30747	252	0	0	0	B336.00	Malignant neoplasm of skin of upper limb and shoulder	Feb-09
30988	105	1	0	0	BB1M.00	"[M]Small cell carcinoma, intermediate cell"	Feb-09
31004	48	1	0	0	BB2G.00	[M]Adenoid squamous cell carcinoma	Feb-09
31026	189	4	0	0	BBF3.00	[M]Spindle cell sarcoma	Feb-09
31042	379	23	0	0	Eu84200	[X]Rett's syndrome	Feb-09
31090	20	3	0	0	BBGP.00	[M]Pigmented dermatofibrosarcoma protuberans	Feb-09
31102	4275	30	0	0	B49z.00	Malignant neoplasm of urinary bladder NOS	Feb-09
31112	250	12	0	0	P732.00	Pulmonary artery atresia	Feb-09

For peer review only - http://bmjopen.bmj.com/site/about/guidelines.xhtml

31188	771	6	0	0	B224.00	"Malignant neoplasm of lower lobe, bronchus or lung"	Feb-09
31210	128	3	0	0	B150100	Hepatoblastoma of liver	Feb-09
31268	293	4	0	0	B223.00	"Malignant neoplasm of middle lobe, bronchus or lung"	Feb-09
31323	158	15	0	0	BBG1.00	[M]Fibrosarcoma NOS	Feb-09
31324	38	1	0	0	B626800	Mast cell malignancy of lymph nodes of multiple sites	Feb-09
31364	118	6	1	0	B050.00	Malignant neoplasm of cheek mucosa	Feb-09
31393	361	14	0	0	B160.11	Carcinoma gallbladder	Feb-09
31399	74	3	0	0	B555.00	Malignant neoplasm of lower limb NOS	Feb-09
31418	455	35	0	0	N233000	Arthrogryposis	Feb-09
31421	230	18	0	0	BBK3100	[M]Rhabdomyosarcoma NOS	Feb-09
31492	335	5	0	0	BBm9.00	[M] Monocytoid B-cell lymphoma	Feb-09
31537	9	0	0	0	BBj1100	"[M]Hodgkin,s disease, lymphocytic predominance, nodular"	Feb-09
31541	16	3	0	0	C392300	Severe combined immunodefiency with reticular dysgenesis	Feb-09
31546	195	2	0	0	B341.00	Malignant neoplasm of central part of female breast	Feb-09
31573	172	1	0	0	B23..00	Malignant neoplasm of pleura	Feb-09
31574	223	18	0	0	BBb0.00	"[M]Glioma, malignant"	Feb-09
31576	73	1	0	0	B627B00	Other types of follicular non-Hodgkin's lymphoma	Feb-09
31586	110	3	0	0	B64y100	Prolymphocytic leukaemia	Feb-09
31608	45	0	0	0	B43y.00	Malignant neoplasm of other site of uterine body	Feb-09
31609	24	2	0	0	BBC4.00	"[M]Granulosa cell tumour, malignant"	Feb-09
31629	198	16	0	0	BBc6.00	[M]Ganglioglioma	Feb-09
31663	20	5	0	0	2826	O/E - salaam attack	Feb-09
31673	5	0	0	0	BBX1.12	"[M]Osteoclastoma, malignant"	Feb-09

For peer review only - http://bmjopen.bmj.com/site/about/guidelines.xhtml

31700	456	3	0	0	B222000	Malignant neoplasm of upper lobe bronchus	Feb-09
31701	162	8	0	0	B651.11	Chronic granulocytic leukaemia	Feb-09
31726	6	0	0	0	BBgM.00	"[M]Malignant lymphoma, small cleaved cell, diffuse"	Feb-09
31741	2	0	0	0	BBj6200	"[M]Hodgkin,s disease, nodular sclerosis, lymphocytic deplet"	Feb-09
31749	18	0	0	0	BBv0.00	[M]Monocytoid B-cell lymphoma	Feb-09
31750	106	3	0	0	BBr0300	[M]Chronic leukaemia NOS	Feb-09
31755	79	5	0	0	G240.00	Secondary malignant hypertension	Feb-09
31772	149	2	0	0	BBGM.00	[M]Dermatofibrosarcoma NOS	Feb-09
31775	P115.00	31	0	0	1	Myelocele	Feb-09
31794	661	3	0	0	B627W00	Unspecified B-cell non-Hodgkin's lymphoma	Feb-09
31818	33	4	0	0	BBK2.00	[M]Myoma and myosarcoma	Feb-09
32022	346	8	0	0	B110.00	Malignant neoplasm of cardia of stomach	Feb-09
32024	15	0	0	0	B030.00	Malignant neoplasm of upper gum	Feb-09
32174	79	1	0	0	B202.00	Malignant neoplasm of maxillary sinus	Feb-09
32213	2	1	0	0	BB0A.00	"[M]Malignant tumour, fusiform cell type"	Feb-09
32240	7	0	11	0	4M22.00	Lymphoma stage III	Feb-09
32246	27	6	0	0	ZV10111	[V]Personal history of malignant neoplasm of bronchus	Feb-09
32294	1	0	0	0	BB5B500	"[M]Glucagonoma, malignant"	Feb-09
32351	22	1	46	0	44a4.00	Squamous cell carcinoma antigen level	Feb-09
32357	137	7	0	0	BBc0.00	[M]Ganglioneuromatous neoplasms	Feb-09
32362	75	1	0	0	B113.00	Malignant neoplasm of fundus of stomach	Feb-09
32372	46	1	0	0	B302100	Malignant neoplasm of thoracic vertebra	Feb-09
32464	188	17	0	0	C300000	Cystinosis	Feb-09
32472	46	0	0	0	BB9H.00	[M]Inflammatory carcinoma	Feb-09

For peer review only

32508	53	0	0	0	P738.00	Atresia of pulmonary artery with septal defect	Feb-09
32603	503	3	0	0	PJyy400	Fragile X syndrome	Feb-09
32641	528	15	0	0	BB5RA00	[M]Merkel cell carcinoma	Feb-09
32768	566	6	0	0	B325100	Malignant melanoma of breast	Feb-09
32874	54	0	0	0	C362z11	Acidaemia NOS	Feb-09
32919	169	8	0	0	SN5y011	Malignant hyperthermia due to anaesthesia	Feb-09
32955	100	1	0	0	B41y.00	Malignant neoplasm of other site of cervix	Feb-09
33259	58	11	0	0	ZV76600	[V]Screening for malignant neoplasm of skin	Feb-09
33271	286	35	0	0	B332200	Malignant neoplasm of pinna NEC	Feb-09
33333	168	2	0	0	B62..00	Other malignant neoplasm of lymphoid and histiocytic tissue	Feb-09
33334	35	2	0	0	F150.00	Werdnig - Hoffmann disease	Feb-09
33344	32	0	0	0	B65z.00	Myeloid leukaemia NOS	Feb-09
33353	101	2	0	0	F11y000	Reye's syndrome	Feb-09
33388	15	0	0	0	B071000	Malignant neoplasm of adenoid	Feb-09
33390	63	6	0	0	C375112	Hurler's syndrome	Feb-09
33444	316	4	0	0	B221100	Malignant neoplasm of hilus of lung	Feb-09
33522	69	1	0	0	PKy6300	Smith - Lemli - Opitz syndrome	Feb-09
33617	429	4	0	0	B43z.00	Malignant neoplasm of body of uterus NOS	Feb-09
33636	104	2	0	0	BBQ7200	"[M]Teratoma, malignant, NOS"	Feb-09
33642	209	2	0	0	PJ2..00	Edward's syndrome - trisomy 18	Feb-09
33682	237	4	0	0	B337400	Malignant neoplasm of skin of lower leg	Feb-09
33775	354	3	0	0	BB5J.00	[M]Adenoid cystic carcinoma	Feb-09
33811	17	1	0	0	PG4D.00	Metaphyseal chondrodysplasia	Feb-09
33833	200	2	0	0	B301.00	Malignant neoplasm of mandible	Feb-09
33843	377	4	0	0	B583.00	Secondary malignant neoplasm of brain and spinal cord	Feb-09

For peer review only - http://bmjopen.bmj.com/site/about/guidelines.xhtml

33869	118	0	0	0	BBgR.00	"[M]Malignant lymphoma, large cell, diffuse NOS"	Feb-09
33871	74	4	0	0	B122.00	Malignant neoplasm of ileum	Feb-09
33897	131	6	0	0	BB4z.00	[M]Transitional cell papilloma or carcinoma NOS	Feb-09
33939	199	0	0	0	P21z.00	Microcephalus NOS	Feb-09
33948	84	1	0	0	PJ32.11	Wolff - Hirschorn syndrome	Feb-09
33997	115	1	0	0	B332000	Malignant neoplasm of skin of auricle (ear)	Feb-09
34000	97	1	0	0	BB80100	[M]Cystadenocarcinoma NOS	Feb-09
34012	208	4	0	0	B08..00	Malignant neoplasm of hypopharynx	Feb-09
34015	182	2	0	0	BB5S200	[M]Bronchiolo-alveolar adenocarcinoma	Feb-09
34030	173	3	0	0	BBL0.00	[M]Endometrial stromal sarcoma	Feb-09
34089	6	0	0	0	B62y400	Malignant lymphoma NOS of lymph nodes of axilla and arm	Feb-09
34096	71	2	0	0	BB5b.00	[M]Granular cell carcinoma	Feb-09
34110	100	3	0	0	BB5R100	"[M]Carcinoid tumour, malignant"	Feb-09
34145	5	0	0	0	B58y600	Secondary malignant neoplasm of testis	Feb-09
34161	61	1	0	0	P225.00	Holoprosencephaly	Feb-09
34252	61	1	0	0	BBb0.12	[M]Gliosarcoma	Feb-09
34259	59	2	0	0	B325300	Malignant melanoma of groin	Feb-09
34269	13	0	0	0	BB69100	[M]Sebaceous adenocarcinoma	Feb-09
34352	121	1	0	0	BBgG.12	[M]Lymphoblastic lymphoma NOS	Feb-09
34388	1117	9	0	0	B17z.00	Malignant neoplasm of pancreas NOS	Feb-09
34395	164	8	0	0	BB24.00	[M]Verrucous carcinoma NOS	Feb-09
34409	4	0	0	0	B010000	Malignant neoplasm of base of tongue dorsal surface	Feb-09
34451	489	11	0	0	B31..00	Malignant neoplasm of connective and other soft tissue	Feb-09
34692	24	3	0	0	B68y.00	Other leukaemia of unspecified cell type	Feb-09
34704	11	0	0	0	F390100	Central core disease	Feb-09
34713	88	2	0	0	BBc0000	[M]Ganglioneuroma	Feb-09

For peer review only - http://bmjopen.bmj.com/site/about/guidelines.xhtml

For peer review only

34742	73	2	0	0	B23z.00	Malignant neoplasm of pleura NOS	Feb-09
34754	46	0	0	0	D200100	Fanconi's familial refractory anaemia	Feb-09
34763	488	16	0	0	BBbT.00	[M]Medulloblastoma NOS	Feb-09
34792	262	7	0	0	F250500	Lennox-Gastaut syndrome	Feb-09
34878	2	0	0	0	B308300	Malignant neoplasm of medial cuneiform	Feb-09
34879	1	0	0	0	BB5J.11	[M]Cylindroid adenocarcinoma	Feb-09
34891	32	0	0	0	BBF2.00	[M]Sarcomatosis NOS	Feb-09
34921	69	1	0	0	P22y111	Joubert syndrome	Feb-09
34950	84	1	0	0	P223.11	Lissencephaly	Feb-09
34984	131	7	0	0	BB80.00	[M]Cystadenoma and carcinoma	Feb-09
35034	101	4	0	0	BBGJ.11	[M]Fibroxanthosarcoma	Feb-09
35039	19	1	0	0	B163.00	"Malignant neoplasm, overlapping lesion of biliary tract"	Feb-09
35071	141	0	0	0	BBQB.00	[M]Mixed germ cell tumour	Feb-09
35113	158	1	0	0	Byu9.00	[X]Malignant neoplasm of urinary tract	Feb-09
35180	189	1	0	0	Byu1.00	[X]Malignant neoplasm of digestive organs	Feb-09
35186	35	1	0	0	ByuC.00	"[X]Malignant neoplasm of ill-defined, secondary and unspeci"	Feb-09
35223	10	0	0	0	BBQ1100	[M]Spermatocytic seminoma	Feb-09
35285	139	0	1	0	ByuA.00	"[X]Malignant neoplasm of eye, brain and other parts of cent"	Feb-09
35325	149	0	0	0	Byu2.00	[X]Malignant neoplasm of respiratory and intrathoracic orga	Feb-09
35348	86	1	0	0	BB5T100	[M]Papillary adenocarcinoma NOS	Feb-09
35357	961	13	0	0	B14..00	"Malignant neoplasm of rectum, rectosigmoid junction and anus"	Feb-09
35364	29	0	0	0	B576000	Secondary malignant neoplasm of retroperitoneum	Feb-09
35420	102	2	0	0	PH3y900	Epidermolysis bullosa dystrophica	Feb-09
35457	251	8	0	0	BB35.00	[M]Basosquamous carcinoma	Feb-09
35467	133	1	0	0	BB5az00	[M]Renal adenoma or carcinoma NOS	Feb-09

For peer review only - http://bmjopen.bmj.com/site/about/guidelines.xhtml

35474	34	0	0	0	BB1C.00	[M]Giant cell carcinoma	Feb-09
35535	138	6	0	0	B173.00	Malignant neoplasm of pancreatic duct	Feb-09
35540	434	10	0	0	F240100	Spastic tetraplegia	Feb-09
35649	54	4	1	0	C31y200	Oxalosis	Feb-09
35665	112	5	0	0	PJ1..00	Patau's syndrome - trisomy 13	Feb-09
35697	45	0	0	0	BBr6.00	[M]Myeloid leukaemias	Feb-09
35747	3	0	0	0	BB5j100	"[M]Endometrioid adenoma, borderline malignancy"	Feb-09
35795	6	0	0	0	B174.00	Malignant neoplasm of Islets of Langerhans	Feb-09
35875	39	5	0	0	B66..00	Monocytic leukaemia	Feb-09
35963	160	1	0	0	B492.00	Malignant neoplasm of lateral wall of urinary bladder	Feb-09
35975	146	6	0	0	BB5z.00	[M]Adenoma or adenocarcinoma NOS	Feb-09
35999	12	0	0	0	B582200	Secondary malignant neoplasm of skin of neck	Feb-09
36031	33	3	0	0	BB5D.00	[M]Hepatobiliary tract adenomas and carcinomas	Feb-09
36114	615	53	0	0	BBg1.00	[M]Malignant lymphoma NOS	Feb-09
36147	553	5	0	0	B153.00	Secondary malignant neoplasm of liver	Feb-09
36161	36	3	0	0	B012.00	"Malignant neoplasm of tongue, tip and lateral border"	Feb-09
36221	49	0	0	0	BB5Sz00	[M]Respiratory tract adenoma or adenocarcinoma NOS	Feb-09
36242	81	0	0	0	BBM7.11	"[M]Cystosarcoma phyllodes, benign"	Feb-09
36286	9	0	0	0	BB5Lz00	[M]Adenomatous or adenocarcinomatous polyp NOS	Feb-09
36325	77	1	0	0	B470300	Teratoma of undescended testis	Feb-09
36371	95	1	0	0	B225.00	Malignant neoplasm of overlapping lesion of bronchus & lung	Feb-09
36401	159	2	0	0	B587.00	Secondary malignant neoplasm of adrenal gland	Feb-09

For peer review only - http://bmjopen.bmj.com/site/about/guidelines.xhtml

36433	F152000	133	0	8	0	Amyotrophic lateral sclerosis	Feb-09
36495	76	3	0	0	B161211	Carcinoma common bile duct	Feb-09
36530	85	3	0	0	BB5S211	[M]Alveolar cell carcinoma	Feb-09
36566	16	0	0	0	F390400	Nemaline body disease	Feb-09
36567	47	0	0	0	PKy6100	Cockayne syndrome	Feb-09
36622	97	0	0	0	C370111	Meconium ileus in cystic fibrosis	Feb-09
36693	75	4	0	0	ZV10600	[V]Personal history of leukaemia	Feb-09
36716	61	1	0	0	B04z.00	Malignant neoplasm of floor of mouth NOS	Feb-09
36731	129	3	0	0	B331000	Malignant neoplasm of canthus	Feb-09
36785	217	28	0	0	BBe1.11	[M]Multiple neurofibromatosis	Feb-09
36839	123	3	0	0	PFy1.00	Larsen's syndrome	Feb-09
36870	48	0	0	0	BBL7111	[M]Adenosarcoma	Feb-09
36876	1	0	0	0	BB5V311	[M]Eosinophil carcinoma	Feb-09
36882	137	3	0	0	BB5X.00	[M]Clear cell adenomas and adenocarcinomas	Feb-09
36899	31	2	0	0	B327800	Malignant melanoma of toe	Feb-09
36949	55	0	0	0	B49y.00	Malignant neoplasm of other site of urinary bladder	Feb-09
37006	31	1	0	0	A789000	HIV disease resulting in mycobacterial infection	Feb-09
37014	180	9	0	0	Eu02200	[X]Dementia in Huntington's disease	Feb-09
37016	43	1	0	0	B33..14	Malignant neoplasm of sebaceous gland	Feb-09
37096	6	0	0	0	B015.00	"Malignant neoplasm of tongue, junctional zone"	Feb-09
37112	7	0	0	0	B6...11	Malignant neoplasm of histiocytic tissue	Feb-09
37137	173	3	0	0	BB51100	[M]Adenocarcinoma in situ in tubulovillous adenoma	Feb-09
37165	339	16	0	0	B334000	Malignant neoplasm of scalp	Feb-09
37182	78	3	0	0	B63..00	Multiple myeloma and immunoproliferative neoplasms	Feb-09
37272	129	1	0	0	B67..00	Other specified leukaemia	Feb-09

For peer review only - http://bmjopen.bmj.com/site/about/guidelines.xhtml

37306	255	18	0	0	ZV10415	[V]Personal history of malignant neoplasm of prostate	Feb-09
37328	287	6	0	0	B450.00	Malignant neoplasm of vagina	Feb-09
37354	74	0	0	0	BB5X100	[M]Clear cell adenocarcinoma NOS	Feb-09
37410	61	0	0	0	BBr2100	[M]Acute lymphoid leukaemia	Feb-09
37461	44	0	0	0	B64y200	Adult T-cell leukaemia	Feb-09
37473	31	1	1	0	BBbW.00	[M]Cerebellar sarcoma NOS	Feb-09
37477	48	2	0	0	BBe7.11	"[M]Schwannoma, malignant"	Feb-09
37510	20	0	0	0	BBLG.00	[M]Carcinoma in pleomorphic adenoma	Feb-09
37516	32	1	0	0	B054.00	Malignant neoplasm of uvula	Feb-09
37539	115	8	0	0	D2...00	Aplastic and other anaemias	Feb-09
37542	2	0	0	0	BBQ7300	[M]Teratocarcinoma	Feb-09
37549	8	0	0	0	B05z000	Kaposi's sarcoma of palate	Feb-09
37553	17	1	0	0	B007.00	"Malignant neoplasm of lip, unspecified"	Feb-09
37590	88	4	0	0	B052.00	Malignant neoplasm of hard palate	Feb-09
37618	70	3	0	0	B551000	Malignant neoplasm of axilla NOS	Feb-09
37680	135	0	0	0	BBGF.00	"[M]Fibrous histiocytoma, malignant"	Feb-09
37688	27	0	0	0	BBA2.00	[M]Acinar cell carcinoma	Feb-09
37723	48	1	0	0	BBr6011	[M]Granulocytic leukaemia NOS	Feb-09
37724	58	0	0	0	B056.00	Malignant neoplasm of retromolar area	Feb-09
37793	30	0	89	0	4M4..00	FIGO staging of gynaecological malignancy	Feb-09
37805	22	0	0	0	B213100	Malignant neoplasm of cricoid cartilage	Feb-09
37810	22	0	0	0	B220z00	Malignant neoplasm of trachea NOS	Feb-09
37842	61	2	0	0	B303000	Malignant neoplasm of rib	Feb-09
37859	37	0	0	0	B110z00	Malignant neoplasm of cardia of stomach NOS	Feb-09
37872	376	6	0	0	B327400	Malignant melanoma of lower leg	Feb-09
37916	32	0	0	0	B05y.00	Malignant neoplasm of other specified mouth parts	Feb-09
37940	58	1	0	0	B072000	Malignant neoplasm of pharyngeal recess	Feb-09

For peer review only - http://bmjopen.bmj.com/site/about/guidelines.xhtml

37969	118	3	0	0	B335100	"Malignant neoplasm of skin of chest, excluding breast"	Feb-09
38286	72	3	0	0	A411.00	Jakob-Creutzfeldt disease	Feb-09
38331	21	0	0	0	B64yz00	Other lymphoid leukaemia NOS	Feb-09
38411	14	0	0	0	7904z00	Other correction of transposition of great vessels NOS	Feb-09
38442	35	0	0	0	BB81200	"[M]Serous cystadenocarcinoma, NOS"	Feb-09
38454	193	4	0	0	BB48.00	[M]Basaloid carcinoma	Feb-09
38475	56	1	0	0	B34yz00	Malignant neoplasm of other site of female breast NOS	Feb-09
38479	77	3	0	0	PFy0.00	Arthrogryposis multiplex congenita	Feb-09
38481	10	0	0	0	BBTK.00	"[M]Epithelioid haemangioendothelioma, malignant"	Feb-09
38488	3	0	0	0	B013z00	Malignant neoplasm of ventral tongue surface NOS	Feb-09
38510	1032	21	0	0	B47z.00	Malignant neoplasm of testis NOS	Feb-09
38551	36	0	0	0	BBb1.00	[M]Gliomatosis cerebri	Feb-09
38575	8	1	0	0	BB62100	[M]Apocrine adenocarcinoma	Feb-09
38608	136	2	0	0	L250.11	Suspect fetal anencephaly	Feb-09
38685	115	1	0	0	BB5fz00	[M]Thyroid adenoma or adenocarcinoma NOS	Feb-09
38689	245	2	0	0	B325.00	Malignant melanoma of trunk (excluding scrotum)	Feb-09
38736	80	0	0	0	B5y..00	Malignant neoplasm of other and unspecified site OS	Feb-09
38756	5	0	0	0	BBP9.00	[M]Cystic mesothelioma	Feb-09
38770	5	0	0	0	BBB7.00	[M]Epithelial-myoepithelial carcinoma	Feb-09
38862	47	1	0	0	B490.00	Malignant neoplasm of trigone of urinary bladder	Feb-09
38914	57	1	0	0	B64z.00	Lymphoid leukaemia NOS	Feb-09
38918	148	1	0	0	B583100	Secondary malignant neoplasm of spinal cord	Feb-09

For peer review only - http://bmjopen.bmj.com/site/about/guidelines.xhtml

38931	50	0	0	0	B4y..00	Malignant neoplasm of genitourinary organ OS	Feb-09
38938	28	1	0	0	B306z00	"Malignant neoplasm of pelvis, sacrum or coccyx NOS"	Feb-09
38939	36	0	0	0	B613.00	"Hodgkin's disease, lymphocytic-histiocytic predominance"	Feb-09
38961	408	2	0	0	B22y.00	Malignant neoplasm of other sites of bronchus or lung	Feb-09
38978	50	0	0	0	B15z.00	Malignant neoplasm of liver and intrahepatic bile ducts NOS	Feb-09
39017	29	1	0	0	PJ2z.00	Edward's syndrome NOS	Feb-09
39023	130	0	0	0	F256.12	West syndrome	Feb-09
39027	103	0	0	0	ByuC000	[X]Malignant neoplasm of other specified sites	Feb-09
39038	88	0	0	0	BB85.00	[M]Signet ring carcinoma	Feb-09
39084	402	10	0	0	B0z2.00	Malignant neoplasm of laryngopharynx	Feb-09
39088	35	0	0	0	B514.00	Malignant neoplasm of occipital lobe	Feb-09
39121	13	1	0	0	BBc0100	[M]Ganglioneuroblastoma	Feb-09
39148	15	0	0	0	BB5Mz00	[M]Tubular adenoma or adenocarcinoma NOS	Feb-09
39187	110	4	0	0	B631.00	Plasma cell leukaemia	Feb-09
39312	28	0	0	0	BBM8.00	[M]Cystosarcoma phyllodes NOS	Feb-09
39386	8	0	0	0	BBb2.11	[M]Mixed glioma	Feb-09
39388	50	0	0	0	BBcC.11	[M]Olfactory neuroblastoma	Feb-09
39413	72	0	0	0	B18y500	Malignant neoplasm of pelvic peritoneum	Feb-09
39430	476	6	0	0	B0zz.00	"Malignant neoplasm of lip, oral cavity and pharynx NOS"	Feb-09
39522	60	4	0	0	BBV..00	[M]Osteomas and osteosarcomas	Feb-09
39554	21	0	0	0	B063.00	Malignant neoplasm of vallecula	Feb-09
39590	3	1	0	0	B206.00	"Malignant neoplasm, overlapping lesion of accessory sinuses"	Feb-09
39629	16	0	0	0	B653100	Granulocytic sarcoma	Feb-09

For peer review only - http://bmjopen.bmj.com/site/about/guidelines.xhtml

39760	129	3	0	0	BB91100	[M]Infiltrating duct and lobular carcinoma	Feb-09
39798	117	1	0	0	B627X00	"Diffuse non-Hodgkin's lymphoma, unspecified"	Feb-09
39870	131	1	0	0	B172.00	Malignant neoplasm of tail of pancreas	Feb-09
39875	46	0	0	0	BB5Nz00	[M]Adenomatous or adenocarcinomatous polyps of the colon NOS	Feb-09
39878	7	0	0	0	B327300	Malignant melanoma of popliteal fossa area	Feb-09
39897	102	1	0	0	B081.00	Malignant neoplasm of pyriform sinus	Feb-09
39899	34	5	0	0	B542100	Malignant neoplasm of craniopharyngeal duct	Feb-09
39906	4	0	0	0	BBgE.00	"[M]Malignant lymphoma, centrocytic"	Feb-09
39923	293	1	0	0	B223100	Malignant neoplasm of middle lobe of lung	Feb-09
39945	86	0	0	0	J600011	Acute liver failure	Feb-09
40014	112	2	0	0	B310100	Malignant neoplasm of soft tissue of face	Feb-09
40240	291	11	0	0	BB5D500	[M]Hepatocellular carcinoma NOS	Feb-09
40292	193	1	0	0	B053.00	Malignant neoplasm of soft palate	Feb-09
40344	32	0	0	0	F200.00	Multiple sclerosis of the brain stem	Feb-09
40359	33	1	0	0	BB94.00	[M]Juvenile breast carcinoma	Feb-09
40420	6	0	0	0	BBr0.00	[M]Leukaemias unspecified	Feb-09
40437	20	0	0	0	B50y.00	Malignant neoplasm of other specified site of eye	Feb-09
40438	40	2	0	0	BB5D111	[M]Bile duct carcinoma	Feb-09
40443	19	0	0	0	B33..15	Malignant neoplasm of sweat gland	Feb-09
40492	17	0	0	0	BBe9.00	"[M]Triton tumour, malignant"	Feb-09
40508	4	0	0	0	BBj6000	"[M]Hodgkin,s disease, nodular sclerosis, lymphocytic predom"	Feb-09
40513	66	1	0	0	BBkz.00	"[M]Lymphoma, nodular or follicular NOS"	Feb-09
40557	136	0	0	0	B01z.00	Malignant neoplasm of tongue NOS	Feb-09
40561	75	4	0	0	ZV10711	[V]Personal history of Hodgkin's disease	Feb-09

For peer review only - http://bmjopen.bmj.com/site/about/guidelines.xhtml

40592	11	0	0	0	Byu5.00	[X]Malignant neoplasm of mesothelial and soft tissue	Feb-09
40595	102	2	0	0	Byu2000	"[X]Malignant neoplasm of bronchus or lung, unspecified"	Feb-09
40598	130	2	0	0	Byu7.00	[X]Malignant neoplasm of female genital organs	Feb-09
40622	29	0	0	0	BB5V711	[M]Mucoid cell carcinoma	Feb-09
40632	150	5	0	0	BB82.00	[M]Mucinous adenoma and adenocarcinoma	Feb-09
40671	93	0	0	0	Byu8.00	[X]Malignant neoplasm of male genital organs	Feb-09
40740	42	0	0	0	ByuD.00	"[X]Malignant neoplasms of lymphoid, haematopoietic and rela"	Feb-09
40749	10	0	0	0	Byu3.00	[X]Malignant neoplasm of bone and articular cartilage	Feb-09
40766	178	2	0	0	BBm5.00	[M] Peripheral T-cell lymphoma NOS	Feb-09
40810	125	1	0	0	B171.00	Malignant neoplasm of body of pancreas	Feb-09
40814	36	3	0	0	B307200	Malignant neoplasm of tibia	Feb-09
40966	24	0	0	0	B306300	Malignant neoplasm of sacral vertebra	Feb-09
40991	47	1	41	0	4M2..00	Lymphoma staging system	Feb-09
41142	21	1	0	0	D204.00	Idiopathic aplastic anaemia	Feb-09
41144	33	0	0	0	B582300	Secondary malignant neoplasm of skin of trunk	Feb-09
41185	20	0	0	0	Eu02400	[X]Dementia in human immunodef virus [HIV] disease	Feb-09
41215	120	4	0	0	B111100	Malignant neoplasm of pyloric canal of stomach	Feb-09
41245	372	5	0	0	F030411	Herpes simplex encephalitis	Feb-09
41278	107	1	0	0	B323000	Malignant melanoma of external surface of cheek	Feb-09
41313	19	0	0	0	BB5D300	[M]Bile duct cystadenocarcinoma	Feb-09

For peer review only - http://bmjopen.bmj.com/site/about/guidelines.xhtml

41362	35	0	0	0	B101.00	Malignant neoplasm of thoracic oesophagus	Feb-09
41369	6	0	0	0	B60..00	Lymphosarcoma and reticulosarcoma	Feb-09
41461	107	9	0	0	PKy0.11	Prader-Willi Syndrome	Feb-09
41488	20	2	0	0	G554100	Constrictive cardiomyopathy	Feb-09
41490	68	0	0	0	B327700	Malignant melanoma of foot	Feb-09
41500	60	2	0	0	BBr2300	[M]Chronic lymphoid leukaemia	Feb-09
41515	69	0	0	0	ByuA100	"[X]Malignant neoplasm/central nervous system, unspecified"	Feb-09
41520	405	1	0	0	B51z.00	Malignant neoplasm of brain NOS	Feb-09
41523	66	0	0	0	B223000	Malignant neoplasm of middle lobe bronchus	Feb-09
41530	7	0	0	0	B01y.00	Malignant neoplasm of other sites of tongue	Feb-09
41571	93	0	0	0	B495.00	Malignant neoplasm of bladder neck	Feb-09
41695	85	0	0	0	BBba.00	[M]Primitive neuroectodermal tumour	Feb-09
41702	100	3	0	0	BB5N.00	[M]Adenomatous and adenocarcinomatous polyps of colon	Feb-09
41734	17	1	0	0	BBr0000	[M]Leukaemia NOS	Feb-09
41754	52	10	0	0	BBg7.00	"[M]Malignant lymphoma, lymphoplasmacytoid type"	Feb-09
41803	51	2	0	0	BBf..00	[M]Granular cell tumours and alveolar soft part sarcoma	Feb-09
41816	63	1	0	0	BB2E.00	"[M]Squamous cell carcinoma, small cell, non-keratinising"	Feb-09
41841	12	0	0	0	BBgB.00	"[M]Malignant lymphoma, follicular centre cell NOS"	Feb-09
41931	87	0	0	0	B550100	Malignant neoplasm of cheek NOS	Feb-09
41958	141	2	0	0	B331200	Malignant neoplasm of lower eyelid	Feb-09
42012	97	1	0	0	B494.00	Malignant neoplasm of posterior wall of urinary bladder	Feb-09
42017	48	1	0	0	C306400	Hyperammonaemia	Feb-09

For peer review only - http://bmjopen.bmj.com/site/about/guidelines.xhtml

42023	13	1	0	0	B497.00	Malignant neoplasm of urachus	Feb-09
42070	178	2	0	0	B345.00	Malignant neoplasm of lower-outer quadrant of female breast	Feb-09
42082	36	0	0	0	BBK3700	[M]Alveolar rhabdomyosarcoma	Feb-09
42153	58	0	0	0	B32y.00	Malignant melanoma of other specified skin site	Feb-09
42169	432	2	0	0	BB5cz00	[M]Parathyroid adenoma or adenocarcinoma NOS	Feb-09
42193	109	0	0	0	B115.00	Malignant neoplasm of lesser curve of stomach unspecified	Feb-09
42198	35	1	0	0	BBj6.00	"[M]Hodgkin's disease, nodular sclerosis NOS"	Feb-09
42218	62	0	0	0	B55y.00	Malignant neoplasm of other specified sites	Feb-09
42273	134	4	0	0	BB5T.00	[M]Papillary adenomas and adenocarcinomas	Feb-09
42297	100	0	0	0	BBrz.00	[M]Leukaemia NOS	Feb-09
42416	589	10	0	0	B105.00	Malignant neoplasm of lower third of oesophagus	Feb-09
42426	190	3	0	0	B511.00	Malignant neoplasm of frontal lobe	Feb-09
42429	9	6	0	0	B33X.00	Malignant neoplasm overlapping lesion of skin	Feb-09
42460	43	0	0	0	B543.00	Malignant neoplasm of pineal gland	Feb-09
42461	139	2	0	0	B61zz00	Hodgkin's disease NOS	Feb-09
42497	6	0	0	0	P100.00	Unspecified spina bifida with hydrocephalus	Feb-09
42539	54	2	0	0	B670.00	Acute erythraemia and erythroleukaemia	Feb-09
42542	83	3	0	0	BB9K.00	[M]Paget's disease and infiltrating breast duct carcinoma	Feb-09
42553	17	1	0	0	BBB3.00	[M]Adenocarcinoma with cartilaginous and osseous metaplasia	Feb-09

For peer review only - http://bmjopen.bmj.com/site/about/guidelines.xhtml

42566	167	0	0	0	B224z00	"Malignant neoplasm of lower lobe, bronchus or lung NOS"	Feb-09
42569	196	3	0	0	B2zz.00	Malignant neoplasm of respiratory tract NOS	Feb-09
42579	13	0	0	0	B62y300	Malignant lymphoma NOS of intra-abdominal lymph nodes	Feb-09
42636	80	3	0	0	C307y11	Methylmalonic acidaemia	Feb-09
42643	33	1	0	0	PK64.00	Proteus syndrome	Feb-09
42707	49	0	0	0	B336100	Malignant neoplasm of skin of upper arm	Feb-09
42714	48	0	0	0	B327500	Malignant melanoma of ankle	Feb-09
42769	10	0	0	0	BBjz.00	[M]Hodgkin's disease NOS	Feb-09
42784	57	0	0	0	P226.00	Microgyria	Feb-09
42856	27	0	0	0	B200z00	Malignant neoplasm of nasal cavities NOS	Feb-09
43005	97	0	0	0	Q200012	Intracranial haemorrhage in fetus or newborn	Feb-09
43087	193	12	0	0	B331.00	Malignant neoplasm of eyelid including canthus	Feb-09
43111	68	1	0	0	B213.00	Malignant neoplasm of laryngeal cartilage	Feb-09
43122	111	1	0	0	B336000	Malignant neoplasm of skin of shoulder	Feb-09
43151	3	0	0	0	Byu3300	"[X]Malignant neoplasm/bone+articular cartilage, unspecified"	Feb-09
43200	67	1	0	0	B06z.00	Malignant neoplasm of oropharynx NOS	Feb-09
43203	132	7	0	0	F100.00	Leucodystrophy	Feb-09
43311	19	1	0	0	ZV10212	[V]Personal history of malignant neoplasm of larynx	Feb-09
43325	87	2	0	0	PKy7300	Rubenstein - Tayi syndrome	Feb-09
43390	173	0	0	0	B12z.00	Malignant neoplasm of small intestine NOS	Feb-09
43392	237	4	0	0	B483.00	"Malignant neoplasm of penis, part unspecified"	Feb-09
43400	135	3	0	0	B03..00	Malignant neoplasm of gum	Feb-09
43415	14	1	0	0	ByuD000	[X]Other Hodgkin's disease	Feb-09

For peer review only - http://bmjopen.bmj.com/site/about/guidelines.xhtml

For peer review only

43431	272	0	0	0	B010.00	Malignant neoplasm of base of tongue	Feb-09
43435	46	0	0	0	B41yz00	Malignant neoplasm of other site of cervix NOS	Feb-09
43450	41	0	0	0	B63z.00	Immunoproliferative neoplasm or myeloma NOS	Feb-09
43463	383	1	0	0	B325700	Malignant melanoma of back	Feb-09
43479	46	0	0	0	B121.00	Malignant neoplasm of jejunum	Feb-09
43490	41	0	0	0	Byu1100	[X]Other specified carcinomas of liver	Feb-09
43548	26	0	0	0	B080.00	Malignant neoplasm of postcricoid region	Feb-09
43565	6	0	0	0	PJ11.00	"Trisomy 13, mosaicism"	Feb-09
43572	142	1	0	0	B114.00	Malignant neoplasm of body of stomach	Feb-09
43582	52	0	0	0	BBQz.00	[M]Germ cell neoplasm NOS	Feb-09
43594	17	0	0	0	BB5Cz00	[M]Gastrinoma or carcinoma NOS	Feb-09
43614	13	0	0	0	B30X.00	"Malignant neoplasm/bones+articular cartilage/limb,unspfd"	Feb-09
43619	159	0	0	0	B334100	Malignant neoplasm of skin of neck	Feb-09
43642	10	0	0	0	B011.00	Malignant neoplasm of dorsal surface of tongue	Feb-09
43712	44	1	0	0	C375311	Sanfilippo syndrome	Feb-09
43715	6	0	0	0	B325600	Malignant melanoma of umbilicus	Feb-09
43717	12	0	0	0	BB24.11	[M]Verrucous epidermoid carcinoma	Feb-09
43761	44	5	0	0	B451.00	Malignant neoplasm of labia majora	Feb-09
43781	8	0	0	0	B011z00	Malignant neoplasm of dorsum of tongue NOS	Feb-09
43930	12	0	0	0	B582000	Secondary malignant neoplasm of skin of head	Feb-09
43940	13	0	0	0	B431.00	Malignant neoplasm of isthmus of uterine body	Feb-09
44074	28	1	0	0	BB84.00	[M]Mucin-producing adenocarcinoma	Feb-09
44089	27	0	0	0	B517.00	Malignant neoplasm of brain stem	Feb-09
44108	69	0	0	0	B18..00	Malignant neoplasm of retroperitoneum and peritoneum	Feb-09

For peer review only - http://bmjopen.bmj.com/site/about/guidelines.xhtml

44139	1	0	0	0	B073.00	Malignant neoplasm of anterior wall of nasopharynx	Feb-09
44157	5	0	0	0	BBE1.13	[M]Melanosarcoma NOS	Feb-09
44169	217	4	0	0	B222z00	"Malignant neoplasm of upper lobe, bronchus or lung NOS"	Feb-09
44196	11	1	0	0	B611.00	Hodgkin's granuloma	Feb-09
44201	11	0	0	0	PB57.00	Microcolon	Feb-09
44267	71	6	1	0	B623.00	Malignant histiocytosis	Feb-09
44272	33	0	0	0	F391B00	Cardiomyopathy in Duchenne muscular dystrophy	Feb-09
44288	132	2	0	0	R109.00	[D]Laboratory evidence of human immunodeficiency virus [HIV]	Feb-09
44318	144	7	0	0	B62xX00	Oth and unspecif peripheral & cutaneous T-cell lymphomas	Feb-09
44399	54	0	0	0	B150z00	Primary malignant neoplasm of liver NOS	Feb-09
44421	20	2	0	0	ZV67A00	[V]Folow-up exam aft other treatment for malignant neoplasm	Feb-09
44452	6	2	0	0	B300C00	Malignant neoplasm of vomer	Feb-09
44529	40	2	0	0	B575.00	Secondary malignant neoplasm of large intestine and rectum	Feb-09
44592	F11xz00	8	0	0	0	Cerebral degeneration other disease NOS	Feb-09
44609	18	0	0	0	B306000	Malignant neoplasm of ilium	Feb-09
44615	129	0	0	0	B586.00	Secondary malignant neoplasm of ovary	Feb-09
44617	13	0	0	0	A789600	HIV disease resulting in Burkitt's lymphoma	Feb-09
44759	9	0	0	0	PG52000	Osteopetrosis - unclassified	Feb-09
44778	94	0	0	0	BB52000	[M]Adenocarcinoma in tubulovillous adenoma	Feb-09
44871	331	0	1	0	C308000	Medium chain acyl-CoA dehydrogenase deficiency	Feb-09
44884	19	2	0	0	B4Ay.00	Malignant neoplasm of other urinary organs	Feb-09

For peer review only - http://bmjopen.bmj.com/site/about/guidelines.xhtml

44930	11	0	0	0	BB81800	[M]Papillary serous cystadenocarcinoma	Feb-09
44939	P251.00	48	0	1	0	Hydromyelia	Feb-09
44996	33	0	0	0	B491.00	Malignant neoplasm of dome of urinary bladder	Feb-09
45071	42	2	0	0	B314.00	Malignant neoplasm of connective and soft tissue of abdomen	Feb-09
45077	223	4	0	0	B335700	Malignant neoplasm of skin of back	Feb-09
45139	45	0	0	0	B323400	Malignant melanoma of external surface of nose	Feb-09
45154	117	4	0	0	B516.00	Malignant neoplasm of cerebellum	Feb-09
45222	118	1	0	0	B343.00	Malignant neoplasm of lower-inner quadrant of female breast	Feb-09
45260	9	0	0	0	Byu9000	"[X]Malignant neoplasm of urinary organ, unspecified"	Feb-09
45262	7	0	0	0	Byu8200	"[X]Malignant neoplasm of male genital organ, unspecified"	Feb-09
45264	20	0	0	0	B620100	"Nodular lymphoma of lymph nodes of head, face and neck"	Feb-09
45267	30	0	0	0	B55z.00	Malignant neoplasm of other and ill defined site NOS	Feb-09
45306	96	0	0	0	B324100	Malignant melanoma of neck	Feb-09
45307	52	0	0	0	B2...11	Carcinoma of respiratory tract and intrathoracic organs	Feb-09
45408	35	0	0	0	B040.00	Malignant neoplasm of anterior portion of floor of mouth	Feb-09
45458	33	1	0	0	BB2F.00	"[M]Squamous cell carcinoma, spindle cell type"	Feb-09
45490	67	0	0	0	B430z00	Malignant neoplasm of corpus uteri NOS	Feb-09
45510	30	0	0	0	BB2M.00	[M]Lymphoepithelial carcinoma	Feb-09
45531	33	1	0	0	BBbE.00	[M]Gemistocytic astrocytoma	Feb-09
45667	27	0	0	0	B501.00	Malignant neoplasm of orbit	Feb-09
45755	161	0	0	0	B326200	Malignant melanoma of fore-arm	Feb-09

For peer review only - http://bmjopen.bmj.com/site/about/guidelines.xhtml

45760	51	0	0	0	B325z00	"Malignant melanoma of trunk, excluding scrotum, NOS"	Feb-09
45766	11	0	0	0	Byu1200	"[X]Malignant neoplasm of intestinal tract, part unspecified"	Feb-09
45793	27	0	0	0	B430300	Malignant neoplasm of myometrium of corpus uteri	Feb-09
45824	7	1	0	0	B58y900	Secondary malignant neoplasm of tongue	Feb-09
45903	23	0	0	0	F100000	Krabbe's disease	Feb-09
45922	15	6	0	0	B508.00	"Malignant neoplasm, overlapping lesion of eye and adnexa"	Feb-09
45986	13	0	0	0	B041.00	Malignant neoplasm of lateral portion of floor of mouth	Feb-09
46008	93	2	0	0	B333z00	Malignant neoplasm skin other and unspec part of face NOS	Feb-09
46048	5	0	0	0	BBr2500	[M]Prolymphocytic leukaemia	Feb-09
46110	9	0	0	0	A75..12	Pfeiffer's disease	Feb-09
46128	F240000	9	0	0	0	Flaccid tetraplegia	Feb-09
46133	11	1	0	0	PJ12.00	"Trisomy 13, translocation"	Feb-09
46153	6	0	0	0	B443.00	Malignant neoplasm of parametrium	Feb-09
46159	6	0	0	0	B142000	Malignant neoplasm of cloacogenic zone	Feb-09
46255	393	4	0	0	B327.00	Malignant melanoma of lower limb and hip	Feb-09
46263	64	4	0	0	BBr6700	[M]Acute myelomonocytic leukaemia	Feb-09
46423	120	0	0	0	B933.11	Cystosarcoma phyllodes	Feb-09
46444	20	1	0	0	BBr4.00	[M]Erythroleukaemias	Feb-09
46458	8	0	0	0	B335600	Malignant neoplasm of skin of perineum	Feb-09
46516	51	3	0	0	C327200	Niemann-Pick disease	Feb-09
46548	42	1	0	0	B071100	Malignant neoplasm of pharyngeal tonsil	Feb-09
46581	140	0	0	0	BBF4.11	[M]Pleomorphic cell sarcoma	Feb-09
46583	6	5	0	0	P234.00	Hydranencephaly	Feb-09
46613	186	1	0	0	B18y.00	Malignant neoplasm of specified parts of peritoneum	Feb-09

For peer review only - http://bmjopen.bmj.com/site/about/guidelines.xhtml

46728	40	1	0	0	B064.00	Malignant neoplasm of anterior epiglottis	Feb-09
46741	3	0	0	0	BBZC.00	[M]Ameloblastic odontosarcoma	Feb-09
46761	53	0	0	0	BB5f600	[M]Papillary and follicular adenocarcinoma	Feb-09
46771	19	1	0	0	BB5D800	"[M]Hepatocellular carcinoma, fibrolamellar"	Feb-09
46779	26	0	0	0	ZV10417	[V]Personal history of malignant neoplasm of uterine body	Feb-09
46787	29	0	0	0	PJ2z.11	TRISOMY 18 NOS	Feb-09
46789	13	1	0	0	B515000	Malignant neoplasm of choroid plexus	Feb-09
46790	196	7	0	0	P10..00	Spina bifida with hydrocephalus	Feb-09
46792	111	1	0	0	B512.00	Malignant neoplasm of temporal lobe	Feb-09
46794	7	0	0	0	PH3y800	Epidermolysis bullosa letalis	Feb-09
46877	47	1	0	0	BBgL.00	"[M]Malignant lymphoma, small lymphocytic NOS"	Feb-09
46905	1	0	0	0	B545200	Malignant neoplasm of coccygeal body	Feb-09
46931	3	0	0	0	BBg4.00	"[M]Malignant lymphoma, stem cell type"	Feb-09
46939	21	1	0	0	B302000	Malignant neoplasm of cervical vertebra	Feb-09
47094	4	0	0	0	B323200	Malignant melanoma of eyebrow	Feb-09
47205	17	12	0	0	B017.00	Malignant overlapping lesion of tongue	Feb-09
47252	59	1	0	0	B323.00	Malignant melanoma of other and unspecified parts of face	Feb-09
47286	20	0	0	0	B551.00	Malignant neoplasm of thorax	Feb-09
47288	63	0	0	0	P102z00	Spina bifida with hydrocephalus - open NOS	Feb-09
47342	29	1	0	0	Q48y000	Congenital renal failure	Feb-09
47556	30	0	0	0	B512z00	Malignant neoplasm of temporal lobe NOS	Feb-09
47668	4	1	0	0	B48y100	Malignant neoplasm of tunica vaginalis	Feb-09
47683	41	0	0	0	ZV10512	[V]Personal history of malignant neoplasm of kidney	Feb-09

For peer review only - http://bmjopen.bmj.com/site/about/guidelines.xhtml

47734	38	0	0	0	BBP5.00	"[M]Epithelioid mesothelioma, malignant"	Feb-09
47767	111	4	0	0	B486.00	Malignant neoplasm of scrotum	Feb-09
47801	6	0	0	0	B49y000	"Malignant neoplasm, overlapping lesion of bladder"	Feb-09
47810	234	8	0	0	B59..00	Malignant neoplasm of unspecified site	Feb-09
47840	5	0	0	0	B545100	Malignant neoplasm of aortic body	Feb-09
47862	96	2	0	0	B213300	Malignant neoplasm of thyroid cartilage	Feb-09
47899	17	1	0	0	B451000	Malignant neoplasm of greater vestibular (Bartholin's) gland	Feb-09
47920	17	0	0	0	BB9B.11	[M]C cell carcinoma	Feb-09
47974	28	4	0	0	F130600	Aicardi Goutieres syndrome	Feb-09
48048	53	1	0	0	BB1B.00	[M]Giant cell and spindle cell carcinoma	Feb-09
48049	22	0	0	0	BBr6800	[M]Chronic myelomonocytic leukaemia	Feb-09
48073	20	2	0	0	B510000	Malignant neoplasm of basal ganglia	Feb-09
48084	19	0	0	0	PKy9400	Zellweger's syndrome	Feb-09
48126	23	7	0	0	F232.11	Tetraplegia - congenital	Feb-09
48145	19	0	0	0	D212000	Anaemia in ovarian carcinoma	Feb-09
48155	17	1	0	0	BBr2.00	[M]Lymphoid leukaemias	Feb-09
48184	24	0	0	0	PG4E.00	Spondylometaphyseal dysplasia	Feb-09
48211	17	0	0	0	F10y000	Alper's disease	Feb-09
48223	31	1	0	0	BB54.00	[M]Scirrhous adenocarcinoma	Feb-09
48231	68	0	0	0	B13y.00	Malignant neoplasm of other specified sites of colon	Feb-09
48237	30	1	0	0	B111000	Malignant neoplasm of prepylorus of stomach	Feb-09
48253	7	0	0	0	BBg8.00	"[M]Malignant lymphoma, immunoblastic type"	Feb-09
48271	16	0	0	0	BBVz.00	[M]Osteoma or osteosarcoma NOS	Feb-09
48275	50	0	0	0	BBK3600	[M]Embryonal rhabdomyosarcoma	Feb-09
48288	11	1	0	0	R210200	[D]Nonspecific sudden infant death	Feb-09
48293	15	0	0	0	C392100	Severe combined immunodeficiency	Feb-09

For peer review only - http://bmjopen.bmj.com/site/about/guidelines.xhtml

48300	51	3	0	0	F10y100	Leigh's disease	Feb-09
48517	85	10	0	0	B310200	Malignant neoplasm of soft tissue of neck	Feb-09
48519	5	0	0	0	B065.00	Malignant neoplasm of junctional region of epiglottis	Feb-09
48531	75	4	0	0	F11x700	Cerebral degeneration due to Jakob - Creutzfeldt disease	Feb-09
48537	8	0	0	0	B17y.00	Malignant neoplasm of other specified sites of pancreas	Feb-09
48609	P118.00	5	0	0	0	Spina bifida without hydrocephalus - closed	Feb-09
48697	11	0	0	0	7A65000	Transposition of valve of vein	Feb-09
48743	57	0	0	0	B482.00	Malignant neoplasm of body of penis	Feb-09
48809	49	0	0	0	B35zz00	Malignant neoplasm of male breast NOS	Feb-09
48820	246	0	0	0	B410.00	Malignant neoplasm of endocervix	Feb-09
48828	13	0	0	0	B582500	Secondary malignant neoplasm of skin of hip and leg	Feb-09
48879	12	0	0	0	J637.00	Hepatic veno-occlusive disease	Feb-09
48952	87	1	0	0	BBc9z00	[M]Retinoblastoma NOS	Feb-09
48991	0	2	4	0	5136	X-ray metastasis control	Feb-09
49023	10	1	0	0	BBY0.11	[M]Endothelial bone sarcoma	Feb-09
49054	28	0	0	0	B304000	Malignant neoplasm of scapula	Feb-09
49131	14	1	0	0	BBg0.00	"[M]Lymphomatous tumour, benign"	Feb-09
49132	7	0	0	0	B517100	Malignant neoplasm of medulla oblongata	Feb-09
49145	8	0	0	0	B58y700	Secondary malignant neoplasm of penis	Feb-09
49148	21	1	0	0	B347.00	"Malignant neoplasm, overlapping lesion of breast"	Feb-09
49168	15	1	0	0	BBb4.00	[M]Subependymal giant cell astrocytoma	Feb-09
49181	19	0	0	0	F101400	Gangliosidosis	Feb-09
49186	118	5	0	0	BBbR.00	"[M]Oligodendroglioma, anaplastic type"	Feb-09
49253	12	0	0	0	BBk0.13	[M]Giant follicular lymphoma	Feb-09

For peer review only - http://bmjopen.bmj.com/site/about/guidelines.xhtml

49262	55	2	0	0	B627200	Follicular non-Hodgkin's large cell lymphoma	Feb-09
49292	12	0	0	0	Byu1300	[X]Malignant neoplsm/ill-defin sites within digestive system	Feb-09
49301	437	30	0	0	B6z..00	Malignant neoplasm lymphatic or haematopoietic tissue NOS	Feb-09
49322	61	0	0	0	F256z00	Infantile spasms NOS	Feb-09
49327	8	0	0	0	BBrA500	[M]Acute megakaryoblastic leukaemia	Feb-09
49341	3	0	3	0	4D56.00	Pleural fluid: malignant cells	Feb-09
49360	30	2	0	0	B031.00	Malignant neoplasm of lower gum	Feb-09
49400	260	1	0	0	B430211	Malignant neoplasm of endometrium	Feb-09
49403	55	3	0	0	B333100	Malignant neoplasm of skin of chin	Feb-09
49463	11	0	0	0	B310400	Malignant neoplasm of tarsus of eyelid	Feb-09
49482	3	0	0	0	F396200	Myopathy due to malignant disease	Feb-09
49491	31	0	0	0	B303100	Malignant neoplasm of sternum	Feb-09
49525	54	1	0	0	B59zX00	"Kaposi's sarcoma, unspecified"	Feb-09
49542	6	0	0	0	C392500	Severe combined immunodef with low or normal B-cell numbers	Feb-09
49605	43	1	0	0	B615.00	"Hodgkin's disease, mixed cellularity"	Feb-09
49629	7	0	0	0	BB5C100	"[M]Gastrinoma, malignant"	Feb-09
49701	21	1	0	0	B302z00	Malignant neoplasm of vertebral column NOS	Feb-09
49714	10	0	0	0	B523.00	Malignant neoplasm of spinal meninges	Feb-09
49725	20	0	0	0	B64y.00	Other lymphoid leukaemia	Feb-09
49758	22	1	0	0	B0zy.00	"Malignant neoplasm of other sites lip, oral cavity, pharynx"	Feb-09
49770	304	7	0	0	C370z00	Cystic fibrosis NOS	Feb-09
49814	59	1	0	0	B325000	Malignant melanoma of axilla	Feb-09
49825	23	0	0	0	BBh0.11	[M]Reticulum cell sarcoma NOS	Feb-09
49828	174	3	0	0	B441.00	Malignant neoplasm of fallopian tube	Feb-09
49862	42	0	0	0	BBV1.11	[M]Osteoblastic sarcoma	Feb-09

For peer review only

49875	18	0	0	0	B52X.00	"Malignant neoplasm of meninges, unspecified"	Feb-09
50035	37	3	0	0	B545.00	Malignant neoplasm of aortic body and other paraganglia	Feb-09
50076	14	1	0	0	A789400	HIV disease resulting in multiple infections	Feb-09
50108	11	1	0	0	BB5Uz00	[M]Villous adenoma or adenocarcinoma NOS	Feb-09
50140	16	0	0	0	BB5K.00	[M]Cribriform carcinoma	Feb-09
50152	62	0	0	0	B306500	Malignant sacral teratoma	Feb-09
50157	28	0	0	0	G210.00	Malignant hypertensive heart disease	Feb-09
50222	17	0	0	0	B311000	Malignant neoplasm of connective and soft tissue of shoulder	Feb-09
50251	5	1	0	0	PF55100	Acrocephalosyndactyly (Pfeiffer)	Feb-09
50285	67	0	0	0	B410z00	Malignant neoplasm of endocervix NOS	Feb-09
50289	2	0	0	0	B241z00	Malignant neoplasm of heart NOS	Feb-09
50290	6	0	0	0	B6z0.00	Kaposi's sarcoma of lymph nodes	Feb-09
50292	5	0	0	0	Byu2500	"[X]Malignant neoplasm of mediastinum, part unspecified"	Feb-09
50296	3	0	0	0	B000100	"Malignant neoplasm of upper lip, lipstick area"	Feb-09
50297	55	0	0	0	B411.00	Malignant neoplasm of exocervix	Feb-09
50298	5	0	0	0	B300500	Malignant neoplasm of orbital bone	Feb-09
50299	2	0	0	0	B300900	Malignant neoplasm of zygomatic bone	Feb-09
50379	47	1	0	0	BBN1.00	[M]Synovial sarcoma NOS	Feb-09
50402	7	0	0	0	B307100	Malignant neoplasm of fibula	Feb-09
50444	20	0	0	0	PB61z00	Biliary atresia NOS	Feb-09
50475	9	0	0	0	B02z.00	Malignant neoplasm of major salivary gland NOS	Feb-09
50505	123	0	0	0	B326000	Malignant melanoma of shoulder	Feb-09
50554	143	1	0	0	Q48E.11	Periventricular leukomalacia	Feb-09
50565	1	0	0	0	P100200	Thoracic spina bifida with hydrocephalus	Feb-09

For peer review only - http://bmjopen.bmj.com/site/about/guidelines.xhtml

50579	4	0	0	0	B214.00	"Malignant neoplasm, overlapping lesion of larynx"	Feb-09
50605	3	0	0	0	BBDB.00	[M]Glomangiosarcoma	Feb-09
50668	14	0	0	0	B627300	Diffuse non-Hodgkin's small cell (diffuse) lymphoma	Feb-09
50681	50	6	0	0	B480.00	Malignant neoplasm of prepuce (foreskin)	Feb-09
50695	9	0	0	0	B627500	Diffuse non-Hodgkin mixed sml & lge cell (diffuse) lymphoma	Feb-09
50696	34	0	0	0	B62y100	"Malignant lymphoma NOS of lymph nodes of head, face and neck"	Feb-09
50777	2	0	0	0	B524600	"Malignant neoplasm,overlap lesion periph nerve & auton ns"	Feb-09
50789	60	0	0	0	B103.00	Malignant neoplasm of upper third of oesophagus	Feb-09
50859	6	0	0	0	BBX1.11	[M]Giant cell bone sarcoma	Feb-09
50898	74	0	0	0	B18y300	Malignant neoplasm of omentum	Feb-09
50928	10	0	0	0	BBr2600	[M]Burkitt's cell leukaemia	Feb-09
50946	5	0	0	0	BB9C.00	[M]Medullary carcinoma with amyloid stroma	Feb-09
50974	67	0	0	0	B14z.00	"Malignant neoplasm rectum,rectosigmoid junction and anus NOS"	Feb-09
51115	96	0	0	0	B522.00	Malignant neoplasm of spinal cord	Feb-09
51209	78	7	0	0	B325800	Malignant melanoma of chest wall	Feb-09
51237	5	0	0	0	B303z00	"Malignant neoplasm of rib, sternum and clavicle NOS"	Feb-09
51255	58	0	0	0	B1zz.00	Malignant neoplasm of digestive tract and peritoneum NOS	Feb-09
51285	12	0	0	0	BBj2.00	"[M]Hodgkin's disease, mixed cellularity"	Feb-09
51323	9	0	0	0	R210z00	[D]Sudden infant death syndrome NOS	Feb-09
51352	10	0	0	0	B592.00	Malignant neoplasms of independent (primary) multiple sites	Feb-09

For peer review only - http://bmjopen.bmj.com/site/about/guidelines.xhtml

51353	8	1	0	0	BBE1000	"[M]Malignant melanoma, regressing"	Feb-09
51551	41	2	0	0	B571.00	Secondary malignant neoplasm of mediastinum	Feb-09
51656	31	0	0	0	BB81E00	[M]Mucinous cystadenocarcinoma NOS	Feb-09
51680	8	0	0	0	BBgV.00	"[M]Malignant lymphoma, small cell, noncleaved, diffuse"	Feb-09
51690	1	0	0	0	B117.00	"Malignant neoplasm, overlapping lesion of stomach"	Feb-09
51708	7	0	0	0	A789X00	"HIV dis reslt/oth mal neopl/lymph,h'matopoetc+reltd tissu"	Feb-09
51786	100	1	0	0	B021.00	Malignant neoplasm of submandibular gland	Feb-09
51795	31	2	0	0	B545000	Malignant neoplasm of glomus jugulare	Feb-09
51818	27	0	0	0	B550300	Malignant neoplasm of jaw NOS	Feb-09
51852	1	0	0	0	BBgD.00	"[M]Malig lymphoma, lymphocytic, intermediate different NOS"	Feb-09
51873	196	5	0	0	B327100	Malignant melanoma of thigh	Feb-09
51878	8	0	0	0	BBcC.00	[M]Aesthesioneuroblastoma	Feb-09
51895	73	1	0	0	BBgz.00	"[M]Lymphoma, diffuse or NOS"	Feb-09
51911	30	1	0	0	P74z800	Atresia of pulmonary vein	Feb-09
51921	7	1	0	0	B306200	Malignant neoplasm of pubis	Feb-09
51926	33	0	0	0	B062000	Malignant neoplasm of faucial pillar	Feb-09
51965	26	0	0	0	B315.00	Malignant neoplasm of connective and soft tissue of pelvis	Feb-09
52029	76	0	0	0	ByuC800	[X]Malignant neoplasm without specification of site	Feb-09
52070	20	1	0	0	BBDE.00	[M]Gangliocytic paraganglioma	Feb-09
52130	P204.00	22	0	0	0	Meningoencephalocele	Feb-09
52258	15	0	0	0	PB33.00	Total intestinal aganglionosis	Feb-09
52316	29	0	0	0	B553.00	Malignant neoplasm of pelvis	Feb-09
52326	41	2	0	0	BB5L100	[M]Adenocarcinoma in adenomatous polyp	Feb-09

For peer review only - http://bmjopen.bmj.com/site/about/guidelines.xhtml

For peer review only

52493	20	1	0	0	BBQ7213	"[M]Teratoblastoma, malignant"	Feb-09
52496	15	3	0	0	BB61.00	[M]Sweat gland adenoma and adenocarcinomas	Feb-09
52511	6	0	0	0	B515.00	Malignant neoplasm of cerebral ventricles	Feb-09
52537	32	1	0	0	B161100	Malignant neoplasm of hepatic duct	Feb-09
52570	2	0	0	0	B487.00	"Malignant neoplasm, overlapping lesion of penis"	Feb-09
52594	59	1	0	0	B4z..00	Malignant neoplasm of genitourinary organ NOS	Feb-09
52677	18	0	0	0	A412.00	Subacute sclerosing panencephalitis	Feb-09
52683	P102.13	5	0	0	0	Myelocele with hydrocephalus	Feb-09
52684	14	0	0	0	BBW9.00	[M]Mesenchymal chondrosarcoma	Feb-09
52942	21	1	0	0	BBr6300	[M]Chronic myeloid leukaemia	Feb-09
53103	6	0	0	0	B410100	Malignant neoplasm of endocervical gland	Feb-09
53129	3	0	0	0	BB5W112	[M]Oncytic adenocarcinoma	Feb-09
53317	57	2	0	0	F383.00	Congenital and developmental myasthenia	Feb-09
53369	14	1	0	0	B327900	Malignant melanoma of great toe	Feb-09
53382	36	0	0	0	F100200	Pelizaeus-Merzbacher disease	Feb-09
53397	197	6	0	0	B61z.00	Hodgkin's disease NOS	Feb-09
53515	114	5	0	0	B332.00	Malignant neoplasm skin of ear and external auricular canal	Feb-09
53517	6	0	0	0	PB33.12	Congenital aganglionic megacolon	Feb-09
53528	8	0	0	0	B581200	Secondary malignant neoplasm of urethra	Feb-09
53551	8	0	0	0	B627600	Diffuse non-Hodgkin's immunoblastic (diffuse) lymphoma	Feb-09
53591	51	0	0	0	B10y.00	Malignant neoplasm of other specified part of oesophagus	Feb-09
53594	7	0	0	0	B300000	Malignant neoplasm of ethmoid bone	Feb-09
53599	8	0	0	0	B300100	Malignant neoplasm of frontal bone	Feb-09
53629	10	0	0	0	B325200	Malignant melanoma of buttock	Feb-09

For peer review only - http://bmjopen.bmj.com/site/about/guidelines.xhtml

53852	165	1	0	0	K05..12	End stage renal failure	Feb-09
53884	131	0	0	0	B060z00	Malignant neoplasm tonsil NOS	Feb-09
53910	21	0	0	0	B453.00	Malignant neoplasm of clitoris	Feb-09
53929	P113000	6	0	0	0	Spinal meningocele of unspecified site	Feb-09
53933	11	0	0	0	PG51z00	Osteogenesis imperfecta NOS	Feb-09
53987	9	0	0	0	BB5Dz00	[M]Hepatobiliary adenoma or carcinoma NOS	Feb-09
54103	85	0	0	0	B16..00	Malignant neoplasm gallbladder and extrahepatic bile ducts	Feb-09
54106	44	2	0	0	Eu02100	[X]Dementia in Creutzfeldt-Jakob disease	Feb-09
54120	5	0	0	0	B584.00	Secondary malignant neoplasm of other part of nervous system	Feb-09
54133	43	0	0	0	B510z00	Malignant neoplasm of cerebrum NOS	Feb-09
54134	45	0	0	0	B223z00	"Malignant neoplasm of middle lobe, bronchus or lung NOS"	Feb-09
54171	74	2	0	0	B104.00	Malignant neoplasm of middle third of oesophagus	Feb-09
54184	98	1	0	0	B4A1z00	Malignant neoplasm of renal pelvis NOS	Feb-09
54186	5	0	0	0	B313100	Malignant neoplasm of diaphragm	Feb-09
54202	6	0	0	0	B35z.00	Malignant neoplasm of other site of male breast	Feb-09
54222	13	0	0	0	B312400	Malignant neoplasm of connective and soft tissue of foot	Feb-09
54234	39	1	0	0	B334.00	Malignant neoplasm of scalp and skin of neck	Feb-09
54253	250	0	0	0	ByuC700	[X]Secondary malignant neoplasm of other specified sites	Feb-09
54267	90	4	0	0	B59z.00	Malignant neoplasm of unspecified site NOS	Feb-09
54276	4	0	0	0	BB1E.00	[M]Pseudosarcomatous carcinoma	Feb-09
54305	64	0	0	0	B327200	Malignant melanoma of knee	Feb-09
54352	90	2	0	0	B336300	Malignant neoplasm of skin of hand	Feb-09

For peer review only - http://bmjopen.bmj.com/site/about/guidelines.xhtml

54493	7	0	0	0	B303500	Malignant neoplasm of xiphoid process	Feb-09
54494	5	0	0	0	B350.00	Malignant neoplasm of nipple and areola of male breast	Feb-09
54585	52	0	0	0	BBr6100	[M]Acute myeloid leukaemia	Feb-09
54613	3	0	0	0	B201200	Malignant neoplasm of tympanic antrum	Feb-09
54627	6	0	0	0	BBR3.00	[M]Choriocarcinoma combined with teratoma	Feb-09
54631	39	1	0	0	B306.00	"Malignant neoplasm of pelvic bones, sacrum and coccyx"	Feb-09
54632	28	0	0	0	B321.00	Malignant melanoma of eyelid including canthus	Feb-09
54636	53	2	0	0	B203.00	Malignant neoplasm of ethmoid sinus	Feb-09
54679	89	1	0	0	B594.00	Secondary malignant neoplasm of unknown site	Feb-09
54685	173	0	0	0	B326100	Malignant melanoma of upper arm	Feb-09
54691	62	0	0	0	B302200	Malignant neoplasm of lumbar vertebra	Feb-09
54714	P00..11	2	0	0	0	Congenital absence of brain	Feb-09
54747	4	0	0	0	B300600	Malignant neoplasm of parietal bone	Feb-09
54749	2	0	0	0	BB81H00	[M]Papillary mucinous cystadenocarcinoma	Feb-09
54793	3	0	0	0	B682.00	Subacute leukaemia NOS	Feb-09
54874	3	0	0	0	BB85100	[M]Metastatic signet ring cell carcinoma	Feb-09
54934	51	3	0	0	F030400	Encephalitis due to herpes simplex virus	Feb-09
54956	26	0	0	0	B50z.00	Malignant neoplasm of eye NOS	Feb-09
55015	130	0	0	0	B05z.00	Malignant neoplasm of mouth NOS	Feb-09
55019	21	1	0	0	B11y.00	Malignant neoplasm of other specified site of stomach	Feb-09
55066	19	0	0	0	B062.00	Malignant neoplasm of tonsillar pillar	Feb-09
55090	20	0	0	0	B58y100	Secondary malignant neoplasm of uterus	Feb-09
55096	33	1	0	0	B582z00	Secondary malignant neoplasm of skin NOS	Feb-09
55098	71	1	0	0	B550000	Malignant neoplasm of head NOS	Feb-09

For peer review only - http://bmjopen.bmj.com/site/about/guidelines.xhtml

55101	16	0	0	0	B553z00	Malignant neoplasm of pelvis NOS	Feb-09
55116	46	2	0	0	BBFz.00	[M]Soft tissue tumour or sarcoma NOS	Feb-09
55246	6	0	0	0	B20z.00	Malignant neoplasm of accessory sinus NOS	Feb-09
55268	30	2	0	0	BBK2100	[M]Myosarcoma	Feb-09
55292	10	0	0	0	B326z00	Malignant melanoma of upper limb or shoulder NOS	Feb-09
55303	23	0	0	0	B614100	"Hodgkin's nodular sclerosis of head, face and neck"	Feb-09
55374	81	0	0	0	B215.00	Malignant neoplasm of epiglottis NOS	Feb-09
55434	42	0	0	0	B116.00	Malignant neoplasm of greater curve of stomach unspecified	Feb-09
55468	7	0	0	0	BB5R600	"[M]Mucocarcinoid tumour, malignant"	Feb-09
55550	34	2	0	0	B331100	Malignant neoplasm of upper eyelid	Feb-09
55588	14	0	0	0	Byu7300	"[X]Malignant neoplasm of female genital organ, unspecified"	Feb-09
55595	7	0	0	0	B300700	Malignant neoplasm of sphenoid bone	Feb-09
55630	8	0	0	0	B07y.00	Malignant neoplasm of other specified site of nasopharynx	Feb-09
55634	11	0	0	0	Q442.00	Neonatal myasthenia gravis	Feb-09
55636	16	0	0	0	F130100	Hallervorden-Spatz disease	Feb-09
55670	61	1	0	0	B333200	Malignant neoplasm of skin of eyebrow	Feb-09
55850	G558.00	7	0	0	0	Cardiomyopathy in disease EC	Feb-09
55881	88	0	0	0	B324000	Malignant melanoma of scalp	Feb-09
55946	7	0	0	0	B574000	Secondary malignant neoplasm of duodenum	Feb-09
55947	34	4	0	0	BBJ7.00	[M]Pleomorphic liposarcoma	Feb-09
55953	5	0	0	0	B300400	Malignant neoplasm of occipital bone	Feb-09
56041	10	0	0	0	BBj1.00	"[M]Hodgkin's disease, lymphocytic predominance"	Feb-09
56121	94	0	0	0	Byu4300	"[X]Malignant neoplasm of skin, unspecified"	Feb-09

For peer review only - http://bmjopen.bmj.com/site/about/guidelines.xhtml

56288	F10..00	304	0	1	0	Cerebral degenerations usually manifest in childhood	Feb-09
56345	49	0	0	0	B57y.00	Secondary malignant neoplasm of other digestive organ	Feb-09
56355	8	1	0	0	B066.00	Malignant neoplasm of lateral wall of oropharynx	Feb-09
56362	P105.00	8	0	0	0	Spina bifida with stenosis of aqueduct of Sylvius	Feb-09
56490	14	0	0	0	B52z.00	Malignant neoplasm of nervous system NOS	Feb-09
56513	111	2	0	0	B307000	Malignant neoplasm of femur	Feb-09
56540	9	2	0	0	D40y400	Leukaemoid reaction	Feb-09
56600	108	0	0	0	BB2A.11	[M]Epidermoid carcinoma NOS	Feb-09
56676	167	9	0	0	BBJ5.00	[M]Myxoid liposarcoma	Feb-09
56709	5	0	0	0	B04y.00	Malignant neoplasm of other sites of floor of mouth	Feb-09
56715	77	1	0	0	B34y.00	Malignant neoplasm of other site of female breast	Feb-09
56718	15	0	0	0	B500z00	Malignant neoplasm of eyeball NOS	Feb-09
56918	67	0	0	0	B1zy.00	Malignant neoplasm other spec digestive tract and peritoneum	Feb-09
56925	36	0	0	0	Byu4000	[X]Malignant melanoma of other+unspecified parts of face	Feb-09
56951	24	0	0	0	F101200	Spielmeyer-Vogt (Batten) disease	Feb-09
56954	13	1	0	0	B337200	Malignant neoplasm of skin of knee	Feb-09
57047	12	0	0	0	B544.00	Malignant neoplasm of carotid body	Feb-09
57084	6	0	0	0	BBQ1000	"[M]Seminoma, anaplastic type"	Feb-09
57113	8	0	0	0	P100300	Lumbar spina bifida with hydrocephalus	Feb-09
57139	39	1	0	0	C30y800	Glutaric aciduria Type 1	Feb-09
57184	4	0	0	0	Byu4200	[X]Oth malignant neoplasm/skin of oth+unspecfd parts of face	Feb-09

For peer review only - http://bmjopen.bmj.com/site/about/guidelines.xhtml

57191	4	2	0	0	Byu8000	[X]Malignant neoplasm/other specified male genital organs	Feb-09
57225	7	0	0	0	B614000	"Hodgkin's disease, nodular sclerosis of unspecified site"	Feb-09
57235	25	0	0	0	B410000	Malignant neoplasm of endocervical canal	Feb-09
57243	2	1	0	0	P102200	Thoracic spina bifida with hydrocephalus - open	Feb-09
57248	9	0	0	0	B082.00	"Malignant neoplasm aryepiglottic fold, hypopharyngeal aspect"	Feb-09
57260	37	1	0	0	B322.00	Malignant melanoma of ear and external auricular canal	Feb-09
57316	34	0	0	0	BBr6600	[M]Acute promyelocytic leukaemia	Feb-09
57336	51	2	0	0	BB16.00	"[M]Epithelioma, malignant"	Feb-09
57422	79	1	0	0	BB5Vz00	[M]Pituitary adenoma or carcinoma NOS	Feb-09
57427	30	1	0	0	B62y000	Malignant lymphoma NOS of unspecified site	Feb-09
57442	534	1	0	0	B337.00	Malignant neoplasm of skin of lower limb and hip	Feb-09
57446	380	1	0	0	B335.00	"Malignant neoplasm of skin of trunk, excluding scrotum"	Feb-09
57481	41	0	0	0	ByuC300	[X]Secondary malignant neoplasm/oth+unspc respiratory organs	Feb-09
57482	30	1	0	0	B311200	Malignant neoplasm of connective and soft tissue of fore-arm	Feb-09
57505	15	0	0	0	BBK3200	[M]Pleomorphic rhabdomyosarcoma	Feb-09
57513	6	0	0	0	BB2C.11	"[M]Epidermoid carcinoma, keratinising type"	Feb-09
57544	6	0	0	0	BBm4.00	[M]True histiocytic lymphoma	Feb-09
57551	2	0	0	0	F381100	Myasthenic syndrome due to other malignancy	Feb-09
57611	P114z00	14	0	0	0	Meningomyelocele NOS	Feb-09

For peer review only - http://bmjopen.bmj.com/site/about/guidelines.xhtml

57632	21	0	0	0	F151z00	Spinal muscular atrophy NOS	Feb-09
57671	7	0	0	0	B672.00	Megakaryocytic leukaemia	Feb-09
57677	36	1	0	0	BBL8.00	[M]Hepatoblastoma	Feb-09
57680	2	0	0	0	BB2A.12	[M]Spinous cell carcinoma	Feb-09
57713	3	0	0	0	BBr8.00	[M]Eosinophilic leukaemias	Feb-09
57719	4	0	0	0	B41y100	Malignant neoplasm of squamocolumnar junction of cervix	Feb-09
57729	6	0	0	0	BBU1.00	[M]Lymphangiosarcoma	Feb-09
57737	5	0	0	0	B62x100	Lymphoepithelioid lymphoma	Feb-09
57756	10	0	0	0	Byu7100	[X]Malignant neoplasm/other specified female genital organs	Feb-09
57796	5	1	0	0	BBN4.00	"[M]Synovial sarcoma, biphasic type"	Feb-09
57802	16	1	0	0	BB5S400	[M]Alveolar adenocarcinoma	Feb-09
57854	6	0	0	0	B553000	Malignant neoplasm of inguinal region NOS	Feb-09
57988	2	0	0	0	B305000	Malignant neoplasm of carpal bone - scaphoid	Feb-09
58015	10	0	0	0	BBgQ.00	[M]Malignant lymphomatous polyposis	Feb-09
58061	15	4	0	0	B452.00	Malignant neoplasm of labia minora	Feb-09
58082	5	0	0	0	B620800	Nodular lymphoma of lymph nodes of multiple sites	Feb-09
58088	11	0	0	0	B151400	Malignant neoplasm of intrahepatic gall duct	Feb-09
58094	21	6	0	0	B412.00	"Malignant neoplasm, overlapping lesion of cervix uteri"	Feb-09
58121	6	0	0	0	B014.00	Malignant neoplasm of anterior 2/3 of tongue unspecified	Feb-09
58131	13	1	0	0	BB93.00	[M]Comedocarcinoma NOS	Feb-09
58601	51	2	0	0	B337100	Malignant neoplasm of skin of thigh	Feb-09
58635	29	0	0	0	PG51300	Osteogenesis imperfecta type I	Feb-09
58671	1	0	0	0	K0A5300	Heredtry nephrpthy NEC difus mesangial prolif glomnephrit	Feb-09

For peer review only

58684	2	0	0	0	B615200	Hodgkin's mixed cellularity of intrathoracic lymph nodes	Feb-09
58798	3	0	0	0	BB47.00	"[M]Transitional cell carcinoma, spindle cell type"	Feb-09
58835	28	1	0	0	BBE1100	"[M]Desmoplastic melanoma, malignant"	Feb-09
58837	14	0	0	0	BBF5.00	[M]Small cell sarcoma	Feb-09
58871	9	0	0	0	B623z00	Malignant histiocytosis NOS	Feb-09
58903	32	0	0	0	B550z00	"Malignant neoplasm of head, neck and face NOS"	Feb-09
58938	G55y000	29	0	0	0	Cardiomyopathy due to drugs and other external agents	Feb-09
58949	8	0	0	0	B308D00	Malignant neoplasm of phalanges of foot	Feb-09
58958	23	0	0	0	B323500	Malignant melanoma of temple	Feb-09
58962	4	0	0	0	B62x500	Malignant immunoproliferative small intestinal disease	Feb-09
58973	39	1	0	0	Byu0.00	"[X]Malignant neoplasm of lip, oral cavity and pharynx"	Feb-09
59001	49	0	0	0	C310112	Pompe's disease	Feb-09
59004	4	0	0	0	B072.00	Malignant neoplasm of lateral wall of nasopharynx	Feb-09
59035	9	0	0	0	F100z00	Leucodystrophy NOS	Feb-09
59036	24	0	0	0	B300.00	Malignant neoplasm of bones of skull and face	Feb-09
59041	33	0	0	0	B500000	Malignant neoplasm of ciliary body	Feb-09
59061	30	0	0	0	B322000	Malignant melanoma of auricle (ear)	Feb-09
59092	24	0	0	0	B111z00	Malignant neoplasm of pylorus of stomach NOS	Feb-09
59097	4	0	0	0	B431000	Malignant neoplasm of lower uterine segment	Feb-09
59115	4	0	0	0	B602100	"Burkitt's lymphoma of lymph nodes of head, face and neck"	Feb-09

For peer review only - http://bmjopen.bmj.com/site/about/guidelines.xhtml

59143	6	0	0	0	BB2D.00	"[M]Squamous cell carcinoma, large cell, non-keratinising"	Feb-09
59152	8	0	0	0	B315200	Malignant neoplasm of connective and soft tissue of perineum	Feb-09
59155	6	0	0	0	Fy04.11	Ondine's curse	Feb-09
59170	18	0	0	0	B51y000	Malignant neoplasm of corpus callosum	Feb-09
59218	18	2	0	0	P10y000	Dandy - Walker syndrome with spina bifida	Feb-09
59223	7	1	0	0	B306100	Malignant neoplasm of ischium	Feb-09
59240	19	0	0	0	BB58.00	"[M]Carcinoma, diffuse type"	Feb-09
59251	17	0	0	0	BBM9.00	"[M]Cystosarcoma phyllodes, malignant"	Feb-09
59284	12	0	0	0	BB82114	[M]Mucous adenocarcinoma	Feb-09
59286	6	0	0	0	B4Ay000	Malignant neoplasm of overlapping lesion of urinary organs	Feb-09
59310	16	1	0	0	BBV1.12	[M]Osteochondrosarcoma	Feb-09
59343	11	4	0	0	C302512	Oculocerebrorenal dystrophy	Feb-09
59362	13	0	0	0	B451z00	Malignant neoplasm of labia majora NOS	Feb-09
59375	27	0	0	0	B583z00	Secondary malignant neoplasm of brain or spinal cord NOS	Feb-09
59381	25	1	0	0	B500100	Malignant neoplasm of iris	Feb-09
59382	76	11	0	0	B310000	Malignant neoplasm of soft tissue of head	Feb-09
59383	11	0	0	0	G240000	Secondary malignant renovascular hypertension	Feb-09
59388	1	0	0	0	B18y100	Malignant neoplasm of mesocaecum	Feb-09
59415	27	0	0	0	BBB6100	"[M]Thymoma, malignant"	Feb-09
59520	2	0	0	0	B300200	Malignant neoplasm of malar bone	Feb-09
59651	4	0	0	0	BBJ8.00	[M]Mixed type liposarcoma	Feb-09
59755	18	0	0	0	B61z200	Hodgkin's disease NOS of intrathoracic lymph nodes	Feb-09
59778	28	0	0	0	B61z100	"Hodgkin's disease NOS of lymph nodes of head, face and neck"	Feb-09

For peer review only - http://bmjopen.bmj.com/site/about/guidelines.xhtml

59823	1	0	0	0	B542.00	Malignant neoplasm pituitary gland and craniopharyngeal duct	Feb-09
59831	25	0	0	0	B340z00	Malignant neoplasm of nipple or areola of female breast NOS	Feb-09
59855	44	0	0	0	F100300	Metachromatic leucodystrophy	Feb-09
59918	5	0	0	0	BB5f200	"[M]Follicular adenocarcinoma, well differentiated type"	Feb-09
59929	3	0	0	0	BBr0z00	"[M]Leukaemia unspecified, NOS"	Feb-09
59956	F10z.00	10	0	0	0	Childhood cerebral degenerations NOS	Feb-09
60035	23	3	0	0	B310300	Malignant neoplasm of cartilage of ear	Feb-09
60045	30	0	0	0	BB5M100	[M]Tubular adenocarcinoma	Feb-09
60052	114	2	0	0	B55yz00	Malignant neoplasm of specified site NOS	Feb-09
60079	8	0	0	0	PJ63z11	Bonnevie-Ullrich syndrome NOS	Feb-09
60092	16	0	0	0	B62y700	Malignant lymphoma NOS of spleen	Feb-09
60127	6	0	0	0	BBJ5.12	[M]Myxoliposarcoma	Feb-09
60134	2	0	0	0	B581000	Secondary malignant neoplasm of ureter	Feb-09
60162	20	0	0	0	Byu5A00	[X]Malignant neoplasm overlapping lesion of skin	Feb-09
60165	4	1	0	0	PFy4.00	Other arthrogryposis syndromes	Feb-09
60242	1	0	0	0	B600000	Reticulosarcoma of unspecified site	Feb-09
60275	8	0	0	0	BBgJ.00	"[M]Malignant lymphoma, centroblastic type NOS"	Feb-09
60312	10	0	0	0	B16y.00	Malignant neoplasm other gallbladder/extrahepatic bile duct	Feb-09
60335	21	1	0	0	B58y400	Secondary malignant neoplasm of vulva	Feb-09
60347	8	0	0	0	BBd2.11	[M]Leptomeningeal sarcoma	Feb-09
60403	8	0	0	0	B303300	Malignant neoplasm of costal cartilage	Feb-09
60504	2	0	0	0	BBgC.12	[M]Lymphocytic lymphosarcoma NOS	Feb-09
60526	12	0	0	0	B336z00	Malignant neoplasm of skin of upper limb or shoulder NOS	Feb-09
60623	3	0	0	0	P102400	Sacral spina bifida with hydrocephalus - open	Feb-09

For peer review only - http://bmjopen.bmj.com/site/about/guidelines.xhtml

60631	13	0	0	0	BBV5.00	[M]Osteosarcoma in Paget's disease of bone	Feb-09
60673	2	0	0	0	PKyz000	"Ullrich - Feichtiger syndrome, chimaera"	Feb-09
60772	37	0	0	0	B450z00	Malignant neoplasm of vagina NOS	Feb-09
60775	11	1	0	0	BB5h100	[M]Adrenal cortical carcinoma	Feb-09
60918	1	0	25	0	4M20.00	Lymphoma stage I	Feb-09
61064	28	2	0	0	B24X.00	"Malignant neoplasm of mediastinum, part unspecified"	Feb-09
61100	10	0	0	0	P51y.11	Transposition of aorta	Feb-09
61149	3	0	0	0	B614300	Hodgkin's nodular sclerosis of intra-abdominal lymph nodes	Feb-09
61194	14	0	0	0	B337z00	Malignant neoplasm of skin of lower limb or hip NOS	Feb-09
61246	14	0	0	0	B327600	Malignant melanoma of heel	Feb-09
61251	2	0	0	0	BBgN.00	"[M]Malign lymphoma,lymphocytic,intermediate differn, diffuse"	Feb-09
61390	10	0	0	0	B540000	Malignant neoplasm of adrenal cortex	Feb-09
61399	29	0	0	0	B510100	Malignant neoplasm of cerebral cortex	Feb-09
61467	1	0	0	0	BB5f300	"[M]Follicular adenocarcinoma, trabecular type"	Feb-09
61500	9	0	0	0	B690.00	Acute myelomonocytic leukaemia	Feb-09
61510	2	1	0	0	B062200	Malignant neoplasm of palatoglossal arch	Feb-09
61542	31	0	0	0	BBQ7400	"[M]Malignant teratoma, undifferentiated type"	Feb-09
61555	11	0	0	0	B180z00	Malignant neoplasm of retroperitoneum NOS	Feb-09
61588	8	0	0	0	BB85000	[M]Signet ring cell carcinoma	Feb-09
61643	24	0	0	0	B151z00	Malignant neoplasm of intrahepatic bile ducts NOS	Feb-09
61650	2	1	0	0	C375200	"Mucopolysaccharidosis, type II"	Feb-09

For peer review only - http://bmjopen.bmj.com/site/about/guidelines.xhtml

61655	1	0	0	0	ZV10211	[V]Personal history of malignant neoplasm - accessory sinus	Feb-09
61662	12	0	0	0	B61z000	"Hodgkin's disease NOS, unspecified site"	Feb-09
61663	8	2	0	0	ZV76500	[V]Screening for malignant neoplasm of oral cavity	Feb-09
61692	2	0	0	0	B004.00	"Malignant neoplasm of lip unspecified, inner aspect"	Feb-09
61693	16	0	0	0	ByuD600	[X]Other myeloid leukaemia	Feb-09
61695	22	1	0	0	B100.00	Malignant neoplasm of cervical oesophagus	Feb-09
61716	4	0	0	0	B524100	"Malignant neoplasm of peripheral nerve,upp limb,incl should"	Feb-09
61741	50	1	0	0	B304200	Malignant neoplasm of humerus	Feb-09
61769	3	0	0	0	PKyC.00	Pena-Shokeir syndrome type I	Feb-09
61783	22	0	0	0	BBbG.11	[M]Juvenile astrocytoma	Feb-09
61930	314	6	0	0	Kyu2.00	[X]Renal failure	Feb-09
61984	9	0	0	0	BB1G.00	[M]Spheroidal cell carcinoma	Feb-09
61997	26	0	0	0	BBj0.00	[M]Hodgkin's disease NOS	Feb-09
62080	10	2	0	0	B332100	Malignant neoplasm of skin of external auditory meatus	Feb-09
62088	44	2	0	0	BBEG.00	[M]Malignant melanoma in Hutchinson's melanotic freckle	Feb-09
62104	9	0	0	0	B300800	Malignant neoplasm of temporal bone	Feb-09
62126	19	0	0	0	B510500	Malignant neoplasm of thalamus	Feb-09
62169	8	0	0	0	P51y.00	Other specified transposition of great vessels	Feb-09
62182	16	0	0	0	B200300	Malignant neoplasm of vestibule of nose	Feb-09
62199	8	0	0	0	BB5W.00	[M]Oxyphilic adenomas and adenocarcinomas	Feb-09
62305	6	0	0	0	B335800	Malignant neoplasm of skin of buttock	Feb-09
62330	2	0	0	0	BBr6z00	[M]Other myeloid leukaemia NOS	Feb-09
62348	10	1	0	0	BBT1.00	[M]Haemangiosarcoma	Feb-09

For peer review only - http://bmjopen.bmj.com/site/about/guidelines.xhtml

62376	P722500	11	0	0	0	Atresia of aorta	Feb-09
62380	2	0	0	0	B601200	Lymphosarcoma of intrathoracic lymph nodes	Feb-09
62395	44	0	0	0	C306100	Citrullinaemia	Feb-09
62396	27	0	0	0	BBF6.00	[M]Epithelioid cell sarcoma	Feb-09
62437	7	0	0	0	B62x400	Malignant reticulosis	Feb-09
62475	17	0	0	0	B326300	Malignant melanoma of hand	Feb-09
62556	13	0	0	0	B24..00	"Malignant neoplasm of thymus, heart and mediastinum"	Feb-09
62567	4	0	0	0	BBc0200	[M]Ganglioneuromatosis	Feb-09
62584	32	0	0	0	B573.00	Secondary malignant neoplasm of other respiratory organs	Feb-09
62630	2	0	0	0	B307z00	Malignant neoplasm of long bones of leg NOS	Feb-09
62644	14	2	0	0	C372011	Lesch - Nyhan syndrome	Feb-09
62761	22	0	0	0	B200200	Malignant neoplasm of septum of nose	Feb-09
62762	5	0	0	0	C375600	"Mucopolysaccharidosis, type VI"	Feb-09
62828	10	0	0	0	B581z00	Secondary malignant neoplasm of other urinary organ NOS	Feb-09
62840	18	0	0	0	B013.00	Malignant neoplasm of ventral surface of tongue	Feb-09
62871	2	0	0	0	BB92.00	"[M]Comedocarcinoma, noninfiltrating"	Feb-09
62909	26	1	0	0	B575100	Secondary malignant neoplasm of rectum	Feb-09
62941	10	1	0	0	BBe2.00	[M]Neurofibrosarcoma	Feb-09
62951	3	0	28	0	5A15.00	Bone tumour/metast.irradiat.	Feb-09
63054	47	0	0	0	B614z00	"Hodgkin's disease, nodular sclerosis NOS"	Feb-09
63102	6	0	0	0	BB5B100	[M]Islet cell carcinoma	Feb-09
63104	9	0	0	0	B501z00	Malignant neoplasm of orbit NOS	Feb-09
63105	10	0	0	0	B62y500	Malignant lymphoma NOS of lymph node inguinal region and leg	Feb-09

For peer review only - http://bmjopen.bmj.com/site/about/guidelines.xhtml

63224	10	0	0	0	B48z.00	Malignant neoplasm of penis and other male genital organ NOS	Feb-09
63239	7	0	0	0	BBm1.00	[M]Malignant histiocytosis	Feb-09
63247	3	0	0	0	BBK3611	[M]Sarcoma botryoides	Feb-09
63286	3	0	0	0	BBN5.00	[M]Clear cell sarcoma of tendons and aponeuroses	Feb-09
63300	1	0	0	0	Byu3200	[X]Malignant neoplasm/overlap lesion/bone+articulr cartilage	Feb-09
63331	5	0	0	0	B485.00	Malignant neoplasm of spermatic cord	Feb-09
63359	7	0	0	0	C315000	Pyruvate dehydrogenase deficiency	Feb-09
63375	47	0	0	0	ByuDE00	[X]Unspecified B-cell non-Hodgkin's lymphoma	Feb-09
63376	15	0	0	0	F402100	Malignant myopia	Feb-09
63390	65	0	0	0	P512.00	Corrected great vessel transposition	Feb-09
63430	7	0	0	0	B241000	Malignant neoplasm of endocardium	Feb-09
63460	6	0	0	0	B213000	Malignant neoplasm of arytenoid cartilage	Feb-09
63470	34	0	0	0	B102.00	Malignant neoplasm of abdominal oesophagus	Feb-09
63475	4	0	0	0	B652.00	Subacute myeloid leukaemia	Feb-09
63518	11	0	0	0	BBLE.00	[M]Adenosarcoma	Feb-09
63568	7	0	0	0	B524000	"Malignant neoplasm of peripheral nerves of head, face & neck"	Feb-09
63570	3	0	0	0	BBr0113	[M]Stem cell leukaemia	Feb-09
63571	18	0	0	0	BBV..12	[M]Parosteal osteosarcoma	Feb-09
63574	11	0	0	0	BBEC.00	[M]Malignant melanoma in junctional naevus	Feb-09
63598	26	0	0	0	ByuE.00	[X]Malignant neoplasms/independent (primary) multiple sites	Feb-09
63625	0	1	0	0	B616400	Hodgkin's lymphocytic depletion lymph nodes axilla and arm	Feb-09
63634	5	1	0	0	PD00000	Bilateral renal agenesis	Feb-09

For peer review only - http://bmjopen.bmj.com/site/about/guidelines.xhtml

63652	5	0	0	0	F103000	Cerebral degeneration in Hunter's disease	Feb-09
63657	37	1	0	0	B503.00	Malignant neoplasm of conjunctiva	Feb-09
63659	1	0	0	0	BBW6.00	[M]Juxtacortical chondrosarcoma	Feb-09
63695	3	0	0	0	B524300	Malignant neoplasm of peripheral nerve of thorax	Feb-09
63699	15	1	0	0	BBk0.00	"[M]Malignant lymphoma, nodular NOS"	Feb-09
63723	12	0	0	0	B601z00	Lymphosarcoma NOS	Feb-09
63786	24	0	0	0	K01w.00	Congenital nephrotic syndrome	Feb-09
63896	5	0	0	0	B582400	Secondary malignant neoplasm of skin of shoulder and arm	Feb-09
63925	19	0	0	0	ByuA200	"[X]Malignant neoplasm of meninges, unspecified"	Feb-09
63973	1	0	0	0	BBm0.00	[M]Microglioma	Feb-09
63979	7	0	0	0	B013100	Malignant neoplasm of frenulum linguae	Feb-09
63988	12	0	0	0	B311500	Malignant neoplasm of connective and soft tissue of thumb	Feb-09
63994	1	0	0	0	BBgS.00	"[M]Malignant lymphoma, large cell, cleaved, diffuse"	Feb-09
63995	3	0	0	0	B123.00	Malignant neoplasm of Meckel's diverticulum	Feb-09
63997	15	0	0	0	B326500	Malignant melanoma of thumb	Feb-09
64036	3	0	0	0	B612.00	Hodgkin's sarcoma	Feb-09
64106	46	0	0	0	B18yz00	Malignant neoplasm of specified parts of peritoneum NOS	Feb-09
64270	14	0	0	0	B337500	Malignant neoplasm of skin of ankle	Feb-09
64309	13	0	0	0	ByuB100	"[X]Malignant neoplasm of endocrine gland, unspecified"	Feb-09
64327	32	0	0	0	B327z00	Malignant melanoma of lower limb or hip NOS	Feb-09
64336	19	0	0	0	ByuD300	[X]Other specified types of non-Hodgkin's lymphoma	Feb-09

For peer review only - http://bmjopen.bmj.com/site/about/guidelines.xhtml

64343	10	0	0	0	BBj6100	"[M]Hodgkin,s disease, nodular sclerosis, mixed cellularity"	Feb-09
64345	22	0	0	0	B311100	"Malignant neoplasm of connective and soft tissue, upper arm"	Feb-09
64406	5	0	0	0	B336500	Malignant neoplasm of skin of thumb	Feb-09
64462	17	0	0	0	B083.00	Malignant neoplasm of posterior pharynx	Feb-09
64470	11	0	0	0	7904y00	Other correction of transposition of great vessels OS	Feb-09
64497	8	0	0	0	Byu7000	"[X]Malignant neoplasm of uterine adnexa, unspecified"	Feb-09
64515	4	0	0	0	ByuDC00	"[X]Diffuse non-Hodgkin's lymphoma, unspecified"	Feb-09
64516	9	0	0	0	B18y400	Malignant neoplasm of parietal peritoneum	Feb-09
64557	4	1	0	0	B517000	Malignant neoplasm of cerebral peduncle	Feb-09
64596	14	0	0	0	BBK0700	[M]Myxoid leiomyosarcoma	Feb-09
64602	17	7	0	0	B470.00	Malignant neoplasm of undescended testis	Feb-09
64618	4	0	0	0	BBr3.00	[M]Plasma cell leukaemias	Feb-09
64625	5	0	0	0	D200111	Fanconi's hypoplastic anaemia	Feb-09
64670	1	0	0	0	B601300	Lymphosarcoma of intra-abdominal lymph nodes	Feb-09
64680	7	0	0	0	B574.00	Secondary malignant neoplasm of small intestine and duodenum	Feb-09
64686	29	0	0	0	B340100	Malignant neoplasm of areola of female breast	Feb-09
64690	34	1	0	0	F390.00	Congenital hereditary muscular dystrophy	Feb-09
64717	24	0	0	0	P10z.00	Spina bifida with hydrocephalus NOS	Feb-09
64796	16	1	0	0	BB82z00	[M]Mucinous adenoma or adenocarcinoma NOS	Feb-09
64810	5	0	0	0	B551z00	Malignant neoplasm of thorax NOS	Feb-09
64817	7	0	0	0	B502.00	Malignant neoplasm of lacrimal gland	Feb-09

For peer review only - http://bmjopen.bmj.com/site/about/guidelines.xhtml

64837	3	2	0	0	G558200	Dystrophic cardiomyopathy	Feb-09
64848	4	0	0	0	B304400	Malignant neoplasm of ulna	Feb-09
64874	22	1	0	0	BBa4.00	[M]Melanotic neuroectodermal tumour	Feb-09
64897	19	0	0	0	ByuE000	[X]Malignant neoplasms/independent(primary)multiple sites	Feb-09
64963	11	0	0	0	BBr0112	[M]Blastic leukaemia	Feb-09
64971	2	0	0	0	B520000	Malignant neoplasm of olfactory bulb	Feb-09
65047	12	0	0	0	BBDA.00	"[M]Phaeochromocytoma, malignant"	Feb-09
65051	4	0	0	0	BB81500	"[M]Papillary cystadenocarcinoma, NOS"	Feb-09
65091	10	0	0	0	PJ33211	18q- syndrome	Feb-09
65106	26	0	0	0	B44z.00	Malignant neoplasm of uterine adnexa NOS	Feb-09
65117	9	0	0	0	A789900	HIV disease resulting in lymphoid interstitial pneumonitis	Feb-09
65121	8	0	0	0	PHz..11	Congenital ectodermal defect	Feb-09
65122	4	0	0	0	B624000	Leukaemic reticuloendotheliosis of unspecified sites	Feb-09
65123	1	0	0	0	B624300	Leukaemic reticuloend of intra-abdominal lymph nodes	Feb-09
65124	5	0	0	0	B151000	Malignant neoplasm of interlobular bile ducts	Feb-09
65159	4	0	0	0	B180100	Malignant neoplasm of perinephric tissue	Feb-09
65164	169	2	0	0	B326.00	Malignant melanoma of upper limb and shoulder	Feb-09
65165	1	0	0	0	ByuD900	[X]Other leukaemia of unspecified cell type	Feb-09
65180	14	0	0	0	B627800	Diffuse non-Hodgkin's lymphoma undifferentiated (diffuse)	Feb-09
65207	6	0	0	0	BB80z00	[M]Cystadenoma or carcinoma NOS	Feb-09
65215	17	0	0	0	B205.00	Malignant neoplasm of sphenoidal sinus	Feb-09
65216	11	0	0	0	BB49.00	[M]Cloacogenic carcinoma	Feb-09

For peer review only - http://bmjopen.bmj.com/site/about/guidelines.xhtml

65241	4	2	0	0	B51y200	"Malignant neoplasm, overlapping lesion of brain"	Feb-09
65246	P118z00	1	0	0	0	Spina bifida without hydrocephalus - closed NOS	Feb-09
65253	3	1	0	0	B560300	Secondary and unspec malignant neoplasm occipital lymph node	Feb-09
65312	6	0	0	0	B11y000	Malignant neoplasm of anterior wall of stomach NEC	Feb-09
65343	1	0	0	0	F102100	Cerebral degeneration in Niemann-Pick disease	Feb-09
65344	9	0	0	0	C370000	Cystic fibrosis with no meconium ileus	Feb-09
65357	1	0	0	0	B507100	Malignant neoplasm of nasolacrimal duct	Feb-09
65372	14	0	0	0	B11yz00	Malignant neoplasm of other specified site of stomach NOS	Feb-09
65405	5	0	0	0	N237300	Pseudosarcomatous fibromatosis	Feb-09
65434	8	0	0	0	B62z.00	Malignant neoplasms of lymphoid and histiocytic tissue NOS	Feb-09
65460	21	1	0	0	B1z1.00	Malignant neoplasm of spleen NEC	Feb-09
65466	2	0	0	0	B592X00	Kaposi's sarcoma of multiple organs	Feb-09
65483	3	0	0	0	B614400	Hodgkin's nodular sclerosis of lymph nodes of axilla and arm	Feb-09
65489	3	0	0	0	B610.00	Hodgkin's paragranuloma	Feb-09
65490	2	0	0	0	B58y411	Secondary cancer of the vulva	Feb-09
65509	5	0	0	0	PJ50300	Trisomy 9	Feb-09
65584	4	0	0	0	BBj1000	"[M]Hodgkin,s disease, lymphocytic predominance, diffuse"	Feb-09
65599	10	0	0	0	B520200	Malignant neoplasm of acoustic nerve	Feb-09
65605	2	0	0	0	B241200	Malignant neoplasm of myocardium	Feb-09
65625	178	0	0	0	B324.00	Malignant melanoma of scalp and neck	Feb-09
65642	2	0	0	0	B623300	Malignant histiocytosis of intra-abdominal lymph nodes	Feb-09
65701	31	1	0	0	B620z00	Nodular lymphoma NOS	Feb-09

For peer review only - http://bmjopen.bmj.com/site/about/guidelines.xhtml

65721	2	0	0	0	B673.00	Mast cell leukaemia	Feb-09
65777	8	1	0	0	B672.11	Thrombocytic leukaemia	Feb-09
65778	F4Gy011	6	0	0	0	Encephalocoele of orbit	Feb-09
65782	4	0	0	0	B337800	Malignant neoplasm of skin of toe	Feb-09
65861	6	0	0	0	BBQ9.00	[M]Dermoid cyst with malignant transformation	Feb-09
65935	PG43.00	7	0	0	0	Asphyxiating thoracic dysplasia	Feb-09
65952	10	0	0	0	BBbU.00	[M]Desmoplastic medulloblastoma	Feb-09
66000	6	0	0	0	BBB5.00	[M]Adenocarcinoma with apocrine metaplasia	Feb-09
66019	6	0	0	0	L254.11	Suspect fetal damage from maternal alcohol	Feb-09
66064	13	1	0	0	BBbM.00	[M]Giant cell glioblastoma	Feb-09
66073	14	0	0	0	C392400	Severe combined immunodef with low T-and B-cell numbers	Feb-09
66089	6	0	0	0	B65yz00	Other myeloid leukaemia NOS	Feb-09
66155	12	0	0	0	C306200	Argininosuccinic aciduria	Feb-09
66163	11	0	0	0	ByuC200	[X]2ndry+unspcf malignant neoplasm lymph nodes/multi regions	Feb-09
66166	5	0	0	0	B124.00	"Malignant neoplasm, overlapping lesion of small intestine"	Feb-09
66270	11	0	0	0	B000000	"Malignant neoplasm of upper lip, external"	Feb-09
66319	5	1	0	0	B335500	Malignant neoplasm of skin of groin	Feb-09
66327	13	0	0	0	B620000	Nodular lymphoma of unspecified site	Feb-09
66367	10	0	0	0	A789700	HIV dis resulting oth types of non-Hodgkin's lymphoma	Feb-09
66368	6	0	0	0	A789100	HIV disease resulting in cytomegaloviral disease	Feb-09
66384	26	0	0	0	B001000	"Malignant neoplasm of lower lip, external"	Feb-09

For peer review only - http://bmjopen.bmj.com/site/about/guidelines.xhtml

66422	1	0	0	0	B074.00	"Malignant neoplasm, overlapping lesion of nasopharynx"	Feb-09
66444	2	0	0	0	Byu2100	"[X]Malignant neoplasm/overlap lesion/heart,mediastinm+pleura"	Feb-09
66447	13	0	0	0	B335A00	Malignant neoplasm of skin of scapular region	Feb-09
66499	23	0	0	0	P00z.00	Anencephalus NOS	Feb-09
66541	3	0	0	0	BB1J.12	[M]Round cell carcinoma	Feb-09
66603	12	0	0	0	BBgK.00	"[M]Malig lymphoma, follicular centre cell, non-cleaved NOS"	Feb-09
66607	6	0	0	0	BBL4.00	"[M]Mixed tumour, malignant, NOS"	Feb-09
66639	14	0	0	0	B303200	Malignant neoplasm of clavicle	Feb-09
66646	6	0	0	0	B26..00	"Malignant neoplasm, overlap lesion of resp & intrathor orgs"	Feb-09
66694	1	0	0	0	BBr6311	[M]Naegeli-type monocytic leukaemia	Feb-09
66699	4	0	0	0	PK83.00	Fetus and newborn affected by maternal use of alcohol	Feb-09
66750	4	0	0	0	B24z.00	"Malignant neoplasm of heart, thymus and mediastinum NOS"	Feb-09
66775	1	0	0	0	B560100	Secondary and unspec malignant neoplasm mastoid lymph nodes	Feb-09
66876	3	0	0	0	BB81E11	[M]Pseudomucinous adenocarcinoma	Feb-09
66908	5	2	0	0	B306400	Malignant neoplasm of coccygeal vertebra	Feb-09
67019	2	0	0	0	BBK1100	[M]Angiomyosarcoma	Feb-09
67029	12	0	0	0	ByuD500	[X]Other lymphoid leukaemia	Feb-09
67034	13	1	0	0	Byu5000	[X]Mesothelioma of other sites	Feb-09
67107	5	0	0	0	B230.00	Malignant neoplasm of parietal pleura	Feb-09
67203	7	0	0	0	BBgG.11	[M]Lymphoblastic lymphosarcoma NOS	Feb-09
67211	1	0	0	0	B523z00	Malignant neoplasm of spinal meninges NOS	Feb-09
67217	8	0	0	0	B55y100	Malignant neoplasm of trunk NOS	Feb-09

For peer review only - http://bmjopen.bmj.com/site/about/guidelines.xhtml

67234	8	0	0	0	PJ21.00	"Trisomy 18, mosaicism"	Feb-09
67236	2	0	0	0	B512000	Malignant neoplasm of hippocampus	Feb-09
67248	3	0	13	0	5A12.00	Thyroid tumour/metast irradiat	Feb-09
67323	25	3	0	0	B06y.00	"Malignant neoplasm of oropharynx, other specified sites"	Feb-09
67339	1	0	0	0	BBp2.00	[M]Malignant mastocytosis	Feb-09
67342	8	0	0	0	BB5U100	[M]Adenocarcinoma in villous adenoma	Feb-09
67351	P113100	15	0	0	0	Cervical spinal meningocele	Feb-09
67446	10	0	0	0	B001.00	"Malignant neoplasm of lower lip, vermilion border"	Feb-09
67451	2	0	0	0	B30W.00	Malignant neoplasm/overlap lesion/bone+articulr cartilage	Feb-09
67497	6	0	0	0	B106.00	"Malignant neoplasm, overlapping lesion of oesophagus"	Feb-09
67504	4	1	0	0	B003000	"Malignant neoplasm of lower lip, buccal aspect"	Feb-09
67506	9	0	0	0	B614200	Hodgkin's nodular sclerosis of intrathoracic lymph nodes	Feb-09
67518	4	0	0	0	ByuD100	[X]Other types of follicular non-Hodgkin's lymphoma	Feb-09
67575	10	0	0	0	A788W00	HIV disease resulting in unspecified malignant neoplasm	Feb-09
67587	18	0	0	0	BBbZ.00	[M]Pleomorphic xanthoastrocytoma	Feb-09
67700	4	0	0	0	B66..12	Monoblastic leukaemia	Feb-09
67701	35	0	0	0	BB94.11	[M]Secretory breast carcinoma	Feb-09
67703	8	0	0	0	B616.00	"Hodgkin's disease, lymphocytic depletion"	Feb-09
67712	8	0	0	0	BBR2.00	[M]Choriocarcinoma	Feb-09
67748	4	0	0	0	B335400	Malignant neoplasm of skin of umbilicus	Feb-09
67762	1	0	0	0	F103100	Cerebral degeneration in mucopolysaccharidoses	Feb-09

For peer review only - http://bmjopen.bmj.com/site/about/guidelines.xhtml

67763	2	0	0	0	B303400	Malignant neoplasm of costo-vertebral joint	Feb-09
67806	62	0	0	0	B323z00	Malignant melanoma of face NOS	Feb-09
67878	P112.00	1	0	0	0	Hydromyelocele	Feb-09
67884	4	0	0	0	B350100	Malignant neoplasm of areola of male breast	Feb-09
67906	10	5	0	0	F431400	Metastatic disseminated retinitis	Feb-09
67912	3	0	0	0	BB26.11	[M]Papillary epidermoid carcinoma	Feb-09
67913	7	1	0	0	BB6A.00	[M]Ceruminous adenoma and adenocarcinoma	Feb-09
67914	3	0	0	0	B337900	Malignant neoplasm of skin of great toe	Feb-09
67927	3	0	0	0	C372300	Lesch-Nyhan syndrome	Feb-09
67934	6	0	0	0	BBLA.00	"[M]Carcinosarcoma, embryonal type"	Feb-09
67949	10	0	0	0	B48y.00	Malignant neoplasm of other male genital organ	Feb-09
67966	3	0	0	0	BBE1.14	[M]Naevocarcinoma	Feb-09
67970	2	0	0	0	BB1L.00	"[M]Small cell carcinoma, fusiform cell type"	Feb-09
68027	4	0	0	0	ByuA000	[X]Malignant neoplasm/other and unspecified cranial nerves	Feb-09
68039	0	0	0	0	B612400	Hodgkin's sarcoma of lymph nodes of axilla and upper limb	Feb-09
68055	32	0	0	0	B307.00	Malignant neoplasm of long bones of leg	Feb-09
68087	42	1	0	0	D20z.00	Aplastic anaemia NOS	Feb-09
68133	21	0	0	0	B323300	Malignant melanoma of forehead	Feb-09
68155	7	0	0	0	B430100	Malignant neoplasm of fundus of corpus uteri	Feb-09
68161	23	0	0	0	B48y000	Malignant neoplasm of seminal vesicle	Feb-09
68181	2	1	0	0	P002.11	Hemianencephaly	Feb-09
68197	5	0	0	0	B337300	Malignant neoplasm of skin of popliteal fossa area	Feb-09
68220	2	0	0	0	BBW4.11	[M]Fibrochondrosarcoma	Feb-09

For peer review only - http://bmjopen.bmj.com/site/about/guidelines.xhtml

68221	P110100	2	0	0	0	Cervical spina bifida without mention of hydrocephalus	Feb-09
68236	3398	6	0	0	B550.00	"Malignant neoplasm of head, neck and face"	Feb-09
68330	8	0	0	0	B613100	"Hodgkin's, lymphocytic-histiocytic pred of head, face, neck"	Feb-09
68332	12	0	0	0	ByuC600	[X]2ndry malignant neoplasm/oth+unspec parts/nervous system	Feb-09
68399	1	0	0	0	B004200	"Malignant neoplasm of lip unspecified, mucosa"	Feb-09
68410	11	0	0	0	B150200	Primary angiosarcoma of liver	Feb-09
68447	8	0	0	0	BBEV.00	"[M]Blue naevus, malignant"	Feb-09
68456	25	0	0	0	BB5V100	[M]Chromophobe carcinoma	Feb-09
68479	4	3	0	0	BBc7.11	[M]Neuroastrocytoma	Feb-09
68480	7	1	0	0	B350000	Malignant neoplasm of nipple of male breast	Feb-09
68612	2	0	0	0	ZV10z00	[V]Personal history of unspecified malignant neoplasm	Feb-09
68641	8	0	0	0	B517z00	Malignant neoplasm of brain stem NOS	Feb-09
68730	25	0	0	0	BBZN.00	[M]Ameloblastic fibrosarcoma	Feb-09
68757	2	0	0	0	BB5f700	[M]Nonencapsulated sclerosing carcinoma	Feb-09
68783	2	0	0	0	BB60100	[M]Skin appendage carcinoma	Feb-09
68787	19	0	0	0	B55y000	Malignant neoplasm of back NOS	Feb-09
68808	11	0	0	0	BBb2.00	[M]Mixed glioma	Feb-09
68824	2	0	0	0	B48y200	"Malignant neoplasm, overlapping lesion male genital orgs"	Feb-09
68956	8	1	0	0	BBX1.00	"[M]Giant cell tumour of bone, malignant"	Feb-09
68964	2	0	0	0	BBgA.00	"[M]Malignant lymphoma, centroblastic-centrocytic, diffuse"	Feb-09

For peer review only - http://bmjopen.bmj.com/site/about/guidelines.xhtml

68999	1	1	0	0	BBK2z00	[M]Myoma or myosarcoma NOS	Feb-09
69017	10	0	0	0	C370100	Cystic fibrosis with meconium ileus	Feb-09
69027	15	0	0	0	D200.00	Constitutional aplastic anaemia	Feb-09
69061	3	0	0	0	D200011	Constitutional aplastic anaemia without malformation	Feb-09
69104	5	1	0	0	B305100	Malignant neoplasm of carpal bone - lunate	Feb-09
69146	7	0	0	0	B300z00	Malignant neoplasm of bones of skull and face NOS	Feb-09
69299	1	1	0	0	BBrA111	[M]Thrombocytic leukaemia	Feb-09
69300	5	0	0	0	BB1F.00	[M]Polygonal cell carcinoma	Feb-09
69301	1	0	0	0	BBg5.00	"[M]Malignant lymphoma, convoluted cell type NOS"	Feb-09
69381	10	0	0	0	C375100	"Mucopolysaccharidosis, type 1"	Feb-09
69421	3	0	0	0	BBDz.00	[M]Paraganglioma or glomus tumour NOS	Feb-09
69436	1	0	0	0	PG51500	Osteogenesis imperfecta type III	Feb-09
69476	14	0	0	0	PJ50200	Trisomy 8	Feb-09
69497	1	0	0	0	B623000	Malignant histiocytosis of unspecified site	Feb-09
69613	11	1	0	0	C377100	Mucolipidosis type III	Feb-09
69671	2	1	0	0	B010.11	Malignant neoplasm of posterior third of tongue	Feb-09
69761	4	0	0	0	B00zz00	"Malignant neoplasm of lip, vermilion border NOS"	Feb-09
69766	8	0	0	0	A788200	HIV infection with persistent generalised lymphadenopathy	Feb-09
69767	3	0	0	0	AyuC600	[X]HIV disease resulting in other non-Hodgkin's lymphoma	Feb-09
69821	11	1	0	0	B18y600	Malignant neoplasm of the pouch of Douglas	Feb-09
69844	11	0	0	0	BBF5.11	[M]Round cell sarcoma	Feb-09
69858	8	0	0	0	P510.00	Total great vessel transposition	Feb-09

For peer review only - http://bmjopen.bmj.com/site/about/guidelines.xhtml

69927	1	0	0	0	B308800	Malignant neoplasm of first metatarsal bone	Feb-09
69951	5	0	0	0	B055100	Malignant neoplasm of roof of mouth	Feb-09
69980	23	2	0	0	BBgC.00	"[M]Malignant lymphoma, lymphocytic, well differentiated NOS"	Feb-09
69981	10	1	0	0	BBe7.00	"[M]Neurilemmoma, malignant"	Feb-09
70104	6	0	0	0	B521z00	Malignant neoplasm of cerebral meninges NOS	Feb-09
70126	9	0	0	0	B520100	Malignant neoplasm of optic nerve	Feb-09
70320	8	0	0	0	BBD4.11	[M]Jugular paraganglioma	Feb-09
70374	1	0	0	0	B600300	Reticulosarcoma of intra-abdominal lymph nodes	Feb-09
70380	6	0	0	0	B335000	Malignant neoplasm of skin of axillary fold	Feb-09
70383	2	0	0	0	BBM0100	"[M]Brenner tumour, malignant"	Feb-09
70439	4	0	0	0	C375300	"Mucopolysaccharidosis, type III"	Feb-09
70463	16	0	0	0	B315000	Malignant neoplasm of connective and soft tissue of buttock	Feb-09
70509	5	0	0	0	B627D00	Diffuse non-Hodgkin's centroblastic lymphoma	Feb-09
70516	9	0	0	0	BB5D.11	[M]Biliary tract adenomas and adenocarcinomas	Feb-09
70572	4	0	0	0	F151000	Unspecified spinal muscular atrophy	Feb-09
70587	8	0	0	0	B337700	Malignant neoplasm of skin of foot	Feb-09
70637	11	0	0	0	B320.00	Malignant melanoma of lip	Feb-09
70696	3	0	0	0	B02y.00	Malignant neoplasm of other major salivary glands	Feb-09
70709	11	0	4	0	4L49.00	Prion protein markers for Creutzfeldt-Jakob disease	Feb-09
70724	7	1	0	0	B653.00	Myeloid sarcoma	Feb-09
70729	3	0	0	0	B431z00	Malignant neoplasm of isthmus of uterine body NOS	Feb-09

For peer review only - http://bmjopen.bmj.com/site/about/guidelines.xhtml

70736	7	0	0	0	B58y300	Secondary malignant neoplasm of vagina	Feb-09
70740	1	0	0	0	BBm1.11	[M]Malignant reticulosis	Feb-09
70819	26	0	0	0	B055.00	Malignant neoplasm of palate unspecified	Feb-09
70824	20	0	0	0	B540z00	Malignant neoplasm of adrenal gland NOS	Feb-09
70842	7	0	0	0	B627100	Follicular non-Hodg mixed sml cleavd & lge cell lymphoma	Feb-09
70855	7	0	0	0	G558000	Cardiomyopathy in Friedreich's ataxia	Feb-09
70928	5	0	0	0	B022.00	Malignant neoplasm of sublingual gland	Feb-09
70935	1	0	0	0	BBr4000	[M]Erythroleukaemia	Feb-09
70942	8	0	0	0	B510400	Malignant neoplasm of hypothalamus	Feb-09
70951	3	0	0	0	BBP0.00	"[M]Mesothelioma, benign"	Feb-09
70988	4	1	0	0	B337000	Malignant neoplasm of skin of hip	Feb-09
71031	1	0	0	0	B600100	"Reticulosarcoma of lymph nodes of head, face and neck"	Feb-09
71117	2	0	0	0	BBg3.00	"[M]Malignant lymphoma, undifferentiated cell type NOS"	Feb-09
71136	8	0	0	0	B323100	Malignant melanoma of chin	Feb-09
71139	26	0	0	0	B51y.00	Malignant neoplasm of other parts of brain	Feb-09
71142	3	0	0	0	B613000	"Hodgkin's, lymphocytic-histiocytic predominance unspec site"	Feb-09
71147	4	0	0	0	B003.00	"Malignant neoplasm of lower lip, inner aspect"	Feb-09
71204	6	1	0	0	B200000	Malignant neoplasm of cartilage of nose	Feb-09
71238	5	0	0	0	B601100	"Lymphosarcoma of lymph nodes of head, face and neck"	Feb-09
71262	4	0	0	0	B62y600	Malignant lymphoma NOS of intrapelvic lymph nodes	Feb-09
71301	5	0	0	0	BBQA100	"[M]Struma ovarii, malignant"	Feb-09
71304	14	0	0	0	B602z00	Burkitt's lymphoma NOS	Feb-09
71377	1	0	0	0	BBr8000	[M]Eosinophilic leukaemia	Feb-09

For peer review only - http://bmjopen.bmj.com/site/about/guidelines.xhtml

71450	2	0	0	0	A788X00	HIV disease resulting/unspcf infectious+parasitic disease	Feb-09
71497	4	0	0	0	BB5W100	[M]Oxyphilic adenocarcinoma	Feb-09
71526	15	0	0	0	PKyF.00	Alstrom syndrome	Feb-09
71584	5	2	0	0	B507.00	Malignant neoplasm of lacrimal duct	Feb-09
71619	1	0	0	0	BBgT.00	"[M]Malignant lymphoma, large cell, noncleaved, diffuse"	Feb-09
71625	1	0	0	0	B601000	Lymphosarcoma of unspecified site	Feb-09
71627	2	0	0	0	BB61200	[M]Sweat gland adenocarcinoma	Feb-09
71652	2	0	0	0	BBgP.00	"[M]Malignant lymphoma, mixed small and large cell, diffuse"	Feb-09
71672	8	0	25	0	4M23.00	Lymphoma stage IV	Feb-09
71810	11	0	0	0	B304.00	Malignant neoplasm of scapula and long bones of upper arm	Feb-09
71850	8	0	0	0	BBr6000	[M]Myeloid leukaemia NOS	Feb-09
71869	11	0	0	0	BBf2.00	[M]Alveolar soft part sarcoma	Feb-09
71895	11	3	0	0	BB56.00	[M]Superficial spreading adenocarcinoma	Feb-09
71946	3	1	0	0	B201300	Malignant neoplasm of mastoid air cells	Feb-09
72018	P118100	0	0	0	0	Cervical spina bifida without hydrocephalus - closed	Feb-09
72127	5	0	0	0	B484.00	Malignant neoplasm of epididymis	Feb-09
72139	1	0	0	0	PJ10.00	"Trisomy 13, meiotic nondisjunction"	Feb-09
72174	1	0	0	0	B4A4.00	Malignant neoplasm of paraurethral glands	Feb-09
72179	1	0	0	0	BBr0200	[M]Subacute leukaemia NOS	Feb-09
72192	14	0	0	0	BB5Xz00	[M]Clear cell adenoma or adenocarcinoma NOS	Feb-09
72196	6	1	0	0	BBgG.00	"[M]Malignant lymphoma, lymphocytic, poorly different NOS"	Feb-09
72197	2	0	0	0	B67y000	Lymphosarcoma cell leukaemia	Feb-09
72212	4	0	0	0	B308200	Malignant neoplasm of calcaneum	Feb-09
72222	1	0	0	0	BBrA100	[M]Megakaryocytic leukaemia	Feb-09

For peer review only - http://bmjopen.bmj.com/site/about/guidelines.xhtml

72224	7	1	0	0	B1z1100	Fibrosarcoma of spleen	Feb-09
72241	1	1	0	0	BBgH.00	[M]Prolymphocytic lymphosarcoma	Feb-09
72265	8	0	0	0	PJ1z.00	Patau's syndrome NOS	Feb-09
72277	4	1	0	0	BB5V700	[M]Basophil carcinoma	Feb-09
72303	1	0	0	0	K01w000	Finnish nephrosis syndrome	Feb-09
72310	1	1	0	0	BBr0400	[M]Aleukaemic leukaemia NOS	Feb-09
72433	3	0	0	0	BBh0.00	[M]Reticulosarcoma NOS	Feb-09
72443	2	0	0	0	BBZ2.00	"[M]Odontogenic tumour, malignant"	Feb-09
72445	4	0	0	0	B161000	Malignant neoplasm of cystic duct	Feb-09
72464	4	0	0	0	B305.12	Malignant neoplasm of metacarpal bones	Feb-09
72522	2	0	0	0	B313200	Malignant neoplasm of great vessels	Feb-09
72668	5	0	0	0	G210100	Malignant hypertensive heart disease with CCF	Feb-09
72707	13	0	0	0	P0...00	Anencephalus and similar anomalies	Feb-09
72723	5	0	0	0	B430000	Malignant neoplasm of cornu of corpus uteri	Feb-09
72725	8	0	0	0	B62y200	Malignant lymphoma NOS of intrathoracic lymph nodes	Feb-09
72774	2	0	0	0	B642.00	Subacute lymphoid leukaemia	Feb-09
73055	P20z.00	10	0	0	0	Encephalocele NOS	Feb-09
73065	8	0	0	0	C370y00	Cystic fibrosis with other manifestations	Feb-09
73066	1	0	0	0	BBrA.00	[M]Miscellaneous leukaemias	Feb-09
73085	2	0	0	0	P10y.00	Other specified spina bifida with hydrocephalus	Feb-09
73088	2	0	0	0	BBr9000	[M]Monocytic leukaemia NOS	Feb-09
73213	11	0	0	0	B581.00	Secondary malignant neoplasm of other urinary organs	Feb-09
73251	0	0	0	0	BBEM.00	[M]Malignant melanoma in giant pigmented naevus	Feb-09
73275	5	0	0	0	BB5N100	[M]Adenocarcinoma in adenomatous polposis coli	Feb-09
73293	7	0	0	0	G240z00	Secondary malignant hypertension NOS	Feb-09

For peer review only - http://bmjopen.bmj.com/site/about/guidelines.xhtml

73296	5	0	0	0	Byu3100	"[X]Malignant neoplasm/bones+articular cartilage/limb,unspfd"	Feb-09
73434	6	0	0	0	BB5L300	[M]Adenocarcinoma in multiple adenomatous polyps	Feb-09
73439	9	0	0	0	B064z00	Malignant neoplasm of anterior epiglottis NOS	Feb-09
73510	10	0	0	0	B550500	Malignant neoplasm of supraclavicular fossa NOS	Feb-09
73530	7	1	0	0	B305.00	Malignant neoplasm of hand bones	Feb-09
73532	2	0	0	0	B613300	"Hodgkin's, lymphocytic-histiocytic pred intra-abdominal node"	Feb-09
73536	5	0	0	0	B327000	Malignant melanoma of hip	Feb-09
73539	26	2	0	0	P51z.00	Great vessel transposition NOS	Feb-09
73556	5	0	0	0	B305z00	Malignant neoplasm of hand bones NOS	Feb-09
73583	25	0	0	0	F14y000	Ataxia-telangiectasia	Feb-09
73608	P117z00	2	0	0	0	Spina bifida without hydrocephalus - open NOS	Feb-09
73612	1	0	0	0	PE02.00	Potter's facies	Feb-09
73614	8	0	0	0	B004000	"Malignant neoplasm of lip unspecified, buccal aspect"	Feb-09
73616	15	0	0	0	B58y200	Secondary malignant neoplasm of cervix uteri	Feb-09
73662	1	0	0	0	BB5Wz00	[M]Oxyphilic adenoma or adenocarcinoma NOS	Feb-09
73712	P115z00	7	0	0	0	Myelocele NOS	Feb-09
73742	7	0	0	0	F427K00	Lipofuscinosis NOS	Feb-09
73744	5	0	0	0	B322z00	Malignant melanoma of ear and external auricular canal NOS	Feb-09
73760	12	0	0	0	B334z00	Malignant neoplasm of scalp or skin of neck NOS	Feb-09
73777	2	0	0	0	B624z00	Leukaemic reticuloendotheliosis NOS	Feb-09
73916	8	0	0	0	BBK0400	[M]Epithelioid leiomyosarcoma	Feb-09

For peer review only - http://bmjopen.bmj.com/site/about/guidelines.xhtml

73962	4	1	0	0	B000.00	"Malignant neoplasm of upper lip, vermilion border"	Feb-09
73988	6	0	0	0	B524500	Malignant neoplasm of peripheral nerve of pelvis	Feb-09
73992	5	0	0	0	B504.00	Malignant neoplasm of cornea	Feb-09
74896	30	0	0	0	B161z00	Malignant neoplasm of extrahepatic bile ducts NOS	Feb-09
85190	12	0	0	0	F562100	Malignant positional vertigo	Feb-09
85988	11	0	0	0	P210.00	Hydromicrocephaly	Feb-09
86046	17	0	0	0	B524400	Malignant neoplasm of peripheral nerve of abdomen	Feb-09
86812	12	0	0	0	B305D00	Malignant neoplasm of phalanges of hand	Feb-09
86820	6	0	0	0	BBP7.00	"[M]Mesothelioma, biphasic type, malignant"	Feb-09
86996	3	0	0	0	B501000	Malignant neoplasm of connective tissue of orbit	Feb-09
86997	8	0	0	0	Byu2400	[X]Malignant neoplasm/ill-defined sites within resp system	Feb-09
87003	1	0	0	0	BBLC100	"[M]Mesenchymoma, malignant"	Feb-09
87113	0	0	0	0	B54X.00	"Malignant neoplasm-pluriglandular involvement,unspecified"	Feb-09
87335	314	15	0	0	B624.12	Hairy cell leukaemia	Feb-09
88022	28	0	0	0	ByuC400	[X]Secondary malignant neoplasm/oth+unspcfd digestive organs	Feb-09
88144	5	0	0	0	B52y.00	Malignant neoplasm of other specified part of nervous system	Feb-09
88362	5	0	0	0	B08y.00	Malignant neoplasm of other specified hypopharyngeal site	Feb-09
89230	0	0	0	0	BBj9.00	[M]Hodgkin's granuloma	Feb-09
89258	5	0	0	0	B524200	"Malignant neoplasm of peripheral nerve of low limb, incl hip"	Feb-09
89329	7	0	0	0	ByuD800	[X]Other specified leukaemias	Feb-09

For peer review only - http://bmjopen.bmj.com/site/about/guidelines.xhtml

89593	7	0	0	0	B151200	Malignant neoplasm of intrahepatic biliary passages	Feb-09
89657	1	0	0	0	B626z00	Malignant mast cell tumour NOS	Feb-09
89762	1	0	0	0	ByuD700	[X]Other monocytic leukaemia	Feb-09
89909	9	0	0	0	B003200	"Malignant neoplasm of lower lip, mucosa"	Feb-09
89916	1	0	0	0	B553100	Malignant neoplasm of presacral region	Feb-09
90124	6	0	0	0	B067.00	Malignant neoplasm of posterior wall of oropharynx	Feb-09
90201	9	0	0	0	B62x000	T-zone lymphoma	Feb-09
90290	16	0	0	0	B18y700	Malignant neoplasm of mesentery	Feb-09
90482	2	0	0	0	P103z11	Thoracolumbar spina bifida with hydrocephalus - closed	Feb-09
90487	6	0	0	0	BBb3.11	[M]Subependymal astrocytoma NOS	Feb-09
90610	8	1	0	0	B002300	"Malignant neoplasm of upper lip, oral aspect"	Feb-09
90659	13	0	0	0	B54y.00	Malignant neoplasm of other specified endocrine gland	Feb-09
91035	2	0	0	0	B010z00	Malignant neoplasm of fixed part of tongue NOS	Feb-09
91037	6	0	0	0	B06yz00	Malignant neoplasm of other specified site of oropharynx NOS	Feb-09
91240	15	0	0	0	B517300	Malignant neoplasm of pons	Feb-09
91457	13	0	0	0	Byu5900	"[X]Malignant neoplasm/connective + soft tissue,unspecified"	Feb-09
91509	13	0	0	0	B471z00	Malignant neoplasm of descended testis NOS	Feb-09
91586	9	0	0	0	B311400	Malignant neoplasm of connective and soft tissue of finger	Feb-09
91600	P113200	8	0	0	0	Thoracic spinal meningocele	Feb-09
91842	3	0	0	0	BB69z00	[M]Sebaceous adenoma or adenocarcinoma NOS	Feb-09

For peer review only - http://bmjopen.bmj.com/site/about/guidelines.xhtml

91843	9	0	0	0	B003100	"Malignant neoplasm of lower lip, frenulum"	Feb-09
91895	4	0	0	0	B064100	Malignant neoplasm of glossoepiglottic fold	Feb-09
91900	5	0	0	0	B61z400	Hodgkin's disease NOS of lymph nodes of axilla and arm	Feb-09
92068	5	0	0	0	B620300	Nodular lymphoma of intra-abdominal lymph nodes	Feb-09
92245	1	0	0	0	B613200	"Hodgkin's, lymphocytic-histiocytic pred intrathoracic nodes"	Feb-09
92329	11	1	0	0	B48yz00	Malignant neoplasm of other male genital organ NOS	Feb-09
92371	5	0	0	0	B304300	Malignant neoplasm of radius	Feb-09
92380	1	0	0	0	B602500	Burkitt's lymphoma of lymph nodes of inguinal region and leg	Feb-09
92382	1	0	0	0	B308B00	Malignant neoplasm of fourth metatarsal bone	Feb-09
92720	3	0	0	0	B243.00	Malignant neoplasm of posterior mediastinum	Feb-09
93133	0	1	0	0	PJ22.00	"Trisomy 18, translocation"	Feb-09
93175	2	0	0	0	BBZ2.11	[M]Intraosseous carcinoma	Feb-09
93177	2	0	0	0	PG52100	Osteopetrosis - congenita type	Feb-09
93218	2	0	0	0	B03z.00	Malignant neoplasm of gum NOS	Feb-09
93342	4	0	0	0	B66z.00	Monocytic leukaemia NOS	Feb-09
93352	15569	886	0	0	B338.00	Squamous cell carcinoma of skin	Feb-09
93372	F103.00	3	0	0	0	Cerebral degeneration in diseases EC	Feb-09
93380	108	1	0	0	C10N100	Cystic fibrosis related diabetes mellitus	Feb-09
93478	4	0	0	0	B138.00	"Malignant neoplasm, overlapping lesion of colon"	Feb-09
93537	3	0	0	0	B517200	Malignant neoplasm of midbrain	Feb-09
93561	8	0	0	0	P601z00	Congenital atresia of pulmonary valve NOS	Feb-09

For peer review only - http://bmjopen.bmj.com/site/about/guidelines.xhtml

93665	6	0	0	0	Byu5300	"[X]Kaposi's sarcoma, unspecified"	Feb-09
93717	3	0	0	0	BBD7.00	"[M]Extra-adrenal paraganglioma, NOS"	Feb-09
93762	6	0	0	0	B42..00	Malignant neoplasm of placenta	Feb-09
93778	2	0	0	0	B1z1z00	Malignant neoplasm of spleen NOS	Feb-09
93842	1	0	0	0	B062300	Malignant neoplasm of palatopharyngeal arch	Feb-09
93902	3	0	0	0	P103.00	Spina bifida with hydrocephalus - closed	Feb-09
93951	3	0	0	0	B613500	"Hodgkin's, lymphocytic-histiocytic pred inguinal and leg"	Feb-09
93963	13	0	0	0	F101600	Sandhoff disease	Feb-09
94005	38	0	0	0	B615z00	"Hodgkin's disease, mixed cellularity NOS"	Feb-09
94083	1	0	0	0	BB5P.00	[M]Solid carcinoma NOS	Feb-09
94174	9	0	0	0	B67y.00	Other and unspecified leukaemia	Feb-09
94220	2	0	0	0	B540100	Malignant neoplasm of adrenal medulla	Feb-09
94239	1	0	0	0	BBp1.00	[M]Mast cell sarcoma	Feb-09
94251	2	0	0	0	B00z100	"Malignant neoplasm of lip, unspecified, lipstick area"	Feb-09
94267	19	0	0	0	BBb3.00	[M]Subependymal glioma	Feb-09
94278	25	0	0	0	B110111	Malignant neoplasm of gastro-oesophageal junction	Feb-09
94279	1	0	0	0	B61z700	Hodgkin's disease NOS of spleen	Feb-09
94286	4	0	0	0	BBG8.11	[M]Congenital fibrosarcoma	Feb-09
94355	3	0	0	0	B55y200	Malignant neoplasm of flank NOS	Feb-09
94390	3	0	0	0	B070.00	Malignant neoplasm of roof of nasopharynx	Feb-09
94407	3	0	0	0	B615100	"Hodgkin's mixed cellularity of lymph nodes head, face, neck"	Feb-09
94415	2	0	0	0	B623100	"Malignant histiocytosis of lymph nodes head, face and neck"	Feb-09
94427	1	0	0	0	B305C00	Malignant neoplasm of fifth metacarpal bone	Feb-09

For peer review only - http://bmjopen.bmj.com/site/about/guidelines.xhtml

94438	3	0	0	0	BB85z00	[M]Signet ring carcinoma NOS	Feb-09
94441	3	0	0	0	B003300	"Malignant neoplasm of lower lip, oral aspect"	Feb-09
94467	1	0	0	0	P0z..00	Anencephalus and similar anomalies NOS	Feb-09
94486	H593.00	1020	0	2	0	Chronic type 2 respiratory failure	Feb-09
94533	4	0	0	0	RyuC000	[X]Sudden infant death syndrome	Feb-09
94597	3	0	0	0	ZV10611	[V]Personal history of lymphoid leukaemia	Feb-09
94598	P115100	2	0	0	0	Cervical myelocele	Feb-09
94776	6	0	0	0	B1z2.00	"Malignant neoplasm, overlapping lesion of digestive system"	Feb-09
94793	1Z1B.00	2761	0	21	0	Chronic kidney disease stage 3 with proteinuria	Feb-09
94810	1	0	0	0	BBB4.00	[M]Adenocarcinoma with spindle cell metaplasia	Feb-09
94873	25	2	0	0	BB2A.13	[M]Squamous cell carcinoma of skin NOS	Feb-09
94925	140	2	0	0	Pyu9D00	[X]Primary ciliary dyskinesia	Feb-09
94935	2	0	14	0	4M21.00	Lymphoma stage II	Feb-09
94946	H592.00	210	0	0	0	Chronic type 1 respiratory failure	Feb-09
94965	1Z15.00	19544	0	31	1	Chronic kidney disease stage 3A	Feb-09
94975	4	0	0	0	B241300	Malignant neoplasm of pericardium	Feb-09
94995	5	0	0	0	B620500	Nodular lymphoma of lymph nodes of inguinal region and leg	Feb-09
95008	1	0	0	0	BB82112	[M]Gelatinous adenocarcinoma	Feb-09
95016	2	0	0	0	B0z1.00	Malignant neoplasm of Waldeyer's ring	Feb-09
95024	4	0	0	0	BBG8.00	[M]Infantile fibrosarcoma	Feb-09
95049	4	0	0	0	B616000	Hodgkin's lymphocytic depletion of unspecified site	Feb-09
95057	3	0	0	0	B34y000	Malignant neoplasm of ectopic site of female breast	Feb-09
95058	1	0	0	0	B600700	Reticulosarcoma of spleen	Feb-09

For peer review only - http://bmjopen.bmj.com/site/about/guidelines.xhtml

95122	1Z1H.00	1243	0	50	0	Chronic kidney disease stage 4 with proteinuria	Feb-09
95123	1Z1C.00	10576	0	6	0	Chronic kidney disease stage 3 without proteinuria	Feb-09
95145	1Z1B.11	67	0	0	0	CKD stage 3 with proteinuria	Feb-09
95150	4	0	0	0	BB81B00	[M]Serous surface papillary carcinoma	Feb-09
95175	1Z1E.00	11978	0	15	0	Chronic kidney disease stage 3A without proteinuria	Feb-09
95176	1Z1E.11	103	0	0	0	CKD stage 3A without proteinuria	Feb-09
95177	1Z1G.00	4533	0	23	0	Chronic kidney disease stage 3B without proteinuria	Feb-09
95178	1Z1F.00	1288	0	20	0	Chronic kidney disease stage 3B with proteinuria	Feb-09
95179	1Z16.00	7458	0	47	0	Chronic kidney disease stage 3B	Feb-09
95180	1Z1F.11	32	0	1	0	CKD stage 3B with proteinuria	Feb-09
95182	3	0	0	0	B308100	Malignant neoplasm of talus	Feb-09
95188	1Z1C.11	175	0	0	0	CKD stage 3 without proteinuria	Feb-09
95323	1	0	0	0	B35z000	Malignant neoplasm of ectopic site of male breast	Feb-09
95338	2	2	0	0	B613600	"Hodgkin's, lymphocytic-histiocytic pred intrapelvic nodes"	Feb-09
95373	3	0	0	0	BBCC100	"[M]Leydig cell tumour, malignant"	Feb-09
95405	1Z1L.00	142	0	1	0	Chronic kidney disease stage 5 without proteinuria	Feb-09
95406	1Z1J.00	1558	0	43	0	Chronic kidney disease stage 4 without proteinuria	Feb-09
95408	1Z1D.00	1692	0	17	0	Chronic kidney disease stage 3A with proteinuria	Feb-09
95421	13	0	0	0	B45y.00	Malignant neoplasm of other specified female genital organ	Feb-09
95429	7	0	0	0	B071.00	Malignant neoplasm of posterior wall of nasopharynx	Feb-09

For peer review only - http://bmjopen.bmj.com/site/about/guidelines.xhtml

95458	8	0	0	0	B300300	Malignant neoplasm of nasal bone	Feb-09
95478	P117200	1	0	0	0	Thoracic spina bifida without hydrocephalus - open	Feb-09
95480	9	0	0	0	B001100	"Malignant neoplasm of lower lip, lipstick area"	Feb-09
95505	6	0	0	0	B41y000	Malignant neoplasm of cervical stump	Feb-09
95508	1Z1K.00	360	0	5	0	Chronic kidney disease stage 5 with proteinuria	Feb-09
95571	1Z1D.11	25	0	0	0	CKD stage 3A with proteinuria	Feb-09
95609	6	0	0	0	BB5B300	"[M]Insulinoma, malignant"	Feb-09
95615	1	0	0	0	F150.11	Infantile spinal muscular atrophy	Feb-09
95629	6	0	0	0	B325500	Malignant melanoma of perineum	Feb-09
95630	1	0	0	0	B62x600	True histiocytic lymphoma	Feb-09
95644	4	0	0	0	B241.00	Malignant neoplasm of heart	Feb-09
95671	13	0	0	0	Byu5700	"[X]Malignant neoplasm of peritoneum, unspecified"	Feb-09
95715	31	0	0	0	B627900	Mucosa-associated lymphoma	Feb-09
95772	9	1	0	0	B051000	Malignant neoplasm of upper buccal sulcus	Feb-09
95783	11	0	0	0	B17yz00	Malignant neoplasm of specified site of pancreas NOS	Feb-09
95792	9	0	0	0	B62zz00	Lymphoid and histiocytic malignancy NOS	Feb-09
95818	4	0	0	0	BBD1.00	"[M]Paraganglioma, malignant"	Feb-09
95972	835	3	0	0	F207.00	Relapsing and remitting multiple sclerosis	Feb-09
96003	4	0	0	0	B055000	Malignant neoplasm of junction of hard and soft palate	Feb-09
96094	40	0	0	0	B119.00	Siewert type III adenocarcinoma	Feb-09
96183	1	0	0	0	BBj4.00	"[M]Hodgkin's disease,lymphocytic depletion,diffuse fibrosis"	Apr-09
96226	4	0	0	0	ByuC100	[X]Malignant neoplasm/overlap lesion/other+ill-defined sites	Apr-09
96231	8	0	0	0	BBGJ.00	"[M]Fibroxanthoma, malignant"	Apr-09

For peer review only - http://bmjopen.bmj.com/site/about/guidelines.xhtml

96246	280	2	0	0	F208.00	Secondary progressive multiple sclerosis	Apr-09
96256	671	13	0	0	F37y100	Axonal sensorimotor neuropathy	Apr-09
96291	19	0	0	0	F204.00	Benign multiple sclerosis	Apr-09
96429	3	0	0	0	B470z00	Malignant neoplasm of undescended testis NOS	Apr-09
96445	0	0	0	0	B300B00	Malignant neoplasm of turbinate	Apr-09
96494	10	0	0	0	BB5Tz00	[M]Papillary adenoma or adenocarcinoma NOS	Apr-09
96515	1	0	0	0	BBP2.00	"[M]Fibrous mesothelioma, benign"	Apr-09
96585	1	0	0	0	B32y000	Overlapping malignant melanoma of skin	Apr-09
96607	164	2	0	0	F206.00	Primary progressive multiple sclerosis	Apr-09
96635	3	0	0	0	B17y000	Malignant neoplasm of ectopic pancreatic tissue	Apr-09
96637	4	0	0	0	790M400	Biventricular repair of hypoplastic left heart syndrome	Apr-09
96709	P110200	3	0	0	0	Thoracic spina bifida without mention of hydrocephalus	Apr-09
96751	1	0	0	0	AyuCB00	"[X]HIV disease result/haematological+immunologic abnorms,NEC"	Apr-09
96782	4	0	0	0	B003z00	"Malignant neoplasm of lower lip, inner aspect NOS"	Apr-09
96783	3	0	0	0	B005.00	Malignant neoplasm of commissure of lip	Apr-09
96798	1	0	0	0	BBd2.12	[M]Meningothelial sarcoma	May-09
96802	8	0	0	0	B11y100	Malignant neoplasm of posterior wall of stomach NEC	May-09
96860	F11x900	42	0	4	0	Cerebral degeneration in Parkinson's disease	May-09
96869	4	1	0	0	B071z00	Malignant neoplasm of posterior wall of nasopharynx NOS	May-09
96893	4	0	0	0	BBrA300	[M]Myeloid sarcoma	Jun-09
97059	64	4	0	0	PKyz700	Angelman's syndrome	Jul-09

For peer review only - http://bmjopen.bmj.com/site/about/guidelines.xhtml

97091	6	0	0	0	ByuC500	[X]2ndry malignant neoplasm/bladder+oth+unsp urinary organs	Aug-09
97092	4	0	0	0	F100100	Schulz's disease	Aug-09
97332	11	0	0	0	B213z00	Malignant neoplasm of laryngeal cartilage NOS	Aug-09
97422	F11x.00	1	0	0	0	Cerebral degeneration in other disease EC	Aug-09
97454	3	1	0	0	F427K11	Lipofuscinosis NEC	Aug-09
97463	4	0	0	0	BBF4.00	[M]Giant cell sarcoma (except of bone)	Aug-09
97499	51	0	0	0	B118.00	Siewert type II adenocarcinoma	Aug-09
97530	7	0	0	0	B051100	Malignant neoplasm of lower buccal sulcus	Aug-09
97547	5	0	0	0	B551200	Malignant neoplasm of intrathoracic site NOS	Aug-09
97577	1	0	0	0	B602300	Burkitt's lymphoma of intra-abdominal lymph nodes	Sep-09
97587	1Z1J.11	47	0	2	0	CKD stage 4 without proteinuria	Sep-09
97593	6	0	0	0	BBZG.00	"[M]Ameloblastoma, malignant"	Sep-09
97683	1Z1L.11	13	0	0	0	CKD stage 5 without proteinuria	Sep-09
97746	8	0	0	0	B61z800	Hodgkin's disease NOS of lymph nodes of multiple sites	Oct-09
97751	6	2	0	0	PG51600	Osteogenesis imperfecta type IV	Oct-09
97832	5	0	0	0	B58y211	Secondary cancer of the cervix	Oct-09
97852	3	0	0	0	BBk7.00	"[M]Malignant lymphoma, centroblastic type, follicular"	Oct-09
97863	2	0	0	0	B615000	"Hodgkin's disease, mixed cellularity of unspecified site"	Oct-09
97875	3	0	0	0	B175.00	"Malignant neoplasm, overlapping lesion of pancreas"	Oct-09
97916	1	0	0	0	Q007111	Fetal alcohol syndrome	Oct-09
97996	8	0	0	0	B44y.00	Malignant neoplasm of other site of uterine adnexa	Nov-09

For peer review only - http://bmjopen.bmj.com/site/about/guidelines.xhtml

98009	0	0	0	0	BBrA312	[M]Granulocytic sarcoma	Nov-09
98020	G558z00	2	0	0	0	"Cardiomyopathy in diseases EC, NOS"	Nov-09
98104	4	0	0	0	B23y.00	Malignant neoplasm of other specified pleura	Dec-09
98142	29	0	0	0	B107.00	Siewert type I adenocarcinoma	Dec-09
98298	1	0	0	0	P100z00	Spina bifida with hydrocephalus NOS	Dec-09
98322	1	0	0	0	BBT7100	"[M]Haemangioendothelioma, malignant"	Jan-10
98361	3	0	0	0	Byu5B00	[X]Kaposi's sarcoma of other sites	Jan-10
98483	2	0	0	0	BBZN.11	[M]Odontogenic fibrosarcoma	Feb-10
98500	5	0	0	0	B002200	"Malignant neoplasm of upper lip, mucosa"	Feb-10
98537	0	0	0	0	B201100	Malignant neoplasm of tympanic cavity	Feb-10
98559	1	0	0	0	BBW8.00	"[M]Chondroblastoma, malignant"	Feb-10
98596	19	0	0	0	ByuD200	[X]Other types of diffuse non-Hodgkin's lymphoma	Mar-10
98617	7	0	0	0	PKyz711	Angelman syndrome	Mar-10
98662	1	0	0	0	PG51400	Osteogenesis imperfecta type II	Mar-10
98740	4	0	0	0	B000z00	"Malignant neoplasm of upper lip, vermilion border NOS"	Mar-10
98753	7	0	0	0	P228300	Aicardi syndrome	Mar-10
98781	1	0	0	0	BB5F.00	[M]Trabecular adenocarcinoma	Mar-10
98797	3	0	0	0	BBLD.00	[M]Embryonal sarcoma	Mar-10
98800	2	0	0	0	BBbG.12	[M]Piloid astrocytoma	Mar-10
98811	1	0	0	0	P102.00	Spina bifida with hydrocephalus - open	Mar-10
98825	2	0	0	0	BB5B600	[M]Mixed islet cell and exocrine adenocarcinoma	Mar-10
98840	1	0	0	0	B610300	Hodgkin's paragranuloma of intra-abdominal lymph nodes	Mar-10
98883	4	0	0	0	BB9D.00	[M]Medullary carcinoma with lymphoid stroma	Apr-10

For peer review only - http://bmjopen.bmj.com/site/about/guidelines.xhtml

98909	2	0	0	0	B611100	"Hodgkin's granuloma of lymph nodes of head, face and neck"	Apr-10
98911	2	0	0	0	B200100	Malignant neoplasm of nasal conchae	Apr-10
98961	1	0	0	0	BBk2.00	"[M]Malignant lymphoma, centroblastic-centrocytic, follicular"	Apr-10
99001	4	1	0	0	B002100	"Malignant neoplasm of upper lip, frenulum"	Apr-10
99012	1	0	0	0	B61z500	Hodgkin's disease NOS of lymph nodes inguinal region and leg	Apr-10
99015	1	0	0	0	B66y.00	Other monocytic leukaemia	Apr-10
99096	1	0	0	0	Byu2300	[X]Malignant neopl/overlapping les/resp+intrathoracic organs	Apr-10
99160	1Z1K.11	18	0	0	0	CKD stage 5 with proteinuria	Apr-10
99185	1	0	0	0	B062100	Malignant neoplasm of glossopalatine fold	Apr-10
99200	1	0	0	0	BBj7.00	"[M]Hodgkin's disease, nodular sclerosis, cellular phase"	Apr-10
99240	1	0	0	0	B600z00	Reticulosarcoma NOS	Apr-10
99257	4	0	0	0	B324z00	Malignant melanoma of scalp and neck NOS	May-10
99281	P117.00	2	0	0	0	Spina bifida without hydrocephalus - open	May-10
99299	P114200	3	0	0	0	Thoracic meningomyelocele	May-10
99312	1Z1H.11	86	0	0	0	CKD stage 4 with proteinuria	May-10
99332	P114000	1	0	0	0	Meningomyelocele of unspecified site	May-10
99386	3	0	0	0	B073200	Malignant neoplasm posterior margin nasal septum and choanae	May-10
99413	5	0	0	0	B67yz00	Other and unspecified leukaemia NOS	May-10
99493	2	0	0	0	B002.00	"Malignant neoplasm of upper lip, inner aspect"	May-10
99511	1	0	0	0	B574200	Secondary malignant neoplasm of ileum	May-10

For peer review only - http://bmjopen.bmj.com/site/about/guidelines.xhtml

99572	1	0	0	0	B312500	Malignant neoplasm of connective and soft tissue of toe	May-10
99621	3	2	0	0	B520.00	Malignant neoplasm of cranial nerves	Jun-10
99655	3	0	0	0	BBg6.00	[M]Lymphosarcoma NOS	Jun-10
99665	3	0	0	0	BBV..11	[M]Juxtacortical osteogenic sarcoma	Jun-10
99702	2	0	0	0	BBn3.00	"[M]Plasma cell tumour, malignant"	Jun-10
99711	7	0	0	0	C302511	Oculocerebrorenal syndrome	Jun-10
99797	1	0	0	0	BBX3.00	[M]Malignant giant cell tumour of soft parts	Jun-10
99887	2	0	0	0	B60y.00	Other specified reticulosarcoma or lymphosarcoma	Jun-10
99896	3	0	0	0	B12y.00	Malignant neoplasm of other specified site small intestine	Jun-10
99913	1	0	0	0	B510300	Malignant neoplasm of globus pallidus	Jun-10
99951	3	0	0	0	B60z.00	Reticulosarcoma or lymphosarcoma NOS	Jun-10
100002	7	0	0	0	B062z00	Malignant neoplasm of tonsillar fossa NOS	Jun-10
100006	3	0	0	0	B602200	Burkitt's lymphoma of intrathoracic lymph nodes	Jun-10
100035	71	6	0	0	1J0L.00	Suspected malignant mesothelioma	Jul-10
100083	123	1	0	0	B546.00	Neuroblastoma	Jul-10
100111	1	0	0	0	BB46.00	[M]Schneiderian carcinoma	Jul-10
100144	6	0	0	0	B004300	"Malignant neoplasm of lip, oral aspect"	Jul-10
100175	2	0	0	0	PKyE.00	Barber-Say syndrome	Jul-10
100267	4	0	0	0	BBZG.11	"[M]Adamantinoma, malignant"	Jul-10
100296	9	0	0	0	B582100	Secondary malignant neoplasm of skin of face	Jul-10
100352	2	0	0	0	B601500	Lymphosarcoma of lymph nodes of inguinal region and leg	Jul-10
100375	3	0	0	0	BBP4.00	"[M]Epithelioid mesothelioma, benign"	Aug-10
100423	1	0	0	0	B610100	"Hodgkin's paragranuloma of lymph nodes of head, face, neck"	Aug-10

For peer review only - http://bmjopen.bmj.com/site/about/guidelines.xhtml

100430	155	1	0	0	66k..00	Cystic fibrosis monitoring	Aug-10
100544	1	0	0	0	BBh2.00	"[M]Reticulosarcoma, nodular"	Aug-10
100582	P20z100	1	0	0	0	Encephalocele of other specified site	Aug-10
100584	3	0	0	0	B110000	Malignant neoplasm of cardiac orifice of stomach	Aug-10
100610	6	0	0	0	C370400	Arthropathy in cystic fibrosis	Sep-10
100615	1	0	0	0	B626500	Mast cell malignancy of lymph nodes inguinal region and leg	Sep-10
100622	1	0	0	0	P51z.11	Transposition of arterial trunk NEC	Sep-10
100625	1	0	0	0	BB5R500	"[M]Carcinoid tumour, nonargentaffin, malignant"	Sep-10
100633	1Z1G.11	33	0	0	0	CKD stage 3B without proteinuria	Sep-10
100721	1	0	0	0	B002z00	"Malignant neoplasm of upper lip, inner aspect NOS"	Sep-10
100733	4	0	0	0	B51yz00	Malignant neoplasm of other part of brain NOS	Sep-10
100786	5	0	0	0	B651000	Chronic eosinophilic leukaemia	Nov-10
100900	497	32	0	0	B927.12	Neurofibromatosis type 1	Nov-10
100906	1	0	0	0	B00z000	"Malignant neoplasm of lip, unspecified, external"	Nov-10
100918	1	0	0	0	B073z00	Malignant neoplasm of anterior wall of nasopharynx NOS	Nov-10
100927	1	0	0	0	BBr4z00	[M]Erythroleukaemia NOS	Nov-10
101013	P110000	1	0	0	0	"Spina bifida without hydrocephalus, site unspecified"	Dec-10
101052	57	1	0	0	B929.00	Neurofibromatosis type 2	Dec-10
101086	1	0	0	0	B520z00	Malignant neoplasm of cranial nerves NOS	Dec-10
101114	96	1	0	0	B627A00	Diffuse non-Hodgkin's large cell lymphoma	Dec-10
101222	2	0	0	0	F151111	Juvenile spinal muscular atrophy	Dec-10
101312	P20..11	2	0	0	0	Hydroencephalocele	Jan-11

For peer review only - http://bmjopen.bmj.com/site/about/guidelines.xhtml

101342	1	0	0	0	BBD3.00	[M]Parasympathetic paraganglioma	Jan-11
101429	1	0	0	0	BBj0.11	"[M]Lymphogranuloma, malignant"	Jan-11
101530	9	0	0	0	B616z00	"Hodgkin's disease, lymphocytic depletion NOS"	Jan-11
101606	1	0	0	0	B662.00	Subacute monocytic leukaemia	Feb-11
101608	7	0	0	0	B4A1100	Malignant neoplasm of ureteropelvic junction	Feb-11
101668	4	0	0	0	Byu5400	"[X]Malignant neoplasm/peripheral nerves of trunk,unspecified"	Feb-11
101707	7	0	0	0	B001z00	"Malignant neoplasm of lower lip, vermilion border NOS"	Feb-11
101715	1	0	0	0	B616700	"Hodgkin's disease, lymphocytic depletion of spleen"	Feb-11
101753	1	0	0	0	B03y.00	Malignant neoplasm of other sites of gum	Feb-11
101778	10	0	0	0	B442.00	Malignant neoplasm of broad ligament	Feb-11
101805	2	0	0	0	B507000	Malignant neoplasm of lacrimal sac	Feb-11
101885	4	0	0	0	B241400	Mesothelioma of pericardium	Mar-11
101923	1	0	0	0	BBJ1.11	[M]Fibroliposarcoma	Mar-11
101988	1	0	0	0	B060100	Malignant neoplasm of palatine tonsil	Apr-11
102102	1	0	0	0	PJ12.11	Partial trisomy 13 in Patau's syndrome	Apr-11
102117	1	0	0	0	AyuC300	[X]HIV disease resulting in multiple infections	Apr-11
102142	2	0	0	0	B013000	Malignant neoplasm of anterior 2/3 of tongue ventral surface	Apr-11
102145	3	0	0	0	B322100	Malignant melanoma of external auditory meatus	Apr-11
102151	1	0	0	0	B060200	Malignant neoplasm of overlapping lesion of tonsil	Apr-11
102171	4	0	0	0	C304400	Sulphite oxidase deficiency	Apr-11
102205	2	0	0	0	B072z00	Malignant neoplasm of lateral wall of nasopharynx NOS	May-11

For peer review only - http://bmjopen.bmj.com/site/about/guidelines.xhtml

102252	2	0	0	0	AyuCC00	[X]HIV disease resulting in other specified conditions	May-11
102338	7	0	0	0	F21y600	Vanishing white matter disease	Jun-11
102593	1	0	0	0	BB96.00	[M]Noninfiltrating intraductal papillary adenocarcinoma	Jul-11
102594	553	1	0	0	B627E00	Diffuse large B-cell lymphoma	Jul-11
102628	P104.00	2	0	0	0	Spina bifida with hydrocephalus of late onset	Jul-11
102688	3	0	0	0	ByuD400	[X]Other malignant immunoproliferative diseases	Aug-11
102783	1	0	0	0	B651200	Chronic neutrophilic leukaemia	Aug-11
102848	1	0	0	0	D200200	Constitutional aplastic anaemia with malformation	Aug-11
102921	3	0	0	0	F10y200	PEHO syndrome	Sep-11
102922	1	0	0	0	C370800	Cystic fibrosis related cirrhosis	Sep-11
102949	6	0	0	0	B312000	Malignant neoplasm of connective and soft tissue of hip	Sep-11
102980	1	0	0	0	P511200	Incomplete great vessel transposition	Sep-11
103017	1	0	0	0	PG51200	Osteogenesis imperfecta - unclassifiable	Sep-11
103034	2	0	0	0	BB5j500	"[M]Endometrioid adenofibroma, malignant"	Sep-11
103139	4	0	0	0	C375400	"Mucopolysaccharidosis, type IV"	Oct-11
103224	9	0	0	0	C370500	Cystic fibrosis with distal intestinal obstruction syndrome	Oct-11
103245	1	0	0	0	B601700	Lymphosarcoma of spleen	Oct-11
103284	1	0	0	0	P102300	Lumbar spina bifida with hydrocephalus - open	Nov-11
103354	2	0	0	0	B308z00	Malignant neoplasm of short bones of leg NOS	Dec-11
103527	P202.00	1	0	0	0	Hydromeningocele - cranial	Jan-12
103582	53	0	0	0	PJ33600	Chromosome 22q11 deletion syndrome	Jan-12
103645	1	0	0	0	B66yz00	Other monocytic leukaemia NOS	Jan-12

For peer review only - http://bmjopen.bmj.com/site/about/guidelines.xhtml

103646	1	0	0	0	PG52200	Osteopetrosis - tarda type	Jan-12
103708	1	0	0	0	BBJ6.00	[M]Round cell liposarcoma	Feb-12
103722	54	1	0	0	F390500	Congenital myopathy	Feb-12
103796	2	0	0	0	B051.00	Malignant neoplasm of vestibule of mouth	Feb-12
103873	1	0	0	0	PJ20.00	"Trisomy 18, meiotic nondisjunction"	Mar-12
103883	1	0	0	0	BBc9100	"[M]Retinoblastoma, undifferentiated type"	Mar-12
103891	1	0	0	0	C375.13	Hurler's syndrome	Mar-12
103900	3	0	0	0	B626000	Mast cell malignancy of unspecified site	Mar-12
103946	2	0	0	0	B220100	Malignant neoplasm of mucosa of trachea	Apr-12
103995	2	0	0	0	B545z00	Malignant neoplasm of aortic body or paraganglia NOS	May-12
104025	1	0	0	0	B337600	Malignant neoplasm of skin of heel	May-12
104054	4	1	0	0	PKyL.00	FG syndrome	May-12
104128	6	0	0	0	B31z000	Kaposi's sarcoma of soft tissue	May-12
104134	1	0	0	0	AyuC400	[X]HIV disease resulting/other infectious+parasitic diseases	May-12
104147	0	0	0	0	BBQ4.11	[M]Infantile embryonal carcinoma	Jun-12
104152	727	8	0	0	B628.00	Follicular lymphoma	Jun-12
104239	333	0	0	0	Q48F.00	Hypoxic ischaemic encephalopathy of newborn	Jun-12
104291	327	7	0	0	B61..11	Hodgkin lymphoma	Jun-12
104324	123	1	0	0	B595.00	Malignant tumour of unknown origin	Jun-12
104325	121	0	0	0	B640000	B-cell acute lymphoblastic leukaemia	Jun-12
104328	118	2	0	0	B641000	B-cell chronic lymphocytic leukaemia	Jun-12
104386	118	0	0	0	B62F000	Small cell B-cell lymphoma	Jun-12
104391	136	0	0	0	B627.11	Non-Hodgkin lymphoma	Jun-12
104412	8	0	0	0	B62F200	Lymphoblastic (diffuse) lymphoma	Jun-12
104475	1	0	0	0	B692.00	Subacute myelomonocytic leukaemia	Jul-12

For peer review only - http://bmjopen.bmj.com/site/about/guidelines.xhtml

104480	1	0	0	0	B58y800	Secondary malignant neoplasm of epididymis and vas deferens	Jul-12
104484	15	0	0	0	B61C.00	Other classical Hodgkin lymphoma	Jul-12
104619	K053.00	13582	0	30	0	Chronic kidney disease stage 3	Jul-12
104620	253	0	0	0	B62F100	Mantle cell lymphoma	Jul-12
104717	1	0	0	0	A789311	HIV disease resulting in Pneumocystis jirovecii pneumonia	Aug-12
104720	17	0	0	0	BBP3.11	[M]Sarcomatoid mesothelioma	Aug-12
104743	1	0	0	0	B613800	"Hodgkin's, lymphocytic-histiocytic pred of multiple sites"	Aug-12
104788	9	1	0	0	B654.00	Acute myeloblastic leukaemia	Aug-12
104790	1	0	0	0	B601800	Lymphosarcoma of lymph nodes of multiple sites	Aug-12
104862	20	0	0	0	B62E300	Cutaneous T-cell lymphoma	Aug-12
104895	16	0	0	0	B617.00	Nodular lymphocyte predominant Hodgkin lymphoma	Aug-12
104934	2	0	0	0	B62Ew00	Other mature T/NK-cell lymphoma	Sep-12
104939	10	0	0	0	B64y500	Adult T-cell lymphoma/leukaemia (HTLV-1-associated)	Sep-12
104963	K054.00	1181	0	14	0	Chronic kidney disease stage 4	Sep-12
104973	1	0	0	0	BB62z00	[M]Apocrine adenoma or adenocarcinoma NOS	Sep-12
104999	2	0	0	0	C375z00	Mucopolysaccharidosis NOS	Sep-12
105020	6	0	0	0	B628300	Follicular lymphoma grade 3a	Sep-12
105035	3	0	0	0	F211.00	Schilder's disease	Oct-12
105038	15	0	0	0	B627G00	Mediastinal (thymic) large B-cell lymphoma	Oct-12
105069	3	0	0	0	B693.00	Juvenile myelomonocytic leukaemia	Oct-12
105073	1	0	0	0	BBN2.00	"[M]Synovial sarcoma, spindle cell type"	Oct-12
105083	13	0	0	0	B62D.00	Histiocytic sarcoma	Oct-12
105085	36	0	0	0	B62E.00	T/NK-cell lymphoma	Oct-12
105095	17	0	0	0	B628100	Follicular lymphoma grade 2	Oct-12

For peer review only - http://bmjopen.bmj.com/site/about/guidelines.xhtml

105151	K055.00	326	0	0	0	Chronic kidney disease stage 5	Nov-12
105166	1	0	0	0	BBDB.11	[M]Glomoid sarcoma	Nov-12
105186	10	0	0	0	P26..00	Disorder of neuronal migration and differentiation	Nov-12
105203	1	0	0	0	B620200	Nodular lymphoma of intrathoracic lymph nodes	Nov-12
105275	1	0	0	0	BBV..13	[M]Periosteal osteogenic sarcoma	Dec-12
105296	7	0	0	0	BBTD200	"[M]Haemangiopericytoma, malignant"	Dec-12
105324	1	0	0	0	A789800	HIV disease resulting in multiple malignant neoplasms	Dec-12
105335	5	0	0	0	B62A.00	Sarcoma of dendritic cells	Dec-12
105375	3	0	0	0	B62E800	Blastic NK-cell lymphoma	Dec-12
105388	97	0	0	0	B498.00	Local recurrence of malignant tumour of urinary bladder	Dec-12
105472	1	0	0	0	B614700	"Hodgkin's disease, nodular sclerosis of spleen"	Dec-12
105475	1	0	0	0	B308.00	Malignant neoplasm of short bones of leg	Dec-12
105488	133	1	0	0	B36..00	Local recurrence of malignant tumour of breast	Dec-12
105559	17	0	0	0	B62E100	"Anaplastic large cell lymphoma, ALK-positive"	Jan-13
105613	3	0	0	0	B161300	Malignant neoplasm of sphincter of Oddi	Jan-13
105636	20	0	0	0	B62E900	Angioimmunoblastic T-cell lymphoma	Jan-13
105679	24	1	0	0	F25G.11	Dravet syndrome	Jan-13
105709	3	0	0	0	B62E600	Enteropathy-associated T-cell lymphoma	Jan-13
105797	1	0	0	0	B304100	Malignant neoplasm of acromion	Feb-13
105841	16	0	0	0	B618.00	Nodular sclerosis classical Hodgkin lymphoma	Mar-13
105889	15	0	0	0	B628000	Follicular lymphoma grade 1	Mar-13
105925	1	0	0	0	B62E700	Subcutaneous panniculitic T-cell lymphoma	Apr-13
105944	1	0	0	0	BBK3300	[M]Mixed cell rhabdomyosarcoma	Apr-13

For peer review only - http://bmjopen.bmj.com/site/about/guidelines.xhtml

105955	12	0	0	0	B62E200	"Anaplastic large cell lymphoma, ALK-negative"	Apr-13
105956	9	0	0	0	C315200	Kearns-Sayre syndrome	Apr-13
105957	6	0	0	0	B651100	"Chronic myeloid leukaemia, BCR/ABL positive"	Apr-13
106012	8	0	0	0	Q48F.11	Perinatal hypoxic - ischaemic encephalopathy	Apr-13
106063	2	0	0	0	B628700	Other types of follicular lymphoma	May-13
106069	1	0	0	0	B305.11	Malignant neoplasm of carpal bones	May-13
106114	3	0	0	0	PJ51400	Trisomy 9p syndrome	May-13
106134	1	0	0	0	BBdB.00	[M]Meningeal sarcomatosis	May-13
106137	1	0	0	0	BBh..00	[M]Reticulosarcomas	May-13
106194	1	0	0	0	B231.00	Malignant neoplasm of visceral pleura	May-13
106197	1	0	0	0	BBr7000	[M]Basophilic leukaemia	May-13
106349	3	0	0	0	B61z.11	Hodgkin lymphoma NOS	Jun-13
106432	200	0	0	0	C370900	Exacerbation of cystic fibrosis	Jun-13
106483	1	0	0	0	BBr6200	[M]Subacute myeloid leukaemia	Aug-13
106519	79	0	0	0	BB1P.00	[M]Non-small cell carcinoma	Aug-13
106569	2	0	0	0	B500200	Malignant neoplasm of crystalline lens	Aug-13
106579	1	0	0	0	Pyu0400	[X]Unspecified spina bifida with hydrocephalus	Aug-13
106597	3	0	0	0	B61B.00	Lymphocyte-rich classical Hodgkin lymphoma	Aug-13
106858	6	0	0	0	1JW..00	Suspected cystic fibrosis	Aug-13
106867	4	0	0	0	B62F.11	Non-follicular lymphoma	Aug-13
106884	6	0	0	0	B62F.00	Nonfollicular lymphoma	Aug-13
106911	1	0	0	0	B613700	"Hodgkin's, lymphocytic-histiocytic predominance of spleen"	Aug-13
106915	1	0	0	0	B073100	Malignant neoplasm of nasopharyngeal soft palate surface	Aug-13
106924	8	0	0	0	B641200	Clinical stage B chronic lymphocytic leukaemia	Aug-13

For peer review only - http://bmjopen.bmj.com/site/about/guidelines.xhtml

106926	6	0	0	0	PKyP.11	Wolfram syndrome	Aug-13
106969	2	0	0	0	B628500	Diffuse follicle centre lymphoma	Sep-13
106970	2	0	0	0	BBk3.00	"[M]Malig lymphoma, lymphocytic, well differentiated,nodular"	Sep-13
107017	9	0	0	0	B641011	Chronic lymphocytic leukaemia of B-cell type	Sep-13
107032	1	0	0	0	B616800	Hodgkin's lymphocytic depletion lymph nodes multiple sites	Sep-13
107052	44	0	0	0	B641100	Clinical stage A chronic lymphocytic leukaemia	Oct-13
107126	3	0	0	0	B553200	Malignant neoplasm of sacrococcygeal region	Oct-13
107144	P114100	3	0	0	0	Cervical meningomyelocele	Oct-13
107162	1	0	0	0	PJ22.11	Partial trisomy 18 in Edward's syndrome	Oct-13
107163	4	0	0	0	B641300	Clinical stage C chronic lymphocytic leukaemia	Oct-13
107166	5	0	0	0	B628200	Follicular lymphoma grade 3	Oct-13
107236	9	0	0	0	B651300	"Atypical chronic myeloid leukaemia, BCR/ABL negative"	Nov-13
107258	1	0	0	0	B011100	Malignant neoplasm of midline of tongue	Nov-13
107299	1	0	0	0	BB5D700	[M]Combined hepatocellular carcinoma and cholangiocarcinoma	Nov-13
107377	P116100	1	0	0	0	Cervical myelocystocele	Dec-13
107643	5	0	0	0	B64y400	T-cell prolymphocytic leukaemia	Jan-14
107773	1	0	0	0	BBr8z00	[M]Eosinophilic leukaemia NOS	Mar-14
107804	1	0	0	0	B61z300	Hodgkin's disease NOS of intra-abdominal lymph nodes	Mar-14
107807	1	0	0	0	AyuC100	[X]HIV disease resulting in other viral infections	Mar-14
107840	1	0	0	0	C308100	Multiple acyl-CoA dehydrogenase deficiencies	Mar-14

For peer review only - http://bmjopen.bmj.com/site/about/guidelines.xhtml

107878	1	0	0	0	B213200	Malignant neoplasm of cuneiform cartilage	Apr-14
107884	1	0	0	0	BBba000	[M]Peripheral neuroectodermal tumour	Apr-14
107916	1	0	0	0	B201000	Malignant neoplasm of auditory (Eustachian) tube	Apr-14
107949	2	0	0	0	B62E500	Hepatosplenic T-cell lymphoma	Apr-14
107973	7	0	0	0	B628400	Follicular lymphoma grade 3b	May-14
108021	1	0	0	0	790Jz00	Other repair of transposition of great arteries NOS	May-14
108041	17	1	0	0	C308200	X-linked adrenoleucodystrophy	May-14
108054	4	1	0	0	A789511	HIV disease resulting in Kaposi sarcoma	May-14
108131	2	0	0	0	F146.00	Early onset cerebellar ataxia with hypogonadism	Jun-14
108182	1	0	0	0	B627400	Diffuse non-Hodgkin's small cleaved cell (diffuse) lymphoma	Jun-14
108316	2	0	0	0	BBrAz00	[M]Miscellaneous leukaemia NOS	Jul-14
108363	71	1	0	0	B509.00	Malignant melanoma of eye	Jul-14
108389	1	0	0	0	B310500	Malignant neoplasm soft tissues of cervical spine	Jul-14
108424	5	0	0	0	B663.00	Acute monoblastic leukaemia	Jul-14
108591	4	0	0	0	K01w100	Drash syndrome	Sep-14
108623	2	0	0	0	C372.11	Lesch - Nyhan syndrome	Sep-14
108638	1	0	0	0	B305A00	Malignant neoplasm of third metacarpal bone	Sep-14
108652	1	0	0	0	PyuAD00	[X]Li-Fraumeni syndrome	Sep-14
108656	3	1	0	0	B64y300	B-cell prolymphocytic leukaemia	Sep-14
108667	2	0	0	0	B1z1000	Angiosarcoma of spleen	Sep-14
108682	1	0	0	0	BBgJ.11	[M]Germinoblastic sarcoma NOS	Oct-14
108715	1	0	0	0	B66..11	Histiocytic leukaemia	Oct-14
108719	6	0	0	0	B628600	Cutaneous follicle centre lymphoma	Oct-14
108773	F103z00	1	0	0	0	Cerebral degeneration in disease NOS	Oct-14

For peer review only - http://bmjopen.bmj.com/site/about/guidelines.xhtml

108775	1	0	0	0	B619.00	Mixed cellularity classical Hodgkin lymphoma	Oct-14
108886	1	0	0	0	B615500	Hodgkin's mixed cellularity of lymph nodes inguinal and leg	Nov-14
108922	1	0	0	0	K01w112	Wilms' tumour + nephrotic syndrome + pseudohermaphroditism	Dec-14
108947	1	0	0	0	PKyz.11	Cockayne's syndrome	Dec-14
108964	2	0	0	0	BBr6900	[M]Juvenile myelomonocytic leukaemia	Dec-14
109002	2	0	0	0	B325400	Malignant melanoma of perianal skin	Dec-14
109110	5	0	0	0	BBD9.11	[M]Chromaffin paraganglioma	Jan-15
109223	1	0	0	0	PJ50311	Trisomy 9 Mosaic Syndrome	Jan-15
109243	1	0	0	0	P100100	Cervical spina bifida with hydrocephalus	Feb-15
109243	P100100	1	0	0	0	Cervical spina bifida with hydrocephalus	Feb-15
109288	11	1	0	0	A411000	Sporadic Creutzfeldt-Jakob disease	Feb-15
109394	1	0	0	0	P00y.00	Other specified anencephalus	Mar-15
109473	0	0	0	0	B521200	Malignant neoplasm of cerebral pia mater	Apr-15
109608	2	0	0	0	C300A00	Congenital Fanconi syndrome	May-15
109657	1Z1Y.00	92	0	0	0	CKD with GFR category G3b & albuminuria category A2	May-15
109714	1	0	0	0	ByuDD00	[X]Oth and unspecif peripheral & cutaneous T-cell lymphomas	Jun-15
109745	2	0	0	0	B32G.00	Malignant melanoma stage IV M1a	Jun-15
109780	1	0	0	0	B62E400	"Extranodal NK/T-cell lymphoma, nasal type"	Jun-15
109804	1Z1T.00	390	0	0	0	CKD with GFR category G3a & albuminuria category A1	Jun-15
109805	1Z1V.00	115	0	1	0	CKD with GFR category G3a & albuminuria category A2	Jun-15
109827	4	0	0	0	B329.00	Malignant melanoma stage IB	Jul-15
109868	2	0	0	0	C327300	Wolman disease	Jul-15
109904	1Z1b.00	26	0	0	0	CKD with GFR category G4 & albuminuria category A2	Jul-15

For peer review only - http://bmjopen.bmj.com/site/about/guidelines.xhtml

109905	1Z1W.00	25	0	0	0	CKD with GFR category G3a & albuminuria category A3	Jul-15
109963	1Z1X.00	160	0	0	0	CKD with GFR category G3b & albuminuria category A1	Jul-15
109980	1Z1a.00	36	0	0	0	CKD with GFR category G4 & albuminuria category A1	Aug-15
109981	1Z1e.00	1	0	0	0	CKD with GFR category G5 & albuminuria category A2	Aug-15
109990	1Z1Z.00	28	0	0	0	CKD with GFR category G3b & albuminuria category A3	Aug-15
110058	2	0	0	0	ZV10613	[V]Personal history of myeloid leukaemia	Sep-15
110133	1Z1d.00	2	0	0	0	CKD with GFR category G5 & albuminuria category A1	Sep-15
110139	4	0	0	0	B32A.00	Malignant melanoma stage IIA	Sep-15
110147	1	0	0	0	BB5D711	[M]Hepatocholangiocarcinoma	Sep-15
110169	1	0	0	0	PKyD.00	Nicolaides-Baraitser syndrome	Oct-15
110180	1	0	0	0	B32B.00	Malignant melanoma stage IIB	Oct-15
110349	1	0	0	0	BBr3z00	[M]Plasma cell leukaemia NOS	Dec-15
110433	1	0	0	0	B574100	Secondary malignant neoplasm of jejunum	Jan-16
110454	1	0	0	0	C370700	Liver disease due to cystic fibrosis	Jan-16
110467	1Z1f.00	9	0	0	0	CKD with GFR category G5 & albuminuria category A3	Jan-16
110483	2	0	0	0	B32H.00	Malignant melanoma stage IV M1b	Jan-16
110563	1	0	0	0	B616500	Hodgkin's lymphocytic depletion lymph nodes inguinal and leg	Feb-16
110626	1Z1c.00	13	0	0	0	CKD with GFR category G4 & albuminuria category A3	Mar-16
110749	2	0	0	0	K01w200	Congenital nephrotic syndrome with focal glomerulosclerosis	Apr-16
110766	2	0	0	0	B521000	Malignant neoplasm of cerebral dura mater	May-16

For peer review only - http://bmjopen.bmj.com/site/about/guidelines.xhtml

110775	1	0	0	0	B151100	Malignant neoplasm of interlobular biliary canals	May-16
110833	2	0	0	0	PJ50400	Trisomy 10	May-16
110838	1	0	0	0	B676.00	Acute erythroid leukaemia	May-16
110903	1	0	0	0	B623800	Malignant histiocytosis of lymph nodes of multiple sites	Jun-16
110961	1	0	0	0	B32F.00	Malignant melanoma stage IIIC	Jun-16
110993	1	0	0	0	B305800	Malignant neoplasm of first metacarpal bone	Jul-16
111036	1	0	0	0	C375.18	Sanfilippo's syndrome	Jul-16
111077	2	0	0	0	C300800	Juvenile nephropathic cystinosis	Jul-16
111079	4	0	0	0	B328.00	Malignant melanoma stage IA	Jul-16
111113	1	0	0	0	BBj3.00	"[M]Hodgkin's disease, lymphocytic depletion NOS"	Jul-16
111120	3	0	0	0	PJ33113	18q deletion syndrome	Jul-16
111162	2	0	0	0	B32J.00	Malignant melanoma stage IV M1c	Jul-16
111172	1	0	0	0	BB5R300	"[M]Carcinoid tumour, argentaffin, malignant"	Aug-16
111238	1	0	0	0	BB5dz00	[M]Mixed cell adenoma or adenocarcinoma NOS	Aug-16
111289	1	0	0	0	B002000	"Malignant neoplasm of upper lip, buccal aspect"	Sep-16
111311	1	0	0	0	B317.00	"Malignant neoplasm, overlap lesion connective & soft tissue"	Sep-16
111370	1	0	0	0	K01wz00	Congenital nephrotic syndrome NOS	Oct-16
111413	2	0	0	0	B32C.00	Malignant melanoma stage IIC	Oct-16
111426	1	0	0	0	B308900	Malignant neoplasm of second metatarsal bone	Oct-16
46733	D104700	148	0	0	0	Beta major thalassaemia	Feb-09
21643	D104811	655	0	8	2	Beta thalassaemia	Feb-09
45151	D104011	11	0	0	0	Thalassaemia major - Cooley's anaemia	Feb-09
31405	D104000	103	0	5	0	Thalassaemia major NEC	Feb-09

For peer review only - http://bmjopen.bmj.com/site/about/guidelines.xhtml

Appendix 2 ICD 10 Codes to identify a child with a Life-limiting condition

A17

A81.0

A81.1

B20

B21

B22

B23

B24

C00

C01

C02

C03

C04

C05

C06

C07

C08

C09

C10

For peer review only - http://bmjopen.bmj.com/site/about/guidelines.xhtml

C11

C12

C13

C14

C15

C16

C17

C18

C19

C20

C21

C22

C23

C24

C25

C26

C30

C31

C32

C33

For peer review only

C34

C35

C36

C37

C38

C39

C40

C41

C43

C44

C45

C46

C47

C48

C49

C50

C51

C52

C53

C54

For peer review only

C55

C56

C57

C58

C59

C60

C61

C62

C63

C64

C65

C66

C67

C68

C69

C70

C71

C72

C73

C74

For peer review only

For peer review only - http://bmjopen.bmj.com/site/about/guidelines.xhtml

C75

C76

C77

C78

C79

C80

C81

C82

C83

C84

C85

C86

C87

C88

C89

C90

C91

C92

C93

C94

For peer review only

C95

C96

C97

D61.9

D70

D76.1

D81

D82.1

D83

D89.1

E31.0

E34.8

E70.2

E71

E72

E74

E75

E76

E77

E79.1

For peer review only

E83.0

E84

E88.0

E88.1

"F80.3

"

F84.2

G10

G11.1

G11.3

G12

G20

G23.0

G23.8

G31.8

G31.9

G35

G40.4

G40.5

G60.0

For peer review only - http://bmjopen.bmj.com/site/about/guidelines.xhtml

G60.1

G70.2

G70.9

G71.0

G71.1

G71.2

G71.3

G80.0

G80.8

G82.3

G82.4

G82.5

G93.4

G93.6

G93.7

H11.1

H49.8

H35.5

I21

I27.0

For peer review only

I42

I61.3

I81

J84.1

J96

J98.4

K55.0

K55.9

K72

K74

K76.5

K86.8

M31.3

M32.1

M89.5

N17

N18

N19

N25.8

P10.1

For peer review only - http://bmjopen.bmj.com/site/about/guidelines.xhtml

For peer review only

P11.2

P21.0

P28.5

P29.0

P29.3

P35.0

P35.1

P35.8

P37.1

Q00.0

Q01

Q03.1

Q03.9

Q04.0

Q04.2

Q04.3

Q04.4

Q04.6

Q04.9

Q21.8

For peer review only

Q22.0

Q22.1

Q22.4

Q22.5

Q22.6

Q23.0

Q23.4

Q23.9

Q25.4

Q39.6

Q41.0

Q41.9

Q43.7

Q44.2

Q74.8

Q44.5

Q44.7

Q60.1

Q60.6

Q78.0

For peer review only - http://bmjopen.bmj.com/site/about/guidelines.xhtml

Q78.5

Q79.2

Q79.3

Q80.4

Q81

Q82.1

Q82.4

Q85.8

Q86.0

Q93.2

Q93.3

Q93.4

Q93.5

Q93.8

Q95.2

T86.0

T86.2

Appendix 3 Read codes to identify a child with a chronic, not life-limiting condition

5600	F172.00	2402	0	130	0	[X] Horners syndrome	Feb-09

For peer review only - http://bmjopen.bmj.com/site/about/guidelines.xhtml

24753	ZS91.12	143	0	5	0	[X]Attention deficit disorder	Feb-09
26285	Eu9y700	2865	0	197	0	[X]Attention deficit disorder	Feb-09
34929	Eu50100	402	0	11	0	[X]Atypical anorexia nervosa	Feb-09
24044	Eu84100	418	0	5	0	[X]Atypical autism	Feb-09
33863	Eu50300	258	0	6	0	[X]Atypical bulimia nervosa	Feb-09
24062	Eu84111	16	0	0	0	[X]Atypical childhood psychosis	Feb-09
91547	Eu20311	4	0	0	0	[X]Atypical schizophrenia	Feb-09
9982	Eu84011	13172	0	379	0	[X]Autistic disorder	Feb-09
51375	Eu84511	9	0	0	0	[X]Autistic psychopathy	Feb-09
42941	Eu84z11	2488	0	11	0	[X]Autistic spectrum disorder	Feb-09
23713	Eu31400	117	0	7	0	"[X]Bipol aff disord, curr epis sev depress, no psychot symp"	Feb-09
28277	Eu31200	153	0	1	0	[X]Bipolar affect disorder cur epi manic with psychotic symp	Feb-09
26299	Eu31100	65	0	0	0	[X]Bipolar affect disorder cur epi manic wout psychotic symp	Feb-09
104065	Eu31800	15	0	0	0	[X]Bipolar affective disorder type I	May-12
103915	Eu31900	262	0	1	0	[X]Bipolar affective disorder type II	Mar-12
44693	Eu31600	124	0	4	0	"[X]Bipolar affective disorder, current episode mixed"	Feb-09
27584	Eu31700	1499	0	1	0	"[X]Bipolar affective disorder, currently in remission"	Feb-09
33751	Eu31z00	624	0	4	0	"[X]Bipolar affective disorder, unspecified"	Feb-09
9581	Eu50200	10720	0	551	4	[X]Bulimia nervosa	Feb-09
72688	Myu1.00	37	0	0	0	[X]Bullous disorders	Feb-09
3637	Eu84000	978	0	21	0	[X]Childhood autism	Feb-09
53820	PyuA.00	12	0	0	0	"[X]Chromosomal abnormalities, not elswhere classified"	Feb-09
543	Eu32z11	738902	0	19968	0	[X]Depression NOS	Feb-09
3291	Eu32z12	16570	0	445	0	[X]Depressive disorder NOS	Feb-09

For peer review only - http://bmjopen.bmj.com/site/about/guidelines.xhtml

6514	Eu80z00	399	0	3	0	[X]Developmental disorder of speech and language unspecified	Feb-09
52212	Cyu2.00	193	0	1	0	[X]Diabetes mellitus	Feb-09
36946	Eu50z00	2380	0	108	0	"[X]Eating disorder, unspecified"	Feb-09
100899	Eu85.00	2066	0	20	0	[X]Global developmental delay	Nov-10
33505	Eu90100	91	0	1	0	[X]Hyperkinetic conduct disorder	Feb-09
45799	Eu90111	8	0	2	0	[X]Hyperkinetic disorder associated with conduct disorder	Feb-09
50015	Eu90z00	79	0	3	0	"[X]Hyperkinetic disorder, unspecified"	Feb-09
97421	Eu90z11	0	0	0	0	[X]Hyperkinetic reaction of childhood or adolescence NOS	Aug-09
96770	Eu90z12	4	0	0	0	[X]Hyperkinetic syndrome NOS	Apr-09
50337	Eu84012	30	0	0	0	[X]Infantile autism	Feb-09
4477	Eu81z11	15741	0	569	0	[X]Learning disability NOS	Feb-09
98346	Eu32500	512	0	2	0	"[X]Major depression, mild"	Jan-10
98252	Eu32600	1500	0	11	0	"[X]Major depression, moderately severe"	Dec-09
98417	Eu32800	323	0	3	0	"[X]Major depression, severe with psychotic symptoms"	Jan-10
98414	Eu32700	1948	0	17	0	"[X]Major depression, severe without psychotic symptoms"	Jan-10
28962	Eu7..00	394	0	1	0	[X]Mental retardation	Feb-09
34174	Eu84112	102	0	2	0	[X]Mental retardation with autistic features	Feb-09
98342	Eu81400	5806	0	21	0	[X]Moderate learning disability	Jan-10
98293	Eu81500	3549	0	24	0	[X]Severe learning disability	Dec-09
36143	Eu72.00	2449	0	1	0	[X]Severe mental retardation	Feb-09
22048	PKy6500	68	0	2	0	Aarskog syndrome	Feb-09
102328	PJyy.11	1	0	0	0	Absence of sex chromosome	Jun-11
72655	P74z100	3	0	0	0	Absence of superior vena cava	Feb-09
8097	2828	7568	0	1399	0	Absence seizure	Feb-09

For peer review only - http://bmjopen.bmj.com/site/about/guidelines.xhtml

71109	PK11.00	3	0	0	0	Absent adrenal gland	Feb-09
25089	PKyz100	4	0	0	0	Acardia	Feb-09
57804	PG41.13	15	0	0	0	Achondroplastic dwarf	Feb-09
69916	C152100	3	0	0	0	Acquired adrenogenital syndrome	Feb-09
91774	G341300	19	0	0	0	Acquired atrioventricular fistula of heart	Feb-09
101375	SN51000	47	0	1	0	Acquired C1 esterase inhibitor deficiency	Jan-11
46025	D307.00	37	0	2	0	Acquired coagulation factor deficiency	Feb-09
37587	D307z00	25	0	0	0	Acquired coagulation factor deficiency NOS	Feb-09
63195	J690100	67	0	1	0	Acquired coeliac disease	Feb-09
4675	F113.00	811	0	60	0	Acquired communicating hydrocephalus	Feb-09
31667	M145300	230	0	16	0	Acquired epidermolysis bullosa	Feb-09
49889	ZS82.00	2	0	0	0	Acquired epileptic aphasia	Feb-09
46546	D307200	15	0	0	0	Acquired factor II deficiency	Feb-09
107206	C300600	5	0	1	0	Acquired Fanconi syndrome	Nov-13
50495	D112z12	8	0	0	0	Acquired haemolytic anaemia with haemoglobinuria NEC	Feb-09
27771	D11..00	152	0	9	1	Acquired haemolytic anaemias	Feb-09
105061	C353500	4	0	0	0	Acquired hypophosphataemia	Oct-12
103789	D307212	0	0	0	0	Acquired hypoprothrombinaemia	Feb-12
3290	C04..00	160727	0	878	7	Acquired hypothyroidism	Feb-09
23639	M211600	136	0	4	0	Acquired ichthyosis	Feb-09
23770	A788.00	1182	0	53	8	Acquired immune deficiency syndrome	Feb-09
110481	K081000	1	0	0	0	Acquired nephrogenic diabetes insipidus	Jan-16
63815	J106500	9	0	0	0	Acquired oesophagocoele	Feb-09
43824	D307211	44	0	2	0	Acquired prothrombin deficiency	Feb-09

For peer review only - http://bmjopen.bmj.com/site/about/guidelines.xhtml

69269	D20X.00	7	0	0	0	"Acquired pure red cell aplasia, unspecified"	Feb-09
104079	K132.11	476	0	127	0	Acquired renal cystic disease	May-12
41699	D210100	8	0	0	0	Acquired sideroblastic anaemia	Feb-09
29933	D11z000	116	0	2	0	Acquired spherocytosis	Feb-09
41840	PF55000	53	0	3	0	Acrocephalosyndactyly (Apert)	Feb-09
48272	PG01.00	8	0	0	0	Acrocephaly	Feb-09
5316	C130200	2971	0	245	1	Acromegaly	Feb-09
62353	PF5E200	32	0	0	0	Acrosyndactyly	Feb-09
11147	C134411	141	0	5	0	ACTH deficiency	Feb-09
43641	C153.11	4	0	0	0	ACTH overproduction	Feb-09
38968	P6z2.00	48	0	0	0	Acyanotic congenital heart disease NOS	Feb-09
59166	PF47.11	8	0	1	0	Adactyly	Feb-09
5524	PG51.13	7	0	0	0	Adair-Dighton syndrome	Feb-09
4042	C154011	239	0	38	0	Addisonian crisis	Feb-09
12227	C154600	668	0	18	0	Addisonian crisis	Feb-09
2813	D010.11	53	0	4	0	Addison's anaemia	Feb-09
4481	C154100	5819	0	428	2	Addison's disease	Feb-09
110474	44lz.00	0	0	0	1	Additional biochemical ratios	Jan-16
9665	PJz1.00	9	0	0	0	Additional chromosome NOS	Feb-09
72804	C392600	3	0	0	0	Adenosine deaminase deficiency	Feb-09
57552	C30yy11	10	0	0	0	Adenosine-deaminase deficiency	Feb-09
1523	F4K4600	2858	0	130	0	Adie's pupil syndrome	Feb-09
42873	C154012	66	0	3	0	Adrenal crisis	Feb-09
41542	C15z.00	215	0	7	0	Adrenal gland disorder NOS	Feb-09
28896	C154z11	74	0	2	0	Adrenal hypofunction	Feb-09
20786	C154z12	444	0	14	0	Adrenal insufficiency NEC	Feb-09
73529	C155000	3	0	0	0	Adrenal medullary insufficiency	Feb-09
32642	C153.12	39	0	0	0	Adrenocortical hyperfunction	Feb-09
57321	C152z00	7	0	0	0	Adrenogenital disorder NOS	Feb-09
31446	C152811	123	0	4	0	Adrenogenital syndrome NOS	Feb-09

For peer review only - http://bmjopen.bmj.com/site/about/guidelines.xhtml

35682	C390.11	148	0	8	0	Agammaglobulinaemia	Feb-09
62598	C390900	2	0	0	0	Agammaglobulinaemia NEC	Feb-09
65511	PB33.11	1	0	0	0	Aganglionic macrocolon	Feb-09
94813	PB3..11	1	0	0	0	Aganglionosis	Feb-09
23457	P220.00	121	0	11	0	"Agenesis of brain, part unspecified"	Feb-09
54877	P22A011	4	0	0	0	Agenesis of cerebellum	Feb-09
40777	P227000	19	0	0	0	Agenesis of cerebrum	Feb-09
67919	P362000	10	0	2	0	Agenesis of cilia	Feb-09
39486	P228011	211	0	2	0	Agenesis of corpus callosum	Feb-09
60930	PF37.00	10	0	0	0	Agenesis of fibula	Feb-09
47576	PB63011	9	0	0	0	Agenesis of gallbladder	Feb-09
56350	PF21z11	14	0	0	0	Agenesis of hand	Feb-09
59444	P853.00	17	0	0	0	Agenesis of lung	Feb-09
93911	P2xz000	3	0	0	0	Agenesis of nerve NEC	Feb-09
67476	P2x0.00	6	0	0	0	"Agenesis of nerve, unspecified"	Feb-09
58710	P22z.12	6	0	0	0	Agenesis of part of brain NEC	Feb-09
53723	P223.00	4	0	0	0	Agyria	Feb-09
40029	PB63500	118	0	2	0	Alagille syndrome	Feb-09
66720	C30y000	24	0	0	2	Alaninaemia	Feb-09
50325	PG52.11	8	0	0	0	Albers - Schonberg syndrome	Feb-09
15624	C302700	956	0	41	0	Albinism	Feb-09
53954	C302.11	25	0	0	0	Albinism	Feb-09
31388	C302712	57	0	4	0	Albino	Feb-09
30601	PG5y011	42	0	3	0	Albright-McCune-Sternberg syndrome	Feb-09
57407	PG5y012	15	0	4	0	Albright's polyostotic dysplasia	Feb-09
15046	PG5y000	35	0	0	0	Albright-Sternberg syndrome	Feb-09
52688	C151.11	70	0	4	0	Aldosteronism	Feb-09
2290	H330.11	13983	0	430	3	Allergic asthma	Feb-09
21232	H33zz12	182	0	0	0	Allergic asthma NEC	Feb-09
1037	M240.00	107630	0	9254	24	Alopecia	Feb-09

For peer review only - http://bmjopen.bmj.com/site/about/guidelines.xhtml

685	M240100	44959	0	2328	2	Alopecia areata	Feb-09
43109	M240900	25	0	1	0	Alopecia disseminata	Feb-09
50780	M240700	3	0	0	0	Alopecia febrilis	Feb-09
48735	M240C00	50	0	3	0	Alopecia follicularis	Feb-09
40947	M240J00	8	0	0	0	Alopecia hereditaria	Feb-09
48719	M240H00	35	0	0	0	Alopecia seborrhoeica	Feb-09
52294	M240600	24	0	0	0	Alopecia senilis	Feb-09
976	M240000	5590	0	250	1	Alopecia unspecified	Feb-09
8866	D104300	848	0	19	0	Alpha thalassaemia	Feb-09
12235	D104400	3655	0	30	114	Alpha trait thalassaemia	Feb-09
3019	C376200	2675	0	117	64	Alpha-1-antitrypsin deficiency	Feb-09
25589	C376100	167	0	8	9	Alpha-1-antitrypsin hepatitis	Feb-09
71522	C327400	14	0	2	0	Alpha-galactosidase A deficiency	Feb-09
24395	PKy9000	592	0	32	0	Alport's syndrome	Feb-09
64601	D014000	3	0	0	0	Amino-acid deficiency anaemia	Feb-09
46357	C300z00	3	0	4	4	Amino-acid transport disorder NOS	Feb-09
32466	46T6.11	18	0	0	11	Aminoaciduria	Feb-09
107595	C373H00	13	0	0	0	Amyloid A amyloidosis	Jan-14
105651	G558400	14	0	0	0	Amyloid cardiomyopathy	Jan-13
30667	G557000	139	0	4	0	Amyloid heart disease	Feb-09
36697	F38y.11	20	0	0	0	Amyotonia congenita	Feb-09
31439	N232000	170	0	5	0	Amyotrophia NOS	Feb-09
57769	PGyy000	5	0	2	2	Amyotrophica congenita	Feb-09
31944	C310412	108	0	2	0	Andersen's disease	Feb-09
24660	C327413	52	0	1	0	Anderson-Fabry disease	Feb-09
93886	C327412	2	0	0	0	Anderson's disease	Feb-09
49161	C1z5.11	145	0	6	0	Androgen insensitivity syndrome	Feb-09
61407	PJ5z.11	8	0	0	0	Aneuploidy NEC	Feb-09
2184	N100.00	35726	0	2753	12	Ankylosing spondylitis	Feb-09
25292	PK1..00	181	0	19	0	Anomalies of adrenal gland	Feb-09
36064	PK1z.00	23	0	0	0	Anomalies of adrenal gland NOS	Feb-09
28318	P22A.00	132	0	3	0	Anomalies of cerebellum	Feb-09

For peer review only - http://bmjopen.bmj.com/site/about/guidelines.xhtml

39724	P227.00	15	0	1	0	Anomalies of cerebrum	Feb-09
32468	P228.00	143	0	7	0	Anomalies of corpus callosum	Feb-09
31416	PG6..00	171	0	7	0	Anomalies of diaphragm	Feb-09
42684	P74..00	34	0	0	0	Anomalies of great veins	Feb-09
46471	P229.00	17	0	0	0	Anomalies of hypothalamus	Feb-09
30055	PB7z.00	45	0	0	0	Anomalies of pancreas NOS	Feb-09
39588	PK27.00	23	0	3	0	Anomalies of parathyroid gland NEC	Feb-09
32424	PK24.00	191	0	12	1	Anomalies of pituitary gland	Feb-09
50786	P76C.00	34	0	1	0	Anomalies of renal artery NEC	Feb-09
45429	PG1z.00	55	0	1	0	Anomalies of spine NOS	Feb-09
26338	PK0..00	151	0	12	0	Anomalies of spleen	Feb-09
59625	PK0z.00	21	0	0	0	Anomalies of spleen NOS	Feb-09
40030	P72..11	103	0	16	0	Anomalies of the aorta excluding coarction	Feb-09
66465	PG3z.11	3	0	0	0	Anomalies of thoracic cage unspecified	Feb-09
39366	PK28.00	37	0	1	0	Anomalies of thymus	Feb-09
57704	PK26.00	45	0	4	0	Anomalies of thyroglossal duct NEC	Feb-09
10638	PK25.00	271	0	44	0	Anomalies of thyroid gland NEC	Feb-09
55080	PAzz.00	5	0	0	0	Anomalies of upper alimentary tract NOS	Feb-09
30315	PD7..00	55	0	0	0	Anomalies of urachus	Feb-09
56889	PD7z.00	2	0	0	0	Anomalies of urachus NOS	Feb-09
103432	P6z1000	2	0	0	0	Anomalous atrial bands	Dec-11
25147	G567.00	224	0	1	0	Anomalous atrioventricular excitation	Feb-09
42803	G567z00	3	0	0	0	Anomalous atrioventricular excitation NOS	Feb-09
109236	P6z1.00	1	0	0	0	Anomalous bands of heart	Jan-15
31373	P6y4400	12	0	1	0	Anomalous coronary artery communication	Feb-09
67286	P721000	1	0	0	0	Anomalous origin of the aortic arch	Feb-09

For peer review only - http://bmjopen.bmj.com/site/about/guidelines.xhtml

101307	P743.00	3	0	0	0	Anomalous portal vein termination	Jan-11
105445	ZR1N.00	3	0	0	0	Anomalous sentences repetition test	Dec-12
63677	P742.11	5	0	0	0	Anomalous termination of right pulmonary vein	Feb-09
64778	P6z1100	8	0	0	0	Anomalous ventricular bands	Feb-09
62230	P720.00	37	0	1	0	"Anomaly of aorta, unspecified"	Feb-09
42389	P761.00	79	0	7	1	Anomaly of artery NEC	Feb-09
44253	P22Az00	19	0	0	0	Anomaly of cerebellum NOS	Feb-09
111258	P227z00	2	0	0	0	Anomaly of cerebrum NOS	Aug-16
60648	P228z00	9	0	0	0	Anomaly of corpus callosum NOS	Feb-09
45291	P740.00	21	0	1	0	"Anomaly of great veins, unspecified"	Feb-09
63530	P831.00	10	0	0	0	Anomaly of laryngeal and tracheal cartilage	Feb-09
55062	P831z00	7	0	0	0	Anomaly of laryngeal or tracheal cartilage NOS	Feb-09
63453	P860.00	16	0	0	0	"Anomaly of lung, unspecified"	Feb-09
100681	PK27z00	3	0	0	0	Anomaly of parathyroid gland NEC NOS	Sep-10
70576	PK24z00	25	0	0	0	Anomaly of pituitary gland NOS	Feb-09
97401	P76Cz00	4	0	0	0	Anomaly of renal artery NEC NOS	Aug-09
60066	PG10.00	29	0	1	0	"Anomaly of spine, unspecified"	Feb-09
74890	P740000	8	0	0	0	"Anomaly of the pulmonary veins, unspecified"	Feb-09
50886	P740100	30	0	0	0	"Anomaly of the vena cava, unspecified"	Feb-09
48842	PK28z00	7	0	0	0	Anomaly of thymus gland NOS	Feb-09
47467	P831200	50	0	6	0	Anomaly of thyroid cartilage	Feb-09
70581	PK25z00	12	0	0	0	Anomaly of thyroid gland NEC NOS	Feb-09
37358	PA10.00	36	0	3	0	"Anomaly of tongue, unspecified"	Feb-09
31020	P831300	13	0	1	0	Anomaly of tracheal cartilage	Feb-09

For peer review only - http://bmjopen.bmj.com/site/about/guidelines.xhtml

23605	P30..00	108	0	6	1	Anophthalmos	Feb-09
37557	P30z.00	6	0	0	0	Anophthalmos NOS	Feb-09
70742	P300z00	2	0	0	0	Anophthalmos NOS	Feb-09
2135	E271.00	26924	0	1771	0	Anorexia nervosa	Feb-09
21889	F15..00	209	0	11	0	Anterior horn cell disease	Feb-09
58729	F15z.00	3	0	0	0	Anterior horn cell disease NOS	Feb-09
43908	C134z11	28	0	0	0	Anterior pituitary hormone deficiency NEC	Feb-09
43347	G121.11	193	0	7	0	Aortic incompetence - rheumatic	Feb-09
1007	G541200	2670	0	168	0	"Aortic incompetence alone, cause unspecified"	Feb-09
14998	G541000	3238	0	146	0	"Aortic incompetence, non-rheumatic"	Feb-09
58810	G541211	5	0	0	0	"Aortic insufficiency alone, cause unspecified"	Feb-09
47887	G541011	10	0	1	0	"Aortic insufficiency, non-rheumatic"	Feb-09
7963	G121.12	909	0	67	3	Aortic regurgitation - rheumatic	Feb-09
1005	G541212	7183	0	121	0	"Aortic regurgitation alone, cause unspecified"	Feb-09
10187	G541012	4918	0	146	0	"Aortic regurgitation, non-rheumatic"	Feb-09
98277	G71A.00	486	0	21	0	Aortic root dilatation	Dec-09
93699	791C000	14	0	0	0	Aortic root replac us pul val auto ri vent pulm art val cond	Feb-09
71668	791C400	196	0	0	0	Aortic root replacement	Feb-09
70353	791C200	26	0	0	0	Aortic root replacement using homograft	Feb-09
91004	791C300	58	0	1	0	Aortic root replacement using mechanical prosthesis	Feb-09
37405	P50..11	31	0	5	0	Aortic septal defect	Feb-09
45505	P501.00	17	0	0	0	Aortic septal defect	Feb-09

For peer review only - http://bmjopen.bmj.com/site/about/guidelines.xhtml

9591	G541500	27546	0	463	0	Aortic stenosis	Feb-09
2343	G541300	14659	0	932	5	"Aortic stenosis alone, cause unspecified"	Feb-09
999	G541100	18331	0	1242	1	"Aortic stenosis, non-rheumatic"	Feb-09
49185	G541700	357	0	2	0	Aortic valve calcification	Feb-09
4548	G541.00	10916	0	644	6	Aortic valve disorders	Feb-09
30610	G541600	2196	0	11	0	Aortic valve sclerosis	Feb-09
10964	G541400	456	0	13	0	Aortic valve stenosis with insufficiency	Feb-09
65330	P501.12	2	0	0	0	Aorticopulmonary septal defect	Feb-09
37402	PF55.11	66	0	32	0	Apert's syndrome	Feb-09
95752	PK15.00	1	0	0	0	Aplasia of adrenal gland	Feb-09
59298	P722100	1	0	0	0	Aplasia of aorta	Feb-09
100305	P221.00	2	0	0	0	"Aplasia of brain, part unspecified"	Jul-10
91846	P22A200	3	0	0	0	Aplasia of cerebellum	Feb-09
103057	P228200	1	0	0	0	Aplasia of corpus callosum	Oct-11
59944	P344600	1	0	0	0	Aplasia of iris	Feb-09
52664	P850.00	4	0	0	0	Aplasia of lung	Feb-09
45473	PGyy400	10	0	1	0	Aplasia of muscle	Feb-09
43166	D201100	10	0	0	0	Aplastic anaemia due to drugs	Feb-09
57859	D201200	5	0	0	0	Aplastic anaemia due to infection	Feb-09
66239	D201400	2	0	0	0	Aplastic anaemia due to toxic cause	Feb-09
78	H33..00	4382665	0	46202	206	Asthma	Feb-09
23481	G581.11	436	0	56	0	Asthma - cardiac	Feb-09
10487	663j.00	12298	0	7	0	Asthma - currently active	Feb-09
232	H33z100	89016	0	4269	5	Asthma attack	Feb-09
8335	H33z111	2604	0	12	0	Asthma attack NOS	Feb-09
11370	1O2..00	12868	0	34	0	Asthma confirmed	Feb-09
4442	H33z.00	42919	0	985	12	Asthma unspecified	Feb-09
52659	F23y400	159	0	1	0	Ataxic diplegic cerebral palsy	Feb-09
38800	ZS42411	24	0	0	0	Ataxic dysarthria	Feb-09
21548	F23y000	141	0	4	0	Ataxic infantile cerebral palsy	Feb-09

For peer review only

68198	P722.00	10	0	0	0	Atresia and stenosis of aorta	Feb-09
51672	PB2..00	22	0	0	0	Atresia and stenosis of large intestine/rectum/anal canal	Feb-09
35968	PB2z.00	41	0	6	0	Atresia and stenosis of large intestine/rectum/anus NOS	Feb-09
27598	PB2..11	27	0	0	0	Atresia large intestine	Feb-09
95045	PD61000	3	0	0	0	Atresia of anterior urethra	Feb-09
31731	PB21000	24	0	0	0	Atresia of anus	Feb-09
99653	PB21300	1	0	0	0	Atresia of appendix	Jun-10
68310	P762.00	3	0	0	0	Atresia of artery NEC	Feb-09
38389	PB61400	4	0	0	0	Atresia of bile duct	Feb-09
97475	PD60000	2	0	0	0	Atresia of bladder neck	Aug-09
93089	P6yy000	4	0	0	0	Atresia of cardiac vein	Feb-09
65385	PC4yA00	7	0	0	0	Atresia of cervix	Feb-09
23593	PB21100	60	0	1	0	Atresia of colon	Feb-09
19039	PB10100	246	0	2	0	Atresia of duodenum	Feb-09
56100	PCy3000	23	0	5	0	Atresia of ejaculatory duct	Feb-09
61727	P832000	2	0	0	0	Atresia of epiglottis	Feb-09
25819	P402000	119	0	6	0	Atresia of external auditory canal	Feb-09
61770	PC1y200	4	0	1	0	Atresia of fallopian tube	Feb-09
51345	P233.00	10	0	0	0	Atresia of foramina of Magendie and Luschka	Feb-09
95015	PCy3.00	2	0	0	0	Atresia of genital organ NEC	Feb-09
62335	PCy3z00	1	0	0	0	Atresia of genital organ NEC NOS	Feb-09
92834	P832100	3	0	0	0	Atresia of glottis	Feb-09
37451	P6yy700	10	0	0	0	Atresia of heart valve NEC	Feb-09
50400	PB61600	8	0	0	0	Atresia of hepatic ducts	Feb-09
53565	PB10200	59	0	0	0	Atresia of ileum	Feb-09
52687	PB10300	86	0	1	0	Atresia of jejunum	Feb-09
31468	PB21.00	36	0	5	0	Atresia of large intestine	Feb-09
72001	P832.00	2	0	0	0	Atresia of larynx and trachea	Feb-09
52894	P8y2.00	5	0	0	0	Atresia of nasopharynx	Feb-09

For peer review only - http://bmjopen.bmj.com/site/about/guidelines.xhtml

33655	PA37.00	133	0	1	0	Atresia of oesophagus with tracheo-oesophageal fistula	Feb-09
73673	P344100	5	0	0	0	Atresia of pupil	Feb-09
46123	PB21200	21	0	0	0	Atresia of rectum	Feb-09
71153	PB21500	9	0	0	0	Atresia of rectum with fistula	Feb-09
33584	PB10.00	115	0	1	0	Atresia of small intestine	Feb-09
40252	PB10000	36	0	3	0	"Atresia of small intestine, unspecified"	Feb-09
68606	P802.00	2	0	0	0	Atresia of the posterior nares	Feb-09
63288	P832300	11	0	0	0	Atresia of trachea	Feb-09
49700	PD20.00	7	0	0	0	Atresia of ureter	Feb-09
27648	K562300	53	0	6	0	Atresia of vagina	Feb-09
36548	PC4yB00	32	0	3	0	Atresia of vagina	Feb-09
73156	K562.12	2	0	0	0	Atresia of vagina	Feb-09
58076	P722z00	36	0	0	0	Atresia or stenosis of aorta NOS	Feb-09
34305	PA22.00	42	0	5	0	"Atresia, salivary duct"	Feb-09
1664	G573000	374276	0	21137	103	Atrial fibrillation	Feb-09
2212	G573.00	146690	0	11825	6	Atrial fibrillation and flutter	Feb-09
23437	G573z00	1241	0	29	0	Atrial fibrillation and flutter NOS	Feb-09
45773	6A9..00	17151	0	49	0	Atrial fibrillation annual review	Feb-09
105554	8CMW200	518	0	1	0	Atrial fibrillation care pathway	Jan-13
104570	1I12.00	82	0	0	0	Atrial fibrillation excluded	Jul-12
57832	9Os..00	5501	0	150	0	Atrial fibrillation monitoring administration	Feb-09
28994	212R.00	22216	0	83	0	Atrial fibrillation resolved	Feb-09
9023	G576300	200	0	17	0	Atrial premature depolarization	Feb-09
3255	P550.00	11410	0	274	1	Atrial septal defect NOS	Feb-09
23708	G361.00	35	0	2	0	Atrial septal defect/curr comp folow acut myocardal infarct	Feb-09
27375	G561z00	173	0	8	0	Atrioventricular block NOS	Feb-09
58032	G561000	80	0	0	0	Atrioventricular block unspecified	Feb-09
28543	ZS91.00	878	0	28	0	Attention deficit disorder	Feb-09

For peer review only - http://bmjopen.bmj.com/site/about/guidelines.xhtml

101067	6A61.00	2631	0	816	0	Attention deficit hyperactivity disorder annual review	Dec-10
34199	E2E0000	84	0	3	0	Attention deficit without hyperactivity	Feb-09
24244	E14y000	4	0	0	0	Atypical childhood psychoses	Feb-09
1276	E140.12	18470	0	1122	1	Autism	Feb-09
7957	J614111	1100	0	35	0	Autoimmune chronic active hepatitis	Feb-09
26410	C394.00	1332	0	57	1	Autoimmune disease NOS	Feb-09
39876	D110z00	238	0	3	0	Autoimmune haemolytic anaemia NOS	Feb-09
3818	D110.00	1773	0	106	2	Autoimmune haemolytic anaemias	Feb-09
18652	J63B.00	2558	0	41	0	Autoimmune hepatitis	Feb-09
31971	C046.00	910	0	4	0	Autoimmune myxoedema	Feb-09
61859	C121000	7	0	1	0	Autoimmune parathyroiditis	Feb-09
52843	C182.00	65	0	2	0	Autoimmune polyglandular failure	Feb-09
16420	42P2.11	378	0	109	459	Auto-immune thrombocytopenia	Feb-09
3857	C052.11	2107	0	108	24	Autoimmune thyroiditis	Feb-09
106346	C353800	2	0	0	0	Autosomal dominant hypophosphataemic rickets	Jun-13
110208	PD12211	1	0	0	0	Autosomal dominant medullary cystic disease	Oct-15
105143	PD11111	56	0	0	0	Autosomal dominant polycystic kidney disease	Nov-12
105794	PD12012	3	0	0	0	Autosomal recessive medullary cystic disease	Feb-13
105919	PD11011	8	0	0	0	Autosomal recessive polycystic kidney disease	Apr-13
40288	C151200	184	0	8	0	Bartter's syndrome	Feb-09
47778	C151.12	17	0	0	0	Bartter's syndrome	Feb-09
32749	F391800	373	0	28	0	Becker muscular dystrophy	Feb-09
73283	G552.11	8	0	1	0	Becker's disease	Feb-09

For peer review only - http://bmjopen.bmj.com/site/about/guidelines.xhtml

12357	PKy9100	175	0	6	0	Beckwith's syndrome	Feb-09
16235	AD61.00	3552	0	263	1	Behcet's syndrome	Feb-09
58818	N012011	14	0	2	0	Behcet's syndrome arthropathy	Feb-09
63333	F390000	21	0	0	0	Benign congenital myopathy	Feb-09
19170	F25y400	1212	0	15	0	Benign Rolandic epilepsy	Feb-09
57144	D104600	54	0	1	0	Beta intermedia thalassaemia	Feb-09
27761	D104800	448	0	5	1	Beta minor thalassaemia	Feb-09
9864	D104500	10190	0	80	23	Beta trait thalassaemia	Feb-09
3300	P641.00	4594	0	140	0	Bicuspid aortic valve	Feb-09
57458	PKy1.11	63	0	4	0	Biedl-Bardet syndrome	Feb-09
55370	D010.12	4	0	0	0	Biermer's congenital pernicious anaemia	Feb-09
54721	P913.00	24	0	1	0	Bilateral complete cleft lip	Feb-09
48948	P903.00	20	0	0	0	Bilateral complete cleft palate	Feb-09
44977	P923.00	20	0	0	0	Bilateral complete cleft palate with cleft lip	Feb-09
71154	PD02000	5	0	0	0	Bilateral congenital absence of kidneys	Feb-09
38373	PE30100	256	0	0	0	Bilateral congenital dislocation of hip	Feb-09
107323	F591A00	36	0	3	0	Bilateral congenital sensorineural hearing loss	Dec-13
61870	PE31100	43	0	0	0	Bilateral congenital subluxation of hip	Feb-09
4035	1C13300	3079	0	380	0	Bilateral deafness	Feb-09
34285	PE34100	160	0	0	0	Bilateral dysplastic hip	Feb-09
106078	P904.00	1	0	0	0	Bilateral incomplete cleft palate	May-13
62690	P924.00	4	0	0	0	Bilateral incomplete cleft palate with cleft lip	Feb-09
9500	PD04000	49	0	0	0	Bilateral renal dysplasia	Feb-09
42925	PD03000	12	0	2	0	Bilateral renal hypoplasia	Feb-09
38774	K091.00	122	0	2	0	Bilateral small kidneys	Feb-09

For peer review only - http://bmjopen.bmj.com/site/about/guidelines.xhtml

50468	PE35100	103	0	0	0	Bilateral unstable hip	Feb-09
40425	PB6..11	93	0	7	0	Bile duct anomalies	Feb-09
53764	PB60200	16	0	1	0	"Bile duct anomaly, unspecified"	Feb-09
16005	J66z.00	676	0	40	2	Bile duct disorder NOS	Feb-09
9494	J616.00	2107	0	74	0	Biliary cirrhosis	Feb-09
58630	J616z00	47	0	0	0	Biliary cirrhosis NOS	Feb-09
91591	J616200	1	0	0	0	Biliary cirrhosis of children	Feb-09
55829	E114400	40	0	1	0	"Bipolar affect disord, currently manic,severe with psychosis"	Feb-09
72026	E115500	6	0	0	0	"Bipolar affect disord, now depressed, part/unspec remission"	Feb-09
63701	E115400	19	0	0	0	"Bipolar affect disord, now depressed, severe with psychosis"	Feb-09
35607	E115300	30	0	0	0	"Bipolar affect disord, now depressed, severe, no psychosis"	Feb-09
59011	E114500	8	0	0	0	"Bipolar affect disord,currently manic, part/unspec remission"	Feb-09
4677	E115.00	524	0	37	0	"Bipolar affective disorder, currently depressed"	Feb-09
35734	E115100	65	0	0	0	"Bipolar affective disorder, currently depressed, mild"	Feb-09
27890	E115200	63	0	1	0	"Bipolar affective disorder, currently depressed, moderate"	Feb-09
37296	E115z00	87	0	3	0	"Bipolar affective disorder, currently depressed, NOS"	Feb-09
3702	E114.00	507	0	38	0	"Bipolar affective disorder, currently manic"	Feb-09
63784	E114600	46	0	1	0	"Bipolar affective disorder, currently manic, full remission"	Feb-09
36126	E114100	55	0	1	0	"Bipolar affective disorder, currently manic, mild"	Feb-09

For peer review only - http://bmjopen.bmj.com/site/about/guidelines.xhtml

46434	E114200	40	0	1	0	"Bipolar affective disorder, currently manic, moderate"	Feb-09
57605	E114z00	64	0	1	0	"Bipolar affective disorder, currently manic, NOS"	Feb-09
35738	E114000	70	0	1	0	"Bipolar affective disorder, currently manic, unspecified"	Feb-09
57465	E115600	75	0	0	0	"Bipolar affective disorder, now depressed, in full remission"	Feb-09
8567	E11..11	6557	0	431	0	Bipolar psychoses	Feb-09
32900	D200311	173	0	5	0	Blackfan - Diamond syndrome	Feb-09
109273	D200.13	0	0	0	0	Blackfan - Diamond syndrome	Feb-15
33762	PG0y000	727	0	65	0	Brachycephaly	Feb-09
20676	G820.00	259	0	2	0	Budd - Chiari syndrome (hepatic vein thrombosis)	Feb-09
29180	G5y9.00	56	0	1	0	"Cardiac septal defect, acquired"	Feb-09
21943	P5...11	70	0	11	0	Cardiac septal defects	Feb-09
509	G5y3.00	16459	0	1620	47	Cardiomegaly	Feb-09
10890	Z7E4400	1867	0	167	0	Cerebellar ataxia	Feb-09
48731	Q200700	12	0	0	0	Cerebral haemorrhage due to birth injury	Feb-09
46545	S62z.00	50	0	0	0	Cerebral haemorrhage following injury NOS	Feb-09
23447	Q200000	97	0	0	0	"Cerebral haemorrhage unspecified, due to birth trauma"	Feb-09
104498	F2B..00	351	0	2	0	Cerebral palsy	Jul-12
104654	F2Bz.00	30	0	0	0	Cerebral palsy NOS	Aug-12
5512	F230100	1189	0	27	0	Cerebral palsy with spastic diplegia	Feb-09
16956	G669.00	511	0	20	0	"Cerebral palsy, not congenital or infantile, acute"	Feb-09
7529	F286.11	19513	0	1701	0	CFS - Chronic fatigue syndrome	Feb-09
108711	K000111	1	0	0	0	CGN - Crescentic glomerulonephritis	Oct-14
5584	F361000	2642	0	294	0	Charcot-Marie-Tooth disease	Feb-09

For peer review only - http://bmjopen.bmj.com/site/about/guidelines.xhtml

96587	4L4C.00	13	0	10	34	Charcot-Marie-Tooth disease type 1A gene detection test	Apr-09
108834	F361012	2	0	0	0	Charcot-Marie-Tooth syndrome	Nov-14
43808	P2x6.00	28	0	0	0	Chiari malformation type I	Feb-09
62968	P101000	195	0	2	0	Chiari malformation type I	Feb-09
1208	H330.12	21212	0	112	3	Childhood asthma	Feb-09
7302	E140.13	343	0	4	0	Childhood autism	Feb-09
3775	E2E..00	7103	0	1175	1	Childhood hyperkinetic syndrome	Feb-09
37395	E14z.11	44	0	2	0	Childhood schizophrenia NOS	Feb-09
31027	P80..00	621	0	27	0	Choanal atresia	Feb-09
60514	P80z.00	14	0	0	0	Choanal atresia NOS	Feb-09
66643	P800.00	10	0	0	0	"Choanal atresia, unspecified"	Feb-09
22756	PG4..00	142	0	11	1	Chondrodysplasia	Feb-09
58035	PG56000	5	0	0	0	Chondrodysplasia calcificans congenita	Feb-09
63146	PG56011	2	0	0	0	Chondrodysplasia calcificans congenita	Feb-09
14917	PG4z.00	44	0	7	0	Chondrodysplasia NOS	Feb-09
18193	PG4C.00	24	0	0	0	Chondrodysplasia punctata	Feb-09
73905	PG40.00	3	0	0	0	"Chondrodysplasia, unspecified"	Feb-09
671	PJ...00	1722	0	52	5	Chromosomal anomalies	Feb-09
20231	PJz..00	509	0	19	1	Chromosomal anomalies NOS	Feb-09
104196	PJ33800	5	0	0	0	Chromosome 4q deletion syndrome	Jun-12
54241	PJ53000	34	0	0	0	Chromosome inversion in normal individual	Feb-09
106938	PJ38.11	3	0	0	0	Chromosome replaced with dicentric	Aug-13
47247	PJ38.12	11	0	0	0	Chromosome replaced with ring	Feb-09
100562	PJ38.00	1	0	0	0	Chromosome replaced with ring or dicentric	Aug-10
9029	J614100	2400	0	106	1	Chronic active hepatitis	Feb-09

For peer review only

106805	H335.00	166	0	5	0	Chronic asthma with fixed airflow obstruction	Aug-13
5798	H312000	1813	0	47	0	Chronic asthmatic bronchitis	Feb-09
4669	K02y200	195	0	7	0	Chronic focal glomerulonephritis	Feb-09
7804	K02..00	1957	0	75	0	Chronic glomerulonephritis	Feb-09
97758	K02y000	2	0	0	0	Chronic glomerulonephritis + diseases EC	Oct-09
15097	K02z.00	184	0	7	0	Chronic glomerulonephritis NOS	Feb-09
104981	K05..13	179	0	0	0	Chronic kidney disease	Sep-12
29013	1Z10.00	22548	0	89	0	Chronic kidney disease stage 1	Feb-09
105392	K051.00	23	0	0	0	Chronic kidney disease stage 1	Dec-12
94789	1Z17.00	132	0	3	0	Chronic kidney disease stage 1 with proteinuria	Feb-09
95572	1Z18.00	228	0	1	0	Chronic kidney disease stage 1 without proteinuria	Feb-09
105383	K052.00	416	0	0	0	Chronic kidney disease stage 2	Dec-12
95146	1Z19.00	500	0	1	0	Chronic kidney disease stage 2 with proteinuria	Feb-09
95121	1Z1A.00	2368	0	4	0	Chronic kidney disease stage 2 without proteinuria	Feb-09
73026	K0A3500	3	0	0	0	Chronic neph syn difus mesangiocapillary glomerulonephritis	Feb-09
60857	K0A3700	18	0	0	0	Chronic nephritic syn diffuse crescentic glomerulonephritis	Feb-09
40413	K0A3100	10	0	0	0	Chronic nephritic syndrm focal+segmental glomerular lesions	Feb-09
21297	K0A3.00	86	0	3	0	Chronic nephritic syndrome	Feb-09
60198	K0A3600	7	0	0	0	"Chronic nephritic syndrome, dense deposit disease"	Feb-09
66505	K0A3000	5	0	0	0	"Chronic nephritic syndrome, minor glomerular abnormality"	Feb-09

For peer review only - http://bmjopen.bmj.com/site/about/guidelines.xhtml

24814	H591.00	102	0	1	0	Chronic respiratory failure	Feb-09
9312	G1...00	1190	0	43	0	Chronic rheumatic heart disease	Feb-09
6863	J61..00	7053	0	402	1	Cirrhosis and chronic liver disease	Feb-09
97980	1Z17.11	5	0	0	0	CKD stage 1 with proteinuria	Nov-09
111022	1Z18.11	1	0	0	0	CKD stage 1 without proteinuria	Jul-16
97979	1Z19.11	22	0	0	0	CKD stage 2 with proteinuria	Nov-09
97978	1Z1A.11	35	0	0	0	CKD stage 2 without proteinuria	Nov-09
110033	1Z1M.00	8	0	0	0	CKD with GFR category G1 & albuminuria category A1	Aug-15
110003	1Z1N.00	5	0	0	0	CKD with GFR category G1 & albuminuria category A2	Aug-15
110484	1Z1P.00	2	0	0	0	CKD with GFR category G1 & albuminuria category A3	Jan-16
110269	1Z1Q.00	86	0	0	0	CKD with GFR category G2 & albuminuria category A1	Nov-15
110108	1Z1R.00	40	0	0	0	CKD with GFR category G2 & albuminuria category A2	Sep-15
110251	1Z1S.00	6	0	0	0	CKD with GFR category G2 & albuminuria category A3	Nov-15
65623	P907.11	23	0	1	0	Cleft hard palate NOS	Feb-09
33902	P92A.00	12	0	0	0	"Cleft hard palate with cleft lip, bilateral"	Feb-09
62321	P92B.00	13	0	0	0	"Cleft hard palate with cleft lip, unilateral"	Feb-09
93972	P928.00	5	0	0	0	"Cleft hard palate with cleft soft palate, unilateral"	Feb-09
64549	P90B.00	2	0	0	0	"Cleft hard palate, bilateral"	Feb-09
38873	P901.11	2	0	0	0	"Cleft hard palate, unilateral"	Feb-09
101223	P90C.00	3	0	0	0	"Cleft hard palate, unilateral"	Dec-10
2027	P91..00	1719	0	65	0	Cleft lip (harelip)	Feb-09
35374	P91z.00	139	0	2	0	Cleft lip NOS	Feb-09
15364	P90z.00	237	0	39	0	Cleft palate NOS	Feb-09

For peer review only - http://bmjopen.bmj.com/site/about/guidelines.xhtml

50430	P9z..00	90	0	1	0	Cleft palate or cleft lip NOS	Feb-09
68398	P92z.00	22	0	2	0	Cleft palate with cleft lip NOS	Feb-09
51301	P920.00	19	0	0	0	"Cleft palate with cleft lip, unspecified"	Feb-09
58885	P900.00	75	0	0	0	"Cleft palate, unspecified"	Feb-09
25740	P908.11	114	0	2	0	Cleft soft palate NOS	Feb-09
42208	P90A.00	22	0	1	0	"Cleft soft palate, bilateral"	Feb-09
97477	P904.11	3	0	0	0	"Cleft soft palate, bilateral"	Aug-09
68166	P906.11	8	0	0	0	"Cleft soft palate, central"	Feb-09
57490	P902.12	2	0	1	0	"Cleft soft palate, unilateral"	Feb-09
1515	J690.00	55378	0	3179	25	Coeliac disease	Feb-09
44310	J690z00	738	0	19	0	Coeliac disease NOS	Feb-09
62236	C392.00	8	0	0	0	Combined immunity deficiency	Feb-09
62328	C392z00	3	0	0	0	Combined immunity deficiency NOS	Feb-09
43049	P56z100	21	0	1	0	Common atrioventricular canal	Feb-09
44896	P56z200	286	0	3	0	Common atrioventricular-type ventricular septal defect	Feb-09
55535	P56z000	35	0	3	0	Common atrium	Feb-09
40765	SB00100	4	0	0	0	Common carotid artery injury	Feb-09
98893	P50..12	3	0	0	0	Common truncus	Apr-10
66857	C398200	2	0	1	0	Common variable immunodef wth autoantibod to B- or T-cells	Feb-09
21975	C398.00	519	0	7	0	Common variable immunodeficiency	Feb-09
57322	C390700	20	0	0	0	Common variable immunodeficiency	Feb-09
30187	PKy5.00	52	0	6	0	Congen malformation syndromes affecting facial appearance	Feb-09
62705	PF6B300	2	0	0	0	Congen overgrowth of whole lower limb	Feb-09
109067	PD8..00	19	0	1	0	Congenital abnormality of the kidney	Jan-15

For peer review only - http://bmjopen.bmj.com/site/about/guidelines.xhtml

59430	PB20000	11	0	0	0	Congenital absence of anus	Feb-09
93826	PB20300	4	0	0	0	Congenital absence of anus with fistula	Feb-09
16417	PB61000	16	0	2	0	Congenital absence of bile duct	Feb-09
39970	PDy0.00	10	0	0	0	Congenital absence of bladder	Feb-09
48901	PF5F.00	32	0	3	0	Congenital absence of both forearm and hand	Feb-09
25316	P22A000	2	0	0	0	Congenital absence of cerebellum	Feb-09
68093	PG13000	2	0	0	0	Congenital absence of cervical vertebra	Feb-09
35942	P4y4.00	5	0	0	0	Congenital absence of chin	Feb-09
57747	PC4y200	4	0	0	0	Congenital absence of clitoris	Feb-09
93830	PG1x000	3	0	0	0	Congenital absence of coccyx	Feb-09
71956	P6y4000	1	0	0	0	Congenital absence of coronary artery	Feb-09
41775	P228000	53	0	2	0	Congenital absence of corpus callosum	Feb-09
60738	PBy0.00	2	0	0	0	Congenital absence of digestive system NOS	Feb-09
68208	P300200	6	0	0	0	Congenital absence of eye	Feb-09
67756	P303.00	3	0	0	0	Congenital absence of eyes	Feb-09
33394	PF29.00	78	0	4	0	Congenital absence of finger	Feb-09
27624	PF25.00	39	0	2	0	Congenital absence of forearm only	Feb-09
42256	PB63000	13	0	0	0	Congenital absence of gallbladder	Feb-09
46835	PF21613	68	0	1	0	Congenital absence of hand	Feb-09
48269	PB61500	2	0	0	0	Congenital absence of hepatic ducts	Feb-09
18406	PB11200	1	0	0	0	Congenital absence of ileum	Feb-09
3314	PD02.00	2413	0	57	0	Congenital absence of kidney	Feb-09
65407	PD02z00	53	0	0	0	Congenital absence of kidney NOS	Feb-09
58855	PB20.00	2	0	0	0	Congenital absence of large intestine	Feb-09
60525	PF31200	8	0	0	0	Congenital absence of leg and foot	Feb-09

For peer review only - http://bmjopen.bmj.com/site/about/guidelines.xhtml

62531	P335.11	1	0	0	0	Congenital absence of lens	Feb-09
28661	PF40.00	6	0	0	0	Congenital absence of limb NOS	Feb-09
98402	PB63100	2	0	0	0	Congenital absence of liver lobe	Jan-10
97185	P853100	3	0	0	0	Congenital absence of lobe of lung	Aug-09
72561	PF35.00	5	0	0	0	Congenital absence of lower leg only	Feb-09
54384	P853.11	5	0	0	0	Congenital absence of lung	Feb-09
68268	P853000	5	0	0	0	Congenital absence of lung fissures	Feb-09
98859	PA35.00	1	0	0	0	Congenital absence of oesophagus	Apr-10
64339	PF39400	8	0	0	0	Congenital absence of other multiple toes	Feb-09
42571	PC00.00	45	0	1	0	Congenital absence of ovary	Feb-09
61638	PF64000	14	0	1	0	Congenital absence of patella	Feb-09
49245	PCy0000	4	0	0	0	Congenital absence of penis	Feb-09
101896	P6yy411	2	0	0	0	Congenital absence of pericardium	Mar-11
111629	PK24100	1	0	0	0	Congenital absence of pituitary gland	Jan-17
103672	P731.11	2	0	0	0	Congenital absence of pulmonary artery	Feb-12
97779	P364111	2	0	0	0	Congenital absence of punctum lacrimale	Oct-09
50443	PB20200	7	0	0	0	Congenital absence of rectum	Feb-09
58095	PB20400	1	0	0	0	Congenital absence of rectum with fistula	Feb-09
42378	PG30.00	23	0	0	0	Congenital absence of rib	Feb-09
43351	PG13300	12	0	1	0	Congenital absence of sacrum	Feb-09
68081	PG1x100	1	0	0	0	Congenital absence of sacrum	Feb-09
38119	PG00.00	1	0	0	0	Congenital absence of skull bones	Feb-09
63291	PG31.00	4	0	0	0	Congenital absence of sternum	Feb-09
73987	PK28100	3	0	0	0	Congenital absence of thymus	Feb-09
3374	PK25100	233	0	26	1	Congenital absence of thyroid gland	Feb-09

For peer review only - http://bmjopen.bmj.com/site/about/guidelines.xhtml

72180	PF24.00	2	0	0	0	Congenital absence of upper arm only	Feb-09
37973	PF21.11	30	0	1	0	Congenital absence part of arm	Feb-09
37277	PF23.00	14	0	0	0	Congenital absence upper arm and forearm with hand present	Feb-09
23416	PA70.11	47	0	2	0	Congenital achalasia of cardia	Feb-09
36786	C152813	9	0	0	0	Congenital adrenal gland hypertrophy NEC	Feb-09
29640	C152000	304	0	14	0	Congenital adrenogenital syndrome	Feb-09
59314	D303000	11	0	0	0	Congenital afibrinogenaemia	Feb-09
16618	PH40.00	94	0	3	0	Congenital alopecia	Feb-09
98667	PH40z00	1	0	0	0	Congenital alopecia NOS	Mar-10
104030	PH40000	2	0	0	0	"Congenital alopecia, unspecified"	May-12
9884	F480011	3463	0	170	0	Congenital amblyopia	Feb-09
53564	PF45.00	15	0	0	0	Congenital amputation of unspecified limb	Feb-09
59307	PF21400	47	0	0	0	Congenital amputation of upper limb	Feb-09
31248	Q455.00	115	0	2	0	Congenital anaemia	Feb-09
99494	Q455000	3	0	0	0	Congenital anaemia from fetal blood loss	May-10
37080	PB59.00	71	0	10	0	Congenital anal fistula	Feb-09
35019	PB24.11	53	0	9	0	Congenital anal stricture	Feb-09
63173	P7yz000	13	0	2	0	Congenital aneurysm NEC	Feb-09
56200	P72z100	23	0	1	0	Congenital aneurysm of aorta	Feb-09
108193	P6yy600	1	0	0	0	Congenital aneurysm of heart	Jun-14
54295	PF6y000	25	0	0	0	Congenital angulation of tibia	Feb-09
44851	P344000	156	0	4	0	Congenital anisocoria	Feb-09
47533	J000200	9	0	1	0	Congenital anodontia	Feb-09
5574	PG1u.00	57	0	0	0	Congenital anomalies of cervical vertebrae NEC	Feb-09

For peer review only - http://bmjopen.bmj.com/site/about/guidelines.xhtml

24218	PF5v.00	20	0	1	0	Congenital anomalies of elbow and upper arm	Feb-09
94280	P36..00	6	0	0	0	"Congenital anomalies of eyelid, lacrimal system and orbit"	Feb-09
94285	P35z.00	3	0	0	0	Congenital anomalies of posterior chamber NOS	Feb-09
40690	PG1v.00	26	0	1	0	Congenital anomalies of thoracic vertebrae NEC	Feb-09
55893	P7y0000	9	0	0	0	"Congenital anomaly of cerebral vessel, unspecified"	Feb-09
41763	PG1uz00	12	0	2	0	Congenital anomaly of cervical vertebrae NEC NOS	Feb-09
37784	P4z1.00	22	0	2	0	Congenital anomaly of face NOS	Feb-09
66463	PG1wz00	12	0	0	0	Congenital anomaly of lumbar vertebra NEC NOS	Feb-09
68894	P6yy500	3	0	0	0	Congenital anomaly of myocardium	Feb-09
93198	P4z0.00	4	0	0	0	Congenital anomaly of neck NOS	Feb-09
108675	P76y.00	3	0	0	0	Congenital anomaly of peripheral vascular system OS	Oct-14
45918	P25y200	3	0	0	0	Congenital anomaly of spinal meninges	Feb-09
71152	PG1vz00	6	0	0	0	Congenital anomaly of thoracic vertebrae NEC NOS	Feb-09
9723	PFz..00	347	0	6	0	Congenital anomaly of unspecified limb NOS	Feb-09
8636	P64..00	65	0	4	0	Congenital aortic valve insufficiency	Feb-09
6843	P64z.00	10	0	0	0	Congenital aortic valve insufficiency NOS	Feb-09
58734	P640.00	18	0	0	0	"Congenital aortic valve insufficiency, unspecified"	Feb-09
6886	P63..00	4086	0	265	0	Congenital aortic valve stenosis	Feb-09
69823	P335.00	19	0	0	0	Congenital aphakia	Feb-09

For peer review only - http://bmjopen.bmj.com/site/about/guidelines.xhtml

94282	PCy1.00	1	0	0	0	Congenital aplasia of genital organ NEC	Feb-09
91739	PCy1000	2	0	0	0	Congenital aplasia of prostate	Feb-09
38020	PCy1300	3	0	0	0	Congenital aplasia of scrotum	Feb-09
58591	P769.00	4	0	0	0	Congenital arterial stricture	Feb-09
95800	P7y0111	3	0	0	0	Congenital arteriovenous fistula of brain	Feb-09
62587	P357100	12	0	1	0	Congenital arteriovenous malformation of retina	Feb-09
66314	F137111	10	0	0	0	Congenital athetosis	Feb-09
59553	PH40.11	4	0	0	0	Congenital atrichosis	Feb-09
22246	F38y.13	751	0	21	0	Congenital benign hypotonia	Feb-09
53454	PE07.00	23	0	5	0	Congenital bent or squashed nose	Feb-09
42721	P421.11	107	0	21	0	Congenital big ears	Feb-09
95892	P226000	8	0	0	0	Congenital bilateral perisylvian syndrome	Feb-09
57166	PDy5.00	8	0	1	0	Congenital bladder hernia	Feb-09
47854	PD60z00	14	0	0	0	Congenital bladder neck obstruction NOS	Feb-09
36666	P7y0200	92	0	2	0	Congenital brain aneurysm NEC	Feb-09
8893	P2y0.00	58	0	0	0	Congenital brain anomaly	Feb-09
56427	P861.00	20	0	1	0	Congenital bronchiectasis	Feb-09
32600	P843.12	100	0	3	0	Congenital bronchogenic cyst	Feb-09
11845	P83yB00	131	0	1	0	Congenital bronchomalacia	Feb-09
56531	PD31.00	42	0	2	0	Congenital calculus of kidney	Feb-09
34007	P6yy200	30	0	0	0	Congenital cardiomegaly	Feb-09
72271	PA70.00	3	0	0	0	Congenital cardiospasm	Feb-09
45926	P33z.00	96	0	6	0	Congenital cataract or lens anomaly NOS	Feb-09
299	P330.00	2282	0	166	0	"Congenital cataract, unspecified"	Feb-09
24424	P7y0100	216	0	17	0	Congenital cerebral arteriovenous aneurysm	Feb-09

For peer review only - http://bmjopen.bmj.com/site/about/guidelines.xhtml

44581	P7y0112	44	0	2	0	Congenital cerebral arteriovenous malformation	Feb-09
39391	P240.00	47	0	2	0	Congenital cerebral cyst	Feb-09
71733	P240z00	1	0	0	0	Congenital cerebral cyst NOS	Feb-09
53474	P20..13	6	0	0	0	Congenital cerebral hernia	Feb-09
2069	F23..00	14415	0	746	2	Congenital cerebral palsy	Feb-09
28306	F23z.00	496	0	11	0	Congenital cerebral palsy NOS	Feb-09
20979	PB6y000	216	0	12	0	Congenital choledochal cyst	Feb-09
34924	P352.00	107	0	4	0	Congenital chorioretinal degeneration	Feb-09
56810	P7yz100	69	0	0	0	Congenital chylothorax	Feb-09
30141	PF58.00	33	0	0	0	Congenital cleft hand	Feb-09
66698	P813.00	12	0	0	0	Congenital cleft nose	Feb-09
105605	P83y700	1	0	0	0	Congenital cleft of posterior cricoid cartilage	Jan-13
41019	PE8y011	38	0	0	0	Congenital club fingers	Feb-09
45651	PE8y000	80	0	5	0	Congenital club hand	Feb-09
23980	PE60.11	58	0	8	0	Congenital clubfoot - valgus	Feb-09
62397	J690000	21	0	0	0	Congenital coeliac disease	Feb-09
48508	PF5G.00	28	0	0	0	Congenital complete absence of upper limb(s)	Feb-09
52310	P6y5100	32	0	0	0	Congenital complete atrio-ventricular heart block	Feb-09
52822	PG15000	4	0	0	0	Congenital complete fusion of spine	Feb-09
31211	PKy7A00	10	0	0	0	Congenital contractural arachnodactyly	Feb-09
66259	P341z00	20	0	1	0	Congenital corneal opacities NOS	Feb-09
71732	P341000	4	0	1	0	Congenital corneal opacity with visual deficit	Feb-09
49901	P6y4500	5	0	0	0	Congenital coronary aneurysm	Feb-09
28705	P6y4411	7	0	3	0	Congenital coronary arterio-venous fistula	Feb-09

For peer review only

96586	P844.00	85	0	0	0	Congenital cystic adenomatoid malformation of the lung	Apr-09
51185	P301.00	17	0	1	0	Congenital cystic eyeball	Feb-09
15917	PD1..00	760	0	46	1	Congenital cystic kidney disease	Feb-09
50331	PD1z.00	34	0	1	0	Congenital cystic kidney disease NOS	Feb-09
27565	PB62.00	293	0	48	20	Congenital cystic liver disease	Feb-09
72000	PB62z00	5	0	0	0	Congenital cystic liver disease NOS	Feb-09
66223	P840.00	11	0	0	0	"Congenital cystic lung disease, unspecified"	Feb-09
33804	P84z.00	12	0	2	0	Congenital cystic lung NOS	Feb-09
60812	P353100	4	0	3	0	Congenital cysts of the posterior segment	Feb-09
59281	PG61.11	3	0	0	0	Congenital defect of diaphragmatic NEC	Feb-09
46289	D010.13	10	0	1	1	Congenital deficiency of intrinsic factor	Feb-09
57275	D303z00	52	0	1	0	Congenital deficiency of other clotting factor NOS	Feb-09
54725	D303y00	15	0	0	0	Congenital deficiency of other clotting factor OS	Feb-09
46026	D303.00	104	0	4	0	Congenital deficiency of other clotting factors	Feb-09
27426	PF6y100	116	0	27	0	Congenital deformity of ankle joint	Feb-09
28542	PF51.00	58	0	3	0	Congenital deformity of clavicle	Feb-09
40517	PF63X00	230	0	15	0	"Congenital deformity of hip, unspecified"	Feb-09
53416	PG1y.11	10	0	0	0	Congenital deformity of lumbosacral joint	Feb-09
51156	PG1y.12	8	0	0	0	Congenital deformity of lumbosacral region	Feb-09

For peer review only - http://bmjopen.bmj.com/site/about/guidelines.xhtml

35801	PGz..11	30	0	1	0	Congenital deformity of musculoskeletal system NEC	Feb-09
27676	PHz2.11	49	0	5	0	Congenital deformity of nail	Feb-09
64527	PF6y200	7	0	0	0	Congenital deformity of sacroiliac joint	Feb-09
49743	PF5w.11	43	0	3	0	Congenital deformity of scapula NEC	Feb-09
54223	PHz0.11	11	0	0	0	Congenital dermal defect	Feb-09
98061	P6y8.00	15	0	0	0	Congenital dextroposition of heart	Nov-09
49768	PG61.00	93	0	0	0	Congenital diaphragmatic hernia	Feb-09
11293	P72z111	55	0	1	0	Congenital dilatation of aorta	Feb-09
22560	PA40.00	281	0	17	0	Congenital dilatation of oesophagus	Feb-09
37835	P83y100	8	0	0	0	Congenital dilatation of trachea	Feb-09
26403	PD24.00	150	0	5	0	Congenital dilatation of ureter	Feb-09
64134	P248.00	50	0	1	0	Congenital dilated lateral ventricles of brain	Feb-09
22571	PD23.11	1047	0	4	0	Congenital dilated renal pelvis	Feb-09
65590	PB6y700	10	0	0	0	Congenital dilation of bile duct	Feb-09
25324	F230.00	543	0	16	0	Congenital diplegia	Feb-09
45551	F230z00	70	0	0	0	Congenital diplegia NOS	Feb-09
9239	PE3..00	5653	0	212	1	Congenital dislocation and subluxation of the hip	Feb-09
22301	PE8y200	500	0	25	0	Congenital dislocation of elbow	Feb-09
933	PE30.00	6905	0	689	1	Congenital dislocation of hip	Feb-09
27301	PE3z.00	528	0	15	0	Congenital dislocation of hip NOS	Feb-09
33576	PE30z00	158	0	1	0	Congenital dislocation of hip NOS	Feb-09
99683	P83y500	4	0	0	0	Congenital diverticulum of trachea	Jun-10
55900	PA43.00	9	0	0	0	Congenital duplication of oesophagus	Feb-09
56752	D21y012	10	0	0	0	Congenital dyserythropoiesis NEC	Feb-09
31040	D21y011	30	0	1	0	Congenital dyserythropoietic anaemia	Feb-09

For peer review only - http://bmjopen.bmj.com/site/about/guidelines.xhtml

53422	D21y000	9	0	0	0	Congenital dyshaematopoietic anaemia	Feb-09
66049	D401000	1	0	0	0	Congenital dysphagocytosis	Feb-09
29474	P337.00	20	0	0	0	Congenital ectopic lens	Feb-09
31998	P361300	14	0	2	0	Congenital ectropion	Feb-09
109860	PA2A000	1	0	0	0	Congenital ectropion of lip	Jul-15
51067	PH03.00	13	0	1	0	Congenital elephantiasis	Feb-09
72048	P20..14	2	0	0	0	Congenital endaural hernia	Feb-09
23752	P6yy900	3	0	0	0	Congenital epicardial cyst	Feb-09
65761	68A4.00	19	0	1	0	Congenital eye disorder screen	Feb-09
67169	P361z00	10	0	0	0	Congenital eyelid deformity NOS	Feb-09
70982	P4z..00	135	0	1	0	Congenital face or neck anomaly NOS	Feb-09
71051	PB5z000	4	0	0	0	Congenital faecal fistula	Feb-09
57813	Q466100	3	0	2	0	Congenital faecoliths causing obstruction	Feb-09
52576	Q402500	26	0	1	0	Congenital falciparum malaria	Feb-09
97903	PF37100	1	0	0	0	Congenital fibular deficiency type II	Oct-09
50307	PE8y600	31	0	2	0	Congenital flexion contracture of hip	Feb-09
29124	PE8y800	56	0	6	0	Congenital flexion contracture of knee	Feb-09
56631	PE8y300	15	0	1	0	Congenital flexion contractures of leg	Feb-09
24561	PG62.00	18	0	1	0	Congenital foramen Morgagni hernia	Feb-09
27309	PE80.11	100	0	8	0	Congenital funnel chest	Feb-09
45452	P602100	1	0	0	0	Congenital fusion of pulmonary valve segment	Feb-09
35806	PG33.00	57	0	1	0	Congenital fusion of ribs	Feb-09
67220	PF6y300	17	0	0	0	Congenital fusion of sacroiliac joint	Feb-09
68179	PH40200	0	0	1	0	Congenital generalised alopecia	Feb-09

For peer review only - http://bmjopen.bmj.com/site/about/guidelines.xhtml

44969	PC...00	38	0	1	0	Congenital genital organ anomalies	Feb-09
50367	PE40.00	23	0	1	0	Congenital genu recurvatum	Feb-09
23407	P320000	908	0	33	0	Congenital glaucoma	Feb-09
42855	P821.00	20	0	1	0	Congenital glottic web of larynx	Feb-09
247	P6z..00	2070	0	122	1	Congenital heart anomaly NOS	Feb-09
11982	P6zz.00	204	0	7	0	Congenital heart anomaly NOS	Feb-09
24533	P6y5.00	239	0	6	0	Congenital heart block	Feb-09
70992	P6y5z00	7	0	0	0	Congenital heart block NOS	Feb-09
68097	P6y5000	6	0	0	0	"Congenital heart block, unspecified"	Feb-09
72576	66g..00	391	0	36	0	Congenital heart condition monitoring	Feb-09
61935	L185.11	6	0	0	0	Congenital heart disease in pregnancy	Feb-09
23754	P5...12	332	0	5	0	"Congenital heart disease, septal and bulbar anomalies"	Feb-09
57298	D107000	3	0	11	11	Congenital Heinz-body anaemia	Feb-09
31853	PKyz600	471	0	26	0	Congenital hemihypertrophy	Feb-09
27966	F231.00	464	0	7	0	Congenital hemiplegia	Feb-09
34903	PB62.11	212	0	19	0	Congenital hepatic cyst	Feb-09
34642	Q48yz11	41	0	1	0	Congenital hepatic fibrosis	Feb-09
40762	Q409000	81	0	4	3	Congenital hepatitis A infection	Feb-09
50245	Q409100	121	0	4	1	Congenital hepatitis B infection	Feb-09
48738	PB6y100	10	0	0	0	Congenital hepatomegaly	Feb-09
101703	P11z.14	2	0	0	0	Congenital hernia of dura mater	Feb-11
43003	Q402000	86	0	2	0	Congenital herpes simplex	Feb-09
43215	PA6..00	234	0	1	0	Congenital hiatus hernia	Feb-09
2889	PF63100	2536	0	148	0	Congenital hip dysplasia	Feb-09
66951	P842.00	9	0	0	0	Congenital honeycomb lung	Feb-09
47776	PA71.00	11	0	1	0	Congenital hourglass stomach	Feb-09
62024	PF5y300	3	0	0	0	Congenital humeral varus	Feb-09
9611	P23..00	1376	0	78	0	Congenital hydrocephalus	Feb-09

For peer review only - http://bmjopen.bmj.com/site/about/guidelines.xhtml

107917	Q402311	2	0	0	0	Congenital hydrocephalus due to toxoplasmosis	Apr-14
28353	P23z.00	29	0	1	0	Congenital hydrocephalus NOS	Feb-09
5379	PD23.00	1375	0	24	0	Congenital hydronephrosis	Feb-09
26681	Q48y300	219	0	10	0	Congenital hypertonia	Feb-09
30154	PH42.00	55	0	11	0	Congenital hypertrichosis	Feb-09
101194	P355200	406	0	3	0	Congenital hypertrophy of retinal pigment epithelium	Dec-10
57161	C390500	27	0	1	0	Congenital hypogammaglobulinaemia	Feb-09
103611	P227100	2	0	0	0	Congenital hypoplasia of cerebrum	Jan-12
37320	D200000	69	0	9	0	Congenital hypoplastic anaemia	Feb-09
10097	C03..00	4305	0	109	28	Congenital hypothyroidism	Feb-09
51481	C03z.00	159	0	4	0	Congenital hypothyroidism NOS	Feb-09
31612	C03y000	39	0	0	0	Congenital hypothyroidism with diffuse goitre	Feb-09
93159	C03y100	2	0	0	0	Congenital hypothyroidism without goitre	Feb-09
8722	Q48y200	1837	0	35	0	Congenital hypotonia	Feb-09
57526	PH1z.11	7	0	0	0	Congenital ichthyosiform erythroderma	Feb-09
36468	PH10.00	133	0	2	0	"Congenital ichthyosis, unspecified"	Feb-09
102167	P6y5200	1	0	0	0	Congenital incomplete atrio-ventricular heart block	Apr-11
106080	Q40V.00	1	0	0	0	"Congenital infectious and parasitic disease, unspecified"	May-13
97269	PE31014	6	0	0	0	Congenital instability of hip joint	Aug-09
107150	P60z000	1	0	0	0	Congenital insufficiency of the pulmonary valve	Oct-13
67830	PB40.00	20	0	0	0	Congenital intestinal adhesions	Feb-09
69811	PB40z00	3	0	0	0	Congenital intestinal adhesions NOS	Feb-09
95632	P240.11	2	0	0	0	Congenital intracerebral cyst	Feb-09

For peer review only - http://bmjopen.bmj.com/site/about/guidelines.xhtml

96787	C0A..00	1	0	0	0	Congenital iodine deficiency syndrome	Apr-09
39166	C0A1.00	13	0	0	0	"Congenital iodine-deficiency syndrome, myxoedematous type"	Feb-09
72331	C0A0.00	1	0	0	0	"Congenital iodine-deficiency syndrome, neurological type"	Feb-09
23783	P340100	59	0	13	0	Congenital keratoconus	Feb-09
36991	PH3y300	216	0	9	0	Congenital keratoderma	Feb-09
49829	P322000	16	0	4	0	Congenital keratoglobus	Feb-09
50077	PF64z00	20	0	0	0	Congenital knee joint deformity NOS	Feb-09
32621	PH52.00	19	0	0	0	Congenital koilonychia	Feb-09
28717	PG18.11	368	0	26	0	Congenital kyphoscoliosis	Feb-09
30673	PC4yE00	526	0	35	0	Congenital labial adhesions	Feb-09
30468	P364.00	432	0	4	0	Congenital lacrimal passage anomalies	Feb-09
45351	P83y300	18	0	3	0	Congenital laryngocele	Feb-09
104555	P831700	58	0	1	0	Congenital laryngomalacia	Jul-12
49133	P6yy300	13	0	1	0	Congenital left ventricular diverticulum	Feb-09
41198	PE4..11	70	0	4	0	Congenital leg bone bowing	Feb-09
35351	P33..11	26	0	2	0	Congenital lens anomaly	Feb-09
95805	PH53.00	8	0	0	0	Congenital leukonychia	Feb-09
70280	Q402100	8	0	0	0	Congenital listeriosis	Feb-09
92345	PH40100	5	0	0	0	Congenital localised alopecia	Feb-09
29414	PG15.11	138	0	8	0	Congenital lumbosacral fusion	Feb-09
35729	PG11.00	97	0	2	0	Congenital lumbosacral spondylolysis	Feb-09
34693	B7J1.11	12	0	0	0	Congenital lymphangioma	Feb-09
27326	PH00.00	264	0	18	0	Congenital lymphoedema	Feb-09
94176	P322112	2	0	0	0	Congenital macrocornea	Feb-09
69316	Q402200	5	0	0	0	Congenital malaria	Feb-09

For peer review only - http://bmjopen.bmj.com/site/about/guidelines.xhtml

67260	PKy5z00	6	0	0	0	Congenital malform syndrome affecting facial appearance NOS	Feb-09
46825	P6W..00	33	0	3	0	Congenital malformation of aortic and mitral valves unsp	Feb-09
52658	PGX..00	33	0	3	0	"Congenital malformation of bony thorax, unspecified"	Feb-09
65092	PH6X.00	17	0	0	0	"Congenital malformation of breast, unspecified"	Feb-09
29738	P7W..00	39	0	1	0	"Congenital malformation of circulatory system, unspecif"	Feb-09
45910	P7X..00	63	0	0	0	"Congenital malformation of great arteries, unspecified"	Feb-09
47726	PB5X.00	12	0	0	0	"Congenital malformation of intestine, unspecified"	Feb-09
36474	P83yX00	22	0	2	0	"Congenital malformation of larynx, unspecified"	Feb-09
37965	PF5rD00	114	0	13	0	Congenital malformation of thumb	Feb-09
50529	P6X..00	19	0	0	0	"Congenital malformation of tricuspid valve, unspecified"	Feb-09
95151	PKy7z00	4	0	0	0	Congenital malformation syndrome involving limbs NOS	Feb-09
73575	PKy6z00	2	0	0	0	Congenital malformation syndrome with short stature NOS	Feb-09
100310	PKy8z00	2	0	0	0	Congenital malformation syndrome+other skeletal changes NOS	Jul-10
58981	PK8..00	13	0	1	0	Congenital malformation syndromes due to known exogen causes	Feb-09
45548	PKy7.00	5	0	0	0	Congenital malformation syndromes involving limbs	Feb-09

For peer review only - http://bmjopen.bmj.com/site/about/guidelines.xhtml

94910	PKy9.00	2	0	0	0	Congenital malformation syndromes with metabolic disturbance	Feb-09
97293	PKy8.00	4	0	0	0	Congenital malformation syndromes with other skeletal change	Aug-09
57953	PKy6.00	13	0	0	0	Congenital malformation syndromes with short stature	Feb-09
48205	P5X..00	33	0	0	0	Congenital malforms of cardiac chambers+connections unsp	Feb-09
39277	PBy2.00	11	0	2	1	Congenital malposition of digestive system NOS	Feb-09
38026	P322100	50	0	1	0	Congenital megalocornea	Feb-09
99424	P33y100	1	0	0	0	Congenital membranous cataract	May-10
41807	PE6z.11	37	0	2	0	Congenital metatarsus valgus	Feb-09
61651	P66..00	8	0	0	0	Congenital mitral insufficiency	Feb-09
57091	P65..00	34	0	2	0	Congenital mitral stenosis	Feb-09
100291	P65z.00	1	0	0	0	Congenital mitral stenosis NOS	Jul-10
46296	PH41.00	22	0	0	0	Congenital monilethrix	Feb-09
33925	F233.00	53	0	0	0	Congenital monoplegia	Feb-09
2709	PGz..00	778	0	46	1	Congenital musculoskeletal anomalies NOS	Feb-09
3298	PE...11	160	0	25	0	Congenital musculoskeletal deformities	Feb-09
33946	PEz..00	95	0	4	0	Congenital musculoskeletal deformity NOS	Feb-09
53113	D400500	14	0	0	0	Congenital neutropenia	Feb-09
40637	F486300	34	0	0	0	Congenital night blindness NOS	Feb-09
50096	F145.00	36	0	1	0	Congenital nonprogressive ataxia	Feb-09
49479	PB61200	13	0	2	0	Congenital obstruction of bile duct	Feb-09
65762	PB22.00	4	0	0	0	Congenital obstruction of large intestine	Feb-09

For peer review only - http://bmjopen.bmj.com/site/about/guidelines.xhtml

66309	PB12.00	20	0	0	0	Congenital obstruction of small intestine	Feb-09
34741	PD61.00	38	0	3	0	Congenital obstruction of urethra	Feb-09
100419	PD61z00	1	0	0	0	Congenital obstruction of urethra NOS	Aug-10
90483	PB23.00	6	0	0	0	Congenital occlusion of anus	Feb-09
62671	PB23z00	2	0	0	0	Congenital occlusion of anus NOS	Feb-09
101348	PB23000	1	0	1	0	Congenital occlusion of anus with fistula	Jan-11
31730	PA32.00	62	0	4	0	Congenital oesophageal fistula	Feb-09
95311	PA32z00	2	0	0	0	Congenital oesophageal fistula NOS	Feb-09
43558	PA45.00	158	0	20	0	Congenital oesophageal pouch	Feb-09
46814	PA3..11	6	0	0	0	Congenital oesophageal ring	Feb-09
49707	PA31.11	35	0	1	0	Congenital oesophageal stenosis	Feb-09
69684	PB40000	4	0	0	0	Congenital omental adhesions	Feb-09
66070	PH54.00	5	0	0	0	Congenital onychauxis	Feb-09
39392	P2xz100	139	0	4	0	Congenital optic atrophy	Feb-09
26024	P356000	95	0	2	0	Congenital optic disc coloboma	Feb-09
50282	P365.00	4	0	0	0	Congenital orbit anomalies	Feb-09
106909	PC0y.11	2	0	0	0	Congenital ovarian dysplasia	Aug-13
53673	PF6B200	1	0	0	0	Congenital overgrowth of foot	Feb-09
46652	PF6B.00	11	0	0	0	Congenital overgrowth of lower limb	Feb-09
35067	PH55.00	60	0	1	0	Congenital pachyonychia	Feb-09
37160	F230000	36	0	2	0	Congenital paraplegia	Feb-09
91259	PG15100	9	0	0	0	Congenital partial fusion of spine - balanced	Feb-09
53028	PG15200	3	0	0	0	Congenital partial fusion of spine - unbalanced	Feb-09
73595	P816.00	2	0	0	0	Congenital perforation of the nasal sinus wall	Feb-09
50362	P6yy400	14	0	0	0	Congenital pericardial defect	Feb-09

For peer review only - http://bmjopen.bmj.com/site/about/guidelines.xhtml

61062	P767.00	7	0	1	0	Congenital peripheral aneurysm	Feb-09
67420	PB40200	5	0	0	0	Congenital peritoneal adhesions	Feb-09
47616	PB40211	2	0	0	0	Congenital peritoneal bands	Feb-09
49742	PA27100	37	0	4	0	Congenital pharyngeal polyp	Feb-09
48046	PH32000	20	0	1	0	Congenital poikiloderma	Feb-09
18195	PB62000	212	0	17	0	Congenital polycystic liver disease	Feb-09
31820	P841.00	6	0	1	0	Congenital polycystic lung	Feb-09
23337	C371000	38	0	2	0	Congenital porphyria	Feb-09
11710	PD67.00	374	0	13	0	Congenital posterior urethral valves	Feb-09
69107	PD62.11	32	0	0	0	Congenital posterior urethral valves	Feb-09
38363	PE2z.11	16	0	1	0	Congenital postural curvature of spine NOS	Feb-09
100736	F591800	19	0	3	0	Congenital prelingual deafness	Sep-10
41025	PDy7.00	44	0	8	0	Congenital prolapse of bladder mucosa	Feb-09
47479	PDy8.00	29	0	2	0	Congenital prolapse of urethra	Feb-09
48936	P42z300	58	0	3	0	Congenital prominent auricle	Feb-09
48469	PF6y600	35	0	2	0	Congenital pseudarthrosis of tibia	Feb-09
44347	P8y4.00	16	0	0	0	Congenital pulmonary lymphangiectasis	Feb-09
22778	P602.00	832	0	10	0	Congenital pulmonary stenosis	Feb-09
33919	P602z00	152	0	0	0	Congenital pulmonary stenosis NOS	Feb-09
7225	D200312	18	0	0	0	Congenital pure red cell aplasia	Feb-09
38655	PB25.11	39	0	2	0	Congenital rectal stricture	Feb-09
61462	D200314	4	0	0	0	Congenital red cell hypoplasia	Feb-09
48260	PB52z12	26	0	1	0	Congenital redundant colon	Feb-09
68819	PB52z11	10	0	0	0	Congenital redundant rectal mucosa	Feb-09
27471	PD01.00	77	0	2	0	Congenital renal atrophy	Feb-09
17523	PD10.00	409	0	31	0	"Congenital renal cyst, single"	Feb-09
49791	P357000	39	0	1	0	Congenital retinal aneurysm	Feb-09
50584	P355100	14	0	0	0	Congenital retinal fold	Feb-09

For peer review only - http://bmjopen.bmj.com/site/about/guidelines.xhtml

48088	PG1x.00	44	0	1	0	Congenital sacrococcygeal anomalies NEC	Feb-09
70583	PG1xz00	12	0	0	0	Congenital sacrococcygeal anomaly NOS	Feb-09
49382	PA23.00	8	0	0	0	Congenital salivary gland fistula	Feb-09
60246	PH3y100	8	0	1	0	Congenital scar	Feb-09
10665	F591400	1042	0	25	0	Congenital sensorineural deafness	Feb-09
94734	PF34100	10	0	1	0	Congenital short femur	Feb-09
43330	D210000	5	0	0	0	Congenital sideroblastic anaemia	Feb-09
31007	PH...11	41	0	6	0	Congenital skin anomalies	Feb-09
61879	PE8y400	1	0	0	0	Congenital spade-like hand	Feb-09
15530	F23..11	644	0	34	0	Congenital spastic cerebral palsy	Feb-09
55593	F233.11	16	0	0	0	Congenital spastic foot	Feb-09
36974	P2y1.00	46	0	1	0	Congenital spinal cord anomaly	Feb-09
22012	PE2..00	660	0	11	0	Congenital spine deformity	Feb-09
29412	PE2z.00	96	0	4	0	Congenital spine deformity NOS	Feb-09
49933	PE20.00	29	0	2	0	"Congenital spine deformity, unspecified"	Feb-09
59741	PK03.00	36	0	0	0	Congenital splenomegaly	Feb-09
1871	PG12.00	1315	0	76	0	Congenital spondylolisthesis	Feb-09
100539	PB24011	2	0	0	0	Congenital stenosis of anus with fistula	Aug-10
49089	PB24111	6	0	0	0	Congenital stenosis of anus without mention of fistula	Feb-09
58189	P722411	3	0	0	0	Congenital stenosis of ascending aorta	Feb-09
71893	P833200	4	0	1	0	Congenital stenosis of bronchus	Feb-09
32484	PC4y800	82	0	3	0	Congenital stenosis of cervical canal	Feb-09
20686	PB13000	47	0	0	0	Congenital stenosis of duodenum	Feb-09
101739	P423100	1	0	0	0	Congenital stenosis of eustachian tube	Feb-11
69712	PB13200	9	0	0	0	Congenital stenosis of ileum	Feb-09

For peer review only - http://bmjopen.bmj.com/site/about/guidelines.xhtml

67021	PB13100	6	0	0	0	Congenital stenosis of jejunum	Feb-09
108445	PB22.11	1	0	0	0	Congenital stenosis of large intestine	Aug-14
61450	P833000	6	0	0	0	Congenital stenosis of larynx	Feb-09
59273	P833.00	5	0	0	0	"Congenital stenosis of larynx, trachea and bronchus"	Feb-09
101122	PB25111	1	0	0	0	Congenital stenosis of rectum without mention of fistula	Dec-10
59399	PB13.00	4	0	0	0	Congenital stenosis of small intestine	Feb-09
99366	PB13z00	1	0	0	0	Congenital stenosis of small intestine NOS	May-10
107172	P803.00	1	0	0	0	Congenital stenosis of the anterior nares	Oct-13
96389	P804.00	5	0	0	0	Congenital stenosis of the posterior nares	Apr-09
48818	P833100	11	0	0	0	Congenital stenosis of trachea	Feb-09
50820	PD22.11	34	0	0	0	Congenital stenosis of ureter	Feb-09
37200	PC4y900	28	0	7	0	Congenital stenosis of vagina	Feb-09
65337	PA73.00	9	0	0	0	Congenital stomach diverticulum	Feb-09
48020	PB24.00	15	0	0	0	Congenital stricture of anus	Feb-09
73497	PB24z00	1	0	0	0	Congenital stricture of anus NOS	Feb-09
41359	PB24000	3	0	0	0	Congenital stricture of anus with fistula	Feb-09
107117	PB24100	3	0	0	0	Congenital stricture of anus without mention of fistula	Oct-13
95810	PB61300	8	0	0	0	Congenital stricture of bile duct	Feb-09
32803	PB61311	31	0	0	0	Congenital stricture of common bile duct	Feb-09
103412	P6y4600	2	0	0	0	Congenital stricture of coronary artery	Dec-11

For peer review only - http://bmjopen.bmj.com/site/about/guidelines.xhtml

102497	P402111	1	0	0	0	Congenital stricture of external auditory canal	Jun-11
95103	P402112	1	0	0	0	Congenital stricture of osseous meatus	Feb-09
111389	P735.11	1	0	0	0	Congenital stricture of pulmonary artery	Oct-16
49501	PB25.00	29	0	1	0	Congenital stricture of rectum	Feb-09
42469	PB25z00	5	0	0	0	Congenital stricture of rectum NOS	Feb-09
69054	PB25000	1	0	0	0	Congenital stricture of rectum with fistula	Feb-09
47939	PB13z11	4	0	0	0	Congenital stricture of small intestine	Feb-09
33355	PD22.00	32	0	2	0	Congenital stricture of ureter	Feb-09
69191	PD22z00	10	0	2	0	Congenital stricture of ureter NOS	Feb-09
97464	PD22000	3	0	0	0	"Congenital stricture of ureter, unspecified"	Aug-09
27646	PD22100	17	0	0	0	Congenital stricture of ureteropelvic junction	Feb-09
96829	PD22200	2	0	0	0	Congenital stricture of ureterovesical orifice	May-09
62647	PC4y911	2	0	0	0	Congenital stricture of vagina	Feb-09
49142	P833300	20	0	0	0	Congenital subglottic stenosis	Feb-09
97956	P822.00	2	0	0	0	Congenital subglottic web of larynx	Nov-09
62586	PE31z00	76	0	0	0	Congenital subluxation of hip NOS	Feb-09
100627	F23y511	7	0	0	0	Congenital suprabulbar paresis	Sep-10
55144	P833400	12	0	1	0	Congenital supraglottic stenosis	Feb-09
99687	PF69000	2	0	0	0	Congenital synostosis of lower limb bones	Jun-10
27420	A90..00	272	0	8	0	Congenital syphilis	Feb-09
60039	A90z.00	6	0	0	0	Congenital syphilis NOS	Feb-09
50581	A900.12	4	0	0	0	Congenital syphilitic choroiditis	Feb-09
69219	A900.14	2	0	0	0	Congenital syphilitic epiphysitis	Feb-09

For peer review only - http://bmjopen.bmj.com/site/about/guidelines.xhtml

46401	A905000	10	0	0	0	Congenital syphilitic gumma	Feb-09
57279	A904200	1	0	0	0	Congenital syphilitic meningitis	Feb-09
22157	A900.16	34	0	6	1	Congenital syphilitic osteochondritis	Feb-09
7815	PE5y000	483	0	8	0	Congenital talipes calcaneovarus	Feb-09
38386	PE7y100	138	0	2	0	Congenital talipes calcaneus	Feb-09
31013	PE6y000	194	0	13	0	Congenital talipes equinovalgus	Feb-09
156	PE51.00	7134	0	439	0	Congenital talipes equinovarus	Feb-09
4334	PE7y200	411	0	25	0	Congenital talipes equinus	Feb-09
29808	PE60.00	589	0	41	0	Congenital talipes valgus	Feb-09
22846	PE50.00	1392	0	155	0	Congenital talipes varus	Feb-09
48940	P252.00	40	0	1	0	Congenital tethering of spinal cord	Feb-09
35125	PE8y111	14	0	0	0	Congenital thoracic wall deformity NEC	Feb-09
55582	D313100	7	0	0	0	Congenital thrombocytopenic purpura	Feb-09
93323	C03z.11	4	0	1	0	Congenital thyroid insufficiency	Feb-09
104835	PF36200	1	0	0	0	Congenital tibial deficiency type III	Aug-12
35042	PA32111	46	0	1	0	Congenital tracheo-oesophageal fistula	Feb-09
12752	P61..00	81	0	1	0	Congenital tricuspid atresia and stenosis	Feb-09
100065	P61z.00	2	0	0	0	Congenital tricuspid atresia or stenosis NOS	Jul-10
69169	P611.00	6	0	0	0	Congenital tricuspid stenosis	Feb-09
55957	Q402400	7	0	0	0	Congenital tuberculosis	Feb-09
110450	PG73.00	34	0	2	0	Congenital umbilical hernia	Jan-16
64939	PF6C100	2	0	0	0	Congenital undergrowth of distal part of limb	Feb-09
62450	PF6C.00	10	0	1	0	Congenital undergrowth of lower limb	Feb-09
54180	PF6C000	2	0	1	0	Congenital undergrowth of proximal part of limb	Feb-09

For peer review only - http://bmjopen.bmj.com/site/about/guidelines.xhtml

59736	PD21.11	13	0	1	0	Congenital ureteric valves	Feb-09
41342	PD62.00	43	0	0	0	Congenital urethral valvular stricture	Feb-09
29419	PDy6.00	96	0	19	0	Congenital urethrorectal fistula	Feb-09
67371	PD63.11	1	0	0	0	Congenital urinary meatus obstruction	Feb-09
31985	PD63.00	73	0	4	0	Congenital urinary meatus stricture	Feb-09
71832	PD63z00	7	0	0	0	Congenital urinary meatus stricture NOS	Feb-09
30143	PC4yC00	274	0	50	0	Congenital vaginal cyst NEC	Feb-09
36791	PF6y500	419	0	49	0	Congenital valgus ankle	Feb-09
35208	P76A.00	97	0	10	0	Congenital varix	Feb-09
36277	PF6y400	76	0	14	0	Congenital varus ankle	Feb-09
50065	PE61000	155	0	2	0	Congenital vertical talus	Feb-09
24918	PD47.00	408	0	8	0	Congenital vesico-uretero-renal reflux	Feb-09
104830	PD64.00	1	0	0	0	Congenital vesicourethral orifice stricture	Aug-12
93368	Q40X.00	4	0	0	0	"Congenital viral disease, unspecified"	Feb-09
67779	Q409.00	22	0	2	0	Congenital viral hepatitis	Feb-09
107622	Q409z00	3	0	0	0	Congenital viral hepatitis NOS	Jan-14
49448	PB5z.11	33	0	1	0	Congenital volvulus	Feb-09
31771	P82..00	87	0	1	0	Congenital web of larynx	Feb-09
70985	P82z.00	4	0	0	0	Congenital web of larynx NOS	Feb-09
111186	P820.00	1	0	0	0	"Congenital web of larynx, unspecified"	Aug-16
35616	P45..00	41	0	6	0	Congenital webbing of neck	Feb-09
99458	P45z.00	1	0	0	0	Congenital webbing of neck NOS	May-10
65125	PE1..11	5	0	1	0	Congenital wry neck	Feb-09
60880	C390512	18	0	1	0	Congenital X-linked agammaglobulinaemia	Feb-09

For peer review only - http://bmjopen.bmj.com/site/about/guidelines.xhtml

68766	G554011	10	0	0	0	Congestive obstructive cardiomyopathy	Feb-09
65402	C1z4100	8	0	1	0	Constitutional dwarfism	Feb-09
72252	D200300	4	0	1	2	Constitutional red cell aplasia and hypoplasia	Feb-09
47438	D200313	17	0	1	0	Constitutional red cell hypoplasia	Feb-09
38896	PF5E000	21	0	9	0	Constriction ring	Feb-09
93784	PF6D000	2	0	0	0	Constriction ring of lower limb	Feb-09
67293	PF6D.00	9	0	0	0	Constriction ring syndrome of lower limb	Feb-09
28775	PF6D300	1	0	0	0	Constriction ring syndrome of lower limb with amputation	Feb-09
24053	PF5E.00	32	0	3	0	Constriction ring syndrome of upper limb	Feb-09
104024	PF5E400	4	0	0	0	Constriction ring with acrosyndactyly and amputation	May-12
93038	PF5E100	2	0	2	0	Constriction ring with lymphoedema	Feb-09
65807	G532z00	15	0	0	0	Constrictive pericarditis NOS	Feb-09
25194	C294200	13	0	1	0	Copper deficiency	Feb-09
94908	4Q73.00	30	0	0	66	Copper level	Feb-09
3113	C154.00	712	0	30	1	Corticoadrenal insufficiency	Feb-09
12396	C154z00	99	0	0	0	Corticoadrenal insufficiency NOS	Feb-09
50508	PKy5B00	8	0	0	0	Costello syndrome	Feb-09
26444	J046100	273	0	7	0	Costen's syndrome	Feb-09
16897	PG03.00	1078	0	77	1	Craniosynostosis	Feb-09
27533	C03z.12	91	0	2	0	Cretinism	Feb-09
67513	C03..11	2	0	0	0	Cretinism	Feb-09
31795	PJ31.00	123	0	2	0	Cri-du-chat syndrome	Feb-09
46738	C374000	85	0	3	0	Crigler - Najjar syndrome	Feb-09
103821	13s7.00	3	0	0	0	Croatian as a second language	Feb-12
6538	J401z11	3927	0	94	0	Crohn's colitis	Feb-09

For peer review only - http://bmjopen.bmj.com/site/about/guidelines.xhtml

593	J40..11	100098	0	5433	53	Crohn's disease	Feb-09
59994	J40z.11	517	0	0	0	Crohn's disease NOS	Feb-09
39278	J400400	94	0	0	0	Crohn's disease of the ileum NOS	Feb-09
66238	J400300	48	0	0	0	Crohn's disease of the ileum unspecified	Feb-09
20688	J401z00	282	0	7	0	Crohn's disease of the large bowel NOS	Feb-09
9359	J400z00	209	0	0	0	Crohn's disease of the small bowel NOS	Feb-09
28476	J400200	582	0	3	0	Crohn's disease of the terminal ileum	Feb-09
25783	PG04.11	118	0	5	0	Crouzon's disease	Feb-09
101374	B7J1000	20	0	1	0	Cystic hygroma of neck	Jan-11
9240	PD11z11	495	0	48	0	Cystic kidney disease NEC	Feb-09
32371	C300200	9	0	0	0	Cystinaemia	Feb-09
60390	C300311	8	0	0	0	Cystine storage disease	Feb-09
27435	C300100	687	0	27	1	Cystinuria	Feb-09
3747	F593.00	1052	0	20	0	"Deaf mutism, NEC"	Feb-09
108703	Fy1..12	2	0	0	0	Deafblind	Oct-14
31374	P40z.11	259	0	5	0	Deafness due to congenital anomaly NEC	Feb-09
686	F59z.00	84378	0	26079	5	Deafness NOS	Feb-09
36588	D303100	91	0	1	0	Deficiency of factor II or prothrombin	Feb-09
15556	D303200	127	0	15	7	Deficiency of factor V or labile factor	Feb-09
45637	D303300	85	0	5	0	Deficiency of factor VII or stable factor	Feb-09
47198	D303400	32	0	1	1	Deficiency of factor X or Stuart-Prower factor	Feb-09
34673	D303500	172	0	13	5	Deficiency of factor XII or Hageman factor	Feb-09

For peer review only - http://bmjopen.bmj.com/site/about/guidelines.xhtml

20174	D303600	23	0	2	2	Deficiency of factor XIII or fibrin stabilizing factor	Feb-09
67298	PJ33000	5	0	0	0	Deletion of long arm of chromosome 13	Feb-09
95440	PJ33100	3	0	0	0	Deletion of long arm of chromosome 18	Feb-09
57806	PJ30.11	4	0	0	0	Deletion of long arm of chromosome 21	Feb-09
97927	PJ33200	4	0	0	0	Deletion of short arm of chromosome 18	Oct-09
66566	PJ32.00	7	0	0	0	Deletion of short arm of chromosome 4	Feb-09
98941	PJ31.11	6	0	0	0	Deletion of short arm of chromosome 5	Apr-10
32943	D104900	30	0	0	0	Delta-beta thalassaemia	Feb-09
106927	PKyP.00	2	0	0	0	"Diab insipidus,diab mell,optic atrophy and deafness"	Aug-13
61670	889A.00	10	0	0	0	Diab mellit insulin-glucose infus acute myocardial infarct	Feb-09
1045	C135.00	3172	0	198	2	Diabetes insipidus	Feb-09
60046	C135.12	12	0	0	0	Diabetes insipidus - pituitary	Feb-09
711	C10..00	813923	0	39051	4	Diabetes mellitus	Feb-09
43453	C10C.00	35	0	0	0	Diabetes mellitus autosomal dominant	Feb-09
36695	C10D.00	339	0	4	0	Diabetes mellitus autosomal dominant type 2	Feb-09
61122	C10H.00	38	0	0	0	Diabetes mellitus induced by non-steroid drugs	Feb-09
72345	C102z00	7	0	0	0	Diabetes mellitus NOS with hyperosmolar coma	Feb-09
42505	C101z00	376	0	8	0	Diabetes mellitus NOS with ketoacidosis	Feb-09

For peer review only

65062	C103z00	13	0	0	0	Diabetes mellitus NOS with ketoacidotic coma	Feb-09
22573	C106z00	257	0	1	0	Diabetes mellitus NOS with neurological manifestation	Feb-09
50972	C100z00	586	0	7	0	Diabetes mellitus NOS with no mention of complication	Feb-09
34283	C105z00	165	0	0	0	Diabetes mellitus NOS with ophthalmic manifestation	Feb-09
70821	C10yz00	15	0	0	0	Diabetes mellitus NOS with other specified manifestation	Feb-09
65025	C107z00	77	0	3	0	Diabetes mellitus NOS with peripheral circulatory disorder	Feb-09
64357	C10zz00	113	0	0	0	Diabetes mellitus NOS with unspecified complication	Feb-09
32403	C107.11	275	0	50	0	Diabetes mellitus with gangrene	Feb-09
21482	C102.00	220	0	34	0	Diabetes mellitus with hyperosmolar coma	Feb-09
1682	C101.00	13381	0	2152	8	Diabetes mellitus with ketoacidosis	Feb-09
15690	C103.00	353	0	58	0	Diabetes mellitus with ketoacidotic coma	Feb-09
35107	C104z00	306	0	10	0	Diabetes mellitus with nephropathy NOS	Feb-09
7795	C106.12	9335	0	219	0	Diabetes mellitus with neuropathy	Feb-09
38986	C100.00	2877	0	34	0	Diabetes mellitus with no mention of complication	Feb-09
33254	C105.00	1791	0	24	0	Diabetes mellitus with ophthalmic manifestation	Feb-09
33343	C10y.00	72	0	1	0	Diabetes mellitus with other specified manifestation	Feb-09
35399	C107.00	3910	0	28	0	Diabetes mellitus with peripheral circulatory disorder	Feb-09

For peer review only - http://bmjopen.bmj.com/site/about/guidelines.xhtml

16491	C106.13	67	0	0	0	Diabetes mellitus with polyneuropathy	Feb-09
16502	C104.00	1441	0	101	0	Diabetes mellitus with renal manifestation	Feb-09
45491	C10z.00	83	0	3	0	Diabetes mellitus with unspecified complication	Feb-09
70448	C107000	2	0	0	0	"Diabetes mellitus, juvenile +peripheral circulatory disorder"	Feb-09
69748	C105000	6	0	0	0	"Diabetes mellitus, juvenile type, + ophthalmic manifestation"	Feb-09
68792	C10z000	7	0	0	0	"Diabetes mellitus, juvenile type, + unspecified complication"	Feb-09
24490	C100000	1681	0	50	0	"Diabetes mellitus, juvenile type, no mention of complication"	Feb-09
40023	C102000	8	0	0	0	"Diabetes mellitus, juvenile type, with hyperosmolar coma"	Feb-09
53200	C101000	108	0	4	0	"Diabetes mellitus, juvenile type, with ketoacidosis"	Feb-09
42567	C103000	6	0	0	0	"Diabetes mellitus, juvenile type, with ketoacidotic coma"	Feb-09
93922	C104000	5	0	0	0	"Diabetes mellitus, juvenile type, with renal manifestation"	Feb-09
67853	C106000	76	0	0	0	"Diabetes mellitus, juvenile, + neurological manifestation"	Feb-09
110997	C10y000	1	0	0	0	"Diabetes mellitus, juvenile, + other specified manifestation"	Jul-16
13071	66AI.00	59237	0	557	73	Diabetic - good control	Feb-09
2378	66AJ.00	135053	0	8917	27	Diabetic - poor control	Feb-09
22023	66AJz00	1614	0	42	0	Diabetic - poor control NOS	Feb-09
8842	66A5.00	73497	0	162	0	Diabetic on insulin	Feb-09
111483	66o6.00	1	0	0	0	Diabetic on insulin and glucagon-like peptide 1	Nov-16

For peer review only - http://bmjopen.bmj.com/site/about/guidelines.xhtml

28769	66AV.00	15408	0	21	0	Diabetic on insulin and oral treatment	Feb-09
110344	66o2.00	41	0	0	0	Diabetic on non-insulin injectable medication	Dec-15
1684	66A4.00	230233	0	445	3	Diabetic on oral treatment	Feb-09
110379	66o5.00	47	0	0	0	Diabetic on oral treatment and glucagon-like peptide 1	Dec-15
101728	66As.00	638	0	0	0	Diabetic on subcutaneous treatment	Feb-11
4987	J34..00	25672	0	508	0	Diaphragmatic hernia	Feb-09
60697	J342.00	30	0	2	2	Diaphragmatic hernia - irreducible	Feb-09
11780	J34z.00	3413	0	10	0	Diaphragmatic hernia NOS	Feb-09
24703	J340.00	12	0	0	0	Diaphragmatic hernia with gangrene	Feb-09
44233	J341.00	16	0	0	0	Diaphragmatic hernia with obstruction	Feb-09
298	13VC.00	16755	0	383	0	Disability	Feb-09
23369	13VC100	439	0	5	0	Disability - moderate	Feb-09
3972	13VC200	248	0	8	0	Disability - severe	Feb-09
27462	13VC000	363	0	2	0	Disability - slight	Feb-09
41742	13VCZ00	531	0	7	0	Disability NOS	Feb-09
3058	13VC.11	4335	0	104	0	Disabled	Feb-09
42815	C313700	681	0	0	0	Disaccharidase deficiency	Feb-09
2667	M154100	6944	0	421	0	Discoid lupus erythematosus	Feb-09
52080	C375X00	5	0	1	0	"Disorder of glucosaminoglycan metabolism, unspecified"	Feb-09
65382	C31yX00	8	0	0	1	"Disorder of glycoprotein metabolism, unspecified"	Feb-09
66726	F391500	8	0	1	0	Distal (Gower's) muscular dystrophy	Feb-09
46117	P58..00	31	0	0	0	Double outlet left ventricle	Feb-09
1778	P511.00	37	0	0	0	Double outlet right ventricle	Feb-09
65318	P511z00	4	0	0	0	Double outlet right ventricle NOS	Feb-09

For peer review only - http://bmjopen.bmj.com/site/about/guidelines.xhtml

1543	PJ0..00	10552	0	1045	780	Down's syndrome - trisomy 21	Feb-09
10759	PJ0z.00	1447	0	13	0	Down's syndrome NOS	Feb-09
15661	G310.11	529	0	16	0	Dressler's syndrome	Feb-09
7766	F4J7000	2490	0	74	0	Duane's syndrome	Feb-09
39293	C374100	106	0	1	0	Dubin - Johnson syndrome	Feb-09
57043	PKy6600	23	0	0	0	Dubowitz syndrome	Feb-09
7470	F152111	50	0	5	0	Duchenne Aran muscular atrophy	Feb-09
108940	PD80.00	243	0	2	0	Duplex kidney	Dec-14
4431	PD34.11	6973	0	218	2	Duplex kidneys	Feb-09
5006	PG41.11	179	0	6	0	Dwarfism	Feb-09
34706	C1z4.00	30	0	0	0	Dwarfism NEC	Feb-09
66781	C1z4z00	1	0	0	0	Dwarfism NEC NOS	Feb-09
4451	PGy2.00	2654	0	259	0	Ehlers-Danlos syndrome	Feb-09
63549	PGy2000	12	0	0	0	Ehlers-Danlos syndrome type I	Feb-09
72787	PGy2100	17	0	0	0	Ehlers-Danlos syndrome type II	Feb-09
28335	PGy2200	143	0	3	0	Ehlers-Danlos syndrome type III	Feb-09
70415	PGy2300	13	0	0	0	Ehlers-Danlos syndrome type IV	Feb-09
53984	PGy2500	3	0	0	0	Ehlers-Danlos syndrome type VI	Feb-09
95643	PGy2600	1	0	0	0	Ehlers-Danlos syndrome type VII	Feb-09
98263	PGy2700	1	0	0	0	Ehlers-Danlos syndrome type VIII	Dec-09
68007	PG55.11	10	0	0	0	Ellis - Van Creveld syndrome	Feb-09
34985	F391A00	32	0	5	0	Emery-Dreifuss muscular dystrophy	Feb-09
37798	PH3y700	257	0	11	0	Epidermolysis bullosa simplex	Feb-09
573	F25..00	391517	0	20275	98	Epilepsy	Feb-09
50012	6674	226	0	6	0	Epilepsy associated problems	Feb-09
22341	1O30.00	1754	0	42	0	Epilepsy confirmed	Feb-09
9747	F25z.00	11242	0	290	1	Epilepsy NOS	Feb-09
1715	F250011	3524	0	1055	1	Epileptic absences	Feb-09
31830	F250300	199	0	17	0	Epileptic seizures - akinetic	Feb-09
24309	F250200	504	0	74	0	Epileptic seizures - atonic	Feb-09
18471	F251200	3391	0	199	0	Epileptic seizures - clonic	Feb-09
4801	F251300	1430	0	80	0	Epileptic seizures - myoclonic	Feb-09

For peer review only - http://bmjopen.bmj.com/site/about/guidelines.xhtml

5152	F251400	3665	0	259	0	Epileptic seizures - tonic	Feb-09
48036	F391100	77	0	2	0	Erb's muscular dystrophy	Feb-09
799	G20..00	2780814	0	16008	35	Essential hypertension	Feb-09
24161	C327411	219	0	15	1	Fabry's disease	Feb-09
35717	C327.12	45	0	3	1	Fabry's disease	Feb-09
24384	K032400	87	0	13	0	Familial glomerulonephritis in Alport's syndrome	Feb-09
3386	C320000	10075	0	265	0	Familial hypercholesterolaemia	Feb-09
3484	C320.11	890	0	3	0	Familial hypercholesterolaemia	Feb-09
23515	C300300	72	0	3	0	Fanconi-de-Toni syndrome	Feb-09
64539	PC72.00	6	0	0	0	Female pseudohermaphroditism	Feb-09
57081	PK84.00	69	0	2	0	Fetal valproate syndrome	Feb-09
39085	F220.00	89	0	12	0	Flaccid hemiplegia	Feb-09
104828	F23y100	1	0	0	0	Flaccid infantile cerebral palsy	Aug-12
46175	F241000	59	0	1	0	Flaccid paraplegia	Feb-09
5525	F255011	3632	0	208	1	Focal epilepsy	Feb-09
107814	K032200	1	0	0	0	Focal glomerulon + focal recurr macroscop glomerulonephritis	Mar-14
105180	F1y0.00	5	0	0	0	Fragile X associated tremor ataxia syndrome	Nov-12
67264	P205.00	8	0	0	0	Frontal encephalocele	Feb-09
65371	C311100	13	0	0	0	Galactokinase deficiency	Feb-09
5561	C311.00	317	0	10	5	Galactosaemia	Feb-09
64321	C311z00	9	0	0	0	Galactosaemia NOS	Feb-09
20730	C311000	9	0	0	0	Galactose-1-phosphate uridyl transferase deficiency	Feb-09
15593	PG71.00	1268	0	17	0	Gastroschisis	Feb-09
4659	E200200	45728	0	1707	0	Generalised anxiety disorder	Feb-09
26144	F251.00	1454	0	13	0	Generalised convulsive epilepsy	Feb-09
40806	F251z00	290	0	1	0	Generalised convulsive epilepsy NOS	Feb-09

For peer review only - http://bmjopen.bmj.com/site/about/guidelines.xhtml

44252	F250z00	82	0	1	0	Generalised nonconvulsive epilepsy NOS	Feb-09
106571	F25H.00	413	0	4	0	Generalised seizure	Aug-13
21934	C310212	179	0	4	0	Glucose-6-phosphatase deficiency	Feb-09
22531	D102000	524	0	265	245	Glucose-6-phosphate dehydrogenase deficiency anaemia	Feb-09
101224	F28y400	11	0	0	0	GLUT1 deficiency syndrome	Dec-10
22111	C309.00	9	0	0	0	Glutaryl CoA dehydrogenase deficiency	Feb-09
24186	C310.13	174	0	9	0	Glycogen storage disease	Feb-09
31383	C310.00	104	0	10	2	Glycogenosis - glycogen storage disease	Feb-09
988	F251000	18606	0	1210	2	Grand mal (major) epilepsy	Feb-09
5668	F251600	19046	0	771	2	Grand mal seizure	Feb-09
5117	F253.00	981	0	31	0	Grand mal status	Feb-09
5257	C020.12	19532	0	614	2	Graves' disease	Feb-09
2983	C350000	10217	0	1033	7	Haemochromatosis	Feb-09
31662	D107600	2	0	0	0	Haemoglobin Zurich disease	Feb-09
31800	D107300	132	0	6	5	Haemoglobin-C disease	Feb-09
7624	D107400	104	0	1	1	Haemoglobin-D disease	Feb-09
32373	D107500	155	0	2	0	Haemoglobin-E disease	Feb-09
7526	D107700	78	0	3	7	Haemoglobin-H disease	Feb-09
71808	D102.00	1	0	0	0	Haemolytic anaemia due to glutathione metabolism disorder	Feb-09
107820	D103000	2	0	0	0	Haemolytic anaemia due to hexokinase deficiency	Mar-14
55561	D103100	37	0	0	0	Haemolytic anaemia due to pyruvate kinase deficiency	Feb-09
3326	D1...00	3122	0	188	2	Haemolytic anaemias	Feb-09
18631	D1z..00	297	0	1	0	Haemolytic anaemias NOS	Feb-09
29323	D111300	1111	0	21	0	Haemolytic-uraemic syndrome	Feb-09

For peer review only - http://bmjopen.bmj.com/site/about/guidelines.xhtml

51041	C37y800	48	0	2	0	Haemophagocytic lymphohistiocytosis	Feb-09
27934	C37y900	17	0	0	0	"Haemophagocytic syndrome, infection-associated"	Feb-09
2833	D300.12	1334	0	55	0	Haemophilia A	Feb-09
31166	D301.12	285	0	4	0	Haemophilia B	Feb-09
56664	D302.11	58	0	1	0	Haemophilia C	Feb-09
5632	D300.00	1516	0	85	2	Haemophilia-A (factor VIII deficiency)	Feb-09
22706	D302.00	392	0	13	1	Haemophilia-C (factor XI deficiency)	Feb-09
69377	PG0E.11	3	0	0	0	Hallerman - Streif syndrome	Feb-09
49217	C300400	12	0	0	0	Hartnup disease	Feb-09
4549	G56..12	16631	0	1578	1	Heart block	Feb-09
95785	P6yyA00	1	0	0	0	Hemicardia	Feb-09
110165	P002.00	2	0	0	0	Hemicephaly	Oct-15
91177	7J02C00	22	0	0	0	Hemicraniotomy	Feb-09
108683	M21yD00	5	0	0	0	Hemifacial atrophy	Oct-14
9371	PE00000	160	0	4	0	Hemifacial microsomia	Feb-09
95697	P24A.00	10	0	0	0	Hemimegalencephaly	Feb-09
807	F22..11	15097	0	1791	0	Hemiparesis	Feb-09
65275	F2Az.00	136	0	1	0	Hemiparesis NOS	Feb-09
1749	F22..00	17585	0	2027	0	Hemiplegia	Feb-09
8492	F22z.00	2414	0	185	0	Hemiplegia NOS	Feb-09
3658	F26y000	6343	0	358	0	Hemiplegic migraine	Feb-09
97623	N093.11	1	0	0	0	Hench - Rosenberg syndrome	Sep-09
25383	J61y400	454	0	4	0	Hepatic fibrosis	Feb-09
71453	J615z15	24	0	0	0	Hepatic fibrosis	Feb-09
100592	J61y600	3	0	0	0	Hepatic fibrosis with hepatic sclerosis	Aug-10
2413	A70z000	7597	0	1138	458	Hepatitis C	Feb-09
10636	J624.00	407	0	14	0	Hepatorenal syndrome	Feb-09

For peer review only - http://bmjopen.bmj.com/site/about/guidelines.xhtml

34092	F1...00	129	0	7	0	Hereditary and degenerative diseases of the CNS	Feb-09
56006	F1z..00	32	0	0	0	Hereditary and degenerative diseases of the CNS NOS	Feb-09
61968	F1y..00	24	0	1	0	Hereditary and degenerative diseases of the CNS OS	Feb-09
101075	SN51100	31	0	6	0	Hereditary C1 esterase inhibitor deficiency	Dec-10
64654	F435.00	7	0	0	0	Hereditary choroid dystrophies	Feb-09
31384	C312.00	58	0	209	208	Hereditary fructose intolerance	Feb-09
14698	D10z.00	560	0	56	2	Hereditary haemolytic anaemia NOS	Feb-09
39456	D10..00	83	0	1	0	Hereditary haemolytic anaemias	Feb-09
4942	G770.00	1536	0	154	0	Hereditary haemorrhagic telangiectasia	Feb-09
46726	D311000	19	0	0	0	Hereditary haemorrhagic thrombasthenia	Feb-09
32527	F368.00	431	0	12	0	Hereditary motor and sensory neuropathy	Feb-09
56910	F368000	54	0	0	0	Hereditary motor and sensory neuropathy type I	Feb-09
35465	F368100	259	0	0	0	Hereditary motor and sensory neuropathy type II	Feb-09
106103	F368200	2	0	0	0	Hereditary motor and sensory neuropathy type III	May-13
51113	K0A5000	4	0	0	0	"Hereditary nephropathy NEC, minor glomerular abnormality"	Feb-09
41239	K0A5100	2	0	0	0	"Hereditary nephropathy NEC,focal+segmnt glomerular lesion"	Feb-09
36205	K0A5.00	44	0	2	0	Hereditary nephropathy not elsewhere classified	Feb-09

For peer review only - http://bmjopen.bmj.com/site/about/guidelines.xhtml

91738	K0A5600	1	0	0	0	"Hereditary nephropathy, NEC, dense deposit disease"	Feb-09
62980	K0A5X00	4	0	0	0	"Hereditary nephropathy, unspecif morphological changes"	Feb-09
68118	F391.00	11	0	0	0	Hereditary progressive muscular dystrophy	Feb-09
22727	F427.00	143	0	6	0	Hereditary retinal dystrophies	Feb-09
97370	F427200	2	0	0	0	Hereditary retinal dystrophies with other diseases	Aug-09
50363	F427z00	531	0	139	0	Hereditary retinal dystrophy NOS	Feb-09
3514	F141.00	748	0	62	0	Hereditary spastic paraplegia	Feb-09
7237	D100.00	3206	0	135	9	Hereditary spherocytosis	Feb-09
58906	D313111	13	0	0	0	Hereditary thrombocytopenia NEC	Feb-09
44270	K0A5200	12	0	0	0	"Hereditry nephropathy NEC,difus membran glomerulnephritis"	Feb-09
61861	C302900	20	0	1	0	Hermansky-Pudlak syndrome	Feb-09
8546	PB30.00	2034	0	82	0	Hirschsprung's disease	Feb-09
56630	PB3..00	17	0	0	0	Hirschsprung's disease and allied congenital conditions	Feb-09
59085	PB3z.00	4	0	0	0	Hirschsprung's disease and allied congenital conditions NOS	Feb-09
70582	PB30z00	65	0	1	0	Hirschsprung's disease NOS	Feb-09
43907	C305000	23	0	0	0	Histidinaemia	Feb-09
55198	C305200	5	0	0	0	Histidinuria	Feb-09
4870	B625.11	240	0	21	1	"Histiocytosis X (acute, progressive)"	Feb-09
51718	C37y500	25	0	0	0	"Histiocytosis X , chronic"	Feb-09
36736	C37y600	105	0	1	0	"Histiocytosis X , unspecified"	Feb-09
37126	C37y700	133	0	2	0	"Histiocytosis, unspecified"	Feb-09
21966	PKy7100	91	0	1	0	Holt - Oram syndrome	Feb-09
33575	C304300	204	0	4	0	Homocystinuria	Feb-09
37808	D104311	46	0	1	0	Homozygous alpha thalassaemia	Feb-09

For peer review only - http://bmjopen.bmj.com/site/about/guidelines.xhtml

44146	D303800	121	0	1	0	Homozygous factor V Leiden mutation	Feb-09
49820	42e0.00	30	0	2	66	Homozygous SS genotype	Feb-09
16167	F17z.11	841	0	97	0	Horner's syndrome	Feb-09
3584	F115.00	7715	0	298	0	Hydrocephalus	Feb-09
97663	P233.12	2	0	0	0	Hydrocephalus with atresia of foramina of Magendie+Luschka	Sep-09
40588	P230.11	6	0	0	0	Hydrocephauls with anomaly of aqueduct of Sylvius	Feb-09
21483	SM31000	16	0	1	0	Hydrochloric acid causing toxic effect	Feb-09
3277	K11..00	33858	0	2602	7	Hydronephrosis	Feb-09
27302	K11z.00	1146	0	20	0	Hydronephrosis NOS	Feb-09
8522	K113.11	2186	0	125	0	Hydronephrosis with pelviureteric junction obstruction	Feb-09
27592	K112.00	225	0	21	0	Hydronephrosis with renal and ureteral calculous obstruction	Feb-09
28159	K11X.00	108	0	3	0	Hydronephrosis with ureteral stricture NEC	Feb-09
10410	K113.00	477	0	12	0	Hydronephrosis with ureteropelvic junction obstruction	Feb-09
31841	K135.00	682	0	13	0	Hydroureter	Feb-09
46116	PD25.00	36	0	0	0	Hydroureter - congenital	Feb-09
17778	K111.00	497	0	7	0	Hydroureteronephrosis	Feb-09
32275	C151.00	437	0	13	0	Hyperaldosteronism	Feb-09
68567	C151z00	31	0	0	0	Hyperaldosteronism NOS	Feb-09
107252	C329.00	7575	0	78	2	Hypercholesterolaemia	Nov-13
34150	D41y000	283	0	27	2	Hypergammaglobulinaemia	Feb-09
10166	C307000	2032	0	66	8	Hyperglycinaemia	Feb-09
31274	C163200	53	0	3	0	Hypergonadotrophic ovarian failure	Feb-09
104314	C304500	15	0	0	0	Hyperhomocysteinaemia	Jun-12
54203	C397.00	17	0	0	0	Hyperimmunoglobulin E syndrome	Feb-09

For peer review only - http://bmjopen.bmj.com/site/about/guidelines.xhtml

111300	K08yB00	2	0	0	0	Hyperkalaemic renal tubular acidosis	Sep-16
58069	E2E1.00	17	0	1	0	Hyperkinesis with developmental delay	Feb-09
45263	E2E2.00	175	0	6	0	Hyperkinetic conduct disorder	Feb-09
41769	E2Ez.00	41	0	4	0	Hyperkinetic syndrome NOS	Feb-09
637	C324.00	338588	0	7473	464	Hyperlipidaemia NOS	Feb-09
48144	C307400	22	0	0	0	Hyperlysinaemia	Feb-09
26371	C352000	27	0	0	0	Hypermagnesaemia	Feb-09
73934	C120100	8	0	0	0	Hyperparathyroid bone disease	Feb-09
3559	C120.00	16259	0	1792	3	Hyperparathyroidism	Feb-09
9423	C353400	706	0	12	0	Hyperphosphataemia	Feb-09
6732	C131000	10204	0	1238	0	Hyperprolactinaemia	Feb-09
30533	C30y400	82	0	12	0	Hyperprolinaemia	Feb-09
10702	D414.00	449	0	25	0	Hypersplenism	Feb-09
57987	G234.00	8	0	1	0	Hyperten heart&renal dis+both(congestv)heart and renal fail	Feb-09
103690	38G3.00	6798	0	0	0	"Hyperten, abnorm renal/liver funct, stroke, BLED score"	Feb-12
7057	G2z..00	41526	0	209	0	Hypertensive disease NOS	Feb-09
63466	G23..00	39	0	0	0	Hypertensive heart and renal disease	Feb-09
68659	G23z.00	6	0	0	0	Hypertensive heart and renal disease NOS	Feb-09
28684	G233.00	46	0	2	0	Hypertensive heart and renal disease with renal failure	Feb-09
31464	G21z.00	119	0	2	0	Hypertensive heart disease NOS	Feb-09
62718	G21z100	70	0	0	0	Hypertensive heart disease NOS with CCF	Feb-09
61166	G21z000	67	0	1	0	Hypertensive heart disease NOS without CCF	Feb-09

For peer review only - http://bmjopen.bmj.com/site/about/guidelines.xhtml

21837	G232.00	29	0	4	0	Hypertensive heart&renal dis wth (congestive) heart failure	Feb-09
4668	G22..00	1876	0	61	0	Hypertensive renal disease	Feb-09
32423	G222.00	82	0	0	0	Hypertensive renal disease with renal failure	Feb-09
1472	C02..11	66918	0	9227	174	Hyperthyroidism	Feb-09
26362	212P.00	331	0	4	0	Hyperthyroidism resolved	Feb-09
53977	C302600	11	0	0	0	Hypertyrosinaemia	Feb-09
91091	7Q0B100	42	0	0	0	Hyperuricaemia drugs Band 1	Feb-09
60584	C303400	6	0	0	0	Hypervalinaemia	Feb-09
64905	C382.00	5	0	0	0	Hypervitaminosis A	Feb-09
32143	C384.00	36	0	2	0	Hypervitaminosis D	Feb-09
21594	C154300	113	0	1	0	Hypoaldosteronism	Feb-09
95041	PG4B100	2	0	0	0	Hypochondrogenesis	Feb-09
29840	PG42.15	137	0	4	0	Hypochondroplasia	Feb-09
94173	PG41000	13	0	0	0	Hypochondroplasia	Feb-09
50305	K032y11	11	0	0	0	Hypocomplementaemic persistent glomerulonephritis NEC	Feb-09
15137	C390000	1831	0	64	0	Hypogammaglobulinaemia NOS	Feb-09
98210	C139.00	1581	0	70	0	Hypogonadotropic hypogonadism	Dec-09
36298	M250.11	37	0	3	0	Hypohidrosis	Feb-09
15096	C121.00	2168	0	136	2	Hypoparathyroidism	Feb-09
63512	C121z00	49	0	0	0	Hypoparathyroidism NOS	Feb-09
7902	C353300	613	0	21	0	Hypophosphataemia	Feb-09
21870	C353211	217	0	5	0	Hypophosphataemic rickets	Feb-09
33347	C353000	366	0	19	1	Hypophosphatasia	Feb-09
22028	C353100	88	0	6	0	Hypophosphatasia rickets	Feb-09
105861	C133.12	1	0	0	0	Hypophyseal dwarfism	Mar-13
8552	C132.11	3039	0	145	4	Hypopituitarism NOS	Feb-09
29818	PK13.00	92	0	6	0	Hypoplasia of adrenal gland	Feb-09
37515	P722200	42	0	1	0	Hypoplasia of aorta	Feb-09

For peer review only - http://bmjopen.bmj.com/site/about/guidelines.xhtml

57932	P710.00	29	0	1	0	"Hypoplasia of aortic arch, unspecified"	Feb-09
63668	P222.00	12	0	0	0	"Hypoplasia of brain, part unspecified"	Feb-09
68063	P6yy100	3	0	0	0	Hypoplasia of cardiac vein	Feb-09
44717	P22A100	29	0	0	0	Hypoplasia of cerebellum	Feb-09
37600	P228100	13	0	0	0	Hypoplasia of corpus callosum	Feb-09
51993	PF4z.13	42	0	2	0	Hypoplasia of limb NOS	Feb-09
24646	PF3z.11	107	0	1	0	Hypoplasia of lower limb	Feb-09
23874	P851.00	156	0	1	0	Hypoplasia of lung	Feb-09
61593	PB72.00	3	0	0	0	Hypoplasia of pancreas	Feb-09
60216	P22z.13	11	0	0	0	Hypoplasia of part of brain NEC	Feb-09
23948	PCy2000	58	0	6	0	Hypoplasia of penis	Feb-09
54488	P601000	17	0	0	0	Hypoplasia of pulmonary valve	Feb-09
68493	PG1y400	10	0	0	0	Hypoplasia of spine	Feb-09
71462	PK06.00	5	0	0	0	Hypoplasia of spleen	Feb-09
54487	P734.00	34	0	0	0	Hypoplasia of the pulmonary artery	Feb-09
22457	PF2z.11	62	0	3	0	Hypoplasia of upper limb	Feb-09
44913	D200.15	46	0	3	0	Hypoplastic anaemia - familial	Feb-09
32715	D201111	11	0	0	0	Hypoplastic anaemia due to drug or chemical substance	Feb-09
65351	D201211	11	0	0	0	Hypoplastic anaemia due to infection	Feb-09
57114	D201412	2	0	1	0	Hypoplastic anaemia due to toxic cause	Feb-09
44767	P6yy.11	27	0	0	0	Hypoplastic aortic orifice or valve	Feb-09
92968	7Q09100	3	0	0	0	Hypoplastic haemolytic and renal anaemia drugs Band 2	Feb-09
16004	C134011	112	0	13	0	Hypoprolactinaemia	Feb-09
5073	C33y000	1163	0	98	4	Hypoproteinaemia	Feb-09
51291	D303111	10	0	2	0	Hypoprothrombinaemia	Feb-09
28386	F4A0400	43	0	2	0	Hypopyon ulcer	Feb-09

For peer review only - http://bmjopen.bmj.com/site/about/guidelines.xhtml

For peer review only

108019	C154700	9	0	0	0	Hyporeninaemic hypoaldosteronism	May-14
36881	C13X.00	53	0	1	0	"Hypothalamic dysfunction, not elsewhere classified"	Feb-09
22732	C13..11	144	0	7	0	Hypothalamus disorders	Feb-09
64145	5761.11	2	0	1	3	Hypothalamus hormone radioass.	Feb-09
18282	C04z.13	447	0	54	0	"Hypothyroid goitre, acquired"	Feb-09
273	C04..13	428907	0	8672	1713	Hypothyroidism	Feb-09
3941	C04z.00	39418	0	249	0	Hypothyroidism NOS	Feb-09
68482	C372000	1	0	2	0	Hypoxanthine-guanine-phosphoribosyltransferase deficiency	Feb-09
20275	C150100	72	0	5	0	Iatrogenic Cushing's syndrome	Feb-09
61520	C110000	17	0	0	0	Iatrogenic hyperinsulinism	Feb-09
50958	C137.11	51	0	3	0	Iatrogenic hypopituitarism	Feb-09
38976	C043z00	240	0	2	0	Iatrogenic hypothyroidism NOS	Feb-09
34459	C137z00	8	0	1	1	Iatrogenic pituitary disorder NOS	Feb-09
56983	C137.00	22	0	2	0	Iatrogenic pituitary disorders	Feb-09
61026	C054.00	13	0	0	0	Iatrogenic thyroiditis	Feb-09
370	PH1..00	1995	0	86	0	Ichthyosis congenita	Feb-09
64322	PH1z.00	72	0	0	0	Ichthyosis congenita NOS	Feb-09
5555	PH14.00	780	0	18	0	Ichthyosis vulgaris	Feb-09
69124	C107300	15	0	0	0	IDDM with peripheral circulatory disorder	Feb-09
18505	C108.11	8393	0	317	0	IDDM-Insulin dependent diabetes mellitus	Feb-09
37401	PB31.00	92	0	2	0	Idiopathic congenital megacolon	Feb-09
94708	F4B2100	5	0	0	0	Idiopathic corneal oedema	Feb-09
70967	C150000	6	0	1	0	Idiopathic Cushing's syndrome	Feb-09
62243	F136000	14	0	1	0	Idiopathic familial dystonia	Feb-09
28229	H563z00	214	0	1	0	Idiopathic fibrosing alveolitis NOS	Feb-09
40750	D00z200	193	0	2	0	Idiopathic hypochromic anaemia	Feb-09
25379	C121200	46	0	0	0	Idiopathic hypoparathyroidism	Feb-09

For peer review only - http://bmjopen.bmj.com/site/about/guidelines.xhtml

40428	N330300	133	0	3	0	Idiopathic osteoporosis	Feb-09
27597	N331600	116	0	6	0	Idiopathic osteoporosis with pathological fracture	Feb-09
48590	C132000	30	0	0	0	Idiopathic panhypopituitarism	Feb-09
103753	H563.13	344	0	8	0	Idiopathic pulmonary fibrosis	Feb-12
27348	H561.00	45	0	6	0	Idiopathic pulmonary haemosiderosis	Feb-09
29966	C350300	47	0	8	0	Idiopathic pulmonary haemosiderosis	Feb-09
31560	B937.12	82	0	2	0	Idiopathic thrombocythaemia	Feb-09
5144	D313.12	2000	0	197	3	Idiopathic thrombocytopenic purpura	Feb-09
12234	D313000	2308	0	47	0	Idiopathic thrombocytopenic purpura	Feb-09
39800	C39X.00	67	0	5	0	"Immunodeficiency associated+major defect, unspecified"	Feb-09
69373	C390600	6	0	0	0	Immunodeficiency with IgM hypergammaglobulinaemia	Feb-09
65617	C395.00	4	0	0	0	Immunodeficiency with short-limbed stature	Feb-09
4855	PB26.00	1267	0	29	0	Imperforate anus	Feb-09
67319	PB26z00	80	0	0	0	Imperforate anus NOS	Feb-09
61401	PB26000	56	0	0	0	Imperforate anus with fistula	Feb-09
68484	PB14.00	3	0	0	0	Imperforate jejunum	Feb-09
98741	PA33.00	0	0	0	0	Imperforate oesophagus	Mar-10
56901	PA27000	2	0	0	0	Imperforate pharynx	Feb-09
49242	PB27.00	22	0	0	0	Imperforate rectum	Feb-09
44953	PB27000	3	0	0	0	Imperforate rectum with fistula	Feb-09
22098	E140.00	1419	0	53	0	Infantile autism	Feb-09
36662	E140z00	78	0	1	0	Infantile autism NOS	Feb-09
5560	F23..12	527	0	8	0	Infantile cerebral palsy	Feb-09

For peer review only - http://bmjopen.bmj.com/site/about/guidelines.xhtml

2019	F234.00	250	0	5	0	Infantile hemiplegia NOS	Feb-09
54014	F392300	6	0	0	0	Infantile myotonia	Feb-09
41668	F460.00	49	0	3	0	"Infantile, juvenile and presenile cataracts"	Feb-09
569	G64..12	3985	0	179	0	Infarction - cerebral	Feb-09
57495	G63..11	37	0	0	0	Infarction - precerebral	Feb-09
24444	G401.11	124	0	1	0	Infarction - pulmonary	Feb-09
26424	G64z400	1122	0	5	0	Infarction of basal ganglia	Feb-09
1796	J4...12	18902	0	1309	14	Inflammatory bowel disease	Feb-09
39809	C108J00	27	0	1	0	Insulin dependent diab mell with neuropathic arthropathy	Feb-09
64446	C108G00	3	0	1	0	Insulin dependent diab mell with peripheral angiopathy	Feb-09
31310	C108900	565	0	20	0	Insulin dependent diabetes maturity onset	Feb-09
97849	C10E912	14	0	0	0	Insulin dependent diabetes maturity onset	Oct-09
1038	C100011	34834	0	2167	5	Insulin dependent diabetes mellitus	Feb-09
1647	C108.00	23978	0	838	7	Insulin dependent diabetes mellitus	Feb-09
51261	C10E.12	1339	0	1	0	Insulin dependent diabetes mellitus	Feb-09
6791	C108800	434	0	18	0	Insulin dependent diabetes mellitus - poor control	Feb-09
72702	C10E812	14	0	0	0	Insulin dependent diabetes mellitus - poor control	Feb-09
65616	C108H00	4	0	0	0	Insulin dependent diabetes mellitus with arthropathy	Feb-09
44260	C108F00	7	0	2	0	Insulin dependent diabetes mellitus with diabetic cataract	Feb-09
100770	C10EF12	1	0	0	0	Insulin dependent diabetes mellitus with diabetic cataract	Nov-10
60499	C108600	10	0	2	0	Insulin dependent diabetes mellitus with gangrene	Feb-09

For peer review only - http://bmjopen.bmj.com/site/about/guidelines.xhtml

109051	C10E612	1	0	0	0	Insulin dependent diabetes mellitus with gangrene	Jan-15
44440	C108E00	51	0	0	0	Insulin dependent diabetes mellitus with hypoglycaemic coma	Feb-09
99716	C10EE12	2	0	0	0	Insulin dependent diabetes mellitus with hypoglycaemic coma	Jun-10
24694	C108B00	1	0	0	0	Insulin dependent diabetes mellitus with mononeuropathy	Feb-09
45276	C10E312	16	0	0	0	Insulin dependent diabetes mellitus with multiple complicat	Feb-09
52104	C108300	11	0	1	0	Insulin dependent diabetes mellitus with multiple complicatn	Feb-09
57621	C108D00	18	0	0	0	Insulin dependent diabetes mellitus with nephropathy	Feb-09
102163	C10ED12	1	0	0	0	Insulin dependent diabetes mellitus with nephropathy	Apr-11
41716	C108C00	11	0	0	0	Insulin dependent diabetes mellitus with polyneuropathy	Feb-09
101311	C10EC12	2	0	0	0	Insulin dependent diabetes mellitus with polyneuropathy	Jan-11
6509	C108700	249	0	41	0	Insulin dependent diabetes mellitus with retinopathy	Feb-09
93875	C10E712	8	0	0	0	Insulin dependent diabetes mellitus with retinopathy	Feb-09
44443	C108500	215	0	3	0	Insulin dependent diabetes mellitus with ulcer	Feb-09
98704	C10E512	3	0	0	0	Insulin dependent diabetes mellitus with ulcer	Mar-10
37648	C109J11	24	0	3	0	Insulin treated non-insulin dependent diabetes mellitus	Feb-09
1407	C10FJ00	13414	0	84	0	Insulin treated Type 2 diabetes mellitus	Feb-09

For peer review only - http://bmjopen.bmj.com/site/about/guidelines.xhtml

18278	C109J00	4211	0	28	0	Insulin treated Type 2 diabetes mellitus	Feb-09
64668	C10FJ11	107	0	0	0	Insulin treated Type II diabetes mellitus	Feb-09
96010	66Ap.00	2595	0	3	0	Insulin treatment initiated	Feb-09
100791	66Ar.00	91	0	0	0	Insulin treatment stopped	Nov-10
52283	C108200	8	0	0	0	Insulin-dependent diabetes mellitus with neurological comps	Feb-09
101735	C10E212	1	0	0	0	Insulin-dependent diabetes mellitus with neurological comps	Feb-11
49276	C108100	18	0	0	0	Insulin-dependent diabetes mellitus with ophthalmic comps	Feb-09
98071	C10E112	2	0	0	0	Insulin-dependent diabetes mellitus with ophthalmic comps	Nov-09
46963	C108000	30	0	1	0	Insulin-dependent diabetes mellitus with renal complications	Feb-09
102946	C10E012	4	0	0	0	Insulin-dependent diabetes mellitus with renal complications	Sep-11
56448	C108A00	21	0	0	0	Insulin-dependent diabetes without complication	Feb-09
99719	C10EA12	2	0	0	0	Insulin-dependent diabetes without complication	Jun-10
109815	H58y600	1	0	0	0	Interstitial lung disease due to collagen vascular disease	Jul-15
104915	H58y700	35	0	1	0	Interstitial lung disease due to connective tissue disease	Sep-12
9355	J69..00	2226	0	216	0	Intestinal malabsorption	Feb-09
42715	J69z.00	212	0	2	0	Intestinal malabsorption NOS	Feb-09
49739	J69y300	10	0	2	0	Intestinal malabsorption of carbohydrate	Feb-09
31392	J69y600	85	0	4	1	Intestinal malabsorption of fat	Feb-09
49191	J69y200	37	0	29	26	Intestinal malabsorption of protein	Feb-09

For peer review only - http://bmjopen.bmj.com/site/about/guidelines.xhtml

37877	F280z11	26	0	0	0	Intracerebral cyst NOS	Feb-09
44479	Q200511	4	0	0	0	Intracerebral haematoma in fetus or newborn	Feb-09
5051	G61..00	14470	0	240	0	Intracerebral haemorrhage	Feb-09
36559	Q200011	26	0	0	0	Intracerebral haemorrhage in fetus or newborn	Feb-09
31060	G61X.00	104	0	0	0	"Intracerebral haemorrhage in hemisphere, unspecified"	Feb-09
3535	G61z.00	1877	0	81	0	Intracerebral haemorrhage NOS	Feb-09
30202	G617.00	676	0	2	0	"Intracerebral haemorrhage, intraventricular"	Feb-09
57315	G618.00	33	0	2	0	"Intracerebral haemorrhage, multiple localized"	Feb-09
66218	C3A..00	5	0	0	0	Iodine-deficiency syndromes	Feb-09
54511	C0AX.00	16	0	0	0	"Iodine-deficiency-related (endemic) goitre, unspecified"	Feb-09
37518	C0A3.00	22	0	1	0	Iodine-deficiency-related diffuse (endemic) goitre	Feb-09
44459	C0A4.00	84	0	8	0	Iodine-deficiency-related multinodular (endemic) goitre	Feb-09
48338	D000.12	118	0	1	0	Iron deficiency anaemia due to blood loss	Feb-09
27726	D000.00	2676	0	101	0	Iron deficiency anaemia due to chronic blood loss	Feb-09
15439	D00zz00	26280	0	657	17	Iron deficiency anaemia NOS	Feb-09
795	D00..00	458744	0	29240	279	Iron deficiency anaemias	Feb-09
100507	PJ33400	10	0	0	0	Jacobsen syndrome	Aug-10
50228	PG43.11	27	0	1	0	Jeune's syndrome	Feb-09
17399	F250400	897	0	32	0	Juvenile absence epilepsy	Feb-09
42405	N045000	118	0	8	0	Juvenile ankylosing spondylitis	Feb-09
12575	N045300	50	0	5	0	Juvenile arthritis in Crohn's disease	Feb-09
28456	N045200	163	0	5	0	Juvenile arthritis in psoriasis	Feb-09

For peer review only - http://bmjopen.bmj.com/site/about/guidelines.xhtml

71083	N045400	4	0	0	0	Juvenile arthritis in ulcerative colitis	Feb-09
50627	M142.00	36	0	0	0	Juvenile dermatitis herpetiformis	Feb-09
32649	N003000	91	0	1	0	Juvenile dermatomyositis	Feb-09
64693	F4B5100	9	0	0	0	Juvenile epithelial corneal dystrophy	Feb-09
105069	B693.00	3	0	0	0	Juvenile myelomonocytic leukaemia	Oct-12
19363	F25A.00	1338	0	12	0	Juvenile myoclonic epilepsy	Feb-09
45320	A904.00	3	0	0	0	Juvenile neurosyphilis	Feb-09
66740	F380100	8	0	0	0	Juvenile or adult myasthenia gravis	Feb-09
6975	N326200	899	0	17	0	Juvenile osteochondritis NOS	Feb-09
63370	N323.11	2	0	0	0	Juvenile osteochondritis of the arm	Feb-09
62237	N323.00	21	0	1	0	Juvenile osteochondritis of the arm and hand	Feb-09
73645	N323z00	1	0	0	0	Juvenile osteochondritis of the arm and hand NOS	Feb-09
101671	N323000	1	0	0	0	"Juvenile osteochondritis of the arm, unspecified"	Feb-11
39819	N323100	3	0	0	0	"Juvenile osteochondritis of the hand, unspecified"	Feb-09
34369	N321.00	370	0	1	1	Juvenile osteochondritis of the hip and pelvis	Feb-09
68673	N321z00	11	0	0	0	Juvenile osteochondritis of the hip and pelvis NOS	Feb-09
66495	N321000	7	0	0	0	"Juvenile osteochondritis of the hip and pelvis, unspecified"	Feb-09
21502	N321400	23	0	2	0	Juvenile osteochondritis of the iliac crest	Feb-09
36725	N320.00	422	0	4	0	Juvenile osteochondritis of the spine	Feb-09
69739	N320z00	16	0	0	0	Juvenile osteochondritis of the spine NOS	Feb-09

For peer review only - http://bmjopen.bmj.com/site/about/guidelines.xhtml

50510	N320000	13	0	0	0	"Juvenile osteochondritis of the spine, unspecified"	Feb-09
94462	N321500	4	0	0	0	Juvenile osteochondritis of the symphysis pubis	Feb-09
94547	N326z00	3	0	0	0	Juvenile osteochondroses NOS	Feb-09
9691	N326300	222	0	4	0	Juvenile osteochondrosis NOS	Feb-09
52681	N328.00	32	0	0	0	Juvenile osteochondrosis of spine	Feb-09
48042	N325.00	61	0	1	0	Juvenile osteochondrosis of the foot	Feb-09
60589	N325z00	14	0	0	0	Juvenile osteochondrosis of the foot NOS	Feb-09
63567	N325000	10	0	0	0	"Juvenile osteochondrosis of the foot, unspecified"	Feb-09
33667	N324.00	2827	0	3	0	Juvenile osteochondrosis of the leg	Feb-09
38898	N324z00	54	0	2	0	"Juvenile osteochondrosis of the leg, NOS"	Feb-09
24028	N324000	24	0	0	0	"Juvenile osteochondrosis of the leg, unspecified"	Feb-09
35026	N324111	15	0	0	0	Juvenile osteochondrosis of the primary patellar centre	Feb-09
48242	N324300	58	0	0	0	Juvenile osteochondrosis of the secondary patellar centre	Feb-09
17810	M142.11	20	0	1	0	Juvenile pemphigoid	Feb-09
7859	M2y4800	8465	0	78	0	Juvenile plantar dermatosis	Feb-09
18380	G759.00	115	0	6	0	Juvenile polyarteritis	Feb-09
96664	J615800	3	0	0	0	Juvenile portal cirrhosis	Apr-09
28450	F427300	109	0	4	0	Juvenile retinoschisis	Feb-09
31360	N045500	352	0	2	0	Juvenile rheumatoid arthritis	Feb-09
4186	N043.00	4230	0	258	0	Juvenile rheumatoid arthritis - Still's disease	Feb-09
27557	N043z00	225	0	2	0	Juvenile rheumatoid arthritis NOS	Feb-09
50644	N043000	37	0	2	0	Juvenile rheumatoid arthropathy unspecified	Feb-09

For peer review only - http://bmjopen.bmj.com/site/about/guidelines.xhtml

31181	N045100	188	0	7	0	Juvenile seronegative polyarthritis	Feb-09
107689	M12A400	80	0	0	0	Juvenile spring eruption	Feb-14
21876	C172111	265	0	16	0	Kallman's syndrome	Feb-09
43444	E140.11	12	0	0	0	Kanner's syndrome	Feb-09
99213	PJy2.12	2	0	0	0	"Karyotype 47, XXX"	Apr-10
9768	PJyy300	119	0	4	2	"Karyotype 47,XYY"	Feb-09
6157	G751000	2136	0	99	0	Kawasaki disease	Feb-09
31452	Q424.00	55	0	4	0	Kernicterus due to isoimmunisation	Feb-09
48012	Q437.00	8	0	0	0	Kernicterus not due to isoimmunisation	Feb-09
61800	Q437z00	7	0	0	0	Kernicterus of newborn NOS	Feb-09
69127	PKy9211	2	0	0	0	Kinky hair syndrome	Feb-09
54490	PJ70.00	29	0	0	0	"Klinefelter's phenotype, karyotype 47XXY"	Feb-09
59439	PJ7z.00	63	0	2	0	Klinefelter's syndrome NOS	Feb-09
96257	PJ72.00	1	0	0	0	"Klinefelter's syndrome, male with 46XX karyotype"	Apr-09
62414	PJ71.12	11	0	0	0	"Klinefelter's syndrome, XXXXY"	Feb-09
56545	PJ71.11	4	0	0	0	"Klinefelter's syndrome, XXXY"	Feb-09
91262	PJ73.00	10	0	0	0	"Klinefelter's syndrome, XXYY"	Feb-09
68109	PJ74.00	26	0	1	0	"Klinefelter's syndrome, XY/XXY mosaic"	Feb-09
67854	PJ71.00	3	0	1	0	"Klinefelter's syndrome,male with more than two X chromosomes"	Feb-09
73985	PG16z00	16	0	0	0	Klippel - Feil syndrome NOS	Feb-09
10491	PKy7200	411	0	18	0	Klippel - Trenaunay - Weber syndrome	Feb-09
4674	PG16.00	519	0	15	0	Klippel-Feil syndrome	Feb-09
59014	N13y100	31	0	0	0	Klippel's disease	Feb-09
71719	F257.00	3	0	0	0	Kojevnikov's epilepsy	Feb-09
35737	C20..00	32	0	2	1	Kwashiorkor	Feb-09
60113	C362300	20	0	0	0	Lactic acidaemia	Feb-09

For peer review only - http://bmjopen.bmj.com/site/about/guidelines.xhtml

37854	C313111	64	0	3	0	Lactose malabsorption	Feb-09
105082	F381011	21	0	0	0	Lambert-Eaton syndrome	Oct-12
59806	ZS82.11	29	0	1	0	Landau-Kleffner syndrome	Feb-09
40000	C37yB00	304	0	1	0	Langerhans' cell histiocytosis	Feb-09
6340	E2F3.11	2040	0	48	0	Language development disorder	Feb-09
99857	ZS7C400	2	0	0	0	Language disorder associated with right hemisphere damage	Jun-10
32831	ZS7C600	42	0	9	0	Language disorder associated with thought disorder	Feb-09
2052	13Z4E00	49540	0	2162	1	Learning difficulties	Feb-09
56376	Z7CD200	466	0	0	0	Learning difficulties	Feb-09
28113	F427J00	102	0	5	0	Leber's congenital amaurosis	Feb-09
45706	F4H1600	137	0	7	0	Leber's hereditary optic atrophy	Feb-09
48037	F4H1711	7	0	0	0	Leber's optic atrophy	Feb-09
53826	G562z00	105	0	7	0	Left bundle branch hemiblock NOS	Feb-09
8933	F222.00	2209	0	147	1	Left hemiplegia	Feb-09
26318	G563.00	283	0	4	0	Left main stem bundle branch block	Feb-09
98383	7904200	1	0	0	0	Left ventricle aorta tunnel right ventricle pul art val con	Jan-10
67657	P542.00	4	0	0	0	Left ventricle to right atrial communication	Feb-09
107397	G5yyD00	792	0	5	0	Left ventricular cardiac dysfunction	Dec-13
884	G581.00	170646	0	11892	23	Left ventricular failure	Feb-09
562	G5y3411	21482	0	436	1	Left ventricular hypertrophy	Feb-09
108457	P69..00	26	0	0	0	Left ventricular outflow tract obstruction	Aug-14
21854	G5yy700	504	0	5	0	Left ventricular thrombosis	Feb-09
69114	C303000	2	0	0	0	Leucinosis	Feb-09
59035	F100z00	9	0	0	0	Leucodystrophy NOS	Feb-09
43857	C10M.00	37	0	2	0	Lipoatrophic diabetes mellitus	Feb-09
34624	C375.14	6	0	2	0	Lipochondrodystrophy	Feb-09
56796	C326z00	39	0	1	0	Lipodystrophy NOS	Feb-09

For peer review only - http://bmjopen.bmj.com/site/about/guidelines.xhtml

39419	A3A2.11	18	0	0	0	"Lipodystrophy, intestinal"	Feb-09
19337	G56y500	1886	0	171	0	Long Q-T syndrome	Feb-09
63395	PB30000	2	0	0	0	Long segment Hirschsprung's disease	Feb-09
47768	C302500	25	0	5	0	Lowe disease	Feb-09
4125	M154.00	6746	0	484	27	Lupus erythematosus	Feb-09
33449	M154000	16	0	0	0	Lupus erythematosus chronicus	Feb-09
40797	M154200	11	0	0	0	Lupus erythematosus migrans	Feb-09
65391	M154300	17	0	0	0	Lupus erythematosus nodularis	Feb-09
7522	M154z00	239	0	4	0	Lupus erythematosus NOS	Feb-09
46148	M154400	77	0	1	0	Lupus erythematosus profundus	Feb-09
44984	M154500	42	0	1	0	Lupus erythematosus tumidus	Feb-09
63955	M154600	2	0	0	0	Lupus erythematosus unguium mutilans	Feb-09
37474	P241.00	61	0	0	0	Macroencephaly	Feb-09
16527	C333.00	337	0	27	0	Macroglobulinaemia	Feb-09
71994	C333z00	6	0	0	0	Macroglobulinaemia NOS	Feb-09
30176	PA14.00	164	0	16	0	Macroglossia	Feb-09
27325	J040.12	120	0	44	0	Macrognathism	Feb-09
73766	J040A00	6	0	0	0	Macrognathism unspecified	Feb-09
72094	P242.00	3	0	0	0	Macrogyria	Feb-09
58909	P37..00	5	0	0	0	Macrophthalmos	Feb-09
61992	PG42012	26	0	0	0	Maffuci's syndrome	Feb-09
30100	C294400	356	0	6	1	Magnesium deficiency	Feb-09
71815	PJ51000	5	0	0	0	Major partial trisomy	Feb-09
31419	J69y700	379	0	32	4	Malabsorption - iron	Feb-09
29513	J69y011	14	0	0	0	Malabsorption due to intolerance to fat	Feb-09
92869	C313911	1	0	0	0	Malabsorption of disaccharide NEC	Feb-09
38805	C313511	24	0	0	0	Malabsorption of glucose	Feb-09
57431	C313011	11	0	0	0	Malabsorption of glucose - galactose	Feb-09

For peer review only - http://bmjopen.bmj.com/site/about/guidelines.xhtml

11934	J69yz13	885	0	50	0	Malabsorption syndrome NOS	Feb-09
54216	PC71.00	20	0	2	0	Male pseudohermaphroditism	Feb-09
37484	PJy6.00	16	0	3	0	Male with structurally abnormal sex chromosome	Feb-09
37070	E110.00	1217	0	13	0	"Manic disorder, single episode"	Feb-09
36611	E110z00	46	0	2	0	"Manic disorder, single episode NOS"	Feb-09
22713	1S42.00	496	0	46	0	Manic mood	Feb-09
26161	E11..13	653	0	27	0	Manic psychoses	Feb-09
109671	C399000	14	0	0	0	Mannose-binding lectin deficiency	May-15
66577	C31y300	32	0	0	0	Mannosidosis	Feb-09
107648	C375000	2	0	0	0	Mannosidosis	Jan-14
37980	G764.11	11	0	0	0	Marable's syndrome	Feb-09
35893	C201.00	4	0	0	0	Marasmic kwashiorkor	Feb-09
103962	A78y300	3	0	0	0	Marburg disease	Apr-12
70356	PKy5600	1	0	0	0	Marchesani syndrome	Feb-09
100022	D112012	1	0	0	0	Marchiafava - Micheli syndrome	Jun-10
56153	F21y000	5	0	0	0	Marchiafava-Bignami disease	Feb-09
3884	P2x4.00	447	0	24	0	Marcus - Gunn syndrome	Feb-09
45341	F4E4113	21	0	0	0	Marcus - Gunn syndrome	Feb-09
936	PKy2.00	4054	0	539	0	Marfan's syndrome	Feb-09
99763	F142000	5	0	1	0	Marie's cerebellar ataxia	Jun-10
11631	C37z.11	885	0	65	0	Marinesco-Sjogren syndrome	Feb-09
62281	C375611	3	0	1	0	Maroteaux - Lamy syndrome	Feb-09
107550	PKy0400	4	0	0	0	Marshall-Smith syndrome	Jan-14
46624	C10C.11	222	0	9	0	Maturity onset diabetes in youth	Feb-09
98392	C10C.12	7	0	0	0	Maturity onset diabetes in youth type 1	Jan-10
59991	C10D.11	31	0	0	0	Maturity onset diabetes in youth type 2	Feb-09
49317	C310000	98	0	6	0	McArdle's disease	Feb-09
42632	PD12.00	53	0	4	0	Medullary cystic disease	Feb-09

For peer review only

41522	PD12z00	5	0	0	0	Medullary cystic disease NOS	Feb-09
64694	PD12y00	4	0	0	0	Medullary cystic disease OS	Feb-09
47135	PD12100	37	0	2	0	"Medullary cystic disease, adult type"	Feb-09
66998	PD12000	4	0	0	0	"Medullary cystic disease, juvenile type"	Feb-09
33400	C156.00	6	0	0	0	Medulloadrenal hyperfunction	Feb-09
35863	J527.00	136	0	5	0	Megacolon excluding Hirschsprung's disease	Feb-09
4816	J527z00	449	0	26	0	Megacolon NOS	Feb-09
65723	D313.14	1	0	0	0	Megakaryocytic hypoplasia	Feb-09
33442	P241.11	64	0	0	0	Megalencephaly	Feb-09
50169	P249.00	52	0	0	0	Megalencephaly	Feb-09
94733	Q48y500	2	0	0	0	Megalencephaly	Feb-09
4475	D01..11	1796	0	102	1	Megaloblastic anaemia	Feb-09
59103	D012112	24	0	0	0	Megaloblastic anaemia due to dietary causes	Feb-09
53799	D01z.11	11	0	0	0	Megaloblastic anaemia NOS	Feb-09
50050	PB56.00	3	0	0	0	Megaloduodenum	Feb-09
71255	PA75.00	1	0	0	0	Megalogastria	Feb-09
40049	K13y700	27	0	0	0	Megaloureter - acquired	Feb-09
5099	PD26.00	120	0	2	0	Megaloureter - congenital	Feb-09
41905	J100.12	7	0	0	0	Megaoesophagus	Feb-09
69752	J100000	2	0	0	1	Megaoesophagus in Chagas' disease	Feb-09
72742	PKyG.00	3	0	0	0	Men ret congen heart dis blepharophim blepharop hypopl teeth	Feb-09
4246	6664	4051	0	128	0	Mental handicap problem	Feb-09
1362	E3...00	8262	0	181	3	Mental retardation	Feb-09
37867	E3z..00	542	0	4	0	Mental retardation NOS	Feb-09
68132	6894	238	0	0	0	Mental retardation screen	Feb-09

For peer review only - http://bmjopen.bmj.com/site/about/guidelines.xhtml

41881	K032y14	246	0	7	0	Mesangiocapillary glomerulonephritis NEC	Feb-09
36342	K032y13	94	0	0	0	Mesangioproliferative glomerulonephritis NEC	Feb-09
12724	C1A0.00	3026	0	48	0	Metabolic syndrome	Feb-09
39481	C10F811	180	0	1	0	Metabolic syndrome X	Feb-09
104331	4Q21000	1	0	1	245	Metadrenaline level	Jun-12
26069	PG45.00	23	0	4	0	Metaphyseal dysostosis	Feb-09
45162	PG45.15	19	0	0	0	Metaphyseal dysplasia	Feb-09
37743	PG44100	2	0	0	0	Metatropic dwarfism	Feb-09
103145	PA15.00	3	0	0	0	Microglossia	Oct-11
15363	J040.15	451	0	21	0	Micrognathism	Feb-09
95850	J040B00	13	0	0	0	Micrognathism unspecified	Feb-09
52652	P336000	4	0	0	0	Microphakia	Feb-09
26462	P31..00	494	0	29	0	Microphthalmos	Feb-09
63439	P31z.00	36	0	1	0	Microphthalmos NOS	Feb-09
48507	P312.00	51	0	2	0	Microphthalmos with other eye anomaly	Feb-09
72284	P310.00	7	0	0	0	"Microphthalmos, unspecified"	Feb-09
161	F26..00	1198522	0	18647	32	Migraine	Feb-09
14700	F26z.00	26163	0	256	1	Migraine NOS	Feb-09
23621	F262z00	803	0	19	0	Migraine variant NOS	Feb-09
103502	F260.11	1592	0	8	0	Migraine with aura	Jan-12
103602	F261.11	325	0	0	0	Migraine without aura	Jan-12
3018	663V100	20542	0	18	0	Mild asthma	Feb-09
33841	F370200	299	0	6	0	Miller-Fisher syndrome	Feb-09
18307	F39X.00	335	0	15	0	"Mitochondrial myopathy, not elsewhere classified"	Feb-09
31727	G133.00	398	0	6	0	Mitral and aortic incompetence	Feb-09
94872	G133.11	4	0	0	0	Mitral and aortic insufficiency	Feb-09
11878	G133.12	3310	0	63	3	Mitral and aortic regurgitation	Feb-09
29158	G13z.00	463	0	16	0	Mitral and aortic valve disease NOS	Feb-09

For peer review only - http://bmjopen.bmj.com/site/about/guidelines.xhtml

21807	G111.11	74	0	12	0	Mitral incompetence - rheumatic	Feb-09
31759	G132.12	13	0	1	0	Mitral incompetence and aortic stenosis	Feb-09
34240	G540100	382	0	5	0	"Mitral incompetence, cause unspecified"	Feb-09
5058	G540000	2806	0	199	0	"Mitral incompetence, non-rheumatic"	Feb-09
33262	G132.00	110	0	4	0	Mitral insufficiency and aortic stenosis	Feb-09
561	G540.16	38256	0	949	3	Mitral regurgitation	Feb-09
22837	G111.12	137	0	0	0	Mitral regurgitation - rheumatic	Feb-09
33907	G132.13	247	0	2	0	Mitral regurgitation and aortic stenosis	Feb-09
1885	G110.00	9886	0	405	2	Mitral stenosis	Feb-09
61250	G131.13	19	0	0	0	Mitral stenosis and aortic incompetence	Feb-09
49355	G131.00	34	0	3	0	Mitral stenosis and aortic insufficiency	Feb-09
50983	G112.12	5	0	0	0	Mitral stenosis with incompetence	Feb-09
44488	G112.00	165	0	0	0	Mitral stenosis with insufficiency	Feb-09
44328	G112.13	72	0	2	0	Mitral stenosis with regurgitation	Feb-09
30443	G11z.00	687	0	11	0	Mitral valve disease NOS	Feb-09
1267	G11..00	5419	0	385	4	Mitral valve diseases	Feb-09
24557	G540z00	427	0	11	0	Mitral valve disorders NOS	Feb-09
2977	G540.00	7682	0	454	5	Mitral valve incompetence	Feb-09
40949	G540.12	31	0	0	0	Mitral valve insufficiency	Feb-09
39916	G540300	307	0	3	0	Mitral valve leaf prolapse	Feb-09
1294	G540.15	11485	0	488	2	Mitral valve prolapse	Feb-09
31839	G540200	799	0	15	0	Mitral valve prolapse	Feb-09
9450	G540.14	3919	0	59	0	Mitral valve regurgitation	Feb-09
25796	H332.00	960	0	2	0	Mixed asthma	Feb-09
31316	E116.00	1458	0	46	0	Mixed bipolar affective disorder	Feb-09

For peer review only - http://bmjopen.bmj.com/site/about/guidelines.xhtml

55064	E116600	92	0	0	0	"Mixed bipolar affective disorder, in full remission"	Feb-09
24689	E116100	31	0	0	0	"Mixed bipolar affective disorder, mild"	Feb-09
63150	E116200	26	0	0	0	"Mixed bipolar affective disorder, moderate"	Feb-09
63583	E116z00	152	0	1	0	"Mixed bipolar affective disorder, NOS"	Feb-09
63651	E116500	7	0	0	0	"Mixed bipolar affective disorder, partial/unspec remission"	Feb-09
54195	E116400	44	0	0	0	"Mixed bipolar affective disorder, severe, with psychosis"	Feb-09
63284	E116300	9	0	0	0	"Mixed bipolar affective disorder, severe, without psychosis"	Feb-09
31535	E116000	237	0	1	0	"Mixed bipolar affective disorder, unspecified"	Feb-09
73492	PJ60.00	8	0	0	0	Mixed gonadal dysgenesis	Feb-09
103752	G561311	13	0	1	0	Mobitz type 1 second degree atrioventricular block	Feb-12
27928	G561300	437	0	18	0	Mobitz type I (Wenckebach) atrioventricular block	Feb-09
13065	663V200	14648	0	13	0	Moderate asthma	Feb-09
302	E310.00	699	0	8	0	"Moderate mental retardation, IQ in range 35-49"	Feb-09
107350	F591C00	446	0	9	0	Moderate sensorineural hearing loss	Dec-13
36560	F326200	108	0	8	0	Moebius congenital oculofacial paralysis	Feb-09
65262	F26y111	3	0	1	0	Moebius' ophthalmoplegic migraine	Feb-09
52270	J094200	11	0	0	0	Moeller's glossodynia exfoliativa	Feb-09
67072	PKy5100	5	0	0	0	Mohr's syndrome	Feb-09

For peer review only - http://bmjopen.bmj.com/site/about/guidelines.xhtml

36276	N043300	127	0	1	0	Monarticular juvenile rheumatoid arthritis	Feb-09
50604	13L2.12	9	0	0	0	Mongol child in family	Feb-09
23489	PJ0..11	171	0	6	0	Mongolism	Feb-09
37105	PJ3..00	25	0	0	0	Monosomies and deletions from the autosomes	Feb-09
98395	PJ3z.00	4	0	0	0	Monosomies and deletions from the autosomes NOS	Jan-10
58584	C375411	31	0	0	0	Morquio - Brailsford syndrome	Feb-09
105345	PJy1300	1	0	0	0	Mosaic including XXXXY	Dec-12
32783	PJy1100	24	0	1	0	Mosaic XO/XX	Feb-09
33269	PJy1000	12	0	2	0	Mosaic XO/XY	Feb-09
64578	PJy1200	2	0	1	0	Mosaic XY/XXY	Feb-09
26140	PJz0.00	93	0	0	0	Mosaicism NOS	Feb-09
23643	PJy5.00	9	0	0	0	"Mosaicism, lines with various numbers of X chromosomes"	Feb-09
65699	F255012	18	0	0	0	Motor epilepsy	Feb-09
93694	PJ9..00	15	0	1	0	Mowat-Wilson syndrome	Feb-09
11946	PD13.11	745	0	25	0	Multicystic kidney	Feb-09
30041	C164.13	227	0	3	0	Multicystic ovaries	Feb-09
18331	PD13.00	251	0	4	0	Multicystic renal dysplasia	Feb-09
16530	PK7..00	134	0	6	0	Multiple congenital anomalies NOS	Feb-09
98724	PK7z.00	4	0	0	0	Multiple congenital anomalies NOS	Mar-10
50980	P841.12	22	0	0	0	Multiple congenital bronchogenic cysts	Feb-09
109441	P240100	1	0	0	0	Multiple congenital cerebral cysts	Mar-15
69886	F201.00	16	0	0	0	Multiple sclerosis of the spinal cord	Feb-09
57338	G544X00	62	0	3	0	"Multiple valve disease, unspecified"	Feb-09
40239	G544.00	46	0	2	0	Multiple valve diseases	Feb-09
22796	ZT4C211	229	0	14	0	Mute	Feb-09

For peer review only - http://bmjopen.bmj.com/site/about/guidelines.xhtml

36541	E2D2211	56	0	4	0	Mutism of childhood or adolescence	Feb-09
27515	F380z00	179	0	2	0	Myasthenia gravis NOS	Feb-09
95005	F381200	1	0	0	0	Myasthenic syndrome due to botulism	Feb-09
39420	F381300	39	0	2	0	Myasthenic syndrome due to diabetic amyotrophy	Feb-09
51640	F381.00	6	0	1	0	Myasthenic syndrome due to disease EC	Feb-09
65825	F381z00	11	0	0	0	Myasthenic syndrome due to disease NOS	Feb-09
61069	F381400	8	0	0	0	Myasthenic syndrome due to hypothyroidism	Feb-09
57551	F381100	2	0	0	0	Myasthenic syndrome due to other malignancy	Feb-09
56973	F381500	6	0	0	0	Myasthenic syndrome due to pernicious anaemia	Feb-09
47695	F381600	3	0	0	0	Myasthenic syndrome due to thyrotoxicosis	Feb-09
94690	F13B.00	191	0	13	0	Myoclonic dystonia	Feb-09
45602	F132200	80	0	4	0	Myoclonic encephalopathy	Feb-09
8487	F132z12	1879	0	176	0	Myoclonic seizure	Feb-09
37897	F132z00	229	0	10	0	Myoclonus NOS	Feb-09
96966	N248000	1417	0	28	0	Myofascial pain syndrome	Jun-09
105696	PG42100	1	0	0	0	Myotonic chondrodysplasia	Jan-13
44867	F392z00	97	0	3	0	Myotonic disorder NOS	Feb-09
11779	F271.00	4394	0	441	0	Narcolepsy	Feb-09
56614	Q488.00	23	0	0	0	Neonatal cerebral ischaemia	Feb-09
48202	Q48A.00	49	0	0	0	Neonatal cerebral leukomalacia	Feb-09
65686	Q444.12	4	0	0	0	Neonatal hypoparathroidism	Feb-09
106500	Q44A.00	1	0	0	0	Neonatal hypoparathyroidism	Aug-13
99435	N000500	14	0	0	0	Neonatal lupus erythematosus	May-10

For peer review only - http://bmjopen.bmj.com/site/about/guidelines.xhtml

37782	F251100	110	0	2	0	Neonatal myoclonic epilepsy	Feb-09
10647	K02..11	392	0	10	0	Nephritis - chronic	Feb-09
4850	K03..11	203	0	6	0	Nephritis and nephropathy unspecified	Feb-09
33580	K03..00	491	0	17	0	Nephritis and nephropathy unspecified	Feb-09
94350	K032z00	3	0	0	0	Nephritis unsp+membranoprolif glomerulonephritis lesion NOS	Feb-09
67193	K032y00	29	0	1	0	Nephritis unsp+OS membranoprolif glomerulonephritis lesion	Feb-09
2773	K0...00	1430	0	97	2	"Nephritis, nephrosis and nephrotic syndrome"	Feb-09
30310	K081.00	249	0	7	0	Nephrogenic diabetes insipidus	Feb-09
42042	PD12011	17	0	0	0	Nephronophthisis	Feb-09
109106	PD12200	2	0	0	0	Nephronophthisis - medullary cystic disease	Jan-15
68112	C373600	7	0	0	0	Nephropathic amyloidosis	Feb-09
11875	K02..12	875	0	41	0	Nephropathy - chronic	Feb-09
41159	K0C1.00	63	0	0	0	Nephropathy induced by other drugs meds and biologl substncs	Feb-09
57784	K0C2.00	13	0	0	0	Nephropathy induced by unspec drug medicament or biol subs	Feb-09
38312	L162.12	16	0	2	1	Nephropathy NOS in pregnancy without hypertension	Feb-09
11873	K03..12	1478	0	43	0	"Nephropathy, unspecified"	Feb-09
21947	K017.00	129	0	1	0	Nephrotic syn difus mesangial prolifertiv glomerulonephritis	Feb-09
21989	K019.00	73	0	1	0	"Nephrotic syn,diffuse mesangiocapillary glomerulonephritis"	Feb-09
50472	K018.00	4	0	0	0	"Nephrotic syn,difus endocapilary proliftv glomerulonephritis"	Feb-09

For peer review only - http://bmjopen.bmj.com/site/about/guidelines.xhtml

2999	K01..00	15444	0	855	5	Nephrotic syndrome	Feb-09
47922	K01x000	80	0	2	0	Nephrotic syndrome in amyloidosis	Feb-09
2471	K01x100	283	0	7	0	Nephrotic syndrome in diabetes mellitus	Feb-09
108816	K01x.00	2	0	0	0	Nephrotic syndrome in diseases EC	Nov-14
99201	K01x200	1	0	0	0	Nephrotic syndrome in malaria	Apr-10
58750	K01x300	7	0	0	0	Nephrotic syndrome in polyarteritis nodosa	Feb-09
47672	K01x400	57	0	3	0	Nephrotic syndrome in systemic lupus erythematosus	Feb-09
27427	K01z.00	305	0	9	0	Nephrotic syndrome NOS	Feb-09
1803	K011.00	515	0	9	0	Nephrotic syndrome with membranous glomerulonephritis	Feb-09
29634	K013.00	180	0	4	0	Nephrotic syndrome with minimal change glomerulonephritis	Feb-09
94373	K01y.00	4	0	0	0	Nephrotic syndrome with other pathological kidney lesions	Feb-09
9840	K010.00	19	0	0	0	Nephrotic syndrome with proliferative glomerulonephritis	Feb-09
56987	K01A.00	12	0	0	0	"Nephrotic syndrome, dense deposit disease"	Feb-09
17365	K01B.00	52	0	0	0	"Nephrotic syndrome, diffuse crescentic glomerulonephritis"	Feb-09
22852	K015.00	208	0	20	0	"Nephrotic syndrome, focal and segmental glomerular lesions"	Feb-09
23913	K014.00	66	0	1	0	"Nephrotic syndrome, minor glomerular abnormality"	Feb-09
99644	K012.00	4	0	0	0	Nephrotic syndrome+membranoproliferative glomerulonephritis	Jun-10
3171	F591211	2006	0	241	0	Nerve deafness	Feb-09
104026	F38..11	42	0	5	0	Neuromuscular disease	May-12

For peer review only - http://bmjopen.bmj.com/site/about/guidelines.xhtml

64871	N082N00	5	0	0	0	Neuromuscular dislocation of the hip	Feb-09
44795	F210.00	149	0	4	0	Neuromyelitis optica	Feb-09
101786	F392400	58	0	1	0	Neuromyotonia	Feb-11
10234	J61y100	32345	0	773	2	Non-alcoholic fatty liver	Feb-09
57897	D111.00	7	0	1	0	Non-autoimmune haemolytic anaemia	Feb-09
94214	D111z00	2	0	0	0	Non-autoimmune haemolytic anaemia NOS	Feb-09
69278	C109E00	18	0	0	0	Non-insulin depend diabetes mellitus with diabetic cataract	Feb-09
40962	C109H00	10	0	0	0	Non-insulin dependent d m with neuropathic arthropathy	Feb-09
506	C100112	95279	0	4539	11	Non-insulin dependent diabetes mellitus	Feb-09
8403	C109700	1745	0	45	0	Non-insulin dependent diabetes mellitus - poor control	Feb-09
24693	C109G00	4	0	0	0	Non-insulin dependent diabetes mellitus with arthropathy	Feb-09
40401	C109500	14	0	1	0	Non-insulin dependent diabetes mellitus with gangrene	Feb-09
43785	C109D00	10	0	0	0	Non-insulin dependent diabetes mellitus with hypoglyca coma	Feb-09
72320	C109A00	3	0	1	0	Non-insulin dependent diabetes mellitus with mononeuropathy	Feb-09
59365	C109C00	60	0	2	0	Non-insulin dependent diabetes mellitus with nephropathy	Feb-09
45467	C109B00	14	0	0	0	Non-insulin dependent diabetes mellitus with polyneuropathy	Feb-09
34912	C109400	229	0	4	0	Non-insulin dependent diabetes mellitus with ulcer	Feb-09

For peer review only - http://bmjopen.bmj.com/site/about/guidelines.xhtml

54212	C109F00	9	0	0	0	Non-insulin-dependent d m with peripheral angiopath	Feb-09
62146	C109300	9	0	2	0	Non-insulin-dependent diabetes mellitus with multiple comps	Feb-09
55842	C109200	15	0	0	0	Non-insulin-dependent diabetes mellitus with neuro comps	Feb-09
50429	C109100	19	0	3	0	Non-insulin-dependent diabetes mellitus with ophthalm comps	Feb-09
52303	C109000	16	0	2	0	Non-insulin-dependent diabetes mellitus with renal comps	Feb-09
29979	C109900	267	0	6	0	Non-insulin-dependent diabetes mellitus without complication	Feb-09
10068	PKy8000	741	0	36	0	Noonan's syndrome	Feb-09
15566	E203z00	1842	0	105	0	Obsessive-compulsive disorder NOS	Feb-09
3208	E203.00	16765	0	2315	0	Obsessive-compulsive disorders	Feb-09
46177	PD2z.00	69	0	2	0	Obstructive defect of renal pelvis or ureter NOS	Feb-09
99128	P6y3.00	10	0	0	0	Obstructive heart anomaly NEC	Apr-10
97818	P6y3z00	4	0	0	0	Obstructive heart anomaly NEC NOS	Oct-09
101873	337C.00	7	0	0	13	Obstructive ventilatory defect	Mar-11
108543	PKy5M00	3	0	0	0	Oculofaciocardiodental syndrome	Aug-14
68816	A213.00	1	0	0	0	Oculoglandular tularaemia	Feb-09
44064	PG0E.00	2	0	0	0	Oculomandibular dysostosis	Feb-09
111653	PKy5900	1	0	0	0	Oculo-palato-digital syndrome	Feb-17
38448	F391700	68	0	3	0	Oculopharyngeal muscular dystrophy	Feb-09
37408	PA3..00	39	0	1	0	"Oesophageal atresia, stenosis and fistula"	Feb-09
16713	J103.00	1924	0	53	0	Oesophageal stricture and stenosis	Feb-09
41141	J103z00	658	0	8	0	Oesophageal stricture and stenosis NOS	Feb-09
37868	PA32100	52	0	1	0	Oesophagotracheal fistula	Feb-09

For peer review only - http://bmjopen.bmj.com/site/about/guidelines.xhtml

61322	PKyG.11	24	0	0	0	Ohdo blepharophimosis syndrome	Feb-09
51998	F259.11	21	0	0	0	Ohtahara syndrome	Feb-09
38418	J32..11	65	0	1	0	Omphalocele	Feb-09
100368	J321000	1	0	0	0	Omphalocele with obstruction	Aug-10
3826	F4H1.00	5675	0	242	0	Optic atrophy	Feb-09
95763	F4H1300	4	0	0	0	Optic atrophy due to retinal dystrophy	Feb-09
41833	F4H1z00	172	0	3	0	Optic atrophy NOS	Feb-09
47676	F4H5z00	15	0	2	0	Optic chiasm disorder NOS	Feb-09
47132	F4H5.00	35	0	2	0	Optic chiasm disorders	Feb-09
63298	SJ01.00	4	0	0	0	Optic chiasm injury	Feb-09
26835	F4H3z00	280	0	2	0	Optic neuritis NOS	Feb-09
49307	PKy5000	29	0	6	0	Oral - facial - digital syndrome	Feb-09
56160	PGy0212	2	0	0	0	Orbinsky syndrome	Feb-09
29616	J08z900	90	0	2	0	Orofacial Crohn's disease	Feb-09
23356	F138100	657	0	29	5	Orofacial dyskinesia	Feb-09
22814	J08zB00	346	0	12	0	Orofacial granulomatosis	Feb-09
3156	N327.00	5189	0	322	1	Osteochondritis dissecans	Feb-09
38335	N327100	51	0	0	0	Osteochondritis dissecans of lateral femoral condyle	Feb-09
42068	N327y00	26	0	2	0	Osteochondritis dissecans of other site	Feb-09
54563	N327000	63	0	0	0	Osteochondritis dissecans of patella	Feb-09
70914	N327400	16	0	0	0	Osteochondritis dissecans of the capitellum	Feb-09
34206	N327800	7	0	0	0	Osteochondritis dissecans of the femoral head	Feb-09
103515	N327300	2	0	0	0	Osteochondritis dissecans of the humeral head	Jan-12
97447	N327500	3	0	0	0	Osteochondritis dissecans of the radial head	Aug-09

For peer review only - http://bmjopen.bmj.com/site/about/guidelines.xhtml

38518	N327900	48	0	0	0	Osteochondritis dissecans of the talus	Feb-09
97466	N327700	2	0	0	0	Osteochondritis dissecans of the wrist	Aug-09
63565	PGW..00	4	0	2	0	Osteochondrodyspl with defct growth tub bone spine unspec	Feb-09
68427	PG5z.11	10	0	1	0	Osteochondrodysplasia	Feb-09
55775	C375.17	19	0	0	0	Osteochondrodystrophy	Feb-09
22861	N32zz00	157	0	2	0	Osteochondropathy NOS	Feb-09
17135	N32z300	969	0	7	0	Osteochondrosis NOS	Feb-09
36753	PG5..00	29	0	2	0	Osteodysplasia	Feb-09
55331	PG5z.00	12	0	1	0	Osteodysplasia NOS	Feb-09
96403	PG50.00	5	0	0	0	"Osteodysplasia, unspecified"	Apr-09
38450	PG5..11	118	0	2	0	Osteodystrophy	Feb-09
26864	N33zD00	318	0	5	0	Osteolysis	Feb-09
22417	N33zH00	172	0	9	0	Osteolytic lesion	Feb-09
29734	N33z.11	101	0	25	0	Osteolytic lesion	Feb-09
4794	B73..12	4464	0	443	0	Osteoma	Feb-09
29735	B30..12	128	0	1	0	Osteoma	Feb-09
10450	C28..11	4171	0	150	8	Osteomalacia	Feb-09
37350	C282.00	144	0	4	0	Osteomalacia unspecified	Feb-09
42657	N334900	398	0	9	0	Osteonecrosis due to drugs	Feb-09
69157	N334C00	7	0	0	0	Osteonecrosis due to haemoglobinopathy	Feb-09
45737	N334A00	181	0	8	0	Osteonecrosis due to previous trauma	Feb-09
58843	N334B00	10	0	0	0	Osteonecrosis in caisson disease	Feb-09
59172	PG42.16	16	0	0	0	Osteopathia striata	Feb-09
54213	N307.00	54	0	5	0	Osteopathy from poliomyelitis	Feb-09
37298	N3...00	200	0	4	0	Osteopathy/chondropathy/acquired musculoskeletal deformity	Feb-09
101249	2126600	389	0	24	0	Osteopenia resolved	Jan-11

For peer review only - http://bmjopen.bmj.com/site/about/guidelines.xhtml

21970	N21z312	130	0	4	0	Osteophyte of unspecified site	Feb-09
39943	PG53.00	71	0	2	0	Osteopoikilosis	Feb-09
277	N330.00	329884	0	14435	329	Osteoporosis	Feb-09
25650	N330D00	67	0	2	0	Osteoporosis due to corticosteroids	Feb-09
31580	N330A00	10	0	0	0	Osteoporosis in endocrine disorders	Feb-09
60433	N330900	17	0	0	0	Osteoporosis in multiple myelomatosis	Feb-09
34798	N330z00	4190	0	116	0	Osteoporosis NOS	Feb-09
33526	N331300	55	0	0	0	Osteoporosis of disuse with pathological fracture	Feb-09
51053	P561.00	53	0	0	0	Ostium primum defect	Feb-09
54243	P55z.00	73	0	0	0	Ostium secundum atrial septal defect NOS	Feb-09
104307	2126800	83	0	8	0	Ostium secundum atrial septal defect resolved	Jun-12
54838	C155z00	27	0	0	0	Other adrenal hypofunction NOS	Feb-09
67273	C152700	1	0	0	0	Other adrenogenital syndrome with salt loss	Feb-09
69764	C152800	15	0	1	0	Other adrenogenital syndrome without mention of salt loss	Feb-09
50220	P72..00	70	0	2	0	Other anomalies of aorta	Feb-09
44781	P72z.00	39	0	0	0	Other anomalies of aorta NOS	Feb-09
50278	PB5..00	12	0	0	0	Other anomalies of intestine	Feb-09
29373	P83..00	22	0	3	0	"Other anomalies of larynx, trachea and bronchus"	Feb-09
36779	P83z.00	6	0	0	0	"Other anomalies of larynx, trachea or bronchus NOS"	Feb-09
11945	P72zz00	60	0	1	0	Other anomaly of aorta NOS	Feb-09
104976	P83yy00	3	0	0	0	Other anomaly of bronchus	Sep-12
63507	P83yw00	12	0	0	0	Other anomaly of larynx	Feb-09
63637	P83y.00	4	0	0	0	"Other anomaly of larynx, trachea and bronchus"	Feb-09

For peer review only - http://bmjopen.bmj.com/site/about/guidelines.xhtml

103042	P83yz00	1	0	0	0	"Other anomaly of larynx, trachea or bronchus NOS"	Oct-11
69913	PG0yz00	9	0	0	0	Other anomaly of skull or face bone NOS	Feb-09
56878	PG1y.00	33	0	1	0	Other anomaly of spine	Feb-09
22768	PG1yz00	107	0	11	1	Other anomaly of spine NOS	Feb-09
70193	P83yx00	5	0	0	0	Other anomaly of trachea	Feb-09
66220	PFyz.00	7	0	1	0	Other anomaly of unspecified limb NOS	Feb-09
71400	F15y.00	5	0	4	0	Other anterior horn cell disease	Feb-09
15488	C134z00	1176	0	255	4	Other anterior pituitary disorder NOS	Feb-09
23560	C131.00	81	0	4	0	Other anterior pituitary hyperfunction	Feb-09
50762	F130z00	35	0	0	0	Other basal ganglia degenerative disease NOS	Feb-09
21863	F130.00	207	0	16	8	Other basal ganglia degenerative diseases	Feb-09
72653	G565300	29	0	6	0	Other bilateral bundle branch block	Feb-09
37613	J66yz00	76	0	5	4	Other bile duct disorder NOS	Feb-09
29066	J66..00	78	0	1	0	Other biliary tract disorders	Feb-09
69100	F427H00	7	0	0	0	Other Bruch's membrane dystrophy	Feb-09
39003	G565z00	146	0	0	0	Other bundle branch block NOS	Feb-09
31133	G57yz00	191	0	6	0	Other cardiac dysrhythmia NOS	Feb-09
7827	G57y.00	2771	0	396	18	Other cardiac dysrhythmias	Feb-09
65499	P7yzz00	8	0	0	0	Other cardiovascular system anomaly NOS	Feb-09
101592	P7yz.00	6	0	0	0	Other cardiovascular system anomaly NOS	Jan-11
34976	F11y.00	30	0	1	0	Other cerebral degeneration	Feb-09
31524	F11yz00	27	0	1	0	Other cerebral degeneration NOS	Feb-09
31892	F11..00	241	0	1	0	Other cerebral degenerations	Feb-09

For peer review only - http://bmjopen.bmj.com/site/about/guidelines.xhtml

104782	F2By.00	5	0	0	0	Other cerebral palsy	Aug-12
71819	E14y.00	1	0	1	0	Other childhood psychoses	Feb-09
66757	E14yz00	4	0	0	0	Other childhood psychoses NOS	Feb-09
60960	K02y.00	12	0	0	0	Other chronic glomerulonephritis	Feb-09
63615	K02yz00	12	0	0	0	Other chronic glomerulonephritis NOS	Feb-09
54113	G41y.00	169	0	0	0	Other chronic pulmonary heart disease	Feb-09
71046	G41yz00	24	0	0	0	Other chronic pulmonary heart disease NOS	Feb-09
26291	PF5r.00	247	0	20	0	Other congenital anomalies of fingers	Feb-09
66010	PF5u.00	23	0	0	0	Other congenital anomalies of forearm	Feb-09
59150	PF6v.00	16	0	1	0	Other congenital anomalies of lower leg	Feb-09
25190	PF6x.00	45	0	2	0	Other congenital anomalies of pelvis	Feb-09
55114	PF6xz00	5	0	0	0	Other congenital anomalies of pelvis NOS	Feb-09
65384	P76..11	8	0	0	0	Other congenital anomalies of peripheral arteries	Feb-09
22070	P76..12	109	0	23	0	Other congenital anomalies of peripheral veins	Feb-09
31378	PG3x.00	72	0	2	0	Other congenital anomalies of ribs	Feb-09
63972	PF5w.00	21	0	0	0	Other congenital anomalies of shoulder	Feb-09
51424	PG3y.00	13	0	0	0	Other congenital anomalies of sternum	Feb-09
92299	PF6w.00	9	0	0	0	Other congenital anomalies of upper leg	Feb-09

For peer review only - http://bmjopen.bmj.com/site/about/guidelines.xhtml

48510	PF5x.00	21	0	2	0	Other congenital anomalies of whole arm	Feb-09
61558	PF5uz00	7	0	0	0	Other congenital anomaly forearm NOS	Feb-09
107541	PK1yz00	1	0	0	0	Other congenital anomaly of adrenal gland NOS	Dec-13
94116	PB6yy00	4	0	0	0	Other congenital anomaly of hepatic or bile ducts	Feb-09
66182	PB6yw00	12	0	0	0	Other congenital anomaly of liver	Feb-09
37517	PA25y00	31	0	3	0	Other congenital anomaly of palate	Feb-09
73738	P76yz00	12	0	0	0	Other congenital anomaly of peripheral vascular system NOS	Feb-09
22437	PCyw.00	247	0	58	0	Other congenital anomaly of testis or scrotum	Feb-09
53178	F23y.00	38	0	0	0	Other congenital cerebral palsy	Feb-09
9778	P7...00	48	0	3	0	Other congenital circulatory system anomalies	Feb-09
59031	PD1yz00	8	0	0	0	Other congenital cystic kidney disease NOS	Feb-09
42998	PB...00	31	0	1	0	Other congenital digestive system anomaly	Feb-09
5621	P6...00	654	0	30	0	Other congenital heart anomalies	Feb-09
63834	PF63z00	38	0	1	0	Other congenital hip joint deformity NOS	Feb-09
14649	PF...00	435	0	42	0	Other congenital limb anomalies	Feb-09
37167	PE9..11	243	0	12	0	Other congenital musculoskeletal deformity	Feb-09
62976	P355.00	29	0	0	0	Other congenital retinal changes	Feb-09
25692	P355z00	234	0	21	0	Other congenital retinal changes NOS	Feb-09
72991	PA...00	5	0	0	0	Other congenital upper alimentary tract anomalies	Feb-09

For peer review only - http://bmjopen.bmj.com/site/about/guidelines.xhtml

68138	7A04.00	3	0	0	0	Other connection from aorta to pulmonary artery	Feb-09
44962	C153.00	9	0	0	0	Other corticoadrenal overactivity	Feb-09
57274	D01z.00	81	0	3	0	Other deficiency anaemias NOS	Feb-09
69820	C13y400	5	0	1	0	Other diencephalic syndrome	Feb-09
95921	PG44z00	2	0	0	0	Other dwarfing syndromes NOS	Feb-09
38307	F25y.00	338	0	10	0	Other forms of epilepsy	Feb-09
9979	F25yz00	364	0	5	0	Other forms of epilepsy NOS	Feb-09
28031	F26y.00	505	0	4	0	Other forms of migraine	Feb-09
28092	F26yz00	453	0	3	0	Other forms of migraine NOS	Feb-09
32176	F4K5600	61	0	0	0	Other forms of nystagmus	Feb-09
73663	P74z.00	2	0	0	0	Other great vein anomalies	Feb-09
73554	P74zz00	6	0	0	0	Other great vein anomaly NOS	Feb-09
39843	G566.00	280	0	1	0	Other heart block	Feb-09
46178	G566z00	168	0	1	0	Other heart block NOS	Feb-09
27304	C111.00	362	0	7	0	Other hyperinsulinism	Feb-09
50491	C111z00	51	0	2	0	Other hyperinsulinism NOS	Feb-09
44026	N10y.00	57	0	4	2	Other inflammatory spondylopathies	Feb-09
37892	N10yz00	37	0	0	0	Other inflammatory spondylopathies NOS	Feb-09
7196	N045.00	3433	0	94	0	Other juvenile arthritis	Feb-09
50425	N326.00	13	0	0	0	Other juvenile osteochondroses	Feb-09
43899	F427700	57	0	0	0	Other pigmented retinal dystrophies	Feb-09
44186	C13y.00	5	0	0	0	Other pituitary disorders + diencephalohypophyseal syndromes	Feb-09
22836	C163300	308	0	20	0	Ovarian hypogonadism	Feb-09
23802	C163.11	24	0	2	0	Ovarian hypogonadism	Feb-09
37902	N310z00	367	0	4	0	Paget's disease NOS	Feb-09
3528	N310.11	13822	0	1224	12	Paget's disease of bone	Feb-09

For peer review only - http://bmjopen.bmj.com/site/about/guidelines.xhtml

56078	N310y00	95	0	5	0	Paget's disease OS	Feb-09
95037	N310A00	1	0	0	0	Paget's disease-carpal bone	Feb-09
50862	N310000	5	0	0	0	Paget's disease-cervical spine	Feb-09
68199	N310500	17	0	0	0	Paget's disease-clavicle	Feb-09
57905	N310E00	38	0	3	0	Paget's disease-femur	Feb-09
72456	N310H00	1	0	0	0	Paget's disease-fibula	Feb-09
63779	N310700	5	0	0	0	Paget's disease-humerus	Feb-09
56021	N310200	48	0	3	0	Paget's disease-lumbar spine	Feb-09
66795	N310x00	16	0	0	0	Paget's disease-multiple sites	Feb-09
56897	N310F00	6	0	0	0	Paget's disease-patella	Feb-09
27501	N310D00	186	0	1	0	Paget's disease-pelvis	Feb-09
65387	N310800	6	0	1	0	Paget's disease-radius	Feb-09
56578	N310300	23	0	0	0	Paget's disease-sacrum	Feb-09
70397	N310600	3	0	0	0	Paget's disease-scapula	Feb-09
51146	N310P00	29	0	1	0	Paget's disease-skull	Feb-09
71514	N310100	4	0	1	0	Paget's disease-thoracic spine	Feb-09
35769	N310G00	34	0	0	0	Paget's disease-tibia	Feb-09
73964	N310900	1	0	0	0	Paget's disease-ulna	Feb-09
5026	C132.00	2554	0	129	14	Panhypopituitarism	Feb-09
33653	C132z00	54	0	0	0	Panhypopituitarism NOS	Feb-09
98998	PKy5011	5	0	2	0	Papillon-Leage-Psaume syndrome	Apr-10
1494	E103.00	27706	0	1069	0	Paranoid schizophrenia	Feb-09
36172	E103500	323	0	0	0	Paranoid schizophrenia in remission	Feb-09
9281	E103z00	346	0	4	0	Paranoid schizophrenia NOS	Feb-09
99040	F230.11	2	0	0	0	Paraplegia - congenital	Apr-10
12386	C332z00	742	0	28	0	Paraproteinaemia NOS	Feb-09
64789	C12z.00	48	0	0	0	Parathyroid disorder NOS	Feb-09
16433	C12..00	456	0	72	2	Parathyroid gland disorders	Feb-09
107472	G573600	155	0	6	0	Paroxysmal atrial flutter	Dec-13
23647	G570100	178	0	1	0	Paroxysmal atrioventricular tachycardia	Feb-09
51845	G570200	51	0	2	0	Paroxysmal junctional tachycardia	Feb-09

For peer review only - http://bmjopen.bmj.com/site/about/guidelines.xhtml

35612	D112100	252	0	9	0	Paroxysmal nocturnal haemoglobinuria	Feb-09
29491	G570300	296	0	23	0	Paroxysmal nodal tachycardia	Feb-09
107057	F139000	6	0	0	0	Paroxysmal non-kinesigenic dyskinesia	Oct-13
4940	G570.00	11026	0	444	0	Paroxysmal supraventricular tachycardia	Feb-09
35124	G570z00	274	0	11	0	Paroxysmal supraventricular tachycardia NOS	Feb-09
1381	G572z00	7791	0	848	1	Paroxysmal tachycardia NOS	Feb-09
25266	G572.00	805	0	14	0	Paroxysmal tachycardia unspecified	Feb-09
24409	F335.11	125	0	8	0	Parsonage - Aldren - Turner syndrome	Feb-09
98870	F255311	3	0	0	0	Partial epilepsy with autonomic symptoms	Apr-10
32288	F254.00	466	0	12	0	Partial epilepsy with impairment of consciousness	Feb-09
31920	F254z00	212	0	8	0	Partial epilepsy with impairment of consciousness NOS	Feb-09
26015	F255.00	391	0	15	0	Partial epilepsy without impairment of consciousness	Feb-09
27526	F255z00	126	0	4	0	Partial epilepsy without impairment of consciousness NOS	Feb-09
26733	F255y00	63	0	1	0	Partial epilepsy without impairment of consciousness OS	Feb-09
68651	7114200	4	0	0	0	Partial parathyroidectomy + parathyroid tissue transposition	Feb-09
43202	7114300	277	0	1	0	Partial parathyroidectomy NEC	Feb-09
101309	PJ02.11	5	0	0	0	Partial trisomy 21 in Down's syndrome	Jan-11
100174	PJ51z00	3	0	0	0	Partial trisomy syndrome NOS	Jul-10
19062	PJ51.00	38	0	0	0	Partial trisomy syndromes	Feb-09

For peer review only - http://bmjopen.bmj.com/site/about/guidelines.xhtml

50985	D402.16	5	0	0	0	Pelger - Huet anomaly	Feb-09
31444	C252.00	45	0	3	0	Pellagra	Feb-09
40319	N216211	125	0	2	0	Pellegrini - Stieda syndrome	Feb-09
58116	M145z00	82	0	1	0	Pemphigoid NOS	Feb-09
53763	M144z00	21	0	0	0	Pemphigus NOS	Feb-09
11892	C030.00	132	0	4	0	Pendred's syndrome	Feb-09
96277	G573400	247	0	2	0	Permanent atrial fibrillation	Apr-09
28901	F361.00	349	0	32	0	Peroneal muscular atrophy	Feb-09
70040	F361z00	10	0	0	0	Peroneal muscular atrophy NOS	Feb-09
2907	F250000	11582	0	1229	1	Petit mal (minor) epilepsy	Feb-09
15607	PK60.00	249	0	27	0	Peutz - Jegher's syndrome	Feb-09
3354	K274.00	24239	0	3840	0	Peyronie's disease	Feb-09
18231	B540.11	968	0	67	4	Phaeochromocytoma	Feb-09
52030	AB68.00	3	0	0	0	Phaeohyphomycosis	Feb-09
4318	C301.00	3036	0	3989	3906	Phenylketonuria	Feb-09
38071	13VM.00	844	0	16	0	Physical disability	Feb-09
3970	6665	1145	0	30	0	Physical handicap problem	Feb-09
46999	Z88..11	7	0	0	0	Physical health handicap	Feb-09
93788	Z77C.32	1	0	0	0	Physical health handicap	Feb-09
11136	F111.00	316	0	13	0	Pick's disease	Feb-09
59140	G532100	1	0	0	0	Pick's disease of heart	Feb-09
24755	C38y.11	152	0	14	0	Pickwickian syndrome	Feb-09
38294	C38y000	45	0	6	0	Pickwickian syndrome	Feb-09
4046	PG0C.00	816	0	16	0	Pierre - Robin syndrome	Feb-09
96210	PG0J.00	7	0	0	0	Pierre Robin association	Apr-09
95730	N321511	3	0	0	0	Pierson's disease	Feb-09
68146	C1zy000	5	0	1	0	Pineal gland dysfunction	Feb-09
39541	710..11	36	0	3	0	Pineal gland operations	Feb-09
2321	B7H2.11	6910	0	352	3	Pituitary adenoma	Feb-09
53682	C150200	36	0	0	0	Pituitary dependent Cushing's syndrome	Feb-09
12449	C13z.00	241	0	12	0	Pituitary disorders NOS	Feb-09

For peer review only - http://bmjopen.bmj.com/site/about/guidelines.xhtml

20287	C133.00	437	0	53	16	Pituitary dwarfism	Feb-09
61409	C133z00	2	0	0	0	Pituitary dwarfism NOS	Feb-09
36838	M163.11	23	0	0	0	Pityriasis circinata	Feb-09
107401	M162311	203	0	3	0	Pityriasis lichenoides chronica	Dec-13
107491	M162111	71	0	6	0	Pityriasis lichenoides et varioliformis acuta	Dec-13
36466	M15y200	172	0	3	0	Pityriasis rubra (Hebra)	Feb-09
48641	M165200	17	0	0	0	Pityriasis streptogenes	Feb-09
62323	G75..00	12	0	0	0	Polyarteritis nodosa and allied conditions	Feb-09
68136	G75z.00	128	0	0	0	Polyarteritis nodosa and allied conditions NOS	Feb-09
1670	N065z11	20288	0	1929	38	Polyarthritis	Feb-09
7454	N065.11	29871	0	2919	4	Polyarthropathy NEC	Feb-09
4504	PD1..13	1782	0	250	0	Polycystic kidney	Feb-09
4503	PD11.00	6901	0	595	3	Polycystic kidney disease	Feb-09
56852	PD11z00	226	0	8	0	Polycystic kidney disease NOS	Feb-09
4505	PD11100	482	0	30	0	"Polycystic kidneys, adult type"	Feb-09
44611	D410300	22	0	0	0	Polycythaemia due to cyanotic respiratory disease	Feb-09
68850	Q454000	4	0	0	0	Polycythaemia due to donor twin transfusion	Feb-09
102672	5A14.00	0	0	0	1	Polycythaemia irradiation	Jul-11
64182	Q454z00	27	0	0	0	Polycythaemia neonatorum NOS	Feb-09
5542	B934.11	2103	0	183	0	Polycythaemia rubra vera	Feb-09
40691	PF0z.00	136	0	0	0	Polydactyly NOS	Feb-09
102958	C320600	75	0	0	0	Polygenic hypercholesterolaemia	Sep-11
39898	C18..00	32	0	0	0	Polyglandular dysfunction and related disorders	Feb-09
47995	C18z.00	10	0	0	0	Polyglandular dysfunction NOS	Feb-09
92901	C183.00	1	0	0	0	Polyglandular hyperfunction	Feb-09
1408	N20..00	300629	0	6946	44	Polymyalgia rheumatica	Feb-09

For peer review only - http://bmjopen.bmj.com/site/about/guidelines.xhtml

15511	N004.00	3141	0	178	1	Polymyositis	Feb-09
93927	N231400	44	0	0	0	Polymyositis ossificans	Feb-09
28294	F326100	80	0	5	0	Polyneuritis cranialis	Feb-09
24222	F376.00	61	0	2	0	Polyneuropathy due to drugs	Feb-09
66336	F374000	15	0	1	0	Polyneuropathy in amyloidosis	Feb-09
73337	F374100	1	0	0	0	Polyneuropathy in beriberi	Feb-09
57313	F371.00	8	0	0	0	Polyneuropathy in collagen vascular disease	Feb-09
71258	F371z00	4	0	0	0	Polyneuropathy in collagen vascular disease NOS	Feb-09
31790	F372.00	1506	0	33	0	Polyneuropathy in diabetes	Feb-09
52089	F374300	1	0	0	0	Polyneuropathy in diphtheria	Feb-09
56272	F374.00	16	0	2	0	Polyneuropathy in disease EC	Feb-09
63555	F374z00	28	0	2	0	Polyneuropathy in disease NOS	Feb-09
44095	F371000	37	0	4	0	Polyneuropathy in disseminated lupus erythematosus	Feb-09
39692	F374400	57	0	0	0	Polyneuropathy in herpes zoster	Feb-09
68960	F374500	6	0	0	0	Polyneuropathy in hypoglycaemia	Feb-09
30537	F373.00	15	0	0	0	Polyneuropathy in malignant disease	Feb-09
100064	F374600	1	0	0	0	Polyneuropathy in mumps	Jul-10
47465	F371100	5	0	0	0	Polyneuropathy in polyarteritis nodosa	Feb-09
58758	F374800	25	0	1	0	Polyneuropathy in porphyria	Feb-09
62401	F371200	21	0	0	0	Polyneuropathy in rheumatoid arthritis	Feb-09
40751	F374900	16	0	0	0	Polyneuropathy in sarcoidosis	Feb-09
24355	F374200	9	0	0	0	Polyneuropathy in vitamin B deficiency	Feb-09
24226	F37z.11	219	0	9	0	Polyneuropathy unspecified	Feb-09
30600	PG54.00	168	0	8	0	Polyostotic fibrous dysplasia	Feb-09
93879	P41..11	10	0	0	0	Polyotia	Feb-09

For peer review only - http://bmjopen.bmj.com/site/about/guidelines.xhtml

38836	PF15.00	55	0	0	0	Polysyndactyly	Feb-09
68991	PF1z.11	1	0	0	0	Polysyndactyly	Feb-09
43743	C371z00	162	0	1	0	Porphyria NOS	Feb-09
47257	J615.11	14	0	0	0	Portal cirrhosis	Feb-09
55454	J615y00	18	0	0	0	Portal cirrhosis unspecified	Feb-09
40963	J61y300	35	0	0	0	Portal fibrosis without cirrhosis	Feb-09
5129	J623.00	4307	0	65	0	Portal hypertension	Feb-09
44881	C137100	36	0	2	0	Post-hypophysectomy hypopituitarism	Feb-09
25424	C1z1011	79	0	22	0	Precocious pubarche	Feb-09
3031	C1z1000	3154	0	645	0	Precocious puberty	Feb-09
15995	C1z1.11	558	0	86	1	Precocious puberty	Feb-09
70360	C152912	3	0	0	0	Precocious puberty with adrenal hyperplasia	Feb-09
22704	C1z1111	873	0	34	0	Precocious thelarche	Feb-09
50665	C391000	40	0	4	0	Predominantly T-cell immuno-deficiency NOS	Feb-09
106756	P711.13	1	0	0	0	Preductal aortic stenosis	Aug-13
72295	P711.00	2	0	0	0	Preductal coarctation of aorta	Feb-09
53970	C373700	26	0	0	0	Primary amyloidosis NEC	Feb-09
5638	J616000	6062	0	300	0	Primary biliary cirrhosis	Feb-09
29028	C151000	247	0	4	0	Primary hyperaldosteronism	Feb-09
18740	C120000	3636	0	136	2	Primary hyperparathyroidism	Feb-09
107704	G20..12	24	0	0	0	Primary hypertension	Feb-14
45133	E312.00	106	0	2	0	Profound mental retardation with IQ less than 20	Feb-09
107607	F591B00	50	0	0	0	Profound sensorineural hearing loss	Jan-14
52673	A413.00	66	0	5	0	Progressive multifocal leucoencephalopathy	Feb-09
49541	A413.11	26	0	1	0	Progressive multifocal leukoencephalopathy	Feb-09
37644	F132100	100	0	1	0	Progressive myoclonic epilepsy	Feb-09

For peer review only - http://bmjopen.bmj.com/site/about/guidelines.xhtml

44058	F427811	60	0	1	0	Progressive rod dystrophy	Feb-09
40553	F130400	72	0	2	0	Progressive supranuclear ophthalmoplegia	Feb-09
9385	F24y000	1339	0	59	0	Progressive supranuclear palsy	Feb-09
44141	N001000	47	0	1	0	Progressive systemic sclerosis	Feb-09
52426	C134000	54	0	5	0	Prolactin deficiency	Feb-09
26102	D30B.00	598	0	25	0	Protein C deficiency	Feb-09
8479	D309.00	1037	0	52	0	Protein S deficiency	Feb-09
22853	PG72.00	118	0	4	0	Prune belly syndrome	Feb-09
46749	C377111	12	0	1	0	Pseudo - Hurler's disease	Feb-09
43115	PG44600	34	0	0	0	Pseudoachondroplasia	Feb-09
60703	ZS42111	15	0	1	0	Pseudobulbar palsy type of dysarthria	Feb-09
2337	D41y200	228	0	14	7	Pseudocholinesterase deficiency	Feb-09
36351	PG42.17	4	0	0	0	Pseudochondroplasia	Feb-09
48532	PC7z100	7	0	0	0	Pseudohermaphrodite NOS	Feb-09
93135	C152814	2	0	0	0	"Pseudohermaphrodite, male with adrenocortical disorder"	Feb-09
26210	F391011	7	0	0	0	Pseudohypertrophic dystrophy	Feb-09
23540	C354300	199	0	6	0	Pseudohypoparathyroidism	Feb-09
49810	F280200	4	0	0	0	Pseudoporencephaly	Feb-09
65126	C371600	54	0	1	0	Pseudoporphyria	Feb-09
60587	C354311	9	0	2	0	Pseudopseudohypoparathyroidism	Feb-09
52610	F4E3411	50	0	3	0	Pseudoptosis	Feb-09
66964	F426500	8	0	0	0	Pseudoretinitis pigmentosa	Feb-09
65405	N237300	6	0	0	0	Pseudosarcomatous fibromatosis	Feb-09
49468	A57y400	21	0	1	0	Pseudoscarlatina	Feb-09
22632	F4J4600	697	0	3	0	Pseudostrabismus	Feb-09
68982	P603.11	2	0	0	0	Pseudotruncus arteriosus	Feb-09
46041	F282.11	43	0	2	0	Pseudotumour cerebri	Feb-09
24805	F4G1111	98	0	3	0	Pseudotumour of orbit	Feb-09
30272	M161200	41	0	0	0	Psoriasis circinata	Feb-09

For peer review only - http://bmjopen.bmj.com/site/about/guidelines.xhtml

42008	M161300	82	0	2	0	Psoriasis diffusa	Feb-09
21633	M161500	19	0	0	0	Psoriasis geographica	Feb-09
65839	M161700	5	0	0	0	Psoriasis gyrata	Feb-09
48257	M161800	11	0	0	0	Psoriasis inveterata	Feb-09
172	M161z00	348556	0	12706	0	Psoriasis NOS	Feb-09
60169	M161900	2	0	0	0	Psoriasis ostracea	Feb-09
24136	M161C00	63	0	2	0	Psoriasis punctata	Feb-09
26368	M160000	191	0	4	0	Psoriasis spondylitica	Feb-09
162	M161000	386002	0	22325	19	Psoriasis unspecified	Feb-09
30210	M161F00	904	0	15	0	Psoriasis vulgaris	Feb-09
96880	M160.11	6793	0	225	0	Psoriatic arthritis	May-09
476	M160.00	37950	0	2913	1	Psoriatic arthropathy	Feb-09
12500	M160z00	1575	0	107	0	Psoriatic arthropathy NOS	Feb-09
23634	F254100	97	0	6	0	Psychomotor epilepsy	Feb-09
37948	1P01.00	928	0	7	0	Psychomotor retardation	Feb-09
36203	F254200	8	0	1	0	Psychosensory epilepsy	Feb-09
43225	E14..00	13	0	1	0	Psychoses with origin in childhood	Feb-09
96225	P736.00	4	0	0	0	Pulmonary arterio-venous aneurysm	Apr-09
47853	P736.11	16	0	0	0	Pulmonary arterio-venous fistula	Feb-09
37807	G42y000	8	0	1	0	Pulmonary arteritis	Feb-09
67928	P731.00	2	0	0	0	Pulmonary artery agenesis	Feb-09
65029	P737.00	18	0	0	0	Pulmonary artery aneurysm	Feb-09
42127	P73z.00	31	0	0	0	Pulmonary artery anomaly NOS	Feb-09
63187	P730.00	9	0	0	0	"Pulmonary artery anomaly, unspecified"	Feb-09
7180	G4...00	230	0	10	0	Pulmonary circulation diseases	Feb-09
30214	H54..00	280	0	17	0	Pulmonary congestion and hypostasis	Feb-09
61229	H54z.00	2	0	0	0	Pulmonary congestion and hypostasis NOS	Feb-09
103472	H563200	4735	0	172	0	Pulmonary fibrosis	Dec-11

For peer review only - http://bmjopen.bmj.com/site/about/guidelines.xhtml

46736	G543200	136	0	3	0	"Pulmonary incompetence, cause unspecified"	Feb-09
23608	G543000	139	0	3	0	"Pulmonary incompetence, non-rheumatic"	Feb-09
9401	P6y2.00	1595	0	74	0	Pulmonary infundibular stenosis	Feb-09
93880	H585000	1	0	0	0	Pulmonary insufficiency following shock	Feb-09
72221	H585100	1	0	0	0	Pulmonary insufficiency following surgery	Feb-09
96754	H585200	3	0	0	0	Pulmonary insufficiency following trauma	Apr-09
38299	G543213	17	0	2	0	"Pulmonary insufficiency, cause unspecified"	Feb-09
15496	G543012	609	0	8	0	"Pulmonary regurgitation, non-rheumatic"	Feb-09
3859	H57y200	2945	0	121	0	Pulmonary sarcoidosis	Feb-09
2669	G543300	3149	0	74	1	"Pulmonary stenosis, cause unspecified"	Feb-09
61878	G543311	107	0	1	0	"Pulmonary stenosis, cause unspecified"	Feb-09
14723	G543100	1408	0	68	0	"Pulmonary stenosis, non-rheumatic"	Feb-09
39992	P60..00	201	0	2	0	Pulmonary valve anomalies	Feb-09
69940	P600.00	22	0	0	0	"Pulmonary valve anomaly, unspecified"	Feb-09
12312	G543.00	632	0	14	0	Pulmonary valve disorders	Feb-09
34932	G543400	225	0	2	0	Pulmonary valve stenosis with insufficiency	Feb-09
53944	K10y.00	128	0	0	0	Pyelonephritis and pyonephrosis unspecified	Feb-09
95710	K10y300	6	0	0	0	Pyelonephritis in diseases EC	Feb-09

For peer review only - http://bmjopen.bmj.com/site/about/guidelines.xhtml

60856	K0A2700	4	0	0	0	Recur+persist haematuria difus crescentic glomerulonephritis	Feb-09
61317	K0A2200	4	0	0	0	Recur+persist haematuria difus membranous glomerulonephritis	Feb-09
68364	K0A2100	2	0	0	0	"Recur+persist haematuria, focal+segmental glomerular lesions"	Feb-09
49642	K0A2300	11	0	0	0	Recur+persist haemuria df mesangial prolif glomerulnephritis	Feb-09
60484	K0A2500	3	0	0	0	Recur+persist hmuria df mesangiocapilary glomerulonephritis	Feb-09
53480	J614300	25	0	0	0	Recurrent hepatitis	Feb-09
25563	E113z00	1910	0	10	0	Recurrent major depressive episode NOS	Feb-09
55384	E113600	518	0	1	0	"Recurrent major depressive episodes, in full remission"	Feb-09
29342	E113100	1615	0	25	0	"Recurrent major depressive episodes, mild"	Feb-09
25697	E113300	1195	0	32	0	"Recurrent major depressive episodes, severe, no psychosis"	Feb-09
24171	E113400	493	0	9	0	"Recurrent major depressive episodes, severe, with psychosis"	Feb-09
35671	E113000	506	0	12	0	"Recurrent major depressive episodes, unspecified"	Feb-09
56273	E113500	226	0	9	0	"Recurrent major depressive episodes,partial/unspec remission"	Feb-09
46415	E111z00	94	0	1	0	Recurrent manic episode NOS	Feb-09
26227	E111.00	215	0	6	0	Recurrent manic episodes	Feb-09
37178	E111600	125	0	1	0	"Recurrent manic episodes, in full remission"	Feb-09
46425	E111100	20	0	0	0	"Recurrent manic episodes, mild"	Feb-09

For peer review only - http://bmjopen.bmj.com/site/about/guidelines.xhtml

27739	E111200	24	0	0	0	"Recurrent manic episodes, moderate"	Feb-09
58863	E111500	2	0	0	0	"Recurrent manic episodes, partial or unspecified remission"	Feb-09
65811	E111300	20	0	0	0	"Recurrent manic episodes, severe without mention psychosis"	Feb-09
32295	E111400	25	0	0	0	"Recurrent manic episodes, severe, with psychosis"	Feb-09
22003	G54z013	93	0	0	0	Regurgitation of unspecified heart valve	Feb-09
8103	A993.00	2493	0	96	0	Reiter's disease / syndrome	Feb-09
2194	A993.11	1829	0	153	7	Reiter's syndrome	Feb-09
45904	K0B2.00	2	0	0	0	Ren tub-interst disordr/blood dis+disordr inv immune mech	Feb-09
67261	K0B4.00	3	0	0	0	Ren tub-interstitl disordr/systemc connectv tiss disorder	Feb-09
48475	K08yz11	2	0	0	0	Renal acidaemia	Feb-09
30097	PD0..00	245	0	9	0	Renal agenesis and dysgenesis	Feb-09
29659	PD0z.00	84	0	6	0	Renal agenesis or dysgenesis NOS	Feb-09
27474	PD00.00	158	0	3	0	"Renal agenesis, unspecified"	Feb-09
55985	PD00z00	17	0	0	0	"Renal agenesis, unspecified NOS"	Feb-09
107765	K0J..00	4	0	0	0	Renal disorders in systemic disease	Mar-14
32963	PD30.12	17	0	0	0	Renal duplication NEC	Feb-09
34648	K080100	4	0	0	0	Renal dwarfism	Feb-09
105657	PD04200	6	0	2	0	Renal dysplasia and retinal aplasia	Jan-13
350	K06..00	38962	0	4706	59	Renal failure unspecified	Feb-09
106860	C353600	4	0	0	0	Renal failure-associated hyperphosphataemia	Aug-13
22876	K071.00	50	0	1	0	Renal fibrosis	Feb-09
41013	K08y300	15	0	0	0	Renal function impairment with growth failure	Feb-09
29638	K080.00	207	0	3	0	Renal osteodystrophy	Feb-09

For peer review only - http://bmjopen.bmj.com/site/about/guidelines.xhtml

34637	K080z00	49	0	0	0	Renal osteodystrophy NOS	Feb-09
44894	D410400	26	0	0	0	Renal polycythaemia	Feb-09
45876	F421200	14	0	2	0	Renal retinopathy	Feb-09
66062	K080300	15	0	0	0	Renal rickets	Feb-09
4480	K07z.00	46	0	2	1	Renal sclerosis NOS	Feb-09
26220	K07..00	59	0	4	0	Renal sclerosis unspecified	Feb-09
110983	K08y412	1	0	0	0	Renal tubular acidaemia	Jul-16
5072	K08y400	651	0	18	0	Renal tubular acidosis	Feb-09
41148	K0B4000	10	0	1	0	Renal tubulo-interstitial disorder in SLE	Feb-09
64622	K0B1.00	4	0	0	0	Renal tubulo-interstitial disorder/ neoplastic diseases	Feb-09
45523	K0B..00	26	0	1	0	Renal tubulo-interstitial disorders in diseases EC	Feb-09
48057	K0B5.00	21	0	0	0	Renal tubulo-interstitial disordrs in transplant rejectn	Feb-09
35157	G770.11	150	0	15	0	Rendu - Osler - Weber disease	Feb-09
25249	H59..00	2542	0	25	0	Respiratory failure	Feb-09
39079	Q319.00	49	0	0	0	Respiratory failure of newborn	Feb-09
46397	P8z..00	33	0	0	0	Respiratory system anomaly NOS	Feb-09
46398	P8...00	38	0	4	0	Respiratory system congenital anomalies	Feb-09
2745	F427600	6052	0	711	2	Retinitis pigmentosa	Feb-09
39637	F421A11	76	0	7	0	Retinitis proliferans	Feb-09
23896	J041.12	144	0	1	0	Retrognathism	Feb-09
26364	J041z00	16	0	0	0	Retrognathism NOS	Feb-09
32211	G121.00	135	0	8	0	Rheumatic aortic insufficiency	Feb-09
9391	G120.00	703	0	26	0	Rheumatic aortic stenosis	Feb-09
63960	G122.00	31	0	2	0	Rheumatic aortic stenosis with insufficiency	Feb-09
50809	G12z.00	54	0	1	0	Rheumatic aortic valve disease NOS	Feb-09
72936	G02z.00	12	0	0	0	Rheumatic chorea NOS	Feb-09

For peer review only - http://bmjopen.bmj.com/site/about/guidelines.xhtml

69995	G020.00	10	0	0	0	Rheumatic chorea with heart involvement	Feb-09
63252	G021.00	9	0	0	0	Rheumatic chorea without mention of heart involvement	Feb-09
100820	66HC.00	9806	0	5	0	Rheumatic disorder annual review invitation	Nov-10
44756	G01..00	119	0	0	0	Rheumatic fever with heart involvement	Feb-09
48189	G00..00	153	0	1	0	Rheumatic fever without heart involvement	Feb-09
107462	GA...00	47	0	2	0	Rheumatic heart disease	Dec-13
53878	G1yz000	101	0	0	0	Rheumatic heart disease unspecified	Feb-09
22262	G1yz100	149	0	11	0	Rheumatic left ventricular failure	Feb-09
51879	G111.00	208	0	19	0	Rheumatic mitral insufficiency	Feb-09
32435	G110.11	223	0	5	0	Rheumatic mitral stenosis	Feb-09
62404	G1y0.00	8	0	0	0	Rheumatic myocarditis	Feb-09
64799	H571.00	8	0	0	0	Rheumatic pneumonia	Feb-09
54088	G141100	2	0	0	0	Rheumatic pulmonary insufficiency	Feb-09
62207	G141000	14	0	0	0	Rheumatic pulmonary stenosis	Feb-09
105626	G141200	1	0	0	0	Rheumatic pulmonary stenosis and insufficiency	Jan-13
44167	G141.00	8	0	0	0	Rheumatic pulmonary valve disease	Feb-09
36768	G141z00	2	0	0	0	Rheumatic pulmonary valve disease NOS	Feb-09
62875	N135100	8	0	0	0	Rheumatic torticollis	Feb-09
60266	G140100	24	0	0	0	Rheumatic tricuspid insufficiency	Feb-09
31505	G140000	5	0	0	0	Rheumatic tricuspid stenosis	Feb-09
62186	G14021Y	1	0	0	0	Rheumatic tricuspid stenosis and incompetence	Feb-09
93114	G140200	1	0	0	0	Rheumatic tricuspid stenosis and insufficiency	Feb-09

For peer review only - http://bmjopen.bmj.com/site/about/guidelines.xhtml

93113	G14021X	7	0	0	0	Rheumatic tricuspid stenosis and regurgitation	Feb-09
72613	G140z00	4	0	0	0	Rheumatic tricuspid valve disease NOS	Feb-09
59275	G14z.11	1	0	0	0	"Rheumatic valvulitis, chronic NOS"	Feb-09
67955	A985z11	1	0	0	0	Rheumatism - gonococcal	Feb-09
33474	N240.00	1221	0	1	0	Rheumatism and fibrositis unspecified	Feb-09
35937	N240z00	1287	0	0	0	Rheumatism or fibrositis NOS	Feb-09
844	N040.00	262585	0	21625	2747	Rheumatoid arthritis	Feb-09
31054	N040S00	380	0	22	0	Rheumatoid arthritis - multiple joint	Feb-09
27603	N04..00	1821	0	30	0	Rheumatoid arthritis and other inflammatory polyarthropathy	Feb-09
105507	66HB000	78562	0	249	0	Rheumatoid arthritis annual review	Dec-12
107340	9mM..00	486	0	1	0	Rheumatoid arthritis monitoring invitation	Dec-13
107435	9mM0.00	8104	0	1	0	Rheumatoid arthritis monitoring invitation first letter	Dec-13
107575	9mM1.00	3203	0	1	0	Rheumatoid arthritis monitoring invitation second letter	Jan-14
107676	9mM2.00	1666	0	0	0	Rheumatoid arthritis monitoring invitation third letter	Feb-14
107797	9mM4.00	482	0	0	0	Rheumatoid arthritis monitoring telephone invitation	Mar-14
107606	9mM3.00	103	0	0	0	Rheumatoid arthritis monitoring verbal invitation	Jan-14
51238	N040K00	16	0	0	0	Rheumatoid arthritis of 1st MTP joint	Feb-09
100914	N040400	3	0	0	0	Rheumatoid arthritis of acromioclavicular joint	Nov-10
51239	N040F00	68	0	1	0	Rheumatoid arthritis of ankle	Feb-09

For peer review only - http://bmjopen.bmj.com/site/about/guidelines.xhtml

44743	N040000	21	0	0	0	Rheumatoid arthritis of cervical spine	Feb-09
63198	N040A00	18	0	0	0	Rheumatoid arthritis of DIP joint of finger	Feb-09
63365	N040600	4	0	0	0	Rheumatoid arthritis of distal radio-ulnar joint	Feb-09
59738	N040500	34	0	0	0	Rheumatoid arthritis of elbow	Feb-09
49067	N040B00	34	0	1	0	Rheumatoid arthritis of hip	Feb-09
107112	N040M00	6	0	0	0	Rheumatoid arthritis of IP joint of toe	Oct-13
50863	N040D00	151	0	3	0	Rheumatoid arthritis of knee	Feb-09
99414	N040L00	4	0	0	0	Rheumatoid arthritis of lesser MTP joint	May-10
42299	N040800	75	0	3	0	Rheumatoid arthritis of MCP joint	Feb-09
71784	N040J00	6	0	0	0	Rheumatoid arthritis of other tarsal joint	Feb-09
41941	N040900	47	0	1	0	Rheumatoid arthritis of PIP joint of finger	Feb-09
100776	N040C00	4	0	0	0	Rheumatoid arthritis of sacro-iliac joint	Nov-10
107963	N040300	1	0	0	0	Rheumatoid arthritis of sternoclavicular joint	Apr-14
73619	N040G00	9	0	0	0	Rheumatoid arthritis of subtalar joint	Feb-09
70658	N040H00	7	0	0	0	Rheumatoid arthritis of talonavicular joint	Feb-09
107791	N040E00	1	0	0	0	Rheumatoid arthritis of tibio-fibular joint	Mar-14
48832	N040700	113	0	0	0	Rheumatoid arthritis of wrist	Feb-09
37431	N042z00	39	0	1	0	Rheumatoid arthropathy + visceral/systemic involvement NOS	Feb-09
43816	G5yA.00	26	0	0	0	Rheumatoid carditis	Feb-09

For peer review only - http://bmjopen.bmj.com/site/about/guidelines.xhtml

31724	N04y000	109	0	11	0	Rheumatoid lung	Feb-09
46436	N042100	33	0	1	0	Rheumatoid lung disease	Feb-09
49787	G5y8.00	6	0	0	0	Rheumatoid myocarditis	Feb-09
30548	N040N00	225	0	3	0	Rheumatoid vasculitis	Feb-09
33375	C260.11	13	0	3	2	Riboflavin deficiency	Feb-09
5074	C28..12	2187	0	148	0	Rickets	Feb-09
51768	P342200	41	0	2	0	Rieger's anomaly	Feb-09
57069	G565200	7	0	1	0	Right BBB with left anterior fascicular block	Feb-09
98675	G565100	2	0	0	0	Right BBB with left posterior fascicular block	Mar-10
10079	G580.12	1165	0	40	0	Right heart failure	Feb-09
3293	F223.00	2081	0	124	1	Right hemiplegia	Feb-09
21851	P603.00	52	0	0	0	Right hypoplastic heart syndrome	Feb-09
98751	G5y3600	98	0	1	0	Right ventricular dilatation	Mar-10
104275	G584.00	169	0	1	0	Right ventricular failure	Jun-12
104266	P6y3100	36	0	0	0	Right ventricular outflow tract obstruction	Jun-12
108180	G5yyE00	70	0	0	0	Right ventricular systolic dysfunction	Jun-14
31616	P2x5.00	9	0	0	0	Riley - Day syndrome	Feb-09
22691	G56y200	51	0	0	0	Romano - Ward syndrome	Feb-09
40502	PH33300	46	0	1	0	Rothmund-Thomson syndrome	Feb-09
46507	C374300	65	0	4	0	Rotor syndrome	Feb-09
109176	F143.11	1	0	0	0	Roussy-Levy syndrome	Jan-15
104100	PKy7311	15	0	0	0	Rubinstein-Taybi syndrome	May-12
32868	PKy6200	316	0	15	0	Russell - Silver syndrome	Feb-09
72928	P103400	1	0	0	0	Sacral spina bifida with hydrocephalus - closed	Feb-09
70923	P118400	9	0	0	0	Sacral spina bifida without hydrocephalus - closed	Feb-09

For peer review only - http://bmjopen.bmj.com/site/about/guidelines.xhtml

71525	P117400	3	0	0	0	Sacral spina bifida without hydrocephalus - open	Feb-09
57986	PF55300	91	0	2	0	Saethre-Chotzen syndrome	Feb-09
109634	E121.11	1	0	0	0	Sander's disease	May-15
44111	J34y.11	52	0	2	0	Sandifer's syndrome	Feb-09
111807	F142100	2	0	0	0	Sanger-Brown cerebellar ataxia	May-17
40613	AD55.00	59	0	2	0	Sarcoid arthropathy	Feb-09
47037	G558300	61	0	2	0	Sarcoid heart disease	Feb-09
34437	G5y7.00	13	0	1	0	Sarcoid myocarditis	Feb-09
3865	AD5..00	29294	0	1573	7	Sarcoidosis	Feb-09
72595	AD54.00	32	0	0	0	Sarcoidosis of inferior turbinates	Feb-09
33980	AD50.00	389	0	6	0	Sarcoidosis of lung	Feb-09
58841	AD52.00	43	0	3	0	Sarcoidosis of lung with sarcoidosis of lymph nodes	Feb-09
49075	AD51.00	81	0	2	0	Sarcoidosis of lymph nodes	Feb-09
27769	AD53.00	243	0	12	0	Sarcoidosis of skin	Feb-09
111668	H32yz11	1	0	0	0	Sawyer - Jones syndrome	Feb-17
69375	C375113	5	0	0	0	Scheie's syndrome	Feb-09
1721	N320100	7224	0	306	1	Scheuermann's disease	Feb-09
56438	E107500	211	0	0	0	Schizo-affective schizophrenia in remission	Feb-09
67943	13Y2.00	11	0	0	0	Schizophrenia association member	Feb-09
58687	E100500	1296	0	0	0	Schizophrenia in remission	Feb-09
8407	E10z.00	11518	0	194	0	Schizophrenia NOS	Feb-09
73295	E100.11	4	0	0	0	Schizophrenia simplex	Feb-09
31950	13L3.12	48	0	0	0	Schizophrenic child	Feb-09
854	E10..00	59295	0	3962	7	Schizophrenic disorders	Feb-09
56692	PG45.12	7	0	0	0	Schmid's metaphyseal dysostosis	Feb-09
48130	C181.12	32	0	1	0	Schmidt's syndrome	Feb-09
36606	P74z600	68	0	3	0	Scimitar syndrome	Feb-09
5318	F4K0.12	7606	0	578	0	Scleritis	Feb-09
15996	F4K0.00	3226	0	76	0	Scleritis and episcleritis	Feb-09

For peer review only - http://bmjopen.bmj.com/site/about/guidelines.xhtml

44859	F4K0z00	557	0	2	0	Scleritis or episcleritis NOS	Feb-09
37538	M210500	123	0	4	0	Sclerodactyly	Feb-09
3670	N001.00	6495	0	419	0	Scleroderma	Feb-09
37665	F4K0500	72	0	0	0	Sclerokeratitis	Feb-09
44791	J661900	142	0	1	0	Sclerosing cholangitis unspecified	Feb-09
40041	N374300	1006	0	40	0	Scoliosis associated with other condition	Feb-09
43192	N374D00	7	0	0	0	Scoliosis in connective tissue anomalies	Feb-09
67445	N374C00	19	0	0	0	Scoliosis in neurofibromatosis	Feb-09
64218	N374A00	29	0	1	0	Scoliosis in skeletal dysplasia	Feb-09
53972	N373900	50	0	0	0	Scoliosis secondary to other treatment	Feb-09
34719	PKy6400	10	0	0	0	Seckel syndrome	Feb-09
22487	C10N.00	146	0	5	0	Secondary diabetes mellitus	Feb-09
94383	C10N000	8	0	0	0	Secondary diabetes mellitus without complication	Feb-09
37119	C151100	24	0	1	0	Secondary hyperaldosteronism	Feb-09
70714	C354111	73	0	0	0	Secondary hypercalcaemia	Feb-09
7329	G24..00	2762	0	20	0	Secondary hypertension	Feb-09
42229	G24zz00	162	0	0	0	Secondary hypertension NOS	Feb-09
51697	C10G.00	339	0	4	0	Secondary pancreatic diabetes mellitus	Feb-09
96506	C10G000	2	0	0	0	Secondary pancreatic diabetes mellitus without complication	Apr-09
34065	G41y000	1476	0	10	0	Secondary pulmonary hypertension	Feb-09
65502	D201z14	8	0	0	0	Secondary red cell aplasia NEC	Feb-09
31774	D201z13	7	0	0	0	Secondary red cell hypoplasia NEC	Feb-09
35667	J661800	50	0	2	0	Secondary sclerosing cholangitis	Feb-09
31550	D210300	7	0	0	0	Secondary sideroblastic anaemia due to disease	Feb-09

For peer review only - http://bmjopen.bmj.com/site/about/guidelines.xhtml

47225	D210400	5	0	0	0	Secondary sideroblastic anaemia due to drugs and toxins	Feb-09
97143	F13C.00	13	0	0	0	Segawa syndrome	Aug-09
13304	Q480.12	1116	0	20	0	Seizures in newborn	Feb-09
8548	C390100	796	0	35	3	Selective IgA immunodeficiency	Feb-09
18700	C390300	122	0	3	3	Selective IgG immunodeficiency	Feb-09
18701	C390200	41	0	5	0	Selective IgM immunodeficiency	Feb-09
536	F591.00	65546	0	1344	0	Sensorineural hearing loss	Feb-09
10112	F591600	8679	0	79	0	"Sensorineural hearing loss, bilateral"	Feb-09
29191	F591700	281	0	38	0	"Sensorineurl hear loss,unilat unrestrict hear/contralat side"	Feb-09
3366	663V300	2734	0	66	0	Severe asthma	Feb-09
233	H33z011	25653	0	4132	4	Severe asthma attack	Feb-09
110345	38B8.00	37	0	0	0	Severe asthma exacerbation risk assessment	Dec-15
15082	Q215.00	1394	0	28	5	Severe birth asphyxia - apgar score less than 4 at 1 minute	Feb-09
98734	F286200	12	0	0	0	Severe chronic fatigue syndrome	Mar-10
107402	28E2.00	149	0	1	0	Severe cognitive impairment	Dec-13
108409	F25G.00	5	0	0	0	Severe myoclonic epilepsy in infancy	Jul-14
107610	F591E00	47	0	1	0	Severe sensorineural hearing loss	Jan-14
4376	PJX..00	25	0	0	0	"Sex chromosome abnormality, male phenotype, unspecified"	Feb-09
34667	PJyz.00	65	0	9	2	Sex chromosome anomaly NOS	Feb-09
55119	PJy1.00	17	0	0	0	Sex chromosome mosaicism	Feb-09
65983	PJy1z00	1	0	0	0	Sex chromosome mosaicism NOS	Feb-09
35014	B622.00	77	0	7	0	Sezary's disease	Feb-09
100532	B622z00	3	0	0	0	Sezary's disease NOS	Aug-10
12841	PJ3y000	42	0	0	0	Shprintzen syndrome	Feb-09
62041	PJ53500	21	0	2	0	Shwachman-Diamond syndrome	Feb-09

For peer review only

35839	F130500	90	0	17	0	Shy-Drager syndrome	Feb-09
2360	N002.00	12690	0	576	1	Sicca (Sjogren's) syndrome	Feb-09
104288	1X0..00	6	0	0	0	Sick Cell Thalas Scr Prog fam orig African or African-Carib	Jun-12
103055	1X1..00	23	0	0	0	Sick Cell Thalas Scr Prog fam orig South Asia (Asian)	Oct-11
107027	K0G..00	5	0	0	0	Sickle cell nephropathy	Sep-13
23519	D106.00	4532	0	489	166	Sickle-cell anaemia	Feb-09
57397	D106z00	75	0	2	0	Sickle-cell anaemia NOS	Feb-09
69964	D106000	14	0	1	0	Sickle-cell anaemia of unspecified type	Feb-09
31370	D106200	1244	0	26	0	Sickle-cell anaemia with crisis	Feb-09
31306	D106300	109	0	8	1	Sickle-cell anaemia with haemoglobin C disease	Feb-09
8119	D106400	55	0	1	1	Sickle-cell anaemia with haemoglobin D disease	Feb-09
93872	D106500	20	0	0	2	Sickle-cell anaemia with haemoglobin E disease	Feb-09
32937	D106100	19	0	1	0	Sickle-cell anaemia with no crisis	Feb-09
31075	D104211	360	0	125	100	Sickle-cell thalassaemia	Feb-09
3616	D105.00	13001	0	1063	741	Sickle-cell trait	Feb-09
99476	PKy5H00	8	0	0	0	Simpson-Golabi-Behmel syndrome	May-10
107846	P240000	1	0	0	0	Single congenital cerebral cyst	Mar-14
62163	P6y4100	26	0	0	0	Single coronary artery	Feb-09
18437	G566000	157	0	7	0	Sinoatrial block	Feb-09
426	G577.00	2593	0	109	1	Sinus arrhythmia	Feb-09
31717	P20..15	22	0	2	0	Sinus pericranii	Feb-09
43560	PK31.00	21	0	1	0	Situs inversus abdominalis	Feb-09
23513	PK3z.00	39	0	8	0	Situs inversus NOS	Feb-09
63861	PK32.00	2	0	0	0	Situs inversus thoracis	Feb-09
110797	PK34.00	1	0	0	0	Situs inversus with levocardia	May-16
72246	PK30.00	6	0	0	0	"Situs inversus, unspecified"	Feb-09

For peer review only - http://bmjopen.bmj.com/site/about/guidelines.xhtml

11632	PH12.11	588	0	19	0	Sjogren - Larsson syndrome	Feb-09
7603	Fy03.00	16408	0	2567	0	Sleep apnoea	Feb-09
23779	H5B..00	2863	0	276	0	Sleep apnoea	Feb-09
60083	PB1..00	9	0	0	0	Small intestine atresia and stenosis	Feb-09
70579	PB10z00	2	0	0	0	Small intestine atresia NOS	Feb-09
25042	PB1z.00	10	0	0	0	Small intestine atresia or stenosis NOS	Feb-09
36871	PJ33300	87	0	4	0	Smith-Magenis syndrome	Feb-09
36314	C1zy211	222	0	13	0	Sotos syndrome	Feb-09
25570	F23y200	221	0	2	0	Spastic cerebral palsy	Feb-09
104775	F230111	49	0	1	0	Spastic diplegic cerebral palsy	Aug-12
59254	ZS42100	32	0	1	0	Spastic dysarthria	Feb-09
105133	F2B1.00	21	0	0	0	Spastic hemiplegic cerebral palsy	Nov-12
9375	F241100	1317	0	48	1	Spastic paraplegia	Feb-09
104580	F2B0.00	101	0	1	0	Spastic quadriplegic cerebral palsy	Jul-12
25990	ZPA2100	992	0	8	0	Special educational needs	Feb-09
22896	ZS...00	1425	0	12	0	Speech and language disorder	Feb-09
43892	ZS5..00	47	0	1	0	Speech and language dyspraxias	Feb-09
28845	ZS6..00	94	0	13	0	Speech and phonology impairments	Feb-09
3417	E2F3.12	6785	0	1109	0	Speech development disorder	Feb-09
110439	1B96.00	40	0	5	0	Speech impairment	Jan-16
3567	E2F3.00	3146	0	44	0	Speech or language developmental disorder	Feb-09
1277	E2F3z00	3822	0	245	1	Speech or language developmental disorder NOS	Feb-09
32923	ZT23.00	548	0	25	0	Speech problem	Feb-09
33403	1B9Z.00	1043	0	8	0	Speech problem NOS	Feb-09
3336	PG17.00	6133	0	142	2	Spina bifida occulta	Feb-09
27436	PD1..14	43	0	1	0	Sponge kidney	Feb-09
4892	H33z000	3720	0	442	0	Status asthmaticus NOS	Feb-09
4093	F253.11	2882	0	577	0	Status epilepticus	Feb-09
22685	F26y200	250	0	1	0	Status migrainosus	Feb-09

For peer review only - http://bmjopen.bmj.com/site/about/guidelines.xhtml

93910	F24y012	1	0	0	0	Steele - Richardson Oszewski syndrome	Feb-09
49034	F24y011	14	0	0	0	Steele Richardson Olszewsk syn	Feb-09
7037	F24y200	94	0	7	0	Steele-Richardson-Olszewski syndrome	Feb-09
16103	C164.12	741	0	51	0	Stein - Leventhal syndrome	Feb-09
50109	PB2..12	5	0	0	0	Stenosis large intestine	Feb-09
16731	P713.11	18	0	0	0	Stenosis of aortic arch	Feb-09
46120	P230.12	31	0	0	0	Stenosis of aqueduct of Sylvius	Feb-09
51675	P74z200	15	0	0	0	Stenosis of inferior vena cava	Feb-09
35852	J50z200	104	0	0	0	Stenosis of intestine NOS	Feb-09
2670	P735.00	785	0	13	0	Stenosis of pulmonary artery	Feb-09
29179	J572.00	135	0	7	0	Stenosis of rectum and anus	Feb-09
53581	J572z00	19	0	2	0	Stenosis of rectum and anus NOS	Feb-09
41197	P74z300	25	0	0	0	Stenosis of superior vena cava	Feb-09
29172	H5y1200	287	0	9	0	Stenosis of trachea	Feb-09
98579	F4J7011	2	0	0	0	Stilling-Turck-Duane syndrome	Feb-10
3769	G56z000	1072	0	156	0	Stokes-Adams syndrome	Feb-09
4749	PK61.00	506	0	20	0	Sturge-Weber syndrome	Feb-09
16539	P6y0.00	411	0	14	0	Subaortic stenosis	Feb-09
73431	J106300	1	0	0	0	Subdiaphragmatic oesophageal diverticulum	Feb-09
105037	P741000	1	0	0	0	Subdiaphragmatic total anomalous pulmonary venous return	Oct-12
36042	C313200	41	0	0	0	Sucrose-isomaltose intolerance	Feb-09
4098	N337100	1745	0	141	0	Sudek's atrophy	Feb-09
111382	D417.12	2	0	0	0	Sulphaemoglobinaemia	Oct-16
36434	J421000	94	0	4	0	Superior mesenteric artery syndrome	Feb-09
8412	G8y2200	219	0	23	0	Superior vena cava syndrome	Feb-09
64216	ZS42113	7	0	0	0	Suprabulbar palsy type of dysarthria	Feb-09

For peer review only - http://bmjopen.bmj.com/site/about/guidelines.xhtml

103039	P741100	1	0	0	0	Supradiaphragmatic total anomalous pulmonary venous return	Oct-11
53964	P722400	43	0	0	0	Supra-valvular aortic stenosis	Feb-09
1536	G57y900	51876	0	4010	0	Supraventricular tachycardia NOS	Feb-09
43838	PF1z.00	265	0	1	0	Syndactyly NOS	Feb-09
41117	PF11.00	33	0	1	0	Syndactyly of fingers without bone fusion	Feb-09
63079	PF10.00	33	0	0	0	"Syndactyly of multiple digits, unspecified"	Feb-09
67014	PF14.00	15	0	1	0	Syndactyly of toes with bone fusion	Feb-09
108734	PF14.13	2	0	0	0	Syndactyly of toes with bone fusion	Oct-14
56836	PF13.00	77	0	3	0	Syndactyly of toes without bone fusion	Feb-09
47358	F160100	23	0	2	0	Syringobulbia	Feb-09
69740	F160.00	17	0	0	0	Syringomyelia and syringobulbia	Feb-09
96785	F160z00	4	0	0	0	Syringomyelia or syringobulbia NOS	Apr-09
43593	P11y.11	21	0	2	0	Syringomyelocele	Feb-09
7871	N000.00	19120	0	1358	47	Systemic lupus erythematosus	Feb-09
45726	ZRq9.00	11	0	1	0	Systemic lupus erythematosus disease activity index	Feb-09
42719	N000z00	347	0	4	0	Systemic lupus erythematosus NOS	Feb-09
29519	N000300	123	0	11	2	Systemic lupus erythematosus with organ or sys involv	Feb-09
28417	N001.12	1671	0	49	0	Systemic sclerosis	Feb-09
110174	N001200	1	0	0	0	Systemic sclerosis induced by drugs and chemicals	Oct-15
37640	G757.00	234	0	4	0	Takayasu's disease	Feb-09
68533	D313211	21	0	1	0	TAR syndrome	Feb-09
40025	P511300	12	0	0	0	Taussig-Bing syndrome	Feb-09
21169	F101300	266	0	135	130	Tay-Sach's disease	Feb-09
3175	F254000	13930	0	664	2	Temporal lobe epilepsy	Feb-09

For peer review only - http://bmjopen.bmj.com/site/about/guidelines.xhtml

36325	B470300	78	0	1	0	Teratoma of undescended testis	Feb-09
15118	B7B..11	312	0	24	0	"Teratoma, benign"	Feb-09
48765	PCy4.12	23	0	0	0	"Testicular agenesis, bilateral"	Feb-09
38998	PCy5.12	59	0	1	0	"Testicular agenesis, unilateral"	Feb-09
63528	C172000	14	0	1	0	Testicular hypofunc- defect adrenocortical hormone synthesis	Feb-09
9577	C172.12	6404	0	377	0	Testicular hypogonadism	Feb-09
4864	P52..00	2444	0	79	1	Tetralogy of Fallot	Feb-09
63046	P52z.00	42	0	0	0	Tetralogy of Fallot NOS	Feb-09
38967	P520.00	34	0	2	0	"Tetralogy of Fallot, unspecified"	Feb-09
1171	D104.00	6076	0	275	84	Thalassaemia	Feb-09
1174	D104100	2813	0	184	105	Thalassaemia minor NEC	Feb-09
4666	D104z00	191	0	0	0	Thalassaemia NOS	Feb-09
54429	D104200	42	0	4	3	Thalassaemia with haemoglobin S disease	Feb-09
56900	PG44200	5	0	0	0	Thanatophoric dwarfism	Feb-09
67262	PG44211	2	0	0	0	Thanatophoric dysplasia	Feb-09
23484	F392111	51	0	6	0	Thomsen's disease	Feb-09
57510	PH33312	7	0	0	0	Thomson's disease	Feb-09
62286	SJ21.00	7	0	0	0	Thoracic cord injury without spinal bone injury	Feb-09
102775	SJ21z00	1	0	0	0	"Thoracic cord injury without spinal bone injury, NOS"	Aug-11
51550	N129200	47	0	2	0	Thoracic disc disorder with myelopathy	Feb-09
23390	N121.00	575	0	15	0	Thoracic disc displacement without myelopathy	Feb-09
51691	N12B100	39	0	0	0	Thoracic disc prolapse with myelopathy	Feb-09
40333	N12C100	65	0	2	0	Thoracic disc prolapse with radiculopathy	Feb-09
103025	C14z.00	3	0	1	0	Thymus disease NOS	Sep-11

For peer review only - http://bmjopen.bmj.com/site/about/guidelines.xhtml

69113	C06y100	6	0	0	0	Thyroid atrophy	Feb-09
14704	C04..12	76375	0	3117	110	Thyroid deficiency	Feb-09
24681	66B8.00	3932	0	5	0	Thyroid dis.treatment changed	Feb-09
38292	66B9.00	986	0	6	0	Thyroid dis.treatment started	Feb-09
35957	C06z.00	1079	0	29	0	Thyroid disorder NOS	Feb-09
42603	AC22.00	2	0	0	0	Thyroid echinococcus granulosus	Feb-09
28530	66B4.00	798	0	9	0	Thyroid eye disease	Feb-09
23014	C04z.12	98	0	0	0	Thyroid insufficiency	Feb-09
1346	C05..00	8097	0	649	11	Thyroiditis	Feb-09
677	C02..00	100352	0	9527	54	Thyrotoxicosis	Feb-09
64856	C02y200	5	0	0	0	Thyrotoxicosis factitia	Feb-09
49508	C024.00	45	0	4	0	Thyrotoxicosis from ectopic thyroid nodule	Feb-09
56270	C024z00	4	0	1	0	Thyrotoxicosis from ectopic thyroid nodule NOS	Feb-09
64656	C024000	1	0	0	0	Thyrotoxicosis from ectopic thyroid nodule with no crisis	Feb-09
26699	C02zz00	2292	0	46	0	Thyrotoxicosis NOS	Feb-09
43136	C02y.00	49	0	0	0	Thyrotoxicosis of other specified origin	Feb-09
34220	C02yz00	38	0	0	0	Thyrotoxicosis of other specified origin NOS	Feb-09
106532	C02y100	2	0	0	0	Thyrotoxicosis of other specified origin with crisis	Aug-13
51273	C02y000	17	0	0	0	Thyrotoxicosis of other specified origin with no crisis	Feb-09
26701	C02z000	68	0	2	0	Thyrotoxicosis without mention of goitre or cause no crisis	Feb-09
15565	C02z.00	1188	0	76	0	Thyrotoxicosis without mention of goitre or other cause	Feb-09
3194	C02z100	253	0	21	0	"Thyrotoxicosis without mention of goitre, cause with crisis"	Feb-09

For peer review only - http://bmjopen.bmj.com/site/about/guidelines.xhtml

18688	F24y100	453	0	8	0	Todd's paralysis	Feb-09
8187	F251500	3156	0	85	0	Tonic-clonic epilepsy	Feb-09
22804	F251011	410	0	7	0	Tonic-clonic epilepsy	Feb-09
68975	PF27100	1	0	0	0	Total absence of ulna	Feb-09
51941	P741.00	61	0	0	0	Total anomalous pulmonary venous return - TAPVR	Feb-09
68784	P741z00	16	0	0	0	Total anomalous pulmonary venous return NOS	Feb-09
95444	790M000	20	0	0	0	Total cavopulmonary con extrac inf cav vein pulmon art con	Feb-09
91455	790M100	16	0	0	0	Total cavopulmonary connection with lateral atrial tunnel	Feb-09
39081	P334000	6	0	0	0	Total congenital cataract	Feb-09
49621	F475100	10	0	0	0	Total internal ophthalmoplegia	Feb-09
66061	PF26200	2	0	0	0	Total radial absence	Feb-09
23315	C020.00	853	0	49	0	Toxic diffuse goitre	Feb-09
49334	C020z00	105	0	1	0	Toxic diffuse goitre NOS	Feb-09
57011	C020100	13	0	0	0	Toxic diffuse goitre with crisis	Feb-09
26702	C020000	69	0	1	0	Toxic diffuse goitre with no crisis	Feb-09
55416	G52y700	8	0	1	0	Toxic myocarditis	Feb-09
63323	F382.00	4	0	0	0	Toxic myoneural disorder	Feb-09
32916	F394.00	19	0	0	0	Toxic myopathy	Feb-09
50893	K0C4.00	11	0	0	0	"Toxic nephropathy, not elsewhere classified"	Feb-09
45081	F37..11	38	0	3	0	Toxic neuropathy	Feb-09
49361	C023z00	57	0	2	0	Toxic nodular goitre NOS	Feb-09
100004	C023100	1	0	0	0	Toxic nodular goitre unspecified with crisis	Jun-10
68512	C023000	6	0	0	0	Toxic nodular goitre unspecified with no crisis	Feb-09
22373	F4H3400	359	0	25	0	Toxic optic neuropathy	Feb-09
53280	C021.00	78	0	3	0	Toxic uninodular goitre	Feb-09

For peer review only - http://bmjopen.bmj.com/site/about/guidelines.xhtml

61498	C021z00	14	0	2	0	Toxic uninodular goitre NOS	Feb-09
26869	C021000	10	0	1	0	Toxic uninodular goitre with no crisis	Feb-09
47068	C294.00	56	0	1	0	Trace element deficiency	Feb-09
68903	Q313400	3	0	0	0	Tracheobronchial haemorrhage origin in the perinatal period	Feb-09
4048	J10y200	941	0	32	0	Tracheo-oesophageal fistula	Feb-09
51167	H5y0400	76	0	2	0	Tracheo-oesophageal fistula following tracheostomy	Feb-09
62637	D011200	27	0	0	0	Transcobalamin II deficiency	Feb-09
93178	ZS78411	2	0	0	0	Transcortical motor aphasia	Feb-09
105929	ZS78611	1	0	1	0	Transcortical sensory aphasia	Apr-13
101822	ZS78600	1	0	1	0	Transcortical sensory dysphasia	Mar-11
65785	PK31.11	3	0	0	0	Transposition of abdominal viscera	Feb-09
59279	PB53100	9	0	0	0	Transposition of appendix	Feb-09
66200	PB53200	2	0	0	0	Transposition of caecum	Feb-09
45857	PB53300	15	0	1	0	Transposition of colon	Feb-09
48206	7927300	78	0	0	0	Transposition of coronary artery NEC	Feb-09
39857	7H40600	18	0	0	0	Transposition of iliopsoas muscle	Feb-09
67877	PB53.00	5	0	0	0	Transposition of intestine	Feb-09
71180	PB53000	1	0	0	0	"Transposition of intestine, unspecified"	Feb-09
71874	P74z700	3	0	1	0	Transposition of pulmonary veins	Feb-09
66199	PA77.00	9	0	0	0	Transposition of stomach	Feb-09
47596	PK30.11	12	0	0	0	Transposition of viscera unspecified	Feb-09
6494	F037.00	2471	0	125	0	Transverse myelitis	Feb-09
42239	G140112	19	0	0	0	Tricuspid incompetence - rheumatic	Feb-09
34869	G140412	107	0	3	0	"Tricuspid incompetence, cause unspecified"	Feb-09
1779	G542000	783	0	15	0	"Tricuspid incompetence, non-rheumatic"	Feb-09

For peer review only - http://bmjopen.bmj.com/site/about/guidelines.xhtml

42128	G140400	318	0	7	0	"Tricuspid insufficiency, cause unspecified"	Feb-09
97738	G542011	3	0	0	0	"Tricuspid insufficiency, non-rheumatic"	Oct-09
21980	G140111	231	0	8	0	Tricuspid regurgitation - rheumatic	Feb-09
9286	G140413	6093	0	42	0	"Tricuspid regurgitation, cause unspecified"	Feb-09
35372	G542012	1335	0	5	0	"Tricuspid regurgitation, non-rheumatic"	Feb-09
72306	G140500	3	0	0	0	"Tricuspid stenosis and insufficiency, cause unspecified"	Feb-09
49551	G140514	34	0	0	0	"Tricuspid stenosis and regurgitation, cause unspecified"	Feb-09
56029	G140300	26	0	0	0	"Tricuspid stenosis, cause unspecified"	Feb-09
35724	G542100	26	0	3	0	"Tricuspid stenosis, non-rheumatic"	Feb-09
16373	G140.00	457	0	28	0	Tricuspid valve disease NEC	Feb-09
43855	G542z00	108	0	0	0	Tricuspid valve disorders NOS	Feb-09
2817	G542.00	297	0	11	0	"Tricuspid valve disorders, non-rheumatic"	Feb-09
103577	F262E00	92	0	0	0	Trigeminal autonomic cephalalgia	Jan-12
38476	PG0B.00	127	0	3	0	Trigonocephaly	Feb-09
107520	PG04.12	1	0	0	0	Trigorhinophalangeal dysplasia	Dec-13
110299	PB6yw13	1	0	0	0	Trilobular liver	Dec-15
92988	PD3C.00	2	0	0	0	Triple kidney with triple pelvis	Feb-09
6377	PJy2.11	73	0	2	0	Triple X female	Feb-09
34913	PJ52300	35	0	0	0	Triploidy	Feb-09
54377	PJ52.00	12	0	0	0	Trisomies of autosomes NEC	Feb-09
70198	PJ50600	7	0	0	0	Trisomy 12	Feb-09
18415	PJ0..12	366	0	12	2	Trisomy 21	Feb-09
61627	PJ0z.11	30	0	0	0	Trisomy 21 NOS	Feb-09

For peer review only - http://bmjopen.bmj.com/site/about/guidelines.xhtml

42701	PJ00.00	10	0	0	0	"Trisomy 21, meiotic nondisjunction"	Feb-09
107919	PJ01.11	1	0	0	0	"Trisomy 21, mitotic nondisjunction"	Apr-14
32010	PJ01.00	29	0	0	0	"Trisomy 21, mosaicism"	Feb-09
61499	PJ02.00	11	0	2	0	"Trisomy 21, translocation"	Feb-09
100024	PJ50800	2	0	0	0	Trisomy 22	Jun-10
37591	PJ50100	3	0	0	0	Trisomy 7	Feb-09
107119	PJ52z00	1	0	0	0	Trisomy of autosomes NEC NOS	Oct-13
46264	PC70.00	43	0	3	0	True hermaphroditism	Feb-09
41371	P502.11	73	0	0	0	Truncus arteriosus	Feb-09
45187	P500.12	28	0	11	0	Truncus arteriosus	Feb-09
62310	C37y300	11	0	3	0	Trypsinogen deficiency	Feb-09
11146	C134300	625	0	41	4	TSH - thyroid-stimulating hormone deficiency	Feb-09
30721	PJ63100	26	0	1	1	"Turner's phenotype, karyotype 45X"	Feb-09
92599	PJ63200	10	0	0	0	"Turner's phenotype, karyotype 46X iso (Xq)"	Feb-09
98244	PJ63000	2	0	0	0	"Turner's phenotype, karyotype normal"	Dec-09
51868	PJ63400	38	0	1	0	"Turner's phenotype, mosaicism 45X/46XX or 45X/46XY"	Feb-09
65206	PJ63600	4	0	0	0	"Turner's phenotype, other variant karyotypes"	Feb-09
64945	PJ63612	3	0	0	0	"Turner's phenotype, partial X deletion karyotype"	Feb-09
97871	PJ63611	1	0	0	0	"Turner's phenotype, ring chromosome karyotype"	Oct-09
4943	PJ63.00	2501	0	161	0	Turner's syndrome	Feb-09
53168	PJ63z00	49	0	2	0	Turner's syndrome NOS	Feb-09

For peer review only - http://bmjopen.bmj.com/site/about/guidelines.xhtml

109385	PJ63300	4	0	0	0	"Turner's,karyotype 46X + abnorm. sex chromosome,not iso(Xq)"	Mar-15
40570	PJ63500	76	0	0	0	"Turner's,mosaic, 45X/other cell line with abn.sex chromosome"	Feb-09
1549	C10E.00	92258	0	2218	2	Type 1 diabetes mellitus	Feb-09
17858	C108.12	6354	0	175	0	Type 1 diabetes mellitus	Feb-09
35288	C10E800	340	0	30	0	Type 1 diabetes mellitus - poor control	Feb-09
45914	C108812	9	0	0	0	Type 1 diabetes mellitus - poor control	Feb-09
109628	C10P011	1	0	0	0	Type 1 diabetes mellitus in remission	May-15
40682	C10E900	82	0	1	0	Type 1 diabetes mellitus maturity onset	Feb-09
97446	C108912	2	0	0	0	Type 1 diabetes mellitus maturity onset	Aug-09
18642	C10EH00	11	0	0	0	Type 1 diabetes mellitus with arthropathy	Feb-09
49554	C10EF00	20	0	0	0	Type 1 diabetes mellitus with diabetic cataract	Feb-09
110400	C108F12	1	0	0	0	Type 1 diabetes mellitus with diabetic cataract	Dec-15
22871	C10EP00	63	0	4	0	Type 1 diabetes mellitus with exudative maculopathy	Feb-09
69993	C10E600	12	0	0	0	Type 1 diabetes mellitus with gangrene	Feb-09
55239	C10EQ00	304	0	5	0	Type 1 diabetes mellitus with gastroparesis	Feb-09
39070	C10EE00	85	0	1	0	Type 1 diabetes mellitus with hypoglycaemic coma	Feb-09
70766	C108E12	2	0	0	0	Type 1 diabetes mellitus with hypoglycaemic coma	Feb-09

For peer review only - http://bmjopen.bmj.com/site/about/guidelines.xhtml

10692	C10EM00	5880	0	106	0	Type 1 diabetes mellitus with ketoacidosis	Feb-09
40837	C10EN00	242	0	26	0	Type 1 diabetes mellitus with ketoacidotic coma	Feb-09
68105	C10EB00	11	0	0	0	Type 1 diabetes mellitus with mononeuropathy	Feb-09
47650	C10E300	37	0	0	0	Type 1 diabetes mellitus with multiple complications	Feb-09
10418	C10ED00	487	0	9	0	Type 1 diabetes mellitus with nephropathy	Feb-09
42831	C10E200	24	0	1	0	Type 1 diabetes mellitus with neurological complications	Feb-09
61829	C108212	6	0	0	0	Type 1 diabetes mellitus with neurological complications	Feb-09
18230	C108J12	3	0	1	0	Type 1 diabetes mellitus with neuropathic arthropathy	Feb-09
54008	C10EJ00	61	0	0	0	Type 1 diabetes mellitus with neuropathic arthropathy	Feb-09
47649	C10E100	21	0	2	0	Type 1 diabetes mellitus with ophthalmic complications	Feb-09
102740	C108112	1	0	0	0	Type 1 diabetes mellitus with ophthalmic complications	Aug-11
93468	C10EG00	5	0	0	0	Type 1 diabetes mellitus with peripheral angiopathy	Feb-09
30294	C10EL00	464	0	5	0	Type 1 diabetes mellitus with persistent microalbuminuria	Feb-09
30323	C10EK00	521	0	5	0	Type 1 diabetes mellitus with persistent proteinuria	Feb-09
46301	C10EC00	34	0	0	0	Type 1 diabetes mellitus with polyneuropathy	Feb-09
21983	C108012	5	0	0	0	Type 1 diabetes mellitus with renal complications	Feb-09

For peer review only - http://bmjopen.bmj.com/site/about/guidelines.xhtml

47582	C10E000	50	0	2	0	Type 1 diabetes mellitus with renal complications	Feb-09
18387	C10E700	536	0	12	0	Type 1 diabetes mellitus with retinopathy	Feb-09
41049	C108712	9	0	0	0	Type 1 diabetes mellitus with retinopathy	Feb-09
68390	C108512	15	0	0	0	Type 1 diabetes mellitus with ulcer	Feb-09
69676	C10EA00	53	0	1	0	Type 1 diabetes mellitus without complication	Feb-09
111106	C108A12	1	0	0	0	Type 1 diabetes mellitus without complication	Jul-16
104453	66At011	114	0	0	0	Type 1 diabetic dietary review	Jun-12
24599	Z271G11	150	0	3	0	Type 1 dip	Feb-09
758	C10F.00	906638	0	12665	21	Type 2 diabetes mellitus	Feb-09
17859	C109.12	46888	0	1121	2	Type 2 diabetes mellitus	Feb-09
25627	C10F700	1307	0	57	0	Type 2 diabetes mellitus - poor control	Feb-09
45913	C109712	45	0	1	0	Type 2 diabetes mellitus - poor control	Feb-09
110611	C10P111	12	0	0	0	Type 2 diabetes mellitus in remission	Mar-16
49869	C109G12	1	0	0	0	Type 2 diabetes mellitus with arthropathy	Feb-09
59253	C10FG00	50	0	2	0	Type 2 diabetes mellitus with arthropathy	Feb-09
44779	C109E12	9	0	1	0	Type 2 diabetes mellitus with diabetic cataract	Feb-09
44982	C10FE00	154	0	5	0	Type 2 diabetes mellitus with diabetic cataract	Feb-09
25591	C10FQ00	189	0	5	0	Type 2 diabetes mellitus with exudative maculopathy	Feb-09

For peer review only - http://bmjopen.bmj.com/site/about/guidelines.xhtml

12736	C10F500	54	0	4	0	Type 2 diabetes mellitus with gangrene	Feb-09
46150	C109512	7	0	0	0	Type 2 diabetes mellitus with gangrene	Feb-09
63690	C10FR00	177	0	5	0	Type 2 diabetes mellitus with gastroparesis	Feb-09
46917	C10FD00	106	0	2	0	Type 2 diabetes mellitus with hypoglycaemic coma	Feb-09
61071	C109D12	7	0	1	0	Type 2 diabetes mellitus with hypoglycaemic coma	Feb-09
32627	C10FN00	855	0	50	0	Type 2 diabetes mellitus with ketoacidosis	Feb-09
51756	C10FP00	30	0	0	0	Type 2 diabetes mellitus with ketoacidotic coma	Feb-09
62674	C10FA00	71	0	0	0	Type 2 diabetes mellitus with mononeuropathy	Feb-09
65267	C10F300	37	0	2	0	Type 2 diabetes mellitus with multiple complications	Feb-09
108005	C109312	56	0	0	0	Type 2 diabetes mellitus with multiple complications	May-14
12640	C10FC00	2067	0	64	0	Type 2 diabetes mellitus with nephropathy	Feb-09
24836	C109C12	10	0	1	0	Type 2 diabetes mellitus with nephropathy	Feb-09
34268	C10F200	176	0	3	0	Type 2 diabetes mellitus with neurological complications	Feb-09
45919	C109212	16	0	0	0	Type 2 diabetes mellitus with neurological complications	Feb-09
35385	C10FH00	214	0	7	0	Type 2 diabetes mellitus with neuropathic arthropathy	Feb-09
66965	C109H12	9	0	0	0	Type 2 diabetes mellitus with neuropathic arthropathy	Feb-09

For peer review only - http://bmjopen.bmj.com/site/about/guidelines.xhtml

47321	C10F100	100	0	1	0	Type 2 diabetes mellitus with ophthalmic complications	Feb-09
70316	C109112	39	0	0	0	Type 2 diabetes mellitus with ophthalmic complications	Feb-09
37806	C10FF00	83	0	2	0	Type 2 diabetes mellitus with peripheral angiopathy	Feb-09
60699	C109F12	4	0	0	0	Type 2 diabetes mellitus with peripheral angiopathy	Feb-09
18390	C10FM00	8573	0	54	0	Type 2 diabetes mellitus with persistent microalbuminuria	Feb-09
26054	C10FL00	4613	0	48	3	Type 2 diabetes mellitus with persistent proteinuria	Feb-09
18425	C10FB00	255	0	4	0	Type 2 diabetes mellitus with polyneuropathy	Feb-09
109865	C109B12	3	0	0	0	Type 2 diabetes mellitus with polyneuropathy	Jul-15
18777	C10F000	231	0	18	0	Type 2 diabetes mellitus with renal complications	Feb-09
18496	C10F600	1868	0	42	2	Type 2 diabetes mellitus with retinopathy	Feb-09
42762	C109612	23	0	1	0	Type 2 diabetes mellitus with retinopathy	Feb-09
49074	C10F400	116	0	4	0	Type 2 diabetes mellitus with ulcer	Feb-09
65704	C109412	14	0	2	0	Type 2 diabetes mellitus with ulcer	Feb-09
47954	C10F900	871	0	6	0	Type 2 diabetes mellitus without complication	Feb-09
105784	C109912	3	0	0	0	Type 2 diabetes mellitus without complication	Feb-13
102611	66At111	243	0	0	0	Type 2 diabetic dietary review	Jul-11
44403	Z271H11	26	0	0	0	Type 2 dip	Feb-09
37957	C10K.00	89	0	2	0	Type A insulin resistance	Feb-09

For peer review only - http://bmjopen.bmj.com/site/about/guidelines.xhtml

56885	C10K000	1	0	0	0	Type A insulin resistance without complication	Feb-09
24423	C108.13	793	0	17	0	Type I diabetes mellitus	Feb-09
46850	C108811	18	0	0	0	Type I diabetes mellitus - poor control	Feb-09
105337	C10E811	8	0	0	0	Type I diabetes mellitus - poor control	Dec-12
108360	C10P000	2	0	0	0	Type I diabetes mellitus in remission	Jul-14
63017	C108911	1	0	0	0	Type I diabetes mellitus maturity onset	Feb-09
96235	C10E911	12	0	0	0	Type I diabetes mellitus maturity onset	Apr-09
62352	C108H11	1	0	0	0	Type I diabetes mellitus with arthropathy	Feb-09
17545	C108F11	5	0	1	0	Type I diabetes mellitus with diabetic cataract	Feb-09
97894	C10EP11	1	0	0	0	Type I diabetes mellitus with exudative maculopathy	Oct-09
102112	C10E611	6	0	0	0	Type I diabetes mellitus with gangrene	Apr-11
108724	C10EQ11	1	0	0	0	Type I diabetes mellitus with gastroparesis	Oct-14
42729	C108E11	14	0	0	0	Type I diabetes mellitus with hypoglycaemic coma	Feb-09
62209	C10EM11	46	0	0	0	Type I diabetes mellitus with ketoacidosis	Feb-09
66145	C10EN11	1	0	0	0	Type I diabetes mellitus with ketoacidotic coma	Feb-09
99231	C108B11	1	0	0	0	Type I diabetes mellitus with mononeuropathy	Apr-10
91942	C10E311	5	0	0	0	Type I diabetes mellitus with multiple complications	Feb-09

For peer review only - http://bmjopen.bmj.com/site/about/guidelines.xhtml

108007	C108311	1	0	0	0	Type I diabetes mellitus with multiple complications	May-14
66872	C108D11	7	0	0	0	Type I diabetes mellitus with nephropathy	Feb-09
49146	C108211	1	0	0	0	Type I diabetes mellitus with neurological complications	Feb-09
60208	C108J11	3	0	0	0	Type I diabetes mellitus with neuropathic arthropathy	Feb-09
99311	C10E111	1	0	0	0	Type I diabetes mellitus with ophthalmic complications	May-10
102620	C10EL11	1	0	0	0	Type I diabetes mellitus with persistent microalbuminuria	Jul-11
91943	C10EC11	1	0	0	0	Type I diabetes mellitus with polyneuropathy	Feb-09
61344	C108011	6	0	0	0	Type I diabetes mellitus with renal complications	Feb-09
109837	C10E011	1	0	0	0	Type I diabetes mellitus with renal complications	Jul-15
38161	C108711	14	0	1	0	Type I diabetes mellitus with retinopathy	Feb-09
95343	C10E711	15	0	0	0	Type I diabetes mellitus with retinopathy	Feb-09
51957	C108511	10	0	1	0	Type I diabetes mellitus with ulcer	Feb-09
93878	C10E511	6	0	0	0	Type I diabetes mellitus with ulcer	Feb-09
62613	C10EA11	4	0	0	0	Type I diabetes mellitus without complication	Feb-09
95992	C108A11	1	0	0	0	Type I diabetes mellitus without complication	Feb-09
102704	66At000	7865	0	8	0	Type I diabetic dietary review	Aug-11
18219	C109.13	4924	0	125	0	Type II diabetes mellitus	Feb-09
22884	C10F.11	5124	0	13	0	Type II diabetes mellitus	Feb-09

For peer review only - http://bmjopen.bmj.com/site/about/guidelines.xhtml

24458	C109711	49	0	2	0	Type II diabetes mellitus - poor control	Feb-09
47315	C10F711	88	0	0	0	Type II diabetes mellitus - poor control	Feb-09
107824	C10P100	27	0	0	0	Type II diabetes mellitus in remission	Mar-14
18143	C109G11	1	0	0	0	Type II diabetes mellitus with arthropathy	Feb-09
103902	C10FG11	17	0	0	0	Type II diabetes mellitus with arthropathy	Mar-12
48192	C109E11	14	0	4	0	Type II diabetes mellitus with diabetic cataract	Feb-09
93727	C10FE11	52	0	1	0	Type II diabetes mellitus with diabetic cataract	Feb-09
111798	C10FQ11	1	0	0	0	Type II diabetes mellitus with exudative maculopathy	May-17
62107	C109511	7	0	1	0	Type II diabetes mellitus with gangrene	Feb-09
104323	C10F511	3	0	0	0	Type II diabetes mellitus with gangrene	Jun-12
56268	C109D11	2	0	0	0	Type II diabetes mellitus with hypoglycaemic coma	Feb-09
98723	C10FD11	22	0	0	0	Type II diabetes mellitus with hypoglycaemic coma	Mar-10
106528	C10FN11	2	0	0	0	Type II diabetes mellitus with ketoacidosis	Aug-13
106061	C10FP11	1	0	0	0	Type II diabetes mellitus with ketoacidotic coma	May-13
50813	C109A11	1	0	0	0	Type II diabetes mellitus with mononeuropathy	Feb-09
95351	C10FA11	11	0	0	0	Type II diabetes mellitus with mononeuropathy	Feb-09

For peer review only - http://bmjopen.bmj.com/site/about/guidelines.xhtml

43227	C10F311	9	0	0	0	Type II diabetes mellitus with multiple complications	Feb-09
64571	C109C11	5	0	1	0	Type II diabetes mellitus with nephropathy	Feb-09
102201	C10FC11	36	0	1	0	Type II diabetes mellitus with nephropathy	May-11
67905	C109211	5	0	0	0	Type II diabetes mellitus with neurological complications	Feb-09
98616	C10F211	2	0	0	0	Type II diabetes mellitus with neurological complications	Mar-10
47816	C109H11	7	0	3	0	Type II diabetes mellitus with neuropathic arthropathy	Feb-09
109197	C10FH11	4	0	0	0	Type II diabetes mellitus with neuropathic arthropathy	Jan-15
59725	C109111	2	0	0	0	Type II diabetes mellitus with ophthalmic complications	Feb-09
100964	C10F111	5	0	0	0	Type II diabetes mellitus with ophthalmic complications	Nov-10
54899	C109F11	4	0	0	0	Type II diabetes mellitus with peripheral angiopathy	Feb-09
104639	C10FF11	24	0	0	0	Type II diabetes mellitus with peripheral angiopathy	Aug-12
60796	C10FL11	60	0	0	0	Type II diabetes mellitus with persistent proteinuria	Feb-09
47409	C109B11	4	0	0	0	Type II diabetes mellitus with polyneuropathy	Feb-09
50527	C10FB11	17	0	0	0	Type II diabetes mellitus with polyneuropathy	Feb-09
50225	C109011	9	0	1	0	Type II diabetes mellitus with renal complications	Feb-09
57278	C10F011	5	0	0	0	Type II diabetes mellitus with renal complications	Feb-09

For peer review only - http://bmjopen.bmj.com/site/about/guidelines.xhtml

49655	C10F611	37	0	0	0	Type II diabetes mellitus with retinopathy	Feb-09
58604	C109611	21	0	5	0	Type II diabetes mellitus with retinopathy	Feb-09
55075	C109411	19	0	1	0	Type II diabetes mellitus with ulcer	Feb-09
91646	C10F411	5	0	0	0	Type II diabetes mellitus with ulcer	Feb-09
53392	C10F911	256	0	0	0	Type II diabetes mellitus without complication	Feb-09
109103	C109911	0	0	0	0	Type II diabetes mellitus without complication	Jan-15
101801	66At100	165941	0	35	0	Type II diabetic dietary review	Feb-11
111509	K08yB11	1	0	0	0	Type IV renal tubular acidosis	Dec-16
31001	C302611	47	0	0	0	Tyrosinaemia	Feb-09
55159	46F7.11	0	0	0	2	Tyrosine cryst.- urine	Feb-09
45295	C302300	5	0	0	0	Tyrosinosis	Feb-09
63807	C302400	11	0	0	0	Tyrosinuria	Feb-09
30433	J411.00	221	0	8	0	Ulcerative (chronic) enterocolitis	Feb-09
42822	J412.00	35	0	2	0	Ulcerative (chronic) ileocolitis	Feb-09
704	J410100	92252	0	4661	46	Ulcerative colitis	Feb-09
1784	J41..12	32854	0	1756	30	Ulcerative colitis and/or proctitis	Feb-09
48732	J410000	39	0	3	0	Ulcerative ileocolitis	Feb-09
104428	J08zF11	172	0	2	0	Ulcerative oral mucositis	Jun-12
104259	J413.00	408	0	4	0	Ulcerative pancolitis	Jun-12
8347	J410300	3226	0	103	0	Ulcerative proctitis	Feb-09
6650	J410.00	8183	0	464	4	Ulcerative proctocolitis	Feb-09
33456	J410z00	135	0	1	0	Ulcerative proctocolitis NOS	Feb-09
24858	J410200	197	0	1	0	Ulcerative rectosigmoiditis	Feb-09
105762	B62C.00	7	0	0	0	Unifocal Langerhans-cell histiocytosis	Feb-13
63930	P911.00	21	0	0	0	Unilateral complete cleft lip	Feb-09
73531	P901.00	3	0	0	0	Unilateral complete cleft palate	Feb-09

For peer review only - http://bmjopen.bmj.com/site/about/guidelines.xhtml

62987	P921.00	16	0	0	0	Unilateral complete cleft palate with cleft lip	Feb-09
102267	J06y700	4	0	0	0	Unilateral condylar mandibular hyperplasia	May-11
103529	J06y800	1	0	0	0	Unilateral condylar mandibular hypoplasia	Jan-12
23958	PD02100	362	0	4	0	Unilateral congenital absence of kidney	Feb-09
45970	PE30000	266	0	3	0	Unilateral congenital dislocation of hip	Feb-09
34019	PE31000	78	0	0	0	Unilateral congenital subluxation of hip	Feb-09
38772	PE34000	135	0	0	0	Unilateral dysplastic hip	Feb-09
68946	F255500	4	0	0	0	Unilateral epilepsy	Feb-09
54012	P912.00	54	0	0	0	Unilateral incomplete cleft lip	Feb-09
73558	P902.00	8	0	0	0	Unilateral incomplete cleft palate	Feb-09
54144	P922.00	5	0	0	0	Unilateral incomplete cleft palate with cleft lip	Feb-09
31961	PD00100	80	0	0	0	Unilateral renal agenesis	Feb-09
54656	PD04111	3	0	0	0	Unilateral renal dysgenesis	Feb-09
24120	PD04100	63	0	2	0	Unilateral renal dysplasia	Feb-09
30650	PD03100	46	0	1	0	Unilateral renal hypoplasia	Feb-09
43919	K090.00	309	0	3	0	Unilateral small kidney	Feb-09
105369	K090100	3	0	1	0	Unilateral small kidney with contralateral hypertrophy	Dec-12
31097	K16X.00	188	0	1	0	"Uninhibited neuropathic bladder, NEC"	Feb-09
62520	K03W.00	8	0	0	0	"Unsp nephrit synd, diff endocap prolif glomerulonephritis"	Feb-09
30301	K03X.00	38	0	1	0	"Unsp nephrit synd, diff mesang prolif glomerulonephritis"	Feb-09

For peer review only

For peer review only - http://bmjopen.bmj.com/site/about/guidelines.xhtml

73574	P2y..00	6	0	0	0	Unspec nervous system anomaly of brain/cord/nervous system	Feb-09
36125	K03U.00	35	0	1	0	"Unspecif nephr synd, diff concentric glomerulonephritis"	Feb-09
25280	PAz2.00	93	0	8	0	Unspecified anomalies of stomach	Feb-09
44752	PDz2.00	103	0	6	0	Unspecified anomaly of bladder	Feb-09
22492	PGz2.00	84	0	7	0	Unspecified anomaly of bones	Feb-09
28247	PGz4.00	53	0	2	0	Unspecified anomaly of connective tissue	Feb-09
26626	P6z0.00	89	0	6	0	Unspecified anomaly of heart valve	Feb-09
46880	PDz3.00	75	0	10	0	Unspecified anomaly of urethra	Feb-09
70721	E117500	6	0	0	0	"Unspecified bipolar affect disord, partial/unspec remission"	Feb-09
24230	E117600	282	0	0	0	"Unspecified bipolar affective disorder, in full remission"	Feb-09
63698	E117100	12	0	0	0	"Unspecified bipolar affective disorder, mild"	Feb-09
68647	E117200	14	0	0	0	"Unspecified bipolar affective disorder, moderate"	Feb-09
27986	E117z00	310	0	5	0	"Unspecified bipolar affective disorder, NOS"	Feb-09
73423	E117300	3	0	0	0	"Unspecified bipolar affective disorder, severe, no psychosis"	Feb-09
49763	E117000	89	0	0	0	"Unspecified bipolar affective disorder, unspecified"	Feb-09
68326	E117400	14	0	0	0	"Unspecified bipolar affective disorder,severe with psychosis"	Feb-09
71029	PJ5z.00	4	0	0	0	Unspecified conditions due to autosomal anomalies	Feb-09
64449	C108z00	3	0	0	0	Unspecified diabetes mellitus with multiple complications	Feb-09
62920	J34y.00	101	0	11	11	Unspecified diaphragmatic hernia	Feb-09

For peer review only - http://bmjopen.bmj.com/site/about/guidelines.xhtml

5182	K03z.00	3309	0	206	3	Unspecified glomerulonephritis NOS	Feb-09
66506	E101000	1	0	0	0	Unspecified hebephrenic schizophrenia	Feb-09
99620	F435000	1	0	0	0	Unspecified hereditary choroid dystrophy	Jun-10
42511	F427000	14	0	0	0	Unspecified hereditary retinal dystrophies	Feb-09
94560	F4J3000	13	0	0	0	Unspecified heterotropia	Feb-09
73638	AB4zz00	2	0	0	0	Unspecified histoplasmosis NOS	Feb-09
96000	A907.00	2	0	0	0	Unspecified late congenital syphilis	Feb-09
69370	P118000	2	0	0	0	Unspecified spina bifida without hydrocephalus - closed	Feb-09
95018	P110z00	2	0	0	0	Unspecified spina bifida without hydrocephalus NOS	Feb-09
66679	PG51.15	12	0	0	0	Van der Hoeve's syndrome	Feb-09
2155	G341000	990	0	27	0	Ventricular cardiac aneurysm	Feb-09
246	P54..00	27151	0	753	1	Ventricular septal defect	Feb-09
23692	P520.11	250	0	14	0	Ventricular septal defect in Fallot's tetralogy	Feb-09
34067	P54z.00	488	0	3	0	Ventricular septal defect NOS	Feb-09
42132	P540.00	220	0	0	0	"Ventricular septal defect, unspecified"	Feb-09
7794	G571.11	3193	0	84	0	Ventricular tachycardia	Feb-09
35762	7010000	119	0	10	0	Ventriculocisternostomy	Feb-09
26280	F28y300	450	0	0	0	Ventriculomegaly	Feb-09
2482	D011100	69	0	5	0	Vit B12 defic anaemia due to malabsorption with proteinuria	Feb-09
5007	C24..00	218	0	2	2	Vitamin A deficiency	Feb-09
31479	C241.00	24	0	1	0	Vitamin A deficiency + Bitot's spot and conjunctival xerosis	Feb-09
50073	C240.00	12	0	0	0	Vitamin A deficiency with conjunctival xerosis	Feb-09

For peer review only - http://bmjopen.bmj.com/site/about/guidelines.xhtml

61766	C242.00	2	0	0	0	Vitamin A deficiency with corneal xerosis	Feb-09
102237	C244.00	1	0	0	0	Vitamin A deficiency with keratomalacia	May-11
66943	C245.00	8	0	3	0	Vitamin A deficiency with night blindness	Feb-09
45889	C24y.00	1	0	0	0	Vitamin A deficiency with other manifestation	Feb-09
49270	C24y100	10	0	0	0	Vitamin A deficiency with xeroderma	Feb-09
49266	C247000	6	0	0	0	Vitamin A deficiency with xerophthalmia	Feb-09
60199	C246.00	18	0	2	0	Vitamin A deficiency with xerophthalmic corneal scars	Feb-09
42792	C26z.00	228	0	0	0	Vitamin B deficiency NOS	Feb-09
94706	42d6.00	69	0	2	906	Vitamin B profile	Feb-09
4667	C25..11	272	0	91	83	Vitamin B1 deficiency	Feb-09
5271	D011.11	4712	0	201	41	Vitamin B12 deficiency anaemia	Feb-09
32953	D011000	222	0	0	0	Vitamin B12 deficiency anaemia due to dietary causes	Feb-09
24059	C260.12	175	0	4	2	Vitamin B2 deficiency	Feb-09
43354	C261.11	48	0	1	1	Vitamin B6 deficiency	Feb-09
48023	C261.00	39	0	0	0	Vitamin B6 deficiency syndrome	Feb-09
45946	C26..00	343	0	4	2	Vitamin B-complex deficiency	Feb-09
37282	C27..12	134	0	2	2	Vitamin C deficiency	Feb-09
104812	D01y000	2	0	0	0	Vitamin C deficiency anaemia	Aug-12
106409	C2B..00	3201	0	7	2	Vitamin D insufficiency	Jun-13
53111	8B73.00	3033	0	14	2	Vitamin D supplements	Feb-09
105896	C28A.00	9	0	0	0	Vitamin D-dependent rickets	Mar-13
51679	C291000	32	0	0	0	Vitamin E deficiency	Feb-09
62257	D01y100	3	0	0	0	Vitamin E deficiency anaemia	Feb-09
100830	C291100	6	0	0	0	Vitamin P deficiency	Nov-10

For peer review only - http://bmjopen.bmj.com/site/about/guidelines.xhtml

33586	C353200	38	0	3	0	Vitamin-D-resistant rickets	Feb-09
29168	F427C00	384	0	3	0	Vitelliform dystrophy	Feb-09
975	M295100	45691	0	4650	4	Vitiligo	Feb-09
65925	C310211	6	0	0	0	Von Gierke's disease	Feb-09
28443	PK62.00	242	0	10	0	Von Hippel-Lindau syndrome	Feb-09
23137	42Qx.00	0	0	0	507	von Willebrand factor activity	Feb-09
34555	42jf.00	1	0	0	618	von Willebrand factor antigen level	Feb-09
34554	42jg.00	0	0	0	213	von Willebrand factor collagen binding assay	Feb-09
109412	42hB.00	0	0	0	2	von Willebrand Normandy screening test	Mar-15
17520	D304.00	4168	0	166	7	Von Willebrand's disease	Feb-09
108235	C333011	1	0	0	0	Waldenstrom macroglobulinaemia	Jun-14
57509	C330000	10	0	1	0	Waldenstrom's hypergammaglobulinaemic purpura	Feb-09
10411	C333000	887	0	29	1	Waldenstrom's macroglobulinaemia	Feb-09
4810	G754.00	3741	0	128	3	Wegener's granulomatosis	Feb-09
66490	C180.11	4	0	0	0	Wermer's syndrome	Feb-09
57803	C1zy400	2	0	1	0	Werner's syndrome	Feb-09
18636	E011200	415	0	18	0	Wernicke-Korsakov syndrome	Feb-09
51158	ZS78D13	7	0	0	0	Wernicke's aphasia	Feb-09
65074	ZS78D00	6	0	0	0	Wernicke's dysphasia	Feb-09
68256	PJ36.00	1	0	0	0	"Whole chromosome monosomy, meiotic nondisjunction"	Feb-09
95598	PJ37.00	2	0	0	0	"Whole chromosome monosomy, mosaicism"	Feb-09
67815	PJ37z00	1	0	0	0	"Whole chromosome monosomy, mosaicism NOS"	Feb-09
101982	PJ50z00	1	0	0	0	Whole chromosome trisomy syndrome NOS	Apr-11
37702	PJ50.00	21	0	2	0	Whole chromosome trisomy syndromes	Feb-09

For peer review only - http://bmjopen.bmj.com/site/about/guidelines.xhtml

For peer review only

107670	PJ50w00	1	0	0	0	"Whole chromosome trisomy, meitotic nondisjunction"	Feb-14
45512	PJ50x00	8	0	0	0	"Whole chromosome trisomy, mosaicism"	Feb-09
32054	PKy9111	72	0	1	0	Wiedemann - Beckwith syndrome	Feb-09
48476	Q317200	26	0	0	0	Wilson-Mikity syndrome	Feb-09
3870	C351011	404	0	28	0	Wilson's disease	Feb-09
31322	C391200	49	0	5	0	Wiskott - Aldrich syndrome	Feb-09
8230	G567400	7350	0	488	1	Wolff-Parkinson-White syndrome	Feb-09
109868	C327300	2	0	0	0	Wolman disease	Jul-15
70109	F151300	7	0	1	0	X-linked bulbo-spinal atrophy	Feb-09
107207	P235.00	1	0	0	0	X-linked hydrocephalus	Nov-13
107983	C353700	7	0	0	0	X-linked hypophosphataemic rickets	May-14
35335	PH15.00	48	0	3	0	X-linked ichthyosis	Feb-09
32782	PJy2.00	67	0	2	0	XXX syndrome	Feb-09
11948	PJy3.00	39	0	0	0	XXY syndrome	Feb-09
41152	PJ64000	21	0	1	0	"XY, female phenotype"	Feb-09
23355	C115.11	239	0	10	0	Zollinger - Ellison syndrome	Feb-09

For peer review only

Appendix 4 ICD10 codes to identify a children with a chronic, not life-limiting condition

ICD 10 code

A15.0

A15.1

A15.2

A15.3

A15.4

A15.5

A15.6

A15.7

A15.8

A15.9

A16.0

A16.1

A16.2

A16.3

A16.4

A16.5

A16.7

A16.8

For peer review only

A16.9

A18.0

A18.1

A18.2

A18.3

A18.4

A18.5

A18.6

A18.7

A18.8

A19.0

A19.1

A19.2

A19.8

A19.9

A50.0

A50.1

A50.2

A50.3

A50.4

For peer review only

For peer review only - http://bmjopen.bmj.com/site/about/guidelines.xhtml

A50.5
A50.6
A50.7
A50.9
A81.2
A81.8
A81.9
B18.0
B18.1
B18.2
B18.8
B18.9
B37.7
B37.1
B37.5
B37.6
B38.1
B39.1
B40.1
B44.0

For peer review only - http://bmjopen.bmj.com/site/about/guidelines.xhtml

B44.7

B45.0

B45.1

B45.2

B45.3

B45.7

B45.8

B45.9

B46.0

B46.1

B46.2

B46.3

B46.4

B46.5

B46.8

B46.9

B48.7

B50.0

B51.0

B52.0

For peer review only

B55.0

B55.1

B55.2

B55.9

B57.2

B57.3

B57.4

B57.5

B58.0

B59

B67.0

B67.1

B67.2

B67.3

B67.4

B67.5

B67.6

B67.7

B67.8

B67.9

For peer review only

For peer review only - http://bmjopen.bmj.com/site/about/guidelines.xhtml

B69.0

B69.1

B69.8

B69.9

B73

B74.0

B74.1

B74.2

B74.3

B74.4

B74.8

B74.9

B78.7

B90.0

B90.1

B90.2

B90.8

B90.9

B91

B92

For peer review only - http://bmjopen.bmj.com/site/about/guidelines.xhtml

B94.0

B94.1

B94.2

B94.8

B94.9

D00.0

D00.1

D00.2

D01.0

D01.1

D01.2

D01.3

D01.4

D01.5

D01.7

D01.9

D02.0

D02.1

D02.2

D02.3

For peer review only

For peer review only - http://bmjopen.bmj.com/site/about/guidelines.xhtml

D02.4

D05.0

D05.1

D05.7

D05.9

D06.0

D06.1

D06.7

D06.9

D07.0

D07.1

D07.2

D07.3

D07.4

D07.5

D07.6

D09.0

D09.1

D09.2

D09.3

For peer review only - http://bmjopen.bmj.com/site/about/guidelines.xhtml

D09.7

D09.9

D12.0

D12.1

D12.2

D12.3

D12.4

D12.5

D12.6

D12.7

D12.8

D12.9

D13.0

D13.1

D13.2

D13.3

D13.4

D13.5

D13.6

D13.7

For peer review only

For peer review only - http://bmjopen.bmj.com/site/about/guidelines.xhtml

D13.9
D14.1
D14.2
D14.3
D14.4
D15.0
D151
D15.2
D15.7
D15.9
D20.0
D20.1
D32.0
D32.1
D34
D35.0
D35.1
D35.2
D35.3
D35.4

For peer review only

For peer review only - http://bmjopen.bmj.com/site/about/guidelines.xhtml

D35.5

D35.6

D35.7

D35.8

D35.9

D37.0

D37.1

D37.2

D37.3

D37.4

D37.5

D37.6

D37.7

D37.9

D38.0

D38.1

D38.2

D38.3

D38.4

D38.5

For peer review only - http://bmjopen.bmj.com/site/about/guidelines.xhtml

D38.6

D39.0

D39.1

D39.2

D39.7

D39.9

D40.0

D40.1

D40.7

D40.9

D41.0

D41.1

D41.2

D41.3

D41.4

D41.7

D41.9

D42.0

D42.1

D42.9

For peer review only

For peer review only - http://bmjopen.bmj.com/site/about/guidelines.xhtml

D44.0

D44.1

D44.2

D44.3

D44.5

D44.7

D44.8

D44.9

D45

D46.0

D46.1

D46.2

D46.4

D46.5

D46.6

D46.7

D46.9

D47.0

D47.1

D47.2

For peer review only

D47.3

D47.4

D47.5

D47.7

D47.9

D55.0

D55.1

D55.2

D55.3

D55.8

D55.9

D56.0

D56.2

D56.4

D56.8

D56.9

D57.0

D57.1

D57.2

D57.8

For peer review only

For peer review only - http://bmjopen.bmj.com/site/about/guidelines.xhtml

D58.0

D58.1

D58.2

D58.8

D58.9

D63.0

D63.8

D66

D68.0

D68.1

D68.2

D68.4

D68.5

D68.6

D68.8

D68.9

D69.0

D69.1

D69.2

D69.3

For peer review only - http://bmjopen.bmj.com/site/about/guidelines.xhtml

D69.4

D69.5

D69.6

D69.8

D69.9

D71

D72.0

D72.1

D72.8

D72.9

D73.0

D73.1

D73.2

D73.3

D73.4

D73.5

D73.8

D73.9

D74.0

D74.8

For peer review only

For peer review only - http://bmjopen.bmj.com/site/about/guidelines.xhtml

D74.9

D75.0

D75.1

D75.8

D75.9

D76.1

D76.2

D76.3

D80.0

D80.1

D80.2

D80.3

D80.4

D80.8

D80.9

D82.0

D82.2

D82.3

D82.4

D82.5

For peer review only

D82.6

D82.7

D82.8

D82.9

D84.0

D84.1

D84.8

D84.9

E00.0

E00.1

E00.2

E00.9

E03.0

E03.1

E07.1

E10

E11

E12

E13

E14

For peer review only

For peer review only - http://bmjopen.bmj.com/site/about/guidelines.xhtml

E22.0

E23.0

E24.4

E25.0

E25.8

E25.9

E26.8

E29.1

E31.1

E31.8

E31.9

E34.0

E34.1

E34.2

E34.5

E35.0

E66.0

E66.1

E66.2

E66.8

For peer review only

E66.9

E70.0

E70.3

E70.8

E70.9

E78.0

E78.1

E78.2

E78.3

E78.4

E78.5

E78.6

E78.8

E78.9

E79.8

E79.9

E80.0

E80.1

E80.2

E80.3

For peer review only - http://bmjopen.bmj.com/site/about/guidelines.xhtml

E80.5

E80.7

E83.1

E83.2

E83.3

E83.4

E83.5

E83.8

E83.9

E85.0

E85.1

E85.2

E85.3

E85.4

E85.8

E85.9

E88.2

E88.3

E88.8

E88.9

For peer review only

For peer review only - http://bmjopen.bmj.com/site/about/guidelines.xhtml

F00.0

F00.1

F00.2

F00.9

F01.0

F01.1

F01.2

F01.3

F01.8

F01.9

F02.1

F02.2

F02.3

F02.4

F02.8

F03

F04

F05.0

F05.1

F05.8

For peer review only

For peer review only - http://bmjopen.bmj.com/site/about/guidelines.xhtml

F05.9

F06.0

F06.1

F06.2

F06.3

F06.4

F06.5

F06.6

F06.7

F06.8

F07.0

F07.1

F07.2

F07.8

F07.9

F09

F10

F11

F12

F13

For peer review only

For peer review only - http://bmjopen.bmj.com/site/about/guidelines.xhtml

F14

F15

F16

F17

F18

F19

F20.0

F20.1

F20.2

F20.3

F20.4

F20.5

F20.6

F20.8

F20.9

F21

F22.0

F22.8

F22.9

F23.0

For peer review only - http://bmjopen.bmj.com/site/about/guidelines.xhtml

F23.1

F23.2

F23.3

F23.8

F23.9

F24

F25.0

F25.1

F25.2

F25.8

F25.9

F28

F29

F30.0

F30.1

F30.2

F30.8

F30.9

F31.0

F31.1

For peer review only - http://bmjopen.bmj.com/site/about/guidelines.xhtml

F31.2

F31.3

F31.4

F31.5

F31.6

F31.7

F31.8

F31.9

F32.0

F32.1

F32.2

F32.3

F32.8

F32.9

F33.0

F33.1

F33.2

F33.3

F33.4

F33.8

For peer review only

For peer review only - http://bmjopen.bmj.com/site/about/guidelines.xhtml

F33.9

F34.0

F34.1

F34.8

F34.9

F38.0

F38.1

F38.8

F39

F40.0

F40.1

F40.2

F40.8

F40.9

F41.0

F41.1

F41.2

F41.3

F41.8

F41.9

For peer review only

F42.0

F42.1

F42.2

F42.8

F42.9

F43.0

F43.1

F43.2

F43.8

F43.9

F44.0

F44.1

F44.2

F44.3

F44.4

F44.5

F44.6

F44.7

F44.8

F44.9

For peer review only - http://bmjopen.bmj.com/site/about/guidelines.xhtml

F45.0

F45.1

F45.2

F45.3

F45.4

F45.8

F45.9

F48.1

F48.8

F48.9

F50.0

F50.1

F50.2

F50.3

F50.4

F50.5

F50.8

F50.9

F53.0

F53.1

For peer review only

F53.8

F53.9

F54

F70

F71

F72

F73

F78

F79

F80.0

F80.1

F80.2

F80.8

F80.9

F82

F83

F84.1

F84.3

F84.4

F84.5

For peer review only

For peer review only - http://bmjopen.bmj.com/site/about/guidelines.xhtml

F84.6

F84.8

F84.9

F88

F89

F90.0

F90.1

F90.8

F90.9

F91.0

F91.1

F91.2

F91.3

F91.8

F91.9

F92.0

F92.8

F92.9

F93.0

F93.1

For peer review only - http://bmjopen.bmj.com/site/about/guidelines.xhtml

F93.2

F93.3

F93.8

F93.9

F94.0

F94.1

F94.2

F94.8

F94.9

F95.0

F95.1

F95.2

F95.8

F95.9

F98.0

F98.1

F98.2

F98.3

F98.4

F98.5

For peer review only

For peer review only - http://bmjopen.bmj.com/site/about/guidelines.xhtml

F98.6

F98.8

F98.9

G00.0

G00.1

G00.2

G00.3

G00.8

G00.9

G01

G02.0

G02.1

G02.8

G03.0

G03.1

G03.2

G03.8

G03.9

G04.0

G04.1

For peer review only

G04.2

G04.8

G04.9

G05.0

G05.1

G05.2

G05.8

G06.0

G06.1

G06.2

G07

G08

G09

G11.0

G11.4

G11.8

G11.9

G13.0

G13.1

G13.2

For peer review only

G13.8

G14

G21.0

G21.1

G21.2

G21.3

G21.4

G21.8

G21.9

G22

G23.1

G23.2

G23.3

G23.9

G24.1

G24.2

G24.3

G24.4

G24.5

G24.8

For peer review only - http://bmjopen.bmj.com/site/about/guidelines.xhtml

For peer review only

G24.9

G25.0

G25.1

G25.2

G25.3

G25.4

G25.5

G25.6

G25.8

G25.9

G26

G30.0

G30.1

G30.8

G30.9

G31.0

G31.1

G31.2

G32.0

G32.8

For peer review only

For peer review only - http://bmjopen.bmj.com/site/about/guidelines.xhtml

G36.0

G36.1

G36.8

G36.9

G37.0

G37.1

G37.2

G37.3

G37.4

G37.5

G37.8

G37.9

G40.0

G40.1

G40.2

G40.3

G40.6

G40.7

G40.8

G40.9

For peer review only
For peer review only - http://bmjopen.bmj.com/site/about/guidelines.xhtml

G41.0

G41.1

G41.2

G41.8

G41.9

G43.0

G43.1

G43.2

G43.3

G43.8

G43.9

G44.0

G44.1

G44.2

G44.3

G44.4

G44.8

G45.0

G45.1

G45.2

For peer review only

For peer review only - http://bmjopen.bmj.com/site/about/guidelines.xhtml

G45.3

G45.4

G45.8

G45.9

G46.0

G46.1

G46.2

G46.3

G46.4

G46.5

G46.6

G46.7

G46.8

G47.0

G47.1

G47.2

G47.3

G47.4

G47.8

G47.9

For peer review only - http://bmjopen.bmj.com/site/about/guidelines.xhtml

G50.0

G50.1

G50.8

G50.9

G51.0

G51.1

G51.2

G51.3

G51.4

G51.8

G51.9

G52.0

G52.1

G52.2

G52.3

G52.7

G52.8

G52.9

G53.0

G53.1

For peer review only - http://bmjopen.bmj.com/site/about/guidelines.xhtml

G53.2

G53.3

G53.8

G54.0

G54.1

G54.2

G54.3

G54.4

G54.5

G54.6

G54.7

G54.8

G54.9

G55.0

G55.1

G55.2

G55.3

G55.8

G56.0

G56.1

For peer review only - http://bmjopen.bmj.com/site/about/guidelines.xhtml

G56.2

G56.3

G56.4

G56.8

G56.9

G57.0

G57.1

G57.2

G57.3

G57.4

G57.5

G57.6

G57.8

G57.9

G58.0

G58.7

G58.8

G58.9

G59.0

G59.8

For peer review only

For peer review only - http://bmjopen.bmj.com/site/about/guidelines.xhtml

G60.2

G60.3

G60.8

G60.9

G61.0

G61.1

G61.8

G61.9

G62.0

G62.1

G62.2

G62.8

G62.9

G63.1

G63.2

G63.3

G63.5

G63.6

G63.8

G64

For peer review only - http://bmjopen.bmj.com/site/about/guidelines.xhtml

G70.0

G70.1

G70.8

G71.8

G71.9

G72.0

G72.1

G72.2

G72.3

G72.4

G72.8

G72.9

G73.0

G73.1

G73.2

G73.3

G73.5

G73.6

G73.7

G80.1

For peer review only - http://bmjopen.bmj.com/site/about/guidelines.xhtml

G80.2

G80.3

G80.4

G80.9

G81.0

G81.1

G81.9

G82.0

G82.1

G82.2

G83.0

G83.1

G83.2

G83.3

G83.4

G83.5

G83.8

G83.9

G90.0

G90.1

For peer review only

For peer review only - http://bmjopen.bmj.com/site/about/guidelines.xhtml

G90.2

G90.4

G90.8

G90.9

G91.0

G91.1

G91.2

G91.3

G91.8

G91.9

G92

G93.0

G93.1

G93.2

G93.3

G93.8

G93.9

G94.1

G94.2

G94.8

For peer review only

For peer review only - http://bmjopen.bmj.com/site/about/guidelines.xhtml

G95.0

G95.1

G95.2

G95.8

G95.9

G96.0

G96.1

G96.8

G96.9

G99.0

G99.1

G99.2

G99.8

H05.1

H05.2

H05.3

H05.4

H05.5

H05.8

H05.9

For peer review only - http://bmjopen.bmj.com/site/about/guidelines.xhtml

H13.3

H17.0

H17.1

H17.8

H17.9

H18.0

H18.1

H18.2

H18.3

H18.4

H18.5

H18.6

H18.7

H18.8

H18.9

H19.3

H21.0

H21.2

H21.2

H21.3

For peer review only

For peer review only - http://bmjopen.bmj.com/site/about/guidelines.xhtml

H21.4

H21.5

H21.8

H21.9

H26.0

H26.1

H26.2

H26.3

H26.4

H26.8

H26.9

H27.0

H27.1

H27.8

H27.9

H28.0

H28.1

H28.2

H31.0

H31.1

For peer review only

H31.2

H31.3

H31.4

H31.8

H31.9

H32.8

H33.0

H33.1

H33.2

H33.3

H33.4

H33.5

H34.0

H34.1

H34.2

H34.8

H34.9

H35.0

H35.1

H35.2

For peer review only

H35.3

H35.4

H35.6

H35.7

H35.8

H35.9

H40.0

H40.1

H40.2

H40.3

H40.4

H40.5

H40.6

H40.8

H40.9

H42.0

H43.0

H43.1

H43.2

H43.3

For peer review only - http://bmjopen.bmj.com/site/about/guidelines.xhtml

H43.8

H43.9

H44.0

H44.1

H44.2

H44.3

H44.4

H44.5

H44.6

H44.7

H44.8

H44.9

H47.0

H47.1

H47.3

H47.4

H47.5

H47.6

H47.7

H54.0

For peer review only

For peer review only - http://bmjopen.bmj.com/site/about/guidelines.xhtml

H54.1

H54.2

H54.4

H60.2

H65.2

H65.3

H65.4

H66.1

H66.2

H66.3

H69.0

H69.8

H69.9

H70.1

H73.1

H74.0

H74.1

H74.2

H74.3

H75.0

For peer review only - http://bmjopen.bmj.com/site/about/guidelines.xhtml

H80.0

H80.1

H80.2

H80.8

H80.9

H81.0

H81.4

H83.0

H83.2

H90.0

H90.3

H90.5

H90.6

H91.0

H91.1

H91.2

H91.3

H91.8

H91.9

I43.1

For peer review only

I60.0

I60.1

I60.2

I60.3

I60.4

I60.5

I60.6

I60.7

I60.8

I60.9

I61.0

I61.1

I61.2

I61.4

I61.5

I61.6

I61.8

I61.9

I62.0

I62.1

For peer review only

For peer review only - http://bmjopen.bmj.com/site/about/guidelines.xhtml

I62.9

I63.0

I63.1

I63.2

I63.3

I63.4

I63.5

I63.6

I63.8

I63.9

I64

I65.0

I65.1

I65.2

I65.3

I65.8

I65.9

I66.0

I66.1

I66.2

For peer review only

I66.3

I66.4

I66.8

I66.9

I67.0

I67.1

I67.2

I67.3

I67.4

I67.5

I67.6

I67.7

I67.8

I67.9

I68.0

I68.2

I68.8

I69.0

I69.1

I69.2

For peer review only - http://bmjopen.bmj.com/site/about/guidelines.xhtml

I69.3

I69.4

I69.8

I72.0

I72.5

I72.9

I79.2

J41.0

J41.1

J41.8

J42

J43.0

J43.1

J43.2

J43.8

J43.9

J44.0

J44.1

J44.8

J44.9

For peer review only

For peer review only - http://bmjopen.bmj.com/site/about/guidelines.xhtml

J45.0

J45.1

J45.8

J45.9

J46

J47

J60

J61

J62.0

J62.8

J63.0

J63.1

J63.2

J63.3

J63.4

J63.5

J63.8

J64

J65

J66.0

For peer review only

J66.1

J66.2

J66.8

J67.0

J67.1

J67.2

J67.3

J67.4

J67.5

J67.6

J67.7

J67.8

J67.9

J68.0

J68.1

J68.2

J68.3

J68.4

J68.8

J68.9

For peer review only
For peer review only - http://bmjopen.bmj.com/site/about/guidelines.xhtml

J69.0

J69.1

J69.8

J70.0

J70.1

J70.2

J70.3

J70.4

J70.8

J70.9

J80

J81

J82

J84.0

J84.8

J84.9

J85.0

J85.1

J85.2

J85.3

For peer review only - http://bmjopen.bmj.com/site/about/guidelines.xhtml

For peer review only

J86.0

J86.9

J98.0

J98.1

J98.2

J98.3

J98.5

J98.6

J98.8

J98.9

J99.0

J99.1

K20

K21.0

K22.0

K22.1

K22.2

K22.3

K22.4

K22.5

For peer review only - http://bmjopen.bmj.com/site/about/guidelines.xhtml

K22.6

K22.7

K22.8

K22.9

K23.0

K23.1

K23.8

K25

K26

K27

K28

K29.0

K29.1

K29.2

K29.3

K29.4

K29.5

K29.6

K29.7

K29.8

For peer review only

K29.9

K31.0

K31.1

K31.2

K31.3

K31.4

K31.5

K31.6

K31.7

K31.8

K31.9

K50.0

K50.1

K50.8

K50.9

K51.0

K51.2

K51.3

K51.4

K51.5

For peer review only

K51.8

K51.9

K52.0

K52.1

K52.2

K52.3

K52.8

K52.9

K55.1

K55.2

K55.8

K57.0

K57.1

K57.2

K57.3

K57.4

K57.5

K57.8

K57.9

K59.2

For peer review only

For peer review only - http://bmjopen.bmj.com/site/about/guidelines.xhtml

K63.0

K63.1

K63.2

K63.3

K66.0

K66.1

K66.8

K66.9

K67.3

K70.0

K70.1

K70.2

K70.3

K70.4

K70.9

K73.0

K73.1

K73.2

K73.8

K73.9

311

For peer review only

K75.0

K75.1

K75.2

K75.3

K75.4

K75.8

K75.9

K76.0

K76.1

K76.2

K76.3

K76.4

K76.6

K76.7

K76.9

K80.0

K80.1

K80.2

K80.3

K80.4

For peer review only - http://bmjopen.bmj.com/site/about/guidelines.xhtml

For peer review only

K80.5
K80.8
K81.0
K81.1
K81.8
K81.9
K82.0
K82.1
K82.2
K82.3
K82.4
K82.8
K82.9
K83.0
K83.1
K83.2
K83.3
K83.4
K83.5
K83.8

For peer review only

K83.9

K85.0

K85.1

K85.2

K85.3

K85.8

K85.9

K86.0

K86.1

K86.2

K86.3

K86.9

K87.0

K90.0

K90.1

K90.2

K90.3

K90.4

K90.8

K90.0

For peer review only

For peer review only - http://bmjopen.bmj.com/site/about/guidelines.xhtml

K93.0

K93.1

L10.0

L10.1

L10.2

L10.3

L10.4

L10.5

L10.8

L10.9

L11.0

L11.8

L11.9

L12.0

L12.1

L12.2

L12.3

L12.8

L12.9

L13.0

For peer review only

For peer review only - http://bmjopen.bmj.com/site/about/guidelines.xhtml

L13.1

L13.8

L13.9

L14

L28.0

L28.1

L28.2

L40.0

L40.1

L40.2

L40.3

L40.4

L40.5

L40.8

L40.9

L41.0

L41.1

L41.3

L41.4

L41.5

For peer review only - http://bmjopen.bmj.com/site/about/guidelines.xhtml

L41.8

L41.9

L42

L43.0

L43.1

L43.2

L43.3

L43.8

L43.9

L44.0

L44.1

L44.2

L44.3

L44.4

L44.8

L44.9

L45

L57.0

L57.1

L57.2

317

For peer review only

L57.3

L57.4

L57.5

L57.8

L57.9

L58.1

L59.0

L59.8

L59.9

L62.0

L87.0

L87.1

L87.2

L87.8

L87.9

L88

L90.0

L90.1

L90.2

L90.3

For peer review only - http://bmjopen.bmj.com/site/about/guidelines.xhtml

L90.4

L90.5

L90.6

L90.8

L90.9

L92.0

L92.1

L92.2

L92.3

L92.8

L92.9

L93.0

L93.1

L93.2

L95.0

L95.1

L95.8

L95.9

L98.5

L99.0

For peer review only - http://bmjopen.bmj.com/site/about/guidelines.xhtml

M01.1

M03.6

M05.0

M05.1

M05.2

M05.3

M05.8

M05.9

M06.0

M06.1

M06.2

M06.3

M06.4

M06.8

M06.9

M07.0

M07.1

M07.2

M07.3

M07.4

For peer review only

For peer review only - http://bmjopen.bmj.com/site/about/guidelines.xhtml

M07.6

M08.0

M08.1

M08.2

M08.3

M08.4

M08.8

M08.9

M09.0

M09.1

M09.2

M09.8

M10.0

M10.1

M10.2

M10.3

M10.4

M10.9

M11.0

M11.1

For peer review only
For peer review only - http://bmjopen.bmj.com/site/about/guidelines.xhtml

M11.2

M11.8

M11.9

M12.0

M12.1

M12.2

M12.3

M12.4

M12.5

M12.8

M13.0

M13.1

M13.8

M13.9

M14.0

M14.2

M14.3

M14.4

M14.5

M14.6

For peer review only

M14.8

M30.0

M30.1

M30.2

M30.3

M30.8

M31.0

M31.1

M31.2

M31.4

M31.5

M31.6

M31.7

M31.8

M31.9

M32.0

M32.8

M32.9

M33.0

M33.1

For peer review only

M33.2

M33.9

M34.0

M34.1

M34.2

M34.8

M34.9

M35.0

M35.1

M35.2

M35.3

M35.4

M35.5

M35.6

M35.7

M35.8

M35.9

M36.0

M36.1

M36.2

For peer review only - http://bmjopen.bmj.com/site/about/guidelines.xhtml

M36.3

M36.4

M40.0

M40.1

M40.2

M40.3

M40.4

M40.5

M41.0

M41.1

M41.2

M41.3

M41.4

M41.5

M41.8

M41.9

M42.0

M42.1

M42.9

M43.0

For peer review only
For peer review only - http://bmjopen.bmj.com/site/about/guidelines.xhtml

M43.1

M43.2

M43.3

M43.4

M43.5

M43.6

M43.8

M43.9

M45

M46.0

M46.1

M46.2

M46.3

M46.4

M46.5

M46.8

M46.9

M47.0

M47.1

M47.2

For peer review only

For peer review only - http://bmjopen.bmj.com/site/about/guidelines.xhtml

M47.8

M47.9

M48.0

M48.1

M48.2

M48.3

M48.4

M48.5

M48.8

M48.9

M49.0

M49.5

M50.0

M50.1

M50.2

M50.3

M50.8

M50.9

M51.0

M51.1

For peer review only

For peer review only - http://bmjopen.bmj.com/site/about/guidelines.xhtml

M51.2

M51.3

M51.4

M51.8

M51.9

M53.0

M53.1

M53.2

M53.3

M53.8

M53.9

M54.0

M54.1

M54.2

M54.3

M54.4

M54.5

M54.6

M54.8

M54.9

For peer review only - http://bmjopen.bmj.com/site/about/guidelines.xhtml

M60.0

M60.1

M60.2

M60.8

M60.9

M61.0

M61.1

M61.2

M61.3

M61.4

M61.5

M61.9

M62.0

M62.1

M62.2

M62.3

M62.4

M62.5

M62.6

M62.8

329

For peer review only

M62.9

M63.8

M80.1

M80.2

M80.3

M80.4

M80.5

M80.8

M80.9

M81.1

M81.2

M81.3

M81.4

M81.5

M81.6

M81.8

M81.9

M82.0

M82.1

M82.8

For peer review only

For peer review only - http://bmjopen.bmj.com/site/about/guidelines.xhtml

M84.0

M84.1

M84.2

M84.8

M84.9

M85.0

M85.1

M85.2

M85.3

M85.4

M85.5

M85.6

M85.8

M85.9

M86.3

M86.4

M86.5

M86.6

M89.0

M89.1

For peer review only

For peer review only - http://bmjopen.bmj.com/site/about/guidelines.xhtml

M89.2

M89.3

M89.4

M89.6

M89.8

M89.9

M90.0

M90.4

M90.6

M90.7

M90.8

M91.0

M91.1

M91.2

M91.3

M91.8

M91.9

M92.0

M92.1

M92.2

For peer review only

For peer review only - http://bmjopen.bmj.com/site/about/guidelines.xhtml

M92.3

M92.4

M92.5

M92.6

M92.7

M92.8

M92.9

M93.0

M93.1

M93.2

M93.8

M93.9

M94.0

M94.1

M94.2

M94.3

M94.8

M94.9

N00

N01

For peer review only

For peer review only - http://bmjopen.bmj.com/site/about/guidelines.xhtml

N02

N03

N04

N05

N07

N08.1

N08.2

N08.3

N08.4

N08.5

N08.8

N11.0

N11.1

N11.8

N11.9

N12

N13.0

N13.1

N13.2

N13.3

For peer review only - http://bmjopen.bmj.com/site/about/guidelines.xhtml

N13.4

N13.5

N13.6

N13.7

N13.8

N13.9

N14.0

N14.1

N14.2

N14.3

N14.4

N15.0

N15.1

N15.8

N15.9

N16.0

N16.1

N16.2

N16.3

N16.4

For peer review only

For peer review only - http://bmjopen.bmj.com/site/about/guidelines.xhtml

N16.5

N16.8

N20.0

N20.1

N20.2

N20.9

N21.0

N21.1

N21.8

N21.9

N22.0

N22.8

N23

N25.0

N25.9

N26

N28.0

N28.1

N28.8

N28.9

For peer review only - http://bmjopen.bmj.com/site/about/guidelines.xhtml

N29.0

N29.8

N31.0

N31.1

N31.2

N31.8

N31.9

N32.0

N32.1

N32.2

N32.3

N32.4

N32.8

N32.9

N33.0

N33.8

N35.0

N35.1

N35.8

N35.9

For peer review only

For peer review only - http://bmjopen.bmj.com/site/about/guidelines.xhtml

N36.0

N36.1

N36.2

N36.3

N36.8

N36.9

N39.1

N39.3

N39.4

N40

N42.0

N42.1

N42.2

N42.3

N42.8

N42.9

N70.1

N70.9

N71.1

N71.9

For peer review only - http://bmjopen.bmj.com/site/about/guidelines.xhtml

N72

N73.0

N73.1

N73.2

N73.3

N73.4

N73.5

N73.6

N73.8

N73.9

N74.0

N74.1

N74.2

N74.3

N74.4

N74.8

N80.0

N80.1

N80.2

N80.3

For peer review only - http://bmjopen.bmj.com/site/about/guidelines.xhtml

N80.4

N80.5

N80.6

N80.8

N80.9

N81.0

N81.1

N81.2

N81.3

N81.4

N81.5

N81.6

N81.8

N81.9

N82.0

N82.1

N82.2

N82.3

N82.4

N82.5

For peer review only

N82.8

N82.9

N85.0

N85.1

N85.2

N85.3

N85.4

N85.5

N85.6

N85.7

N85.8

N85.9

N87.0

N87.1

N87.2

N87.9

N88.0

N88.1

N88.2

N88.3

For peer review only

For peer review only - http://bmjopen.bmj.com/site/about/guidelines.xhtml

N88.4

N88.8

N88.9

O35.4

P10.0

P10.2

P10.3

P10.4

P10.8

P10.9

P27.0

P27.1

P27.8

P27.9

P35.2

P35.9

P37.0

P37.1

P52.0

P52.1

For peer review only
For peer review only - http://bmjopen.bmj.com/site/about/guidelines.xhtml

P52.2

P52.3

P52.5

P52.6

P52.8

P52.9

P57.0

P57.8

P57.9

P75

P90

P91.1

P96.0

Q00.1

Q00.1

Q02

Q03.0

Q03.8

Q04.1

Q04.5

For peer review only - http://bmjopen.bmj.com/site/about/guidelines.xhtml

Q04.8

Q06.0

Q06.1

Q06.2

Q06.3

Q06.4

Q06.8

Q06.9

Q07.8

Q07.9

Q10.4

Q10.7

Q11.0

Q11.1

Q11.2

Q11.3

Q12.0

Q12.1

Q12.2

Q12.3

For peer review only

For peer review only - http://bmjopen.bmj.com/site/about/guidelines.xhtml

Q12.4

Q12.8

Q12.9

Q13.0

Q13.1

Q13.2

Q13.3

Q13.4

Q13.8

Q13.9

Q14.0

Q14.1

Q14.2

Q14.3

Q14.8

Q14.9

Q15.0

Q15.8

Q15.9

Q16.0

For peer review only

For peer review only - http://bmjopen.bmj.com/site/about/guidelines.xhtml

Q16.1

Q16.2

Q16.3

Q16.4

Q16.5

Q16.9

Q18.8

Q20.1

Q20.2

Q20.5

Q21.0

Q21.1

Q21.2

Q21.4

Q21.9

Q22.2

Q22.3

Q22.8

Q22.9

Q23.1

For peer review only

For peer review only - http://bmjopen.bmj.com/site/about/guidelines.xhtml

Q23.3

Q23.8

Q24.0

Q24.1

Q24.2

Q24.3

Q24.4

Q24.5

Q24.6

Q24.8

Q24.9

Q25.0

Q25.1

Q25.2

Q25.3

Q25.7

Q25.8

Q25.9

Q26.0

Q26.1

For peer review only
For peer review only - http://bmjopen.bmj.com/site/about/guidelines.xhtml

Q26.3

Q26.5

Q26.9

Q27.0

Q27.1

Q27.2

Q27.3

Q27.4

Q27.8

Q27.9

Q28.0

Q28.1

Q28.3

Q28.9

Q30.0

Q30.1

Q30.2

Q30.3

Q30.8

Q30.9

For peer review only

Q31.0

Q31.1

Q31.2

Q31.3

Q31.5

Q31.8

Q31.9

Q32.0

Q32.2

Q32.3

Q32.4

Q33.0

Q33.1

Q33.2

Q33.3

Q33.4

Q33.5

Q33.8

Q33.9

Q34.0

For peer review only - http://bmjopen.bmj.com/site/about/guidelines.xhtml

Q34.1

Q34.8

Q34.9

Q35.1

Q35.3

Q35.5

Q35.7

Q35.9

Q36.0

Q36.1

Q36.9

Q37.0

Q37.1

Q37.2

Q37.3

Q37.4

Q37.5

Q37.8

Q37.9

Q38.0

For peer review only

Q38.3

Q38.4

Q38.6

Q38.7

Q38.8

Q39.0

Q39.1

Q39.2

Q39.3

Q39.4

Q39.5

Q39.8

Q39.9

Q40.2

Q40.3

Q40.8

Q41.1

Q41.2

Q41.8

Q42.0

For peer review only

For peer review only - http://bmjopen.bmj.com/site/about/guidelines.xhtml

Q42.1

Q42.2

Q42.3

Q42.8

Q42.9

Q43.1

Q43.3

Q43.4

Q43.5

Q43.6

Q43.9

Q44.0

Q44.1

Q44.3

Q44.4

Q44.5

Q44.7

Q45.0

Q45.1

Q45.2

For peer review only

For peer review only - http://bmjopen.bmj.com/site/about/guidelines.xhtml

Q45.3

Q45.8

Q45.9

Q50.0

Q51.0

Q51.1

Q51.2

Q51.3

Q51.4

Q51.5

Q51.6

Q51.7

Q51.8

Q51.9

Q52.1

Q52.2

Q52.4

Q54.0

Q54.1

Q54.2

For peer review only

For peer review only - http://bmjopen.bmj.com/site/about/guidelines.xhtml

Q54.3

Q54.8

Q54.9

Q55.0

Q55.5

Q56.0

Q56.1

Q56.2

Q56.3

Q56.4

Q60.2

Q60.4

Q60.5

Q61.0

Q61.1

Q61.2

Q61.3

Q61.5

Q61.8

Q62.0

354

For peer review only

Q62.1

Q62.3

Q62.4

Q62.5

Q62.6

Q62.8

Q63.0

Q63.1

Q63.2

Q63.8

Q63.9

Q64.0

Q64.1

Q64.3

Q64.4

Q64.5

Q64.6

Q64.7

Q64.8

Q64.9

For peer review only

For peer review only - http://bmjopen.bmj.com/site/about/guidelines.xhtml

Q65.0

Q65.1

Q65.2

Q65.8

Q65.9

Q67.5

Q68.2

Q71.0

Q71.1

Q71.2

Q71.3

Q71.4

Q71.5

Q71.6

Q71.8

Q71.9

Q72.0

Q72.1

Q72.2

Q72.3

For peer review only - http://bmjopen.bmj.com/site/about/guidelines.xhtml

Q72.4

Q72.5

Q72.6

Q72.7

Q72.8

Q72.9

Q73.0

Q73.1

Q73.8

Q74.0

Q74.1

Q74.2

Q74.8

Q74.9

Q75.1

Q75.3

Q75.4

Q75.5

Q75.8

Q75.9

For peer review only - http://bmjopen.bmj.com/site/about/guidelines.xhtml

Q76.1

Q76.2

Q76.3

Q76.4

Q77.0

Q77.1

Q77.5

Q77.6

Q77.7

Q77.8

Q77.9

Q78.0

Q78.1

Q78.2

Q78.3

Q78.4

Q78.6

Q78.8

Q79.0

Q79.4

For peer review only

For peer review only - http://bmjopen.bmj.com/site/about/guidelines.xhtml

Q79.5

Q79.6

Q79.8

Q80.0

Q80.1

Q80.2

Q80.3

Q80.8

Q80.9

Q82.0

Q82.2

Q82.3

Q82.9

Q85.0

Q85.9

Q85.0

Q86.1

Q86.2

Q86.8

Q87.3

For peer review only
For peer review only - http://bmjopen.bmj.com/site/about/guidelines.xhtml

Q87.4

Q87.5

Q89.1

Q89.2

Q89.3

Q89.4

Q89.7

Q89.8

Q89.9

Q90.0

Q90.1

Q90.2

Q90.9

Q92.2

Q92.3

Q92.5

Q92.6

Q92.9

Q93.0

Q93.1

For peer review only

Q93.6

Q93.7

Q93.9

Q97.0

Q97.1

Q97.2

Q97.3

Q97.8

Q97.9

Q98.0

Q99.0

Q99.1

Q99.2

Q99.8

Q99.9

For peer review only

For peer review only - http://bmjopen.bmj.com/site/about/guidelines.xhtml

STROBE Statement—Checklist of items that should be included in reports of ***cohort studies***

	Item No	Recommendation	Page No
**Title and abstract**	1	(*a*) Indicate the study's design with a commonly used term in the title or the abstract<br>(*b*) Provide in the abstract an informative and balanced summary of what was done and what was found	1
**Introduction**			
Background/rationale	2	Explain the scientific background and rationale for the investigation being reported	3-4
Objectives	3	State specific objectives, including any prespecified hypotheses	4
**Methods**			
Study design	4	Present key elements of study design early in the paper	5 + abstract + Title
Setting	5	Describe the setting, locations, and relevant dates, including periods of recruitment, exposure, follow-up, and data collection	5
Participants	6	(*a*) Give the eligibility criteria, and the sources and methods of selection of participants. Describe methods of follow-up<br>(*b*) For matched studies, give matching criteria and number of exposed and unexposed	5-7 + Table 1
Variables	7	Clearly define all outcomes, exposures, predictors, potential confounders, and effect modifiers. Give diagnostic criteria, if applicable	8 -9
Data sources/ measurement	8*	For each variable of interest, give sources of data and details of methods of assessment (measurement). Describe comparability of assessment methods if there is more than one group	8 -9
Bias	9	Describe any efforts to address potential sources of bias	9-10
Study size	10	Explain how the study size was arrived at	5,10
Quantitative variables	11	Explain how quantitative variables were handled in the analyses. If applicable, describe which groupings were chosen and why	9-10
Statistical methods	12	(*a*) Describe all statistical methods, including those used to control for confounding<br>(*b*) Describe any methods used to examine subgroups and interactions<br>(*c*) Explain how missing data were addressed<br>(*d*) If applicable, explain how loss to follow-up was addressed<br>(*e*) Describe any sensitivity analyses	9-10
**Results**			
Participants	13*	(a) Report numbers of individuals at each stage of study—eg numbers potentially eligible, examined for eligibility, confirmed eligible, included in the study, completing follow-up, and analysed<br>(b) Give reasons for non-participation at each stage<br>(c) Consider use of a flow diagram	
Descriptive data	14*	(a) Give characteristics of study participants (eg demographic, clinical, social) and information on exposures and potential confounders<br>(b) Indicate number of participants with missing data for each variable of interest<br>(c) Summarise follow-up time (eg, average and total amount)	
Outcome data	15*	Report numbers of outcome events or summary measures over time	

For peer review only

For peer review only

For peer review only - http://bmjopen.bmj.com/site/about/guidelines.xhtml

Main results	16	(*a*) Give unadjusted estimates and, if applicable, confounder-adjusted estimates and their precision (eg, 95% confidence interval). Make clear which confounders were adjusted for and why they were included<br>(*b*) Report category boundaries when continuous variables were categorized<br>(*c*) If relevant, consider translating estimates of relative risk into absolute risk for a meaningful time period	
Other analyses	17	Report other analyses done—eg analyses of subgroups and interactions, and sensitivity analyses	
**Discussion**			
Key results	18	Summarise key results with reference to study objectives	
Limitations	19	Discuss limitations of the study, taking into account sources of potential bias or imprecision. Discuss both direction and magnitude of any potential bias	
Interpretation	20	Give a cautious overall interpretation of results considering objectives, limitations, multiplicity of analyses, results from similar studies, and other relevant evidence	
Generalisability	21	Discuss the generalisability (external validity) of the study results	
**Other information**			
Funding	22	Give the source of funding and the role of the funders for the present study and, if applicable, for the original study on which the present article is based	

*Give information separately for exposed and unexposed groups.

Note: An Explanation and Elaboration article discusses each checklist item and gives methodological background and published examples of transparent reporting. The STROBE checklist is best used in conjunction with this article (freely available on the Web sites of PLoS Medicine at http://www.plosmedicine.org/, Annals of Internal Medicine at http://www.annals.org/, and Epidemiology at http://www.epidem.com/). Information on the STROBE Initiative is available at http://www.strobe-statement.org.

For peer review only

BMJ Open

The health of mothers of children with a life-limiting condition; a protocol for comparative cohort study using the Clinical Practice Research Datalink

Journal:	*BMJ Open*
Manuscript ID	bmjopen-2019-034024.R1
Article Type:	Protocol
Date Submitted by the Author:	04-Mar-2020

Complete List of Authors:	Fraser, Lorna; University of York,<br>Murtagh, Fliss; Wolfson Palliative Care Research Centre, Hull York Medical School, University of Hull, UK,<br>Sheldon, Trevor; University of York, health Sciences<br>Gilbody, Simon; The University of York, Department of Health Sciences<br>Hewitt, Catherine; The University of York, Health Sciences

**Primary Subject Heading**:	General practice / Family practice
Secondary Subject Heading:	Paediatrics
Keywords:	Developmental neurology & neurodisability < PAEDIATRICS, Life-limiting, maternal health

SCHOLARONE™
Manuscripts

I, the Submitting Author has the right to grant and does grant on behalf of all authors of the Work (as defined in the below author licence), an exclusive licence and/or a non-exclusive licence for contributions from authors who are: i) UK Crown employees; ii) where BMJ has agreed a CC-BY licence shall apply, and/or iii) in accordance with the terms applicable for US Federal Government officers or employees acting as part of their official duties; on a worldwide, perpetual, irrevocable, royalty-free basis to BMJ Publishing Group Ltd ("BMJ") its licensees and where the relevant Journal is co-owned by BMJ to the co-owners of the Journal, to publish the Work in this journal and any other BMJ products and to exploit all rights, as set out in our licence.

The Submitting Author accepts and understands that any supply made under these terms is made by BMJ to the Submitting Author unless you are acting as an employee on behalf of your employer or a postgraduate student of an affiliated institution which is paying any applicable article publishing charge ("APC") for Open Access articles. Where the Submitting Author wishes to make the Work available on an Open Access basis (and intends to pay the relevant APC), the terms of reuse of such Open Access shall be governed by a Creative Commons licence – details of these licences and which Creative Commons licence will apply to this Work are set out in our licence referred to above.

Other than as permitted in any relevant BMJ Author's Self Archiving Policies, I confirm this Work has not been accepted for publication elsewhere, is not being considered for publication elsewhere and does not duplicate material already published. I confirm all authors consent to publication of this Work and authorise the granting of this licence.

For peer review only

TITLE: The health of mothers of children with a life-limiting condition; a protocol for comparative cohort study using the **Clinical Practice Research Datalink**

Fraser LK[1,2], Murtagh FEM[3], Sheldon TA[1], Gilbody S[1], Hewitt CE[1]

[1] Department of Health Sciences, University of York, UK

[2]Martin House Research Centre, University of York, UK

[3] Wolfson Palliative Care Centre, Hull York Medical School, Hull, UK

Corresponding Author:

Dr Lorna Fraser

Department of Health Sciences

Area 2 Seebohm Rowntree Building

Heslington

YORK

YO10 5DD

lorna.fraser@york.ac.uk

01904 321889

Word Count: 2489 words.

Tables :1

For peer review only

For peer review only - http://bmjopen.bmj.com/site/about/guidelines.xhtml

ABSTRACT

Introduction

There are now nearly 50,000 children with a life-limiting or life-threatening conditions in the UK. These include conditions where there is no reasonable hope of cure and from which they will die, as well as conditions for which curative treatment may be feasible but can fail, e.g. cancer or heart failure. Having a child with a life-limiting condition involves being a coordinator and provider of healthcare in addition to the responsibilities and pressures of parenting a child who is expected to die young. This adversely affects the health and wellbeing of these mothers and affects their ability to care for their child, but the extent of the impact is poorly understood.

This study aims to quantify the incidence and nature of mental and physical morbidity in mothers of children with a life-limiting condition, their healthcare use and to assess whether there is a relationship between the health of the mother and the child's condition.

Methods and analysis

A comparative cohort study using data from the Clinical Practice Research Datalink (CPRD) and linked hospital data will include three groups of children and their mothers (those with a life-limiting condition, those with a chronic conditions and those with no long term health condition total =20,000 mother-child dyads). Incidence rates and incidence rate ratios will be used to quantify and compare the outcomes between groups with multivariable regression modelling used to assess the relationship between the child's disease trajectory and mother's health.

Ethics and dissemination

This study protocol has approval from the Independent Scientific Advisory Committee for the UK Medicines and Healthcare products Regulatory Agency Database Research. The results of this study will be reported according to the STROBE and RECORD guidelines. There will also

For peer review only - http://bmjopen.bmj.com/site/about/guidelines.xhtml

be a lay summary for parents which will be available to download from the Martin House Research Centre website (www.york.ac.uk/mhrc).

For peer review only

For peer review only - http://bmjopen.bmj.com/site/about/guidelines.xhtml

ARTICLE SUMMARY

Strengths and Limitations of this study

This study is utilising a large, longitudinal, nationally representative primary care data source.

The Clinical Practice Research Datalink data enables the identification of mother and child dyads and linkage to secondary care data.

This study is reliant on identification of the cohort of interest by diagnostic coding therefore on the quality of diagnostic coding within the CPRD and HES datasets.

INTRODUCTION

The number of children with life-limiting or life-threatening conditions has been rising, with latest figures estimating 49,000 children and young people with a life-limiting condition in the UK.[1] These include conditions for which there is no reasonable hope of cure and from which children or young people will die, as well as conditions for which curative treatment may be feasible but can fail, such as cancer or heart failure. In children and young people, more than 300 diagnoses are life-limiting or life-threatening[2] , including duchenne muscular dystrophy, severe cerebral palsy, neurodegenerative conditions, and severe congenital anomalies.

Although many of the individual diagnoses are rare, collectively life-limiting conditions affect more children and young people than more common single long term conditions, such as diabetes mellitus[3] . Many of these children are living longer due to the use of medical technologies, e.g. ventilation and gastrostomy feeding, and more aggressive treatment of complications and they are often high users of healthcare services[4] . The parents of these children have an added layer of 'pressure' from having a child with a chronic condition who also has a shortened life expectancy[5] . Having a child with a life-limiting condition often involves being a coordinator and provider of healthcare as well as being a parent, and this responsibility exists 24 hours a day, 7 days a week, especially for mothers. The health of these mothers is important, both for their own well-being, and also in terms of their ability to care

For peer review only - http://bmjopen.bmj.com/site/about/guidelines.xhtml

for their child. Currently the National Health Service and statutory services for these children rarely extend to providing care for the parents. The focus is on mothers rather than fathers in this study as mothers are the main carer for the majority of these children.

The need for further research on the emotional and psychological support and interventions for parents or carers of children with a life-limiting condition was highlighted recently in National Institute for Health and Clinical Excellence (NICE) guidelines on end of life care in children and young people[6] . This stated that research was needed on 'What emotional support do children and young people with life-limiting conditions and their parents or carers need, and how would they like these needs to be addressed? The NICE guidelines[6] also noted that no studies had quantified the psychological/ mental health of mothers of children with life-limiting condition. There have been some attempts to quantify these in children with special needs[7] or specific disabilities[8 9] which have shown higher levels of parental distress or emotional problems than parents of healthy children (36% cf 20%). These however, do not address the specific needs of those with life-limiting conditions or the added burden that parents of children with a life-limiting condition face, knowing their child is likely to die.

Evidence about the physical health of mothers with life-limiting condition is also sparse, although two studies in mothers of children with disabilities found higher prevalence of physical conditions compared to mothers of healthy children. For example, prevalence of back pain and hypertension was 35.2% and 24.7% respectively in mothers of children with disabilities, compared to 26.7% and 19.1% in mothers of healthy children. However, these studies were cross sectional, self-reported and were not explicitly for mothers of children with a life-limiting condition [9 10]. To date, no study using a nationally representative sample has assessed the incidence or prevalence of mental and physical health conditions in mothers of children with a life-limiting condition. Quantifying the extent and nature of both physical and mental health conditions in these mothers is important when trying to develop and target appropriate preventative or treatment interventions for this population.

This study aims to quantify the incidence and nature of mental and physical morbidity in mothers of children with a life-limiting condition, their healthcare use and to assess whether there is a relationship between the health of the mother and the child's condition.

For peer review only - http://bmjopen.bmj.com/site/about/guidelines.xhtml

METHODS AND ANALYSIS

Research Questions

1. What is the nature and incidence of mental and physical morbidity in mothers of children with a life-limiting condition?

2. What is the relationship between the health of the mother and the child's condition?

3. How many primary care visits do these mothers have compared to other mothers?

4. How many hospital admissions do these mothers have compared to other mothers?

5. What are the resource costs of healthcare for these mothers?

Data sources and sample selection

This will be an observational comparative cohort study design using data from the Clinical Practice Research Datalink (CPRD). The CPRD dataset contains anonymized, longitudinal records of primary care from a representative sample of GP practices across the UK (covering approximately 8.5% of the UK population)[11]. Individuals within the primary care CPRD dataset are also linked to secondary care data (inpatient, outpatient, A & E Hospital Episodes Statistics (HES) and the Mental Health Minimum Dataset (MHMDS))[12 13] and Office for National Statistics death certificate data. The CPRDprovides a unique opportunity to assess the relationship between maternal morbidity and their child health due to the longitudinal nature of data collection (since 1987) and the ability to link mothers and children via their mother-baby link algorithm[14].

The index children will be extracted from the CPRD mother baby linkage, where the mother has at least 1 years registration and where they eligible for HES linkage (resident in England). Children will be grouped in to those with a life-limiting condition (n~5000) or chronic condition that is not life-limiting e.g. diabetes, asthma (n~5000) and they will be identified within the CPRD dataset between 2007-2017, using READ codes in the CPRD data and ICD10 codes in the HES data[1] (see Table 1) . The development of these ICD-10 code list have been

For peer review only - http://bmjopen.bmj.com/site/about/guidelines.xhtml

described previously for life-limiting[1] and chronic conditions[15]. Subjects will be matched by year of birth, sex and region to the control group of healthy children (n~10,000) with no long term conditions.

All primary and the linked secondary care data will be extracted for each mother and child dyad.

Table 1 Cohort identification processes

Groups	Inclusion criteria	Exclusion Criteria
1. Life-limiting or life-threatening condition	• Children from the source population who have prevalent diagnosis event records for life-limiting conditions, either in the Clinical or Referral files in CPRD GOLD based on the Read codes detailed in Supplementary material, or in HES APC based on the ICD-10 codes in Supplementary material.<br>• Children have above events before the end of the study period (31/12/2017).<br>• Children have above events within their UTS follow-up period.<br>• Children are aged 18 years or less on the diagnosis event date.	None
2. Chronic condition that is not life-limiting	One matching control will be provided for each case in Group 1. The controls will comprise of patients from the source population who fulfil the following criteria:<br>• Children from the source population who have diagnosis event records for other chronic conditions in the Clinical or Referral files in CPRD GOLD based	•Children with a diagnosis on or before 31/12/2017 of life-limiting conditions, either in the Clinical or Referral files in CPRD GOLD based on the Read codes detailed in Appendix 1, or in HES APC based on the ICD-10 codes in Appendix 2.

For peer review only

	on the Read codes detailed in Supplementary material, or in HES APC based on the ICD-10 codes in Supplementary material.<br>• Children have above events before the end of the study period of (31/12/2017).<br>• Children have above events within their UTS follow-up period.<br>• Children are aged 18 years or less on the diagnosis date.	• Children who have deregistered from the CPRD on or before 01/04/2007<br>• Children who are siblings of children in the case population (Group 1), based on having a link to the same mother in the Mother Baby Link
3. No long term conditions	Up to two matching controls will be provided for each case in Group 1. The controls will comprise of patients from the source population who fulfil the following criteria:<br>• Children from the source population who have at least one day of registration during follow-up start and end.	• Children with a diagnosis of life-limiting conditions on or before 31/12/2017, either in the Clinical or Referral files in CPRD GOLD based on the Read codes detailed in Appendix 1, or in HES APC based on the ICD-10 codes in Appendix 2, will be excluded<br>• Children with a diagnosis of other chronic conditions on or before 31/12/2017, in the Clinical or Referral files in CPRD GOLD based on the Read codes detailed in Appendix 3, or in HES APC based on the ICD-10 codes in Appendix 4, will be excluded.<br>• Children who have deregistered from the CPRD on or before 01/04/2007<br>• Children who are siblings of children in the case population (Group 1), based on

For peer review only

		having a link to the same mother in the Mother Baby Link

Sample size

A sample size calculation based on 80% power, 5% significance to detect a 5% difference in prevalence in maternal anxiety/depression between the groups (from 15-20% prevalence) indicates a minimum of 1,131 participants required in each group. Given the heterogeneity in the life-limiting condition group, and to facilitate analyses within life-limiting condition subgroups, we will aim to have 5,000 participants in each of the life-limiting condition and chronic disease groups, with 10,000 in the control (no long-term condition) group. The total sample size will be 40,000 (20,000 children and 20,000 mothers).

Exposures

The key exposure is the disease status of the child (life-limiting condition/chronic disease (asthma or diabetes)/ no long-term condition). These groups will be identified using previously developed READ and ICD 10 diagnostic code lists (see supplementary material). The use of both READ and ICD 10 codes will enable identification of relevant diagnoses from either the primary care records (READ) or hospital admission data (ICD 10).

Outcomes

The outcomes for the mothers will be recorded in either the primary care data or the linked secondary care datasets. The dates of these outcomes will be assessed in relation to the date of the child's diagnoses.

Incidence of mental health and physical health conditions

- Maternal mental health diagnoses, including both common mental illness and severe mental illness. These will be identified using diagnostic READ codes and relevant

For peer review only - http://bmjopen.bmj.com/site/about/guidelines.xhtml

prescription data. A previously developed READ code algorithm[16] will be utilised. The linked MHMDS will indicate the presence of a more serious mental health conditions.

- Maternal physical diagnoses, including e.g. obesity, hypertension, musculoskeletal problems, and cardiovascular disease. These will be identified using diagnostic READ codes, relevant prescription data, HES data, and related biometric data (including blood pressure, BMI, and total cholesterol).

The association of maternal health with the child's disease trajectory

- The number of A&E attendances, hospital admissions and length of stay for the children (HES data).

Healthcare use and resources of the mother

- Referrals to other services, especially secondary services for mothers (CPRD and HES data).
- Uptake of cervical screening by mothers (CPRD data).
- The number of primary care attendances (GP, practice nurse etc.) per year by mothers (CPRD data)
- The number and nature of prescribed medication for mothers (Prescription data).
- The number of A&E attendances, hospital admissions and length of stay for mothers (HES data).

Confounding

The following clinical and demographic confounders will be considered in the analyses:

- Ethnicity, this will be classified according to the census 2011 categories[17] using data from CPRD and HES data.
- Pre-existing comorbidities of the mother
- Child's diagnoses: grouped into disease categories[1]
- Socioeconomic data is based on the index of multiple deprivation category based on the local superoutput area of residence[18]

For peer review only

- Region of residence.

Statistical methods

All analyses will be undertaken using STATA version 15[19]. All analyses will adjust for known clinical and demographic confounders of importance (age, co-morbidities, socioeconomic status, previous use of primary care services, etc.). The left and right censored nature of these data will be taken into account when undertaking these analyses.

Incidence of mental health and physical health conditions

Incidence rates of the physical and mental health conditions will be calculated in each group of mothers by dividing the number of cases in each group by the person-time at risk in each group. Incident cases will be counted only after the diagnoses in the child. A comparison with the incidence of these conditions in the three groups of mothers will be undertaken using incidence rate ratios.

The association of maternal health with the child's disease trajectory

The presence of physical or mental health diagnoses in the mother will be the outcome variable of interest in a multivariable model with a key covariate being the presence of prolonged or repeated hospital admission for the child. A prolonged admission is one that lasts > 14 days and repeated admissions will be defined as more than 2 admissions in a 6 month time period.

Healthcare use and resources of the mother

Using multivariable Poisson regression modelling for the count of GP visits, referrals and hospital admissions with the key exposure of disease status of the child (life-limiting condition /Chronic disease/ no long-term condition). Multilevel modelling will be used to account for repeated measures within individuals. For the analyses of healthcare costs, we will use information from Unit Costs of Health and Social Care[20] , the NHS national tariffs[21] and Department of Health Reference Costs[22] .

For peer review only

Missing data

We expect there to be missing data for some of the key covariates in the data e.g. ethnicity. We will assess the level of missingness across all sources of these variable (HES and CPRD). If appropriate then we will use multiple imputation techniques [23] to address this in order to avoid the potential bias from only undertaking complete case analyses.

PATIENT AND PUBLIC INVOLVEMENT

The views of parents and carers of children with a life-limiting condition have informed the development of this study. Initial ideas about the topic area and planned study were discussed with a group of 10 parents of children with a life-limiting condition at Martin House Children's Hospice. They helped to refine the research question and future stages of this programme of work. The Family Advisory Board of the Martin House Research Centre (www.york.ac.uk/mhrc) will have involvement in this study with an emphasis on the preparation of the lay summaries and dissemination to parents and children.

ETHICS AND DISSEMINATION

This study protocol has approval from the Independent Scientific Advisory Committee for the UK Medicines and Healthcare products Regulatory Agency Database Research (protocol 18_313). They have NREC approval for release of pseudonymised data for observational research.

This study will be complete by Feb 2021.

The results of this study will be reported according to the STROBE and RECORD guidelines (ref). There were also be a lay summary for parents which will be available to download from the Martin House Research Centre website (www.york.ac.uk/mhrc).

For peer review only

For peer review only - http://bmjopen.bmj.com/site/about/guidelines.xhtml

Authors' contributions: LF had the original idea for this study and wrote the first draft of this manuscript. FM, SG, TS and CH contributed to the development of this idea, study design and revised the manuscript. All authors approved the final submitted version of this manuscript.

Funding statement: This paper is independent research arising from a Career Development Fellowship held by Lorna Fraser (CDF-2018-11-ST2-002) supported by the National Institute for Health Research. The views expressed in this publication are those of the author(s) and not necessarily those of the NHS, the National Institute for Health Research or the Department of Health.

Competing interests statement: none declared

Ethics approval: This study protocol has approval from the Independent Scientific Advisory Committee for the UK Medicines and Healthcare products Regulatory Agency Database Research (protocol 18_313).

Data sharing statement: the clinical codes used for this study are provided as supplementary material.

For peer review only

For peer review only - http://bmjopen.bmj.com/site/about/guidelines.xhtml

REFERENCES

1. Fraser LK, Miller M, Hain R, et al. Rising National Prevalence of Life-Limiting Conditions in Children in England. *Pediatrics* 2012;129(4):E923-E29. doi: 10.1542/peds.2011-2846

2. Hain R, Devins M, Hastings R, et al. Paediatric palliative care: development and pilot study of a 'Directory' of life-limiting conditions. *Bmc Palliative Care* 2013;12 doi: 10.1186/1472-684x-12-43

3. Royal College of Paediatrics and Child Health. National Paediatric Diabetes Audit, 2014.

4. Feudtner C, DiGiuseppe DL, Neff JM. Hospital care for children and young adults with complex chronic conditions in the last year of life. *Pediatric Research* 2003;53(4):266A-66A.

5. Fraser LK, Jarvis SW, Moran N, et al. Children in Scotland requiring Palliative Care: identifying numbers and needs (The ChiSP Study). York, UK: University of York, 2015.

6. National Institute for Health and Crae Excellence (NICE). End of life care for infants, children and young people with life-limiting conditions: planning and management, 2016.

7. Thurston S, Paul L, Loney P, et al. Associations and costs of parental symptoms of psychiatric distress in a multi-diagnosis group of children with special needs. *Journal of Intellectual Disability Research* 2011;55:263-80. doi: 10.1111/j.1365-2788.2010.01356.x

8. Brehaut JC, Kohen DE, Raina P, et al. The Health of Primary Caregivers of Children With Cerebral Palsy: How Does It Compare With That of Other Canadian Caregivers? *Pediatrics* 2004;114(2):e182-e91. doi: 10.1542/peds.114.2.e182

9. Lach LM, Kohen DE, Garner RE, et al. The health and psychosocial functioning of caregivers of children with neurodevelopmental disorders. *Disability and Rehabilitation* 2009;31(8):607-18. doi: 10.1080/09638280802242163

10. Lee MH, Park C, Matthews AK, et al. Differences in physical health, and health behaviors between family caregivers of children with and without disabilities. *Disability and Health Journal* 2017;10(4):565-70. doi: 10.1016/j.dhjo.2017.03.007

11. Herrett E, Gallagher AM, Bhaskaran K, et al. Data Resource Profile: Clinical Practice Research Datalink (CPRD). *International Journal of Epidemiology* 2015;44(3):827-36. doi: 10.1093/ije/dyv098

12. Clinical Practice Research Datalink. Hospital Episode Statistics (HES) Admitted Patient Care and GOLD Documentation (Set 14): Medicines & Healthcare products Regulatory Agency, 2017.

For peer review only

For peer review only - http://bmjopen.bmj.com/site/about/guidelines.xhtml

13. Health & Social Care Information Centre. Methodology for creation of the HES Patient ID (HESID), 2014.

14. CPRD. Clinical Practice Research Datalink 2014 [Available from: http://www.cprd.com/intro.asp accessed 29/01/2014.

15. Hardelid P, Dattani N, Gilbert R. Estimating the prevalence of chronic conditions in children who die in England, Scotland and Wales: a data linkage cohort study. *BMJ Open* 2014;4(8):e005331. doi: 10.1136/bmjopen-2014-005331

16. Prady SL, Pickett KE, Petherick ES, et al. Evaluation of ethnic disparities in detection of depression and anxiety in primary care during the maternal period: combined analysis of routine and cohort data. *British Journal of Psychiatry* 2016;208(5):453-61. doi: 10.1192/bjp.bp.114.158832

17. NOMIS. Census 2011 - Ethnic group by sex by age: NOMIS, 2013.

18. Government DfCaL. The English Indices of Deprivation 2015, 2015.

19. StataCorp. Stata Statistical Software: Release 14. College Station, TX: StataCorp LP, 2017.

20. Personal Social Services Research Unit. Unit Costs of Health and Social Care 2014, 2014.

21. Department of Health. Payment by Results Guidance for 2013-14, 2013.

22. Department of health. Reference costs 2013-14, 2014.

23. Sterne JAC, White IR, Carlin JB, et al. Multiple imputation for missing data in epidemiological and clinical research: potential and pitfalls. *BMJ* 2009;338:b2393. doi: 10.1136/bmj.b2393

For peer review only - http://bmjopen.bmj.com/site/about/guidelines.xhtml

Supplementary Material

Appendix 1 Read Codes to identify a child with a Life-limiting condition

medcode	clinicalevents	referralevents	testevents	immunisationevents	readcode	readterm	databasebuild
241	217428	9975	65	0	G30..00	Acute myocardial infarction	Feb-09
245	7800	168	4	0	G410.00	Primary pulmonary hypertension	Feb-09
318	411	23	0	0	B210.00	Malignant neoplasm of glottis	Feb-09
319	4215	149	0	0	B21..00	Malignant neoplasm of larynx	Feb-09
348	54604	2944	8	0	B34..11	Ca female breast	Feb-09
512	61437	4071	14	0	K05..00	Chronic renal failure	Feb-09
579	9051	803	14	0	BBE1.00	[M]Malignant melanoma NOS	Feb-09
684	86980	7892	6	0	F20..00	Multiple sclerosis	Feb-09
765	3287	381	1	0	BBe1.00	[M]Neurofibromatosis NOS	Feb-09
780	123877	3483	21	0	B46..00	Malignant neoplasm of prostate	Feb-09
865	30087	2172	10	0	B32..00	Malignant melanoma of skin	Feb-09
1056	8129	525	4	0	B5z..00	Malignant neoplasm of other and unspecified site NOS	Feb-09
1062	16003	663	2	0	B10..00	Malignant neoplasm of oesophagus	Feb-09
1204	4721	258	1	0	G30..14	Heart attack	Feb-09
1220	35966	1430	2	0	B13..00	Malignant neoplasm of colon	Feb-09
1391	312	6	0	0	C327100	Gaucher's disease	Feb-09
1481	48	1	0	0	B600.00	Reticulosarcoma	Feb-09
1483	12690	675	2	0	BBg1.11	[M]Lymphoma NOS	Feb-09
1599	4047	172	1	0	B4A0.00	Malignant neoplasm of kidney parenchyma	Feb-09
1624	38351	2463	10	0	BB2A.00	[M]Squamous cell carcinoma NOS	Feb-09
1800	21118	946	2	0	B141.00	Malignant neoplasm of rectum	Feb-09
1950	9157	203	0	0	BB4..00	[M]Transitional cell papillomas and carcinomas	Feb-09
1952	166	3	0	0	B580.00	Secondary malignant neoplasm of kidney	Feb-09
1986	11636	589	3	0	B440.11	Cancer of ovary	Feb-09
2123	944	54	1	0	BBc1.00	[M]Neuroblastoma NOS	Feb-09

For peer review only - http://bmjopen.bmj.com/site/about/guidelines.xhtml

2272	28720	722	23	0	BB5..11	[M]Adenocarcinomas	Feb-09
2298	2427	120	0	0	F203.00	Exacerbation of multiple sclerosis	Feb-09
2462	10288	433	13	0	B61..00	Hodgkin's disease	Feb-09
2492	9864	1312	66	0	B33z.00	Malignant neoplasm of skin NOS	Feb-09
2587	35784	1469	5	0	B22z.11	Lung cancer	Feb-09
2744	3591	129	2	0	B40..00	"Malignant neoplasm of uterus, part unspecified"	Feb-09
2747	7108	210	1	0	B41..00	Malignant neoplasm of cervix uteri	Feb-09
2755	19616	1231	106	0	B....11	Cancers	Feb-09
2815	6116	113	1	0	B133.00	Malignant neoplasm of sigmoid colon	Feb-09
2816	833	34	0	0	P51..00	Transposition of great vessels	Feb-09
2835	6829	291	5393	0	43C3.11	HIV positive	Feb-09
2890	7205	174	2	0	B430200	Malignant neoplasm of endometrium of corpus uteri	Feb-09
2961	1999	106	0	0	B47z.11	Seminoma of testis	Feb-09
3152	13083	265	0	0	BB13.00	"[M]Carcinoma, metastatic, NOS"	Feb-09
3197	22344	1073	2	0	BB03.00	"[M]Neoplasm, metastatic"	Feb-09
3204	20442	926	6	0	G55..00	Cardiomyopathy	Feb-09
3213	294	19	0	0	B430.00	"Malignant neoplasm of corpus uteri, excluding isthmus"	Feb-09
3230	4027	195	0	0	B41..11	Cervical carcinoma (uterus)	Feb-09
3357	1876	129	0	0	B1...11	Carcinoma of digestive organs and peritoneum	Feb-09
3371	3101	115	0	0	BBg2.11	[M]Non Hodgkins lymphoma	Feb-09
3541	536	56	0	0	B48..00	Malignant neoplasm of penis and other male genital organs	Feb-09
3591	4247	308	0	0	F134.00	Huntington's chorea	Feb-09
3604	14600	525	0	0	B627.00	Non - Hodgkin's lymphoma	Feb-09
3710	407	34	0	0	BBB1.00	[M]Adenolymphoma	Feb-09
3811	6639	194	0	0	B134.00	Malignant neoplasm of caecum	Feb-09
3862	244	16	0	0	P601.00	Congenital atresia of the pulmonary valve	Feb-09

For peer review only - http://bmjopen.bmj.com/site/about/guidelines.xhtml

For peer review only

3903	34821	1333	3	0	B22z.00	Malignant neoplasm of bronchus or lung NOS	Feb-09
3947	6445	288	10	0	P1...00	Spina bifida	Feb-09
3968	128623	3421	8	0	B34..00	Malignant neoplasm of female breast	Feb-09
3969	21	0	0	0	BB9M.00	[M]Intracystic carcinoma NOS	Feb-09
4072	544	43	0	0	B680.00	Acute leukaemia NOS	Feb-09
4118	360	14	0	0	BBV9.00	[M]Myxoid chondrosarcoma	Feb-09
4137	5072	154	1	0	B570.00	Secondary malignant neoplasm of lung	Feb-09
4158	1619	149	0	0	PG51.00	Osteogenesis imperfecta	Feb-09
4165	641	70	0	0	F140.00	Friedreich's ataxia	Feb-09
4217	770	26	0	0	BB5c.00	[M]Parathyroid adenomas and adenocarcinomas	Feb-09
4218	48	0	0	0	B541.00	Malignant neoplasm of parathyroid gland	Feb-09
4222	2158	123	0	0	B64..11	Lymphatic leukaemia	Feb-09
4250	3215	381	0	0	B68z.00	Leukaemia NOS	Feb-09
4251	2752	114	0	0	B640.00	Acute lymphoid leukaemia	Feb-09
4272	983	58	0	0	PH3y200	Epidermolysis bullosa	Feb-09
4388	897	21	0	0	B020.00	Malignant neoplasm of parotid gland	Feb-09
4403	13552	429	0	0	B577.11	Liver metastases	Feb-09
4413	6444	194	0	0	B650.00	Acute myeloid leukaemia	Feb-09
4473	590	13	0	0	BBY0.00	[M]Ewing's sarcoma	Feb-09
4478	1446	87	0	0	F256.00	Infantile spasms	Feb-09
4479	1238	93	0	0	PK5..00	Tuberous sclerosis	Feb-09
4554	1713	44	0	0	B454.00	Malignant neoplasm of vulva unspecified	Feb-09
4632	14309	947	1	0	B33..00	Other malignant neoplasm of skin	Feb-09
4637	722	14	0	0	BBr..00	[M]Leukaemias	Feb-09
4673	P20..00	254	0	8	0	Encephalocele	Feb-09
4796	F152.00	10361	0	689	0	Motor neurone disease	Feb-09
4852	642	90	0	0	BB24.12	[M]Verrucous squamous cell carcinoma	Feb-09
4865	7627	274	1	0	B10z.11	Oesophageal cancer	Feb-09
4944	14705	788	3	0	B630.00	Multiple myeloma	Feb-09
5052	250	13	0	0	BBV1.13	[M]Osteogenic sarcoma NOS	Feb-09

For peer review only - http://bmjopen.bmj.com/site/about/guidelines.xhtml

5136	80	10	1	0	B911013	Choriocarcinoma	Feb-09
5137	302	81	1	0	B624.11	Leukaemic reticuloendotheliosis	Feb-09
5179	232	11	1	0	B620.00	Nodular lymphoma (Brill - Symmers disease)	Feb-09
5198	3093	125	0	0	B583000	Secondary malignant neoplasm of brain	Feb-09
5199	5722	87	0	0	B583200	Cerebral metastasis	Feb-09
5306	235	15	0	0	P100000	"Spina bifida with hydrocephalus, unspecified"	Feb-09
5393	672	51	0	0	F391000	Duchenne muscular dystrophy	Feb-09
5395	2491	73	0	0	A540.00	Eczema herpeticum - Kaposi's varicelliform eruption	Feb-09
5431	P11..00	87	0	1	0	Spina bifida without mention of hydrocephalus	Feb-09
5443	305	6	0	0	F122.00	Malignant neuroleptic syndrome	Feb-09
5455	2673	45	0	0	BB53.00	"[M]Adenocarcinoma, metastatic, NOS"	Feb-09
5637	5356	129	2	0	B53..00	Malignant neoplasm of thyroid gland	Feb-09
5712	2991	84	0	0	P21..00	Microcephalus	Feb-09
5842	6910	159	0	0	B58..00	Secondary malignant neoplasm of other specified sites	Feb-09
5901	9334	321	0	0	B141.12	Rectal carcinoma	Feb-09
5915	536	12	0	0	BBrA400	[M]Hairy cell leukaemia	Feb-09
5932	493	19	0	0	BB06.00	"[M]Tumour cells, uncertain whether benign or malignant"	Feb-09
5964	1625	125	2	0	F39B.00	Muscular dystrophy	Feb-09
6170	10811	117	0	0	B590.11	Carcinomatosis	Feb-09
6220	13732	778	263	0	C370.00	Cystic fibrosis	Feb-09
6316	245	4	0	0	BBr0100	[M]Acute leukaemia NOS	Feb-09
6436	3624	40	0	0	BB43.00	[M]Transitional cell carcinoma NOS	Feb-09
6471	1998	50	0	0	B57..11	Metastases of respiratory and/or digestive systems	Feb-09
6515	P211.00	231	0	2	0	Micrencephaly	Feb-09
6712	3881	65	1	0	K050.00	End stage renal failure	Feb-09

For peer review only - http://bmjopen.bmj.com/site/about/guidelines.xhtml

6746	2004	25	1	0	BB5M.00	[M]Tubular adenomas and adenocarcinomas	Feb-09
6806	187	6	0	0	B12..00	Malignant neoplasm of small intestine and duodenum	Feb-09
6883	951	15	0	0	Q317000	Perinatal bronchopulmonary dysplasia	Feb-09
6920	640	22	0	0	BB5U.00	[M]Villous adenomas and adenocarcinomas	Feb-09
6935	745	5	0	0	B131.00	Malignant neoplasm of transverse colon	Feb-09
6966	267	1	0	0	BB1D.00	[M]Spindle cell carcinoma	Feb-09
7046	2915	13	0	0	B43..00	Malignant neoplasm of body of uterus	Feb-09
7176	2417	106	0	0	B65..00	Myeloid leukaemia	Feb-09
7219	2385	46	0	0	B141.11	Carcinoma of rectum	Feb-09
7319	240	0	0	0	BB9G.00	[M]Infiltrating ductular carcinoma	Feb-09
7476	942	17	0	0	BBQ1.00	[M]Seminomas	Feb-09
7484	4152	107	0	0	B226.00	Mesothelioma	Feb-09
7535	3403	77	0	0	G554400	Primary dilated cardiomyopathy	Feb-09
7593	3116	266	0	0	H51y700	Malignant pleural effusion	Feb-09
7654	7216	308	2	0	B585.00	Secondary malignant neoplasm of bone and bone marrow	Feb-09
7740	236	10	0	0	B470200	Seminoma of undescended testis	Feb-09
7805	9705	177	0	0	B440.00	Malignant neoplasm of ovary	Feb-09
7830	2203	37	0	0	B56..11	Lymph node metastases	Feb-09
7856	362	15	0	0	BBJH.00	[M]Dedifferentiated liposarcoma	Feb-09
7940	453	8	0	0	ByuDF11	[X]Non-Hodgkin's lymphoma NOS	Feb-09
7941	183	2	0	0	BBW4.00	[M]Chondrosarcoma NOS	Feb-09
7945	134	0	0	0	F256000	Hypsarrhythmia	Feb-09
7982	224	2	0	0	B161200	Malignant neoplasm of common bile duct	Feb-09
8010	3501	202	0	0	G551.00	Hypertrophic obstructive cardiomyopathy	Feb-09
8032	1365	37	0	0	BB5B.00	[M]Pancreatic adenomas and carcinomas	Feb-09
8085	2979	102	0	0	BBF1.00	[M]Sarcoma NOS	Feb-09
8088	18	1	0	0	BBG3.00	[M]Fibromyxosarcoma	Feb-09
8101	2854	120	0	0	BB5a.00	[M]Renal adenoma and carcinoma	Feb-09

For peer review only - http://bmjopen.bmj.com/site/about/guidelines.xhtml

8154	800	49	0	0	B576200	Malignant ascites	Feb-09
8166	14157	485	1	0	B17..00	Malignant neoplasm of pancreas	Feb-09
8267	29	1	0	0	F363.00	Refsum's disease	Feb-09
8281	11	0	0	0	A789A00	HIV disease resulting in wasting syndrome	Feb-09
8328	326	7	0	0	BBbC.00	"[M]Astrocytoma, anaplastic type"	Feb-09
8351	907	12	0	0	BB91.00	[M]Infiltrating duct carcinoma	Feb-09
8386	10022	372	1	0	B11..00	Malignant neoplasm of stomach	Feb-09
8523	3902	212	0	0	BBb0.11	[M]Glioma NOS	Feb-09
8547	3113	135	1	0	BBbB.00	[M]Astrocytoma NOS	Feb-09
8550	98	2	0	0	B542000	Malignant neoplasm of pituitary gland	Feb-09
8600	2847	117	0	0	1D18.00	Pain from metastases	Feb-09
8606	2207	45	0	0	BB5j.00	[M]Endometrioid adenomas and carcinomas	Feb-09
8625	16007	1249	4	0	B641.00	Chronic lymphoid leukaemia	Feb-09
8627	492	5	1	0	BB07.00	"[M]Tumour cells, malignant"	Feb-09
8649	455	11	0	0	ByuDF00	"[X]Non-Hodgkin's lymphoma, unspecified type"	Feb-09
8660	139	2	0	0	BBV1.00	[M]Osteosarcoma NOS	Feb-09
8695	6720	163	2	0	BB12.00	[M]Carcinoma NOS	Feb-09
8711	3485	112	3	0	BB5D100	[M]Cholangiocarcinoma	Feb-09
8771	1081	5	0	0	B170.00	Malignant neoplasm of head of pancreas	Feb-09
8918	229	4	0	0	B15..00	Malignant neoplasm of liver and intrahepatic bile ducts	Feb-09
8930	4784	25	0	0	BB52.00	[M]Adenocarcinoma NOS	Feb-09
9011	368	9	0	0	P543.00	Eisenmenger's complex	Feb-09
9030	179	4	0	0	B55..00	Malignant neoplasm of other and ill-defined sites	Feb-09
9088	480	10	1	0	B130.00	Malignant neoplasm of hepatic flexure of colon	Feb-09
9118	6778	234	0	0	B13z.11	Colonic cancer	Feb-09
9156	201	4	0	0	BB1K.00	[M]Oat cell carcinoma	Feb-09
9179	703	69	0	0	F151.00	Spinal muscular atrophy	Feb-09

For peer review only - http://bmjopen.bmj.com/site/about/guidelines.xhtml

9237	578	4	0	0	B21z.00	Malignant neoplasm of larynx NOS	Feb-09
9271	2219	87	0	0	F240.00	Quadriplegia	Feb-09
9291	2384	46	0	0	BB1J.00	[M]Small cell carcinoma NOS	Feb-09
9366	297	7	0	0	BB13.11	[M]Secondary carcinoma	Feb-09
9402	734	15	0	0	G55y.11	Secondary dilated cardiomyopathy	Feb-09
9444	142	7	0	0	ZV10400	[V]Personal history of malignant neoplasm of genital organ	Feb-09
9447	969	3	0	0	BB5j200	[M]Endometrioid carcinoma	Feb-09
9470	10661	57	3	0	B34z.00	Malignant neoplasm of female breast NOS	Feb-09
9476	729	18	0	0	B471100	Teratoma of descended testis	Feb-09
9491	632	23	0	0	B142.11	Anal carcinoma	Feb-09
9505	71	2	0	0	B582600	Secondary malignant neoplasm of skin of breast	Feb-09
9575	3391	54	1	0	BBbL.11	[M]Glioblastoma multiforme	Feb-09
9600	1246	24	0	0	B232.00	Mesothelioma of pleura	Feb-09
9618	801	46	0	0	B56..00	Secondary and unspecified malignant neoplasm of lymph nodes	Feb-09
9622	41	4	0	0	B525.00	Malignant neoplasm of cauda equina	Feb-09
9712	820	9	0	0	BB4A.00	[M]Papillary transitional cell carcinoma	Feb-09
9722	P114.00	268	0	3	0	Meningomyelocele	Feb-09
9859	261	1	0	0	BBQ1z00	[M]Seminoma NOS	Feb-09
9902	1265	48	0	0	B3...11	"Carcinoma of bone, connective tissue, skin and breast"	Feb-09
9984	168	6	0	0	B00..11	Carcinoma of lip	Feb-09
10166	1986	62	8	0	C307000	Hyperglycinaemia	Feb-09
10283	3195	152	0	0	B01..00	Malignant neoplasm of tongue	Feb-09
10358	1844	16	0	0	B222.00	"Malignant neoplasm of upper lobe, bronchus or lung"	Feb-09
10368	972	21	0	0	B11..11	Gastric neoplasm	Feb-09
10439	120	7	0	0	ZV76.00	[V]Screening for malignant neoplasm	Feb-09
10541	264	2	0	0	BB22.00	[M]Papillary carcinoma NOS	Feb-09

For peer review only

10588	1685	50	0	0	BBK0200	[M]Leiomyosarcoma NOS	Feb-09
10628	882	38	0	0	PJyy200	Fragile X chromosome	Feb-09
10668	2130	21	0	0	BB5a000	[M]Renal cell carcinoma	Feb-09
10698	76	2	0	0	B450100	Malignant neoplasm of vaginal vault	Feb-09
10726	3296	148	0	0	B651.00	Chronic myeloid leukaemia	Feb-09
10851	779	26	0	0	B51..11	Cerebral tumour - malignant	Feb-09
10864	565	6	0	0	B132.00	Malignant neoplasm of descending colon	Feb-09
10913	72	0	0	0	BBD0.00	[M]Paraganglioma NOS	Feb-09
10946	1278	22	0	0	B136.00	Malignant neoplasm of ascending colon	Feb-09
10949	433	11	0	0	B162.00	Malignant neoplasm of ampulla of Vater	Feb-09
10955	407	17	0	0	C391100	Di George syndrome	Feb-09
10956	487	23	0	0	PKy9300	Prader - Willi syndrome	Feb-09
10995	2057	31	0	0	B5...00	Malignant neoplasm of other and unspecified sites	Feb-09
11035	475	4	0	0	B593.00	Primary malignant neoplasm of unknown site	Feb-09
11513	297	15	0	0	PH30.00	Congenital ectodermal dysplasia	Feb-09
11531	431	15	0	0	P00..00	Anencephalus	Feb-09
11628	5160	168	0	0	B1z0.11	Cancer of bowel	Feb-09
11773	3006	40	0	0	7L1A.11	Dialysis for renal failure	Feb-09
11991	58	0	0	0	B454.11	Primary vulval cancer	Feb-09
12067	227	20	0	0	B927.11	Von Recklinghausen's disease	Feb-09
12106	966	14	0	0	ZV10.00	[V]Personal history of malignant neoplasm	Feb-09
12146	17	4	0	0	BBr2000	[M]Lymphoid leukaemia NOS	Feb-09
12204	220	10	0	0	C354A00	Metastatic calcification	Feb-09
12309	524	15	0	0	BBb..00	[M]Gliomas	Feb-09
12323	1428	3	0	0	B6...00	Malignant neoplasm of lymphatic and haemopoietic tissue	Feb-09
12335	6535	469	2	0	B62y.00	Malignant lymphoma NOS	Feb-09
12388	249	3	0	0	BB43.11	[M]Urothelial carcinoma	Feb-09
12389	514	9	0	0	B4A1.00	Malignant neoplasm of renal pelvis	Feb-09

For peer review only - http://bmjopen.bmj.com/site/about/guidelines.xhtml

12427	293	8	0	0	BB9F.00	[M]Lobular carcinoma NOS	Feb-09
12464	142	1	0	0	B62x200	Peripheral T-cell lymphoma	Feb-09
12480	321	39	0	0	BB9K000	[M]Paget's disease and intraductal carcinoma of breast	Feb-09
12490	74	2	0	0	B550200	Malignant neoplasm of nose NOS	Feb-09
12497	143	0	0	0	BB82100	[M]Mucinous adenocarcinoma	Feb-09
12499	1207	3	0	0	Byu6.00	[X]Malignant neoplasm of breast	Feb-09
12539	1040	61	0	0	B3...12	Sarcoma of bone and connective tissue	Feb-09
12580	173	0	0	0	BBB0.00	[M]Adenosquamous carcinoma	Feb-09
12582	1332	11	0	0	B224100	Malignant neoplasm of lower lobe of lung	Feb-09
12609	220	3	0	0	BB19.00	"[M]Carcinoma, anaplastic type, NOS"	Feb-09
12870	2717	45	0	0	B221.00	Malignant neoplasm of main bronchus	Feb-09
13243	10271	325	1	0	B22..00	"Malignant neoplasm of trachea, bronchus and lung"	Feb-09
13252	1710	16	0	0	B4...00	Malignant neoplasm of genitourinary organ	Feb-09
13569	20097	639	1	0	B590.00	Disseminated malignancy NOS	Feb-09
13574	10	0	0	0	BB36.00	[M]Metatypical carcinoma	Feb-09
13575	32	1	31	0	4F32.00	Ascitic fluid: malignant cells	Feb-09
13581	149	0	0	0	R210.00	[D]Sudden infant death syndrome	Feb-09
14242	159	13	240	0	42D4.00	RBC's - sickle cells present	Feb-09
14712	565	42	0	0	B00..00	Malignant neoplasm of lip	Feb-09
14792	674	42	0	0	B05..00	Malignant neoplasm of other and unspecified parts of mouth	Feb-09
14800	5086	263	1	0	B11z.00	Malignant neoplasm of stomach NOS	Feb-09
15027	844	45	0	0	B62yz00	Malignant lymphoma NOS	Feb-09
15036	49	2	0	0	B626.00	Malignant mast cell tumours	Feb-09
15103	4814	199	3	0	B577.00	Secondary malignant neoplasm of liver	Feb-09
15148	2504	66	1	0	B47..00	Malignant neoplasm of testis	Feb-09
15182	411	35	6	0	B31z.00	"Malignant neoplasm of connective and soft tissue, site NOS"	Feb-09
15221	187	8	0	0	B220.00	Malignant neoplasm of trachea	Feb-09

For peer review only - http://bmjopen.bmj.com/site/about/guidelines.xhtml

15223	633	16	0	0	B4A2.00	Malignant neoplasm of ureter	Feb-09
15343	1275	95	1	0	PG52.00	Osteopetrosis	Feb-09
15422	2316	216	3	0	D20..00	Aplastic anaemia	Feb-09
15504	150	5	0	0	B62y800	Malignant lymphoma NOS of lymph nodes of multiple sites	Feb-09
15543	104	9	0	0	BB01.00	"[M]Neoplasm, uncertain whether benign or malignant"	Feb-09
15644	173	6	0	0	B4A3.00	Malignant neoplasm of urethra	Feb-09
15658	87	10	0	0	D201.00	Acquired aplastic anaemia	Feb-09
15684	33	4	1	0	B204.00	Malignant neoplasm of frontal sinus	Feb-09
15709	1697	725	0	0	B1...00	Malignant neoplasm of digestive organs and peritoneum	Feb-09
15711	137	2	0	0	B510.00	Malignant neoplasm cerebrum (excluding lobes and ventricles)	Feb-09
15868	95	12	0	0	B335z00	"Malignant neoplasm of skin of trunk, excluding scrotum, NOS"	Feb-09
15907	224	20	5	0	B16z.00	Malignant neoplasm gallbladder/extrahepatic bile ducts NOS	Feb-09
15976	240	19	0	0	B552.00	Malignant neoplasm of abdomen	Feb-09
15989	1174	80	0	0	B47z.12	Teratoma of testis	Feb-09
15991	424	15	0	0	B506.00	Malignant neoplasm of choroid	Feb-09
16038	69	1	0	0	C303300	Maple syrup urine disease	Feb-09
16075	394	22	0	0	B30z.00	Malignant neoplasm of bone and articular cartilage NOS	Feb-09
16087	423	12	0	0	PKy4.00	William syndrome	Feb-09
16105	842	12	0	0	B160.00	Malignant neoplasm of gallbladder	Feb-09
16117	1296	88	0	0	F240.11	Tetraplegia	Feb-09
16118	F392000	1938	0	149	1	Dystrophia myotonica (Steinert's disease)	Feb-09
16126	357	12	0	0	B150000	Primary carcinoma of liver	Feb-09
16146	42	4	0	0	BBB2.00	[M]Adenocarcinoma with squamous metaplasia	Feb-09

For peer review only - http://bmjopen.bmj.com/site/about/guidelines.xhtml

16202	652	24	0	0	B333400	Malignant neoplasm of skin of nose (external)	Feb-09
16213	341	33	0	0	B572.00	Secondary malignant neoplasm of pleura	Feb-09
16241	1580	43	0	0	B060.00	Malignant neoplasm of tonsil	Feb-09
16280	158	1	0	0	B550400	Malignant neoplasm of neck NOS	Feb-09
16297	654	30	0	0	B0z0.00	Malignant neoplasm of pharynx unspecified	Feb-09
16298	48	0	0	0	B18z.00	Malignant neoplasm of retroperitoneum and peritoneum NOS	Feb-09
16416	400	56	1	0	B681.00	Chronic leukaemia NOS	Feb-09
16443	45	3	0	0	C375211	Hunter's syndrome	Feb-09
16460	554	30	0	0	BBg2.00	"[M]Malignant lymphoma, non Hodgkin's type"	Feb-09
16500	809	32	0	0	B58z.00	Secondary malignant neoplasm of other specified site NOS	Feb-09
16677	143	3	0	0	BB9B.00	[M]Medullary carcinoma NOS	Feb-09
16692	1029	9	0	0	BB14.00	[M]Carcinomatosis	Feb-09
16704	73	1	0	0	B302.00	Malignant neoplasm of vertebral column	Feb-09
16711	519	34	0	0	PKy7B00	Stickler syndrome	Feb-09
16723	678	27	0	0	BB5S212	[M]Bronchiolar carcinoma	Feb-09
16760	371	4	0	0	B58y000	Secondary malignant neoplasm of breast	Feb-09
16774	412	9	0	0	BBmD.00	[M] Cutaneous lymphoma	Feb-09
16854	2691	189	0	0	B927.00	Neurofibromatosis - Von Recklinghausen's disease	Feb-09
16915	96	1	0	0	B151.00	Malignant neoplasm of intrahepatic bile ducts	Feb-09
16967	32	0	0	0	B432.00	Malignant neoplasm of overlapping lesion of corpus uteri	Feb-09
17151	1215	68	0	0	BB81.11	[M]Ovarian cystadenoma or carcinoma	Feb-09
17178	525	12	0	0	BBg..00	"[M]Lymphomas, NOS or diffuse"	Feb-09
17182	1343	8	0	0	B627C11	Follicular lymphoma NOS	Feb-09
17212	143	1	0	0	BBLH.00	[M]Rhabdoid sarcoma	Feb-09

For peer review only - http://bmjopen.bmj.com/site/about/guidelines.xhtml

17314	758	81	0	0	BBL7112	[M]Wilms' tumour	Feb-09
17366	296	24	0	0	BBF..00	[M]Soft tissue tumours and sarcomas NOS	Feb-09
17391	142	4	0	0	B221000	Malignant neoplasm of carina of bronchus	Feb-09
17460	93	3	0	0	B627700	Diffuse non-Hodgkin's lymphoblastic (diffuse) lymphoma	Feb-09
17468	289	5	0	0	BBQ..00	[M]Germ cell neoplasms	Feb-09
17475	128	6	0	0	B300A00	Malignant neoplasm of maxilla	Feb-09
17559	550	22	0	0	B1z0.00	"Malignant neoplasm of intestinal tract, part unspecified"	Feb-09
17841	147	0	0	0	B481.00	Malignant neoplasm of glans penis	Feb-09
17874	184	7	0	0	B181.00	Mesothelioma of peritoneum	Feb-09
17887	982	16	0	0	B62x.00	Malignant lymphoma otherwise specified	Feb-09
17912	24	10	0	0	B042.00	"Malignant neoplasm, overlapping lesion of floor of mouth"	Feb-09
18245	152	8	0	0	B330.00	Malignant neoplasm of skin of lip	Feb-09
18255	141	19	1	0	BB5L.00	[M]Adenomatous and adenocarcinomatous polyps	Feb-09
18270	1315	40	0	0	7G03K00	Excision malignant skin tumour	Feb-09
18314	255	5	0	0	B30..00	Malignant neoplasm of bone and articular cartilage	Feb-09
18321	20	1	0	0	PG56012	Conradi - Hunermann syndrome	Feb-09
18354	86	2	0	0	B33y.00	Malignant neoplasm of other specified skin sites	Feb-09
18383	603	7	0	0	BBmH.00	[M] Large cell lymphoma	Feb-09
18537	224	8	0	0	P233.11	Dandy - Walker syndrome	Feb-09
18613	383	57	1	0	B120.00	Malignant neoplasm of duodenum	Feb-09
18616	313	0	0	0	B58y.00	Secondary malignant neoplasm of other specified sites	Feb-09
18617	3533	53	0	0	B51..00	Malignant neoplasm of brain	Feb-09

For peer review only - http://bmjopen.bmj.com/site/about/guidelines.xhtml

18618	31	1	0	0	B335300	Malignant neoplasm of skin of abdominal wall	Feb-09
18619	351	2	0	0	B137.00	Malignant neoplasm of splenic flexure of colon	Feb-09
18632	305	2	0	0	B135.00	Malignant neoplasm of appendix	Feb-09
18676	1030	33	0	0	B585000	Pathological fracture due to metastatic bone disease	Feb-09
18678	226	0	0	0	B224000	Malignant neoplasm of lower lobe bronchus	Feb-09
18712	2030	46	0	0	B4A..11	Renal malignant neoplasm	Feb-09
18744	465	6	0	0	BBn0.11	[M]Multiple myeloma	Feb-09
18771	72	0	0	0	BBLJ.00	[M]Clear cell sarcoma of kidney	Feb-09
18882	26	16	1	0	B006.00	Malignant neoplasm of overlapping lesion of lip	Feb-09
18905	8	0	0	0	C370300	Cystic fibrosis with intestinal manifestations	Feb-09
18914	187	13	0	0	C370200	Cystic fibrosis with pulmonary manifestations	Feb-09
19007	218	0	0	0	Q48E.00	Periventricular leucomalacia	Feb-09
19038	32	0	0	0	PJ1z.11	Trisomy 13 NOS	Feb-09
19041	947	23	0	0	BB29.12	[M]Intraepidermal carcinoma NOS	Feb-09
19083	1453	209	0	0	1J04.00	Suspected lymphoma	Feb-09
19091	1049	154	0	0	BB5..00	[M]Adenomas and adenocarcinomas	Feb-09
19140	3	0	0	0	B614800	Hodgkin's nodular sclerosis of lymph nodes of multiple sites	Feb-09
19141	439	7	0	0	B44..00	Malignant neoplasm of ovary and other uterine adnexa	Feb-09
19144	222	10	0	0	Byu4.00	[X]Melanoma and other malignant neoplasms of skin	Feb-09
19162	41	0	0	0	B493.00	Malignant neoplasm of anterior wall of urinary bladder	Feb-09
19226	142	3	0	0	B513.00	Malignant neoplasm of parietal lobe	Feb-09

For peer review only - http://bmjopen.bmj.com/site/about/guidelines.xhtml

19263	417	21	0	0	BB5f.00	[M]Thyroid adenoma and adenocarcinoma	Feb-09
19318	116	3	0	0	B112.00	Malignant neoplasm of pyloric antrum of stomach	Feb-09
19321	25	1	0	0	B311300	Malignant neoplasm of connective and soft tissue of hand	Feb-09
19334	231	3	0	0	BBL9.00	[M]Carcinosarcoma NOS	Feb-09
19372	1294	108	0	0	B64..00	Lymphoid leukaemia	Feb-09
19415	732	23	0	0	B0...00	"Malignant neoplasm of lip, oral cavity and pharynx"	Feb-09
19423	663	14	0	0	B35..00	Malignant neoplasm of male breast	Feb-09
19437	481	12	0	0	B30z000	Osteosarcoma	Feb-09
19444	520	7	0	0	Byu4100	"[X]Malignant melanoma of skin, unspecified"	Feb-09
19475	54	1	0	0	B471.00	Malignant neoplasm of descended testis	Feb-09
19584	413	3	1	0	Q401.00	Congenital cytomegalovirus infection	Feb-09
19657	158	6	0	0	B911000	Malignant hydatidiform mole	Feb-09
19678	996	118	0	0	BB29.13	[M]Intraepithelial squamous cell carcinoma	Feb-09
19692	286	85	0	0	1J02.00	Suspected leukaemia	Feb-09
19731	26	1	0	0	BB5N.11	[M]Adenoma or or adenocarcinoma in polyposis coli	Feb-09
19945	340	41	27	0	B582.00	Secondary malignant neoplasm of skin	Feb-09
19974	42	6	0	0	B660.00	Acute monocytic leukaemia	Feb-09
20092	562	18	0	0	B04..00	Malignant neoplasm of floor of mouth	Feb-09
20151	371	18	9	0	Q400.00	Congenital rubella	Feb-09
20160	382	13	0	0	B50..00	Malignant neoplasm of eye	Feb-09
20166	41	1	0	0	B45z.00	Malignant neoplasm of female genital organ NOS	Feb-09
20292	299	7	0	0	B02..00	Malignant neoplasm of major salivary glands	Feb-09
20437	350	9	0	0	BBk..00	"[M]Lymphomas, nodular or follicular"	Feb-09

For peer review only - http://bmjopen.bmj.com/site/about/guidelines.xhtml

20440	221	9	0	0	B69..00	Myelomonocytic leukaemia	Feb-09
20493	1686	19	0	0	F20z.00	Multiple sclerosis NOS	Feb-09
20635	32	1	0	0	BBr2011	[M]Lymphatic leukaemia	Feb-09
20685	91	1	0	0	B346.00	Malignant neoplasm of axillary tail of female breast	Feb-09
20699	135	1	0	0	7H19500	Closure of gastrochisis	Feb-09
20710	843	30	0	0	BBj..00	[M]Hodgkin's disease	Feb-09
20772	281	7	0	0	P67..00	Hypoplastic left heart syndrome	Feb-09
20807	184	18	0	0	BB26.00	[M]Papillary squamous cell carcinoma	Feb-09
20822	132	3	0	0	Q48y100	Congenital cardiac failure	Feb-09
21217	64	2	0	0	BB1N.00	[M]Small cell-large cell carcinoma	Feb-09
21249	196	2	0	0	F232.00	Congenital quadriplegia	Feb-09
21327	310	7	0	0	B333500	Malignant neoplasm of skin of temple	Feb-09
21330	128	2	0	0	B180.00	Malignant neoplasm of retroperitoneum	Feb-09
21402	404	6	0	0	B602.00	Burkitt's lymphoma	Feb-09
21418	140	6	0	0	PKy6011	Cornelia de Lange syndrome	Feb-09
21447	14	0	0	0	BBV3.00	[M]Fibroblastic osteosarcoma	Feb-09
21463	272	2	0	0	BBgC.11	[M]Lymphocytic lymphoma NOS	Feb-09
21549	739	16	0	0	B627C00	Follicular non-Hodgkin's lymphoma	Feb-09
21590	352	11	0	0	B58y500	Secondary malignant neoplasm of prostate	Feb-09
21609	131	3	0	0	BB18.00	"[M]Carcinoma, undifferentiated type, NOS"	Feb-09
21620	86	0	0	0	B111.00	Malignant neoplasm of pylorus of stomach	Feb-09
21659	269	4	0	0	BB5Bz00	[M]Pancreatic adenoma or carcinoma NOS	Feb-09
21682	24	0	0	0	BBQ7500	"[M]Malignant teratoma, intermediate type"	Feb-09
21698	631	6	0	0	B221z00	Malignant neoplasm of main bronchus NOS	Feb-09
21715	297	5	0	0	Byu5011	[X]Mesothelioma of lung	Feb-09

For peer review only - http://bmjopen.bmj.com/site/about/guidelines.xhtml

21723	55	4	0	0	D201z00	Acquired aplastic anaemia NOS	Feb-09
21732	40	0	0	0	BBH1.00	[M]Myxosarcoma	Feb-09
21741	64	1	0	0	BB5f100	[M]Follicular adenocarcinoma NOS	Feb-09
21769	458	12	0	0	J625.11	[X] Liver failure	Feb-09
21770	836	71	0	0	BBPX.00	"[M]Mesothelioma, unspecified"	Feb-09
21786	1506	23	0	0	B471000	Seminoma of descended testis	Feb-09
21802	238	1	0	0	P1z..00	Spina bifida NOS	Feb-09
21833	854	4	0	0	BB91.11	[M]Duct carcinoma NOS	Feb-09
21847	356	2	0	0	BB5f111	[M]Follicular carcinoma	Feb-09
21852	184	34	0	0	G554200	Familial cardiomyopathy	Feb-09
21868	2570	18	0	0	BB02.00	"[M]Neoplasm, malignant"	Feb-09
21914	262	7	0	0	BB11.11	[M]Intraepithelial carcinoma NOS	Feb-09
22050	507	8	0	0	B691.00	Chronic myelomonocytic leukaemia	Feb-09
22071	8	0	0	0	BBr0111	[M]Blast cell leukaemia	Feb-09
22146	91	0	0	0	B581100	Secondary malignant neoplasm of bladder	Feb-09
22156	208	2	0	0	BB08.00	"[M]Malignant tumour, small cell type"	Feb-09
22158	88	1	0	0	B630000	"Malignant plasma cell neoplasm, extramedullary plasmacytoma"	Feb-09
22163	673	13	0	0	B134.11	Carcinoma of caecum	Feb-09
22174	10	1	0	0	F390z00	Congenital hereditary muscular dystrophy NOS	Feb-09
22187	2226	46	0	0	B150300	Hepatocellular carcinoma	Feb-09
22267	215	19	0	0	BB04.00	"[M]Neoplasm, malig, uncertain whether primary or metastatic"	Feb-09
22290	27	3	0	0	B313.00	Malignant neoplasm of connective and soft tissue of thorax	Feb-09
22441	28	1	0	0	B212.00	Malignant neoplasm of subglottis	Feb-09
22524	318	0	0	0	B58yz00	Secondary malignant neoplasm of other specified site NOS	Feb-09
22561	187	13	0	0	BBV4.00	[M]Telangiectatic osteosarcoma	Feb-09
22650	307	10	0	0	BBT1.11	[M]Angiosarcoma	Feb-09

For peer review only - http://bmjopen.bmj.com/site/about/guidelines.xhtml

22692	124	6	0	0	BBEG000	"[M]Acral lentiginous melanoma, malignant"	Feb-09
22728	168	1	0	0	SP08700	Acute graft-versus-host disease	Feb-09
22851	173	11	1	0	C375.00	Mucopolysaccharidosis	Feb-09
22893	485	8	0	0	B06..00	Malignant neoplasm of oropharynx	Feb-09
22894	150	3	0	0	B110100	Malignant neoplasm of cardio-oesophageal junction of stomach	Feb-09
22993	1078	20	0	0	G55z.00	Cardiomyopathy NOS	Feb-09
23083	888	11	0	0	BBbL.00	[M]Glioblastoma NOS	Feb-09
23320	53	3	0	0	N233011	Arthrogryposis multiplex congenita.	Feb-09
23380	162	19	0	0	B340000	Malignant neoplasm of nipple of female breast	Feb-09
23389	286	14	0	0	B200.00	Malignant neoplasm of nasal cavities	Feb-09
23399	919	7	0	0	B344.00	Malignant neoplasm of upper-outer quadrant of female breast	Feb-09
23415	97	9	0	0	F256100	Salaam attacks	Feb-09
23433	109	0	0	0	B161.00	Malignant neoplasm of extrahepatic bile ducts	Feb-09
23455	74	5	0	0	BBe1.12	[M]Von Recklinghausen's disease	Feb-09
23480	145	16	0	0	B335900	Malignant neoplasm of perianal skin	Feb-09
23566	40	1	0	0	Q490.00	Neonatal cardiac failure	Feb-09
23711	127	5	0	0	BBg1000	"[M]Malignant lymphoma, diffuse NOS"	Feb-09
23730	99	16	0	0	F202.00	Generalised multiple sclerosis	Feb-09
23775	223	20	0	0	J62y.12	Liver failure NOS	Feb-09
23861	57	5	0	0	B551100	Malignant neoplasm of chest wall NOS	Feb-09
23936	36	2	0	0	ZV10411	[V]Personal history of malignant neoplasm of cervix uteri	Feb-09
23945	544	22	0	0	PK80.00	Fetal alcohol syndrome	Feb-09
23951	43	0	0	0	A789200	HIV disease resulting in candidiasis	Feb-09
23960	17	1	0	0	PD03011	Potter's syndrome	Feb-09
24048	13	0	0	0	B180200	Malignant neoplasm of retrocaecal tissue	Feb-09

For peer review only - http://bmjopen.bmj.com/site/about/guidelines.xhtml

24293	1136	14	0	0	BB2B.00	"[M]Squamous cell carcinoma, metastatic NOS"	Feb-09
24301	46	1	0	0	B57..12	Secondary carcinoma of respiratory and/or digestive systems	Feb-09
24312	29	0	0	0	BB62.00	[M]Apocrine adenoma and adenocarcinomas	Feb-09
24370	983	54	1	0	B142.00	Malignant neoplasm of anal canal	Feb-09
24374	478	23	0	0	B0...11	"Carcinoma of lip, oral cavity and pharynx"	Feb-09
24375	693	37	0	0	B339.00	Dermatofibrosarcoma protuberans	Feb-09
24397	140	4	0	0	B061.00	Malignant neoplasm of tonsillar fossa	Feb-09
24437	830	29	0	0	PB5z.12	Short bowel syndrome	Feb-09
24511	89	6	0	0	BB09.00	"[M]Malignant tumour, giant cell type"	Feb-09
24539	55	2	0	0	BBV2.00	[M]Chondroblastic osteosarcoma	Feb-09
24551	49	2	0	0	BBE1.11	[M]Melanocarcinoma	Feb-09
24675	446	5	0	0	B07..00	Malignant neoplasm of nasopharynx	Feb-09
24852	100	0	0	0	B016.00	Malignant neoplasm of lingual tonsil	Feb-09
24924	59	2	0	0	BBD..00	[M]Paragangliomas and glomus tumours	Feb-09
25191	518	39	0	0	B68..00	Leukaemia of unspecified cell type	Feb-09
25245	13	2	0	0	B336400	Malignant neoplasm of skin of finger	Feb-09
25282	P203.11	68	0	3	0	Meningocele - cranial	Feb-09
25304	140	5	0	0	Q402300	Congenital toxoplasmosis	Feb-09
25306	196	6	0	0	PKyz511	Angelman syndrome	Feb-09
25429	37	0	0	0	F390300	Myotubular myopathy	Feb-09
25535	566	20	1	0	B150.00	Primary malignant neoplasm of liver	Feb-09
25597	237	2	0	0	PB61.00	Biliary atresia	Feb-09
25602	13	2	0	0	B326400	Malignant melanoma of finger	Feb-09
25641	149	7	0	0	BB5D513	[M]Liver cell carcinoma	Feb-09
25657	29	0	0	0	A752.00	Pfeiffer's disease	Feb-09
25695	171	3	0	0	SP08800	Chronic graft-versus-host disease	Feb-09
25835	45573	21	1	0	ZV76200	[V]Screening for malignant neoplasm of cervix	Feb-09

For peer review only - http://bmjopen.bmj.com/site/about/guidelines.xhtml

25886	2578	12	0	0	B222100	Malignant neoplasm of upper lobe of lung	Feb-09
25961	165	3	0	0	BB17.00	[M]Large cell carcinoma NOS	Feb-09
26034	125	0	0	0	B591.00	Other malignant neoplasm NOS	Feb-09
26111	237	4	0	0	M162800	Lymphomatoid papulosis	Feb-09
26120	186	6	0	0	BB5V.00	[M]Pituitary adenomas and carcinomas	Feb-09
26134	10	0	0	0	B064000	"Malignant neoplasm of epiglottis, free border"	Feb-09
26165	408	8	0	0	B211.00	Malignant neoplasm of supraglottis	Feb-09
26253	940	8	0	0	BB5R900	[M]Neuroendocrine carcinoma	Feb-09
26393	865	24	0	0	B152.00	Malignant neoplasm of liver unspecified	Feb-09
26413	100	3	0	0	BB1A.00	[M]Pleomorphic carcinoma	Feb-09
26448	4	0	0	0	B060000	Malignant neoplasm of faucial tonsil	Feb-09
26454	13	0	0	0	B45X.00	Malignant neoplasm/overlapping lesion/feml genital organs	Feb-09
26478	260	7	0	0	SN5y000	Malignant hyperpyrexia due to anaesthetic	Feb-09
26813	74	0	0	0	B21y.00	"Malignant neoplasm of larynx, other specified site"	Feb-09
26814	207	4	0	0	BB5D512	"[M]Hepatoma, malignant"	Feb-09
26848	20	1	0	0	BB5S.00	[M]Respiratory tract adenomas and adenocarcinomas	Feb-09
26853	342	14	0	0	B340.00	Malignant neoplasm of nipple and areola of female breast	Feb-09
26858	137	5	0	0	BB5C.00	[M]Gastrinoma and carcinomas	Feb-09
27280	45	4	0	0	PKy0.12	Prader-Willi syndrome	Feb-09
27330	61	7	0	0	B624.00	Leukaemic reticuloendotheliosis	Feb-09
27363	19	0	0	0	BBd2.00	"[M]Meningioma, malignant"	Feb-09
27370	172	5	0	0	B333.00	Malignant neoplasm skin of other and unspecified parts face	Feb-09
27377	494	62	0	0	F152200	Progressive bulbar palsy	Feb-09
27391	311	20	0	0	B576100	Secondary malignant neoplasm of peritoneum	Feb-09

For peer review only

27416	197	8	0	0	B601.00	Lymphosarcoma	Feb-09
27439	335	23	0	0	BBTA.00	[M]Kaposi's sarcoma	Feb-09
27449	48	2	0	0	B554.00	Malignant neoplasm of upper limb NOS	Feb-09
27458	129	18	0	0	B661.00	Chronic monocytic leukaemia	Feb-09
27475	195	0	0	0	13M3.00	Sudden infant death	Feb-09
27483	149	6	0	0	B240.00	Malignant neoplasm of thymus	Feb-09
27509	95	0	0	0	BBP1.00	"[M]Mesothelioma, malignant"	Feb-09
27520	65	0	0	0	B651z00	Chronic myeloid leukaemia NOS	Feb-09
27528	12	0	0	0	B303.00	"Malignant neoplasm of ribs, sternum and clavicle"	Feb-09
27540	46	0	0	0	B4A1000	Malignant neoplasm of renal calyces	Feb-09
27562	5	0	0	0	BBk0.12	[M]Follicular lymphosarcoma NOS	Feb-09
27617	7	0	0	0	B45y000	Malignant neoplasm of overlapping lesion of vulva	Feb-09
27641	215	1	0	0	A789300	HIV disease resulting in Pneumocystis carinii pneumonia	Feb-09
27651	183	2	0	0	B58..11	Secondary carcinoma of other specified sites	Feb-09
27653	149	1	0	0	BBbz.00	[M]Glioma NOS	Feb-09
27664	253	2	0	0	B65y100	Acute promyelocytic leukaemia	Feb-09
27683	112	11	0	0	G558100	Cardiomyopathy in myotonic dystrophy	Feb-09
27686	P203.00	89	0	0	0	Meningocele - cerebral	Feb-09
27715	15	0	0	0	B242.00	Malignant neoplasm of anterior mediastinum	Feb-09
27728	202	3	0	0	BB90.00	"[M]Intraductal carcinoma, noninfiltrating NOS"	Feb-09
27744	969	36	0	0	BBbQ.00	[M]Oligodendroglioma NOS	Feb-09
27748	131	4	0	0	BBbB.11	[M]Astrocytic glioma	Feb-09
27790	865	59	0	0	B641.11	Chronic lymphatic leukaemia	Feb-09
27846	46	0	0	0	BBbF.00	[M]Fibrillary astrocytoma	Feb-09
27849	12	0	0	0	BB5U200	[M]Villous adenocarcinoma	Feb-09
27853	45	0	0	0	A789500	HIV disease resulting in Kaposi's sarcoma	Feb-09

For peer review only - http://bmjopen.bmj.com/site/about/guidelines.xhtml

27855	1087	13	1	0	B140.00	Malignant neoplasm of rectosigmoid junction	Feb-09
27897	267	2	0	0	B143.00	Malignant neoplasm of anus unspecified	Feb-09
27931	66	4	0	0	B33z000	Kaposi's sarcoma of skin	Feb-09
27965	23	1	0	0	BBv2.00	[M]AngiocentricT-cell lymphoma	Feb-09
28003	144	2	0	0	B420.00	Choriocarcinoma	Feb-09
28069	43	2	0	0	B505.00	Malignant neoplasm of retina	Feb-09
28148	204	0	0	0	B540.00	Malignant neoplasm of adrenal gland	Feb-09
28163	2504	28	0	0	B13z.00	Malignant neoplasm of colon NOS	Feb-09
28178	863	71	0	0	BBa0.00	[M]Craniopharyngioma	Feb-09
28210	33	2	0	0	F391300	Other limb-girdle muscular dystrophy	Feb-09
28241	155	0	0	0	B496.00	Malignant neoplasm of ureteric orifice	Feb-09
28272	472	14	0	0	BB57.00	"[M]Adenocarcinoma, intestinal type"	Feb-09
28291	165	19	0	0	BB69.00	[M]Sebaceous adenoma and adenocarcinoma	Feb-09
28311	1295	7	0	0	B41z.00	Malignant neoplasm of cervix uteri NOS	Feb-09
28344	60	1	0	0	BBb3.12	[M]Subependymal astrocytoma NOS	Feb-09
28355	222	4	0	0	7904	Other correction of transposition of great vessels	Feb-09
28388	189	1	0	0	BB5jz00	[M]Endometrioid adenoma or carcinoma NOS	Feb-09
28451	28	0	0	0	B08z.00	Malignant neoplasm of hypopharynx NOS	Feb-09
28556	815	48	0	0	B32z.00	Malignant melanoma of skin NOS	Feb-09
28559	18	0	0	0	B055z00	Malignant neoplasm of palate NOS	Feb-09
28599	484	21	0	0	BBJ1.00	[M]Liposarcoma NOS	Feb-09
28625	72	0	0	0	BB71.00	[M]Mucoepidermoid carcinoma	Feb-09
28628	72	1	0	0	BBJ3.00	"[M]Liposarcoma, well differentiated type"	Feb-09
28639	119	1	0	0	B627000	Follicular non-Hodgkin's small cleaved cell lymphoma	Feb-09
28663	26	3	0	0	PKy9200	Menke's syndrome	Feb-09
28665	42	0	0	0	B07z.00	Malignant neoplasm of nasopharynx NOS	Feb-09

For peer review only - http://bmjopen.bmj.com/site/about/guidelines.xhtml

28727	138	1	0	0	B575000	Secondary malignant neoplasm of colon	Feb-09
28824	95	1	0	0	P240200	Schizencephaly	Feb-09
28836	523	37	0	0	BBc9.00	[M]Retinoblastomas	Feb-09
28881	8	1	0	0	ZV10513	[V]Personal history of malignant neoplasm of kidney	Feb-09
28919	44	2	0	0	B521.00	Malignant neoplasm of cerebral meninges	Feb-09
28941	88	0	0	0	BBQ3.00	[M]Embryonal carcinoma NOS	Feb-09
28955	F392100	319	0	23	0	Myotonia congenita (Thomsen's disease)	Feb-09
29008	51	0	0	0	BB5W111	[M]Hurthle cell adenocarcinoma	Feb-09
29160	30	2	0	0	B313000	Malignant neoplasm of connective and soft tissue of axilla	Feb-09
29178	515	1	0	0	B614.00	"Hodgkin's disease, nodular sclerosis"	Feb-09
29283	11	0	0	0	B2zy.00	Malignant neoplasm of other site of respiratory tract	Feb-09
29284	82	9	0	0	ZV10112	[V]Personal history of malignant neoplasm of lung	Feb-09
29335	117	2	0	0	BBr2700	[M]Adult T-cell leukaemia/lymphoma	Feb-09
29337	80	2	0	0	BBVA.00	[M] Small cell osteosarcoma	Feb-09
29462	1773	8	0	0	B4Az.00	Malignant neoplasm of kidney or urinary organs NOS	Feb-09
29580	16	1	0	0	BBCA.00	[M]Sertoli cell carcinoma	Feb-09
29787	244	19	0	0	BB2C.00	"[M]Squamous cell carcinoma, keratinising type NOS"	Feb-09
29826	266	1	0	0	B342.00	Malignant neoplasm of upper-inner quadrant of female breast	Feb-09
29876	7	0	0	0	B613z00	"Hodgkin's, lymphocytic-histiocytic predominance NOS"	Feb-09
29945	93	0	0	0	BBR4.00	"[M]Malignant teratoma, trophoblastic"	Feb-09
30119	8	0	0	0	C1zy100	Progeria	Feb-09
30165	3	1	0	0	B18y200	Malignant neoplasm of mesorectum	Feb-09

For peer review only - http://bmjopen.bmj.com/site/about/guidelines.xhtml

30189	81	0	0	0	BB91000	[M]Intraductal papillary adenocarcinoma with invasion	Feb-09
30273	381	13	0	0	BBbG.00	[M]Pilocytic astrocytoma	Feb-09
30283	1419	10	0	0	7B2C700	Intravesical install chemotherapeutic agent for malignancy	Feb-09
30322	73	9	0	0	ZV10500	[V]Personal history of malignant neoplasm of urinary organ	Feb-09
30402	77	6	0	0	B050.11	Malignant neoplasm of buccal mucosa	Feb-09
30416	12	0	0	0	BB82111	[M]Colloid adenocarcinoma	Feb-09
30526	47	0	0	0	Byu5100	"[X]Mesothelioma, unspecified"	Feb-09
30537	15	0	0	0	F373.00	Polyneuropathy in malignant disease	Feb-09
30543	120	1	0	0	B335200	Malignant neoplasm of skin of breast	Feb-09
30576	395	6	0	0	B333300	Malignant neoplasm of skin of forehead	Feb-09
30577	80	2	0	0	B336200	Malignant neoplasm of skin of fore-arm	Feb-09
30632	44	1	0	0	B67z.00	Other specified leukaemia NOS	Feb-09
30645	431	7	0	0	B333000	"Malignant neoplasm of skin of cheek, external"	Feb-09
30646	27	0	0	0	B6y..00	Malignant neoplasm lymphatic or haematopoietic tissue OS	Feb-09
30700	2796	53	0	0	B10z.00	Malignant neoplasm of oesophagus NOS	Feb-09
30747	252	0	0	0	B336.00	Malignant neoplasm of skin of upper limb and shoulder	Feb-09
30988	105	1	0	0	BB1M.00	"[M]Small cell carcinoma, intermediate cell"	Feb-09
31004	48	1	0	0	BB2G.00	[M]Adenoid squamous cell carcinoma	Feb-09
31026	189	4	0	0	BBF3.00	[M]Spindle cell sarcoma	Feb-09
31042	379	23	0	0	Eu84200	[X]Rett's syndrome	Feb-09
31090	20	3	0	0	BBGP.00	[M]Pigmented dermatofibrosarcoma protuberans	Feb-09
31102	4275	30	0	0	B49z.00	Malignant neoplasm of urinary bladder NOS	Feb-09
31112	250	12	0	0	P732.00	Pulmonary artery atresia	Feb-09

For peer review only - http://bmjopen.bmj.com/site/about/guidelines.xhtml

31188	771	6	0	0	B224.00	"Malignant neoplasm of lower lobe, bronchus or lung"	Feb-09
31210	128	3	0	0	B150100	Hepatoblastoma of liver	Feb-09
31268	293	4	0	0	B223.00	"Malignant neoplasm of middle lobe, bronchus or lung"	Feb-09
31323	158	15	0	0	BBG1.00	[M]Fibrosarcoma NOS	Feb-09
31324	38	1	0	0	B626800	Mast cell malignancy of lymph nodes of multiple sites	Feb-09
31364	118	6	1	0	B050.00	Malignant neoplasm of cheek mucosa	Feb-09
31393	361	14	0	0	B160.11	Carcinoma gallbladder	Feb-09
31399	74	3	0	0	B555.00	Malignant neoplasm of lower limb NOS	Feb-09
31418	455	35	0	0	N233000	Arthrogryposis	Feb-09
31421	230	18	0	0	BBK3100	[M]Rhabdomyosarcoma NOS	Feb-09
31492	335	5	0	0	BBm9.00	[M] Monocytoid B-cell lymphoma	Feb-09
31537	9	0	0	0	BBj1100	"[M]Hodgkin,s disease, lymphocytic predominance, nodular"	Feb-09
31541	16	3	0	0	C392300	Severe combined immunodefiency with reticular dysgenesis	Feb-09
31546	195	2	0	0	B341.00	Malignant neoplasm of central part of female breast	Feb-09
31573	172	1	0	0	B23..00	Malignant neoplasm of pleura	Feb-09
31574	223	18	0	0	BBb0.00	"[M]Glioma, malignant"	Feb-09
31576	73	1	0	0	B627B00	Other types of follicular non-Hodgkin's lymphoma	Feb-09
31586	110	3	0	0	B64y100	Prolymphocytic leukaemia	Feb-09
31608	45	0	0	0	B43y.00	Malignant neoplasm of other site of uterine body	Feb-09
31609	24	2	0	0	BBC4.00	"[M]Granulosa cell tumour, malignant"	Feb-09
31629	198	16	0	0	BBc6.00	[M]Ganglioglioma	Feb-09
31663	20	5	0	0	2826	O/E - salaam attack	Feb-09
31673	5	0	0	0	BBX1.12	"[M]Osteoclastoma, malignant"	Feb-09

For peer review only - http://bmjopen.bmj.com/site/about/guidelines.xhtml

31700	456	3	0	0	B222000	Malignant neoplasm of upper lobe bronchus	Feb-09
31701	162	8	0	0	B651.11	Chronic granulocytic leukaemia	Feb-09
31726	6	0	0	0	BBgM.00	"[M]Malignant lymphoma, small cleaved cell, diffuse"	Feb-09
31741	2	0	0	0	BBj6200	"[M]Hodgkin,s disease, nodular sclerosis, lymphocytic deplet"	Feb-09
31749	18	0	0	0	BBv0.00	[M]Monocytoid B-cell lymphoma	Feb-09
31750	106	3	0	0	BBr0300	[M]Chronic leukaemia NOS	Feb-09
31755	79	5	0	0	G240.00	Secondary malignant hypertension	Feb-09
31772	149	2	0	0	BBGM.00	[M]Dermatofibrosarcoma NOS	Feb-09
31775	P115.00	31	0	0	1	Myelocele	Feb-09
31794	661	3	0	0	B627W00	Unspecified B-cell non-Hodgkin's lymphoma	Feb-09
31818	33	4	0	0	BBK2.00	[M]Myoma and myosarcoma	Feb-09
32022	346	8	0	0	B110.00	Malignant neoplasm of cardia of stomach	Feb-09
32024	15	0	0	0	B030.00	Malignant neoplasm of upper gum	Feb-09
32174	79	1	0	0	B202.00	Malignant neoplasm of maxillary sinus	Feb-09
32213	2	1	0	0	BB0A.00	"[M]Malignant tumour, fusiform cell type"	Feb-09
32240	7	0	11	0	4M22.00	Lymphoma stage III	Feb-09
32246	27	6	0	0	ZV10111	[V]Personal history of malignant neoplasm of bronchus	Feb-09
32294	1	0	0	0	BB5B500	"[M]Glucagonoma, malignant"	Feb-09
32351	22	1	46	0	44a4.00	Squamous cell carcinoma antigen level	Feb-09
32357	137	7	0	0	BBc0.00	[M]Ganglioneuromatous neoplasms	Feb-09
32362	75	1	0	0	B113.00	Malignant neoplasm of fundus of stomach	Feb-09
32372	46	1	0	0	B302100	Malignant neoplasm of thoracic vertebra	Feb-09
32464	188	17	0	0	C300000	Cystinosis	Feb-09
32472	46	0	0	0	BB9H.00	[M]Inflammatory carcinoma	Feb-09

For peer review only - http://bmjopen.bmj.com/site/about/guidelines.xhtml

32508	53	0	0	0	P738.00	Atresia of pulmonary artery with septal defect	Feb-09
32603	503	3	0	0	PJyy400	Fragile X syndrome	Feb-09
32641	528	15	0	0	BB5RA00	[M]Merkel cell carcinoma	Feb-09
32768	566	6	0	0	B325100	Malignant melanoma of breast	Feb-09
32874	54	0	0	0	C362z11	Acidaemia NOS	Feb-09
32919	169	8	0	0	SN5y011	Malignant hyperthermia due to anaesthesia	Feb-09
32955	100	1	0	0	B41y.00	Malignant neoplasm of other site of cervix	Feb-09
33259	58	11	0	0	ZV76600	[V]Screening for malignant neoplasm of skin	Feb-09
33271	286	35	0	0	B332200	Malignant neoplasm of pinna NEC	Feb-09
33333	168	2	0	0	B62..00	Other malignant neoplasm of lymphoid and histiocytic tissue	Feb-09
33334	35	2	0	0	F150.00	Werdnig - Hoffmann disease	Feb-09
33344	32	0	0	0	B65z.00	Myeloid leukaemia NOS	Feb-09
33353	101	2	0	0	F11y000	Reye's syndrome	Feb-09
33388	15	0	0	0	B071000	Malignant neoplasm of adenoid	Feb-09
33390	63	6	0	0	C375112	Hurler's syndrome	Feb-09
33444	316	4	0	0	B221100	Malignant neoplasm of hilus of lung	Feb-09
33522	69	1	0	0	PKy6300	Smith - Lemli - Opitz syndrome	Feb-09
33617	429	4	0	0	B43z.00	Malignant neoplasm of body of uterus NOS	Feb-09
33636	104	2	0	0	BBQ7200	"[M]Teratoma, malignant, NOS"	Feb-09
33642	209	2	0	0	PJ2..00	Edward's syndrome - trisomy 18	Feb-09
33682	237	4	0	0	B337400	Malignant neoplasm of skin of lower leg	Feb-09
33775	354	3	0	0	BB5J.00	[M]Adenoid cystic carcinoma	Feb-09
33811	17	1	0	0	PG4D.00	Metaphyseal chondrodysplasia	Feb-09
33833	200	2	0	0	B301.00	Malignant neoplasm of mandible	Feb-09
33843	377	4	0	0	B583.00	Secondary malignant neoplasm of brain and spinal cord	Feb-09

For peer review only - http://bmjopen.bmj.com/site/about/guidelines.xhtml

33869	118	0	0	0	BBgR.00	"[M]Malignant lymphoma, large cell, diffuse NOS"	Feb-09
33871	74	4	0	0	B122.00	Malignant neoplasm of ileum	Feb-09
33897	131	6	0	0	BB4z.00	[M]Transitional cell papilloma or carcinoma NOS	Feb-09
33939	199	0	0	0	P21z.00	Microcephalus NOS	Feb-09
33948	84	1	0	0	PJ32.11	Wolff - Hirschorn syndrome	Feb-09
33997	115	1	0	0	B332000	Malignant neoplasm of skin of auricle (ear)	Feb-09
34000	97	1	0	0	BB80100	[M]Cystadenocarcinoma NOS	Feb-09
34012	208	4	0	0	B08..00	Malignant neoplasm of hypopharynx	Feb-09
34015	182	2	0	0	BB5S200	[M]Bronchiolo-alveolar adenocarcinoma	Feb-09
34030	173	3	0	0	BBL0.00	[M]Endometrial stromal sarcoma	Feb-09
34089	6	0	0	0	B62y400	Malignant lymphoma NOS of lymph nodes of axilla and arm	Feb-09
34096	71	2	0	0	BB5b.00	[M]Granular cell carcinoma	Feb-09
34110	100	3	0	0	BB5R100	"[M]Carcinoid tumour, malignant"	Feb-09
34145	5	0	0	0	B58y600	Secondary malignant neoplasm of testis	Feb-09
34161	61	1	0	0	P225.00	Holoprosencephaly	Feb-09
34252	61	1	0	0	BBb0.12	[M]Gliosarcoma	Feb-09
34259	59	2	0	0	B325300	Malignant melanoma of groin	Feb-09
34269	13	0	0	0	BB69100	[M]Sebaceous adenocarcinoma	Feb-09
34352	121	1	0	0	BBgG.12	[M]Lymphoblastic lymphoma NOS	Feb-09
34388	1117	9	0	0	B17z.00	Malignant neoplasm of pancreas NOS	Feb-09
34395	164	8	0	0	BB24.00	[M]Verrucous carcinoma NOS	Feb-09
34409	4	0	0	0	B010000	Malignant neoplasm of base of tongue dorsal surface	Feb-09
34451	489	11	0	0	B31..00	Malignant neoplasm of connective and other soft tissue	Feb-09
34692	24	3	0	0	B68y.00	Other leukaemia of unspecified cell type	Feb-09
34704	11	0	0	0	F390100	Central core disease	Feb-09
34713	88	2	0	0	BBc0000	[M]Ganglioneuroma	Feb-09

For peer review only - http://bmjopen.bmj.com/site/about/guidelines.xhtml

For peer review only

34742	73	2	0	0	B23z.00	Malignant neoplasm of pleura NOS	Feb-09
34754	46	0	0	0	D200100	Fanconi's familial refractory anaemia	Feb-09
34763	488	16	0	0	BBbT.00	[M]Medulloblastoma NOS	Feb-09
34792	262	7	0	0	F250500	Lennox-Gastaut syndrome	Feb-09
34878	2	0	0	0	B308300	Malignant neoplasm of medial cuneiform	Feb-09
34879	1	0	0	0	BB5J.11	[M]Cylindroid adenocarcinoma	Feb-09
34891	32	0	0	0	BBF2.00	[M]Sarcomatosis NOS	Feb-09
34921	69	1	0	0	P22y111	Joubert syndrome	Feb-09
34950	84	1	0	0	P223.11	Lissencephaly	Feb-09
34984	131	7	0	0	BB80.00	[M]Cystadenoma and carcinoma	Feb-09
35034	101	4	0	0	BBGJ.11	[M]Fibroxanthosarcoma	Feb-09
35039	19	1	0	0	B163.00	"Malignant neoplasm, overlapping lesion of biliary tract"	Feb-09
35071	141	0	0	0	BBQB.00	[M]Mixed germ cell tumour	Feb-09
35113	158	1	0	0	Byu9.00	[X]Malignant neoplasm of urinary tract	Feb-09
35180	189	1	0	0	Byu1.00	[X]Malignant neoplasm of digestive organs	Feb-09
35186	35	1	0	0	ByuC.00	"[X]Malignant neoplasm of ill-defined, secondary and unspeci"	Feb-09
35223	10	0	0	0	BBQ1100	[M]Spermatocytic seminoma	Feb-09
35285	139	0	1	0	ByuA.00	"[X]Malignant neoplasm of eye, brain and other parts of cent"	Feb-09
35325	149	0	0	0	Byu2.00	[X]Malignant neoplasm of respiratory and intrathoracic orga	Feb-09
35348	86	1	0	0	BB5T100	[M]Papillary adenocarcinoma NOS	Feb-09
35357	961	13	0	0	B14..00	"Malignant neoplasm of rectum, rectosigmoid junction and anus"	Feb-09
35364	29	0	0	0	B576000	Secondary malignant neoplasm of retroperitoneum	Feb-09
35420	102	2	0	0	PH3y900	Epidermolysis bullosa dystrophica	Feb-09
35457	251	8	0	0	BB35.00	[M]Basosquamous carcinoma	Feb-09
35467	133	1	0	0	BB5az00	[M]Renal adenoma or carcinoma NOS	Feb-09

For peer review only - http://bmjopen.bmj.com/site/about/guidelines.xhtml

35474	34	0	0	0	BB1C.00	[M]Giant cell carcinoma	Feb-09
35535	138	6	0	0	B173.00	Malignant neoplasm of pancreatic duct	Feb-09
35540	434	10	0	0	F240100	Spastic tetraplegia	Feb-09
35649	54	4	1	0	C31y200	Oxalosis	Feb-09
35665	112	5	0	0	PJ1..00	Patau's syndrome - trisomy 13	Feb-09
35697	45	0	0	0	BBr6.00	[M]Myeloid leukaemias	Feb-09
35747	3	0	0	0	BB5j100	"[M]Endometrioid adenoma, borderline malignancy"	Feb-09
35795	6	0	0	0	B174.00	Malignant neoplasm of Islets of Langerhans	Feb-09
35875	39	5	0	0	B66..00	Monocytic leukaemia	Feb-09
35963	160	1	0	0	B492.00	Malignant neoplasm of lateral wall of urinary bladder	Feb-09
35975	146	6	0	0	BB5z.00	[M]Adenoma or adenocarcinoma NOS	Feb-09
35999	12	0	0	0	B582200	Secondary malignant neoplasm of skin of neck	Feb-09
36031	33	3	0	0	BB5D.00	[M]Hepatobiliary tract adenomas and carcinomas	Feb-09
36114	615	53	0	0	BBg1.00	[M]Malignant lymphoma NOS	Feb-09
36147	553	5	0	0	B153.00	Secondary malignant neoplasm of liver	Feb-09
36161	36	3	0	0	B012.00	"Malignant neoplasm of tongue, tip and lateral border"	Feb-09
36221	49	0	0	0	BB5Sz00	[M]Respiratory tract adenoma or adenocarcinoma NOS	Feb-09
36242	81	0	0	0	BBM7.11	"[M]Cystosarcoma phyllodes, benign"	Feb-09
36286	9	0	0	0	BB5Lz00	[M]Adenomatous or adenocarcinomatous polyp NOS	Feb-09
36325	77	1	0	0	B470300	Teratoma of undescended testis	Feb-09
36371	95	1	0	0	B225.00	Malignant neoplasm of overlapping lesion of bronchus & lung	Feb-09
36401	159	2	0	0	B587.00	Secondary malignant neoplasm of adrenal gland	Feb-09

For peer review only - http://bmjopen.bmj.com/site/about/guidelines.xhtml

36433	F152000	133	0	8	0	Amyotrophic lateral sclerosis	Feb-09
36495	76	3	0	0	B161211	Carcinoma common bile duct	Feb-09
36530	85	3	0	0	BB5S211	[M]Alveolar cell carcinoma	Feb-09
36566	16	0	0	0	F390400	Nemaline body disease	Feb-09
36567	47	0	0	0	PKy6100	Cockayne syndrome	Feb-09
36622	97	0	0	0	C370111	Meconium ileus in cystic fibrosis	Feb-09
36693	75	4	0	0	ZV10600	[V]Personal history of leukaemia	Feb-09
36716	61	1	0	0	B04z.00	Malignant neoplasm of floor of mouth NOS	Feb-09
36731	129	3	0	0	B331000	Malignant neoplasm of canthus	Feb-09
36785	217	28	0	0	BBe1.11	[M]Multiple neurofibromatosis	Feb-09
36839	123	3	0	0	PFy1.00	Larsen's syndrome	Feb-09
36870	48	0	0	0	BBL7111	[M]Adenosarcoma	Feb-09
36876	1	0	0	0	BB5V311	[M]Eosinophil carcinoma	Feb-09
36882	137	3	0	0	BB5X.00	[M]Clear cell adenomas and adenocarcinomas	Feb-09
36899	31	2	0	0	B327800	Malignant melanoma of toe	Feb-09
36949	55	0	0	0	B49y.00	Malignant neoplasm of other site of urinary bladder	Feb-09
37006	31	1	0	0	A789000	HIV disease resulting in mycobacterial infection	Feb-09
37014	180	9	0	0	Eu02200	[X]Dementia in Huntington's disease	Feb-09
37016	43	1	0	0	B33..14	Malignant neoplasm of sebaceous gland	Feb-09
37096	6	0	0	0	B015.00	"Malignant neoplasm of tongue, junctional zone"	Feb-09
37112	7	0	0	0	B6...11	Malignant neoplasm of histiocytic tissue	Feb-09
37137	173	3	0	0	BB51100	[M]Adenocarcinoma in situ in tubulovillous adenoma	Feb-09
37165	339	16	0	0	B334000	Malignant neoplasm of scalp	Feb-09
37182	78	3	0	0	B63..00	Multiple myeloma and immunoproliferative neoplasms	Feb-09
37272	129	1	0	0	B67..00	Other specified leukaemia	Feb-09

For peer review only - http://bmjopen.bmj.com/site/about/guidelines.xhtml

37306	255	18	0	0	ZV10415	[V]Personal history of malignant neoplasm of prostate	Feb-09
37328	287	6	0	0	B450.00	Malignant neoplasm of vagina	Feb-09
37354	74	0	0	0	BB5X100	[M]Clear cell adenocarcinoma NOS	Feb-09
37410	61	0	0	0	BBr2100	[M]Acute lymphoid leukaemia	Feb-09
37461	44	0	0	0	B64y200	Adult T-cell leukaemia	Feb-09
37473	31	1	1	0	BBbW.00	[M]Cerebellar sarcoma NOS	Feb-09
37477	48	2	0	0	BBe7.11	"[M]Schwannoma, malignant"	Feb-09
37510	20	0	0	0	BBLG.00	[M]Carcinoma in pleomorphic adenoma	Feb-09
37516	32	1	0	0	B054.00	Malignant neoplasm of uvula	Feb-09
37539	115	8	0	0	D2...00	Aplastic and other anaemias	Feb-09
37542	2	0	0	0	BBQ7300	[M]Teratocarcinoma	Feb-09
37549	8	0	0	0	B05z000	Kaposi's sarcoma of palate	Feb-09
37553	17	1	0	0	B007.00	"Malignant neoplasm of lip, unspecified"	Feb-09
37590	88	4	0	0	B052.00	Malignant neoplasm of hard palate	Feb-09
37618	70	3	0	0	B551000	Malignant neoplasm of axilla NOS	Feb-09
37680	135	0	0	0	BBGF.00	"[M]Fibrous histiocytoma, malignant"	Feb-09
37688	27	0	0	0	BBA2.00	[M]Acinar cell carcinoma	Feb-09
37723	48	1	0	0	BBr6011	[M]Granulocytic leukaemia NOS	Feb-09
37724	58	0	0	0	B056.00	Malignant neoplasm of retromolar area	Feb-09
37793	30	0	89	0	4M4..00	FIGO staging of gynaecological malignancy	Feb-09
37805	22	0	0	0	B213100	Malignant neoplasm of cricoid cartilage	Feb-09
37810	22	0	0	0	B220z00	Malignant neoplasm of trachea NOS	Feb-09
37842	61	2	0	0	B303000	Malignant neoplasm of rib	Feb-09
37859	37	0	0	0	B110z00	Malignant neoplasm of cardia of stomach NOS	Feb-09
37872	376	6	0	0	B327400	Malignant melanoma of lower leg	Feb-09
37916	32	0	0	0	B05y.00	Malignant neoplasm of other specified mouth parts	Feb-09
37940	58	1	0	0	B072000	Malignant neoplasm of pharyngeal recess	Feb-09

For peer review only - http://bmjopen.bmj.com/site/about/guidelines.xhtml

37969	118	3	0	0	B335100	"Malignant neoplasm of skin of chest, excluding breast"	Feb-09
38286	72	3	0	0	A411.00	Jakob-Creutzfeldt disease	Feb-09
38331	21	0	0	0	B64yz00	Other lymphoid leukaemia NOS	Feb-09
38411	14	0	0	0	7904z00	Other correction of transposition of great vessels NOS	Feb-09
38442	35	0	0	0	BB81200	"[M]Serous cystadenocarcinoma, NOS"	Feb-09
38454	193	4	0	0	BB48.00	[M]Basaloid carcinoma	Feb-09
38475	56	1	0	0	B34yz00	Malignant neoplasm of other site of female breast NOS	Feb-09
38479	77	3	0	0	PFy0.00	Arthrogryposis multiplex congenita	Feb-09
38481	10	0	0	0	BBTK.00	"[M]Epithelioid haemangioendothelioma, malignant"	Feb-09
38488	3	0	0	0	B013z00	Malignant neoplasm of ventral tongue surface NOS	Feb-09
38510	1032	21	0	0	B47z.00	Malignant neoplasm of testis NOS	Feb-09
38551	36	0	0	0	BBb1.00	[M]Gliomatosis cerebri	Feb-09
38575	8	1	0	0	BB62100	[M]Apocrine adenocarcinoma	Feb-09
38608	136	2	0	0	L250.11	Suspect fetal anencephaly	Feb-09
38685	115	1	0	0	BB5fz00	[M]Thyroid adenoma or adenocarcinoma NOS	Feb-09
38689	245	2	0	0	B325.00	Malignant melanoma of trunk (excluding scrotum)	Feb-09
38736	80	0	0	0	B5y..00	Malignant neoplasm of other and unspecified site OS	Feb-09
38756	5	0	0	0	BBP9.00	[M]Cystic mesothelioma	Feb-09
38770	5	0	0	0	BBB7.00	[M]Epithelial-myoepithelial carcinoma	Feb-09
38862	47	1	0	0	B490.00	Malignant neoplasm of trigone of urinary bladder	Feb-09
38914	57	1	0	0	B64z.00	Lymphoid leukaemia NOS	Feb-09
38918	148	1	0	0	B583100	Secondary malignant neoplasm of spinal cord	Feb-09

For peer review only - http://bmjopen.bmj.com/site/about/guidelines.xhtml

38931	50	0	0	0	B4y..00	Malignant neoplasm of genitourinary organ OS	Feb-09
38938	28	1	0	0	B306z00	"Malignant neoplasm of pelvis, sacrum or coccyx NOS"	Feb-09
38939	36	0	0	0	B613.00	"Hodgkin's disease, lymphocytic-histiocytic predominance"	Feb-09
38961	408	2	0	0	B22y.00	Malignant neoplasm of other sites of bronchus or lung	Feb-09
38978	50	0	0	0	B15z.00	Malignant neoplasm of liver and intrahepatic bile ducts NOS	Feb-09
39017	29	1	0	0	PJ2z.00	Edward's syndrome NOS	Feb-09
39023	130	0	0	0	F256.12	West syndrome	Feb-09
39027	103	0	0	0	ByuC000	[X]Malignant neoplasm of other specified sites	Feb-09
39038	88	0	0	0	BB85.00	[M]Signet ring carcinoma	Feb-09
39084	402	10	0	0	B0z2.00	Malignant neoplasm of laryngopharynx	Feb-09
39088	35	0	0	0	B514.00	Malignant neoplasm of occipital lobe	Feb-09
39121	13	1	0	0	BBc0100	[M]Ganglioneuroblastoma	Feb-09
39148	15	0	0	0	BB5Mz00	[M]Tubular adenoma or adenocarcinoma NOS	Feb-09
39187	110	4	0	0	B631.00	Plasma cell leukaemia	Feb-09
39312	28	0	0	0	BBM8.00	[M]Cystosarcoma phyllodes NOS	Feb-09
39386	8	0	0	0	BBb2.11	[M]Mixed glioma	Feb-09
39388	50	0	0	0	BBcC.11	[M]Olfactory neuroblastoma	Feb-09
39413	72	0	0	0	B18y500	Malignant neoplasm of pelvic peritoneum	Feb-09
39430	476	6	0	0	B0zz.00	"Malignant neoplasm of lip, oral cavity and pharynx NOS"	Feb-09
39522	60	4	0	0	BBV..00	[M]Osteomas and osteosarcomas	Feb-09
39554	21	0	0	0	B063.00	Malignant neoplasm of vallecula	Feb-09
39590	3	1	0	0	B206.00	"Malignant neoplasm, overlapping lesion of accessory sinuses"	Feb-09
39629	16	0	0	0	B653100	Granulocytic sarcoma	Feb-09

For peer review only - http://bmjopen.bmj.com/site/about/guidelines.xhtml

39760	129	3	0	0	BB91100	[M]Infiltrating duct and lobular carcinoma	Feb-09
39798	117	1	0	0	B627X00	"Diffuse non-Hodgkin's lymphoma, unspecified"	Feb-09
39870	131	1	0	0	B172.00	Malignant neoplasm of tail of pancreas	Feb-09
39875	46	0	0	0	BB5Nz00	[M]Adenomatous or adenocarcinomatous polyps of the colon NOS	Feb-09
39878	7	0	0	0	B327300	Malignant melanoma of popliteal fossa area	Feb-09
39897	102	1	0	0	B081.00	Malignant neoplasm of pyriform sinus	Feb-09
39899	34	5	0	0	B542100	Malignant neoplasm of craniopharyngeal duct	Feb-09
39906	4	0	0	0	BBgE.00	"[M]Malignant lymphoma, centrocytic"	Feb-09
39923	293	1	0	0	B223100	Malignant neoplasm of middle lobe of lung	Feb-09
39945	86	0	0	0	J600011	Acute liver failure	Feb-09
40014	112	2	0	0	B310100	Malignant neoplasm of soft tissue of face	Feb-09
40240	291	11	0	0	BB5D500	[M]Hepatocellular carcinoma NOS	Feb-09
40292	193	1	0	0	B053.00	Malignant neoplasm of soft palate	Feb-09
40344	32	0	0	0	F200.00	Multiple sclerosis of the brain stem	Feb-09
40359	33	1	0	0	BB94.00	[M]Juvenile breast carcinoma	Feb-09
40420	6	0	0	0	BBr0.00	[M]Leukaemias unspecified	Feb-09
40437	20	0	0	0	B50y.00	Malignant neoplasm of other specified site of eye	Feb-09
40438	40	2	0	0	BB5D111	[M]Bile duct carcinoma	Feb-09
40443	19	0	0	0	B33..15	Malignant neoplasm of sweat gland	Feb-09
40492	17	0	0	0	BBe9.00	"[M]Triton tumour, malignant"	Feb-09
40508	4	0	0	0	BBj6000	"[M]Hodgkin,s disease, nodular sclerosis, lymphocytic predom"	Feb-09
40513	66	1	0	0	BBkz.00	"[M]Lymphoma, nodular or follicular NOS"	Feb-09
40557	136	0	0	0	B01z.00	Malignant neoplasm of tongue NOS	Feb-09
40561	75	4	0	0	ZV10711	[V]Personal history of Hodgkin's disease	Feb-09

For peer review only - http://bmjopen.bmj.com/site/about/guidelines.xhtml

40592	11	0	0	0	Byu5.00	[X]Malignant neoplasm of mesothelial and soft tissue	Feb-09
40595	102	2	0	0	Byu2000	"[X]Malignant neoplasm of bronchus or lung, unspecified"	Feb-09
40598	130	2	0	0	Byu7.00	[X]Malignant neoplasm of female genital organs	Feb-09
40622	29	0	0	0	BB5V711	[M]Mucoid cell carcinoma	Feb-09
40632	150	5	0	0	BB82.00	[M]Mucinous adenoma and adenocarcinoma	Feb-09
40671	93	0	0	0	Byu8.00	[X]Malignant neoplasm of male genital organs	Feb-09
40740	42	0	0	0	ByuD.00	"[X]Malignant neoplasms of lymphoid, haematopoietic and rela"	Feb-09
40749	10	0	0	0	Byu3.00	[X]Malignant neoplasm of bone and articular cartilage	Feb-09
40766	178	2	0	0	BBm5.00	[M] Peripheral T-cell lymphoma NOS	Feb-09
40810	125	1	0	0	B171.00	Malignant neoplasm of body of pancreas	Feb-09
40814	36	3	0	0	B307200	Malignant neoplasm of tibia	Feb-09
40966	24	0	0	0	B306300	Malignant neoplasm of sacral vertebra	Feb-09
40991	47	1	41	0	4M2..00	Lymphoma staging system	Feb-09
41142	21	1	0	0	D204.00	Idiopathic aplastic anaemia	Feb-09
41144	33	0	0	0	B582300	Secondary malignant neoplasm of skin of trunk	Feb-09
41185	20	0	0	0	Eu02400	[X]Dementia in human immunodef virus [HIV] disease	Feb-09
41215	120	4	0	0	B111100	Malignant neoplasm of pyloric canal of stomach	Feb-09
41245	372	5	0	0	F030411	Herpes simplex encephalitis	Feb-09
41278	107	1	0	0	B323000	Malignant melanoma of external surface of cheek	Feb-09
41313	19	0	0	0	BB5D300	[M]Bile duct cystadenocarcinoma	Feb-09

For peer review only

41362	35	0	0	0	B101.00	Malignant neoplasm of thoracic oesophagus	Feb-09
41369	6	0	0	0	B60..00	Lymphosarcoma and reticulosarcoma	Feb-09
41461	107	9	0	0	PKy0.11	Prader-Willi Syndrome	Feb-09
41488	20	2	0	0	G554100	Constrictive cardiomyopathy	Feb-09
41490	68	0	0	0	B327700	Malignant melanoma of foot	Feb-09
41500	60	2	0	0	BBr2300	[M]Chronic lymphoid leukaemia	Feb-09
41515	69	0	0	0	ByuA100	"[X]Malignant neoplasm/central nervous system, unspecified"	Feb-09
41520	405	1	0	0	B51z.00	Malignant neoplasm of brain NOS	Feb-09
41523	66	0	0	0	B223000	Malignant neoplasm of middle lobe bronchus	Feb-09
41530	7	0	0	0	B01y.00	Malignant neoplasm of other sites of tongue	Feb-09
41571	93	0	0	0	B495.00	Malignant neoplasm of bladder neck	Feb-09
41695	85	0	0	0	BBba.00	[M]Primitive neuroectodermal tumour	Feb-09
41702	100	3	0	0	BB5N.00	[M]Adenomatous and adenocarcinomatous polyps of colon	Feb-09
41734	17	1	0	0	BBr0000	[M]Leukaemia NOS	Feb-09
41754	52	10	0	0	BBg7.00	"[M]Malignant lymphoma, lymphoplasmacytoid type"	Feb-09
41803	51	2	0	0	BBf..00	[M]Granular cell tumours and alveolar soft part sarcoma	Feb-09
41816	63	1	0	0	BB2E.00	"[M]Squamous cell carcinoma, small cell, non-keratinising"	Feb-09
41841	12	0	0	0	BBgB.00	"[M]Malignant lymphoma, follicular centre cell NOS"	Feb-09
41931	87	0	0	0	B550100	Malignant neoplasm of cheek NOS	Feb-09
41958	141	2	0	0	B331200	Malignant neoplasm of lower eyelid	Feb-09
42012	97	1	0	0	B494.00	Malignant neoplasm of posterior wall of urinary bladder	Feb-09
42017	48	1	0	0	C306400	Hyperammonaemia	Feb-09

For peer review only - http://bmjopen.bmj.com/site/about/guidelines.xhtml

42023	13	1	0	0	B497.00	Malignant neoplasm of urachus	Feb-09
42070	178	2	0	0	B345.00	Malignant neoplasm of lower-outer quadrant of female breast	Feb-09
42082	36	0	0	0	BBK3700	[M]Alveolar rhabdomyosarcoma	Feb-09
42153	58	0	0	0	B32y.00	Malignant melanoma of other specified skin site	Feb-09
42169	432	2	0	0	BB5cz00	[M]Parathyroid adenoma or adenocarcinoma NOS	Feb-09
42193	109	0	0	0	B115.00	Malignant neoplasm of lesser curve of stomach unspecified	Feb-09
42198	35	1	0	0	BBj6.00	"[M]Hodgkin's disease, nodular sclerosis NOS"	Feb-09
42218	62	0	0	0	B55y.00	Malignant neoplasm of other specified sites	Feb-09
42273	134	4	0	0	BB5T.00	[M]Papillary adenomas and adenocarcinomas	Feb-09
42297	100	0	0	0	BBrz.00	[M]Leukaemia NOS	Feb-09
42416	589	10	0	0	B105.00	Malignant neoplasm of lower third of oesophagus	Feb-09
42426	190	3	0	0	B511.00	Malignant neoplasm of frontal lobe	Feb-09
42429	9	6	0	0	B33X.00	Malignant neoplasm overlapping lesion of skin	Feb-09
42460	43	0	0	0	B543.00	Malignant neoplasm of pineal gland	Feb-09
42461	139	2	0	0	B61zz00	Hodgkin's disease NOS	Feb-09
42497	6	0	0	0	P100.00	Unspecified spina bifida with hydrocephalus	Feb-09
42539	54	2	0	0	B670.00	Acute erythraemia and erythroleukaemia	Feb-09
42542	83	3	0	0	BB9K.00	[M]Paget's disease and infiltrating breast duct carcinoma	Feb-09
42553	17	1	0	0	BBB3.00	[M]Adenocarcinoma with cartilaginous and osseous metaplasia	Feb-09

For peer review only - http://bmjopen.bmj.com/site/about/guidelines.xhtml

42566	167	0	0	0	B224z00	"Malignant neoplasm of lower lobe, bronchus or lung NOS"	Feb-09
42569	196	3	0	0	B2zz.00	Malignant neoplasm of respiratory tract NOS	Feb-09
42579	13	0	0	0	B62y300	Malignant lymphoma NOS of intra-abdominal lymph nodes	Feb-09
42636	80	3	0	0	C307y11	Methylmalonic acidaemia	Feb-09
42643	33	1	0	0	PK64.00	Proteus syndrome	Feb-09
42707	49	0	0	0	B336100	Malignant neoplasm of skin of upper arm	Feb-09
42714	48	0	0	0	B327500	Malignant melanoma of ankle	Feb-09
42769	10	0	0	0	BBjz.00	[M]Hodgkin's disease NOS	Feb-09
42784	57	0	0	0	P226.00	Microgyria	Feb-09
42856	27	0	0	0	B200z00	Malignant neoplasm of nasal cavities NOS	Feb-09
43005	97	0	0	0	Q200012	Intracranial haemorrhage in fetus or newborn	Feb-09
43087	193	12	0	0	B331.00	Malignant neoplasm of eyelid including canthus	Feb-09
43111	68	1	0	0	B213.00	Malignant neoplasm of laryngeal cartilage	Feb-09
43122	111	1	0	0	B336000	Malignant neoplasm of skin of shoulder	Feb-09
43151	3	0	0	0	Byu3300	"[X]Malignant neoplasm/bone+articular cartilage, unspecified"	Feb-09
43200	67	1	0	0	B06z.00	Malignant neoplasm of oropharynx NOS	Feb-09
43203	132	7	0	0	F100.00	Leucodystrophy	Feb-09
43311	19	1	0	0	ZV10212	[V]Personal history of malignant neoplasm of larynx	Feb-09
43325	87	2	0	0	PKy7300	Rubenstein - Tayi syndrome	Feb-09
43390	173	0	0	0	B12z.00	Malignant neoplasm of small intestine NOS	Feb-09
43392	237	4	0	0	B483.00	"Malignant neoplasm of penis, part unspecified"	Feb-09
43400	135	3	0	0	B03..00	Malignant neoplasm of gum	Feb-09
43415	14	1	0	0	ByuD000	[X]Other Hodgkin's disease	Feb-09

For peer review only - http://bmjopen.bmj.com/site/about/guidelines.xhtml

43431	272	0	0	0	B010.00	Malignant neoplasm of base of tongue	Feb-09
43435	46	0	0	0	B41yz00	Malignant neoplasm of other site of cervix NOS	Feb-09
43450	41	0	0	0	B63z.00	Immunoproliferative neoplasm or myeloma NOS	Feb-09
43463	383	1	0	0	B325700	Malignant melanoma of back	Feb-09
43479	46	0	0	0	B121.00	Malignant neoplasm of jejunum	Feb-09
43490	41	0	0	0	Byu1100	[X]Other specified carcinomas of liver	Feb-09
43548	26	0	0	0	B080.00	Malignant neoplasm of postcricoid region	Feb-09
43565	6	0	0	0	PJ11.00	"Trisomy 13, mosaicism"	Feb-09
43572	142	1	0	0	B114.00	Malignant neoplasm of body of stomach	Feb-09
43582	52	0	0	0	BBQz.00	[M]Germ cell neoplasm NOS	Feb-09
43594	17	0	0	0	BB5Cz00	[M]Gastrinoma or carcinoma NOS	Feb-09
43614	13	0	0	0	B30X.00	"Malignant neoplasm/bones+articular cartilage/limb,unspfd"	Feb-09
43619	159	0	0	0	B334100	Malignant neoplasm of skin of neck	Feb-09
43642	10	0	0	0	B011.00	Malignant neoplasm of dorsal surface of tongue	Feb-09
43712	44	1	0	0	C375311	Sanfilippo syndrome	Feb-09
43715	6	0	0	0	B325600	Malignant melanoma of umbilicus	Feb-09
43717	12	0	0	0	BB24.11	[M]Verrucous epidermoid carcinoma	Feb-09
43761	44	5	0	0	B451.00	Malignant neoplasm of labia majora	Feb-09
43781	8	0	0	0	B011z00	Malignant neoplasm of dorsum of tongue NOS	Feb-09
43930	12	0	0	0	B582000	Secondary malignant neoplasm of skin of head	Feb-09
43940	13	0	0	0	B431.00	Malignant neoplasm of isthmus of uterine body	Feb-09
44074	28	1	0	0	BB84.00	[M]Mucin-producing adenocarcinoma	Feb-09
44089	27	0	0	0	B517.00	Malignant neoplasm of brain stem	Feb-09
44108	69	0	0	0	B18..00	Malignant neoplasm of retroperitoneum and peritoneum	Feb-09

For peer review only - http://bmjopen.bmj.com/site/about/guidelines.xhtml

44139	1	0	0	0	B073.00	Malignant neoplasm of anterior wall of nasopharynx	Feb-09
44157	5	0	0	0	BBE1.13	[M]Melanosarcoma NOS	Feb-09
44169	217	4	0	0	B222z00	"Malignant neoplasm of upper lobe, bronchus or lung NOS"	Feb-09
44196	11	1	0	0	B611.00	Hodgkin's granuloma	Feb-09
44201	11	0	0	0	PB57.00	Microcolon	Feb-09
44267	71	6	1	0	B623.00	Malignant histiocytosis	Feb-09
44272	33	0	0	0	F391B00	Cardiomyopathy in Duchenne muscular dystrophy	Feb-09
44288	132	2	0	0	R109.00	[D]Laboratory evidence of human immunodeficiency virus [HIV]	Feb-09
44318	144	7	0	0	B62xX00	Oth and unspecif peripheral & cutaneous T-cell lymphomas	Feb-09
44399	54	0	0	0	B150z00	Primary malignant neoplasm of liver NOS	Feb-09
44421	20	2	0	0	ZV67A00	[V]Folow-up exam aft other treatment for malignant neoplasm	Feb-09
44452	6	2	0	0	B300C00	Malignant neoplasm of vomer	Feb-09
44529	40	2	0	0	B575.00	Secondary malignant neoplasm of large intestine and rectum	Feb-09
44592	F11xz00	8	0	0	0	Cerebral degeneration other disease NOS	Feb-09
44609	18	0	0	0	B306000	Malignant neoplasm of ilium	Feb-09
44615	129	0	0	0	B586.00	Secondary malignant neoplasm of ovary	Feb-09
44617	13	0	0	0	A789600	HIV disease resulting in Burkitt's lymphoma	Feb-09
44759	9	0	0	0	PG52000	Osteopetrosis - unclassified	Feb-09
44778	94	0	0	0	BB52000	[M]Adenocarcinoma in tubulovillous adenoma	Feb-09
44871	331	0	1	0	C308000	Medium chain acyl-CoA dehydrogenase deficiency	Feb-09
44884	19	2	0	0	B4Ay.00	Malignant neoplasm of other urinary organs	Feb-09

For peer review only - http://bmjopen.bmj.com/site/about/guidelines.xhtml

44930	11	0	0	0	BB81800	[M]Papillary serous cystadenocarcinoma	Feb-09
44939	P251.00	48	0	1	0	Hydromyelia	Feb-09
44996	33	0	0	0	B491.00	Malignant neoplasm of dome of urinary bladder	Feb-09
45071	42	2	0	0	B314.00	Malignant neoplasm of connective and soft tissue of abdomen	Feb-09
45077	223	4	0	0	B335700	Malignant neoplasm of skin of back	Feb-09
45139	45	0	0	0	B323400	Malignant melanoma of external surface of nose	Feb-09
45154	117	4	0	0	B516.00	Malignant neoplasm of cerebellum	Feb-09
45222	118	1	0	0	B343.00	Malignant neoplasm of lower-inner quadrant of female breast	Feb-09
45260	9	0	0	0	Byu9000	"[X]Malignant neoplasm of urinary organ, unspecified"	Feb-09
45262	7	0	0	0	Byu8200	"[X]Malignant neoplasm of male genital organ, unspecified"	Feb-09
45264	20	0	0	0	B620100	"Nodular lymphoma of lymph nodes of head, face and neck"	Feb-09
45267	30	0	0	0	B55z.00	Malignant neoplasm of other and ill defined site NOS	Feb-09
45306	96	0	0	0	B324100	Malignant melanoma of neck	Feb-09
45307	52	0	0	0	B2...11	Carcinoma of respiratory tract and intrathoracic organs	Feb-09
45408	35	0	0	0	B040.00	Malignant neoplasm of anterior portion of floor of mouth	Feb-09
45458	33	1	0	0	BB2F.00	"[M]Squamous cell carcinoma, spindle cell type"	Feb-09
45490	67	0	0	0	B430z00	Malignant neoplasm of corpus uteri NOS	Feb-09
45510	30	0	0	0	BB2M.00	[M]Lymphoepithelial carcinoma	Feb-09
45531	33	1	0	0	BBbE.00	[M]Gemistocytic astrocytoma	Feb-09
45667	27	0	0	0	B501.00	Malignant neoplasm of orbit	Feb-09
45755	161	0	0	0	B326200	Malignant melanoma of fore-arm	Feb-09

For peer review only - http://bmjopen.bmj.com/site/about/guidelines.xhtml

45760	51	0	0	0	B325z00	"Malignant melanoma of trunk, excluding scrotum, NOS"	Feb-09
45766	11	0	0	0	Byu1200	"[X]Malignant neoplasm of intestinal tract, part unspecified"	Feb-09
45793	27	0	0	0	B430300	Malignant neoplasm of myometrium of corpus uteri	Feb-09
45824	7	1	0	0	B58y900	Secondary malignant neoplasm of tongue	Feb-09
45903	23	0	0	0	F100000	Krabbe's disease	Feb-09
45922	15	6	0	0	B508.00	"Malignant neoplasm, overlapping lesion of eye and adnexa"	Feb-09
45986	13	0	0	0	B041.00	Malignant neoplasm of lateral portion of floor of mouth	Feb-09
46008	93	2	0	0	B333z00	Malignant neoplasm skin other and unspec part of face NOS	Feb-09
46048	5	0	0	0	BBr2500	[M]Prolymphocytic leukaemia	Feb-09
46110	9	0	0	0	A75..12	Pfeiffer's disease	Feb-09
46128	F240000	9	0	0	0	Flaccid tetraplegia	Feb-09
46133	11	1	0	0	PJ12.00	"Trisomy 13, translocation"	Feb-09
46153	6	0	0	0	B443.00	Malignant neoplasm of parametrium	Feb-09
46159	6	0	0	0	B142000	Malignant neoplasm of cloacogenic zone	Feb-09
46255	393	4	0	0	B327.00	Malignant melanoma of lower limb and hip	Feb-09
46263	64	4	0	0	BBr6700	[M]Acute myelomonocytic leukaemia	Feb-09
46423	120	0	0	0	B933.11	Cystosarcoma phyllodes	Feb-09
46444	20	1	0	0	BBr4.00	[M]Erythroleukaemias	Feb-09
46458	8	0	0	0	B335600	Malignant neoplasm of skin of perineum	Feb-09
46516	51	3	0	0	C327200	Niemann-Pick disease	Feb-09
46548	42	1	0	0	B071100	Malignant neoplasm of pharyngeal tonsil	Feb-09
46581	140	0	0	0	BBF4.11	[M]Pleomorphic cell sarcoma	Feb-09
46583	6	5	0	0	P234.00	Hydranencephaly	Feb-09
46613	186	1	0	0	B18y.00	Malignant neoplasm of specified parts of peritoneum	Feb-09

For peer review only - http://bmjopen.bmj.com/site/about/guidelines.xhtml

46728	40	1	0	0	B064.00	Malignant neoplasm of anterior epiglottis	Feb-09
46741	3	0	0	0	BBZC.00	[M]Ameloblastic odontosarcoma	Feb-09
46761	53	0	0	0	BB5f600	[M]Papillary and follicular adenocarcinoma	Feb-09
46771	19	1	0	0	BB5D800	"[M]Hepatocellular carcinoma, fibrolamellar"	Feb-09
46779	26	0	0	0	ZV10417	[V]Personal history of malignant neoplasm of uterine body	Feb-09
46787	29	0	0	0	PJ2z.11	TRISOMY 18 NOS	Feb-09
46789	13	1	0	0	B515000	Malignant neoplasm of choroid plexus	Feb-09
46790	196	7	0	0	P10..00	Spina bifida with hydrocephalus	Feb-09
46792	111	1	0	0	B512.00	Malignant neoplasm of temporal lobe	Feb-09
46794	7	0	0	0	PH3y800	Epidermolysis bullosa letalis	Feb-09
46877	47	1	0	0	BBgL.00	"[M]Malignant lymphoma, small lymphocytic NOS"	Feb-09
46905	1	0	0	0	B545200	Malignant neoplasm of coccygeal body	Feb-09
46931	3	0	0	0	BBg4.00	"[M]Malignant lymphoma, stem cell type"	Feb-09
46939	21	1	0	0	B302000	Malignant neoplasm of cervical vertebra	Feb-09
47094	4	0	0	0	B323200	Malignant melanoma of eyebrow	Feb-09
47205	17	12	0	0	B017.00	Malignant overlapping lesion of tongue	Feb-09
47252	59	1	0	0	B323.00	Malignant melanoma of other and unspecified parts of face	Feb-09
47286	20	0	0	0	B551.00	Malignant neoplasm of thorax	Feb-09
47288	63	0	0	0	P102z00	Spina bifida with hydrocephalus - open NOS	Feb-09
47342	29	1	0	0	Q48y000	Congenital renal failure	Feb-09
47556	30	0	0	0	B512z00	Malignant neoplasm of temporal lobe NOS	Feb-09
47668	4	1	0	0	B48y100	Malignant neoplasm of tunica vaginalis	Feb-09
47683	41	0	0	0	ZV10512	[V]Personal history of malignant neoplasm of kidney	Feb-09

For peer review only - http://bmjopen.bmj.com/site/about/guidelines.xhtml

47734	38	0	0	0	BBP5.00	"[M]Epithelioid mesothelioma, malignant"	Feb-09
47767	111	4	0	0	B486.00	Malignant neoplasm of scrotum	Feb-09
47801	6	0	0	0	B49y000	"Malignant neoplasm, overlapping lesion of bladder"	Feb-09
47810	234	8	0	0	B59..00	Malignant neoplasm of unspecified site	Feb-09
47840	5	0	0	0	B545100	Malignant neoplasm of aortic body	Feb-09
47862	96	2	0	0	B213300	Malignant neoplasm of thyroid cartilage	Feb-09
47899	17	1	0	0	B451000	Malignant neoplasm of greater vestibular (Bartholin's) gland	Feb-09
47920	17	0	0	0	BB9B.11	[M]C cell carcinoma	Feb-09
47974	28	4	0	0	F130600	Aicardi Goutieres syndrome	Feb-09
48048	53	1	0	0	BB1B.00	[M]Giant cell and spindle cell carcinoma	Feb-09
48049	22	0	0	0	BBr6800	[M]Chronic myelomonocytic leukaemia	Feb-09
48073	20	2	0	0	B510000	Malignant neoplasm of basal ganglia	Feb-09
48084	19	0	0	0	PKy9400	Zellweger's syndrome	Feb-09
48126	23	7	0	0	F232.11	Tetraplegia - congenital	Feb-09
48145	19	0	0	0	D212000	Anaemia in ovarian carcinoma	Feb-09
48155	17	1	0	0	BBr2.00	[M]Lymphoid leukaemias	Feb-09
48184	24	0	0	0	PG4E.00	Spondylometaphyseal dysplasia	Feb-09
48211	17	0	0	0	F10y000	Alper's disease	Feb-09
48223	31	1	0	0	BB54.00	[M]Scirrhous adenocarcinoma	Feb-09
48231	68	0	0	0	B13y.00	Malignant neoplasm of other specified sites of colon	Feb-09
48237	30	1	0	0	B111000	Malignant neoplasm of prepylorus of stomach	Feb-09
48253	7	0	0	0	BBg8.00	"[M]Malignant lymphoma, immunoblastic type"	Feb-09
48271	16	0	0	0	BBVz.00	[M]Osteoma or osteosarcoma NOS	Feb-09
48275	50	0	0	0	BBK3600	[M]Embryonal rhabdomyosarcoma	Feb-09
48288	11	1	0	0	R210200	[D]Nonspecific sudden infant death	Feb-09
48293	15	0	0	0	C392100	Severe combined immunodeficiency	Feb-09

For peer review only - http://bmjopen.bmj.com/site/about/guidelines.xhtml

48300	51	3	0	0	F10y100	Leigh's disease	Feb-09
48517	85	10	0	0	B310200	Malignant neoplasm of soft tissue of neck	Feb-09
48519	5	0	0	0	B065.00	Malignant neoplasm of junctional region of epiglottis	Feb-09
48531	75	4	0	0	F11x700	Cerebral degeneration due to Jakob - Creutzfeldt disease	Feb-09
48537	8	0	0	0	B17y.00	Malignant neoplasm of other specified sites of pancreas	Feb-09
48609	P118.00	5	0	0	0	Spina bifida without hydrocephalus - closed	Feb-09
48697	11	0	0	0	7A65000	Transposition of valve of vein	Feb-09
48743	57	0	0	0	B482.00	Malignant neoplasm of body of penis	Feb-09
48809	49	0	0	0	B35zz00	Malignant neoplasm of male breast NOS	Feb-09
48820	246	0	0	0	B410.00	Malignant neoplasm of endocervix	Feb-09
48828	13	0	0	0	B582500	Secondary malignant neoplasm of skin of hip and leg	Feb-09
48879	12	0	0	0	J637.00	Hepatic veno-occlusive disease	Feb-09
48952	87	1	0	0	BBc9z00	[M]Retinoblastoma NOS	Feb-09
48991	0	2	4	0	5136	X-ray metastasis control	Feb-09
49023	10	1	0	0	BBY0.11	[M]Endothelial bone sarcoma	Feb-09
49054	28	0	0	0	B304000	Malignant neoplasm of scapula	Feb-09
49131	14	1	0	0	BBg0.00	"[M]Lymphomatous tumour, benign"	Feb-09
49132	7	0	0	0	B517100	Malignant neoplasm of medulla oblongata	Feb-09
49145	8	0	0	0	B58y700	Secondary malignant neoplasm of penis	Feb-09
49148	21	1	0	0	B347.00	"Malignant neoplasm, overlapping lesion of breast"	Feb-09
49168	15	1	0	0	BBb4.00	[M]Subependymal giant cell astrocytoma	Feb-09
49181	19	0	0	0	F101400	Gangliosidosis	Feb-09
49186	118	5	0	0	BBbR.00	"[M]Oligodendroglioma, anaplastic type"	Feb-09
49253	12	0	0	0	BBk0.13	[M]Giant follicular lymphoma	Feb-09

For peer review only - http://bmjopen.bmj.com/site/about/guidelines.xhtml

49262	55	2	0	0	B627200	Follicular non-Hodgkin's large cell lymphoma	Feb-09
49292	12	0	0	0	Byu1300	[X]Malignant neoplsm/ill-defin sites within digestive system	Feb-09
49301	437	30	0	0	B6z..00	Malignant neoplasm lymphatic or haematopoietic tissue NOS	Feb-09
49322	61	0	0	0	F256z00	Infantile spasms NOS	Feb-09
49327	8	0	0	0	BBrA500	[M]Acute megakaryoblastic leukaemia	Feb-09
49341	3	0	3	0	4D56.00	Pleural fluid: malignant cells	Feb-09
49360	30	2	0	0	B031.00	Malignant neoplasm of lower gum	Feb-09
49400	260	1	0	0	B430211	Malignant neoplasm of endometrium	Feb-09
49403	55	3	0	0	B333100	Malignant neoplasm of skin of chin	Feb-09
49463	11	0	0	0	B310400	Malignant neoplasm of tarsus of eyelid	Feb-09
49482	3	0	0	0	F396200	Myopathy due to malignant disease	Feb-09
49491	31	0	0	0	B303100	Malignant neoplasm of sternum	Feb-09
49525	54	1	0	0	B59zX00	"Kaposi's sarcoma, unspecified"	Feb-09
49542	6	0	0	0	C392500	Severe combined immunodef with low or normal B-cell numbers	Feb-09
49605	43	1	0	0	B615.00	"Hodgkin's disease, mixed cellularity"	Feb-09
49629	7	0	0	0	BB5C100	"[M]Gastrinoma, malignant"	Feb-09
49701	21	1	0	0	B302z00	Malignant neoplasm of vertebral column NOS	Feb-09
49714	10	0	0	0	B523.00	Malignant neoplasm of spinal meninges	Feb-09
49725	20	0	0	0	B64y.00	Other lymphoid leukaemia	Feb-09
49758	22	1	0	0	B0zy.00	"Malignant neoplasm of other sites lip, oral cavity, pharynx"	Feb-09
49770	304	7	0	0	C370z00	Cystic fibrosis NOS	Feb-09
49814	59	1	0	0	B325000	Malignant melanoma of axilla	Feb-09
49825	23	0	0	0	BBh0.11	[M]Reticulum cell sarcoma NOS	Feb-09
49828	174	3	0	0	B441.00	Malignant neoplasm of fallopian tube	Feb-09
49862	42	0	0	0	BBV1.11	[M]Osteoblastic sarcoma	Feb-09

For peer review only - http://bmjopen.bmj.com/site/about/guidelines.xhtml

49875	18	0	0	0	B52X.00	"Malignant neoplasm of meninges, unspecified"	Feb-09
50035	37	3	0	0	B545.00	Malignant neoplasm of aortic body and other paraganglia	Feb-09
50076	14	1	0	0	A789400	HIV disease resulting in multiple infections	Feb-09
50108	11	1	0	0	BB5Uz00	[M]Villous adenoma or adenocarcinoma NOS	Feb-09
50140	16	0	0	0	BB5K.00	[M]Cribriform carcinoma	Feb-09
50152	62	0	0	0	B306500	Malignant sacral teratoma	Feb-09
50157	28	0	0	0	G210.00	Malignant hypertensive heart disease	Feb-09
50222	17	0	0	0	B311000	Malignant neoplasm of connective and soft tissue of shoulder	Feb-09
50251	5	1	0	0	PF55100	Acrocephalosyndactyly (Pfeiffer)	Feb-09
50285	67	0	0	0	B410z00	Malignant neoplasm of endocervix NOS	Feb-09
50289	2	0	0	0	B241z00	Malignant neoplasm of heart NOS	Feb-09
50290	6	0	0	0	B6z0.00	Kaposi's sarcoma of lymph nodes	Feb-09
50292	5	0	0	0	Byu2500	"[X]Malignant neoplasm of mediastinum, part unspecified"	Feb-09
50296	3	0	0	0	B000100	"Malignant neoplasm of upper lip, lipstick area"	Feb-09
50297	55	0	0	0	B411.00	Malignant neoplasm of exocervix	Feb-09
50298	5	0	0	0	B300500	Malignant neoplasm of orbital bone	Feb-09
50299	2	0	0	0	B300900	Malignant neoplasm of zygomatic bone	Feb-09
50379	47	1	0	0	BBN1.00	[M]Synovial sarcoma NOS	Feb-09
50402	7	0	0	0	B307100	Malignant neoplasm of fibula	Feb-09
50444	20	0	0	0	PB61z00	Biliary atresia NOS	Feb-09
50475	9	0	0	0	B02z.00	Malignant neoplasm of major salivary gland NOS	Feb-09
50505	123	0	0	0	B326000	Malignant melanoma of shoulder	Feb-09
50554	143	1	0	0	Q48E.11	Periventricular leukomalacia	Feb-09
50565	1	0	0	0	P100200	Thoracic spina bifida with hydrocephalus	Feb-09

For peer review only - http://bmjopen.bmj.com/site/about/guidelines.xhtml

50579	4	0	0	0	B214.00	"Malignant neoplasm, overlapping lesion of larynx"	Feb-09
50605	3	0	0	0	BBDB.00	[M]Glomangiosarcoma	Feb-09
50668	14	0	0	0	B627300	Diffuse non-Hodgkin's small cell (diffuse) lymphoma	Feb-09
50681	50	6	0	0	B480.00	Malignant neoplasm of prepuce (foreskin)	Feb-09
50695	9	0	0	0	B627500	Diffuse non-Hodgkin mixed sml & lge cell (diffuse) lymphoma	Feb-09
50696	34	0	0	0	B62y100	"Malignant lymphoma NOS of lymph nodes of head, face and neck"	Feb-09
50777	2	0	0	0	B524600	"Malignant neoplasm,overlap lesion periph nerve & auton ns"	Feb-09
50789	60	0	0	0	B103.00	Malignant neoplasm of upper third of oesophagus	Feb-09
50859	6	0	0	0	BBX1.11	[M]Giant cell bone sarcoma	Feb-09
50898	74	0	0	0	B18y300	Malignant neoplasm of omentum	Feb-09
50928	10	0	0	0	BBr2600	[M]Burkitt's cell leukaemia	Feb-09
50946	5	0	0	0	BB9C.00	[M]Medullary carcinoma with amyloid stroma	Feb-09
50974	67	0	0	0	B14z.00	"Malignant neoplasm rectum,rectosigmoid junction and anus NOS"	Feb-09
51115	96	0	0	0	B522.00	Malignant neoplasm of spinal cord	Feb-09
51209	78	7	0	0	B325800	Malignant melanoma of chest wall	Feb-09
51237	5	0	0	0	B303z00	"Malignant neoplasm of rib, sternum and clavicle NOS"	Feb-09
51255	58	0	0	0	B1zz.00	Malignant neoplasm of digestive tract and peritoneum NOS	Feb-09
51285	12	0	0	0	BBj2.00	"[M]Hodgkin's disease, mixed cellularity"	Feb-09
51323	9	0	0	0	R210z00	[D]Sudden infant death syndrome NOS	Feb-09
51352	10	0	0	0	B592.00	Malignant neoplasms of independent (primary) multiple sites	Feb-09

For peer review only - http://bmjopen.bmj.com/site/about/guidelines.xhtml

51353	8	1	0	0	BBE1000	"[M]Malignant melanoma, regressing"	Feb-09
51551	41	2	0	0	B571.00	Secondary malignant neoplasm of mediastinum	Feb-09
51656	31	0	0	0	BB81E00	[M]Mucinous cystadenocarcinoma NOS	Feb-09
51680	8	0	0	0	BBgV.00	"[M]Malignant lymphoma, small cell, noncleaved, diffuse"	Feb-09
51690	1	0	0	0	B117.00	"Malignant neoplasm, overlapping lesion of stomach"	Feb-09
51708	7	0	0	0	A789X00	"HIV dis reslt/oth mal neopl/lymph,h'matopoetc+reltd tissu"	Feb-09
51786	100	1	0	0	B021.00	Malignant neoplasm of submandibular gland	Feb-09
51795	31	2	0	0	B545000	Malignant neoplasm of glomus jugulare	Feb-09
51818	27	0	0	0	B550300	Malignant neoplasm of jaw NOS	Feb-09
51852	1	0	0	0	BBgD.00	"[M]Malig lymphoma, lymphocytic, intermediate different NOS"	Feb-09
51873	196	5	0	0	B327100	Malignant melanoma of thigh	Feb-09
51878	8	0	0	0	BBcC.00	[M]Aesthesioneuroblastoma	Feb-09
51895	73	1	0	0	BBgz.00	"[M]Lymphoma, diffuse or NOS"	Feb-09
51911	30	1	0	0	P74z800	Atresia of pulmonary vein	Feb-09
51921	7	1	0	0	B306200	Malignant neoplasm of pubis	Feb-09
51926	33	0	0	0	B062000	Malignant neoplasm of faucial pillar	Feb-09
51965	26	0	0	0	B315.00	Malignant neoplasm of connective and soft tissue of pelvis	Feb-09
52029	76	0	0	0	ByuC800	[X]Malignant neoplasm without specification of site	Feb-09
52070	20	1	0	0	BBDE.00	[M]Gangliocytic paraganglioma	Feb-09
52130	P204.00	22	0	0	0	Meningoencephalocele	Feb-09
52258	15	0	0	0	PB33.00	Total intestinal aganglionosis	Feb-09
52316	29	0	0	0	B553.00	Malignant neoplasm of pelvis	Feb-09
52326	41	2	0	0	BB5L100	[M]Adenocarcinoma in adenomatous polyp	Feb-09

For peer review only - http://bmjopen.bmj.com/site/about/guidelines.xhtml

52493	20	1	0	0	BBQ7213	"[M]Teratoblastoma, malignant"	Feb-09
52496	15	3	0	0	BB61.00	[M]Sweat gland adenoma and adenocarcinomas	Feb-09
52511	6	0	0	0	B515.00	Malignant neoplasm of cerebral ventricles	Feb-09
52537	32	1	0	0	B161100	Malignant neoplasm of hepatic duct	Feb-09
52570	2	0	0	0	B487.00	"Malignant neoplasm, overlapping lesion of penis"	Feb-09
52594	59	1	0	0	B4z..00	Malignant neoplasm of genitourinary organ NOS	Feb-09
52677	18	0	0	0	A412.00	Subacute sclerosing panencephalitis	Feb-09
52683	P102.13	5	0	0	0	Myelocele with hydrocephalus	Feb-09
52684	14	0	0	0	BBW9.00	[M]Mesenchymal chondrosarcoma	Feb-09
52942	21	1	0	0	BBr6300	[M]Chronic myeloid leukaemia	Feb-09
53103	6	0	0	0	B410100	Malignant neoplasm of endocervical gland	Feb-09
53129	3	0	0	0	BB5W112	[M]Oncytic adenocarcinoma	Feb-09
53317	57	2	0	0	F383.00	Congenital and developmental myasthenia	Feb-09
53369	14	1	0	0	B327900	Malignant melanoma of great toe	Feb-09
53382	36	0	0	0	F100200	Pelizaeus-Merzbacher disease	Feb-09
53397	197	6	0	0	B61z.00	Hodgkin's disease NOS	Feb-09
53515	114	5	0	0	B332.00	Malignant neoplasm skin of ear and external auricular canal	Feb-09
53517	6	0	0	0	PB33.12	Congenital aganglionic megacolon	Feb-09
53528	8	0	0	0	B581200	Secondary malignant neoplasm of urethra	Feb-09
53551	8	0	0	0	B627600	Diffuse non-Hodgkin's immunoblastic (diffuse) lymphoma	Feb-09
53591	51	0	0	0	B10y.00	Malignant neoplasm of other specified part of oesophagus	Feb-09
53594	7	0	0	0	B300000	Malignant neoplasm of ethmoid bone	Feb-09
53599	8	0	0	0	B300100	Malignant neoplasm of frontal bone	Feb-09
53629	10	0	0	0	B325200	Malignant melanoma of buttock	Feb-09

For peer review only - http://bmjopen.bmj.com/site/about/guidelines.xhtml

53852	165	1	0	0	K05..12	End stage renal failure	Feb-09
53884	131	0	0	0	B060z00	Malignant neoplasm tonsil NOS	Feb-09
53910	21	0	0	0	B453.00	Malignant neoplasm of clitoris	Feb-09
53929	P113000	6	0	0	0	Spinal meningocele of unspecified site	Feb-09
53933	11	0	0	0	PG51z00	Osteogenesis imperfecta NOS	Feb-09
53987	9	0	0	0	BB5Dz00	[M]Hepatobiliary adenoma or carcinoma NOS	Feb-09
54103	85	0	0	0	B16..00	Malignant neoplasm gallbladder and extrahepatic bile ducts	Feb-09
54106	44	2	0	0	Eu02100	[X]Dementia in Creutzfeldt-Jakob disease	Feb-09
54120	5	0	0	0	B584.00	Secondary malignant neoplasm of other part of nervous system	Feb-09
54133	43	0	0	0	B510z00	Malignant neoplasm of cerebrum NOS	Feb-09
54134	45	0	0	0	B223z00	"Malignant neoplasm of middle lobe, bronchus or lung NOS"	Feb-09
54171	74	2	0	0	B104.00	Malignant neoplasm of middle third of oesophagus	Feb-09
54184	98	1	0	0	B4A1z00	Malignant neoplasm of renal pelvis NOS	Feb-09
54186	5	0	0	0	B313100	Malignant neoplasm of diaphragm	Feb-09
54202	6	0	0	0	B35z.00	Malignant neoplasm of other site of male breast	Feb-09
54222	13	0	0	0	B312400	Malignant neoplasm of connective and soft tissue of foot	Feb-09
54234	39	1	0	0	B334.00	Malignant neoplasm of scalp and skin of neck	Feb-09
54253	250	0	0	0	ByuC700	[X]Secondary malignant neoplasm of other specified sites	Feb-09
54267	90	4	0	0	B59z.00	Malignant neoplasm of unspecified site NOS	Feb-09
54276	4	0	0	0	BB1E.00	[M]Pseudosarcomatous carcinoma	Feb-09
54305	64	0	0	0	B327200	Malignant melanoma of knee	Feb-09
54352	90	2	0	0	B336300	Malignant neoplasm of skin of hand	Feb-09

For peer review only - http://bmjopen.bmj.com/site/about/guidelines.xhtml

54493	7	0	0	0	B303500	Malignant neoplasm of xiphoid process	Feb-09
54494	5	0	0	0	B350.00	Malignant neoplasm of nipple and areola of male breast	Feb-09
54585	52	0	0	0	BBr6100	[M]Acute myeloid leukaemia	Feb-09
54613	3	0	0	0	B201200	Malignant neoplasm of tympanic antrum	Feb-09
54627	6	0	0	0	BBR3.00	[M]Choriocarcinoma combined with teratoma	Feb-09
54631	39	1	0	0	B306.00	"Malignant neoplasm of pelvic bones, sacrum and coccyx"	Feb-09
54632	28	0	0	0	B321.00	Malignant melanoma of eyelid including canthus	Feb-09
54636	53	2	0	0	B203.00	Malignant neoplasm of ethmoid sinus	Feb-09
54679	89	1	0	0	B594.00	Secondary malignant neoplasm of unknown site	Feb-09
54685	173	0	0	0	B326100	Malignant melanoma of upper arm	Feb-09
54691	62	0	0	0	B302200	Malignant neoplasm of lumbar vertebra	Feb-09
54714	P00..11	2	0	0	0	Congenital absence of brain	Feb-09
54747	4	0	0	0	B300600	Malignant neoplasm of parietal bone	Feb-09
54749	2	0	0	0	BB81H00	[M]Papillary mucinous cystadenocarcinoma	Feb-09
54793	3	0	0	0	B682.00	Subacute leukaemia NOS	Feb-09
54874	3	0	0	0	BB85100	[M]Metastatic signet ring cell carcinoma	Feb-09
54934	51	3	0	0	F030400	Encephalitis due to herpes simplex virus	Feb-09
54956	26	0	0	0	B50z.00	Malignant neoplasm of eye NOS	Feb-09
55015	130	0	0	0	B05z.00	Malignant neoplasm of mouth NOS	Feb-09
55019	21	1	0	0	B11y.00	Malignant neoplasm of other specified site of stomach	Feb-09
55066	19	0	0	0	B062.00	Malignant neoplasm of tonsillar pillar	Feb-09
55090	20	0	0	0	B58y100	Secondary malignant neoplasm of uterus	Feb-09
55096	33	1	0	0	B582z00	Secondary malignant neoplasm of skin NOS	Feb-09
55098	71	1	0	0	B550000	Malignant neoplasm of head NOS	Feb-09

For peer review only - http://bmjopen.bmj.com/site/about/guidelines.xhtml

55101	16	0	0	0	B553z00	Malignant neoplasm of pelvis NOS	Feb-09
55116	46	2	0	0	BBFz.00	[M]Soft tissue tumour or sarcoma NOS	Feb-09
55246	6	0	0	0	B20z.00	Malignant neoplasm of accessory sinus NOS	Feb-09
55268	30	2	0	0	BBK2100	[M]Myosarcoma	Feb-09
55292	10	0	0	0	B326z00	Malignant melanoma of upper limb or shoulder NOS	Feb-09
55303	23	0	0	0	B614100	"Hodgkin's nodular sclerosis of head, face and neck"	Feb-09
55374	81	0	0	0	B215.00	Malignant neoplasm of epiglottis NOS	Feb-09
55434	42	0	0	0	B116.00	Malignant neoplasm of greater curve of stomach unspecified	Feb-09
55468	7	0	0	0	BB5R600	"[M]Mucocarcinoid tumour, malignant"	Feb-09
55550	34	2	0	0	B331100	Malignant neoplasm of upper eyelid	Feb-09
55588	14	0	0	0	Byu7300	"[X]Malignant neoplasm of female genital organ, unspecified"	Feb-09
55595	7	0	0	0	B300700	Malignant neoplasm of sphenoid bone	Feb-09
55630	8	0	0	0	B07y.00	Malignant neoplasm of other specified site of nasopharynx	Feb-09
55634	11	0	0	0	Q442.00	Neonatal myasthenia gravis	Feb-09
55636	16	0	0	0	F130100	Hallervorden-Spatz disease	Feb-09
55670	61	1	0	0	B333200	Malignant neoplasm of skin of eyebrow	Feb-09
55850	G558.00	7	0	0	0	Cardiomyopathy in disease EC	Feb-09
55881	88	0	0	0	B324000	Malignant melanoma of scalp	Feb-09
55946	7	0	0	0	B574000	Secondary malignant neoplasm of duodenum	Feb-09
55947	34	4	0	0	BBJ7.00	[M]Pleomorphic liposarcoma	Feb-09
55953	5	0	0	0	B300400	Malignant neoplasm of occipital bone	Feb-09
56041	10	0	0	0	BBj1.00	"[M]Hodgkin's disease, lymphocytic predominance"	Feb-09
56121	94	0	0	0	Byu4300	"[X]Malignant neoplasm of skin, unspecified"	Feb-09

For peer review only - http://bmjopen.bmj.com/site/about/guidelines.xhtml

56288	F10..00	304	0	1	0	Cerebral degenerations usually manifest in childhood	Feb-09
56345	49	0	0	0	B57y.00	Secondary malignant neoplasm of other digestive organ	Feb-09
56355	8	1	0	0	B066.00	Malignant neoplasm of lateral wall of oropharynx	Feb-09
56362	P105.00	8	0	0	0	Spina bifida with stenosis of aqueduct of Sylvius	Feb-09
56490	14	0	0	0	B52z.00	Malignant neoplasm of nervous system NOS	Feb-09
56513	111	2	0	0	B307000	Malignant neoplasm of femur	Feb-09
56540	9	2	0	0	D40y400	Leukaemoid reaction	Feb-09
56600	108	0	0	0	BB2A.11	[M]Epidermoid carcinoma NOS	Feb-09
56676	167	9	0	0	BBJ5.00	[M]Myxoid liposarcoma	Feb-09
56709	5	0	0	0	B04y.00	Malignant neoplasm of other sites of floor of mouth	Feb-09
56715	77	1	0	0	B34y.00	Malignant neoplasm of other site of female breast	Feb-09
56718	15	0	0	0	B500z00	Malignant neoplasm of eyeball NOS	Feb-09
56918	67	0	0	0	B1zy.00	Malignant neoplasm other spec digestive tract and peritoneum	Feb-09
56925	36	0	0	0	Byu4000	[X]Malignant melanoma of other+unspecified parts of face	Feb-09
56951	24	0	0	0	F101200	Spielmeyer-Vogt (Batten) disease	Feb-09
56954	13	1	0	0	B337200	Malignant neoplasm of skin of knee	Feb-09
57047	12	0	0	0	B544.00	Malignant neoplasm of carotid body	Feb-09
57084	6	0	0	0	BBQ1000	"[M]Seminoma, anaplastic type"	Feb-09
57113	8	0	0	0	P100300	Lumbar spina bifida with hydrocephalus	Feb-09
57139	39	1	0	0	C30y800	Glutaric aciduria Type 1	Feb-09
57184	4	0	0	0	Byu4200	[X]Oth malignant neoplasm/skin of oth+unspecfd parts of face	Feb-09

For peer review only - http://bmjopen.bmj.com/site/about/guidelines.xhtml

57191	4	2	0	0	Byu8000	[X]Malignant neoplasm/other specified male genital organs	Feb-09
57225	7	0	0	0	B614000	"Hodgkin's disease, nodular sclerosis of unspecified site"	Feb-09
57235	25	0	0	0	B410000	Malignant neoplasm of endocervical canal	Feb-09
57243	2	1	0	0	P102200	Thoracic spina bifida with hydrocephalus - open	Feb-09
57248	9	0	0	0	B082.00	"Malignant neoplasm aryepiglottic fold, hypopharyngeal aspect"	Feb-09
57260	37	1	0	0	B322.00	Malignant melanoma of ear and external auricular canal	Feb-09
57316	34	0	0	0	BBr6600	[M]Acute promyelocytic leukaemia	Feb-09
57336	51	2	0	0	BB16.00	"[M]Epithelioma, malignant"	Feb-09
57422	79	1	0	0	BB5Vz00	[M]Pituitary adenoma or carcinoma NOS	Feb-09
57427	30	1	0	0	B62y000	Malignant lymphoma NOS of unspecified site	Feb-09
57442	534	1	0	0	B337.00	Malignant neoplasm of skin of lower limb and hip	Feb-09
57446	380	1	0	0	B335.00	"Malignant neoplasm of skin of trunk, excluding scrotum"	Feb-09
57481	41	0	0	0	ByuC300	[X]Secondary malignant neoplasm/oth+unspc respiratory organs	Feb-09
57482	30	1	0	0	B311200	Malignant neoplasm of connective and soft tissue of fore-arm	Feb-09
57505	15	0	0	0	BBK3200	[M]Pleomorphic rhabdomyosarcoma	Feb-09
57513	6	0	0	0	BB2C.11	"[M]Epidermoid carcinoma, keratinising type"	Feb-09
57544	6	0	0	0	BBm4.00	[M]True histiocytic lymphoma	Feb-09
57551	2	0	0	0	F381100	Myasthenic syndrome due to other malignancy	Feb-09
57611	P114z00	14	0	0	0	Meningomyelocele NOS	Feb-09

For peer review only - http://bmjopen.bmj.com/site/about/guidelines.xhtml

57632	21	0	0	0	F151z00	Spinal muscular atrophy NOS	Feb-09
57671	7	0	0	0	B672.00	Megakaryocytic leukaemia	Feb-09
57677	36	1	0	0	BBL8.00	[M]Hepatoblastoma	Feb-09
57680	2	0	0	0	BB2A.12	[M]Spinous cell carcinoma	Feb-09
57713	3	0	0	0	BBr8.00	[M]Eosinophilic leukaemias	Feb-09
57719	4	0	0	0	B41y100	Malignant neoplasm of squamocolumnar junction of cervix	Feb-09
57729	6	0	0	0	BBU1.00	[M]Lymphangiosarcoma	Feb-09
57737	5	0	0	0	B62x100	Lymphoepithelioid lymphoma	Feb-09
57756	10	0	0	0	Byu7100	[X]Malignant neoplasm/other specified female genital organs	Feb-09
57796	5	1	0	0	BBN4.00	"[M]Synovial sarcoma, biphasic type"	Feb-09
57802	16	1	0	0	BB5S400	[M]Alveolar adenocarcinoma	Feb-09
57854	6	0	0	0	B553000	Malignant neoplasm of inguinal region NOS	Feb-09
57988	2	0	0	0	B305000	Malignant neoplasm of carpal bone - scaphoid	Feb-09
58015	10	0	0	0	BBgQ.00	[M]Malignant lymphomatous polyposis	Feb-09
58061	15	4	0	0	B452.00	Malignant neoplasm of labia minora	Feb-09
58082	5	0	0	0	B620800	Nodular lymphoma of lymph nodes of multiple sites	Feb-09
58088	11	0	0	0	B151400	Malignant neoplasm of intrahepatic gall duct	Feb-09
58094	21	6	0	0	B412.00	"Malignant neoplasm, overlapping lesion of cervix uteri"	Feb-09
58121	6	0	0	0	B014.00	Malignant neoplasm of anterior 2/3 of tongue unspecified	Feb-09
58131	13	1	0	0	BB93.00	[M]Comedocarcinoma NOS	Feb-09
58601	51	2	0	0	B337100	Malignant neoplasm of skin of thigh	Feb-09
58635	29	0	0	0	PG51300	Osteogenesis imperfecta type I	Feb-09
58671	1	0	0	0	K0A5300	Heredtry nephrpthy NEC difus mesangial prolif glomnephrit	Feb-09

For peer review only - http://bmjopen.bmj.com/site/about/guidelines.xhtml

58684	2	0	0	0	B615200	Hodgkin's mixed cellularity of intrathoracic lymph nodes	Feb-09
58798	3	0	0	0	BB47.00	"[M]Transitional cell carcinoma, spindle cell type"	Feb-09
58835	28	1	0	0	BBE1100	"[M]Desmoplastic melanoma, malignant"	Feb-09
58837	14	0	0	0	BBF5.00	[M]Small cell sarcoma	Feb-09
58871	9	0	0	0	B623z00	Malignant histiocytosis NOS	Feb-09
58903	32	0	0	0	B550z00	"Malignant neoplasm of head, neck and face NOS"	Feb-09
58938	G55y000	29	0	0	0	Cardiomyopathy due to drugs and other external agents	Feb-09
58949	8	0	0	0	B308D00	Malignant neoplasm of phalanges of foot	Feb-09
58958	23	0	0	0	B323500	Malignant melanoma of temple	Feb-09
58962	4	0	0	0	B62x500	Malignant immunoproliferative small intestinal disease	Feb-09
58973	39	1	0	0	Byu0.00	"[X]Malignant neoplasm of lip, oral cavity and pharynx"	Feb-09
59001	49	0	0	0	C310112	Pompe's disease	Feb-09
59004	4	0	0	0	B072.00	Malignant neoplasm of lateral wall of nasopharynx	Feb-09
59035	9	0	0	0	F100z00	Leucodystrophy NOS	Feb-09
59036	24	0	0	0	B300.00	Malignant neoplasm of bones of skull and face	Feb-09
59041	33	0	0	0	B500000	Malignant neoplasm of ciliary body	Feb-09
59061	30	0	0	0	B322000	Malignant melanoma of auricle (ear)	Feb-09
59092	24	0	0	0	B111z00	Malignant neoplasm of pylorus of stomach NOS	Feb-09
59097	4	0	0	0	B431000	Malignant neoplasm of lower uterine segment	Feb-09
59115	4	0	0	0	B602100	"Burkitt's lymphoma of lymph nodes of head, face and neck"	Feb-09

For peer review only - http://bmjopen.bmj.com/site/about/guidelines.xhtml

59143	6	0	0	0	BB2D.00	"[M]Squamous cell carcinoma, large cell, non-keratinising"	Feb-09
59152	8	0	0	0	B315200	Malignant neoplasm of connective and soft tissue of perineum	Feb-09
59155	6	0	0	0	Fy04.11	Ondine's curse	Feb-09
59170	18	0	0	0	B51y000	Malignant neoplasm of corpus callosum	Feb-09
59218	18	2	0	0	P10y000	Dandy - Walker syndrome with spina bifida	Feb-09
59223	7	1	0	0	B306100	Malignant neoplasm of ischium	Feb-09
59240	19	0	0	0	BB58.00	"[M]Carcinoma, diffuse type"	Feb-09
59251	17	0	0	0	BBM9.00	"[M]Cystosarcoma phyllodes, malignant"	Feb-09
59284	12	0	0	0	BB82114	[M]Mucous adenocarcinoma	Feb-09
59286	6	0	0	0	B4Ay000	Malignant neoplasm of overlapping lesion of urinary organs	Feb-09
59310	16	1	0	0	BBV1.12	[M]Osteochondrosarcoma	Feb-09
59343	11	4	0	0	C302512	Oculocerebrorenal dystrophy	Feb-09
59362	13	0	0	0	B451z00	Malignant neoplasm of labia majora NOS	Feb-09
59375	27	0	0	0	B583z00	Secondary malignant neoplasm of brain or spinal cord NOS	Feb-09
59381	25	1	0	0	B500100	Malignant neoplasm of iris	Feb-09
59382	76	11	0	0	B310000	Malignant neoplasm of soft tissue of head	Feb-09
59383	11	0	0	0	G240000	Secondary malignant renovascular hypertension	Feb-09
59388	1	0	0	0	B18y100	Malignant neoplasm of mesocaecum	Feb-09
59415	27	0	0	0	BBB6100	"[M]Thymoma, malignant"	Feb-09
59520	2	0	0	0	B300200	Malignant neoplasm of malar bone	Feb-09
59651	4	0	0	0	BBJ8.00	[M]Mixed type liposarcoma	Feb-09
59755	18	0	0	0	B61z200	Hodgkin's disease NOS of intrathoracic lymph nodes	Feb-09
59778	28	0	0	0	B61z100	"Hodgkin's disease NOS of lymph nodes of head, face and neck"	Feb-09

For peer review only - http://bmjopen.bmj.com/site/about/guidelines.xhtml

59823	1	0	0	0	B542.00	Malignant neoplasm pituitary gland and craniopharyngeal duct	Feb-09
59831	25	0	0	0	B340z00	Malignant neoplasm of nipple or areola of female breast NOS	Feb-09
59855	44	0	0	0	F100300	Metachromatic leucodystrophy	Feb-09
59918	5	0	0	0	BB5f200	"[M]Follicular adenocarcinoma, well differentiated type"	Feb-09
59929	3	0	0	0	BBr0z00	"[M]Leukaemia unspecified, NOS"	Feb-09
59956	F10z.00	10	0	0	0	Childhood cerebral degenerations NOS	Feb-09
60035	23	3	0	0	B310300	Malignant neoplasm of cartilage of ear	Feb-09
60045	30	0	0	0	BB5M100	[M]Tubular adenocarcinoma	Feb-09
60052	114	2	0	0	B55yz00	Malignant neoplasm of specified site NOS	Feb-09
60079	8	0	0	0	PJ63z11	Bonnevie-Ullrich syndrome NOS	Feb-09
60092	16	0	0	0	B62y700	Malignant lymphoma NOS of spleen	Feb-09
60127	6	0	0	0	BBJ5.12	[M]Myxoliposarcoma	Feb-09
60134	2	0	0	0	B581000	Secondary malignant neoplasm of ureter	Feb-09
60162	20	0	0	0	Byu5A00	[X]Malignant neoplasm overlapping lesion of skin	Feb-09
60165	4	1	0	0	PFy4.00	Other arthrogryposis syndromes	Feb-09
60242	1	0	0	0	B600000	Reticulosarcoma of unspecified site	Feb-09
60275	8	0	0	0	BBgJ.00	"[M]Malignant lymphoma, centroblastic type NOS"	Feb-09
60312	10	0	0	0	B16y.00	Malignant neoplasm other gallbladder/extrahepatic bile duct	Feb-09
60335	21	1	0	0	B58y400	Secondary malignant neoplasm of vulva	Feb-09
60347	8	0	0	0	BBd2.11	[M]Leptomeningeal sarcoma	Feb-09
60403	8	0	0	0	B303300	Malignant neoplasm of costal cartilage	Feb-09
60504	2	0	0	0	BBgC.12	[M]Lymphocytic lymphosarcoma NOS	Feb-09
60526	12	0	0	0	B336z00	Malignant neoplasm of skin of upper limb or shoulder NOS	Feb-09
60623	3	0	0	0	P102400	Sacral spina bifida with hydrocephalus - open	Feb-09

For peer review only - http://bmjopen.bmj.com/site/about/guidelines.xhtml

60631	13	0	0	0	BBV5.00	[M]Osteosarcoma in Paget's disease of bone	Feb-09
60673	2	0	0	0	PKyz000	"Ullrich - Feichtiger syndrome, chimaera"	Feb-09
60772	37	0	0	0	B450z00	Malignant neoplasm of vagina NOS	Feb-09
60775	11	1	0	0	BB5h100	[M]Adrenal cortical carcinoma	Feb-09
60918	1	0	25	0	4M20.00	Lymphoma stage I	Feb-09
61064	28	2	0	0	B24X.00	"Malignant neoplasm of mediastinum, part unspecified"	Feb-09
61100	10	0	0	0	P51y.11	Transposition of aorta	Feb-09
61149	3	0	0	0	B614300	Hodgkin's nodular sclerosis of intra-abdominal lymph nodes	Feb-09
61194	14	0	0	0	B337z00	Malignant neoplasm of skin of lower limb or hip NOS	Feb-09
61246	14	0	0	0	B327600	Malignant melanoma of heel	Feb-09
61251	2	0	0	0	BBgN.00	"[M]Malign lymphoma,lymphocytic,intermediate differn, diffuse"	Feb-09
61390	10	0	0	0	B540000	Malignant neoplasm of adrenal cortex	Feb-09
61399	29	0	0	0	B510100	Malignant neoplasm of cerebral cortex	Feb-09
61467	1	0	0	0	BB5f300	"[M]Follicular adenocarcinoma, trabecular type"	Feb-09
61500	9	0	0	0	B690.00	Acute myelomonocytic leukaemia	Feb-09
61510	2	1	0	0	B062200	Malignant neoplasm of palatoglossal arch	Feb-09
61542	31	0	0	0	BBQ7400	"[M]Malignant teratoma, undifferentiated type"	Feb-09
61555	11	0	0	0	B180z00	Malignant neoplasm of retroperitoneum NOS	Feb-09
61588	8	0	0	0	BB85000	[M]Signet ring cell carcinoma	Feb-09
61643	24	0	0	0	B151z00	Malignant neoplasm of intrahepatic bile ducts NOS	Feb-09
61650	2	1	0	0	C375200	"Mucopolysaccharidosis, type II"	Feb-09

For peer review only - http://bmjopen.bmj.com/site/about/guidelines.xhtml

61655	1	0	0	0	ZV10211	[V]Personal history of malignant neoplasm - accessory sinus	Feb-09
61662	12	0	0	0	B61z000	"Hodgkin's disease NOS, unspecified site"	Feb-09
61663	8	2	0	0	ZV76500	[V]Screening for malignant neoplasm of oral cavity	Feb-09
61692	2	0	0	0	B004.00	"Malignant neoplasm of lip unspecified, inner aspect"	Feb-09
61693	16	0	0	0	ByuD600	[X]Other myeloid leukaemia	Feb-09
61695	22	1	0	0	B100.00	Malignant neoplasm of cervical oesophagus	Feb-09
61716	4	0	0	0	B524100	"Malignant neoplasm of peripheral nerve,upp limb,incl should"	Feb-09
61741	50	1	0	0	B304200	Malignant neoplasm of humerus	Feb-09
61769	3	0	0	0	PKyC.00	Pena-Shokeir syndrome type I	Feb-09
61783	22	0	0	0	BBbG.11	[M]Juvenile astrocytoma	Feb-09
61930	314	6	0	0	Kyu2.00	[X]Renal failure	Feb-09
61984	9	0	0	0	BB1G.00	[M]Spheroidal cell carcinoma	Feb-09
61997	26	0	0	0	BBj0.00	[M]Hodgkin's disease NOS	Feb-09
62080	10	2	0	0	B332100	Malignant neoplasm of skin of external auditory meatus	Feb-09
62088	44	2	0	0	BBEG.00	[M]Malignant melanoma in Hutchinson's melanotic freckle	Feb-09
62104	9	0	0	0	B300800	Malignant neoplasm of temporal bone	Feb-09
62126	19	0	0	0	B510500	Malignant neoplasm of thalamus	Feb-09
62169	8	0	0	0	P51y.00	Other specified transposition of great vessels	Feb-09
62182	16	0	0	0	B200300	Malignant neoplasm of vestibule of nose	Feb-09
62199	8	0	0	0	BB5W.00	[M]Oxyphilic adenomas and adenocarcinomas	Feb-09
62305	6	0	0	0	B335800	Malignant neoplasm of skin of buttock	Feb-09
62330	2	0	0	0	BBr6z00	[M]Other myeloid leukaemia NOS	Feb-09
62348	10	1	0	0	BBT1.00	[M]Haemangiosarcoma	Feb-09

For peer review only - http://bmjopen.bmj.com/site/about/guidelines.xhtml

62376	P722500	11	0	0	0	Atresia of aorta	Feb-09
62380	2	0	0	0	B601200	Lymphosarcoma of intrathoracic lymph nodes	Feb-09
62395	44	0	0	0	C306100	Citrullinaemia	Feb-09
62396	27	0	0	0	BBF6.00	[M]Epithelioid cell sarcoma	Feb-09
62437	7	0	0	0	B62x400	Malignant reticulosis	Feb-09
62475	17	0	0	0	B326300	Malignant melanoma of hand	Feb-09
62556	13	0	0	0	B24..00	"Malignant neoplasm of thymus, heart and mediastinum"	Feb-09
62567	4	0	0	0	BBc0200	[M]Ganglioneuromatosis	Feb-09
62584	32	0	0	0	B573.00	Secondary malignant neoplasm of other respiratory organs	Feb-09
62630	2	0	0	0	B307z00	Malignant neoplasm of long bones of leg NOS	Feb-09
62644	14	2	0	0	C372011	Lesch - Nyhan syndrome	Feb-09
62761	22	0	0	0	B200200	Malignant neoplasm of septum of nose	Feb-09
62762	5	0	0	0	C375600	"Mucopolysaccharidosis, type VI"	Feb-09
62828	10	0	0	0	B581z00	Secondary malignant neoplasm of other urinary organ NOS	Feb-09
62840	18	0	0	0	B013.00	Malignant neoplasm of ventral surface of tongue	Feb-09
62871	2	0	0	0	BB92.00	"[M]Comedocarcinoma, noninfiltrating"	Feb-09
62909	26	1	0	0	B575100	Secondary malignant neoplasm of rectum	Feb-09
62941	10	1	0	0	BBe2.00	[M]Neurofibrosarcoma	Feb-09
62951	3	0	28	0	5A15.00	Bone tumour/metast.irradiat.	Feb-09
63054	47	0	0	0	B614z00	"Hodgkin's disease, nodular sclerosis NOS"	Feb-09
63102	6	0	0	0	BB5B100	[M]Islet cell carcinoma	Feb-09
63104	9	0	0	0	B501z00	Malignant neoplasm of orbit NOS	Feb-09
63105	10	0	0	0	B62y500	Malignant lymphoma NOS of lymph node inguinal region and leg	Feb-09

For peer review only - http://bmjopen.bmj.com/site/about/guidelines.xhtml

63224	10	0	0	0	B48z.00	Malignant neoplasm of penis and other male genital organ NOS	Feb-09
63239	7	0	0	0	BBm1.00	[M]Malignant histiocytosis	Feb-09
63247	3	0	0	0	BBK3611	[M]Sarcoma botryoides	Feb-09
63286	3	0	0	0	BBN5.00	[M]Clear cell sarcoma of tendons and aponeuroses	Feb-09
63300	1	0	0	0	Byu3200	[X]Malignant neoplasm/overlap lesion/bone+articulr cartilage	Feb-09
63331	5	0	0	0	B485.00	Malignant neoplasm of spermatic cord	Feb-09
63359	7	0	0	0	C315000	Pyruvate dehydrogenase deficiency	Feb-09
63375	47	0	0	0	ByuDE00	[X]Unspecified B-cell non-Hodgkin's lymphoma	Feb-09
63376	15	0	0	0	F402100	Malignant myopia	Feb-09
63390	65	0	0	0	P512.00	Corrected great vessel transposition	Feb-09
63430	7	0	0	0	B241000	Malignant neoplasm of endocardium	Feb-09
63460	6	0	0	0	B213000	Malignant neoplasm of arytenoid cartilage	Feb-09
63470	34	0	0	0	B102.00	Malignant neoplasm of abdominal oesophagus	Feb-09
63475	4	0	0	0	B652.00	Subacute myeloid leukaemia	Feb-09
63518	11	0	0	0	BBLE.00	[M]Adenosarcoma	Feb-09
63568	7	0	0	0	B524000	"Malignant neoplasm of peripheral nerves of head, face & neck"	Feb-09
63570	3	0	0	0	BBr0113	[M]Stem cell leukaemia	Feb-09
63571	18	0	0	0	BBV..12	[M]Parosteal osteosarcoma	Feb-09
63574	11	0	0	0	BBEC.00	[M]Malignant melanoma in junctional naevus	Feb-09
63598	26	0	0	0	ByuE.00	[X]Malignant neoplasms/independent (primary) multiple sites	Feb-09
63625	0	1	0	0	B616400	Hodgkin's lymphocytic depletion lymph nodes axilla and arm	Feb-09
63634	5	1	0	0	PD00000	Bilateral renal agenesis	Feb-09

For peer review only - http://bmjopen.bmj.com/site/about/guidelines.xhtml

63652	5	0	0	0	F103000	Cerebral degeneration in Hunter's disease	Feb-09
63657	37	1	0	0	B503.00	Malignant neoplasm of conjunctiva	Feb-09
63659	1	0	0	0	BBW6.00	[M]Juxtacortical chondrosarcoma	Feb-09
63695	3	0	0	0	B524300	Malignant neoplasm of peripheral nerve of thorax	Feb-09
63699	15	1	0	0	BBk0.00	"[M]Malignant lymphoma, nodular NOS"	Feb-09
63723	12	0	0	0	B601z00	Lymphosarcoma NOS	Feb-09
63786	24	0	0	0	K01w.00	Congenital nephrotic syndrome	Feb-09
63896	5	0	0	0	B582400	Secondary malignant neoplasm of skin of shoulder and arm	Feb-09
63925	19	0	0	0	ByuA200	"[X]Malignant neoplasm of meninges, unspecified"	Feb-09
63973	1	0	0	0	BBm0.00	[M]Microglioma	Feb-09
63979	7	0	0	0	B013100	Malignant neoplasm of frenulum linguae	Feb-09
63988	12	0	0	0	B311500	Malignant neoplasm of connective and soft tissue of thumb	Feb-09
63994	1	0	0	0	BBgS.00	"[M]Malignant lymphoma, large cell, cleaved, diffuse"	Feb-09
63995	3	0	0	0	B123.00	Malignant neoplasm of Meckel's diverticulum	Feb-09
63997	15	0	0	0	B326500	Malignant melanoma of thumb	Feb-09
64036	3	0	0	0	B612.00	Hodgkin's sarcoma	Feb-09
64106	46	0	0	0	B18yz00	Malignant neoplasm of specified parts of peritoneum NOS	Feb-09
64270	14	0	0	0	B337500	Malignant neoplasm of skin of ankle	Feb-09
64309	13	0	0	0	ByuB100	"[X]Malignant neoplasm of endocrine gland, unspecified"	Feb-09
64327	32	0	0	0	B327z00	Malignant melanoma of lower limb or hip NOS	Feb-09
64336	19	0	0	0	ByuD300	[X]Other specified types of non-Hodgkin's lymphoma	Feb-09

For peer review only - http://bmjopen.bmj.com/site/about/guidelines.xhtml

64343	10	0	0	0	BBj6100	"[M]Hodgkin,s disease, nodular sclerosis, mixed cellularity"	Feb-09
64345	22	0	0	0	B311100	"Malignant neoplasm of connective and soft tissue, upper arm"	Feb-09
64406	5	0	0	0	B336500	Malignant neoplasm of skin of thumb	Feb-09
64462	17	0	0	0	B083.00	Malignant neoplasm of posterior pharynx	Feb-09
64470	11	0	0	0	7904y00	Other correction of transposition of great vessels OS	Feb-09
64497	8	0	0	0	Byu7000	"[X]Malignant neoplasm of uterine adnexa, unspecified"	Feb-09
64515	4	0	0	0	ByuDC00	"[X]Diffuse non-Hodgkin's lymphoma, unspecified"	Feb-09
64516	9	0	0	0	B18y400	Malignant neoplasm of parietal peritoneum	Feb-09
64557	4	1	0	0	B517000	Malignant neoplasm of cerebral peduncle	Feb-09
64596	14	0	0	0	BBK0700	[M]Myxoid leiomyosarcoma	Feb-09
64602	17	7	0	0	B470.00	Malignant neoplasm of undescended testis	Feb-09
64618	4	0	0	0	BBr3.00	[M]Plasma cell leukaemias	Feb-09
64625	5	0	0	0	D200111	Fanconi's hypoplastic anaemia	Feb-09
64670	1	0	0	0	B601300	Lymphosarcoma of intra-abdominal lymph nodes	Feb-09
64680	7	0	0	0	B574.00	Secondary malignant neoplasm of small intestine and duodenum	Feb-09
64686	29	0	0	0	B340100	Malignant neoplasm of areola of female breast	Feb-09
64690	34	1	0	0	F390.00	Congenital hereditary muscular dystrophy	Feb-09
64717	24	0	0	0	P10z.00	Spina bifida with hydrocephalus NOS	Feb-09
64796	16	1	0	0	BB82z00	[M]Mucinous adenoma or adenocarcinoma NOS	Feb-09
64810	5	0	0	0	B551z00	Malignant neoplasm of thorax NOS	Feb-09
64817	7	0	0	0	B502.00	Malignant neoplasm of lacrimal gland	Feb-09

For peer review only - http://bmjopen.bmj.com/site/about/guidelines.xhtml

64837	3	2	0	0	G558200	Dystrophic cardiomyopathy	Feb-09
64848	4	0	0	0	B304400	Malignant neoplasm of ulna	Feb-09
64874	22	1	0	0	BBa4.00	[M]Melanotic neuroectodermal tumour	Feb-09
64897	19	0	0	0	ByuE000	[X]Malignant neoplasms/independent(primary)multiple sites	Feb-09
64963	11	0	0	0	BBr0112	[M]Blastic leukaemia	Feb-09
64971	2	0	0	0	B520000	Malignant neoplasm of olfactory bulb	Feb-09
65047	12	0	0	0	BBDA.00	"[M]Phaeochromocytoma, malignant"	Feb-09
65051	4	0	0	0	BB81500	"[M]Papillary cystadenocarcinoma, NOS"	Feb-09
65091	10	0	0	0	PJ33211	18q- syndrome	Feb-09
65106	26	0	0	0	B44z.00	Malignant neoplasm of uterine adnexa NOS	Feb-09
65117	9	0	0	0	A789900	HIV disease resulting in lymphoid interstitial pneumonitis	Feb-09
65121	8	0	0	0	PHz..11	Congenital ectodermal defect	Feb-09
65122	4	0	0	0	B624000	Leukaemic reticuloendotheliosis of unspecified sites	Feb-09
65123	1	0	0	0	B624300	Leukaemic reticuloend of intra-abdominal lymph nodes	Feb-09
65124	5	0	0	0	B151000	Malignant neoplasm of interlobular bile ducts	Feb-09
65159	4	0	0	0	B180100	Malignant neoplasm of perinephric tissue	Feb-09
65164	169	2	0	0	B326.00	Malignant melanoma of upper limb and shoulder	Feb-09
65165	1	0	0	0	ByuD900	[X]Other leukaemia of unspecified cell type	Feb-09
65180	14	0	0	0	B627800	Diffuse non-Hodgkin's lymphoma undifferentiated (diffuse)	Feb-09
65207	6	0	0	0	BB80z00	[M]Cystadenoma or carcinoma NOS	Feb-09
65215	17	0	0	0	B205.00	Malignant neoplasm of sphenoidal sinus	Feb-09
65216	11	0	0	0	BB49.00	[M]Cloacogenic carcinoma	Feb-09

For peer review only - http://bmjopen.bmj.com/site/about/guidelines.xhtml

65241	4	2	0	0	B51y200	"Malignant neoplasm, overlapping lesion of brain"	Feb-09
65246	P118z00	1	0	0	0	Spina bifida without hydrocephalus - closed NOS	Feb-09
65253	3	1	0	0	B560300	Secondary and unspec malignant neoplasm occipital lymph node	Feb-09
65312	6	0	0	0	B11y000	Malignant neoplasm of anterior wall of stomach NEC	Feb-09
65343	1	0	0	0	F102100	Cerebral degeneration in Niemann-Pick disease	Feb-09
65344	9	0	0	0	C370000	Cystic fibrosis with no meconium ileus	Feb-09
65357	1	0	0	0	B507100	Malignant neoplasm of nasolacrimal duct	Feb-09
65372	14	0	0	0	B11yz00	Malignant neoplasm of other specified site of stomach NOS	Feb-09
65405	5	0	0	0	N237300	Pseudosarcomatous fibromatosis	Feb-09
65434	8	0	0	0	B62z.00	Malignant neoplasms of lymphoid and histiocytic tissue NOS	Feb-09
65460	21	1	0	0	B1z1.00	Malignant neoplasm of spleen NEC	Feb-09
65466	2	0	0	0	B592X00	Kaposi's sarcoma of multiple organs	Feb-09
65483	3	0	0	0	B614400	Hodgkin's nodular sclerosis of lymph nodes of axilla and arm	Feb-09
65489	3	0	0	0	B610.00	Hodgkin's paragranuloma	Feb-09
65490	2	0	0	0	B58y411	Secondary cancer of the vulva	Feb-09
65509	5	0	0	0	PJ50300	Trisomy 9	Feb-09
65584	4	0	0	0	BBj1000	"[M]Hodgkin,s disease, lymphocytic predominance, diffuse"	Feb-09
65599	10	0	0	0	B520200	Malignant neoplasm of acoustic nerve	Feb-09
65605	2	0	0	0	B241200	Malignant neoplasm of myocardium	Feb-09
65625	178	0	0	0	B324.00	Malignant melanoma of scalp and neck	Feb-09
65642	2	0	0	0	B623300	Malignant histiocytosis of intra-abdominal lymph nodes	Feb-09
65701	31	1	0	0	B620z00	Nodular lymphoma NOS	Feb-09

For peer review only - http://bmjopen.bmj.com/site/about/guidelines.xhtml

65721	2	0	0	0	B673.00	Mast cell leukaemia	Feb-09
65777	8	1	0	0	B672.11	Thrombocytic leukaemia	Feb-09
65778	F4Gy011	6	0	0	0	Encephalocoele of orbit	Feb-09
65782	4	0	0	0	B337800	Malignant neoplasm of skin of toe	Feb-09
65861	6	0	0	0	BBQ9.00	[M]Dermoid cyst with malignant transformation	Feb-09
65935	PG43.00	7	0	0	0	Asphyxiating thoracic dysplasia	Feb-09
65952	10	0	0	0	BBbU.00	[M]Desmoplastic medulloblastoma	Feb-09
66000	6	0	0	0	BBB5.00	[M]Adenocarcinoma with apocrine metaplasia	Feb-09
66019	6	0	0	0	L254.11	Suspect fetal damage from maternal alcohol	Feb-09
66064	13	1	0	0	BBbM.00	[M]Giant cell glioblastoma	Feb-09
66073	14	0	0	0	C392400	Severe combined immunodef with low T- and B-cell numbers	Feb-09
66089	6	0	0	0	B65yz00	Other myeloid leukaemia NOS	Feb-09
66155	12	0	0	0	C306200	Argininosuccinic aciduria	Feb-09
66163	11	0	0	0	ByuC200	[X]2ndry+unspcf malignant neoplasm lymph nodes/multi regions	Feb-09
66166	5	0	0	0	B124.00	"Malignant neoplasm, overlapping lesion of small intestine"	Feb-09
66270	11	0	0	0	B000000	"Malignant neoplasm of upper lip, external"	Feb-09
66319	5	1	0	0	B335500	Malignant neoplasm of skin of groin	Feb-09
66327	13	0	0	0	B620000	Nodular lymphoma of unspecified site	Feb-09
66367	10	0	0	0	A789700	HIV dis resulting oth types of non-Hodgkin's lymphoma	Feb-09
66368	6	0	0	0	A789100	HIV disease resulting in cytomegaloviral disease	Feb-09
66384	26	0	0	0	B001000	"Malignant neoplasm of lower lip, external"	Feb-09

For peer review only - http://bmjopen.bmj.com/site/about/guidelines.xhtml

66422	1	0	0	0	B074.00	"Malignant neoplasm, overlapping lesion of nasopharynx"	Feb-09
66444	2	0	0	0	Byu2100	"[X]Malignant neoplasm/overlap lesion/heart,mediastinm+pleura"	Feb-09
66447	13	0	0	0	B335A00	Malignant neoplasm of skin of scapular region	Feb-09
66499	23	0	0	0	P00z.00	Anencephalus NOS	Feb-09
66541	3	0	0	0	BB1J.12	[M]Round cell carcinoma	Feb-09
66603	12	0	0	0	BBgK.00	"[M]Malig lymphoma, follicular centre cell, non-cleaved NOS"	Feb-09
66607	6	0	0	0	BBL4.00	"[M]Mixed tumour, malignant, NOS"	Feb-09
66639	14	0	0	0	B303200	Malignant neoplasm of clavicle	Feb-09
66646	6	0	0	0	B26..00	"Malignant neoplasm, overlap lesion of resp & intrathor orgs"	Feb-09
66694	1	0	0	0	BBr6311	[M]Naegeli-type monocytic leukaemia	Feb-09
66699	4	0	0	0	PK83.00	Fetus and newborn affected by maternal use of alcohol	Feb-09
66750	4	0	0	0	B24z.00	"Malignant neoplasm of heart, thymus and mediastinum NOS"	Feb-09
66775	1	0	0	0	B560100	Secondary and unspec malignant neoplasm mastoid lymph nodes	Feb-09
66876	3	0	0	0	BB81E11	[M]Pseudomucinous adenocarcinoma	Feb-09
66908	5	2	0	0	B306400	Malignant neoplasm of coccygeal vertebra	Feb-09
67019	2	0	0	0	BBK1100	[M]Angiomyosarcoma	Feb-09
67029	12	0	0	0	ByuD500	[X]Other lymphoid leukaemia	Feb-09
67034	13	1	0	0	Byu5000	[X]Mesothelioma of other sites	Feb-09
67107	5	0	0	0	B230.00	Malignant neoplasm of parietal pleura	Feb-09
67203	7	0	0	0	BBgG.11	[M]Lymphoblastic lymphosarcoma NOS	Feb-09
67211	1	0	0	0	B523z00	Malignant neoplasm of spinal meninges NOS	Feb-09
67217	8	0	0	0	B55y100	Malignant neoplasm of trunk NOS	Feb-09

For peer review only - http://bmjopen.bmj.com/site/about/guidelines.xhtml

For peer review only

67234	8	0	0	0	PJ21.00	"Trisomy 18, mosaicism"	Feb-09
67236	2	0	0	0	B512000	Malignant neoplasm of hippocampus	Feb-09
67248	3	0	13	0	5A12.00	Thyroid tumour/metast irradiat	Feb-09
67323	25	3	0	0	B06y.00	"Malignant neoplasm of oropharynx, other specified sites"	Feb-09
67339	1	0	0	0	BBp2.00	[M]Malignant mastocytosis	Feb-09
67342	8	0	0	0	BB5U100	[M]Adenocarcinoma in villous adenoma	Feb-09
67351	P113100	15	0	0	0	Cervical spinal meningocele	Feb-09
67446	10	0	0	0	B001.00	"Malignant neoplasm of lower lip, vermilion border"	Feb-09
67451	2	0	0	0	B30W.00	Malignant neoplasm/overlap lesion/bone+articulr cartilage	Feb-09
67497	6	0	0	0	B106.00	"Malignant neoplasm, overlapping lesion of oesophagus"	Feb-09
67504	4	1	0	0	B003000	"Malignant neoplasm of lower lip, buccal aspect"	Feb-09
67506	9	0	0	0	B614200	Hodgkin's nodular sclerosis of intrathoracic lymph nodes	Feb-09
67518	4	0	0	0	ByuD100	[X]Other types of follicular non-Hodgkin's lymphoma	Feb-09
67575	10	0	0	0	A788W00	HIV disease resulting in unspecified malignant neoplasm	Feb-09
67587	18	0	0	0	BBbZ.00	[M]Pleomorphic xanthoastrocytoma	Feb-09
67700	4	0	0	0	B66..12	Monoblastic leukaemia	Feb-09
67701	35	0	0	0	BB94.11	[M]Secretory breast carcinoma	Feb-09
67703	8	0	0	0	B616.00	"Hodgkin's disease, lymphocytic depletion"	Feb-09
67712	8	0	0	0	BBR2.00	[M]Choriocarcinoma	Feb-09
67748	4	0	0	0	B335400	Malignant neoplasm of skin of umbilicus	Feb-09
67762	1	0	0	0	F103100	Cerebral degeneration in mucopolysaccharidoses	Feb-09

For peer review only - http://bmjopen.bmj.com/site/about/guidelines.xhtml

67763	2	0	0	0	B303400	Malignant neoplasm of costo-vertebral joint	Feb-09
67806	62	0	0	0	B323z00	Malignant melanoma of face NOS	Feb-09
67878	P112.00	1	0	0	0	Hydromyelocele	Feb-09
67884	4	0	0	0	B350100	Malignant neoplasm of areola of male breast	Feb-09
67906	10	5	0	0	F431400	Metastatic disseminated retinitis	Feb-09
67912	3	0	0	0	BB26.11	[M]Papillary epidermoid carcinoma	Feb-09
67913	7	1	0	0	BB6A.00	[M]Ceruminous adenoma and adenocarcinoma	Feb-09
67914	3	0	0	0	B337900	Malignant neoplasm of skin of great toe	Feb-09
67927	3	0	0	0	C372300	Lesch-Nyhan syndrome	Feb-09
67934	6	0	0	0	BBLA.00	"[M]Carcinosarcoma, embryonal type"	Feb-09
67949	10	0	0	0	B48y.00	Malignant neoplasm of other male genital organ	Feb-09
67966	3	0	0	0	BBE1.14	[M]Naevocarcinoma	Feb-09
67970	2	0	0	0	BB1L.00	"[M]Small cell carcinoma, fusiform cell type"	Feb-09
68027	4	0	0	0	ByuA000	[X]Malignant neoplasm/other and unspecified cranial nerves	Feb-09
68039	0	0	0	0	B612400	Hodgkin's sarcoma of lymph nodes of axilla and upper limb	Feb-09
68055	32	0	0	0	B307.00	Malignant neoplasm of long bones of leg	Feb-09
68087	42	1	0	0	D20z.00	Aplastic anaemia NOS	Feb-09
68133	21	0	0	0	B323300	Malignant melanoma of forehead	Feb-09
68155	7	0	0	0	B430100	Malignant neoplasm of fundus of corpus uteri	Feb-09
68161	23	0	0	0	B48y000	Malignant neoplasm of seminal vesicle	Feb-09
68181	2	1	0	0	P002.11	Hemianencephaly	Feb-09
68197	5	0	0	0	B337300	Malignant neoplasm of skin of popliteal fossa area	Feb-09
68220	2	0	0	0	BBW4.11	[M]Fibrochondrosarcoma	Feb-09

For peer review only - http://bmjopen.bmj.com/site/about/guidelines.xhtml

68221	P110100	2	0	0	0	Cervical spina bifida without mention of hydrocephalus	Feb-09
68236	3398	6	0	0	B550.00	"Malignant neoplasm of head, neck and face"	Feb-09
68330	8	0	0	0	B613100	"Hodgkin's, lymphocytic-histiocytic pred of head, face, neck"	Feb-09
68332	12	0	0	0	ByuC600	[X]2ndry malignant neoplasm/oth+unspec parts/nervous system	Feb-09
68399	1	0	0	0	B004200	"Malignant neoplasm of lip unspecified, mucosa"	Feb-09
68410	11	0	0	0	B150200	Primary angiosarcoma of liver	Feb-09
68447	8	0	0	0	BBEV.00	"[M]Blue naevus, malignant"	Feb-09
68456	25	0	0	0	BB5V100	[M]Chromophobe carcinoma	Feb-09
68479	4	3	0	0	BBc7.11	[M]Neuroastrocytoma	Feb-09
68480	7	1	0	0	B350000	Malignant neoplasm of nipple of male breast	Feb-09
68612	2	0	0	0	ZV10z00	[V]Personal history of unspecified malignant neoplasm	Feb-09
68641	8	0	0	0	B517z00	Malignant neoplasm of brain stem NOS	Feb-09
68730	25	0	0	0	BBZN.00	[M]Ameloblastic fibrosarcoma	Feb-09
68757	2	0	0	0	BB5f700	[M]Nonencapsulated sclerosing carcinoma	Feb-09
68783	2	0	0	0	BB60100	[M]Skin appendage carcinoma	Feb-09
68787	19	0	0	0	B55y000	Malignant neoplasm of back NOS	Feb-09
68808	11	0	0	0	BBb2.00	[M]Mixed glioma	Feb-09
68824	2	0	0	0	B48y200	"Malignant neoplasm, overlapping lesion male genital orgs"	Feb-09
68956	8	1	0	0	BBX1.00	"[M]Giant cell tumour of bone, malignant"	Feb-09
68964	2	0	0	0	BBgA.00	"[M]Malignant lymphoma, centroblastic-centrocytic, diffuse"	Feb-09

For peer review only - http://bmjopen.bmj.com/site/about/guidelines.xhtml

68999	1	1	0	0	BBK2z00	[M]Myoma or myosarcoma NOS	Feb-09
69017	10	0	0	0	C370100	Cystic fibrosis with meconium ileus	Feb-09
69027	15	0	0	0	D200.00	Constitutional aplastic anaemia	Feb-09
69061	3	0	0	0	D200011	Constitutional aplastic anaemia without malformation	Feb-09
69104	5	1	0	0	B305100	Malignant neoplasm of carpal bone - lunate	Feb-09
69146	7	0	0	0	B300z00	Malignant neoplasm of bones of skull and face NOS	Feb-09
69299	1	1	0	0	BBrA111	[M]Thrombocytic leukaemia	Feb-09
69300	5	0	0	0	BB1F.00	[M]Polygonal cell carcinoma	Feb-09
69301	1	0	0	0	BBg5.00	"[M]Malignant lymphoma, convoluted cell type NOS"	Feb-09
69381	10	0	0	0	C375100	"Mucopolysaccharidosis, type 1"	Feb-09
69421	3	0	0	0	BBDz.00	[M]Paraganglioma or glomus tumour NOS	Feb-09
69436	1	0	0	0	PG51500	Osteogenesis imperfecta type III	Feb-09
69476	14	0	0	0	PJ50200	Trisomy 8	Feb-09
69497	1	0	0	0	B623000	Malignant histiocytosis of unspecified site	Feb-09
69613	11	1	0	0	C377100	Mucolipidosis type III	Feb-09
69671	2	1	0	0	B010.11	Malignant neoplasm of posterior third of tongue	Feb-09
69761	4	0	0	0	B00zz00	"Malignant neoplasm of lip, vermilion border NOS"	Feb-09
69766	8	0	0	0	A788200	HIV infection with persistent generalised lymphadenopathy	Feb-09
69767	3	0	0	0	AyuC600	[X]HIV disease resulting in other non-Hodgkin's lymphoma	Feb-09
69821	11	1	0	0	B18y600	Malignant neoplasm of the pouch of Douglas	Feb-09
69844	11	0	0	0	BBF5.11	[M]Round cell sarcoma	Feb-09
69858	8	0	0	0	P510.00	Total great vessel transposition	Feb-09

For peer review only - http://bmjopen.bmj.com/site/about/guidelines.xhtml

69927	1	0	0	0	B308800	Malignant neoplasm of first metatarsal bone	Feb-09
69951	5	0	0	0	B055100	Malignant neoplasm of roof of mouth	Feb-09
69980	23	2	0	0	BBgC.00	"[M]Malignant lymphoma, lymphocytic, well differentiated NOS"	Feb-09
69981	10	1	0	0	BBe7.00	"[M]Neurilemmoma, malignant"	Feb-09
70104	6	0	0	0	B521z00	Malignant neoplasm of cerebral meninges NOS	Feb-09
70126	9	0	0	0	B520100	Malignant neoplasm of optic nerve	Feb-09
70320	8	0	0	0	BBD4.11	[M]Jugular paraganglioma	Feb-09
70374	1	0	0	0	B600300	Reticulosarcoma of intra-abdominal lymph nodes	Feb-09
70380	6	0	0	0	B335000	Malignant neoplasm of skin of axillary fold	Feb-09
70383	2	0	0	0	BBM0100	"[M]Brenner tumour, malignant"	Feb-09
70439	4	0	0	0	C375300	"Mucopolysaccharidosis, type III"	Feb-09
70463	16	0	0	0	B315000	Malignant neoplasm of connective and soft tissue of buttock	Feb-09
70509	5	0	0	0	B627D00	Diffuse non-Hodgkin's centroblastic lymphoma	Feb-09
70516	9	0	0	0	BB5D.11	[M]Biliary tract adenomas and adenocarcinomas	Feb-09
70572	4	0	0	0	F151000	Unspecified spinal muscular atrophy	Feb-09
70587	8	0	0	0	B337700	Malignant neoplasm of skin of foot	Feb-09
70637	11	0	0	0	B320.00	Malignant melanoma of lip	Feb-09
70696	3	0	0	0	B02y.00	Malignant neoplasm of other major salivary glands	Feb-09
70709	11	0	4	0	4L49.00	Prion protein markers for Creutzfeldt-Jakob disease	Feb-09
70724	7	1	0	0	B653.00	Myeloid sarcoma	Feb-09
70729	3	0	0	0	B431z00	Malignant neoplasm of isthmus of uterine body NOS	Feb-09

For peer review only - http://bmjopen.bmj.com/site/about/guidelines.xhtml

70736	7	0	0	0	B58y300	Secondary malignant neoplasm of vagina	Feb-09
70740	1	0	0	0	BBm1.11	[M]Malignant reticulosis	Feb-09
70819	26	0	0	0	B055.00	Malignant neoplasm of palate unspecified	Feb-09
70824	20	0	0	0	B540z00	Malignant neoplasm of adrenal gland NOS	Feb-09
70842	7	0	0	0	B627100	Follicular non-Hodg mixed sml cleavd & lge cell lymphoma	Feb-09
70855	7	0	0	0	G558000	Cardiomyopathy in Friedreich's ataxia	Feb-09
70928	5	0	0	0	B022.00	Malignant neoplasm of sublingual gland	Feb-09
70935	1	0	0	0	BBr4000	[M]Erythroleukaemia	Feb-09
70942	8	0	0	0	B510400	Malignant neoplasm of hypothalamus	Feb-09
70951	3	0	0	0	BBP0.00	"[M]Mesothelioma, benign"	Feb-09
70988	4	1	0	0	B337000	Malignant neoplasm of skin of hip	Feb-09
71031	1	0	0	0	B600100	"Reticulosarcoma of lymph nodes of head, face and neck"	Feb-09
71117	2	0	0	0	BBg3.00	"[M]Malignant lymphoma, undifferentiated cell type NOS"	Feb-09
71136	8	0	0	0	B323100	Malignant melanoma of chin	Feb-09
71139	26	0	0	0	B51y.00	Malignant neoplasm of other parts of brain	Feb-09
71142	3	0	0	0	B613000	"Hodgkin's, lymphocytic-histiocytic predominance unspec site"	Feb-09
71147	4	0	0	0	B003.00	"Malignant neoplasm of lower lip, inner aspect"	Feb-09
71204	6	1	0	0	B200000	Malignant neoplasm of cartilage of nose	Feb-09
71238	5	0	0	0	B601100	"Lymphosarcoma of lymph nodes of head, face and neck"	Feb-09
71262	4	0	0	0	B62y600	Malignant lymphoma NOS of intrapelvic lymph nodes	Feb-09
71301	5	0	0	0	BBQA100	"[M]Struma ovarii, malignant"	Feb-09
71304	14	0	0	0	B602z00	Burkitt's lymphoma NOS	Feb-09
71377	1	0	0	0	BBr8000	[M]Eosinophilic leukaemia	Feb-09

For peer review only - http://bmjopen.bmj.com/site/about/guidelines.xhtml

71450	2	0	0	0	A788X00	HIV disease resulting/unspcf infectious+parasitic disease	Feb-09
71497	4	0	0	0	BB5W100	[M]Oxyphilic adenocarcinoma	Feb-09
71526	15	0	0	0	PKyF.00	Alstrom syndrome	Feb-09
71584	5	2	0	0	B507.00	Malignant neoplasm of lacrimal duct	Feb-09
71619	1	0	0	0	BBgT.00	"[M]Malignant lymphoma, large cell, noncleaved, diffuse"	Feb-09
71625	1	0	0	0	B601000	Lymphosarcoma of unspecified site	Feb-09
71627	2	0	0	0	BB61200	[M]Sweat gland adenocarcinoma	Feb-09
71652	2	0	0	0	BBgP.00	"[M]Malignant lymphoma, mixed small and large cell, diffuse"	Feb-09
71672	8	0	25	0	4M23.00	Lymphoma stage IV	Feb-09
71810	11	0	0	0	B304.00	Malignant neoplasm of scapula and long bones of upper arm	Feb-09
71850	8	0	0	0	BBr6000	[M]Myeloid leukaemia NOS	Feb-09
71869	11	0	0	0	BBf2.00	[M]Alveolar soft part sarcoma	Feb-09
71895	11	3	0	0	BB56.00	[M]Superficial spreading adenocarcinoma	Feb-09
71946	3	1	0	0	B201300	Malignant neoplasm of mastoid air cells	Feb-09
72018	P118100	0	0	0	0	Cervical spina bifida without hydrocephalus - closed	Feb-09
72127	5	0	0	0	B484.00	Malignant neoplasm of epididymis	Feb-09
72139	1	0	0	0	PJ10.00	"Trisomy 13, meiotic nondisjunction"	Feb-09
72174	1	0	0	0	B4A4.00	Malignant neoplasm of paraurethral glands	Feb-09
72179	1	0	0	0	BBr0200	[M]Subacute leukaemia NOS	Feb-09
72192	14	0	0	0	BB5Xz00	[M]Clear cell adenoma or adenocarcinoma NOS	Feb-09
72196	6	1	0	0	BBgG.00	"[M]Malignant lymphoma, lymphocytic, poorly different NOS"	Feb-09
72197	2	0	0	0	B67y000	Lymphosarcoma cell leukaemia	Feb-09
72212	4	0	0	0	B308200	Malignant neoplasm of calcaneum	Feb-09
72222	1	0	0	0	BBrA100	[M]Megakaryocytic leukaemia	Feb-09

For peer review only - http://bmjopen.bmj.com/site/about/guidelines.xhtml

72224	7	1	0	0	B1z1100	Fibrosarcoma of spleen	Feb-09
72241	1	1	0	0	BBgH.00	[M]Prolymphocytic lymphosarcoma	Feb-09
72265	8	0	0	0	PJ1z.00	Patau's syndrome NOS	Feb-09
72277	4	1	0	0	BB5V700	[M]Basophil carcinoma	Feb-09
72303	1	0	0	0	K01w000	Finnish nephrosis syndrome	Feb-09
72310	1	1	0	0	BBr0400	[M]Aleukaemic leukaemia NOS	Feb-09
72433	3	0	0	0	BBh0.00	[M]Reticulosarcoma NOS	Feb-09
72443	2	0	0	0	BBZ2.00	"[M]Odontogenic tumour, malignant"	Feb-09
72445	4	0	0	0	B161000	Malignant neoplasm of cystic duct	Feb-09
72464	4	0	0	0	B305.12	Malignant neoplasm of metacarpal bones	Feb-09
72522	2	0	0	0	B313200	Malignant neoplasm of great vessels	Feb-09
72668	5	0	0	0	G210100	Malignant hypertensive heart disease with CCF	Feb-09
72707	13	0	0	0	P0...00	Anencephalus and similar anomalies	Feb-09
72723	5	0	0	0	B430000	Malignant neoplasm of cornu of corpus uteri	Feb-09
72725	8	0	0	0	B62y200	Malignant lymphoma NOS of intrathoracic lymph nodes	Feb-09
72774	2	0	0	0	B642.00	Subacute lymphoid leukaemia	Feb-09
73055	P20z.00	10	0	0	0	Encephalocele NOS	Feb-09
73065	8	0	0	0	C370y00	Cystic fibrosis with other manifestations	Feb-09
73066	1	0	0	0	BBrA.00	[M]Miscellaneous leukaemias	Feb-09
73085	2	0	0	0	P10y.00	Other specified spina bifida with hydrocephalus	Feb-09
73088	2	0	0	0	BBr9000	[M]Monocytic leukaemia NOS	Feb-09
73213	11	0	0	0	B581.00	Secondary malignant neoplasm of other urinary organs	Feb-09
73251	0	0	0	0	BBEM.00	[M]Malignant melanoma in giant pigmented naevus	Feb-09
73275	5	0	0	0	BB5N100	[M]Adenocarcinoma in adenomatous polposis coli	Feb-09
73293	7	0	0	0	G240z00	Secondary malignant hypertension NOS	Feb-09

For peer review only - http://bmjopen.bmj.com/site/about/guidelines.xhtml

73296	5	0	0	0	Byu3100	"[X]Malignant neoplasm/bones+articular cartilage/limb,unspfd"	Feb-09
73434	6	0	0	0	BB5L300	[M]Adenocarcinoma in multiple adenomatous polyps	Feb-09
73439	9	0	0	0	B064z00	Malignant neoplasm of anterior epiglottis NOS	Feb-09
73510	10	0	0	0	B550500	Malignant neoplasm of supraclavicular fossa NOS	Feb-09
73530	7	1	0	0	B305.00	Malignant neoplasm of hand bones	Feb-09
73532	2	0	0	0	B613300	"Hodgkin's, lymphocytic-histiocytic pred intra-abdominal node"	Feb-09
73536	5	0	0	0	B327000	Malignant melanoma of hip	Feb-09
73539	26	2	0	0	P51z.00	Great vessel transposition NOS	Feb-09
73556	5	0	0	0	B305z00	Malignant neoplasm of hand bones NOS	Feb-09
73583	25	0	0	0	F14y000	Ataxia-telangiectasia	Feb-09
73608	P117z00	2	0	0	0	Spina bifida without hydrocephalus - open NOS	Feb-09
73612	1	0	0	0	PE02.00	Potter's facies	Feb-09
73614	8	0	0	0	B004000	"Malignant neoplasm of lip unspecified, buccal aspect"	Feb-09
73616	15	0	0	0	B58y200	Secondary malignant neoplasm of cervix uteri	Feb-09
73662	1	0	0	0	BB5Wz00	[M]Oxyphilic adenoma or adenocarcinoma NOS	Feb-09
73712	P115z00	7	0	0	0	Myelocele NOS	Feb-09
73742	7	0	0	0	F427K00	Lipofuscinosis NOS	Feb-09
73744	5	0	0	0	B322z00	Malignant melanoma of ear and external auricular canal NOS	Feb-09
73760	12	0	0	0	B334z00	Malignant neoplasm of scalp or skin of neck NOS	Feb-09
73777	2	0	0	0	B624z00	Leukaemic reticuloendotheliosis NOS	Feb-09
73916	8	0	0	0	BBK0400	[M]Epithelioid leiomyosarcoma	Feb-09

For peer review only - http://bmjopen.bmj.com/site/about/guidelines.xhtml

73962	4	1	0	0	B000.00	"Malignant neoplasm of upper lip, vermilion border"	Feb-09
73988	6	0	0	0	B524500	Malignant neoplasm of peripheral nerve of pelvis	Feb-09
73992	5	0	0	0	B504.00	Malignant neoplasm of cornea	Feb-09
74896	30	0	0	0	B161z00	Malignant neoplasm of extrahepatic bile ducts NOS	Feb-09
85190	12	0	0	0	F562100	Malignant positional vertigo	Feb-09
85988	11	0	0	0	P210.00	Hydromicrocephaly	Feb-09
86046	17	0	0	0	B524400	Malignant neoplasm of peripheral nerve of abdomen	Feb-09
86812	12	0	0	0	B305D00	Malignant neoplasm of phalanges of hand	Feb-09
86820	6	0	0	0	BBP7.00	"[M]Mesothelioma, biphasic type, malignant"	Feb-09
86996	3	0	0	0	B501000	Malignant neoplasm of connective tissue of orbit	Feb-09
86997	8	0	0	0	Byu2400	[X]Malignant neoplasm/ill-defined sites within resp system	Feb-09
87003	1	0	0	0	BBLC100	"[M]Mesenchymoma, malignant"	Feb-09
87113	0	0	0	0	B54X.00	"Malignant neoplasm-pluriglandular involvement,unspecified"	Feb-09
87335	314	15	0	0	B624.12	Hairy cell leukaemia	Feb-09
88022	28	0	0	0	ByuC400	[X]Secondary malignant neoplasm/oth+unspcfd digestive organs	Feb-09
88144	5	0	0	0	B52y.00	Malignant neoplasm of other specified part of nervous system	Feb-09
88362	5	0	0	0	B08y.00	Malignant neoplasm of other specified hypopharyngeal site	Feb-09
89230	0	0	0	0	BBj9.00	[M]Hodgkin's granuloma	Feb-09
89258	5	0	0	0	B524200	"Malignant neoplasm of peripheral nerve of low limb, incl hip"	Feb-09
89329	7	0	0	0	ByuD800	[X]Other specified leukaemias	Feb-09

For peer review only - http://bmjopen.bmj.com/site/about/guidelines.xhtml

89593	7	0	0	0	B151200	Malignant neoplasm of intrahepatic biliary passages	Feb-09
89657	1	0	0	0	B626z00	Malignant mast cell tumour NOS	Feb-09
89762	1	0	0	0	ByuD700	[X]Other monocytic leukaemia	Feb-09
89909	9	0	0	0	B003200	"Malignant neoplasm of lower lip, mucosa"	Feb-09
89916	1	0	0	0	B553100	Malignant neoplasm of presacral region	Feb-09
90124	6	0	0	0	B067.00	Malignant neoplasm of posterior wall of oropharynx	Feb-09
90201	9	0	0	0	B62x000	T-zone lymphoma	Feb-09
90290	16	0	0	0	B18y700	Malignant neoplasm of mesentery	Feb-09
90482	2	0	0	0	P103z11	Thoracolumbar spina bifida with hydrocephalus - closed	Feb-09
90487	6	0	0	0	BBb3.11	[M]Subependymal astrocytoma NOS	Feb-09
90610	8	1	0	0	B002300	"Malignant neoplasm of upper lip, oral aspect"	Feb-09
90659	13	0	0	0	B54y.00	Malignant neoplasm of other specified endocrine gland	Feb-09
91035	2	0	0	0	B010z00	Malignant neoplasm of fixed part of tongue NOS	Feb-09
91037	6	0	0	0	B06yz00	Malignant neoplasm of other specified site of oropharynx NOS	Feb-09
91240	15	0	0	0	B517300	Malignant neoplasm of pons	Feb-09
91457	13	0	0	0	Byu5900	"[X]Malignant neoplasm/connective + soft tissue,unspecified"	Feb-09
91509	13	0	0	0	B471z00	Malignant neoplasm of descended testis NOS	Feb-09
91586	9	0	0	0	B311400	Malignant neoplasm of connective and soft tissue of finger	Feb-09
91600	P113200	8	0	0	0	Thoracic spinal meningocele	Feb-09
91842	3	0	0	0	BB69z00	[M]Sebaceous adenoma or adenocarcinoma NOS	Feb-09

For peer review only - http://bmjopen.bmj.com/site/about/guidelines.xhtml

91843	9	0	0	0	B003100	"Malignant neoplasm of lower lip, frenulum"	Feb-09
91895	4	0	0	0	B064100	Malignant neoplasm of glossoepiglottic fold	Feb-09
91900	5	0	0	0	B61z400	Hodgkin's disease NOS of lymph nodes of axilla and arm	Feb-09
92068	5	0	0	0	B620300	Nodular lymphoma of intra-abdominal lymph nodes	Feb-09
92245	1	0	0	0	B613200	"Hodgkin's, lymphocytic-histiocytic pred intrathoracic nodes"	Feb-09
92329	11	1	0	0	B48yz00	Malignant neoplasm of other male genital organ NOS	Feb-09
92371	5	0	0	0	B304300	Malignant neoplasm of radius	Feb-09
92380	1	0	0	0	B602500	Burkitt's lymphoma of lymph nodes of inguinal region and leg	Feb-09
92382	1	0	0	0	B308B00	Malignant neoplasm of fourth metatarsal bone	Feb-09
92720	3	0	0	0	B243.00	Malignant neoplasm of posterior mediastinum	Feb-09
93133	0	1	0	0	PJ22.00	"Trisomy 18, translocation"	Feb-09
93175	2	0	0	0	BBZ2.11	[M]Intraosseous carcinoma	Feb-09
93177	2	0	0	0	PG52100	Osteopetrosis - congenita type	Feb-09
93218	2	0	0	0	B03z.00	Malignant neoplasm of gum NOS	Feb-09
93342	4	0	0	0	B66z.00	Monocytic leukaemia NOS	Feb-09
93352	15569	886	0	0	B338.00	Squamous cell carcinoma of skin	Feb-09
93372	F103.00	3	0	0	0	Cerebral degeneration in diseases EC	Feb-09
93380	108	1	0	0	C10N100	Cystic fibrosis related diabetes mellitus	Feb-09
93478	4	0	0	0	B138.00	"Malignant neoplasm, overlapping lesion of colon"	Feb-09
93537	3	0	0	0	B517200	Malignant neoplasm of midbrain	Feb-09
93561	8	0	0	0	P601z00	Congenital atresia of pulmonary valve NOS	Feb-09

For peer review only - http://bmjopen.bmj.com/site/about/guidelines.xhtml

93665	6	0	0	0	Byu5300	"[X]Kaposi's sarcoma, unspecified"	Feb-09
93717	3	0	0	0	BBD7.00	"[M]Extra-adrenal paraganglioma, NOS"	Feb-09
93762	6	0	0	0	B42..00	Malignant neoplasm of placenta	Feb-09
93778	2	0	0	0	B1z1z00	Malignant neoplasm of spleen NOS	Feb-09
93842	1	0	0	0	B062300	Malignant neoplasm of palatopharyngeal arch	Feb-09
93902	3	0	0	0	P103.00	Spina bifida with hydrocephalus - closed	Feb-09
93951	3	0	0	0	B613500	"Hodgkin's, lymphocytic-histiocytic pred inguinal and leg"	Feb-09
93963	13	0	0	0	F101600	Sandhoff disease	Feb-09
94005	38	0	0	0	B615z00	"Hodgkin's disease, mixed cellularity NOS"	Feb-09
94083	1	0	0	0	BB5P.00	[M]Solid carcinoma NOS	Feb-09
94174	9	0	0	0	B67y.00	Other and unspecified leukaemia	Feb-09
94220	2	0	0	0	B540100	Malignant neoplasm of adrenal medulla	Feb-09
94239	1	0	0	0	BBp1.00	[M]Mast cell sarcoma	Feb-09
94251	2	0	0	0	B00z100	"Malignant neoplasm of lip, unspecified, lipstick area"	Feb-09
94267	19	0	0	0	BBb3.00	[M]Subependymal glioma	Feb-09
94278	25	0	0	0	B110111	Malignant neoplasm of gastro-oesophageal junction	Feb-09
94279	1	0	0	0	B61z700	Hodgkin's disease NOS of spleen	Feb-09
94286	4	0	0	0	BBG8.11	[M]Congenital fibrosarcoma	Feb-09
94355	3	0	0	0	B55y200	Malignant neoplasm of flank NOS	Feb-09
94390	3	0	0	0	B070.00	Malignant neoplasm of roof of nasopharynx	Feb-09
94407	3	0	0	0	B615100	"Hodgkin's mixed cellularity of lymph nodes head, face, neck"	Feb-09
94415	2	0	0	0	B623100	"Malignant histiocytosis of lymph nodes head, face and neck"	Feb-09
94427	1	0	0	0	B305C00	Malignant neoplasm of fifth metacarpal bone	Feb-09

For peer review only - http://bmjopen.bmj.com/site/about/guidelines.xhtml

94438	3	0	0	0	BB85z00	[M]Signet ring carcinoma NOS	Feb-09
94441	3	0	0	0	B003300	"Malignant neoplasm of lower lip, oral aspect"	Feb-09
94467	1	0	0	0	P0z..00	Anencephalus and similar anomalies NOS	Feb-09
94486	H593.00	1020	0	2	0	Chronic type 2 respiratory failure	Feb-09
94533	4	0	0	0	RyuC000	[X]Sudden infant death syndrome	Feb-09
94597	3	0	0	0	ZV10611	[V]Personal history of lymphoid leukaemia	Feb-09
94598	P115100	2	0	0	0	Cervical myelocele	Feb-09
94776	6	0	0	0	B1z2.00	"Malignant neoplasm, overlapping lesion of digestive system"	Feb-09
94793	1Z1B.00	2761	0	21	0	Chronic kidney disease stage 3 with proteinuria	Feb-09
94810	1	0	0	0	BBB4.00	[M]Adenocarcinoma with spindle cell metaplasia	Feb-09
94873	25	2	0	0	BB2A.13	[M]Squamous cell carcinoma of skin NOS	Feb-09
94925	140	2	0	0	Pyu9D00	[X]Primary ciliary dyskinesia	Feb-09
94935	2	0	14	0	4M21.00	Lymphoma stage II	Feb-09
94946	H592.00	210	0	0	0	Chronic type 1 respiratory failure	Feb-09
94965	1Z15.00	19544	0	31	1	Chronic kidney disease stage 3A	Feb-09
94975	4	0	0	0	B241300	Malignant neoplasm of pericardium	Feb-09
94995	5	0	0	0	B620500	Nodular lymphoma of lymph nodes of inguinal region and leg	Feb-09
95008	1	0	0	0	BB82112	[M]Gelatinous adenocarcinoma	Feb-09
95016	2	0	0	0	B0z1.00	Malignant neoplasm of Waldeyer's ring	Feb-09
95024	4	0	0	0	BBG8.00	[M]Infantile fibrosarcoma	Feb-09
95049	4	0	0	0	B616000	Hodgkin's lymphocytic depletion of unspecified site	Feb-09
95057	3	0	0	0	B34y000	Malignant neoplasm of ectopic site of female breast	Feb-09
95058	1	0	0	0	B600700	Reticulosarcoma of spleen	Feb-09

For peer review only - http://bmjopen.bmj.com/site/about/guidelines.xhtml

95122	1Z1H.00	1243	0	50	0	Chronic kidney disease stage 4 with proteinuria	Feb-09
95123	1Z1C.00	10576	0	6	0	Chronic kidney disease stage 3 without proteinuria	Feb-09
95145	1Z1B.11	67	0	0	0	CKD stage 3 with proteinuria	Feb-09
95150	4	0	0	0	BB81B00	[M]Serous surface papillary carcinoma	Feb-09
95175	1Z1E.00	11978	0	15	0	Chronic kidney disease stage 3A without proteinuria	Feb-09
95176	1Z1E.11	103	0	0	0	CKD stage 3A without proteinuria	Feb-09
95177	1Z1G.00	4533	0	23	0	Chronic kidney disease stage 3B without proteinuria	Feb-09
95178	1Z1F.00	1288	0	20	0	Chronic kidney disease stage 3B with proteinuria	Feb-09
95179	1Z16.00	7458	0	47	0	Chronic kidney disease stage 3B	Feb-09
95180	1Z1F.11	32	0	1	0	CKD stage 3B with proteinuria	Feb-09
95182	3	0	0	0	B308100	Malignant neoplasm of talus	Feb-09
95188	1Z1C.11	175	0	0	0	CKD stage 3 without proteinuria	Feb-09
95323	1	0	0	0	B35z000	Malignant neoplasm of ectopic site of male breast	Feb-09
95338	2	2	0	0	B613600	"Hodgkin's, lymphocytic-histiocytic pred intrapelvic nodes"	Feb-09
95373	3	0	0	0	BBCC100	"[M]Leydig cell tumour, malignant"	Feb-09
95405	1Z1L.00	142	0	1	0	Chronic kidney disease stage 5 without proteinuria	Feb-09
95406	1Z1J.00	1558	0	43	0	Chronic kidney disease stage 4 without proteinuria	Feb-09
95408	1Z1D.00	1692	0	17	0	Chronic kidney disease stage 3A with proteinuria	Feb-09
95421	13	0	0	0	B45y.00	Malignant neoplasm of other specified female genital organ	Feb-09
95429	7	0	0	0	B071.00	Malignant neoplasm of posterior wall of nasopharynx	Feb-09

For peer review only - http://bmjopen.bmj.com/site/about/guidelines.xhtml

95458	8	0	0	0	B300300	Malignant neoplasm of nasal bone	Feb-09
95478	P117200	1	0	0	0	Thoracic spina bifida without hydrocephalus - open	Feb-09
95480	9	0	0	0	B001100	"Malignant neoplasm of lower lip, lipstick area"	Feb-09
95505	6	0	0	0	B41y000	Malignant neoplasm of cervical stump	Feb-09
95508	1Z1K.00	360	0	5	0	Chronic kidney disease stage 5 with proteinuria	Feb-09
95571	1Z1D.11	25	0	0	0	CKD stage 3A with proteinuria	Feb-09
95609	6	0	0	0	BB5B300	"[M]Insulinoma, malignant"	Feb-09
95615	1	0	0	0	F150.11	Infantile spinal muscular atrophy	Feb-09
95629	6	0	0	0	B325500	Malignant melanoma of perineum	Feb-09
95630	1	0	0	0	B62x600	True histiocytic lymphoma	Feb-09
95644	4	0	0	0	B241.00	Malignant neoplasm of heart	Feb-09
95671	13	0	0	0	Byu5700	"[X]Malignant neoplasm of peritoneum, unspecified"	Feb-09
95715	31	0	0	0	B627900	Mucosa-associated lymphoma	Feb-09
95772	9	1	0	0	B051000	Malignant neoplasm of upper buccal sulcus	Feb-09
95783	11	0	0	0	B17yz00	Malignant neoplasm of specified site of pancreas NOS	Feb-09
95792	9	0	0	0	B62zz00	Lymphoid and histiocytic malignancy NOS	Feb-09
95818	4	0	0	0	BBD1.00	"[M]Paraganglioma, malignant"	Feb-09
95972	835	3	0	0	F207.00	Relapsing and remitting multiple sclerosis	Feb-09
96003	4	0	0	0	B055000	Malignant neoplasm of junction of hard and soft palate	Feb-09
96094	40	0	0	0	B119.00	Siewert type III adenocarcinoma	Feb-09
96183	1	0	0	0	BBj4.00	"[M]Hodgkin's disease,lymphocytic depletion,diffuse fibrosis"	Apr-09
96226	4	0	0	0	ByuC100	[X]Malignant neoplasm/overlap lesion/other+ill-defined sites	Apr-09
96231	8	0	0	0	BBGJ.00	"[M]Fibroxanthoma, malignant"	Apr-09

For peer review only - http://bmjopen.bmj.com/site/about/guidelines.xhtml

96246	280	2	0	0	F208.00	Secondary progressive multiple sclerosis	Apr-09
96256	671	13	0	0	F37y100	Axonal sensorimotor neuropathy	Apr-09
96291	19	0	0	0	F204.00	Benign multiple sclerosis	Apr-09
96429	3	0	0	0	B470z00	Malignant neoplasm of undescended testis NOS	Apr-09
96445	0	0	0	0	B300B00	Malignant neoplasm of turbinate	Apr-09
96494	10	0	0	0	BB5Tz00	[M]Papillary adenoma or adenocarcinoma NOS	Apr-09
96515	1	0	0	0	BBP2.00	"[M]Fibrous mesothelioma, benign"	Apr-09
96585	1	0	0	0	B32y000	Overlapping malignant melanoma of skin	Apr-09
96607	164	2	0	0	F206.00	Primary progressive multiple sclerosis	Apr-09
96635	3	0	0	0	B17y000	Malignant neoplasm of ectopic pancreatic tissue	Apr-09
96637	4	0	0	0	790M400	Biventricular repair of hypoplastic left heart syndrome	Apr-09
96709	P110200	3	0	0	0	Thoracic spina bifida without mention of hydrocephalus	Apr-09
96751	1	0	0	0	AyuCB00	"[X]HIV disease result/haematological+immunologic abnorms,NEC"	Apr-09
96782	4	0	0	0	B003z00	"Malignant neoplasm of lower lip, inner aspect NOS"	Apr-09
96783	3	0	0	0	B005.00	Malignant neoplasm of commissure of lip	Apr-09
96798	1	0	0	0	BBd2.12	[M]Meningothelial sarcoma	May-09
96802	8	0	0	0	B11y100	Malignant neoplasm of posterior wall of stomach NEC	May-09
96860	F11x900	42	0	4	0	Cerebral degeneration in Parkinson's disease	May-09
96869	4	1	0	0	B071z00	Malignant neoplasm of posterior wall of nasopharynx NOS	May-09
96893	4	0	0	0	BBrA300	[M]Myeloid sarcoma	Jun-09
97059	64	4	0	0	PKyz700	Angelman's syndrome	Jul-09

For peer review only - http://bmjopen.bmj.com/site/about/guidelines.xhtml

97091	6	0	0	0	ByuC500	[X]2ndry malignant neoplasm/bladder+oth+unsp urinary organs	Aug-09
97092	4	0	0	0	F100100	Schulz's disease	Aug-09
97332	11	0	0	0	B213z00	Malignant neoplasm of laryngeal cartilage NOS	Aug-09
97422	F11x.00	1	0	0	0	Cerebral degeneration in other disease EC	Aug-09
97454	3	1	0	0	F427K11	Lipofuscinosis NEC	Aug-09
97463	4	0	0	0	BBF4.00	[M]Giant cell sarcoma (except of bone)	Aug-09
97499	51	0	0	0	B118.00	Siewert type II adenocarcinoma	Aug-09
97530	7	0	0	0	B051100	Malignant neoplasm of lower buccal sulcus	Aug-09
97547	5	0	0	0	B551200	Malignant neoplasm of intrathoracic site NOS	Aug-09
97577	1	0	0	0	B602300	Burkitt's lymphoma of intra-abdominal lymph nodes	Sep-09
97587	1Z1J.11	47	0	2	0	CKD stage 4 without proteinuria	Sep-09
97593	6	0	0	0	BBZG.00	"[M]Ameloblastoma, malignant"	Sep-09
97683	1Z1L.11	13	0	0	0	CKD stage 5 without proteinuria	Sep-09
97746	8	0	0	0	B61z800	Hodgkin's disease NOS of lymph nodes of multiple sites	Oct-09
97751	6	2	0	0	PG51600	Osteogenesis imperfecta type IV	Oct-09
97832	5	0	0	0	B58y211	Secondary cancer of the cervix	Oct-09
97852	3	0	0	0	BBk7.00	"[M]Malignant lymphoma, centroblastic type, follicular"	Oct-09
97863	2	0	0	0	B615000	"Hodgkin's disease, mixed cellularity of unspecified site"	Oct-09
97875	3	0	0	0	B175.00	"Malignant neoplasm, overlapping lesion of pancreas"	Oct-09
97916	1	0	0	0	Q007111	Fetal alcohol syndrome	Oct-09
97996	8	0	0	0	B44y.00	Malignant neoplasm of other site of uterine adnexa	Nov-09

For peer review only - http://bmjopen.bmj.com/site/about/guidelines.xhtml

For peer review only

98009	0	0	0	0	BBrA312	[M]Granulocytic sarcoma	Nov-09
98020	G558z00	2	0	0	0	"Cardiomyopathy in diseases EC, NOS"	Nov-09
98104	4	0	0	0	B23y.00	Malignant neoplasm of other specified pleura	Dec-09
98142	29	0	0	0	B107.00	Siewert type I adenocarcinoma	Dec-09
98298	1	0	0	0	P100z00	Spina bifida with hydrocephalus NOS	Dec-09
98322	1	0	0	0	BBT7100	"[M]Haemangioendothelioma, malignant"	Jan-10
98361	3	0	0	0	Byu5B00	[X]Kaposi's sarcoma of other sites	Jan-10
98483	2	0	0	0	BBZN.11	[M]Odontogenic fibrosarcoma	Feb-10
98500	5	0	0	0	B002200	"Malignant neoplasm of upper lip, mucosa"	Feb-10
98537	0	0	0	0	B201100	Malignant neoplasm of tympanic cavity	Feb-10
98559	1	0	0	0	BBW8.00	"[M]Chondroblastoma, malignant"	Feb-10
98596	19	0	0	0	ByuD200	[X]Other types of diffuse non-Hodgkin's lymphoma	Mar-10
98617	7	0	0	0	PKyz711	Angelman syndrome	Mar-10
98662	1	0	0	0	PG51400	Osteogenesis imperfecta type II	Mar-10
98740	4	0	0	0	B000z00	"Malignant neoplasm of upper lip, vermilion border NOS"	Mar-10
98753	7	0	0	0	P228300	Aicardi syndrome	Mar-10
98781	1	0	0	0	BB5F.00	[M]Trabecular adenocarcinoma	Mar-10
98797	3	0	0	0	BBLD.00	[M]Embryonal sarcoma	Mar-10
98800	2	0	0	0	BBbG.12	[M]Piloid astrocytoma	Mar-10
98811	1	0	0	0	P102.00	Spina bifida with hydrocephalus - open	Mar-10
98825	2	0	0	0	BB5B600	[M]Mixed islet cell and exocrine adenocarcinoma	Mar-10
98840	1	0	0	0	B610300	Hodgkin's paragranuloma of intra-abdominal lymph nodes	Mar-10
98883	4	0	0	0	BB9D.00	[M]Medullary carcinoma with lymphoid stroma	Apr-10

For peer review only - http://bmjopen.bmj.com/site/about/guidelines.xhtml

98909	2	0	0	0	B611100	"Hodgkin's granuloma of lymph nodes of head, face and neck"	Apr-10
98911	2	0	0	0	B200100	Malignant neoplasm of nasal conchae	Apr-10
98961	1	0	0	0	BBk2.00	"[M]Malignant lymphoma, centroblastic-centrocytic, follicular"	Apr-10
99001	4	1	0	0	B002100	"Malignant neoplasm of upper lip, frenulum"	Apr-10
99012	1	0	0	0	B61z500	Hodgkin's disease NOS of lymph nodes inguinal region and leg	Apr-10
99015	1	0	0	0	B66y.00	Other monocytic leukaemia	Apr-10
99096	1	0	0	0	Byu2300	[X]Malignant neopl/overlapping les/resp+intrathoracic organs	Apr-10
99160	1Z1K.11	18	0	0	0	CKD stage 5 with proteinuria	Apr-10
99185	1	0	0	0	B062100	Malignant neoplasm of glossopalatine fold	Apr-10
99200	1	0	0	0	BBj7.00	"[M]Hodgkin's disease, nodular sclerosis, cellular phase"	Apr-10
99240	1	0	0	0	B600z00	Reticulosarcoma NOS	Apr-10
99257	4	0	0	0	B324z00	Malignant melanoma of scalp and neck NOS	May-10
99281	P117.00	2	0	0	0	Spina bifida without hydrocephalus - open	May-10
99299	P114200	3	0	0	0	Thoracic meningomyelocele	May-10
99312	1Z1H.11	86	0	0	0	CKD stage 4 with proteinuria	May-10
99332	P114000	1	0	0	0	Meningomyelocele of unspecified site	May-10
99386	3	0	0	0	B073200	Malignant neoplasm posterior margin nasal septum and choanae	May-10
99413	5	0	0	0	B67yz00	Other and unspecified leukaemia NOS	May-10
99493	2	0	0	0	B002.00	"Malignant neoplasm of upper lip, inner aspect"	May-10
99511	1	0	0	0	B574200	Secondary malignant neoplasm of ileum	May-10

For peer review only - http://bmjopen.bmj.com/site/about/guidelines.xhtml

99572	1	0	0	0	B312500	Malignant neoplasm of connective and soft tissue of toe	May-10
99621	3	2	0	0	B520.00	Malignant neoplasm of cranial nerves	Jun-10
99655	3	0	0	0	BBg6.00	[M]Lymphosarcoma NOS	Jun-10
99665	3	0	0	0	BBV..11	[M]Juxtacortical osteogenic sarcoma	Jun-10
99702	2	0	0	0	BBn3.00	"[M]Plasma cell tumour, malignant"	Jun-10
99711	7	0	0	0	C302511	Oculocerebrorenal syndrome	Jun-10
99797	1	0	0	0	BBX3.00	[M]Malignant giant cell tumour of soft parts	Jun-10
99887	2	0	0	0	B60y.00	Other specified reticulosarcoma or lymphosarcoma	Jun-10
99896	3	0	0	0	B12y.00	Malignant neoplasm of other specified site small intestine	Jun-10
99913	1	0	0	0	B510300	Malignant neoplasm of globus pallidus	Jun-10
99951	3	0	0	0	B60z.00	Reticulosarcoma or lymphosarcoma NOS	Jun-10
100002	7	0	0	0	B062z00	Malignant neoplasm of tonsillar fossa NOS	Jun-10
100006	3	0	0	0	B602200	Burkitt's lymphoma of intrathoracic lymph nodes	Jun-10
100035	71	6	0	0	1J0L.00	Suspected malignant mesothelioma	Jul-10
100083	123	1	0	0	B546.00	Neuroblastoma	Jul-10
100111	1	0	0	0	BB46.00	[M]Schneiderian carcinoma	Jul-10
100144	6	0	0	0	B004300	"Malignant neoplasm of lip, oral aspect"	Jul-10
100175	2	0	0	0	PKyE.00	Barber-Say syndrome	Jul-10
100267	4	0	0	0	BBZG.11	"[M]Adamantinoma, malignant"	Jul-10
100296	9	0	0	0	B582100	Secondary malignant neoplasm of skin of face	Jul-10
100352	2	0	0	0	B601500	Lymphosarcoma of lymph nodes of inguinal region and leg	Jul-10
100375	3	0	0	0	BBP4.00	"[M]Epithelioid mesothelioma, benign"	Aug-10
100423	1	0	0	0	B610100	"Hodgkin's paragranuloma of lymph nodes of head, face, neck"	Aug-10

For peer review only - http://bmjopen.bmj.com/site/about/guidelines.xhtml

100430	155	1	0	0	66k..00	Cystic fibrosis monitoring	Aug-10
100544	1	0	0	0	BBh2.00	"[M]Reticulosarcoma, nodular"	Aug-10
100582	P20z100	1	0	0	0	Encephalocele of other specified site	Aug-10
100584	3	0	0	0	B110000	Malignant neoplasm of cardiac orifice of stomach	Aug-10
100610	6	0	0	0	C370400	Arthropathy in cystic fibrosis	Sep-10
100615	1	0	0	0	B626500	Mast cell malignancy of lymph nodes inguinal region and leg	Sep-10
100622	1	0	0	0	P51z.11	Transposition of arterial trunk NEC	Sep-10
100625	1	0	0	0	BB5R500	"[M]Carcinoid tumour, nonargentaffin, malignant"	Sep-10
100633	1Z1G.11	33	0	0	0	CKD stage 3B without proteinuria	Sep-10
100721	1	0	0	0	B002z00	"Malignant neoplasm of upper lip, inner aspect NOS"	Sep-10
100733	4	0	0	0	B51yz00	Malignant neoplasm of other part of brain NOS	Sep-10
100786	5	0	0	0	B651000	Chronic eosinophilic leukaemia	Nov-10
100900	497	32	0	0	B927.12	Neurofibromatosis type 1	Nov-10
100906	1	0	0	0	B00z000	"Malignant neoplasm of lip, unspecified, external"	Nov-10
100918	1	0	0	0	B073z00	Malignant neoplasm of anterior wall of nasopharynx NOS	Nov-10
100927	1	0	0	0	BBr4z00	[M]Erythroleukaemia NOS	Nov-10
101013	P110000	1	0	0	0	"Spina bifida without hydrocephalus, site unspecified"	Dec-10
101052	57	1	0	0	B929.00	Neurofibromatosis type 2	Dec-10
101086	1	0	0	0	B520z00	Malignant neoplasm of cranial nerves NOS	Dec-10
101114	96	1	0	0	B627A00	Diffuse non-Hodgkin's large cell lymphoma	Dec-10
101222	2	0	0	0	F151111	Juvenile spinal muscular atrophy	Dec-10
101312	P20..11	2	0	0	0	Hydroencephalocele	Jan-11

For peer review only - http://bmjopen.bmj.com/site/about/guidelines.xhtml

101342	1	0	0	0	BBD3.00	[M]Parasympathetic paraganglioma	Jan-11
101429	1	0	0	0	BBj0.11	"[M]Lymphogranuloma, malignant"	Jan-11
101530	9	0	0	0	B616z00	"Hodgkin's disease, lymphocytic depletion NOS"	Jan-11
101606	1	0	0	0	B662.00	Subacute monocytic leukaemia	Feb-11
101608	7	0	0	0	B4A1100	Malignant neoplasm of ureteropelvic junction	Feb-11
101668	4	0	0	0	Byu5400	"[X]Malignant neoplasm/peripheral nerves of trunk,unspecified"	Feb-11
101707	7	0	0	0	B001z00	"Malignant neoplasm of lower lip, vermilion border NOS"	Feb-11
101715	1	0	0	0	B616700	"Hodgkin's disease, lymphocytic depletion of spleen"	Feb-11
101753	1	0	0	0	B03y.00	Malignant neoplasm of other sites of gum	Feb-11
101778	10	0	0	0	B442.00	Malignant neoplasm of broad ligament	Feb-11
101805	2	0	0	0	B507000	Malignant neoplasm of lacrimal sac	Feb-11
101885	4	0	0	0	B241400	Mesothelioma of pericardium	Mar-11
101923	1	0	0	0	BBJ1.11	[M]Fibroliposarcoma	Mar-11
101988	1	0	0	0	B060100	Malignant neoplasm of palatine tonsil	Apr-11
102102	1	0	0	0	PJ12.11	Partial trisomy 13 in Patau's syndrome	Apr-11
102117	1	0	0	0	AyuC300	[X]HIV disease resulting in multiple infections	Apr-11
102142	2	0	0	0	B013000	Malignant neoplasm of anterior 2/3 of tongue ventral surface	Apr-11
102145	3	0	0	0	B322100	Malignant melanoma of external auditory meatus	Apr-11
102151	1	0	0	0	B060200	Malignant neoplasm of overlapping lesion of tonsil	Apr-11
102171	4	0	0	0	C304400	Sulphite oxidase deficiency	Apr-11
102205	2	0	0	0	B072z00	Malignant neoplasm of lateral wall of nasopharynx NOS	May-11

For peer review only - http://bmjopen.bmj.com/site/about/guidelines.xhtml

102252	2	0	0	0	AyuCC00	[X]HIV disease resulting in other specified conditions	May-11
102338	7	0	0	0	F21y600	Vanishing white matter disease	Jun-11
102593	1	0	0	0	BB96.00	[M]Noninfiltrating intraductal papillary adenocarcinoma	Jul-11
102594	553	1	0	0	B627E00	Diffuse large B-cell lymphoma	Jul-11
102628	P104.00	2	0	0	0	Spina bifida with hydrocephalus of late onset	Jul-11
102688	3	0	0	0	ByuD400	[X]Other malignant immunoproliferative diseases	Aug-11
102783	1	0	0	0	B651200	Chronic neutrophilic leukaemia	Aug-11
102848	1	0	0	0	D200200	Constitutional aplastic anaemia with malformation	Aug-11
102921	3	0	0	0	F10y200	PEHO syndrome	Sep-11
102922	1	0	0	0	C370800	Cystic fibrosis related cirrhosis	Sep-11
102949	6	0	0	0	B312000	Malignant neoplasm of connective and soft tissue of hip	Sep-11
102980	1	0	0	0	P511200	Incomplete great vessel transposition	Sep-11
103017	1	0	0	0	PG51200	Osteogenesis imperfecta - unclassifiable	Sep-11
103034	2	0	0	0	BB5j500	"[M]Endometrioid adenofibroma, malignant"	Sep-11
103139	4	0	0	0	C375400	"Mucopolysaccharidosis, type IV"	Oct-11
103224	9	0	0	0	C370500	Cystic fibrosis with distal intestinal obstruction syndrome	Oct-11
103245	1	0	0	0	B601700	Lymphosarcoma of spleen	Oct-11
103284	1	0	0	0	P102300	Lumbar spina bifida with hydrocephalus - open	Nov-11
103354	2	0	0	0	B308z00	Malignant neoplasm of short bones of leg NOS	Dec-11
103527	P202.00	1	0	0	0	Hydromeningocele - cranial	Jan-12
103582	53	0	0	0	PJ33600	Chromosome 22q11 deletion syndrome	Jan-12
103645	1	0	0	0	B66yz00	Other monocytic leukaemia NOS	Jan-12

For peer review only - http://bmjopen.bmj.com/site/about/guidelines.xhtml

103646	1	0	0	0	PG52200	Osteopetrosis - tarda type	Jan-12
103708	1	0	0	0	BBJ6.00	[M]Round cell liposarcoma	Feb-12
103722	54	1	0	0	F390500	Congenital myopathy	Feb-12
103796	2	0	0	0	B051.00	Malignant neoplasm of vestibule of mouth	Feb-12
103873	1	0	0	0	PJ20.00	"Trisomy 18, meiotic nondisjunction"	Mar-12
103883	1	0	0	0	BBc9100	"[M]Retinoblastoma, undifferentiated type"	Mar-12
103891	1	0	0	0	C375.13	Hurler's syndrome	Mar-12
103900	3	0	0	0	B626000	Mast cell malignancy of unspecified site	Mar-12
103946	2	0	0	0	B220100	Malignant neoplasm of mucosa of trachea	Apr-12
103995	2	0	0	0	B545z00	Malignant neoplasm of aortic body or paraganglia NOS	May-12
104025	1	0	0	0	B337600	Malignant neoplasm of skin of heel	May-12
104054	4	1	0	0	PKyL.00	FG syndrome	May-12
104128	6	0	0	0	B31z000	Kaposi's sarcoma of soft tissue	May-12
104134	1	0	0	0	AyuC400	[X]HIV disease resulting/other infectious+parasitic diseases	May-12
104147	0	0	0	0	BBQ4.11	[M]Infantile embryonal carcinoma	Jun-12
104152	727	8	0	0	B628.00	Follicular lymphoma	Jun-12
104239	333	0	0	0	Q48F.00	Hypoxic ischaemic encephalopathy of newborn	Jun-12
104291	327	7	0	0	B61..11	Hodgkin lymphoma	Jun-12
104324	123	1	0	0	B595.00	Malignant tumour of unknown origin	Jun-12
104325	121	0	0	0	B640000	B-cell acute lymphoblastic leukaemia	Jun-12
104328	118	2	0	0	B641000	B-cell chronic lymphocytic leukaemia	Jun-12
104386	118	0	0	0	B62F000	Small cell B-cell lymphoma	Jun-12
104391	136	0	0	0	B627.11	Non-Hodgkin lymphoma	Jun-12
104412	8	0	0	0	B62F200	Lymphoblastic (diffuse) lymphoma	Jun-12
104475	1	0	0	0	B692.00	Subacute myelomonocytic leukaemia	Jul-12

For peer review only - http://bmjopen.bmj.com/site/about/guidelines.xhtml

104480	1	0	0	0	B58y800	Secondary malignant neoplasm of epididymis and vas deferens	Jul-12
104484	15	0	0	0	B61C.00	Other classical Hodgkin lymphoma	Jul-12
104619	K053.00	13582	0	30	0	Chronic kidney disease stage 3	Jul-12
104620	253	0	0	0	B62F100	Mantle cell lymphoma	Jul-12
104717	1	0	0	0	A789311	HIV disease resulting in Pneumocystis jirovecii pneumonia	Aug-12
104720	17	0	0	0	BBP3.11	[M]Sarcomatoid mesothelioma	Aug-12
104743	1	0	0	0	B613800	"Hodgkin's, lymphocytic-histiocytic pred of multiple sites"	Aug-12
104788	9	1	0	0	B654.00	Acute myeloblastic leukaemia	Aug-12
104790	1	0	0	0	B601800	Lymphosarcoma of lymph nodes of multiple sites	Aug-12
104862	20	0	0	0	B62E300	Cutaneous T-cell lymphoma	Aug-12
104895	16	0	0	0	B617.00	Nodular lymphocyte predominant Hodgkin lymphoma	Aug-12
104934	2	0	0	0	B62Ew00	Other mature T/NK-cell lymphoma	Sep-12
104939	10	0	0	0	B64y500	Adult T-cell lymphoma/leukaemia (HTLV-1-associated)	Sep-12
104963	K054.00	1181	0	14	0	Chronic kidney disease stage 4	Sep-12
104973	1	0	0	0	BB62z00	[M]Apocrine adenoma or adenocarcinoma NOS	Sep-12
104999	2	0	0	0	C375z00	Mucopolysaccharidosis NOS	Sep-12
105020	6	0	0	0	B628300	Follicular lymphoma grade 3a	Sep-12
105035	3	0	0	0	F211.00	Schilder's disease	Oct-12
105038	15	0	0	0	B627G00	Mediastinal (thymic) large B-cell lymphoma	Oct-12
105069	3	0	0	0	B693.00	Juvenile myelomonocytic leukaemia	Oct-12
105073	1	0	0	0	BBN2.00	"[M]Synovial sarcoma, spindle cell type"	Oct-12
105083	13	0	0	0	B62D.00	Histiocytic sarcoma	Oct-12
105085	36	0	0	0	B62E.00	T/NK-cell lymphoma	Oct-12
105095	17	0	0	0	B628100	Follicular lymphoma grade 2	Oct-12

For peer review only - http://bmjopen.bmj.com/site/about/guidelines.xhtml

105151	K055.00	326	0	0	0	Chronic kidney disease stage 5	Nov-12
105166	1	0	0	0	BBDB.11	[M]Glomoid sarcoma	Nov-12
105186	10	0	0	0	P26..00	Disorder of neuronal migration and differentiation	Nov-12
105203	1	0	0	0	B620200	Nodular lymphoma of intrathoracic lymph nodes	Nov-12
105275	1	0	0	0	BBV..13	[M]Periosteal osteogenic sarcoma	Dec-12
105296	7	0	0	0	BBTD200	"[M]Haemangiopericytoma, malignant"	Dec-12
105324	1	0	0	0	A789800	HIV disease resulting in multiple malignant neoplasms	Dec-12
105335	5	0	0	0	B62A.00	Sarcoma of dendritic cells	Dec-12
105375	3	0	0	0	B62E800	Blastic NK-cell lymphoma	Dec-12
105388	97	0	0	0	B498.00	Local recurrence of malignant tumour of urinary bladder	Dec-12
105472	1	0	0	0	B614700	"Hodgkin's disease, nodular sclerosis of spleen"	Dec-12
105475	1	0	0	0	B308.00	Malignant neoplasm of short bones of leg	Dec-12
105488	133	1	0	0	B36..00	Local recurrence of malignant tumour of breast	Dec-12
105559	17	0	0	0	B62E100	"Anaplastic large cell lymphoma, ALK-positive"	Jan-13
105613	3	0	0	0	B161300	Malignant neoplasm of sphincter of Oddi	Jan-13
105636	20	0	0	0	B62E900	Angioimmunoblastic T-cell lymphoma	Jan-13
105679	24	1	0	0	F25G.11	Dravet syndrome	Jan-13
105709	3	0	0	0	B62E600	Enteropathy-associated T-cell lymphoma	Jan-13
105797	1	0	0	0	B304100	Malignant neoplasm of acromion	Feb-13
105841	16	0	0	0	B618.00	Nodular sclerosis classical Hodgkin lymphoma	Mar-13
105889	15	0	0	0	B628000	Follicular lymphoma grade 1	Mar-13
105925	1	0	0	0	B62E700	Subcutaneous panniculitic T-cell lymphoma	Apr-13
105944	1	0	0	0	BBK3300	[M]Mixed cell rhabdomyosarcoma	Apr-13

For peer review only - http://bmjopen.bmj.com/site/about/guidelines.xhtml

105955	12	0	0	0	B62E200	"Anaplastic large cell lymphoma, ALK-negative"	Apr-13
105956	9	0	0	0	C315200	Kearns-Sayre syndrome	Apr-13
105957	6	0	0	0	B651100	"Chronic myeloid leukaemia, BCR/ABL positive"	Apr-13
106012	8	0	0	0	Q48F.11	Perinatal hypoxic - ischaemic encephalopathy	Apr-13
106063	2	0	0	0	B628700	Other types of follicular lymphoma	May-13
106069	1	0	0	0	B305.11	Malignant neoplasm of carpal bones	May-13
106114	3	0	0	0	PJ51400	Trisomy 9p syndrome	May-13
106134	1	0	0	0	BBdB.00	[M]Meningeal sarcomatosis	May-13
106137	1	0	0	0	BBh..00	[M]Reticulosarcomas	May-13
106194	1	0	0	0	B231.00	Malignant neoplasm of visceral pleura	May-13
106197	1	0	0	0	BBr7000	[M]Basophilic leukaemia	May-13
106349	3	0	0	0	B61z.11	Hodgkin lymphoma NOS	Jun-13
106432	200	0	0	0	C370900	Exacerbation of cystic fibrosis	Jun-13
106483	1	0	0	0	BBr6200	[M]Subacute myeloid leukaemia	Aug-13
106519	79	0	0	0	BB1P.00	[M]Non-small cell carcinoma	Aug-13
106569	2	0	0	0	B500200	Malignant neoplasm of crystalline lens	Aug-13
106579	1	0	0	0	Pyu0400	[X]Unspecified spina bifida with hydrocephalus	Aug-13
106597	3	0	0	0	B61B.00	Lymphocyte-rich classical Hodgkin lymphoma	Aug-13
106858	6	0	0	0	1JW..00	Suspected cystic fibrosis	Aug-13
106867	4	0	0	0	B62F.11	Non-follicular lymphoma	Aug-13
106884	6	0	0	0	B62F.00	Nonfollicular lymphoma	Aug-13
106911	1	0	0	0	B613700	"Hodgkin's, lymphocytic-histiocytic predominance of spleen"	Aug-13
106915	1	0	0	0	B073100	Malignant neoplasm of nasopharyngeal soft palate surface	Aug-13
106924	8	0	0	0	B641200	Clinical stage B chronic lymphocytic leukaemia	Aug-13

For peer review only - http://bmjopen.bmj.com/site/about/guidelines.xhtml

106926	6	0	0	0	PKyP.11	Wolfram syndrome	Aug-13
106969	2	0	0	0	B628500	Diffuse follicle centre lymphoma	Sep-13
106970	2	0	0	0	BBk3.00	"[M]Malig lymphoma, lymphocytic, well differentiated,nodular"	Sep-13
107017	9	0	0	0	B641011	Chronic lymphocytic leukaemia of B-cell type	Sep-13
107032	1	0	0	0	B616800	Hodgkin's lymphocytic depletion lymph nodes multiple sites	Sep-13
107052	44	0	0	0	B641100	Clinical stage A chronic lymphocytic leukaemia	Oct-13
107126	3	0	0	0	B553200	Malignant neoplasm of sacrococcygeal region	Oct-13
107144	P114100	3	0	0	0	Cervical meningomyelocele	Oct-13
107162	1	0	0	0	PJ22.11	Partial trisomy 18 in Edward's syndrome	Oct-13
107163	4	0	0	0	B641300	Clinical stage C chronic lymphocytic leukaemia	Oct-13
107166	5	0	0	0	B628200	Follicular lymphoma grade 3	Oct-13
107236	9	0	0	0	B651300	"Atypical chronic myeloid leukaemia, BCR/ABL negative"	Nov-13
107258	1	0	0	0	B011100	Malignant neoplasm of midline of tongue	Nov-13
107299	1	0	0	0	BB5D700	[M]Combined hepatocellular carcinoma and cholangiocarcinoma	Nov-13
107377	P116100	1	0	0	0	Cervical myelocystocele	Dec-13
107643	5	0	0	0	B64y400	T-cell prolymphocytic leukaemia	Jan-14
107773	1	0	0	0	BBr8z00	[M]Eosinophilic leukaemia NOS	Mar-14
107804	1	0	0	0	B61z300	Hodgkin's disease NOS of intra-abdominal lymph nodes	Mar-14
107807	1	0	0	0	AyuC100	[X]HIV disease resulting in other viral infections	Mar-14
107840	1	0	0	0	C308100	Multiple acyl-CoA dehydrogenase deficiencies	Mar-14

For peer review only - http://bmjopen.bmj.com/site/about/guidelines.xhtml

107878	1	0	0	0	B213200	Malignant neoplasm of cuneiform cartilage	Apr-14
107884	1	0	0	0	BBba000	[M]Peripheral neuroectodermal tumour	Apr-14
107916	1	0	0	0	B201000	Malignant neoplasm of auditory (Eustachian) tube	Apr-14
107949	2	0	0	0	B62E500	Hepatosplenic T-cell lymphoma	Apr-14
107973	7	0	0	0	B628400	Follicular lymphoma grade 3b	May-14
108021	1	0	0	0	790Jz00	Other repair of transposition of great arteries NOS	May-14
108041	17	1	0	0	C308200	X-linked adrenoleucodystrophy	May-14
108054	4	1	0	0	A789511	HIV disease resulting in Kaposi sarcoma	May-14
108131	2	0	0	0	F146.00	Early onset cerebellar ataxia with hypogonadism	Jun-14
108182	1	0	0	0	B627400	Diffuse non-Hodgkin's small cleaved cell (diffuse) lymphoma	Jun-14
108316	2	0	0	0	BBrAz00	[M]Miscellaneous leukaemia NOS	Jul-14
108363	71	1	0	0	B509.00	Malignant melanoma of eye	Jul-14
108389	1	0	0	0	B310500	Malignant neoplasm soft tissues of cervical spine	Jul-14
108424	5	0	0	0	B663.00	Acute monoblastic leukaemia	Jul-14
108591	4	0	0	0	K01w100	Drash syndrome	Sep-14
108623	2	0	0	0	C372.11	Lesch - Nyhan syndrome	Sep-14
108638	1	0	0	0	B305A00	Malignant neoplasm of third metacarpal bone	Sep-14
108652	1	0	0	0	PyuAD00	[X]Li-Fraumeni syndrome	Sep-14
108656	3	1	0	0	B64y300	B-cell prolymphocytic leukaemia	Sep-14
108667	2	0	0	0	B1z1000	Angiosarcoma of spleen	Sep-14
108682	1	0	0	0	BBgJ.11	[M]Germinoblastic sarcoma NOS	Oct-14
108715	1	0	0	0	B66..11	Histiocytic leukaemia	Oct-14
108719	6	0	0	0	B628600	Cutaneous follicle centre lymphoma	Oct-14
108773	F103z00	1	0	0	0	Cerebral degeneration in disease NOS	Oct-14

For peer review only - http://bmjopen.bmj.com/site/about/guidelines.xhtml

108775	1	0	0	0	B619.00	Mixed cellularity classical Hodgkin lymphoma	Oct-14
108886	1	0	0	0	B615500	Hodgkin's mixed cellularity of lymph nodes inguinal and leg	Nov-14
108922	1	0	0	0	K01w112	Wilms' tumour + nephrotic syndrome + pseudohermaphroditism	Dec-14
108947	1	0	0	0	PKyz.11	Cockayne's syndrome	Dec-14
108964	2	0	0	0	BBr6900	[M]Juvenile myelomonocytic leukaemia	Dec-14
109002	2	0	0	0	B325400	Malignant melanoma of perianal skin	Dec-14
109110	5	0	0	0	BBD9.11	[M]Chromaffin paraganglioma	Jan-15
109223	1	0	0	0	PJ50311	Trisomy 9 Mosaic Syndrome	Jan-15
109243	1	0	0	0	P100100	Cervical spina bifida with hydrocephalus	Feb-15
109243	P100100	1	0	0	0	Cervical spina bifida with hydrocephalus	Feb-15
109288	11	1	0	0	A411000	Sporadic Creutzfeldt-Jakob disease	Feb-15
109394	1	0	0	0	P00y.00	Other specified anencephalus	Mar-15
109473	0	0	0	0	B521200	Malignant neoplasm of cerebral pia mater	Apr-15
109608	2	0	0	0	C300A00	Congenital Fanconi syndrome	May-15
109657	1Z1Y.00	92	0	0	0	CKD with GFR category G3b & albuminuria category A2	May-15
109714	1	0	0	0	ByuDD00	[X]Oth and unspecif peripheral & cutaneous T-cell lymphomas	Jun-15
109745	2	0	0	0	B32G.00	Malignant melanoma stage IV M1a	Jun-15
109780	1	0	0	0	B62E400	"Extranodal NK/T-cell lymphoma, nasal type"	Jun-15
109804	1Z1T.00	390	0	0	0	CKD with GFR category G3a & albuminuria category A1	Jun-15
109805	1Z1V.00	115	0	1	0	CKD with GFR category G3a & albuminuria category A2	Jun-15
109827	4	0	0	0	B329.00	Malignant melanoma stage IB	Jul-15
109868	2	0	0	0	C327300	Wolman disease	Jul-15
109904	1Z1b.00	26	0	0	0	CKD with GFR category G4 & albuminuria category A2	Jul-15

For peer review only - http://bmjopen.bmj.com/site/about/guidelines.xhtml

109905	1Z1W.00	25	0	0	0	CKD with GFR category G3a & albuminuria category A3	Jul-15
109963	1Z1X.00	160	0	0	0	CKD with GFR category G3b & albuminuria category A1	Jul-15
109980	1Z1a.00	36	0	0	0	CKD with GFR category G4 & albuminuria category A1	Aug-15
109981	1Z1e.00	1	0	0	0	CKD with GFR category G5 & albuminuria category A2	Aug-15
109990	1Z1Z.00	28	0	0	0	CKD with GFR category G3b & albuminuria category A3	Aug-15
110058	2	0	0	0	ZV10613	[V]Personal history of myeloid leukaemia	Sep-15
110133	1Z1d.00	2	0	0	0	CKD with GFR category G5 & albuminuria category A1	Sep-15
110139	4	0	0	0	B32A.00	Malignant melanoma stage IIA	Sep-15
110147	1	0	0	0	BB5D711	[M]Hepatocholangiocarcinoma	Sep-15
110169	1	0	0	0	PKyD.00	Nicolaides-Baraitser syndrome	Oct-15
110180	1	0	0	0	B32B.00	Malignant melanoma stage IIB	Oct-15
110349	1	0	0	0	BBr3z00	[M]Plasma cell leukaemia NOS	Dec-15
110433	1	0	0	0	B574100	Secondary malignant neoplasm of jejunum	Jan-16
110454	1	0	0	0	C370700	Liver disease due to cystic fibrosis	Jan-16
110467	1Z1f.00	9	0	0	0	CKD with GFR category G5 & albuminuria category A3	Jan-16
110483	2	0	0	0	B32H.00	Malignant melanoma stage IV M1b	Jan-16
110563	1	0	0	0	B616500	Hodgkin's lymphocytic depletion lymph nodes inguinal and leg	Feb-16
110626	1Z1c.00	13	0	0	0	CKD with GFR category G4 & albuminuria category A3	Mar-16
110749	2	0	0	0	K01w200	Congenital nephrotic syndrome with focal glomerulosclerosis	Apr-16
110766	2	0	0	0	B521000	Malignant neoplasm of cerebral dura mater	May-16

For peer review only - http://bmjopen.bmj.com/site/about/guidelines.xhtml

110775	1	0	0	0	B151100	Malignant neoplasm of interlobular biliary canals	May-16
110833	2	0	0	0	PJ50400	Trisomy 10	May-16
110838	1	0	0	0	B676.00	Acute erythroid leukaemia	May-16
110903	1	0	0	0	B623800	Malignant histiocytosis of lymph nodes of multiple sites	Jun-16
110961	1	0	0	0	B32F.00	Malignant melanoma stage IIIC	Jun-16
110993	1	0	0	0	B305800	Malignant neoplasm of first metacarpal bone	Jul-16
111036	1	0	0	0	C375.18	Sanfilippo's syndrome	Jul-16
111077	2	0	0	0	C300800	Juvenile nephropathic cystinosis	Jul-16
111079	4	0	0	0	B328.00	Malignant melanoma stage IA	Jul-16
111113	1	0	0	0	BBj3.00	"[M]Hodgkin's disease, lymphocytic depletion NOS"	Jul-16
111120	3	0	0	0	PJ33113	18q deletion syndrome	Jul-16
111162	2	0	0	0	B32J.00	Malignant melanoma stage IV M1c	Jul-16
111172	1	0	0	0	BB5R300	"[M]Carcinoid tumour, argentaffin, malignant"	Aug-16
111238	1	0	0	0	BB5dz00	[M]Mixed cell adenoma or adenocarcinoma NOS	Aug-16
111289	1	0	0	0	B002000	"Malignant neoplasm of upper lip, buccal aspect"	Sep-16
111311	1	0	0	0	B317.00	"Malignant neoplasm, overlap lesion connective & soft tissue"	Sep-16
111370	1	0	0	0	K01wz00	Congenital nephrotic syndrome NOS	Oct-16
111413	2	0	0	0	B32C.00	Malignant melanoma stage IIC	Oct-16
111426	1	0	0	0	B308900	Malignant neoplasm of second metatarsal bone	Oct-16
46733	D104700	148	0	0	0	Beta major thalassaemia	Feb-09
21643	D104811	655	0	8	2	Beta thalassaemia	Feb-09
45151	D104011	11	0	0	0	Thalassaemia major - Cooley's anaemia	Feb-09
31405	D104000	103	0	5	0	Thalassaemia major NEC	Feb-09

For peer review only - http://bmjopen.bmj.com/site/about/guidelines.xhtml

Appendix 2 ICD 10 Codes to identify a child with a Life-limiting condition

A17

A81.0

A81.1

B20

B21

B22

B23

B24

C00

C01

C02

C03

C04

C05

C06

C07

C08

C09

C10

For peer review only
For peer review only - http://bmjopen.bmj.com/site/about/guidelines.xhtml

C11

C12

C13

C14

C15

C16

C17

C18

C19

C20

C21

C22

C23

C24

C25

C26

C30

C31

C32

C33

For peer review only

For peer review only - http://bmjopen.bmj.com/site/about/guidelines.xhtml

C34

C35

C36

C37

C38

C39

C40

C41

C43

C44

C45

C46

C47

C48

C49

C50

C51

C52

C53

C54

For peer review only - http://bmjopen.bmj.com/site/about/guidelines.xhtml

C55

C56

C57

C58

C59

C60

C61

C62

C63

C64

C65

C66

C67

C68

C69

C70

C71

C72

C73

C74

For peer review only - http://bmjopen.bmj.com/site/about/guidelines.xhtml

C75

C76

C77

C78

C79

C80

C81

C82

C83

C84

C85

C86

C87

C88

C89

C90

C91

C92

C93

C94

For peer review only

C95

C96

C97

D61.9

D70

D76.1

D81

D82.1

D83

D89.1

E31.0

E34.8

E70.2

E71

E72

E74

E75

E76

E77

E79.1

For peer review only

For peer review only - http://bmjopen.bmj.com/site/about/guidelines.xhtml

E83.0

E84

E88.0

E88.1

"F80.3

"

F84.2

G10

G11.1

G11.3

G12

G20

G23.0

G23.8

G31.8

G31.9

G35

G40.4

G40.5

G60.0

For peer review only

For peer review only - http://bmjopen.bmj.com/site/about/guidelines.xhtml

G60.1

G70.2

G70.9

G71.0

G71.1

G71.2

G71.3

G80.0

G80.8

G82.3

G82.4

G82.5

G93.4

G93.6

G93.7

H11.1

H49.8

H35.5

I21

I27.0

For peer review only
For peer review only - http://bmjopen.bmj.com/site/about/guidelines.xhtml

I42

I61.3

I81

J84.1

J96

J98.4

K55.0

K55.9

K72

K74

K76.5

K86.8

M31.3

M32.1

M89.5

N17

N18

N19

N25.8

P10.1

For peer review only - http://bmjopen.bmj.com/site/about/guidelines.xhtml

For peer review only

P11.2

P21.0

P28.5

P29.0

P29.3

P35.0

P35.1

P35.8

P37.1

Q00.0

Q01

Q03.1

Q03.9

Q04.0

Q04.2

Q04.3

Q04.4

Q04.6

Q04.9

Q21.8

For peer review only
For peer review only - http://bmjopen.bmj.com/site/about/guidelines.xhtml

Q22.0

Q22.1

Q22.4

Q22.5

Q22.6

Q23.0

Q23.4

Q23.9

Q25.4

Q39.6

Q41.0

Q41.9

Q43.7

Q44.2

Q74.8

Q44.5

Q44.7

Q60.1

Q60.6

Q78.0

For peer review only - http://bmjopen.bmj.com/site/about/guidelines.xhtml

Q78.5

Q79.2

Q79.3

Q80.4

Q81

Q82.1

Q82.4

Q85.8

Q86.0

Q93.2

Q93.3

Q93.4

Q93.5

Q93.8

Q95.2

T86.0

T86.2

Appendix 3 Read codes to identify a child with a chronic, not life-limiting condition

5600	F172.00	2402	0	130	0	[X] Horners syndrome	Feb-09

For peer review only

24753	ZS91.12	143	0	5	0	[X]Attention deficit disorder	Feb-09
26285	Eu9y700	2865	0	197	0	[X]Attention deficit disorder	Feb-09
34929	Eu50100	402	0	11	0	[X]Atypical anorexia nervosa	Feb-09
24044	Eu84100	418	0	5	0	[X]Atypical autism	Feb-09
33863	Eu50300	258	0	6	0	[X]Atypical bulimia nervosa	Feb-09
24062	Eu84111	16	0	0	0	[X]Atypical childhood psychosis	Feb-09
91547	Eu20311	4	0	0	0	[X]Atypical schizophrenia	Feb-09
9982	Eu84011	13172	0	379	0	[X]Autistic disorder	Feb-09
51375	Eu84511	9	0	0	0	[X]Autistic psychopathy	Feb-09
42941	Eu84z11	2488	0	11	0	[X]Autistic spectrum disorder	Feb-09
23713	Eu31400	117	0	7	0	"[X]Bipol aff disord, curr epis sev depress, no psychot symp"	Feb-09
28277	Eu31200	153	0	1	0	[X]Bipolar affect disorder cur epi manic with psychotic symp	Feb-09
26299	Eu31100	65	0	0	0	[X]Bipolar affect disorder cur epi manic wout psychotic symp	Feb-09
104065	Eu31800	15	0	0	0	[X]Bipolar affective disorder type I	May-12
103915	Eu31900	262	0	1	0	[X]Bipolar affective disorder type II	Mar-12
44693	Eu31600	124	0	4	0	"[X]Bipolar affective disorder, current episode mixed"	Feb-09
27584	Eu31700	1499	0	1	0	"[X]Bipolar affective disorder, currently in remission"	Feb-09
33751	Eu31z00	624	0	4	0	"[X]Bipolar affective disorder, unspecified"	Feb-09
9581	Eu50200	10720	0	551	4	[X]Bulimia nervosa	Feb-09
72688	Myu1.00	37	0	0	0	[X]Bullous disorders	Feb-09
3637	Eu84000	978	0	21	0	[X]Childhood autism	Feb-09
53820	PyuA.00	12	0	0	0	"[X]Chromosomal abnormalities, not elswhere classified"	Feb-09
543	Eu32z11	738902	0	19968	0	[X]Depression NOS	Feb-09
3291	Eu32z12	16570	0	445	0	[X]Depressive disorder NOS	Feb-09

For peer review only - http://bmjopen.bmj.com/site/about/guidelines.xhtml

6514	Eu80z00	399	0	3	0	[X]Developmental disorder of speech and language unspecified	Feb-09
52212	Cyu2.00	193	0	1	0	[X]Diabetes mellitus	Feb-09
36946	Eu50z00	2380	0	108	0	"[X]Eating disorder, unspecified"	Feb-09
100899	Eu85.00	2066	0	20	0	[X]Global developmental delay	Nov-10
33505	Eu90100	91	0	1	0	[X]Hyperkinetic conduct disorder	Feb-09
45799	Eu90111	8	0	2	0	[X]Hyperkinetic disorder associated with conduct disorder	Feb-09
50015	Eu90z00	79	0	3	0	"[X]Hyperkinetic disorder, unspecified"	Feb-09
97421	Eu90z11	0	0	0	0	[X]Hyperkinetic reaction of childhood or adolescence NOS	Aug-09
96770	Eu90z12	4	0	0	0	[X]Hyperkinetic syndrome NOS	Apr-09
50337	Eu84012	30	0	0	0	[X]Infantile autism	Feb-09
4477	Eu81z11	15741	0	569	0	[X]Learning disability NOS	Feb-09
98346	Eu32500	512	0	2	0	"[X]Major depression, mild"	Jan-10
98252	Eu32600	1500	0	11	0	"[X]Major depression, moderately severe"	Dec-09
98417	Eu32800	323	0	3	0	"[X]Major depression, severe with psychotic symptoms"	Jan-10
98414	Eu32700	1948	0	17	0	"[X]Major depression, severe without psychotic symptoms"	Jan-10
28962	Eu7..00	394	0	1	0	[X]Mental retardation	Feb-09
34174	Eu84112	102	0	2	0	[X]Mental retardation with autistic features	Feb-09
98342	Eu81400	5806	0	21	0	[X]Moderate learning disability	Jan-10
98293	Eu81500	3549	0	24	0	[X]Severe learning disability	Dec-09
36143	Eu72.00	2449	0	1	0	[X]Severe mental retardation	Feb-09
22048	PKy6500	68	0	2	0	Aarskog syndrome	Feb-09
102328	PJyy.11	1	0	0	0	Absence of sex chromosome	Jun-11
72655	P74z100	3	0	0	0	Absence of superior vena cava	Feb-09
8097	2828	7568	0	1399	0	Absence seizure	Feb-09

For peer review only - http://bmjopen.bmj.com/site/about/guidelines.xhtml

For peer review only

71109	PK11.00	3	0	0	0	Absent adrenal gland	Feb-09
25089	PKyz100	4	0	0	0	Acardia	Feb-09
57804	PG41.13	15	0	0	0	Achondroplastic dwarf	Feb-09
69916	C152100	3	0	0	0	Acquired adrenogenital syndrome	Feb-09
91774	G341300	19	0	0	0	Acquired atrioventricular fistula of heart	Feb-09
101375	SN51000	47	0	1	0	Acquired C1 esterase inhibitor deficiency	Jan-11
46025	D307.00	37	0	2	0	Acquired coagulation factor deficiency	Feb-09
37587	D307z00	25	0	0	0	Acquired coagulation factor deficiency NOS	Feb-09
63195	J690100	67	0	1	0	Acquired coeliac disease	Feb-09
4675	F113.00	811	0	60	0	Acquired communicating hydrocephalus	Feb-09
31667	M145300	230	0	16	0	Acquired epidermolysis bullosa	Feb-09
49889	ZS82.00	2	0	0	0	Acquired epileptic aphasia	Feb-09
46546	D307200	15	0	0	0	Acquired factor II deficiency	Feb-09
107206	C300600	5	0	1	0	Acquired Fanconi syndrome	Nov-13
50495	D112z12	8	0	0	0	Acquired haemolytic anaemia with haemoglobinuria NEC	Feb-09
27771	D11..00	152	0	9	1	Acquired haemolytic anaemias	Feb-09
105061	C353500	4	0	0	0	Acquired hypophosphataemia	Oct-12
103789	D307212	0	0	0	0	Acquired hypoprothrombinaemia	Feb-12
3290	C04..00	160727	0	878	7	Acquired hypothyroidism	Feb-09
23639	M211600	136	0	4	0	Acquired ichthyosis	Feb-09
23770	A788.00	1182	0	53	8	Acquired immune deficiency syndrome	Feb-09
110481	K081000	1	0	0	0	Acquired nephrogenic diabetes insipidus	Jan-16
63815	J106500	9	0	0	0	Acquired oesophagocoele	Feb-09
43824	D307211	44	0	2	0	Acquired prothrombin deficiency	Feb-09

For peer review only - http://bmjopen.bmj.com/site/about/guidelines.xhtml

69269	D20X.00	7	0	0	0	"Acquired pure red cell aplasia, unspecified"	Feb-09
104079	K132.11	476	0	127	0	Acquired renal cystic disease	May-12
41699	D210100	8	0	0	0	Acquired sideroblastic anaemia	Feb-09
29933	D11z000	116	0	2	0	Acquired spherocytosis	Feb-09
41840	PF55000	53	0	3	0	Acrocephalosyndactyly (Apert)	Feb-09
48272	PG01.00	8	0	0	0	Acrocephaly	Feb-09
5316	C130200	2971	0	245	1	Acromegaly	Feb-09
62353	PF5E200	32	0	0	0	Acrosyndactyly	Feb-09
11147	C134411	141	0	5	0	ACTH deficiency	Feb-09
43641	C153.11	4	0	0	0	ACTH overproduction	Feb-09
38968	P6z2.00	48	0	0	0	Acyanotic congenital heart disease NOS	Feb-09
59166	PF47.11	8	0	1	0	Adactyly	Feb-09
5524	PG51.13	7	0	0	0	Adair-Dighton syndrome	Feb-09
4042	C154011	239	0	38	0	Addisonian crisis	Feb-09
12227	C154600	668	0	18	0	Addisonian crisis	Feb-09
2813	D010.11	53	0	4	0	Addison's anaemia	Feb-09
4481	C154100	5819	0	428	2	Addison's disease	Feb-09
110474	44lz.00	0	0	0	1	Additional biochemical ratios	Jan-16
9665	PJz1.00	9	0	0	0	Additional chromosome NOS	Feb-09
72804	C392600	3	0	0	0	Adenosine deaminase deficiency	Feb-09
57552	C30yy11	10	0	0	0	Adenosine-deaminase deficiency	Feb-09
1523	F4K4600	2858	0	130	0	Adie's pupil syndrome	Feb-09
42873	C154012	66	0	3	0	Adrenal crisis	Feb-09
41542	C15z.00	215	0	7	0	Adrenal gland disorder NOS	Feb-09
28896	C154z11	74	0	2	0	Adrenal hypofunction	Feb-09
20786	C154z12	444	0	14	0	Adrenal insufficiency NEC	Feb-09
73529	C155000	3	0	0	0	Adrenal medullary insufficiency	Feb-09
32642	C153.12	39	0	0	0	Adrenocortical hyperfunction	Feb-09
57321	C152z00	7	0	0	0	Adrenogenital disorder NOS	Feb-09
31446	C152811	123	0	4	0	Adrenogenital syndrome NOS	Feb-09

For peer review only - http://bmjopen.bmj.com/site/about/guidelines.xhtml

35682	C390.11	148	0	8	0	Agammaglobulinaemia	Feb-09
62598	C390900	2	0	0	0	Agammaglobulinaemia NEC	Feb-09
65511	PB33.11	1	0	0	0	Aganglionic macrocolon	Feb-09
94813	PB3..11	1	0	0	0	Aganglionosis	Feb-09
23457	P220.00	121	0	11	0	"Agenesis of brain, part unspecified"	Feb-09
54877	P22A011	4	0	0	0	Agenesis of cerebellum	Feb-09
40777	P227000	19	0	0	0	Agenesis of cerebrum	Feb-09
67919	P362000	10	0	2	0	Agenesis of cilia	Feb-09
39486	P228011	211	0	2	0	Agenesis of corpus callosum	Feb-09
60930	PF37.00	10	0	0	0	Agenesis of fibula	Feb-09
47576	PB63011	9	0	0	0	Agenesis of gallbladder	Feb-09
56350	PF21z11	14	0	0	0	Agenesis of hand	Feb-09
59444	P853.00	17	0	0	0	Agenesis of lung	Feb-09
93911	P2xz000	3	0	0	0	Agenesis of nerve NEC	Feb-09
67476	P2x0.00	6	0	0	0	"Agenesis of nerve, unspecified"	Feb-09
58710	P22z.12	6	0	0	0	Agenesis of part of brain NEC	Feb-09
53723	P223.00	4	0	0	0	Agyria	Feb-09
40029	PB63500	118	0	2	0	Alagille syndrome	Feb-09
66720	C30y000	24	0	0	2	Alaninaemia	Feb-09
50325	PG52.11	8	0	0	0	Albers - Schonberg syndrome	Feb-09
15624	C302700	956	0	41	0	Albinism	Feb-09
53954	C302.11	25	0	0	0	Albinism	Feb-09
31388	C302712	57	0	4	0	Albino	Feb-09
30601	PG5y011	42	0	3	0	Albright-McCune-Sternberg syndrome	Feb-09
57407	PG5y012	15	0	4	0	Albright's polyostotic dysplasia	Feb-09
15046	PG5y000	35	0	0	0	Albright-Sternberg syndrome	Feb-09
52688	C151.11	70	0	4	0	Aldosteronism	Feb-09
2290	H330.11	13983	0	430	3	Allergic asthma	Feb-09
21232	H33zz12	182	0	0	0	Allergic asthma NEC	Feb-09
1037	M240.00	107630	0	9254	24	Alopecia	Feb-09

For peer review only - http://bmjopen.bmj.com/site/about/guidelines.xhtml

685	M240100	44959	0	2328	2	Alopecia areata	Feb-09
43109	M240900	25	0	1	0	Alopecia disseminata	Feb-09
50780	M240700	3	0	0	0	Alopecia febrilis	Feb-09
48735	M240C00	50	0	3	0	Alopecia follicularis	Feb-09
40947	M240J00	8	0	0	0	Alopecia hereditaria	Feb-09
48719	M240H00	35	0	0	0	Alopecia seborrhoeica	Feb-09
52294	M240600	24	0	0	0	Alopecia senilis	Feb-09
976	M240000	5590	0	250	1	Alopecia unspecified	Feb-09
8866	D104300	848	0	19	0	Alpha thalassaemia	Feb-09
12235	D104400	3655	0	30	114	Alpha trait thalassaemia	Feb-09
3019	C376200	2675	0	117	64	Alpha-1-antitrypsin deficiency	Feb-09
25589	C376100	167	0	8	9	Alpha-1-antitrypsin hepatitis	Feb-09
71522	C327400	14	0	2	0	Alpha-galactosidase A deficiency	Feb-09
24395	PKy9000	592	0	32	0	Alport's syndrome	Feb-09
64601	D014000	3	0	0	0	Amino-acid deficiency anaemia	Feb-09
46357	C300z00	3	0	4	4	Amino-acid transport disorder NOS	Feb-09
32466	46T6.11	18	0	0	11	Aminoaciduria	Feb-09
107595	C373H00	13	0	0	0	Amyloid A amyloidosis	Jan-14
105651	G558400	14	0	0	0	Amyloid cardiomyopathy	Jan-13
30667	G557000	139	0	4	0	Amyloid heart disease	Feb-09
36697	F38y.11	20	0	0	0	Amyotonia congenita	Feb-09
31439	N232000	170	0	5	0	Amyotrophia NOS	Feb-09
57769	PGyy000	5	0	2	2	Amyotrophica congenita	Feb-09
31944	C310412	108	0	2	0	Andersen's disease	Feb-09
24660	C327413	52	0	1	0	Anderson-Fabry disease	Feb-09
93886	C327412	2	0	0	0	Anderson's disease	Feb-09
49161	C1z5.11	145	0	6	0	Androgen insensitivity syndrome	Feb-09
61407	PJ5z.11	8	0	0	0	Aneuploidy NEC	Feb-09
2184	N100.00	35726	0	2753	12	Ankylosing spondylitis	Feb-09
25292	PK1..00	181	0	19	0	Anomalies of adrenal gland	Feb-09
36064	PK1z.00	23	0	0	0	Anomalies of adrenal gland NOS	Feb-09
28318	P22A.00	132	0	3	0	Anomalies of cerebellum	Feb-09

For peer review only - http://bmjopen.bmj.com/site/about/guidelines.xhtml

39724	P227.00	15	0	1	0	Anomalies of cerebrum	Feb-09
32468	P228.00	143	0	7	0	Anomalies of corpus callosum	Feb-09
31416	PG6..00	171	0	7	0	Anomalies of diaphragm	Feb-09
42684	P74..00	34	0	0	0	Anomalies of great veins	Feb-09
46471	P229.00	17	0	0	0	Anomalies of hypothalamus	Feb-09
30055	PB7z.00	45	0	0	0	Anomalies of pancreas NOS	Feb-09
39588	PK27.00	23	0	3	0	Anomalies of parathyroid gland NEC	Feb-09
32424	PK24.00	191	0	12	1	Anomalies of pituitary gland	Feb-09
50786	P76C.00	34	0	1	0	Anomalies of renal artery NEC	Feb-09
45429	PG1z.00	55	0	1	0	Anomalies of spine NOS	Feb-09
26338	PK0..00	151	0	12	0	Anomalies of spleen	Feb-09
59625	PK0z.00	21	0	0	0	Anomalies of spleen NOS	Feb-09
40030	P72..11	103	0	16	0	Anomalies of the aorta excluding coarction	Feb-09
66465	PG3z.11	3	0	0	0	Anomalies of thoracic cage unspecified	Feb-09
39366	PK28.00	37	0	1	0	Anomalies of thymus	Feb-09
57704	PK26.00	45	0	4	0	Anomalies of thyroglossal duct NEC	Feb-09
10638	PK25.00	271	0	44	0	Anomalies of thyroid gland NEC	Feb-09
55080	PAzz.00	5	0	0	0	Anomalies of upper alimentary tract NOS	Feb-09
30315	PD7..00	55	0	0	0	Anomalies of urachus	Feb-09
56889	PD7z.00	2	0	0	0	Anomalies of urachus NOS	Feb-09
103432	P6z1000	2	0	0	0	Anomalous atrial bands	Dec-11
25147	G567.00	224	0	1	0	Anomalous atrioventricular excitation	Feb-09
42803	G567z00	3	0	0	0	Anomalous atrioventricular excitation NOS	Feb-09
109236	P6z1.00	1	0	0	0	Anomalous bands of heart	Jan-15
31373	P6y4400	12	0	1	0	Anomalous coronary artery communication	Feb-09
67286	P721000	1	0	0	0	Anomalous origin of the aortic arch	Feb-09

For peer review only - http://bmjopen.bmj.com/site/about/guidelines.xhtml

101307	P743.00	3	0	0	0	Anomalous portal vein termination	Jan-11
105445	ZR1N.00	3	0	0	0	Anomalous sentences repetition test	Dec-12
63677	P742.11	5	0	0	0	Anomalous termination of right pulmonary vein	Feb-09
64778	P6z1100	8	0	0	0	Anomalous ventricular bands	Feb-09
62230	P720.00	37	0	1	0	"Anomaly of aorta, unspecified"	Feb-09
42389	P761.00	79	0	7	1	Anomaly of artery NEC	Feb-09
44253	P22Az00	19	0	0	0	Anomaly of cerebellum NOS	Feb-09
111258	P227z00	2	0	0	0	Anomaly of cerebrum NOS	Aug-16
60648	P228z00	9	0	0	0	Anomaly of corpus callosum NOS	Feb-09
45291	P740.00	21	0	1	0	"Anomaly of great veins, unspecified"	Feb-09
63530	P831.00	10	0	0	0	Anomaly of laryngeal and tracheal cartilage	Feb-09
55062	P831z00	7	0	0	0	Anomaly of laryngeal or tracheal cartilage NOS	Feb-09
63453	P860.00	16	0	0	0	"Anomaly of lung, unspecified"	Feb-09
100681	PK27z00	3	0	0	0	Anomaly of parathyroid gland NEC NOS	Sep-10
70576	PK24z00	25	0	0	0	Anomaly of pituitary gland NOS	Feb-09
97401	P76Cz00	4	0	0	0	Anomaly of renal artery NEC NOS	Aug-09
60066	PG10.00	29	0	1	0	"Anomaly of spine, unspecified"	Feb-09
74890	P740000	8	0	0	0	"Anomaly of the pulmonary veins, unspecified"	Feb-09
50886	P740100	30	0	0	0	"Anomaly of the vena cava, unspecified"	Feb-09
48842	PK28z00	7	0	0	0	Anomaly of thymus gland NOS	Feb-09
47467	P831200	50	0	6	0	Anomaly of thyroid cartilage	Feb-09
70581	PK25z00	12	0	0	0	Anomaly of thyroid gland NEC NOS	Feb-09
37358	PA10.00	36	0	3	0	"Anomaly of tongue, unspecified"	Feb-09
31020	P831300	13	0	1	0	Anomaly of tracheal cartilage	Feb-09

For peer review only - http://bmjopen.bmj.com/site/about/guidelines.xhtml

23605	P30..00	108	0	6	1	Anophthalmos	Feb-09
37557	P30z.00	6	0	0	0	Anophthalmos NOS	Feb-09
70742	P300z00	2	0	0	0	Anophthalmos NOS	Feb-09
2135	E271.00	26924	0	1771	0	Anorexia nervosa	Feb-09
21889	F15..00	209	0	11	0	Anterior horn cell disease	Feb-09
58729	F15z.00	3	0	0	0	Anterior horn cell disease NOS	Feb-09
43908	C134z11	28	0	0	0	Anterior pituitary hormone deficiency NEC	Feb-09
43347	G121.11	193	0	7	0	Aortic incompetence - rheumatic	Feb-09
1007	G541200	2670	0	168	0	"Aortic incompetence alone, cause unspecified"	Feb-09
14998	G541000	3238	0	146	0	"Aortic incompetence, non-rheumatic"	Feb-09
58810	G541211	5	0	0	0	"Aortic insufficiency alone, cause unspecified"	Feb-09
47887	G541011	10	0	1	0	"Aortic insufficiency, non-rheumatic"	Feb-09
7963	G121.12	909	0	67	3	Aortic regurgitation - rheumatic	Feb-09
1005	G541212	7183	0	121	0	"Aortic regurgitation alone, cause unspecified"	Feb-09
10187	G541012	4918	0	146	0	"Aortic regurgitation, non-rheumatic"	Feb-09
98277	G71A.00	486	0	21	0	Aortic root dilatation	Dec-09
93699	791C000	14	0	0	0	Aortic root replac us pul val auto ri vent pulm art val cond	Feb-09
71668	791C400	196	0	0	0	Aortic root replacement	Feb-09
70353	791C200	26	0	0	0	Aortic root replacement using homograft	Feb-09
91004	791C300	58	0	1	0	Aortic root replacement using mechanical prosthesis	Feb-09
37405	P50..11	31	0	5	0	Aortic septal defect	Feb-09
45505	P501.00	17	0	0	0	Aortic septal defect	Feb-09

For peer review only - http://bmjopen.bmj.com/site/about/guidelines.xhtml

9591	G541500	27546	0	463	0	Aortic stenosis	Feb-09
2343	G541300	14659	0	932	5	"Aortic stenosis alone, cause unspecified"	Feb-09
999	G541100	18331	0	1242	1	"Aortic stenosis, non-rheumatic"	Feb-09
49185	G541700	357	0	2	0	Aortic valve calcification	Feb-09
4548	G541.00	10916	0	644	6	Aortic valve disorders	Feb-09
30610	G541600	2196	0	11	0	Aortic valve sclerosis	Feb-09
10964	G541400	456	0	13	0	Aortic valve stenosis with insufficiency	Feb-09
65330	P501.12	2	0	0	0	Aorticopulmonary septal defect	Feb-09
37402	PF55.11	66	0	32	0	Apert's syndrome	Feb-09
95752	PK15.00	1	0	0	0	Aplasia of adrenal gland	Feb-09
59298	P722100	1	0	0	0	Aplasia of aorta	Feb-09
100305	P221.00	2	0	0	0	"Aplasia of brain, part unspecified"	Jul-10
91846	P22A200	3	0	0	0	Aplasia of cerebellum	Feb-09
103057	P228200	1	0	0	0	Aplasia of corpus callosum	Oct-11
59944	P344600	1	0	0	0	Aplasia of iris	Feb-09
52664	P850.00	4	0	0	0	Aplasia of lung	Feb-09
45473	PGyy400	10	0	1	0	Aplasia of muscle	Feb-09
43166	D201100	10	0	0	0	Aplastic anaemia due to drugs	Feb-09
57859	D201200	5	0	0	0	Aplastic anaemia due to infection	Feb-09
66239	D201400	2	0	0	0	Aplastic anaemia due to toxic cause	Feb-09
78	H33..00	4382665	0	46202	206	Asthma	Feb-09
23481	G581.11	436	0	56	0	Asthma - cardiac	Feb-09
10487	663j.00	12298	0	7	0	Asthma - currently active	Feb-09
232	H33z100	89016	0	4269	5	Asthma attack	Feb-09
8335	H33z111	2604	0	12	0	Asthma attack NOS	Feb-09
11370	1O2..00	12868	0	34	0	Asthma confirmed	Feb-09
4442	H33z.00	42919	0	985	12	Asthma unspecified	Feb-09
52659	F23y400	159	0	1	0	Ataxic diplegic cerebral palsy	Feb-09
38800	ZS42411	24	0	0	0	Ataxic dysarthria	Feb-09
21548	F23y000	141	0	4	0	Ataxic infantile cerebral palsy	Feb-09

For peer review only - http://bmjopen.bmj.com/site/about/guidelines.xhtml

68198	P722.00	10	0	0	0	Atresia and stenosis of aorta	Feb-09
51672	PB2..00	22	0	0	0	Atresia and stenosis of large intestine/rectum/anal canal	Feb-09
35968	PB2z.00	41	0	6	0	Atresia and stenosis of large intestine/rectum/anus NOS	Feb-09
27598	PB2..11	27	0	0	0	Atresia large intestine	Feb-09
95045	PD61000	3	0	0	0	Atresia of anterior urethra	Feb-09
31731	PB21000	24	0	0	0	Atresia of anus	Feb-09
99653	PB21300	1	0	0	0	Atresia of appendix	Jun-10
68310	P762.00	3	0	0	0	Atresia of artery NEC	Feb-09
38389	PB61400	4	0	0	0	Atresia of bile duct	Feb-09
97475	PD60000	2	0	0	0	Atresia of bladder neck	Aug-09
93089	P6yy000	4	0	0	0	Atresia of cardiac vein	Feb-09
65385	PC4yA00	7	0	0	0	Atresia of cervix	Feb-09
23593	PB21100	60	0	1	0	Atresia of colon	Feb-09
19039	PB10100	246	0	2	0	Atresia of duodenum	Feb-09
56100	PCy3000	23	0	5	0	Atresia of ejaculatory duct	Feb-09
61727	P832000	2	0	0	0	Atresia of epiglottis	Feb-09
25819	P402000	119	0	6	0	Atresia of external auditory canal	Feb-09
61770	PC1y200	4	0	1	0	Atresia of fallopian tube	Feb-09
51345	P233.00	10	0	0	0	Atresia of foramina of Magendie and Luschka	Feb-09
95015	PCy3.00	2	0	0	0	Atresia of genital organ NEC	Feb-09
62335	PCy3z00	1	0	0	0	Atresia of genital organ NEC NOS	Feb-09
92834	P832100	3	0	0	0	Atresia of glottis	Feb-09
37451	P6yy700	10	0	0	0	Atresia of heart valve NEC	Feb-09
50400	PB61600	8	0	0	0	Atresia of hepatic ducts	Feb-09
53565	PB10200	59	0	0	0	Atresia of ileum	Feb-09
52687	PB10300	86	0	1	0	Atresia of jejunum	Feb-09
31468	PB21.00	36	0	5	0	Atresia of large intestine	Feb-09
72001	P832.00	2	0	0	0	Atresia of larynx and trachea	Feb-09
52894	P8y2.00	5	0	0	0	Atresia of nasopharynx	Feb-09

For peer review only - http://bmjopen.bmj.com/site/about/guidelines.xhtml

33655	PA37.00	133	0	1	0	Atresia of oesophagus with tracheo-oesophageal fistula	Feb-09
73673	P344100	5	0	0	0	Atresia of pupil	Feb-09
46123	PB21200	21	0	0	0	Atresia of rectum	Feb-09
71153	PB21500	9	0	0	0	Atresia of rectum with fistula	Feb-09
33584	PB10.00	115	0	1	0	Atresia of small intestine	Feb-09
40252	PB10000	36	0	3	0	"Atresia of small intestine, unspecified"	Feb-09
68606	P802.00	2	0	0	0	Atresia of the posterior nares	Feb-09
63288	P832300	11	0	0	0	Atresia of trachea	Feb-09
49700	PD20.00	7	0	0	0	Atresia of ureter	Feb-09
27648	K562300	53	0	6	0	Atresia of vagina	Feb-09
36548	PC4yB00	32	0	3	0	Atresia of vagina	Feb-09
73156	K562.12	2	0	0	0	Atresia of vagina	Feb-09
58076	P722z00	36	0	0	0	Atresia or stenosis of aorta NOS	Feb-09
34305	PA22.00	42	0	5	0	"Atresia, salivary duct"	Feb-09
1664	G573000	374276	0	21137	103	Atrial fibrillation	Feb-09
2212	G573.00	146690	0	11825	6	Atrial fibrillation and flutter	Feb-09
23437	G573z00	1241	0	29	0	Atrial fibrillation and flutter NOS	Feb-09
45773	6A9..00	17151	0	49	0	Atrial fibrillation annual review	Feb-09
105554	8CMW200	518	0	1	0	Atrial fibrillation care pathway	Jan-13
104570	1I12.00	82	0	0	0	Atrial fibrillation excluded	Jul-12
57832	9Os..00	5501	0	150	0	Atrial fibrillation monitoring administration	Feb-09
28994	212R.00	22216	0	83	0	Atrial fibrillation resolved	Feb-09
9023	G576300	200	0	17	0	Atrial premature depolarization	Feb-09
3255	P550.00	11410	0	274	1	Atrial septal defect NOS	Feb-09
23708	G361.00	35	0	2	0	Atrial septal defect/curr comp folow acut myocardial infarct	Feb-09
27375	G561z00	173	0	8	0	Atrioventricular block NOS	Feb-09
58032	G561000	80	0	0	0	Atrioventricular block unspecified	Feb-09
28543	ZS91.00	878	0	28	0	Attention deficit disorder	Feb-09

For peer review only - http://bmjopen.bmj.com/site/about/guidelines.xhtml

101067	6A61.00	2631	0	816	0	Attention deficit hyperactivity disorder annual review	Dec-10
34199	E2E0000	84	0	3	0	Attention deficit without hyperactivity	Feb-09
24244	E14y000	4	0	0	0	Atypical childhood psychoses	Feb-09
1276	E140.12	18470	0	1122	1	Autism	Feb-09
7957	J614111	1100	0	35	0	Autoimmune chronic active hepatitis	Feb-09
26410	C394.00	1332	0	57	1	Autoimmune disease NOS	Feb-09
39876	D110z00	238	0	3	0	Autoimmune haemolytic anaemia NOS	Feb-09
3818	D110.00	1773	0	106	2	Autoimmune haemolytic anaemias	Feb-09
18652	J63B.00	2558	0	41	0	Autoimmune hepatitis	Feb-09
31971	C046.00	910	0	4	0	Autoimmune myxoedema	Feb-09
61859	C121000	7	0	1	0	Autoimmune parathyroiditis	Feb-09
52843	C182.00	65	0	2	0	Autoimmune polyglandular failure	Feb-09
16420	42P2.11	378	0	109	459	Auto-immune thrombocytopenia	Feb-09
3857	C052.11	2107	0	108	24	Autoimmune thyroiditis	Feb-09
106346	C353800	2	0	0	0	Autosomal dominant hypophosphataemic rickets	Jun-13
110208	PD12211	1	0	0	0	Autosomal dominant medullary cystic disease	Oct-15
105143	PD11111	56	0	0	0	Autosomal dominant polycystic kidney disease	Nov-12
105794	PD12012	3	0	0	0	Autosomal recessive medullary cystic disease	Feb-13
105919	PD11011	8	0	0	0	Autosomal recessive polycystic kidney disease	Apr-13
40288	C151200	184	0	8	0	Bartter's syndrome	Feb-09
47778	C151.12	17	0	0	0	Bartter's syndrome	Feb-09
32749	F391800	373	0	28	0	Becker muscular dystrophy	Feb-09
73283	G552.11	8	0	1	0	Becker's disease	Feb-09

For peer review only

12357	PKy9100	175	0	6	0	Beckwith's syndrome	Feb-09
16235	AD61.00	3552	0	263	1	Behcet's syndrome	Feb-09
58818	N012011	14	0	2	0	Behcet's syndrome arthropathy	Feb-09
63333	F390000	21	0	0	0	Benign congenital myopathy	Feb-09
19170	F25y400	1212	0	15	0	Benign Rolandic epilepsy	Feb-09
57144	D104600	54	0	1	0	Beta intermedia thalassaemia	Feb-09
27761	D104800	448	0	5	1	Beta minor thalassaemia	Feb-09
9864	D104500	10190	0	80	23	Beta trait thalassaemia	Feb-09
3300	P641.00	4594	0	140	0	Bicuspid aortic valve	Feb-09
57458	PKy1.11	63	0	4	0	Biedl-Bardet syndrome	Feb-09
55370	D010.12	4	0	0	0	Biermer's congenital pernicious anaemia	Feb-09
54721	P913.00	24	0	1	0	Bilateral complete cleft lip	Feb-09
48948	P903.00	20	0	0	0	Bilateral complete cleft palate	Feb-09
44977	P923.00	20	0	0	0	Bilateral complete cleft palate with cleft lip	Feb-09
71154	PD02000	5	0	0	0	Bilateral congenital absence of kidneys	Feb-09
38373	PE30100	256	0	0	0	Bilateral congenital dislocation of hip	Feb-09
107323	F591A00	36	0	3	0	Bilateral congenital sensorineural hearing loss	Dec-13
61870	PE31100	43	0	0	0	Bilateral congenital subluxation of hip	Feb-09
4035	1C13300	3079	0	380	0	Bilateral deafness	Feb-09
34285	PE34100	160	0	0	0	Bilateral dysplastic hip	Feb-09
106078	P904.00	1	0	0	0	Bilateral incomplete cleft palate	May-13
62690	P924.00	4	0	0	0	Bilateral incomplete cleft palate with cleft lip	Feb-09
9500	PD04000	49	0	0	0	Bilateral renal dysplasia	Feb-09
42925	PD03000	12	0	2	0	Bilateral renal hypoplasia	Feb-09
38774	K091.00	122	0	2	0	Bilateral small kidneys	Feb-09

For peer review only - http://bmjopen.bmj.com/site/about/guidelines.xhtml

50468	PE35100	103	0	0	0	Bilateral unstable hip	Feb-09
40425	PB6..11	93	0	7	0	Bile duct anomalies	Feb-09
53764	PB60200	16	0	1	0	"Bile duct anomaly, unspecified"	Feb-09
16005	J66z.00	676	0	40	2	Bile duct disorder NOS	Feb-09
9494	J616.00	2107	0	74	0	Biliary cirrhosis	Feb-09
58630	J616z00	47	0	0	0	Biliary cirrhosis NOS	Feb-09
91591	J616200	1	0	0	0	Biliary cirrhosis of children	Feb-09
55829	E114400	40	0	1	0	"Bipolar affect disord, currently manic,severe with psychosis"	Feb-09
72026	E115500	6	0	0	0	"Bipolar affect disord, now depressed, part/unspec remission"	Feb-09
63701	E115400	19	0	0	0	"Bipolar affect disord, now depressed, severe with psychosis"	Feb-09
35607	E115300	30	0	0	0	"Bipolar affect disord, now depressed, severe, no psychosis"	Feb-09
59011	E114500	8	0	0	0	"Bipolar affect disord,currently manic, part/unspec remission"	Feb-09
4677	E115.00	524	0	37	0	"Bipolar affective disorder, currently depressed"	Feb-09
35734	E115100	65	0	0	0	"Bipolar affective disorder, currently depressed, mild"	Feb-09
27890	E115200	63	0	1	0	"Bipolar affective disorder, currently depressed, moderate"	Feb-09
37296	E115z00	87	0	3	0	"Bipolar affective disorder, currently depressed, NOS"	Feb-09
3702	E114.00	507	0	38	0	"Bipolar affective disorder, currently manic"	Feb-09
63784	E114600	46	0	1	0	"Bipolar affective disorder, currently manic, full remission"	Feb-09
36126	E114100	55	0	1	0	"Bipolar affective disorder, currently manic, mild"	Feb-09

For peer review only - http://bmjopen.bmj.com/site/about/guidelines.xhtml

46434	E114200	40	0	1	0	"Bipolar affective disorder, currently manic, moderate"	Feb-09
57605	E114z00	64	0	1	0	"Bipolar affective disorder, currently manic, NOS"	Feb-09
35738	E114000	70	0	1	0	"Bipolar affective disorder, currently manic, unspecified"	Feb-09
57465	E115600	75	0	0	0	"Bipolar affective disorder, now depressed, in full remission"	Feb-09
8567	E11..11	6557	0	431	0	Bipolar psychoses	Feb-09
32900	D200311	173	0	5	0	Blackfan - Diamond syndrome	Feb-09
109273	D200.13	0	0	0	0	Blackfan - Diamond syndrome	Feb-15
33762	PG0y000	727	0	65	0	Brachycephaly	Feb-09
20676	G820.00	259	0	2	0	Budd - Chiari syndrome (hepatic vein thrombosis)	Feb-09
29180	G5y9.00	56	0	1	0	"Cardiac septal defect, acquired"	Feb-09
21943	P5...11	70	0	11	0	Cardiac septal defects	Feb-09
509	G5y3.00	16459	0	1620	47	Cardiomegaly	Feb-09
10890	Z7E4400	1867	0	167	0	Cerebellar ataxia	Feb-09
48731	Q200700	12	0	0	0	Cerebral haemorrhage due to birth injury	Feb-09
46545	S62z.00	50	0	0	0	Cerebral haemorrhage following injury NOS	Feb-09
23447	Q200000	97	0	0	0	"Cerebral haemorrhage unspecified, due to birth trauma"	Feb-09
104498	F2B..00	351	0	2	0	Cerebral palsy	Jul-12
104654	F2Bz.00	30	0	0	0	Cerebral palsy NOS	Aug-12
5512	F230100	1189	0	27	0	Cerebral palsy with spastic diplegia	Feb-09
16956	G669.00	511	0	20	0	"Cerebral palsy, not congenital or infantile, acute"	Feb-09
7529	F286.11	19513	0	1701	0	CFS - Chronic fatigue syndrome	Feb-09
108711	K000111	1	0	0	0	CGN - Crescentic glomerulonephritis	Oct-14
5584	F361000	2642	0	294	0	Charcot-Marie-Tooth disease	Feb-09

For peer review only - http://bmjopen.bmj.com/site/about/guidelines.xhtml

96587	4L4C.00	13	0	10	34	Charcot-Marie-Tooth disease type 1A gene detection test	Apr-09
108834	F361012	2	0	0	0	Charcot-Marie-Tooth syndrome	Nov-14
43808	P2x6.00	28	0	0	0	Chiari malformation type I	Feb-09
62968	P101000	195	0	2	0	Chiari malformation type I	Feb-09
1208	H330.12	21212	0	112	3	Childhood asthma	Feb-09
7302	E140.13	343	0	4	0	Childhood autism	Feb-09
3775	E2E..00	7103	0	1175	1	Childhood hyperkinetic syndrome	Feb-09
37395	E14z.11	44	0	2	0	Childhood schizophrenia NOS	Feb-09
31027	P80..00	621	0	27	0	Choanal atresia	Feb-09
60514	P80z.00	14	0	0	0	Choanal atresia NOS	Feb-09
66643	P800.00	10	0	0	0	"Choanal atresia, unspecified"	Feb-09
22756	PG4..00	142	0	11	1	Chondrodysplasia	Feb-09
58035	PG56000	5	0	0	0	Chondrodysplasia calcificans congenita	Feb-09
63146	PG56011	2	0	0	0	Chondrodysplasia calcificans congenita	Feb-09
14917	PG4z.00	44	0	7	0	Chondrodysplasia NOS	Feb-09
18193	PG4C.00	24	0	0	0	Chondrodysplasia punctata	Feb-09
73905	PG40.00	3	0	0	0	"Chondrodysplasia, unspecified"	Feb-09
671	PJ...00	1722	0	52	5	Chromosomal anomalies	Feb-09
20231	PJz..00	509	0	19	1	Chromosomal anomalies NOS	Feb-09
104196	PJ33800	5	0	0	0	Chromosome 4q deletion syndrome	Jun-12
54241	PJ53000	34	0	0	0	Chromosome inversion in normal individual	Feb-09
106938	PJ38.11	3	0	0	0	Chromosome replaced with dicentric	Aug-13
47247	PJ38.12	11	0	0	0	Chromosome replaced with ring	Feb-09
100562	PJ38.00	1	0	0	0	Chromosome replaced with ring or dicentric	Aug-10
9029	J614100	2400	0	106	1	Chronic active hepatitis	Feb-09

For peer review only - http://bmjopen.bmj.com/site/about/guidelines.xhtml

106805	H335.00	166	0	5	0	Chronic asthma with fixed airflow obstruction	Aug-13
5798	H312000	1813	0	47	0	Chronic asthmatic bronchitis	Feb-09
4669	K02y200	195	0	7	0	Chronic focal glomerulonephritis	Feb-09
7804	K02..00	1957	0	75	0	Chronic glomerulonephritis	Feb-09
97758	K02y000	2	0	0	0	Chronic glomerulonephritis + diseases EC	Oct-09
15097	K02z.00	184	0	7	0	Chronic glomerulonephritis NOS	Feb-09
104981	K05..13	179	0	0	0	Chronic kidney disease	Sep-12
29013	1Z10.00	22548	0	89	0	Chronic kidney disease stage 1	Feb-09
105392	K051.00	23	0	0	0	Chronic kidney disease stage 1	Dec-12
94789	1Z17.00	132	0	3	0	Chronic kidney disease stage 1 with proteinuria	Feb-09
95572	1Z18.00	228	0	1	0	Chronic kidney disease stage 1 without proteinuria	Feb-09
105383	K052.00	416	0	0	0	Chronic kidney disease stage 2	Dec-12
95146	1Z19.00	500	0	1	0	Chronic kidney disease stage 2 with proteinuria	Feb-09
95121	1Z1A.00	2368	0	4	0	Chronic kidney disease stage 2 without proteinuria	Feb-09
73026	K0A3500	3	0	0	0	Chronic neph syn difus mesangiocapillary glomerulonephritis	Feb-09
60857	K0A3700	18	0	0	0	Chronic nephritic syn diffuse crescentic glomerulonephritis	Feb-09
40413	K0A3100	10	0	0	0	Chronic nephritic syndrm focal+segmental glomerular lesions	Feb-09
21297	K0A3.00	86	0	3	0	Chronic nephritic syndrome	Feb-09
60198	K0A3600	7	0	0	0	"Chronic nephritic syndrome, dense deposit disease"	Feb-09
66505	K0A3000	5	0	0	0	"Chronic nephritic syndrome, minor glomerular abnormality"	Feb-09

For peer review only - http://bmjopen.bmj.com/site/about/guidelines.xhtml

24814	H591.00	102	0	1	0	Chronic respiratory failure	Feb-09
9312	G1...00	1190	0	43	0	Chronic rheumatic heart disease	Feb-09
6863	J61..00	7053	0	402	1	Cirrhosis and chronic liver disease	Feb-09
97980	1Z17.11	5	0	0	0	CKD stage 1 with proteinuria	Nov-09
111022	1Z18.11	1	0	0	0	CKD stage 1 without proteinuria	Jul-16
97979	1Z19.11	22	0	0	0	CKD stage 2 with proteinuria	Nov-09
97978	1Z1A.11	35	0	0	0	CKD stage 2 without proteinuria	Nov-09
110033	1Z1M.00	8	0	0	0	CKD with GFR category G1 & albuminuria category A1	Aug-15
110003	1Z1N.00	5	0	0	0	CKD with GFR category G1 & albuminuria category A2	Aug-15
110484	1Z1P.00	2	0	0	0	CKD with GFR category G1 & albuminuria category A3	Jan-16
110269	1Z1Q.00	86	0	0	0	CKD with GFR category G2 & albuminuria category A1	Nov-15
110108	1Z1R.00	40	0	0	0	CKD with GFR category G2 & albuminuria category A2	Sep-15
110251	1Z1S.00	6	0	0	0	CKD with GFR category G2 & albuminuria category A3	Nov-15
65623	P907.11	23	0	1	0	Cleft hard palate NOS	Feb-09
33902	P92A.00	12	0	0	0	"Cleft hard palate with cleft lip, bilateral"	Feb-09
62321	P92B.00	13	0	0	0	"Cleft hard palate with cleft lip, unilateral"	Feb-09
93972	P928.00	5	0	0	0	"Cleft hard palate with cleft soft palate, unilateral"	Feb-09
64549	P90B.00	2	0	0	0	"Cleft hard palate, bilateral"	Feb-09
38873	P901.11	2	0	0	0	"Cleft hard palate, unilateral"	Feb-09
101223	P90C.00	3	0	0	0	"Cleft hard palate, unilateral"	Dec-10
2027	P91..00	1719	0	65	0	Cleft lip (harelip)	Feb-09
35374	P91z.00	139	0	2	0	Cleft lip NOS	Feb-09
15364	P90z.00	237	0	39	0	Cleft palate NOS	Feb-09

For peer review only - http://bmjopen.bmj.com/site/about/guidelines.xhtml

50430	P9z..00	90	0	1	0	Cleft palate or cleft lip NOS	Feb-09
68398	P92z.00	22	0	2	0	Cleft palate with cleft lip NOS	Feb-09
51301	P920.00	19	0	0	0	"Cleft palate with cleft lip, unspecified"	Feb-09
58885	P900.00	75	0	0	0	"Cleft palate, unspecified"	Feb-09
25740	P908.11	114	0	2	0	Cleft soft palate NOS	Feb-09
42208	P90A.00	22	0	1	0	"Cleft soft palate, bilateral"	Feb-09
97477	P904.11	3	0	0	0	"Cleft soft palate, bilateral"	Aug-09
68166	P906.11	8	0	0	0	"Cleft soft palate, central"	Feb-09
57490	P902.12	2	0	1	0	"Cleft soft palate, unilateral"	Feb-09
1515	J690.00	55378	0	3179	25	Coeliac disease	Feb-09
44310	J690z00	738	0	19	0	Coeliac disease NOS	Feb-09
62236	C392.00	8	0	0	0	Combined immunity deficiency	Feb-09
62328	C392z00	3	0	0	0	Combined immunity deficiency NOS	Feb-09
43049	P56z100	21	0	1	0	Common atrioventricular canal	Feb-09
44896	P56z200	286	0	3	0	Common atrioventricular-type ventricular septal defect	Feb-09
55535	P56z000	35	0	3	0	Common atrium	Feb-09
40765	SB00100	4	0	0	0	Common carotid artery injury	Feb-09
98893	P50..12	3	0	0	0	Common truncus	Apr-10
66857	C398200	2	0	1	0	Common variable immunodef wth autoantibod to B- or T-cells	Feb-09
21975	C398.00	519	0	7	0	Common variable immunodeficiency	Feb-09
57322	C390700	20	0	0	0	Common variable immunodeficiency	Feb-09
30187	PKy5.00	52	0	6	0	Congen malformation syndromes affecting facial appearance	Feb-09
62705	PF6B300	2	0	0	0	Congen overgrowth of whole lower limb	Feb-09
109067	PD8..00	19	0	1	0	Congenital abnormality of the kidney	Jan-15

For peer review only - http://bmjopen.bmj.com/site/about/guidelines.xhtml

59430	PB20000	11	0	0	0	Congenital absence of anus	Feb-09
93826	PB20300	4	0	0	0	Congenital absence of anus with fistula	Feb-09
16417	PB61000	16	0	2	0	Congenital absence of bile duct	Feb-09
39970	PDy0.00	10	0	0	0	Congenital absence of bladder	Feb-09
48901	PF5F.00	32	0	3	0	Congenital absence of both forearm and hand	Feb-09
25316	P22A000	2	0	0	0	Congenital absence of cerebellum	Feb-09
68093	PG13000	2	0	0	0	Congenital absence of cervical vertebra	Feb-09
35942	P4y4.00	5	0	0	0	Congenital absence of chin	Feb-09
57747	PC4y200	4	0	0	0	Congenital absence of clitoris	Feb-09
93830	PG1x000	3	0	0	0	Congenital absence of coccyx	Feb-09
71956	P6y4000	1	0	0	0	Congenital absence of coronary artery	Feb-09
41775	P228000	53	0	2	0	Congenital absence of corpus callosum	Feb-09
60738	PBy0.00	2	0	0	0	Congenital absence of digestive system NOS	Feb-09
68208	P300200	6	0	0	0	Congenital absence of eye	Feb-09
67756	P303.00	3	0	0	0	Congenital absence of eyes	Feb-09
33394	PF29.00	78	0	4	0	Congenital absence of finger	Feb-09
27624	PF25.00	39	0	2	0	Congenital absence of forearm only	Feb-09
42256	PB63000	13	0	0	0	Congenital absence of gallbladder	Feb-09
46835	PF21613	68	0	1	0	Congenital absence of hand	Feb-09
48269	PB61500	2	0	0	0	Congenital absence of hepatic ducts	Feb-09
18406	PB11200	1	0	0	0	Congenital absence of ileum	Feb-09
3314	PD02.00	2413	0	57	0	Congenital absence of kidney	Feb-09
65407	PD02z00	53	0	0	0	Congenital absence of kidney NOS	Feb-09
58855	PB20.00	2	0	0	0	Congenital absence of large intestine	Feb-09
60525	PF31200	8	0	0	0	Congenital absence of leg and foot	Feb-09

For peer review only - http://bmjopen.bmj.com/site/about/guidelines.xhtml

62531	P335.11	1	0	0	0	Congenital absence of lens	Feb-09
28661	PF40.00	6	0	0	0	Congenital absence of limb NOS	Feb-09
98402	PB63100	2	0	0	0	Congenital absence of liver lobe	Jan-10
97185	P853100	3	0	0	0	Congenital absence of lobe of lung	Aug-09
72561	PF35.00	5	0	0	0	Congenital absence of lower leg only	Feb-09
54384	P853.11	5	0	0	0	Congenital absence of lung	Feb-09
68268	P853000	5	0	0	0	Congenital absence of lung fissures	Feb-09
98859	PA35.00	1	0	0	0	Congenital absence of oesophagus	Apr-10
64339	PF39400	8	0	0	0	Congenital absence of other multiple toes	Feb-09
42571	PC00.00	45	0	1	0	Congenital absence of ovary	Feb-09
61638	PF64000	14	0	1	0	Congenital absence of patella	Feb-09
49245	PCy0000	4	0	0	0	Congenital absence of penis	Feb-09
101896	P6yy411	2	0	0	0	Congenital absence of pericardium	Mar-11
111629	PK24100	1	0	0	0	Congenital absence of pituitary gland	Jan-17
103672	P731.11	2	0	0	0	Congenital absence of pulmonary artery	Feb-12
97779	P364111	2	0	0	0	Congenital absence of punctum lacrimale	Oct-09
50443	PB20200	7	0	0	0	Congenital absence of rectum	Feb-09
58095	PB20400	1	0	0	0	Congenital absence of rectum with fistula	Feb-09
42378	PG30.00	23	0	0	0	Congenital absence of rib	Feb-09
43351	PG13300	12	0	1	0	Congenital absence of sacrum	Feb-09
68081	PG1x100	1	0	0	0	Congenital absence of sacrum	Feb-09
38119	PG00.00	1	0	0	0	Congenital absence of skull bones	Feb-09
63291	PG31.00	4	0	0	0	Congenital absence of sternum	Feb-09
73987	PK28100	3	0	0	0	Congenital absence of thymus	Feb-09
3374	PK25100	233	0	26	1	Congenital absence of thyroid gland	Feb-09

For peer review only - http://bmjopen.bmj.com/site/about/guidelines.xhtml

72180	PF24.00	2	0	0	0	Congenital absence of upper arm only	Feb-09
37973	PF21.11	30	0	1	0	Congenital absence part of arm	Feb-09
37277	PF23.00	14	0	0	0	Congenital absence upper arm and forearm with hand present	Feb-09
23416	PA70.11	47	0	2	0	Congenital achalasia of cardia	Feb-09
36786	C152813	9	0	0	0	Congenital adrenal gland hypertrophy NEC	Feb-09
29640	C152000	304	0	14	0	Congenital adrenogenital syndrome	Feb-09
59314	D303000	11	0	0	0	Congenital afibrinogenaemia	Feb-09
16618	PH40.00	94	0	3	0	Congenital alopecia	Feb-09
98667	PH40z00	1	0	0	0	Congenital alopecia NOS	Mar-10
104030	PH40000	2	0	0	0	"Congenital alopecia, unspecified"	May-12
9884	F480011	3463	0	170	0	Congenital amblyopia	Feb-09
53564	PF45.00	15	0	0	0	Congenital amputation of unspecified limb	Feb-09
59307	PF21400	47	0	0	0	Congenital amputation of upper limb	Feb-09
31248	Q455.00	115	0	2	0	Congenital anaemia	Feb-09
99494	Q455000	3	0	0	0	Congenital anaemia from fetal blood loss	May-10
37080	PB59.00	71	0	10	0	Congenital anal fistula	Feb-09
35019	PB24.11	53	0	9	0	Congenital anal stricture	Feb-09
63173	P7yz000	13	0	2	0	Congenital aneurysm NEC	Feb-09
56200	P72z100	23	0	1	0	Congenital aneurysm of aorta	Feb-09
108193	P6yy600	1	0	0	0	Congenital aneurysm of heart	Jun-14
54295	PF6y000	25	0	0	0	Congenital angulation of tibia	Feb-09
44851	P344000	156	0	4	0	Congenital anisocoria	Feb-09
47533	J000200	9	0	1	0	Congenital anodontia	Feb-09
5574	PG1u.00	57	0	0	0	Congenital anomalies of cervical vertebrae NEC	Feb-09

For peer review only - http://bmjopen.bmj.com/site/about/guidelines.xhtml

24218	PF5v.00	20	0	1	0	Congenital anomalies of elbow and upper arm	Feb-09
94280	P36..00	6	0	0	0	"Congenital anomalies of eyelid, lacrimal system and orbit"	Feb-09
94285	P35z.00	3	0	0	0	Congenital anomalies of posterior chamber NOS	Feb-09
40690	PG1v.00	26	0	1	0	Congenital anomalies of thoracic vertebrae NEC	Feb-09
55893	P7y0000	9	0	0	0	"Congenital anomaly of cerebral vessel, unspecified"	Feb-09
41763	PG1uz00	12	0	2	0	Congenital anomaly of cervical vertebrae NEC NOS	Feb-09
37784	P4z1.00	22	0	2	0	Congenital anomaly of face NOS	Feb-09
66463	PG1wz00	12	0	0	0	Congenital anomaly of lumbar vertebra NEC NOS	Feb-09
68894	P6yy500	3	0	0	0	Congenital anomaly of myocardium	Feb-09
93198	P4z0.00	4	0	0	0	Congenital anomaly of neck NOS	Feb-09
108675	P76y.00	3	0	0	0	Congenital anomaly of peripheral vascular system OS	Oct-14
45918	P25y200	3	0	0	0	Congenital anomaly of spinal meninges	Feb-09
71152	PG1vz00	6	0	0	0	Congenital anomaly of thoracic vertebrae NEC NOS	Feb-09
9723	PFz..00	347	0	6	0	Congenital anomaly of unspecified limb NOS	Feb-09
8636	P64..00	65	0	4	0	Congenital aortic valve insufficiency	Feb-09
6843	P64z.00	10	0	0	0	Congenital aortic valve insufficiency NOS	Feb-09
58734	P640.00	18	0	0	0	"Congenital aortic valve insufficiency, unspecified"	Feb-09
6886	P63..00	4086	0	265	0	Congenital aortic valve stenosis	Feb-09
69823	P335.00	19	0	0	0	Congenital aphakia	Feb-09

For peer review only - http://bmjopen.bmj.com/site/about/guidelines.xhtml

94282	PCy1.00	1	0	0	0	Congenital aplasia of genital organ NEC	Feb-09
91739	PCy1000	2	0	0	0	Congenital aplasia of prostate	Feb-09
38020	PCy1300	3	0	0	0	Congenital aplasia of scrotum	Feb-09
58591	P769.00	4	0	0	0	Congenital arterial stricture	Feb-09
95800	P7y0111	3	0	0	0	Congenital arteriovenous fistula of brain	Feb-09
62587	P357100	12	0	1	0	Congenital arteriovenous malformation of retina	Feb-09
66314	F137111	10	0	0	0	Congenital athetosis	Feb-09
59553	PH40.11	4	0	0	0	Congenital atrichosis	Feb-09
22246	F38y.13	751	0	21	0	Congenital benign hypotonia	Feb-09
53454	PE07.00	23	0	5	0	Congenital bent or squashed nose	Feb-09
42721	P421.11	107	0	21	0	Congenital big ears	Feb-09
95892	P226000	8	0	0	0	Congenital bilateral perisylvian syndrome	Feb-09
57166	PDy5.00	8	0	1	0	Congenital bladder hernia	Feb-09
47854	PD60z00	14	0	0	0	Congenital bladder neck obstruction NOS	Feb-09
36666	P7y0200	92	0	2	0	Congenital brain aneurysm NEC	Feb-09
8893	P2y0.00	58	0	0	0	Congenital brain anomaly	Feb-09
56427	P861.00	20	0	1	0	Congenital bronchiectasis	Feb-09
32600	P843.12	100	0	3	0	Congenital bronchogenic cyst	Feb-09
11845	P83yB00	131	0	1	0	Congenital bronchomalacia	Feb-09
56531	PD31.00	42	0	2	0	Congenital calculus of kidney	Feb-09
34007	P6yy200	30	0	0	0	Congenital cardiomegaly	Feb-09
72271	PA70.00	3	0	0	0	Congenital cardiospasm	Feb-09
45926	P33z.00	96	0	6	0	Congenital cataract or lens anomaly NOS	Feb-09
299	P330.00	2282	0	166	0	"Congenital cataract, unspecified"	Feb-09
24424	P7y0100	216	0	17	0	Congenital cerebral arteriovenous aneurysm	Feb-09

For peer review only - http://bmjopen.bmj.com/site/about/guidelines.xhtml

44581	P7y0112	44	0	2	0	Congenital cerebral arteriovenous malformation	Feb-09
39391	P240.00	47	0	2	0	Congenital cerebral cyst	Feb-09
71733	P240z00	1	0	0	0	Congenital cerebral cyst NOS	Feb-09
53474	P20..13	6	0	0	0	Congenital cerebral hernia	Feb-09
2069	F23..00	14415	0	746	2	Congenital cerebral palsy	Feb-09
28306	F23z.00	496	0	11	0	Congenital cerebral palsy NOS	Feb-09
20979	PB6y000	216	0	12	0	Congenital choledochal cyst	Feb-09
34924	P352.00	107	0	4	0	Congenital chorioretinal degeneration	Feb-09
56810	P7yz100	69	0	0	0	Congenital chylothorax	Feb-09
30141	PF58.00	33	0	0	0	Congenital cleft hand	Feb-09
66698	P813.00	12	0	0	0	Congenital cleft nose	Feb-09
105605	P83y700	1	0	0	0	Congenital cleft of posterior cricoid cartilage	Jan-13
41019	PE8y011	38	0	0	0	Congenital club fingers	Feb-09
45651	PE8y000	80	0	5	0	Congenital club hand	Feb-09
23980	PE60.11	58	0	8	0	Congenital clubfoot - valgus	Feb-09
62397	J690000	21	0	0	0	Congenital coeliac disease	Feb-09
48508	PF5G.00	28	0	0	0	Congenital complete absence of upper limb(s)	Feb-09
52310	P6y5100	32	0	0	0	Congenital complete atrio-ventricular heart block	Feb-09
52822	PG15000	4	0	0	0	Congenital complete fusion of spine	Feb-09
31211	PKy7A00	10	0	0	0	Congenital contractural arachnodactyly	Feb-09
66259	P341z00	20	0	1	0	Congenital corneal opacities NOS	Feb-09
71732	P341000	4	0	1	0	Congenital corneal opacity with visual deficit	Feb-09
49901	P6y4500	5	0	0	0	Congenital coronary aneurysm	Feb-09
28705	P6y4411	7	0	3	0	Congenital coronary arterio-venous fistula	Feb-09

For peer review only - http://bmjopen.bmj.com/site/about/guidelines.xhtml

For peer review only

96586	P844.00	85	0	0	0	Congenital cystic adenomatoid malformation of the lung	Apr-09
51185	P301.00	17	0	1	0	Congenital cystic eyeball	Feb-09
15917	PD1..00	760	0	46	1	Congenital cystic kidney disease	Feb-09
50331	PD1z.00	34	0	1	0	Congenital cystic kidney disease NOS	Feb-09
27565	PB62.00	293	0	48	20	Congenital cystic liver disease	Feb-09
72000	PB62z00	5	0	0	0	Congenital cystic liver disease NOS	Feb-09
66223	P840.00	11	0	0	0	"Congenital cystic lung disease, unspecified"	Feb-09
33804	P84z.00	12	0	2	0	Congenital cystic lung NOS	Feb-09
60812	P353100	4	0	3	0	Congenital cysts of the posterior segment	Feb-09
59281	PG61.11	3	0	0	0	Congenital defect of diaphragmatic NEC	Feb-09
46289	D010.13	10	0	1	1	Congenital deficiency of intrinsic factor	Feb-09
57275	D303z00	52	0	1	0	Congenital deficiency of other clotting factor NOS	Feb-09
54725	D303y00	15	0	0	0	Congenital deficiency of other clotting factor OS	Feb-09
46026	D303.00	104	0	4	0	Congenital deficiency of other clotting factors	Feb-09
27426	PF6y100	116	0	27	0	Congenital deformity of ankle joint	Feb-09
28542	PF51.00	58	0	3	0	Congenital deformity of clavicle	Feb-09
40517	PF63X00	230	0	15	0	"Congenital deformity of hip, unspecified"	Feb-09
53416	PG1y.11	10	0	0	0	Congenital deformity of lumbosacral joint	Feb-09
51156	PG1y.12	8	0	0	0	Congenital deformity of lumbosacral region	Feb-09

For peer review only - http://bmjopen.bmj.com/site/about/guidelines.xhtml

35801	PGz..11	30	0	1	0	Congenital deformity of musculoskeletal system NEC	Feb-09
27676	PHz2.11	49	0	5	0	Congenital deformity of nail	Feb-09
64527	PF6y200	7	0	0	0	Congenital deformity of sacroiliac joint	Feb-09
49743	PF5w.11	43	0	3	0	Congenital deformity of scapula NEC	Feb-09
54223	PHz0.11	11	0	0	0	Congenital dermal defect	Feb-09
98061	P6y8.00	15	0	0	0	Congenital dextroposition of heart	Nov-09
49768	PG61.00	93	0	0	0	Congenital diaphragmatic hernia	Feb-09
11293	P72z111	55	0	1	0	Congenital dilatation of aorta	Feb-09
22560	PA40.00	281	0	17	0	Congenital dilatation of oesophagus	Feb-09
37835	P83y100	8	0	0	0	Congenital dilatation of trachea	Feb-09
26403	PD24.00	150	0	5	0	Congenital dilatation of ureter	Feb-09
64134	P248.00	50	0	1	0	Congenital dilated lateral ventricles of brain	Feb-09
22571	PD23.11	1047	0	4	0	Congenital dilated renal pelvis	Feb-09
65590	PB6y700	10	0	0	0	Congenital dilation of bile duct	Feb-09
25324	F230.00	543	0	16	0	Congenital diplegia	Feb-09
45551	F230z00	70	0	0	0	Congenital diplegia NOS	Feb-09
9239	PE3..00	5653	0	212	1	Congenital dislocation and subluxation of the hip	Feb-09
22301	PE8y200	500	0	25	0	Congenital dislocation of elbow	Feb-09
933	PE30.00	6905	0	689	1	Congenital dislocation of hip	Feb-09
27301	PE3z.00	528	0	15	0	Congenital dislocation of hip NOS	Feb-09
33576	PE30z00	158	0	1	0	Congenital dislocation of hip NOS	Feb-09
99683	P83y500	4	0	0	0	Congenital diverticulum of trachea	Jun-10
55900	PA43.00	9	0	0	0	Congenital duplication of oesophagus	Feb-09
56752	D21y012	10	0	0	0	Congenital dyserythropoiesis NEC	Feb-09
31040	D21y011	30	0	1	0	Congenital dyserythropoietic anaemia	Feb-09

For peer review only - http://bmjopen.bmj.com/site/about/guidelines.xhtml

53422	D21y000	9	0	0	0	Congenital dyshaematopoietic anaemia	Feb-09
66049	D401000	1	0	0	0	Congenital dysphagocytosis	Feb-09
29474	P337.00	20	0	0	0	Congenital ectopic lens	Feb-09
31998	P361300	14	0	2	0	Congenital ectropion	Feb-09
109860	PA2A000	1	0	0	0	Congenital ectropion of lip	Jul-15
51067	PH03.00	13	0	1	0	Congenital elephantiasis	Feb-09
72048	P20..14	2	0	0	0	Congenital endaural hernia	Feb-09
23752	P6yy900	3	0	0	0	Congenital epicardial cyst	Feb-09
65761	68A4.00	19	0	1	0	Congenital eye disorder screen	Feb-09
67169	P361z00	10	0	0	0	Congenital eyelid deformity NOS	Feb-09
70982	P4z..00	135	0	1	0	Congenital face or neck anomaly NOS	Feb-09
71051	PB5z000	4	0	0	0	Congenital faecal fistula	Feb-09
57813	Q466100	3	0	2	0	Congenital faecoliths causing obstruction	Feb-09
52576	Q402500	26	0	1	0	Congenital falciparum malaria	Feb-09
97903	PF37100	1	0	0	0	Congenital fibular deficiency type II	Oct-09
50307	PE8y600	31	0	2	0	Congenital flexion contracture of hip	Feb-09
29124	PE8y800	56	0	6	0	Congenital flexion contracture of knee	Feb-09
56631	PE8y300	15	0	1	0	Congenital flexion contractures of leg	Feb-09
24561	PG62.00	18	0	1	0	Congenital foramen Morgagni hernia	Feb-09
27309	PE80.11	100	0	8	0	Congenital funnel chest	Feb-09
45452	P602100	1	0	0	0	Congenital fusion of pulmonary valve segment	Feb-09
35806	PG33.00	57	0	1	0	Congenital fusion of ribs	Feb-09
67220	PF6y300	17	0	0	0	Congenital fusion of sacroiliac joint	Feb-09
68179	PH40200	0	0	1	0	Congenital generalised alopecia	Feb-09

For peer review only - http://bmjopen.bmj.com/site/about/guidelines.xhtml

44969	PC...00	38	0	1	0	Congenital genital organ anomalies	Feb-09
50367	PE40.00	23	0	1	0	Congenital genu recurvatum	Feb-09
23407	P320000	908	0	33	0	Congenital glaucoma	Feb-09
42855	P821.00	20	0	1	0	Congenital glottic web of larynx	Feb-09
247	P6z..00	2070	0	122	1	Congenital heart anomaly NOS	Feb-09
11982	P6zz.00	204	0	7	0	Congenital heart anomaly NOS	Feb-09
24533	P6y5.00	239	0	6	0	Congenital heart block	Feb-09
70992	P6y5z00	7	0	0	0	Congenital heart block NOS	Feb-09
68097	P6y5000	6	0	0	0	"Congenital heart block, unspecified"	Feb-09
72576	66g..00	391	0	36	0	Congenital heart condition monitoring	Feb-09
61935	L185.11	6	0	0	0	Congenital heart disease in pregnancy	Feb-09
23754	P5...12	332	0	5	0	"Congenital heart disease, septal and bulbar anomalies"	Feb-09
57298	D107000	3	0	11	11	Congenital Heinz-body anaemia	Feb-09
31853	PKyz600	471	0	26	0	Congenital hemihypertrophy	Feb-09
27966	F231.00	464	0	7	0	Congenital hemiplegia	Feb-09
34903	PB62.11	212	0	19	0	Congenital hepatic cyst	Feb-09
34642	Q48yz11	41	0	1	0	Congenital hepatic fibrosis	Feb-09
40762	Q409000	81	0	4	3	Congenital hepatitis A infection	Feb-09
50245	Q409100	121	0	4	1	Congenital hepatitis B infection	Feb-09
48738	PB6y100	10	0	0	0	Congenital hepatomegaly	Feb-09
101703	P11z.14	2	0	0	0	Congenital hernia of dura mater	Feb-11
43003	Q402000	86	0	2	0	Congenital herpes simplex	Feb-09
43215	PA6..00	234	0	1	0	Congenital hiatus hernia	Feb-09
2889	PF63100	2536	0	148	0	Congenital hip dysplasia	Feb-09
66951	P842.00	9	0	0	0	Congenital honeycomb lung	Feb-09
47776	PA71.00	11	0	1	0	Congenital hourglass stomach	Feb-09
62024	PF5y300	3	0	0	0	Congenital humeral varus	Feb-09
9611	P23..00	1376	0	78	0	Congenital hydrocephalus	Feb-09

For peer review only - http://bmjopen.bmj.com/site/about/guidelines.xhtml

107917	Q402311	2	0	0	0	Congenital hydrocephalus due to toxoplasmosis	Apr-14
28353	P23z.00	29	0	1	0	Congenital hydrocephalus NOS	Feb-09
5379	PD23.00	1375	0	24	0	Congenital hydronephrosis	Feb-09
26681	Q48y300	219	0	10	0	Congenital hypertonia	Feb-09
30154	PH42.00	55	0	11	0	Congenital hypertrichosis	Feb-09
101194	P355200	406	0	3	0	Congenital hypertrophy of retinal pigment epithelium	Dec-10
57161	C390500	27	0	1	0	Congenital hypogammaglobulinaemia	Feb-09
103611	P227100	2	0	0	0	Congenital hypoplasia of cerebrum	Jan-12
37320	D200000	69	0	9	0	Congenital hypoplastic anaemia	Feb-09
10097	C03..00	4305	0	109	28	Congenital hypothyroidism	Feb-09
51481	C03z.00	159	0	4	0	Congenital hypothyroidism NOS	Feb-09
31612	C03y000	39	0	0	0	Congenital hypothyroidism with diffuse goitre	Feb-09
93159	C03y100	2	0	0	0	Congenital hypothyroidism without goitre	Feb-09
8722	Q48y200	1837	0	35	0	Congenital hypotonia	Feb-09
57526	PH1z.11	7	0	0	0	Congenital ichthyosiform erythroderma	Feb-09
36468	PH10.00	133	0	2	0	"Congenital ichthyosis, unspecified"	Feb-09
102167	P6y5200	1	0	0	0	Congenital incomplete atrio-ventricular heart block	Apr-11
106080	Q40V.00	1	0	0	0	"Congenital infectious and parasitic disease, unspecified"	May-13
97269	PE31014	6	0	0	0	Congenital instability of hip joint	Aug-09
107150	P60z000	1	0	0	0	Congenital insufficiency of the pulmonary valve	Oct-13
67830	PB40.00	20	0	0	0	Congenital intestinal adhesions	Feb-09
69811	PB40z00	3	0	0	0	Congenital intestinal adhesions NOS	Feb-09
95632	P240.11	2	0	0	0	Congenital intracerebral cyst	Feb-09

For peer review only - http://bmjopen.bmj.com/site/about/guidelines.xhtml

96787	C0A..00	1	0	0	0	Congenital iodine deficiency syndrome	Apr-09
39166	C0A1.00	13	0	0	0	"Congenital iodine-deficiency syndrome, myxoedematous type"	Feb-09
72331	C0A0.00	1	0	0	0	"Congenital iodine-deficiency syndrome, neurological type"	Feb-09
23783	P340100	59	0	13	0	Congenital keratoconus	Feb-09
36991	PH3y300	216	0	9	0	Congenital keratoderma	Feb-09
49829	P322000	16	0	4	0	Congenital keratoglobus	Feb-09
50077	PF64z00	20	0	0	0	Congenital knee joint deformity NOS	Feb-09
32621	PH52.00	19	0	0	0	Congenital koilonychia	Feb-09
28717	PG18.11	368	0	26	0	Congenital kyphoscoliosis	Feb-09
30673	PC4yE00	526	0	35	0	Congenital labial adhesions	Feb-09
30468	P364.00	432	0	4	0	Congenital lacrimal passage anomalies	Feb-09
45351	P83y300	18	0	3	0	Congenital laryngocele	Feb-09
104555	P831700	58	0	1	0	Congenital laryngomalacia	Jul-12
49133	P6yy300	13	0	1	0	Congenital left ventricular diverticulum	Feb-09
41198	PE4..11	70	0	4	0	Congenital leg bone bowing	Feb-09
35351	P33..11	26	0	2	0	Congenital lens anomaly	Feb-09
95805	PH53.00	8	0	0	0	Congenital leukonychia	Feb-09
70280	Q402100	8	0	0	0	Congenital listeriosis	Feb-09
92345	PH40100	5	0	0	0	Congenital localised alopecia	Feb-09
29414	PG15.11	138	0	8	0	Congenital lumbosacral fusion	Feb-09
35729	PG11.00	97	0	2	0	Congenital lumbosacral spondylolysis	Feb-09
34693	B7J1.11	12	0	0	0	Congenital lymphangioma	Feb-09
27326	PH00.00	264	0	18	0	Congenital lymphoedema	Feb-09
94176	P322112	2	0	0	0	Congenital macrocornea	Feb-09
69316	Q402200	5	0	0	0	Congenital malaria	Feb-09

For peer review only - http://bmjopen.bmj.com/site/about/guidelines.xhtml

67260	PKy5z00	6	0	0	0	Congenital malform syndrome affecting facial appearance NOS	Feb-09
46825	P6W..00	33	0	3	0	Congenital malformation of aortic and mitral valves unsp	Feb-09
52658	PGX..00	33	0	3	0	"Congenital malformation of bony thorax, unspecified"	Feb-09
65092	PH6X.00	17	0	0	0	"Congenital malformation of breast, unspecified"	Feb-09
29738	P7W..00	39	0	1	0	"Congenital malformation of circulatory system, unspecif"	Feb-09
45910	P7X..00	63	0	0	0	"Congenital malformation of great arteries, unspecified"	Feb-09
47726	PB5X.00	12	0	0	0	"Congenital malformation of intestine, unspecified"	Feb-09
36474	P83yX00	22	0	2	0	"Congenital malformation of larynx, unspecified"	Feb-09
37965	PF5rD00	114	0	13	0	Congenital malformation of thumb	Feb-09
50529	P6X..00	19	0	0	0	"Congenital malformation of tricuspid valve, unspecified"	Feb-09
95151	PKy7z00	4	0	0	0	Congenital malformation syndrome involving limbs NOS	Feb-09
73575	PKy6z00	2	0	0	0	Congenital malformation syndrome with short stature NOS	Feb-09
100310	PKy8z00	2	0	0	0	Congenital malformation syndrome+other skeletal changes NOS	Jul-10
58981	PK8..00	13	0	1	0	Congenital malformation syndromes due to known exogen causes	Feb-09
45548	PKy7.00	5	0	0	0	Congenital malformation syndromes involving limbs	Feb-09

For peer review only - http://bmjopen.bmj.com/site/about/guidelines.xhtml

94910	PKy9.00	2	0	0	0	Congenital malformation syndromes with metabolic disturbance	Feb-09
97293	PKy8.00	4	0	0	0	Congenital malformation syndromes with other skeletal change	Aug-09
57953	PKy6.00	13	0	0	0	Congenital malformation syndromes with short stature	Feb-09
48205	P5X..00	33	0	0	0	Congenital malforms of cardiac chambers+connections unsp	Feb-09
39277	PBy2.00	11	0	2	1	Congenital malposition of digestive system NOS	Feb-09
38026	P322100	50	0	1	0	Congenital megalocornea	Feb-09
99424	P33y100	1	0	0	0	Congenital membranous cataract	May-10
41807	PE6z.11	37	0	2	0	Congenital metatarsus valgus	Feb-09
61651	P66..00	8	0	0	0	Congenital mitral insufficiency	Feb-09
57091	P65..00	34	0	2	0	Congenital mitral stenosis	Feb-09
100291	P65z.00	1	0	0	0	Congenital mitral stenosis NOS	Jul-10
46296	PH41.00	22	0	0	0	Congenital monilethrix	Feb-09
33925	F233.00	53	0	0	0	Congenital monoplegia	Feb-09
2709	PGz..00	778	0	46	1	Congenital musculoskeletal anomalies NOS	Feb-09
3298	PE...11	160	0	25	0	Congenital musculoskeletal deformities	Feb-09
33946	PEz..00	95	0	4	0	Congenital musculoskeletal deformity NOS	Feb-09
53113	D400500	14	0	0	0	Congenital neutropenia	Feb-09
40637	F486300	34	0	0	0	Congenital night blindness NOS	Feb-09
50096	F145.00	36	0	1	0	Congenital nonprogressive ataxia	Feb-09
49479	PB61200	13	0	2	0	Congenital obstruction of bile duct	Feb-09
65762	PB22.00	4	0	0	0	Congenital obstruction of large intestine	Feb-09

For peer review only - http://bmjopen.bmj.com/site/about/guidelines.xhtml

66309	PB12.00	20	0	0	0	Congenital obstruction of small intestine	Feb-09
34741	PD61.00	38	0	3	0	Congenital obstruction of urethra	Feb-09
100419	PD61z00	1	0	0	0	Congenital obstruction of urethra NOS	Aug-10
90483	PB23.00	6	0	0	0	Congenital occlusion of anus	Feb-09
62671	PB23z00	2	0	0	0	Congenital occlusion of anus NOS	Feb-09
101348	PB23000	1	0	1	0	Congenital occlusion of anus with fistula	Jan-11
31730	PA32.00	62	0	4	0	Congenital oesophageal fistula	Feb-09
95311	PA32z00	2	0	0	0	Congenital oesophageal fistula NOS	Feb-09
43558	PA45.00	158	0	20	0	Congenital oesophageal pouch	Feb-09
46814	PA3..11	6	0	0	0	Congenital oesophageal ring	Feb-09
49707	PA31.11	35	0	1	0	Congenital oesophageal stenosis	Feb-09
69684	PB40000	4	0	0	0	Congenital omental adhesions	Feb-09
66070	PH54.00	5	0	0	0	Congenital onychauxis	Feb-09
39392	P2xz100	139	0	4	0	Congenital optic atrophy	Feb-09
26024	P356000	95	0	2	0	Congenital optic disc coloboma	Feb-09
50282	P365.00	4	0	0	0	Congenital orbit anomalies	Feb-09
106909	PC0y.11	2	0	0	0	Congenital ovarian dysplasia	Aug-13
53673	PF6B200	1	0	0	0	Congenital overgrowth of foot	Feb-09
46652	PF6B.00	11	0	0	0	Congenital overgrowth of lower limb	Feb-09
35067	PH55.00	60	0	1	0	Congenital pachyonychia	Feb-09
37160	F230000	36	0	2	0	Congenital paraplegia	Feb-09
91259	PG15100	9	0	0	0	Congenital partial fusion of spine - balanced	Feb-09
53028	PG15200	3	0	0	0	Congenital partial fusion of spine - unbalanced	Feb-09
73595	P816.00	2	0	0	0	Congenital perforation of the nasal sinus wall	Feb-09
50362	P6yy400	14	0	0	0	Congenital pericardial defect	Feb-09

For peer review only - http://bmjopen.bmj.com/site/about/guidelines.xhtml

61062	P767.00	7	0	1	0	Congenital peripheral aneurysm	Feb-09
67420	PB40200	5	0	0	0	Congenital peritoneal adhesions	Feb-09
47616	PB40211	2	0	0	0	Congenital peritoneal bands	Feb-09
49742	PA27100	37	0	4	0	Congenital pharyngeal polyp	Feb-09
48046	PH32000	20	0	1	0	Congenital poikiloderma	Feb-09
18195	PB62000	212	0	17	0	Congenital polycystic liver disease	Feb-09
31820	P841.00	6	0	1	0	Congenital polycystic lung	Feb-09
23337	C371000	38	0	2	0	Congenital porphyria	Feb-09
11710	PD67.00	374	0	13	0	Congenital posterior urethral valves	Feb-09
69107	PD62.11	32	0	0	0	Congenital posterior urethral valves	Feb-09
38363	PE2z.11	16	0	1	0	Congenital postural curvature of spine NOS	Feb-09
100736	F591800	19	0	3	0	Congenital prelingual deafness	Sep-10
41025	PDy7.00	44	0	8	0	Congenital prolapse of bladder mucosa	Feb-09
47479	PDy8.00	29	0	2	0	Congenital prolapse of urethra	Feb-09
48936	P42z300	58	0	3	0	Congenital prominent auricle	Feb-09
48469	PF6y600	35	0	2	0	Congenital pseudarthrosis of tibia	Feb-09
44347	P8y4.00	16	0	0	0	Congenital pulmonary lymphangiectasis	Feb-09
22778	P602.00	832	0	10	0	Congenital pulmonary stenosis	Feb-09
33919	P602z00	152	0	0	0	Congenital pulmonary stenosis NOS	Feb-09
7225	D200312	18	0	0	0	Congenital pure red cell aplasia	Feb-09
38655	PB25.11	39	0	2	0	Congenital rectal stricture	Feb-09
61462	D200314	4	0	0	0	Congenital red cell hypoplasia	Feb-09
48260	PB52z12	26	0	1	0	Congenital redundant colon	Feb-09
68819	PB52z11	10	0	0	0	Congenital redundant rectal mucosa	Feb-09
27471	PD01.00	77	0	2	0	Congenital renal atrophy	Feb-09
17523	PD10.00	409	0	31	0	"Congenital renal cyst, single"	Feb-09
49791	P357000	39	0	1	0	Congenital retinal aneurysm	Feb-09
50584	P355100	14	0	0	0	Congenital retinal fold	Feb-09

For peer review only - http://bmjopen.bmj.com/site/about/guidelines.xhtml

48088	PG1x.00	44	0	1	0	Congenital sacrococcygeal anomalies NEC	Feb-09
70583	PG1xz00	12	0	0	0	Congenital sacrococcygeal anomaly NOS	Feb-09
49382	PA23.00	8	0	0	0	Congenital salivary gland fistula	Feb-09
60246	PH3y100	8	0	1	0	Congenital scar	Feb-09
10665	F591400	1042	0	25	0	Congenital sensorineural deafness	Feb-09
94734	PF34100	10	0	1	0	Congenital short femur	Feb-09
43330	D210000	5	0	0	0	Congenital sideroblastic anaemia	Feb-09
31007	PH...11	41	0	6	0	Congenital skin anomalies	Feb-09
61879	PE8y400	1	0	0	0	Congenital spade-like hand	Feb-09
15530	F23..11	644	0	34	0	Congenital spastic cerebral palsy	Feb-09
55593	F233.11	16	0	0	0	Congenital spastic foot	Feb-09
36974	P2y1.00	46	0	1	0	Congenital spinal cord anomaly	Feb-09
22012	PE2..00	660	0	11	0	Congenital spine deformity	Feb-09
29412	PE2z.00	96	0	4	0	Congenital spine deformity NOS	Feb-09
49933	PE20.00	29	0	2	0	"Congenital spine deformity, unspecified"	Feb-09
59741	PK03.00	36	0	0	0	Congenital splenomegaly	Feb-09
1871	PG12.00	1315	0	76	0	Congenital spondylolisthesis	Feb-09
100539	PB24011	2	0	0	0	Congenital stenosis of anus with fistula	Aug-10
49089	PB24111	6	0	0	0	Congenital stenosis of anus without mention of fistula	Feb-09
58189	P722411	3	0	0	0	Congenital stenosis of ascending aorta	Feb-09
71893	P833200	4	0	1	0	Congenital stenosis of bronchus	Feb-09
32484	PC4y800	82	0	3	0	Congenital stenosis of cervical canal	Feb-09
20686	PB13000	47	0	0	0	Congenital stenosis of duodenum	Feb-09
101739	P423100	1	0	0	0	Congenital stenosis of eustachian tube	Feb-11
69712	PB13200	9	0	0	0	Congenital stenosis of ileum	Feb-09

For peer review only

67021	PB13100	6	0	0	0	Congenital stenosis of jejunum	Feb-09
108445	PB22.11	1	0	0	0	Congenital stenosis of large intestine	Aug-14
61450	P833000	6	0	0	0	Congenital stenosis of larynx	Feb-09
59273	P833.00	5	0	0	0	"Congenital stenosis of larynx, trachea and bronchus"	Feb-09
101122	PB25111	1	0	0	0	Congenital stenosis of rectum without mention of fistula	Dec-10
59399	PB13.00	4	0	0	0	Congenital stenosis of small intestine	Feb-09
99366	PB13z00	1	0	0	0	Congenital stenosis of small intestine NOS	May-10
107172	P803.00	1	0	0	0	Congenital stenosis of the anterior nares	Oct-13
96389	P804.00	5	0	0	0	Congenital stenosis of the posterior nares	Apr-09
48818	P833100	11	0	0	0	Congenital stenosis of trachea	Feb-09
50820	PD22.11	34	0	0	0	Congenital stenosis of ureter	Feb-09
37200	PC4y900	28	0	7	0	Congenital stenosis of vagina	Feb-09
65337	PA73.00	9	0	0	0	Congenital stomach diverticulum	Feb-09
48020	PB24.00	15	0	0	0	Congenital stricture of anus	Feb-09
73497	PB24z00	1	0	0	0	Congenital stricture of anus NOS	Feb-09
41359	PB24000	3	0	0	0	Congenital stricture of anus with fistula	Feb-09
107117	PB24100	3	0	0	0	Congenital stricture of anus without mention of fistula	Oct-13
95810	PB61300	8	0	0	0	Congenital stricture of bile duct	Feb-09
32803	PB61311	31	0	0	0	Congenital stricture of common bile duct	Feb-09
103412	P6y4600	2	0	0	0	Congenital stricture of coronary artery	Dec-11

For peer review only - http://bmjopen.bmj.com/site/about/guidelines.xhtml

For peer review only

102497	P402111	1	0	0	0	Congenital stricture of external auditory canal	Jun-11
95103	P402112	1	0	0	0	Congenital stricture of osseous meatus	Feb-09
111389	P735.11	1	0	0	0	Congenital stricture of pulmonary artery	Oct-16
49501	PB25.00	29	0	1	0	Congenital stricture of rectum	Feb-09
42469	PB25z00	5	0	0	0	Congenital stricture of rectum NOS	Feb-09
69054	PB25000	1	0	0	0	Congenital stricture of rectum with fistula	Feb-09
47939	PB13z11	4	0	0	0	Congenital stricture of small intestine	Feb-09
33355	PD22.00	32	0	2	0	Congenital stricture of ureter	Feb-09
69191	PD22z00	10	0	2	0	Congenital stricture of ureter NOS	Feb-09
97464	PD22000	3	0	0	0	"Congenital stricture of ureter, unspecified"	Aug-09
27646	PD22100	17	0	0	0	Congenital stricture of ureteropelvic junction	Feb-09
96829	PD22200	2	0	0	0	Congenital stricture of ureterovesical orifice	May-09
62647	PC4y911	2	0	0	0	Congenital stricture of vagina	Feb-09
49142	P833300	20	0	0	0	Congenital subglottic stenosis	Feb-09
97956	P822.00	2	0	0	0	Congenital subglottic web of larynx	Nov-09
62586	PE31z00	76	0	0	0	Congenital subluxation of hip NOS	Feb-09
100627	F23y511	7	0	0	0	Congenital suprabulbar paresis	Sep-10
55144	P833400	12	0	1	0	Congenital supraglottic stenosis	Feb-09
99687	PF69000	2	0	0	0	Congenital synostosis of lower limb bones	Jun-10
27420	A90..00	272	0	8	0	Congenital syphilis	Feb-09
60039	A90z.00	6	0	0	0	Congenital syphilis NOS	Feb-09
50581	A900.12	4	0	0	0	Congenital syphilitic choroiditis	Feb-09
69219	A900.14	2	0	0	0	Congenital syphilitic epiphysitis	Feb-09

For peer review only - http://bmjopen.bmj.com/site/about/guidelines.xhtml

46401	A905000	10	0	0	0	Congenital syphilitic gumma	Feb-09
57279	A904200	1	0	0	0	Congenital syphilitic meningitis	Feb-09
22157	A900.16	34	0	6	1	Congenital syphilitic osteochondritis	Feb-09
7815	PE5y000	483	0	8	0	Congenital talipes calcaneovarus	Feb-09
38386	PE7y100	138	0	2	0	Congenital talipes calcaneus	Feb-09
31013	PE6y000	194	0	13	0	Congenital talipes equinovalgus	Feb-09
156	PE51.00	7134	0	439	0	Congenital talipes equinovarus	Feb-09
4334	PE7y200	411	0	25	0	Congenital talipes equinus	Feb-09
29808	PE60.00	589	0	41	0	Congenital talipes valgus	Feb-09
22846	PE50.00	1392	0	155	0	Congenital talipes varus	Feb-09
48940	P252.00	40	0	1	0	Congenital tethering of spinal cord	Feb-09
35125	PE8y111	14	0	0	0	Congenital thoracic wall deformity NEC	Feb-09
55582	D313100	7	0	0	0	Congenital thrombocytopenic purpura	Feb-09
93323	C03z.11	4	0	1	0	Congenital thyroid insufficiency	Feb-09
104835	PF36200	1	0	0	0	Congenital tibial deficiency type III	Aug-12
35042	PA32111	46	0	1	0	Congenital tracheo-oesophageal fistula	Feb-09
12752	P61..00	81	0	1	0	Congenital tricuspid atresia and stenosis	Feb-09
100065	P61z.00	2	0	0	0	Congenital tricuspid atresia or stenosis NOS	Jul-10
69169	P611.00	6	0	0	0	Congenital tricuspid stenosis	Feb-09
55957	Q402400	7	0	0	0	Congenital tuberculosis	Feb-09
110450	PG73.00	34	0	2	0	Congenital umbilical hernia	Jan-16
64939	PF6C100	2	0	0	0	Congenital undergrowth of distal part of limb	Feb-09
62450	PF6C.00	10	0	1	0	Congenital undergrowth of lower limb	Feb-09
54180	PF6C000	2	0	1	0	Congenital undergrowth of proximal part of limb	Feb-09

For peer review only - http://bmjopen.bmj.com/site/about/guidelines.xhtml

59736	PD21.11	13	0	1	0	Congenital ureteric valves	Feb-09
41342	PD62.00	43	0	0	0	Congenital urethral valvular stricture	Feb-09
29419	PDy6.00	96	0	19	0	Congenital urethrorectal fistula	Feb-09
67371	PD63.11	1	0	0	0	Congenital urinary meatus obstruction	Feb-09
31985	PD63.00	73	0	4	0	Congenital urinary meatus stricture	Feb-09
71832	PD63z00	7	0	0	0	Congenital urinary meatus stricture NOS	Feb-09
30143	PC4yC00	274	0	50	0	Congenital vaginal cyst NEC	Feb-09
36791	PF6y500	419	0	49	0	Congenital valgus ankle	Feb-09
35208	P76A.00	97	0	10	0	Congenital varix	Feb-09
36277	PF6y400	76	0	14	0	Congenital varus ankle	Feb-09
50065	PE61000	155	0	2	0	Congenital vertical talus	Feb-09
24918	PD47.00	408	0	8	0	Congenital vesico-uretero-renal reflux	Feb-09
104830	PD64.00	1	0	0	0	Congenital vesicourethral orifice stricture	Aug-12
93368	Q40X.00	4	0	0	0	"Congenital viral disease, unspecified"	Feb-09
67779	Q409.00	22	0	2	0	Congenital viral hepatitis	Feb-09
107622	Q409z00	3	0	0	0	Congenital viral hepatitis NOS	Jan-14
49448	PB5z.11	33	0	1	0	Congenital volvulus	Feb-09
31771	P82..00	87	0	1	0	Congenital web of larynx	Feb-09
70985	P82z.00	4	0	0	0	Congenital web of larynx NOS	Feb-09
111186	P820.00	1	0	0	0	"Congenital web of larynx, unspecified"	Aug-16
35616	P45..00	41	0	6	0	Congenital webbing of neck	Feb-09
99458	P45z.00	1	0	0	0	Congenital webbing of neck NOS	May-10
65125	PE1..11	5	0	1	0	Congenital wry neck	Feb-09
60880	C390512	18	0	1	0	Congenital X-linked agammaglobulinaemia	Feb-09

For peer review only - http://bmjopen.bmj.com/site/about/guidelines.xhtml

For peer review only

68766	G554011	10	0	0	0	Congestive obstructive cardiomyopathy	Feb-09
65402	C1z4100	8	0	1	0	Constitutional dwarfism	Feb-09
72252	D200300	4	0	1	2	Constitutional red cell aplasia and hypoplasia	Feb-09
47438	D200313	17	0	1	0	Constitutional red cell hypoplasia	Feb-09
38896	PF5E000	21	0	9	0	Constriction ring	Feb-09
93784	PF6D000	2	0	0	0	Constriction ring of lower limb	Feb-09
67293	PF6D.00	9	0	0	0	Constriction ring syndrome of lower limb	Feb-09
28775	PF6D300	1	0	0	0	Constriction ring syndrome of lower limb with amputation	Feb-09
24053	PF5E.00	32	0	3	0	Constriction ring syndrome of upper limb	Feb-09
104024	PF5E400	4	0	0	0	Constriction ring with acrosyndactyly and amputation	May-12
93038	PF5E100	2	0	2	0	Constriction ring with lymphoedema	Feb-09
65807	G532z00	15	0	0	0	Constrictive pericarditis NOS	Feb-09
25194	C294200	13	0	1	0	Copper deficiency	Feb-09
94908	4Q73.00	30	0	0	66	Copper level	Feb-09
3113	C154.00	712	0	30	1	Corticoadrenal insufficiency	Feb-09
12396	C154z00	99	0	0	0	Corticoadrenal insufficiency NOS	Feb-09
50508	PKy5B00	8	0	0	0	Costello syndrome	Feb-09
26444	J046100	273	0	7	0	Costen's syndrome	Feb-09
16897	PG03.00	1078	0	77	1	Craniosynostosis	Feb-09
27533	C03z.12	91	0	2	0	Cretinism	Feb-09
67513	C03..11	2	0	0	0	Cretinism	Feb-09
31795	PJ31.00	123	0	2	0	Cri-du-chat syndrome	Feb-09
46738	C374000	85	0	3	0	Crigler - Najjar syndrome	Feb-09
103821	13s7.00	3	0	0	0	Croatian as a second language	Feb-12
6538	J401z11	3927	0	94	0	Crohn's colitis	Feb-09

For peer review only - http://bmjopen.bmj.com/site/about/guidelines.xhtml

593	J40..11	100098	0	5433	53	Crohn's disease	Feb-09
59994	J40z.11	517	0	0	0	Crohn's disease NOS	Feb-09
39278	J400400	94	0	0	0	Crohn's disease of the ileum NOS	Feb-09
66238	J400300	48	0	0	0	Crohn's disease of the ileum unspecified	Feb-09
20688	J401z00	282	0	7	0	Crohn's disease of the large bowel NOS	Feb-09
9359	J400z00	209	0	0	0	Crohn's disease of the small bowel NOS	Feb-09
28476	J400200	582	0	3	0	Crohn's disease of the terminal ileum	Feb-09
25783	PG04.11	118	0	5	0	Crouzon's disease	Feb-09
101374	B7J1000	20	0	1	0	Cystic hygroma of neck	Jan-11
9240	PD11z11	495	0	48	0	Cystic kidney disease NEC	Feb-09
32371	C300200	9	0	0	0	Cystinaemia	Feb-09
60390	C300311	8	0	0	0	Cystine storage disease	Feb-09
27435	C300100	687	0	27	1	Cystinuria	Feb-09
3747	F593.00	1052	0	20	0	"Deaf mutism, NEC"	Feb-09
108703	Fy1..12	2	0	0	0	Deafblind	Oct-14
31374	P40z.11	259	0	5	0	Deafness due to congenital anomaly NEC	Feb-09
686	F59z.00	84378	0	26079	5	Deafness NOS	Feb-09
36588	D303100	91	0	1	0	Deficiency of factor II or prothrombin	Feb-09
15556	D303200	127	0	15	7	Deficiency of factor V or labile factor	Feb-09
45637	D303300	85	0	5	0	Deficiency of factor VII or stable factor	Feb-09
47198	D303400	32	0	1	1	Deficiency of factor X or Stuart-Prower factor	Feb-09
34673	D303500	172	0	13	5	Deficiency of factor XII or Hageman factor	Feb-09

For peer review only - http://bmjopen.bmj.com/site/about/guidelines.xhtml

20174	D303600	23	0	2	2	Deficiency of factor XIII or fibrin stabilizing factor	Feb-09
67298	PJ33000	5	0	0	0	Deletion of long arm of chromosome 13	Feb-09
95440	PJ33100	3	0	0	0	Deletion of long arm of chromosome 18	Feb-09
57806	PJ30.11	4	0	0	0	Deletion of long arm of chromosome 21	Feb-09
97927	PJ33200	4	0	0	0	Deletion of short arm of chromosome 18	Oct-09
66566	PJ32.00	7	0	0	0	Deletion of short arm of chromosome 4	Feb-09
98941	PJ31.11	6	0	0	0	Deletion of short arm of chromosome 5	Apr-10
32943	D104900	30	0	0	0	Delta-beta thalassaemia	Feb-09
106927	PKyP.00	2	0	0	0	"Diab insipidus,diab mell,optic atrophy and deafness"	Aug-13
61670	889A.00	10	0	0	0	Diab mellit insulin-glucose infus acute myocardial infarct	Feb-09
1045	C135.00	3172	0	198	2	Diabetes insipidus	Feb-09
60046	C135.12	12	0	0	0	Diabetes insipidus - pituitary	Feb-09
711	C10..00	813923	0	39051	4	Diabetes mellitus	Feb-09
43453	C10C.00	35	0	0	0	Diabetes mellitus autosomal dominant	Feb-09
36695	C10D.00	339	0	4	0	Diabetes mellitus autosomal dominant type 2	Feb-09
61122	C10H.00	38	0	0	0	Diabetes mellitus induced by non-steroid drugs	Feb-09
72345	C102z00	7	0	0	0	Diabetes mellitus NOS with hyperosmolar coma	Feb-09
42505	C101z00	376	0	8	0	Diabetes mellitus NOS with ketoacidosis	Feb-09

For peer review only

65062	C103z00	13	0	0	0	Diabetes mellitus NOS with ketoacidotic coma	Feb-09
22573	C106z00	257	0	1	0	Diabetes mellitus NOS with neurological manifestation	Feb-09
50972	C100z00	586	0	7	0	Diabetes mellitus NOS with no mention of complication	Feb-09
34283	C105z00	165	0	0	0	Diabetes mellitus NOS with ophthalmic manifestation	Feb-09
70821	C10yz00	15	0	0	0	Diabetes mellitus NOS with other specified manifestation	Feb-09
65025	C107z00	77	0	3	0	Diabetes mellitus NOS with peripheral circulatory disorder	Feb-09
64357	C10zz00	113	0	0	0	Diabetes mellitus NOS with unspecified complication	Feb-09
32403	C107.11	275	0	50	0	Diabetes mellitus with gangrene	Feb-09
21482	C102.00	220	0	34	0	Diabetes mellitus with hyperosmolar coma	Feb-09
1682	C101.00	13381	0	2152	8	Diabetes mellitus with ketoacidosis	Feb-09
15690	C103.00	353	0	58	0	Diabetes mellitus with ketoacidotic coma	Feb-09
35107	C104z00	306	0	10	0	Diabetes mellitus with nephropathy NOS	Feb-09
7795	C106.12	9335	0	219	0	Diabetes mellitus with neuropathy	Feb-09
38986	C100.00	2877	0	34	0	Diabetes mellitus with no mention of complication	Feb-09
33254	C105.00	1791	0	24	0	Diabetes mellitus with ophthalmic manifestation	Feb-09
33343	C10y.00	72	0	1	0	Diabetes mellitus with other specified manifestation	Feb-09
35399	C107.00	3910	0	28	0	Diabetes mellitus with peripheral circulatory disorder	Feb-09

For peer review only - http://bmjopen.bmj.com/site/about/guidelines.xhtml

16491	C106.13	67	0	0	0	Diabetes mellitus with polyneuropathy	Feb-09
16502	C104.00	1441	0	101	0	Diabetes mellitus with renal manifestation	Feb-09
45491	C10z.00	83	0	3	0	Diabetes mellitus with unspecified complication	Feb-09
70448	C107000	2	0	0	0	"Diabetes mellitus, juvenile +peripheral circulatory disorder"	Feb-09
69748	C105000	6	0	0	0	"Diabetes mellitus, juvenile type, + ophthalmic manifestation"	Feb-09
68792	C10z000	7	0	0	0	"Diabetes mellitus, juvenile type, + unspecified complication"	Feb-09
24490	C100000	1681	0	50	0	"Diabetes mellitus, juvenile type, no mention of complication"	Feb-09
40023	C102000	8	0	0	0	"Diabetes mellitus, juvenile type, with hyperosmolar coma"	Feb-09
53200	C101000	108	0	4	0	"Diabetes mellitus, juvenile type, with ketoacidosis"	Feb-09
42567	C103000	6	0	0	0	"Diabetes mellitus, juvenile type, with ketoacidotic coma"	Feb-09
93922	C104000	5	0	0	0	"Diabetes mellitus, juvenile type, with renal manifestation"	Feb-09
67853	C106000	76	0	0	0	"Diabetes mellitus, juvenile, + neurological manifestation"	Feb-09
110997	C10y000	1	0	0	0	"Diabetes mellitus, juvenile, + other specified manifestation"	Jul-16
13071	66AI.00	59237	0	557	73	Diabetic - good control	Feb-09
2378	66AJ.00	135053	0	8917	27	Diabetic - poor control	Feb-09
22023	66AJz00	1614	0	42	0	Diabetic - poor control NOS	Feb-09
8842	66A5.00	73497	0	162	0	Diabetic on insulin	Feb-09
111483	66o6.00	1	0	0	0	Diabetic on insulin and glucagon-like peptide 1	Nov-16

For peer review only - http://bmjopen.bmj.com/site/about/guidelines.xhtml

28769	66AV.00	15408	0	21	0	Diabetic on insulin and oral treatment	Feb-09
110344	66o2.00	41	0	0	0	Diabetic on non-insulin injectable medication	Dec-15
1684	66A4.00	230233	0	445	3	Diabetic on oral treatment	Feb-09
110379	66o5.00	47	0	0	0	Diabetic on oral treatment and glucagon-like peptide 1	Dec-15
101728	66As.00	638	0	0	0	Diabetic on subcutaneous treatment	Feb-11
4987	J34..00	25672	0	508	0	Diaphragmatic hernia	Feb-09
60697	J342.00	30	0	2	2	Diaphragmatic hernia - irreducible	Feb-09
11780	J34z.00	3413	0	10	0	Diaphragmatic hernia NOS	Feb-09
24703	J340.00	12	0	0	0	Diaphragmatic hernia with gangrene	Feb-09
44233	J341.00	16	0	0	0	Diaphragmatic hernia with obstruction	Feb-09
298	13VC.00	16755	0	383	0	Disability	Feb-09
23369	13VC100	439	0	5	0	Disability - moderate	Feb-09
3972	13VC200	248	0	8	0	Disability - severe	Feb-09
27462	13VC000	363	0	2	0	Disability - slight	Feb-09
41742	13VCZ00	531	0	7	0	Disability NOS	Feb-09
3058	13VC.11	4335	0	104	0	Disabled	Feb-09
42815	C313700	681	0	0	0	Disaccharidase deficiency	Feb-09
2667	M154100	6944	0	421	0	Discoid lupus erythematosus	Feb-09
52080	C375X00	5	0	1	0	"Disorder of glucosaminoglycan metabolism, unspecified"	Feb-09
65382	C31yX00	8	0	0	1	"Disorder of glycoprotein metabolism, unspecified"	Feb-09
66726	F391500	8	0	1	0	Distal (Gower's) muscular dystrophy	Feb-09
46117	P58..00	31	0	0	0	Double outlet left ventricle	Feb-09
1778	P511.00	37	0	0	0	Double outlet right ventricle	Feb-09
65318	P511z00	4	0	0	0	Double outlet right ventricle NOS	Feb-09

For peer review only - http://bmjopen.bmj.com/site/about/guidelines.xhtml

1543	PJ0..00	10552	0	1045	780	Down's syndrome - trisomy 21	Feb-09
10759	PJ0z.00	1447	0	13	0	Down's syndrome NOS	Feb-09
15661	G310.11	529	0	16	0	Dressler's syndrome	Feb-09
7766	F4J7000	2490	0	74	0	Duane's syndrome	Feb-09
39293	C374100	106	0	1	0	Dubin - Johnson syndrome	Feb-09
57043	PKy6600	23	0	0	0	Dubowitz syndrome	Feb-09
7470	F152111	50	0	5	0	Duchenne Aran muscular atrophy	Feb-09
108940	PD80.00	243	0	2	0	Duplex kidney	Dec-14
4431	PD34.11	6973	0	218	2	Duplex kidneys	Feb-09
5006	PG41.11	179	0	6	0	Dwarfism	Feb-09
34706	C1z4.00	30	0	0	0	Dwarfism NEC	Feb-09
66781	C1z4z00	1	0	0	0	Dwarfism NEC NOS	Feb-09
4451	PGy2.00	2654	0	259	0	Ehlers-Danlos syndrome	Feb-09
63549	PGy2000	12	0	0	0	Ehlers-Danlos syndrome type I	Feb-09
72787	PGy2100	17	0	0	0	Ehlers-Danlos syndrome type II	Feb-09
28335	PGy2200	143	0	3	0	Ehlers-Danlos syndrome type III	Feb-09
70415	PGy2300	13	0	0	0	Ehlers-Danlos syndrome type IV	Feb-09
53984	PGy2500	3	0	0	0	Ehlers-Danlos syndrome type VI	Feb-09
95643	PGy2600	1	0	0	0	Ehlers-Danlos syndrome type VII	Feb-09
98263	PGy2700	1	0	0	0	Ehlers-Danlos syndrome type VIII	Dec-09
68007	PG55.11	10	0	0	0	Ellis - Van Creveld syndrome	Feb-09
34985	F391A00	32	0	5	0	Emery-Dreifuss muscular dystrophy	Feb-09
37798	PH3y700	257	0	11	0	Epidermolysis bullosa simplex	Feb-09
573	F25..00	391517	0	20275	98	Epilepsy	Feb-09
50012	6674	226	0	6	0	Epilepsy associated problems	Feb-09
22341	1O30.00	1754	0	42	0	Epilepsy confirmed	Feb-09
9747	F25z.00	11242	0	290	1	Epilepsy NOS	Feb-09
1715	F250011	3524	0	1055	1	Epileptic absences	Feb-09
31830	F250300	199	0	17	0	Epileptic seizures - akinetic	Feb-09
24309	F250200	504	0	74	0	Epileptic seizures - atonic	Feb-09
18471	F251200	3391	0	199	0	Epileptic seizures - clonic	Feb-09
4801	F251300	1430	0	80	0	Epileptic seizures - myoclonic	Feb-09

For peer review only - http://bmjopen.bmj.com/site/about/guidelines.xhtml

5152	F251400	3665	0	259	0	Epileptic seizures - tonic	Feb-09
48036	F391100	77	0	2	0	Erb's muscular dystrophy	Feb-09
799	G20..00	2780814	0	16008	35	Essential hypertension	Feb-09
24161	C327411	219	0	15	1	Fabry's disease	Feb-09
35717	C327.12	45	0	3	1	Fabry's disease	Feb-09
24384	K032400	87	0	13	0	Familial glomerulonephritis in Alport's syndrome	Feb-09
3386	C320000	10075	0	265	0	Familial hypercholesterolaemia	Feb-09
3484	C320.11	890	0	3	0	Familial hypercholesterolaemia	Feb-09
23515	C300300	72	0	3	0	Fanconi-de-Toni syndrome	Feb-09
64539	PC72.00	6	0	0	0	Female pseudohermaphroditism	Feb-09
57081	PK84.00	69	0	2	0	Fetal valproate syndrome	Feb-09
39085	F220.00	89	0	12	0	Flaccid hemiplegia	Feb-09
104828	F23y100	1	0	0	0	Flaccid infantile cerebral palsy	Aug-12
46175	F241000	59	0	1	0	Flaccid paraplegia	Feb-09
5525	F255011	3632	0	208	1	Focal epilepsy	Feb-09
107814	K032200	1	0	0	0	Focal glomerulon + focal recurr macroscop glomerulonephritis	Mar-14
105180	F1y0.00	5	0	0	0	Fragile X associated tremor ataxia syndrome	Nov-12
67264	P205.00	8	0	0	0	Frontal encephalocele	Feb-09
65371	C311100	13	0	0	0	Galactokinase deficiency	Feb-09
5561	C311.00	317	0	10	5	Galactosaemia	Feb-09
64321	C311z00	9	0	0	0	Galactosaemia NOS	Feb-09
20730	C311000	9	0	0	0	Galactose-1-phosphate uridyl transferase deficiency	Feb-09
15593	PG71.00	1268	0	17	0	Gastroschisis	Feb-09
4659	E200200	45728	0	1707	0	Generalised anxiety disorder	Feb-09
26144	F251.00	1454	0	13	0	Generalised convulsive epilepsy	Feb-09
40806	F251z00	290	0	1	0	Generalised convulsive epilepsy NOS	Feb-09

For peer review only - http://bmjopen.bmj.com/site/about/guidelines.xhtml

44252	F250z00	82	0	1	0	Generalised nonconvulsive epilepsy NOS	Feb-09
106571	F25H.00	413	0	4	0	Generalised seizure	Aug-13
21934	C310212	179	0	4	0	Glucose-6-phosphatase deficiency	Feb-09
22531	D102000	524	0	265	245	Glucose-6-phosphate dehydrogenase deficiency anaemia	Feb-09
101224	F28y400	11	0	0	0	GLUT1 deficiency syndrome	Dec-10
22111	C309.00	9	0	0	0	Glutaryl CoA dehydrogenase deficiency	Feb-09
24186	C310.13	174	0	9	0	Glycogen storage disease	Feb-09
31383	C310.00	104	0	10	2	Glycogenosis - glycogen storage disease	Feb-09
988	F251000	18606	0	1210	2	Grand mal (major) epilepsy	Feb-09
5668	F251600	19046	0	771	2	Grand mal seizure	Feb-09
5117	F253.00	981	0	31	0	Grand mal status	Feb-09
5257	C020.12	19532	0	614	2	Graves' disease	Feb-09
2983	C350000	10217	0	1033	7	Haemochromatosis	Feb-09
31662	D107600	2	0	0	0	Haemoglobin Zurich disease	Feb-09
31800	D107300	132	0	6	5	Haemoglobin-C disease	Feb-09
7624	D107400	104	0	1	1	Haemoglobin-D disease	Feb-09
32373	D107500	155	0	2	0	Haemoglobin-E disease	Feb-09
7526	D107700	78	0	3	7	Haemoglobin-H disease	Feb-09
71808	D102.00	1	0	0	0	Haemolytic anaemia due to glutathione metabolism disorder	Feb-09
107820	D103000	2	0	0	0	Haemolytic anaemia due to hexokinase deficiency	Mar-14
55561	D103100	37	0	0	0	Haemolytic anaemia due to pyruvate kinase deficiency	Feb-09
3326	D1...00	3122	0	188	2	Haemolytic anaemias	Feb-09
18631	D1z..00	297	0	1	0	Haemolytic anaemias NOS	Feb-09
29323	D111300	1111	0	21	0	Haemolytic-uraemic syndrome	Feb-09

For peer review only - http://bmjopen.bmj.com/site/about/guidelines.xhtml

51041	C37y800	48	0	2	0	Haemophagocytic lymphohistiocytosis	Feb-09
27934	C37y900	17	0	0	0	"Haemophagocytic syndrome, infection-associated"	Feb-09
2833	D300.12	1334	0	55	0	Haemophilia A	Feb-09
31166	D301.12	285	0	4	0	Haemophilia B	Feb-09
56664	D302.11	58	0	1	0	Haemophilia C	Feb-09
5632	D300.00	1516	0	85	2	Haemophilia-A (factor VIII deficiency)	Feb-09
22706	D302.00	392	0	13	1	Haemophilia-C (factor XI deficiency)	Feb-09
69377	PG0E.11	3	0	0	0	Hallerman - Streif syndrome	Feb-09
49217	C300400	12	0	0	0	Hartnup disease	Feb-09
4549	G56..12	16631	0	1578	1	Heart block	Feb-09
95785	P6yyA00	1	0	0	0	Hemicardia	Feb-09
110165	P002.00	2	0	0	0	Hemicephaly	Oct-15
91177	7J02C00	22	0	0	0	Hemicraniotomy	Feb-09
108683	M21yD00	5	0	0	0	Hemifacial atrophy	Oct-14
9371	PE00000	160	0	4	0	Hemifacial microsomia	Feb-09
95697	P24A.00	10	0	0	0	Hemimegalencephaly	Feb-09
807	F22..11	15097	0	1791	0	Hemiparesis	Feb-09
65275	F2Az.00	136	0	1	0	Hemiparesis NOS	Feb-09
1749	F22..00	17585	0	2027	0	Hemiplegia	Feb-09
8492	F22z.00	2414	0	185	0	Hemiplegia NOS	Feb-09
3658	F26y000	6343	0	358	0	Hemiplegic migraine	Feb-09
97623	N093.11	1	0	0	0	Hench - Rosenberg syndrome	Sep-09
25383	J61y400	454	0	4	0	Hepatic fibrosis	Feb-09
71453	J615z15	24	0	0	0	Hepatic fibrosis	Feb-09
100592	J61y600	3	0	0	0	Hepatic fibrosis with hepatic sclerosis	Aug-10
2413	A70z000	7597	0	1138	458	Hepatitis C	Feb-09
10636	J624.00	407	0	14	0	Hepatorenal syndrome	Feb-09

For peer review only - http://bmjopen.bmj.com/site/about/guidelines.xhtml

34092	F1...00	129	0	7	0	Hereditary and degenerative diseases of the CNS	Feb-09
56006	F1z..00	32	0	0	0	Hereditary and degenerative diseases of the CNS NOS	Feb-09
61968	F1y..00	24	0	1	0	Hereditary and degenerative diseases of the CNS OS	Feb-09
101075	SN51100	31	0	6	0	Hereditary C1 esterase inhibitor deficiency	Dec-10
64654	F435.00	7	0	0	0	Hereditary choroid dystrophies	Feb-09
31384	C312.00	58	0	209	208	Hereditary fructose intolerance	Feb-09
14698	D10z.00	560	0	56	2	Hereditary haemolytic anaemia NOS	Feb-09
39456	D10..00	83	0	1	0	Hereditary haemolytic anaemias	Feb-09
4942	G770.00	1536	0	154	0	Hereditary haemorrhagic telangiectasia	Feb-09
46726	D311000	19	0	0	0	Hereditary haemorrhagic thrombasthenia	Feb-09
32527	F368.00	431	0	12	0	Hereditary motor and sensory neuropathy	Feb-09
56910	F368000	54	0	0	0	Hereditary motor and sensory neuropathy type I	Feb-09
35465	F368100	259	0	0	0	Hereditary motor and sensory neuropathy type II	Feb-09
106103	F368200	2	0	0	0	Hereditary motor and sensory neuropathy type III	May-13
51113	K0A5000	4	0	0	0	"Hereditary nephropathy NEC, minor glomerular abnormality"	Feb-09
41239	K0A5100	2	0	0	0	"Hereditary nephropathy NEC,focal+segmnt glomerular lesion"	Feb-09
36205	K0A5.00	44	0	2	0	Hereditary nephropathy not elsewhere classified	Feb-09

For peer review only - http://bmjopen.bmj.com/site/about/guidelines.xhtml

91738	K0A5600	1	0	0	0	"Hereditary nephropathy, NEC, dense deposit disease"	Feb-09
62980	K0A5X00	4	0	0	0	"Hereditary nephropathy, unspecif morphological changes"	Feb-09
68118	F391.00	11	0	0	0	Hereditary progressive muscular dystrophy	Feb-09
22727	F427.00	143	0	6	0	Hereditary retinal dystrophies	Feb-09
97370	F427200	2	0	0	0	Hereditary retinal dystrophies with other diseases	Aug-09
50363	F427z00	531	0	139	0	Hereditary retinal dystrophy NOS	Feb-09
3514	F141.00	748	0	62	0	Hereditary spastic paraplegia	Feb-09
7237	D100.00	3206	0	135	9	Hereditary spherocytosis	Feb-09
58906	D313111	13	0	0	0	Hereditary thrombocytopenia NEC	Feb-09
44270	K0A5200	12	0	0	0	"Hereditry nephropathy NEC,difus membran glomerulnephritis"	Feb-09
61861	C302900	20	0	1	0	Hermansky-Pudlak syndrome	Feb-09
8546	PB30.00	2034	0	82	0	Hirschsprung's disease	Feb-09
56630	PB3..00	17	0	0	0	Hirschsprung's disease and allied congenital conditions	Feb-09
59085	PB3z.00	4	0	0	0	Hirschsprung's disease and allied congenital conditions NOS	Feb-09
70582	PB30z00	65	0	1	0	Hirschsprung's disease NOS	Feb-09
43907	C305000	23	0	0	0	Histidinaemia	Feb-09
55198	C305200	5	0	0	0	Histidinuria	Feb-09
4870	B625.11	240	0	21	1	"Histiocytosis X (acute, progressive)"	Feb-09
51718	C37y500	25	0	0	0	"Histiocytosis X , chronic"	Feb-09
36736	C37y600	105	0	1	0	"Histiocytosis X , unspecified"	Feb-09
37126	C37y700	133	0	2	0	"Histiocytosis, unspecified"	Feb-09
21966	PKy7100	91	0	1	0	Holt - Oram syndrome	Feb-09
33575	C304300	204	0	4	0	Homocystinuria	Feb-09
37808	D104311	46	0	1	0	Homozygous alpha thalassaemia	Feb-09

For peer review only - http://bmjopen.bmj.com/site/about/guidelines.xhtml

For peer review only

44146	D303800	121	0	1	0	Homozygous factor V Leiden mutation	Feb-09
49820	42e0.00	30	0	2	66	Homozygous SS genotype	Feb-09
16167	F17z.11	841	0	97	0	Horner's syndrome	Feb-09
3584	F115.00	7715	0	298	0	Hydrocephalus	Feb-09
97663	P233.12	2	0	0	0	Hydrocephalus with atresia of foramina of Magendie+Luschka	Sep-09
40588	P230.11	6	0	0	0	Hydrocephauls with anomaly of aqueduct of Sylvius	Feb-09
21483	SM31000	16	0	1	0	Hydrochloric acid causing toxic effect	Feb-09
3277	K11..00	33858	0	2602	7	Hydronephrosis	Feb-09
27302	K11z.00	1146	0	20	0	Hydronephrosis NOS	Feb-09
8522	K113.11	2186	0	125	0	Hydronephrosis with pelviureteric junction obstruction	Feb-09
27592	K112.00	225	0	21	0	Hydronephrosis with renal and ureteral calculous obstruction	Feb-09
28159	K11X.00	108	0	3	0	Hydronephrosis with ureteral stricture NEC	Feb-09
10410	K113.00	477	0	12	0	Hydronephrosis with ureteropelvic junction obstruction	Feb-09
31841	K135.00	682	0	13	0	Hydroureter	Feb-09
46116	PD25.00	36	0	0	0	Hydroureter - congenital	Feb-09
17778	K111.00	497	0	7	0	Hydroureteronephrosis	Feb-09
32275	C151.00	437	0	13	0	Hyperaldosteronism	Feb-09
68567	C151z00	31	0	0	0	Hyperaldosteronism NOS	Feb-09
107252	C329.00	7575	0	78	2	Hypercholesterolaemia	Nov-13
34150	D41y000	283	0	27	2	Hypergammaglobulinaemia	Feb-09
10166	C307000	2032	0	66	8	Hyperglycinaemia	Feb-09
31274	C163200	53	0	3	0	Hypergonadotrophic ovarian failure	Feb-09
104314	C304500	15	0	0	0	Hyperhomocysteinaemia	Jun-12
54203	C397.00	17	0	0	0	Hyperimmunoglobulin E syndrome	Feb-09

For peer review only - http://bmjopen.bmj.com/site/about/guidelines.xhtml

111300	K08yB00	2	0	0	0	Hyperkalaemic renal tubular acidosis	Sep-16
58069	E2E1.00	17	0	1	0	Hyperkinesis with developmental delay	Feb-09
45263	E2E2.00	175	0	6	0	Hyperkinetic conduct disorder	Feb-09
41769	E2Ez.00	41	0	4	0	Hyperkinetic syndrome NOS	Feb-09
637	C324.00	338588	0	7473	464	Hyperlipidaemia NOS	Feb-09
48144	C307400	22	0	0	0	Hyperlysinaemia	Feb-09
26371	C352000	27	0	0	0	Hypermagnesaemia	Feb-09
73934	C120100	8	0	0	0	Hyperparathyroid bone disease	Feb-09
3559	C120.00	16259	0	1792	3	Hyperparathyroidism	Feb-09
9423	C353400	706	0	12	0	Hyperphosphataemia	Feb-09
6732	C131000	10204	0	1238	0	Hyperprolactinaemia	Feb-09
30533	C30y400	82	0	12	0	Hyperprolinaemia	Feb-09
10702	D414.00	449	0	25	0	Hypersplenism	Feb-09
57987	G234.00	8	0	1	0	Hyperten heart&renal dis+both(congestv)heart and renal fail	Feb-09
103690	38G3.00	6798	0	0	0	"Hyperten, abnorm renal/liver funct, stroke, BLED score"	Feb-12
7057	G2z..00	41526	0	209	0	Hypertensive disease NOS	Feb-09
63466	G23..00	39	0	0	0	Hypertensive heart and renal disease	Feb-09
68659	G23z.00	6	0	0	0	Hypertensive heart and renal disease NOS	Feb-09
28684	G233.00	46	0	2	0	Hypertensive heart and renal disease with renal failure	Feb-09
31464	G21z.00	119	0	2	0	Hypertensive heart disease NOS	Feb-09
62718	G21z100	70	0	0	0	Hypertensive heart disease NOS with CCF	Feb-09
61166	G21z000	67	0	1	0	Hypertensive heart disease NOS without CCF	Feb-09

For peer review only - http://bmjopen.bmj.com/site/about/guidelines.xhtml

21837	G232.00	29	0	4	0	Hypertensive heart&renal dis wth (congestive) heart failure	Feb-09
4668	G22..00	1876	0	61	0	Hypertensive renal disease	Feb-09
32423	G222.00	82	0	0	0	Hypertensive renal disease with renal failure	Feb-09
1472	C02..11	66918	0	9227	174	Hyperthyroidism	Feb-09
26362	212P.00	331	0	4	0	Hyperthyroidism resolved	Feb-09
53977	C302600	11	0	0	0	Hypertyrosinaemia	Feb-09
91091	7Q0B100	42	0	0	0	Hyperuricaemia drugs Band 1	Feb-09
60584	C303400	6	0	0	0	Hypervalinaemia	Feb-09
64905	C382.00	5	0	0	0	Hypervitaminosis A	Feb-09
32143	C384.00	36	0	2	0	Hypervitaminosis D	Feb-09
21594	C154300	113	0	1	0	Hypoaldosteronism	Feb-09
95041	PG4B100	2	0	0	0	Hypochondrogenesis	Feb-09
29840	PG42.15	137	0	4	0	Hypochondroplasia	Feb-09
94173	PG41000	13	0	0	0	Hypochondroplasia	Feb-09
50305	K032y11	11	0	0	0	Hypocomplementaemic persistent glomerulonephritis NEC	Feb-09
15137	C390000	1831	0	64	0	Hypogammaglobulinaemia NOS	Feb-09
98210	C139.00	1581	0	70	0	Hypogonadotropic hypogonadism	Dec-09
36298	M250.11	37	0	3	0	Hypohidrosis	Feb-09
15096	C121.00	2168	0	136	2	Hypoparathyroidism	Feb-09
63512	C121z00	49	0	0	0	Hypoparathyroidism NOS	Feb-09
7902	C353300	613	0	21	0	Hypophosphataemia	Feb-09
21870	C353211	217	0	5	0	Hypophosphataemic rickets	Feb-09
33347	C353000	366	0	19	1	Hypophosphatasia	Feb-09
22028	C353100	88	0	6	0	Hypophosphatasia rickets	Feb-09
105861	C133.12	1	0	0	0	Hypophyseal dwarfism	Mar-13
8552	C132.11	3039	0	145	4	Hypopituitarism NOS	Feb-09
29818	PK13.00	92	0	6	0	Hypoplasia of adrenal gland	Feb-09
37515	P722200	42	0	1	0	Hypoplasia of aorta	Feb-09

For peer review only - http://bmjopen.bmj.com/site/about/guidelines.xhtml

For peer review only

57932	P710.00	29	0	1	0	"Hypoplasia of aortic arch, unspecified"	Feb-09
63668	P222.00	12	0	0	0	"Hypoplasia of brain, part unspecified"	Feb-09
68063	P6yy100	3	0	0	0	Hypoplasia of cardiac vein	Feb-09
44717	P22A100	29	0	0	0	Hypoplasia of cerebellum	Feb-09
37600	P228100	13	0	0	0	Hypoplasia of corpus callosum	Feb-09
51993	PF4z.13	42	0	2	0	Hypoplasia of limb NOS	Feb-09
24646	PF3z.11	107	0	1	0	Hypoplasia of lower limb	Feb-09
23874	P851.00	156	0	1	0	Hypoplasia of lung	Feb-09
61593	PB72.00	3	0	0	0	Hypoplasia of pancreas	Feb-09
60216	P22z.13	11	0	0	0	Hypoplasia of part of brain NEC	Feb-09
23948	PCy2000	58	0	6	0	Hypoplasia of penis	Feb-09
54488	P601000	17	0	0	0	Hypoplasia of pulmonary valve	Feb-09
68493	PG1y400	10	0	0	0	Hypoplasia of spine	Feb-09
71462	PK06.00	5	0	0	0	Hypoplasia of spleen	Feb-09
54487	P734.00	34	0	0	0	Hypoplasia of the pulmonary artery	Feb-09
22457	PF2z.11	62	0	3	0	Hypoplasia of upper limb	Feb-09
44913	D200.15	46	0	3	0	Hypoplastic anaemia - familial	Feb-09
32715	D201111	11	0	0	0	Hypoplastic anaemia due to drug or chemical substance	Feb-09
65351	D201211	11	0	0	0	Hypoplastic anaemia due to infection	Feb-09
57114	D201412	2	0	1	0	Hypoplastic anaemia due to toxic cause	Feb-09
44767	P6yy.11	27	0	0	0	Hypoplastic aortic orifice or valve	Feb-09
92968	7Q09100	3	0	0	0	Hypoplastic haemolytic and renal anaemia drugs Band 2	Feb-09
16004	C134011	112	0	13	0	Hypoprolactinaemia	Feb-09
5073	C33y000	1163	0	98	4	Hypoproteinaemia	Feb-09
51291	D303111	10	0	2	0	Hypoprothrombinaemia	Feb-09
28386	F4A0400	43	0	2	0	Hypopyon ulcer	Feb-09

For peer review only - http://bmjopen.bmj.com/site/about/guidelines.xhtml

108019	C154700	9	0	0	0	Hyporeninaemic hypoaldosteronism	May-14
36881	C13X.00	53	0	1	0	"Hypothalamic dysfunction, not elsewhere classified"	Feb-09
22732	C13..11	144	0	7	0	Hypothalamus disorders	Feb-09
64145	5761.11	2	0	1	3	Hypothalamus hormone radioass.	Feb-09
18282	C04z.13	447	0	54	0	"Hypothyroid goitre, acquired"	Feb-09
273	C04..13	428907	0	8672	1713	Hypothyroidism	Feb-09
3941	C04z.00	39418	0	249	0	Hypothyroidism NOS	Feb-09
68482	C372000	1	0	2	0	Hypoxanthine-guanine-phosphoribosyltransferase deficiency	Feb-09
20275	C150100	72	0	5	0	Iatrogenic Cushing's syndrome	Feb-09
61520	C110000	17	0	0	0	Iatrogenic hyperinsulinism	Feb-09
50958	C137.11	51	0	3	0	Iatrogenic hypopituitarism	Feb-09
38976	C043z00	240	0	2	0	Iatrogenic hypothyroidism NOS	Feb-09
34459	C137z00	8	0	1	1	Iatrogenic pituitary disorder NOS	Feb-09
56983	C137.00	22	0	2	0	Iatrogenic pituitary disorders	Feb-09
61026	C054.00	13	0	0	0	Iatrogenic thyroiditis	Feb-09
370	PH1..00	1995	0	86	0	Ichthyosis congenita	Feb-09
64322	PH1z.00	72	0	0	0	Ichthyosis congenita NOS	Feb-09
5555	PH14.00	780	0	18	0	Ichthyosis vulgaris	Feb-09
69124	C107300	15	0	0	0	IDDM with peripheral circulatory disorder	Feb-09
18505	C108.11	8393	0	317	0	IDDM-Insulin dependent diabetes mellitus	Feb-09
37401	PB31.00	92	0	2	0	Idiopathic congenital megacolon	Feb-09
94708	F4B2100	5	0	0	0	Idiopathic corneal oedema	Feb-09
70967	C150000	6	0	1	0	Idiopathic Cushing's syndrome	Feb-09
62243	F136000	14	0	1	0	Idiopathic familial dystonia	Feb-09
28229	H563z00	214	0	1	0	Idiopathic fibrosing alveolitis NOS	Feb-09
40750	D00z200	193	0	2	0	Idiopathic hypochromic anaemia	Feb-09
25379	C121200	46	0	0	0	Idiopathic hypoparathyroidism	Feb-09

For peer review only - http://bmjopen.bmj.com/site/about/guidelines.xhtml

40428	N330300	133	0	3	0	Idiopathic osteoporosis	Feb-09
27597	N331600	116	0	6	0	Idiopathic osteoporosis with pathological fracture	Feb-09
48590	C132000	30	0	0	0	Idiopathic panhypopituitarism	Feb-09
103753	H563.13	344	0	8	0	Idiopathic pulmonary fibrosis	Feb-12
27348	H561.00	45	0	6	0	Idiopathic pulmonary haemosiderosis	Feb-09
29966	C350300	47	0	8	0	Idiopathic pulmonary haemosiderosis	Feb-09
31560	B937.12	82	0	2	0	Idiopathic thrombocythaemia	Feb-09
5144	D313.12	2000	0	197	3	Idiopathic thrombocytopenic purpura	Feb-09
12234	D313000	2308	0	47	0	Idiopathic thrombocytopenic purpura	Feb-09
39800	C39X.00	67	0	5	0	"Immunodeficiency associated+major defect, unspecified"	Feb-09
69373	C390600	6	0	0	0	Immunodeficiency with IgM hypergammaglobulinaemia	Feb-09
65617	C395.00	4	0	0	0	Immunodeficiency with short-limbed stature	Feb-09
4855	PB26.00	1267	0	29	0	Imperforate anus	Feb-09
67319	PB26z00	80	0	0	0	Imperforate anus NOS	Feb-09
61401	PB26000	56	0	0	0	Imperforate anus with fistula	Feb-09
68484	PB14.00	3	0	0	0	Imperforate jejunum	Feb-09
98741	PA33.00	0	0	0	0	Imperforate oesophagus	Mar-10
56901	PA27000	2	0	0	0	Imperforate pharynx	Feb-09
49242	PB27.00	22	0	0	0	Imperforate rectum	Feb-09
44953	PB27000	3	0	0	0	Imperforate rectum with fistula	Feb-09
22098	E140.00	1419	0	53	0	Infantile autism	Feb-09
36662	E140z00	78	0	1	0	Infantile autism NOS	Feb-09
5560	F23..12	527	0	8	0	Infantile cerebral palsy	Feb-09

For peer review only - http://bmjopen.bmj.com/site/about/guidelines.xhtml

2019	F234.00	250	0	5	0	Infantile hemiplegia NOS	Feb-09
54014	F392300	6	0	0	0	Infantile myotonia	Feb-09
41668	F460.00	49	0	3	0	"Infantile, juvenile and presenile cataracts"	Feb-09
569	G64..12	3985	0	179	0	Infarction - cerebral	Feb-09
57495	G63..11	37	0	0	0	Infarction - precerebral	Feb-09
24444	G401.11	124	0	1	0	Infarction - pulmonary	Feb-09
26424	G64z400	1122	0	5	0	Infarction of basal ganglia	Feb-09
1796	J4...12	18902	0	1309	14	Inflammatory bowel disease	Feb-09
39809	C108J00	27	0	1	0	Insulin dependent diab mell with neuropathic arthropathy	Feb-09
64446	C108G00	3	0	1	0	Insulin dependent diab mell with peripheral angiopathy	Feb-09
31310	C108900	565	0	20	0	Insulin dependent diabetes maturity onset	Feb-09
97849	C10E912	14	0	0	0	Insulin dependent diabetes maturity onset	Oct-09
1038	C100011	34834	0	2167	5	Insulin dependent diabetes mellitus	Feb-09
1647	C108.00	23978	0	838	7	Insulin dependent diabetes mellitus	Feb-09
51261	C10E.12	1339	0	1	0	Insulin dependent diabetes mellitus	Feb-09
6791	C108800	434	0	18	0	Insulin dependent diabetes mellitus - poor control	Feb-09
72702	C10E812	14	0	0	0	Insulin dependent diabetes mellitus - poor control	Feb-09
65616	C108H00	4	0	0	0	Insulin dependent diabetes mellitus with arthropathy	Feb-09
44260	C108F00	7	0	2	0	Insulin dependent diabetes mellitus with diabetic cataract	Feb-09
100770	C10EF12	1	0	0	0	Insulin dependent diabetes mellitus with diabetic cataract	Nov-10
60499	C108600	10	0	2	0	Insulin dependent diabetes mellitus with gangrene	Feb-09

For peer review only - http://bmjopen.bmj.com/site/about/guidelines.xhtml

109051	C10E612	1	0	0	0	Insulin dependent diabetes mellitus with gangrene	Jan-15
44440	C108E00	51	0	0	0	Insulin dependent diabetes mellitus with hypoglycaemic coma	Feb-09
99716	C10EE12	2	0	0	0	Insulin dependent diabetes mellitus with hypoglycaemic coma	Jun-10
24694	C108B00	1	0	0	0	Insulin dependent diabetes mellitus with mononeuropathy	Feb-09
45276	C10E312	16	0	0	0	Insulin dependent diabetes mellitus with multiple complicat	Feb-09
52104	C108300	11	0	1	0	Insulin dependent diabetes mellitus with multiple complicatn	Feb-09
57621	C108D00	18	0	0	0	Insulin dependent diabetes mellitus with nephropathy	Feb-09
102163	C10ED12	1	0	0	0	Insulin dependent diabetes mellitus with nephropathy	Apr-11
41716	C108C00	11	0	0	0	Insulin dependent diabetes mellitus with polyneuropathy	Feb-09
101311	C10EC12	2	0	0	0	Insulin dependent diabetes mellitus with polyneuropathy	Jan-11
6509	C108700	249	0	41	0	Insulin dependent diabetes mellitus with retinopathy	Feb-09
93875	C10E712	8	0	0	0	Insulin dependent diabetes mellitus with retinopathy	Feb-09
44443	C108500	215	0	3	0	Insulin dependent diabetes mellitus with ulcer	Feb-09
98704	C10E512	3	0	0	0	Insulin dependent diabetes mellitus with ulcer	Mar-10
37648	C109J11	24	0	3	0	Insulin treated non-insulin dependent diabetes mellitus	Feb-09
1407	C10FJ00	13414	0	84	0	Insulin treated Type 2 diabetes mellitus	Feb-09

For peer review only - http://bmjopen.bmj.com/site/about/guidelines.xhtml

18278	C109J00	4211	0	28	0	Insulin treated Type 2 diabetes mellitus	Feb-09
64668	C10FJ11	107	0	0	0	Insulin treated Type II diabetes mellitus	Feb-09
96010	66Ap.00	2595	0	3	0	Insulin treatment initiated	Feb-09
100791	66Ar.00	91	0	0	0	Insulin treatment stopped	Nov-10
52283	C108200	8	0	0	0	Insulin-dependent diabetes mellitus with neurological comps	Feb-09
101735	C10E212	1	0	0	0	Insulin-dependent diabetes mellitus with neurological comps	Feb-11
49276	C108100	18	0	0	0	Insulin-dependent diabetes mellitus with ophthalmic comps	Feb-09
98071	C10E112	2	0	0	0	Insulin-dependent diabetes mellitus with ophthalmic comps	Nov-09
46963	C108000	30	0	1	0	Insulin-dependent diabetes mellitus with renal complications	Feb-09
102946	C10E012	4	0	0	0	Insulin-dependent diabetes mellitus with renal complications	Sep-11
56448	C108A00	21	0	0	0	Insulin-dependent diabetes without complication	Feb-09
99719	C10EA12	2	0	0	0	Insulin-dependent diabetes without complication	Jun-10
109815	H58y600	1	0	0	0	Interstitial lung disease due to collagen vascular disease	Jul-15
104915	H58y700	35	0	1	0	Interstitial lung disease due to connective tissue disease	Sep-12
9355	J69..00	2226	0	216	0	Intestinal malabsorption	Feb-09
42715	J69z.00	212	0	2	0	Intestinal malabsorption NOS	Feb-09
49739	J69y300	10	0	2	0	Intestinal malabsorption of carbohydrate	Feb-09
31392	J69y600	85	0	4	1	Intestinal malabsorption of fat	Feb-09
49191	J69y200	37	0	29	26	Intestinal malabsorption of protein	Feb-09

For peer review only - http://bmjopen.bmj.com/site/about/guidelines.xhtml

37877	F280z11	26	0	0	0	Intracerebral cyst NOS	Feb-09
44479	Q200511	4	0	0	0	Intracerebral haematoma in fetus or newborn	Feb-09
5051	G61..00	14470	0	240	0	Intracerebral haemorrhage	Feb-09
36559	Q200011	26	0	0	0	Intracerebral haemorrhage in fetus or newborn	Feb-09
31060	G61X.00	104	0	0	0	"Intracerebral haemorrhage in hemisphere, unspecified"	Feb-09
3535	G61z.00	1877	0	81	0	Intracerebral haemorrhage NOS	Feb-09
30202	G617.00	676	0	2	0	"Intracerebral haemorrhage, intraventricular"	Feb-09
57315	G618.00	33	0	2	0	"Intracerebral haemorrhage, multiple localized"	Feb-09
66218	C3A..00	5	0	0	0	Iodine-deficiency syndromes	Feb-09
54511	C0AX.00	16	0	0	0	"Iodine-deficiency-related (endemic) goitre, unspecified"	Feb-09
37518	C0A3.00	22	0	1	0	Iodine-deficiency-related diffuse (endemic) goitre	Feb-09
44459	C0A4.00	84	0	8	0	Iodine-deficiency-related multinodular (endemic) goitre	Feb-09
48338	D000.12	118	0	1	0	Iron deficiency anaemia due to blood loss	Feb-09
27726	D000.00	2676	0	101	0	Iron deficiency anaemia due to chronic blood loss	Feb-09
15439	D00zz00	26280	0	657	17	Iron deficiency anaemia NOS	Feb-09
795	D00..00	458744	0	29240	279	Iron deficiency anaemias	Feb-09
100507	PJ33400	10	0	0	0	Jacobsen syndrome	Aug-10
50228	PG43.11	27	0	1	0	Jeune's syndrome	Feb-09
17399	F250400	897	0	32	0	Juvenile absence epilepsy	Feb-09
42405	N045000	118	0	8	0	Juvenile ankylosing spondylitis	Feb-09
12575	N045300	50	0	5	0	Juvenile arthritis in Crohn's disease	Feb-09
28456	N045200	163	0	5	0	Juvenile arthritis in psoriasis	Feb-09

For peer review only - http://bmjopen.bmj.com/site/about/guidelines.xhtml

71083	N045400	4	0	0	0	Juvenile arthritis in ulcerative colitis	Feb-09
50627	M142.00	36	0	0	0	Juvenile dermatitis herpetiformis	Feb-09
32649	N003000	91	0	1	0	Juvenile dermatomyositis	Feb-09
64693	F4B5100	9	0	0	0	Juvenile epithelial corneal dystrophy	Feb-09
105069	B693.00	3	0	0	0	Juvenile myelomonocytic leukaemia	Oct-12
19363	F25A.00	1338	0	12	0	Juvenile myoclonic epilepsy	Feb-09
45320	A904.00	3	0	0	0	Juvenile neurosyphilis	Feb-09
66740	F380100	8	0	0	0	Juvenile or adult myasthenia gravis	Feb-09
6975	N326200	899	0	17	0	Juvenile osteochondritis NOS	Feb-09
63370	N323.11	2	0	0	0	Juvenile osteochondritis of the arm	Feb-09
62237	N323.00	21	0	1	0	Juvenile osteochondritis of the arm and hand	Feb-09
73645	N323z00	1	0	0	0	Juvenile osteochondritis of the arm and hand NOS	Feb-09
101671	N323000	1	0	0	0	"Juvenile osteochondritis of the arm, unspecified"	Feb-11
39819	N323100	3	0	0	0	"Juvenile osteochondritis of the hand, unspecified"	Feb-09
34369	N321.00	370	0	1	1	Juvenile osteochondritis of the hip and pelvis	Feb-09
68673	N321z00	11	0	0	0	Juvenile osteochondritis of the hip and pelvis NOS	Feb-09
66495	N321000	7	0	0	0	"Juvenile osteochondritis of the hip and pelvis, unspecified"	Feb-09
21502	N321400	23	0	2	0	Juvenile osteochondritis of the iliac crest	Feb-09
36725	N320.00	422	0	4	0	Juvenile osteochondritis of the spine	Feb-09
69739	N320z00	16	0	0	0	Juvenile osteochondritis of the spine NOS	Feb-09

For peer review only - http://bmjopen.bmj.com/site/about/guidelines.xhtml

50510	N320000	13	0	0	0	"Juvenile osteochondritis of the spine, unspecified"	Feb-09
94462	N321500	4	0	0	0	Juvenile osteochondritis of the symphysis pubis	Feb-09
94547	N326z00	3	0	0	0	Juvenile osteochondroses NOS	Feb-09
9691	N326300	222	0	4	0	Juvenile osteochondrosis NOS	Feb-09
52681	N328.00	32	0	0	0	Juvenile osteochondrosis of spine	Feb-09
48042	N325.00	61	0	1	0	Juvenile osteochondrosis of the foot	Feb-09
60589	N325z00	14	0	0	0	Juvenile osteochondrosis of the foot NOS	Feb-09
63567	N325000	10	0	0	0	"Juvenile osteochondrosis of the foot, unspecified"	Feb-09
33667	N324.00	2827	0	3	0	Juvenile osteochondrosis of the leg	Feb-09
38898	N324z00	54	0	2	0	"Juvenile osteochondrosis of the leg, NOS"	Feb-09
24028	N324000	24	0	0	0	"Juvenile osteochondrosis of the leg, unspecified"	Feb-09
35026	N324111	15	0	0	0	Juvenile osteochondrosis of the primary patellar centre	Feb-09
48242	N324300	58	0	0	0	Juvenile osteochondrosis of the secondary patellar centre	Feb-09
17810	M142.11	20	0	1	0	Juvenile pemphigoid	Feb-09
7859	M2y4800	8465	0	78	0	Juvenile plantar dermatosis	Feb-09
18380	G759.00	115	0	6	0	Juvenile polyarteritis	Feb-09
96664	J615800	3	0	0	0	Juvenile portal cirrhosis	Apr-09
28450	F427300	109	0	4	0	Juvenile retinoschisis	Feb-09
31360	N045500	352	0	2	0	Juvenile rheumatoid arthritis	Feb-09
4186	N043.00	4230	0	258	0	Juvenile rheumatoid arthritis - Still's disease	Feb-09
27557	N043z00	225	0	2	0	Juvenile rheumatoid arthritis NOS	Feb-09
50644	N043000	37	0	2	0	Juvenile rheumatoid arthropathy unspecified	Feb-09

For peer review only - http://bmjopen.bmj.com/site/about/guidelines.xhtml

31181	N045100	188	0	7	0	Juvenile seronegative polyarthritis	Feb-09
107689	M12A400	80	0	0	0	Juvenile spring eruption	Feb-14
21876	C172111	265	0	16	0	Kallman's syndrome	Feb-09
43444	E140.11	12	0	0	0	Kanner's syndrome	Feb-09
99213	PJy2.12	2	0	0	0	"Karyotype 47, XXX"	Apr-10
9768	PJyy300	119	0	4	2	"Karyotype 47,XYY"	Feb-09
6157	G751000	2136	0	99	0	Kawasaki disease	Feb-09
31452	Q424.00	55	0	4	0	Kernicterus due to isoimmunisation	Feb-09
48012	Q437.00	8	0	0	0	Kernicterus not due to isoimmunisation	Feb-09
61800	Q437z00	7	0	0	0	Kernicterus of newborn NOS	Feb-09
69127	PKy9211	2	0	0	0	Kinky hair syndrome	Feb-09
54490	PJ70.00	29	0	0	0	"Klinefelter's phenotype, karyotype 47XXY"	Feb-09
59439	PJ7z.00	63	0	2	0	Klinefelter's syndrome NOS	Feb-09
96257	PJ72.00	1	0	0	0	"Klinefelter's syndrome, male with 46XX karyotype"	Apr-09
62414	PJ71.12	11	0	0	0	"Klinefelter's syndrome, XXXXY"	Feb-09
56545	PJ71.11	4	0	0	0	"Klinefelter's syndrome, XXXY"	Feb-09
91262	PJ73.00	10	0	0	0	"Klinefelter's syndrome, XXYY"	Feb-09
68109	PJ74.00	26	0	1	0	"Klinefelter's syndrome, XY/XXY mosaic"	Feb-09
67854	PJ71.00	3	0	1	0	"Klinefelter's syndrome,male with more than two X chromosomes"	Feb-09
73985	PG16z00	16	0	0	0	Klippel - Feil syndrome NOS	Feb-09
10491	PKy7200	411	0	18	0	Klippel - Trenaunay - Weber syndrome	Feb-09
4674	PG16.00	519	0	15	0	Klippel-Feil syndrome	Feb-09
59014	N13y100	31	0	0	0	Klippel's disease	Feb-09
71719	F257.00	3	0	0	0	Kojevnikov's epilepsy	Feb-09
35737	C20..00	32	0	2	1	Kwashiorkor	Feb-09
60113	C362300	20	0	0	0	Lactic acidaemia	Feb-09

For peer review only - http://bmjopen.bmj.com/site/about/guidelines.xhtml

37854	C313111	64	0	3	0	Lactose malabsorption	Feb-09
105082	F381011	21	0	0	0	Lambert-Eaton syndrome	Oct-12
59806	ZS82.11	29	0	1	0	Landau-Kleffner syndrome	Feb-09
40000	C37yB00	304	0	1	0	Langerhans' cell histiocytosis	Feb-09
6340	E2F3.11	2040	0	48	0	Language development disorder	Feb-09
99857	ZS7C400	2	0	0	0	Language disorder associated with right hemisphere damage	Jun-10
32831	ZS7C600	42	0	9	0	Language disorder associated with thought disorder	Feb-09
2052	13Z4E00	49540	0	2162	1	Learning difficulties	Feb-09
56376	Z7CD200	466	0	0	0	Learning difficulties	Feb-09
28113	F427J00	102	0	5	0	Leber's congenital amaurosis	Feb-09
45706	F4H1600	137	0	7	0	Leber's hereditary optic atrophy	Feb-09
48037	F4H1711	7	0	0	0	Leber's optic atrophy	Feb-09
53826	G562z00	105	0	7	0	Left bundle branch hemiblock NOS	Feb-09
8933	F222.00	2209	0	147	1	Left hemiplegia	Feb-09
26318	G563.00	283	0	4	0	Left main stem bundle branch block	Feb-09
98383	7904200	1	0	0	0	Left ventricle aorta tunnel right ventricle pul art val con	Jan-10
67657	P542.00	4	0	0	0	Left ventricle to right atrial communication	Feb-09
107397	G5yyD00	792	0	5	0	Left ventricular cardiac dysfunction	Dec-13
884	G581.00	170646	0	11892	23	Left ventricular failure	Feb-09
562	G5y3411	21482	0	436	1	Left ventricular hypertrophy	Feb-09
108457	P69..00	26	0	0	0	Left ventricular outflow tract obstruction	Aug-14
21854	G5yy700	504	0	5	0	Left ventricular thrombosis	Feb-09
69114	C303000	2	0	0	0	Leucinosis	Feb-09
59035	F100z00	9	0	0	0	Leucodystrophy NOS	Feb-09
43857	C10M.00	37	0	2	0	Lipoatrophic diabetes mellitus	Feb-09
34624	C375.14	6	0	2	0	Lipochondrodystrophy	Feb-09
56796	C326z00	39	0	1	0	Lipodystrophy NOS	Feb-09

For peer review only - http://bmjopen.bmj.com/site/about/guidelines.xhtml

39419	A3A2.11	18	0	0	0	"Lipodystrophy, intestinal"	Feb-09
19337	G56y500	1886	0	171	0	Long Q-T syndrome	Feb-09
63395	PB30000	2	0	0	0	Long segment Hirschsprung's disease	Feb-09
47768	C302500	25	0	5	0	Lowe disease	Feb-09
4125	M154.00	6746	0	484	27	Lupus erythematosus	Feb-09
33449	M154000	16	0	0	0	Lupus erythematosus chronicus	Feb-09
40797	M154200	11	0	0	0	Lupus erythematosus migrans	Feb-09
65391	M154300	17	0	0	0	Lupus erythematosus nodularis	Feb-09
7522	M154z00	239	0	4	0	Lupus erythematosus NOS	Feb-09
46148	M154400	77	0	1	0	Lupus erythematosus profundus	Feb-09
44984	M154500	42	0	1	0	Lupus erythematosus tumidus	Feb-09
63955	M154600	2	0	0	0	Lupus erythematosus unguium mutilans	Feb-09
37474	P241.00	61	0	0	0	Macroencephaly	Feb-09
16527	C333.00	337	0	27	0	Macroglobulinaemia	Feb-09
71994	C333z00	6	0	0	0	Macroglobulinaemia NOS	Feb-09
30176	PA14.00	164	0	16	0	Macroglossia	Feb-09
27325	J040.12	120	0	44	0	Macrognathism	Feb-09
73766	J040A00	6	0	0	0	Macrognathism unspecified	Feb-09
72094	P242.00	3	0	0	0	Macrogyria	Feb-09
58909	P37..00	5	0	0	0	Macrophthalmos	Feb-09
61992	PG42012	26	0	0	0	Maffuci's syndrome	Feb-09
30100	C294400	356	0	6	1	Magnesium deficiency	Feb-09
71815	PJ51000	5	0	0	0	Major partial trisomy	Feb-09
31419	J69y700	379	0	32	4	Malabsorption - iron	Feb-09
29513	J69y011	14	0	0	0	Malabsorption due to intolerance to fat	Feb-09
92869	C313911	1	0	0	0	Malabsorption of disaccharide NEC	Feb-09
38805	C313511	24	0	0	0	Malabsorption of glucose	Feb-09
57431	C313011	11	0	0	0	Malabsorption of glucose - galactose	Feb-09

For peer review only - http://bmjopen.bmj.com/site/about/guidelines.xhtml

11934	J69yz13	885	0	50	0	Malabsorption syndrome NOS	Feb-09
54216	PC71.00	20	0	2	0	Male pseudohermaphroditism	Feb-09
37484	PJy6.00	16	0	3	0	Male with structurally abnormal sex chromosome	Feb-09
37070	E110.00	1217	0	13	0	"Manic disorder, single episode"	Feb-09
36611	E110z00	46	0	2	0	"Manic disorder, single episode NOS"	Feb-09
22713	1S42.00	496	0	46	0	Manic mood	Feb-09
26161	E11..13	653	0	27	0	Manic psychoses	Feb-09
109671	C399000	14	0	0	0	Mannose-binding lectin deficiency	May-15
66577	C31y300	32	0	0	0	Mannosidosis	Feb-09
107648	C375000	2	0	0	0	Mannosidosis	Jan-14
37980	G764.11	11	0	0	0	Marable's syndrome	Feb-09
35893	C201.00	4	0	0	0	Marasmic kwashiorkor	Feb-09
103962	A78y300	3	0	0	0	Marburg disease	Apr-12
70356	PKy5600	1	0	0	0	Marchesani syndrome	Feb-09
100022	D112012	1	0	0	0	Marchiafava - Micheli syndrome	Jun-10
56153	F21y000	5	0	0	0	Marchiafava-Bignami disease	Feb-09
3884	P2x4.00	447	0	24	0	Marcus - Gunn syndrome	Feb-09
45341	F4E4113	21	0	0	0	Marcus - Gunn syndrome	Feb-09
936	PKy2.00	4054	0	539	0	Marfan's syndrome	Feb-09
99763	F142000	5	0	1	0	Marie's cerebellar ataxia	Jun-10
11631	C37z.11	885	0	65	0	Marinesco-Sjogren syndrome	Feb-09
62281	C375611	3	0	1	0	Maroteaux - Lamy syndrome	Feb-09
107550	PKy0400	4	0	0	0	Marshall-Smith syndrome	Jan-14
46624	C10C.11	222	0	9	0	Maturity onset diabetes in youth	Feb-09
98392	C10C.12	7	0	0	0	Maturity onset diabetes in youth type 1	Jan-10
59991	C10D.11	31	0	0	0	Maturity onset diabetes in youth type 2	Feb-09
49317	C310000	98	0	6	0	McArdle's disease	Feb-09
42632	PD12.00	53	0	4	0	Medullary cystic disease	Feb-09

For peer review only - http://bmjopen.bmj.com/site/about/guidelines.xhtml

41522	PD12z00	5	0	0	0	Medullary cystic disease NOS	Feb-09
64694	PD12y00	4	0	0	0	Medullary cystic disease OS	Feb-09
47135	PD12100	37	0	2	0	"Medullary cystic disease, adult type"	Feb-09
66998	PD12000	4	0	0	0	"Medullary cystic disease, juvenile type"	Feb-09
33400	C156.00	6	0	0	0	Medulloadrenal hyperfunction	Feb-09
35863	J527.00	136	0	5	0	Megacolon excluding Hirschsprung's disease	Feb-09
4816	J527z00	449	0	26	0	Megacolon NOS	Feb-09
65723	D313.14	1	0	0	0	Megakaryocytic hypoplasia	Feb-09
33442	P241.11	64	0	0	0	Megalencephaly	Feb-09
50169	P249.00	52	0	0	0	Megalencephaly	Feb-09
94733	Q48y500	2	0	0	0	Megalencephaly	Feb-09
4475	D01..11	1796	0	102	1	Megaloblastic anaemia	Feb-09
59103	D012112	24	0	0	0	Megaloblastic anaemia due to dietary causes	Feb-09
53799	D01z.11	11	0	0	0	Megaloblastic anaemia NOS	Feb-09
50050	PB56.00	3	0	0	0	Megaloduodenum	Feb-09
71255	PA75.00	1	0	0	0	Megalogastria	Feb-09
40049	K13y700	27	0	0	0	Megaloureter - acquired	Feb-09
5099	PD26.00	120	0	2	0	Megaloureter - congenital	Feb-09
41905	J100.12	7	0	0	0	Megaoesophagus	Feb-09
69752	J100000	2	0	0	1	Megaoesophagus in Chagas' disease	Feb-09
72742	PKyG.00	3	0	0	0	Men ret congen heart dis blepharophim blepharop hypopl teeth	Feb-09
4246	6664	4051	0	128	0	Mental handicap problem	Feb-09
1362	E3...00	8262	0	181	3	Mental retardation	Feb-09
37867	E3z..00	542	0	4	0	Mental retardation NOS	Feb-09
68132	6894	238	0	0	0	Mental retardation screen	Feb-09

For peer review only - http://bmjopen.bmj.com/site/about/guidelines.xhtml

41881	K032y14	246	0	7	0	Mesangiocapillary glomerulonephritis NEC	Feb-09
36342	K032y13	94	0	0	0	Mesangioproliferative glomerulonephritis NEC	Feb-09
12724	C1A0.00	3026	0	48	0	Metabolic syndrome	Feb-09
39481	C10F811	180	0	1	0	Metabolic syndrome X	Feb-09
104331	4Q21000	1	0	1	245	Metadrenaline level	Jun-12
26069	PG45.00	23	0	4	0	Metaphyseal dysostosis	Feb-09
45162	PG45.15	19	0	0	0	Metaphyseal dysplasia	Feb-09
37743	PG44100	2	0	0	0	Metatropic dwarfism	Feb-09
103145	PA15.00	3	0	0	0	Microglossia	Oct-11
15363	J040.15	451	0	21	0	Micrognathism	Feb-09
95850	J040B00	13	0	0	0	Micrognathism unspecified	Feb-09
52652	P336000	4	0	0	0	Microphakia	Feb-09
26462	P31..00	494	0	29	0	Microphthalmos	Feb-09
63439	P31z.00	36	0	1	0	Microphthalmos NOS	Feb-09
48507	P312.00	51	0	2	0	Microphthalmos with other eye anomaly	Feb-09
72284	P310.00	7	0	0	0	"Microphthalmos, unspecified"	Feb-09
161	F26..00	1198522	0	18647	32	Migraine	Feb-09
14700	F26z.00	26163	0	256	1	Migraine NOS	Feb-09
23621	F262z00	803	0	19	0	Migraine variant NOS	Feb-09
103502	F260.11	1592	0	8	0	Migraine with aura	Jan-12
103602	F261.11	325	0	0	0	Migraine without aura	Jan-12
3018	663V100	20542	0	18	0	Mild asthma	Feb-09
33841	F370200	299	0	6	0	Miller-Fisher syndrome	Feb-09
18307	F39X.00	335	0	15	0	"Mitochondrial myopathy, not elsewhere classified"	Feb-09
31727	G133.00	398	0	6	0	Mitral and aortic incompetence	Feb-09
94872	G133.11	4	0	0	0	Mitral and aortic insufficiency	Feb-09
11878	G133.12	3310	0	63	3	Mitral and aortic regurgitation	Feb-09
29158	G13z.00	463	0	16	0	Mitral and aortic valve disease NOS	Feb-09

For peer review only - http://bmjopen.bmj.com/site/about/guidelines.xhtml

21807	G111.11	74	0	12	0	Mitral incompetence - rheumatic	Feb-09
31759	G132.12	13	0	1	0	Mitral incompetence and aortic stenosis	Feb-09
34240	G540100	382	0	5	0	"Mitral incompetence, cause unspecified"	Feb-09
5058	G540000	2806	0	199	0	"Mitral incompetence, non-rheumatic"	Feb-09
33262	G132.00	110	0	4	0	Mitral insufficiency and aortic stenosis	Feb-09
561	G540.16	38256	0	949	3	Mitral regurgitation	Feb-09
22837	G111.12	137	0	0	0	Mitral regurgitation - rheumatic	Feb-09
33907	G132.13	247	0	2	0	Mitral regurgitation and aortic stenosis	Feb-09
1885	G110.00	9886	0	405	2	Mitral stenosis	Feb-09
61250	G131.13	19	0	0	0	Mitral stenosis and aortic incompetence	Feb-09
49355	G131.00	34	0	3	0	Mitral stenosis and aortic insufficiency	Feb-09
50983	G112.12	5	0	0	0	Mitral stenosis with incompetence	Feb-09
44488	G112.00	165	0	0	0	Mitral stenosis with insufficiency	Feb-09
44328	G112.13	72	0	2	0	Mitral stenosis with regurgitation	Feb-09
30443	G11z.00	687	0	11	0	Mitral valve disease NOS	Feb-09
1267	G11..00	5419	0	385	4	Mitral valve diseases	Feb-09
24557	G540z00	427	0	11	0	Mitral valve disorders NOS	Feb-09
2977	G540.00	7682	0	454	5	Mitral valve incompetence	Feb-09
40949	G540.12	31	0	0	0	Mitral valve insufficiency	Feb-09
39916	G540300	307	0	3	0	Mitral valve leaf prolapse	Feb-09
1294	G540.15	11485	0	488	2	Mitral valve prolapse	Feb-09
31839	G540200	799	0	15	0	Mitral valve prolapse	Feb-09
9450	G540.14	3919	0	59	0	Mitral valve regurgitation	Feb-09
25796	H332.00	960	0	2	0	Mixed asthma	Feb-09
31316	E116.00	1458	0	46	0	Mixed bipolar affective disorder	Feb-09

For peer review only - http://bmjopen.bmj.com/site/about/guidelines.xhtml

55064	E116600	92	0	0	0	"Mixed bipolar affective disorder, in full remission"	Feb-09
24689	E116100	31	0	0	0	"Mixed bipolar affective disorder, mild"	Feb-09
63150	E116200	26	0	0	0	"Mixed bipolar affective disorder, moderate"	Feb-09
63583	E116z00	152	0	1	0	"Mixed bipolar affective disorder, NOS"	Feb-09
63651	E116500	7	0	0	0	"Mixed bipolar affective disorder, partial/unspec remission"	Feb-09
54195	E116400	44	0	0	0	"Mixed bipolar affective disorder, severe, with psychosis"	Feb-09
63284	E116300	9	0	0	0	"Mixed bipolar affective disorder, severe, without psychosis"	Feb-09
31535	E116000	237	0	1	0	"Mixed bipolar affective disorder, unspecified"	Feb-09
73492	PJ60.00	8	0	0	0	Mixed gonadal dysgenesis	Feb-09
103752	G561311	13	0	1	0	Mobitz type 1 second degree atrioventricular block	Feb-12
27928	G561300	437	0	18	0	Mobitz type I (Wenckebach) atrioventricular block	Feb-09
13065	663V200	14648	0	13	0	Moderate asthma	Feb-09
302	E310.00	699	0	8	0	"Moderate mental retardation, IQ in range 35-49"	Feb-09
107350	F591C00	446	0	9	0	Moderate sensorineural hearing loss	Dec-13
36560	F326200	108	0	8	0	Moebius congenital oculofacial paralysis	Feb-09
65262	F26y111	3	0	1	0	Moebius' ophthalmoplegic migraine	Feb-09
52270	J094200	11	0	0	0	Moeller's glossodynia exfoliativa	Feb-09
67072	PKy5100	5	0	0	0	Mohr's syndrome	Feb-09

For peer review only - http://bmjopen.bmj.com/site/about/guidelines.xhtml

36276	N043300	127	0	1	0	Monarticular juvenile rheumatoid arthritis	Feb-09
50604	13L2.12	9	0	0	0	Mongol child in family	Feb-09
23489	PJ0..11	171	0	6	0	Mongolism	Feb-09
37105	PJ3..00	25	0	0	0	Monosomies and deletions from the autosomes	Feb-09
98395	PJ3z.00	4	0	0	0	Monosomies and deletions from the autosomes NOS	Jan-10
58584	C375411	31	0	0	0	Morquio - Brailsford syndrome	Feb-09
105345	PJy1300	1	0	0	0	Mosaic including XXXXY	Dec-12
32783	PJy1100	24	0	1	0	Mosaic XO/XX	Feb-09
33269	PJy1000	12	0	2	0	Mosaic XO/XY	Feb-09
64578	PJy1200	2	0	1	0	Mosaic XY/XXY	Feb-09
26140	PJz0.00	93	0	0	0	Mosaicism NOS	Feb-09
23643	PJy5.00	9	0	0	0	"Mosaicism, lines with various numbers of X chromosomes"	Feb-09
65699	F255012	18	0	0	0	Motor epilepsy	Feb-09
93694	PJ9..00	15	0	1	0	Mowat-Wilson syndrome	Feb-09
11946	PD13.11	745	0	25	0	Multicystic kidney	Feb-09
30041	C164.13	227	0	3	0	Multicystic ovaries	Feb-09
18331	PD13.00	251	0	4	0	Multicystic renal dysplasia	Feb-09
16530	PK7..00	134	0	6	0	Multiple congenital anomalies NOS	Feb-09
98724	PK7z.00	4	0	0	0	Multiple congenital anomalies NOS	Mar-10
50980	P841.12	22	0	0	0	Multiple congenital bronchogenic cysts	Feb-09
109441	P240100	1	0	0	0	Multiple congenital cerebral cysts	Mar-15
69886	F201.00	16	0	0	0	Multiple sclerosis of the spinal cord	Feb-09
57338	G544X00	62	0	3	0	"Multiple valve disease, unspecified"	Feb-09
40239	G544.00	46	0	2	0	Multiple valve diseases	Feb-09
22796	ZT4C211	229	0	14	0	Mute	Feb-09

For peer review only - http://bmjopen.bmj.com/site/about/guidelines.xhtml

36541	E2D2211	56	0	4	0	Mutism of childhood or adolescence	Feb-09
27515	F380z00	179	0	2	0	Myasthenia gravis NOS	Feb-09
95005	F381200	1	0	0	0	Myasthenic syndrome due to botulism	Feb-09
39420	F381300	39	0	2	0	Myasthenic syndrome due to diabetic amyotrophy	Feb-09
51640	F381.00	6	0	1	0	Myasthenic syndrome due to disease EC	Feb-09
65825	F381z00	11	0	0	0	Myasthenic syndrome due to disease NOS	Feb-09
61069	F381400	8	0	0	0	Myasthenic syndrome due to hypothyroidism	Feb-09
57551	F381100	2	0	0	0	Myasthenic syndrome due to other malignancy	Feb-09
56973	F381500	6	0	0	0	Myasthenic syndrome due to pernicious anaemia	Feb-09
47695	F381600	3	0	0	0	Myasthenic syndrome due to thyrotoxicosis	Feb-09
94690	F13B.00	191	0	13	0	Myoclonic dystonia	Feb-09
45602	F132200	80	0	4	0	Myoclonic encephalopathy	Feb-09
8487	F132z12	1879	0	176	0	Myoclonic seizure	Feb-09
37897	F132z00	229	0	10	0	Myoclonus NOS	Feb-09
96966	N248000	1417	0	28	0	Myofascial pain syndrome	Jun-09
105696	PG42100	1	0	0	0	Myotonic chondrodysplasia	Jan-13
44867	F392z00	97	0	3	0	Myotonic disorder NOS	Feb-09
11779	F271.00	4394	0	441	0	Narcolepsy	Feb-09
56614	Q488.00	23	0	0	0	Neonatal cerebral ischaemia	Feb-09
48202	Q48A.00	49	0	0	0	Neonatal cerebral leukomalacia	Feb-09
65686	Q444.12	4	0	0	0	Neonatal hypoparathroidism	Feb-09
106500	Q44A.00	1	0	0	0	Neonatal hypoparathyroidism	Aug-13
99435	N000500	14	0	0	0	Neonatal lupus erythematosus	May-10

For peer review only - http://bmjopen.bmj.com/site/about/guidelines.xhtml

37782	F251100	110	0	2	0	Neonatal myoclonic epilepsy	Feb-09
10647	K02..11	392	0	10	0	Nephritis - chronic	Feb-09
4850	K03..11	203	0	6	0	Nephritis and nephropathy unspecified	Feb-09
33580	K03..00	491	0	17	0	Nephritis and nephropathy unspecified	Feb-09
94350	K032z00	3	0	0	0	Nephritis unsp+membranoprolif glomerulonephritis lesion NOS	Feb-09
67193	K032y00	29	0	1	0	Nephritis unsp+OS membranoprolif glomerulonephritis lesion	Feb-09
2773	K0...00	1430	0	97	2	"Nephritis, nephrosis and nephrotic syndrome"	Feb-09
30310	K081.00	249	0	7	0	Nephrogenic diabetes insipidus	Feb-09
42042	PD12011	17	0	0	0	Nephronophthisis	Feb-09
109106	PD12200	2	0	0	0	Nephronophthisis - medullary cystic disease	Jan-15
68112	C373600	7	0	0	0	Nephropathic amyloidosis	Feb-09
11875	K02..12	875	0	41	0	Nephropathy - chronic	Feb-09
41159	K0C1.00	63	0	0	0	Nephropathy induced by other drugs meds and biologl substncs	Feb-09
57784	K0C2.00	13	0	0	0	Nephropathy induced by unspec drug medicament or biol subs	Feb-09
38312	L162.12	16	0	2	1	Nephropathy NOS in pregnancy without hypertension	Feb-09
11873	K03..12	1478	0	43	0	"Nephropathy, unspecified"	Feb-09
21947	K017.00	129	0	1	0	Nephrotic syn difus mesangial prolifertiv glomerulonephritis	Feb-09
21989	K019.00	73	0	1	0	"Nephrotic syn,diffuse mesangiocapillary glomerulonephritis"	Feb-09
50472	K018.00	4	0	0	0	"Nephrotic syn,difus endocapilary proliftv glomerulonephritis"	Feb-09

For peer review only - http://bmjopen.bmj.com/site/about/guidelines.xhtml

2999	K01..00	15444	0	855	5	Nephrotic syndrome	Feb-09
47922	K01x000	80	0	2	0	Nephrotic syndrome in amyloidosis	Feb-09
2471	K01x100	283	0	7	0	Nephrotic syndrome in diabetes mellitus	Feb-09
108816	K01x.00	2	0	0	0	Nephrotic syndrome in diseases EC	Nov-14
99201	K01x200	1	0	0	0	Nephrotic syndrome in malaria	Apr-10
58750	K01x300	7	0	0	0	Nephrotic syndrome in polyarteritis nodosa	Feb-09
47672	K01x400	57	0	3	0	Nephrotic syndrome in systemic lupus erythematosus	Feb-09
27427	K01z.00	305	0	9	0	Nephrotic syndrome NOS	Feb-09
1803	K011.00	515	0	9	0	Nephrotic syndrome with membranous glomerulonephritis	Feb-09
29634	K013.00	180	0	4	0	Nephrotic syndrome with minimal change glomerulonephritis	Feb-09
94373	K01y.00	4	0	0	0	Nephrotic syndrome with other pathological kidney lesions	Feb-09
9840	K010.00	19	0	0	0	Nephrotic syndrome with proliferative glomerulonephritis	Feb-09
56987	K01A.00	12	0	0	0	"Nephrotic syndrome, dense deposit disease"	Feb-09
17365	K01B.00	52	0	0	0	"Nephrotic syndrome, diffuse crescentic glomerulonephritis"	Feb-09
22852	K015.00	208	0	20	0	"Nephrotic syndrome, focal and segmental glomerular lesions"	Feb-09
23913	K014.00	66	0	1	0	"Nephrotic syndrome, minor glomerular abnormality"	Feb-09
99644	K012.00	4	0	0	0	Nephrotic syndrome+membranoproliferative glomerulonephritis	Jun-10
3171	F591211	2006	0	241	0	Nerve deafness	Feb-09
104026	F38..11	42	0	5	0	Neuromuscular disease	May-12

For peer review only - http://bmjopen.bmj.com/site/about/guidelines.xhtml

64871	N082N00	5	0	0	0	Neuromuscular dislocation of the hip	Feb-09
44795	F210.00	149	0	4	0	Neuromyelitis optica	Feb-09
101786	F392400	58	0	1	0	Neuromyotonia	Feb-11
10234	J61y100	32345	0	773	2	Non-alcoholic fatty liver	Feb-09
57897	D111.00	7	0	1	0	Non-autoimmune haemolytic anaemia	Feb-09
94214	D111z00	2	0	0	0	Non-autoimmune haemolytic anaemia NOS	Feb-09
69278	C109E00	18	0	0	0	Non-insulin depend diabetes mellitus with diabetic cataract	Feb-09
40962	C109H00	10	0	0	0	Non-insulin dependent d m with neuropathic arthropathy	Feb-09
506	C100112	95279	0	4539	11	Non-insulin dependent diabetes mellitus	Feb-09
8403	C109700	1745	0	45	0	Non-insulin dependent diabetes mellitus - poor control	Feb-09
24693	C109G00	4	0	0	0	Non-insulin dependent diabetes mellitus with arthropathy	Feb-09
40401	C109500	14	0	1	0	Non-insulin dependent diabetes mellitus with gangrene	Feb-09
43785	C109D00	10	0	0	0	Non-insulin dependent diabetes mellitus with hypoglyca coma	Feb-09
72320	C109A00	3	0	1	0	Non-insulin dependent diabetes mellitus with mononeuropathy	Feb-09
59365	C109C00	60	0	2	0	Non-insulin dependent diabetes mellitus with nephropathy	Feb-09
45467	C109B00	14	0	0	0	Non-insulin dependent diabetes mellitus with polyneuropathy	Feb-09
34912	C109400	229	0	4	0	Non-insulin dependent diabetes mellitus with ulcer	Feb-09

For peer review only - http://bmjopen.bmj.com/site/about/guidelines.xhtml

54212	C109F00	9	0	0	0	Non-insulin-dependent d m with peripheral angiopath	Feb-09
62146	C109300	9	0	2	0	Non-insulin-dependent diabetes mellitus with multiple comps	Feb-09
55842	C109200	15	0	0	0	Non-insulin-dependent diabetes mellitus with neuro comps	Feb-09
50429	C109100	19	0	3	0	Non-insulin-dependent diabetes mellitus with ophthalm comps	Feb-09
52303	C109000	16	0	2	0	Non-insulin-dependent diabetes mellitus with renal comps	Feb-09
29979	C109900	267	0	6	0	Non-insulin-dependent diabetes mellitus without complication	Feb-09
10068	PKy8000	741	0	36	0	Noonan's syndrome	Feb-09
15566	E203z00	1842	0	105	0	Obsessive-compulsive disorder NOS	Feb-09
3208	E203.00	16765	0	2315	0	Obsessive-compulsive disorders	Feb-09
46177	PD2z.00	69	0	2	0	Obstructive defect of renal pelvis or ureter NOS	Feb-09
99128	P6y3.00	10	0	0	0	Obstructive heart anomaly NEC	Apr-10
97818	P6y3z00	4	0	0	0	Obstructive heart anomaly NEC NOS	Oct-09
101873	337C.00	7	0	0	13	Obstructive ventilatory defect	Mar-11
108543	PKy5M00	3	0	0	0	Oculofaciocardiodental syndrome	Aug-14
68816	A213.00	1	0	0	0	Oculoglandular tularaemia	Feb-09
44064	PG0E.00	2	0	0	0	Oculomandibular dysostosis	Feb-09
111653	PKy5900	1	0	0	0	Oculo-palato-digital syndrome	Feb-17
38448	F391700	68	0	3	0	Oculopharyngeal muscular dystrophy	Feb-09
37408	PA3..00	39	0	1	0	"Oesophageal atresia, stenosis and fistula"	Feb-09
16713	J103.00	1924	0	53	0	Oesophageal stricture and stenosis	Feb-09
41141	J103z00	658	0	8	0	Oesophageal stricture and stenosis NOS	Feb-09
37868	PA32100	52	0	1	0	Oesophagotracheal fistula	Feb-09

For peer review only - http://bmjopen.bmj.com/site/about/guidelines.xhtml

61322	PKyG.11	24	0	0	0	Ohdo blepharophimosis syndrome	Feb-09
51998	F259.11	21	0	0	0	Ohtahara syndrome	Feb-09
38418	J32..11	65	0	1	0	Omphalocele	Feb-09
100368	J321000	1	0	0	0	Omphalocele with obstruction	Aug-10
3826	F4H1.00	5675	0	242	0	Optic atrophy	Feb-09
95763	F4H1300	4	0	0	0	Optic atrophy due to retinal dystrophy	Feb-09
41833	F4H1z00	172	0	3	0	Optic atrophy NOS	Feb-09
47676	F4H5z00	15	0	2	0	Optic chiasm disorder NOS	Feb-09
47132	F4H5.00	35	0	2	0	Optic chiasm disorders	Feb-09
63298	SJ01.00	4	0	0	0	Optic chiasm injury	Feb-09
26835	F4H3z00	280	0	2	0	Optic neuritis NOS	Feb-09
49307	PKy5000	29	0	6	0	Oral - facial - digital syndrome	Feb-09
56160	PGy0212	2	0	0	0	Orbinsky syndrome	Feb-09
29616	J08z900	90	0	2	0	Orofacial Crohn's disease	Feb-09
23356	F138100	657	0	29	5	Orofacial dyskinesia	Feb-09
22814	J08zB00	346	0	12	0	Orofacial granulomatosis	Feb-09
3156	N327.00	5189	0	322	1	Osteochondritis dissecans	Feb-09
38335	N327100	51	0	0	0	Osteochondritis dissecans of lateral femoral condyle	Feb-09
42068	N327y00	26	0	2	0	Osteochondritis dissecans of other site	Feb-09
54563	N327000	63	0	0	0	Osteochondritis dissecans of patella	Feb-09
70914	N327400	16	0	0	0	Osteochondritis dissecans of the capitellum	Feb-09
34206	N327800	7	0	0	0	Osteochondritis dissecans of the femoral head	Feb-09
103515	N327300	2	0	0	0	Osteochondritis dissecans of the humeral head	Jan-12
97447	N327500	3	0	0	0	Osteochondritis dissecans of the radial head	Aug-09

For peer review only - http://bmjopen.bmj.com/site/about/guidelines.xhtml

38518	N327900	48	0	0	0	Osteochondritis dissecans of the talus	Feb-09
97466	N327700	2	0	0	0	Osteochondritis dissecans of the wrist	Aug-09
63565	PGW..00	4	0	2	0	Osteochondrodyspl with defct growth tub bone spine unspec	Feb-09
68427	PG5z.11	10	0	1	0	Osteochondrodysplasia	Feb-09
55775	C375.17	19	0	0	0	Osteochondrodystrophy	Feb-09
22861	N32zz00	157	0	2	0	Osteochondropathy NOS	Feb-09
17135	N32z300	969	0	7	0	Osteochondrosis NOS	Feb-09
36753	PG5..00	29	0	2	0	Osteodysplasia	Feb-09
55331	PG5z.00	12	0	1	0	Osteodysplasia NOS	Feb-09
96403	PG50.00	5	0	0	0	"Osteodysplasia, unspecified"	Apr-09
38450	PG5..11	118	0	2	0	Osteodystrophy	Feb-09
26864	N33zD00	318	0	5	0	Osteolysis	Feb-09
22417	N33zH00	172	0	9	0	Osteolytic lesion	Feb-09
29734	N33z.11	101	0	25	0	Osteolytic lesion	Feb-09
4794	B73..12	4464	0	443	0	Osteoma	Feb-09
29735	B30..12	128	0	1	0	Osteoma	Feb-09
10450	C28..11	4171	0	150	8	Osteomalacia	Feb-09
37350	C282.00	144	0	4	0	Osteomalacia unspecified	Feb-09
42657	N334900	398	0	9	0	Osteonecrosis due to drugs	Feb-09
69157	N334C00	7	0	0	0	Osteonecrosis due to haemoglobinopathy	Feb-09
45737	N334A00	181	0	8	0	Osteonecrosis due to previous trauma	Feb-09
58843	N334B00	10	0	0	0	Osteonecrosis in caisson disease	Feb-09
59172	PG42.16	16	0	0	0	Osteopathia striata	Feb-09
54213	N307.00	54	0	5	0	Osteopathy from poliomyelitis	Feb-09
37298	N3...00	200	0	4	0	Osteopathy/chondropathy/acquired musculoskeletal deformity	Feb-09
101249	2126600	389	0	24	0	Osteopenia resolved	Jan-11

For peer review only - http://bmjopen.bmj.com/site/about/guidelines.xhtml

21970	N21z312	130	0	4	0	Osteophyte of unspecified site	Feb-09
39943	PG53.00	71	0	2	0	Osteopoikilosis	Feb-09
277	N330.00	329884	0	14435	329	Osteoporosis	Feb-09
25650	N330D00	67	0	2	0	Osteoporosis due to corticosteroids	Feb-09
31580	N330A00	10	0	0	0	Osteoporosis in endocrine disorders	Feb-09
60433	N330900	17	0	0	0	Osteoporosis in multiple myelomatosis	Feb-09
34798	N330z00	4190	0	116	0	Osteoporosis NOS	Feb-09
33526	N331300	55	0	0	0	Osteoporosis of disuse with pathological fracture	Feb-09
51053	P561.00	53	0	0	0	Ostium primum defect	Feb-09
54243	P55z.00	73	0	0	0	Ostium secundum atrial septal defect NOS	Feb-09
104307	2126800	83	0	8	0	Ostium secundum atrial septal defect resolved	Jun-12
54838	C155z00	27	0	0	0	Other adrenal hypofunction NOS	Feb-09
67273	C152700	1	0	0	0	Other adrenogenital syndrome with salt loss	Feb-09
69764	C152800	15	0	1	0	Other adrenogenital syndrome without mention of salt loss	Feb-09
50220	P72..00	70	0	2	0	Other anomalies of aorta	Feb-09
44781	P72z.00	39	0	0	0	Other anomalies of aorta NOS	Feb-09
50278	PB5..00	12	0	0	0	Other anomalies of intestine	Feb-09
29373	P83..00	22	0	3	0	"Other anomalies of larynx, trachea and bronchus"	Feb-09
36779	P83z.00	6	0	0	0	"Other anomalies of larynx, trachea or bronchus NOS"	Feb-09
11945	P72zz00	60	0	1	0	Other anomaly of aorta NOS	Feb-09
104976	P83yy00	3	0	0	0	Other anomaly of bronchus	Sep-12
63507	P83yw00	12	0	0	0	Other anomaly of larynx	Feb-09
63637	P83y.00	4	0	0	0	"Other anomaly of larynx, trachea and bronchus"	Feb-09

For peer review only - http://bmjopen.bmj.com/site/about/guidelines.xhtml

103042	P83yz00	1	0	0	0	"Other anomaly of larynx, trachea or bronchus NOS"	Oct-11
69913	PG0yz00	9	0	0	0	Other anomaly of skull or face bone NOS	Feb-09
56878	PG1y.00	33	0	1	0	Other anomaly of spine	Feb-09
22768	PG1yz00	107	0	11	1	Other anomaly of spine NOS	Feb-09
70193	P83yx00	5	0	0	0	Other anomaly of trachea	Feb-09
66220	PFyz.00	7	0	1	0	Other anomaly of unspecified limb NOS	Feb-09
71400	F15y.00	5	0	4	0	Other anterior horn cell disease	Feb-09
15488	C134z00	1176	0	255	4	Other anterior pituitary disorder NOS	Feb-09
23560	C131.00	81	0	4	0	Other anterior pituitary hyperfunction	Feb-09
50762	F130z00	35	0	0	0	Other basal ganglia degenerative disease NOS	Feb-09
21863	F130.00	207	0	16	8	Other basal ganglia degenerative diseases	Feb-09
72653	G565300	29	0	6	0	Other bilateral bundle branch block	Feb-09
37613	J66yz00	76	0	5	4	Other bile duct disorder NOS	Feb-09
29066	J66..00	78	0	1	0	Other biliary tract disorders	Feb-09
69100	F427H00	7	0	0	0	Other Bruch's membrane dystrophy	Feb-09
39003	G565z00	146	0	0	0	Other bundle branch block NOS	Feb-09
31133	G57yz00	191	0	6	0	Other cardiac dysrhythmia NOS	Feb-09
7827	G57y.00	2771	0	396	18	Other cardiac dysrhythmias	Feb-09
65499	P7yzz00	8	0	0	0	Other cardiovascular system anomaly NOS	Feb-09
101592	P7yz.00	6	0	0	0	Other cardiovascular system anomaly NOS	Jan-11
34976	F11y.00	30	0	1	0	Other cerebral degeneration	Feb-09
31524	F11yz00	27	0	1	0	Other cerebral degeneration NOS	Feb-09
31892	F11..00	241	0	1	0	Other cerebral degenerations	Feb-09

For peer review only - http://bmjopen.bmj.com/site/about/guidelines.xhtml

104782	F2By.00	5	0	0	0	Other cerebral palsy	Aug-12
71819	E14y.00	1	0	1	0	Other childhood psychoses	Feb-09
66757	E14yz00	4	0	0	0	Other childhood psychoses NOS	Feb-09
60960	K02y.00	12	0	0	0	Other chronic glomerulonephritis	Feb-09
63615	K02yz00	12	0	0	0	Other chronic glomerulonephritis NOS	Feb-09
54113	G41y.00	169	0	0	0	Other chronic pulmonary heart disease	Feb-09
71046	G41yz00	24	0	0	0	Other chronic pulmonary heart disease NOS	Feb-09
26291	PF5r.00	247	0	20	0	Other congenital anomalies of fingers	Feb-09
66010	PF5u.00	23	0	0	0	Other congenital anomalies of forearm	Feb-09
59150	PF6v.00	16	0	1	0	Other congenital anomalies of lower leg	Feb-09
25190	PF6x.00	45	0	2	0	Other congenital anomalies of pelvis	Feb-09
55114	PF6xz00	5	0	0	0	Other congenital anomalies of pelvis NOS	Feb-09
65384	P76..11	8	0	0	0	Other congenital anomalies of peripheral arteries	Feb-09
22070	P76..12	109	0	23	0	Other congenital anomalies of peripheral veins	Feb-09
31378	PG3x.00	72	0	2	0	Other congenital anomalies of ribs	Feb-09
63972	PF5w.00	21	0	0	0	Other congenital anomalies of shoulder	Feb-09
51424	PG3y.00	13	0	0	0	Other congenital anomalies of sternum	Feb-09
92299	PF6w.00	9	0	0	0	Other congenital anomalies of upper leg	Feb-09

For peer review only - http://bmjopen.bmj.com/site/about/guidelines.xhtml

48510	PF5x.00	21	0	2	0	Other congenital anomalies of whole arm	Feb-09
61558	PF5uz00	7	0	0	0	Other congenital anomaly forearm NOS	Feb-09
107541	PK1yz00	1	0	0	0	Other congenital anomaly of adrenal gland NOS	Dec-13
94116	PB6yy00	4	0	0	0	Other congenital anomaly of hepatic or bile ducts	Feb-09
66182	PB6yw00	12	0	0	0	Other congenital anomaly of liver	Feb-09
37517	PA25y00	31	0	3	0	Other congenital anomaly of palate	Feb-09
73738	P76yz00	12	0	0	0	Other congenital anomaly of peripheral vascular system NOS	Feb-09
22437	PCyw.00	247	0	58	0	Other congenital anomaly of testis or scrotum	Feb-09
53178	F23y.00	38	0	0	0	Other congenital cerebral palsy	Feb-09
9778	P7...00	48	0	3	0	Other congenital circulatory system anomalies	Feb-09
59031	PD1yz00	8	0	0	0	Other congenital cystic kidney disease NOS	Feb-09
42998	PB...00	31	0	1	0	Other congenital digestive system anomaly	Feb-09
5621	P6...00	654	0	30	0	Other congenital heart anomalies	Feb-09
63834	PF63z00	38	0	1	0	Other congenital hip joint deformity NOS	Feb-09
14649	PF...00	435	0	42	0	Other congenital limb anomalies	Feb-09
37167	PE9..11	243	0	12	0	Other congenital musculoskeletal deformity	Feb-09
62976	P355.00	29	0	0	0	Other congenital retinal changes	Feb-09
25692	P355z00	234	0	21	0	Other congenital retinal changes NOS	Feb-09
72991	PA...00	5	0	0	0	Other congenital upper alimentary tract anomalies	Feb-09

For peer review only - http://bmjopen.bmj.com/site/about/guidelines.xhtml

68138	7A04.00	3	0	0	0	Other connection from aorta to pulmonary artery	Feb-09
44962	C153.00	9	0	0	0	Other corticoadrenal overactivity	Feb-09
57274	D01z.00	81	0	3	0	Other deficiency anaemias NOS	Feb-09
69820	C13y400	5	0	1	0	Other diencephalic syndrome	Feb-09
95921	PG44z00	2	0	0	0	Other dwarfing syndromes NOS	Feb-09
38307	F25y.00	338	0	10	0	Other forms of epilepsy	Feb-09
9979	F25yz00	364	0	5	0	Other forms of epilepsy NOS	Feb-09
28031	F26y.00	505	0	4	0	Other forms of migraine	Feb-09
28092	F26yz00	453	0	3	0	Other forms of migraine NOS	Feb-09
32176	F4K5600	61	0	0	0	Other forms of nystagmus	Feb-09
73663	P74z.00	2	0	0	0	Other great vein anomalies	Feb-09
73554	P74zz00	6	0	0	0	Other great vein anomaly NOS	Feb-09
39843	G566.00	280	0	1	0	Other heart block	Feb-09
46178	G566z00	168	0	1	0	Other heart block NOS	Feb-09
27304	C111.00	362	0	7	0	Other hyperinsulinism	Feb-09
50491	C111z00	51	0	2	0	Other hyperinsulinism NOS	Feb-09
44026	N10y.00	57	0	4	2	Other inflammatory spondylopathies	Feb-09
37892	N10yz00	37	0	0	0	Other inflammatory spondylopathies NOS	Feb-09
7196	N045.00	3433	0	94	0	Other juvenile arthritis	Feb-09
50425	N326.00	13	0	0	0	Other juvenile osteochondroses	Feb-09
43899	F427700	57	0	0	0	Other pigmented retinal dystrophies	Feb-09
44186	C13y.00	5	0	0	0	Other pituitary disorders + diencephalohypophyseal syndromes	Feb-09
22836	C163300	308	0	20	0	Ovarian hypogonadism	Feb-09
23802	C163.11	24	0	2	0	Ovarian hypogonadism	Feb-09
37902	N310z00	367	0	4	0	Paget's disease NOS	Feb-09
3528	N310.11	13822	0	1224	12	Paget's disease of bone	Feb-09

For peer review only - http://bmjopen.bmj.com/site/about/guidelines.xhtml

56078	N310y00	95	0	5	0	Paget's disease OS	Feb-09
95037	N310A00	1	0	0	0	Paget's disease-carpal bone	Feb-09
50862	N310000	5	0	0	0	Paget's disease-cervical spine	Feb-09
68199	N310500	17	0	0	0	Paget's disease-clavicle	Feb-09
57905	N310E00	38	0	3	0	Paget's disease-femur	Feb-09
72456	N310H00	1	0	0	0	Paget's disease-fibula	Feb-09
63779	N310700	5	0	0	0	Paget's disease-humerus	Feb-09
56021	N310200	48	0	3	0	Paget's disease-lumbar spine	Feb-09
66795	N310x00	16	0	0	0	Paget's disease-multiple sites	Feb-09
56897	N310F00	6	0	0	0	Paget's disease-patella	Feb-09
27501	N310D00	186	0	1	0	Paget's disease-pelvis	Feb-09
65387	N310800	6	0	1	0	Paget's disease-radius	Feb-09
56578	N310300	23	0	0	0	Paget's disease-sacrum	Feb-09
70397	N310600	3	0	0	0	Paget's disease-scapula	Feb-09
51146	N310P00	29	0	1	0	Paget's disease-skull	Feb-09
71514	N310100	4	0	1	0	Paget's disease-thoracic spine	Feb-09
35769	N310G00	34	0	0	0	Paget's disease-tibia	Feb-09
73964	N310900	1	0	0	0	Paget's disease-ulna	Feb-09
5026	C132.00	2554	0	129	14	Panhypopituitarism	Feb-09
33653	C132z00	54	0	0	0	Panhypopituitarism NOS	Feb-09
98998	PKy5011	5	0	2	0	Papillon-Leage-Psaume syndrome	Apr-10
1494	E103.00	27706	0	1069	0	Paranoid schizophrenia	Feb-09
36172	E103500	323	0	0	0	Paranoid schizophrenia in remission	Feb-09
9281	E103z00	346	0	4	0	Paranoid schizophrenia NOS	Feb-09
99040	F230.11	2	0	0	0	Paraplegia - congenital	Apr-10
12386	C332z00	742	0	28	0	Paraproteinaemia NOS	Feb-09
64789	C12z.00	48	0	0	0	Parathyroid disorder NOS	Feb-09
16433	C12..00	456	0	72	2	Parathyroid gland disorders	Feb-09
107472	G573600	155	0	6	0	Paroxysmal atrial flutter	Dec-13
23647	G570100	178	0	1	0	Paroxysmal atrioventricular tachycardia	Feb-09
51845	G570200	51	0	2	0	Paroxysmal junctional tachycardia	Feb-09

For peer review only - http://bmjopen.bmj.com/site/about/guidelines.xhtml

35612	D112100	252	0	9	0	Paroxysmal nocturnal haemoglobinuria	Feb-09
29491	G570300	296	0	23	0	Paroxysmal nodal tachycardia	Feb-09
107057	F139000	6	0	0	0	Paroxysmal non-kinesigenic dyskinesia	Oct-13
4940	G570.00	11026	0	444	0	Paroxysmal supraventricular tachycardia	Feb-09
35124	G570z00	274	0	11	0	Paroxysmal supraventricular tachycardia NOS	Feb-09
1381	G572z00	7791	0	848	1	Paroxysmal tachycardia NOS	Feb-09
25266	G572.00	805	0	14	0	Paroxysmal tachycardia unspecified	Feb-09
24409	F335.11	125	0	8	0	Parsonage - Aldren - Turner syndrome	Feb-09
98870	F255311	3	0	0	0	Partial epilepsy with autonomic symptoms	Apr-10
32288	F254.00	466	0	12	0	Partial epilepsy with impairment of consciousness	Feb-09
31920	F254z00	212	0	8	0	Partial epilepsy with impairment of consciousness NOS	Feb-09
26015	F255.00	391	0	15	0	Partial epilepsy without impairment of consciousness	Feb-09
27526	F255z00	126	0	4	0	Partial epilepsy without impairment of consciousness NOS	Feb-09
26733	F255y00	63	0	1	0	Partial epilepsy without impairment of consciousness OS	Feb-09
68651	7114200	4	0	0	0	Partial parathyroidectomy + parathyroid tissue transposition	Feb-09
43202	7114300	277	0	1	0	Partial parathyroidectomy NEC	Feb-09
101309	PJ02.11	5	0	0	0	Partial trisomy 21 in Down's syndrome	Jan-11
100174	PJ51z00	3	0	0	0	Partial trisomy syndrome NOS	Jul-10
19062	PJ51.00	38	0	0	0	Partial trisomy syndromes	Feb-09

For peer review only - http://bmjopen.bmj.com/site/about/guidelines.xhtml

50985	D402.16	5	0	0	0	Pelger - Huet anomaly	Feb-09
31444	C252.00	45	0	3	0	Pellagra	Feb-09
40319	N216211	125	0	2	0	Pellegrini - Stieda syndrome	Feb-09
58116	M145z00	82	0	1	0	Pemphigoid NOS	Feb-09
53763	M144z00	21	0	0	0	Pemphigus NOS	Feb-09
11892	C030.00	132	0	4	0	Pendred's syndrome	Feb-09
96277	G573400	247	0	2	0	Permanent atrial fibrillation	Apr-09
28901	F361.00	349	0	32	0	Peroneal muscular atrophy	Feb-09
70040	F361z00	10	0	0	0	Peroneal muscular atrophy NOS	Feb-09
2907	F250000	11582	0	1229	1	Petit mal (minor) epilepsy	Feb-09
15607	PK60.00	249	0	27	0	Peutz - Jegher's syndrome	Feb-09
3354	K274.00	24239	0	3840	0	Peyronie's disease	Feb-09
18231	B540.11	968	0	67	4	Phaeochromocytoma	Feb-09
52030	AB68.00	3	0	0	0	Phaeohyphomycosis	Feb-09
4318	C301.00	3036	0	3989	3906	Phenylketonuria	Feb-09
38071	13VM.00	844	0	16	0	Physical disability	Feb-09
3970	6665	1145	0	30	0	Physical handicap problem	Feb-09
46999	Z88..11	7	0	0	0	Physical health handicap	Feb-09
93788	Z77C.32	1	0	0	0	Physical health handicap	Feb-09
11136	F111.00	316	0	13	0	Pick's disease	Feb-09
59140	G532100	1	0	0	0	Pick's disease of heart	Feb-09
24755	C38y.11	152	0	14	0	Pickwickian syndrome	Feb-09
38294	C38y000	45	0	6	0	Pickwickian syndrome	Feb-09
4046	PG0C.00	816	0	16	0	Pierre - Robin syndrome	Feb-09
96210	PG0J.00	7	0	0	0	Pierre Robin association	Apr-09
95730	N321511	3	0	0	0	Pierson's disease	Feb-09
68146	C1zy000	5	0	1	0	Pineal gland dysfunction	Feb-09
39541	710..11	36	0	3	0	Pineal gland operations	Feb-09
2321	B7H2.11	6910	0	352	3	Pituitary adenoma	Feb-09
53682	C150200	36	0	0	0	Pituitary dependent Cushing's syndrome	Feb-09
12449	C13z.00	241	0	12	0	Pituitary disorders NOS	Feb-09

For peer review only - http://bmjopen.bmj.com/site/about/guidelines.xhtml

20287	C133.00	437	0	53	16	Pituitary dwarfism	Feb-09
61409	C133z00	2	0	0	0	Pituitary dwarfism NOS	Feb-09
36838	M163.11	23	0	0	0	Pityriasis circinata	Feb-09
107401	M162311	203	0	3	0	Pityriasis lichenoides chronica	Dec-13
107491	M162111	71	0	6	0	Pityriasis lichenoides et varioliformis acuta	Dec-13
36466	M15y200	172	0	3	0	Pityriasis rubra (Hebra)	Feb-09
48641	M165200	17	0	0	0	Pityriasis streptogenes	Feb-09
62323	G75..00	12	0	0	0	Polyarteritis nodosa and allied conditions	Feb-09
68136	G75z.00	128	0	0	0	Polyarteritis nodosa and allied conditions NOS	Feb-09
1670	N065z11	20288	0	1929	38	Polyarthritis	Feb-09
7454	N065.11	29871	0	2919	4	Polyarthropathy NEC	Feb-09
4504	PD1..13	1782	0	250	0	Polycystic kidney	Feb-09
4503	PD11.00	6901	0	595	3	Polycystic kidney disease	Feb-09
56852	PD11z00	226	0	8	0	Polycystic kidney disease NOS	Feb-09
4505	PD11100	482	0	30	0	"Polycystic kidneys, adult type"	Feb-09
44611	D410300	22	0	0	0	Polycythaemia due to cyanotic respiratory disease	Feb-09
68850	Q454000	4	0	0	0	Polycythaemia due to donor twin transfusion	Feb-09
102672	5A14.00	0	0	0	1	Polycythaemia irradiation	Jul-11
64182	Q454z00	27	0	0	0	Polycythaemia neonatorum NOS	Feb-09
5542	B934.11	2103	0	183	0	Polycythaemia rubra vera	Feb-09
40691	PF0z.00	136	0	0	0	Polydactyly NOS	Feb-09
102958	C320600	75	0	0	0	Polygenic hypercholesterolaemia	Sep-11
39898	C18..00	32	0	0	0	Polyglandular dysfunction and related disorders	Feb-09
47995	C18z.00	10	0	0	0	Polyglandular dysfunction NOS	Feb-09
92901	C183.00	1	0	0	0	Polyglandular hyperfunction	Feb-09
1408	N20..00	300629	0	6946	44	Polymyalgia rheumatica	Feb-09

For peer review only - http://bmjopen.bmj.com/site/about/guidelines.xhtml

15511	N004.00	3141	0	178	1	Polymyositis	Feb-09
93927	N231400	44	0	0	0	Polymyositis ossificans	Feb-09
28294	F326100	80	0	5	0	Polyneuritis cranialis	Feb-09
24222	F376.00	61	0	2	0	Polyneuropathy due to drugs	Feb-09
66336	F374000	15	0	1	0	Polyneuropathy in amyloidosis	Feb-09
73337	F374100	1	0	0	0	Polyneuropathy in beriberi	Feb-09
57313	F371.00	8	0	0	0	Polyneuropathy in collagen vascular disease	Feb-09
71258	F371z00	4	0	0	0	Polyneuropathy in collagen vascular disease NOS	Feb-09
31790	F372.00	1506	0	33	0	Polyneuropathy in diabetes	Feb-09
52089	F374300	1	0	0	0	Polyneuropathy in diphtheria	Feb-09
56272	F374.00	16	0	2	0	Polyneuropathy in disease EC	Feb-09
63555	F374z00	28	0	2	0	Polyneuropathy in disease NOS	Feb-09
44095	F371000	37	0	4	0	Polyneuropathy in disseminated lupus erythematosus	Feb-09
39692	F374400	57	0	0	0	Polyneuropathy in herpes zoster	Feb-09
68960	F374500	6	0	0	0	Polyneuropathy in hypoglycaemia	Feb-09
30537	F373.00	15	0	0	0	Polyneuropathy in malignant disease	Feb-09
100064	F374600	1	0	0	0	Polyneuropathy in mumps	Jul-10
47465	F371100	5	0	0	0	Polyneuropathy in polyarteritis nodosa	Feb-09
58758	F374800	25	0	1	0	Polyneuropathy in porphyria	Feb-09
62401	F371200	21	0	0	0	Polyneuropathy in rheumatoid arthritis	Feb-09
40751	F374900	16	0	0	0	Polyneuropathy in sarcoidosis	Feb-09
24355	F374200	9	0	0	0	Polyneuropathy in vitamin B deficiency	Feb-09
24226	F37z.11	219	0	9	0	Polyneuropathy unspecified	Feb-09
30600	PG54.00	168	0	8	0	Polyostotic fibrous dysplasia	Feb-09
93879	P41..11	10	0	0	0	Polyotia	Feb-09

For peer review only - http://bmjopen.bmj.com/site/about/guidelines.xhtml

38836	PF15.00	55	0	0	0	Polysyndactyly	Feb-09
68991	PF1z.11	1	0	0	0	Polysyndactyly	Feb-09
43743	C371z00	162	0	1	0	Porphyria NOS	Feb-09
47257	J615.11	14	0	0	0	Portal cirrhosis	Feb-09
55454	J615y00	18	0	0	0	Portal cirrhosis unspecified	Feb-09
40963	J61y300	35	0	0	0	Portal fibrosis without cirrhosis	Feb-09
5129	J623.00	4307	0	65	0	Portal hypertension	Feb-09
44881	C137100	36	0	2	0	Post-hypophysectomy hypopituitarism	Feb-09
25424	C1z1011	79	0	22	0	Precocious pubarche	Feb-09
3031	C1z1000	3154	0	645	0	Precocious puberty	Feb-09
15995	C1z1.11	558	0	86	1	Precocious puberty	Feb-09
70360	C152912	3	0	0	0	Precocious puberty with adrenal hyperplasia	Feb-09
22704	C1z1111	873	0	34	0	Precocious thelarche	Feb-09
50665	C391000	40	0	4	0	Predominantly T-cell immuno-deficiency NOS	Feb-09
106756	P711.13	1	0	0	0	Preductal aortic stenosis	Aug-13
72295	P711.00	2	0	0	0	Preductal coarctation of aorta	Feb-09
53970	C373700	26	0	0	0	Primary amyloidosis NEC	Feb-09
5638	J616000	6062	0	300	0	Primary biliary cirrhosis	Feb-09
29028	C151000	247	0	4	0	Primary hyperaldosteronism	Feb-09
18740	C120000	3636	0	136	2	Primary hyperparathyroidism	Feb-09
107704	G20..12	24	0	0	0	Primary hypertension	Feb-14
45133	E312.00	106	0	2	0	Profound mental retardation with IQ less than 20	Feb-09
107607	F591B00	50	0	0	0	Profound sensorineural hearing loss	Jan-14
52673	A413.00	66	0	5	0	Progressive multifocal leucoencephalopathy	Feb-09
49541	A413.11	26	0	1	0	Progressive multifocal leukoencephalopathy	Feb-09
37644	F132100	100	0	1	0	Progressive myoclonic epilepsy	Feb-09

For peer review only - http://bmjopen.bmj.com/site/about/guidelines.xhtml

44058	F427811	60	0	1	0	Progressive rod dystrophy	Feb-09
40553	F130400	72	0	2	0	Progressive supranuclear ophthalmoplegia	Feb-09
9385	F24y000	1339	0	59	0	Progressive supranuclear palsy	Feb-09
44141	N001000	47	0	1	0	Progressive systemic sclerosis	Feb-09
52426	C134000	54	0	5	0	Prolactin deficiency	Feb-09
26102	D30B.00	598	0	25	0	Protein C deficiency	Feb-09
8479	D309.00	1037	0	52	0	Protein S deficiency	Feb-09
22853	PG72.00	118	0	4	0	Prune belly syndrome	Feb-09
46749	C377111	12	0	1	0	Pseudo - Hurler's disease	Feb-09
43115	PG44600	34	0	0	0	Pseudoachondroplasia	Feb-09
60703	ZS42111	15	0	1	0	Pseudobulbar palsy type of dysarthria	Feb-09
2337	D41y200	228	0	14	7	Pseudocholinesterase deficiency	Feb-09
36351	PG42.17	4	0	0	0	Pseudochondroplasia	Feb-09
48532	PC7z100	7	0	0	0	Pseudohermaphrodite NOS	Feb-09
93135	C152814	2	0	0	0	"Pseudohermaphrodite, male with adrenocortical disorder"	Feb-09
26210	F391011	7	0	0	0	Pseudohypertrophic dystrophy	Feb-09
23540	C354300	199	0	6	0	Pseudohypoparathyroidism	Feb-09
49810	F280200	4	0	0	0	Pseudoporencephaly	Feb-09
65126	C371600	54	0	1	0	Pseudoporphyria	Feb-09
60587	C354311	9	0	2	0	Pseudopseudohypoparathyroidism	Feb-09
52610	F4E3411	50	0	3	0	Pseudoptosis	Feb-09
66964	F426500	8	0	0	0	Pseudoretinitis pigmentosa	Feb-09
65405	N237300	6	0	0	0	Pseudosarcomatous fibromatosis	Feb-09
49468	A57y400	21	0	1	0	Pseudoscarlatina	Feb-09
22632	F4J4600	697	0	3	0	Pseudostrabismus	Feb-09
68982	P603.11	2	0	0	0	Pseudotruncus arteriosus	Feb-09
46041	F282.11	43	0	2	0	Pseudotumour cerebri	Feb-09
24805	F4G1111	98	0	3	0	Pseudotumour of orbit	Feb-09
30272	M161200	41	0	0	0	Psoriasis circinata	Feb-09

For peer review only - http://bmjopen.bmj.com/site/about/guidelines.xhtml

42008	M161300	82	0	2	0	Psoriasis diffusa	Feb-09
21633	M161500	19	0	0	0	Psoriasis geographica	Feb-09
65839	M161700	5	0	0	0	Psoriasis gyrata	Feb-09
48257	M161800	11	0	0	0	Psoriasis inveterata	Feb-09
172	M161z00	348556	0	12706	0	Psoriasis NOS	Feb-09
60169	M161900	2	0	0	0	Psoriasis ostracea	Feb-09
24136	M161C00	63	0	2	0	Psoriasis punctata	Feb-09
26368	M160000	191	0	4	0	Psoriasis spondylitica	Feb-09
162	M161000	386002	0	22325	19	Psoriasis unspecified	Feb-09
30210	M161F00	904	0	15	0	Psoriasis vulgaris	Feb-09
96880	M160.11	6793	0	225	0	Psoriatic arthritis	May-09
476	M160.00	37950	0	2913	1	Psoriatic arthropathy	Feb-09
12500	M160z00	1575	0	107	0	Psoriatic arthropathy NOS	Feb-09
23634	F254100	97	0	6	0	Psychomotor epilepsy	Feb-09
37948	1P01.00	928	0	7	0	Psychomotor retardation	Feb-09
36203	F254200	8	0	1	0	Psychosensory epilepsy	Feb-09
43225	E14..00	13	0	1	0	Psychoses with origin in childhood	Feb-09
96225	P736.00	4	0	0	0	Pulmonary arterio-venous aneurysm	Apr-09
47853	P736.11	16	0	0	0	Pulmonary arterio-venous fistula	Feb-09
37807	G42y000	8	0	1	0	Pulmonary arteritis	Feb-09
67928	P731.00	2	0	0	0	Pulmonary artery agenesis	Feb-09
65029	P737.00	18	0	0	0	Pulmonary artery aneurysm	Feb-09
42127	P73z.00	31	0	0	0	Pulmonary artery anomaly NOS	Feb-09
63187	P730.00	9	0	0	0	"Pulmonary artery anomaly, unspecified"	Feb-09
7180	G4...00	230	0	10	0	Pulmonary circulation diseases	Feb-09
30214	H54..00	280	0	17	0	Pulmonary congestion and hypostasis	Feb-09
61229	H54z.00	2	0	0	0	Pulmonary congestion and hypostasis NOS	Feb-09
103472	H563200	4735	0	172	0	Pulmonary fibrosis	Dec-11

For peer review only - http://bmjopen.bmj.com/site/about/guidelines.xhtml

For peer review only

46736	G543200	136	0	3	0	"Pulmonary incompetence, cause unspecified"	Feb-09
23608	G543000	139	0	3	0	"Pulmonary incompetence, non-rheumatic"	Feb-09
9401	P6y2.00	1595	0	74	0	Pulmonary infundibular stenosis	Feb-09
93880	H585000	1	0	0	0	Pulmonary insufficiency following shock	Feb-09
72221	H585100	1	0	0	0	Pulmonary insufficiency following surgery	Feb-09
96754	H585200	3	0	0	0	Pulmonary insufficiency following trauma	Apr-09
38299	G543213	17	0	2	0	"Pulmonary insufficiency, cause unspecified"	Feb-09
15496	G543012	609	0	8	0	"Pulmonary regurgitation, non-rheumatic"	Feb-09
3859	H57y200	2945	0	121	0	Pulmonary sarcoidosis	Feb-09
2669	G543300	3149	0	74	1	"Pulmonary stenosis, cause unspecified"	Feb-09
61878	G543311	107	0	1	0	"Pulmonary stenosis, cause unspecified"	Feb-09
14723	G543100	1408	0	68	0	"Pulmonary stenosis, non-rheumatic"	Feb-09
39992	P60..00	201	0	2	0	Pulmonary valve anomalies	Feb-09
69940	P600.00	22	0	0	0	"Pulmonary valve anomaly, unspecified"	Feb-09
12312	G543.00	632	0	14	0	Pulmonary valve disorders	Feb-09
34932	G543400	225	0	2	0	Pulmonary valve stenosis with insufficiency	Feb-09
53944	K10y.00	128	0	0	0	Pyelonephritis and pyonephrosis unspecified	Feb-09
95710	K10y300	6	0	0	0	Pyelonephritis in diseases EC	Feb-09

For peer review only - http://bmjopen.bmj.com/site/about/guidelines.xhtml

60856	K0A2700	4	0	0	0	Recur+persist haematuria difus crescentic glomerulonephritis	Feb-09
61317	K0A2200	4	0	0	0	Recur+persist haematuria difus membranous glomerulonephritis	Feb-09
68364	K0A2100	2	0	0	0	"Recur+persist haematuria, focal+segmental glomerular lesions"	Feb-09
49642	K0A2300	11	0	0	0	Recur+persist haemuria df mesangial prolif glomerulnephritis	Feb-09
60484	K0A2500	3	0	0	0	Recur+persist hmuria df mesangiocapilary glomerulonephritis	Feb-09
53480	J614300	25	0	0	0	Recurrent hepatitis	Feb-09
25563	E113z00	1910	0	10	0	Recurrent major depressive episode NOS	Feb-09
55384	E113600	518	0	1	0	"Recurrent major depressive episodes, in full remission"	Feb-09
29342	E113100	1615	0	25	0	"Recurrent major depressive episodes, mild"	Feb-09
25697	E113300	1195	0	32	0	"Recurrent major depressive episodes, severe, no psychosis"	Feb-09
24171	E113400	493	0	9	0	"Recurrent major depressive episodes, severe, with psychosis"	Feb-09
35671	E113000	506	0	12	0	"Recurrent major depressive episodes, unspecified"	Feb-09
56273	E113500	226	0	9	0	"Recurrent major depressive episodes,partial/unspec remission"	Feb-09
46415	E111z00	94	0	1	0	Recurrent manic episode NOS	Feb-09
26227	E111.00	215	0	6	0	Recurrent manic episodes	Feb-09
37178	E111600	125	0	1	0	"Recurrent manic episodes, in full remission"	Feb-09
46425	E111100	20	0	0	0	"Recurrent manic episodes, mild"	Feb-09

For peer review only - http://bmjopen.bmj.com/site/about/guidelines.xhtml

27739	E111200	24	0	0	0	"Recurrent manic episodes, moderate"	Feb-09
58863	E111500	2	0	0	0	"Recurrent manic episodes, partial or unspecified remission"	Feb-09
65811	E111300	20	0	0	0	"Recurrent manic episodes, severe without mention psychosis"	Feb-09
32295	E111400	25	0	0	0	"Recurrent manic episodes, severe, with psychosis"	Feb-09
22003	G54z013	93	0	0	0	Regurgitation of unspecified heart valve	Feb-09
8103	A993.00	2493	0	96	0	Reiter's disease / syndrome	Feb-09
2194	A993.11	1829	0	153	7	Reiter's syndrome	Feb-09
45904	K0B2.00	2	0	0	0	Ren tub-interst disordr/blood dis+disordr inv immune mech	Feb-09
67261	K0B4.00	3	0	0	0	Ren tub-interstitl disordr/systemc connectv tiss disorder	Feb-09
48475	K08yz11	2	0	0	0	Renal acidaemia	Feb-09
30097	PD0..00	245	0	9	0	Renal agenesis and dysgenesis	Feb-09
29659	PD0z.00	84	0	6	0	Renal agenesis or dysgenesis NOS	Feb-09
27474	PD00.00	158	0	3	0	"Renal agenesis, unspecified"	Feb-09
55985	PD00z00	17	0	0	0	"Renal agenesis, unspecified NOS"	Feb-09
107765	K0J..00	4	0	0	0	Renal disorders in systemic disease	Mar-14
32963	PD30.12	17	0	0	0	Renal duplication NEC	Feb-09
34648	K080100	4	0	0	0	Renal dwarfism	Feb-09
105657	PD04200	6	0	2	0	Renal dysplasia and retinal aplasia	Jan-13
350	K06..00	38962	0	4706	59	Renal failure unspecified	Feb-09
106860	C353600	4	0	0	0	Renal failure-associated hyperphosphataemia	Aug-13
22876	K071.00	50	0	1	0	Renal fibrosis	Feb-09
41013	K08y300	15	0	0	0	Renal function impairment with growth failure	Feb-09
29638	K080.00	207	0	3	0	Renal osteodystrophy	Feb-09

For peer review only - http://bmjopen.bmj.com/site/about/guidelines.xhtml

34637	K080z00	49	0	0	0	Renal osteodystrophy NOS	Feb-09
44894	D410400	26	0	0	0	Renal polycythaemia	Feb-09
45876	F421200	14	0	2	0	Renal retinopathy	Feb-09
66062	K080300	15	0	0	0	Renal rickets	Feb-09
4480	K07z.00	46	0	2	1	Renal sclerosis NOS	Feb-09
26220	K07..00	59	0	4	0	Renal sclerosis unspecified	Feb-09
110983	K08y412	1	0	0	0	Renal tubular acidaemia	Jul-16
5072	K08y400	651	0	18	0	Renal tubular acidosis	Feb-09
41148	K0B4000	10	0	1	0	Renal tubulo-interstitial disorder in SLE	Feb-09
64622	K0B1.00	4	0	0	0	Renal tubulo-interstitial disorder/ neoplastic diseases	Feb-09
45523	K0B..00	26	0	1	0	Renal tubulo-interstitial disorders in diseases EC	Feb-09
48057	K0B5.00	21	0	0	0	Renal tubulo-interstitial disordrs in transplant rejectn	Feb-09
35157	G770.11	150	0	15	0	Rendu - Osler - Weber disease	Feb-09
25249	H59..00	2542	0	25	0	Respiratory failure	Feb-09
39079	Q319.00	49	0	0	0	Respiratory failure of newborn	Feb-09
46397	P8z..00	33	0	0	0	Respiratory system anomaly NOS	Feb-09
46398	P8...00	38	0	4	0	Respiratory system congenital anomalies	Feb-09
2745	F427600	6052	0	711	2	Retinitis pigmentosa	Feb-09
39637	F421A11	76	0	7	0	Retinitis proliferans	Feb-09
23896	J041.12	144	0	1	0	Retrognathism	Feb-09
26364	J041z00	16	0	0	0	Retrognathism NOS	Feb-09
32211	G121.00	135	0	8	0	Rheumatic aortic insufficiency	Feb-09
9391	G120.00	703	0	26	0	Rheumatic aortic stenosis	Feb-09
63960	G122.00	31	0	2	0	Rheumatic aortic stenosis with insufficiency	Feb-09
50809	G12z.00	54	0	1	0	Rheumatic aortic valve disease NOS	Feb-09
72936	G02z.00	12	0	0	0	Rheumatic chorea NOS	Feb-09

For peer review only - http://bmjopen.bmj.com/site/about/guidelines.xhtml

69995	G020.00	10	0	0	0	Rheumatic chorea with heart involvement	Feb-09
63252	G021.00	9	0	0	0	Rheumatic chorea without mention of heart involvement	Feb-09
100820	66HC.00	9806	0	5	0	Rheumatic disorder annual review invitation	Nov-10
44756	G01..00	119	0	0	0	Rheumatic fever with heart involvement	Feb-09
48189	G00..00	153	0	1	0	Rheumatic fever without heart involvement	Feb-09
107462	GA...00	47	0	2	0	Rheumatic heart disease	Dec-13
53878	G1yz000	101	0	0	0	Rheumatic heart disease unspecified	Feb-09
22262	G1yz100	149	0	11	0	Rheumatic left ventricular failure	Feb-09
51879	G111.00	208	0	19	0	Rheumatic mitral insufficiency	Feb-09
32435	G110.11	223	0	5	0	Rheumatic mitral stenosis	Feb-09
62404	G1y0.00	8	0	0	0	Rheumatic myocarditis	Feb-09
64799	H571.00	8	0	0	0	Rheumatic pneumonia	Feb-09
54088	G141100	2	0	0	0	Rheumatic pulmonary insufficiency	Feb-09
62207	G141000	14	0	0	0	Rheumatic pulmonary stenosis	Feb-09
105626	G141200	1	0	0	0	Rheumatic pulmonary stenosis and insufficiency	Jan-13
44167	G141.00	8	0	0	0	Rheumatic pulmonary valve disease	Feb-09
36768	G141z00	2	0	0	0	Rheumatic pulmonary valve disease NOS	Feb-09
62875	N135100	8	0	0	0	Rheumatic torticollis	Feb-09
60266	G140100	24	0	0	0	Rheumatic tricuspid insufficiency	Feb-09
31505	G140000	5	0	0	0	Rheumatic tricuspid stenosis	Feb-09
62186	G14021Y	1	0	0	0	Rheumatic tricuspid stenosis and incompetence	Feb-09
93114	G140200	1	0	0	0	Rheumatic tricuspid stenosis and insufficiency	Feb-09

For peer review only - http://bmjopen.bmj.com/site/about/guidelines.xhtml

93113	G14021X	7	0	0	0	Rheumatic tricuspid stenosis and regurgitation	Feb-09
72613	G140z00	4	0	0	0	Rheumatic tricuspid valve disease NOS	Feb-09
59275	G14z.11	1	0	0	0	"Rheumatic valvulitis, chronic NOS"	Feb-09
67955	A985z11	1	0	0	0	Rheumatism - gonococcal	Feb-09
33474	N240.00	1221	0	1	0	Rheumatism and fibrositis unspecified	Feb-09
35937	N240z00	1287	0	0	0	Rheumatism or fibrositis NOS	Feb-09
844	N040.00	262585	0	21625	2747	Rheumatoid arthritis	Feb-09
31054	N040S00	380	0	22	0	Rheumatoid arthritis - multiple joint	Feb-09
27603	N04..00	1821	0	30	0	Rheumatoid arthritis and other inflammatory polyarthropathy	Feb-09
105507	66HB000	78562	0	249	0	Rheumatoid arthritis annual review	Dec-12
107340	9mM..00	486	0	1	0	Rheumatoid arthritis monitoring invitation	Dec-13
107435	9mM0.00	8104	0	1	0	Rheumatoid arthritis monitoring invitation first letter	Dec-13
107575	9mM1.00	3203	0	1	0	Rheumatoid arthritis monitoring invitation second letter	Jan-14
107676	9mM2.00	1666	0	0	0	Rheumatoid arthritis monitoring invitation third letter	Feb-14
107797	9mM4.00	482	0	0	0	Rheumatoid arthritis monitoring telephone invitation	Mar-14
107606	9mM3.00	103	0	0	0	Rheumatoid arthritis monitoring verbal invitation	Jan-14
51238	N040K00	16	0	0	0	Rheumatoid arthritis of 1st MTP joint	Feb-09
100914	N040400	3	0	0	0	Rheumatoid arthritis of acromioclavicular joint	Nov-10
51239	N040F00	68	0	1	0	Rheumatoid arthritis of ankle	Feb-09

For peer review only - http://bmjopen.bmj.com/site/about/guidelines.xhtml

44743	N040000	21	0	0	0	Rheumatoid arthritis of cervical spine	Feb-09
63198	N040A00	18	0	0	0	Rheumatoid arthritis of DIP joint of finger	Feb-09
63365	N040600	4	0	0	0	Rheumatoid arthritis of distal radio-ulnar joint	Feb-09
59738	N040500	34	0	0	0	Rheumatoid arthritis of elbow	Feb-09
49067	N040B00	34	0	1	0	Rheumatoid arthritis of hip	Feb-09
107112	N040M00	6	0	0	0	Rheumatoid arthritis of IP joint of toe	Oct-13
50863	N040D00	151	0	3	0	Rheumatoid arthritis of knee	Feb-09
99414	N040L00	4	0	0	0	Rheumatoid arthritis of lesser MTP joint	May-10
42299	N040800	75	0	3	0	Rheumatoid arthritis of MCP joint	Feb-09
71784	N040J00	6	0	0	0	Rheumatoid arthritis of other tarsal joint	Feb-09
41941	N040900	47	0	1	0	Rheumatoid arthritis of PIP joint of finger	Feb-09
100776	N040C00	4	0	0	0	Rheumatoid arthritis of sacro-iliac joint	Nov-10
107963	N040300	1	0	0	0	Rheumatoid arthritis of sternoclavicular joint	Apr-14
73619	N040G00	9	0	0	0	Rheumatoid arthritis of subtalar joint	Feb-09
70658	N040H00	7	0	0	0	Rheumatoid arthritis of talonavicular joint	Feb-09
107791	N040E00	1	0	0	0	Rheumatoid arthritis of tibio-fibular joint	Mar-14
48832	N040700	113	0	0	0	Rheumatoid arthritis of wrist	Feb-09
37431	N042z00	39	0	1	0	Rheumatoid arthropathy + visceral/systemic involvement NOS	Feb-09
43816	G5yA.00	26	0	0	0	Rheumatoid carditis	Feb-09

For peer review only

For peer review only - http://bmjopen.bmj.com/site/about/guidelines.xhtml

31724	N04y000	109	0	11	0	Rheumatoid lung	Feb-09
46436	N042100	33	0	1	0	Rheumatoid lung disease	Feb-09
49787	G5y8.00	6	0	0	0	Rheumatoid myocarditis	Feb-09
30548	N040N00	225	0	3	0	Rheumatoid vasculitis	Feb-09
33375	C260.11	13	0	3	2	Riboflavin deficiency	Feb-09
5074	C28..12	2187	0	148	0	Rickets	Feb-09
51768	P342200	41	0	2	0	Rieger's anomaly	Feb-09
57069	G565200	7	0	1	0	Right BBB with left anterior fascicular block	Feb-09
98675	G565100	2	0	0	0	Right BBB with left posterior fascicular block	Mar-10
10079	G580.12	1165	0	40	0	Right heart failure	Feb-09
3293	F223.00	2081	0	124	1	Right hemiplegia	Feb-09
21851	P603.00	52	0	0	0	Right hypoplastic heart syndrome	Feb-09
98751	G5y3600	98	0	1	0	Right ventricular dilatation	Mar-10
104275	G584.00	169	0	1	0	Right ventricular failure	Jun-12
104266	P6y3100	36	0	0	0	Right ventricular outflow tract obstruction	Jun-12
108180	G5yyE00	70	0	0	0	Right ventricular systolic dysfunction	Jun-14
31616	P2x5.00	9	0	0	0	Riley - Day syndrome	Feb-09
22691	G56y200	51	0	0	0	Romano - Ward syndrome	Feb-09
40502	PH33300	46	0	1	0	Rothmund-Thomson syndrome	Feb-09
46507	C374300	65	0	4	0	Rotor syndrome	Feb-09
109176	F143.11	1	0	0	0	Roussy-Levy syndrome	Jan-15
104100	PKy7311	15	0	0	0	Rubinstein-Taybi syndrome	May-12
32868	PKy6200	316	0	15	0	Russell - Silver syndrome	Feb-09
72928	P103400	1	0	0	0	Sacral spina bifida with hydrocephalus - closed	Feb-09
70923	P118400	9	0	0	0	Sacral spina bifida without hydrocephalus - closed	Feb-09

For peer review only - http://bmjopen.bmj.com/site/about/guidelines.xhtml

71525	P117400	3	0	0	0	Sacral spina bifida without hydrocephalus - open	Feb-09
57986	PF55300	91	0	2	0	Saethre-Chotzen syndrome	Feb-09
109634	E121.11	1	0	0	0	Sander's disease	May-15
44111	J34y.11	52	0	2	0	Sandifer's syndrome	Feb-09
111807	F142100	2	0	0	0	Sanger-Brown cerebellar ataxia	May-17
40613	AD55.00	59	0	2	0	Sarcoid arthropathy	Feb-09
47037	G558300	61	0	2	0	Sarcoid heart disease	Feb-09
34437	G5y7.00	13	0	1	0	Sarcoid myocarditis	Feb-09
3865	AD5..00	29294	0	1573	7	Sarcoidosis	Feb-09
72595	AD54.00	32	0	0	0	Sarcoidosis of inferior turbinates	Feb-09
33980	AD50.00	389	0	6	0	Sarcoidosis of lung	Feb-09
58841	AD52.00	43	0	3	0	Sarcoidosis of lung with sarcoidosis of lymph nodes	Feb-09
49075	AD51.00	81	0	2	0	Sarcoidosis of lymph nodes	Feb-09
27769	AD53.00	243	0	12	0	Sarcoidosis of skin	Feb-09
111668	H32yz11	1	0	0	0	Sawyer - Jones syndrome	Feb-17
69375	C375113	5	0	0	0	Scheie's syndrome	Feb-09
1721	N320100	7224	0	306	1	Scheuermann's disease	Feb-09
56438	E107500	211	0	0	0	Schizo-affective schizophrenia in remission	Feb-09
67943	13Y2.00	11	0	0	0	Schizophrenia association member	Feb-09
58687	E100500	1296	0	0	0	Schizophrenia in remission	Feb-09
8407	E10z.00	11518	0	194	0	Schizophrenia NOS	Feb-09
73295	E100.11	4	0	0	0	Schizophrenia simplex	Feb-09
31950	13L3.12	48	0	0	0	Schizophrenic child	Feb-09
854	E10..00	59295	0	3962	7	Schizophrenic disorders	Feb-09
56692	PG45.12	7	0	0	0	Schmid's metaphyseal dysostosis	Feb-09
48130	C181.12	32	0	1	0	Schmidt's syndrome	Feb-09
36606	P74z600	68	0	3	0	Scimitar syndrome	Feb-09
5318	F4K0.12	7606	0	578	0	Scleritis	Feb-09
15996	F4K0.00	3226	0	76	0	Scleritis and episcleritis	Feb-09

For peer review only - http://bmjopen.bmj.com/site/about/guidelines.xhtml

44859	F4K0z00	557	0	2	0	Scleritis or episcleritis NOS	Feb-09
37538	M210500	123	0	4	0	Sclerodactyly	Feb-09
3670	N001.00	6495	0	419	0	Scleroderma	Feb-09
37665	F4K0500	72	0	0	0	Sclerokeratitis	Feb-09
44791	J661900	142	0	1	0	Sclerosing cholangitis unspecified	Feb-09
40041	N374300	1006	0	40	0	Scoliosis associated with other condition	Feb-09
43192	N374D00	7	0	0	0	Scoliosis in connective tissue anomalies	Feb-09
67445	N374C00	19	0	0	0	Scoliosis in neurofibromatosis	Feb-09
64218	N374A00	29	0	1	0	Scoliosis in skeletal dysplasia	Feb-09
53972	N373900	50	0	0	0	Scoliosis secondary to other treatment	Feb-09
34719	PKy6400	10	0	0	0	Seckel syndrome	Feb-09
22487	C10N.00	146	0	5	0	Secondary diabetes mellitus	Feb-09
94383	C10N000	8	0	0	0	Secondary diabetes mellitus without complication	Feb-09
37119	C151100	24	0	1	0	Secondary hyperaldosteronism	Feb-09
70714	C354111	73	0	0	0	Secondary hypercalcaemia	Feb-09
7329	G24..00	2762	0	20	0	Secondary hypertension	Feb-09
42229	G24zz00	162	0	0	0	Secondary hypertension NOS	Feb-09
51697	C10G.00	339	0	4	0	Secondary pancreatic diabetes mellitus	Feb-09
96506	C10G000	2	0	0	0	Secondary pancreatic diabetes mellitus without complication	Apr-09
34065	G41y000	1476	0	10	0	Secondary pulmonary hypertension	Feb-09
65502	D201z14	8	0	0	0	Secondary red cell aplasia NEC	Feb-09
31774	D201z13	7	0	0	0	Secondary red cell hypoplasia NEC	Feb-09
35667	J661800	50	0	2	0	Secondary sclerosing cholangitis	Feb-09
31550	D210300	7	0	0	0	Secondary sideroblastic anaemia due to disease	Feb-09

For peer review only - http://bmjopen.bmj.com/site/about/guidelines.xhtml

47225	D210400	5	0	0	0	Secondary sideroblastic anaemia due to drugs and toxins	Feb-09
97143	F13C.00	13	0	0	0	Segawa syndrome	Aug-09
13304	Q480.12	1116	0	20	0	Seizures in newborn	Feb-09
8548	C390100	796	0	35	3	Selective IgA immunodeficiency	Feb-09
18700	C390300	122	0	3	3	Selective IgG immunodeficiency	Feb-09
18701	C390200	41	0	5	0	Selective IgM immunodeficiency	Feb-09
536	F591.00	65546	0	1344	0	Sensorineural hearing loss	Feb-09
10112	F591600	8679	0	79	0	"Sensorineural hearing loss, bilateral"	Feb-09
29191	F591700	281	0	38	0	"Sensorineurl hear loss,unilat unrestrict hear/contralat side"	Feb-09
3366	663V300	2734	0	66	0	Severe asthma	Feb-09
233	H33z011	25653	0	4132	4	Severe asthma attack	Feb-09
110345	38B8.00	37	0	0	0	Severe asthma exacerbation risk assessment	Dec-15
15082	Q215.00	1394	0	28	5	Severe birth asphyxia - apgar score less than 4 at 1 minute	Feb-09
98734	F286200	12	0	0	0	Severe chronic fatigue syndrome	Mar-10
107402	28E2.00	149	0	1	0	Severe cognitive impairment	Dec-13
108409	F25G.00	5	0	0	0	Severe myoclonic epilepsy in infancy	Jul-14
107610	F591E00	47	0	1	0	Severe sensorineural hearing loss	Jan-14
4376	PJX..00	25	0	0	0	"Sex chromosome abnormality, male phenotype, unspecified"	Feb-09
34667	PJyz.00	65	0	9	2	Sex chromosome anomaly NOS	Feb-09
55119	PJy1.00	17	0	0	0	Sex chromosome mosaicism	Feb-09
65983	PJy1z00	1	0	0	0	Sex chromosome mosaicism NOS	Feb-09
35014	B622.00	77	0	7	0	Sezary's disease	Feb-09
100532	B622z00	3	0	0	0	Sezary's disease NOS	Aug-10
12841	PJ3y000	42	0	0	0	Shprintzen syndrome	Feb-09
62041	PJ53500	21	0	2	0	Shwachman-Diamond syndrome	Feb-09

For peer review only - http://bmjopen.bmj.com/site/about/guidelines.xhtml

35839	F130500	90	0	17	0	Shy-Drager syndrome	Feb-09
2360	N002.00	12690	0	576	1	Sicca (Sjogren's) syndrome	Feb-09
104288	1X0..00	6	0	0	0	Sick Cell Thalas Scr Prog fam orig African or African-Carib	Jun-12
103055	1X1..00	23	0	0	0	Sick Cell Thalas Scr Prog fam orig South Asia (Asian)	Oct-11
107027	K0G..00	5	0	0	0	Sickle cell nephropathy	Sep-13
23519	D106.00	4532	0	489	166	Sickle-cell anaemia	Feb-09
57397	D106z00	75	0	2	0	Sickle-cell anaemia NOS	Feb-09
69964	D106000	14	0	1	0	Sickle-cell anaemia of unspecified type	Feb-09
31370	D106200	1244	0	26	0	Sickle-cell anaemia with crisis	Feb-09
31306	D106300	109	0	8	1	Sickle-cell anaemia with haemoglobin C disease	Feb-09
8119	D106400	55	0	1	1	Sickle-cell anaemia with haemoglobin D disease	Feb-09
93872	D106500	20	0	0	2	Sickle-cell anaemia with haemoglobin E disease	Feb-09
32937	D106100	19	0	1	0	Sickle-cell anaemia with no crisis	Feb-09
31075	D104211	360	0	125	100	Sickle-cell thalassaemia	Feb-09
3616	D105.00	13001	0	1063	741	Sickle-cell trait	Feb-09
99476	PKy5H00	8	0	0	0	Simpson-Golabi-Behmel syndrome	May-10
107846	P240000	1	0	0	0	Single congenital cerebral cyst	Mar-14
62163	P6y4100	26	0	0	0	Single coronary artery	Feb-09
18437	G566000	157	0	7	0	Sinoatrial block	Feb-09
426	G577.00	2593	0	109	1	Sinus arrhythmia	Feb-09
31717	P20..15	22	0	2	0	Sinus pericranii	Feb-09
43560	PK31.00	21	0	1	0	Situs inversus abdominalis	Feb-09
23513	PK3z.00	39	0	8	0	Situs inversus NOS	Feb-09
63861	PK32.00	2	0	0	0	Situs inversus thoracis	Feb-09
110797	PK34.00	1	0	0	0	Situs inversus with levocardia	May-16
72246	PK30.00	6	0	0	0	"Situs inversus, unspecified"	Feb-09

For peer review only - http://bmjopen.bmj.com/site/about/guidelines.xhtml

11632	PH12.11	588	0	19	0	Sjogren - Larsson syndrome	Feb-09
7603	Fy03.00	16408	0	2567	0	Sleep apnoea	Feb-09
23779	H5B..00	2863	0	276	0	Sleep apnoea	Feb-09
60083	PB1..00	9	0	0	0	Small intestine atresia and stenosis	Feb-09
70579	PB10z00	2	0	0	0	Small intestine atresia NOS	Feb-09
25042	PB1z.00	10	0	0	0	Small intestine atresia or stenosis NOS	Feb-09
36871	PJ33300	87	0	4	0	Smith-Magenis syndrome	Feb-09
36314	C1zy211	222	0	13	0	Sotos syndrome	Feb-09
25570	F23y200	221	0	2	0	Spastic cerebral palsy	Feb-09
104775	F230111	49	0	1	0	Spastic diplegic cerebral palsy	Aug-12
59254	ZS42100	32	0	1	0	Spastic dysarthria	Feb-09
105133	F2B1.00	21	0	0	0	Spastic hemiplegic cerebral palsy	Nov-12
9375	F241100	1317	0	48	1	Spastic paraplegia	Feb-09
104580	F2B0.00	101	0	1	0	Spastic quadriplegic cerebral palsy	Jul-12
25990	ZPA2100	992	0	8	0	Special educational needs	Feb-09
22896	ZS...00	1425	0	12	0	Speech and language disorder	Feb-09
43892	ZS5..00	47	0	1	0	Speech and language dyspraxias	Feb-09
28845	ZS6..00	94	0	13	0	Speech and phonology impairments	Feb-09
3417	E2F3.12	6785	0	1109	0	Speech development disorder	Feb-09
110439	1B96.00	40	0	5	0	Speech impairment	Jan-16
3567	E2F3.00	3146	0	44	0	Speech or language developmental disorder	Feb-09
1277	E2F3z00	3822	0	245	1	Speech or language developmental disorder NOS	Feb-09
32923	ZT23.00	548	0	25	0	Speech problem	Feb-09
33403	1B9Z.00	1043	0	8	0	Speech problem NOS	Feb-09
3336	PG17.00	6133	0	142	2	Spina bifida occulta	Feb-09
27436	PD1..14	43	0	1	0	Sponge kidney	Feb-09
4892	H33z000	3720	0	442	0	Status asthmaticus NOS	Feb-09
4093	F253.11	2882	0	577	0	Status epilepticus	Feb-09
22685	F26y200	250	0	1	0	Status migrainosus	Feb-09

For peer review only - http://bmjopen.bmj.com/site/about/guidelines.xhtml

93910	F24y012	1	0	0	0	Steele - Richardson Oszewski syndrome	Feb-09
49034	F24y011	14	0	0	0	Steele Richardson Olszewsk syn	Feb-09
7037	F24y200	94	0	7	0	Steele-Richardson-Olszewski syndrome	Feb-09
16103	C164.12	741	0	51	0	Stein - Leventhal syndrome	Feb-09
50109	PB2..12	5	0	0	0	Stenosis large intestine	Feb-09
16731	P713.11	18	0	0	0	Stenosis of aortic arch	Feb-09
46120	P230.12	31	0	0	0	Stenosis of aqueduct of Sylvius	Feb-09
51675	P74z200	15	0	0	0	Stenosis of inferior vena cava	Feb-09
35852	J50z200	104	0	0	0	Stenosis of intestine NOS	Feb-09
2670	P735.00	785	0	13	0	Stenosis of pulmonary artery	Feb-09
29179	J572.00	135	0	7	0	Stenosis of rectum and anus	Feb-09
53581	J572z00	19	0	2	0	Stenosis of rectum and anus NOS	Feb-09
41197	P74z300	25	0	0	0	Stenosis of superior vena cava	Feb-09
29172	H5y1200	287	0	9	0	Stenosis of trachea	Feb-09
98579	F4J7011	2	0	0	0	Stilling-Turck-Duane syndrome	Feb-10
3769	G56z000	1072	0	156	0	Stokes-Adams syndrome	Feb-09
4749	PK61.00	506	0	20	0	Sturge-Weber syndrome	Feb-09
16539	P6y0.00	411	0	14	0	Subaortic stenosis	Feb-09
73431	J106300	1	0	0	0	Subdiaphragmatic oesophageal diverticulum	Feb-09
105037	P741000	1	0	0	0	Subdiaphragmatic total anomalous pulmonary venous return	Oct-12
36042	C313200	41	0	0	0	Sucrose-isomaltose intolerance	Feb-09
4098	N337100	1745	0	141	0	Sudek's atrophy	Feb-09
111382	D417.12	2	0	0	0	Sulphaemoglobinaemia	Oct-16
36434	J421000	94	0	4	0	Superior mesenteric artery syndrome	Feb-09
8412	G8y2200	219	0	23	0	Superior vena cava syndrome	Feb-09
64216	ZS42113	7	0	0	0	Suprabulbar palsy type of dysarthria	Feb-09

For peer review only - http://bmjopen.bmj.com/site/about/guidelines.xhtml

103039	P741100	1	0	0	0	Supradiaphragmatic total anomalous pulmonary venous return	Oct-11
53964	P722400	43	0	0	0	Supra-valvular aortic stenosis	Feb-09
1536	G57y900	51876	0	4010	0	Supraventricular tachycardia NOS	Feb-09
43838	PF1z.00	265	0	1	0	Syndactyly NOS	Feb-09
41117	PF11.00	33	0	1	0	Syndactyly of fingers without bone fusion	Feb-09
63079	PF10.00	33	0	0	0	"Syndactyly of multiple digits, unspecified"	Feb-09
67014	PF14.00	15	0	1	0	Syndactyly of toes with bone fusion	Feb-09
108734	PF14.13	2	0	0	0	Syndactyly of toes with bone fusion	Oct-14
56836	PF13.00	77	0	3	0	Syndactyly of toes without bone fusion	Feb-09
47358	F160100	23	0	2	0	Syringobulbia	Feb-09
69740	F160.00	17	0	0	0	Syringomyelia and syringobulbia	Feb-09
96785	F160z00	4	0	0	0	Syringomyelia or syringobulbia NOS	Apr-09
43593	P11y.11	21	0	2	0	Syringomyelocele	Feb-09
7871	N000.00	19120	0	1358	47	Systemic lupus erythematosus	Feb-09
45726	ZRq9.00	11	0	1	0	Systemic lupus erythematosus disease activity index	Feb-09
42719	N000z00	347	0	4	0	Systemic lupus erythematosus NOS	Feb-09
29519	N000300	123	0	11	2	Systemic lupus erythematosus with organ or sys involv	Feb-09
28417	N001.12	1671	0	49	0	Systemic sclerosis	Feb-09
110174	N001200	1	0	0	0	Systemic sclerosis induced by drugs and chemicals	Oct-15
37640	G757.00	234	0	4	0	Takayasu's disease	Feb-09
68533	D313211	21	0	1	0	TAR syndrome	Feb-09
40025	P511300	12	0	0	0	Taussig-Bing syndrome	Feb-09
21169	F101300	266	0	135	130	Tay-Sach's disease	Feb-09
3175	F254000	13930	0	664	2	Temporal lobe epilepsy	Feb-09

For peer review only - http://bmjopen.bmj.com/site/about/guidelines.xhtml

36325	B470300	78	0	1	0	Teratoma of undescended testis	Feb-09
15118	B7B..11	312	0	24	0	"Teratoma, benign"	Feb-09
48765	PCy4.12	23	0	0	0	"Testicular agenesis, bilateral"	Feb-09
38998	PCy5.12	59	0	1	0	"Testicular agenesis, unilateral"	Feb-09
63528	C172000	14	0	1	0	Testicular hypofunc- defect adrenocortical hormone synthesis	Feb-09
9577	C172.12	6404	0	377	0	Testicular hypogonadism	Feb-09
4864	P52..00	2444	0	79	1	Tetralogy of Fallot	Feb-09
63046	P52z.00	42	0	0	0	Tetralogy of Fallot NOS	Feb-09
38967	P520.00	34	0	2	0	"Tetralogy of Fallot, unspecified"	Feb-09
1171	D104.00	6076	0	275	84	Thalassaemia	Feb-09
1174	D104100	2813	0	184	105	Thalassaemia minor NEC	Feb-09
4666	D104z00	191	0	0	0	Thalassaemia NOS	Feb-09
54429	D104200	42	0	4	3	Thalassaemia with haemoglobin S disease	Feb-09
56900	PG44200	5	0	0	0	Thanatophoric dwarfism	Feb-09
67262	PG44211	2	0	0	0	Thanatophoric dysplasia	Feb-09
23484	F392111	51	0	6	0	Thomsen's disease	Feb-09
57510	PH33312	7	0	0	0	Thomson's disease	Feb-09
62286	SJ21.00	7	0	0	0	Thoracic cord injury without spinal bone injury	Feb-09
102775	SJ21z00	1	0	0	0	"Thoracic cord injury without spinal bone injury, NOS"	Aug-11
51550	N129200	47	0	2	0	Thoracic disc disorder with myelopathy	Feb-09
23390	N121.00	575	0	15	0	Thoracic disc displacement without myelopathy	Feb-09
51691	N12B100	39	0	0	0	Thoracic disc prolapse with myelopathy	Feb-09
40333	N12C100	65	0	2	0	Thoracic disc prolapse with radiculopathy	Feb-09
103025	C14z.00	3	0	1	0	Thymus disease NOS	Sep-11

For peer review only - http://bmjopen.bmj.com/site/about/guidelines.xhtml

69113	C06y100	6	0	0	0	Thyroid atrophy	Feb-09
14704	C04..12	76375	0	3117	110	Thyroid deficiency	Feb-09
24681	66B8.00	3932	0	5	0	Thyroid dis.treatment changed	Feb-09
38292	66B9.00	986	0	6	0	Thyroid dis.treatment started	Feb-09
35957	C06z.00	1079	0	29	0	Thyroid disorder NOS	Feb-09
42603	AC22.00	2	0	0	0	Thyroid echinococcus granulosus	Feb-09
28530	66B4.00	798	0	9	0	Thyroid eye disease	Feb-09
23014	C04z.12	98	0	0	0	Thyroid insufficiency	Feb-09
1346	C05..00	8097	0	649	11	Thyroiditis	Feb-09
677	C02..00	100352	0	9527	54	Thyrotoxicosis	Feb-09
64856	C02y200	5	0	0	0	Thyrotoxicosis factitia	Feb-09
49508	C024.00	45	0	4	0	Thyrotoxicosis from ectopic thyroid nodule	Feb-09
56270	C024z00	4	0	1	0	Thyrotoxicosis from ectopic thyroid nodule NOS	Feb-09
64656	C024000	1	0	0	0	Thyrotoxicosis from ectopic thyroid nodule with no crisis	Feb-09
26699	C02zz00	2292	0	46	0	Thyrotoxicosis NOS	Feb-09
43136	C02y.00	49	0	0	0	Thyrotoxicosis of other specified origin	Feb-09
34220	C02yz00	38	0	0	0	Thyrotoxicosis of other specified origin NOS	Feb-09
106532	C02y100	2	0	0	0	Thyrotoxicosis of other specified origin with crisis	Aug-13
51273	C02y000	17	0	0	0	Thyrotoxicosis of other specified origin with no crisis	Feb-09
26701	C02z000	68	0	2	0	Thyrotoxicosis without mention of goitre or cause no crisis	Feb-09
15565	C02z.00	1188	0	76	0	Thyrotoxicosis without mention of goitre or other cause	Feb-09
3194	C02z100	253	0	21	0	"Thyrotoxicosis without mention of goitre, cause with crisis"	Feb-09

For peer review only - http://bmjopen.bmj.com/site/about/guidelines.xhtml

18688	F24y100	453	0	8	0	Todd's paralysis	Feb-09
8187	F251500	3156	0	85	0	Tonic-clonic epilepsy	Feb-09
22804	F251011	410	0	7	0	Tonic-clonic epilepsy	Feb-09
68975	PF27100	1	0	0	0	Total absence of ulna	Feb-09
51941	P741.00	61	0	0	0	Total anomalous pulmonary venous return - TAPVR	Feb-09
68784	P741z00	16	0	0	0	Total anomalous pulmonary venous return NOS	Feb-09
95444	790M000	20	0	0	0	Total cavopulmonary con extrac inf cav vein pulmon art con	Feb-09
91455	790M100	16	0	0	0	Total cavopulmonary connection with lateral atrial tunnel	Feb-09
39081	P334000	6	0	0	0	Total congenital cataract	Feb-09
49621	F475100	10	0	0	0	Total internal ophthalmoplegia	Feb-09
66061	PF26200	2	0	0	0	Total radial absence	Feb-09
23315	C020.00	853	0	49	0	Toxic diffuse goitre	Feb-09
49334	C020z00	105	0	1	0	Toxic diffuse goitre NOS	Feb-09
57011	C020100	13	0	0	0	Toxic diffuse goitre with crisis	Feb-09
26702	C020000	69	0	1	0	Toxic diffuse goitre with no crisis	Feb-09
55416	G52y700	8	0	1	0	Toxic myocarditis	Feb-09
63323	F382.00	4	0	0	0	Toxic myoneural disorder	Feb-09
32916	F394.00	19	0	0	0	Toxic myopathy	Feb-09
50893	K0C4.00	11	0	0	0	"Toxic nephropathy, not elsewhere classified"	Feb-09
45081	F37..11	38	0	3	0	Toxic neuropathy	Feb-09
49361	C023z00	57	0	2	0	Toxic nodular goitre NOS	Feb-09
100004	C023100	1	0	0	0	Toxic nodular goitre unspecified with crisis	Jun-10
68512	C023000	6	0	0	0	Toxic nodular goitre unspecified with no crisis	Feb-09
22373	F4H3400	359	0	25	0	Toxic optic neuropathy	Feb-09
53280	C021.00	78	0	3	0	Toxic uninodular goitre	Feb-09

For peer review only - http://bmjopen.bmj.com/site/about/guidelines.xhtml

For peer review only

61498	C021z00	14	0	2	0	Toxic uninodular goitre NOS	Feb-09
26869	C021000	10	0	1	0	Toxic uninodular goitre with no crisis	Feb-09
47068	C294.00	56	0	1	0	Trace element deficiency	Feb-09
68903	Q313400	3	0	0	0	Tracheobronchial haemorrhage origin in the perinatal period	Feb-09
4048	J10y200	941	0	32	0	Tracheo-oesophageal fistula	Feb-09
51167	H5y0400	76	0	2	0	Tracheo-oesophageal fistula following tracheostomy	Feb-09
62637	D011200	27	0	0	0	Transcobalamin II deficiency	Feb-09
93178	ZS78411	2	0	0	0	Transcortical motor aphasia	Feb-09
105929	ZS78611	1	0	1	0	Transcortical sensory aphasia	Apr-13
101822	ZS78600	1	0	1	0	Transcortical sensory dysphasia	Mar-11
65785	PK31.11	3	0	0	0	Transposition of abdominal viscera	Feb-09
59279	PB53100	9	0	0	0	Transposition of appendix	Feb-09
66200	PB53200	2	0	0	0	Transposition of caecum	Feb-09
45857	PB53300	15	0	1	0	Transposition of colon	Feb-09
48206	7927300	78	0	0	0	Transposition of coronary artery NEC	Feb-09
39857	7H40600	18	0	0	0	Transposition of iliopsoas muscle	Feb-09
67877	PB53.00	5	0	0	0	Transposition of intestine	Feb-09
71180	PB53000	1	0	0	0	"Transposition of intestine, unspecified"	Feb-09
71874	P74z700	3	0	1	0	Transposition of pulmonary veins	Feb-09
66199	PA77.00	9	0	0	0	Transposition of stomach	Feb-09
47596	PK30.11	12	0	0	0	Transposition of viscera unspecified	Feb-09
6494	F037.00	2471	0	125	0	Transverse myelitis	Feb-09
42239	G140112	19	0	0	0	Tricuspid incompetence - rheumatic	Feb-09
34869	G140412	107	0	3	0	"Tricuspid incompetence, cause unspecified"	Feb-09
1779	G542000	783	0	15	0	"Tricuspid incompetence, non-rheumatic"	Feb-09

For peer review only - http://bmjopen.bmj.com/site/about/guidelines.xhtml

42128	G140400	318	0	7	0	"Tricuspid insufficiency, cause unspecified"	Feb-09
97738	G542011	3	0	0	0	"Tricuspid insufficiency, non-rheumatic"	Oct-09
21980	G140111	231	0	8	0	Tricuspid regurgitation - rheumatic	Feb-09
9286	G140413	6093	0	42	0	"Tricuspid regurgitation, cause unspecified"	Feb-09
35372	G542012	1335	0	5	0	"Tricuspid regurgitation, non-rheumatic"	Feb-09
72306	G140500	3	0	0	0	"Tricuspid stenosis and insufficiency, cause unspecified"	Feb-09
49551	G140514	34	0	0	0	"Tricuspid stenosis and regurgitation, cause unspecified"	Feb-09
56029	G140300	26	0	0	0	"Tricuspid stenosis, cause unspecified"	Feb-09
35724	G542100	26	0	3	0	"Tricuspid stenosis, non-rheumatic"	Feb-09
16373	G140.00	457	0	28	0	Tricuspid valve disease NEC	Feb-09
43855	G542z00	108	0	0	0	Tricuspid valve disorders NOS	Feb-09
2817	G542.00	297	0	11	0	"Tricuspid valve disorders, non-rheumatic"	Feb-09
103577	F262E00	92	0	0	0	Trigeminal autonomic cephalalgia	Jan-12
38476	PG0B.00	127	0	3	0	Trigonocephaly	Feb-09
107520	PG04.12	1	0	0	0	Trigorhinophalangeal dysplasia	Dec-13
110299	PB6yw13	1	0	0	0	Trilobular liver	Dec-15
92988	PD3C.00	2	0	0	0	Triple kidney with triple pelvis	Feb-09
6377	PJy2.11	73	0	2	0	Triple X female	Feb-09
34913	PJ52300	35	0	0	0	Triploidy	Feb-09
54377	PJ52.00	12	0	0	0	Trisomies of autosomes NEC	Feb-09
70198	PJ50600	7	0	0	0	Trisomy 12	Feb-09
18415	PJ0..12	366	0	12	2	Trisomy 21	Feb-09
61627	PJ0z.11	30	0	0	0	Trisomy 21 NOS	Feb-09

For peer review only - http://bmjopen.bmj.com/site/about/guidelines.xhtml

42701	PJ00.00	10	0	0	0	"Trisomy 21, meiotic nondisjunction"	Feb-09
107919	PJ01.11	1	0	0	0	"Trisomy 21, mitotic nondisjunction"	Apr-14
32010	PJ01.00	29	0	0	0	"Trisomy 21, mosaicism"	Feb-09
61499	PJ02.00	11	0	2	0	"Trisomy 21, translocation"	Feb-09
100024	PJ50800	2	0	0	0	Trisomy 22	Jun-10
37591	PJ50100	3	0	0	0	Trisomy 7	Feb-09
107119	PJ52z00	1	0	0	0	Trisomy of autosomes NEC NOS	Oct-13
46264	PC70.00	43	0	3	0	True hermaphroditism	Feb-09
41371	P502.11	73	0	0	0	Truncus arteriosus	Feb-09
45187	P500.12	28	0	11	0	Truncus arteriosus	Feb-09
62310	C37y300	11	0	3	0	Trypsinogen deficiency	Feb-09
11146	C134300	625	0	41	4	TSH - thyroid-stimulating hormone deficiency	Feb-09
30721	PJ63100	26	0	1	1	"Turner's phenotype, karyotype 45X"	Feb-09
92599	PJ63200	10	0	0	0	"Turner's phenotype, karyotype 46X iso (Xq)"	Feb-09
98244	PJ63000	2	0	0	0	"Turner's phenotype, karyotype normal"	Dec-09
51868	PJ63400	38	0	1	0	"Turner's phenotype, mosaicism 45X/46XX or 45X/46XY"	Feb-09
65206	PJ63600	4	0	0	0	"Turner's phenotype, other variant karyotypes"	Feb-09
64945	PJ63612	3	0	0	0	"Turner's phenotype, partial X deletion karyotype"	Feb-09
97871	PJ63611	1	0	0	0	"Turner's phenotype, ring chromosome karyotype"	Oct-09
4943	PJ63.00	2501	0	161	0	Turner's syndrome	Feb-09
53168	PJ63z00	49	0	2	0	Turner's syndrome NOS	Feb-09

For peer review only - http://bmjopen.bmj.com/site/about/guidelines.xhtml

109385	PJ63300	4	0	0	0	"Turner's,karyotype 46X + abnorm. sex chromosome,not iso(Xq)"	Mar-15
40570	PJ63500	76	0	0	0	"Turner's,mosaic, 45X/other cell line with abn.sex chromosome"	Feb-09
1549	C10E.00	92258	0	2218	2	Type 1 diabetes mellitus	Feb-09
17858	C108.12	6354	0	175	0	Type 1 diabetes mellitus	Feb-09
35288	C10E800	340	0	30	0	Type 1 diabetes mellitus - poor control	Feb-09
45914	C108812	9	0	0	0	Type 1 diabetes mellitus - poor control	Feb-09
109628	C10P011	1	0	0	0	Type 1 diabetes mellitus in remission	May-15
40682	C10E900	82	0	1	0	Type 1 diabetes mellitus maturity onset	Feb-09
97446	C108912	2	0	0	0	Type 1 diabetes mellitus maturity onset	Aug-09
18642	C10EH00	11	0	0	0	Type 1 diabetes mellitus with arthropathy	Feb-09
49554	C10EF00	20	0	0	0	Type 1 diabetes mellitus with diabetic cataract	Feb-09
110400	C108F12	1	0	0	0	Type 1 diabetes mellitus with diabetic cataract	Dec-15
22871	C10EP00	63	0	4	0	Type 1 diabetes mellitus with exudative maculopathy	Feb-09
69993	C10E600	12	0	0	0	Type 1 diabetes mellitus with gangrene	Feb-09
55239	C10EQ00	304	0	5	0	Type 1 diabetes mellitus with gastroparesis	Feb-09
39070	C10EE00	85	0	1	0	Type 1 diabetes mellitus with hypoglycaemic coma	Feb-09
70766	C108E12	2	0	0	0	Type 1 diabetes mellitus with hypoglycaemic coma	Feb-09

For peer review only - http://bmjopen.bmj.com/site/about/guidelines.xhtml

10692	C10EM00	5880	0	106	0	Type 1 diabetes mellitus with ketoacidosis	Feb-09
40837	C10EN00	242	0	26	0	Type 1 diabetes mellitus with ketoacidotic coma	Feb-09
68105	C10EB00	11	0	0	0	Type 1 diabetes mellitus with mononeuropathy	Feb-09
47650	C10E300	37	0	0	0	Type 1 diabetes mellitus with multiple complications	Feb-09
10418	C10ED00	487	0	9	0	Type 1 diabetes mellitus with nephropathy	Feb-09
42831	C10E200	24	0	1	0	Type 1 diabetes mellitus with neurological complications	Feb-09
61829	C108212	6	0	0	0	Type 1 diabetes mellitus with neurological complications	Feb-09
18230	C108J12	3	0	1	0	Type 1 diabetes mellitus with neuropathic arthropathy	Feb-09
54008	C10EJ00	61	0	0	0	Type 1 diabetes mellitus with neuropathic arthropathy	Feb-09
47649	C10E100	21	0	2	0	Type 1 diabetes mellitus with ophthalmic complications	Feb-09
102740	C108112	1	0	0	0	Type 1 diabetes mellitus with ophthalmic complications	Aug-11
93468	C10EG00	5	0	0	0	Type 1 diabetes mellitus with peripheral angiopathy	Feb-09
30294	C10EL00	464	0	5	0	Type 1 diabetes mellitus with persistent microalbuminuria	Feb-09
30323	C10EK00	521	0	5	0	Type 1 diabetes mellitus with persistent proteinuria	Feb-09
46301	C10EC00	34	0	0	0	Type 1 diabetes mellitus with polyneuropathy	Feb-09
21983	C108012	5	0	0	0	Type 1 diabetes mellitus with renal complications	Feb-09

For peer review only - http://bmjopen.bmj.com/site/about/guidelines.xhtml

47582	C10E000	50	0	2	0	Type 1 diabetes mellitus with renal complications	Feb-09
18387	C10E700	536	0	12	0	Type 1 diabetes mellitus with retinopathy	Feb-09
41049	C108712	9	0	0	0	Type 1 diabetes mellitus with retinopathy	Feb-09
68390	C108512	15	0	0	0	Type 1 diabetes mellitus with ulcer	Feb-09
69676	C10EA00	53	0	1	0	Type 1 diabetes mellitus without complication	Feb-09
111106	C108A12	1	0	0	0	Type 1 diabetes mellitus without complication	Jul-16
104453	66At011	114	0	0	0	Type 1 diabetic dietary review	Jun-12
24599	Z271G11	150	0	3	0	Type 1 dip	Feb-09
758	C10F.00	906638	0	12665	21	Type 2 diabetes mellitus	Feb-09
17859	C109.12	46888	0	1121	2	Type 2 diabetes mellitus	Feb-09
25627	C10F700	1307	0	57	0	Type 2 diabetes mellitus - poor control	Feb-09
45913	C109712	45	0	1	0	Type 2 diabetes mellitus - poor control	Feb-09
110611	C10P111	12	0	0	0	Type 2 diabetes mellitus in remission	Mar-16
49869	C109G12	1	0	0	0	Type 2 diabetes mellitus with arthropathy	Feb-09
59253	C10FG00	50	0	2	0	Type 2 diabetes mellitus with arthropathy	Feb-09
44779	C109E12	9	0	1	0	Type 2 diabetes mellitus with diabetic cataract	Feb-09
44982	C10FE00	154	0	5	0	Type 2 diabetes mellitus with diabetic cataract	Feb-09
25591	C10FQ00	189	0	5	0	Type 2 diabetes mellitus with exudative maculopathy	Feb-09

For peer review only - http://bmjopen.bmj.com/site/about/guidelines.xhtml

12736	C10F500	54	0	4	0	Type 2 diabetes mellitus with gangrene	Feb-09
46150	C109512	7	0	0	0	Type 2 diabetes mellitus with gangrene	Feb-09
63690	C10FR00	177	0	5	0	Type 2 diabetes mellitus with gastroparesis	Feb-09
46917	C10FD00	106	0	2	0	Type 2 diabetes mellitus with hypoglycaemic coma	Feb-09
61071	C109D12	7	0	1	0	Type 2 diabetes mellitus with hypoglycaemic coma	Feb-09
32627	C10FN00	855	0	50	0	Type 2 diabetes mellitus with ketoacidosis	Feb-09
51756	C10FP00	30	0	0	0	Type 2 diabetes mellitus with ketoacidotic coma	Feb-09
62674	C10FA00	71	0	0	0	Type 2 diabetes mellitus with mononeuropathy	Feb-09
65267	C10F300	37	0	2	0	Type 2 diabetes mellitus with multiple complications	Feb-09
108005	C109312	56	0	0	0	Type 2 diabetes mellitus with multiple complications	May-14
12640	C10FC00	2067	0	64	0	Type 2 diabetes mellitus with nephropathy	Feb-09
24836	C109C12	10	0	1	0	Type 2 diabetes mellitus with nephropathy	Feb-09
34268	C10F200	176	0	3	0	Type 2 diabetes mellitus with neurological complications	Feb-09
45919	C109212	16	0	0	0	Type 2 diabetes mellitus with neurological complications	Feb-09
35385	C10FH00	214	0	7	0	Type 2 diabetes mellitus with neuropathic arthropathy	Feb-09
66965	C109H12	9	0	0	0	Type 2 diabetes mellitus with neuropathic arthropathy	Feb-09

For peer review only - http://bmjopen.bmj.com/site/about/guidelines.xhtml

47321	C10F100	100	0	1	0	Type 2 diabetes mellitus with ophthalmic complications	Feb-09
70316	C109112	39	0	0	0	Type 2 diabetes mellitus with ophthalmic complications	Feb-09
37806	C10FF00	83	0	2	0	Type 2 diabetes mellitus with peripheral angiopathy	Feb-09
60699	C109F12	4	0	0	0	Type 2 diabetes mellitus with peripheral angiopathy	Feb-09
18390	C10FM00	8573	0	54	0	Type 2 diabetes mellitus with persistent microalbuminuria	Feb-09
26054	C10FL00	4613	0	48	3	Type 2 diabetes mellitus with persistent proteinuria	Feb-09
18425	C10FB00	255	0	4	0	Type 2 diabetes mellitus with polyneuropathy	Feb-09
109865	C109B12	3	0	0	0	Type 2 diabetes mellitus with polyneuropathy	Jul-15
18777	C10F000	231	0	18	0	Type 2 diabetes mellitus with renal complications	Feb-09
18496	C10F600	1868	0	42	2	Type 2 diabetes mellitus with retinopathy	Feb-09
42762	C109612	23	0	1	0	Type 2 diabetes mellitus with retinopathy	Feb-09
49074	C10F400	116	0	4	0	Type 2 diabetes mellitus with ulcer	Feb-09
65704	C109412	14	0	2	0	Type 2 diabetes mellitus with ulcer	Feb-09
47954	C10F900	871	0	6	0	Type 2 diabetes mellitus without complication	Feb-09
105784	C109912	3	0	0	0	Type 2 diabetes mellitus without complication	Feb-13
102611	66At111	243	0	0	0	Type 2 diabetic dietary review	Jul-11
44403	Z271H11	26	0	0	0	Type 2 dip	Feb-09
37957	C10K.00	89	0	2	0	Type A insulin resistance	Feb-09

For peer review only - http://bmjopen.bmj.com/site/about/guidelines.xhtml

56885	C10K000	1	0	0	0	Type A insulin resistance without complication	Feb-09
24423	C108.13	793	0	17	0	Type I diabetes mellitus	Feb-09
46850	C108811	18	0	0	0	Type I diabetes mellitus - poor control	Feb-09
105337	C10E811	8	0	0	0	Type I diabetes mellitus - poor control	Dec-12
108360	C10P000	2	0	0	0	Type I diabetes mellitus in remission	Jul-14
63017	C108911	1	0	0	0	Type I diabetes mellitus maturity onset	Feb-09
96235	C10E911	12	0	0	0	Type I diabetes mellitus maturity onset	Apr-09
62352	C108H11	1	0	0	0	Type I diabetes mellitus with arthropathy	Feb-09
17545	C108F11	5	0	1	0	Type I diabetes mellitus with diabetic cataract	Feb-09
97894	C10EP11	1	0	0	0	Type I diabetes mellitus with exudative maculopathy	Oct-09
102112	C10E611	6	0	0	0	Type I diabetes mellitus with gangrene	Apr-11
108724	C10EQ11	1	0	0	0	Type I diabetes mellitus with gastroparesis	Oct-14
42729	C108E11	14	0	0	0	Type I diabetes mellitus with hypoglycaemic coma	Feb-09
62209	C10EM11	46	0	0	0	Type I diabetes mellitus with ketoacidosis	Feb-09
66145	C10EN11	1	0	0	0	Type I diabetes mellitus with ketoacidotic coma	Feb-09
99231	C108B11	1	0	0	0	Type I diabetes mellitus with mononeuropathy	Apr-10
91942	C10E311	5	0	0	0	Type I diabetes mellitus with multiple complications	Feb-09

For peer review only - http://bmjopen.bmj.com/site/about/guidelines.xhtml

108007	C108311	1	0	0	0	Type I diabetes mellitus with multiple complications	May-14
66872	C108D11	7	0	0	0	Type I diabetes mellitus with nephropathy	Feb-09
49146	C108211	1	0	0	0	Type I diabetes mellitus with neurological complications	Feb-09
60208	C108J11	3	0	0	0	Type I diabetes mellitus with neuropathic arthropathy	Feb-09
99311	C10E111	1	0	0	0	Type I diabetes mellitus with ophthalmic complications	May-10
102620	C10EL11	1	0	0	0	Type I diabetes mellitus with persistent microalbuminuria	Jul-11
91943	C10EC11	1	0	0	0	Type I diabetes mellitus with polyneuropathy	Feb-09
61344	C108011	6	0	0	0	Type I diabetes mellitus with renal complications	Feb-09
109837	C10E011	1	0	0	0	Type I diabetes mellitus with renal complications	Jul-15
38161	C108711	14	0	1	0	Type I diabetes mellitus with retinopathy	Feb-09
95343	C10E711	15	0	0	0	Type I diabetes mellitus with retinopathy	Feb-09
51957	C108511	10	0	1	0	Type I diabetes mellitus with ulcer	Feb-09
93878	C10E511	6	0	0	0	Type I diabetes mellitus with ulcer	Feb-09
62613	C10EA11	4	0	0	0	Type I diabetes mellitus without complication	Feb-09
95992	C108A11	1	0	0	0	Type I diabetes mellitus without complication	Feb-09
102704	66At000	7865	0	8	0	Type I diabetic dietary review	Aug-11
18219	C109.13	4924	0	125	0	Type II diabetes mellitus	Feb-09
22884	C10F.11	5124	0	13	0	Type II diabetes mellitus	Feb-09

For peer review only - http://bmjopen.bmj.com/site/about/guidelines.xhtml

24458	C109711	49	0	2	0	Type II diabetes mellitus - poor control	Feb-09
47315	C10F711	88	0	0	0	Type II diabetes mellitus - poor control	Feb-09
107824	C10P100	27	0	0	0	Type II diabetes mellitus in remission	Mar-14
18143	C109G11	1	0	0	0	Type II diabetes mellitus with arthropathy	Feb-09
103902	C10FG11	17	0	0	0	Type II diabetes mellitus with arthropathy	Mar-12
48192	C109E11	14	0	4	0	Type II diabetes mellitus with diabetic cataract	Feb-09
93727	C10FE11	52	0	1	0	Type II diabetes mellitus with diabetic cataract	Feb-09
111798	C10FQ11	1	0	0	0	Type II diabetes mellitus with exudative maculopathy	May-17
62107	C109511	7	0	1	0	Type II diabetes mellitus with gangrene	Feb-09
104323	C10F511	3	0	0	0	Type II diabetes mellitus with gangrene	Jun-12
56268	C109D11	2	0	0	0	Type II diabetes mellitus with hypoglycaemic coma	Feb-09
98723	C10FD11	22	0	0	0	Type II diabetes mellitus with hypoglycaemic coma	Mar-10
106528	C10FN11	2	0	0	0	Type II diabetes mellitus with ketoacidosis	Aug-13
106061	C10FP11	1	0	0	0	Type II diabetes mellitus with ketoacidotic coma	May-13
50813	C109A11	1	0	0	0	Type II diabetes mellitus with mononeuropathy	Feb-09
95351	C10FA11	11	0	0	0	Type II diabetes mellitus with mononeuropathy	Feb-09

For peer review only - http://bmjopen.bmj.com/site/about/guidelines.xhtml

43227	C10F311	9	0	0	0	Type II diabetes mellitus with multiple complications	Feb-09
64571	C109C11	5	0	1	0	Type II diabetes mellitus with nephropathy	Feb-09
102201	C10FC11	36	0	1	0	Type II diabetes mellitus with nephropathy	May-11
67905	C109211	5	0	0	0	Type II diabetes mellitus with neurological complications	Feb-09
98616	C10F211	2	0	0	0	Type II diabetes mellitus with neurological complications	Mar-10
47816	C109H11	7	0	3	0	Type II diabetes mellitus with neuropathic arthropathy	Feb-09
109197	C10FH11	4	0	0	0	Type II diabetes mellitus with neuropathic arthropathy	Jan-15
59725	C109111	2	0	0	0	Type II diabetes mellitus with ophthalmic complications	Feb-09
100964	C10F111	5	0	0	0	Type II diabetes mellitus with ophthalmic complications	Nov-10
54899	C109F11	4	0	0	0	Type II diabetes mellitus with peripheral angiopathy	Feb-09
104639	C10FF11	24	0	0	0	Type II diabetes mellitus with peripheral angiopathy	Aug-12
60796	C10FL11	60	0	0	0	Type II diabetes mellitus with persistent proteinuria	Feb-09
47409	C109B11	4	0	0	0	Type II diabetes mellitus with polyneuropathy	Feb-09
50527	C10FB11	17	0	0	0	Type II diabetes mellitus with polyneuropathy	Feb-09
50225	C109011	9	0	1	0	Type II diabetes mellitus with renal complications	Feb-09
57278	C10F011	5	0	0	0	Type II diabetes mellitus with renal complications	Feb-09

For peer review only - http://bmjopen.bmj.com/site/about/guidelines.xhtml

For peer review only

49655	C10F611	37	0	0	0	Type II diabetes mellitus with retinopathy	Feb-09
58604	C109611	21	0	5	0	Type II diabetes mellitus with retinopathy	Feb-09
55075	C109411	19	0	1	0	Type II diabetes mellitus with ulcer	Feb-09
91646	C10F411	5	0	0	0	Type II diabetes mellitus with ulcer	Feb-09
53392	C10F911	256	0	0	0	Type II diabetes mellitus without complication	Feb-09
109103	C109911	0	0	0	0	Type II diabetes mellitus without complication	Jan-15
101801	66At100	165941	0	35	0	Type II diabetic dietary review	Feb-11
111509	K08yB11	1	0	0	0	Type IV renal tubular acidosis	Dec-16
31001	C302611	47	0	0	0	Tyrosinaemia	Feb-09
55159	46F7.11	0	0	0	2	Tyrosine cryst.- urine	Feb-09
45295	C302300	5	0	0	0	Tyrosinosis	Feb-09
63807	C302400	11	0	0	0	Tyrosinuria	Feb-09
30433	J411.00	221	0	8	0	Ulcerative (chronic) enterocolitis	Feb-09
42822	J412.00	35	0	2	0	Ulcerative (chronic) ileocolitis	Feb-09
704	J410100	92252	0	4661	46	Ulcerative colitis	Feb-09
1784	J41..12	32854	0	1756	30	Ulcerative colitis and/or proctitis	Feb-09
48732	J410000	39	0	3	0	Ulcerative ileocolitis	Feb-09
104428	J08zF11	172	0	2	0	Ulcerative oral mucositis	Jun-12
104259	J413.00	408	0	4	0	Ulcerative pancolitis	Jun-12
8347	J410300	3226	0	103	0	Ulcerative proctitis	Feb-09
6650	J410.00	8183	0	464	4	Ulcerative proctocolitis	Feb-09
33456	J410z00	135	0	1	0	Ulcerative proctocolitis NOS	Feb-09
24858	J410200	197	0	1	0	Ulcerative rectosigmoiditis	Feb-09
105762	B62C.00	7	0	0	0	Unifocal Langerhans-cell histiocytosis	Feb-13
63930	P911.00	21	0	0	0	Unilateral complete cleft lip	Feb-09
73531	P901.00	3	0	0	0	Unilateral complete cleft palate	Feb-09

For peer review only - http://bmjopen.bmj.com/site/about/guidelines.xhtml

62987	P921.00	16	0	0	0	Unilateral complete cleft palate with cleft lip	Feb-09
102267	J06y700	4	0	0	0	Unilateral condylar mandibular hyperplasia	May-11
103529	J06y800	1	0	0	0	Unilateral condylar mandibular hypoplasia	Jan-12
23958	PD02100	362	0	4	0	Unilateral congenital absence of kidney	Feb-09
45970	PE30000	266	0	3	0	Unilateral congenital dislocation of hip	Feb-09
34019	PE31000	78	0	0	0	Unilateral congenital subluxation of hip	Feb-09
38772	PE34000	135	0	0	0	Unilateral dysplastic hip	Feb-09
68946	F255500	4	0	0	0	Unilateral epilepsy	Feb-09
54012	P912.00	54	0	0	0	Unilateral incomplete cleft lip	Feb-09
73558	P902.00	8	0	0	0	Unilateral incomplete cleft palate	Feb-09
54144	P922.00	5	0	0	0	Unilateral incomplete cleft palate with cleft lip	Feb-09
31961	PD00100	80	0	0	0	Unilateral renal agenesis	Feb-09
54656	PD04111	3	0	0	0	Unilateral renal dysgenesis	Feb-09
24120	PD04100	63	0	2	0	Unilateral renal dysplasia	Feb-09
30650	PD03100	46	0	1	0	Unilateral renal hypoplasia	Feb-09
43919	K090.00	309	0	3	0	Unilateral small kidney	Feb-09
105369	K090100	3	0	1	0	Unilateral small kidney with contralateral hypertrophy	Dec-12
31097	K16X.00	188	0	1	0	"Uninhibited neuropathic bladder, NEC"	Feb-09
62520	K03W.00	8	0	0	0	"Unsp nephrit synd, diff endocap prolif glomerulonephritis"	Feb-09
30301	K03X.00	38	0	1	0	"Unsp nephrit synd, diff mesang prolif glomerulonephritis"	Feb-09

For peer review only - http://bmjopen.bmj.com/site/about/guidelines.xhtml

73574	P2y..00	6	0	0	0	Unspec nervous system anomaly of brain/cord/nervous system	Feb-09
36125	K03U.00	35	0	1	0	"Unspecif nephr synd, diff concentric glomerulonephritis"	Feb-09
25280	PAz2.00	93	0	8	0	Unspecified anomalies of stomach	Feb-09
44752	PDz2.00	103	0	6	0	Unspecified anomaly of bladder	Feb-09
22492	PGz2.00	84	0	7	0	Unspecified anomaly of bones	Feb-09
28247	PGz4.00	53	0	2	0	Unspecified anomaly of connective tissue	Feb-09
26626	P6z0.00	89	0	6	0	Unspecified anomaly of heart valve	Feb-09
46880	PDz3.00	75	0	10	0	Unspecified anomaly of urethra	Feb-09
70721	E117500	6	0	0	0	"Unspecified bipolar affect disord, partial/unspec remission"	Feb-09
24230	E117600	282	0	0	0	"Unspecified bipolar affective disorder, in full remission"	Feb-09
63698	E117100	12	0	0	0	"Unspecified bipolar affective disorder, mild"	Feb-09
68647	E117200	14	0	0	0	"Unspecified bipolar affective disorder, moderate"	Feb-09
27986	E117z00	310	0	5	0	"Unspecified bipolar affective disorder, NOS"	Feb-09
73423	E117300	3	0	0	0	"Unspecified bipolar affective disorder, severe, no psychosis"	Feb-09
49763	E117000	89	0	0	0	"Unspecified bipolar affective disorder, unspecified"	Feb-09
68326	E117400	14	0	0	0	"Unspecified bipolar affective disorder,severe with psychosis"	Feb-09
71029	PJ5z.00	4	0	0	0	Unspecified conditions due to autosomal anomalies	Feb-09
64449	C108z00	3	0	0	0	Unspecified diabetes mellitus with multiple complications	Feb-09
62920	J34y.00	101	0	11	11	Unspecified diaphragmatic hernia	Feb-09

For peer review only - http://bmjopen.bmj.com/site/about/guidelines.xhtml

5182	K03z.00	3309	0	206	3	Unspecified glomerulonephritis NOS	Feb-09
66506	E101000	1	0	0	0	Unspecified hebephrenic schizophrenia	Feb-09
99620	F435000	1	0	0	0	Unspecified hereditary choroid dystrophy	Jun-10
42511	F427000	14	0	0	0	Unspecified hereditary retinal dystrophies	Feb-09
94560	F4J3000	13	0	0	0	Unspecified heterotropia	Feb-09
73638	AB4zz00	2	0	0	0	Unspecified histoplasmosis NOS	Feb-09
96000	A907.00	2	0	0	0	Unspecified late congenital syphilis	Feb-09
69370	P118000	2	0	0	0	Unspecified spina bifida without hydrocephalus - closed	Feb-09
95018	P110z00	2	0	0	0	Unspecified spina bifida without hydrocephalus NOS	Feb-09
66679	PG51.15	12	0	0	0	Van der Hoeve's syndrome	Feb-09
2155	G341000	990	0	27	0	Ventricular cardiac aneurysm	Feb-09
246	P54..00	27151	0	753	1	Ventricular septal defect	Feb-09
23692	P520.11	250	0	14	0	Ventricular septal defect in Fallot's tetralogy	Feb-09
34067	P54z.00	488	0	3	0	Ventricular septal defect NOS	Feb-09
42132	P540.00	220	0	0	0	"Ventricular septal defect, unspecified"	Feb-09
7794	G571.11	3193	0	84	0	Ventricular tachycardia	Feb-09
35762	7010000	119	0	10	0	Ventriculocisternostomy	Feb-09
26280	F28y300	450	0	0	0	Ventriculomegaly	Feb-09
2482	D011100	69	0	5	0	Vit B12 defic anaemia due to malabsorption with proteinuria	Feb-09
5007	C24..00	218	0	2	2	Vitamin A deficiency	Feb-09
31479	C241.00	24	0	1	0	Vitamin A deficiency + Bitot's spot and conjunctival xerosis	Feb-09
50073	C240.00	12	0	0	0	Vitamin A deficiency with conjunctival xerosis	Feb-09

For peer review only - http://bmjopen.bmj.com/site/about/guidelines.xhtml

61766	C242.00	2	0	0	0	Vitamin A deficiency with corneal xerosis	Feb-09
102237	C244.00	1	0	0	0	Vitamin A deficiency with keratomalacia	May-11
66943	C245.00	8	0	3	0	Vitamin A deficiency with night blindness	Feb-09
45889	C24y.00	1	0	0	0	Vitamin A deficiency with other manifestation	Feb-09
49270	C24y100	10	0	0	0	Vitamin A deficiency with xeroderma	Feb-09
49266	C247000	6	0	0	0	Vitamin A deficiency with xerophthalmia	Feb-09
60199	C246.00	18	0	2	0	Vitamin A deficiency with xerophthalmic corneal scars	Feb-09
42792	C26z.00	228	0	0	0	Vitamin B deficiency NOS	Feb-09
94706	42d6.00	69	0	2	906	Vitamin B profile	Feb-09
4667	C25..11	272	0	91	83	Vitamin B1 deficiency	Feb-09
5271	D011.11	4712	0	201	41	Vitamin B12 deficiency anaemia	Feb-09
32953	D011000	222	0	0	0	Vitamin B12 deficiency anaemia due to dietary causes	Feb-09
24059	C260.12	175	0	4	2	Vitamin B2 deficiency	Feb-09
43354	C261.11	48	0	1	1	Vitamin B6 deficiency	Feb-09
48023	C261.00	39	0	0	0	Vitamin B6 deficiency syndrome	Feb-09
45946	C26..00	343	0	4	2	Vitamin B-complex deficiency	Feb-09
37282	C27..12	134	0	2	2	Vitamin C deficiency	Feb-09
104812	D01y000	2	0	0	0	Vitamin C deficiency anaemia	Aug-12
106409	C2B..00	3201	0	7	2	Vitamin D insufficiency	Jun-13
53111	8B73.00	3033	0	14	2	Vitamin D supplements	Feb-09
105896	C28A.00	9	0	0	0	Vitamin D-dependent rickets	Mar-13
51679	C291000	32	0	0	0	Vitamin E deficiency	Feb-09
62257	D01y100	3	0	0	0	Vitamin E deficiency anaemia	Feb-09
100830	C291100	6	0	0	0	Vitamin P deficiency	Nov-10

For peer review only - http://bmjopen.bmj.com/site/about/guidelines.xhtml

33586	C353200	38	0	3	0	Vitamin-D-resistant rickets	Feb-09
29168	F427C00	384	0	3	0	Vitelliform dystrophy	Feb-09
975	M295100	45691	0	4650	4	Vitiligo	Feb-09
65925	C310211	6	0	0	0	Von Gierke's disease	Feb-09
28443	PK62.00	242	0	10	0	Von Hippel-Lindau syndrome	Feb-09
23137	42Qx.00	0	0	0	507	von Willebrand factor activity	Feb-09
34555	42jf.00	1	0	0	618	von Willebrand factor antigen level	Feb-09
34554	42jg.00	0	0	0	213	von Willebrand factor collagen binding assay	Feb-09
109412	42hB.00	0	0	0	2	von Willebrand Normandy screening test	Mar-15
17520	D304.00	4168	0	166	7	Von Willebrand's disease	Feb-09
108235	C333011	1	0	0	0	Waldenstrom macroglobulinaemia	Jun-14
57509	C330000	10	0	1	0	Waldenstrom's hypergammaglobulinaemic purpura	Feb-09
10411	C333000	887	0	29	1	Waldenstrom's macroglobulinaemia	Feb-09
4810	G754.00	3741	0	128	3	Wegener's granulomatosis	Feb-09
66490	C180.11	4	0	0	0	Wermer's syndrome	Feb-09
57803	C1zy400	2	0	1	0	Werner's syndrome	Feb-09
18636	E011200	415	0	18	0	Wernicke-Korsakov syndrome	Feb-09
51158	ZS78D13	7	0	0	0	Wernicke's aphasia	Feb-09
65074	ZS78D00	6	0	0	0	Wernicke's dysphasia	Feb-09
68256	PJ36.00	1	0	0	0	"Whole chromosome monosomy, meiotic nondisjunction"	Feb-09
95598	PJ37.00	2	0	0	0	"Whole chromosome monosomy, mosaicism"	Feb-09
67815	PJ37z00	1	0	0	0	"Whole chromosome monosomy, mosaicism NOS"	Feb-09
101982	PJ50z00	1	0	0	0	Whole chromosome trisomy syndrome NOS	Apr-11
37702	PJ50.00	21	0	2	0	Whole chromosome trisomy syndromes	Feb-09

For peer review only

107670	PJ50w00	1	0	0	0	"Whole chromosome trisomy, meitotic nondisjunction"	Feb-14
45512	PJ50x00	8	0	0	0	"Whole chromosome trisomy, mosaicism"	Feb-09
32054	PKy9111	72	0	1	0	Wiedemann - Beckwith syndrome	Feb-09
48476	Q317200	26	0	0	0	Wilson-Mikity syndrome	Feb-09
3870	C351011	404	0	28	0	Wilson's disease	Feb-09
31322	C391200	49	0	5	0	Wiskott - Aldrich syndrome	Feb-09
8230	G567400	7350	0	488	1	Wolff-Parkinson-White syndrome	Feb-09
109868	C327300	2	0	0	0	Wolman disease	Jul-15
70109	F151300	7	0	1	0	X-linked bulbo-spinal atrophy	Feb-09
107207	P235.00	1	0	0	0	X-linked hydrocephalus	Nov-13
107983	C353700	7	0	0	0	X-linked hypophosphataemic rickets	May-14
35335	PH15.00	48	0	3	0	X-linked ichthyosis	Feb-09
32782	PJy2.00	67	0	2	0	XXX syndrome	Feb-09
11948	PJy3.00	39	0	0	0	XXY syndrome	Feb-09
41152	PJ64000	21	0	1	0	"XY, female phenotype"	Feb-09
23355	C115.11	239	0	10	0	Zollinger - Ellison syndrome	Feb-09

For peer review only

Appendix 4 ICD10 codes to identify a children with a chronic, not life-limiting condition

ICD 10 code

A15.0

A15.1

A15.2

A15.3

A15.4

A15.5

A15.6

A15.7

A15.8

A15.9

A16.0

A16.1

A16.2

A16.3

A16.4

A16.5

A16.7

A16.8

For peer review only - http://bmjopen.bmj.com/site/about/guidelines.xhtml

A16.9

A18.0

A18.1

A18.2

A18.3

A18.4

A18.5

A18.6

A18.7

A18.8

A19.0

A19.1

A19.2

A19.8

A19.9

A50.0

A50.1

A50.2

A50.3

A50.4

For peer review only - http://bmjopen.bmj.com/site/about/guidelines.xhtml

A50.5

A50.6

A50.7

A50.9

A81.2

A81.8

A81.9

B18.0

B18.1

B18.2

B18.8

B18.9

B37.7

B37.1

B37.5

B37.6

B38.1

B39.1

B40.1

B44.0

For peer review only - http://bmjopen.bmj.com/site/about/guidelines.xhtml

B44.7

B45.0

B45.1

B45.2

B45.3

B45.7

B45.8

B45.9

B46.0

B46.1

B46.2

B46.3

B46.4

B46.5

B46.8

B46.9

B48.7

B50.0

B51.0

B52.0

For peer review only - http://bmjopen.bmj.com/site/about/guidelines.xhtml

B55.0

B55.1

B55.2

B55.9

B57.2

B57.3

B57.4

B57.5

B58.0

B59

B67.0

B67.1

B67.2

B67.3

B67.4

B67.5

B67.6

B67.7

B67.8

B67.9

For peer review only

B69.0

B69.1

B69.8

B69.9

B73

B74.0

B74.1

B74.2

B74.3

B74.4

B74.8

B74.9

B78.7

B90.0

B90.1

B90.2

B90.8

B90.9

B91

B92

For peer review only

For peer review only - http://bmjopen.bmj.com/site/about/guidelines.xhtml

B94.0

B94.1

B94.2

B94.8

B94.9

D00.0

D00.1

D00.2

D01.0

D01.1

D01.2

D01.3

D01.4

D01.5

D01.7

D01.9

D02.0

D02.1

D02.2

D02.3

For peer review only

For peer review only - http://bmjopen.bmj.com/site/about/guidelines.xhtml

D02.4

D05.0

D05.1

D05.7

D05.9

D06.0

D06.1

D06.7

D06.9

D07.0

D07.1

D07.2

D07.3

D07.4

D07.5

D07.6

D09.0

D09.1

D09.2

D09.3

For peer review only

For peer review only - http://bmjopen.bmj.com/site/about/guidelines.xhtml

D09.7

D09.9

D12.0

D12.1

D12.2

D12.3

D12.4

D12.5

D12.6

D12.7

D12.8

D12.9

D13.0

D13.1

D13.2

D13.3

D13.4

D13.5

D13.6

D13.7

For peer review only

For peer review only - http://bmjopen.bmj.com/site/about/guidelines.xhtml

D13.9

D14.1

D14.2

D14.3

D14.4

D15.0

D151

D15.2

D15.7

D15.9

D20.0

D20.1

D32.0

D32.1

D34

D35.0

D35.1

D35.2

D35.3

D35.4

For peer review only
For peer review only - http://bmjopen.bmj.com/site/about/guidelines.xhtml

D35.5

D35.6

D35.7

D35.8

D35.9

D37.0

D37.1

D37.2

D37.3

D37.4

D37.5

D37.6

D37.7

D37.9

D38.0

D38.1

D38.2

D38.3

D38.4

D38.5

For peer review only - http://bmjopen.bmj.com/site/about/guidelines.xhtml

D38.6

D39.0

D39.1

D39.2

D39.7

D39.9

D40.0

D40.1

D40.7

D40.9

D41.0

D41.1

D41.2

D41.3

D41.4

D41.7

D41.9

D42.0

D42.1

D42.9

For peer review only
For peer review only - http://bmjopen.bmj.com/site/about/guidelines.xhtml

D44.0

D44.1

D44.2

D44.3

D44.5

D44.7

D44.8

D44.9

D45

D46.0

D46.1

D46.2

D46.4

D46.5

D46.6

D46.7

D46.9

D47.0

D47.1

D47.2

For peer review only - http://bmjopen.bmj.com/site/about/guidelines.xhtml

D47.3

D47.4

D47.5

D47.7

D47.9

D55.0

D55.1

D55.2

D55.3

D55.8

D55.9

D56.0

D56.2

D56.4

D56.8

D56.9

D57.0

D57.1

D57.2

D57.8

For peer review only - http://bmjopen.bmj.com/site/about/guidelines.xhtml

D58.0

D58.1

D58.2

D58.8

D58.9

D63.0

D63.8

D66

D68.0

D68.1

D68.2

D68.4

D68.5

D68.6

D68.8

D68.9

D69.0

D69.1

D69.2

D69.3

For peer review only - http://bmjopen.bmj.com/site/about/guidelines.xhtml

D69.4

D69.5

D69.6

D69.8

D69.9

D71

D72.0

D72.1

D72.8

D72.9

D73.0

D73.1

D73.2

D73.3

D73.4

D73.5

D73.8

D73.9

D74.0

D74.8

For peer review only
For peer review only - http://bmjopen.bmj.com/site/about/guidelines.xhtml

D74.9

D75.0

D75.1

D75.8

D75.9

D76.1

D76.2

D76.3

D80.0

D80.1

D80.2

D80.3

D80.4

D80.8

D80.9

D82.0

D82.2

D82.3

D82.4

D82.5

For peer review only

D82.6

D82.7

D82.8

D82.9

D84.0

D84.1

D84.8

D84.9

E00.0

E00.1

E00.2

E00.9

E03.0

E03.1

E07.1

E10

E11

E12

E13

E14

For peer review only - http://bmjopen.bmj.com/site/about/guidelines.xhtml

E22.0

E23.0

E24.4

E25.0

E25.8

E25.9

E26.8

E29.1

E31.1

E31.8

E31.9

E34.0

E34.1

E34.2

E34.5

E35.0

E66.0

E66.1

E66.2

E66.8

For peer review only

E66.9

E70.0

E70.3

E70.8

E70.9

E78.0

E78.1

E78.2

E78.3

E78.4

E78.5

E78.6

E78.8

E78.9

E79.8

E79.9

E80.0

E80.1

E80.2

E80.3

For peer review only

For peer review only - http://bmjopen.bmj.com/site/about/guidelines.xhtml

E80.5
E80.7
E83.1
E83.2
E83.3
E83.4
E83.5
E83.8
E83.9
E85.0
E85.1
E85.2
E85.3
E85.4
E85.8
E85.9
E88.2
E88.3
E88.8
E88.9

For peer review only

F00.0

F00.1

F00.2

F00.9

F01.0

F01.1

F01.2

F01.3

F01.8

F01.9

F02.1

F02.2

F02.3

F02.4

F02.8

F03

F04

F05.0

F05.1

F05.8

For peer review only
For peer review only - http://bmjopen.bmj.com/site/about/guidelines.xhtml

F05.9

F06.0

F06.1

F06.2

F06.3

F06.4

F06.5

F06.6

F06.7

F06.8

F07.0

F07.1

F07.2

F07.8

F07.9

F09

F10

F11

F12

F13

For peer review only - http://bmjopen.bmj.com/site/about/guidelines.xhtml

F14

F15

F16

F17

F18

F19

F20.0

F20.1

F20.2

F20.3

F20.4

F20.5

F20.6

F20.8

F20.9

F21

F22.0

F22.8

F22.9

F23.0

For peer review only - http://bmjopen.bmj.com/site/about/guidelines.xhtml

F23.1

F23.2

F23.3

F23.8

F23.9

F24

F25.0

F25.1

F25.2

F25.8

F25.9

F28

F29

F30.0

F30.1

F30.2

F30.8

F30.9

F31.0

F31.1

For peer review only - http://bmjopen.bmj.com/site/about/guidelines.xhtml

F31.2

F31.3

F31.4

F31.5

F31.6

F31.7

F31.8

F31.9

F32.0

F32.1

F32.2

F32.3

F32.8

F32.9

F33.0

F33.1

F33.2

F33.3

F33.4

F33.8

For peer review only

For peer review only - http://bmjopen.bmj.com/site/about/guidelines.xhtml

F33.9

F34.0

F34.1

F34.8

F34.9

F38.0

F38.1

F38.8

F39

F40.0

F40.1

F40.2

F40.8

F40.9

F41.0

F41.1

F41.2

F41.3

F41.8

F41.9

For peer review only

For peer review only - http://bmjopen.bmj.com/site/about/guidelines.xhtml

F42.0

F42.1

F42.2

F42.8

F42.9

F43.0

F43.1

F43.2

F43.8

F43.9

F44.0

F44.1

F44.2

F44.3

F44.4

F44.5

F44.6

F44.7

F44.8

F44.9

For peer review only

F45.0

F45.1

F45.2

F45.3

F45.4

F45.8

F45.9

F48.1

F48.8

F48.9

F50.0

F50.1

F50.2

F50.3

F50.4

F50.5

F50.8

F50.9

F53.0

F53.1

For peer review only - http://bmjopen.bmj.com/site/about/guidelines.xhtml
For peer review only

F53.8

F53.9

F54

F70

F71

F72

F73

F78

F79

F80.0

F80.1

F80.2

F80.8

F80.9

F82

F83

F84.1

F84.3

F84.4

F84.5

For peer review only

For peer review only - http://bmjopen.bmj.com/site/about/guidelines.xhtml

F84.6

F84.8

F84.9

F88

F89

F90.0

F90.1

F90.8

F90.9

F91.0

F91.1

F91.2

F91.3

F91.8

F91.9

F92.0

F92.8

F92.9

F93.0

F93.1

For peer review only

F93.2

F93.3

F93.8

F93.9

F94.0

F94.1

F94.2

F94.8

F94.9

F95.0

F95.1

F95.2

F95.8

F95.9

F98.0

F98.1

F98.2

F98.3

F98.4

F98.5

For peer review only

For peer review only - http://bmjopen.bmj.com/site/about/guidelines.xhtml

F98.6

F98.8

F98.9

G00.0

G00.1

G00.2

G00.3

G00.8

G00.9

G01

G02.0

G02.1

G02.8

G03.0

G03.1

G03.2

G03.8

G03.9

G04.0

G04.1

For peer review only

For peer review only - http://bmjopen.bmj.com/site/about/guidelines.xhtml

G04.2

G04.8

G04.9

G05.0

G05.1

G05.2

G05.8

G06.0

G06.1

G06.2

G07

G08

G09

G11.0

G11.4

G11.8

G11.9

G13.0

G13.1

G13.2

For peer review only

For peer review only - http://bmjopen.bmj.com/site/about/guidelines.xhtml

G13.8

G14

G21.0

G21.1

G21.2

G21.3

G21.4

G21.8

G21.9

G22

G23.1

G23.2

G23.3

G23.9

G24.1

G24.2

G24.3

G24.4

G24.5

G24.8

For peer review only - http://bmjopen.bmj.com/site/about/guidelines.xhtml

G24.9

G25.0

G25.1

G25.2

G25.3

G25.4

G25.5

G25.6

G25.8

G25.9

G26

G30.0

G30.1

G30.8

G30.9

G31.0

G31.1

G31.2

G32.0

G32.8

For peer review only - http://bmjopen.bmj.com/site/about/guidelines.xhtml

G36.0

G36.1

G36.8

G36.9

G37.0

G37.1

G37.2

G37.3

G37.4

G37.5

G37.8

G37.9

G40.0

G40.1

G40.2

G40.3

G40.6

G40.7

G40.8

G40.9

For peer review only - http://bmjopen.bmj.com/site/about/guidelines.xhtml

G41.0

G41.1

G41.2

G41.8

G41.9

G43.0

G43.1

G43.2

G43.3

G43.8

G43.9

G44.0

G44.1

G44.2

G44.3

G44.4

G44.8

G45.0

G45.1

G45.2

For peer review only

For peer review only - http://bmjopen.bmj.com/site/about/guidelines.xhtml

G45.3

G45.4

G45.8

G45.9

G46.0

G46.1

G46.2

G46.3

G46.4

G46.5

G46.6

G46.7

G46.8

G47.0

G47.1

G47.2

G47.3

G47.4

G47.8

G47.9

For peer review only - http://bmjopen.bmj.com/site/about/guidelines.xhtml

G50.0

G50.1

G50.8

G50.9

G51.0

G51.1

G51.2

G51.3

G51.4

G51.8

G51.9

G52.0

G52.1

G52.2

G52.3

G52.7

G52.8

G52.9

G53.0

G53.1

For peer review only

For peer review only - http://bmjopen.bmj.com/site/about/guidelines.xhtml

G53.2

G53.3

G53.8

G54.0

G54.1

G54.2

G54.3

G54.4

G54.5

G54.6

G54.7

G54.8

G54.9

G55.0

G55.1

G55.2

G55.3

G55.8

G56.0

G56.1

For peer review only - http://bmjopen.bmj.com/site/about/guidelines.xhtml

G56.2

G56.3

G56.4

G56.8

G56.9

G57.0

G57.1

G57.2

G57.3

G57.4

G57.5

G57.6

G57.8

G57.9

G58.0

G58.7

G58.8

G58.9

G59.0

G59.8

For peer review only
For peer review only - http://bmjopen.bmj.com/site/about/guidelines.xhtml

G60.2

G60.3

G60.8

G60.9

G61.0

G61.1

G61.8

G61.9

G62.0

G62.1

G62.2

G62.8

G62.9

G63.1

G63.2

G63.3

G63.5

G63.6

G63.8

G64

For peer review only - http://bmjopen.bmj.com/site/about/guidelines.xhtml

G70.0

G70.1

G70.8

G71.8

G71.9

G72.0

G72.1

G72.2

G72.3

G72.4

G72.8

G72.9

G73.0

G73.1

G73.2

G73.3

G73.5

G73.6

G73.7

G80.1

For peer review only

For peer review only - http://bmjopen.bmj.com/site/about/guidelines.xhtml

G80.2

G80.3

G80.4

G80.9

G81.0

G81.1

G81.9

G82.0

G82.1

G82.2

G83.0

G83.1

G83.2

G83.3

G83.4

G83.5

G83.8

G83.9

G90.0

G90.1

For peer review only

G90.2

G90.4

G90.8

G90.9

G91.0

G91.1

G91.2

G91.3

G91.8

G91.9

G92

G93.0

G93.1

G93.2

G93.3

G93.8

G93.9

G94.1

G94.2

G94.8

For peer review only

For peer review only - http://bmjopen.bmj.com/site/about/guidelines.xhtml

G95.0

G95.1

G95.2

G95.8

G95.9

G96.0

G96.1

G96.8

G96.9

G99.0

G99.1

G99.2

G99.8

H05.1

H05.2

H05.3

H05.4

H05.5

H05.8

H05.9

For peer review only - http://bmjopen.bmj.com/site/about/guidelines.xhtml

H13.3

H17.0

H17.1

H17.8

H17.9

H18.0

H18.1

H18.2

H18.3

H18.4

H18.5

H18.6

H18.7

H18.8

H18.9

H19.3

H21.0

H21.2

H21.2

H21.3

For peer review only - http://bmjopen.bmj.com/site/about/guidelines.xhtml

H21.4

H21.5

H21.8

H21.9

H26.0

H26.1

H26.2

H26.3

H26.4

H26.8

H26.9

H27.0

H27.1

H27.8

H27.9

H28.0

H28.1

H28.2

H31.0

H31.1

For peer review only - http://bmjopen.bmj.com/site/about/guidelines.xhtml

H31.2

H31.3

H31.4

H31.8

H31.9

H32.8

H33.0

H33.1

H33.2

H33.3

H33.4

H33.5

H34.0

H34.1

H34.2

H34.8

H34.9

H35.0

H35.1

H35.2

For peer review only

H35.3

H35.4

H35.6

H35.7

H35.8

H35.9

H40.0

H40.1

H40.2

H40.3

H40.4

H40.5

H40.6

H40.8

H40.9

H42.0

H43.0

H43.1

H43.2

H43.3

For peer review only - http://bmjopen.bmj.com/site/about/guidelines.xhtml

H43.8

H43.9

H44.0

H44.1

H44.2

H44.3

H44.4

H44.5

H44.6

H44.7

H44.8

H44.9

H47.0

H47.1

H47.3

H47.4

H47.5

H47.6

H47.7

H54.0

For peer review only - http://bmjopen.bmj.com/site/about/guidelines.xhtml

H54.1

H54.2

H54.4

H60.2

H65.2

H65.3

H65.4

H66.1

H66.2

H66.3

H69.0

H69.8

H69.9

H70.1

H73.1

H74.0

H74.1

H74.2

H74.3

H75.0

For peer review only - http://bmjopen.bmj.com/site/about/guidelines.xhtml

H80.0

H80.1

H80.2

H80.8

H80.9

H81.0

H81.4

H83.0

H83.2

H90.0

H90.3

H90.5

H90.6

H91.0

H91.1

H91.2

H91.3

H91.8

H91.9

I43.1

For peer review only

For peer review only - http://bmjopen.bmj.com/site/about/guidelines.xhtml

I60.0

I60.1

I60.2

I60.3

I60.4

I60.5

I60.6

I60.7

I60.8

I60.9

I61.0

I61.1

I61.2

I61.4

I61.5

I61.6

I61.8

I61.9

I62.0

I62.1

For peer review only

For peer review only - http://bmjopen.bmj.com/site/about/guidelines.xhtml

I62.9

I63.0

I63.1

I63.2

I63.3

I63.4

I63.5

I63.6

I63.8

I63.9

I64

I65.0

I65.1

I65.2

I65.3

I65.8

I65.9

I66.0

I66.1

I66.2

For peer review only

For peer review only - http://bmjopen.bmj.com/site/about/guidelines.xhtml

I66.3

I66.4

I66.8

I66.9

I67.0

I67.1

I67.2

I67.3

I67.4

I67.5

I67.6

I67.7

I67.8

I67.9

I68.0

I68.2

I68.8

I69.0

I69.1

I69.2

For peer review only

For peer review only - http://bmjopen.bmj.com/site/about/guidelines.xhtml

I69.3

I69.4

I69.8

I72.0

I72.5

I72.9

I79.2

J41.0

J41.1

J41.8

J42

J43.0

J43.1

J43.2

J43.8

J43.9

J44.0

J44.1

J44.8

J44.9

For peer review only

For peer review only - http://bmjopen.bmj.com/site/about/guidelines.xhtml

J45.0

J45.1

J45.8

J45.9

J46

J47

J60

J61

J62.0

J62.8

J63.0

J63.1

J63.2

J63.3

J63.4

J63.5

J63.8

J64

J65

J66.0

For peer review only

J66.1

J66.2

J66.8

J67.0

J67.1

J67.2

J67.3

J67.4

J67.5

J67.6

J67.7

J67.8

J67.9

J68.0

J68.1

J68.2

J68.3

J68.4

J68.8

J68.9

For peer review only

J69.0

J69.1

J69.8

J70.0

J70.1

J70.2

J70.3

J70.4

J70.8

J70.9

J80

J81

J82

J84.0

J84.8

J84.9

J85.0

J85.1

J85.2

J85.3

For peer review only - http://bmjopen.bmj.com/site/about/guidelines.xhtml

J86.0

J86.9

J98.0

J98.1

J98.2

J98.3

J98.5

J98.6

J98.8

J98.9

J99.0

J99.1

K20

K21.0

K22.0

K22.1

K22.2

K22.3

K22.4

K22.5

For peer review only - http://bmjopen.bmj.com/site/about/guidelines.xhtml

K22.6

K22.7

K22.8

K22.9

K23.0

K23.1

K23.8

K25

K26

K27

K28

K29.0

K29.1

K29.2

K29.3

K29.4

K29.5

K29.6

K29.7

K29.8

For peer review only - http://bmjopen.bmj.com/site/about/guidelines.xhtml

K29.9

K31.0

K31.1

K31.2

K31.3

K31.4

K31.5

K31.6

K31.7

K31.8

K31.9

K50.0

K50.1

K50.8

K50.9

K51.0

K51.2

K51.3

K51.4

K51.5

For peer review only

For peer review only - http://bmjopen.bmj.com/site/about/guidelines.xhtml

K51.8

K51.9

K52.0

K52.1

K52.2

K52.3

K52.8

K52.9

K55.1

K55.2

K55.8

K57.0

K57.1

K57.2

K57.3

K57.4

K57.5

K57.8

K57.9

K59.2

For peer review only - http://bmjopen.bmj.com/site/about/guidelines.xhtml

K63.0

K63.1

K63.2

K63.3

K66.0

K66.1

K66.8

K66.9

K67.3

K70.0

K70.1

K70.2

K70.3

K70.4

K70.9

K73.0

K73.1

K73.2

K73.8

K73.9

For peer review only - http://bmjopen.bmj.com/site/about/guidelines.xhtml

K75.0

K75.1

K75.2

K75.3

K75.4

K75.8

K75.9

K76.0

K76.1

K76.2

K76.3

K76.4

K76.6

K76.7

K76.9

K80.0

K80.1

K80.2

K80.3

K80.4

For peer review only - http://bmjopen.bmj.com/site/about/guidelines.xhtml

K80.5

K80.8

K81.0

K81.1

K81.8

K81.9

K82.0

K82.1

K82.2

K82.3

K82.4

K82.8

K82.9

K83.0

K83.1

K83.2

K83.3

K83.4

K83.5

K83.8

For peer review only - http://bmjopen.bmj.com/site/about/guidelines.xhtml

For peer review only

K83.9

K85.0

K85.1

K85.2

K85.3

K85.8

K85.9

K86.0

K86.1

K86.2

K86.3

K86.9

K87.0

K90.0

K90.1

K90.2

K90.3

K90.4

K90.8

K90.0

For peer review only - http://bmjopen.bmj.com/site/about/guidelines.xhtml

K93.0

K93.1

L10.0

L10.1

L10.2

L10.3

L10.4

L10.5

L10.8

L10.9

L11.0

L11.8

L11.9

L12.0

L12.1

L12.2

L12.3

L12.8

L12.9

L13.0

For peer review only

For peer review only - http://bmjopen.bmj.com/site/about/guidelines.xhtml

L13.1

L13.8

L13.9

L14

L28.0

L28.1

L28.2

L40.0

L40.1

L40.2

L40.3

L40.4

L40.5

L40.8

L40.9

L41.0

L41.1

L41.3

L41.4

L41.5

For peer review only - http://bmjopen.bmj.com/site/about/guidelines.xhtml

L41.8

L41.9

L42

L43.0

L43.1

L43.2

L43.3

L43.8

L43.9

L44.0

L44.1

L44.2

L44.3

L44.4

L44.8

L44.9

L45

L57.0

L57.1

L57.2

For peer review only - http://bmjopen.bmj.com/site/about/guidelines.xhtml

For peer review only

L57.3

L57.4

L57.5

L57.8

L57.9

L58.1

L59.0

L59.8

L59.9

L62.0

L87.0

L87.1

L87.2

L87.8

L87.9

L88

L90.0

L90.1

L90.2

L90.3

For peer review only - http://bmjopen.bmj.com/site/about/guidelines.xhtml

L90.4

L90.5

L90.6

L90.8

L90.9

L92.0

L92.1

L92.2

L92.3

L92.8

L92.9

L93.0

L93.1

L93.2

L95.0

L95.1

L95.8

L95.9

L98.5

L99.0

For peer review only

M01.1

M03.6

M05.0

M05.1

M05.2

M05.3

M05.8

M05.9

M06.0

M06.1

M06.2

M06.3

M06.4

M06.8

M06.9

M07.0

M07.1

M07.2

M07.3

M07.4

For peer review only

For peer review only - http://bmjopen.bmj.com/site/about/guidelines.xhtml

M07.6

M08.0

M08.1

M08.2

M08.3

M08.4

M08.8

M08.9

M09.0

M09.1

M09.2

M09.8

M10.0

M10.1

M10.2

M10.3

M10.4

M10.9

M11.0

M11.1

For peer review only - http://bmjopen.bmj.com/site/about/guidelines.xhtml

M11.2

M11.8

M11.9

M12.0

M12.1

M12.2

M12.3

M12.4

M12.5

M12.8

M13.0

M13.1

M13.8

M13.9

M14.0

M14.2

M14.3

M14.4

M14.5

M14.6

For peer review only - http://bmjopen.bmj.com/site/about/guidelines.xhtml

M14.8

M30.0

M30.1

M30.2

M30.3

M30.8

M31.0

M31.1

M31.2

M31.4

M31.5

M31.6

M31.7

M31.8

M31.9

M32.0

M32.8

M32.9

M33.0

M33.1

For peer review only - http://bmjopen.bmj.com/site/about/guidelines.xhtml

M33.2

M33.9

M34.0

M34.1

M34.2

M34.8

M34.9

M35.0

M35.1

M35.2

M35.3

M35.4

M35.5

M35.6

M35.7

M35.8

M35.9

M36.0

M36.1

M36.2

For peer review only

M36.3

M36.4

M40.0

M40.1

M40.2

M40.3

M40.4

M40.5

M41.0

M41.1

M41.2

M41.3

M41.4

M41.5

M41.8

M41.9

M42.0

M42.1

M42.9

M43.0

For peer review only - http://bmjopen.bmj.com/site/about/guidelines.xhtml

M43.1

M43.2

M43.3

M43.4

M43.5

M43.6

M43.8

M43.9

M45

M46.0

M46.1

M46.2

M46.3

M46.4

M46.5

M46.8

M46.9

M47.0

M47.1

M47.2

For peer review only - http://bmjopen.bmj.com/site/about/guidelines.xhtml

M47.8

M47.9

M48.0

M48.1

M48.2

M48.3

M48.4

M48.5

M48.8

M48.9

M49.0

M49.5

M50.0

M50.1

M50.2

M50.3

M50.8

M50.9

M51.0

M51.1

For peer review only

For peer review only - http://bmjopen.bmj.com/site/about/guidelines.xhtml

M51.2

M51.3

M51.4

M51.8

M51.9

M53.0

M53.1

M53.2

M53.3

M53.8

M53.9

M54.0

M54.1

M54.2

M54.3

M54.4

M54.5

M54.6

M54.8

M54.9

For peer review only

For peer review only - http://bmjopen.bmj.com/site/about/guidelines.xhtml

M60.0

M60.1

M60.2

M60.8

M60.9

M61.0

M61.1

M61.2

M61.3

M61.4

M61.5

M61.9

M62.0

M62.1

M62.2

M62.3

M62.4

M62.5

M62.6

M62.8

For peer review only

M62.9

M63.8

M80.1

M80.2

M80.3

M80.4

M80.5

M80.8

M80.9

M81.1

M81.2

M81.3

M81.4

M81.5

M81.6

M81.8

M81.9

M82.0

M82.1

M82.8

For peer review only - http://bmjopen.bmj.com/site/about/guidelines.xhtml

M84.0

M84.1

M84.2

M84.8

M84.9

M85.0

M85.1

M85.2

M85.3

M85.4

M85.5

M85.6

M85.8

M85.9

M86.3

M86.4

M86.5

M86.6

M89.0

M89.1

For peer review only - http://bmjopen.bmj.com/site/about/guidelines.xhtml

M89.2

M89.3

M89.4

M89.6

M89.8

M89.9

M90.0

M90.4

M90.6

M90.7

M90.8

M91.0

M91.1

M91.2

M91.3

M91.8

M91.9

M92.0

M92.1

M92.2

For peer review only
For peer review only - http://bmjopen.bmj.com/site/about/guidelines.xhtml

M92.3

M92.4

M92.5

M92.6

M92.7

M92.8

M92.9

M93.0

M93.1

M93.2

M93.8

M93.9

M94.0

M94.1

M94.2

M94.3

M94.8

M94.9

N00

N01

For peer review only - http://bmjopen.bmj.com/site/about/guidelines.xhtml

N02

N03

N04

N05

N07

N08.1

N08.2

N08.3

N08.4

N08.5

N08.8

N11.0

N11.1

N11.8

N11.9

N12

N13.0

N13.1

N13.2

N13.3

For peer review only - http://bmjopen.bmj.com/site/about/guidelines.xhtml

N13.4

N13.5

N13.6

N13.7

N13.8

N13.9

N14.0

N14.1

N14.2

N14.3

N14.4

N15.0

N15.1

N15.8

N15.9

N16.0

N16.1

N16.2

N16.3

N16.4

For peer review only - http://bmjopen.bmj.com/site/about/guidelines.xhtml
For peer review only

N16.5

N16.8

N20.0

N20.1

N20.2

N20.9

N21.0

N21.1

N21.8

N21.9

N22.0

N22.8

N23

N25.0

N25.9

N26

N28.0

N28.1

N28.8

N28.9

For peer review only - http://bmjopen.bmj.com/site/about/guidelines.xhtml

N29.0

N29.8

N31.0

N31.1

N31.2

N31.8

N31.9

N32.0

N32.1

N32.2

N32.3

N32.4

N32.8

N32.9

N33.0

N33.8

N35.0

N35.1

N35.8

N35.9

For peer review only - http://bmjopen.bmj.com/site/about/guidelines.xhtml

N36.0

N36.1

N36.2

N36.3

N36.8

N36.9

N39.1

N39.3

N39.4

N40

N42.0

N42.1

N42.2

N42.3

N42.8

N42.9

N70.1

N70.9

N71.1

N71.9

338

For peer review only

N72

N73.0

N73.1

N73.2

N73.3

N73.4

N73.5

N73.6

N73.8

N73.9

N74.0

N74.1

N74.2

N74.3

N74.4

N74.8

N80.0

N80.1

N80.2

N80.3

For peer review only

For peer review only - http://bmjopen.bmj.com/site/about/guidelines.xhtml

N80.4

N80.5

N80.6

N80.8

N80.9

N81.0

N81.1

N81.2

N81.3

N81.4

N81.5

N81.6

N81.8

N81.9

N82.0

N82.1

N82.2

N82.3

N82.4

N82.5

For peer review only

For peer review only - http://bmjopen.bmj.com/site/about/guidelines.xhtml

N82.8

N82.9

N85.0

N85.1

N85.2

N85.3

N85.4

N85.5

N85.6

N85.7

N85.8

N85.9

N87.0

N87.1

N87.2

N87.9

N88.0

N88.1

N88.2

N88.3

For peer review only

For peer review only - http://bmjopen.bmj.com/site/about/guidelines.xhtml

N88.4

N88.8

N88.9

O35.4

P10.0

P10.2

P10.3

P10.4

P10.8

P10.9

P27.0

P27.1

P27.8

P27.9

P35.2

P35.9

P37.0

P37.1

P52.0

P52.1

For peer review only

For peer review only - http://bmjopen.bmj.com/site/about/guidelines.xhtml

P52.2

P52.3

P52.5

P52.6

P52.8

P52.9

P57.0

P57.8

P57.9

P75

P90

P91.1

P96.0

Q00.1

Q00.1

Q02

Q03.0

Q03.8

Q04.1

Q04.5

For peer review only - http://bmjopen.bmj.com/site/about/guidelines.xhtml

Q04.8

Q06.0

Q06.1

Q06.2

Q06.3

Q06.4

Q06.8

Q06.9

Q07.8

Q07.9

Q10.4

Q10.7

Q11.0

Q11.1

Q11.2

Q11.3

Q12.0

Q12.1

Q12.2

Q12.3

For peer review only - http://bmjopen.bmj.com/site/about/guidelines.xhtml

For peer review only

Q12.4

Q12.8

Q12.9

Q13.0

Q13.1

Q13.2

Q13.3

Q13.4

Q13.8

Q13.9

Q14.0

Q14.1

Q14.2

Q14.3

Q14.8

Q14.9

Q15.0

Q15.8

Q15.9

Q16.0

For peer review only

For peer review only - http://bmjopen.bmj.com/site/about/guidelines.xhtml

Q16.1

Q16.2

Q16.3

Q16.4

Q16.5

Q16.9

Q18.8

Q20.1

Q20.2

Q20.5

Q21.0

Q21.1

Q21.2

Q21.4

Q21.9

Q22.2

Q22.3

Q22.8

Q22.9

Q23.1

For peer review only

For peer review only - http://bmjopen.bmj.com/site/about/guidelines.xhtml

Q23.3

Q23.8

Q24.0

Q24.1

Q24.2

Q24.3

Q24.4

Q24.5

Q24.6

Q24.8

Q24.9

Q25.0

Q25.1

Q25.2

Q25.3

Q25.7

Q25.8

Q25.9

Q26.0

Q26.1

For peer review only - http://bmjopen.bmj.com/site/about/guidelines.xhtml

Q26.3
Q26.5
Q26.9
Q27.0
Q27.1
Q27.2
Q27.3
Q27.4
Q27.8
Q27.9
Q28.0
Q28.1
Q28.3
Q28.9
Q30.0
Q30.1
Q30.2
Q30.3
Q30.8
Q30.9

For peer review only
For peer review only - http://bmjopen.bmj.com/site/about/guidelines.xhtml

Q31.0

Q31.1

Q31.2

Q31.3

Q31.5

Q31.8

Q31.9

Q32.0

Q32.2

Q32.3

Q32.4

Q33.0

Q33.1

Q33.2

Q33.3

Q33.4

Q33.5

Q33.8

Q33.9

Q34.0

For peer review only - http://bmjopen.bmj.com/site/about/guidelines.xhtml

Q34.1

Q34.8

Q34.9

Q35.1

Q35.3

Q35.5

Q35.7

Q35.9

Q36.0

Q36.1

Q36.9

Q37.0

Q37.1

Q37.2

Q37.3

Q37.4

Q37.5

Q37.8

Q37.9

Q38.0

For peer review only

For peer review only - http://bmjopen.bmj.com/site/about/guidelines.xhtml

Q38.3

Q38.4

Q38.6

Q38.7

Q38.8

Q39.0

Q39.1

Q39.2

Q39.3

Q39.4

Q39.5

Q39.8

Q39.9

Q40.2

Q40.3

Q40.8

Q41.1

Q41.2

Q41.8

Q42.0

For peer review only

Q42.1

Q42.2

Q42.3

Q42.8

Q42.9

Q43.1

Q43.3

Q43.4

Q43.5

Q43.6

Q43.9

Q44.0

Q44.1

Q44.3

Q44.4

Q44.5

Q44.7

Q45.0

Q45.1

Q45.2

For peer review only

For peer review only - http://bmjopen.bmj.com/site/about/guidelines.xhtml

Q45.3

Q45.8

Q45.9

Q50.0

Q51.0

Q51.1

Q51.2

Q51.3

Q51.4

Q51.5

Q51.6

Q51.7

Q51.8

Q51.9

Q52.1

Q52.2

Q52.4

Q54.0

Q54.1

Q54.2

For peer review only

For peer review only - http://bmjopen.bmj.com/site/about/guidelines.xhtml

Q54.3

Q54.8

Q54.9

Q55.0

Q55.5

Q56.0

Q56.1

Q56.2

Q56.3

Q56.4

Q60.2

Q60.4

Q60.5

Q61.0

Q61.1

Q61.2

Q61.3

Q61.5

Q61.8

Q62.0

For peer review only

For peer review only - http://bmjopen.bmj.com/site/about/guidelines.xhtml

Q62.1

Q62.3

Q62.4

Q62.5

Q62.6

Q62.8

Q63.0

Q63.1

Q63.2

Q63.8

Q63.9

Q64.0

Q64.1

Q64.3

Q64.4

Q64.5

Q64.6

Q64.7

Q64.8

Q64.9

For peer review only

Q65.0
Q65.1
Q65.2
Q65.8
Q65.9
Q67.5
Q68.2
Q71.0
Q71.1
Q71.2
Q71.3
Q71.4
Q71.5
Q71.6
Q71.8
Q71.9
Q72.0
Q72.1
Q72.2
Q72.3

For peer review only

For peer review only - http://bmjopen.bmj.com/site/about/guidelines.xhtml

Q72.4

Q72.5

Q72.6

Q72.7

Q72.8

Q72.9

Q73.0

Q73.1

Q73.8

Q74.0

Q74.1

Q74.2

Q74.8

Q74.9

Q75.1

Q75.3

Q75.4

Q75.5

Q75.8

Q75.9

For peer review only

Q76.1

Q76.2

Q76.3

Q76.4

Q77.0

Q77.1

Q77.5

Q77.6

Q77.7

Q77.8

Q77.9

Q78.0

Q78.1

Q78.2

Q78.3

Q78.4

Q78.6

Q78.8

Q79.0

Q79.4

For peer review only

For peer review only - http://bmjopen.bmj.com/site/about/guidelines.xhtml

Q79.5

Q79.6

Q79.8

Q80.0

Q80.1

Q80.2

Q80.3

Q80.8

Q80.9

Q82.0

Q82.2

Q82.3

Q82.9

Q85.0

Q85.9

Q85.0

Q86.1

Q86.2

Q86.8

Q87.3

For peer review only - http://bmjopen.bmj.com/site/about/guidelines.xhtml

Q87.4

Q87.5

Q89.1

Q89.2

Q89.3

Q89.4

Q89.7

Q89.8

Q89.9

Q90.0

Q90.1

Q90.2

Q90.9

Q92.2

Q92.3

Q92.5

Q92.6

Q92.9

Q93.0

Q93.1

For peer review only - http://bmjopen.bmj.com/site/about/guidelines.xhtml

Q93.6

Q93.7

Q93.9

Q97.0

Q97.1

Q97.2

Q97.3

Q97.8

Q97.9

Q98.0

Q99.0

Q99.1

Q99.2

Q99.8

Q99.9

For peer review only

For peer review only - http://bmjopen.bmj.com/site/about/guidelines.xhtml

STROBE Statement—Checklist of items that should be included in reports of ***cohort studies***

	Item No	Recommendation	Page No
**Title and abstract**	1	(*a*) Indicate the study's design with a commonly used term in the title or the abstract<br>(*b*) Provide in the abstract an informative and balanced summary of what was done and what was found	1
**Introduction**			
Background/rationale	2	Explain the scientific background and rationale for the investigation being reported	3-4
Objectives	3	State specific objectives, including any prespecified hypotheses	4
**Methods**			
Study design	4	Present key elements of study design early in the paper	5 + abstract + Title
Setting	5	Describe the setting, locations, and relevant dates, including periods of recruitment, exposure, follow-up, and data collection	5
Participants	6	(*a*) Give the eligibility criteria, and the sources and methods of selection of participants. Describe methods of follow-up<br>(*b*) For matched studies, give matching criteria and number of exposed and unexposed	5-7 + Table 1
Variables	7	Clearly define all outcomes, exposures, predictors, potential confounders, and effect modifiers. Give diagnostic criteria, if applicable	8 -9
Data sources/ measurement	8*	For each variable of interest, give sources of data and details of methods of assessment (measurement). Describe comparability of assessment methods if there is more than one group	8 -9
Bias	9	Describe any efforts to address potential sources of bias	9-10
Study size	10	Explain how the study size was arrived at	5,10
Quantitative variables	11	Explain how quantitative variables were handled in the analyses. If applicable, describe which groupings were chosen and why	9-10
Statistical methods	12	(*a*) Describe all statistical methods, including those used to control for confounding<br>(*b*) Describe any methods used to examine subgroups and interactions<br>(*c*) Explain how missing data were addressed<br>(*d*) If applicable, explain how loss to follow-up was addressed<br>(*e*) Describe any sensitivity analyses	9-10
**Results**			
Participants	13*	(a) Report numbers of individuals at each stage of study—eg numbers potentially eligible, examined for eligibility, confirmed eligible, included in the study, completing follow-up, and analysed<br>(b) Give reasons for non-participation at each stage<br>(c) Consider use of a flow diagram	
Descriptive data	14*	(a) Give characteristics of study participants (eg demographic, clinical, social) and information on exposures and potential confounders<br>(b) Indicate number of participants with missing data for each variable of interest<br>(c) Summarise follow-up time (eg, average and total amount)	
Outcome data	15*	Report numbers of outcome events or summary measures over time	

For peer review only

For peer review only - http://bmjopen.bmj.com/site/about/guidelines.xhtml

For peer review only

For peer review only - http://bmjopen.bmj.com/site/about/guidelines.xhtml

Main results	16	(*a*) Give unadjusted estimates and, if applicable, confounder-adjusted estimates and their precision (eg, 95% confidence interval). Make clear which confounders were adjusted for and why they were included<br>(*b*) Report category boundaries when continuous variables were categorized<br>(*c*) If relevant, consider translating estimates of relative risk into absolute risk for a meaningful time period	
Other analyses	17	Report other analyses done—eg analyses of subgroups and interactions, and sensitivity analyses	
**Discussion**			
Key results	18	Summarise key results with reference to study objectives	
Limitations	19	Discuss limitations of the study, taking into account sources of potential bias or imprecision. Discuss both direction and magnitude of any potential bias	
Interpretation	20	Give a cautious overall interpretation of results considering objectives, limitations, multiplicity of analyses, results from similar studies, and other relevant evidence	
Generalisability	21	Discuss the generalisability (external validity) of the study results	
**Other information**			
Funding	22	Give the source of funding and the role of the funders for the present study and, if applicable, for the original study on which the present article is based	

*Give information separately for exposed and unexposed groups.

Note: An Explanation and Elaboration article discusses each checklist item and gives methodological background and published examples of transparent reporting. The STROBE checklist is best used in conjunction with this article (freely available on the Web sites of PLoS Medicine at http://www.plosmedicine.org/, Annals of Internal Medicine at http://www.annals.org/, and Epidemiology at http://www.epidem.com/). Information on the STROBE Initiative is available at http://www.strobe-statement.org.

For peer review only

For peer review only - http://bmjopen.bmj.com/site/about/guidelines.xhtml

BMJ Open

The health of mothers of children with a life-limiting condition; a protocol for comparative cohort study using the Clinical Practice Research Datalink

Journal:	*BMJ Open*
Manuscript ID	bmjopen-2019-034024.R2
Article Type:	Protocol
Date Submitted by the Author:	09-Jun-2020
Complete List of Authors:	Fraser, Lorna; University of York, Health Sciences<br>Murtagh, Fliss; Wolfson Palliative Care Research Centre, Hull York Medical School, University of Hull, UK,<br>Sheldon, Trevor; University of York, health Sciences<br>Gilbody, Simon; The University of York, Department of Health Sciences<br>Hewitt, Catherine; The University of York, Health Sciences
**Primary Subject Heading**:	General practice / Family practice
Secondary Subject Heading:	Paediatrics
Keywords:	Developmental neurology & neurodisability < PAEDIATRICS, Life-limiting, maternal health

SCHOLARONE™
Manuscripts

I, the Submitting Author has the right to grant and does grant on behalf of all authors of the Work (as defined in the below author licence), an exclusive licence and/or a non-exclusive licence for contributions from authors who are: i) UK Crown employees; ii) where BMJ has agreed a CC-BY licence shall apply, and/or iii) in accordance with the terms applicable for US Federal Government officers or employees acting as part of their official duties; on a worldwide, perpetual, irrevocable, royalty-free basis to BMJ Publishing Group Ltd ("BMJ") its licensees and where the relevant Journal is co-owned by BMJ to the co-owners of the Journal, to publish the Work in this journal and any other BMJ products and to exploit all rights, as set out in our licence.

The Submitting Author accepts and understands that any supply made under these terms is made by BMJ to the Submitting Author unless you are acting as an employee on behalf of your employer or a postgraduate student of an affiliated institution which is paying any applicable article publishing charge ("APC") for Open Access articles. Where the Submitting Author wishes to make the Work available on an Open Access basis (and intends to pay the relevant APC), the terms of reuse of such Open Access shall be governed by a Creative Commons licence – details of these licences and which Creative Commons licence will apply to this Work are set out in our licence referred to above.

Other than as permitted in any relevant BMJ Author's Self Archiving Policies, I confirm this Work has not been accepted for publication elsewhere, is not being considered for publication elsewhere and does not duplicate material already published. I confirm all authors consent to publication of this Work and authorise the granting of this licence.

For peer review only

TITLE: The health of mothers of children with a life-limiting condition; a protocol for comparative cohort study using the **Clinical Practice Research Datalink**

Fraser LK[1,2], Murtagh FEM[3], Sheldon TA[1], Gilbody S[1], Hewitt CE[1]

[1] Department of Health Sciences, University of York, UK

[2]Martin House Research Centre, University of York, UK

[3] Wolfson Palliative Care Centre, Hull York Medical School, Hull, UK

Corresponding Author:

Dr Lorna Fraser

Department of Health Sciences

Area 2 Seebohm Rowntree Building

Heslington

YORK

YO10 5DD

lorna.fraser@york.ac.uk

01904 321889

Word Count: 2489 words.

Tables :1

For peer review only

For peer review only - http://bmjopen.bmj.com/site/about/guidelines.xhtml

ABSTRACT

Introduction

There are now nearly 50,000 children with a life-limiting or life-threatening conditions in the UK. These include conditions where there is no reasonable hope of cure and from which they will die, as well as conditions for which curative treatment may be feasible but can fail, e.g. cancer or heart failure. Having a child with a life-limiting condition involves being a coordinator and provider of healthcare in addition to the responsibilities and pressures of parenting a child who is expected to die young. This adversely affects the health and wellbeing of these mothers and affects their ability to care for their child, but the extent of the impact is poorly understood.

This study aims to quantify the incidence and nature of mental and physical morbidity in mothers of children with a life-limiting condition, their healthcare use and to assess whether there is a relationship between the health of the mother and the child's condition.

Methods and analysis

A comparative cohort study using data from the Clinical Practice Research Datalink (CPRD) and linked hospital data will include three groups of children and their mothers (those with a life-limiting condition, those with a chronic conditions and those with no long term health condition total =20,000 mother-child dyads). Incidence rates and incidence rate ratios will be used to quantify and compare the outcomes between groups with multivariable regression modelling used to assess the relationship between the child's disease trajectory and mother's health.

Ethics and dissemination

This study protocol has approval from the Independent Scientific Advisory Committee for the UK Medicines and Healthcare products Regulatory Agency Database Research. The results of this study will be reported according to the STROBE and RECORD guidelines. There will also

For peer review only - http://bmjopen.bmj.com/site/about/guidelines.xhtml

be a lay summary for parents which will be available to download from the Martin House Research Centre website (www.york.ac.uk/mhrc).

For peer review only

For peer review only - http://bmjopen.bmj.com/site/about/guidelines.xhtml

ARTICLE SUMMARY

Strengths and Limitations of this study

This study is utilising a large, longitudinal, nationally representative primary care data source.

The Clinical Practice Research Datalink data enables the identification of mother and child dyads and linkage to secondary care data.

This study is reliant on identification of the cohort of interest by diagnostic coding therefore on the quality of diagnostic coding within the CPRD and HES datasets.

INTRODUCTION

The number of children with life-limiting or life-threatening conditions has been rising, with latest figures estimating 49,000 children and young people with a life-limiting condition in the UK.[1] These include conditions for which there is no reasonable hope of cure and from which children or young people will die, as well as conditions for which curative treatment may be feasible but can fail, such as cancer or heart failure. In children and young people, more than 300 diagnoses are life-limiting or life-threatening[2] , including duchenne muscular dystrophy, severe cerebral palsy, neurodegenerative conditions, and severe congenital anomalies.

Although many of the individual diagnoses are rare, collectively life-limiting conditions affect more children and young people than more common single long term conditions, such as diabetes mellitus[3] . Many of these children are living longer due to the use of medical technologies, e.g. ventilation and gastrostomy feeding, and more aggressive treatment of complications and they are often high users of healthcare services[4] . The parents of these children have an added layer of 'pressure' from having a child with a chronic condition who also has a shortened life expectancy[5] . Having a child with a life-limiting condition often involves being a coordinator and provider of healthcare as well as being a parent, and this responsibility exists 24 hours a day, 7 days a week, especially for mothers. The health of these mothers is important, both for their own well-being, and also in terms of their ability to care

For peer review only - http://bmjopen.bmj.com/site/about/guidelines.xhtml

for their child. Currently the National Health Service and statutory services for these children rarely extend to providing care for the parents. The focus is on mothers rather than fathers in this study as mothers are the main carer for the majority of these children.

The need for further research on the emotional and psychological support and interventions for parents or carers of children with a life-limiting condition was highlighted recently in National Institute for Health and Clinical Excellence (NICE) guidelines on end of life care in children and young people[6] . This stated that research was needed on 'What emotional support do children and young people with life-limiting conditions and their parents or carers need, and how would they like these needs to be addressed? The NICE guidelines[6] also noted that no studies had quantified the psychological/ mental health of mothers of children with life-limiting condition. There have been some attempts to quantify these in children with special needs[7] or specific disabilities[8 9] which have shown higher levels of parental distress or emotional problems than parents of healthy children (36% cf 20%). These however, do not address the specific needs of those with life-limiting conditions or the added burden that parents of children with a life-limiting condition face, knowing their child is likely to die.

Evidence about the physical health of mothers with life-limiting condition is also sparse, although two studies in mothers of children with disabilities found higher prevalence of physical conditions compared to mothers of healthy children. For example, prevalence of back pain and hypertension was 35.2% and 24.7% respectively in mothers of children with disabilities, compared to 26.7% and 19.1% in mothers of healthy children. However, these studies were cross sectional, self-reported and were not explicitly for mothers of children with a life-limiting condition [9 10]. To date, no study using a nationally representative sample has assessed the incidence or prevalence of mental and physical health conditions in mothers of children with a life-limiting condition. Quantifying the extent and nature of both physical and mental health conditions in these mothers is important when trying to develop and target appropriate preventative or treatment interventions for this population.

This study aims to quantify the incidence and nature of mental and physical morbidity in mothers of children with a life-limiting condition, their healthcare use and to assess whether there is a relationship between the health of the mother and the child's condition.

For peer review only - http://bmjopen.bmj.com/site/about/guidelines.xhtml

METHODS AND ANALYSIS

Research Questions

1. What is the nature and incidence of mental and physical morbidity in mothers of children with a life-limiting condition?

2. What is the relationship between the health of the mother and the child's condition?

3. How many primary care visits do these mothers have compared to other mothers?

4. How many hospital admissions do these mothers have compared to other mothers?

5. What are the resource costs of healthcare for these mothers?

Data sources and sample selection

This will be an observational comparative cohort study design using data from the Clinical Practice Research Datalink (CPRD). The CPRD dataset contains anonymized, longitudinal records of primary care from a representative sample of GP practices across the UK (covering approximately 8.5% of the UK population)[11]. Individuals within the primary care CPRD dataset are also linked to secondary care data (inpatient, outpatient, A & E Hospital Episodes Statistics (HES) and the Mental Health Minimum Dataset (MHMDS))[12 13] and Office for National Statistics death certificate data. The CPRD provides a unique opportunity to assess the relationship between maternal morbidity and their child health due to the longitudinal nature of data collection (since 1987) and the ability to link mothers and children via their mother-baby link algorithm[14]. It is not currently possible to link babies to fathers in this dataset.

The index children will be extracted from the CPRD mother baby linkage, where the mother has at least 1 years registration and where they eligible for HES linkage (resident in England). Children will be grouped in to those with a life-limiting condition (n~5000) or chronic condition that is not life-limiting e.g. diabetes, asthma (n~5000) and they will be identified within the CPRD dataset between 2007-2017, using READ codes in the CPRD data and ICD10 codes in the HES data[1] (see Table 1) . The development of these ICD-10 code list have been

For peer review only - http://bmjopen.bmj.com/site/about/guidelines.xhtml

described previously for life-limiting[1] and chronic conditions[15]. Subjects will be matched by year of birth, sex and region to the control group of healthy children (n~10,000) with no long term conditions.

All primary and the linked secondary care data will be extracted for each mother and child dyad.

Table 1 Cohort identification processes

Groups	Inclusion criteria	Exclusion Criteria
1. Life-limiting or life-threatening condition	• Children from the source population who have prevalent diagnosis event records for life-limiting conditions, either in the Clinical or Referral files in CPRD GOLD based on the Read codes detailed in Supplementary material, or in HES APC based on the ICD-10 codes in Supplementary material.<br>• Children have above events before the end of the study period (31/12/2017).<br>• Children have above events within their UTS follow-up period.<br>• Children are aged 18 years or less on the diagnosis event date.	None
2. Chronic condition that is not life-limiting	One matching control will be provided for each case in Group 1. The controls will comprise of patients from the source population who fulfil the following criteria:<br>• Children from the source population who have diagnosis event records for other chronic conditions in the Clinical or Referral files in CPRD GOLD based	•Children with a diagnosis on or before 31/12/2017 of life-limiting conditions, either in the Clinical or Referral files in CPRD GOLD based on the Read codes detailed in Appendix 1, or in HES APC based on the ICD-10 codes in Appendix 2.

For peer review only - http://bmjopen.bmj.com/site/about/guidelines.xhtml

	on the Read codes detailed in Supplementary material, or in HES APC based on the ICD-10 codes in Supplementary material.<br>• Children have above events before the end of the study period of (31/12/2017).<br>• Children have above events within their UTS follow-up period.<br>• Children are aged 18 years or less on the diagnosis date.	• Children who have deregistered from the CPRD on or before 01/04/2007<br><br>• Children who are siblings of children in the case population (Group 1), based on having a link to the same mother in the Mother Baby Link
3. No long term conditions	Up to two matching controls will be provided for each case in Group 1. The controls will comprise of patients from the source population who fulfil the following criteria:<br><br>• Children from the source population who have at least one day of registration during follow-up start and end.	• Children with a diagnosis of life-limiting conditions on or before 31/12/2017, either in the Clinical or Referral files in CPRD GOLD based on the Read codes detailed in Appendix 1, or in HES APC based on the ICD-10 codes in Appendix 2, will be excluded<br>• Children with a diagnosis of other chronic conditions on or before 31/12/2017, in the Clinical or Referral files in CPRD GOLD based on the Read codes detailed in Appendix 3, or in HES APC based on the ICD-10 codes in Appendix 4, will be excluded.<br>• Children who have deregistered from the CPRD on or before 01/04/2007<br>• Children who are siblings of children in the case population (Group 1), based on

For peer review only - http://bmjopen.bmj.com/site/about/guidelines.xhtml

		having a link to the same mother in the Mother Baby Link

Sample size

A sample size calculation based on 80% power, 5% significance to detect

an incidence rate ratio of 1.4 (with mean incidence rate of 0.03 in maternal anxiety/depression) indicated a minimum of 3,260 participants required in each group. The final cohort includes all children with a life-limiting condition in the CPRD dataset. The cohort for analyses contained a total of 35,683 mothers, of whom 8,950 had a child with a life-limiting condition, 8,868 had a child with a chronic condition and 17,865 had a child with no long-term condition.

Exposures

The key exposure is the disease status of the child (life-limiting condition/chronic disease (asthma or diabetes)/ no long-term condition). These groups will be identified using previously developed READ and ICD 10 diagnostic code lists (see supplementary material). The use of both READ and ICD 10 codes will enable identification of relevant diagnoses from either the primary care records (READ) or hospital admission data (ICD 10).

Outcomes

The outcomes for the mothers will be recorded in either the primary care data or the linked secondary care datasets. The dates of these outcomes will be assessed in relation to the date of the child's diagnoses.

For peer review only - http://bmjopen.bmj.com/site/about/guidelines.xhtml

Incidence of mental health and physical health conditions

- Maternal mental health diagnoses, including both common mental illness and severe mental illness. These will be identified using diagnostic READ codes and relevant prescription data. A previously developed READ code algorithm[16] will be utilised. The linked MHMDS will indicate the presence of a more serious mental health conditions.
- Maternal physical diagnoses, including e.g. obesity, hypertension, musculoskeletal problems, and cardiovascular disease. These will be identified using diagnostic READ codes, relevant prescription data, HES data, and related biometric data (including blood pressure, BMI, and total cholesterol).

The association of maternal health with the child's disease trajectory

- The number of A&E attendances, hospital admissions and length of stay for the children (HES data).

Healthcare use and resources of the mother

- Referrals to other services, especially secondary services for mothers (CPRD and HES data).
- Uptake of cervical screening by mothers (CPRD data).
- The number of primary care attendances (GP, practice nurse etc.) per year by mothers (CPRD data)
- The number and nature of prescribed medication for mothers (Prescription data).
- The number of A&E attendances, hospital admissions and length of stay for mothers (HES data).

Confounding

The following clinical and demographic confounders will be considered in the analyses:

- Ethnicity, this will be classified according to the census 2011 categories[17] using data from CPRD and HES data.
- Pre-existing comorbidities of the mother

For peer review only

- Child's diagnoses: grouped into disease categories[1]
- Socioeconomic data is based on the index of multiple deprivation category based on the local superoutput area of residence[18]

- Region of residence (recognising that the geographical coverage of CPRD is not uniform across England).

Statistical methods

All analyses will be undertaken using STATA version 15[19]. All analyses will adjust for known clinical and demographic confounders of importance (age, co-morbidities, socioeconomic status, previous use of primary care services, etc.). The left and right censored nature of these data will be taken into account when undertaking these analyses.

Incidence of mental health and physical health conditions

Incidence rates of the physical and mental health conditions will be calculated in each group of mothers by dividing the number of cases in each group by the person-time at risk in each group. Incident cases will be counted only after the diagnoses in the child. A comparison with the incidence of these conditions in the three groups of mothers will be undertaken using incidence rate ratios.

The association of maternal health with the child's disease trajectory

The presence of physical or mental health diagnoses in the mother will be the outcome variable of interest in a multivariable model with a key covariate being the presence of prolonged or repeated hospital admission for the child. A prolonged admission is one that lasts > 14 days and repeated admissions will be defined as more than 2 admissions in a 6 month time period.

Healthcare use and resources of the mother

For peer review only - http://bmjopen.bmj.com/site/about/guidelines.xhtml

Using multivariable Poisson regression modelling for the count of GP visits, referrals and hospital admissions with the key exposure of disease status of the child (life-limiting condition /Chronic disease/ no long-term condition). Multilevel modelling will be used to account for repeated measures within individuals. For the analyses of healthcare costs, we will use information from Unit Costs of Health and Social Care[20] , the NHS national tariffs[21] and Department of Health Reference Costs[22] .

Missing data

We expect there to be missing data for some of the key covariates in the data e.g. ethnicity. We will assess the level of missingness across all sources of these variable (HES and CPRD). If appropriate then we will use multiple imputation techniques [23] to address this in order to avoid the potential bias from only undertaking complete case analyses.

PATIENT AND PUBLIC INVOLVEMENT

The views of parents and carers of children with a life-limiting condition have informed the development of this study. Initial ideas about the topic area and planned study were discussed with a group of 10 parents of children with a life-limiting condition at Martin House Children's Hospice. They helped to refine the research question and future stages of this programme of work. The Family Advisory Board of the Martin House Research Centre (www.york.ac.uk/mhrc) will have involvement in this study with an emphasis on the preparation of the lay summaries and dissemination to parents and children.

ETHICS AND DISSEMINATION

This study protocol has approval from the Independent Scientific Advisory Committee for the UK Medicines and Healthcare products Regulatory Agency Database Research (protocol 18_313). They have NREC approval for release of pseudonymised data for observational research.

This study will be complete by Feb 2021.

For peer review only - http://bmjopen.bmj.com/site/about/guidelines.xhtml

The results of this study will be reported according to the STROBE and RECORD guidelines (ref). There were also be a lay summary for parents which will be available to download from the Martin House Research Centre website (www.york.ac.uk/mhrc).

Authors' contributions: LF had the original idea for this study and wrote the first draft of this manuscript. FM, SG, TS and CH contributed to the development of this idea, study design and revised the manuscript. All authors approved the final submitted version of this manuscript.

Funding statement: This paper is independent research arising from a Career Development Fellowship held by Lorna Fraser (CDF-2018-11-ST2-002) supported by the National Institute for Health Research. The views expressed in this publication are those of the author(s) and not necessarily those of the NHS, the National Institute for Health Research or the Department of Health.

Competing interests statement: none declared

Ethics approval: This study protocol has approval from the Independent Scientific Advisory Committee for the UK Medicines and Healthcare products Regulatory Agency Database Research (protocol 18_313).

Data sharing statement: the clinical codes used for this study are provided as supplementary material.

For peer review only - http://bmjopen.bmj.com/site/about/guidelines.xhtml

REFERENCES

1. Fraser LK, Miller M, Hain R, et al. Rising National Prevalence of Life-Limiting Conditions in Children in England. *Pediatrics* 2012;129(4):E923-E29. doi: 10.1542/peds.2011-2846

2. Hain R, Devins M, Hastings R, et al. Paediatric palliative care: development and pilot study of a 'Directory' of life-limiting conditions. *Bmc Palliative Care* 2013;12 doi: 10.1186/1472-684x-12-43

3. Royal College of Paediatrics and Child Health. National Paediatric Diabetes Audit, 2014.

4. Feudtner C, DiGiuseppe DL, Neff JM. Hospital care for children and young adults with complex chronic conditions in the last year of life. *Pediatric Research* 2003;53(4):266A-66A.

5. Fraser LK, Jarvis SW, Moran N, et al. Children in Scotland requiring Palliative Care: identifying numbers and needs (The ChiSP Study). York, UK: University of York, 2015.

6. National Institute for Health and Crae Excellence (NICE). End of life care for infants, children and young people with life-limiting conditions: planning and management, 2016.

7. Thurston S, Paul L, Loney P, et al. Associations and costs of parental symptoms of psychiatric distress in a multi-diagnosis group of children with special needs. *Journal of Intellectual Disability Research* 2011;55:263-80. doi: 10.1111/j.1365-2788.2010.01356.x

8. Brehaut JC, Kohen DE, Raina P, et al. The Health of Primary Caregivers of Children With Cerebral Palsy: How Does It Compare With That of Other Canadian Caregivers? *Pediatrics* 2004;114(2):e182-e91. doi: 10.1542/peds.114.2.e182

9. Lach LM, Kohen DE, Garner RE, et al. The health and psychosocial functioning of caregivers of children with neurodevelopmental disorders. *Disability and Rehabilitation* 2009;31(8):607-18. doi: 10.1080/09638280802242163

10. Lee MH, Park C, Matthews AK, et al. Differences in physical health, and health behaviors between family caregivers of children with and without disabilities. *Disability and Health Journal* 2017;10(4):565-70. doi: 10.1016/j.dhjo.2017.03.007

11. Herrett E, Gallagher AM, Bhaskaran K, et al. Data Resource Profile: Clinical Practice Research Datalink (CPRD). *International Journal of Epidemiology* 2015;44(3):827-36. doi: 10.1093/ije/dyv098

12. Clinical Practice Research Datalink. Hospital Episode Statistics (HES) Admitted Patient Care and GOLD Documentation (Set 14): Medicines & Healthcare products Regulatory Agency, 2017.

For peer review only

13. Health & Social Care Information Centre. Methodology for creation of the HES Patient ID (HESID), 2014.

14. CPRD. Clinical Practice Research Datalink 2014 [Available from: http://www.cprd.com/intro.asp accessed 29/01/2014.

15. Hardelid P, Dattani N, Gilbert R. Estimating the prevalence of chronic conditions in children who die in England, Scotland and Wales: a data linkage cohort study. *BMJ Open* 2014;4(8):e005331. doi: 10.1136/bmjopen-2014-005331

16. Prady SL, Pickett KE, Petherick ES, et al. Evaluation of ethnic disparities in detection of depression and anxiety in primary care during the maternal period: combined analysis of routine and cohort data. *British Journal of Psychiatry* 2016;208(5):453-61. doi: 10.1192/bjp.bp.114.158832

17. NOMIS. Census 2011 - Ethnic group by sex by age: NOMIS, 2013.

18. Government DfCaL. The English Indices of Deprivation 2015, 2015.

19. StataCorp. Stata Statistical Software: Release 14. College Station, TX: StataCorp LP, 2017.

20. Personal Social Services Research Unit. Unit Costs of Health and Social Care 2014, 2014.

21. Department of Health. Payment by Results Guidance for 2013-14, 2013.

22. Department of health. Reference costs 2013-14, 2014.

23. Sterne JAC, White IR, Carlin JB, et al. Multiple imputation for missing data in epidemiological and clinical research: potential and pitfalls. *BMJ* 2009;338:b2393. doi: 10.1136/bmj.b2393

For peer review only

For peer review only - http://bmjopen.bmj.com/site/about/guidelines.xhtml

Supplementary Material

Appendix 1 Read Codes to identify a child with a Life-limiting condition

medcode	clinicalevents	referralevents	testevents	immunisationevents	readcode	readterm	databasebuild
241	217428	9975	65	0	G30..00	Acute myocardial infarction	Feb-09
245	7800	168	4	0	G410.00	Primary pulmonary hypertension	Feb-09
318	411	23	0	0	B210.00	Malignant neoplasm of glottis	Feb-09
319	4215	149	0	0	B21..00	Malignant neoplasm of larynx	Feb-09
348	54604	2944	8	0	B34..11	Ca female breast	Feb-09
512	61437	4071	14	0	K05..00	Chronic renal failure	Feb-09
579	9051	803	14	0	BBE1.00	[M]Malignant melanoma NOS	Feb-09
684	86980	7892	6	0	F20..00	Multiple sclerosis	Feb-09
765	3287	381	1	0	BBe1.00	[M]Neurofibromatosis NOS	Feb-09
780	123877	3483	21	0	B46..00	Malignant neoplasm of prostate	Feb-09
865	30087	2172	10	0	B32..00	Malignant melanoma of skin	Feb-09
1056	8129	525	4	0	B5z..00	Malignant neoplasm of other and unspecified site NOS	Feb-09
1062	16003	663	2	0	B10..00	Malignant neoplasm of oesophagus	Feb-09
1204	4721	258	1	0	G30..14	Heart attack	Feb-09
1220	35966	1430	2	0	B13..00	Malignant neoplasm of colon	Feb-09
1391	312	6	0	0	C327100	Gaucher's disease	Feb-09
1481	48	1	0	0	B600.00	Reticulosarcoma	Feb-09
1483	12690	675	2	0	BBg1.11	[M]Lymphoma NOS	Feb-09
1599	4047	172	1	0	B4A0.00	Malignant neoplasm of kidney parenchyma	Feb-09
1624	38351	2463	10	0	BB2A.00	[M]Squamous cell carcinoma NOS	Feb-09
1800	21118	946	2	0	B141.00	Malignant neoplasm of rectum	Feb-09
1950	9157	203	0	0	BB4..00	[M]Transitional cell papillomas and carcinomas	Feb-09
1952	166	3	0	0	B580.00	Secondary malignant neoplasm of kidney	Feb-09
1986	11636	589	3	0	B440.11	Cancer of ovary	Feb-09
2123	944	54	1	0	BBc1.00	[M]Neuroblastoma NOS	Feb-09

For peer review only - http://bmjopen.bmj.com/site/about/guidelines.xhtml

2272	28720	722	23	0	BB5..11	[M]Adenocarcinomas	Feb-09
2298	2427	120	0	0	F203.00	Exacerbation of multiple sclerosis	Feb-09
2462	10288	433	13	0	B61..00	Hodgkin's disease	Feb-09
2492	9864	1312	66	0	B33z.00	Malignant neoplasm of skin NOS	Feb-09
2587	35784	1469	5	0	B22z.11	Lung cancer	Feb-09
2744	3591	129	2	0	B40..00	"Malignant neoplasm of uterus, part unspecified"	Feb-09
2747	7108	210	1	0	B41..00	Malignant neoplasm of cervix uteri	Feb-09
2755	19616	1231	106	0	B....11	Cancers	Feb-09
2815	6116	113	1	0	B133.00	Malignant neoplasm of sigmoid colon	Feb-09
2816	833	34	0	0	P51..00	Transposition of great vessels	Feb-09
2835	6829	291	5393	0	43C3.11	HIV positive	Feb-09
2890	7205	174	2	0	B430200	Malignant neoplasm of endometrium of corpus uteri	Feb-09
2961	1999	106	0	0	B47z.11	Seminoma of testis	Feb-09
3152	13083	265	0	0	BB13.00	"[M]Carcinoma, metastatic, NOS"	Feb-09
3197	22344	1073	2	0	BB03.00	"[M]Neoplasm, metastatic"	Feb-09
3204	20442	926	6	0	G55..00	Cardiomyopathy	Feb-09
3213	294	19	0	0	B430.00	"Malignant neoplasm of corpus uteri, excluding isthmus"	Feb-09
3230	4027	195	0	0	B41..11	Cervical carcinoma (uterus)	Feb-09
3357	1876	129	0	0	B1...11	Carcinoma of digestive organs and peritoneum	Feb-09
3371	3101	115	0	0	BBg2.11	[M]Non Hodgkins lymphoma	Feb-09
3541	536	56	0	0	B48..00	Malignant neoplasm of penis and other male genital organs	Feb-09
3591	4247	308	0	0	F134.00	Huntington's chorea	Feb-09
3604	14600	525	0	0	B627.00	Non - Hodgkin's lymphoma	Feb-09
3710	407	34	0	0	BBB1.00	[M]Adenolymphoma	Feb-09
3811	6639	194	0	0	B134.00	Malignant neoplasm of caecum	Feb-09
3862	244	16	0	0	P601.00	Congenital atresia of the pulmonary valve	Feb-09

For peer review only - http://bmjopen.bmj.com/site/about/guidelines.xhtml

3903	34821	1333	3	0	B22z.00	Malignant neoplasm of bronchus or lung NOS	Feb-09
3947	6445	288	10	0	P1...00	Spina bifida	Feb-09
3968	128623	3421	8	0	B34..00	Malignant neoplasm of female breast	Feb-09
3969	21	0	0	0	BB9M.00	[M]Intracystic carcinoma NOS	Feb-09
4072	544	43	0	0	B680.00	Acute leukaemia NOS	Feb-09
4118	360	14	0	0	BBV9.00	[M]Myxoid chondrosarcoma	Feb-09
4137	5072	154	1	0	B570.00	Secondary malignant neoplasm of lung	Feb-09
4158	1619	149	0	0	PG51.00	Osteogenesis imperfecta	Feb-09
4165	641	70	0	0	F140.00	Friedreich's ataxia	Feb-09
4217	770	26	0	0	BB5c.00	[M]Parathyroid adenomas and adenocarcinomas	Feb-09
4218	48	0	0	0	B541.00	Malignant neoplasm of parathyroid gland	Feb-09
4222	2158	123	0	0	B64..11	Lymphatic leukaemia	Feb-09
4250	3215	381	0	0	B68z.00	Leukaemia NOS	Feb-09
4251	2752	114	0	0	B640.00	Acute lymphoid leukaemia	Feb-09
4272	983	58	0	0	PH3y200	Epidermolysis bullosa	Feb-09
4388	897	21	0	0	B020.00	Malignant neoplasm of parotid gland	Feb-09
4403	13552	429	0	0	B577.11	Liver metastases	Feb-09
4413	6444	194	0	0	B650.00	Acute myeloid leukaemia	Feb-09
4473	590	13	0	0	BBY0.00	[M]Ewing's sarcoma	Feb-09
4478	1446	87	0	0	F256.00	Infantile spasms	Feb-09
4479	1238	93	0	0	PK5..00	Tuberous sclerosis	Feb-09
4554	1713	44	0	0	B454.00	Malignant neoplasm of vulva unspecified	Feb-09
4632	14309	947	1	0	B33..00	Other malignant neoplasm of skin	Feb-09
4637	722	14	0	0	BBr..00	[M]Leukaemias	Feb-09
4673	P20..00	254	0	8	0	Encephalocele	Feb-09
4796	F152.00	10361	0	689	0	Motor neurone disease	Feb-09
4852	642	90	0	0	BB24.12	[M]Verrucous squamous cell carcinoma	Feb-09
4865	7627	274	1	0	B10z.11	Oesophageal cancer	Feb-09
4944	14705	788	3	0	B630.00	Multiple myeloma	Feb-09
5052	250	13	0	0	BBV1.13	[M]Osteogenic sarcoma NOS	Feb-09

For peer review only - http://bmjopen.bmj.com/site/about/guidelines.xhtml

5136	80	10	1	0	B911013	Choriocarcinoma	Feb-09
5137	302	81	1	0	B624.11	Leukaemic reticuloendotheliosis	Feb-09
5179	232	11	1	0	B620.00	Nodular lymphoma (Brill - Symmers disease)	Feb-09
5198	3093	125	0	0	B583000	Secondary malignant neoplasm of brain	Feb-09
5199	5722	87	0	0	B583200	Cerebral metastasis	Feb-09
5306	235	15	0	0	P100000	"Spina bifida with hydrocephalus, unspecified"	Feb-09
5393	672	51	0	0	F391000	Duchenne muscular dystrophy	Feb-09
5395	2491	73	0	0	A540.00	Eczema herpeticum - Kaposi's varicelliform eruption	Feb-09
5431	P11..00	87	0	1	0	Spina bifida without mention of hydrocephalus	Feb-09
5443	305	6	0	0	F122.00	Malignant neuroleptic syndrome	Feb-09
5455	2673	45	0	0	BB53.00	"[M]Adenocarcinoma, metastatic, NOS"	Feb-09
5637	5356	129	2	0	B53..00	Malignant neoplasm of thyroid gland	Feb-09
5712	2991	84	0	0	P21..00	Microcephalus	Feb-09
5842	6910	159	0	0	B58..00	Secondary malignant neoplasm of other specified sites	Feb-09
5901	9334	321	0	0	B141.12	Rectal carcinoma	Feb-09
5915	536	12	0	0	BBrA400	[M]Hairy cell leukaemia	Feb-09
5932	493	19	0	0	BB06.00	"[M]Tumour cells, uncertain whether benign or malignant"	Feb-09
5964	1625	125	2	0	F39B.00	Muscular dystrophy	Feb-09
6170	10811	117	0	0	B590.11	Carcinomatosis	Feb-09
6220	13732	778	263	0	C370.00	Cystic fibrosis	Feb-09
6316	245	4	0	0	BBr0100	[M]Acute leukaemia NOS	Feb-09
6436	3624	40	0	0	BB43.00	[M]Transitional cell carcinoma NOS	Feb-09
6471	1998	50	0	0	B57..11	Metastases of respiratory and/or digestive systems	Feb-09
6515	P211.00	231	0	2	0	Micrencephaly	Feb-09
6712	3881	65	1	0	K050.00	End stage renal failure	Feb-09

For peer review only - http://bmjopen.bmj.com/site/about/guidelines.xhtml

6746	2004	25	1	0	BB5M.00	[M]Tubular adenomas and adenocarcinomas	Feb-09
6806	187	6	0	0	B12..00	Malignant neoplasm of small intestine and duodenum	Feb-09
6883	951	15	0	0	Q317000	Perinatal bronchopulmonary dysplasia	Feb-09
6920	640	22	0	0	BB5U.00	[M]Villous adenomas and adenocarcinomas	Feb-09
6935	745	5	0	0	B131.00	Malignant neoplasm of transverse colon	Feb-09
6966	267	1	0	0	BB1D.00	[M]Spindle cell carcinoma	Feb-09
7046	2915	13	0	0	B43..00	Malignant neoplasm of body of uterus	Feb-09
7176	2417	106	0	0	B65..00	Myeloid leukaemia	Feb-09
7219	2385	46	0	0	B141.11	Carcinoma of rectum	Feb-09
7319	240	0	0	0	BB9G.00	[M]Infiltrating ductular carcinoma	Feb-09
7476	942	17	0	0	BBQ1.00	[M]Seminomas	Feb-09
7484	4152	107	0	0	B226.00	Mesothelioma	Feb-09
7535	3403	77	0	0	G554400	Primary dilated cardiomyopathy	Feb-09
7593	3116	266	0	0	H51y700	Malignant pleural effusion	Feb-09
7654	7216	308	2	0	B585.00	Secondary malignant neoplasm of bone and bone marrow	Feb-09
7740	236	10	0	0	B470200	Seminoma of undescended testis	Feb-09
7805	9705	177	0	0	B440.00	Malignant neoplasm of ovary	Feb-09
7830	2203	37	0	0	B56..11	Lymph node metastases	Feb-09
7856	362	15	0	0	BBJH.00	[M]Dedifferentiated liposarcoma	Feb-09
7940	453	8	0	0	ByuDF11	[X]Non-Hodgkin's lymphoma NOS	Feb-09
7941	183	2	0	0	BBW4.00	[M]Chondrosarcoma NOS	Feb-09
7945	134	0	0	0	F256000	Hypsarrhythmia	Feb-09
7982	224	2	0	0	B161200	Malignant neoplasm of common bile duct	Feb-09
8010	3501	202	0	0	G551.00	Hypertrophic obstructive cardiomyopathy	Feb-09
8032	1365	37	0	0	BB5B.00	[M]Pancreatic adenomas and carcinomas	Feb-09
8085	2979	102	0	0	BBF1.00	[M]Sarcoma NOS	Feb-09
8088	18	1	0	0	BBG3.00	[M]Fibromyxosarcoma	Feb-09
8101	2854	120	0	0	BB5a.00	[M]Renal adenoma and carcinoma	Feb-09

For peer review only

8154	800	49	0	0	B576200	Malignant ascites	Feb-09
8166	14157	485	1	0	B17..00	Malignant neoplasm of pancreas	Feb-09
8267	29	1	0	0	F363.00	Refsum's disease	Feb-09
8281	11	0	0	0	A789A00	HIV disease resulting in wasting syndrome	Feb-09
8328	326	7	0	0	BBbC.00	"[M]Astrocytoma, anaplastic type"	Feb-09
8351	907	12	0	0	BB91.00	[M]Infiltrating duct carcinoma	Feb-09
8386	10022	372	1	0	B11..00	Malignant neoplasm of stomach	Feb-09
8523	3902	212	0	0	BBb0.11	[M]Glioma NOS	Feb-09
8547	3113	135	1	0	BBbB.00	[M]Astrocytoma NOS	Feb-09
8550	98	2	0	0	B542000	Malignant neoplasm of pituitary gland	Feb-09
8600	2847	117	0	0	1D18.00	Pain from metastases	Feb-09
8606	2207	45	0	0	BB5j.00	[M]Endometrioid adenomas and carcinomas	Feb-09
8625	16007	1249	4	0	B641.00	Chronic lymphoid leukaemia	Feb-09
8627	492	5	1	0	BB07.00	"[M]Tumour cells, malignant"	Feb-09
8649	455	11	0	0	ByuDF00	"[X]Non-Hodgkin's lymphoma, unspecified type"	Feb-09
8660	139	2	0	0	BBV1.00	[M]Osteosarcoma NOS	Feb-09
8695	6720	163	2	0	BB12.00	[M]Carcinoma NOS	Feb-09
8711	3485	112	3	0	BB5D100	[M]Cholangiocarcinoma	Feb-09
8771	1081	5	0	0	B170.00	Malignant neoplasm of head of pancreas	Feb-09
8918	229	4	0	0	B15..00	Malignant neoplasm of liver and intrahepatic bile ducts	Feb-09
8930	4784	25	0	0	BB52.00	[M]Adenocarcinoma NOS	Feb-09
9011	368	9	0	0	P543.00	Eisenmenger's complex	Feb-09
9030	179	4	0	0	B55..00	Malignant neoplasm of other and ill-defined sites	Feb-09
9088	480	10	1	0	B130.00	Malignant neoplasm of hepatic flexure of colon	Feb-09
9118	6778	234	0	0	B13z.11	Colonic cancer	Feb-09
9156	201	4	0	0	BB1K.00	[M]Oat cell carcinoma	Feb-09
9179	703	69	0	0	F151.00	Spinal muscular atrophy	Feb-09

For peer review only - http://bmjopen.bmj.com/site/about/guidelines.xhtml

9237	578	4	0	0	B21z.00	Malignant neoplasm of larynx NOS	Feb-09
9271	2219	87	0	0	F240.00	Quadriplegia	Feb-09
9291	2384	46	0	0	BB1J.00	[M]Small cell carcinoma NOS	Feb-09
9366	297	7	0	0	BB13.11	[M]Secondary carcinoma	Feb-09
9402	734	15	0	0	G55y.11	Secondary dilated cardiomyopathy	Feb-09
9444	142	7	0	0	ZV10400	[V]Personal history of malignant neoplasm of genital organ	Feb-09
9447	969	3	0	0	BB5j200	[M]Endometrioid carcinoma	Feb-09
9470	10661	57	3	0	B34z.00	Malignant neoplasm of female breast NOS	Feb-09
9476	729	18	0	0	B471100	Teratoma of descended testis	Feb-09
9491	632	23	0	0	B142.11	Anal carcinoma	Feb-09
9505	71	2	0	0	B582600	Secondary malignant neoplasm of skin of breast	Feb-09
9575	3391	54	1	0	BBbL.11	[M]Glioblastoma multiforme	Feb-09
9600	1246	24	0	0	B232.00	Mesothelioma of pleura	Feb-09
9618	801	46	0	0	B56..00	Secondary and unspecified malignant neoplasm of lymph nodes	Feb-09
9622	41	4	0	0	B525.00	Malignant neoplasm of cauda equina	Feb-09
9712	820	9	0	0	BB4A.00	[M]Papillary transitional cell carcinoma	Feb-09
9722	P114.00	268	0	3	0	Meningomyelocele	Feb-09
9859	261	1	0	0	BBQ1z00	[M]Seminoma NOS	Feb-09
9902	1265	48	0	0	B3...11	"Carcinoma of bone, connective tissue, skin and breast"	Feb-09
9984	168	6	0	0	B00..11	Carcinoma of lip	Feb-09
10166	1986	62	8	0	C307000	Hyperglycinaemia	Feb-09
10283	3195	152	0	0	B01..00	Malignant neoplasm of tongue	Feb-09
10358	1844	16	0	0	B222.00	"Malignant neoplasm of upper lobe, bronchus or lung"	Feb-09
10368	972	21	0	0	B11..11	Gastric neoplasm	Feb-09
10439	120	7	0	0	ZV76.00	[V]Screening for malignant neoplasm	Feb-09
10541	264	2	0	0	BB22.00	[M]Papillary carcinoma NOS	Feb-09

For peer review only - http://bmjopen.bmj.com/site/about/guidelines.xhtml

10588	1685	50	0	0	BBK0200	[M]Leiomyosarcoma NOS	Feb-09
10628	882	38	0	0	PJyy200	Fragile X chromosome	Feb-09
10668	2130	21	0	0	BB5a000	[M]Renal cell carcinoma	Feb-09
10698	76	2	0	0	B450100	Malignant neoplasm of vaginal vault	Feb-09
10726	3296	148	0	0	B651.00	Chronic myeloid leukaemia	Feb-09
10851	779	26	0	0	B51..11	Cerebral tumour - malignant	Feb-09
10864	565	6	0	0	B132.00	Malignant neoplasm of descending colon	Feb-09
10913	72	0	0	0	BBD0.00	[M]Paraganglioma NOS	Feb-09
10946	1278	22	0	0	B136.00	Malignant neoplasm of ascending colon	Feb-09
10949	433	11	0	0	B162.00	Malignant neoplasm of ampulla of Vater	Feb-09
10955	407	17	0	0	C391100	Di George syndrome	Feb-09
10956	487	23	0	0	PKy9300	Prader - Willi syndrome	Feb-09
10995	2057	31	0	0	B5...00	Malignant neoplasm of other and unspecified sites	Feb-09
11035	475	4	0	0	B593.00	Primary malignant neoplasm of unknown site	Feb-09
11513	297	15	0	0	PH30.00	Congenital ectodermal dysplasia	Feb-09
11531	431	15	0	0	P00..00	Anencephalus	Feb-09
11628	5160	168	0	0	B1z0.11	Cancer of bowel	Feb-09
11773	3006	40	0	0	7L1A.11	Dialysis for renal failure	Feb-09
11991	58	0	0	0	B454.11	Primary vulval cancer	Feb-09
12067	227	20	0	0	B927.11	Von Recklinghausen's disease	Feb-09
12106	966	14	0	0	ZV10.00	[V]Personal history of malignant neoplasm	Feb-09
12146	17	4	0	0	BBr2000	[M]Lymphoid leukaemia NOS	Feb-09
12204	220	10	0	0	C354A00	Metastatic calcification	Feb-09
12309	524	15	0	0	BBb..00	[M]Gliomas	Feb-09
12323	1428	3	0	0	B6...00	Malignant neoplasm of lymphatic and haemopoietic tissue	Feb-09
12335	6535	469	2	0	B62y.00	Malignant lymphoma NOS	Feb-09
12388	249	3	0	0	BB43.11	[M]Urothelial carcinoma	Feb-09
12389	514	9	0	0	B4A1.00	Malignant neoplasm of renal pelvis	Feb-09

For peer review only - http://bmjopen.bmj.com/site/about/guidelines.xhtml

12427	293	8	0	0	BB9F.00	[M]Lobular carcinoma NOS	Feb-09
12464	142	1	0	0	B62x200	Peripheral T-cell lymphoma	Feb-09
12480	321	39	0	0	BB9K000	[M]Paget's disease and intraductal carcinoma of breast	Feb-09
12490	74	2	0	0	B550200	Malignant neoplasm of nose NOS	Feb-09
12497	143	0	0	0	BB82100	[M]Mucinous adenocarcinoma	Feb-09
12499	1207	3	0	0	Byu6.00	[X]Malignant neoplasm of breast	Feb-09
12539	1040	61	0	0	B3...12	Sarcoma of bone and connective tissue	Feb-09
12580	173	0	0	0	BBB0.00	[M]Adenosquamous carcinoma	Feb-09
12582	1332	11	0	0	B224100	Malignant neoplasm of lower lobe of lung	Feb-09
12609	220	3	0	0	BB19.00	"[M]Carcinoma, anaplastic type, NOS"	Feb-09
12870	2717	45	0	0	B221.00	Malignant neoplasm of main bronchus	Feb-09
13243	10271	325	1	0	B22..00	"Malignant neoplasm of trachea, bronchus and lung"	Feb-09
13252	1710	16	0	0	B4...00	Malignant neoplasm of genitourinary organ	Feb-09
13569	20097	639	1	0	B590.00	Disseminated malignancy NOS	Feb-09
13574	10	0	0	0	BB36.00	[M]Metatypical carcinoma	Feb-09
13575	32	1	31	0	4F32.00	Ascitic fluid: malignant cells	Feb-09
13581	149	0	0	0	R210.00	[D]Sudden infant death syndrome	Feb-09
14242	159	13	240	0	42D4.00	RBC's - sickle cells present	Feb-09
14712	565	42	0	0	B00..00	Malignant neoplasm of lip	Feb-09
14792	674	42	0	0	B05..00	Malignant neoplasm of other and unspecified parts of mouth	Feb-09
14800	5086	263	1	0	B11z.00	Malignant neoplasm of stomach NOS	Feb-09
15027	844	45	0	0	B62yz00	Malignant lymphoma NOS	Feb-09
15036	49	2	0	0	B626.00	Malignant mast cell tumours	Feb-09
15103	4814	199	3	0	B577.00	Secondary malignant neoplasm of liver	Feb-09
15148	2504	66	1	0	B47..00	Malignant neoplasm of testis	Feb-09
15182	411	35	6	0	B31z.00	"Malignant neoplasm of connective and soft tissue, site NOS"	Feb-09
15221	187	8	0	0	B220.00	Malignant neoplasm of trachea	Feb-09

For peer review only - http://bmjopen.bmj.com/site/about/guidelines.xhtml

15223	633	16	0	0	B4A2.00	Malignant neoplasm of ureter	Feb-09
15343	1275	95	1	0	PG52.00	Osteopetrosis	Feb-09
15422	2316	216	3	0	D20..00	Aplastic anaemia	Feb-09
15504	150	5	0	0	B62y800	Malignant lymphoma NOS of lymph nodes of multiple sites	Feb-09
15543	104	9	0	0	BB01.00	"[M]Neoplasm, uncertain whether benign or malignant"	Feb-09
15644	173	6	0	0	B4A3.00	Malignant neoplasm of urethra	Feb-09
15658	87	10	0	0	D201.00	Acquired aplastic anaemia	Feb-09
15684	33	4	1	0	B204.00	Malignant neoplasm of frontal sinus	Feb-09
15709	1697	725	0	0	B1...00	Malignant neoplasm of digestive organs and peritoneum	Feb-09
15711	137	2	0	0	B510.00	Malignant neoplasm cerebrum (excluding lobes and ventricles)	Feb-09
15868	95	12	0	0	B335z00	"Malignant neoplasm of skin of trunk, excluding scrotum, NOS"	Feb-09
15907	224	20	5	0	B16z.00	Malignant neoplasm gallbladder/extrahepatic bile ducts NOS	Feb-09
15976	240	19	0	0	B552.00	Malignant neoplasm of abdomen	Feb-09
15989	1174	80	0	0	B47z.12	Teratoma of testis	Feb-09
15991	424	15	0	0	B506.00	Malignant neoplasm of choroid	Feb-09
16038	69	1	0	0	C303300	Maple syrup urine disease	Feb-09
16075	394	22	0	0	B30z.00	Malignant neoplasm of bone and articular cartilage NOS	Feb-09
16087	423	12	0	0	PKy4.00	William syndrome	Feb-09
16105	842	12	0	0	B160.00	Malignant neoplasm of gallbladder	Feb-09
16117	1296	88	0	0	F240.11	Tetraplegia	Feb-09
16118	F392000	1938	0	149	1	Dystrophia myotonica (Steinert's disease)	Feb-09
16126	357	12	0	0	B150000	Primary carcinoma of liver	Feb-09
16146	42	4	0	0	BBB2.00	[M]Adenocarcinoma with squamous metaplasia	Feb-09

For peer review only - http://bmjopen.bmj.com/site/about/guidelines.xhtml

16202	652	24	0	0	B333400	Malignant neoplasm of skin of nose (external)	Feb-09
16213	341	33	0	0	B572.00	Secondary malignant neoplasm of pleura	Feb-09
16241	1580	43	0	0	B060.00	Malignant neoplasm of tonsil	Feb-09
16280	158	1	0	0	B550400	Malignant neoplasm of neck NOS	Feb-09
16297	654	30	0	0	B0z0.00	Malignant neoplasm of pharynx unspecified	Feb-09
16298	48	0	0	0	B18z.00	Malignant neoplasm of retroperitoneum and peritoneum NOS	Feb-09
16416	400	56	1	0	B681.00	Chronic leukaemia NOS	Feb-09
16443	45	3	0	0	C375211	Hunter's syndrome	Feb-09
16460	554	30	0	0	BBg2.00	"[M]Malignant lymphoma, non Hodgkin's type"	Feb-09
16500	809	32	0	0	B58z.00	Secondary malignant neoplasm of other specified site NOS	Feb-09
16677	143	3	0	0	BB9B.00	[M]Medullary carcinoma NOS	Feb-09
16692	1029	9	0	0	BB14.00	[M]Carcinomatosis	Feb-09
16704	73	1	0	0	B302.00	Malignant neoplasm of vertebral column	Feb-09
16711	519	34	0	0	PKy7B00	Stickler syndrome	Feb-09
16723	678	27	0	0	BB5S212	[M]Bronchiolar carcinoma	Feb-09
16760	371	4	0	0	B58y000	Secondary malignant neoplasm of breast	Feb-09
16774	412	9	0	0	BBmD.00	[M] Cutaneous lymphoma	Feb-09
16854	2691	189	0	0	B927.00	Neurofibromatosis - Von Recklinghausen's disease	Feb-09
16915	96	1	0	0	B151.00	Malignant neoplasm of intrahepatic bile ducts	Feb-09
16967	32	0	0	0	B432.00	Malignant neoplasm of overlapping lesion of corpus uteri	Feb-09
17151	1215	68	0	0	BB81.11	[M]Ovarian cystadenoma or carcinoma	Feb-09
17178	525	12	0	0	BBg..00	"[M]Lymphomas, NOS or diffuse"	Feb-09
17182	1343	8	0	0	B627C11	Follicular lymphoma NOS	Feb-09
17212	143	1	0	0	BBLH.00	[M]Rhabdoid sarcoma	Feb-09

For peer review only - http://bmjopen.bmj.com/site/about/guidelines.xhtml

17314	758	81	0	0	BBL7112	[M]Wilms' tumour	Feb-09
17366	296	24	0	0	BBF..00	[M]Soft tissue tumours and sarcomas NOS	Feb-09
17391	142	4	0	0	B221000	Malignant neoplasm of carina of bronchus	Feb-09
17460	93	3	0	0	B627700	Diffuse non-Hodgkin's lymphoblastic (diffuse) lymphoma	Feb-09
17468	289	5	0	0	BBQ..00	[M]Germ cell neoplasms	Feb-09
17475	128	6	0	0	B300A00	Malignant neoplasm of maxilla	Feb-09
17559	550	22	0	0	B1z0.00	"Malignant neoplasm of intestinal tract, part unspecified"	Feb-09
17841	147	0	0	0	B481.00	Malignant neoplasm of glans penis	Feb-09
17874	184	7	0	0	B181.00	Mesothelioma of peritoneum	Feb-09
17887	982	16	0	0	B62x.00	Malignant lymphoma otherwise specified	Feb-09
17912	24	10	0	0	B042.00	"Malignant neoplasm, overlapping lesion of floor of mouth"	Feb-09
18245	152	8	0	0	B330.00	Malignant neoplasm of skin of lip	Feb-09
18255	141	19	1	0	BB5L.00	[M]Adenomatous and adenocarcinomatous polyps	Feb-09
18270	1315	40	0	0	7G03K00	Excision malignant skin tumour	Feb-09
18314	255	5	0	0	B30..00	Malignant neoplasm of bone and articular cartilage	Feb-09
18321	20	1	0	0	PG56012	Conradi - Hunermann syndrome	Feb-09
18354	86	2	0	0	B33y.00	Malignant neoplasm of other specified skin sites	Feb-09
18383	603	7	0	0	BBmH.00	[M] Large cell lymphoma	Feb-09
18537	224	8	0	0	P233.11	Dandy - Walker syndrome	Feb-09
18613	383	57	1	0	B120.00	Malignant neoplasm of duodenum	Feb-09
18616	313	0	0	0	B58y.00	Secondary malignant neoplasm of other specified sites	Feb-09
18617	3533	53	0	0	B51..00	Malignant neoplasm of brain	Feb-09

For peer review only - http://bmjopen.bmj.com/site/about/guidelines.xhtml

18618	31	1	0	0	B335300	Malignant neoplasm of skin of abdominal wall	Feb-09
18619	351	2	0	0	B137.00	Malignant neoplasm of splenic flexure of colon	Feb-09
18632	305	2	0	0	B135.00	Malignant neoplasm of appendix	Feb-09
18676	1030	33	0	0	B585000	Pathological fracture due to metastatic bone disease	Feb-09
18678	226	0	0	0	B224000	Malignant neoplasm of lower lobe bronchus	Feb-09
18712	2030	46	0	0	B4A..11	Renal malignant neoplasm	Feb-09
18744	465	6	0	0	BBn0.11	[M]Multiple myeloma	Feb-09
18771	72	0	0	0	BBLJ.00	[M]Clear cell sarcoma of kidney	Feb-09
18882	26	16	1	0	B006.00	Malignant neoplasm of overlapping lesion of lip	Feb-09
18905	8	0	0	0	C370300	Cystic fibrosis with intestinal manifestations	Feb-09
18914	187	13	0	0	C370200	Cystic fibrosis with pulmonary manifestations	Feb-09
19007	218	0	0	0	Q48E.00	Periventricular leucomalacia	Feb-09
19038	32	0	0	0	PJ1z.11	Trisomy 13 NOS	Feb-09
19041	947	23	0	0	BB29.12	[M]Intraepidermal carcinoma NOS	Feb-09
19083	1453	209	0	0	1J04.00	Suspected lymphoma	Feb-09
19091	1049	154	0	0	BB5..00	[M]Adenomas and adenocarcinomas	Feb-09
19140	3	0	0	0	B614800	Hodgkin's nodular sclerosis of lymph nodes of multiple sites	Feb-09
19141	439	7	0	0	B44..00	Malignant neoplasm of ovary and other uterine adnexa	Feb-09
19144	222	10	0	0	Byu4.00	[X]Melanoma and other malignant neoplasms of skin	Feb-09
19162	41	0	0	0	B493.00	Malignant neoplasm of anterior wall of urinary bladder	Feb-09
19226	142	3	0	0	B513.00	Malignant neoplasm of parietal lobe	Feb-09

For peer review only - http://bmjopen.bmj.com/site/about/guidelines.xhtml

19263	417	21	0	0	BB5f.00	[M]Thyroid adenoma and adenocarcinoma	Feb-09
19318	116	3	0	0	B112.00	Malignant neoplasm of pyloric antrum of stomach	Feb-09
19321	25	1	0	0	B311300	Malignant neoplasm of connective and soft tissue of hand	Feb-09
19334	231	3	0	0	BBL9.00	[M]Carcinosarcoma NOS	Feb-09
19372	1294	108	0	0	B64..00	Lymphoid leukaemia	Feb-09
19415	732	23	0	0	B0...00	"Malignant neoplasm of lip, oral cavity and pharynx"	Feb-09
19423	663	14	0	0	B35..00	Malignant neoplasm of male breast	Feb-09
19437	481	12	0	0	B30z000	Osteosarcoma	Feb-09
19444	520	7	0	0	Byu4100	"[X]Malignant melanoma of skin, unspecified"	Feb-09
19475	54	1	0	0	B471.00	Malignant neoplasm of descended testis	Feb-09
19584	413	3	1	0	Q401.00	Congenital cytomegalovirus infection	Feb-09
19657	158	6	0	0	B911000	Malignant hydatidiform mole	Feb-09
19678	996	118	0	0	BB29.13	[M]Intraepithelial squamous cell carcinoma	Feb-09
19692	286	85	0	0	1J02.00	Suspected leukaemia	Feb-09
19731	26	1	0	0	BB5N.11	[M]Adenoma or or adenocarcinoma in polyposis coli	Feb-09
19945	340	41	27	0	B582.00	Secondary malignant neoplasm of skin	Feb-09
19974	42	6	0	0	B660.00	Acute monocytic leukaemia	Feb-09
20092	562	18	0	0	B04..00	Malignant neoplasm of floor of mouth	Feb-09
20151	371	18	9	0	Q400.00	Congenital rubella	Feb-09
20160	382	13	0	0	B50..00	Malignant neoplasm of eye	Feb-09
20166	41	1	0	0	B45z.00	Malignant neoplasm of female genital organ NOS	Feb-09
20292	299	7	0	0	B02..00	Malignant neoplasm of major salivary glands	Feb-09
20437	350	9	0	0	BBk..00	"[M]Lymphomas, nodular or follicular"	Feb-09

For peer review only - http://bmjopen.bmj.com/site/about/guidelines.xhtml

20440	221	9	0	0	B69..00	Myelomonocytic leukaemia	Feb-09
20493	1686	19	0	0	F20z.00	Multiple sclerosis NOS	Feb-09
20635	32	1	0	0	BBr2011	[M]Lymphatic leukaemia	Feb-09
20685	91	1	0	0	B346.00	Malignant neoplasm of axillary tail of female breast	Feb-09
20699	135	1	0	0	7H19500	Closure of gastrochisis	Feb-09
20710	843	30	0	0	BBj..00	[M]Hodgkin's disease	Feb-09
20772	281	7	0	0	P67..00	Hypoplastic left heart syndrome	Feb-09
20807	184	18	0	0	BB26.00	[M]Papillary squamous cell carcinoma	Feb-09
20822	132	3	0	0	Q48y100	Congenital cardiac failure	Feb-09
21217	64	2	0	0	BB1N.00	[M]Small cell-large cell carcinoma	Feb-09
21249	196	2	0	0	F232.00	Congenital quadriplegia	Feb-09
21327	310	7	0	0	B333500	Malignant neoplasm of skin of temple	Feb-09
21330	128	2	0	0	B180.00	Malignant neoplasm of retroperitoneum	Feb-09
21402	404	6	0	0	B602.00	Burkitt's lymphoma	Feb-09
21418	140	6	0	0	PKy6011	Cornelia de Lange syndrome	Feb-09
21447	14	0	0	0	BBV3.00	[M]Fibroblastic osteosarcoma	Feb-09
21463	272	2	0	0	BBgC.11	[M]Lymphocytic lymphoma NOS	Feb-09
21549	739	16	0	0	B627C00	Follicular non-Hodgkin's lymphoma	Feb-09
21590	352	11	0	0	B58y500	Secondary malignant neoplasm of prostate	Feb-09
21609	131	3	0	0	BB18.00	"[M]Carcinoma, undifferentiated type, NOS"	Feb-09
21620	86	0	0	0	B111.00	Malignant neoplasm of pylorus of stomach	Feb-09
21659	269	4	0	0	BB5Bz00	[M]Pancreatic adenoma or carcinoma NOS	Feb-09
21682	24	0	0	0	BBQ7500	"[M]Malignant teratoma, intermediate type"	Feb-09
21698	631	6	0	0	B221z00	Malignant neoplasm of main bronchus NOS	Feb-09
21715	297	5	0	0	Byu5011	[X]Mesothelioma of lung	Feb-09

For peer review only - http://bmjopen.bmj.com/site/about/guidelines.xhtml

21723	55	4	0	0	D201z00	Acquired aplastic anaemia NOS	Feb-09
21732	40	0	0	0	BBH1.00	[M]Myxosarcoma	Feb-09
21741	64	1	0	0	BB5f100	[M]Follicular adenocarcinoma NOS	Feb-09
21769	458	12	0	0	J625.11	[X] Liver failure	Feb-09
21770	836	71	0	0	BBPX.00	"[M]Mesothelioma, unspecified"	Feb-09
21786	1506	23	0	0	B471000	Seminoma of descended testis	Feb-09
21802	238	1	0	0	P1z..00	Spina bifida NOS	Feb-09
21833	854	4	0	0	BB91.11	[M]Duct carcinoma NOS	Feb-09
21847	356	2	0	0	BB5f111	[M]Follicular carcinoma	Feb-09
21852	184	34	0	0	G554200	Familial cardiomyopathy	Feb-09
21868	2570	18	0	0	BB02.00	"[M]Neoplasm, malignant"	Feb-09
21914	262	7	0	0	BB11.11	[M]Intraepithelial carcinoma NOS	Feb-09
22050	507	8	0	0	B691.00	Chronic myelomonocytic leukaemia	Feb-09
22071	8	0	0	0	BBr0111	[M]Blast cell leukaemia	Feb-09
22146	91	0	0	0	B581100	Secondary malignant neoplasm of bladder	Feb-09
22156	208	2	0	0	BB08.00	"[M]Malignant tumour, small cell type"	Feb-09
22158	88	1	0	0	B630000	"Malignant plasma cell neoplasm, extramedullary plasmacytoma"	Feb-09
22163	673	13	0	0	B134.11	Carcinoma of caecum	Feb-09
22174	10	1	0	0	F390z00	Congenital hereditary muscular dystrophy NOS	Feb-09
22187	2226	46	0	0	B150300	Hepatocellular carcinoma	Feb-09
22267	215	19	0	0	BB04.00	"[M]Neoplasm, malig, uncertain whether primary or metastatic"	Feb-09
22290	27	3	0	0	B313.00	Malignant neoplasm of connective and soft tissue of thorax	Feb-09
22441	28	1	0	0	B212.00	Malignant neoplasm of subglottis	Feb-09
22524	318	0	0	0	B58yz00	Secondary malignant neoplasm of other specified site NOS	Feb-09
22561	187	13	0	0	BBV4.00	[M]Telangiectatic osteosarcoma	Feb-09
22650	307	10	0	0	BBT1.11	[M]Angiosarcoma	Feb-09

For peer review only - http://bmjopen.bmj.com/site/about/guidelines.xhtml

22692	124	6	0	0	BBEG000	"[M]Acral lentiginous melanoma, malignant"	Feb-09
22728	168	1	0	0	SP08700	Acute graft-versus-host disease	Feb-09
22851	173	11	1	0	C375.00	Mucopolysaccharidosis	Feb-09
22893	485	8	0	0	B06..00	Malignant neoplasm of oropharynx	Feb-09
22894	150	3	0	0	B110100	Malignant neoplasm of cardio-oesophageal junction of stomach	Feb-09
22993	1078	20	0	0	G55z.00	Cardiomyopathy NOS	Feb-09
23083	888	11	0	0	BBbL.00	[M]Glioblastoma NOS	Feb-09
23320	53	3	0	0	N233011	Arthrogryposis multiplex congenita.	Feb-09
23380	162	19	0	0	B340000	Malignant neoplasm of nipple of female breast	Feb-09
23389	286	14	0	0	B200.00	Malignant neoplasm of nasal cavities	Feb-09
23399	919	7	0	0	B344.00	Malignant neoplasm of upper-outer quadrant of female breast	Feb-09
23415	97	9	0	0	F256100	Salaam attacks	Feb-09
23433	109	0	0	0	B161.00	Malignant neoplasm of extrahepatic bile ducts	Feb-09
23455	74	5	0	0	BBe1.12	[M]Von Recklinghausen's disease	Feb-09
23480	145	16	0	0	B335900	Malignant neoplasm of perianal skin	Feb-09
23566	40	1	0	0	Q490.00	Neonatal cardiac failure	Feb-09
23711	127	5	0	0	BBg1000	"[M]Malignant lymphoma, diffuse NOS"	Feb-09
23730	99	16	0	0	F202.00	Generalised multiple sclerosis	Feb-09
23775	223	20	0	0	J62y.12	Liver failure NOS	Feb-09
23861	57	5	0	0	B551100	Malignant neoplasm of chest wall NOS	Feb-09
23936	36	2	0	0	ZV10411	[V]Personal history of malignant neoplasm of cervix uteri	Feb-09
23945	544	22	0	0	PK80.00	Fetal alcohol syndrome	Feb-09
23951	43	0	0	0	A789200	HIV disease resulting in candidiasis	Feb-09
23960	17	1	0	0	PD03011	Potter's syndrome	Feb-09
24048	13	0	0	0	B180200	Malignant neoplasm of retrocaecal tissue	Feb-09

For peer review only - http://bmjopen.bmj.com/site/about/guidelines.xhtml

24293	1136	14	0	0	BB2B.00	"[M]Squamous cell carcinoma, metastatic NOS"	Feb-09
24301	46	1	0	0	B57..12	Secondary carcinoma of respiratory and/or digestive systems	Feb-09
24312	29	0	0	0	BB62.00	[M]Apocrine adenoma and adenocarcinomas	Feb-09
24370	983	54	1	0	B142.00	Malignant neoplasm of anal canal	Feb-09
24374	478	23	0	0	B0...11	"Carcinoma of lip, oral cavity and pharynx"	Feb-09
24375	693	37	0	0	B339.00	Dermatofibrosarcoma protuberans	Feb-09
24397	140	4	0	0	B061.00	Malignant neoplasm of tonsillar fossa	Feb-09
24437	830	29	0	0	PB5z.12	Short bowel syndrome	Feb-09
24511	89	6	0	0	BB09.00	"[M]Malignant tumour, giant cell type"	Feb-09
24539	55	2	0	0	BBV2.00	[M]Chondroblastic osteosarcoma	Feb-09
24551	49	2	0	0	BBE1.11	[M]Melanocarcinoma	Feb-09
24675	446	5	0	0	B07..00	Malignant neoplasm of nasopharynx	Feb-09
24852	100	0	0	0	B016.00	Malignant neoplasm of lingual tonsil	Feb-09
24924	59	2	0	0	BBD..00	[M]Paragangliomas and glomus tumours	Feb-09
25191	518	39	0	0	B68..00	Leukaemia of unspecified cell type	Feb-09
25245	13	2	0	0	B336400	Malignant neoplasm of skin of finger	Feb-09
25282	P203.11	68	0	3	0	Meningocele - cranial	Feb-09
25304	140	5	0	0	Q402300	Congenital toxoplasmosis	Feb-09
25306	196	6	0	0	PKyz511	Angelman syndrome	Feb-09
25429	37	0	0	0	F390300	Myotubular myopathy	Feb-09
25535	566	20	1	0	B150.00	Primary malignant neoplasm of liver	Feb-09
25597	237	2	0	0	PB61.00	Biliary atresia	Feb-09
25602	13	2	0	0	B326400	Malignant melanoma of finger	Feb-09
25641	149	7	0	0	BB5D513	[M]Liver cell carcinoma	Feb-09
25657	29	0	0	0	A752.00	Pfeiffer's disease	Feb-09
25695	171	3	0	0	SP08800	Chronic graft-versus-host disease	Feb-09
25835	45573	21	1	0	ZV76200	[V]Screening for malignant neoplasm of cervix	Feb-09

For peer review only - http://bmjopen.bmj.com/site/about/guidelines.xhtml

For peer review only

25886	2578	12	0	0	B222100	Malignant neoplasm of upper lobe of lung	Feb-09
25961	165	3	0	0	BB17.00	[M]Large cell carcinoma NOS	Feb-09
26034	125	0	0	0	B591.00	Other malignant neoplasm NOS	Feb-09
26111	237	4	0	0	M162800	Lymphomatoid papulosis	Feb-09
26120	186	6	0	0	BB5V.00	[M]Pituitary adenomas and carcinomas	Feb-09
26134	10	0	0	0	B064000	"Malignant neoplasm of epiglottis, free border"	Feb-09
26165	408	8	0	0	B211.00	Malignant neoplasm of supraglottis	Feb-09
26253	940	8	0	0	BB5R900	[M]Neuroendocrine carcinoma	Feb-09
26393	865	24	0	0	B152.00	Malignant neoplasm of liver unspecified	Feb-09
26413	100	3	0	0	BB1A.00	[M]Pleomorphic carcinoma	Feb-09
26448	4	0	0	0	B060000	Malignant neoplasm of faucial tonsil	Feb-09
26454	13	0	0	0	B45X.00	Malignant neoplasm/overlapping lesion/feml genital organs	Feb-09
26478	260	7	0	0	SN5y000	Malignant hyperpyrexia due to anaesthetic	Feb-09
26813	74	0	0	0	B21y.00	"Malignant neoplasm of larynx, other specified site"	Feb-09
26814	207	4	0	0	BB5D512	"[M]Hepatoma, malignant"	Feb-09
26848	20	1	0	0	BB5S.00	[M]Respiratory tract adenomas and adenocarcinomas	Feb-09
26853	342	14	0	0	B340.00	Malignant neoplasm of nipple and areola of female breast	Feb-09
26858	137	5	0	0	BB5C.00	[M]Gastrinoma and carcinomas	Feb-09
27280	45	4	0	0	PKy0.12	Prader-Willi syndrome	Feb-09
27330	61	7	0	0	B624.00	Leukaemic reticuloendotheliosis	Feb-09
27363	19	0	0	0	BBd2.00	"[M]Meningioma, malignant"	Feb-09
27370	172	5	0	0	B333.00	Malignant neoplasm skin of other and unspecified parts face	Feb-09
27377	494	62	0	0	F152200	Progressive bulbar palsy	Feb-09
27391	311	20	0	0	B576100	Secondary malignant neoplasm of peritoneum	Feb-09

For peer review only - http://bmjopen.bmj.com/site/about/guidelines.xhtml

For peer review only

27416	197	8	0	0	B601.00	Lymphosarcoma	Feb-09
27439	335	23	0	0	BBTA.00	[M]Kaposi's sarcoma	Feb-09
27449	48	2	0	0	B554.00	Malignant neoplasm of upper limb NOS	Feb-09
27458	129	18	0	0	B661.00	Chronic monocytic leukaemia	Feb-09
27475	195	0	0	0	13M3.00	Sudden infant death	Feb-09
27483	149	6	0	0	B240.00	Malignant neoplasm of thymus	Feb-09
27509	95	0	0	0	BBP1.00	"[M]Mesothelioma, malignant"	Feb-09
27520	65	0	0	0	B651z00	Chronic myeloid leukaemia NOS	Feb-09
27528	12	0	0	0	B303.00	"Malignant neoplasm of ribs, sternum and clavicle"	Feb-09
27540	46	0	0	0	B4A1000	Malignant neoplasm of renal calyces	Feb-09
27562	5	0	0	0	BBk0.12	[M]Follicular lymphosarcoma NOS	Feb-09
27617	7	0	0	0	B45y000	Malignant neoplasm of overlapping lesion of vulva	Feb-09
27641	215	1	0	0	A789300	HIV disease resulting in Pneumocystis carinii pneumonia	Feb-09
27651	183	2	0	0	B58..11	Secondary carcinoma of other specified sites	Feb-09
27653	149	1	0	0	BBbz.00	[M]Glioma NOS	Feb-09
27664	253	2	0	0	B65y100	Acute promyelocytic leukaemia	Feb-09
27683	112	11	0	0	G558100	Cardiomyopathy in myotonic dystrophy	Feb-09
27686	P203.00	89	0	0	0	Meningocele - cerebral	Feb-09
27715	15	0	0	0	B242.00	Malignant neoplasm of anterior mediastinum	Feb-09
27728	202	3	0	0	BB90.00	"[M]Intraductal carcinoma, noninfiltrating NOS"	Feb-09
27744	969	36	0	0	BBbQ.00	[M]Oligodendroglioma NOS	Feb-09
27748	131	4	0	0	BBbB.11	[M]Astrocytic glioma	Feb-09
27790	865	59	0	0	B641.11	Chronic lymphatic leukaemia	Feb-09
27846	46	0	0	0	BBbF.00	[M]Fibrillary astrocytoma	Feb-09
27849	12	0	0	0	BB5U200	[M]Villous adenocarcinoma	Feb-09
27853	45	0	0	0	A789500	HIV disease resulting in Kaposi's sarcoma	Feb-09

For peer review only - http://bmjopen.bmj.com/site/about/guidelines.xhtml

27855	1087	13	1	0	B140.00	Malignant neoplasm of rectosigmoid junction	Feb-09
27897	267	2	0	0	B143.00	Malignant neoplasm of anus unspecified	Feb-09
27931	66	4	0	0	B33z000	Kaposi's sarcoma of skin	Feb-09
27965	23	1	0	0	BBv2.00	[M]AngiocentricT-cell lymphoma	Feb-09
28003	144	2	0	0	B420.00	Choriocarcinoma	Feb-09
28069	43	2	0	0	B505.00	Malignant neoplasm of retina	Feb-09
28148	204	0	0	0	B540.00	Malignant neoplasm of adrenal gland	Feb-09
28163	2504	28	0	0	B13z.00	Malignant neoplasm of colon NOS	Feb-09
28178	863	71	0	0	BBa0.00	[M]Craniopharyngioma	Feb-09
28210	33	2	0	0	F391300	Other limb-girdle muscular dystrophy	Feb-09
28241	155	0	0	0	B496.00	Malignant neoplasm of ureteric orifice	Feb-09
28272	472	14	0	0	BB57.00	"[M]Adenocarcinoma, intestinal type"	Feb-09
28291	165	19	0	0	BB69.00	[M]Sebaceous adenoma and adenocarcinoma	Feb-09
28311	1295	7	0	0	B41z.00	Malignant neoplasm of cervix uteri NOS	Feb-09
28344	60	1	0	0	BBb3.12	[M]Subependymal astrocytoma NOS	Feb-09
28355	222	4	0	0	7904	Other correction of transposition of great vessels	Feb-09
28388	189	1	0	0	BB5jz00	[M]Endometrioid adenoma or carcinoma NOS	Feb-09
28451	28	0	0	0	B08z.00	Malignant neoplasm of hypopharynx NOS	Feb-09
28556	815	48	0	0	B32z.00	Malignant melanoma of skin NOS	Feb-09
28559	18	0	0	0	B055z00	Malignant neoplasm of palate NOS	Feb-09
28599	484	21	0	0	BBJ1.00	[M]Liposarcoma NOS	Feb-09
28625	72	0	0	0	BB71.00	[M]Mucoepidermoid carcinoma	Feb-09
28628	72	1	0	0	BBJ3.00	"[M]Liposarcoma, well differentiated type"	Feb-09
28639	119	1	0	0	B627000	Follicular non-Hodgkin's small cleaved cell lymphoma	Feb-09
28663	26	3	0	0	PKy9200	Menke's syndrome	Feb-09
28665	42	0	0	0	B07z.00	Malignant neoplasm of nasopharynx NOS	Feb-09

For peer review only - http://bmjopen.bmj.com/site/about/guidelines.xhtml

28727	138	1	0	0	B575000	Secondary malignant neoplasm of colon	Feb-09
28824	95	1	0	0	P240200	Schizencephaly	Feb-09
28836	523	37	0	0	BBc9.00	[M]Retinoblastomas	Feb-09
28881	8	1	0	0	ZV10513	[V]Personal history of malignant neoplasm of kidney	Feb-09
28919	44	2	0	0	B521.00	Malignant neoplasm of cerebral meninges	Feb-09
28941	88	0	0	0	BBQ3.00	[M]Embryonal carcinoma NOS	Feb-09
28955	F392100	319	0	23	0	Myotonia congenita (Thomsen's disease)	Feb-09
29008	51	0	0	0	BB5W111	[M]Hurthle cell adenocarcinoma	Feb-09
29160	30	2	0	0	B313000	Malignant neoplasm of connective and soft tissue of axilla	Feb-09
29178	515	1	0	0	B614.00	"Hodgkin's disease, nodular sclerosis"	Feb-09
29283	11	0	0	0	B2zy.00	Malignant neoplasm of other site of respiratory tract	Feb-09
29284	82	9	0	0	ZV10112	[V]Personal history of malignant neoplasm of lung	Feb-09
29335	117	2	0	0	BBr2700	[M]Adult T-cell leukaemia/lymphoma	Feb-09
29337	80	2	0	0	BBVA.00	[M] Small cell osteosarcoma	Feb-09
29462	1773	8	0	0	B4Az.00	Malignant neoplasm of kidney or urinary organs NOS	Feb-09
29580	16	1	0	0	BBCA.00	[M]Sertoli cell carcinoma	Feb-09
29787	244	19	0	0	BB2C.00	"[M]Squamous cell carcinoma, keratinising type NOS"	Feb-09
29826	266	1	0	0	B342.00	Malignant neoplasm of upper-inner quadrant of female breast	Feb-09
29876	7	0	0	0	B613z00	"Hodgkin's, lymphocytic-histiocytic predominance NOS"	Feb-09
29945	93	0	0	0	BBR4.00	"[M]Malignant teratoma, trophoblastic"	Feb-09
30119	8	0	0	0	C1zy100	Progeria	Feb-09
30165	3	1	0	0	B18y200	Malignant neoplasm of mesorectum	Feb-09

For peer review only - http://bmjopen.bmj.com/site/about/guidelines.xhtml

30189	81	0	0	0	BB91000	[M]Intraductal papillary adenocarcinoma with invasion	Feb-09
30273	381	13	0	0	BBbG.00	[M]Pilocytic astrocytoma	Feb-09
30283	1419	10	0	0	7B2C700	Intravesical install chemotherapeutic agent for malignancy	Feb-09
30322	73	9	0	0	ZV10500	[V]Personal history of malignant neoplasm of urinary organ	Feb-09
30402	77	6	0	0	B050.11	Malignant neoplasm of buccal mucosa	Feb-09
30416	12	0	0	0	BB82111	[M]Colloid adenocarcinoma	Feb-09
30526	47	0	0	0	Byu5100	"[X]Mesothelioma, unspecified"	Feb-09
30537	15	0	0	0	F373.00	Polyneuropathy in malignant disease	Feb-09
30543	120	1	0	0	B335200	Malignant neoplasm of skin of breast	Feb-09
30576	395	6	0	0	B333300	Malignant neoplasm of skin of forehead	Feb-09
30577	80	2	0	0	B336200	Malignant neoplasm of skin of fore-arm	Feb-09
30632	44	1	0	0	B67z.00	Other specified leukaemia NOS	Feb-09
30645	431	7	0	0	B333000	"Malignant neoplasm of skin of cheek, external"	Feb-09
30646	27	0	0	0	B6y..00	Malignant neoplasm lymphatic or haematopoietic tissue OS	Feb-09
30700	2796	53	0	0	B10z.00	Malignant neoplasm of oesophagus NOS	Feb-09
30747	252	0	0	0	B336.00	Malignant neoplasm of skin of upper limb and shoulder	Feb-09
30988	105	1	0	0	BB1M.00	"[M]Small cell carcinoma, intermediate cell"	Feb-09
31004	48	1	0	0	BB2G.00	[M]Adenoid squamous cell carcinoma	Feb-09
31026	189	4	0	0	BBF3.00	[M]Spindle cell sarcoma	Feb-09
31042	379	23	0	0	Eu84200	[X]Rett's syndrome	Feb-09
31090	20	3	0	0	BBGP.00	[M]Pigmented dermatofibrosarcoma protuberans	Feb-09
31102	4275	30	0	0	B49z.00	Malignant neoplasm of urinary bladder NOS	Feb-09
31112	250	12	0	0	P732.00	Pulmonary artery atresia	Feb-09

For peer review only - http://bmjopen.bmj.com/site/about/guidelines.xhtml

31188	771	6	0	0	B224.00	"Malignant neoplasm of lower lobe, bronchus or lung"	Feb-09
31210	128	3	0	0	B150100	Hepatoblastoma of liver	Feb-09
31268	293	4	0	0	B223.00	"Malignant neoplasm of middle lobe, bronchus or lung"	Feb-09
31323	158	15	0	0	BBG1.00	[M]Fibrosarcoma NOS	Feb-09
31324	38	1	0	0	B626800	Mast cell malignancy of lymph nodes of multiple sites	Feb-09
31364	118	6	1	0	B050.00	Malignant neoplasm of cheek mucosa	Feb-09
31393	361	14	0	0	B160.11	Carcinoma gallbladder	Feb-09
31399	74	3	0	0	B555.00	Malignant neoplasm of lower limb NOS	Feb-09
31418	455	35	0	0	N233000	Arthrogryposis	Feb-09
31421	230	18	0	0	BBK3100	[M]Rhabdomyosarcoma NOS	Feb-09
31492	335	5	0	0	BBm9.00	[M] Monocytoid B-cell lymphoma	Feb-09
31537	9	0	0	0	BBj1100	"[M]Hodgkin,s disease, lymphocytic predominance, nodular"	Feb-09
31541	16	3	0	0	C392300	Severe combined immunodefiency with reticular dysgenesis	Feb-09
31546	195	2	0	0	B341.00	Malignant neoplasm of central part of female breast	Feb-09
31573	172	1	0	0	B23..00	Malignant neoplasm of pleura	Feb-09
31574	223	18	0	0	BBb0.00	"[M]Glioma, malignant"	Feb-09
31576	73	1	0	0	B627B00	Other types of follicular non-Hodgkin's lymphoma	Feb-09
31586	110	3	0	0	B64y100	Prolymphocytic leukaemia	Feb-09
31608	45	0	0	0	B43y.00	Malignant neoplasm of other site of uterine body	Feb-09
31609	24	2	0	0	BBC4.00	"[M]Granulosa cell tumour, malignant"	Feb-09
31629	198	16	0	0	BBc6.00	[M]Ganglioglioma	Feb-09
31663	20	5	0	0	2826	O/E - salaam attack	Feb-09
31673	5	0	0	0	BBX1.12	"[M]Osteoclastoma, malignant"	Feb-09

For peer review only - http://bmjopen.bmj.com/site/about/guidelines.xhtml

31700	456	3	0	0	B222000	Malignant neoplasm of upper lobe bronchus	Feb-09
31701	162	8	0	0	B651.11	Chronic granulocytic leukaemia	Feb-09
31726	6	0	0	0	BBgM.00	"[M]Malignant lymphoma, small cleaved cell, diffuse"	Feb-09
31741	2	0	0	0	BBj6200	"[M]Hodgkin,s disease, nodular sclerosis, lymphocytic deplet"	Feb-09
31749	18	0	0	0	BBv0.00	[M]Monocytoid B-cell lymphoma	Feb-09
31750	106	3	0	0	BBr0300	[M]Chronic leukaemia NOS	Feb-09
31755	79	5	0	0	G240.00	Secondary malignant hypertension	Feb-09
31772	149	2	0	0	BBGM.00	[M]Dermatofibrosarcoma NOS	Feb-09
31775	P115.00	31	0	0	1	Myelocele	Feb-09
31794	661	3	0	0	B627W00	Unspecified B-cell non-Hodgkin's lymphoma	Feb-09
31818	33	4	0	0	BBK2.00	[M]Myoma and myosarcoma	Feb-09
32022	346	8	0	0	B110.00	Malignant neoplasm of cardia of stomach	Feb-09
32024	15	0	0	0	B030.00	Malignant neoplasm of upper gum	Feb-09
32174	79	1	0	0	B202.00	Malignant neoplasm of maxillary sinus	Feb-09
32213	2	1	0	0	BB0A.00	"[M]Malignant tumour, fusiform cell type"	Feb-09
32240	7	0	11	0	4M22.00	Lymphoma stage III	Feb-09
32246	27	6	0	0	ZV10111	[V]Personal history of malignant neoplasm of bronchus	Feb-09
32294	1	0	0	0	BB5B500	"[M]Glucagonoma, malignant"	Feb-09
32351	22	1	46	0	44a4.00	Squamous cell carcinoma antigen level	Feb-09
32357	137	7	0	0	BBc0.00	[M]Ganglioneuromatous neoplasms	Feb-09
32362	75	1	0	0	B113.00	Malignant neoplasm of fundus of stomach	Feb-09
32372	46	1	0	0	B302100	Malignant neoplasm of thoracic vertebra	Feb-09
32464	188	17	0	0	C300000	Cystinosis	Feb-09
32472	46	0	0	0	BB9H.00	[M]Inflammatory carcinoma	Feb-09

For peer review only - http://bmjopen.bmj.com/site/about/guidelines.xhtml

32508	53	0	0	0	P738.00	Atresia of pulmonary artery with septal defect	Feb-09
32603	503	3	0	0	PJyy400	Fragile X syndrome	Feb-09
32641	528	15	0	0	BB5RA00	[M]Merkel cell carcinoma	Feb-09
32768	566	6	0	0	B325100	Malignant melanoma of breast	Feb-09
32874	54	0	0	0	C362z11	Acidaemia NOS	Feb-09
32919	169	8	0	0	SN5y011	Malignant hyperthermia due to anaesthesia	Feb-09
32955	100	1	0	0	B41y.00	Malignant neoplasm of other site of cervix	Feb-09
33259	58	11	0	0	ZV76600	[V]Screening for malignant neoplasm of skin	Feb-09
33271	286	35	0	0	B332200	Malignant neoplasm of pinna NEC	Feb-09
33333	168	2	0	0	B62..00	Other malignant neoplasm of lymphoid and histiocytic tissue	Feb-09
33334	35	2	0	0	F150.00	Werdnig - Hoffmann disease	Feb-09
33344	32	0	0	0	B65z.00	Myeloid leukaemia NOS	Feb-09
33353	101	2	0	0	F11y000	Reye's syndrome	Feb-09
33388	15	0	0	0	B071000	Malignant neoplasm of adenoid	Feb-09
33390	63	6	0	0	C375112	Hurler's syndrome	Feb-09
33444	316	4	0	0	B221100	Malignant neoplasm of hilus of lung	Feb-09
33522	69	1	0	0	PKy6300	Smith - Lemli - Opitz syndrome	Feb-09
33617	429	4	0	0	B43z.00	Malignant neoplasm of body of uterus NOS	Feb-09
33636	104	2	0	0	BBQ7200	"[M]Teratoma, malignant, NOS"	Feb-09
33642	209	2	0	0	PJ2..00	Edward's syndrome - trisomy 18	Feb-09
33682	237	4	0	0	B337400	Malignant neoplasm of skin of lower leg	Feb-09
33775	354	3	0	0	BB5J.00	[M]Adenoid cystic carcinoma	Feb-09
33811	17	1	0	0	PG4D.00	Metaphyseal chondrodysplasia	Feb-09
33833	200	2	0	0	B301.00	Malignant neoplasm of mandible	Feb-09
33843	377	4	0	0	B583.00	Secondary malignant neoplasm of brain and spinal cord	Feb-09

For peer review only - http://bmjopen.bmj.com/site/about/guidelines.xhtml

33869	118	0	0	0	BBgR.00	"[M]Malignant lymphoma, large cell, diffuse NOS"	Feb-09
33871	74	4	0	0	B122.00	Malignant neoplasm of ileum	Feb-09
33897	131	6	0	0	BB4z.00	[M]Transitional cell papilloma or carcinoma NOS	Feb-09
33939	199	0	0	0	P21z.00	Microcephalus NOS	Feb-09
33948	84	1	0	0	PJ32.11	Wolff - Hirschorn syndrome	Feb-09
33997	115	1	0	0	B332000	Malignant neoplasm of skin of auricle (ear)	Feb-09
34000	97	1	0	0	BB80100	[M]Cystadenocarcinoma NOS	Feb-09
34012	208	4	0	0	B08..00	Malignant neoplasm of hypopharynx	Feb-09
34015	182	2	0	0	BB5S200	[M]Bronchiolo-alveolar adenocarcinoma	Feb-09
34030	173	3	0	0	BBL0.00	[M]Endometrial stromal sarcoma	Feb-09
34089	6	0	0	0	B62y400	Malignant lymphoma NOS of lymph nodes of axilla and arm	Feb-09
34096	71	2	0	0	BB5b.00	[M]Granular cell carcinoma	Feb-09
34110	100	3	0	0	BB5R100	"[M]Carcinoid tumour, malignant"	Feb-09
34145	5	0	0	0	B58y600	Secondary malignant neoplasm of testis	Feb-09
34161	61	1	0	0	P225.00	Holoprosencephaly	Feb-09
34252	61	1	0	0	BBb0.12	[M]Gliosarcoma	Feb-09
34259	59	2	0	0	B325300	Malignant melanoma of groin	Feb-09
34269	13	0	0	0	BB69100	[M]Sebaceous adenocarcinoma	Feb-09
34352	121	1	0	0	BBgG.12	[M]Lymphoblastic lymphoma NOS	Feb-09
34388	1117	9	0	0	B17z.00	Malignant neoplasm of pancreas NOS	Feb-09
34395	164	8	0	0	BB24.00	[M]Verrucous carcinoma NOS	Feb-09
34409	4	0	0	0	B010000	Malignant neoplasm of base of tongue dorsal surface	Feb-09
34451	489	11	0	0	B31..00	Malignant neoplasm of connective and other soft tissue	Feb-09
34692	24	3	0	0	B68y.00	Other leukaemia of unspecified cell type	Feb-09
34704	11	0	0	0	F390100	Central core disease	Feb-09
34713	88	2	0	0	BBc0000	[M]Ganglioneuroma	Feb-09

For peer review only - http://bmjopen.bmj.com/site/about/guidelines.xhtml

34742	73	2	0	0	B23z.00	Malignant neoplasm of pleura NOS	Feb-09
34754	46	0	0	0	D200100	Fanconi's familial refractory anaemia	Feb-09
34763	488	16	0	0	BBbT.00	[M]Medulloblastoma NOS	Feb-09
34792	262	7	0	0	F250500	Lennox-Gastaut syndrome	Feb-09
34878	2	0	0	0	B308300	Malignant neoplasm of medial cuneiform	Feb-09
34879	1	0	0	0	BB5J.11	[M]Cylindroid adenocarcinoma	Feb-09
34891	32	0	0	0	BBF2.00	[M]Sarcomatosis NOS	Feb-09
34921	69	1	0	0	P22y111	Joubert syndrome	Feb-09
34950	84	1	0	0	P223.11	Lissencephaly	Feb-09
34984	131	7	0	0	BB80.00	[M]Cystadenoma and carcinoma	Feb-09
35034	101	4	0	0	BBGJ.11	[M]Fibroxanthosarcoma	Feb-09
35039	19	1	0	0	B163.00	"Malignant neoplasm, overlapping lesion of biliary tract"	Feb-09
35071	141	0	0	0	BBQB.00	[M]Mixed germ cell tumour	Feb-09
35113	158	1	0	0	Byu9.00	[X]Malignant neoplasm of urinary tract	Feb-09
35180	189	1	0	0	Byu1.00	[X]Malignant neoplasm of digestive organs	Feb-09
35186	35	1	0	0	ByuC.00	"[X]Malignant neoplasm of ill-defined, secondary and unspeci"	Feb-09
35223	10	0	0	0	BBQ1100	[M]Spermatocytic seminoma	Feb-09
35285	139	0	1	0	ByuA.00	"[X]Malignant neoplasm of eye, brain and other parts of cent"	Feb-09
35325	149	0	0	0	Byu2.00	[X]Malignant neoplasm of respiratory and intrathoracic orga	Feb-09
35348	86	1	0	0	BB5T100	[M]Papillary adenocarcinoma NOS	Feb-09
35357	961	13	0	0	B14..00	"Malignant neoplasm of rectum, rectosigmoid junction and anus"	Feb-09
35364	29	0	0	0	B576000	Secondary malignant neoplasm of retroperitoneum	Feb-09
35420	102	2	0	0	PH3y900	Epidermolysis bullosa dystrophica	Feb-09
35457	251	8	0	0	BB35.00	[M]Basosquamous carcinoma	Feb-09
35467	133	1	0	0	BB5az00	[M]Renal adenoma or carcinoma NOS	Feb-09

For peer review only - http://bmjopen.bmj.com/site/about/guidelines.xhtml

35474	34	0	0	0	BB1C.00	[M]Giant cell carcinoma	Feb-09
35535	138	6	0	0	B173.00	Malignant neoplasm of pancreatic duct	Feb-09
35540	434	10	0	0	F240100	Spastic tetraplegia	Feb-09
35649	54	4	1	0	C31y200	Oxalosis	Feb-09
35665	112	5	0	0	PJ1..00	Patau's syndrome - trisomy 13	Feb-09
35697	45	0	0	0	BBr6.00	[M]Myeloid leukaemias	Feb-09
35747	3	0	0	0	BB5j100	"[M]Endometrioid adenoma, borderline malignancy"	Feb-09
35795	6	0	0	0	B174.00	Malignant neoplasm of Islets of Langerhans	Feb-09
35875	39	5	0	0	B66..00	Monocytic leukaemia	Feb-09
35963	160	1	0	0	B492.00	Malignant neoplasm of lateral wall of urinary bladder	Feb-09
35975	146	6	0	0	BB5z.00	[M]Adenoma or adenocarcinoma NOS	Feb-09
35999	12	0	0	0	B582200	Secondary malignant neoplasm of skin of neck	Feb-09
36031	33	3	0	0	BB5D.00	[M]Hepatobiliary tract adenomas and carcinomas	Feb-09
36114	615	53	0	0	BBg1.00	[M]Malignant lymphoma NOS	Feb-09
36147	553	5	0	0	B153.00	Secondary malignant neoplasm of liver	Feb-09
36161	36	3	0	0	B012.00	"Malignant neoplasm of tongue, tip and lateral border"	Feb-09
36221	49	0	0	0	BB5Sz00	[M]Respiratory tract adenoma or adenocarcinoma NOS	Feb-09
36242	81	0	0	0	BBM7.11	"[M]Cystosarcoma phyllodes, benign"	Feb-09
36286	9	0	0	0	BB5Lz00	[M]Adenomatous or adenocarcinomatous polyp NOS	Feb-09
36325	77	1	0	0	B470300	Teratoma of undescended testis	Feb-09
36371	95	1	0	0	B225.00	Malignant neoplasm of overlapping lesion of bronchus & lung	Feb-09
36401	159	2	0	0	B587.00	Secondary malignant neoplasm of adrenal gland	Feb-09

For peer review only - http://bmjopen.bmj.com/site/about/guidelines.xhtml

36433	F152000	133	0	8	0	Amyotrophic lateral sclerosis	Feb-09
36495	76	3	0	0	B161211	Carcinoma common bile duct	Feb-09
36530	85	3	0	0	BB5S211	[M]Alveolar cell carcinoma	Feb-09
36566	16	0	0	0	F390400	Nemaline body disease	Feb-09
36567	47	0	0	0	PKy6100	Cockayne syndrome	Feb-09
36622	97	0	0	0	C370111	Meconium ileus in cystic fibrosis	Feb-09
36693	75	4	0	0	ZV10600	[V]Personal history of leukaemia	Feb-09
36716	61	1	0	0	B04z.00	Malignant neoplasm of floor of mouth NOS	Feb-09
36731	129	3	0	0	B331000	Malignant neoplasm of canthus	Feb-09
36785	217	28	0	0	BBe1.11	[M]Multiple neurofibromatosis	Feb-09
36839	123	3	0	0	PFy1.00	Larsen's syndrome	Feb-09
36870	48	0	0	0	BBL7111	[M]Adenosarcoma	Feb-09
36876	1	0	0	0	BB5V311	[M]Eosinophil carcinoma	Feb-09
36882	137	3	0	0	BB5X.00	[M]Clear cell adenomas and adenocarcinomas	Feb-09
36899	31	2	0	0	B327800	Malignant melanoma of toe	Feb-09
36949	55	0	0	0	B49y.00	Malignant neoplasm of other site of urinary bladder	Feb-09
37006	31	1	0	0	A789000	HIV disease resulting in mycobacterial infection	Feb-09
37014	180	9	0	0	Eu02200	[X]Dementia in Huntington's disease	Feb-09
37016	43	1	0	0	B33..14	Malignant neoplasm of sebaceous gland	Feb-09
37096	6	0	0	0	B015.00	"Malignant neoplasm of tongue, junctional zone"	Feb-09
37112	7	0	0	0	B6...11	Malignant neoplasm of histiocytic tissue	Feb-09
37137	173	3	0	0	BB51100	[M]Adenocarcinoma in situ in tubulovillous adenoma	Feb-09
37165	339	16	0	0	B334000	Malignant neoplasm of scalp	Feb-09
37182	78	3	0	0	B63..00	Multiple myeloma and immunoproliferative neoplasms	Feb-09
37272	129	1	0	0	B67..00	Other specified leukaemia	Feb-09

For peer review only - http://bmjopen.bmj.com/site/about/guidelines.xhtml

37306	255	18	0	0	ZV10415	[V]Personal history of malignant neoplasm of prostate	Feb-09
37328	287	6	0	0	B450.00	Malignant neoplasm of vagina	Feb-09
37354	74	0	0	0	BB5X100	[M]Clear cell adenocarcinoma NOS	Feb-09
37410	61	0	0	0	BBr2100	[M]Acute lymphoid leukaemia	Feb-09
37461	44	0	0	0	B64y200	Adult T-cell leukaemia	Feb-09
37473	31	1	1	0	BBbW.00	[M]Cerebellar sarcoma NOS	Feb-09
37477	48	2	0	0	BBe7.11	"[M]Schwannoma, malignant"	Feb-09
37510	20	0	0	0	BBLG.00	[M]Carcinoma in pleomorphic adenoma	Feb-09
37516	32	1	0	0	B054.00	Malignant neoplasm of uvula	Feb-09
37539	115	8	0	0	D2...00	Aplastic and other anaemias	Feb-09
37542	2	0	0	0	BBQ7300	[M]Teratocarcinoma	Feb-09
37549	8	0	0	0	B05z000	Kaposi's sarcoma of palate	Feb-09
37553	17	1	0	0	B007.00	"Malignant neoplasm of lip, unspecified"	Feb-09
37590	88	4	0	0	B052.00	Malignant neoplasm of hard palate	Feb-09
37618	70	3	0	0	B551000	Malignant neoplasm of axilla NOS	Feb-09
37680	135	0	0	0	BBGF.00	"[M]Fibrous histiocytoma, malignant"	Feb-09
37688	27	0	0	0	BBA2.00	[M]Acinar cell carcinoma	Feb-09
37723	48	1	0	0	BBr6011	[M]Granulocytic leukaemia NOS	Feb-09
37724	58	0	0	0	B056.00	Malignant neoplasm of retromolar area	Feb-09
37793	30	0	89	0	4M4..00	FIGO staging of gynaecological malignancy	Feb-09
37805	22	0	0	0	B213100	Malignant neoplasm of cricoid cartilage	Feb-09
37810	22	0	0	0	B220z00	Malignant neoplasm of trachea NOS	Feb-09
37842	61	2	0	0	B303000	Malignant neoplasm of rib	Feb-09
37859	37	0	0	0	B110z00	Malignant neoplasm of cardia of stomach NOS	Feb-09
37872	376	6	0	0	B327400	Malignant melanoma of lower leg	Feb-09
37916	32	0	0	0	B05y.00	Malignant neoplasm of other specified mouth parts	Feb-09
37940	58	1	0	0	B072000	Malignant neoplasm of pharyngeal recess	Feb-09

For peer review only - http://bmjopen.bmj.com/site/about/guidelines.xhtml

37969	118	3	0	0	B335100	"Malignant neoplasm of skin of chest, excluding breast"	Feb-09
38286	72	3	0	0	A411.00	Jakob-Creutzfeldt disease	Feb-09
38331	21	0	0	0	B64yz00	Other lymphoid leukaemia NOS	Feb-09
38411	14	0	0	0	7904z00	Other correction of transposition of great vessels NOS	Feb-09
38442	35	0	0	0	BB81200	"[M]Serous cystadenocarcinoma, NOS"	Feb-09
38454	193	4	0	0	BB48.00	[M]Basaloid carcinoma	Feb-09
38475	56	1	0	0	B34yz00	Malignant neoplasm of other site of female breast NOS	Feb-09
38479	77	3	0	0	PFy0.00	Arthrogryposis multiplex congenita	Feb-09
38481	10	0	0	0	BBTK.00	"[M]Epithelioid haemangioendothelioma, malignant"	Feb-09
38488	3	0	0	0	B013z00	Malignant neoplasm of ventral tongue surface NOS	Feb-09
38510	1032	21	0	0	B47z.00	Malignant neoplasm of testis NOS	Feb-09
38551	36	0	0	0	BBb1.00	[M]Gliomatosis cerebri	Feb-09
38575	8	1	0	0	BB62100	[M]Apocrine adenocarcinoma	Feb-09
38608	136	2	0	0	L250.11	Suspect fetal anencephaly	Feb-09
38685	115	1	0	0	BB5fz00	[M]Thyroid adenoma or adenocarcinoma NOS	Feb-09
38689	245	2	0	0	B325.00	Malignant melanoma of trunk (excluding scrotum)	Feb-09
38736	80	0	0	0	B5y..00	Malignant neoplasm of other and unspecified site OS	Feb-09
38756	5	0	0	0	BBP9.00	[M]Cystic mesothelioma	Feb-09
38770	5	0	0	0	BBB7.00	[M]Epithelial-myoepithelial carcinoma	Feb-09
38862	47	1	0	0	B490.00	Malignant neoplasm of trigone of urinary bladder	Feb-09
38914	57	1	0	0	B64z.00	Lymphoid leukaemia NOS	Feb-09
38918	148	1	0	0	B583100	Secondary malignant neoplasm of spinal cord	Feb-09

For peer review only - http://bmjopen.bmj.com/site/about/guidelines.xhtml

38931	50	0	0	0	B4y..00	Malignant neoplasm of genitourinary organ OS	Feb-09
38938	28	1	0	0	B306z00	"Malignant neoplasm of pelvis, sacrum or coccyx NOS"	Feb-09
38939	36	0	0	0	B613.00	"Hodgkin's disease, lymphocytic-histiocytic predominance"	Feb-09
38961	408	2	0	0	B22y.00	Malignant neoplasm of other sites of bronchus or lung	Feb-09
38978	50	0	0	0	B15z.00	Malignant neoplasm of liver and intrahepatic bile ducts NOS	Feb-09
39017	29	1	0	0	PJ2z.00	Edward's syndrome NOS	Feb-09
39023	130	0	0	0	F256.12	West syndrome	Feb-09
39027	103	0	0	0	ByuC000	[X]Malignant neoplasm of other specified sites	Feb-09
39038	88	0	0	0	BB85.00	[M]Signet ring carcinoma	Feb-09
39084	402	10	0	0	B0z2.00	Malignant neoplasm of laryngopharynx	Feb-09
39088	35	0	0	0	B514.00	Malignant neoplasm of occipital lobe	Feb-09
39121	13	1	0	0	BBc0100	[M]Ganglioneuroblastoma	Feb-09
39148	15	0	0	0	BB5Mz00	[M]Tubular adenoma or adenocarcinoma NOS	Feb-09
39187	110	4	0	0	B631.00	Plasma cell leukaemia	Feb-09
39312	28	0	0	0	BBM8.00	[M]Cystosarcoma phyllodes NOS	Feb-09
39386	8	0	0	0	BBb2.11	[M]Mixed glioma	Feb-09
39388	50	0	0	0	BBcC.11	[M]Olfactory neuroblastoma	Feb-09
39413	72	0	0	0	B18y500	Malignant neoplasm of pelvic peritoneum	Feb-09
39430	476	6	0	0	B0zz.00	"Malignant neoplasm of lip, oral cavity and pharynx NOS"	Feb-09
39522	60	4	0	0	BBV..00	[M]Osteomas and osteosarcomas	Feb-09
39554	21	0	0	0	B063.00	Malignant neoplasm of vallecula	Feb-09
39590	3	1	0	0	B206.00	"Malignant neoplasm, overlapping lesion of accessory sinuses"	Feb-09
39629	16	0	0	0	B653100	Granulocytic sarcoma	Feb-09

For peer review only - http://bmjopen.bmj.com/site/about/guidelines.xhtml

39760	129	3	0	0	BB91100	[M]Infiltrating duct and lobular carcinoma	Feb-09
39798	117	1	0	0	B627X00	"Diffuse non-Hodgkin's lymphoma, unspecified"	Feb-09
39870	131	1	0	0	B172.00	Malignant neoplasm of tail of pancreas	Feb-09
39875	46	0	0	0	BB5Nz00	[M]Adenomatous or adenocarcinomatous polyps of the colon NOS	Feb-09
39878	7	0	0	0	B327300	Malignant melanoma of popliteal fossa area	Feb-09
39897	102	1	0	0	B081.00	Malignant neoplasm of pyriform sinus	Feb-09
39899	34	5	0	0	B542100	Malignant neoplasm of craniopharyngeal duct	Feb-09
39906	4	0	0	0	BBgE.00	"[M]Malignant lymphoma, centrocytic"	Feb-09
39923	293	1	0	0	B223100	Malignant neoplasm of middle lobe of lung	Feb-09
39945	86	0	0	0	J600011	Acute liver failure	Feb-09
40014	112	2	0	0	B310100	Malignant neoplasm of soft tissue of face	Feb-09
40240	291	11	0	0	BB5D500	[M]Hepatocellular carcinoma NOS	Feb-09
40292	193	1	0	0	B053.00	Malignant neoplasm of soft palate	Feb-09
40344	32	0	0	0	F200.00	Multiple sclerosis of the brain stem	Feb-09
40359	33	1	0	0	BB94.00	[M]Juvenile breast carcinoma	Feb-09
40420	6	0	0	0	BBr0.00	[M]Leukaemias unspecified	Feb-09
40437	20	0	0	0	B50y.00	Malignant neoplasm of other specified site of eye	Feb-09
40438	40	2	0	0	BB5D111	[M]Bile duct carcinoma	Feb-09
40443	19	0	0	0	B33..15	Malignant neoplasm of sweat gland	Feb-09
40492	17	0	0	0	BBe9.00	"[M]Triton tumour, malignant"	Feb-09
40508	4	0	0	0	BBj6000	"[M]Hodgkin,s disease, nodular sclerosis, lymphocytic predom"	Feb-09
40513	66	1	0	0	BBkz.00	"[M]Lymphoma, nodular or follicular NOS"	Feb-09
40557	136	0	0	0	B01z.00	Malignant neoplasm of tongue NOS	Feb-09
40561	75	4	0	0	ZV10711	[V]Personal history of Hodgkin's disease	Feb-09

For peer review only - http://bmjopen.bmj.com/site/about/guidelines.xhtml

40592	11	0	0	0	Byu5.00	[X]Malignant neoplasm of mesothelial and soft tissue	Feb-09
40595	102	2	0	0	Byu2000	"[X]Malignant neoplasm of bronchus or lung, unspecified"	Feb-09
40598	130	2	0	0	Byu7.00	[X]Malignant neoplasm of female genital organs	Feb-09
40622	29	0	0	0	BB5V711	[M]Mucoid cell carcinoma	Feb-09
40632	150	5	0	0	BB82.00	[M]Mucinous adenoma and adenocarcinoma	Feb-09
40671	93	0	0	0	Byu8.00	[X]Malignant neoplasm of male genital organs	Feb-09
40740	42	0	0	0	ByuD.00	"[X]Malignant neoplasms of lymphoid, haematopoietic and rela"	Feb-09
40749	10	0	0	0	Byu3.00	[X]Malignant neoplasm of bone and articular cartilage	Feb-09
40766	178	2	0	0	BBm5.00	[M] Peripheral T-cell lymphoma NOS	Feb-09
40810	125	1	0	0	B171.00	Malignant neoplasm of body of pancreas	Feb-09
40814	36	3	0	0	B307200	Malignant neoplasm of tibia	Feb-09
40966	24	0	0	0	B306300	Malignant neoplasm of sacral vertebra	Feb-09
40991	47	1	41	0	4M2..00	Lymphoma staging system	Feb-09
41142	21	1	0	0	D204.00	Idiopathic aplastic anaemia	Feb-09
41144	33	0	0	0	B582300	Secondary malignant neoplasm of skin of trunk	Feb-09
41185	20	0	0	0	Eu02400	[X]Dementia in human immunodef virus [HIV] disease	Feb-09
41215	120	4	0	0	B111100	Malignant neoplasm of pyloric canal of stomach	Feb-09
41245	372	5	0	0	F030411	Herpes simplex encephalitis	Feb-09
41278	107	1	0	0	B323000	Malignant melanoma of external surface of cheek	Feb-09
41313	19	0	0	0	BB5D300	[M]Bile duct cystadenocarcinoma	Feb-09

For peer review only - http://bmjopen.bmj.com/site/about/guidelines.xhtml

41362	35	0	0	0	B101.00	Malignant neoplasm of thoracic oesophagus	Feb-09
41369	6	0	0	0	B60..00	Lymphosarcoma and reticulosarcoma	Feb-09
41461	107	9	0	0	PKy0.11	Prader-Willi Syndrome	Feb-09
41488	20	2	0	0	G554100	Constrictive cardiomyopathy	Feb-09
41490	68	0	0	0	B327700	Malignant melanoma of foot	Feb-09
41500	60	2	0	0	BBr2300	[M]Chronic lymphoid leukaemia	Feb-09
41515	69	0	0	0	ByuA100	"[X]Malignant neoplasm/central nervous system, unspecified"	Feb-09
41520	405	1	0	0	B51z.00	Malignant neoplasm of brain NOS	Feb-09
41523	66	0	0	0	B223000	Malignant neoplasm of middle lobe bronchus	Feb-09
41530	7	0	0	0	B01y.00	Malignant neoplasm of other sites of tongue	Feb-09
41571	93	0	0	0	B495.00	Malignant neoplasm of bladder neck	Feb-09
41695	85	0	0	0	BBba.00	[M]Primitive neuroectodermal tumour	Feb-09
41702	100	3	0	0	BB5N.00	[M]Adenomatous and adenocarcinomatous polyps of colon	Feb-09
41734	17	1	0	0	BBr0000	[M]Leukaemia NOS	Feb-09
41754	52	10	0	0	BBg7.00	"[M]Malignant lymphoma, lymphoplasmacytoid type"	Feb-09
41803	51	2	0	0	BBf..00	[M]Granular cell tumours and alveolar soft part sarcoma	Feb-09
41816	63	1	0	0	BB2E.00	"[M]Squamous cell carcinoma, small cell, non-keratinising"	Feb-09
41841	12	0	0	0	BBgB.00	"[M]Malignant lymphoma, follicular centre cell NOS"	Feb-09
41931	87	0	0	0	B550100	Malignant neoplasm of cheek NOS	Feb-09
41958	141	2	0	0	B331200	Malignant neoplasm of lower eyelid	Feb-09
42012	97	1	0	0	B494.00	Malignant neoplasm of posterior wall of urinary bladder	Feb-09
42017	48	1	0	0	C306400	Hyperammonaemia	Feb-09

For peer review only - http://bmjopen.bmj.com/site/about/guidelines.xhtml

42023	13	1	0	0	B497.00	Malignant neoplasm of urachus	Feb-09
42070	178	2	0	0	B345.00	Malignant neoplasm of lower-outer quadrant of female breast	Feb-09
42082	36	0	0	0	BBK3700	[M]Alveolar rhabdomyosarcoma	Feb-09
42153	58	0	0	0	B32y.00	Malignant melanoma of other specified skin site	Feb-09
42169	432	2	0	0	BB5cz00	[M]Parathyroid adenoma or adenocarcinoma NOS	Feb-09
42193	109	0	0	0	B115.00	Malignant neoplasm of lesser curve of stomach unspecified	Feb-09
42198	35	1	0	0	BBj6.00	"[M]Hodgkin's disease, nodular sclerosis NOS"	Feb-09
42218	62	0	0	0	B55y.00	Malignant neoplasm of other specified sites	Feb-09
42273	134	4	0	0	BB5T.00	[M]Papillary adenomas and adenocarcinomas	Feb-09
42297	100	0	0	0	BBrz.00	[M]Leukaemia NOS	Feb-09
42416	589	10	0	0	B105.00	Malignant neoplasm of lower third of oesophagus	Feb-09
42426	190	3	0	0	B511.00	Malignant neoplasm of frontal lobe	Feb-09
42429	9	6	0	0	B33X.00	Malignant neoplasm overlapping lesion of skin	Feb-09
42460	43	0	0	0	B543.00	Malignant neoplasm of pineal gland	Feb-09
42461	139	2	0	0	B61zz00	Hodgkin's disease NOS	Feb-09
42497	6	0	0	0	P100.00	Unspecified spina bifida with hydrocephalus	Feb-09
42539	54	2	0	0	B670.00	Acute erythraemia and erythroleukaemia	Feb-09
42542	83	3	0	0	BB9K.00	[M]Paget's disease and infiltrating breast duct carcinoma	Feb-09
42553	17	1	0	0	BBB3.00	[M]Adenocarcinoma with cartilaginous and osseous metaplasia	Feb-09

For peer review only - http://bmjopen.bmj.com/site/about/guidelines.xhtml

42566	167	0	0	0	B224z00	"Malignant neoplasm of lower lobe, bronchus or lung NOS"	Feb-09
42569	196	3	0	0	B2zz.00	Malignant neoplasm of respiratory tract NOS	Feb-09
42579	13	0	0	0	B62y300	Malignant lymphoma NOS of intra-abdominal lymph nodes	Feb-09
42636	80	3	0	0	C307y11	Methylmalonic acidaemia	Feb-09
42643	33	1	0	0	PK64.00	Proteus syndrome	Feb-09
42707	49	0	0	0	B336100	Malignant neoplasm of skin of upper arm	Feb-09
42714	48	0	0	0	B327500	Malignant melanoma of ankle	Feb-09
42769	10	0	0	0	BBjz.00	[M]Hodgkin's disease NOS	Feb-09
42784	57	0	0	0	P226.00	Microgyria	Feb-09
42856	27	0	0	0	B200z00	Malignant neoplasm of nasal cavities NOS	Feb-09
43005	97	0	0	0	Q200012	Intracranial haemorrhage in fetus or newborn	Feb-09
43087	193	12	0	0	B331.00	Malignant neoplasm of eyelid including canthus	Feb-09
43111	68	1	0	0	B213.00	Malignant neoplasm of laryngeal cartilage	Feb-09
43122	111	1	0	0	B336000	Malignant neoplasm of skin of shoulder	Feb-09
43151	3	0	0	0	Byu3300	"[X]Malignant neoplasm/bone+articular cartilage, unspecified"	Feb-09
43200	67	1	0	0	B06z.00	Malignant neoplasm of oropharynx NOS	Feb-09
43203	132	7	0	0	F100.00	Leucodystrophy	Feb-09
43311	19	1	0	0	ZV10212	[V]Personal history of malignant neoplasm of larynx	Feb-09
43325	87	2	0	0	PKy7300	Rubenstein - Tayi syndrome	Feb-09
43390	173	0	0	0	B12z.00	Malignant neoplasm of small intestine NOS	Feb-09
43392	237	4	0	0	B483.00	"Malignant neoplasm of penis, part unspecified"	Feb-09
43400	135	3	0	0	B03..00	Malignant neoplasm of gum	Feb-09
43415	14	1	0	0	ByuD000	[X]Other Hodgkin's disease	Feb-09

For peer review only - http://bmjopen.bmj.com/site/about/guidelines.xhtml

43431	272	0	0	0	B010.00	Malignant neoplasm of base of tongue	Feb-09
43435	46	0	0	0	B41yz00	Malignant neoplasm of other site of cervix NOS	Feb-09
43450	41	0	0	0	B63z.00	Immunoproliferative neoplasm or myeloma NOS	Feb-09
43463	383	1	0	0	B325700	Malignant melanoma of back	Feb-09
43479	46	0	0	0	B121.00	Malignant neoplasm of jejunum	Feb-09
43490	41	0	0	0	Byu1100	[X]Other specified carcinomas of liver	Feb-09
43548	26	0	0	0	B080.00	Malignant neoplasm of postcricoid region	Feb-09
43565	6	0	0	0	PJ11.00	"Trisomy 13, mosaicism"	Feb-09
43572	142	1	0	0	B114.00	Malignant neoplasm of body of stomach	Feb-09
43582	52	0	0	0	BBQz.00	[M]Germ cell neoplasm NOS	Feb-09
43594	17	0	0	0	BB5Cz00	[M]Gastrinoma or carcinoma NOS	Feb-09
43614	13	0	0	0	B30X.00	"Malignant neoplasm/bones+articular cartilage/limb,unspfd"	Feb-09
43619	159	0	0	0	B334100	Malignant neoplasm of skin of neck	Feb-09
43642	10	0	0	0	B011.00	Malignant neoplasm of dorsal surface of tongue	Feb-09
43712	44	1	0	0	C375311	Sanfilippo syndrome	Feb-09
43715	6	0	0	0	B325600	Malignant melanoma of umbilicus	Feb-09
43717	12	0	0	0	BB24.11	[M]Verrucous epidermoid carcinoma	Feb-09
43761	44	5	0	0	B451.00	Malignant neoplasm of labia majora	Feb-09
43781	8	0	0	0	B011z00	Malignant neoplasm of dorsum of tongue NOS	Feb-09
43930	12	0	0	0	B582000	Secondary malignant neoplasm of skin of head	Feb-09
43940	13	0	0	0	B431.00	Malignant neoplasm of isthmus of uterine body	Feb-09
44074	28	1	0	0	BB84.00	[M]Mucin-producing adenocarcinoma	Feb-09
44089	27	0	0	0	B517.00	Malignant neoplasm of brain stem	Feb-09
44108	69	0	0	0	B18..00	Malignant neoplasm of retroperitoneum and peritoneum	Feb-09

For peer review only - http://bmjopen.bmj.com/site/about/guidelines.xhtml

44139	1	0	0	0	B073.00	Malignant neoplasm of anterior wall of nasopharynx	Feb-09
44157	5	0	0	0	BBE1.13	[M]Melanosarcoma NOS	Feb-09
44169	217	4	0	0	B222z00	"Malignant neoplasm of upper lobe, bronchus or lung NOS"	Feb-09
44196	11	1	0	0	B611.00	Hodgkin's granuloma	Feb-09
44201	11	0	0	0	PB57.00	Microcolon	Feb-09
44267	71	6	1	0	B623.00	Malignant histiocytosis	Feb-09
44272	33	0	0	0	F391B00	Cardiomyopathy in Duchenne muscular dystrophy	Feb-09
44288	132	2	0	0	R109.00	[D]Laboratory evidence of human immunodeficiency virus [HIV]	Feb-09
44318	144	7	0	0	B62xX00	Oth and unspecif peripheral & cutaneous T-cell lymphomas	Feb-09
44399	54	0	0	0	B150z00	Primary malignant neoplasm of liver NOS	Feb-09
44421	20	2	0	0	ZV67A00	[V]Folow-up exam aft other treatment for malignant neoplasm	Feb-09
44452	6	2	0	0	B300C00	Malignant neoplasm of vomer	Feb-09
44529	40	2	0	0	B575.00	Secondary malignant neoplasm of large intestine and rectum	Feb-09
44592	F11xz00	8	0	0	0	Cerebral degeneration other disease NOS	Feb-09
44609	18	0	0	0	B306000	Malignant neoplasm of ilium	Feb-09
44615	129	0	0	0	B586.00	Secondary malignant neoplasm of ovary	Feb-09
44617	13	0	0	0	A789600	HIV disease resulting in Burkitt's lymphoma	Feb-09
44759	9	0	0	0	PG52000	Osteopetrosis - unclassified	Feb-09
44778	94	0	0	0	BB52000	[M]Adenocarcinoma in tubulovillous adenoma	Feb-09
44871	331	0	1	0	C308000	Medium chain acyl-CoA dehydrogenase deficiency	Feb-09
44884	19	2	0	0	B4Ay.00	Malignant neoplasm of other urinary organs	Feb-09

For peer review only - http://bmjopen.bmj.com/site/about/guidelines.xhtml

44930	11	0	0	0	BB81800	[M]Papillary serous cystadenocarcinoma	Feb-09
44939	P251.00	48	0	1	0	Hydromyelia	Feb-09
44996	33	0	0	0	B491.00	Malignant neoplasm of dome of urinary bladder	Feb-09
45071	42	2	0	0	B314.00	Malignant neoplasm of connective and soft tissue of abdomen	Feb-09
45077	223	4	0	0	B335700	Malignant neoplasm of skin of back	Feb-09
45139	45	0	0	0	B323400	Malignant melanoma of external surface of nose	Feb-09
45154	117	4	0	0	B516.00	Malignant neoplasm of cerebellum	Feb-09
45222	118	1	0	0	B343.00	Malignant neoplasm of lower-inner quadrant of female breast	Feb-09
45260	9	0	0	0	Byu9000	"[X]Malignant neoplasm of urinary organ, unspecified"	Feb-09
45262	7	0	0	0	Byu8200	"[X]Malignant neoplasm of male genital organ, unspecified"	Feb-09
45264	20	0	0	0	B620100	"Nodular lymphoma of lymph nodes of head, face and neck"	Feb-09
45267	30	0	0	0	B55z.00	Malignant neoplasm of other and ill defined site NOS	Feb-09
45306	96	0	0	0	B324100	Malignant melanoma of neck	Feb-09
45307	52	0	0	0	B2...11	Carcinoma of respiratory tract and intrathoracic organs	Feb-09
45408	35	0	0	0	B040.00	Malignant neoplasm of anterior portion of floor of mouth	Feb-09
45458	33	1	0	0	BB2F.00	"[M]Squamous cell carcinoma, spindle cell type"	Feb-09
45490	67	0	0	0	B430z00	Malignant neoplasm of corpus uteri NOS	Feb-09
45510	30	0	0	0	BB2M.00	[M]Lymphoepithelial carcinoma	Feb-09
45531	33	1	0	0	BBbE.00	[M]Gemistocytic astrocytoma	Feb-09
45667	27	0	0	0	B501.00	Malignant neoplasm of orbit	Feb-09
45755	161	0	0	0	B326200	Malignant melanoma of fore-arm	Feb-09

For peer review only - http://bmjopen.bmj.com/site/about/guidelines.xhtml

45760	51	0	0	0	B325z00	"Malignant melanoma of trunk, excluding scrotum, NOS"	Feb-09
45766	11	0	0	0	Byu1200	"[X]Malignant neoplasm of intestinal tract, part unspecified"	Feb-09
45793	27	0	0	0	B430300	Malignant neoplasm of myometrium of corpus uteri	Feb-09
45824	7	1	0	0	B58y900	Secondary malignant neoplasm of tongue	Feb-09
45903	23	0	0	0	F100000	Krabbe's disease	Feb-09
45922	15	6	0	0	B508.00	"Malignant neoplasm, overlapping lesion of eye and adnexa"	Feb-09
45986	13	0	0	0	B041.00	Malignant neoplasm of lateral portion of floor of mouth	Feb-09
46008	93	2	0	0	B333z00	Malignant neoplasm skin other and unspec part of face NOS	Feb-09
46048	5	0	0	0	BBr2500	[M]Prolymphocytic leukaemia	Feb-09
46110	9	0	0	0	A75..12	Pfeiffer's disease	Feb-09
46128	F240000	9	0	0	0	Flaccid tetraplegia	Feb-09
46133	11	1	0	0	PJ12.00	"Trisomy 13, translocation"	Feb-09
46153	6	0	0	0	B443.00	Malignant neoplasm of parametrium	Feb-09
46159	6	0	0	0	B142000	Malignant neoplasm of cloacogenic zone	Feb-09
46255	393	4	0	0	B327.00	Malignant melanoma of lower limb and hip	Feb-09
46263	64	4	0	0	BBr6700	[M]Acute myelomonocytic leukaemia	Feb-09
46423	120	0	0	0	B933.11	Cystosarcoma phyllodes	Feb-09
46444	20	1	0	0	BBr4.00	[M]Erythroleukaemias	Feb-09
46458	8	0	0	0	B335600	Malignant neoplasm of skin of perineum	Feb-09
46516	51	3	0	0	C327200	Niemann-Pick disease	Feb-09
46548	42	1	0	0	B071100	Malignant neoplasm of pharyngeal tonsil	Feb-09
46581	140	0	0	0	BBF4.11	[M]Pleomorphic cell sarcoma	Feb-09
46583	6	5	0	0	P234.00	Hydranencephaly	Feb-09
46613	186	1	0	0	B18y.00	Malignant neoplasm of specified parts of peritoneum	Feb-09

For peer review only - http://bmjopen.bmj.com/site/about/guidelines.xhtml

For peer review only

46728	40	1	0	0	B064.00	Malignant neoplasm of anterior epiglottis	Feb-09
46741	3	0	0	0	BBZC.00	[M]Ameloblastic odontosarcoma	Feb-09
46761	53	0	0	0	BB5f600	[M]Papillary and follicular adenocarcinoma	Feb-09
46771	19	1	0	0	BB5D800	"[M]Hepatocellular carcinoma, fibrolamellar"	Feb-09
46779	26	0	0	0	ZV10417	[V]Personal history of malignant neoplasm of uterine body	Feb-09
46787	29	0	0	0	PJ2z.11	TRISOMY 18 NOS	Feb-09
46789	13	1	0	0	B515000	Malignant neoplasm of choroid plexus	Feb-09
46790	196	7	0	0	P10..00	Spina bifida with hydrocephalus	Feb-09
46792	111	1	0	0	B512.00	Malignant neoplasm of temporal lobe	Feb-09
46794	7	0	0	0	PH3y800	Epidermolysis bullosa letalis	Feb-09
46877	47	1	0	0	BBgL.00	"[M]Malignant lymphoma, small lymphocytic NOS"	Feb-09
46905	1	0	0	0	B545200	Malignant neoplasm of coccygeal body	Feb-09
46931	3	0	0	0	BBg4.00	"[M]Malignant lymphoma, stem cell type"	Feb-09
46939	21	1	0	0	B302000	Malignant neoplasm of cervical vertebra	Feb-09
47094	4	0	0	0	B323200	Malignant melanoma of eyebrow	Feb-09
47205	17	12	0	0	B017.00	Malignant overlapping lesion of tongue	Feb-09
47252	59	1	0	0	B323.00	Malignant melanoma of other and unspecified parts of face	Feb-09
47286	20	0	0	0	B551.00	Malignant neoplasm of thorax	Feb-09
47288	63	0	0	0	P102z00	Spina bifida with hydrocephalus - open NOS	Feb-09
47342	29	1	0	0	Q48y000	Congenital renal failure	Feb-09
47556	30	0	0	0	B512z00	Malignant neoplasm of temporal lobe NOS	Feb-09
47668	4	1	0	0	B48y100	Malignant neoplasm of tunica vaginalis	Feb-09
47683	41	0	0	0	ZV10512	[V]Personal history of malignant neoplasm of kidney	Feb-09

For peer review only - http://bmjopen.bmj.com/site/about/guidelines.xhtml

47734	38	0	0	0	BBP5.00	"[M]Epithelioid mesothelioma, malignant"	Feb-09
47767	111	4	0	0	B486.00	Malignant neoplasm of scrotum	Feb-09
47801	6	0	0	0	B49y000	"Malignant neoplasm, overlapping lesion of bladder"	Feb-09
47810	234	8	0	0	B59..00	Malignant neoplasm of unspecified site	Feb-09
47840	5	0	0	0	B545100	Malignant neoplasm of aortic body	Feb-09
47862	96	2	0	0	B213300	Malignant neoplasm of thyroid cartilage	Feb-09
47899	17	1	0	0	B451000	Malignant neoplasm of greater vestibular (Bartholin's) gland	Feb-09
47920	17	0	0	0	BB9B.11	[M]C cell carcinoma	Feb-09
47974	28	4	0	0	F130600	Aicardi Goutieres syndrome	Feb-09
48048	53	1	0	0	BB1B.00	[M]Giant cell and spindle cell carcinoma	Feb-09
48049	22	0	0	0	BBr6800	[M]Chronic myelomonocytic leukaemia	Feb-09
48073	20	2	0	0	B510000	Malignant neoplasm of basal ganglia	Feb-09
48084	19	0	0	0	PKy9400	Zellweger's syndrome	Feb-09
48126	23	7	0	0	F232.11	Tetraplegia - congenital	Feb-09
48145	19	0	0	0	D212000	Anaemia in ovarian carcinoma	Feb-09
48155	17	1	0	0	BBr2.00	[M]Lymphoid leukaemias	Feb-09
48184	24	0	0	0	PG4E.00	Spondylometaphyseal dysplasia	Feb-09
48211	17	0	0	0	F10y000	Alper's disease	Feb-09
48223	31	1	0	0	BB54.00	[M]Scirrhous adenocarcinoma	Feb-09
48231	68	0	0	0	B13y.00	Malignant neoplasm of other specified sites of colon	Feb-09
48237	30	1	0	0	B111000	Malignant neoplasm of prepylorus of stomach	Feb-09
48253	7	0	0	0	BBg8.00	"[M]Malignant lymphoma, immunoblastic type"	Feb-09
48271	16	0	0	0	BBVz.00	[M]Osteoma or osteosarcoma NOS	Feb-09
48275	50	0	0	0	BBK3600	[M]Embryonal rhabdomyosarcoma	Feb-09
48288	11	1	0	0	R210200	[D]Nonspecific sudden infant death	Feb-09
48293	15	0	0	0	C392100	Severe combined immunodeficiency	Feb-09

For peer review only - http://bmjopen.bmj.com/site/about/guidelines.xhtml

48300	51	3	0	0	F10y100	Leigh's disease	Feb-09
48517	85	10	0	0	B310200	Malignant neoplasm of soft tissue of neck	Feb-09
48519	5	0	0	0	B065.00	Malignant neoplasm of junctional region of epiglottis	Feb-09
48531	75	4	0	0	F11x700	Cerebral degeneration due to Jakob - Creutzfeldt disease	Feb-09
48537	8	0	0	0	B17y.00	Malignant neoplasm of other specified sites of pancreas	Feb-09
48609	P118.00	5	0	0	0	Spina bifida without hydrocephalus - closed	Feb-09
48697	11	0	0	0	7A65000	Transposition of valve of vein	Feb-09
48743	57	0	0	0	B482.00	Malignant neoplasm of body of penis	Feb-09
48809	49	0	0	0	B35zz00	Malignant neoplasm of male breast NOS	Feb-09
48820	246	0	0	0	B410.00	Malignant neoplasm of endocervix	Feb-09
48828	13	0	0	0	B582500	Secondary malignant neoplasm of skin of hip and leg	Feb-09
48879	12	0	0	0	J637.00	Hepatic veno-occlusive disease	Feb-09
48952	87	1	0	0	BBc9z00	[M]Retinoblastoma NOS	Feb-09
48991	0	2	4	0	5136	X-ray metastasis control	Feb-09
49023	10	1	0	0	BBY0.11	[M]Endothelial bone sarcoma	Feb-09
49054	28	0	0	0	B304000	Malignant neoplasm of scapula	Feb-09
49131	14	1	0	0	BBg0.00	"[M]Lymphomatous tumour, benign"	Feb-09
49132	7	0	0	0	B517100	Malignant neoplasm of medulla oblongata	Feb-09
49145	8	0	0	0	B58y700	Secondary malignant neoplasm of penis	Feb-09
49148	21	1	0	0	B347.00	"Malignant neoplasm, overlapping lesion of breast"	Feb-09
49168	15	1	0	0	BBb4.00	[M]Subependymal giant cell astrocytoma	Feb-09
49181	19	0	0	0	F101400	Gangliosidosis	Feb-09
49186	118	5	0	0	BBbR.00	"[M]Oligodendroglioma, anaplastic type"	Feb-09
49253	12	0	0	0	BBk0.13	[M]Giant follicular lymphoma	Feb-09

For peer review only - http://bmjopen.bmj.com/site/about/guidelines.xhtml

49262	55	2	0	0	B627200	Follicular non-Hodgkin's large cell lymphoma	Feb-09
49292	12	0	0	0	Byu1300	[X]Malignant neoplsm/ill-defin sites within digestive system	Feb-09
49301	437	30	0	0	B6z..00	Malignant neoplasm lymphatic or haematopoietic tissue NOS	Feb-09
49322	61	0	0	0	F256z00	Infantile spasms NOS	Feb-09
49327	8	0	0	0	BBrA500	[M]Acute megakaryoblastic leukaemia	Feb-09
49341	3	0	3	0	4D56.00	Pleural fluid: malignant cells	Feb-09
49360	30	2	0	0	B031.00	Malignant neoplasm of lower gum	Feb-09
49400	260	1	0	0	B430211	Malignant neoplasm of endometrium	Feb-09
49403	55	3	0	0	B333100	Malignant neoplasm of skin of chin	Feb-09
49463	11	0	0	0	B310400	Malignant neoplasm of tarsus of eyelid	Feb-09
49482	3	0	0	0	F396200	Myopathy due to malignant disease	Feb-09
49491	31	0	0	0	B303100	Malignant neoplasm of sternum	Feb-09
49525	54	1	0	0	B59zX00	"Kaposi's sarcoma, unspecified"	Feb-09
49542	6	0	0	0	C392500	Severe combined immunodef with low or normal B-cell numbers	Feb-09
49605	43	1	0	0	B615.00	"Hodgkin's disease, mixed cellularity"	Feb-09
49629	7	0	0	0	BB5C100	"[M]Gastrinoma, malignant"	Feb-09
49701	21	1	0	0	B302z00	Malignant neoplasm of vertebral column NOS	Feb-09
49714	10	0	0	0	B523.00	Malignant neoplasm of spinal meninges	Feb-09
49725	20	0	0	0	B64y.00	Other lymphoid leukaemia	Feb-09
49758	22	1	0	0	B0zy.00	"Malignant neoplasm of other sites lip, oral cavity, pharynx"	Feb-09
49770	304	7	0	0	C370z00	Cystic fibrosis NOS	Feb-09
49814	59	1	0	0	B325000	Malignant melanoma of axilla	Feb-09
49825	23	0	0	0	BBh0.11	[M]Reticulum cell sarcoma NOS	Feb-09
49828	174	3	0	0	B441.00	Malignant neoplasm of fallopian tube	Feb-09
49862	42	0	0	0	BBV1.11	[M]Osteoblastic sarcoma	Feb-09

For peer review only - http://bmjopen.bmj.com/site/about/guidelines.xhtml

49875	18	0	0	0	B52X.00	"Malignant neoplasm of meninges, unspecified"	Feb-09
50035	37	3	0	0	B545.00	Malignant neoplasm of aortic body and other paraganglia	Feb-09
50076	14	1	0	0	A789400	HIV disease resulting in multiple infections	Feb-09
50108	11	1	0	0	BB5Uz00	[M]Villous adenoma or adenocarcinoma NOS	Feb-09
50140	16	0	0	0	BB5K.00	[M]Cribriform carcinoma	Feb-09
50152	62	0	0	0	B306500	Malignant sacral teratoma	Feb-09
50157	28	0	0	0	G210.00	Malignant hypertensive heart disease	Feb-09
50222	17	0	0	0	B311000	Malignant neoplasm of connective and soft tissue of shoulder	Feb-09
50251	5	1	0	0	PF55100	Acrocephalosyndactyly (Pfeiffer)	Feb-09
50285	67	0	0	0	B410z00	Malignant neoplasm of endocervix NOS	Feb-09
50289	2	0	0	0	B241z00	Malignant neoplasm of heart NOS	Feb-09
50290	6	0	0	0	B6z0.00	Kaposi's sarcoma of lymph nodes	Feb-09
50292	5	0	0	0	Byu2500	"[X]Malignant neoplasm of mediastinum, part unspecified"	Feb-09
50296	3	0	0	0	B000100	"Malignant neoplasm of upper lip, lipstick area"	Feb-09
50297	55	0	0	0	B411.00	Malignant neoplasm of exocervix	Feb-09
50298	5	0	0	0	B300500	Malignant neoplasm of orbital bone	Feb-09
50299	2	0	0	0	B300900	Malignant neoplasm of zygomatic bone	Feb-09
50379	47	1	0	0	BBN1.00	[M]Synovial sarcoma NOS	Feb-09
50402	7	0	0	0	B307100	Malignant neoplasm of fibula	Feb-09
50444	20	0	0	0	PB61z00	Biliary atresia NOS	Feb-09
50475	9	0	0	0	B02z.00	Malignant neoplasm of major salivary gland NOS	Feb-09
50505	123	0	0	0	B326000	Malignant melanoma of shoulder	Feb-09
50554	143	1	0	0	Q48E.11	Periventricular leukomalacia	Feb-09
50565	1	0	0	0	P100200	Thoracic spina bifida with hydrocephalus	Feb-09

For peer review only - http://bmjopen.bmj.com/site/about/guidelines.xhtml

50579	4	0	0	0	B214.00	"Malignant neoplasm, overlapping lesion of larynx"	Feb-09
50605	3	0	0	0	BBDB.00	[M]Glomangiosarcoma	Feb-09
50668	14	0	0	0	B627300	Diffuse non-Hodgkin's small cell (diffuse) lymphoma	Feb-09
50681	50	6	0	0	B480.00	Malignant neoplasm of prepuce (foreskin)	Feb-09
50695	9	0	0	0	B627500	Diffuse non-Hodgkin mixed sml & lge cell (diffuse) lymphoma	Feb-09
50696	34	0	0	0	B62y100	"Malignant lymphoma NOS of lymph nodes of head, face and neck"	Feb-09
50777	2	0	0	0	B524600	"Malignant neoplasm,overlap lesion periph nerve & auton ns"	Feb-09
50789	60	0	0	0	B103.00	Malignant neoplasm of upper third of oesophagus	Feb-09
50859	6	0	0	0	BBX1.11	[M]Giant cell bone sarcoma	Feb-09
50898	74	0	0	0	B18y300	Malignant neoplasm of omentum	Feb-09
50928	10	0	0	0	BBr2600	[M]Burkitt's cell leukaemia	Feb-09
50946	5	0	0	0	BB9C.00	[M]Medullary carcinoma with amyloid stroma	Feb-09
50974	67	0	0	0	B14z.00	"Malignant neoplasm rectum,rectosigmoid junction and anus NOS"	Feb-09
51115	96	0	0	0	B522.00	Malignant neoplasm of spinal cord	Feb-09
51209	78	7	0	0	B325800	Malignant melanoma of chest wall	Feb-09
51237	5	0	0	0	B303z00	"Malignant neoplasm of rib, sternum and clavicle NOS"	Feb-09
51255	58	0	0	0	B1zz.00	Malignant neoplasm of digestive tract and peritoneum NOS	Feb-09
51285	12	0	0	0	BBj2.00	"[M]Hodgkin's disease, mixed cellularity"	Feb-09
51323	9	0	0	0	R210z00	[D]Sudden infant death syndrome NOS	Feb-09
51352	10	0	0	0	B592.00	Malignant neoplasms of independent (primary) multiple sites	Feb-09

For peer review only - http://bmjopen.bmj.com/site/about/guidelines.xhtml

51353	8	1	0	0	BBE1000	"[M]Malignant melanoma, regressing"	Feb-09
51551	41	2	0	0	B571.00	Secondary malignant neoplasm of mediastinum	Feb-09
51656	31	0	0	0	BB81E00	[M]Mucinous cystadenocarcinoma NOS	Feb-09
51680	8	0	0	0	BBgV.00	"[M]Malignant lymphoma, small cell, noncleaved, diffuse"	Feb-09
51690	1	0	0	0	B117.00	"Malignant neoplasm, overlapping lesion of stomach"	Feb-09
51708	7	0	0	0	A789X00	"HIV dis reslt/oth mal neopl/lymph,h'matopoetc+reltd tissu"	Feb-09
51786	100	1	0	0	B021.00	Malignant neoplasm of submandibular gland	Feb-09
51795	31	2	0	0	B545000	Malignant neoplasm of glomus jugulare	Feb-09
51818	27	0	0	0	B550300	Malignant neoplasm of jaw NOS	Feb-09
51852	1	0	0	0	BBgD.00	"[M]Malig lymphoma, lymphocytic, intermediate different NOS"	Feb-09
51873	196	5	0	0	B327100	Malignant melanoma of thigh	Feb-09
51878	8	0	0	0	BBcC.00	[M]Aesthesioneuroblastoma	Feb-09
51895	73	1	0	0	BBgz.00	"[M]Lymphoma, diffuse or NOS"	Feb-09
51911	30	1	0	0	P74z800	Atresia of pulmonary vein	Feb-09
51921	7	1	0	0	B306200	Malignant neoplasm of pubis	Feb-09
51926	33	0	0	0	B062000	Malignant neoplasm of faucial pillar	Feb-09
51965	26	0	0	0	B315.00	Malignant neoplasm of connective and soft tissue of pelvis	Feb-09
52029	76	0	0	0	ByuC800	[X]Malignant neoplasm without specification of site	Feb-09
52070	20	1	0	0	BBDE.00	[M]Gangliocytic paraganglioma	Feb-09
52130	P204.00	22	0	0	0	Meningoencephalocele	Feb-09
52258	15	0	0	0	PB33.00	Total intestinal aganglionosis	Feb-09
52316	29	0	0	0	B553.00	Malignant neoplasm of pelvis	Feb-09
52326	41	2	0	0	BB5L100	[M]Adenocarcinoma in adenomatous polyp	Feb-09

For peer review only

For peer review only - http://bmjopen.bmj.com/site/about/guidelines.xhtml

52493	20	1	0	0	BBQ7213	"[M]Teratoblastoma, malignant"	Feb-09
52496	15	3	0	0	BB61.00	[M]Sweat gland adenoma and adenocarcinomas	Feb-09
52511	6	0	0	0	B515.00	Malignant neoplasm of cerebral ventricles	Feb-09
52537	32	1	0	0	B161100	Malignant neoplasm of hepatic duct	Feb-09
52570	2	0	0	0	B487.00	"Malignant neoplasm, overlapping lesion of penis"	Feb-09
52594	59	1	0	0	B4z..00	Malignant neoplasm of genitourinary organ NOS	Feb-09
52677	18	0	0	0	A412.00	Subacute sclerosing panencephalitis	Feb-09
52683	P102.13	5	0	0	0	Myelocele with hydrocephalus	Feb-09
52684	14	0	0	0	BBW9.00	[M]Mesenchymal chondrosarcoma	Feb-09
52942	21	1	0	0	BBr6300	[M]Chronic myeloid leukaemia	Feb-09
53103	6	0	0	0	B410100	Malignant neoplasm of endocervical gland	Feb-09
53129	3	0	0	0	BB5W112	[M]Oncytic adenocarcinoma	Feb-09
53317	57	2	0	0	F383.00	Congenital and developmental myasthenia	Feb-09
53369	14	1	0	0	B327900	Malignant melanoma of great toe	Feb-09
53382	36	0	0	0	F100200	Pelizaeus-Merzbacher disease	Feb-09
53397	197	6	0	0	B61z.00	Hodgkin's disease NOS	Feb-09
53515	114	5	0	0	B332.00	Malignant neoplasm skin of ear and external auricular canal	Feb-09
53517	6	0	0	0	PB33.12	Congenital aganglionic megacolon	Feb-09
53528	8	0	0	0	B581200	Secondary malignant neoplasm of urethra	Feb-09
53551	8	0	0	0	B627600	Diffuse non-Hodgkin's immunoblastic (diffuse) lymphoma	Feb-09
53591	51	0	0	0	B10y.00	Malignant neoplasm of other specified part of oesophagus	Feb-09
53594	7	0	0	0	B300000	Malignant neoplasm of ethmoid bone	Feb-09
53599	8	0	0	0	B300100	Malignant neoplasm of frontal bone	Feb-09
53629	10	0	0	0	B325200	Malignant melanoma of buttock	Feb-09

For peer review only - http://bmjopen.bmj.com/site/about/guidelines.xhtml

53852	165	1	0	0	K05..12	End stage renal failure	Feb-09
53884	131	0	0	0	B060z00	Malignant neoplasm tonsil NOS	Feb-09
53910	21	0	0	0	B453.00	Malignant neoplasm of clitoris	Feb-09
53929	P113000	6	0	0	0	Spinal meningocele of unspecified site	Feb-09
53933	11	0	0	0	PG51z00	Osteogenesis imperfecta NOS	Feb-09
53987	9	0	0	0	BB5Dz00	[M]Hepatobiliary adenoma or carcinoma NOS	Feb-09
54103	85	0	0	0	B16..00	Malignant neoplasm gallbladder and extrahepatic bile ducts	Feb-09
54106	44	2	0	0	Eu02100	[X]Dementia in Creutzfeldt-Jakob disease	Feb-09
54120	5	0	0	0	B584.00	Secondary malignant neoplasm of other part of nervous system	Feb-09
54133	43	0	0	0	B510z00	Malignant neoplasm of cerebrum NOS	Feb-09
54134	45	0	0	0	B223z00	"Malignant neoplasm of middle lobe, bronchus or lung NOS"	Feb-09
54171	74	2	0	0	B104.00	Malignant neoplasm of middle third of oesophagus	Feb-09
54184	98	1	0	0	B4A1z00	Malignant neoplasm of renal pelvis NOS	Feb-09
54186	5	0	0	0	B313100	Malignant neoplasm of diaphragm	Feb-09
54202	6	0	0	0	B35z.00	Malignant neoplasm of other site of male breast	Feb-09
54222	13	0	0	0	B312400	Malignant neoplasm of connective and soft tissue of foot	Feb-09
54234	39	1	0	0	B334.00	Malignant neoplasm of scalp and skin of neck	Feb-09
54253	250	0	0	0	ByuC700	[X]Secondary malignant neoplasm of other specified sites	Feb-09
54267	90	4	0	0	B59z.00	Malignant neoplasm of unspecified site NOS	Feb-09
54276	4	0	0	0	BB1E.00	[M]Pseudosarcomatous carcinoma	Feb-09
54305	64	0	0	0	B327200	Malignant melanoma of knee	Feb-09
54352	90	2	0	0	B336300	Malignant neoplasm of skin of hand	Feb-09

For peer review only - http://bmjopen.bmj.com/site/about/guidelines.xhtml

54493	7	0	0	0	B303500	Malignant neoplasm of xiphoid process	Feb-09
54494	5	0	0	0	B350.00	Malignant neoplasm of nipple and areola of male breast	Feb-09
54585	52	0	0	0	BBr6100	[M]Acute myeloid leukaemia	Feb-09
54613	3	0	0	0	B201200	Malignant neoplasm of tympanic antrum	Feb-09
54627	6	0	0	0	BBR3.00	[M]Choriocarcinoma combined with teratoma	Feb-09
54631	39	1	0	0	B306.00	"Malignant neoplasm of pelvic bones, sacrum and coccyx"	Feb-09
54632	28	0	0	0	B321.00	Malignant melanoma of eyelid including canthus	Feb-09
54636	53	2	0	0	B203.00	Malignant neoplasm of ethmoid sinus	Feb-09
54679	89	1	0	0	B594.00	Secondary malignant neoplasm of unknown site	Feb-09
54685	173	0	0	0	B326100	Malignant melanoma of upper arm	Feb-09
54691	62	0	0	0	B302200	Malignant neoplasm of lumbar vertebra	Feb-09
54714	P00..11	2	0	0	0	Congenital absence of brain	Feb-09
54747	4	0	0	0	B300600	Malignant neoplasm of parietal bone	Feb-09
54749	2	0	0	0	BB81H00	[M]Papillary mucinous cystadenocarcinoma	Feb-09
54793	3	0	0	0	B682.00	Subacute leukaemia NOS	Feb-09
54874	3	0	0	0	BB85100	[M]Metastatic signet ring cell carcinoma	Feb-09
54934	51	3	0	0	F030400	Encephalitis due to herpes simplex virus	Feb-09
54956	26	0	0	0	B50z.00	Malignant neoplasm of eye NOS	Feb-09
55015	130	0	0	0	B05z.00	Malignant neoplasm of mouth NOS	Feb-09
55019	21	1	0	0	B11y.00	Malignant neoplasm of other specified site of stomach	Feb-09
55066	19	0	0	0	B062.00	Malignant neoplasm of tonsillar pillar	Feb-09
55090	20	0	0	0	B58y100	Secondary malignant neoplasm of uterus	Feb-09
55096	33	1	0	0	B582z00	Secondary malignant neoplasm of skin NOS	Feb-09
55098	71	1	0	0	B550000	Malignant neoplasm of head NOS	Feb-09

For peer review only - http://bmjopen.bmj.com/site/about/guidelines.xhtml

55101	16	0	0	0	B553z00	Malignant neoplasm of pelvis NOS	Feb-09
55116	46	2	0	0	BBFz.00	[M]Soft tissue tumour or sarcoma NOS	Feb-09
55246	6	0	0	0	B20z.00	Malignant neoplasm of accessory sinus NOS	Feb-09
55268	30	2	0	0	BBK2100	[M]Myosarcoma	Feb-09
55292	10	0	0	0	B326z00	Malignant melanoma of upper limb or shoulder NOS	Feb-09
55303	23	0	0	0	B614100	"Hodgkin's nodular sclerosis of head, face and neck"	Feb-09
55374	81	0	0	0	B215.00	Malignant neoplasm of epiglottis NOS	Feb-09
55434	42	0	0	0	B116.00	Malignant neoplasm of greater curve of stomach unspecified	Feb-09
55468	7	0	0	0	BB5R600	"[M]Mucocarcinoid tumour, malignant"	Feb-09
55550	34	2	0	0	B331100	Malignant neoplasm of upper eyelid	Feb-09
55588	14	0	0	0	Byu7300	"[X]Malignant neoplasm of female genital organ, unspecified"	Feb-09
55595	7	0	0	0	B300700	Malignant neoplasm of sphenoid bone	Feb-09
55630	8	0	0	0	B07y.00	Malignant neoplasm of other specified site of nasopharynx	Feb-09
55634	11	0	0	0	Q442.00	Neonatal myasthenia gravis	Feb-09
55636	16	0	0	0	F130100	Hallervorden-Spatz disease	Feb-09
55670	61	1	0	0	B333200	Malignant neoplasm of skin of eyebrow	Feb-09
55850	G558.00	7	0	0	0	Cardiomyopathy in disease EC	Feb-09
55881	88	0	0	0	B324000	Malignant melanoma of scalp	Feb-09
55946	7	0	0	0	B574000	Secondary malignant neoplasm of duodenum	Feb-09
55947	34	4	0	0	BBJ7.00	[M]Pleomorphic liposarcoma	Feb-09
55953	5	0	0	0	B300400	Malignant neoplasm of occipital bone	Feb-09
56041	10	0	0	0	BBj1.00	"[M]Hodgkin's disease, lymphocytic predominance"	Feb-09
56121	94	0	0	0	Byu4300	"[X]Malignant neoplasm of skin, unspecified"	Feb-09

For peer review only - http://bmjopen.bmj.com/site/about/guidelines.xhtml

56288	F10..00	304	0	1	0	Cerebral degenerations usually manifest in childhood	Feb-09
56345	49	0	0	0	B57y.00	Secondary malignant neoplasm of other digestive organ	Feb-09
56355	8	1	0	0	B066.00	Malignant neoplasm of lateral wall of oropharynx	Feb-09
56362	P105.00	8	0	0	0	Spina bifida with stenosis of aqueduct of Sylvius	Feb-09
56490	14	0	0	0	B52z.00	Malignant neoplasm of nervous system NOS	Feb-09
56513	111	2	0	0	B307000	Malignant neoplasm of femur	Feb-09
56540	9	2	0	0	D40y400	Leukaemoid reaction	Feb-09
56600	108	0	0	0	BB2A.11	[M]Epidermoid carcinoma NOS	Feb-09
56676	167	9	0	0	BBJ5.00	[M]Myxoid liposarcoma	Feb-09
56709	5	0	0	0	B04y.00	Malignant neoplasm of other sites of floor of mouth	Feb-09
56715	77	1	0	0	B34y.00	Malignant neoplasm of other site of female breast	Feb-09
56718	15	0	0	0	B500z00	Malignant neoplasm of eyeball NOS	Feb-09
56918	67	0	0	0	B1zy.00	Malignant neoplasm other spec digestive tract and peritoneum	Feb-09
56925	36	0	0	0	Byu4000	[X]Malignant melanoma of other+unspecified parts of face	Feb-09
56951	24	0	0	0	F101200	Spielmeyer-Vogt (Batten) disease	Feb-09
56954	13	1	0	0	B337200	Malignant neoplasm of skin of knee	Feb-09
57047	12	0	0	0	B544.00	Malignant neoplasm of carotid body	Feb-09
57084	6	0	0	0	BBQ1000	"[M]Seminoma, anaplastic type"	Feb-09
57113	8	0	0	0	P100300	Lumbar spina bifida with hydrocephalus	Feb-09
57139	39	1	0	0	C30y800	Glutaric aciduria Type 1	Feb-09
57184	4	0	0	0	Byu4200	[X]Oth malignant neoplasm/skin of oth+unspecfd parts of face	Feb-09

For peer review only - http://bmjopen.bmj.com/site/about/guidelines.xhtml

57191	4	2	0	0	Byu8000	[X]Malignant neoplasm/other specified male genital organs	Feb-09
57225	7	0	0	0	B614000	"Hodgkin's disease, nodular sclerosis of unspecified site"	Feb-09
57235	25	0	0	0	B410000	Malignant neoplasm of endocervical canal	Feb-09
57243	2	1	0	0	P102200	Thoracic spina bifida with hydrocephalus - open	Feb-09
57248	9	0	0	0	B082.00	"Malignant neoplasm aryepiglottic fold, hypopharyngeal aspect"	Feb-09
57260	37	1	0	0	B322.00	Malignant melanoma of ear and external auricular canal	Feb-09
57316	34	0	0	0	BBr6600	[M]Acute promyelocytic leukaemia	Feb-09
57336	51	2	0	0	BB16.00	"[M]Epithelioma, malignant"	Feb-09
57422	79	1	0	0	BB5Vz00	[M]Pituitary adenoma or carcinoma NOS	Feb-09
57427	30	1	0	0	B62y000	Malignant lymphoma NOS of unspecified site	Feb-09
57442	534	1	0	0	B337.00	Malignant neoplasm of skin of lower limb and hip	Feb-09
57446	380	1	0	0	B335.00	"Malignant neoplasm of skin of trunk, excluding scrotum"	Feb-09
57481	41	0	0	0	ByuC300	[X]Secondary malignant neoplasm/oth+unspc respiratory organs	Feb-09
57482	30	1	0	0	B311200	Malignant neoplasm of connective and soft tissue of fore-arm	Feb-09
57505	15	0	0	0	BBK3200	[M]Pleomorphic rhabdomyosarcoma	Feb-09
57513	6	0	0	0	BB2C.11	"[M]Epidermoid carcinoma, keratinising type"	Feb-09
57544	6	0	0	0	BBm4.00	[M]True histiocytic lymphoma	Feb-09
57551	2	0	0	0	F381100	Myasthenic syndrome due to other malignancy	Feb-09
57611	P114z00	14	0	0	0	Meningomyelocele NOS	Feb-09

For peer review only - http://bmjopen.bmj.com/site/about/guidelines.xhtml

57632	21	0	0	0	F151z00	Spinal muscular atrophy NOS	Feb-09
57671	7	0	0	0	B672.00	Megakaryocytic leukaemia	Feb-09
57677	36	1	0	0	BBL8.00	[M]Hepatoblastoma	Feb-09
57680	2	0	0	0	BB2A.12	[M]Spinous cell carcinoma	Feb-09
57713	3	0	0	0	BBr8.00	[M]Eosinophilic leukaemias	Feb-09
57719	4	0	0	0	B41y100	Malignant neoplasm of squamocolumnar junction of cervix	Feb-09
57729	6	0	0	0	BBU1.00	[M]Lymphangiosarcoma	Feb-09
57737	5	0	0	0	B62x100	Lymphoepithelioid lymphoma	Feb-09
57756	10	0	0	0	Byu7100	[X]Malignant neoplasm/other specified female genital organs	Feb-09
57796	5	1	0	0	BBN4.00	"[M]Synovial sarcoma, biphasic type"	Feb-09
57802	16	1	0	0	BB5S400	[M]Alveolar adenocarcinoma	Feb-09
57854	6	0	0	0	B553000	Malignant neoplasm of inguinal region NOS	Feb-09
57988	2	0	0	0	B305000	Malignant neoplasm of carpal bone - scaphoid	Feb-09
58015	10	0	0	0	BBgQ.00	[M]Malignant lymphomatous polyposis	Feb-09
58061	15	4	0	0	B452.00	Malignant neoplasm of labia minora	Feb-09
58082	5	0	0	0	B620800	Nodular lymphoma of lymph nodes of multiple sites	Feb-09
58088	11	0	0	0	B151400	Malignant neoplasm of intrahepatic gall duct	Feb-09
58094	21	6	0	0	B412.00	"Malignant neoplasm, overlapping lesion of cervix uteri"	Feb-09
58121	6	0	0	0	B014.00	Malignant neoplasm of anterior 2/3 of tongue unspecified	Feb-09
58131	13	1	0	0	BB93.00	[M]Comedocarcinoma NOS	Feb-09
58601	51	2	0	0	B337100	Malignant neoplasm of skin of thigh	Feb-09
58635	29	0	0	0	PG51300	Osteogenesis imperfecta type I	Feb-09
58671	1	0	0	0	K0A5300	Heredtry nephrpthy NEC difus mesangial prolif glomnephrit	Feb-09

For peer review only - http://bmjopen.bmj.com/site/about/guidelines.xhtml

58684	2	0	0	0	B615200	Hodgkin's mixed cellularity of intrathoracic lymph nodes	Feb-09
58798	3	0	0	0	BB47.00	"[M]Transitional cell carcinoma, spindle cell type"	Feb-09
58835	28	1	0	0	BBE1100	"[M]Desmoplastic melanoma, malignant"	Feb-09
58837	14	0	0	0	BBF5.00	[M]Small cell sarcoma	Feb-09
58871	9	0	0	0	B623z00	Malignant histiocytosis NOS	Feb-09
58903	32	0	0	0	B550z00	"Malignant neoplasm of head, neck and face NOS"	Feb-09
58938	G55y000	29	0	0	0	Cardiomyopathy due to drugs and other external agents	Feb-09
58949	8	0	0	0	B308D00	Malignant neoplasm of phalanges of foot	Feb-09
58958	23	0	0	0	B323500	Malignant melanoma of temple	Feb-09
58962	4	0	0	0	B62x500	Malignant immunoproliferative small intestinal disease	Feb-09
58973	39	1	0	0	Byu0.00	"[X]Malignant neoplasm of lip, oral cavity and pharynx"	Feb-09
59001	49	0	0	0	C310112	Pompe's disease	Feb-09
59004	4	0	0	0	B072.00	Malignant neoplasm of lateral wall of nasopharynx	Feb-09
59035	9	0	0	0	F100z00	Leucodystrophy NOS	Feb-09
59036	24	0	0	0	B300.00	Malignant neoplasm of bones of skull and face	Feb-09
59041	33	0	0	0	B500000	Malignant neoplasm of ciliary body	Feb-09
59061	30	0	0	0	B322000	Malignant melanoma of auricle (ear)	Feb-09
59092	24	0	0	0	B111z00	Malignant neoplasm of pylorus of stomach NOS	Feb-09
59097	4	0	0	0	B431000	Malignant neoplasm of lower uterine segment	Feb-09
59115	4	0	0	0	B602100	"Burkitt's lymphoma of lymph nodes of head, face and neck"	Feb-09

For peer review only - http://bmjopen.bmj.com/site/about/guidelines.xhtml

59143	6	0	0	0	BB2D.00	"[M]Squamous cell carcinoma, large cell, non-keratinising"	Feb-09
59152	8	0	0	0	B315200	Malignant neoplasm of connective and soft tissue of perineum	Feb-09
59155	6	0	0	0	Fy04.11	Ondine's curse	Feb-09
59170	18	0	0	0	B51y000	Malignant neoplasm of corpus callosum	Feb-09
59218	18	2	0	0	P10y000	Dandy - Walker syndrome with spina bifida	Feb-09
59223	7	1	0	0	B306100	Malignant neoplasm of ischium	Feb-09
59240	19	0	0	0	BB58.00	"[M]Carcinoma, diffuse type"	Feb-09
59251	17	0	0	0	BBM9.00	"[M]Cystosarcoma phyllodes, malignant"	Feb-09
59284	12	0	0	0	BB82114	[M]Mucous adenocarcinoma	Feb-09
59286	6	0	0	0	B4Ay000	Malignant neoplasm of overlapping lesion of urinary organs	Feb-09
59310	16	1	0	0	BBV1.12	[M]Osteochondrosarcoma	Feb-09
59343	11	4	0	0	C302512	Oculocerebrorenal dystrophy	Feb-09
59362	13	0	0	0	B451z00	Malignant neoplasm of labia majora NOS	Feb-09
59375	27	0	0	0	B583z00	Secondary malignant neoplasm of brain or spinal cord NOS	Feb-09
59381	25	1	0	0	B500100	Malignant neoplasm of iris	Feb-09
59382	76	11	0	0	B310000	Malignant neoplasm of soft tissue of head	Feb-09
59383	11	0	0	0	G240000	Secondary malignant renovascular hypertension	Feb-09
59388	1	0	0	0	B18y100	Malignant neoplasm of mesocaecum	Feb-09
59415	27	0	0	0	BBB6100	"[M]Thymoma, malignant"	Feb-09
59520	2	0	0	0	B300200	Malignant neoplasm of malar bone	Feb-09
59651	4	0	0	0	BBJ8.00	[M]Mixed type liposarcoma	Feb-09
59755	18	0	0	0	B61z200	Hodgkin's disease NOS of intrathoracic lymph nodes	Feb-09
59778	28	0	0	0	B61z100	"Hodgkin's disease NOS of lymph nodes of head, face and neck"	Feb-09

For peer review only - http://bmjopen.bmj.com/site/about/guidelines.xhtml

59823	1	0	0	0	B542.00	Malignant neoplasm pituitary gland and craniopharyngeal duct	Feb-09
59831	25	0	0	0	B340z00	Malignant neoplasm of nipple or areola of female breast NOS	Feb-09
59855	44	0	0	0	F100300	Metachromatic leucodystrophy	Feb-09
59918	5	0	0	0	BB5f200	"[M]Follicular adenocarcinoma, well differentiated type"	Feb-09
59929	3	0	0	0	BBr0z00	"[M]Leukaemia unspecified, NOS"	Feb-09
59956	F10z.00	10	0	0	0	Childhood cerebral degenerations NOS	Feb-09
60035	23	3	0	0	B310300	Malignant neoplasm of cartilage of ear	Feb-09
60045	30	0	0	0	BB5M100	[M]Tubular adenocarcinoma	Feb-09
60052	114	2	0	0	B55yz00	Malignant neoplasm of specified site NOS	Feb-09
60079	8	0	0	0	PJ63z11	Bonnevie-Ullrich syndrome NOS	Feb-09
60092	16	0	0	0	B62y700	Malignant lymphoma NOS of spleen	Feb-09
60127	6	0	0	0	BBJ5.12	[M]Myxoliposarcoma	Feb-09
60134	2	0	0	0	B581000	Secondary malignant neoplasm of ureter	Feb-09
60162	20	0	0	0	Byu5A00	[X]Malignant neoplasm overlapping lesion of skin	Feb-09
60165	4	1	0	0	PFy4.00	Other arthrogryposis syndromes	Feb-09
60242	1	0	0	0	B600000	Reticulosarcoma of unspecified site	Feb-09
60275	8	0	0	0	BBgJ.00	"[M]Malignant lymphoma, centroblastic type NOS"	Feb-09
60312	10	0	0	0	B16y.00	Malignant neoplasm other gallbladder/extrahepatic bile duct	Feb-09
60335	21	1	0	0	B58y400	Secondary malignant neoplasm of vulva	Feb-09
60347	8	0	0	0	BBd2.11	[M]Leptomeningeal sarcoma	Feb-09
60403	8	0	0	0	B303300	Malignant neoplasm of costal cartilage	Feb-09
60504	2	0	0	0	BBgC.12	[M]Lymphocytic lymphosarcoma NOS	Feb-09
60526	12	0	0	0	B336z00	Malignant neoplasm of skin of upper limb or shoulder NOS	Feb-09
60623	3	0	0	0	P102400	Sacral spina bifida with hydrocephalus - open	Feb-09

For peer review only - http://bmjopen.bmj.com/site/about/guidelines.xhtml

60631	13	0	0	0	BBV5.00	[M]Osteosarcoma in Paget's disease of bone	Feb-09
60673	2	0	0	0	PKyz000	"Ullrich - Feichtiger syndrome, chimaera"	Feb-09
60772	37	0	0	0	B450z00	Malignant neoplasm of vagina NOS	Feb-09
60775	11	1	0	0	BB5h100	[M]Adrenal cortical carcinoma	Feb-09
60918	1	0	25	0	4M20.00	Lymphoma stage I	Feb-09
61064	28	2	0	0	B24X.00	"Malignant neoplasm of mediastinum, part unspecified"	Feb-09
61100	10	0	0	0	P51y.11	Transposition of aorta	Feb-09
61149	3	0	0	0	B614300	Hodgkin's nodular sclerosis of intra-abdominal lymph nodes	Feb-09
61194	14	0	0	0	B337z00	Malignant neoplasm of skin of lower limb or hip NOS	Feb-09
61246	14	0	0	0	B327600	Malignant melanoma of heel	Feb-09
61251	2	0	0	0	BBgN.00	"[M]Malign lymphoma,lymphocytic,intermediate differn, diffuse"	Feb-09
61390	10	0	0	0	B540000	Malignant neoplasm of adrenal cortex	Feb-09
61399	29	0	0	0	B510100	Malignant neoplasm of cerebral cortex	Feb-09
61467	1	0	0	0	BB5f300	"[M]Follicular adenocarcinoma, trabecular type"	Feb-09
61500	9	0	0	0	B690.00	Acute myelomonocytic leukaemia	Feb-09
61510	2	1	0	0	B062200	Malignant neoplasm of palatoglossal arch	Feb-09
61542	31	0	0	0	BBQ7400	"[M]Malignant teratoma, undifferentiated type"	Feb-09
61555	11	0	0	0	B180z00	Malignant neoplasm of retroperitoneum NOS	Feb-09
61588	8	0	0	0	BB85000	[M]Signet ring cell carcinoma	Feb-09
61643	24	0	0	0	B151z00	Malignant neoplasm of intrahepatic bile ducts NOS	Feb-09
61650	2	1	0	0	C375200	"Mucopolysaccharidosis, type II"	Feb-09

For peer review only - http://bmjopen.bmj.com/site/about/guidelines.xhtml

61655	1	0	0	0	ZV10211	[V]Personal history of malignant neoplasm - accessory sinus	Feb-09
61662	12	0	0	0	B61z000	"Hodgkin's disease NOS, unspecified site"	Feb-09
61663	8	2	0	0	ZV76500	[V]Screening for malignant neoplasm of oral cavity	Feb-09
61692	2	0	0	0	B004.00	"Malignant neoplasm of lip unspecified, inner aspect"	Feb-09
61693	16	0	0	0	ByuD600	[X]Other myeloid leukaemia	Feb-09
61695	22	1	0	0	B100.00	Malignant neoplasm of cervical oesophagus	Feb-09
61716	4	0	0	0	B524100	"Malignant neoplasm of peripheral nerve,upp limb,incl should"	Feb-09
61741	50	1	0	0	B304200	Malignant neoplasm of humerus	Feb-09
61769	3	0	0	0	PKyC.00	Pena-Shokeir syndrome type I	Feb-09
61783	22	0	0	0	BBbG.11	[M]Juvenile astrocytoma	Feb-09
61930	314	6	0	0	Kyu2.00	[X]Renal failure	Feb-09
61984	9	0	0	0	BB1G.00	[M]Spheroidal cell carcinoma	Feb-09
61997	26	0	0	0	BBj0.00	[M]Hodgkin's disease NOS	Feb-09
62080	10	2	0	0	B332100	Malignant neoplasm of skin of external auditory meatus	Feb-09
62088	44	2	0	0	BBEG.00	[M]Malignant melanoma in Hutchinson's melanotic freckle	Feb-09
62104	9	0	0	0	B300800	Malignant neoplasm of temporal bone	Feb-09
62126	19	0	0	0	B510500	Malignant neoplasm of thalamus	Feb-09
62169	8	0	0	0	P51y.00	Other specified transposition of great vessels	Feb-09
62182	16	0	0	0	B200300	Malignant neoplasm of vestibule of nose	Feb-09
62199	8	0	0	0	BB5W.00	[M]Oxyphilic adenomas and adenocarcinomas	Feb-09
62305	6	0	0	0	B335800	Malignant neoplasm of skin of buttock	Feb-09
62330	2	0	0	0	BBr6z00	[M]Other myeloid leukaemia NOS	Feb-09
62348	10	1	0	0	BBT1.00	[M]Haemangiosarcoma	Feb-09

For peer review only - http://bmjopen.bmj.com/site/about/guidelines.xhtml

62376	P722500	11	0	0	0	Atresia of aorta	Feb-09
62380	2	0	0	0	B601200	Lymphosarcoma of intrathoracic lymph nodes	Feb-09
62395	44	0	0	0	C306100	Citrullinaemia	Feb-09
62396	27	0	0	0	BBF6.00	[M]Epithelioid cell sarcoma	Feb-09
62437	7	0	0	0	B62x400	Malignant reticulosis	Feb-09
62475	17	0	0	0	B326300	Malignant melanoma of hand	Feb-09
62556	13	0	0	0	B24..00	"Malignant neoplasm of thymus, heart and mediastinum"	Feb-09
62567	4	0	0	0	BBc0200	[M]Ganglioneuromatosis	Feb-09
62584	32	0	0	0	B573.00	Secondary malignant neoplasm of other respiratory organs	Feb-09
62630	2	0	0	0	B307z00	Malignant neoplasm of long bones of leg NOS	Feb-09
62644	14	2	0	0	C372011	Lesch - Nyhan syndrome	Feb-09
62761	22	0	0	0	B200200	Malignant neoplasm of septum of nose	Feb-09
62762	5	0	0	0	C375600	"Mucopolysaccharidosis, type VI"	Feb-09
62828	10	0	0	0	B581z00	Secondary malignant neoplasm of other urinary organ NOS	Feb-09
62840	18	0	0	0	B013.00	Malignant neoplasm of ventral surface of tongue	Feb-09
62871	2	0	0	0	BB92.00	"[M]Comedocarcinoma, noninfiltrating"	Feb-09
62909	26	1	0	0	B575100	Secondary malignant neoplasm of rectum	Feb-09
62941	10	1	0	0	BBe2.00	[M]Neurofibrosarcoma	Feb-09
62951	3	0	28	0	5A15.00	Bone tumour/metast.irradiat.	Feb-09
63054	47	0	0	0	B614z00	"Hodgkin's disease, nodular sclerosis NOS"	Feb-09
63102	6	0	0	0	BB5B100	[M]Islet cell carcinoma	Feb-09
63104	9	0	0	0	B501z00	Malignant neoplasm of orbit NOS	Feb-09
63105	10	0	0	0	B62y500	Malignant lymphoma NOS of lymph node inguinal region and leg	Feb-09

For peer review only - http://bmjopen.bmj.com/site/about/guidelines.xhtml

63224	10	0	0	0	B48z.00	Malignant neoplasm of penis and other male genital organ NOS	Feb-09
63239	7	0	0	0	BBm1.00	[M]Malignant histiocytosis	Feb-09
63247	3	0	0	0	BBK3611	[M]Sarcoma botryoides	Feb-09
63286	3	0	0	0	BBN5.00	[M]Clear cell sarcoma of tendons and aponeuroses	Feb-09
63300	1	0	0	0	Byu3200	[X]Malignant neoplasm/overlap lesion/bone+articulr cartilage	Feb-09
63331	5	0	0	0	B485.00	Malignant neoplasm of spermatic cord	Feb-09
63359	7	0	0	0	C315000	Pyruvate dehydrogenase deficiency	Feb-09
63375	47	0	0	0	ByuDE00	[X]Unspecified B-cell non-Hodgkin's lymphoma	Feb-09
63376	15	0	0	0	F402100	Malignant myopia	Feb-09
63390	65	0	0	0	P512.00	Corrected great vessel transposition	Feb-09
63430	7	0	0	0	B241000	Malignant neoplasm of endocardium	Feb-09
63460	6	0	0	0	B213000	Malignant neoplasm of arytenoid cartilage	Feb-09
63470	34	0	0	0	B102.00	Malignant neoplasm of abdominal oesophagus	Feb-09
63475	4	0	0	0	B652.00	Subacute myeloid leukaemia	Feb-09
63518	11	0	0	0	BBLE.00	[M]Adenosarcoma	Feb-09
63568	7	0	0	0	B524000	"Malignant neoplasm of peripheral nerves of head, face & neck"	Feb-09
63570	3	0	0	0	BBr0113	[M]Stem cell leukaemia	Feb-09
63571	18	0	0	0	BBV..12	[M]Parosteal osteosarcoma	Feb-09
63574	11	0	0	0	BBEC.00	[M]Malignant melanoma in junctional naevus	Feb-09
63598	26	0	0	0	ByuE.00	[X]Malignant neoplasms/independent (primary) multiple sites	Feb-09
63625	0	1	0	0	B616400	Hodgkin's lymphocytic depletion lymph nodes axilla and arm	Feb-09
63634	5	1	0	0	PD00000	Bilateral renal agenesis	Feb-09

For peer review only - http://bmjopen.bmj.com/site/about/guidelines.xhtml

63652	5	0	0	0	F103000	Cerebral degeneration in Hunter's disease	Feb-09
63657	37	1	0	0	B503.00	Malignant neoplasm of conjunctiva	Feb-09
63659	1	0	0	0	BBW6.00	[M]Juxtacortical chondrosarcoma	Feb-09
63695	3	0	0	0	B524300	Malignant neoplasm of peripheral nerve of thorax	Feb-09
63699	15	1	0	0	BBk0.00	"[M]Malignant lymphoma, nodular NOS"	Feb-09
63723	12	0	0	0	B601z00	Lymphosarcoma NOS	Feb-09
63786	24	0	0	0	K01w.00	Congenital nephrotic syndrome	Feb-09
63896	5	0	0	0	B582400	Secondary malignant neoplasm of skin of shoulder and arm	Feb-09
63925	19	0	0	0	ByuA200	"[X]Malignant neoplasm of meninges, unspecified"	Feb-09
63973	1	0	0	0	BBm0.00	[M]Microglioma	Feb-09
63979	7	0	0	0	B013100	Malignant neoplasm of frenulum linguae	Feb-09
63988	12	0	0	0	B311500	Malignant neoplasm of connective and soft tissue of thumb	Feb-09
63994	1	0	0	0	BBgS.00	"[M]Malignant lymphoma, large cell, cleaved, diffuse"	Feb-09
63995	3	0	0	0	B123.00	Malignant neoplasm of Meckel's diverticulum	Feb-09
63997	15	0	0	0	B326500	Malignant melanoma of thumb	Feb-09
64036	3	0	0	0	B612.00	Hodgkin's sarcoma	Feb-09
64106	46	0	0	0	B18yz00	Malignant neoplasm of specified parts of peritoneum NOS	Feb-09
64270	14	0	0	0	B337500	Malignant neoplasm of skin of ankle	Feb-09
64309	13	0	0	0	ByuB100	"[X]Malignant neoplasm of endocrine gland, unspecified"	Feb-09
64327	32	0	0	0	B327z00	Malignant melanoma of lower limb or hip NOS	Feb-09
64336	19	0	0	0	ByuD300	[X]Other specified types of non-Hodgkin's lymphoma	Feb-09

For peer review only - http://bmjopen.bmj.com/site/about/guidelines.xhtml

64343	10	0	0	0	BBj6100	"[M]Hodgkin,s disease, nodular sclerosis, mixed cellularity"	Feb-09
64345	22	0	0	0	B311100	"Malignant neoplasm of connective and soft tissue, upper arm"	Feb-09
64406	5	0	0	0	B336500	Malignant neoplasm of skin of thumb	Feb-09
64462	17	0	0	0	B083.00	Malignant neoplasm of posterior pharynx	Feb-09
64470	11	0	0	0	7904y00	Other correction of transposition of great vessels OS	Feb-09
64497	8	0	0	0	Byu7000	"[X]Malignant neoplasm of uterine adnexa, unspecified"	Feb-09
64515	4	0	0	0	ByuDC00	"[X]Diffuse non-Hodgkin's lymphoma, unspecified"	Feb-09
64516	9	0	0	0	B18y400	Malignant neoplasm of parietal peritoneum	Feb-09
64557	4	1	0	0	B517000	Malignant neoplasm of cerebral peduncle	Feb-09
64596	14	0	0	0	BBK0700	[M]Myxoid leiomyosarcoma	Feb-09
64602	17	7	0	0	B470.00	Malignant neoplasm of undescended testis	Feb-09
64618	4	0	0	0	BBr3.00	[M]Plasma cell leukaemias	Feb-09
64625	5	0	0	0	D200111	Fanconi's hypoplastic anaemia	Feb-09
64670	1	0	0	0	B601300	Lymphosarcoma of intra-abdominal lymph nodes	Feb-09
64680	7	0	0	0	B574.00	Secondary malignant neoplasm of small intestine and duodenum	Feb-09
64686	29	0	0	0	B340100	Malignant neoplasm of areola of female breast	Feb-09
64690	34	1	0	0	F390.00	Congenital hereditary muscular dystrophy	Feb-09
64717	24	0	0	0	P10z.00	Spina bifida with hydrocephalus NOS	Feb-09
64796	16	1	0	0	BB82z00	[M]Mucinous adenoma or adenocarcinoma NOS	Feb-09
64810	5	0	0	0	B551z00	Malignant neoplasm of thorax NOS	Feb-09
64817	7	0	0	0	B502.00	Malignant neoplasm of lacrimal gland	Feb-09

For peer review only - http://bmjopen.bmj.com/site/about/guidelines.xhtml

64837	3	2	0	0	G558200	Dystrophic cardiomyopathy	Feb-09
64848	4	0	0	0	B304400	Malignant neoplasm of ulna	Feb-09
64874	22	1	0	0	BBa4.00	[M]Melanotic neuroectodermal tumour	Feb-09
64897	19	0	0	0	ByuE000	[X]Malignant neoplasms/independent(primary)multiple sites	Feb-09
64963	11	0	0	0	BBr0112	[M]Blastic leukaemia	Feb-09
64971	2	0	0	0	B520000	Malignant neoplasm of olfactory bulb	Feb-09
65047	12	0	0	0	BBDA.00	"[M]Phaeochromocytoma, malignant"	Feb-09
65051	4	0	0	0	BB81500	"[M]Papillary cystadenocarcinoma, NOS"	Feb-09
65091	10	0	0	0	PJ33211	18q- syndrome	Feb-09
65106	26	0	0	0	B44z.00	Malignant neoplasm of uterine adnexa NOS	Feb-09
65117	9	0	0	0	A789900	HIV disease resulting in lymphoid interstitial pneumonitis	Feb-09
65121	8	0	0	0	PHz..11	Congenital ectodermal defect	Feb-09
65122	4	0	0	0	B624000	Leukaemic reticuloendotheliosis of unspecified sites	Feb-09
65123	1	0	0	0	B624300	Leukaemic reticuloend of intra-abdominal lymph nodes	Feb-09
65124	5	0	0	0	B151000	Malignant neoplasm of interlobular bile ducts	Feb-09
65159	4	0	0	0	B180100	Malignant neoplasm of perinephric tissue	Feb-09
65164	169	2	0	0	B326.00	Malignant melanoma of upper limb and shoulder	Feb-09
65165	1	0	0	0	ByuD900	[X]Other leukaemia of unspecified cell type	Feb-09
65180	14	0	0	0	B627800	Diffuse non-Hodgkin's lymphoma undifferentiated (diffuse)	Feb-09
65207	6	0	0	0	BB80z00	[M]Cystadenoma or carcinoma NOS	Feb-09
65215	17	0	0	0	B205.00	Malignant neoplasm of sphenoidal sinus	Feb-09
65216	11	0	0	0	BB49.00	[M]Cloacogenic carcinoma	Feb-09

For peer review only - http://bmjopen.bmj.com/site/about/guidelines.xhtml

65241	4	2	0	0	B51y200	"Malignant neoplasm, overlapping lesion of brain"	Feb-09
65246	P118z00	1	0	0	0	Spina bifida without hydrocephalus - closed NOS	Feb-09
65253	3	1	0	0	B560300	Secondary and unspec malignant neoplasm occipital lymph node	Feb-09
65312	6	0	0	0	B11y000	Malignant neoplasm of anterior wall of stomach NEC	Feb-09
65343	1	0	0	0	F102100	Cerebral degeneration in Niemann-Pick disease	Feb-09
65344	9	0	0	0	C370000	Cystic fibrosis with no meconium ileus	Feb-09
65357	1	0	0	0	B507100	Malignant neoplasm of nasolacrimal duct	Feb-09
65372	14	0	0	0	B11yz00	Malignant neoplasm of other specified site of stomach NOS	Feb-09
65405	5	0	0	0	N237300	Pseudosarcomatous fibromatosis	Feb-09
65434	8	0	0	0	B62z.00	Malignant neoplasms of lymphoid and histiocytic tissue NOS	Feb-09
65460	21	1	0	0	B1z1.00	Malignant neoplasm of spleen NEC	Feb-09
65466	2	0	0	0	B592X00	Kaposi's sarcoma of multiple organs	Feb-09
65483	3	0	0	0	B614400	Hodgkin's nodular sclerosis of lymph nodes of axilla and arm	Feb-09
65489	3	0	0	0	B610.00	Hodgkin's paragranuloma	Feb-09
65490	2	0	0	0	B58y411	Secondary cancer of the vulva	Feb-09
65509	5	0	0	0	PJ50300	Trisomy 9	Feb-09
65584	4	0	0	0	BBj1000	"[M]Hodgkin,s disease, lymphocytic predominance, diffuse"	Feb-09
65599	10	0	0	0	B520200	Malignant neoplasm of acoustic nerve	Feb-09
65605	2	0	0	0	B241200	Malignant neoplasm of myocardium	Feb-09
65625	178	0	0	0	B324.00	Malignant melanoma of scalp and neck	Feb-09
65642	2	0	0	0	B623300	Malignant histiocytosis of intra-abdominal lymph nodes	Feb-09
65701	31	1	0	0	B620z00	Nodular lymphoma NOS	Feb-09

For peer review only - http://bmjopen.bmj.com/site/about/guidelines.xhtml

65721	2	0	0	0	B673.00	Mast cell leukaemia	Feb-09
65777	8	1	0	0	B672.11	Thrombocytic leukaemia	Feb-09
65778	F4Gy011	6	0	0	0	Encephalocoele of orbit	Feb-09
65782	4	0	0	0	B337800	Malignant neoplasm of skin of toe	Feb-09
65861	6	0	0	0	BBQ9.00	[M]Dermoid cyst with malignant transformation	Feb-09
65935	PG43.00	7	0	0	0	Asphyxiating thoracic dysplasia	Feb-09
65952	10	0	0	0	BBbU.00	[M]Desmoplastic medulloblastoma	Feb-09
66000	6	0	0	0	BBB5.00	[M]Adenocarcinoma with apocrine metaplasia	Feb-09
66019	6	0	0	0	L254.11	Suspect fetal damage from maternal alcohol	Feb-09
66064	13	1	0	0	BBbM.00	[M]Giant cell glioblastoma	Feb-09
66073	14	0	0	0	C392400	Severe combined immunodef with low T-and B-cell numbers	Feb-09
66089	6	0	0	0	B65yz00	Other myeloid leukaemia NOS	Feb-09
66155	12	0	0	0	C306200	Argininosuccinic aciduria	Feb-09
66163	11	0	0	0	ByuC200	[X]2ndry+unspcf malignant neoplasm lymph nodes/multi regions	Feb-09
66166	5	0	0	0	B124.00	"Malignant neoplasm, overlapping lesion of small intestine"	Feb-09
66270	11	0	0	0	B000000	"Malignant neoplasm of upper lip, external"	Feb-09
66319	5	1	0	0	B335500	Malignant neoplasm of skin of groin	Feb-09
66327	13	0	0	0	B620000	Nodular lymphoma of unspecified site	Feb-09
66367	10	0	0	0	A789700	HIV dis resulting oth types of non-Hodgkin's lymphoma	Feb-09
66368	6	0	0	0	A789100	HIV disease resulting in cytomegaloviral disease	Feb-09
66384	26	0	0	0	B001000	"Malignant neoplasm of lower lip, external"	Feb-09

For peer review only - http://bmjopen.bmj.com/site/about/guidelines.xhtml

66422	1	0	0	0	B074.00	"Malignant neoplasm, overlapping lesion of nasopharynx"	Feb-09
66444	2	0	0	0	Byu2100	"[X]Malignant neoplasm/overlap lesion/heart,mediastinm+pleura"	Feb-09
66447	13	0	0	0	B335A00	Malignant neoplasm of skin of scapular region	Feb-09
66499	23	0	0	0	P00z.00	Anencephalus NOS	Feb-09
66541	3	0	0	0	BB1J.12	[M]Round cell carcinoma	Feb-09
66603	12	0	0	0	BBgK.00	"[M]Malig lymphoma, follicular centre cell, non-cleaved NOS"	Feb-09
66607	6	0	0	0	BBL4.00	"[M]Mixed tumour, malignant, NOS"	Feb-09
66639	14	0	0	0	B303200	Malignant neoplasm of clavicle	Feb-09
66646	6	0	0	0	B26..00	"Malignant neoplasm, overlap lesion of resp & intrathor orgs"	Feb-09
66694	1	0	0	0	BBr6311	[M]Naegeli-type monocytic leukaemia	Feb-09
66699	4	0	0	0	PK83.00	Fetus and newborn affected by maternal use of alcohol	Feb-09
66750	4	0	0	0	B24z.00	"Malignant neoplasm of heart, thymus and mediastinum NOS"	Feb-09
66775	1	0	0	0	B560100	Secondary and unspec malignant neoplasm mastoid lymph nodes	Feb-09
66876	3	0	0	0	BB81E11	[M]Pseudomucinous adenocarcinoma	Feb-09
66908	5	2	0	0	B306400	Malignant neoplasm of coccygeal vertebra	Feb-09
67019	2	0	0	0	BBK1100	[M]Angiomyosarcoma	Feb-09
67029	12	0	0	0	ByuD500	[X]Other lymphoid leukaemia	Feb-09
67034	13	1	0	0	Byu5000	[X]Mesothelioma of other sites	Feb-09
67107	5	0	0	0	B230.00	Malignant neoplasm of parietal pleura	Feb-09
67203	7	0	0	0	BBgG.11	[M]Lymphoblastic lymphosarcoma NOS	Feb-09
67211	1	0	0	0	B523z00	Malignant neoplasm of spinal meninges NOS	Feb-09
67217	8	0	0	0	B55y100	Malignant neoplasm of trunk NOS	Feb-09

For peer review only - http://bmjopen.bmj.com/site/about/guidelines.xhtml

67234	8	0	0	0	PJ21.00	"Trisomy 18, mosaicism"	Feb-09
67236	2	0	0	0	B512000	Malignant neoplasm of hippocampus	Feb-09
67248	3	0	13	0	5A12.00	Thyroid tumour/metast irradiat	Feb-09
67323	25	3	0	0	B06y.00	"Malignant neoplasm of oropharynx, other specified sites"	Feb-09
67339	1	0	0	0	BBp2.00	[M]Malignant mastocytosis	Feb-09
67342	8	0	0	0	BB5U100	[M]Adenocarcinoma in villous adenoma	Feb-09
67351	P113100	15	0	0	0	Cervical spinal meningocele	Feb-09
67446	10	0	0	0	B001.00	"Malignant neoplasm of lower lip, vermilion border"	Feb-09
67451	2	0	0	0	B30W.00	Malignant neoplasm/overlap lesion/bone+articulr cartilage	Feb-09
67497	6	0	0	0	B106.00	"Malignant neoplasm, overlapping lesion of oesophagus"	Feb-09
67504	4	1	0	0	B003000	"Malignant neoplasm of lower lip, buccal aspect"	Feb-09
67506	9	0	0	0	B614200	Hodgkin's nodular sclerosis of intrathoracic lymph nodes	Feb-09
67518	4	0	0	0	ByuD100	[X]Other types of follicular non-Hodgkin's lymphoma	Feb-09
67575	10	0	0	0	A788W00	HIV disease resulting in unspecified malignant neoplasm	Feb-09
67587	18	0	0	0	BBbZ.00	[M]Pleomorphic xanthoastrocytoma	Feb-09
67700	4	0	0	0	B66..12	Monoblastic leukaemia	Feb-09
67701	35	0	0	0	BB94.11	[M]Secretory breast carcinoma	Feb-09
67703	8	0	0	0	B616.00	"Hodgkin's disease, lymphocytic depletion"	Feb-09
67712	8	0	0	0	BBR2.00	[M]Choriocarcinoma	Feb-09
67748	4	0	0	0	B335400	Malignant neoplasm of skin of umbilicus	Feb-09
67762	1	0	0	0	F103100	Cerebral degeneration in mucopolysaccharidoses	Feb-09

For peer review only - http://bmjopen.bmj.com/site/about/guidelines.xhtml

67763	2	0	0	0	B303400	Malignant neoplasm of costo-vertebral joint	Feb-09
67806	62	0	0	0	B323z00	Malignant melanoma of face NOS	Feb-09
67878	P112.00	1	0	0	0	Hydromyelocele	Feb-09
67884	4	0	0	0	B350100	Malignant neoplasm of areola of male breast	Feb-09
67906	10	5	0	0	F431400	Metastatic disseminated retinitis	Feb-09
67912	3	0	0	0	BB26.11	[M]Papillary epidermoid carcinoma	Feb-09
67913	7	1	0	0	BB6A.00	[M]Ceruminous adenoma and adenocarcinoma	Feb-09
67914	3	0	0	0	B337900	Malignant neoplasm of skin of great toe	Feb-09
67927	3	0	0	0	C372300	Lesch-Nyhan syndrome	Feb-09
67934	6	0	0	0	BBLA.00	"[M]Carcinosarcoma, embryonal type"	Feb-09
67949	10	0	0	0	B48y.00	Malignant neoplasm of other male genital organ	Feb-09
67966	3	0	0	0	BBE1.14	[M]Naevocarcinoma	Feb-09
67970	2	0	0	0	BB1L.00	"[M]Small cell carcinoma, fusiform cell type"	Feb-09
68027	4	0	0	0	ByuA000	[X]Malignant neoplasm/other and unspecified cranial nerves	Feb-09
68039	0	0	0	0	B612400	Hodgkin's sarcoma of lymph nodes of axilla and upper limb	Feb-09
68055	32	0	0	0	B307.00	Malignant neoplasm of long bones of leg	Feb-09
68087	42	1	0	0	D20z.00	Aplastic anaemia NOS	Feb-09
68133	21	0	0	0	B323300	Malignant melanoma of forehead	Feb-09
68155	7	0	0	0	B430100	Malignant neoplasm of fundus of corpus uteri	Feb-09
68161	23	0	0	0	B48y000	Malignant neoplasm of seminal vesicle	Feb-09
68181	2	1	0	0	P002.11	Hemianencephaly	Feb-09
68197	5	0	0	0	B337300	Malignant neoplasm of skin of popliteal fossa area	Feb-09
68220	2	0	0	0	BBW4.11	[M]Fibrochondrosarcoma	Feb-09

For peer review only - http://bmjopen.bmj.com/site/about/guidelines.xhtml

68221	P110100	2	0	0	0	Cervical spina bifida without mention of hydrocephalus	Feb-09
68236	3398	6	0	0	B550.00	"Malignant neoplasm of head, neck and face"	Feb-09
68330	8	0	0	0	B613100	"Hodgkin's, lymphocytic-histiocytic pred of head, face, neck"	Feb-09
68332	12	0	0	0	ByuC600	[X]2ndry malignant neoplasm/oth+unspec parts/nervous system	Feb-09
68399	1	0	0	0	B004200	"Malignant neoplasm of lip unspecified, mucosa"	Feb-09
68410	11	0	0	0	B150200	Primary angiosarcoma of liver	Feb-09
68447	8	0	0	0	BBEV.00	"[M]Blue naevus, malignant"	Feb-09
68456	25	0	0	0	BB5V100	[M]Chromophobe carcinoma	Feb-09
68479	4	3	0	0	BBc7.11	[M]Neuroastrocytoma	Feb-09
68480	7	1	0	0	B350000	Malignant neoplasm of nipple of male breast	Feb-09
68612	2	0	0	0	ZV10z00	[V]Personal history of unspecified malignant neoplasm	Feb-09
68641	8	0	0	0	B517z00	Malignant neoplasm of brain stem NOS	Feb-09
68730	25	0	0	0	BBZN.00	[M]Ameloblastic fibrosarcoma	Feb-09
68757	2	0	0	0	BB5f700	[M]Nonencapsulated sclerosing carcinoma	Feb-09
68783	2	0	0	0	BB60100	[M]Skin appendage carcinoma	Feb-09
68787	19	0	0	0	B55y000	Malignant neoplasm of back NOS	Feb-09
68808	11	0	0	0	BBb2.00	[M]Mixed glioma	Feb-09
68824	2	0	0	0	B48y200	"Malignant neoplasm, overlapping lesion male genital orgs"	Feb-09
68956	8	1	0	0	BBX1.00	"[M]Giant cell tumour of bone, malignant"	Feb-09
68964	2	0	0	0	BBgA.00	"[M]Malignant lymphoma, centroblastic-centrocytic, diffuse"	Feb-09

For peer review only - http://bmjopen.bmj.com/site/about/guidelines.xhtml

68999	1	1	0	0	BBK2z00	[M]Myoma or myosarcoma NOS	Feb-09
69017	10	0	0	0	C370100	Cystic fibrosis with meconium ileus	Feb-09
69027	15	0	0	0	D200.00	Constitutional aplastic anaemia	Feb-09
69061	3	0	0	0	D200011	Constitutional aplastic anaemia without malformation	Feb-09
69104	5	1	0	0	B305100	Malignant neoplasm of carpal bone - lunate	Feb-09
69146	7	0	0	0	B300z00	Malignant neoplasm of bones of skull and face NOS	Feb-09
69299	1	1	0	0	BBrA111	[M]Thrombocytic leukaemia	Feb-09
69300	5	0	0	0	BB1F.00	[M]Polygonal cell carcinoma	Feb-09
69301	1	0	0	0	BBg5.00	"[M]Malignant lymphoma, convoluted cell type NOS"	Feb-09
69381	10	0	0	0	C375100	"Mucopolysaccharidosis, type 1"	Feb-09
69421	3	0	0	0	BBDz.00	[M]Paraganglioma or glomus tumour NOS	Feb-09
69436	1	0	0	0	PG51500	Osteogenesis imperfecta type III	Feb-09
69476	14	0	0	0	PJ50200	Trisomy 8	Feb-09
69497	1	0	0	0	B623000	Malignant histiocytosis of unspecified site	Feb-09
69613	11	1	0	0	C377100	Mucolipidosis type III	Feb-09
69671	2	1	0	0	B010.11	Malignant neoplasm of posterior third of tongue	Feb-09
69761	4	0	0	0	B00zz00	"Malignant neoplasm of lip, vermilion border NOS"	Feb-09
69766	8	0	0	0	A788200	HIV infection with persistent generalised lymphadenopathy	Feb-09
69767	3	0	0	0	AyuC600	[X]HIV disease resulting in other non-Hodgkin's lymphoma	Feb-09
69821	11	1	0	0	B18y600	Malignant neoplasm of the pouch of Douglas	Feb-09
69844	11	0	0	0	BBF5.11	[M]Round cell sarcoma	Feb-09
69858	8	0	0	0	P510.00	Total great vessel transposition	Feb-09

For peer review only - http://bmjopen.bmj.com/site/about/guidelines.xhtml

69927	1	0	0	0	B308800	Malignant neoplasm of first metatarsal bone	Feb-09
69951	5	0	0	0	B055100	Malignant neoplasm of roof of mouth	Feb-09
69980	23	2	0	0	BBgC.00	"[M]Malignant lymphoma, lymphocytic, well differentiated NOS"	Feb-09
69981	10	1	0	0	BBe7.00	"[M]Neurilemmoma, malignant"	Feb-09
70104	6	0	0	0	B521z00	Malignant neoplasm of cerebral meninges NOS	Feb-09
70126	9	0	0	0	B520100	Malignant neoplasm of optic nerve	Feb-09
70320	8	0	0	0	BBD4.11	[M]Jugular paraganglioma	Feb-09
70374	1	0	0	0	B600300	Reticulosarcoma of intra-abdominal lymph nodes	Feb-09
70380	6	0	0	0	B335000	Malignant neoplasm of skin of axillary fold	Feb-09
70383	2	0	0	0	BBM0100	"[M]Brenner tumour, malignant"	Feb-09
70439	4	0	0	0	C375300	"Mucopolysaccharidosis, type III"	Feb-09
70463	16	0	0	0	B315000	Malignant neoplasm of connective and soft tissue of buttock	Feb-09
70509	5	0	0	0	B627D00	Diffuse non-Hodgkin's centroblastic lymphoma	Feb-09
70516	9	0	0	0	BB5D.11	[M]Biliary tract adenomas and adenocarcinomas	Feb-09
70572	4	0	0	0	F151000	Unspecified spinal muscular atrophy	Feb-09
70587	8	0	0	0	B337700	Malignant neoplasm of skin of foot	Feb-09
70637	11	0	0	0	B320.00	Malignant melanoma of lip	Feb-09
70696	3	0	0	0	B02y.00	Malignant neoplasm of other major salivary glands	Feb-09
70709	11	0	4	0	4L49.00	Prion protein markers for Creutzfeldt-Jakob disease	Feb-09
70724	7	1	0	0	B653.00	Myeloid sarcoma	Feb-09
70729	3	0	0	0	B431z00	Malignant neoplasm of isthmus of uterine body NOS	Feb-09

For peer review only - http://bmjopen.bmj.com/site/about/guidelines.xhtml

70736	7	0	0	0	B58y300	Secondary malignant neoplasm of vagina	Feb-09
70740	1	0	0	0	BBm1.11	[M]Malignant reticulosis	Feb-09
70819	26	0	0	0	B055.00	Malignant neoplasm of palate unspecified	Feb-09
70824	20	0	0	0	B540z00	Malignant neoplasm of adrenal gland NOS	Feb-09
70842	7	0	0	0	B627100	Follicular non-Hodg mixed sml cleavd & lge cell lymphoma	Feb-09
70855	7	0	0	0	G558000	Cardiomyopathy in Friedreich's ataxia	Feb-09
70928	5	0	0	0	B022.00	Malignant neoplasm of sublingual gland	Feb-09
70935	1	0	0	0	BBr4000	[M]Erythroleukaemia	Feb-09
70942	8	0	0	0	B510400	Malignant neoplasm of hypothalamus	Feb-09
70951	3	0	0	0	BBP0.00	"[M]Mesothelioma, benign"	Feb-09
70988	4	1	0	0	B337000	Malignant neoplasm of skin of hip	Feb-09
71031	1	0	0	0	B600100	"Reticulosarcoma of lymph nodes of head, face and neck"	Feb-09
71117	2	0	0	0	BBg3.00	"[M]Malignant lymphoma, undifferentiated cell type NOS"	Feb-09
71136	8	0	0	0	B323100	Malignant melanoma of chin	Feb-09
71139	26	0	0	0	B51y.00	Malignant neoplasm of other parts of brain	Feb-09
71142	3	0	0	0	B613000	"Hodgkin's, lymphocytic-histiocytic predominance unspec site"	Feb-09
71147	4	0	0	0	B003.00	"Malignant neoplasm of lower lip, inner aspect"	Feb-09
71204	6	1	0	0	B200000	Malignant neoplasm of cartilage of nose	Feb-09
71238	5	0	0	0	B601100	"Lymphosarcoma of lymph nodes of head, face and neck"	Feb-09
71262	4	0	0	0	B62y600	Malignant lymphoma NOS of intrapelvic lymph nodes	Feb-09
71301	5	0	0	0	BBQA100	"[M]Struma ovarii, malignant"	Feb-09
71304	14	0	0	0	B602z00	Burkitt's lymphoma NOS	Feb-09
71377	1	0	0	0	BBr8000	[M]Eosinophilic leukaemia	Feb-09

For peer review only - http://bmjopen.bmj.com/site/about/guidelines.xhtml

71450	2	0	0	0	A788X00	HIV disease resulting/unspcf infectious+parasitic disease	Feb-09
71497	4	0	0	0	BB5W100	[M]Oxyphilic adenocarcinoma	Feb-09
71526	15	0	0	0	PKyF.00	Alstrom syndrome	Feb-09
71584	5	2	0	0	B507.00	Malignant neoplasm of lacrimal duct	Feb-09
71619	1	0	0	0	BBgT.00	"[M]Malignant lymphoma, large cell, noncleaved, diffuse"	Feb-09
71625	1	0	0	0	B601000	Lymphosarcoma of unspecified site	Feb-09
71627	2	0	0	0	BB61200	[M]Sweat gland adenocarcinoma	Feb-09
71652	2	0	0	0	BBgP.00	"[M]Malignant lymphoma, mixed small and large cell, diffuse"	Feb-09
71672	8	0	25	0	4M23.00	Lymphoma stage IV	Feb-09
71810	11	0	0	0	B304.00	Malignant neoplasm of scapula and long bones of upper arm	Feb-09
71850	8	0	0	0	BBr6000	[M]Myeloid leukaemia NOS	Feb-09
71869	11	0	0	0	BBf2.00	[M]Alveolar soft part sarcoma	Feb-09
71895	11	3	0	0	BB56.00	[M]Superficial spreading adenocarcinoma	Feb-09
71946	3	1	0	0	B201300	Malignant neoplasm of mastoid air cells	Feb-09
72018	P118100	0	0	0	0	Cervical spina bifida without hydrocephalus - closed	Feb-09
72127	5	0	0	0	B484.00	Malignant neoplasm of epididymis	Feb-09
72139	1	0	0	0	PJ10.00	"Trisomy 13, meiotic nondisjunction"	Feb-09
72174	1	0	0	0	B4A4.00	Malignant neoplasm of paraurethral glands	Feb-09
72179	1	0	0	0	BBr0200	[M]Subacute leukaemia NOS	Feb-09
72192	14	0	0	0	BB5Xz00	[M]Clear cell adenoma or adenocarcinoma NOS	Feb-09
72196	6	1	0	0	BBgG.00	"[M]Malignant lymphoma, lymphocytic, poorly different NOS"	Feb-09
72197	2	0	0	0	B67y000	Lymphosarcoma cell leukaemia	Feb-09
72212	4	0	0	0	B308200	Malignant neoplasm of calcaneum	Feb-09
72222	1	0	0	0	BBrA100	[M]Megakaryocytic leukaemia	Feb-09

For peer review only - http://bmjopen.bmj.com/site/about/guidelines.xhtml

72224	7	1	0	0	B1z1100	Fibrosarcoma of spleen	Feb-09
72241	1	1	0	0	BBgH.00	[M]Prolymphocytic lymphosarcoma	Feb-09
72265	8	0	0	0	PJ1z.00	Patau's syndrome NOS	Feb-09
72277	4	1	0	0	BB5V700	[M]Basophil carcinoma	Feb-09
72303	1	0	0	0	K01w000	Finnish nephrosis syndrome	Feb-09
72310	1	1	0	0	BBr0400	[M]Aleukaemic leukaemia NOS	Feb-09
72433	3	0	0	0	BBh0.00	[M]Reticulosarcoma NOS	Feb-09
72443	2	0	0	0	BBZ2.00	"[M]Odontogenic tumour, malignant"	Feb-09
72445	4	0	0	0	B161000	Malignant neoplasm of cystic duct	Feb-09
72464	4	0	0	0	B305.12	Malignant neoplasm of metacarpal bones	Feb-09
72522	2	0	0	0	B313200	Malignant neoplasm of great vessels	Feb-09
72668	5	0	0	0	G210100	Malignant hypertensive heart disease with CCF	Feb-09
72707	13	0	0	0	P0...00	Anencephalus and similar anomalies	Feb-09
72723	5	0	0	0	B430000	Malignant neoplasm of cornu of corpus uteri	Feb-09
72725	8	0	0	0	B62y200	Malignant lymphoma NOS of intrathoracic lymph nodes	Feb-09
72774	2	0	0	0	B642.00	Subacute lymphoid leukaemia	Feb-09
73055	P20z.00	10	0	0	0	Encephalocele NOS	Feb-09
73065	8	0	0	0	C370y00	Cystic fibrosis with other manifestations	Feb-09
73066	1	0	0	0	BBrA.00	[M]Miscellaneous leukaemias	Feb-09
73085	2	0	0	0	P10y.00	Other specified spina bifida with hydrocephalus	Feb-09
73088	2	0	0	0	BBr9000	[M]Monocytic leukaemia NOS	Feb-09
73213	11	0	0	0	B581.00	Secondary malignant neoplasm of other urinary organs	Feb-09
73251	0	0	0	0	BBEM.00	[M]Malignant melanoma in giant pigmented naevus	Feb-09
73275	5	0	0	0	BB5N100	[M]Adenocarcinoma in adenomatous polposis coli	Feb-09
73293	7	0	0	0	G240z00	Secondary malignant hypertension NOS	Feb-09

For peer review only - http://bmjopen.bmj.com/site/about/guidelines.xhtml

73296	5	0	0	0	Byu3100	"[X]Malignant neoplasm/bones+articular cartilage/limb,unspfd"	Feb-09
73434	6	0	0	0	BB5L300	[M]Adenocarcinoma in multiple adenomatous polyps	Feb-09
73439	9	0	0	0	B064z00	Malignant neoplasm of anterior epiglottis NOS	Feb-09
73510	10	0	0	0	B550500	Malignant neoplasm of supraclavicular fossa NOS	Feb-09
73530	7	1	0	0	B305.00	Malignant neoplasm of hand bones	Feb-09
73532	2	0	0	0	B613300	"Hodgkin's, lymphocytic-histiocytic pred intra-abdominal node"	Feb-09
73536	5	0	0	0	B327000	Malignant melanoma of hip	Feb-09
73539	26	2	0	0	P51z.00	Great vessel transposition NOS	Feb-09
73556	5	0	0	0	B305z00	Malignant neoplasm of hand bones NOS	Feb-09
73583	25	0	0	0	F14y000	Ataxia-telangiectasia	Feb-09
73608	P117z00	2	0	0	0	Spina bifida without hydrocephalus - open NOS	Feb-09
73612	1	0	0	0	PE02.00	Potter's facies	Feb-09
73614	8	0	0	0	B004000	"Malignant neoplasm of lip unspecified, buccal aspect"	Feb-09
73616	15	0	0	0	B58y200	Secondary malignant neoplasm of cervix uteri	Feb-09
73662	1	0	0	0	BB5Wz00	[M]Oxyphilic adenoma or adenocarcinoma NOS	Feb-09
73712	P115z00	7	0	0	0	Myelocele NOS	Feb-09
73742	7	0	0	0	F427K00	Lipofuscinosis NOS	Feb-09
73744	5	0	0	0	B322z00	Malignant melanoma of ear and external auricular canal NOS	Feb-09
73760	12	0	0	0	B334z00	Malignant neoplasm of scalp or skin of neck NOS	Feb-09
73777	2	0	0	0	B624z00	Leukaemic reticuloendotheliosis NOS	Feb-09
73916	8	0	0	0	BBK0400	[M]Epithelioid leiomyosarcoma	Feb-09

For peer review only - http://bmjopen.bmj.com/site/about/guidelines.xhtml

73962	4	1	0	0	B000.00	"Malignant neoplasm of upper lip, vermilion border"	Feb-09
73988	6	0	0	0	B524500	Malignant neoplasm of peripheral nerve of pelvis	Feb-09
73992	5	0	0	0	B504.00	Malignant neoplasm of cornea	Feb-09
74896	30	0	0	0	B161z00	Malignant neoplasm of extrahepatic bile ducts NOS	Feb-09
85190	12	0	0	0	F562100	Malignant positional vertigo	Feb-09
85988	11	0	0	0	P210.00	Hydromicrocephaly	Feb-09
86046	17	0	0	0	B524400	Malignant neoplasm of peripheral nerve of abdomen	Feb-09
86812	12	0	0	0	B305D00	Malignant neoplasm of phalanges of hand	Feb-09
86820	6	0	0	0	BBP7.00	"[M]Mesothelioma, biphasic type, malignant"	Feb-09
86996	3	0	0	0	B501000	Malignant neoplasm of connective tissue of orbit	Feb-09
86997	8	0	0	0	Byu2400	[X]Malignant neoplasm/ill-defined sites within resp system	Feb-09
87003	1	0	0	0	BBLC100	"[M]Mesenchymoma, malignant"	Feb-09
87113	0	0	0	0	B54X.00	"Malignant neoplasm-pluriglandular involvement,unspecified"	Feb-09
87335	314	15	0	0	B624.12	Hairy cell leukaemia	Feb-09
88022	28	0	0	0	ByuC400	[X]Secondary malignant neoplasm/oth+unspcfd digestive organs	Feb-09
88144	5	0	0	0	B52y.00	Malignant neoplasm of other specified part of nervous system	Feb-09
88362	5	0	0	0	B08y.00	Malignant neoplasm of other specified hypopharyngeal site	Feb-09
89230	0	0	0	0	BBj9.00	[M]Hodgkin's granuloma	Feb-09
89258	5	0	0	0	B524200	"Malignant neoplasm of peripheral nerve of low limb, incl hip"	Feb-09
89329	7	0	0	0	ByuD800	[X]Other specified leukaemias	Feb-09

For peer review only - http://bmjopen.bmj.com/site/about/guidelines.xhtml

89593	7	0	0	0	B151200	Malignant neoplasm of intrahepatic biliary passages	Feb-09
89657	1	0	0	0	B626z00	Malignant mast cell tumour NOS	Feb-09
89762	1	0	0	0	ByuD700	[X]Other monocytic leukaemia	Feb-09
89909	9	0	0	0	B003200	"Malignant neoplasm of lower lip, mucosa"	Feb-09
89916	1	0	0	0	B553100	Malignant neoplasm of presacral region	Feb-09
90124	6	0	0	0	B067.00	Malignant neoplasm of posterior wall of oropharynx	Feb-09
90201	9	0	0	0	B62x000	T-zone lymphoma	Feb-09
90290	16	0	0	0	B18y700	Malignant neoplasm of mesentery	Feb-09
90482	2	0	0	0	P103z11	Thoracolumbar spina bifida with hydrocephalus - closed	Feb-09
90487	6	0	0	0	BBb3.11	[M]Subependymal astrocytoma NOS	Feb-09
90610	8	1	0	0	B002300	"Malignant neoplasm of upper lip, oral aspect"	Feb-09
90659	13	0	0	0	B54y.00	Malignant neoplasm of other specified endocrine gland	Feb-09
91035	2	0	0	0	B010z00	Malignant neoplasm of fixed part of tongue NOS	Feb-09
91037	6	0	0	0	B06yz00	Malignant neoplasm of other specified site of oropharynx NOS	Feb-09
91240	15	0	0	0	B517300	Malignant neoplasm of pons	Feb-09
91457	13	0	0	0	Byu5900	"[X]Malignant neoplasm/connective + soft tissue,unspecified"	Feb-09
91509	13	0	0	0	B471z00	Malignant neoplasm of descended testis NOS	Feb-09
91586	9	0	0	0	B311400	Malignant neoplasm of connective and soft tissue of finger	Feb-09
91600	P113200	8	0	0	0	Thoracic spinal meningocele	Feb-09
91842	3	0	0	0	BB69z00	[M]Sebaceous adenoma or adenocarcinoma NOS	Feb-09

For peer review only - http://bmjopen.bmj.com/site/about/guidelines.xhtml

91843	9	0	0	0	B003100	"Malignant neoplasm of lower lip, frenulum"	Feb-09
91895	4	0	0	0	B064100	Malignant neoplasm of glossoepiglottic fold	Feb-09
91900	5	0	0	0	B61z400	Hodgkin's disease NOS of lymph nodes of axilla and arm	Feb-09
92068	5	0	0	0	B620300	Nodular lymphoma of intra-abdominal lymph nodes	Feb-09
92245	1	0	0	0	B613200	"Hodgkin's, lymphocytic-histiocytic pred intrathoracic nodes"	Feb-09
92329	11	1	0	0	B48yz00	Malignant neoplasm of other male genital organ NOS	Feb-09
92371	5	0	0	0	B304300	Malignant neoplasm of radius	Feb-09
92380	1	0	0	0	B602500	Burkitt's lymphoma of lymph nodes of inguinal region and leg	Feb-09
92382	1	0	0	0	B308B00	Malignant neoplasm of fourth metatarsal bone	Feb-09
92720	3	0	0	0	B243.00	Malignant neoplasm of posterior mediastinum	Feb-09
93133	0	1	0	0	PJ22.00	"Trisomy 18, translocation"	Feb-09
93175	2	0	0	0	BBZ2.11	[M]Intraosseous carcinoma	Feb-09
93177	2	0	0	0	PG52100	Osteopetrosis - congenita type	Feb-09
93218	2	0	0	0	B03z.00	Malignant neoplasm of gum NOS	Feb-09
93342	4	0	0	0	B66z.00	Monocytic leukaemia NOS	Feb-09
93352	15569	886	0	0	B338.00	Squamous cell carcinoma of skin	Feb-09
93372	F103.00	3	0	0	0	Cerebral degeneration in diseases EC	Feb-09
93380	108	1	0	0	C10N100	Cystic fibrosis related diabetes mellitus	Feb-09
93478	4	0	0	0	B138.00	"Malignant neoplasm, overlapping lesion of colon"	Feb-09
93537	3	0	0	0	B517200	Malignant neoplasm of midbrain	Feb-09
93561	8	0	0	0	P601z00	Congenital atresia of pulmonary valve NOS	Feb-09

For peer review only - http://bmjopen.bmj.com/site/about/guidelines.xhtml

93665	6	0	0	0	Byu5300	"[X]Kaposi's sarcoma, unspecified"	Feb-09
93717	3	0	0	0	BBD7.00	"[M]Extra-adrenal paraganglioma, NOS"	Feb-09
93762	6	0	0	0	B42..00	Malignant neoplasm of placenta	Feb-09
93778	2	0	0	0	B1z1z00	Malignant neoplasm of spleen NOS	Feb-09
93842	1	0	0	0	B062300	Malignant neoplasm of palatopharyngeal arch	Feb-09
93902	3	0	0	0	P103.00	Spina bifida with hydrocephalus - closed	Feb-09
93951	3	0	0	0	B613500	"Hodgkin's, lymphocytic-histiocytic pred inguinal and leg"	Feb-09
93963	13	0	0	0	F101600	Sandhoff disease	Feb-09
94005	38	0	0	0	B615z00	"Hodgkin's disease, mixed cellularity NOS"	Feb-09
94083	1	0	0	0	BB5P.00	[M]Solid carcinoma NOS	Feb-09
94174	9	0	0	0	B67y.00	Other and unspecified leukaemia	Feb-09
94220	2	0	0	0	B540100	Malignant neoplasm of adrenal medulla	Feb-09
94239	1	0	0	0	BBp1.00	[M]Mast cell sarcoma	Feb-09
94251	2	0	0	0	B00z100	"Malignant neoplasm of lip, unspecified, lipstick area"	Feb-09
94267	19	0	0	0	BBb3.00	[M]Subependymal glioma	Feb-09
94278	25	0	0	0	B110111	Malignant neoplasm of gastro-oesophageal junction	Feb-09
94279	1	0	0	0	B61z700	Hodgkin's disease NOS of spleen	Feb-09
94286	4	0	0	0	BBG8.11	[M]Congenital fibrosarcoma	Feb-09
94355	3	0	0	0	B55y200	Malignant neoplasm of flank NOS	Feb-09
94390	3	0	0	0	B070.00	Malignant neoplasm of roof of nasopharynx	Feb-09
94407	3	0	0	0	B615100	"Hodgkin's mixed cellularity of lymph nodes head, face, neck"	Feb-09
94415	2	0	0	0	B623100	"Malignant histiocytosis of lymph nodes head, face and neck"	Feb-09
94427	1	0	0	0	B305C00	Malignant neoplasm of fifth metacarpal bone	Feb-09

For peer review only - http://bmjopen.bmj.com/site/about/guidelines.xhtml

94438	3	0	0	0	BB85z00	[M]Signet ring carcinoma NOS	Feb-09
94441	3	0	0	0	B003300	"Malignant neoplasm of lower lip, oral aspect"	Feb-09
94467	1	0	0	0	P0z..00	Anencephalus and similar anomalies NOS	Feb-09
94486	H593.00	1020	0	2	0	Chronic type 2 respiratory failure	Feb-09
94533	4	0	0	0	RyuC000	[X]Sudden infant death syndrome	Feb-09
94597	3	0	0	0	ZV10611	[V]Personal history of lymphoid leukaemia	Feb-09
94598	P115100	2	0	0	0	Cervical myelocele	Feb-09
94776	6	0	0	0	B1z2.00	"Malignant neoplasm, overlapping lesion of digestive system"	Feb-09
94793	1Z1B.00	2761	0	21	0	Chronic kidney disease stage 3 with proteinuria	Feb-09
94810	1	0	0	0	BBB4.00	[M]Adenocarcinoma with spindle cell metaplasia	Feb-09
94873	25	2	0	0	BB2A.13	[M]Squamous cell carcinoma of skin NOS	Feb-09
94925	140	2	0	0	Pyu9D00	[X]Primary ciliary dyskinesia	Feb-09
94935	2	0	14	0	4M21.00	Lymphoma stage II	Feb-09
94946	H592.00	210	0	0	0	Chronic type 1 respiratory failure	Feb-09
94965	1Z15.00	19544	0	31	1	Chronic kidney disease stage 3A	Feb-09
94975	4	0	0	0	B241300	Malignant neoplasm of pericardium	Feb-09
94995	5	0	0	0	B620500	Nodular lymphoma of lymph nodes of inguinal region and leg	Feb-09
95008	1	0	0	0	BB82112	[M]Gelatinous adenocarcinoma	Feb-09
95016	2	0	0	0	B0z1.00	Malignant neoplasm of Waldeyer's ring	Feb-09
95024	4	0	0	0	BBG8.00	[M]Infantile fibrosarcoma	Feb-09
95049	4	0	0	0	B616000	Hodgkin's lymphocytic depletion of unspecified site	Feb-09
95057	3	0	0	0	B34y000	Malignant neoplasm of ectopic site of female breast	Feb-09
95058	1	0	0	0	B600700	Reticulosarcoma of spleen	Feb-09

For peer review only - http://bmjopen.bmj.com/site/about/guidelines.xhtml

95122	1Z1H.00	1243	0	50	0	Chronic kidney disease stage 4 with proteinuria	Feb-09
95123	1Z1C.00	10576	0	6	0	Chronic kidney disease stage 3 without proteinuria	Feb-09
95145	1Z1B.11	67	0	0	0	CKD stage 3 with proteinuria	Feb-09
95150	4	0	0	0	BB81B00	[M]Serous surface papillary carcinoma	Feb-09
95175	1Z1E.00	11978	0	15	0	Chronic kidney disease stage 3A without proteinuria	Feb-09
95176	1Z1E.11	103	0	0	0	CKD stage 3A without proteinuria	Feb-09
95177	1Z1G.00	4533	0	23	0	Chronic kidney disease stage 3B without proteinuria	Feb-09
95178	1Z1F.00	1288	0	20	0	Chronic kidney disease stage 3B with proteinuria	Feb-09
95179	1Z16.00	7458	0	47	0	Chronic kidney disease stage 3B	Feb-09
95180	1Z1F.11	32	0	1	0	CKD stage 3B with proteinuria	Feb-09
95182	3	0	0	0	B308100	Malignant neoplasm of talus	Feb-09
95188	1Z1C.11	175	0	0	0	CKD stage 3 without proteinuria	Feb-09
95323	1	0	0	0	B35z000	Malignant neoplasm of ectopic site of male breast	Feb-09
95338	2	2	0	0	B613600	"Hodgkin's, lymphocytic-histiocytic pred intrapelvic nodes"	Feb-09
95373	3	0	0	0	BBCC100	"[M]Leydig cell tumour, malignant"	Feb-09
95405	1Z1L.00	142	0	1	0	Chronic kidney disease stage 5 without proteinuria	Feb-09
95406	1Z1J.00	1558	0	43	0	Chronic kidney disease stage 4 without proteinuria	Feb-09
95408	1Z1D.00	1692	0	17	0	Chronic kidney disease stage 3A with proteinuria	Feb-09
95421	13	0	0	0	B45y.00	Malignant neoplasm of other specified female genital organ	Feb-09
95429	7	0	0	0	B071.00	Malignant neoplasm of posterior wall of nasopharynx	Feb-09

For peer review only - http://bmjopen.bmj.com/site/about/guidelines.xhtml

For peer review only

95458	8	0	0	0	B300300	Malignant neoplasm of nasal bone	Feb-09
95478	P117200	1	0	0	0	Thoracic spina bifida without hydrocephalus - open	Feb-09
95480	9	0	0	0	B001100	"Malignant neoplasm of lower lip, lipstick area"	Feb-09
95505	6	0	0	0	B41y000	Malignant neoplasm of cervical stump	Feb-09
95508	1Z1K.00	360	0	5	0	Chronic kidney disease stage 5 with proteinuria	Feb-09
95571	1Z1D.11	25	0	0	0	CKD stage 3A with proteinuria	Feb-09
95609	6	0	0	0	BB5B300	"[M]Insulinoma, malignant"	Feb-09
95615	1	0	0	0	F150.11	Infantile spinal muscular atrophy	Feb-09
95629	6	0	0	0	B325500	Malignant melanoma of perineum	Feb-09
95630	1	0	0	0	B62x600	True histiocytic lymphoma	Feb-09
95644	4	0	0	0	B241.00	Malignant neoplasm of heart	Feb-09
95671	13	0	0	0	Byu5700	"[X]Malignant neoplasm of peritoneum, unspecified"	Feb-09
95715	31	0	0	0	B627900	Mucosa-associated lymphoma	Feb-09
95772	9	1	0	0	B051000	Malignant neoplasm of upper buccal sulcus	Feb-09
95783	11	0	0	0	B17yz00	Malignant neoplasm of specified site of pancreas NOS	Feb-09
95792	9	0	0	0	B62zz00	Lymphoid and histiocytic malignancy NOS	Feb-09
95818	4	0	0	0	BBD1.00	"[M]Paraganglioma, malignant"	Feb-09
95972	835	3	0	0	F207.00	Relapsing and remitting multiple sclerosis	Feb-09
96003	4	0	0	0	B055000	Malignant neoplasm of junction of hard and soft palate	Feb-09
96094	40	0	0	0	B119.00	Siewert type III adenocarcinoma	Feb-09
96183	1	0	0	0	BBj4.00	"[M]Hodgkin's disease,lymphocytic depletion,diffuse fibrosis"	Apr-09
96226	4	0	0	0	ByuC100	[X]Malignant neoplasm/overlap lesion/other+ill-defined sites	Apr-09
96231	8	0	0	0	BBGJ.00	"[M]Fibroxanthoma, malignant"	Apr-09

For peer review only - http://bmjopen.bmj.com/site/about/guidelines.xhtml

96246	280	2	0	0	F208.00	Secondary progressive multiple sclerosis	Apr-09
96256	671	13	0	0	F37y100	Axonal sensorimotor neuropathy	Apr-09
96291	19	0	0	0	F204.00	Benign multiple sclerosis	Apr-09
96429	3	0	0	0	B470z00	Malignant neoplasm of undescended testis NOS	Apr-09
96445	0	0	0	0	B300B00	Malignant neoplasm of turbinate	Apr-09
96494	10	0	0	0	BB5Tz00	[M]Papillary adenoma or adenocarcinoma NOS	Apr-09
96515	1	0	0	0	BBP2.00	"[M]Fibrous mesothelioma, benign"	Apr-09
96585	1	0	0	0	B32y000	Overlapping malignant melanoma of skin	Apr-09
96607	164	2	0	0	F206.00	Primary progressive multiple sclerosis	Apr-09
96635	3	0	0	0	B17y000	Malignant neoplasm of ectopic pancreatic tissue	Apr-09
96637	4	0	0	0	790M400	Biventricular repair of hypoplastic left heart syndrome	Apr-09
96709	P110200	3	0	0	0	Thoracic spina bifida without mention of hydrocephalus	Apr-09
96751	1	0	0	0	AyuCB00	"[X]HIV disease result/haematological+immunologic abnorms,NEC"	Apr-09
96782	4	0	0	0	B003z00	"Malignant neoplasm of lower lip, inner aspect NOS"	Apr-09
96783	3	0	0	0	B005.00	Malignant neoplasm of commissure of lip	Apr-09
96798	1	0	0	0	BBd2.12	[M]Meningothelial sarcoma	May-09
96802	8	0	0	0	B11y100	Malignant neoplasm of posterior wall of stomach NEC	May-09
96860	F11x900	42	0	4	0	Cerebral degeneration in Parkinson's disease	May-09
96869	4	1	0	0	B071z00	Malignant neoplasm of posterior wall of nasopharynx NOS	May-09
96893	4	0	0	0	BBrA300	[M]Myeloid sarcoma	Jun-09
97059	64	4	0	0	PKyz700	Angelman's syndrome	Jul-09

For peer review only - http://bmjopen.bmj.com/site/about/guidelines.xhtml

97091	6	0	0	0	ByuC500	[X]2ndry malignant neoplasm/bladder+oth+unsp urinary organs	Aug-09
97092	4	0	0	0	F100100	Schulz's disease	Aug-09
97332	11	0	0	0	B213z00	Malignant neoplasm of laryngeal cartilage NOS	Aug-09
97422	F11x.00	1	0	0	0	Cerebral degeneration in other disease EC	Aug-09
97454	3	1	0	0	F427K11	Lipofuscinosis NEC	Aug-09
97463	4	0	0	0	BBF4.00	[M]Giant cell sarcoma (except of bone)	Aug-09
97499	51	0	0	0	B118.00	Siewert type II adenocarcinoma	Aug-09
97530	7	0	0	0	B051100	Malignant neoplasm of lower buccal sulcus	Aug-09
97547	5	0	0	0	B551200	Malignant neoplasm of intrathoracic site NOS	Aug-09
97577	1	0	0	0	B602300	Burkitt's lymphoma of intra-abdominal lymph nodes	Sep-09
97587	1Z1J.11	47	0	2	0	CKD stage 4 without proteinuria	Sep-09
97593	6	0	0	0	BBZG.00	"[M]Ameloblastoma, malignant"	Sep-09
97683	1Z1L.11	13	0	0	0	CKD stage 5 without proteinuria	Sep-09
97746	8	0	0	0	B61z800	Hodgkin's disease NOS of lymph nodes of multiple sites	Oct-09
97751	6	2	0	0	PG51600	Osteogenesis imperfecta type IV	Oct-09
97832	5	0	0	0	B58y211	Secondary cancer of the cervix	Oct-09
97852	3	0	0	0	BBk7.00	"[M]Malignant lymphoma, centroblastic type, follicular"	Oct-09
97863	2	0	0	0	B615000	"Hodgkin's disease, mixed cellularity of unspecified site"	Oct-09
97875	3	0	0	0	B175.00	"Malignant neoplasm, overlapping lesion of pancreas"	Oct-09
97916	1	0	0	0	Q007111	Fetal alcohol syndrome	Oct-09
97996	8	0	0	0	B44y.00	Malignant neoplasm of other site of uterine adnexa	Nov-09

For peer review only - http://bmjopen.bmj.com/site/about/guidelines.xhtml

98009	0	0	0	0	BBrA312	[M]Granulocytic sarcoma	Nov-09
98020	G558z00	2	0	0	0	"Cardiomyopathy in diseases EC, NOS"	Nov-09
98104	4	0	0	0	B23y.00	Malignant neoplasm of other specified pleura	Dec-09
98142	29	0	0	0	B107.00	Siewert type I adenocarcinoma	Dec-09
98298	1	0	0	0	P100z00	Spina bifida with hydrocephalus NOS	Dec-09
98322	1	0	0	0	BBT7100	"[M]Haemangioendothelioma, malignant"	Jan-10
98361	3	0	0	0	Byu5B00	[X]Kaposi's sarcoma of other sites	Jan-10
98483	2	0	0	0	BBZN.11	[M]Odontogenic fibrosarcoma	Feb-10
98500	5	0	0	0	B002200	"Malignant neoplasm of upper lip, mucosa"	Feb-10
98537	0	0	0	0	B201100	Malignant neoplasm of tympanic cavity	Feb-10
98559	1	0	0	0	BBW8.00	"[M]Chondroblastoma, malignant"	Feb-10
98596	19	0	0	0	ByuD200	[X]Other types of diffuse non-Hodgkin's lymphoma	Mar-10
98617	7	0	0	0	PKyz711	Angelman syndrome	Mar-10
98662	1	0	0	0	PG51400	Osteogenesis imperfecta type II	Mar-10
98740	4	0	0	0	B000z00	"Malignant neoplasm of upper lip, vermilion border NOS"	Mar-10
98753	7	0	0	0	P228300	Aicardi syndrome	Mar-10
98781	1	0	0	0	BB5F.00	[M]Trabecular adenocarcinoma	Mar-10
98797	3	0	0	0	BBLD.00	[M]Embryonal sarcoma	Mar-10
98800	2	0	0	0	BBbG.12	[M]Piloid astrocytoma	Mar-10
98811	1	0	0	0	P102.00	Spina bifida with hydrocephalus - open	Mar-10
98825	2	0	0	0	BB5B600	[M]Mixed islet cell and exocrine adenocarcinoma	Mar-10
98840	1	0	0	0	B610300	Hodgkin's paragranuloma of intra-abdominal lymph nodes	Mar-10
98883	4	0	0	0	BB9D.00	[M]Medullary carcinoma with lymphoid stroma	Apr-10

For peer review only - http://bmjopen.bmj.com/site/about/guidelines.xhtml

98909	2	0	0	0	B611100	"Hodgkin's granuloma of lymph nodes of head, face and neck"	Apr-10
98911	2	0	0	0	B200100	Malignant neoplasm of nasal conchae	Apr-10
98961	1	0	0	0	BBk2.00	"[M]Malignant lymphoma, centroblastic-centrocytic, follicular"	Apr-10
99001	4	1	0	0	B002100	"Malignant neoplasm of upper lip, frenulum"	Apr-10
99012	1	0	0	0	B61z500	Hodgkin's disease NOS of lymph nodes inguinal region and leg	Apr-10
99015	1	0	0	0	B66y.00	Other monocytic leukaemia	Apr-10
99096	1	0	0	0	Byu2300	[X]Malignant neopl/overlapping les/resp+intrathoracic organs	Apr-10
99160	1Z1K.11	18	0	0	0	CKD stage 5 with proteinuria	Apr-10
99185	1	0	0	0	B062100	Malignant neoplasm of glossopalatine fold	Apr-10
99200	1	0	0	0	BBj7.00	"[M]Hodgkin's disease, nodular sclerosis, cellular phase"	Apr-10
99240	1	0	0	0	B600z00	Reticulosarcoma NOS	Apr-10
99257	4	0	0	0	B324z00	Malignant melanoma of scalp and neck NOS	May-10
99281	P117.00	2	0	0	0	Spina bifida without hydrocephalus - open	May-10
99299	P114200	3	0	0	0	Thoracic meningomyelocele	May-10
99312	1Z1H.11	86	0	0	0	CKD stage 4 with proteinuria	May-10
99332	P114000	1	0	0	0	Meningomyelocele of unspecified site	May-10
99386	3	0	0	0	B073200	Malignant neoplasm posterior margin nasal septum and choanae	May-10
99413	5	0	0	0	B67yz00	Other and unspecified leukaemia NOS	May-10
99493	2	0	0	0	B002.00	"Malignant neoplasm of upper lip, inner aspect"	May-10
99511	1	0	0	0	B574200	Secondary malignant neoplasm of ileum	May-10

For peer review only - http://bmjopen.bmj.com/site/about/guidelines.xhtml

99572	1	0	0	0	B312500	Malignant neoplasm of connective and soft tissue of toe	May-10
99621	3	2	0	0	B520.00	Malignant neoplasm of cranial nerves	Jun-10
99655	3	0	0	0	BBg6.00	[M]Lymphosarcoma NOS	Jun-10
99665	3	0	0	0	BBV..11	[M]Juxtacortical osteogenic sarcoma	Jun-10
99702	2	0	0	0	BBn3.00	"[M]Plasma cell tumour, malignant"	Jun-10
99711	7	0	0	0	C302511	Oculocerebrorenal syndrome	Jun-10
99797	1	0	0	0	BBX3.00	[M]Malignant giant cell tumour of soft parts	Jun-10
99887	2	0	0	0	B60y.00	Other specified reticulosarcoma or lymphosarcoma	Jun-10
99896	3	0	0	0	B12y.00	Malignant neoplasm of other specified site small intestine	Jun-10
99913	1	0	0	0	B510300	Malignant neoplasm of globus pallidus	Jun-10
99951	3	0	0	0	B60z.00	Reticulosarcoma or lymphosarcoma NOS	Jun-10
100002	7	0	0	0	B062z00	Malignant neoplasm of tonsillar fossa NOS	Jun-10
100006	3	0	0	0	B602200	Burkitt's lymphoma of intrathoracic lymph nodes	Jun-10
100035	71	6	0	0	1J0L.00	Suspected malignant mesothelioma	Jul-10
100083	123	1	0	0	B546.00	Neuroblastoma	Jul-10
100111	1	0	0	0	BB46.00	[M]Schneiderian carcinoma	Jul-10
100144	6	0	0	0	B004300	"Malignant neoplasm of lip, oral aspect"	Jul-10
100175	2	0	0	0	PKyE.00	Barber-Say syndrome	Jul-10
100267	4	0	0	0	BBZG.11	"[M]Adamantinoma, malignant"	Jul-10
100296	9	0	0	0	B582100	Secondary malignant neoplasm of skin of face	Jul-10
100352	2	0	0	0	B601500	Lymphosarcoma of lymph nodes of inguinal region and leg	Jul-10
100375	3	0	0	0	BBP4.00	"[M]Epithelioid mesothelioma, benign"	Aug-10
100423	1	0	0	0	B610100	"Hodgkin's paragranuloma of lymph nodes of head, face, neck"	Aug-10

For peer review only - http://bmjopen.bmj.com/site/about/guidelines.xhtml

100430	155	1	0	0	66k..00	Cystic fibrosis monitoring	Aug-10
100544	1	0	0	0	BBh2.00	"[M]Reticulosarcoma, nodular"	Aug-10
100582	P20z100	1	0	0	0	Encephalocele of other specified site	Aug-10
100584	3	0	0	0	B110000	Malignant neoplasm of cardiac orifice of stomach	Aug-10
100610	6	0	0	0	C370400	Arthropathy in cystic fibrosis	Sep-10
100615	1	0	0	0	B626500	Mast cell malignancy of lymph nodes inguinal region and leg	Sep-10
100622	1	0	0	0	P51z.11	Transposition of arterial trunk NEC	Sep-10
100625	1	0	0	0	BB5R500	"[M]Carcinoid tumour, nonargentaffin, malignant"	Sep-10
100633	1Z1G.11	33	0	0	0	CKD stage 3B without proteinuria	Sep-10
100721	1	0	0	0	B002z00	"Malignant neoplasm of upper lip, inner aspect NOS"	Sep-10
100733	4	0	0	0	B51yz00	Malignant neoplasm of other part of brain NOS	Sep-10
100786	5	0	0	0	B651000	Chronic eosinophilic leukaemia	Nov-10
100900	497	32	0	0	B927.12	Neurofibromatosis type 1	Nov-10
100906	1	0	0	0	B00z000	"Malignant neoplasm of lip, unspecified, external"	Nov-10
100918	1	0	0	0	B073z00	Malignant neoplasm of anterior wall of nasopharynx NOS	Nov-10
100927	1	0	0	0	BBr4z00	[M]Erythroleukaemia NOS	Nov-10
101013	P110000	1	0	0	0	"Spina bifida without hydrocephalus, site unspecified"	Dec-10
101052	57	1	0	0	B929.00	Neurofibromatosis type 2	Dec-10
101086	1	0	0	0	B520z00	Malignant neoplasm of cranial nerves NOS	Dec-10
101114	96	1	0	0	B627A00	Diffuse non-Hodgkin's large cell lymphoma	Dec-10
101222	2	0	0	0	F151111	Juvenile spinal muscular atrophy	Dec-10
101312	P20..11	2	0	0	0	Hydroencephalocele	Jan-11

For peer review only - http://bmjopen.bmj.com/site/about/guidelines.xhtml

101342	1	0	0	0	BBD3.00	[M]Parasympathetic paraganglioma	Jan-11
101429	1	0	0	0	BBj0.11	"[M]Lymphogranuloma, malignant"	Jan-11
101530	9	0	0	0	B616z00	"Hodgkin's disease, lymphocytic depletion NOS"	Jan-11
101606	1	0	0	0	B662.00	Subacute monocytic leukaemia	Feb-11
101608	7	0	0	0	B4A1100	Malignant neoplasm of ureteropelvic junction	Feb-11
101668	4	0	0	0	Byu5400	"[X]Malignant neoplasm/peripheral nerves of trunk,unspecified"	Feb-11
101707	7	0	0	0	B001z00	"Malignant neoplasm of lower lip, vermilion border NOS"	Feb-11
101715	1	0	0	0	B616700	"Hodgkin's disease, lymphocytic depletion of spleen"	Feb-11
101753	1	0	0	0	B03y.00	Malignant neoplasm of other sites of gum	Feb-11
101778	10	0	0	0	B442.00	Malignant neoplasm of broad ligament	Feb-11
101805	2	0	0	0	B507000	Malignant neoplasm of lacrimal sac	Feb-11
101885	4	0	0	0	B241400	Mesothelioma of pericardium	Mar-11
101923	1	0	0	0	BBJ1.11	[M]Fibroliposarcoma	Mar-11
101988	1	0	0	0	B060100	Malignant neoplasm of palatine tonsil	Apr-11
102102	1	0	0	0	PJ12.11	Partial trisomy 13 in Patau's syndrome	Apr-11
102117	1	0	0	0	AyuC300	[X]HIV disease resulting in multiple infections	Apr-11
102142	2	0	0	0	B013000	Malignant neoplasm of anterior 2/3 of tongue ventral surface	Apr-11
102145	3	0	0	0	B322100	Malignant melanoma of external auditory meatus	Apr-11
102151	1	0	0	0	B060200	Malignant neoplasm of overlapping lesion of tonsil	Apr-11
102171	4	0	0	0	C304400	Sulphite oxidase deficiency	Apr-11
102205	2	0	0	0	B072z00	Malignant neoplasm of lateral wall of nasopharynx NOS	May-11

For peer review only - http://bmjopen.bmj.com/site/about/guidelines.xhtml

102252	2	0	0	0	AyuCC00	[X]HIV disease resulting in other specified conditions	May-11
102338	7	0	0	0	F21y600	Vanishing white matter disease	Jun-11
102593	1	0	0	0	BB96.00	[M]Noninfiltrating intraductal papillary adenocarcinoma	Jul-11
102594	553	1	0	0	B627E00	Diffuse large B-cell lymphoma	Jul-11
102628	P104.00	2	0	0	0	Spina bifida with hydrocephalus of late onset	Jul-11
102688	3	0	0	0	ByuD400	[X]Other malignant immunoproliferative diseases	Aug-11
102783	1	0	0	0	B651200	Chronic neutrophilic leukaemia	Aug-11
102848	1	0	0	0	D200200	Constitutional aplastic anaemia with malformation	Aug-11
102921	3	0	0	0	F10y200	PEHO syndrome	Sep-11
102922	1	0	0	0	C370800	Cystic fibrosis related cirrhosis	Sep-11
102949	6	0	0	0	B312000	Malignant neoplasm of connective and soft tissue of hip	Sep-11
102980	1	0	0	0	P511200	Incomplete great vessel transposition	Sep-11
103017	1	0	0	0	PG51200	Osteogenesis imperfecta - unclassifiable	Sep-11
103034	2	0	0	0	BB5j500	"[M]Endometrioid adenofibroma, malignant"	Sep-11
103139	4	0	0	0	C375400	"Mucopolysaccharidosis, type IV"	Oct-11
103224	9	0	0	0	C370500	Cystic fibrosis with distal intestinal obstruction syndrome	Oct-11
103245	1	0	0	0	B601700	Lymphosarcoma of spleen	Oct-11
103284	1	0	0	0	P102300	Lumbar spina bifida with hydrocephalus - open	Nov-11
103354	2	0	0	0	B308z00	Malignant neoplasm of short bones of leg NOS	Dec-11
103527	P202.00	1	0	0	0	Hydromeningocele - cranial	Jan-12
103582	53	0	0	0	PJ33600	Chromosome 22q11 deletion syndrome	Jan-12
103645	1	0	0	0	B66yz00	Other monocytic leukaemia NOS	Jan-12

For peer review only - http://bmjopen.bmj.com/site/about/guidelines.xhtml

103646	1	0	0	0	PG52200	Osteopetrosis - tarda type	Jan-12
103708	1	0	0	0	BBJ6.00	[M]Round cell liposarcoma	Feb-12
103722	54	1	0	0	F390500	Congenital myopathy	Feb-12
103796	2	0	0	0	B051.00	Malignant neoplasm of vestibule of mouth	Feb-12
103873	1	0	0	0	PJ20.00	"Trisomy 18, meiotic nondisjunction"	Mar-12
103883	1	0	0	0	BBc9100	"[M]Retinoblastoma, undifferentiated type"	Mar-12
103891	1	0	0	0	C375.13	Hurler's syndrome	Mar-12
103900	3	0	0	0	B626000	Mast cell malignancy of unspecified site	Mar-12
103946	2	0	0	0	B220100	Malignant neoplasm of mucosa of trachea	Apr-12
103995	2	0	0	0	B545z00	Malignant neoplasm of aortic body or paraganglia NOS	May-12
104025	1	0	0	0	B337600	Malignant neoplasm of skin of heel	May-12
104054	4	1	0	0	PKyL.00	FG syndrome	May-12
104128	6	0	0	0	B31z000	Kaposi's sarcoma of soft tissue	May-12
104134	1	0	0	0	AyuC400	[X]HIV disease resulting/other infectious+parasitic diseases	May-12
104147	0	0	0	0	BBQ4.11	[M]Infantile embryonal carcinoma	Jun-12
104152	727	8	0	0	B628.00	Follicular lymphoma	Jun-12
104239	333	0	0	0	Q48F.00	Hypoxic ischaemic encephalopathy of newborn	Jun-12
104291	327	7	0	0	B61..11	Hodgkin lymphoma	Jun-12
104324	123	1	0	0	B595.00	Malignant tumour of unknown origin	Jun-12
104325	121	0	0	0	B640000	B-cell acute lymphoblastic leukaemia	Jun-12
104328	118	2	0	0	B641000	B-cell chronic lymphocytic leukaemia	Jun-12
104386	118	0	0	0	B62F000	Small cell B-cell lymphoma	Jun-12
104391	136	0	0	0	B627.11	Non-Hodgkin lymphoma	Jun-12
104412	8	0	0	0	B62F200	Lymphoblastic (diffuse) lymphoma	Jun-12
104475	1	0	0	0	B692.00	Subacute myelomonocytic leukaemia	Jul-12

For peer review only

104480	1	0	0	0	B58y800	Secondary malignant neoplasm of epididymis and vas deferens	Jul-12
104484	15	0	0	0	B61C.00	Other classical Hodgkin lymphoma	Jul-12
104619	K053.00	13582	0	30	0	Chronic kidney disease stage 3	Jul-12
104620	253	0	0	0	B62F100	Mantle cell lymphoma	Jul-12
104717	1	0	0	0	A789311	HIV disease resulting in Pneumocystis jirovecii pneumonia	Aug-12
104720	17	0	0	0	BBP3.11	[M]Sarcomatoid mesothelioma	Aug-12
104743	1	0	0	0	B613800	"Hodgkin's, lymphocytic-histiocytic pred of multiple sites"	Aug-12
104788	9	1	0	0	B654.00	Acute myeloblastic leukaemia	Aug-12
104790	1	0	0	0	B601800	Lymphosarcoma of lymph nodes of multiple sites	Aug-12
104862	20	0	0	0	B62E300	Cutaneous T-cell lymphoma	Aug-12
104895	16	0	0	0	B617.00	Nodular lymphocyte predominant Hodgkin lymphoma	Aug-12
104934	2	0	0	0	B62Ew00	Other mature T/NK-cell lymphoma	Sep-12
104939	10	0	0	0	B64y500	Adult T-cell lymphoma/leukaemia (HTLV-1-associated)	Sep-12
104963	K054.00	1181	0	14	0	Chronic kidney disease stage 4	Sep-12
104973	1	0	0	0	BB62z00	[M]Apocrine adenoma or adenocarcinoma NOS	Sep-12
104999	2	0	0	0	C375z00	Mucopolysaccharidosis NOS	Sep-12
105020	6	0	0	0	B628300	Follicular lymphoma grade 3a	Sep-12
105035	3	0	0	0	F211.00	Schilder's disease	Oct-12
105038	15	0	0	0	B627G00	Mediastinal (thymic) large B-cell lymphoma	Oct-12
105069	3	0	0	0	B693.00	Juvenile myelomonocytic leukaemia	Oct-12
105073	1	0	0	0	BBN2.00	"[M]Synovial sarcoma, spindle cell type"	Oct-12
105083	13	0	0	0	B62D.00	Histiocytic sarcoma	Oct-12
105085	36	0	0	0	B62E.00	T/NK-cell lymphoma	Oct-12
105095	17	0	0	0	B628100	Follicular lymphoma grade 2	Oct-12

For peer review only - http://bmjopen.bmj.com/site/about/guidelines.xhtml

105151	K055.00	326	0	0	0	Chronic kidney disease stage 5	Nov-12
105166	1	0	0	0	BBDB.11	[M]Glomoid sarcoma	Nov-12
105186	10	0	0	0	P26..00	Disorder of neuronal migration and differentiation	Nov-12
105203	1	0	0	0	B620200	Nodular lymphoma of intrathoracic lymph nodes	Nov-12
105275	1	0	0	0	BBV..13	[M]Periosteal osteogenic sarcoma	Dec-12
105296	7	0	0	0	BBTD200	"[M]Haemangiopericytoma, malignant"	Dec-12
105324	1	0	0	0	A789800	HIV disease resulting in multiple malignant neoplasms	Dec-12
105335	5	0	0	0	B62A.00	Sarcoma of dendritic cells	Dec-12
105375	3	0	0	0	B62E800	Blastic NK-cell lymphoma	Dec-12
105388	97	0	0	0	B498.00	Local recurrence of malignant tumour of urinary bladder	Dec-12
105472	1	0	0	0	B614700	"Hodgkin's disease, nodular sclerosis of spleen"	Dec-12
105475	1	0	0	0	B308.00	Malignant neoplasm of short bones of leg	Dec-12
105488	133	1	0	0	B36..00	Local recurrence of malignant tumour of breast	Dec-12
105559	17	0	0	0	B62E100	"Anaplastic large cell lymphoma, ALK-positive"	Jan-13
105613	3	0	0	0	B161300	Malignant neoplasm of sphincter of Oddi	Jan-13
105636	20	0	0	0	B62E900	Angioimmunoblastic T-cell lymphoma	Jan-13
105679	24	1	0	0	F25G.11	Dravet syndrome	Jan-13
105709	3	0	0	0	B62E600	Enteropathy-associated T-cell lymphoma	Jan-13
105797	1	0	0	0	B304100	Malignant neoplasm of acromion	Feb-13
105841	16	0	0	0	B618.00	Nodular sclerosis classical Hodgkin lymphoma	Mar-13
105889	15	0	0	0	B628000	Follicular lymphoma grade 1	Mar-13
105925	1	0	0	0	B62E700	Subcutaneous panniculitic T-cell lymphoma	Apr-13
105944	1	0	0	0	BBK3300	[M]Mixed cell rhabdomyosarcoma	Apr-13

For peer review only - http://bmjopen.bmj.com/site/about/guidelines.xhtml

105955	12	0	0	0	B62E200	"Anaplastic large cell lymphoma, ALK-negative"	Apr-13
105956	9	0	0	0	C315200	Kearns-Sayre syndrome	Apr-13
105957	6	0	0	0	B651100	"Chronic myeloid leukaemia, BCR/ABL positive"	Apr-13
106012	8	0	0	0	Q48F.11	Perinatal hypoxic - ischaemic encephalopathy	Apr-13
106063	2	0	0	0	B628700	Other types of follicular lymphoma	May-13
106069	1	0	0	0	B305.11	Malignant neoplasm of carpal bones	May-13
106114	3	0	0	0	PJ51400	Trisomy 9p syndrome	May-13
106134	1	0	0	0	BBdB.00	[M]Meningeal sarcomatosis	May-13
106137	1	0	0	0	BBh..00	[M]Reticulosarcomas	May-13
106194	1	0	0	0	B231.00	Malignant neoplasm of visceral pleura	May-13
106197	1	0	0	0	BBr7000	[M]Basophilic leukaemia	May-13
106349	3	0	0	0	B61z.11	Hodgkin lymphoma NOS	Jun-13
106432	200	0	0	0	C370900	Exacerbation of cystic fibrosis	Jun-13
106483	1	0	0	0	BBr6200	[M]Subacute myeloid leukaemia	Aug-13
106519	79	0	0	0	BB1P.00	[M]Non-small cell carcinoma	Aug-13
106569	2	0	0	0	B500200	Malignant neoplasm of crystalline lens	Aug-13
106579	1	0	0	0	Pyu0400	[X]Unspecified spina bifida with hydrocephalus	Aug-13
106597	3	0	0	0	B61B.00	Lymphocyte-rich classical Hodgkin lymphoma	Aug-13
106858	6	0	0	0	1JW..00	Suspected cystic fibrosis	Aug-13
106867	4	0	0	0	B62F.11	Non-follicular lymphoma	Aug-13
106884	6	0	0	0	B62F.00	Nonfollicular lymphoma	Aug-13
106911	1	0	0	0	B613700	"Hodgkin's, lymphocytic-histiocytic predominance of spleen"	Aug-13
106915	1	0	0	0	B073100	Malignant neoplasm of nasopharyngeal soft palate surface	Aug-13
106924	8	0	0	0	B641200	Clinical stage B chronic lymphocytic leukaemia	Aug-13

For peer review only - http://bmjopen.bmj.com/site/about/guidelines.xhtml

106926	6	0	0	0	PKyP.11	Wolfram syndrome	Aug-13
106969	2	0	0	0	B628500	Diffuse follicle centre lymphoma	Sep-13
106970	2	0	0	0	BBk3.00	"[M]Malig lymphoma, lymphocytic, well differentiated,nodular"	Sep-13
107017	9	0	0	0	B641011	Chronic lymphocytic leukaemia of B-cell type	Sep-13
107032	1	0	0	0	B616800	Hodgkin's lymphocytic depletion lymph nodes multiple sites	Sep-13
107052	44	0	0	0	B641100	Clinical stage A chronic lymphocytic leukaemia	Oct-13
107126	3	0	0	0	B553200	Malignant neoplasm of sacrococcygeal region	Oct-13
107144	P114100	3	0	0	0	Cervical meningomyelocele	Oct-13
107162	1	0	0	0	PJ22.11	Partial trisomy 18 in Edward's syndrome	Oct-13
107163	4	0	0	0	B641300	Clinical stage C chronic lymphocytic leukaemia	Oct-13
107166	5	0	0	0	B628200	Follicular lymphoma grade 3	Oct-13
107236	9	0	0	0	B651300	"Atypical chronic myeloid leukaemia, BCR/ABL negative"	Nov-13
107258	1	0	0	0	B011100	Malignant neoplasm of midline of tongue	Nov-13
107299	1	0	0	0	BB5D700	[M]Combined hepatocellular carcinoma and cholangiocarcinoma	Nov-13
107377	P116100	1	0	0	0	Cervical myelocystocele	Dec-13
107643	5	0	0	0	B64y400	T-cell prolymphocytic leukaemia	Jan-14
107773	1	0	0	0	BBr8z00	[M]Eosinophilic leukaemia NOS	Mar-14
107804	1	0	0	0	B61z300	Hodgkin's disease NOS of intra-abdominal lymph nodes	Mar-14
107807	1	0	0	0	AyuC100	[X]HIV disease resulting in other viral infections	Mar-14
107840	1	0	0	0	C308100	Multiple acyl-CoA dehydrogenase deficiencies	Mar-14

For peer review only - http://bmjopen.bmj.com/site/about/guidelines.xhtml

107878	1	0	0	0	B213200	Malignant neoplasm of cuneiform cartilage	Apr-14
107884	1	0	0	0	BBba000	[M]Peripheral neuroectodermal tumour	Apr-14
107916	1	0	0	0	B201000	Malignant neoplasm of auditory (Eustachian) tube	Apr-14
107949	2	0	0	0	B62E500	Hepatosplenic T-cell lymphoma	Apr-14
107973	7	0	0	0	B628400	Follicular lymphoma grade 3b	May-14
108021	1	0	0	0	790Jz00	Other repair of transposition of great arteries NOS	May-14
108041	17	1	0	0	C308200	X-linked adrenoleucodystrophy	May-14
108054	4	1	0	0	A789511	HIV disease resulting in Kaposi sarcoma	May-14
108131	2	0	0	0	F146.00	Early onset cerebellar ataxia with hypogonadism	Jun-14
108182	1	0	0	0	B627400	Diffuse non-Hodgkin's small cleaved cell (diffuse) lymphoma	Jun-14
108316	2	0	0	0	BBrAz00	[M]Miscellaneous leukaemia NOS	Jul-14
108363	71	1	0	0	B509.00	Malignant melanoma of eye	Jul-14
108389	1	0	0	0	B310500	Malignant neoplasm soft tissues of cervical spine	Jul-14
108424	5	0	0	0	B663.00	Acute monoblastic leukaemia	Jul-14
108591	4	0	0	0	K01w100	Drash syndrome	Sep-14
108623	2	0	0	0	C372.11	Lesch - Nyhan syndrome	Sep-14
108638	1	0	0	0	B305A00	Malignant neoplasm of third metacarpal bone	Sep-14
108652	1	0	0	0	PyuAD00	[X]Li-Fraumeni syndrome	Sep-14
108656	3	1	0	0	B64y300	B-cell prolymphocytic leukaemia	Sep-14
108667	2	0	0	0	B1z1000	Angiosarcoma of spleen	Sep-14
108682	1	0	0	0	BBgJ.11	[M]Germinoblastic sarcoma NOS	Oct-14
108715	1	0	0	0	B66..11	Histiocytic leukaemia	Oct-14
108719	6	0	0	0	B628600	Cutaneous follicle centre lymphoma	Oct-14
108773	F103z00	1	0	0	0	Cerebral degeneration in disease NOS	Oct-14

For peer review only - http://bmjopen.bmj.com/site/about/guidelines.xhtml

108775	1	0	0	0	B619.00	Mixed cellularity classical Hodgkin lymphoma	Oct-14
108886	1	0	0	0	B615500	Hodgkin's mixed cellularity of lymph nodes inguinal and leg	Nov-14
108922	1	0	0	0	K01w112	Wilms' tumour + nephrotic syndrome + pseudohermaphroditism	Dec-14
108947	1	0	0	0	PKyz.11	Cockayne's syndrome	Dec-14
108964	2	0	0	0	BBr6900	[M]Juvenile myelomonocytic leukaemia	Dec-14
109002	2	0	0	0	B325400	Malignant melanoma of perianal skin	Dec-14
109110	5	0	0	0	BBD9.11	[M]Chromaffin paraganglioma	Jan-15
109223	1	0	0	0	PJ50311	Trisomy 9 Mosaic Syndrome	Jan-15
109243	1	0	0	0	P100100	Cervical spina bifida with hydrocephalus	Feb-15
109243	P100100	1	0	0	0	Cervical spina bifida with hydrocephalus	Feb-15
109288	11	1	0	0	A411000	Sporadic Creutzfeldt-Jakob disease	Feb-15
109394	1	0	0	0	P00y.00	Other specified anencephalus	Mar-15
109473	0	0	0	0	B521200	Malignant neoplasm of cerebral pia mater	Apr-15
109608	2	0	0	0	C300A00	Congenital Fanconi syndrome	May-15
109657	1Z1Y.00	92	0	0	0	CKD with GFR category G3b & albuminuria category A2	May-15
109714	1	0	0	0	ByuDD00	[X]Oth and unspecif peripheral & cutaneous T-cell lymphomas	Jun-15
109745	2	0	0	0	B32G.00	Malignant melanoma stage IV M1a	Jun-15
109780	1	0	0	0	B62E400	"Extranodal NK/T-cell lymphoma, nasal type"	Jun-15
109804	1Z1T.00	390	0	0	0	CKD with GFR category G3a & albuminuria category A1	Jun-15
109805	1Z1V.00	115	0	1	0	CKD with GFR category G3a & albuminuria category A2	Jun-15
109827	4	0	0	0	B329.00	Malignant melanoma stage IB	Jul-15
109868	2	0	0	0	C327300	Wolman disease	Jul-15
109904	1Z1b.00	26	0	0	0	CKD with GFR category G4 & albuminuria category A2	Jul-15

For peer review only - http://bmjopen.bmj.com/site/about/guidelines.xhtml

109905	1Z1W.00	25	0	0	0	CKD with GFR category G3a & albuminuria category A3	Jul-15
109963	1Z1X.00	160	0	0	0	CKD with GFR category G3b & albuminuria category A1	Jul-15
109980	1Z1a.00	36	0	0	0	CKD with GFR category G4 & albuminuria category A1	Aug-15
109981	1Z1e.00	1	0	0	0	CKD with GFR category G5 & albuminuria category A2	Aug-15
109990	1Z1Z.00	28	0	0	0	CKD with GFR category G3b & albuminuria category A3	Aug-15
110058	2	0	0	0	ZV10613	[V]Personal history of myeloid leukaemia	Sep-15
110133	1Z1d.00	2	0	0	0	CKD with GFR category G5 & albuminuria category A1	Sep-15
110139	4	0	0	0	B32A.00	Malignant melanoma stage IIA	Sep-15
110147	1	0	0	0	BB5D711	[M]Hepatocholangiocarcinoma	Sep-15
110169	1	0	0	0	PKyD.00	Nicolaides-Baraitser syndrome	Oct-15
110180	1	0	0	0	B32B.00	Malignant melanoma stage IIB	Oct-15
110349	1	0	0	0	BBr3z00	[M]Plasma cell leukaemia NOS	Dec-15
110433	1	0	0	0	B574100	Secondary malignant neoplasm of jejunum	Jan-16
110454	1	0	0	0	C370700	Liver disease due to cystic fibrosis	Jan-16
110467	1Z1f.00	9	0	0	0	CKD with GFR category G5 & albuminuria category A3	Jan-16
110483	2	0	0	0	B32H.00	Malignant melanoma stage IV M1b	Jan-16
110563	1	0	0	0	B616500	Hodgkin's lymphocytic depletion lymph nodes inguinal and leg	Feb-16
110626	1Z1c.00	13	0	0	0	CKD with GFR category G4 & albuminuria category A3	Mar-16
110749	2	0	0	0	K01w200	Congenital nephrotic syndrome with focal glomerulosclerosis	Apr-16
110766	2	0	0	0	B521000	Malignant neoplasm of cerebral dura mater	May-16

For peer review only - http://bmjopen.bmj.com/site/about/guidelines.xhtml

110775	1	0	0	0	B151100	Malignant neoplasm of interlobular biliary canals	May-16
110833	2	0	0	0	PJ50400	Trisomy 10	May-16
110838	1	0	0	0	B676.00	Acute erythroid leukaemia	May-16
110903	1	0	0	0	B623800	Malignant histiocytosis of lymph nodes of multiple sites	Jun-16
110961	1	0	0	0	B32F.00	Malignant melanoma stage IIIC	Jun-16
110993	1	0	0	0	B305800	Malignant neoplasm of first metacarpal bone	Jul-16
111036	1	0	0	0	C375.18	Sanfilippo's syndrome	Jul-16
111077	2	0	0	0	C300800	Juvenile nephropathic cystinosis	Jul-16
111079	4	0	0	0	B328.00	Malignant melanoma stage IA	Jul-16
111113	1	0	0	0	BBj3.00	"[M]Hodgkin's disease, lymphocytic depletion NOS"	Jul-16
111120	3	0	0	0	PJ33113	18q deletion syndrome	Jul-16
111162	2	0	0	0	B32J.00	Malignant melanoma stage IV M1c	Jul-16
111172	1	0	0	0	BB5R300	"[M]Carcinoid tumour, argentaffin, malignant"	Aug-16
111238	1	0	0	0	BB5dz00	[M]Mixed cell adenoma or adenocarcinoma NOS	Aug-16
111289	1	0	0	0	B002000	"Malignant neoplasm of upper lip, buccal aspect"	Sep-16
111311	1	0	0	0	B317.00	"Malignant neoplasm, overlap lesion connective & soft tissue"	Sep-16
111370	1	0	0	0	K01wz00	Congenital nephrotic syndrome NOS	Oct-16
111413	2	0	0	0	B32C.00	Malignant melanoma stage IIC	Oct-16
111426	1	0	0	0	B308900	Malignant neoplasm of second metatarsal bone	Oct-16
46733	D104700	148	0	0	0	Beta major thalassaemia	Feb-09
21643	D104811	655	0	8	2	Beta thalassaemia	Feb-09
45151	D104011	11	0	0	0	Thalassaemia major - Cooley's anaemia	Feb-09
31405	D104000	103	0	5	0	Thalassaemia major NEC	Feb-09

For peer review only - http://bmjopen.bmj.com/site/about/guidelines.xhtml

Appendix 2 ICD 10 Codes to identify a child with a Life-limiting condition

A17

A81.0

A81.1

B20

B21

B22

B23

B24

C00

C01

C02

C03

C04

C05

C06

C07

C08

C09

C10

For peer review only - http://bmjopen.bmj.com/site/about/guidelines.xhtml

C11

C12

C13

C14

C15

C16

C17

C18

C19

C20

C21

C22

C23

C24

C25

C26

C30

C31

C32

C33

For peer review only

For peer review only - http://bmjopen.bmj.com/site/about/guidelines.xhtml

C34

C35

C36

C37

C38

C39

C40

C41

C43

C44

C45

C46

C47

C48

C49

C50

C51

C52

C53

C54

For peer review only - http://bmjopen.bmj.com/site/about/guidelines.xhtml

C55

C56

C57

C58

C59

C60

C61

C62

C63

C64

C65

C66

C67

C68

C69

C70

C71

C72

C73

C74

For peer review only

For peer review only - http://bmjopen.bmj.com/site/about/guidelines.xhtml

C75

C76

C77

C78

C79

C80

C81

C82

C83

C84

C85

C86

C87

C88

C89

C90

C91

C92

C93

C94

For peer review only

C95

C96

C97

D61.9

D70

D76.1

D81

D82.1

D83

D89.1

E31.0

E34.8

E70.2

E71

E72

E74

E75

E76

E77

E79.1

For peer review only

For peer review only - http://bmjopen.bmj.com/site/about/guidelines.xhtml

E83.0

E84

E88.0

E88.1

"F80.3

"

F84.2

G10

G11.1

G11.3

G12

G20

G23.0

G23.8

G31.8

G31.9

G35

G40.4

G40.5

G60.0

For peer review only - http://bmjopen.bmj.com/site/about/guidelines.xhtml

G60.1

G70.2

G70.9

G71.0

G71.1

G71.2

G71.3

G80.0

G80.8

G82.3

G82.4

G82.5

G93.4

G93.6

G93.7

H11.1

H49.8

H35.5

I21

I27.0

For peer review only

For peer review only - http://bmjopen.bmj.com/site/about/guidelines.xhtml

I42

I61.3

I81

J84.1

J96

J98.4

K55.0

K55.9

K72

K74

K76.5

K86.8

M31.3

M32.1

M89.5

N17

N18

N19

N25.8

P10.1

For peer review only - http://bmjopen.bmj.com/site/about/guidelines.xhtml

P11.2

P21.0

P28.5

P29.0

P29.3

P35.0

P35.1

P35.8

P37.1

Q00.0

Q01

Q03.1

Q03.9

Q04.0

Q04.2

Q04.3

Q04.4

Q04.6

Q04.9

Q21.8

For peer review only

For peer review only - http://bmjopen.bmj.com/site/about/guidelines.xhtml

Q22.0

Q22.1

Q22.4

Q22.5

Q22.6

Q23.0

Q23.4

Q23.9

Q25.4

Q39.6

Q41.0

Q41.9

Q43.7

Q44.2

Q74.8

Q44.5

Q44.7

Q60.1

Q60.6

Q78.0

For peer review only

Q78.5

Q79.2

Q79.3

Q80.4

Q81

Q82.1

Q82.4

Q85.8

Q86.0

Q93.2

Q93.3

Q93.4

Q93.5

Q93.8

Q95.2

T86.0

T86.2

Appendix 3 Read codes to identify a child with a chronic, not life-limiting condition

5600	F172.00	2402	0	130	0	[X] Horners syndrome	Feb-09

24753	ZS91.12	143	0	5	0	[X]Attention deficit disorder	Feb-09
26285	Eu9y700	2865	0	197	0	[X]Attention deficit disorder	Feb-09
34929	Eu50100	402	0	11	0	[X]Atypical anorexia nervosa	Feb-09
24044	Eu84100	418	0	5	0	[X]Atypical autism	Feb-09
33863	Eu50300	258	0	6	0	[X]Atypical bulimia nervosa	Feb-09
24062	Eu84111	16	0	0	0	[X]Atypical childhood psychosis	Feb-09
91547	Eu20311	4	0	0	0	[X]Atypical schizophrenia	Feb-09
9982	Eu84011	13172	0	379	0	[X]Autistic disorder	Feb-09
51375	Eu84511	9	0	0	0	[X]Autistic psychopathy	Feb-09
42941	Eu84z11	2488	0	11	0	[X]Autistic spectrum disorder	Feb-09
23713	Eu31400	117	0	7	0	"[X]Bipol aff disord, curr epis sev depress, no psychot symp"	Feb-09
28277	Eu31200	153	0	1	0	[X]Bipolar affect disorder cur epi manic with psychotic symp	Feb-09
26299	Eu31100	65	0	0	0	[X]Bipolar affect disorder cur epi manic wout psychotic symp	Feb-09
104065	Eu31800	15	0	0	0	[X]Bipolar affective disorder type I	May-12
103915	Eu31900	262	0	1	0	[X]Bipolar affective disorder type II	Mar-12
44693	Eu31600	124	0	4	0	"[X]Bipolar affective disorder, current episode mixed"	Feb-09
27584	Eu31700	1499	0	1	0	"[X]Bipolar affective disorder, currently in remission"	Feb-09
33751	Eu31z00	624	0	4	0	"[X]Bipolar affective disorder, unspecified"	Feb-09
9581	Eu50200	10720	0	551	4	[X]Bulimia nervosa	Feb-09
72688	Myu1.00	37	0	0	0	[X]Bullous disorders	Feb-09
3637	Eu84000	978	0	21	0	[X]Childhood autism	Feb-09
53820	PyuA.00	12	0	0	0	"[X]Chromosomal abnormalities, not elswhere classified"	Feb-09
543	Eu32z11	738902	0	19968	0	[X]Depression NOS	Feb-09
3291	Eu32z12	16570	0	445	0	[X]Depressive disorder NOS	Feb-09

For peer review only - http://bmjopen.bmj.com/site/about/guidelines.xhtml

6514	Eu80z00	399	0	3	0	[X]Developmental disorder of speech and language unspecified	Feb-09
52212	Cyu2.00	193	0	1	0	[X]Diabetes mellitus	Feb-09
36946	Eu50z00	2380	0	108	0	"[X]Eating disorder, unspecified"	Feb-09
100899	Eu85.00	2066	0	20	0	[X]Global developmental delay	Nov-10
33505	Eu90100	91	0	1	0	[X]Hyperkinetic conduct disorder	Feb-09
45799	Eu90111	8	0	2	0	[X]Hyperkinetic disorder associated with conduct disorder	Feb-09
50015	Eu90z00	79	0	3	0	"[X]Hyperkinetic disorder, unspecified"	Feb-09
97421	Eu90z11	0	0	0	0	[X]Hyperkinetic reaction of childhood or adolescence NOS	Aug-09
96770	Eu90z12	4	0	0	0	[X]Hyperkinetic syndrome NOS	Apr-09
50337	Eu84012	30	0	0	0	[X]Infantile autism	Feb-09
4477	Eu81z11	15741	0	569	0	[X]Learning disability NOS	Feb-09
98346	Eu32500	512	0	2	0	"[X]Major depression, mild"	Jan-10
98252	Eu32600	1500	0	11	0	"[X]Major depression, moderately severe"	Dec-09
98417	Eu32800	323	0	3	0	"[X]Major depression, severe with psychotic symptoms"	Jan-10
98414	Eu32700	1948	0	17	0	"[X]Major depression, severe without psychotic symptoms"	Jan-10
28962	Eu7..00	394	0	1	0	[X]Mental retardation	Feb-09
34174	Eu84112	102	0	2	0	[X]Mental retardation with autistic features	Feb-09
98342	Eu81400	5806	0	21	0	[X]Moderate learning disability	Jan-10
98293	Eu81500	3549	0	24	0	[X]Severe learning disability	Dec-09
36143	Eu72.00	2449	0	1	0	[X]Severe mental retardation	Feb-09
22048	PKy6500	68	0	2	0	Aarskog syndrome	Feb-09
102328	PJyy.11	1	0	0	0	Absence of sex chromosome	Jun-11
72655	P74z100	3	0	0	0	Absence of superior vena cava	Feb-09
8097	2828	7568	0	1399	0	Absence seizure	Feb-09

For peer review only - http://bmjopen.bmj.com/site/about/guidelines.xhtml

71109	PK11.00	3	0	0	0	Absent adrenal gland	Feb-09
25089	PKyz100	4	0	0	0	Acardia	Feb-09
57804	PG41.13	15	0	0	0	Achondroplastic dwarf	Feb-09
69916	C152100	3	0	0	0	Acquired adrenogenital syndrome	Feb-09
91774	G341300	19	0	0	0	Acquired atrioventricular fistula of heart	Feb-09
101375	SN51000	47	0	1	0	Acquired C1 esterase inhibitor deficiency	Jan-11
46025	D307.00	37	0	2	0	Acquired coagulation factor deficiency	Feb-09
37587	D307z00	25	0	0	0	Acquired coagulation factor deficiency NOS	Feb-09
63195	J690100	67	0	1	0	Acquired coeliac disease	Feb-09
4675	F113.00	811	0	60	0	Acquired communicating hydrocephalus	Feb-09
31667	M145300	230	0	16	0	Acquired epidermolysis bullosa	Feb-09
49889	ZS82.00	2	0	0	0	Acquired epileptic aphasia	Feb-09
46546	D307200	15	0	0	0	Acquired factor II deficiency	Feb-09
107206	C300600	5	0	1	0	Acquired Fanconi syndrome	Nov-13
50495	D112z12	8	0	0	0	Acquired haemolytic anaemia with haemoglobinuria NEC	Feb-09
27771	D11..00	152	0	9	1	Acquired haemolytic anaemias	Feb-09
105061	C353500	4	0	0	0	Acquired hypophosphataemia	Oct-12
103789	D307212	0	0	0	0	Acquired hypoprothrombinaemia	Feb-12
3290	C04..00	160727	0	878	7	Acquired hypothyroidism	Feb-09
23639	M211600	136	0	4	0	Acquired ichthyosis	Feb-09
23770	A788.00	1182	0	53	8	Acquired immune deficiency syndrome	Feb-09
110481	K081000	1	0	0	0	Acquired nephrogenic diabetes insipidus	Jan-16
63815	J106500	9	0	0	0	Acquired oesophagocoele	Feb-09
43824	D307211	44	0	2	0	Acquired prothrombin deficiency	Feb-09

For peer review only - http://bmjopen.bmj.com/site/about/guidelines.xhtml

69269	D20X.00	7	0	0	0	"Acquired pure red cell aplasia, unspecified"	Feb-09
104079	K132.11	476	0	127	0	Acquired renal cystic disease	May-12
41699	D210100	8	0	0	0	Acquired sideroblastic anaemia	Feb-09
29933	D11z000	116	0	2	0	Acquired spherocytosis	Feb-09
41840	PF55000	53	0	3	0	Acrocephalosyndactyly (Apert)	Feb-09
48272	PG01.00	8	0	0	0	Acrocephaly	Feb-09
5316	C130200	2971	0	245	1	Acromegaly	Feb-09
62353	PF5E200	32	0	0	0	Acrosyndactyly	Feb-09
11147	C134411	141	0	5	0	ACTH deficiency	Feb-09
43641	C153.11	4	0	0	0	ACTH overproduction	Feb-09
38968	P6z2.00	48	0	0	0	Acyanotic congenital heart disease NOS	Feb-09
59166	PF47.11	8	0	1	0	Adactyly	Feb-09
5524	PG51.13	7	0	0	0	Adair-Dighton syndrome	Feb-09
4042	C154011	239	0	38	0	Addisonian crisis	Feb-09
12227	C154600	668	0	18	0	Addisonian crisis	Feb-09
2813	D010.11	53	0	4	0	Addison's anaemia	Feb-09
4481	C154100	5819	0	428	2	Addison's disease	Feb-09
110474	44lz.00	0	0	0	1	Additional biochemical ratios	Jan-16
9665	PJz1.00	9	0	0	0	Additional chromosome NOS	Feb-09
72804	C392600	3	0	0	0	Adenosine deaminase deficiency	Feb-09
57552	C30yy11	10	0	0	0	Adenosine-deaminase deficiency	Feb-09
1523	F4K4600	2858	0	130	0	Adie's pupil syndrome	Feb-09
42873	C154012	66	0	3	0	Adrenal crisis	Feb-09
41542	C15z.00	215	0	7	0	Adrenal gland disorder NOS	Feb-09
28896	C154z11	74	0	2	0	Adrenal hypofunction	Feb-09
20786	C154z12	444	0	14	0	Adrenal insufficiency NEC	Feb-09
73529	C155000	3	0	0	0	Adrenal medullary insufficiency	Feb-09
32642	C153.12	39	0	0	0	Adrenocortical hyperfunction	Feb-09
57321	C152z00	7	0	0	0	Adrenogenital disorder NOS	Feb-09
31446	C152811	123	0	4	0	Adrenogenital syndrome NOS	Feb-09

For peer review only - http://bmjopen.bmj.com/site/about/guidelines.xhtml

35682	C390.11	148	0	8	0	Agammaglobulinaemia	Feb-09
62598	C390900	2	0	0	0	Agammaglobulinaemia NEC	Feb-09
65511	PB33.11	1	0	0	0	Aganglionic macrocolon	Feb-09
94813	PB3..11	1	0	0	0	Aganglionosis	Feb-09
23457	P220.00	121	0	11	0	"Agenesis of brain, part unspecified"	Feb-09
54877	P22A011	4	0	0	0	Agenesis of cerebellum	Feb-09
40777	P227000	19	0	0	0	Agenesis of cerebrum	Feb-09
67919	P362000	10	0	2	0	Agenesis of cilia	Feb-09
39486	P228011	211	0	2	0	Agenesis of corpus callosum	Feb-09
60930	PF37.00	10	0	0	0	Agenesis of fibula	Feb-09
47576	PB63011	9	0	0	0	Agenesis of gallbladder	Feb-09
56350	PF21z11	14	0	0	0	Agenesis of hand	Feb-09
59444	P853.00	17	0	0	0	Agenesis of lung	Feb-09
93911	P2xz000	3	0	0	0	Agenesis of nerve NEC	Feb-09
67476	P2x0.00	6	0	0	0	"Agenesis of nerve, unspecified"	Feb-09
58710	P22z.12	6	0	0	0	Agenesis of part of brain NEC	Feb-09
53723	P223.00	4	0	0	0	Agyria	Feb-09
40029	PB63500	118	0	2	0	Alagille syndrome	Feb-09
66720	C30y000	24	0	0	2	Alaninaemia	Feb-09
50325	PG52.11	8	0	0	0	Albers - Schonberg syndrome	Feb-09
15624	C302700	956	0	41	0	Albinism	Feb-09
53954	C302.11	25	0	0	0	Albinism	Feb-09
31388	C302712	57	0	4	0	Albino	Feb-09
30601	PG5y011	42	0	3	0	Albright-McCune-Sternberg syndrome	Feb-09
57407	PG5y012	15	0	4	0	Albright's polyostotic dysplasia	Feb-09
15046	PG5y000	35	0	0	0	Albright-Sternberg syndrome	Feb-09
52688	C151.11	70	0	4	0	Aldosteronism	Feb-09
2290	H330.11	13983	0	430	3	Allergic asthma	Feb-09
21232	H33zz12	182	0	0	0	Allergic asthma NEC	Feb-09
1037	M240.00	107630	0	9254	24	Alopecia	Feb-09

For peer review only - http://bmjopen.bmj.com/site/about/guidelines.xhtml

685	M240100	44959	0	2328	2	Alopecia areata	Feb-09
43109	M240900	25	0	1	0	Alopecia disseminata	Feb-09
50780	M240700	3	0	0	0	Alopecia febrilis	Feb-09
48735	M240C00	50	0	3	0	Alopecia follicularis	Feb-09
40947	M240J00	8	0	0	0	Alopecia hereditaria	Feb-09
48719	M240H00	35	0	0	0	Alopecia seborrhoeica	Feb-09
52294	M240600	24	0	0	0	Alopecia senilis	Feb-09
976	M240000	5590	0	250	1	Alopecia unspecified	Feb-09
8866	D104300	848	0	19	0	Alpha thalassaemia	Feb-09
12235	D104400	3655	0	30	114	Alpha trait thalassaemia	Feb-09
3019	C376200	2675	0	117	64	Alpha-1-antitrypsin deficiency	Feb-09
25589	C376100	167	0	8	9	Alpha-1-antitrypsin hepatitis	Feb-09
71522	C327400	14	0	2	0	Alpha-galactosidase A deficiency	Feb-09
24395	PKy9000	592	0	32	0	Alport's syndrome	Feb-09
64601	D014000	3	0	0	0	Amino-acid deficiency anaemia	Feb-09
46357	C300z00	3	0	4	4	Amino-acid transport disorder NOS	Feb-09
32466	46T6.11	18	0	0	11	Aminoaciduria	Feb-09
107595	C373H00	13	0	0	0	Amyloid A amyloidosis	Jan-14
105651	G558400	14	0	0	0	Amyloid cardiomyopathy	Jan-13
30667	G557000	139	0	4	0	Amyloid heart disease	Feb-09
36697	F38y.11	20	0	0	0	Amyotonia congenita	Feb-09
31439	N232000	170	0	5	0	Amyotrophia NOS	Feb-09
57769	PGyy000	5	0	2	2	Amyotrophica congenita	Feb-09
31944	C310412	108	0	2	0	Andersen's disease	Feb-09
24660	C327413	52	0	1	0	Anderson-Fabry disease	Feb-09
93886	C327412	2	0	0	0	Anderson's disease	Feb-09
49161	C1z5.11	145	0	6	0	Androgen insensitivity syndrome	Feb-09
61407	PJ5z.11	8	0	0	0	Aneuploidy NEC	Feb-09
2184	N100.00	35726	0	2753	12	Ankylosing spondylitis	Feb-09
25292	PK1..00	181	0	19	0	Anomalies of adrenal gland	Feb-09
36064	PK1z.00	23	0	0	0	Anomalies of adrenal gland NOS	Feb-09
28318	P22A.00	132	0	3	0	Anomalies of cerebellum	Feb-09

For peer review only - http://bmjopen.bmj.com/site/about/guidelines.xhtml

39724	P227.00	15	0	1	0	Anomalies of cerebrum	Feb-09
32468	P228.00	143	0	7	0	Anomalies of corpus callosum	Feb-09
31416	PG6..00	171	0	7	0	Anomalies of diaphragm	Feb-09
42684	P74..00	34	0	0	0	Anomalies of great veins	Feb-09
46471	P229.00	17	0	0	0	Anomalies of hypothalamus	Feb-09
30055	PB7z.00	45	0	0	0	Anomalies of pancreas NOS	Feb-09
39588	PK27.00	23	0	3	0	Anomalies of parathyroid gland NEC	Feb-09
32424	PK24.00	191	0	12	1	Anomalies of pituitary gland	Feb-09
50786	P76C.00	34	0	1	0	Anomalies of renal artery NEC	Feb-09
45429	PG1z.00	55	0	1	0	Anomalies of spine NOS	Feb-09
26338	PK0..00	151	0	12	0	Anomalies of spleen	Feb-09
59625	PK0z.00	21	0	0	0	Anomalies of spleen NOS	Feb-09
40030	P72..11	103	0	16	0	Anomalies of the aorta excluding coarction	Feb-09
66465	PG3z.11	3	0	0	0	Anomalies of thoracic cage unspecified	Feb-09
39366	PK28.00	37	0	1	0	Anomalies of thymus	Feb-09
57704	PK26.00	45	0	4	0	Anomalies of thyroglossal duct NEC	Feb-09
10638	PK25.00	271	0	44	0	Anomalies of thyroid gland NEC	Feb-09
55080	PAzz.00	5	0	0	0	Anomalies of upper alimentary tract NOS	Feb-09
30315	PD7..00	55	0	0	0	Anomalies of urachus	Feb-09
56889	PD7z.00	2	0	0	0	Anomalies of urachus NOS	Feb-09
103432	P6z1000	2	0	0	0	Anomalous atrial bands	Dec-11
25147	G567.00	224	0	1	0	Anomalous atrioventricular excitation	Feb-09
42803	G567z00	3	0	0	0	Anomalous atrioventricular excitation NOS	Feb-09
109236	P6z1.00	1	0	0	0	Anomalous bands of heart	Jan-15
31373	P6y4400	12	0	1	0	Anomalous coronary artery communication	Feb-09
67286	P721000	1	0	0	0	Anomalous origin of the aortic arch	Feb-09

For peer review only - http://bmjopen.bmj.com/site/about/guidelines.xhtml

101307	P743.00	3	0	0	0	Anomalous portal vein termination	Jan-11
105445	ZR1N.00	3	0	0	0	Anomalous sentences repetition test	Dec-12
63677	P742.11	5	0	0	0	Anomalous termination of right pulmonary vein	Feb-09
64778	P6z1100	8	0	0	0	Anomalous ventricular bands	Feb-09
62230	P720.00	37	0	1	0	"Anomaly of aorta, unspecified"	Feb-09
42389	P761.00	79	0	7	1	Anomaly of artery NEC	Feb-09
44253	P22Az00	19	0	0	0	Anomaly of cerebellum NOS	Feb-09
111258	P227z00	2	0	0	0	Anomaly of cerebrum NOS	Aug-16
60648	P228z00	9	0	0	0	Anomaly of corpus callosum NOS	Feb-09
45291	P740.00	21	0	1	0	"Anomaly of great veins, unspecified"	Feb-09
63530	P831.00	10	0	0	0	Anomaly of laryngeal and tracheal cartilage	Feb-09
55062	P831z00	7	0	0	0	Anomaly of laryngeal or tracheal cartilage NOS	Feb-09
63453	P860.00	16	0	0	0	"Anomaly of lung, unspecified"	Feb-09
100681	PK27z00	3	0	0	0	Anomaly of parathyroid gland NEC NOS	Sep-10
70576	PK24z00	25	0	0	0	Anomaly of pituitary gland NOS	Feb-09
97401	P76Cz00	4	0	0	0	Anomaly of renal artery NEC NOS	Aug-09
60066	PG10.00	29	0	1	0	"Anomaly of spine, unspecified"	Feb-09
74890	P740000	8	0	0	0	"Anomaly of the pulmonary veins, unspecified"	Feb-09
50886	P740100	30	0	0	0	"Anomaly of the vena cava, unspecified"	Feb-09
48842	PK28z00	7	0	0	0	Anomaly of thymus gland NOS	Feb-09
47467	P831200	50	0	6	0	Anomaly of thyroid cartilage	Feb-09
70581	PK25z00	12	0	0	0	Anomaly of thyroid gland NEC NOS	Feb-09
37358	PA10.00	36	0	3	0	"Anomaly of tongue, unspecified"	Feb-09
31020	P831300	13	0	1	0	Anomaly of tracheal cartilage	Feb-09

For peer review only - http://bmjopen.bmj.com/site/about/guidelines.xhtml

23605	P30..00	108	0	6	1	Anophthalmos	Feb-09
37557	P30z.00	6	0	0	0	Anophthalmos NOS	Feb-09
70742	P300z00	2	0	0	0	Anophthalmos NOS	Feb-09
2135	E271.00	26924	0	1771	0	Anorexia nervosa	Feb-09
21889	F15..00	209	0	11	0	Anterior horn cell disease	Feb-09
58729	F15z.00	3	0	0	0	Anterior horn cell disease NOS	Feb-09
43908	C134z11	28	0	0	0	Anterior pituitary hormone deficiency NEC	Feb-09
43347	G121.11	193	0	7	0	Aortic incompetence - rheumatic	Feb-09
1007	G541200	2670	0	168	0	"Aortic incompetence alone, cause unspecified"	Feb-09
14998	G541000	3238	0	146	0	"Aortic incompetence, non-rheumatic"	Feb-09
58810	G541211	5	0	0	0	"Aortic insufficiency alone, cause unspecified"	Feb-09
47887	G541011	10	0	1	0	"Aortic insufficiency, non-rheumatic"	Feb-09
7963	G121.12	909	0	67	3	Aortic regurgitation - rheumatic	Feb-09
1005	G541212	7183	0	121	0	"Aortic regurgitation alone, cause unspecified"	Feb-09
10187	G541012	4918	0	146	0	"Aortic regurgitation, non-rheumatic"	Feb-09
98277	G71A.00	486	0	21	0	Aortic root dilatation	Dec-09
93699	791C000	14	0	0	0	Aortic root replac us pul val auto ri vent pulm art val cond	Feb-09
71668	791C400	196	0	0	0	Aortic root replacement	Feb-09
70353	791C200	26	0	0	0	Aortic root replacement using homograft	Feb-09
91004	791C300	58	0	1	0	Aortic root replacement using mechanical prosthesis	Feb-09
37405	P50..11	31	0	5	0	Aortic septal defect	Feb-09
45505	P501.00	17	0	0	0	Aortic septal defect	Feb-09

For peer review only - http://bmjopen.bmj.com/site/about/guidelines.xhtml

9591	G541500	27546	0	463	0	Aortic stenosis	Feb-09
2343	G541300	14659	0	932	5	"Aortic stenosis alone, cause unspecified"	Feb-09
999	G541100	18331	0	1242	1	"Aortic stenosis, non-rheumatic"	Feb-09
49185	G541700	357	0	2	0	Aortic valve calcification	Feb-09
4548	G541.00	10916	0	644	6	Aortic valve disorders	Feb-09
30610	G541600	2196	0	11	0	Aortic valve sclerosis	Feb-09
10964	G541400	456	0	13	0	Aortic valve stenosis with insufficiency	Feb-09
65330	P501.12	2	0	0	0	Aorticopulmonary septal defect	Feb-09
37402	PF55.11	66	0	32	0	Apert's syndrome	Feb-09
95752	PK15.00	1	0	0	0	Aplasia of adrenal gland	Feb-09
59298	P722100	1	0	0	0	Aplasia of aorta	Feb-09
100305	P221.00	2	0	0	0	"Aplasia of brain, part unspecified"	Jul-10
91846	P22A200	3	0	0	0	Aplasia of cerebellum	Feb-09
103057	P228200	1	0	0	0	Aplasia of corpus callosum	Oct-11
59944	P344600	1	0	0	0	Aplasia of iris	Feb-09
52664	P850.00	4	0	0	0	Aplasia of lung	Feb-09
45473	PGyy400	10	0	1	0	Aplasia of muscle	Feb-09
43166	D201100	10	0	0	0	Aplastic anaemia due to drugs	Feb-09
57859	D201200	5	0	0	0	Aplastic anaemia due to infection	Feb-09
66239	D201400	2	0	0	0	Aplastic anaemia due to toxic cause	Feb-09
78	H33..00	4382665	0	46202	206	Asthma	Feb-09
23481	G581.11	436	0	56	0	Asthma - cardiac	Feb-09
10487	663j.00	12298	0	7	0	Asthma - currently active	Feb-09
232	H33z100	89016	0	4269	5	Asthma attack	Feb-09
8335	H33z111	2604	0	12	0	Asthma attack NOS	Feb-09
11370	1O2..00	12868	0	34	0	Asthma confirmed	Feb-09
4442	H33z.00	42919	0	985	12	Asthma unspecified	Feb-09
52659	F23y400	159	0	1	0	Ataxic diplegic cerebral palsy	Feb-09
38800	ZS42411	24	0	0	0	Ataxic dysarthria	Feb-09
21548	F23y000	141	0	4	0	Ataxic infantile cerebral palsy	Feb-09

For peer review only - http://bmjopen.bmj.com/site/about/guidelines.xhtml

68198	P722.00	10	0	0	0	Atresia and stenosis of aorta	Feb-09
51672	PB2..00	22	0	0	0	Atresia and stenosis of large intestine/rectum/anal canal	Feb-09
35968	PB2z.00	41	0	6	0	Atresia and stenosis of large intestine/rectum/anus NOS	Feb-09
27598	PB2..11	27	0	0	0	Atresia large intestine	Feb-09
95045	PD61000	3	0	0	0	Atresia of anterior urethra	Feb-09
31731	PB21000	24	0	0	0	Atresia of anus	Feb-09
99653	PB21300	1	0	0	0	Atresia of appendix	Jun-10
68310	P762.00	3	0	0	0	Atresia of artery NEC	Feb-09
38389	PB61400	4	0	0	0	Atresia of bile duct	Feb-09
97475	PD60000	2	0	0	0	Atresia of bladder neck	Aug-09
93089	P6yy000	4	0	0	0	Atresia of cardiac vein	Feb-09
65385	PC4yA00	7	0	0	0	Atresia of cervix	Feb-09
23593	PB21100	60	0	1	0	Atresia of colon	Feb-09
19039	PB10100	246	0	2	0	Atresia of duodenum	Feb-09
56100	PCy3000	23	0	5	0	Atresia of ejaculatory duct	Feb-09
61727	P832000	2	0	0	0	Atresia of epiglottis	Feb-09
25819	P402000	119	0	6	0	Atresia of external auditory canal	Feb-09
61770	PC1y200	4	0	1	0	Atresia of fallopian tube	Feb-09
51345	P233.00	10	0	0	0	Atresia of foramina of Magendie and Luschka	Feb-09
95015	PCy3.00	2	0	0	0	Atresia of genital organ NEC	Feb-09
62335	PCy3z00	1	0	0	0	Atresia of genital organ NEC NOS	Feb-09
92834	P832100	3	0	0	0	Atresia of glottis	Feb-09
37451	P6yy700	10	0	0	0	Atresia of heart valve NEC	Feb-09
50400	PB61600	8	0	0	0	Atresia of hepatic ducts	Feb-09
53565	PB10200	59	0	0	0	Atresia of ileum	Feb-09
52687	PB10300	86	0	1	0	Atresia of jejunum	Feb-09
31468	PB21.00	36	0	5	0	Atresia of large intestine	Feb-09
72001	P832.00	2	0	0	0	Atresia of larynx and trachea	Feb-09
52894	P8y2.00	5	0	0	0	Atresia of nasopharynx	Feb-09

For peer review only - http://bmjopen.bmj.com/site/about/guidelines.xhtml

33655	PA37.00	133	0	1	0	Atresia of oesophagus with tracheo-oesophageal fistula	Feb-09
73673	P344100	5	0	0	0	Atresia of pupil	Feb-09
46123	PB21200	21	0	0	0	Atresia of rectum	Feb-09
71153	PB21500	9	0	0	0	Atresia of rectum with fistula	Feb-09
33584	PB10.00	115	0	1	0	Atresia of small intestine	Feb-09
40252	PB10000	36	0	3	0	"Atresia of small intestine, unspecified"	Feb-09
68606	P802.00	2	0	0	0	Atresia of the posterior nares	Feb-09
63288	P832300	11	0	0	0	Atresia of trachea	Feb-09
49700	PD20.00	7	0	0	0	Atresia of ureter	Feb-09
27648	K562300	53	0	6	0	Atresia of vagina	Feb-09
36548	PC4yB00	32	0	3	0	Atresia of vagina	Feb-09
73156	K562.12	2	0	0	0	Atresia of vagina	Feb-09
58076	P722z00	36	0	0	0	Atresia or stenosis of aorta NOS	Feb-09
34305	PA22.00	42	0	5	0	"Atresia, salivary duct"	Feb-09
1664	G573000	374276	0	21137	103	Atrial fibrillation	Feb-09
2212	G573.00	146690	0	11825	6	Atrial fibrillation and flutter	Feb-09
23437	G573z00	1241	0	29	0	Atrial fibrillation and flutter NOS	Feb-09
45773	6A9..00	17151	0	49	0	Atrial fibrillation annual review	Feb-09
105554	8CMW200	518	0	1	0	Atrial fibrillation care pathway	Jan-13
104570	1I12.00	82	0	0	0	Atrial fibrillation excluded	Jul-12
57832	9Os..00	5501	0	150	0	Atrial fibrillation monitoring administration	Feb-09
28994	212R.00	22216	0	83	0	Atrial fibrillation resolved	Feb-09
9023	G576300	200	0	17	0	Atrial premature depolarization	Feb-09
3255	P550.00	11410	0	274	1	Atrial septal defect NOS	Feb-09
23708	G361.00	35	0	2	0	Atrial septal defect/curr comp folow acut myocardial infarct	Feb-09
27375	G561z00	173	0	8	0	Atrioventricular block NOS	Feb-09
58032	G561000	80	0	0	0	Atrioventricular block unspecified	Feb-09
28543	ZS91.00	878	0	28	0	Attention deficit disorder	Feb-09

For peer review only - http://bmjopen.bmj.com/site/about/guidelines.xhtml

101067	6A61.00	2631	0	816	0	Attention deficit hyperactivity disorder annual review	Dec-10
34199	E2E0000	84	0	3	0	Attention deficit without hyperactivity	Feb-09
24244	E14y000	4	0	0	0	Atypical childhood psychoses	Feb-09
1276	E140.12	18470	0	1122	1	Autism	Feb-09
7957	J614111	1100	0	35	0	Autoimmune chronic active hepatitis	Feb-09
26410	C394.00	1332	0	57	1	Autoimmune disease NOS	Feb-09
39876	D110z00	238	0	3	0	Autoimmune haemolytic anaemia NOS	Feb-09
3818	D110.00	1773	0	106	2	Autoimmune haemolytic anaemias	Feb-09
18652	J63B.00	2558	0	41	0	Autoimmune hepatitis	Feb-09
31971	C046.00	910	0	4	0	Autoimmune myxoedema	Feb-09
61859	C121000	7	0	1	0	Autoimmune parathyroiditis	Feb-09
52843	C182.00	65	0	2	0	Autoimmune polyglandular failure	Feb-09
16420	42P2.11	378	0	109	459	Auto-immune thrombocytopenia	Feb-09
3857	C052.11	2107	0	108	24	Autoimmune thyroiditis	Feb-09
106346	C353800	2	0	0	0	Autosomal dominant hypophosphataemic rickets	Jun-13
110208	PD12211	1	0	0	0	Autosomal dominant medullary cystic disease	Oct-15
105143	PD11111	56	0	0	0	Autosomal dominant polycystic kidney disease	Nov-12
105794	PD12012	3	0	0	0	Autosomal recessive medullary cystic disease	Feb-13
105919	PD11011	8	0	0	0	Autosomal recessive polycystic kidney disease	Apr-13
40288	C151200	184	0	8	0	Bartter's syndrome	Feb-09
47778	C151.12	17	0	0	0	Bartter's syndrome	Feb-09
32749	F391800	373	0	28	0	Becker muscular dystrophy	Feb-09
73283	G552.11	8	0	1	0	Becker's disease	Feb-09

For peer review only

12357	PKy9100	175	0	6	0	Beckwith's syndrome	Feb-09
16235	AD61.00	3552	0	263	1	Behcet's syndrome	Feb-09
58818	N012011	14	0	2	0	Behcet's syndrome arthropathy	Feb-09
63333	F390000	21	0	0	0	Benign congenital myopathy	Feb-09
19170	F25y400	1212	0	15	0	Benign Rolandic epilepsy	Feb-09
57144	D104600	54	0	1	0	Beta intermedia thalassaemia	Feb-09
27761	D104800	448	0	5	1	Beta minor thalassaemia	Feb-09
9864	D104500	10190	0	80	23	Beta trait thalassaemia	Feb-09
3300	P641.00	4594	0	140	0	Bicuspid aortic valve	Feb-09
57458	PKy1.11	63	0	4	0	Biedl-Bardet syndrome	Feb-09
55370	D010.12	4	0	0	0	Biermer's congenital pernicious anaemia	Feb-09
54721	P913.00	24	0	1	0	Bilateral complete cleft lip	Feb-09
48948	P903.00	20	0	0	0	Bilateral complete cleft palate	Feb-09
44977	P923.00	20	0	0	0	Bilateral complete cleft palate with cleft lip	Feb-09
71154	PD02000	5	0	0	0	Bilateral congenital absence of kidneys	Feb-09
38373	PE30100	256	0	0	0	Bilateral congenital dislocation of hip	Feb-09
107323	F591A00	36	0	3	0	Bilateral congenital sensorineural hearing loss	Dec-13
61870	PE31100	43	0	0	0	Bilateral congenital subluxation of hip	Feb-09
4035	1C13300	3079	0	380	0	Bilateral deafness	Feb-09
34285	PE34100	160	0	0	0	Bilateral dysplastic hip	Feb-09
106078	P904.00	1	0	0	0	Bilateral incomplete cleft palate	May-13
62690	P924.00	4	0	0	0	Bilateral incomplete cleft palate with cleft lip	Feb-09
9500	PD04000	49	0	0	0	Bilateral renal dysplasia	Feb-09
42925	PD03000	12	0	2	0	Bilateral renal hypoplasia	Feb-09
38774	K091.00	122	0	2	0	Bilateral small kidneys	Feb-09

For peer review only - http://bmjopen.bmj.com/site/about/guidelines.xhtml

50468	PE35100	103	0	0	0	Bilateral unstable hip	Feb-09
40425	PB6..11	93	0	7	0	Bile duct anomalies	Feb-09
53764	PB60200	16	0	1	0	"Bile duct anomaly, unspecified"	Feb-09
16005	J66z.00	676	0	40	2	Bile duct disorder NOS	Feb-09
9494	J616.00	2107	0	74	0	Biliary cirrhosis	Feb-09
58630	J616z00	47	0	0	0	Biliary cirrhosis NOS	Feb-09
91591	J616200	1	0	0	0	Biliary cirrhosis of children	Feb-09
55829	E114400	40	0	1	0	"Bipolar affect disord, currently manic,severe with psychosis"	Feb-09
72026	E115500	6	0	0	0	"Bipolar affect disord, now depressed, part/unspec remission"	Feb-09
63701	E115400	19	0	0	0	"Bipolar affect disord, now depressed, severe with psychosis"	Feb-09
35607	E115300	30	0	0	0	"Bipolar affect disord, now depressed, severe, no psychosis"	Feb-09
59011	E114500	8	0	0	0	"Bipolar affect disord,currently manic, part/unspec remission"	Feb-09
4677	E115.00	524	0	37	0	"Bipolar affective disorder, currently depressed"	Feb-09
35734	E115100	65	0	0	0	"Bipolar affective disorder, currently depressed, mild"	Feb-09
27890	E115200	63	0	1	0	"Bipolar affective disorder, currently depressed, moderate"	Feb-09
37296	E115z00	87	0	3	0	"Bipolar affective disorder, currently depressed, NOS"	Feb-09
3702	E114.00	507	0	38	0	"Bipolar affective disorder, currently manic"	Feb-09
63784	E114600	46	0	1	0	"Bipolar affective disorder, currently manic, full remission"	Feb-09
36126	E114100	55	0	1	0	"Bipolar affective disorder, currently manic, mild"	Feb-09

For peer review only

46434	E114200	40	0	1	0	"Bipolar affective disorder, currently manic, moderate"	Feb-09
57605	E114z00	64	0	1	0	"Bipolar affective disorder, currently manic, NOS"	Feb-09
35738	E114000	70	0	1	0	"Bipolar affective disorder, currently manic, unspecified"	Feb-09
57465	E115600	75	0	0	0	"Bipolar affective disorder, now depressed, in full remission"	Feb-09
8567	E11..11	6557	0	431	0	Bipolar psychoses	Feb-09
32900	D200311	173	0	5	0	Blackfan - Diamond syndrome	Feb-09
109273	D200.13	0	0	0	0	Blackfan - Diamond syndrome	Feb-15
33762	PG0y000	727	0	65	0	Brachycephaly	Feb-09
20676	G820.00	259	0	2	0	Budd - Chiari syndrome (hepatic vein thrombosis)	Feb-09
29180	G5y9.00	56	0	1	0	"Cardiac septal defect, acquired"	Feb-09
21943	P5...11	70	0	11	0	Cardiac septal defects	Feb-09
509	G5y3.00	16459	0	1620	47	Cardiomegaly	Feb-09
10890	Z7E4400	1867	0	167	0	Cerebellar ataxia	Feb-09
48731	Q200700	12	0	0	0	Cerebral haemorrhage due to birth injury	Feb-09
46545	S62z.00	50	0	0	0	Cerebral haemorrhage following injury NOS	Feb-09
23447	Q200000	97	0	0	0	"Cerebral haemorrhage unspecified, due to birth trauma"	Feb-09
104498	F2B..00	351	0	2	0	Cerebral palsy	Jul-12
104654	F2Bz.00	30	0	0	0	Cerebral palsy NOS	Aug-12
5512	F230100	1189	0	27	0	Cerebral palsy with spastic diplegia	Feb-09
16956	G669.00	511	0	20	0	"Cerebral palsy, not congenital or infantile, acute"	Feb-09
7529	F286.11	19513	0	1701	0	CFS - Chronic fatigue syndrome	Feb-09
108711	K000111	1	0	0	0	CGN - Crescentic glomerulonephritis	Oct-14
5584	F361000	2642	0	294	0	Charcot-Marie-Tooth disease	Feb-09

For peer review only - http://bmjopen.bmj.com/site/about/guidelines.xhtml

96587	4L4C.00	13	0	10	34	Charcot-Marie-Tooth disease type 1A gene detection test	Apr-09
108834	F361012	2	0	0	0	Charcot-Marie-Tooth syndrome	Nov-14
43808	P2x6.00	28	0	0	0	Chiari malformation type I	Feb-09
62968	P101000	195	0	2	0	Chiari malformation type I	Feb-09
1208	H330.12	21212	0	112	3	Childhood asthma	Feb-09
7302	E140.13	343	0	4	0	Childhood autism	Feb-09
3775	E2E..00	7103	0	1175	1	Childhood hyperkinetic syndrome	Feb-09
37395	E14z.11	44	0	2	0	Childhood schizophrenia NOS	Feb-09
31027	P80..00	621	0	27	0	Choanal atresia	Feb-09
60514	P80z.00	14	0	0	0	Choanal atresia NOS	Feb-09
66643	P800.00	10	0	0	0	"Choanal atresia, unspecified"	Feb-09
22756	PG4..00	142	0	11	1	Chondrodysplasia	Feb-09
58035	PG56000	5	0	0	0	Chondrodysplasia calcificans congenita	Feb-09
63146	PG56011	2	0	0	0	Chondrodysplasia calcificans congenita	Feb-09
14917	PG4z.00	44	0	7	0	Chondrodysplasia NOS	Feb-09
18193	PG4C.00	24	0	0	0	Chondrodysplasia punctata	Feb-09
73905	PG40.00	3	0	0	0	"Chondrodysplasia, unspecified"	Feb-09
671	PJ...00	1722	0	52	5	Chromosomal anomalies	Feb-09
20231	PJz..00	509	0	19	1	Chromosomal anomalies NOS	Feb-09
104196	PJ33800	5	0	0	0	Chromosome 4q deletion syndrome	Jun-12
54241	PJ53000	34	0	0	0	Chromosome inversion in normal individual	Feb-09
106938	PJ38.11	3	0	0	0	Chromosome replaced with dicentric	Aug-13
47247	PJ38.12	11	0	0	0	Chromosome replaced with ring	Feb-09
100562	PJ38.00	1	0	0	0	Chromosome replaced with ring or dicentric	Aug-10
9029	J614100	2400	0	106	1	Chronic active hepatitis	Feb-09

For peer review only

106805	H335.00	166	0	5	0	Chronic asthma with fixed airflow obstruction	Aug-13
5798	H312000	1813	0	47	0	Chronic asthmatic bronchitis	Feb-09
4669	K02y200	195	0	7	0	Chronic focal glomerulonephritis	Feb-09
7804	K02..00	1957	0	75	0	Chronic glomerulonephritis	Feb-09
97758	K02y000	2	0	0	0	Chronic glomerulonephritis + diseases EC	Oct-09
15097	K02z.00	184	0	7	0	Chronic glomerulonephritis NOS	Feb-09
104981	K05..13	179	0	0	0	Chronic kidney disease	Sep-12
29013	1Z10.00	22548	0	89	0	Chronic kidney disease stage 1	Feb-09
105392	K051.00	23	0	0	0	Chronic kidney disease stage 1	Dec-12
94789	1Z17.00	132	0	3	0	Chronic kidney disease stage 1 with proteinuria	Feb-09
95572	1Z18.00	228	0	1	0	Chronic kidney disease stage 1 without proteinuria	Feb-09
105383	K052.00	416	0	0	0	Chronic kidney disease stage 2	Dec-12
95146	1Z19.00	500	0	1	0	Chronic kidney disease stage 2 with proteinuria	Feb-09
95121	1Z1A.00	2368	0	4	0	Chronic kidney disease stage 2 without proteinuria	Feb-09
73026	K0A3500	3	0	0	0	Chronic neph syn difus mesangiocapillary glomerulonephritis	Feb-09
60857	K0A3700	18	0	0	0	Chronic nephritic syn diffuse crescentic glomerulonephritis	Feb-09
40413	K0A3100	10	0	0	0	Chronic nephritic syndrm focal+segmental glomerular lesions	Feb-09
21297	K0A3.00	86	0	3	0	Chronic nephritic syndrome	Feb-09
60198	K0A3600	7	0	0	0	"Chronic nephritic syndrome, dense deposit disease"	Feb-09
66505	K0A3000	5	0	0	0	"Chronic nephritic syndrome, minor glomerular abnormality"	Feb-09

For peer review only - http://bmjopen.bmj.com/site/about/guidelines.xhtml

24814	H591.00	102	0	1	0	Chronic respiratory failure	Feb-09
9312	G1...00	1190	0	43	0	Chronic rheumatic heart disease	Feb-09
6863	J61..00	7053	0	402	1	Cirrhosis and chronic liver disease	Feb-09
97980	1Z17.11	5	0	0	0	CKD stage 1 with proteinuria	Nov-09
111022	1Z18.11	1	0	0	0	CKD stage 1 without proteinuria	Jul-16
97979	1Z19.11	22	0	0	0	CKD stage 2 with proteinuria	Nov-09
97978	1Z1A.11	35	0	0	0	CKD stage 2 without proteinuria	Nov-09
110033	1Z1M.00	8	0	0	0	CKD with GFR category G1 & albuminuria category A1	Aug-15
110003	1Z1N.00	5	0	0	0	CKD with GFR category G1 & albuminuria category A2	Aug-15
110484	1Z1P.00	2	0	0	0	CKD with GFR category G1 & albuminuria category A3	Jan-16
110269	1Z1Q.00	86	0	0	0	CKD with GFR category G2 & albuminuria category A1	Nov-15
110108	1Z1R.00	40	0	0	0	CKD with GFR category G2 & albuminuria category A2	Sep-15
110251	1Z1S.00	6	0	0	0	CKD with GFR category G2 & albuminuria category A3	Nov-15
65623	P907.11	23	0	1	0	Cleft hard palate NOS	Feb-09
33902	P92A.00	12	0	0	0	"Cleft hard palate with cleft lip, bilateral"	Feb-09
62321	P92B.00	13	0	0	0	"Cleft hard palate with cleft lip, unilateral"	Feb-09
93972	P928.00	5	0	0	0	"Cleft hard palate with cleft soft palate, unilateral"	Feb-09
64549	P90B.00	2	0	0	0	"Cleft hard palate, bilateral"	Feb-09
38873	P901.11	2	0	0	0	"Cleft hard palate, unilateral"	Feb-09
101223	P90C.00	3	0	0	0	"Cleft hard palate, unilateral"	Dec-10
2027	P91..00	1719	0	65	0	Cleft lip (harelip)	Feb-09
35374	P91z.00	139	0	2	0	Cleft lip NOS	Feb-09
15364	P90z.00	237	0	39	0	Cleft palate NOS	Feb-09

For peer review only - http://bmjopen.bmj.com/site/about/guidelines.xhtml

50430	P9z..00	90	0	1	0	Cleft palate or cleft lip NOS	Feb-09
68398	P92z.00	22	0	2	0	Cleft palate with cleft lip NOS	Feb-09
51301	P920.00	19	0	0	0	"Cleft palate with cleft lip, unspecified"	Feb-09
58885	P900.00	75	0	0	0	"Cleft palate, unspecified"	Feb-09
25740	P908.11	114	0	2	0	Cleft soft palate NOS	Feb-09
42208	P90A.00	22	0	1	0	"Cleft soft palate, bilateral"	Feb-09
97477	P904.11	3	0	0	0	"Cleft soft palate, bilateral"	Aug-09
68166	P906.11	8	0	0	0	"Cleft soft palate, central"	Feb-09
57490	P902.12	2	0	1	0	"Cleft soft palate, unilateral"	Feb-09
1515	J690.00	55378	0	3179	25	Coeliac disease	Feb-09
44310	J690z00	738	0	19	0	Coeliac disease NOS	Feb-09
62236	C392.00	8	0	0	0	Combined immunity deficiency	Feb-09
62328	C392z00	3	0	0	0	Combined immunity deficiency NOS	Feb-09
43049	P56z100	21	0	1	0	Common atrioventricular canal	Feb-09
44896	P56z200	286	0	3	0	Common atrioventricular-type ventricular septal defect	Feb-09
55535	P56z000	35	0	3	0	Common atrium	Feb-09
40765	SB00100	4	0	0	0	Common carotid artery injury	Feb-09
98893	P50..12	3	0	0	0	Common truncus	Apr-10
66857	C398200	2	0	1	0	Common variable immunodef wth autoantibod to B- or T-cells	Feb-09
21975	C398.00	519	0	7	0	Common variable immunodeficiency	Feb-09
57322	C390700	20	0	0	0	Common variable immunodeficiency	Feb-09
30187	PKy5.00	52	0	6	0	Congen malformation syndromes affecting facial appearance	Feb-09
62705	PF6B300	2	0	0	0	Congen overgrowth of whole lower limb	Feb-09
109067	PD8..00	19	0	1	0	Congenital abnormality of the kidney	Jan-15

For peer review only - http://bmjopen.bmj.com/site/about/guidelines.xhtml

59430	PB20000	11	0	0	0	Congenital absence of anus	Feb-09
93826	PB20300	4	0	0	0	Congenital absence of anus with fistula	Feb-09
16417	PB61000	16	0	2	0	Congenital absence of bile duct	Feb-09
39970	PDy0.00	10	0	0	0	Congenital absence of bladder	Feb-09
48901	PF5F.00	32	0	3	0	Congenital absence of both forearm and hand	Feb-09
25316	P22A000	2	0	0	0	Congenital absence of cerebellum	Feb-09
68093	PG13000	2	0	0	0	Congenital absence of cervical vertebra	Feb-09
35942	P4y4.00	5	0	0	0	Congenital absence of chin	Feb-09
57747	PC4y200	4	0	0	0	Congenital absence of clitoris	Feb-09
93830	PG1x000	3	0	0	0	Congenital absence of coccyx	Feb-09
71956	P6y4000	1	0	0	0	Congenital absence of coronary artery	Feb-09
41775	P228000	53	0	2	0	Congenital absence of corpus callosum	Feb-09
60738	PBy0.00	2	0	0	0	Congenital absence of digestive system NOS	Feb-09
68208	P300200	6	0	0	0	Congenital absence of eye	Feb-09
67756	P303.00	3	0	0	0	Congenital absence of eyes	Feb-09
33394	PF29.00	78	0	4	0	Congenital absence of finger	Feb-09
27624	PF25.00	39	0	2	0	Congenital absence of forearm only	Feb-09
42256	PB63000	13	0	0	0	Congenital absence of gallbladder	Feb-09
46835	PF21613	68	0	1	0	Congenital absence of hand	Feb-09
48269	PB61500	2	0	0	0	Congenital absence of hepatic ducts	Feb-09
18406	PB11200	1	0	0	0	Congenital absence of ileum	Feb-09
3314	PD02.00	2413	0	57	0	Congenital absence of kidney	Feb-09
65407	PD02z00	53	0	0	0	Congenital absence of kidney NOS	Feb-09
58855	PB20.00	2	0	0	0	Congenital absence of large intestine	Feb-09
60525	PF31200	8	0	0	0	Congenital absence of leg and foot	Feb-09

For peer review only - http://bmjopen.bmj.com/site/about/guidelines.xhtml

62531	P335.11	1	0	0	0	Congenital absence of lens	Feb-09
28661	PF40.00	6	0	0	0	Congenital absence of limb NOS	Feb-09
98402	PB63100	2	0	0	0	Congenital absence of liver lobe	Jan-10
97185	P853100	3	0	0	0	Congenital absence of lobe of lung	Aug-09
72561	PF35.00	5	0	0	0	Congenital absence of lower leg only	Feb-09
54384	P853.11	5	0	0	0	Congenital absence of lung	Feb-09
68268	P853000	5	0	0	0	Congenital absence of lung fissures	Feb-09
98859	PA35.00	1	0	0	0	Congenital absence of oesophagus	Apr-10
64339	PF39400	8	0	0	0	Congenital absence of other multiple toes	Feb-09
42571	PC00.00	45	0	1	0	Congenital absence of ovary	Feb-09
61638	PF64000	14	0	1	0	Congenital absence of patella	Feb-09
49245	PCy0000	4	0	0	0	Congenital absence of penis	Feb-09
101896	P6yy411	2	0	0	0	Congenital absence of pericardium	Mar-11
111629	PK24100	1	0	0	0	Congenital absence of pituitary gland	Jan-17
103672	P731.11	2	0	0	0	Congenital absence of pulmonary artery	Feb-12
97779	P364111	2	0	0	0	Congenital absence of punctum lacrimale	Oct-09
50443	PB20200	7	0	0	0	Congenital absence of rectum	Feb-09
58095	PB20400	1	0	0	0	Congenital absence of rectum with fistula	Feb-09
42378	PG30.00	23	0	0	0	Congenital absence of rib	Feb-09
43351	PG13300	12	0	1	0	Congenital absence of sacrum	Feb-09
68081	PG1x100	1	0	0	0	Congenital absence of sacrum	Feb-09
38119	PG00.00	1	0	0	0	Congenital absence of skull bones	Feb-09
63291	PG31.00	4	0	0	0	Congenital absence of sternum	Feb-09
73987	PK28100	3	0	0	0	Congenital absence of thymus	Feb-09
3374	PK25100	233	0	26	1	Congenital absence of thyroid gland	Feb-09

For peer review only - http://bmjopen.bmj.com/site/about/guidelines.xhtml

72180	PF24.00	2	0	0	0	Congenital absence of upper arm only	Feb-09
37973	PF21.11	30	0	1	0	Congenital absence part of arm	Feb-09
37277	PF23.00	14	0	0	0	Congenital absence upper arm and forearm with hand present	Feb-09
23416	PA70.11	47	0	2	0	Congenital achalasia of cardia	Feb-09
36786	C152813	9	0	0	0	Congenital adrenal gland hypertrophy NEC	Feb-09
29640	C152000	304	0	14	0	Congenital adrenogenital syndrome	Feb-09
59314	D303000	11	0	0	0	Congenital afibrinogenaemia	Feb-09
16618	PH40.00	94	0	3	0	Congenital alopecia	Feb-09
98667	PH40z00	1	0	0	0	Congenital alopecia NOS	Mar-10
104030	PH40000	2	0	0	0	"Congenital alopecia, unspecified"	May-12
9884	F480011	3463	0	170	0	Congenital amblyopia	Feb-09
53564	PF45.00	15	0	0	0	Congenital amputation of unspecified limb	Feb-09
59307	PF21400	47	0	0	0	Congenital amputation of upper limb	Feb-09
31248	Q455.00	115	0	2	0	Congenital anaemia	Feb-09
99494	Q455000	3	0	0	0	Congenital anaemia from fetal blood loss	May-10
37080	PB59.00	71	0	10	0	Congenital anal fistula	Feb-09
35019	PB24.11	53	0	9	0	Congenital anal stricture	Feb-09
63173	P7yz000	13	0	2	0	Congenital aneurysm NEC	Feb-09
56200	P72z100	23	0	1	0	Congenital aneurysm of aorta	Feb-09
108193	P6yy600	1	0	0	0	Congenital aneurysm of heart	Jun-14
54295	PF6y000	25	0	0	0	Congenital angulation of tibia	Feb-09
44851	P344000	156	0	4	0	Congenital anisocoria	Feb-09
47533	J000200	9	0	1	0	Congenital anodontia	Feb-09
5574	PG1u.00	57	0	0	0	Congenital anomalies of cervical vertebrae NEC	Feb-09

For peer review only

24218	PF5v.00	20	0	1	0	Congenital anomalies of elbow and upper arm	Feb-09
94280	P36..00	6	0	0	0	"Congenital anomalies of eyelid, lacrimal system and orbit"	Feb-09
94285	P35z.00	3	0	0	0	Congenital anomalies of posterior chamber NOS	Feb-09
40690	PG1v.00	26	0	1	0	Congenital anomalies of thoracic vertebrae NEC	Feb-09
55893	P7y0000	9	0	0	0	"Congenital anomaly of cerebral vessel, unspecified"	Feb-09
41763	PG1uz00	12	0	2	0	Congenital anomaly of cervical vertebrae NEC NOS	Feb-09
37784	P4z1.00	22	0	2	0	Congenital anomaly of face NOS	Feb-09
66463	PG1wz00	12	0	0	0	Congenital anomaly of lumbar vertebra NEC NOS	Feb-09
68894	P6yy500	3	0	0	0	Congenital anomaly of myocardium	Feb-09
93198	P4z0.00	4	0	0	0	Congenital anomaly of neck NOS	Feb-09
108675	P76y.00	3	0	0	0	Congenital anomaly of peripheral vascular system OS	Oct-14
45918	P25y200	3	0	0	0	Congenital anomaly of spinal meninges	Feb-09
71152	PG1vz00	6	0	0	0	Congenital anomaly of thoracic vertebrae NEC NOS	Feb-09
9723	PFz..00	347	0	6	0	Congenital anomaly of unspecified limb NOS	Feb-09
8636	P64..00	65	0	4	0	Congenital aortic valve insufficiency	Feb-09
6843	P64z.00	10	0	0	0	Congenital aortic valve insufficiency NOS	Feb-09
58734	P640.00	18	0	0	0	"Congenital aortic valve insufficiency, unspecified"	Feb-09
6886	P63..00	4086	0	265	0	Congenital aortic valve stenosis	Feb-09
69823	P335.00	19	0	0	0	Congenital aphakia	Feb-09

For peer review only - http://bmjopen.bmj.com/site/about/guidelines.xhtml

94282	PCy1.00	1	0	0	0	Congenital aplasia of genital organ NEC	Feb-09
91739	PCy1000	2	0	0	0	Congenital aplasia of prostate	Feb-09
38020	PCy1300	3	0	0	0	Congenital aplasia of scrotum	Feb-09
58591	P769.00	4	0	0	0	Congenital arterial stricture	Feb-09
95800	P7y0111	3	0	0	0	Congenital arteriovenous fistula of brain	Feb-09
62587	P357100	12	0	1	0	Congenital arteriovenous malformation of retina	Feb-09
66314	F137111	10	0	0	0	Congenital athetosis	Feb-09
59553	PH40.11	4	0	0	0	Congenital atrichosis	Feb-09
22246	F38y.13	751	0	21	0	Congenital benign hypotonia	Feb-09
53454	PE07.00	23	0	5	0	Congenital bent or squashed nose	Feb-09
42721	P421.11	107	0	21	0	Congenital big ears	Feb-09
95892	P226000	8	0	0	0	Congenital bilateral perisylvian syndrome	Feb-09
57166	PDy5.00	8	0	1	0	Congenital bladder hernia	Feb-09
47854	PD60z00	14	0	0	0	Congenital bladder neck obstruction NOS	Feb-09
36666	P7y0200	92	0	2	0	Congenital brain aneurysm NEC	Feb-09
8893	P2y0.00	58	0	0	0	Congenital brain anomaly	Feb-09
56427	P861.00	20	0	1	0	Congenital bronchiectasis	Feb-09
32600	P843.12	100	0	3	0	Congenital bronchogenic cyst	Feb-09
11845	P83yB00	131	0	1	0	Congenital bronchomalacia	Feb-09
56531	PD31.00	42	0	2	0	Congenital calculus of kidney	Feb-09
34007	P6yy200	30	0	0	0	Congenital cardiomegaly	Feb-09
72271	PA70.00	3	0	0	0	Congenital cardiospasm	Feb-09
45926	P33z.00	96	0	6	0	Congenital cataract or lens anomaly NOS	Feb-09
299	P330.00	2282	0	166	0	"Congenital cataract, unspecified"	Feb-09
24424	P7y0100	216	0	17	0	Congenital cerebral arteriovenous aneurysm	Feb-09

For peer review only - http://bmjopen.bmj.com/site/about/guidelines.xhtml

44581	P7y0112	44	0	2	0	Congenital cerebral arteriovenous malformation	Feb-09
39391	P240.00	47	0	2	0	Congenital cerebral cyst	Feb-09
71733	P240z00	1	0	0	0	Congenital cerebral cyst NOS	Feb-09
53474	P20..13	6	0	0	0	Congenital cerebral hernia	Feb-09
2069	F23..00	14415	0	746	2	Congenital cerebral palsy	Feb-09
28306	F23z.00	496	0	11	0	Congenital cerebral palsy NOS	Feb-09
20979	PB6y000	216	0	12	0	Congenital choledochal cyst	Feb-09
34924	P352.00	107	0	4	0	Congenital chorioretinal degeneration	Feb-09
56810	P7yz100	69	0	0	0	Congenital chylothorax	Feb-09
30141	PF58.00	33	0	0	0	Congenital cleft hand	Feb-09
66698	P813.00	12	0	0	0	Congenital cleft nose	Feb-09
105605	P83y700	1	0	0	0	Congenital cleft of posterior cricoid cartilage	Jan-13
41019	PE8y011	38	0	0	0	Congenital club fingers	Feb-09
45651	PE8y000	80	0	5	0	Congenital club hand	Feb-09
23980	PE60.11	58	0	8	0	Congenital clubfoot - valgus	Feb-09
62397	J690000	21	0	0	0	Congenital coeliac disease	Feb-09
48508	PF5G.00	28	0	0	0	Congenital complete absence of upper limb(s)	Feb-09
52310	P6y5100	32	0	0	0	Congenital complete atrio-ventricular heart block	Feb-09
52822	PG15000	4	0	0	0	Congenital complete fusion of spine	Feb-09
31211	PKy7A00	10	0	0	0	Congenital contractural arachnodactyly	Feb-09
66259	P341z00	20	0	1	0	Congenital corneal opacities NOS	Feb-09
71732	P341000	4	0	1	0	Congenital corneal opacity with visual deficit	Feb-09
49901	P6y4500	5	0	0	0	Congenital coronary aneurysm	Feb-09
28705	P6y4411	7	0	3	0	Congenital coronary arterio-venous fistula	Feb-09

For peer review only - http://bmjopen.bmj.com/site/about/guidelines.xhtml

96586	P844.00	85	0	0	0	Congenital cystic adenomatoid malformation of the lung	Apr-09
51185	P301.00	17	0	1	0	Congenital cystic eyeball	Feb-09
15917	PD1..00	760	0	46	1	Congenital cystic kidney disease	Feb-09
50331	PD1z.00	34	0	1	0	Congenital cystic kidney disease NOS	Feb-09
27565	PB62.00	293	0	48	20	Congenital cystic liver disease	Feb-09
72000	PB62z00	5	0	0	0	Congenital cystic liver disease NOS	Feb-09
66223	P840.00	11	0	0	0	"Congenital cystic lung disease, unspecified"	Feb-09
33804	P84z.00	12	0	2	0	Congenital cystic lung NOS	Feb-09
60812	P353100	4	0	3	0	Congenital cysts of the posterior segment	Feb-09
59281	PG61.11	3	0	0	0	Congenital defect of diaphragmatic NEC	Feb-09
46289	D010.13	10	0	1	1	Congenital deficiency of intrinsic factor	Feb-09
57275	D303z00	52	0	1	0	Congenital deficiency of other clotting factor NOS	Feb-09
54725	D303y00	15	0	0	0	Congenital deficiency of other clotting factor OS	Feb-09
46026	D303.00	104	0	4	0	Congenital deficiency of other clotting factors	Feb-09
27426	PF6y100	116	0	27	0	Congenital deformity of ankle joint	Feb-09
28542	PF51.00	58	0	3	0	Congenital deformity of clavicle	Feb-09
40517	PF63X00	230	0	15	0	"Congenital deformity of hip, unspecified"	Feb-09
53416	PG1y.11	10	0	0	0	Congenital deformity of lumbosacral joint	Feb-09
51156	PG1y.12	8	0	0	0	Congenital deformity of lumbosacral region	Feb-09

For peer review only - http://bmjopen.bmj.com/site/about/guidelines.xhtml

35801	PGz..11	30	0	1	0	Congenital deformity of musculoskeletal system NEC	Feb-09
27676	PHz2.11	49	0	5	0	Congenital deformity of nail	Feb-09
64527	PF6y200	7	0	0	0	Congenital deformity of sacroiliac joint	Feb-09
49743	PF5w.11	43	0	3	0	Congenital deformity of scapula NEC	Feb-09
54223	PHz0.11	11	0	0	0	Congenital dermal defect	Feb-09
98061	P6y8.00	15	0	0	0	Congenital dextroposition of heart	Nov-09
49768	PG61.00	93	0	0	0	Congenital diaphragmatic hernia	Feb-09
11293	P72z111	55	0	1	0	Congenital dilatation of aorta	Feb-09
22560	PA40.00	281	0	17	0	Congenital dilatation of oesophagus	Feb-09
37835	P83y100	8	0	0	0	Congenital dilatation of trachea	Feb-09
26403	PD24.00	150	0	5	0	Congenital dilatation of ureter	Feb-09
64134	P248.00	50	0	1	0	Congenital dilated lateral ventricles of brain	Feb-09
22571	PD23.11	1047	0	4	0	Congenital dilated renal pelvis	Feb-09
65590	PB6y700	10	0	0	0	Congenital dilation of bile duct	Feb-09
25324	F230.00	543	0	16	0	Congenital diplegia	Feb-09
45551	F230z00	70	0	0	0	Congenital diplegia NOS	Feb-09
9239	PE3..00	5653	0	212	1	Congenital dislocation and subluxation of the hip	Feb-09
22301	PE8y200	500	0	25	0	Congenital dislocation of elbow	Feb-09
933	PE30.00	6905	0	689	1	Congenital dislocation of hip	Feb-09
27301	PE3z.00	528	0	15	0	Congenital dislocation of hip NOS	Feb-09
33576	PE30z00	158	0	1	0	Congenital dislocation of hip NOS	Feb-09
99683	P83y500	4	0	0	0	Congenital diverticulum of trachea	Jun-10
55900	PA43.00	9	0	0	0	Congenital duplication of oesophagus	Feb-09
56752	D21y012	10	0	0	0	Congenital dyserythropoiesis NEC	Feb-09
31040	D21y011	30	0	1	0	Congenital dyserythropoietic anaemia	Feb-09

For peer review only - http://bmjopen.bmj.com/site/about/guidelines.xhtml

For peer review only

53422	D21y000	9	0	0	0	Congenital dyshaematopoietic anaemia	Feb-09
66049	D401000	1	0	0	0	Congenital dysphagocytosis	Feb-09
29474	P337.00	20	0	0	0	Congenital ectopic lens	Feb-09
31998	P361300	14	0	2	0	Congenital ectropion	Feb-09
109860	PA2A000	1	0	0	0	Congenital ectropion of lip	Jul-15
51067	PH03.00	13	0	1	0	Congenital elephantiasis	Feb-09
72048	P20..14	2	0	0	0	Congenital endaural hernia	Feb-09
23752	P6yy900	3	0	0	0	Congenital epicardial cyst	Feb-09
65761	68A4.00	19	0	1	0	Congenital eye disorder screen	Feb-09
67169	P361z00	10	0	0	0	Congenital eyelid deformity NOS	Feb-09
70982	P4z..00	135	0	1	0	Congenital face or neck anomaly NOS	Feb-09
71051	PB5z000	4	0	0	0	Congenital faecal fistula	Feb-09
57813	Q466100	3	0	2	0	Congenital faecoliths causing obstruction	Feb-09
52576	Q402500	26	0	1	0	Congenital falciparum malaria	Feb-09
97903	PF37100	1	0	0	0	Congenital fibular deficiency type II	Oct-09
50307	PE8y600	31	0	2	0	Congenital flexion contracture of hip	Feb-09
29124	PE8y800	56	0	6	0	Congenital flexion contracture of knee	Feb-09
56631	PE8y300	15	0	1	0	Congenital flexion contractures of leg	Feb-09
24561	PG62.00	18	0	1	0	Congenital foramen Morgagni hernia	Feb-09
27309	PE80.11	100	0	8	0	Congenital funnel chest	Feb-09
45452	P602100	1	0	0	0	Congenital fusion of pulmonary valve segment	Feb-09
35806	PG33.00	57	0	1	0	Congenital fusion of ribs	Feb-09
67220	PF6y300	17	0	0	0	Congenital fusion of sacroiliac joint	Feb-09
68179	PH40200	0	0	1	0	Congenital generalised alopecia	Feb-09

For peer review only - http://bmjopen.bmj.com/site/about/guidelines.xhtml

44969	PC...00	38	0	1	0	Congenital genital organ anomalies	Feb-09
50367	PE40.00	23	0	1	0	Congenital genu recurvatum	Feb-09
23407	P320000	908	0	33	0	Congenital glaucoma	Feb-09
42855	P821.00	20	0	1	0	Congenital glottic web of larynx	Feb-09
247	P6z..00	2070	0	122	1	Congenital heart anomaly NOS	Feb-09
11982	P6zz.00	204	0	7	0	Congenital heart anomaly NOS	Feb-09
24533	P6y5.00	239	0	6	0	Congenital heart block	Feb-09
70992	P6y5z00	7	0	0	0	Congenital heart block NOS	Feb-09
68097	P6y5000	6	0	0	0	"Congenital heart block, unspecified"	Feb-09
72576	66g..00	391	0	36	0	Congenital heart condition monitoring	Feb-09
61935	L185.11	6	0	0	0	Congenital heart disease in pregnancy	Feb-09
23754	P5...12	332	0	5	0	"Congenital heart disease, septal and bulbar anomalies"	Feb-09
57298	D107000	3	0	11	11	Congenital Heinz-body anaemia	Feb-09
31853	PKyz600	471	0	26	0	Congenital hemihypertrophy	Feb-09
27966	F231.00	464	0	7	0	Congenital hemiplegia	Feb-09
34903	PB62.11	212	0	19	0	Congenital hepatic cyst	Feb-09
34642	Q48yz11	41	0	1	0	Congenital hepatic fibrosis	Feb-09
40762	Q409000	81	0	4	3	Congenital hepatitis A infection	Feb-09
50245	Q409100	121	0	4	1	Congenital hepatitis B infection	Feb-09
48738	PB6y100	10	0	0	0	Congenital hepatomegaly	Feb-09
101703	P11z.14	2	0	0	0	Congenital hernia of dura mater	Feb-11
43003	Q402000	86	0	2	0	Congenital herpes simplex	Feb-09
43215	PA6..00	234	0	1	0	Congenital hiatus hernia	Feb-09
2889	PF63100	2536	0	148	0	Congenital hip dysplasia	Feb-09
66951	P842.00	9	0	0	0	Congenital honeycomb lung	Feb-09
47776	PA71.00	11	0	1	0	Congenital hourglass stomach	Feb-09
62024	PF5y300	3	0	0	0	Congenital humeral varus	Feb-09
9611	P23..00	1376	0	78	0	Congenital hydrocephalus	Feb-09

For peer review only - http://bmjopen.bmj.com/site/about/guidelines.xhtml

107917	Q402311	2	0	0	0	Congenital hydrocephalus due to toxoplasmosis	Apr-14
28353	P23z.00	29	0	1	0	Congenital hydrocephalus NOS	Feb-09
5379	PD23.00	1375	0	24	0	Congenital hydronephrosis	Feb-09
26681	Q48y300	219	0	10	0	Congenital hypertonia	Feb-09
30154	PH42.00	55	0	11	0	Congenital hypertrichosis	Feb-09
101194	P355200	406	0	3	0	Congenital hypertrophy of retinal pigment epithelium	Dec-10
57161	C390500	27	0	1	0	Congenital hypogammaglobulinaemia	Feb-09
103611	P227100	2	0	0	0	Congenital hypoplasia of cerebrum	Jan-12
37320	D200000	69	0	9	0	Congenital hypoplastic anaemia	Feb-09
10097	C03..00	4305	0	109	28	Congenital hypothyroidism	Feb-09
51481	C03z.00	159	0	4	0	Congenital hypothyroidism NOS	Feb-09
31612	C03y000	39	0	0	0	Congenital hypothyroidism with diffuse goitre	Feb-09
93159	C03y100	2	0	0	0	Congenital hypothyroidism without goitre	Feb-09
8722	Q48y200	1837	0	35	0	Congenital hypotonia	Feb-09
57526	PH1z.11	7	0	0	0	Congenital ichthyosiform erythroderma	Feb-09
36468	PH10.00	133	0	2	0	"Congenital ichthyosis, unspecified"	Feb-09
102167	P6y5200	1	0	0	0	Congenital incomplete atrio-ventricular heart block	Apr-11
106080	Q40V.00	1	0	0	0	"Congenital infectious and parasitic disease, unspecified"	May-13
97269	PE31014	6	0	0	0	Congenital instability of hip joint	Aug-09
107150	P60z000	1	0	0	0	Congenital insufficiency of the pulmonary valve	Oct-13
67830	PB40.00	20	0	0	0	Congenital intestinal adhesions	Feb-09
69811	PB40z00	3	0	0	0	Congenital intestinal adhesions NOS	Feb-09
95632	P240.11	2	0	0	0	Congenital intracerebral cyst	Feb-09

For peer review only - http://bmjopen.bmj.com/site/about/guidelines.xhtml

96787	C0A..00	1	0	0	0	Congenital iodine deficiency syndrome	Apr-09
39166	C0A1.00	13	0	0	0	"Congenital iodine-deficiency syndrome, myxoedematous type"	Feb-09
72331	C0A0.00	1	0	0	0	"Congenital iodine-deficiency syndrome, neurological type"	Feb-09
23783	P340100	59	0	13	0	Congenital keratoconus	Feb-09
36991	PH3y300	216	0	9	0	Congenital keratoderma	Feb-09
49829	P322000	16	0	4	0	Congenital keratoglobus	Feb-09
50077	PF64z00	20	0	0	0	Congenital knee joint deformity NOS	Feb-09
32621	PH52.00	19	0	0	0	Congenital koilonychia	Feb-09
28717	PG18.11	368	0	26	0	Congenital kyphoscoliosis	Feb-09
30673	PC4yE00	526	0	35	0	Congenital labial adhesions	Feb-09
30468	P364.00	432	0	4	0	Congenital lacrimal passage anomalies	Feb-09
45351	P83y300	18	0	3	0	Congenital laryngocele	Feb-09
104555	P831700	58	0	1	0	Congenital laryngomalacia	Jul-12
49133	P6yy300	13	0	1	0	Congenital left ventricular diverticulum	Feb-09
41198	PE4..11	70	0	4	0	Congenital leg bone bowing	Feb-09
35351	P33..11	26	0	2	0	Congenital lens anomaly	Feb-09
95805	PH53.00	8	0	0	0	Congenital leukonychia	Feb-09
70280	Q402100	8	0	0	0	Congenital listeriosis	Feb-09
92345	PH40100	5	0	0	0	Congenital localised alopecia	Feb-09
29414	PG15.11	138	0	8	0	Congenital lumbosacral fusion	Feb-09
35729	PG11.00	97	0	2	0	Congenital lumbosacral spondylolysis	Feb-09
34693	B7J1.11	12	0	0	0	Congenital lymphangioma	Feb-09
27326	PH00.00	264	0	18	0	Congenital lymphoedema	Feb-09
94176	P322112	2	0	0	0	Congenital macrocornea	Feb-09
69316	Q402200	5	0	0	0	Congenital malaria	Feb-09

For peer review only - http://bmjopen.bmj.com/site/about/guidelines.xhtml

67260	PKy5z00	6	0	0	0	Congenital malform syndrome affecting facial appearance NOS	Feb-09
46825	P6W..00	33	0	3	0	Congenital malformation of aortic and mitral valves unsp	Feb-09
52658	PGX..00	33	0	3	0	"Congenital malformation of bony thorax, unspecified"	Feb-09
65092	PH6X.00	17	0	0	0	"Congenital malformation of breast, unspecified"	Feb-09
29738	P7W..00	39	0	1	0	"Congenital malformation of circulatory system, unspecif"	Feb-09
45910	P7X..00	63	0	0	0	"Congenital malformation of great arteries, unspecified"	Feb-09
47726	PB5X.00	12	0	0	0	"Congenital malformation of intestine, unspecified"	Feb-09
36474	P83yX00	22	0	2	0	"Congenital malformation of larynx, unspecified"	Feb-09
37965	PF5rD00	114	0	13	0	Congenital malformation of thumb	Feb-09
50529	P6X..00	19	0	0	0	"Congenital malformation of tricuspid valve, unspecified"	Feb-09
95151	PKy7z00	4	0	0	0	Congenital malformation syndrome involving limbs NOS	Feb-09
73575	PKy6z00	2	0	0	0	Congenital malformation syndrome with short stature NOS	Feb-09
100310	PKy8z00	2	0	0	0	Congenital malformation syndrome+other skeletal changes NOS	Jul-10
58981	PK8..00	13	0	1	0	Congenital malformation syndromes due to known exogen causes	Feb-09
45548	PKy7.00	5	0	0	0	Congenital malformation syndromes involving limbs	Feb-09

For peer review only - http://bmjopen.bmj.com/site/about/guidelines.xhtml

94910	PKy9.00	2	0	0	0	Congenital malformation syndromes with metabolic disturbance	Feb-09
97293	PKy8.00	4	0	0	0	Congenital malformation syndromes with other skeletal change	Aug-09
57953	PKy6.00	13	0	0	0	Congenital malformation syndromes with short stature	Feb-09
48205	P5X..00	33	0	0	0	Congenital malforms of cardiac chambers+connections unsp	Feb-09
39277	PBy2.00	11	0	2	1	Congenital malposition of digestive system NOS	Feb-09
38026	P322100	50	0	1	0	Congenital megalocornea	Feb-09
99424	P33y100	1	0	0	0	Congenital membranous cataract	May-10
41807	PE6z.11	37	0	2	0	Congenital metatarsus valgus	Feb-09
61651	P66..00	8	0	0	0	Congenital mitral insufficiency	Feb-09
57091	P65..00	34	0	2	0	Congenital mitral stenosis	Feb-09
100291	P65z.00	1	0	0	0	Congenital mitral stenosis NOS	Jul-10
46296	PH41.00	22	0	0	0	Congenital monilethrix	Feb-09
33925	F233.00	53	0	0	0	Congenital monoplegia	Feb-09
2709	PGz..00	778	0	46	1	Congenital musculoskeletal anomalies NOS	Feb-09
3298	PE...11	160	0	25	0	Congenital musculoskeletal deformities	Feb-09
33946	PEz..00	95	0	4	0	Congenital musculoskeletal deformity NOS	Feb-09
53113	D400500	14	0	0	0	Congenital neutropenia	Feb-09
40637	F486300	34	0	0	0	Congenital night blindness NOS	Feb-09
50096	F145.00	36	0	1	0	Congenital nonprogressive ataxia	Feb-09
49479	PB61200	13	0	2	0	Congenital obstruction of bile duct	Feb-09
65762	PB22.00	4	0	0	0	Congenital obstruction of large intestine	Feb-09

For peer review only - http://bmjopen.bmj.com/site/about/guidelines.xhtml

66309	PB12.00	20	0	0	0	Congenital obstruction of small intestine	Feb-09
34741	PD61.00	38	0	3	0	Congenital obstruction of urethra	Feb-09
100419	PD61z00	1	0	0	0	Congenital obstruction of urethra NOS	Aug-10
90483	PB23.00	6	0	0	0	Congenital occlusion of anus	Feb-09
62671	PB23z00	2	0	0	0	Congenital occlusion of anus NOS	Feb-09
101348	PB23000	1	0	1	0	Congenital occlusion of anus with fistula	Jan-11
31730	PA32.00	62	0	4	0	Congenital oesophageal fistula	Feb-09
95311	PA32z00	2	0	0	0	Congenital oesophageal fistula NOS	Feb-09
43558	PA45.00	158	0	20	0	Congenital oesophageal pouch	Feb-09
46814	PA3..11	6	0	0	0	Congenital oesophageal ring	Feb-09
49707	PA31.11	35	0	1	0	Congenital oesophageal stenosis	Feb-09
69684	PB40000	4	0	0	0	Congenital omental adhesions	Feb-09
66070	PH54.00	5	0	0	0	Congenital onychauxis	Feb-09
39392	P2xz100	139	0	4	0	Congenital optic atrophy	Feb-09
26024	P356000	95	0	2	0	Congenital optic disc coloboma	Feb-09
50282	P365.00	4	0	0	0	Congenital orbit anomalies	Feb-09
106909	PC0y.11	2	0	0	0	Congenital ovarian dysplasia	Aug-13
53673	PF6B200	1	0	0	0	Congenital overgrowth of foot	Feb-09
46652	PF6B.00	11	0	0	0	Congenital overgrowth of lower limb	Feb-09
35067	PH55.00	60	0	1	0	Congenital pachyonychia	Feb-09
37160	F230000	36	0	2	0	Congenital paraplegia	Feb-09
91259	PG15100	9	0	0	0	Congenital partial fusion of spine - balanced	Feb-09
53028	PG15200	3	0	0	0	Congenital partial fusion of spine - unbalanced	Feb-09
73595	P816.00	2	0	0	0	Congenital perforation of the nasal sinus wall	Feb-09
50362	P6yy400	14	0	0	0	Congenital pericardial defect	Feb-09

For peer review only - http://bmjopen.bmj.com/site/about/guidelines.xhtml

61062	P767.00	7	0	1	0	Congenital peripheral aneurysm	Feb-09
67420	PB40200	5	0	0	0	Congenital peritoneal adhesions	Feb-09
47616	PB40211	2	0	0	0	Congenital peritoneal bands	Feb-09
49742	PA27100	37	0	4	0	Congenital pharyngeal polyp	Feb-09
48046	PH32000	20	0	1	0	Congenital poikiloderma	Feb-09
18195	PB62000	212	0	17	0	Congenital polycystic liver disease	Feb-09
31820	P841.00	6	0	1	0	Congenital polycystic lung	Feb-09
23337	C371000	38	0	2	0	Congenital porphyria	Feb-09
11710	PD67.00	374	0	13	0	Congenital posterior urethral valves	Feb-09
69107	PD62.11	32	0	0	0	Congenital posterior urethral valves	Feb-09
38363	PE2z.11	16	0	1	0	Congenital postural curvature of spine NOS	Feb-09
100736	F591800	19	0	3	0	Congenital prelingual deafness	Sep-10
41025	PDy7.00	44	0	8	0	Congenital prolapse of bladder mucosa	Feb-09
47479	PDy8.00	29	0	2	0	Congenital prolapse of urethra	Feb-09
48936	P42z300	58	0	3	0	Congenital prominent auricle	Feb-09
48469	PF6y600	35	0	2	0	Congenital pseudarthrosis of tibia	Feb-09
44347	P8y4.00	16	0	0	0	Congenital pulmonary lymphangiectasis	Feb-09
22778	P602.00	832	0	10	0	Congenital pulmonary stenosis	Feb-09
33919	P602z00	152	0	0	0	Congenital pulmonary stenosis NOS	Feb-09
7225	D200312	18	0	0	0	Congenital pure red cell aplasia	Feb-09
38655	PB25.11	39	0	2	0	Congenital rectal stricture	Feb-09
61462	D200314	4	0	0	0	Congenital red cell hypoplasia	Feb-09
48260	PB52z12	26	0	1	0	Congenital redundant colon	Feb-09
68819	PB52z11	10	0	0	0	Congenital redundant rectal mucosa	Feb-09
27471	PD01.00	77	0	2	0	Congenital renal atrophy	Feb-09
17523	PD10.00	409	0	31	0	"Congenital renal cyst, single"	Feb-09
49791	P357000	39	0	1	0	Congenital retinal aneurysm	Feb-09
50584	P355100	14	0	0	0	Congenital retinal fold	Feb-09

For peer review only - http://bmjopen.bmj.com/site/about/guidelines.xhtml

48088	PG1x.00	44	0	1	0	Congenital sacrococcygeal anomalies NEC	Feb-09
70583	PG1xz00	12	0	0	0	Congenital sacrococcygeal anomaly NOS	Feb-09
49382	PA23.00	8	0	0	0	Congenital salivary gland fistula	Feb-09
60246	PH3y100	8	0	1	0	Congenital scar	Feb-09
10665	F591400	1042	0	25	0	Congenital sensorineural deafness	Feb-09
94734	PF34100	10	0	1	0	Congenital short femur	Feb-09
43330	D210000	5	0	0	0	Congenital sideroblastic anaemia	Feb-09
31007	PH...11	41	0	6	0	Congenital skin anomalies	Feb-09
61879	PE8y400	1	0	0	0	Congenital spade-like hand	Feb-09
15530	F23..11	644	0	34	0	Congenital spastic cerebral palsy	Feb-09
55593	F233.11	16	0	0	0	Congenital spastic foot	Feb-09
36974	P2y1.00	46	0	1	0	Congenital spinal cord anomaly	Feb-09
22012	PE2..00	660	0	11	0	Congenital spine deformity	Feb-09
29412	PE2z.00	96	0	4	0	Congenital spine deformity NOS	Feb-09
49933	PE20.00	29	0	2	0	"Congenital spine deformity, unspecified"	Feb-09
59741	PK03.00	36	0	0	0	Congenital splenomegaly	Feb-09
1871	PG12.00	1315	0	76	0	Congenital spondylolisthesis	Feb-09
100539	PB24011	2	0	0	0	Congenital stenosis of anus with fistula	Aug-10
49089	PB24111	6	0	0	0	Congenital stenosis of anus without mention of fistula	Feb-09
58189	P722411	3	0	0	0	Congenital stenosis of ascending aorta	Feb-09
71893	P833200	4	0	1	0	Congenital stenosis of bronchus	Feb-09
32484	PC4y800	82	0	3	0	Congenital stenosis of cervical canal	Feb-09
20686	PB13000	47	0	0	0	Congenital stenosis of duodenum	Feb-09
101739	P423100	1	0	0	0	Congenital stenosis of eustachian tube	Feb-11
69712	PB13200	9	0	0	0	Congenital stenosis of ileum	Feb-09

For peer review only - http://bmjopen.bmj.com/site/about/guidelines.xhtml

67021	PB13100	6	0	0	0	Congenital stenosis of jejunum	Feb-09
108445	PB22.11	1	0	0	0	Congenital stenosis of large intestine	Aug-14
61450	P833000	6	0	0	0	Congenital stenosis of larynx	Feb-09
59273	P833.00	5	0	0	0	"Congenital stenosis of larynx, trachea and bronchus"	Feb-09
101122	PB25111	1	0	0	0	Congenital stenosis of rectum without mention of fistula	Dec-10
59399	PB13.00	4	0	0	0	Congenital stenosis of small intestine	Feb-09
99366	PB13z00	1	0	0	0	Congenital stenosis of small intestine NOS	May-10
107172	P803.00	1	0	0	0	Congenital stenosis of the anterior nares	Oct-13
96389	P804.00	5	0	0	0	Congenital stenosis of the posterior nares	Apr-09
48818	P833100	11	0	0	0	Congenital stenosis of trachea	Feb-09
50820	PD22.11	34	0	0	0	Congenital stenosis of ureter	Feb-09
37200	PC4y900	28	0	7	0	Congenital stenosis of vagina	Feb-09
65337	PA73.00	9	0	0	0	Congenital stomach diverticulum	Feb-09
48020	PB24.00	15	0	0	0	Congenital stricture of anus	Feb-09
73497	PB24z00	1	0	0	0	Congenital stricture of anus NOS	Feb-09
41359	PB24000	3	0	0	0	Congenital stricture of anus with fistula	Feb-09
107117	PB24100	3	0	0	0	Congenital stricture of anus without mention of fistula	Oct-13
95810	PB61300	8	0	0	0	Congenital stricture of bile duct	Feb-09
32803	PB61311	31	0	0	0	Congenital stricture of common bile duct	Feb-09
103412	P6y4600	2	0	0	0	Congenital stricture of coronary artery	Dec-11

For peer review only - http://bmjopen.bmj.com/site/about/guidelines.xhtml

For peer review only

102497	P402111	1	0	0	0	Congenital stricture of external auditory canal	Jun-11
95103	P402112	1	0	0	0	Congenital stricture of osseous meatus	Feb-09
111389	P735.11	1	0	0	0	Congenital stricture of pulmonary artery	Oct-16
49501	PB25.00	29	0	1	0	Congenital stricture of rectum	Feb-09
42469	PB25z00	5	0	0	0	Congenital stricture of rectum NOS	Feb-09
69054	PB25000	1	0	0	0	Congenital stricture of rectum with fistula	Feb-09
47939	PB13z11	4	0	0	0	Congenital stricture of small intestine	Feb-09
33355	PD22.00	32	0	2	0	Congenital stricture of ureter	Feb-09
69191	PD22z00	10	0	2	0	Congenital stricture of ureter NOS	Feb-09
97464	PD22000	3	0	0	0	"Congenital stricture of ureter, unspecified"	Aug-09
27646	PD22100	17	0	0	0	Congenital stricture of ureteropelvic junction	Feb-09
96829	PD22200	2	0	0	0	Congenital stricture of ureterovesical orifice	May-09
62647	PC4y911	2	0	0	0	Congenital stricture of vagina	Feb-09
49142	P833300	20	0	0	0	Congenital subglottic stenosis	Feb-09
97956	P822.00	2	0	0	0	Congenital subglottic web of larynx	Nov-09
62586	PE31z00	76	0	0	0	Congenital subluxation of hip NOS	Feb-09
100627	F23y511	7	0	0	0	Congenital suprabulbar paresis	Sep-10
55144	P833400	12	0	1	0	Congenital supraglottic stenosis	Feb-09
99687	PF69000	2	0	0	0	Congenital synostosis of lower limb bones	Jun-10
27420	A90..00	272	0	8	0	Congenital syphilis	Feb-09
60039	A90z.00	6	0	0	0	Congenital syphilis NOS	Feb-09
50581	A900.12	4	0	0	0	Congenital syphilitic choroiditis	Feb-09
69219	A900.14	2	0	0	0	Congenital syphilitic epiphysitis	Feb-09

For peer review only - http://bmjopen.bmj.com/site/about/guidelines.xhtml

46401	A905000	10	0	0	0	Congenital syphilitic gumma	Feb-09
57279	A904200	1	0	0	0	Congenital syphilitic meningitis	Feb-09
22157	A900.16	34	0	6	1	Congenital syphilitic osteochondritis	Feb-09
7815	PE5y000	483	0	8	0	Congenital talipes calcaneovarus	Feb-09
38386	PE7y100	138	0	2	0	Congenital talipes calcaneus	Feb-09
31013	PE6y000	194	0	13	0	Congenital talipes equinovalgus	Feb-09
156	PE51.00	7134	0	439	0	Congenital talipes equinovarus	Feb-09
4334	PE7y200	411	0	25	0	Congenital talipes equinus	Feb-09
29808	PE60.00	589	0	41	0	Congenital talipes valgus	Feb-09
22846	PE50.00	1392	0	155	0	Congenital talipes varus	Feb-09
48940	P252.00	40	0	1	0	Congenital tethering of spinal cord	Feb-09
35125	PE8y111	14	0	0	0	Congenital thoracic wall deformity NEC	Feb-09
55582	D313100	7	0	0	0	Congenital thrombocytopenic purpura	Feb-09
93323	C03z.11	4	0	1	0	Congenital thyroid insufficiency	Feb-09
104835	PF36200	1	0	0	0	Congenital tibial deficiency type III	Aug-12
35042	PA32111	46	0	1	0	Congenital tracheo-oesophageal fistula	Feb-09
12752	P61..00	81	0	1	0	Congenital tricuspid atresia and stenosis	Feb-09
100065	P61z.00	2	0	0	0	Congenital tricuspid atresia or stenosis NOS	Jul-10
69169	P611.00	6	0	0	0	Congenital tricuspid stenosis	Feb-09
55957	Q402400	7	0	0	0	Congenital tuberculosis	Feb-09
110450	PG73.00	34	0	2	0	Congenital umbilical hernia	Jan-16
64939	PF6C100	2	0	0	0	Congenital undergrowth of distal part of limb	Feb-09
62450	PF6C.00	10	0	1	0	Congenital undergrowth of lower limb	Feb-09
54180	PF6C000	2	0	1	0	Congenital undergrowth of proximal part of limb	Feb-09

For peer review only - http://bmjopen.bmj.com/site/about/guidelines.xhtml

59736	PD21.11	13	0	1	0	Congenital ureteric valves	Feb-09
41342	PD62.00	43	0	0	0	Congenital urethral valvular stricture	Feb-09
29419	PDy6.00	96	0	19	0	Congenital urethrorectal fistula	Feb-09
67371	PD63.11	1	0	0	0	Congenital urinary meatus obstruction	Feb-09
31985	PD63.00	73	0	4	0	Congenital urinary meatus stricture	Feb-09
71832	PD63z00	7	0	0	0	Congenital urinary meatus stricture NOS	Feb-09
30143	PC4yC00	274	0	50	0	Congenital vaginal cyst NEC	Feb-09
36791	PF6y500	419	0	49	0	Congenital valgus ankle	Feb-09
35208	P76A.00	97	0	10	0	Congenital varix	Feb-09
36277	PF6y400	76	0	14	0	Congenital varus ankle	Feb-09
50065	PE61000	155	0	2	0	Congenital vertical talus	Feb-09
24918	PD47.00	408	0	8	0	Congenital vesico-uretero-renal reflux	Feb-09
104830	PD64.00	1	0	0	0	Congenital vesicourethral orifice stricture	Aug-12
93368	Q40X.00	4	0	0	0	"Congenital viral disease, unspecified"	Feb-09
67779	Q409.00	22	0	2	0	Congenital viral hepatitis	Feb-09
107622	Q409z00	3	0	0	0	Congenital viral hepatitis NOS	Jan-14
49448	PB5z.11	33	0	1	0	Congenital volvulus	Feb-09
31771	P82..00	87	0	1	0	Congenital web of larynx	Feb-09
70985	P82z.00	4	0	0	0	Congenital web of larynx NOS	Feb-09
111186	P820.00	1	0	0	0	"Congenital web of larynx, unspecified"	Aug-16
35616	P45..00	41	0	6	0	Congenital webbing of neck	Feb-09
99458	P45z.00	1	0	0	0	Congenital webbing of neck NOS	May-10
65125	PE1..11	5	0	1	0	Congenital wry neck	Feb-09
60880	C390512	18	0	1	0	Congenital X-linked agammaglobulinaemia	Feb-09

For peer review only - http://bmjopen.bmj.com/site/about/guidelines.xhtml

68766	G554011	10	0	0	0	Congestive obstructive cardiomyopathy	Feb-09
65402	C1z4100	8	0	1	0	Constitutional dwarfism	Feb-09
72252	D200300	4	0	1	2	Constitutional red cell aplasia and hypoplasia	Feb-09
47438	D200313	17	0	1	0	Constitutional red cell hypoplasia	Feb-09
38896	PF5E000	21	0	9	0	Constriction ring	Feb-09
93784	PF6D000	2	0	0	0	Constriction ring of lower limb	Feb-09
67293	PF6D.00	9	0	0	0	Constriction ring syndrome of lower limb	Feb-09
28775	PF6D300	1	0	0	0	Constriction ring syndrome of lower limb with amputation	Feb-09
24053	PF5E.00	32	0	3	0	Constriction ring syndrome of upper limb	Feb-09
104024	PF5E400	4	0	0	0	Constriction ring with acrosyndactyly and amputation	May-12
93038	PF5E100	2	0	2	0	Constriction ring with lymphoedema	Feb-09
65807	G532z00	15	0	0	0	Constrictive pericarditis NOS	Feb-09
25194	C294200	13	0	1	0	Copper deficiency	Feb-09
94908	4Q73.00	30	0	0	66	Copper level	Feb-09
3113	C154.00	712	0	30	1	Corticoadrenal insufficiency	Feb-09
12396	C154z00	99	0	0	0	Corticoadrenal insufficiency NOS	Feb-09
50508	PKy5B00	8	0	0	0	Costello syndrome	Feb-09
26444	J046100	273	0	7	0	Costen's syndrome	Feb-09
16897	PG03.00	1078	0	77	1	Craniosynostosis	Feb-09
27533	C03z.12	91	0	2	0	Cretinism	Feb-09
67513	C03..11	2	0	0	0	Cretinism	Feb-09
31795	PJ31.00	123	0	2	0	Cri-du-chat syndrome	Feb-09
46738	C374000	85	0	3	0	Crigler - Najjar syndrome	Feb-09
103821	13s7.00	3	0	0	0	Croatian as a second language	Feb-12
6538	J401z11	3927	0	94	0	Crohn's colitis	Feb-09

For peer review only - http://bmjopen.bmj.com/site/about/guidelines.xhtml

593	J40..11	100098	0	5433	53	Crohn's disease	Feb-09
59994	J40z.11	517	0	0	0	Crohn's disease NOS	Feb-09
39278	J400400	94	0	0	0	Crohn's disease of the ileum NOS	Feb-09
66238	J400300	48	0	0	0	Crohn's disease of the ileum unspecified	Feb-09
20688	J401z00	282	0	7	0	Crohn's disease of the large bowel NOS	Feb-09
9359	J400z00	209	0	0	0	Crohn's disease of the small bowel NOS	Feb-09
28476	J400200	582	0	3	0	Crohn's disease of the terminal ileum	Feb-09
25783	PG04.11	118	0	5	0	Crouzon's disease	Feb-09
101374	B7J1000	20	0	1	0	Cystic hygroma of neck	Jan-11
9240	PD11z11	495	0	48	0	Cystic kidney disease NEC	Feb-09
32371	C300200	9	0	0	0	Cystinaemia	Feb-09
60390	C300311	8	0	0	0	Cystine storage disease	Feb-09
27435	C300100	687	0	27	1	Cystinuria	Feb-09
3747	F593.00	1052	0	20	0	"Deaf mutism, NEC"	Feb-09
108703	Fy1..12	2	0	0	0	Deafblind	Oct-14
31374	P40z.11	259	0	5	0	Deafness due to congenital anomaly NEC	Feb-09
686	F59z.00	84378	0	26079	5	Deafness NOS	Feb-09
36588	D303100	91	0	1	0	Deficiency of factor II or prothrombin	Feb-09
15556	D303200	127	0	15	7	Deficiency of factor V or labile factor	Feb-09
45637	D303300	85	0	5	0	Deficiency of factor VII or stable factor	Feb-09
47198	D303400	32	0	1	1	Deficiency of factor X or Stuart-Prower factor	Feb-09
34673	D303500	172	0	13	5	Deficiency of factor XII or Hageman factor	Feb-09

For peer review only - http://bmjopen.bmj.com/site/about/guidelines.xhtml

20174	D303600	23	0	2	2	Deficiency of factor XIII or fibrin stabilizing factor	Feb-09
67298	PJ33000	5	0	0	0	Deletion of long arm of chromosome 13	Feb-09
95440	PJ33100	3	0	0	0	Deletion of long arm of chromosome 18	Feb-09
57806	PJ30.11	4	0	0	0	Deletion of long arm of chromosome 21	Feb-09
97927	PJ33200	4	0	0	0	Deletion of short arm of chromosome 18	Oct-09
66566	PJ32.00	7	0	0	0	Deletion of short arm of chromosome 4	Feb-09
98941	PJ31.11	6	0	0	0	Deletion of short arm of chromosome 5	Apr-10
32943	D104900	30	0	0	0	Delta-beta thalassaemia	Feb-09
106927	PKyP.00	2	0	0	0	"Diab insipidus,diab mell,optic atrophy and deafness"	Aug-13
61670	889A.00	10	0	0	0	Diab mellit insulin-glucose infus acute myocardial infarct	Feb-09
1045	C135.00	3172	0	198	2	Diabetes insipidus	Feb-09
60046	C135.12	12	0	0	0	Diabetes insipidus - pituitary	Feb-09
711	C10..00	813923	0	39051	4	Diabetes mellitus	Feb-09
43453	C10C.00	35	0	0	0	Diabetes mellitus autosomal dominant	Feb-09
36695	C10D.00	339	0	4	0	Diabetes mellitus autosomal dominant type 2	Feb-09
61122	C10H.00	38	0	0	0	Diabetes mellitus induced by non-steroid drugs	Feb-09
72345	C102z00	7	0	0	0	Diabetes mellitus NOS with hyperosmolar coma	Feb-09
42505	C101z00	376	0	8	0	Diabetes mellitus NOS with ketoacidosis	Feb-09

For peer review only - http://bmjopen.bmj.com/site/about/guidelines.xhtml

65062	C103z00	13	0	0	0	Diabetes mellitus NOS with ketoacidotic coma	Feb-09
22573	C106z00	257	0	1	0	Diabetes mellitus NOS with neurological manifestation	Feb-09
50972	C100z00	586	0	7	0	Diabetes mellitus NOS with no mention of complication	Feb-09
34283	C105z00	165	0	0	0	Diabetes mellitus NOS with ophthalmic manifestation	Feb-09
70821	C10yz00	15	0	0	0	Diabetes mellitus NOS with other specified manifestation	Feb-09
65025	C107z00	77	0	3	0	Diabetes mellitus NOS with peripheral circulatory disorder	Feb-09
64357	C10zz00	113	0	0	0	Diabetes mellitus NOS with unspecified complication	Feb-09
32403	C107.11	275	0	50	0	Diabetes mellitus with gangrene	Feb-09
21482	C102.00	220	0	34	0	Diabetes mellitus with hyperosmolar coma	Feb-09
1682	C101.00	13381	0	2152	8	Diabetes mellitus with ketoacidosis	Feb-09
15690	C103.00	353	0	58	0	Diabetes mellitus with ketoacidotic coma	Feb-09
35107	C104z00	306	0	10	0	Diabetes mellitus with nephropathy NOS	Feb-09
7795	C106.12	9335	0	219	0	Diabetes mellitus with neuropathy	Feb-09
38986	C100.00	2877	0	34	0	Diabetes mellitus with no mention of complication	Feb-09
33254	C105.00	1791	0	24	0	Diabetes mellitus with ophthalmic manifestation	Feb-09
33343	C10y.00	72	0	1	0	Diabetes mellitus with other specified manifestation	Feb-09
35399	C107.00	3910	0	28	0	Diabetes mellitus with peripheral circulatory disorder	Feb-09

For peer review only - http://bmjopen.bmj.com/site/about/guidelines.xhtml

16491	C106.13	67	0	0	0	Diabetes mellitus with polyneuropathy	Feb-09
16502	C104.00	1441	0	101	0	Diabetes mellitus with renal manifestation	Feb-09
45491	C10z.00	83	0	3	0	Diabetes mellitus with unspecified complication	Feb-09
70448	C107000	2	0	0	0	"Diabetes mellitus, juvenile +peripheral circulatory disorder"	Feb-09
69748	C105000	6	0	0	0	"Diabetes mellitus, juvenile type, + ophthalmic manifestation"	Feb-09
68792	C10z000	7	0	0	0	"Diabetes mellitus, juvenile type, + unspecified complication"	Feb-09
24490	C100000	1681	0	50	0	"Diabetes mellitus, juvenile type, no mention of complication"	Feb-09
40023	C102000	8	0	0	0	"Diabetes mellitus, juvenile type, with hyperosmolar coma"	Feb-09
53200	C101000	108	0	4	0	"Diabetes mellitus, juvenile type, with ketoacidosis"	Feb-09
42567	C103000	6	0	0	0	"Diabetes mellitus, juvenile type, with ketoacidotic coma"	Feb-09
93922	C104000	5	0	0	0	"Diabetes mellitus, juvenile type, with renal manifestation"	Feb-09
67853	C106000	76	0	0	0	"Diabetes mellitus, juvenile, + neurological manifestation"	Feb-09
110997	C10y000	1	0	0	0	"Diabetes mellitus, juvenile, + other specified manifestation"	Jul-16
13071	66AI.00	59237	0	557	73	Diabetic - good control	Feb-09
2378	66AJ.00	135053	0	8917	27	Diabetic - poor control	Feb-09
22023	66AJz00	1614	0	42	0	Diabetic - poor control NOS	Feb-09
8842	66A5.00	73497	0	162	0	Diabetic on insulin	Feb-09
111483	66o6.00	1	0	0	0	Diabetic on insulin and glucagon-like peptide 1	Nov-16

For peer review only - http://bmjopen.bmj.com/site/about/guidelines.xhtml

28769	66AV.00	15408	0	21	0	Diabetic on insulin and oral treatment	Feb-09
110344	66o2.00	41	0	0	0	Diabetic on non-insulin injectable medication	Dec-15
1684	66A4.00	230233	0	445	3	Diabetic on oral treatment	Feb-09
110379	66o5.00	47	0	0	0	Diabetic on oral treatment and glucagon-like peptide 1	Dec-15
101728	66As.00	638	0	0	0	Diabetic on subcutaneous treatment	Feb-11
4987	J34..00	25672	0	508	0	Diaphragmatic hernia	Feb-09
60697	J342.00	30	0	2	2	Diaphragmatic hernia - irreducible	Feb-09
11780	J34z.00	3413	0	10	0	Diaphragmatic hernia NOS	Feb-09
24703	J340.00	12	0	0	0	Diaphragmatic hernia with gangrene	Feb-09
44233	J341.00	16	0	0	0	Diaphragmatic hernia with obstruction	Feb-09
298	13VC.00	16755	0	383	0	Disability	Feb-09
23369	13VC100	439	0	5	0	Disability - moderate	Feb-09
3972	13VC200	248	0	8	0	Disability - severe	Feb-09
27462	13VC000	363	0	2	0	Disability - slight	Feb-09
41742	13VCZ00	531	0	7	0	Disability NOS	Feb-09
3058	13VC.11	4335	0	104	0	Disabled	Feb-09
42815	C313700	681	0	0	0	Disaccharidase deficiency	Feb-09
2667	M154100	6944	0	421	0	Discoid lupus erythematosus	Feb-09
52080	C375X00	5	0	1	0	"Disorder of glucosaminoglycan metabolism, unspecified"	Feb-09
65382	C31yX00	8	0	0	1	"Disorder of glycoprotein metabolism, unspecified"	Feb-09
66726	F391500	8	0	1	0	Distal (Gower's) muscular dystrophy	Feb-09
46117	P58..00	31	0	0	0	Double outlet left ventricle	Feb-09
1778	P511.00	37	0	0	0	Double outlet right ventricle	Feb-09
65318	P511z00	4	0	0	0	Double outlet right ventricle NOS	Feb-09

For peer review only - http://bmjopen.bmj.com/site/about/guidelines.xhtml

1543	PJ0..00	10552	0	1045	780	Down's syndrome - trisomy 21	Feb-09
10759	PJ0z.00	1447	0	13	0	Down's syndrome NOS	Feb-09
15661	G310.11	529	0	16	0	Dressler's syndrome	Feb-09
7766	F4J7000	2490	0	74	0	Duane's syndrome	Feb-09
39293	C374100	106	0	1	0	Dubin - Johnson syndrome	Feb-09
57043	PKy6600	23	0	0	0	Dubowitz syndrome	Feb-09
7470	F152111	50	0	5	0	Duchenne Aran muscular atrophy	Feb-09
108940	PD80.00	243	0	2	0	Duplex kidney	Dec-14
4431	PD34.11	6973	0	218	2	Duplex kidneys	Feb-09
5006	PG41.11	179	0	6	0	Dwarfism	Feb-09
34706	C1z4.00	30	0	0	0	Dwarfism NEC	Feb-09
66781	C1z4z00	1	0	0	0	Dwarfism NEC NOS	Feb-09
4451	PGy2.00	2654	0	259	0	Ehlers-Danlos syndrome	Feb-09
63549	PGy2000	12	0	0	0	Ehlers-Danlos syndrome type I	Feb-09
72787	PGy2100	17	0	0	0	Ehlers-Danlos syndrome type II	Feb-09
28335	PGy2200	143	0	3	0	Ehlers-Danlos syndrome type III	Feb-09
70415	PGy2300	13	0	0	0	Ehlers-Danlos syndrome type IV	Feb-09
53984	PGy2500	3	0	0	0	Ehlers-Danlos syndrome type VI	Feb-09
95643	PGy2600	1	0	0	0	Ehlers-Danlos syndrome type VII	Feb-09
98263	PGy2700	1	0	0	0	Ehlers-Danlos syndrome type VIII	Dec-09
68007	PG55.11	10	0	0	0	Ellis - Van Creveld syndrome	Feb-09
34985	F391A00	32	0	5	0	Emery-Dreifuss muscular dystrophy	Feb-09
37798	PH3y700	257	0	11	0	Epidermolysis bullosa simplex	Feb-09
573	F25..00	391517	0	20275	98	Epilepsy	Feb-09
50012	6674	226	0	6	0	Epilepsy associated problems	Feb-09
22341	1O30.00	1754	0	42	0	Epilepsy confirmed	Feb-09
9747	F25z.00	11242	0	290	1	Epilepsy NOS	Feb-09
1715	F250011	3524	0	1055	1	Epileptic absences	Feb-09
31830	F250300	199	0	17	0	Epileptic seizures - akinetic	Feb-09
24309	F250200	504	0	74	0	Epileptic seizures - atonic	Feb-09
18471	F251200	3391	0	199	0	Epileptic seizures - clonic	Feb-09
4801	F251300	1430	0	80	0	Epileptic seizures - myoclonic	Feb-09

For peer review only - http://bmjopen.bmj.com/site/about/guidelines.xhtml

5152	F251400	3665	0	259	0	Epileptic seizures - tonic	Feb-09
48036	F391100	77	0	2	0	Erb's muscular dystrophy	Feb-09
799	G20..00	2780814	0	16008	35	Essential hypertension	Feb-09
24161	C327411	219	0	15	1	Fabry's disease	Feb-09
35717	C327.12	45	0	3	1	Fabry's disease	Feb-09
24384	K032400	87	0	13	0	Familial glomerulonephritis in Alport's syndrome	Feb-09
3386	C320000	10075	0	265	0	Familial hypercholesterolaemia	Feb-09
3484	C320.11	890	0	3	0	Familial hypercholesterolaemia	Feb-09
23515	C300300	72	0	3	0	Fanconi-de-Toni syndrome	Feb-09
64539	PC72.00	6	0	0	0	Female pseudohermaphroditism	Feb-09
57081	PK84.00	69	0	2	0	Fetal valproate syndrome	Feb-09
39085	F220.00	89	0	12	0	Flaccid hemiplegia	Feb-09
104828	F23y100	1	0	0	0	Flaccid infantile cerebral palsy	Aug-12
46175	F241000	59	0	1	0	Flaccid paraplegia	Feb-09
5525	F255011	3632	0	208	1	Focal epilepsy	Feb-09
107814	K032200	1	0	0	0	Focal glomerulon + focal recurr macroscop glomerulonephritis	Mar-14
105180	F1y0.00	5	0	0	0	Fragile X associated tremor ataxia syndrome	Nov-12
67264	P205.00	8	0	0	0	Frontal encephalocele	Feb-09
65371	C311100	13	0	0	0	Galactokinase deficiency	Feb-09
5561	C311.00	317	0	10	5	Galactosaemia	Feb-09
64321	C311z00	9	0	0	0	Galactosaemia NOS	Feb-09
20730	C311000	9	0	0	0	Galactose-1-phosphate uridyl transferase deficiency	Feb-09
15593	PG71.00	1268	0	17	0	Gastroschisis	Feb-09
4659	E200200	45728	0	1707	0	Generalised anxiety disorder	Feb-09
26144	F251.00	1454	0	13	0	Generalised convulsive epilepsy	Feb-09
40806	F251z00	290	0	1	0	Generalised convulsive epilepsy NOS	Feb-09

For peer review only - http://bmjopen.bmj.com/site/about/guidelines.xhtml

44252	F250z00	82	0	1	0	Generalised nonconvulsive epilepsy NOS	Feb-09
106571	F25H.00	413	0	4	0	Generalised seizure	Aug-13
21934	C310212	179	0	4	0	Glucose-6-phosphatase deficiency	Feb-09
22531	D102000	524	0	265	245	Glucose-6-phosphate dehydrogenase deficiency anaemia	Feb-09
101224	F28y400	11	0	0	0	GLUT1 deficiency syndrome	Dec-10
22111	C309.00	9	0	0	0	Glutaryl CoA dehydrogenase deficiency	Feb-09
24186	C310.13	174	0	9	0	Glycogen storage disease	Feb-09
31383	C310.00	104	0	10	2	Glycogenosis - glycogen storage disease	Feb-09
988	F251000	18606	0	1210	2	Grand mal (major) epilepsy	Feb-09
5668	F251600	19046	0	771	2	Grand mal seizure	Feb-09
5117	F253.00	981	0	31	0	Grand mal status	Feb-09
5257	C020.12	19532	0	614	2	Graves' disease	Feb-09
2983	C350000	10217	0	1033	7	Haemochromatosis	Feb-09
31662	D107600	2	0	0	0	Haemoglobin Zurich disease	Feb-09
31800	D107300	132	0	6	5	Haemoglobin-C disease	Feb-09
7624	D107400	104	0	1	1	Haemoglobin-D disease	Feb-09
32373	D107500	155	0	2	0	Haemoglobin-E disease	Feb-09
7526	D107700	78	0	3	7	Haemoglobin-H disease	Feb-09
71808	D102.00	1	0	0	0	Haemolytic anaemia due to glutathione metabolism disorder	Feb-09
107820	D103000	2	0	0	0	Haemolytic anaemia due to hexokinase deficiency	Mar-14
55561	D103100	37	0	0	0	Haemolytic anaemia due to pyruvate kinase deficiency	Feb-09
3326	D1...00	3122	0	188	2	Haemolytic anaemias	Feb-09
18631	D1z..00	297	0	1	0	Haemolytic anaemias NOS	Feb-09
29323	D111300	1111	0	21	0	Haemolytic-uraemic syndrome	Feb-09

For peer review only - http://bmjopen.bmj.com/site/about/guidelines.xhtml

51041	C37y800	48	0	2	0	Haemophagocytic lymphohistiocytosis	Feb-09
27934	C37y900	17	0	0	0	"Haemophagocytic syndrome, infection-associated"	Feb-09
2833	D300.12	1334	0	55	0	Haemophilia A	Feb-09
31166	D301.12	285	0	4	0	Haemophilia B	Feb-09
56664	D302.11	58	0	1	0	Haemophilia C	Feb-09
5632	D300.00	1516	0	85	2	Haemophilia-A (factor VIII deficiency)	Feb-09
22706	D302.00	392	0	13	1	Haemophilia-C (factor XI deficiency)	Feb-09
69377	PG0E.11	3	0	0	0	Hallerman - Streif syndrome	Feb-09
49217	C300400	12	0	0	0	Hartnup disease	Feb-09
4549	G56..12	16631	0	1578	1	Heart block	Feb-09
95785	P6yyA00	1	0	0	0	Hemicardia	Feb-09
110165	P002.00	2	0	0	0	Hemicephaly	Oct-15
91177	7J02C00	22	0	0	0	Hemicraniotomy	Feb-09
108683	M21yD00	5	0	0	0	Hemifacial atrophy	Oct-14
9371	PE00000	160	0	4	0	Hemifacial microsomia	Feb-09
95697	P24A.00	10	0	0	0	Hemimegalencephaly	Feb-09
807	F22..11	15097	0	1791	0	Hemiparesis	Feb-09
65275	F2Az.00	136	0	1	0	Hemiparesis NOS	Feb-09
1749	F22..00	17585	0	2027	0	Hemiplegia	Feb-09
8492	F22z.00	2414	0	185	0	Hemiplegia NOS	Feb-09
3658	F26y000	6343	0	358	0	Hemiplegic migraine	Feb-09
97623	N093.11	1	0	0	0	Hench - Rosenberg syndrome	Sep-09
25383	J61y400	454	0	4	0	Hepatic fibrosis	Feb-09
71453	J615z15	24	0	0	0	Hepatic fibrosis	Feb-09
100592	J61y600	3	0	0	0	Hepatic fibrosis with hepatic sclerosis	Aug-10
2413	A70z000	7597	0	1138	458	Hepatitis C	Feb-09
10636	J624.00	407	0	14	0	Hepatorenal syndrome	Feb-09

For peer review only - http://bmjopen.bmj.com/site/about/guidelines.xhtml

34092	F1...00	129	0	7	0	Hereditary and degenerative diseases of the CNS	Feb-09
56006	F1z..00	32	0	0	0	Hereditary and degenerative diseases of the CNS NOS	Feb-09
61968	F1y..00	24	0	1	0	Hereditary and degenerative diseases of the CNS OS	Feb-09
101075	SN51100	31	0	6	0	Hereditary C1 esterase inhibitor deficiency	Dec-10
64654	F435.00	7	0	0	0	Hereditary choroid dystrophies	Feb-09
31384	C312.00	58	0	209	208	Hereditary fructose intolerance	Feb-09
14698	D10z.00	560	0	56	2	Hereditary haemolytic anaemia NOS	Feb-09
39456	D10..00	83	0	1	0	Hereditary haemolytic anaemias	Feb-09
4942	G770.00	1536	0	154	0	Hereditary haemorrhagic telangiectasia	Feb-09
46726	D311000	19	0	0	0	Hereditary haemorrhagic thrombasthenia	Feb-09
32527	F368.00	431	0	12	0	Hereditary motor and sensory neuropathy	Feb-09
56910	F368000	54	0	0	0	Hereditary motor and sensory neuropathy type I	Feb-09
35465	F368100	259	0	0	0	Hereditary motor and sensory neuropathy type II	Feb-09
106103	F368200	2	0	0	0	Hereditary motor and sensory neuropathy type III	May-13
51113	K0A5000	4	0	0	0	"Hereditary nephropathy NEC, minor glomerular abnormality"	Feb-09
41239	K0A5100	2	0	0	0	"Hereditary nephropathy NEC,focal+segmnt glomerular lesion"	Feb-09
36205	K0A5.00	44	0	2	0	Hereditary nephropathy not elsewhere classified	Feb-09

For peer review only - http://bmjopen.bmj.com/site/about/guidelines.xhtml

91738	K0A5600	1	0	0	0	"Hereditary nephropathy, NEC, dense deposit disease"	Feb-09
62980	K0A5X00	4	0	0	0	"Hereditary nephropathy, unspecif morphological changes"	Feb-09
68118	F391.00	11	0	0	0	Hereditary progressive muscular dystrophy	Feb-09
22727	F427.00	143	0	6	0	Hereditary retinal dystrophies	Feb-09
97370	F427200	2	0	0	0	Hereditary retinal dystrophies with other diseases	Aug-09
50363	F427z00	531	0	139	0	Hereditary retinal dystrophy NOS	Feb-09
3514	F141.00	748	0	62	0	Hereditary spastic paraplegia	Feb-09
7237	D100.00	3206	0	135	9	Hereditary spherocytosis	Feb-09
58906	D313111	13	0	0	0	Hereditary thrombocytopenia NEC	Feb-09
44270	K0A5200	12	0	0	0	"Hereditry nephropathy NEC,difus membran glomerulnephritis"	Feb-09
61861	C302900	20	0	1	0	Hermansky-Pudlak syndrome	Feb-09
8546	PB30.00	2034	0	82	0	Hirschsprung's disease	Feb-09
56630	PB3..00	17	0	0	0	Hirschsprung's disease and allied congenital conditions	Feb-09
59085	PB3z.00	4	0	0	0	Hirschsprung's disease and allied congenital conditions NOS	Feb-09
70582	PB30z00	65	0	1	0	Hirschsprung's disease NOS	Feb-09
43907	C305000	23	0	0	0	Histidinaemia	Feb-09
55198	C305200	5	0	0	0	Histidinuria	Feb-09
4870	B625.11	240	0	21	1	"Histiocytosis X (acute, progressive)"	Feb-09
51718	C37y500	25	0	0	0	"Histiocytosis X , chronic"	Feb-09
36736	C37y600	105	0	1	0	"Histiocytosis X , unspecified"	Feb-09
37126	C37y700	133	0	2	0	"Histiocytosis, unspecified"	Feb-09
21966	PKy7100	91	0	1	0	Holt - Oram syndrome	Feb-09
33575	C304300	204	0	4	0	Homocystinuria	Feb-09
37808	D104311	46	0	1	0	Homozygous alpha thalassaemia	Feb-09

For peer review only - http://bmjopen.bmj.com/site/about/guidelines.xhtml

44146	D303800	121	0	1	0	Homozygous factor V Leiden mutation	Feb-09
49820	42e0.00	30	0	2	66	Homozygous SS genotype	Feb-09
16167	F17z.11	841	0	97	0	Horner's syndrome	Feb-09
3584	F115.00	7715	0	298	0	Hydrocephalus	Feb-09
97663	P233.12	2	0	0	0	Hydrocephalus with atresia of foramina of Magendie+Luschka	Sep-09
40588	P230.11	6	0	0	0	Hydrocephauls with anomaly of aqueduct of Sylvius	Feb-09
21483	SM31000	16	0	1	0	Hydrochloric acid causing toxic effect	Feb-09
3277	K11..00	33858	0	2602	7	Hydronephrosis	Feb-09
27302	K11z.00	1146	0	20	0	Hydronephrosis NOS	Feb-09
8522	K113.11	2186	0	125	0	Hydronephrosis with pelviureteric junction obstruction	Feb-09
27592	K112.00	225	0	21	0	Hydronephrosis with renal and ureteral calculous obstruction	Feb-09
28159	K11X.00	108	0	3	0	Hydronephrosis with ureteral stricture NEC	Feb-09
10410	K113.00	477	0	12	0	Hydronephrosis with ureteropelvic junction obstruction	Feb-09
31841	K135.00	682	0	13	0	Hydroureter	Feb-09
46116	PD25.00	36	0	0	0	Hydroureter - congenital	Feb-09
17778	K111.00	497	0	7	0	Hydroureteronephrosis	Feb-09
32275	C151.00	437	0	13	0	Hyperaldosteronism	Feb-09
68567	C151z00	31	0	0	0	Hyperaldosteronism NOS	Feb-09
107252	C329.00	7575	0	78	2	Hypercholesterolaemia	Nov-13
34150	D41y000	283	0	27	2	Hypergammaglobulinaemia	Feb-09
10166	C307000	2032	0	66	8	Hyperglycinaemia	Feb-09
31274	C163200	53	0	3	0	Hypergonadotrophic ovarian failure	Feb-09
104314	C304500	15	0	0	0	Hyperhomocysteinaemia	Jun-12
54203	C397.00	17	0	0	0	Hyperimmunoglobulin E syndrome	Feb-09

For peer review only - http://bmjopen.bmj.com/site/about/guidelines.xhtml

111300	K08yB00	2	0	0	0	Hyperkalaemic renal tubular acidosis	Sep-16
58069	E2E1.00	17	0	1	0	Hyperkinesis with developmental delay	Feb-09
45263	E2E2.00	175	0	6	0	Hyperkinetic conduct disorder	Feb-09
41769	E2Ez.00	41	0	4	0	Hyperkinetic syndrome NOS	Feb-09
637	C324.00	338588	0	7473	464	Hyperlipidaemia NOS	Feb-09
48144	C307400	22	0	0	0	Hyperlysinaemia	Feb-09
26371	C352000	27	0	0	0	Hypermagnesaemia	Feb-09
73934	C120100	8	0	0	0	Hyperparathyroid bone disease	Feb-09
3559	C120.00	16259	0	1792	3	Hyperparathyroidism	Feb-09
9423	C353400	706	0	12	0	Hyperphosphataemia	Feb-09
6732	C131000	10204	0	1238	0	Hyperprolactinaemia	Feb-09
30533	C30y400	82	0	12	0	Hyperprolinaemia	Feb-09
10702	D414.00	449	0	25	0	Hypersplenism	Feb-09
57987	G234.00	8	0	1	0	Hyperten heart&renal dis+both(congestv)heart and renal fail	Feb-09
103690	38G3.00	6798	0	0	0	"Hyperten, abnorm renal/liver funct, stroke, BLED score"	Feb-12
7057	G2z..00	41526	0	209	0	Hypertensive disease NOS	Feb-09
63466	G23..00	39	0	0	0	Hypertensive heart and renal disease	Feb-09
68659	G23z.00	6	0	0	0	Hypertensive heart and renal disease NOS	Feb-09
28684	G233.00	46	0	2	0	Hypertensive heart and renal disease with renal failure	Feb-09
31464	G21z.00	119	0	2	0	Hypertensive heart disease NOS	Feb-09
62718	G21z100	70	0	0	0	Hypertensive heart disease NOS with CCF	Feb-09
61166	G21z000	67	0	1	0	Hypertensive heart disease NOS without CCF	Feb-09

For peer review only - http://bmjopen.bmj.com/site/about/guidelines.xhtml

21837	G232.00	29	0	4	0	Hypertensive heart&renal dis wth (congestive) heart failure	Feb-09
4668	G22..00	1876	0	61	0	Hypertensive renal disease	Feb-09
32423	G222.00	82	0	0	0	Hypertensive renal disease with renal failure	Feb-09
1472	C02..11	66918	0	9227	174	Hyperthyroidism	Feb-09
26362	212P.00	331	0	4	0	Hyperthyroidism resolved	Feb-09
53977	C302600	11	0	0	0	Hypertyrosinaemia	Feb-09
91091	7Q0B100	42	0	0	0	Hyperuricaemia drugs Band 1	Feb-09
60584	C303400	6	0	0	0	Hypervalinaemia	Feb-09
64905	C382.00	5	0	0	0	Hypervitaminosis A	Feb-09
32143	C384.00	36	0	2	0	Hypervitaminosis D	Feb-09
21594	C154300	113	0	1	0	Hypoaldosteronism	Feb-09
95041	PG4B100	2	0	0	0	Hypochondrogenesis	Feb-09
29840	PG42.15	137	0	4	0	Hypochondroplasia	Feb-09
94173	PG41000	13	0	0	0	Hypochondroplasia	Feb-09
50305	K032y11	11	0	0	0	Hypocomplementaemic persistent glomerulonephritis NEC	Feb-09
15137	C390000	1831	0	64	0	Hypogammaglobulinaemia NOS	Feb-09
98210	C139.00	1581	0	70	0	Hypogonadotropic hypogonadism	Dec-09
36298	M250.11	37	0	3	0	Hypohidrosis	Feb-09
15096	C121.00	2168	0	136	2	Hypoparathyroidism	Feb-09
63512	C121z00	49	0	0	0	Hypoparathyroidism NOS	Feb-09
7902	C353300	613	0	21	0	Hypophosphataemia	Feb-09
21870	C353211	217	0	5	0	Hypophosphataemic rickets	Feb-09
33347	C353000	366	0	19	1	Hypophosphatasia	Feb-09
22028	C353100	88	0	6	0	Hypophosphatasia rickets	Feb-09
105861	C133.12	1	0	0	0	Hypophyseal dwarfism	Mar-13
8552	C132.11	3039	0	145	4	Hypopituitarism NOS	Feb-09
29818	PK13.00	92	0	6	0	Hypoplasia of adrenal gland	Feb-09
37515	P722200	42	0	1	0	Hypoplasia of aorta	Feb-09

For peer review only - http://bmjopen.bmj.com/site/about/guidelines.xhtml

57932	P710.00	29	0	1	0	"Hypoplasia of aortic arch, unspecified"	Feb-09
63668	P222.00	12	0	0	0	"Hypoplasia of brain, part unspecified"	Feb-09
68063	P6yy100	3	0	0	0	Hypoplasia of cardiac vein	Feb-09
44717	P22A100	29	0	0	0	Hypoplasia of cerebellum	Feb-09
37600	P228100	13	0	0	0	Hypoplasia of corpus callosum	Feb-09
51993	PF4z.13	42	0	2	0	Hypoplasia of limb NOS	Feb-09
24646	PF3z.11	107	0	1	0	Hypoplasia of lower limb	Feb-09
23874	P851.00	156	0	1	0	Hypoplasia of lung	Feb-09
61593	PB72.00	3	0	0	0	Hypoplasia of pancreas	Feb-09
60216	P22z.13	11	0	0	0	Hypoplasia of part of brain NEC	Feb-09
23948	PCy2000	58	0	6	0	Hypoplasia of penis	Feb-09
54488	P601000	17	0	0	0	Hypoplasia of pulmonary valve	Feb-09
68493	PG1y400	10	0	0	0	Hypoplasia of spine	Feb-09
71462	PK06.00	5	0	0	0	Hypoplasia of spleen	Feb-09
54487	P734.00	34	0	0	0	Hypoplasia of the pulmonary artery	Feb-09
22457	PF2z.11	62	0	3	0	Hypoplasia of upper limb	Feb-09
44913	D200.15	46	0	3	0	Hypoplastic anaemia - familial	Feb-09
32715	D201111	11	0	0	0	Hypoplastic anaemia due to drug or chemical substance	Feb-09
65351	D201211	11	0	0	0	Hypoplastic anaemia due to infection	Feb-09
57114	D201412	2	0	1	0	Hypoplastic anaemia due to toxic cause	Feb-09
44767	P6yy.11	27	0	0	0	Hypoplastic aortic orifice or valve	Feb-09
92968	7Q09100	3	0	0	0	Hypoplastic haemolytic and renal anaemia drugs Band 2	Feb-09
16004	C134011	112	0	13	0	Hypoprolactinaemia	Feb-09
5073	C33y000	1163	0	98	4	Hypoproteinaemia	Feb-09
51291	D303111	10	0	2	0	Hypoprothrombinaemia	Feb-09
28386	F4A0400	43	0	2	0	Hypopyon ulcer	Feb-09

For peer review only - http://bmjopen.bmj.com/site/about/guidelines.xhtml

108019	C154700	9	0	0	0	Hyporeninaemic hypoaldosteronism	May-14
36881	C13X.00	53	0	1	0	"Hypothalamic dysfunction, not elsewhere classified"	Feb-09
22732	C13..11	144	0	7	0	Hypothalamus disorders	Feb-09
64145	5761.11	2	0	1	3	Hypothalamus hormone radioass.	Feb-09
18282	C04z.13	447	0	54	0	"Hypothyroid goitre, acquired"	Feb-09
273	C04..13	428907	0	8672	1713	Hypothyroidism	Feb-09
3941	C04z.00	39418	0	249	0	Hypothyroidism NOS	Feb-09
68482	C372000	1	0	2	0	Hypoxanthine-guanine-phosphoribosyltransferase deficiency	Feb-09
20275	C150100	72	0	5	0	Iatrogenic Cushing's syndrome	Feb-09
61520	C110000	17	0	0	0	Iatrogenic hyperinsulinism	Feb-09
50958	C137.11	51	0	3	0	Iatrogenic hypopituitarism	Feb-09
38976	C043z00	240	0	2	0	Iatrogenic hypothyroidism NOS	Feb-09
34459	C137z00	8	0	1	1	Iatrogenic pituitary disorder NOS	Feb-09
56983	C137.00	22	0	2	0	Iatrogenic pituitary disorders	Feb-09
61026	C054.00	13	0	0	0	Iatrogenic thyroiditis	Feb-09
370	PH1..00	1995	0	86	0	Ichthyosis congenita	Feb-09
64322	PH1z.00	72	0	0	0	Ichthyosis congenita NOS	Feb-09
5555	PH14.00	780	0	18	0	Ichthyosis vulgaris	Feb-09
69124	C107300	15	0	0	0	IDDM with peripheral circulatory disorder	Feb-09
18505	C108.11	8393	0	317	0	IDDM-Insulin dependent diabetes mellitus	Feb-09
37401	PB31.00	92	0	2	0	Idiopathic congenital megacolon	Feb-09
94708	F4B2100	5	0	0	0	Idiopathic corneal oedema	Feb-09
70967	C150000	6	0	1	0	Idiopathic Cushing's syndrome	Feb-09
62243	F136000	14	0	1	0	Idiopathic familial dystonia	Feb-09
28229	H563z00	214	0	1	0	Idiopathic fibrosing alveolitis NOS	Feb-09
40750	D00z200	193	0	2	0	Idiopathic hypochromic anaemia	Feb-09
25379	C121200	46	0	0	0	Idiopathic hypoparathyroidism	Feb-09

For peer review only - http://bmjopen.bmj.com/site/about/guidelines.xhtml

40428	N330300	133	0	3	0	Idiopathic osteoporosis	Feb-09
27597	N331600	116	0	6	0	Idiopathic osteoporosis with pathological fracture	Feb-09
48590	C132000	30	0	0	0	Idiopathic panhypopituitarism	Feb-09
103753	H563.13	344	0	8	0	Idiopathic pulmonary fibrosis	Feb-12
27348	H561.00	45	0	6	0	Idiopathic pulmonary haemosiderosis	Feb-09
29966	C350300	47	0	8	0	Idiopathic pulmonary haemosiderosis	Feb-09
31560	B937.12	82	0	2	0	Idiopathic thrombocythaemia	Feb-09
5144	D313.12	2000	0	197	3	Idiopathic thrombocytopenic purpura	Feb-09
12234	D313000	2308	0	47	0	Idiopathic thrombocytopenic purpura	Feb-09
39800	C39X.00	67	0	5	0	"Immunodeficiency associated+major defect, unspecified"	Feb-09
69373	C390600	6	0	0	0	Immunodeficiency with IgM hypergammaglobulinaemia	Feb-09
65617	C395.00	4	0	0	0	Immunodeficiency with short-limbed stature	Feb-09
4855	PB26.00	1267	0	29	0	Imperforate anus	Feb-09
67319	PB26z00	80	0	0	0	Imperforate anus NOS	Feb-09
61401	PB26000	56	0	0	0	Imperforate anus with fistula	Feb-09
68484	PB14.00	3	0	0	0	Imperforate jejunum	Feb-09
98741	PA33.00	0	0	0	0	Imperforate oesophagus	Mar-10
56901	PA27000	2	0	0	0	Imperforate pharynx	Feb-09
49242	PB27.00	22	0	0	0	Imperforate rectum	Feb-09
44953	PB27000	3	0	0	0	Imperforate rectum with fistula	Feb-09
22098	E140.00	1419	0	53	0	Infantile autism	Feb-09
36662	E140z00	78	0	1	0	Infantile autism NOS	Feb-09
5560	F23..12	527	0	8	0	Infantile cerebral palsy	Feb-09

For peer review only - http://bmjopen.bmj.com/site/about/guidelines.xhtml

For peer review only

2019	F234.00	250	0	5	0	Infantile hemiplegia NOS	Feb-09
54014	F392300	6	0	0	0	Infantile myotonia	Feb-09
41668	F460.00	49	0	3	0	"Infantile, juvenile and presenile cataracts"	Feb-09
569	G64..12	3985	0	179	0	Infarction - cerebral	Feb-09
57495	G63..11	37	0	0	0	Infarction - precerebral	Feb-09
24444	G401.11	124	0	1	0	Infarction - pulmonary	Feb-09
26424	G64z400	1122	0	5	0	Infarction of basal ganglia	Feb-09
1796	J4...12	18902	0	1309	14	Inflammatory bowel disease	Feb-09
39809	C108J00	27	0	1	0	Insulin dependent diab mell with neuropathic arthropathy	Feb-09
64446	C108G00	3	0	1	0	Insulin dependent diab mell with peripheral angiopathy	Feb-09
31310	C108900	565	0	20	0	Insulin dependent diabetes maturity onset	Feb-09
97849	C10E912	14	0	0	0	Insulin dependent diabetes maturity onset	Oct-09
1038	C100011	34834	0	2167	5	Insulin dependent diabetes mellitus	Feb-09
1647	C108.00	23978	0	838	7	Insulin dependent diabetes mellitus	Feb-09
51261	C10E.12	1339	0	1	0	Insulin dependent diabetes mellitus	Feb-09
6791	C108800	434	0	18	0	Insulin dependent diabetes mellitus - poor control	Feb-09
72702	C10E812	14	0	0	0	Insulin dependent diabetes mellitus - poor control	Feb-09
65616	C108H00	4	0	0	0	Insulin dependent diabetes mellitus with arthropathy	Feb-09
44260	C108F00	7	0	2	0	Insulin dependent diabetes mellitus with diabetic cataract	Feb-09
100770	C10EF12	1	0	0	0	Insulin dependent diabetes mellitus with diabetic cataract	Nov-10
60499	C108600	10	0	2	0	Insulin dependent diabetes mellitus with gangrene	Feb-09

For peer review only - http://bmjopen.bmj.com/site/about/guidelines.xhtml

109051	C10E612	1	0	0	0	Insulin dependent diabetes mellitus with gangrene	Jan-15
44440	C108E00	51	0	0	0	Insulin dependent diabetes mellitus with hypoglycaemic coma	Feb-09
99716	C10EE12	2	0	0	0	Insulin dependent diabetes mellitus with hypoglycaemic coma	Jun-10
24694	C108B00	1	0	0	0	Insulin dependent diabetes mellitus with mononeuropathy	Feb-09
45276	C10E312	16	0	0	0	Insulin dependent diabetes mellitus with multiple complicat	Feb-09
52104	C108300	11	0	1	0	Insulin dependent diabetes mellitus with multiple complicatn	Feb-09
57621	C108D00	18	0	0	0	Insulin dependent diabetes mellitus with nephropathy	Feb-09
102163	C10ED12	1	0	0	0	Insulin dependent diabetes mellitus with nephropathy	Apr-11
41716	C108C00	11	0	0	0	Insulin dependent diabetes mellitus with polyneuropathy	Feb-09
101311	C10EC12	2	0	0	0	Insulin dependent diabetes mellitus with polyneuropathy	Jan-11
6509	C108700	249	0	41	0	Insulin dependent diabetes mellitus with retinopathy	Feb-09
93875	C10E712	8	0	0	0	Insulin dependent diabetes mellitus with retinopathy	Feb-09
44443	C108500	215	0	3	0	Insulin dependent diabetes mellitus with ulcer	Feb-09
98704	C10E512	3	0	0	0	Insulin dependent diabetes mellitus with ulcer	Mar-10
37648	C109J11	24	0	3	0	Insulin treated non-insulin dependent diabetes mellitus	Feb-09
1407	C10FJ00	13414	0	84	0	Insulin treated Type 2 diabetes mellitus	Feb-09

For peer review only - http://bmjopen.bmj.com/site/about/guidelines.xhtml

For peer review only

18278	C109J00	4211	0	28	0	Insulin treated Type 2 diabetes mellitus	Feb-09
64668	C10FJ11	107	0	0	0	Insulin treated Type II diabetes mellitus	Feb-09
96010	66Ap.00	2595	0	3	0	Insulin treatment initiated	Feb-09
100791	66Ar.00	91	0	0	0	Insulin treatment stopped	Nov-10
52283	C108200	8	0	0	0	Insulin-dependent diabetes mellitus with neurological comps	Feb-09
101735	C10E212	1	0	0	0	Insulin-dependent diabetes mellitus with neurological comps	Feb-11
49276	C108100	18	0	0	0	Insulin-dependent diabetes mellitus with ophthalmic comps	Feb-09
98071	C10E112	2	0	0	0	Insulin-dependent diabetes mellitus with ophthalmic comps	Nov-09
46963	C108000	30	0	1	0	Insulin-dependent diabetes mellitus with renal complications	Feb-09
102946	C10E012	4	0	0	0	Insulin-dependent diabetes mellitus with renal complications	Sep-11
56448	C108A00	21	0	0	0	Insulin-dependent diabetes without complication	Feb-09
99719	C10EA12	2	0	0	0	Insulin-dependent diabetes without complication	Jun-10
109815	H58y600	1	0	0	0	Interstitial lung disease due to collagen vascular disease	Jul-15
104915	H58y700	35	0	1	0	Interstitial lung disease due to connective tissue disease	Sep-12
9355	J69..00	2226	0	216	0	Intestinal malabsorption	Feb-09
42715	J69z.00	212	0	2	0	Intestinal malabsorption NOS	Feb-09
49739	J69y300	10	0	2	0	Intestinal malabsorption of carbohydrate	Feb-09
31392	J69y600	85	0	4	1	Intestinal malabsorption of fat	Feb-09
49191	J69y200	37	0	29	26	Intestinal malabsorption of protein	Feb-09

For peer review only - http://bmjopen.bmj.com/site/about/guidelines.xhtml

37877	F280z11	26	0	0	0	Intracerebral cyst NOS	Feb-09
44479	Q200511	4	0	0	0	Intracerebral haematoma in fetus or newborn	Feb-09
5051	G61..00	14470	0	240	0	Intracerebral haemorrhage	Feb-09
36559	Q200011	26	0	0	0	Intracerebral haemorrhage in fetus or newborn	Feb-09
31060	G61X.00	104	0	0	0	"Intracerebral haemorrhage in hemisphere, unspecified"	Feb-09
3535	G61z.00	1877	0	81	0	Intracerebral haemorrhage NOS	Feb-09
30202	G617.00	676	0	2	0	"Intracerebral haemorrhage, intraventricular"	Feb-09
57315	G618.00	33	0	2	0	"Intracerebral haemorrhage, multiple localized"	Feb-09
66218	C3A..00	5	0	0	0	Iodine-deficiency syndromes	Feb-09
54511	C0AX.00	16	0	0	0	"Iodine-deficiency-related (endemic) goitre, unspecified"	Feb-09
37518	C0A3.00	22	0	1	0	Iodine-deficiency-related diffuse (endemic) goitre	Feb-09
44459	C0A4.00	84	0	8	0	Iodine-deficiency-related multinodular (endemic) goitre	Feb-09
48338	D000.12	118	0	1	0	Iron deficiency anaemia due to blood loss	Feb-09
27726	D000.00	2676	0	101	0	Iron deficiency anaemia due to chronic blood loss	Feb-09
15439	D00zz00	26280	0	657	17	Iron deficiency anaemia NOS	Feb-09
795	D00..00	458744	0	29240	279	Iron deficiency anaemias	Feb-09
100507	PJ33400	10	0	0	0	Jacobsen syndrome	Aug-10
50228	PG43.11	27	0	1	0	Jeune's syndrome	Feb-09
17399	F250400	897	0	32	0	Juvenile absence epilepsy	Feb-09
42405	N045000	118	0	8	0	Juvenile ankylosing spondylitis	Feb-09
12575	N045300	50	0	5	0	Juvenile arthritis in Crohn's disease	Feb-09
28456	N045200	163	0	5	0	Juvenile arthritis in psoriasis	Feb-09

For peer review only - http://bmjopen.bmj.com/site/about/guidelines.xhtml

71083	N045400	4	0	0	0	Juvenile arthritis in ulcerative colitis	Feb-09
50627	M142.00	36	0	0	0	Juvenile dermatitis herpetiformis	Feb-09
32649	N003000	91	0	1	0	Juvenile dermatomyositis	Feb-09
64693	F4B5100	9	0	0	0	Juvenile epithelial corneal dystrophy	Feb-09
105069	B693.00	3	0	0	0	Juvenile myelomonocytic leukaemia	Oct-12
19363	F25A.00	1338	0	12	0	Juvenile myoclonic epilepsy	Feb-09
45320	A904.00	3	0	0	0	Juvenile neurosyphilis	Feb-09
66740	F380100	8	0	0	0	Juvenile or adult myasthenia gravis	Feb-09
6975	N326200	899	0	17	0	Juvenile osteochondritis NOS	Feb-09
63370	N323.11	2	0	0	0	Juvenile osteochondritis of the arm	Feb-09
62237	N323.00	21	0	1	0	Juvenile osteochondritis of the arm and hand	Feb-09
73645	N323z00	1	0	0	0	Juvenile osteochondritis of the arm and hand NOS	Feb-09
101671	N323000	1	0	0	0	"Juvenile osteochondritis of the arm, unspecified"	Feb-11
39819	N323100	3	0	0	0	"Juvenile osteochondritis of the hand, unspecified"	Feb-09
34369	N321.00	370	0	1	1	Juvenile osteochondritis of the hip and pelvis	Feb-09
68673	N321z00	11	0	0	0	Juvenile osteochondritis of the hip and pelvis NOS	Feb-09
66495	N321000	7	0	0	0	"Juvenile osteochondritis of the hip and pelvis, unspecified"	Feb-09
21502	N321400	23	0	2	0	Juvenile osteochondritis of the iliac crest	Feb-09
36725	N320.00	422	0	4	0	Juvenile osteochondritis of the spine	Feb-09
69739	N320z00	16	0	0	0	Juvenile osteochondritis of the spine NOS	Feb-09

For peer review only - http://bmjopen.bmj.com/site/about/guidelines.xhtml

50510	N320000	13	0	0	0	"Juvenile osteochondritis of the spine, unspecified"	Feb-09
94462	N321500	4	0	0	0	Juvenile osteochondritis of the symphysis pubis	Feb-09
94547	N326z00	3	0	0	0	Juvenile osteochondroses NOS	Feb-09
9691	N326300	222	0	4	0	Juvenile osteochondrosis NOS	Feb-09
52681	N328.00	32	0	0	0	Juvenile osteochondrosis of spine	Feb-09
48042	N325.00	61	0	1	0	Juvenile osteochondrosis of the foot	Feb-09
60589	N325z00	14	0	0	0	Juvenile osteochondrosis of the foot NOS	Feb-09
63567	N325000	10	0	0	0	"Juvenile osteochondrosis of the foot, unspecified"	Feb-09
33667	N324.00	2827	0	3	0	Juvenile osteochondrosis of the leg	Feb-09
38898	N324z00	54	0	2	0	"Juvenile osteochondrosis of the leg, NOS"	Feb-09
24028	N324000	24	0	0	0	"Juvenile osteochondrosis of the leg, unspecified"	Feb-09
35026	N324111	15	0	0	0	Juvenile osteochondrosis of the primary patellar centre	Feb-09
48242	N324300	58	0	0	0	Juvenile osteochondrosis of the secondary patellar centre	Feb-09
17810	M142.11	20	0	1	0	Juvenile pemphigoid	Feb-09
7859	M2y4800	8465	0	78	0	Juvenile plantar dermatosis	Feb-09
18380	G759.00	115	0	6	0	Juvenile polyarteritis	Feb-09
96664	J615800	3	0	0	0	Juvenile portal cirrhosis	Apr-09
28450	F427300	109	0	4	0	Juvenile retinoschisis	Feb-09
31360	N045500	352	0	2	0	Juvenile rheumatoid arthritis	Feb-09
4186	N043.00	4230	0	258	0	Juvenile rheumatoid arthritis - Still's disease	Feb-09
27557	N043z00	225	0	2	0	Juvenile rheumatoid arthritis NOS	Feb-09
50644	N043000	37	0	2	0	Juvenile rheumatoid arthropathy unspecified	Feb-09

For peer review only - http://bmjopen.bmj.com/site/about/guidelines.xhtml

31181	N045100	188	0	7	0	Juvenile seronegative polyarthritis	Feb-09
107689	M12A400	80	0	0	0	Juvenile spring eruption	Feb-14
21876	C172111	265	0	16	0	Kallman's syndrome	Feb-09
43444	E140.11	12	0	0	0	Kanner's syndrome	Feb-09
99213	PJy2.12	2	0	0	0	"Karyotype 47, XXX"	Apr-10
9768	PJyy300	119	0	4	2	"Karyotype 47,XYY"	Feb-09
6157	G751000	2136	0	99	0	Kawasaki disease	Feb-09
31452	Q424.00	55	0	4	0	Kernicterus due to isoimmunisation	Feb-09
48012	Q437.00	8	0	0	0	Kernicterus not due to isoimmunisation	Feb-09
61800	Q437z00	7	0	0	0	Kernicterus of newborn NOS	Feb-09
69127	PKy9211	2	0	0	0	Kinky hair syndrome	Feb-09
54490	PJ70.00	29	0	0	0	"Klinefelter's phenotype, karyotype 47XXY"	Feb-09
59439	PJ7z.00	63	0	2	0	Klinefelter's syndrome NOS	Feb-09
96257	PJ72.00	1	0	0	0	"Klinefelter's syndrome, male with 46XX karyotype"	Apr-09
62414	PJ71.12	11	0	0	0	"Klinefelter's syndrome, XXXXY"	Feb-09
56545	PJ71.11	4	0	0	0	"Klinefelter's syndrome, XXXY"	Feb-09
91262	PJ73.00	10	0	0	0	"Klinefelter's syndrome, XXYY"	Feb-09
68109	PJ74.00	26	0	1	0	"Klinefelter's syndrome, XY/XXY mosaic"	Feb-09
67854	PJ71.00	3	0	1	0	"Klinefelter's syndrome,male with more than two X chromosomes"	Feb-09
73985	PG16z00	16	0	0	0	Klippel - Feil syndrome NOS	Feb-09
10491	PKy7200	411	0	18	0	Klippel - Trenaunay - Weber syndrome	Feb-09
4674	PG16.00	519	0	15	0	Klippel-Feil syndrome	Feb-09
59014	N13y100	31	0	0	0	Klippel's disease	Feb-09
71719	F257.00	3	0	0	0	Kojevnikov's epilepsy	Feb-09
35737	C20..00	32	0	2	1	Kwashiorkor	Feb-09
60113	C362300	20	0	0	0	Lactic acidaemia	Feb-09

For peer review only - http://bmjopen.bmj.com/site/about/guidelines.xhtml

37854	C313111	64	0	3	0	Lactose malabsorption	Feb-09
105082	F381011	21	0	0	0	Lambert-Eaton syndrome	Oct-12
59806	ZS82.11	29	0	1	0	Landau-Kleffner syndrome	Feb-09
40000	C37yB00	304	0	1	0	Langerhans' cell histiocytosis	Feb-09
6340	E2F3.11	2040	0	48	0	Language development disorder	Feb-09
99857	ZS7C400	2	0	0	0	Language disorder associated with right hemisphere damage	Jun-10
32831	ZS7C600	42	0	9	0	Language disorder associated with thought disorder	Feb-09
2052	13Z4E00	49540	0	2162	1	Learning difficulties	Feb-09
56376	Z7CD200	466	0	0	0	Learning difficulties	Feb-09
28113	F427J00	102	0	5	0	Leber's congenital amaurosis	Feb-09
45706	F4H1600	137	0	7	0	Leber's hereditary optic atrophy	Feb-09
48037	F4H1711	7	0	0	0	Leber's optic atrophy	Feb-09
53826	G562z00	105	0	7	0	Left bundle branch hemiblock NOS	Feb-09
8933	F222.00	2209	0	147	1	Left hemiplegia	Feb-09
26318	G563.00	283	0	4	0	Left main stem bundle branch block	Feb-09
98383	7904200	1	0	0	0	Left ventricle aorta tunnel right ventricle pul art val con	Jan-10
67657	P542.00	4	0	0	0	Left ventricle to right atrial communication	Feb-09
107397	G5yyD00	792	0	5	0	Left ventricular cardiac dysfunction	Dec-13
884	G581.00	170646	0	11892	23	Left ventricular failure	Feb-09
562	G5y3411	21482	0	436	1	Left ventricular hypertrophy	Feb-09
108457	P69..00	26	0	0	0	Left ventricular outflow tract obstruction	Aug-14
21854	G5yy700	504	0	5	0	Left ventricular thrombosis	Feb-09
69114	C303000	2	0	0	0	Leucinosis	Feb-09
59035	F100z00	9	0	0	0	Leucodystrophy NOS	Feb-09
43857	C10M.00	37	0	2	0	Lipoatrophic diabetes mellitus	Feb-09
34624	C375.14	6	0	2	0	Lipochondrodystrophy	Feb-09
56796	C326z00	39	0	1	0	Lipodystrophy NOS	Feb-09

For peer review only - http://bmjopen.bmj.com/site/about/guidelines.xhtml

39419	A3A2.11	18	0	0	0	"Lipodystrophy, intestinal"	Feb-09
19337	G56y500	1886	0	171	0	Long Q-T syndrome	Feb-09
63395	PB30000	2	0	0	0	Long segment Hirschsprung's disease	Feb-09
47768	C302500	25	0	5	0	Lowe disease	Feb-09
4125	M154.00	6746	0	484	27	Lupus erythematosus	Feb-09
33449	M154000	16	0	0	0	Lupus erythematosus chronicus	Feb-09
40797	M154200	11	0	0	0	Lupus erythematosus migrans	Feb-09
65391	M154300	17	0	0	0	Lupus erythematosus nodularis	Feb-09
7522	M154z00	239	0	4	0	Lupus erythematosus NOS	Feb-09
46148	M154400	77	0	1	0	Lupus erythematosus profundus	Feb-09
44984	M154500	42	0	1	0	Lupus erythematosus tumidus	Feb-09
63955	M154600	2	0	0	0	Lupus erythematosus unguium mutilans	Feb-09
37474	P241.00	61	0	0	0	Macroencephaly	Feb-09
16527	C333.00	337	0	27	0	Macroglobulinaemia	Feb-09
71994	C333z00	6	0	0	0	Macroglobulinaemia NOS	Feb-09
30176	PA14.00	164	0	16	0	Macroglossia	Feb-09
27325	J040.12	120	0	44	0	Macrognathism	Feb-09
73766	J040A00	6	0	0	0	Macrognathism unspecified	Feb-09
72094	P242.00	3	0	0	0	Macrogyria	Feb-09
58909	P37..00	5	0	0	0	Macrophthalmos	Feb-09
61992	PG42012	26	0	0	0	Maffuci's syndrome	Feb-09
30100	C294400	356	0	6	1	Magnesium deficiency	Feb-09
71815	PJ51000	5	0	0	0	Major partial trisomy	Feb-09
31419	J69y700	379	0	32	4	Malabsorption - iron	Feb-09
29513	J69y011	14	0	0	0	Malabsorption due to intolerance to fat	Feb-09
92869	C313911	1	0	0	0	Malabsorption of disaccharide NEC	Feb-09
38805	C313511	24	0	0	0	Malabsorption of glucose	Feb-09
57431	C313011	11	0	0	0	Malabsorption of glucose - galactose	Feb-09

For peer review only - http://bmjopen.bmj.com/site/about/guidelines.xhtml

11934	J69yz13	885	0	50	0	Malabsorption syndrome NOS	Feb-09
54216	PC71.00	20	0	2	0	Male pseudohermaphroditism	Feb-09
37484	PJy6.00	16	0	3	0	Male with structurally abnormal sex chromosome	Feb-09
37070	E110.00	1217	0	13	0	"Manic disorder, single episode"	Feb-09
36611	E110z00	46	0	2	0	"Manic disorder, single episode NOS"	Feb-09
22713	1S42.00	496	0	46	0	Manic mood	Feb-09
26161	E11..13	653	0	27	0	Manic psychoses	Feb-09
109671	C399000	14	0	0	0	Mannose-binding lectin deficiency	May-15
66577	C31y300	32	0	0	0	Mannosidosis	Feb-09
107648	C375000	2	0	0	0	Mannosidosis	Jan-14
37980	G764.11	11	0	0	0	Marable's syndrome	Feb-09
35893	C201.00	4	0	0	0	Marasmic kwashiorkor	Feb-09
103962	A78y300	3	0	0	0	Marburg disease	Apr-12
70356	PKy5600	1	0	0	0	Marchesani syndrome	Feb-09
100022	D112012	1	0	0	0	Marchiafava - Micheli syndrome	Jun-10
56153	F21y000	5	0	0	0	Marchiafava-Bignami disease	Feb-09
3884	P2x4.00	447	0	24	0	Marcus - Gunn syndrome	Feb-09
45341	F4E4113	21	0	0	0	Marcus - Gunn syndrome	Feb-09
936	PKy2.00	4054	0	539	0	Marfan's syndrome	Feb-09
99763	F142000	5	0	1	0	Marie's cerebellar ataxia	Jun-10
11631	C37z.11	885	0	65	0	Marinesco-Sjogren syndrome	Feb-09
62281	C375611	3	0	1	0	Maroteaux - Lamy syndrome	Feb-09
107550	PKy0400	4	0	0	0	Marshall-Smith syndrome	Jan-14
46624	C10C.11	222	0	9	0	Maturity onset diabetes in youth	Feb-09
98392	C10C.12	7	0	0	0	Maturity onset diabetes in youth type 1	Jan-10
59991	C10D.11	31	0	0	0	Maturity onset diabetes in youth type 2	Feb-09
49317	C310000	98	0	6	0	McArdle's disease	Feb-09
42632	PD12.00	53	0	4	0	Medullary cystic disease	Feb-09

For peer review only - http://bmjopen.bmj.com/site/about/guidelines.xhtml

41522	PD12z00	5	0	0	0	Medullary cystic disease NOS	Feb-09
64694	PD12y00	4	0	0	0	Medullary cystic disease OS	Feb-09
47135	PD12100	37	0	2	0	"Medullary cystic disease, adult type"	Feb-09
66998	PD12000	4	0	0	0	"Medullary cystic disease, juvenile type"	Feb-09
33400	C156.00	6	0	0	0	Medulloadrenal hyperfunction	Feb-09
35863	J527.00	136	0	5	0	Megacolon excluding Hirschsprung's disease	Feb-09
4816	J527z00	449	0	26	0	Megacolon NOS	Feb-09
65723	D313.14	1	0	0	0	Megakaryocytic hypoplasia	Feb-09
33442	P241.11	64	0	0	0	Megalencephaly	Feb-09
50169	P249.00	52	0	0	0	Megalencephaly	Feb-09
94733	Q48y500	2	0	0	0	Megalencephaly	Feb-09
4475	D01..11	1796	0	102	1	Megaloblastic anaemia	Feb-09
59103	D012112	24	0	0	0	Megaloblastic anaemia due to dietary causes	Feb-09
53799	D01z.11	11	0	0	0	Megaloblastic anaemia NOS	Feb-09
50050	PB56.00	3	0	0	0	Megaloduodenum	Feb-09
71255	PA75.00	1	0	0	0	Megalogastria	Feb-09
40049	K13y700	27	0	0	0	Megaloureter - acquired	Feb-09
5099	PD26.00	120	0	2	0	Megaloureter - congenital	Feb-09
41905	J100.12	7	0	0	0	Megaoesophagus	Feb-09
69752	J100000	2	0	0	1	Megaoesophagus in Chagas' disease	Feb-09
72742	PKyG.00	3	0	0	0	Men ret congen heart dis blepharophim blepharop hypopl teeth	Feb-09
4246	6664	4051	0	128	0	Mental handicap problem	Feb-09
1362	E3...00	8262	0	181	3	Mental retardation	Feb-09
37867	E3z..00	542	0	4	0	Mental retardation NOS	Feb-09
68132	6894	238	0	0	0	Mental retardation screen	Feb-09

For peer review only - http://bmjopen.bmj.com/site/about/guidelines.xhtml

41881	K032y14	246	0	7	0	Mesangiocapillary glomerulonephritis NEC	Feb-09
36342	K032y13	94	0	0	0	Mesangioproliferative glomerulonephritis NEC	Feb-09
12724	C1A0.00	3026	0	48	0	Metabolic syndrome	Feb-09
39481	C10F811	180	0	1	0	Metabolic syndrome X	Feb-09
104331	4Q21000	1	0	1	245	Metadrenaline level	Jun-12
26069	PG45.00	23	0	4	0	Metaphyseal dysostosis	Feb-09
45162	PG45.15	19	0	0	0	Metaphyseal dysplasia	Feb-09
37743	PG44100	2	0	0	0	Metatropic dwarfism	Feb-09
103145	PA15.00	3	0	0	0	Microglossia	Oct-11
15363	J040.15	451	0	21	0	Micrognathism	Feb-09
95850	J040B00	13	0	0	0	Micrognathism unspecified	Feb-09
52652	P336000	4	0	0	0	Microphakia	Feb-09
26462	P31..00	494	0	29	0	Microphthalmos	Feb-09
63439	P31z.00	36	0	1	0	Microphthalmos NOS	Feb-09
48507	P312.00	51	0	2	0	Microphthalmos with other eye anomaly	Feb-09
72284	P310.00	7	0	0	0	"Microphthalmos, unspecified"	Feb-09
161	F26..00	1198522	0	18647	32	Migraine	Feb-09
14700	F26z.00	26163	0	256	1	Migraine NOS	Feb-09
23621	F262z00	803	0	19	0	Migraine variant NOS	Feb-09
103502	F260.11	1592	0	8	0	Migraine with aura	Jan-12
103602	F261.11	325	0	0	0	Migraine without aura	Jan-12
3018	663V100	20542	0	18	0	Mild asthma	Feb-09
33841	F370200	299	0	6	0	Miller-Fisher syndrome	Feb-09
18307	F39X.00	335	0	15	0	"Mitochondrial myopathy, not elsewhere classified"	Feb-09
31727	G133.00	398	0	6	0	Mitral and aortic incompetence	Feb-09
94872	G133.11	4	0	0	0	Mitral and aortic insufficiency	Feb-09
11878	G133.12	3310	0	63	3	Mitral and aortic regurgitation	Feb-09
29158	G13z.00	463	0	16	0	Mitral and aortic valve disease NOS	Feb-09

For peer review only

21807	G111.11	74	0	12	0	Mitral incompetence - rheumatic	Feb-09
31759	G132.12	13	0	1	0	Mitral incompetence and aortic stenosis	Feb-09
34240	G540100	382	0	5	0	"Mitral incompetence, cause unspecified"	Feb-09
5058	G540000	2806	0	199	0	"Mitral incompetence, non-rheumatic"	Feb-09
33262	G132.00	110	0	4	0	Mitral insufficiency and aortic stenosis	Feb-09
561	G540.16	38256	0	949	3	Mitral regurgitation	Feb-09
22837	G111.12	137	0	0	0	Mitral regurgitation - rheumatic	Feb-09
33907	G132.13	247	0	2	0	Mitral regurgitation and aortic stenosis	Feb-09
1885	G110.00	9886	0	405	2	Mitral stenosis	Feb-09
61250	G131.13	19	0	0	0	Mitral stenosis and aortic incompetence	Feb-09
49355	G131.00	34	0	3	0	Mitral stenosis and aortic insufficiency	Feb-09
50983	G112.12	5	0	0	0	Mitral stenosis with incompetence	Feb-09
44488	G112.00	165	0	0	0	Mitral stenosis with insufficiency	Feb-09
44328	G112.13	72	0	2	0	Mitral stenosis with regurgitation	Feb-09
30443	G11z.00	687	0	11	0	Mitral valve disease NOS	Feb-09
1267	G11..00	5419	0	385	4	Mitral valve diseases	Feb-09
24557	G540z00	427	0	11	0	Mitral valve disorders NOS	Feb-09
2977	G540.00	7682	0	454	5	Mitral valve incompetence	Feb-09
40949	G540.12	31	0	0	0	Mitral valve insufficiency	Feb-09
39916	G540300	307	0	3	0	Mitral valve leaf prolapse	Feb-09
1294	G540.15	11485	0	488	2	Mitral valve prolapse	Feb-09
31839	G540200	799	0	15	0	Mitral valve prolapse	Feb-09
9450	G540.14	3919	0	59	0	Mitral valve regurgitation	Feb-09
25796	H332.00	960	0	2	0	Mixed asthma	Feb-09
31316	E116.00	1458	0	46	0	Mixed bipolar affective disorder	Feb-09

For peer review only - http://bmjopen.bmj.com/site/about/guidelines.xhtml

55064	E116600	92	0	0	0	"Mixed bipolar affective disorder, in full remission"	Feb-09
24689	E116100	31	0	0	0	"Mixed bipolar affective disorder, mild"	Feb-09
63150	E116200	26	0	0	0	"Mixed bipolar affective disorder, moderate"	Feb-09
63583	E116z00	152	0	1	0	"Mixed bipolar affective disorder, NOS"	Feb-09
63651	E116500	7	0	0	0	"Mixed bipolar affective disorder, partial/unspec remission"	Feb-09
54195	E116400	44	0	0	0	"Mixed bipolar affective disorder, severe, with psychosis"	Feb-09
63284	E116300	9	0	0	0	"Mixed bipolar affective disorder, severe, without psychosis"	Feb-09
31535	E116000	237	0	1	0	"Mixed bipolar affective disorder, unspecified"	Feb-09
73492	PJ60.00	8	0	0	0	Mixed gonadal dysgenesis	Feb-09
103752	G561311	13	0	1	0	Mobitz type 1 second degree atrioventricular block	Feb-12
27928	G561300	437	0	18	0	Mobitz type I (Wenckebach) atrioventricular block	Feb-09
13065	663V200	14648	0	13	0	Moderate asthma	Feb-09
302	E310.00	699	0	8	0	"Moderate mental retardation, IQ in range 35-49"	Feb-09
107350	F591C00	446	0	9	0	Moderate sensorineural hearing loss	Dec-13
36560	F326200	108	0	8	0	Moebius congenital oculofacial paralysis	Feb-09
65262	F26y111	3	0	1	0	Moebius' ophthalmoplegic migraine	Feb-09
52270	J094200	11	0	0	0	Moeller's glossodynia exfoliativa	Feb-09
67072	PKy5100	5	0	0	0	Mohr's syndrome	Feb-09

For peer review only - http://bmjopen.bmj.com/site/about/guidelines.xhtml

36276	N043300	127	0	1	0	Monarticular juvenile rheumatoid arthritis	Feb-09
50604	13L2.12	9	0	0	0	Mongol child in family	Feb-09
23489	PJ0..11	171	0	6	0	Mongolism	Feb-09
37105	PJ3..00	25	0	0	0	Monosomies and deletions from the autosomes	Feb-09
98395	PJ3z.00	4	0	0	0	Monosomies and deletions from the autosomes NOS	Jan-10
58584	C375411	31	0	0	0	Morquio - Brailsford syndrome	Feb-09
105345	PJy1300	1	0	0	0	Mosaic including XXXXY	Dec-12
32783	PJy1100	24	0	1	0	Mosaic XO/XX	Feb-09
33269	PJy1000	12	0	2	0	Mosaic XO/XY	Feb-09
64578	PJy1200	2	0	1	0	Mosaic XY/XXY	Feb-09
26140	PJz0.00	93	0	0	0	Mosaicism NOS	Feb-09
23643	PJy5.00	9	0	0	0	"Mosaicism, lines with various numbers of X chromosomes"	Feb-09
65699	F255012	18	0	0	0	Motor epilepsy	Feb-09
93694	PJ9..00	15	0	1	0	Mowat-Wilson syndrome	Feb-09
11946	PD13.11	745	0	25	0	Multicystic kidney	Feb-09
30041	C164.13	227	0	3	0	Multicystic ovaries	Feb-09
18331	PD13.00	251	0	4	0	Multicystic renal dysplasia	Feb-09
16530	PK7..00	134	0	6	0	Multiple congenital anomalies NOS	Feb-09
98724	PK7z.00	4	0	0	0	Multiple congenital anomalies NOS	Mar-10
50980	P841.12	22	0	0	0	Multiple congenital bronchogenic cysts	Feb-09
109441	P240100	1	0	0	0	Multiple congenital cerebral cysts	Mar-15
69886	F201.00	16	0	0	0	Multiple sclerosis of the spinal cord	Feb-09
57338	G544X00	62	0	3	0	"Multiple valve disease, unspecified"	Feb-09
40239	G544.00	46	0	2	0	Multiple valve diseases	Feb-09
22796	ZT4C211	229	0	14	0	Mute	Feb-09

For peer review only - http://bmjopen.bmj.com/site/about/guidelines.xhtml

36541	E2D2211	56	0	4	0	Mutism of childhood or adolescence	Feb-09
27515	F380z00	179	0	2	0	Myasthenia gravis NOS	Feb-09
95005	F381200	1	0	0	0	Myasthenic syndrome due to botulism	Feb-09
39420	F381300	39	0	2	0	Myasthenic syndrome due to diabetic amyotrophy	Feb-09
51640	F381.00	6	0	1	0	Myasthenic syndrome due to disease EC	Feb-09
65825	F381z00	11	0	0	0	Myasthenic syndrome due to disease NOS	Feb-09
61069	F381400	8	0	0	0	Myasthenic syndrome due to hypothyroidism	Feb-09
57551	F381100	2	0	0	0	Myasthenic syndrome due to other malignancy	Feb-09
56973	F381500	6	0	0	0	Myasthenic syndrome due to pernicious anaemia	Feb-09
47695	F381600	3	0	0	0	Myasthenic syndrome due to thyrotoxicosis	Feb-09
94690	F13B.00	191	0	13	0	Myoclonic dystonia	Feb-09
45602	F132200	80	0	4	0	Myoclonic encephalopathy	Feb-09
8487	F132z12	1879	0	176	0	Myoclonic seizure	Feb-09
37897	F132z00	229	0	10	0	Myoclonus NOS	Feb-09
96966	N248000	1417	0	28	0	Myofascial pain syndrome	Jun-09
105696	PG42100	1	0	0	0	Myotonic chondrodysplasia	Jan-13
44867	F392z00	97	0	3	0	Myotonic disorder NOS	Feb-09
11779	F271.00	4394	0	441	0	Narcolepsy	Feb-09
56614	Q488.00	23	0	0	0	Neonatal cerebral ischaemia	Feb-09
48202	Q48A.00	49	0	0	0	Neonatal cerebral leukomalacia	Feb-09
65686	Q444.12	4	0	0	0	Neonatal hypoparathroidism	Feb-09
106500	Q44A.00	1	0	0	0	Neonatal hypoparathyroidism	Aug-13
99435	N000500	14	0	0	0	Neonatal lupus erythematosus	May-10

For peer review only - http://bmjopen.bmj.com/site/about/guidelines.xhtml

37782	F251100	110	0	2	0	Neonatal myoclonic epilepsy	Feb-09
10647	K02..11	392	0	10	0	Nephritis - chronic	Feb-09
4850	K03..11	203	0	6	0	Nephritis and nephropathy unspecified	Feb-09
33580	K03..00	491	0	17	0	Nephritis and nephropathy unspecified	Feb-09
94350	K032z00	3	0	0	0	Nephritis unsp+membranoprolif glomerulonephritis lesion NOS	Feb-09
67193	K032y00	29	0	1	0	Nephritis unsp+OS membranoprolif glomerulonephritis lesion	Feb-09
2773	K0...00	1430	0	97	2	"Nephritis, nephrosis and nephrotic syndrome"	Feb-09
30310	K081.00	249	0	7	0	Nephrogenic diabetes insipidus	Feb-09
42042	PD12011	17	0	0	0	Nephronophthisis	Feb-09
109106	PD12200	2	0	0	0	Nephronophthisis - medullary cystic disease	Jan-15
68112	C373600	7	0	0	0	Nephropathic amyloidosis	Feb-09
11875	K02..12	875	0	41	0	Nephropathy - chronic	Feb-09
41159	K0C1.00	63	0	0	0	Nephropathy induced by other drugs meds and biologl substncs	Feb-09
57784	K0C2.00	13	0	0	0	Nephropathy induced by unspec drug medicament or biol subs	Feb-09
38312	L162.12	16	0	2	1	Nephropathy NOS in pregnancy without hypertension	Feb-09
11873	K03..12	1478	0	43	0	"Nephropathy, unspecified"	Feb-09
21947	K017.00	129	0	1	0	Nephrotic syn difus mesangial prolifertiv glomerulonephritis	Feb-09
21989	K019.00	73	0	1	0	"Nephrotic syn,diffuse mesangiocapillary glomerulonephritis"	Feb-09
50472	K018.00	4	0	0	0	"Nephrotic syn,difus endocapilary proliftv glomerulonephritis"	Feb-09

For peer review only - http://bmjopen.bmj.com/site/about/guidelines.xhtml
For peer review only

2999	K01..00	15444	0	855	5	Nephrotic syndrome	Feb-09
47922	K01x000	80	0	2	0	Nephrotic syndrome in amyloidosis	Feb-09
2471	K01x100	283	0	7	0	Nephrotic syndrome in diabetes mellitus	Feb-09
108816	K01x.00	2	0	0	0	Nephrotic syndrome in diseases EC	Nov-14
99201	K01x200	1	0	0	0	Nephrotic syndrome in malaria	Apr-10
58750	K01x300	7	0	0	0	Nephrotic syndrome in polyarteritis nodosa	Feb-09
47672	K01x400	57	0	3	0	Nephrotic syndrome in systemic lupus erythematosus	Feb-09
27427	K01z.00	305	0	9	0	Nephrotic syndrome NOS	Feb-09
1803	K011.00	515	0	9	0	Nephrotic syndrome with membranous glomerulonephritis	Feb-09
29634	K013.00	180	0	4	0	Nephrotic syndrome with minimal change glomerulonephritis	Feb-09
94373	K01y.00	4	0	0	0	Nephrotic syndrome with other pathological kidney lesions	Feb-09
9840	K010.00	19	0	0	0	Nephrotic syndrome with proliferative glomerulonephritis	Feb-09
56987	K01A.00	12	0	0	0	"Nephrotic syndrome, dense deposit disease"	Feb-09
17365	K01B.00	52	0	0	0	"Nephrotic syndrome, diffuse crescentic glomerulonephritis"	Feb-09
22852	K015.00	208	0	20	0	"Nephrotic syndrome, focal and segmental glomerular lesions"	Feb-09
23913	K014.00	66	0	1	0	"Nephrotic syndrome, minor glomerular abnormality"	Feb-09
99644	K012.00	4	0	0	0	Nephrotic syndrome+membranoproliferative glomerulonephritis	Jun-10
3171	F591211	2006	0	241	0	Nerve deafness	Feb-09
104026	F38..11	42	0	5	0	Neuromuscular disease	May-12

For peer review only - http://bmjopen.bmj.com/site/about/guidelines.xhtml

64871	N082N00	5	0	0	0	Neuromuscular dislocation of the hip	Feb-09
44795	F210.00	149	0	4	0	Neuromyelitis optica	Feb-09
101786	F392400	58	0	1	0	Neuromyotonia	Feb-11
10234	J61y100	32345	0	773	2	Non-alcoholic fatty liver	Feb-09
57897	D111.00	7	0	1	0	Non-autoimmune haemolytic anaemia	Feb-09
94214	D111z00	2	0	0	0	Non-autoimmune haemolytic anaemia NOS	Feb-09
69278	C109E00	18	0	0	0	Non-insulin depend diabetes mellitus with diabetic cataract	Feb-09
40962	C109H00	10	0	0	0	Non-insulin dependent d m with neuropathic arthropathy	Feb-09
506	C100112	95279	0	4539	11	Non-insulin dependent diabetes mellitus	Feb-09
8403	C109700	1745	0	45	0	Non-insulin dependent diabetes mellitus - poor control	Feb-09
24693	C109G00	4	0	0	0	Non-insulin dependent diabetes mellitus with arthropathy	Feb-09
40401	C109500	14	0	1	0	Non-insulin dependent diabetes mellitus with gangrene	Feb-09
43785	C109D00	10	0	0	0	Non-insulin dependent diabetes mellitus with hypoglyca coma	Feb-09
72320	C109A00	3	0	1	0	Non-insulin dependent diabetes mellitus with mononeuropathy	Feb-09
59365	C109C00	60	0	2	0	Non-insulin dependent diabetes mellitus with nephropathy	Feb-09
45467	C109B00	14	0	0	0	Non-insulin dependent diabetes mellitus with polyneuropathy	Feb-09
34912	C109400	229	0	4	0	Non-insulin dependent diabetes mellitus with ulcer	Feb-09

For peer review only - http://bmjopen.bmj.com/site/about/guidelines.xhtml

54212	C109F00	9	0	0	0	Non-insulin-dependent d m with peripheral angiopath	Feb-09
62146	C109300	9	0	2	0	Non-insulin-dependent diabetes mellitus with multiple comps	Feb-09
55842	C109200	15	0	0	0	Non-insulin-dependent diabetes mellitus with neuro comps	Feb-09
50429	C109100	19	0	3	0	Non-insulin-dependent diabetes mellitus with ophthalm comps	Feb-09
52303	C109000	16	0	2	0	Non-insulin-dependent diabetes mellitus with renal comps	Feb-09
29979	C109900	267	0	6	0	Non-insulin-dependent diabetes mellitus without complication	Feb-09
10068	PKy8000	741	0	36	0	Noonan's syndrome	Feb-09
15566	E203z00	1842	0	105	0	Obsessive-compulsive disorder NOS	Feb-09
3208	E203.00	16765	0	2315	0	Obsessive-compulsive disorders	Feb-09
46177	PD2z.00	69	0	2	0	Obstructive defect of renal pelvis or ureter NOS	Feb-09
99128	P6y3.00	10	0	0	0	Obstructive heart anomaly NEC	Apr-10
97818	P6y3z00	4	0	0	0	Obstructive heart anomaly NEC NOS	Oct-09
101873	337C.00	7	0	0	13	Obstructive ventilatory defect	Mar-11
108543	PKy5M00	3	0	0	0	Oculofaciocardiodental syndrome	Aug-14
68816	A213.00	1	0	0	0	Oculoglandular tularaemia	Feb-09
44064	PG0E.00	2	0	0	0	Oculomandibular dysostosis	Feb-09
111653	PKy5900	1	0	0	0	Oculo-palato-digital syndrome	Feb-17
38448	F391700	68	0	3	0	Oculopharyngeal muscular dystrophy	Feb-09
37408	PA3..00	39	0	1	0	"Oesophageal atresia, stenosis and fistula"	Feb-09
16713	J103.00	1924	0	53	0	Oesophageal stricture and stenosis	Feb-09
41141	J103z00	658	0	8	0	Oesophageal stricture and stenosis NOS	Feb-09
37868	PA32100	52	0	1	0	Oesophagotracheal fistula	Feb-09

For peer review only - http://bmjopen.bmj.com/site/about/guidelines.xhtml

61322	PKyG.11	24	0	0	0	Ohdo blepharophimosis syndrome	Feb-09
51998	F259.11	21	0	0	0	Ohtahara syndrome	Feb-09
38418	J32..11	65	0	1	0	Omphalocele	Feb-09
100368	J321000	1	0	0	0	Omphalocele with obstruction	Aug-10
3826	F4H1.00	5675	0	242	0	Optic atrophy	Feb-09
95763	F4H1300	4	0	0	0	Optic atrophy due to retinal dystrophy	Feb-09
41833	F4H1z00	172	0	3	0	Optic atrophy NOS	Feb-09
47676	F4H5z00	15	0	2	0	Optic chiasm disorder NOS	Feb-09
47132	F4H5.00	35	0	2	0	Optic chiasm disorders	Feb-09
63298	SJ01.00	4	0	0	0	Optic chiasm injury	Feb-09
26835	F4H3z00	280	0	2	0	Optic neuritis NOS	Feb-09
49307	PKy5000	29	0	6	0	Oral - facial - digital syndrome	Feb-09
56160	PGy0212	2	0	0	0	Orbinsky syndrome	Feb-09
29616	J08z900	90	0	2	0	Orofacial Crohn's disease	Feb-09
23356	F138100	657	0	29	5	Orofacial dyskinesia	Feb-09
22814	J08zB00	346	0	12	0	Orofacial granulomatosis	Feb-09
3156	N327.00	5189	0	322	1	Osteochondritis dissecans	Feb-09
38335	N327100	51	0	0	0	Osteochondritis dissecans of lateral femoral condyle	Feb-09
42068	N327y00	26	0	2	0	Osteochondritis dissecans of other site	Feb-09
54563	N327000	63	0	0	0	Osteochondritis dissecans of patella	Feb-09
70914	N327400	16	0	0	0	Osteochondritis dissecans of the capitellum	Feb-09
34206	N327800	7	0	0	0	Osteochondritis dissecans of the femoral head	Feb-09
103515	N327300	2	0	0	0	Osteochondritis dissecans of the humeral head	Jan-12
97447	N327500	3	0	0	0	Osteochondritis dissecans of the radial head	Aug-09

For peer review only - http://bmjopen.bmj.com/site/about/guidelines.xhtml

38518	N327900	48	0	0	0	Osteochondritis dissecans of the talus	Feb-09
97466	N327700	2	0	0	0	Osteochondritis dissecans of the wrist	Aug-09
63565	PGW..00	4	0	2	0	Osteochondrodyspl with defct growth tub bone spine unspec	Feb-09
68427	PG5z.11	10	0	1	0	Osteochondrodysplasia	Feb-09
55775	C375.17	19	0	0	0	Osteochondrodystrophy	Feb-09
22861	N32zz00	157	0	2	0	Osteochondropathy NOS	Feb-09
17135	N32z300	969	0	7	0	Osteochondrosis NOS	Feb-09
36753	PG5..00	29	0	2	0	Osteodysplasia	Feb-09
55331	PG5z.00	12	0	1	0	Osteodysplasia NOS	Feb-09
96403	PG50.00	5	0	0	0	"Osteodysplasia, unspecified"	Apr-09
38450	PG5..11	118	0	2	0	Osteodystrophy	Feb-09
26864	N33zD00	318	0	5	0	Osteolysis	Feb-09
22417	N33zH00	172	0	9	0	Osteolytic lesion	Feb-09
29734	N33z.11	101	0	25	0	Osteolytic lesion	Feb-09
4794	B73..12	4464	0	443	0	Osteoma	Feb-09
29735	B30..12	128	0	1	0	Osteoma	Feb-09
10450	C28..11	4171	0	150	8	Osteomalacia	Feb-09
37350	C282.00	144	0	4	0	Osteomalacia unspecified	Feb-09
42657	N334900	398	0	9	0	Osteonecrosis due to drugs	Feb-09
69157	N334C00	7	0	0	0	Osteonecrosis due to haemoglobinopathy	Feb-09
45737	N334A00	181	0	8	0	Osteonecrosis due to previous trauma	Feb-09
58843	N334B00	10	0	0	0	Osteonecrosis in caisson disease	Feb-09
59172	PG42.16	16	0	0	0	Osteopathia striata	Feb-09
54213	N307.00	54	0	5	0	Osteopathy from poliomyelitis	Feb-09
37298	N3...00	200	0	4	0	Osteopathy/chondropathy/acquired musculoskeletal deformity	Feb-09
101249	2126600	389	0	24	0	Osteopenia resolved	Jan-11

For peer review only - http://bmjopen.bmj.com/site/about/guidelines.xhtml

21970	N21z312	130	0	4	0	Osteophyte of unspecified site	Feb-09
39943	PG53.00	71	0	2	0	Osteopoikilosis	Feb-09
277	N330.00	329884	0	14435	329	Osteoporosis	Feb-09
25650	N330D00	67	0	2	0	Osteoporosis due to corticosteroids	Feb-09
31580	N330A00	10	0	0	0	Osteoporosis in endocrine disorders	Feb-09
60433	N330900	17	0	0	0	Osteoporosis in multiple myelomatosis	Feb-09
34798	N330z00	4190	0	116	0	Osteoporosis NOS	Feb-09
33526	N331300	55	0	0	0	Osteoporosis of disuse with pathological fracture	Feb-09
51053	P561.00	53	0	0	0	Ostium primum defect	Feb-09
54243	P55z.00	73	0	0	0	Ostium secundum atrial septal defect NOS	Feb-09
104307	2126800	83	0	8	0	Ostium secundum atrial septal defect resolved	Jun-12
54838	C155z00	27	0	0	0	Other adrenal hypofunction NOS	Feb-09
67273	C152700	1	0	0	0	Other adrenogenital syndrome with salt loss	Feb-09
69764	C152800	15	0	1	0	Other adrenogenital syndrome without mention of salt loss	Feb-09
50220	P72..00	70	0	2	0	Other anomalies of aorta	Feb-09
44781	P72z.00	39	0	0	0	Other anomalies of aorta NOS	Feb-09
50278	PB5..00	12	0	0	0	Other anomalies of intestine	Feb-09
29373	P83..00	22	0	3	0	"Other anomalies of larynx, trachea and bronchus"	Feb-09
36779	P83z.00	6	0	0	0	"Other anomalies of larynx, trachea or bronchus NOS"	Feb-09
11945	P72zz00	60	0	1	0	Other anomaly of aorta NOS	Feb-09
104976	P83yy00	3	0	0	0	Other anomaly of bronchus	Sep-12
63507	P83yw00	12	0	0	0	Other anomaly of larynx	Feb-09
63637	P83y.00	4	0	0	0	"Other anomaly of larynx, trachea and bronchus"	Feb-09

For peer review only - http://bmjopen.bmj.com/site/about/guidelines.xhtml

103042	P83yz00	1	0	0	0	"Other anomaly of larynx, trachea or bronchus NOS"	Oct-11
69913	PG0yz00	9	0	0	0	Other anomaly of skull or face bone NOS	Feb-09
56878	PG1y.00	33	0	1	0	Other anomaly of spine	Feb-09
22768	PG1yz00	107	0	11	1	Other anomaly of spine NOS	Feb-09
70193	P83yx00	5	0	0	0	Other anomaly of trachea	Feb-09
66220	PFyz.00	7	0	1	0	Other anomaly of unspecified limb NOS	Feb-09
71400	F15y.00	5	0	4	0	Other anterior horn cell disease	Feb-09
15488	C134z00	1176	0	255	4	Other anterior pituitary disorder NOS	Feb-09
23560	C131.00	81	0	4	0	Other anterior pituitary hyperfunction	Feb-09
50762	F130z00	35	0	0	0	Other basal ganglia degenerative disease NOS	Feb-09
21863	F130.00	207	0	16	8	Other basal ganglia degenerative diseases	Feb-09
72653	G565300	29	0	6	0	Other bilateral bundle branch block	Feb-09
37613	J66yz00	76	0	5	4	Other bile duct disorder NOS	Feb-09
29066	J66..00	78	0	1	0	Other biliary tract disorders	Feb-09
69100	F427H00	7	0	0	0	Other Bruch's membrane dystrophy	Feb-09
39003	G565z00	146	0	0	0	Other bundle branch block NOS	Feb-09
31133	G57yz00	191	0	6	0	Other cardiac dysrhythmia NOS	Feb-09
7827	G57y.00	2771	0	396	18	Other cardiac dysrhythmias	Feb-09
65499	P7yzz00	8	0	0	0	Other cardiovascular system anomaly NOS	Feb-09
101592	P7yz.00	6	0	0	0	Other cardiovascular system anomaly NOS	Jan-11
34976	F11y.00	30	0	1	0	Other cerebral degeneration	Feb-09
31524	F11yz00	27	0	1	0	Other cerebral degeneration NOS	Feb-09
31892	F11..00	241	0	1	0	Other cerebral degenerations	Feb-09

For peer review only - http://bmjopen.bmj.com/site/about/guidelines.xhtml

104782	F2By.00	5	0	0	0	Other cerebral palsy	Aug-12
71819	E14y.00	1	0	1	0	Other childhood psychoses	Feb-09
66757	E14yz00	4	0	0	0	Other childhood psychoses NOS	Feb-09
60960	K02y.00	12	0	0	0	Other chronic glomerulonephritis	Feb-09
63615	K02yz00	12	0	0	0	Other chronic glomerulonephritis NOS	Feb-09
54113	G41y.00	169	0	0	0	Other chronic pulmonary heart disease	Feb-09
71046	G41yz00	24	0	0	0	Other chronic pulmonary heart disease NOS	Feb-09
26291	PF5r.00	247	0	20	0	Other congenital anomalies of fingers	Feb-09
66010	PF5u.00	23	0	0	0	Other congenital anomalies of forearm	Feb-09
59150	PF6v.00	16	0	1	0	Other congenital anomalies of lower leg	Feb-09
25190	PF6x.00	45	0	2	0	Other congenital anomalies of pelvis	Feb-09
55114	PF6xz00	5	0	0	0	Other congenital anomalies of pelvis NOS	Feb-09
65384	P76..11	8	0	0	0	Other congenital anomalies of peripheral arteries	Feb-09
22070	P76..12	109	0	23	0	Other congenital anomalies of peripheral veins	Feb-09
31378	PG3x.00	72	0	2	0	Other congenital anomalies of ribs	Feb-09
63972	PF5w.00	21	0	0	0	Other congenital anomalies of shoulder	Feb-09
51424	PG3y.00	13	0	0	0	Other congenital anomalies of sternum	Feb-09
92299	PF6w.00	9	0	0	0	Other congenital anomalies of upper leg	Feb-09

For peer review only - http://bmjopen.bmj.com/site/about/guidelines.xhtml

48510	PF5x.00	21	0	2	0	Other congenital anomalies of whole arm	Feb-09
61558	PF5uz00	7	0	0	0	Other congenital anomaly forearm NOS	Feb-09
107541	PK1yz00	1	0	0	0	Other congenital anomaly of adrenal gland NOS	Dec-13
94116	PB6yy00	4	0	0	0	Other congenital anomaly of hepatic or bile ducts	Feb-09
66182	PB6yw00	12	0	0	0	Other congenital anomaly of liver	Feb-09
37517	PA25y00	31	0	3	0	Other congenital anomaly of palate	Feb-09
73738	P76yz00	12	0	0	0	Other congenital anomaly of peripheral vascular system NOS	Feb-09
22437	PCyw.00	247	0	58	0	Other congenital anomaly of testis or scrotum	Feb-09
53178	F23y.00	38	0	0	0	Other congenital cerebral palsy	Feb-09
9778	P7...00	48	0	3	0	Other congenital circulatory system anomalies	Feb-09
59031	PD1yz00	8	0	0	0	Other congenital cystic kidney disease NOS	Feb-09
42998	PB...00	31	0	1	0	Other congenital digestive system anomaly	Feb-09
5621	P6...00	654	0	30	0	Other congenital heart anomalies	Feb-09
63834	PF63z00	38	0	1	0	Other congenital hip joint deformity NOS	Feb-09
14649	PF...00	435	0	42	0	Other congenital limb anomalies	Feb-09
37167	PE9..11	243	0	12	0	Other congenital musculoskeletal deformity	Feb-09
62976	P355.00	29	0	0	0	Other congenital retinal changes	Feb-09
25692	P355z00	234	0	21	0	Other congenital retinal changes NOS	Feb-09
72991	PA...00	5	0	0	0	Other congenital upper alimentary tract anomalies	Feb-09

For peer review only

68138	7A04.00	3	0	0	0	Other connection from aorta to pulmonary artery	Feb-09
44962	C153.00	9	0	0	0	Other corticoadrenal overactivity	Feb-09
57274	D01z.00	81	0	3	0	Other deficiency anaemias NOS	Feb-09
69820	C13y400	5	0	1	0	Other diencephalic syndrome	Feb-09
95921	PG44z00	2	0	0	0	Other dwarfing syndromes NOS	Feb-09
38307	F25y.00	338	0	10	0	Other forms of epilepsy	Feb-09
9979	F25yz00	364	0	5	0	Other forms of epilepsy NOS	Feb-09
28031	F26y.00	505	0	4	0	Other forms of migraine	Feb-09
28092	F26yz00	453	0	3	0	Other forms of migraine NOS	Feb-09
32176	F4K5600	61	0	0	0	Other forms of nystagmus	Feb-09
73663	P74z.00	2	0	0	0	Other great vein anomalies	Feb-09
73554	P74zz00	6	0	0	0	Other great vein anomaly NOS	Feb-09
39843	G566.00	280	0	1	0	Other heart block	Feb-09
46178	G566z00	168	0	1	0	Other heart block NOS	Feb-09
27304	C111.00	362	0	7	0	Other hyperinsulinism	Feb-09
50491	C111z00	51	0	2	0	Other hyperinsulinism NOS	Feb-09
44026	N10y.00	57	0	4	2	Other inflammatory spondylopathies	Feb-09
37892	N10yz00	37	0	0	0	Other inflammatory spondylopathies NOS	Feb-09
7196	N045.00	3433	0	94	0	Other juvenile arthritis	Feb-09
50425	N326.00	13	0	0	0	Other juvenile osteochondroses	Feb-09
43899	F427700	57	0	0	0	Other pigmented retinal dystrophies	Feb-09
44186	C13y.00	5	0	0	0	Other pituitary disorders + diencephalohypophyseal syndromes	Feb-09
22836	C163300	308	0	20	0	Ovarian hypogonadism	Feb-09
23802	C163.11	24	0	2	0	Ovarian hypogonadism	Feb-09
37902	N310z00	367	0	4	0	Paget's disease NOS	Feb-09
3528	N310.11	13822	0	1224	12	Paget's disease of bone	Feb-09

For peer review only - http://bmjopen.bmj.com/site/about/guidelines.xhtml

56078	N310y00	95	0	5	0	Paget's disease OS	Feb-09
95037	N310A00	1	0	0	0	Paget's disease-carpal bone	Feb-09
50862	N310000	5	0	0	0	Paget's disease-cervical spine	Feb-09
68199	N310500	17	0	0	0	Paget's disease-clavicle	Feb-09
57905	N310E00	38	0	3	0	Paget's disease-femur	Feb-09
72456	N310H00	1	0	0	0	Paget's disease-fibula	Feb-09
63779	N310700	5	0	0	0	Paget's disease-humerus	Feb-09
56021	N310200	48	0	3	0	Paget's disease-lumbar spine	Feb-09
66795	N310x00	16	0	0	0	Paget's disease-multiple sites	Feb-09
56897	N310F00	6	0	0	0	Paget's disease-patella	Feb-09
27501	N310D00	186	0	1	0	Paget's disease-pelvis	Feb-09
65387	N310800	6	0	1	0	Paget's disease-radius	Feb-09
56578	N310300	23	0	0	0	Paget's disease-sacrum	Feb-09
70397	N310600	3	0	0	0	Paget's disease-scapula	Feb-09
51146	N310P00	29	0	1	0	Paget's disease-skull	Feb-09
71514	N310100	4	0	1	0	Paget's disease-thoracic spine	Feb-09
35769	N310G00	34	0	0	0	Paget's disease-tibia	Feb-09
73964	N310900	1	0	0	0	Paget's disease-ulna	Feb-09
5026	C132.00	2554	0	129	14	Panhypopituitarism	Feb-09
33653	C132z00	54	0	0	0	Panhypopituitarism NOS	Feb-09
98998	PKy5011	5	0	2	0	Papillon-Leage-Psaume syndrome	Apr-10
1494	E103.00	27706	0	1069	0	Paranoid schizophrenia	Feb-09
36172	E103500	323	0	0	0	Paranoid schizophrenia in remission	Feb-09
9281	E103z00	346	0	4	0	Paranoid schizophrenia NOS	Feb-09
99040	F230.11	2	0	0	0	Paraplegia - congenital	Apr-10
12386	C332z00	742	0	28	0	Paraproteinaemia NOS	Feb-09
64789	C12z.00	48	0	0	0	Parathyroid disorder NOS	Feb-09
16433	C12..00	456	0	72	2	Parathyroid gland disorders	Feb-09
107472	G573600	155	0	6	0	Paroxysmal atrial flutter	Dec-13
23647	G570100	178	0	1	0	Paroxysmal atrioventricular tachycardia	Feb-09
51845	G570200	51	0	2	0	Paroxysmal junctional tachycardia	Feb-09

For peer review only - http://bmjopen.bmj.com/site/about/guidelines.xhtml

35612	D112100	252	0	9	0	Paroxysmal nocturnal haemoglobinuria	Feb-09
29491	G570300	296	0	23	0	Paroxysmal nodal tachycardia	Feb-09
107057	F139000	6	0	0	0	Paroxysmal non-kinesigenic dyskinesia	Oct-13
4940	G570.00	11026	0	444	0	Paroxysmal supraventricular tachycardia	Feb-09
35124	G570z00	274	0	11	0	Paroxysmal supraventricular tachycardia NOS	Feb-09
1381	G572z00	7791	0	848	1	Paroxysmal tachycardia NOS	Feb-09
25266	G572.00	805	0	14	0	Paroxysmal tachycardia unspecified	Feb-09
24409	F335.11	125	0	8	0	Parsonage - Aldren - Turner syndrome	Feb-09
98870	F255311	3	0	0	0	Partial epilepsy with autonomic symptoms	Apr-10
32288	F254.00	466	0	12	0	Partial epilepsy with impairment of consciousness	Feb-09
31920	F254z00	212	0	8	0	Partial epilepsy with impairment of consciousness NOS	Feb-09
26015	F255.00	391	0	15	0	Partial epilepsy without impairment of consciousness	Feb-09
27526	F255z00	126	0	4	0	Partial epilepsy without impairment of consciousness NOS	Feb-09
26733	F255y00	63	0	1	0	Partial epilepsy without impairment of consciousness OS	Feb-09
68651	7114200	4	0	0	0	Partial parathyroidectomy + parathyroid tissue transposition	Feb-09
43202	7114300	277	0	1	0	Partial parathyroidectomy NEC	Feb-09
101309	PJ02.11	5	0	0	0	Partial trisomy 21 in Down's syndrome	Jan-11
100174	PJ51z00	3	0	0	0	Partial trisomy syndrome NOS	Jul-10
19062	PJ51.00	38	0	0	0	Partial trisomy syndromes	Feb-09

For peer review only - http://bmjopen.bmj.com/site/about/guidelines.xhtml

For peer review only

50985	D402.16	5	0	0	0	Pelger - Huet anomaly	Feb-09
31444	C252.00	45	0	3	0	Pellagra	Feb-09
40319	N216211	125	0	2	0	Pellegrini - Stieda syndrome	Feb-09
58116	M145z00	82	0	1	0	Pemphigoid NOS	Feb-09
53763	M144z00	21	0	0	0	Pemphigus NOS	Feb-09
11892	C030.00	132	0	4	0	Pendred's syndrome	Feb-09
96277	G573400	247	0	2	0	Permanent atrial fibrillation	Apr-09
28901	F361.00	349	0	32	0	Peroneal muscular atrophy	Feb-09
70040	F361z00	10	0	0	0	Peroneal muscular atrophy NOS	Feb-09
2907	F250000	11582	0	1229	1	Petit mal (minor) epilepsy	Feb-09
15607	PK60.00	249	0	27	0	Peutz - Jegher's syndrome	Feb-09
3354	K274.00	24239	0	3840	0	Peyronie's disease	Feb-09
18231	B540.11	968	0	67	4	Phaeochromocytoma	Feb-09
52030	AB68.00	3	0	0	0	Phaeohyphomycosis	Feb-09
4318	C301.00	3036	0	3989	3906	Phenylketonuria	Feb-09
38071	13VM.00	844	0	16	0	Physical disability	Feb-09
3970	6665	1145	0	30	0	Physical handicap problem	Feb-09
46999	Z88..11	7	0	0	0	Physical health handicap	Feb-09
93788	Z77C.32	1	0	0	0	Physical health handicap	Feb-09
11136	F111.00	316	0	13	0	Pick's disease	Feb-09
59140	G532100	1	0	0	0	Pick's disease of heart	Feb-09
24755	C38y.11	152	0	14	0	Pickwickian syndrome	Feb-09
38294	C38y000	45	0	6	0	Pickwickian syndrome	Feb-09
4046	PG0C.00	816	0	16	0	Pierre - Robin syndrome	Feb-09
96210	PG0J.00	7	0	0	0	Pierre Robin association	Apr-09
95730	N321511	3	0	0	0	Pierson's disease	Feb-09
68146	C1zy000	5	0	1	0	Pineal gland dysfunction	Feb-09
39541	710..11	36	0	3	0	Pineal gland operations	Feb-09
2321	B7H2.11	6910	0	352	3	Pituitary adenoma	Feb-09
53682	C150200	36	0	0	0	Pituitary dependent Cushing's syndrome	Feb-09
12449	C13z.00	241	0	12	0	Pituitary disorders NOS	Feb-09

For peer review only - http://bmjopen.bmj.com/site/about/guidelines.xhtml

20287	C133.00	437	0	53	16	Pituitary dwarfism	Feb-09
61409	C133z00	2	0	0	0	Pituitary dwarfism NOS	Feb-09
36838	M163.11	23	0	0	0	Pityriasis circinata	Feb-09
107401	M162311	203	0	3	0	Pityriasis lichenoides chronica	Dec-13
107491	M162111	71	0	6	0	Pityriasis lichenoides et varioliformis acuta	Dec-13
36466	M15y200	172	0	3	0	Pityriasis rubra (Hebra)	Feb-09
48641	M165200	17	0	0	0	Pityriasis streptogenes	Feb-09
62323	G75..00	12	0	0	0	Polyarteritis nodosa and allied conditions	Feb-09
68136	G75z.00	128	0	0	0	Polyarteritis nodosa and allied conditions NOS	Feb-09
1670	N065z11	20288	0	1929	38	Polyarthritis	Feb-09
7454	N065.11	29871	0	2919	4	Polyarthropathy NEC	Feb-09
4504	PD1..13	1782	0	250	0	Polycystic kidney	Feb-09
4503	PD11.00	6901	0	595	3	Polycystic kidney disease	Feb-09
56852	PD11z00	226	0	8	0	Polycystic kidney disease NOS	Feb-09
4505	PD11100	482	0	30	0	"Polycystic kidneys, adult type"	Feb-09
44611	D410300	22	0	0	0	Polycythaemia due to cyanotic respiratory disease	Feb-09
68850	Q454000	4	0	0	0	Polycythaemia due to donor twin transfusion	Feb-09
102672	5A14.00	0	0	0	1	Polycythaemia irradiation	Jul-11
64182	Q454z00	27	0	0	0	Polycythaemia neonatorum NOS	Feb-09
5542	B934.11	2103	0	183	0	Polycythaemia rubra vera	Feb-09
40691	PF0z.00	136	0	0	0	Polydactyly NOS	Feb-09
102958	C320600	75	0	0	0	Polygenic hypercholesterolaemia	Sep-11
39898	C18..00	32	0	0	0	Polyglandular dysfunction and related disorders	Feb-09
47995	C18z.00	10	0	0	0	Polyglandular dysfunction NOS	Feb-09
92901	C183.00	1	0	0	0	Polyglandular hyperfunction	Feb-09
1408	N20..00	300629	0	6946	44	Polymyalgia rheumatica	Feb-09

For peer review only - http://bmjopen.bmj.com/site/about/guidelines.xhtml

15511	N004.00	3141	0	178	1	Polymyositis	Feb-09
93927	N231400	44	0	0	0	Polymyositis ossificans	Feb-09
28294	F326100	80	0	5	0	Polyneuritis cranialis	Feb-09
24222	F376.00	61	0	2	0	Polyneuropathy due to drugs	Feb-09
66336	F374000	15	0	1	0	Polyneuropathy in amyloidosis	Feb-09
73337	F374100	1	0	0	0	Polyneuropathy in beriberi	Feb-09
57313	F371.00	8	0	0	0	Polyneuropathy in collagen vascular disease	Feb-09
71258	F371z00	4	0	0	0	Polyneuropathy in collagen vascular disease NOS	Feb-09
31790	F372.00	1506	0	33	0	Polyneuropathy in diabetes	Feb-09
52089	F374300	1	0	0	0	Polyneuropathy in diphtheria	Feb-09
56272	F374.00	16	0	2	0	Polyneuropathy in disease EC	Feb-09
63555	F374z00	28	0	2	0	Polyneuropathy in disease NOS	Feb-09
44095	F371000	37	0	4	0	Polyneuropathy in disseminated lupus erythematosus	Feb-09
39692	F374400	57	0	0	0	Polyneuropathy in herpes zoster	Feb-09
68960	F374500	6	0	0	0	Polyneuropathy in hypoglycaemia	Feb-09
30537	F373.00	15	0	0	0	Polyneuropathy in malignant disease	Feb-09
100064	F374600	1	0	0	0	Polyneuropathy in mumps	Jul-10
47465	F371100	5	0	0	0	Polyneuropathy in polyarteritis nodosa	Feb-09
58758	F374800	25	0	1	0	Polyneuropathy in porphyria	Feb-09
62401	F371200	21	0	0	0	Polyneuropathy in rheumatoid arthritis	Feb-09
40751	F374900	16	0	0	0	Polyneuropathy in sarcoidosis	Feb-09
24355	F374200	9	0	0	0	Polyneuropathy in vitamin B deficiency	Feb-09
24226	F37z.11	219	0	9	0	Polyneuropathy unspecified	Feb-09
30600	PG54.00	168	0	8	0	Polyostotic fibrous dysplasia	Feb-09
93879	P41..11	10	0	0	0	Polyotia	Feb-09

For peer review only - http://bmjopen.bmj.com/site/about/guidelines.xhtml

38836	PF15.00	55	0	0	0	Polysyndactyly	Feb-09
68991	PF1z.11	1	0	0	0	Polysyndactyly	Feb-09
43743	C371z00	162	0	1	0	Porphyria NOS	Feb-09
47257	J615.11	14	0	0	0	Portal cirrhosis	Feb-09
55454	J615y00	18	0	0	0	Portal cirrhosis unspecified	Feb-09
40963	J61y300	35	0	0	0	Portal fibrosis without cirrhosis	Feb-09
5129	J623.00	4307	0	65	0	Portal hypertension	Feb-09
44881	C137100	36	0	2	0	Post-hypophysectomy hypopituitarism	Feb-09
25424	C1z1011	79	0	22	0	Precocious pubarche	Feb-09
3031	C1z1000	3154	0	645	0	Precocious puberty	Feb-09
15995	C1z1.11	558	0	86	1	Precocious puberty	Feb-09
70360	C152912	3	0	0	0	Precocious puberty with adrenal hyperplasia	Feb-09
22704	C1z1111	873	0	34	0	Precocious thelarche	Feb-09
50665	C391000	40	0	4	0	Predominantly T-cell immuno-deficiency NOS	Feb-09
106756	P711.13	1	0	0	0	Preductal aortic stenosis	Aug-13
72295	P711.00	2	0	0	0	Preductal coarctation of aorta	Feb-09
53970	C373700	26	0	0	0	Primary amyloidosis NEC	Feb-09
5638	J616000	6062	0	300	0	Primary biliary cirrhosis	Feb-09
29028	C151000	247	0	4	0	Primary hyperaldosteronism	Feb-09
18740	C120000	3636	0	136	2	Primary hyperparathyroidism	Feb-09
107704	G20..12	24	0	0	0	Primary hypertension	Feb-14
45133	E312.00	106	0	2	0	Profound mental retardation with IQ less than 20	Feb-09
107607	F591B00	50	0	0	0	Profound sensorineural hearing loss	Jan-14
52673	A413.00	66	0	5	0	Progressive multifocal leucoencephalopathy	Feb-09
49541	A413.11	26	0	1	0	Progressive multifocal leukoencephalopathy	Feb-09
37644	F132100	100	0	1	0	Progressive myoclonic epilepsy	Feb-09

For peer review only - http://bmjopen.bmj.com/site/about/guidelines.xhtml

44058	F427811	60	0	1	0	Progressive rod dystrophy	Feb-09
40553	F130400	72	0	2	0	Progressive supranuclear ophthalmoplegia	Feb-09
9385	F24y000	1339	0	59	0	Progressive supranuclear palsy	Feb-09
44141	N001000	47	0	1	0	Progressive systemic sclerosis	Feb-09
52426	C134000	54	0	5	0	Prolactin deficiency	Feb-09
26102	D30B.00	598	0	25	0	Protein C deficiency	Feb-09
8479	D309.00	1037	0	52	0	Protein S deficiency	Feb-09
22853	PG72.00	118	0	4	0	Prune belly syndrome	Feb-09
46749	C377111	12	0	1	0	Pseudo - Hurler's disease	Feb-09
43115	PG44600	34	0	0	0	Pseudoachondroplasia	Feb-09
60703	ZS42111	15	0	1	0	Pseudobulbar palsy type of dysarthria	Feb-09
2337	D41y200	228	0	14	7	Pseudocholinesterase deficiency	Feb-09
36351	PG42.17	4	0	0	0	Pseudochondroplasia	Feb-09
48532	PC7z100	7	0	0	0	Pseudohermaphrodite NOS	Feb-09
93135	C152814	2	0	0	0	"Pseudohermaphrodite, male with adrenocortical disorder"	Feb-09
26210	F391011	7	0	0	0	Pseudohypertrophic dystrophy	Feb-09
23540	C354300	199	0	6	0	Pseudohypoparathyroidism	Feb-09
49810	F280200	4	0	0	0	Pseudoporencephaly	Feb-09
65126	C371600	54	0	1	0	Pseudoporphyria	Feb-09
60587	C354311	9	0	2	0	Pseudopseudohypoparathyroidism	Feb-09
52610	F4E3411	50	0	3	0	Pseudoptosis	Feb-09
66964	F426500	8	0	0	0	Pseudoretinitis pigmentosa	Feb-09
65405	N237300	6	0	0	0	Pseudosarcomatous fibromatosis	Feb-09
49468	A57y400	21	0	1	0	Pseudoscarlatina	Feb-09
22632	F4J4600	697	0	3	0	Pseudostrabismus	Feb-09
68982	P603.11	2	0	0	0	Pseudotruncus arteriosus	Feb-09
46041	F282.11	43	0	2	0	Pseudotumour cerebri	Feb-09
24805	F4G1111	98	0	3	0	Pseudotumour of orbit	Feb-09
30272	M161200	41	0	0	0	Psoriasis circinata	Feb-09

For peer review only - http://bmjopen.bmj.com/site/about/guidelines.xhtml

42008	M161300	82	0	2	0	Psoriasis diffusa	Feb-09
21633	M161500	19	0	0	0	Psoriasis geographica	Feb-09
65839	M161700	5	0	0	0	Psoriasis gyrata	Feb-09
48257	M161800	11	0	0	0	Psoriasis inveterata	Feb-09
172	M161z00	348556	0	12706	0	Psoriasis NOS	Feb-09
60169	M161900	2	0	0	0	Psoriasis ostracea	Feb-09
24136	M161C00	63	0	2	0	Psoriasis punctata	Feb-09
26368	M160000	191	0	4	0	Psoriasis spondylitica	Feb-09
162	M161000	386002	0	22325	19	Psoriasis unspecified	Feb-09
30210	M161F00	904	0	15	0	Psoriasis vulgaris	Feb-09
96880	M160.11	6793	0	225	0	Psoriatic arthritis	May-09
476	M160.00	37950	0	2913	1	Psoriatic arthropathy	Feb-09
12500	M160z00	1575	0	107	0	Psoriatic arthropathy NOS	Feb-09
23634	F254100	97	0	6	0	Psychomotor epilepsy	Feb-09
37948	1P01.00	928	0	7	0	Psychomotor retardation	Feb-09
36203	F254200	8	0	1	0	Psychosensory epilepsy	Feb-09
43225	E14..00	13	0	1	0	Psychoses with origin in childhood	Feb-09
96225	P736.00	4	0	0	0	Pulmonary arterio-venous aneurysm	Apr-09
47853	P736.11	16	0	0	0	Pulmonary arterio-venous fistula	Feb-09
37807	G42y000	8	0	1	0	Pulmonary arteritis	Feb-09
67928	P731.00	2	0	0	0	Pulmonary artery agenesis	Feb-09
65029	P737.00	18	0	0	0	Pulmonary artery aneurysm	Feb-09
42127	P73z.00	31	0	0	0	Pulmonary artery anomaly NOS	Feb-09
63187	P730.00	9	0	0	0	"Pulmonary artery anomaly, unspecified"	Feb-09
7180	G4...00	230	0	10	0	Pulmonary circulation diseases	Feb-09
30214	H54..00	280	0	17	0	Pulmonary congestion and hypostasis	Feb-09
61229	H54z.00	2	0	0	0	Pulmonary congestion and hypostasis NOS	Feb-09
103472	H563200	4735	0	172	0	Pulmonary fibrosis	Dec-11

For peer review only - http://bmjopen.bmj.com/site/about/guidelines.xhtml

46736	G543200	136	0	3	0	"Pulmonary incompetence, cause unspecified"	Feb-09
23608	G543000	139	0	3	0	"Pulmonary incompetence, non-rheumatic"	Feb-09
9401	P6y2.00	1595	0	74	0	Pulmonary infundibular stenosis	Feb-09
93880	H585000	1	0	0	0	Pulmonary insufficiency following shock	Feb-09
72221	H585100	1	0	0	0	Pulmonary insufficiency following surgery	Feb-09
96754	H585200	3	0	0	0	Pulmonary insufficiency following trauma	Apr-09
38299	G543213	17	0	2	0	"Pulmonary insufficiency, cause unspecified"	Feb-09
15496	G543012	609	0	8	0	"Pulmonary regurgitation, non-rheumatic"	Feb-09
3859	H57y200	2945	0	121	0	Pulmonary sarcoidosis	Feb-09
2669	G543300	3149	0	74	1	"Pulmonary stenosis, cause unspecified"	Feb-09
61878	G543311	107	0	1	0	"Pulmonary stenosis, cause unspecified"	Feb-09
14723	G543100	1408	0	68	0	"Pulmonary stenosis, non-rheumatic"	Feb-09
39992	P60..00	201	0	2	0	Pulmonary valve anomalies	Feb-09
69940	P600.00	22	0	0	0	"Pulmonary valve anomaly, unspecified"	Feb-09
12312	G543.00	632	0	14	0	Pulmonary valve disorders	Feb-09
34932	G543400	225	0	2	0	Pulmonary valve stenosis with insufficiency	Feb-09
53944	K10y.00	128	0	0	0	Pyelonephritis and pyonephrosis unspecified	Feb-09
95710	K10y300	6	0	0	0	Pyelonephritis in diseases EC	Feb-09

For peer review only

60856	K0A2700	4	0	0	0	Recur+persist haematuria difus crescentic glomerulonephritis	Feb-09
61317	K0A2200	4	0	0	0	Recur+persist haematuria difus membranous glomerulonephritis	Feb-09
68364	K0A2100	2	0	0	0	"Recur+persist haematuria, focal+segmental glomerular lesions"	Feb-09
49642	K0A2300	11	0	0	0	Recur+persist haemuria df mesangial prolif glomerulnephritis	Feb-09
60484	K0A2500	3	0	0	0	Recur+persist hmuria df mesangiocapilary glomerulonephritis	Feb-09
53480	J614300	25	0	0	0	Recurrent hepatitis	Feb-09
25563	E113z00	1910	0	10	0	Recurrent major depressive episode NOS	Feb-09
55384	E113600	518	0	1	0	"Recurrent major depressive episodes, in full remission"	Feb-09
29342	E113100	1615	0	25	0	"Recurrent major depressive episodes, mild"	Feb-09
25697	E113300	1195	0	32	0	"Recurrent major depressive episodes, severe, no psychosis"	Feb-09
24171	E113400	493	0	9	0	"Recurrent major depressive episodes, severe, with psychosis"	Feb-09
35671	E113000	506	0	12	0	"Recurrent major depressive episodes, unspecified"	Feb-09
56273	E113500	226	0	9	0	"Recurrent major depressive episodes,partial/unspec remission"	Feb-09
46415	E111z00	94	0	1	0	Recurrent manic episode NOS	Feb-09
26227	E111.00	215	0	6	0	Recurrent manic episodes	Feb-09
37178	E111600	125	0	1	0	"Recurrent manic episodes, in full remission"	Feb-09
46425	E111100	20	0	0	0	"Recurrent manic episodes, mild"	Feb-09

For peer review only - http://bmjopen.bmj.com/site/about/guidelines.xhtml

27739	E111200	24	0	0	0	"Recurrent manic episodes, moderate"	Feb-09
58863	E111500	2	0	0	0	"Recurrent manic episodes, partial or unspecified remission"	Feb-09
65811	E111300	20	0	0	0	"Recurrent manic episodes, severe without mention psychosis"	Feb-09
32295	E111400	25	0	0	0	"Recurrent manic episodes, severe, with psychosis"	Feb-09
22003	G54z013	93	0	0	0	Regurgitation of unspecified heart valve	Feb-09
8103	A993.00	2493	0	96	0	Reiter's disease / syndrome	Feb-09
2194	A993.11	1829	0	153	7	Reiter's syndrome	Feb-09
45904	K0B2.00	2	0	0	0	Ren tub-interst disordr/blood dis+disordr inv immune mech	Feb-09
67261	K0B4.00	3	0	0	0	Ren tub-interstitl disordr/systemc connectv tiss disorder	Feb-09
48475	K08yz11	2	0	0	0	Renal acidaemia	Feb-09
30097	PD0..00	245	0	9	0	Renal agenesis and dysgenesis	Feb-09
29659	PD0z.00	84	0	6	0	Renal agenesis or dysgenesis NOS	Feb-09
27474	PD00.00	158	0	3	0	"Renal agenesis, unspecified"	Feb-09
55985	PD00z00	17	0	0	0	"Renal agenesis, unspecified NOS"	Feb-09
107765	K0J..00	4	0	0	0	Renal disorders in systemic disease	Mar-14
32963	PD30.12	17	0	0	0	Renal duplication NEC	Feb-09
34648	K080100	4	0	0	0	Renal dwarfism	Feb-09
105657	PD04200	6	0	2	0	Renal dysplasia and retinal aplasia	Jan-13
350	K06..00	38962	0	4706	59	Renal failure unspecified	Feb-09
106860	C353600	4	0	0	0	Renal failure-associated hyperphosphataemia	Aug-13
22876	K071.00	50	0	1	0	Renal fibrosis	Feb-09
41013	K08y300	15	0	0	0	Renal function impairment with growth failure	Feb-09
29638	K080.00	207	0	3	0	Renal osteodystrophy	Feb-09

For peer review only - http://bmjopen.bmj.com/site/about/guidelines.xhtml

34637	K080z00	49	0	0	0	Renal osteodystrophy NOS	Feb-09
44894	D410400	26	0	0	0	Renal polycythaemia	Feb-09
45876	F421200	14	0	2	0	Renal retinopathy	Feb-09
66062	K080300	15	0	0	0	Renal rickets	Feb-09
4480	K07z.00	46	0	2	1	Renal sclerosis NOS	Feb-09
26220	K07..00	59	0	4	0	Renal sclerosis unspecified	Feb-09
110983	K08y412	1	0	0	0	Renal tubular acidaemia	Jul-16
5072	K08y400	651	0	18	0	Renal tubular acidosis	Feb-09
41148	K0B4000	10	0	1	0	Renal tubulo-interstitial disorder in SLE	Feb-09
64622	K0B1.00	4	0	0	0	Renal tubulo-interstitial disorder/ neoplastic diseases	Feb-09
45523	K0B..00	26	0	1	0	Renal tubulo-interstitial disorders in diseases EC	Feb-09
48057	K0B5.00	21	0	0	0	Renal tubulo-interstitial disordrs in transplant rejectn	Feb-09
35157	G770.11	150	0	15	0	Rendu - Osler - Weber disease	Feb-09
25249	H59..00	2542	0	25	0	Respiratory failure	Feb-09
39079	Q319.00	49	0	0	0	Respiratory failure of newborn	Feb-09
46397	P8z..00	33	0	0	0	Respiratory system anomaly NOS	Feb-09
46398	P8...00	38	0	4	0	Respiratory system congenital anomalies	Feb-09
2745	F427600	6052	0	711	2	Retinitis pigmentosa	Feb-09
39637	F421A11	76	0	7	0	Retinitis proliferans	Feb-09
23896	J041.12	144	0	1	0	Retrognathism	Feb-09
26364	J041z00	16	0	0	0	Retrognathism NOS	Feb-09
32211	G121.00	135	0	8	0	Rheumatic aortic insufficiency	Feb-09
9391	G120.00	703	0	26	0	Rheumatic aortic stenosis	Feb-09
63960	G122.00	31	0	2	0	Rheumatic aortic stenosis with insufficiency	Feb-09
50809	G12z.00	54	0	1	0	Rheumatic aortic valve disease NOS	Feb-09
72936	G02z.00	12	0	0	0	Rheumatic chorea NOS	Feb-09

For peer review only - http://bmjopen.bmj.com/site/about/guidelines.xhtml

69995	G020.00	10	0	0	0	Rheumatic chorea with heart involvement	Feb-09
63252	G021.00	9	0	0	0	Rheumatic chorea without mention of heart involvement	Feb-09
100820	66HC.00	9806	0	5	0	Rheumatic disorder annual review invitation	Nov-10
44756	G01..00	119	0	0	0	Rheumatic fever with heart involvement	Feb-09
48189	G00..00	153	0	1	0	Rheumatic fever without heart involvement	Feb-09
107462	GA...00	47	0	2	0	Rheumatic heart disease	Dec-13
53878	G1yz000	101	0	0	0	Rheumatic heart disease unspecified	Feb-09
22262	G1yz100	149	0	11	0	Rheumatic left ventricular failure	Feb-09
51879	G111.00	208	0	19	0	Rheumatic mitral insufficiency	Feb-09
32435	G110.11	223	0	5	0	Rheumatic mitral stenosis	Feb-09
62404	G1y0.00	8	0	0	0	Rheumatic myocarditis	Feb-09
64799	H571.00	8	0	0	0	Rheumatic pneumonia	Feb-09
54088	G141100	2	0	0	0	Rheumatic pulmonary insufficiency	Feb-09
62207	G141000	14	0	0	0	Rheumatic pulmonary stenosis	Feb-09
105626	G141200	1	0	0	0	Rheumatic pulmonary stenosis and insufficiency	Jan-13
44167	G141.00	8	0	0	0	Rheumatic pulmonary valve disease	Feb-09
36768	G141z00	2	0	0	0	Rheumatic pulmonary valve disease NOS	Feb-09
62875	N135100	8	0	0	0	Rheumatic torticollis	Feb-09
60266	G140100	24	0	0	0	Rheumatic tricuspid insufficiency	Feb-09
31505	G140000	5	0	0	0	Rheumatic tricuspid stenosis	Feb-09
62186	G14021Y	1	0	0	0	Rheumatic tricuspid stenosis and incompetence	Feb-09
93114	G140200	1	0	0	0	Rheumatic tricuspid stenosis and insufficiency	Feb-09

For peer review only - http://bmjopen.bmj.com/site/about/guidelines.xhtml

93113	G14021X	7	0	0	0	Rheumatic tricuspid stenosis and regurgitation	Feb-09
72613	G140z00	4	0	0	0	Rheumatic tricuspid valve disease NOS	Feb-09
59275	G14z.11	1	0	0	0	"Rheumatic valvulitis, chronic NOS"	Feb-09
67955	A985z11	1	0	0	0	Rheumatism - gonococcal	Feb-09
33474	N240.00	1221	0	1	0	Rheumatism and fibrositis unspecified	Feb-09
35937	N240z00	1287	0	0	0	Rheumatism or fibrositis NOS	Feb-09
844	N040.00	262585	0	21625	2747	Rheumatoid arthritis	Feb-09
31054	N040S00	380	0	22	0	Rheumatoid arthritis - multiple joint	Feb-09
27603	N04..00	1821	0	30	0	Rheumatoid arthritis and other inflammatory polyarthropathy	Feb-09
105507	66HB000	78562	0	249	0	Rheumatoid arthritis annual review	Dec-12
107340	9mM..00	486	0	1	0	Rheumatoid arthritis monitoring invitation	Dec-13
107435	9mM0.00	8104	0	1	0	Rheumatoid arthritis monitoring invitation first letter	Dec-13
107575	9mM1.00	3203	0	1	0	Rheumatoid arthritis monitoring invitation second letter	Jan-14
107676	9mM2.00	1666	0	0	0	Rheumatoid arthritis monitoring invitation third letter	Feb-14
107797	9mM4.00	482	0	0	0	Rheumatoid arthritis monitoring telephone invitation	Mar-14
107606	9mM3.00	103	0	0	0	Rheumatoid arthritis monitoring verbal invitation	Jan-14
51238	N040K00	16	0	0	0	Rheumatoid arthritis of 1st MTP joint	Feb-09
100914	N040400	3	0	0	0	Rheumatoid arthritis of acromioclavicular joint	Nov-10
51239	N040F00	68	0	1	0	Rheumatoid arthritis of ankle	Feb-09

For peer review only - http://bmjopen.bmj.com/site/about/guidelines.xhtml

For peer review only

44743	N040000	21	0	0	0	Rheumatoid arthritis of cervical spine	Feb-09
63198	N040A00	18	0	0	0	Rheumatoid arthritis of DIP joint of finger	Feb-09
63365	N040600	4	0	0	0	Rheumatoid arthritis of distal radio-ulnar joint	Feb-09
59738	N040500	34	0	0	0	Rheumatoid arthritis of elbow	Feb-09
49067	N040B00	34	0	1	0	Rheumatoid arthritis of hip	Feb-09
107112	N040M00	6	0	0	0	Rheumatoid arthritis of IP joint of toe	Oct-13
50863	N040D00	151	0	3	0	Rheumatoid arthritis of knee	Feb-09
99414	N040L00	4	0	0	0	Rheumatoid arthritis of lesser MTP joint	May-10
42299	N040800	75	0	3	0	Rheumatoid arthritis of MCP joint	Feb-09
71784	N040J00	6	0	0	0	Rheumatoid arthritis of other tarsal joint	Feb-09
41941	N040900	47	0	1	0	Rheumatoid arthritis of PIP joint of finger	Feb-09
100776	N040C00	4	0	0	0	Rheumatoid arthritis of sacro-iliac joint	Nov-10
107963	N040300	1	0	0	0	Rheumatoid arthritis of sternoclavicular joint	Apr-14
73619	N040G00	9	0	0	0	Rheumatoid arthritis of subtalar joint	Feb-09
70658	N040H00	7	0	0	0	Rheumatoid arthritis of talonavicular joint	Feb-09
107791	N040E00	1	0	0	0	Rheumatoid arthritis of tibio-fibular joint	Mar-14
48832	N040700	113	0	0	0	Rheumatoid arthritis of wrist	Feb-09
37431	N042z00	39	0	1	0	Rheumatoid arthropathy + visceral/systemic involvement NOS	Feb-09
43816	G5yA.00	26	0	0	0	Rheumatoid carditis	Feb-09

For peer review only - http://bmjopen.bmj.com/site/about/guidelines.xhtml

31724	N04y000	109	0	11	0	Rheumatoid lung	Feb-09
46436	N042100	33	0	1	0	Rheumatoid lung disease	Feb-09
49787	G5y8.00	6	0	0	0	Rheumatoid myocarditis	Feb-09
30548	N040N00	225	0	3	0	Rheumatoid vasculitis	Feb-09
33375	C260.11	13	0	3	2	Riboflavin deficiency	Feb-09
5074	C28..12	2187	0	148	0	Rickets	Feb-09
51768	P342200	41	0	2	0	Rieger's anomaly	Feb-09
57069	G565200	7	0	1	0	Right BBB with left anterior fascicular block	Feb-09
98675	G565100	2	0	0	0	Right BBB with left posterior fascicular block	Mar-10
10079	G580.12	1165	0	40	0	Right heart failure	Feb-09
3293	F223.00	2081	0	124	1	Right hemiplegia	Feb-09
21851	P603.00	52	0	0	0	Right hypoplastic heart syndrome	Feb-09
98751	G5y3600	98	0	1	0	Right ventricular dilatation	Mar-10
104275	G584.00	169	0	1	0	Right ventricular failure	Jun-12
104266	P6y3100	36	0	0	0	Right ventricular outflow tract obstruction	Jun-12
108180	G5yyE00	70	0	0	0	Right ventricular systolic dysfunction	Jun-14
31616	P2x5.00	9	0	0	0	Riley - Day syndrome	Feb-09
22691	G56y200	51	0	0	0	Romano - Ward syndrome	Feb-09
40502	PH33300	46	0	1	0	Rothmund-Thomson syndrome	Feb-09
46507	C374300	65	0	4	0	Rotor syndrome	Feb-09
109176	F143.11	1	0	0	0	Roussy-Levy syndrome	Jan-15
104100	PKy7311	15	0	0	0	Rubinstein-Taybi syndrome	May-12
32868	PKy6200	316	0	15	0	Russell - Silver syndrome	Feb-09
72928	P103400	1	0	0	0	Sacral spina bifida with hydrocephalus - closed	Feb-09
70923	P118400	9	0	0	0	Sacral spina bifida without hydrocephalus - closed	Feb-09

For peer review only - http://bmjopen.bmj.com/site/about/guidelines.xhtml

71525	P117400	3	0	0	0	Sacral spina bifida without hydrocephalus - open	Feb-09
57986	PF55300	91	0	2	0	Saethre-Chotzen syndrome	Feb-09
109634	E121.11	1	0	0	0	Sander's disease	May-15
44111	J34y.11	52	0	2	0	Sandifer's syndrome	Feb-09
111807	F142100	2	0	0	0	Sanger-Brown cerebellar ataxia	May-17
40613	AD55.00	59	0	2	0	Sarcoid arthropathy	Feb-09
47037	G558300	61	0	2	0	Sarcoid heart disease	Feb-09
34437	G5y7.00	13	0	1	0	Sarcoid myocarditis	Feb-09
3865	AD5..00	29294	0	1573	7	Sarcoidosis	Feb-09
72595	AD54.00	32	0	0	0	Sarcoidosis of inferior turbinates	Feb-09
33980	AD50.00	389	0	6	0	Sarcoidosis of lung	Feb-09
58841	AD52.00	43	0	3	0	Sarcoidosis of lung with sarcoidosis of lymph nodes	Feb-09
49075	AD51.00	81	0	2	0	Sarcoidosis of lymph nodes	Feb-09
27769	AD53.00	243	0	12	0	Sarcoidosis of skin	Feb-09
111668	H32yz11	1	0	0	0	Sawyer - Jones syndrome	Feb-17
69375	C375113	5	0	0	0	Scheie's syndrome	Feb-09
1721	N320100	7224	0	306	1	Scheuermann's disease	Feb-09
56438	E107500	211	0	0	0	Schizo-affective schizophrenia in remission	Feb-09
67943	13Y2.00	11	0	0	0	Schizophrenia association member	Feb-09
58687	E100500	1296	0	0	0	Schizophrenia in remission	Feb-09
8407	E10z.00	11518	0	194	0	Schizophrenia NOS	Feb-09
73295	E100.11	4	0	0	0	Schizophrenia simplex	Feb-09
31950	13L3.12	48	0	0	0	Schizophrenic child	Feb-09
854	E10..00	59295	0	3962	7	Schizophrenic disorders	Feb-09
56692	PG45.12	7	0	0	0	Schmid's metaphyseal dysostosis	Feb-09
48130	C181.12	32	0	1	0	Schmidt's syndrome	Feb-09
36606	P74z600	68	0	3	0	Scimitar syndrome	Feb-09
5318	F4K0.12	7606	0	578	0	Scleritis	Feb-09
15996	F4K0.00	3226	0	76	0	Scleritis and episcleritis	Feb-09

For peer review only - http://bmjopen.bmj.com/site/about/guidelines.xhtml

44859	F4K0z00	557	0	2	0	Scleritis or episcleritis NOS	Feb-09
37538	M210500	123	0	4	0	Sclerodactyly	Feb-09
3670	N001.00	6495	0	419	0	Scleroderma	Feb-09
37665	F4K0500	72	0	0	0	Sclerokeratitis	Feb-09
44791	J661900	142	0	1	0	Sclerosing cholangitis unspecified	Feb-09
40041	N374300	1006	0	40	0	Scoliosis associated with other condition	Feb-09
43192	N374D00	7	0	0	0	Scoliosis in connective tissue anomalies	Feb-09
67445	N374C00	19	0	0	0	Scoliosis in neurofibromatosis	Feb-09
64218	N374A00	29	0	1	0	Scoliosis in skeletal dysplasia	Feb-09
53972	N373900	50	0	0	0	Scoliosis secondary to other treatment	Feb-09
34719	PKy6400	10	0	0	0	Seckel syndrome	Feb-09
22487	C10N.00	146	0	5	0	Secondary diabetes mellitus	Feb-09
94383	C10N000	8	0	0	0	Secondary diabetes mellitus without complication	Feb-09
37119	C151100	24	0	1	0	Secondary hyperaldosteronism	Feb-09
70714	C354111	73	0	0	0	Secondary hypercalcaemia	Feb-09
7329	G24..00	2762	0	20	0	Secondary hypertension	Feb-09
42229	G24zz00	162	0	0	0	Secondary hypertension NOS	Feb-09
51697	C10G.00	339	0	4	0	Secondary pancreatic diabetes mellitus	Feb-09
96506	C10G000	2	0	0	0	Secondary pancreatic diabetes mellitus without complication	Apr-09
34065	G41y000	1476	0	10	0	Secondary pulmonary hypertension	Feb-09
65502	D201z14	8	0	0	0	Secondary red cell aplasia NEC	Feb-09
31774	D201z13	7	0	0	0	Secondary red cell hypoplasia NEC	Feb-09
35667	J661800	50	0	2	0	Secondary sclerosing cholangitis	Feb-09
31550	D210300	7	0	0	0	Secondary sideroblastic anaemia due to disease	Feb-09

For peer review only - http://bmjopen.bmj.com/site/about/guidelines.xhtml

47225	D210400	5	0	0	0	Secondary sideroblastic anaemia due to drugs and toxins	Feb-09
97143	F13C.00	13	0	0	0	Segawa syndrome	Aug-09
13304	Q480.12	1116	0	20	0	Seizures in newborn	Feb-09
8548	C390100	796	0	35	3	Selective IgA immunodeficiency	Feb-09
18700	C390300	122	0	3	3	Selective IgG immunodeficiency	Feb-09
18701	C390200	41	0	5	0	Selective IgM immunodeficiency	Feb-09
536	F591.00	65546	0	1344	0	Sensorineural hearing loss	Feb-09
10112	F591600	8679	0	79	0	"Sensorineural hearing loss, bilateral"	Feb-09
29191	F591700	281	0	38	0	"Sensorineurl hear loss,unilat unrestrict hear/contralat side"	Feb-09
3366	663V300	2734	0	66	0	Severe asthma	Feb-09
233	H33z011	25653	0	4132	4	Severe asthma attack	Feb-09
110345	38B8.00	37	0	0	0	Severe asthma exacerbation risk assessment	Dec-15
15082	Q215.00	1394	0	28	5	Severe birth asphyxia - apgar score less than 4 at 1 minute	Feb-09
98734	F286200	12	0	0	0	Severe chronic fatigue syndrome	Mar-10
107402	28E2.00	149	0	1	0	Severe cognitive impairment	Dec-13
108409	F25G.00	5	0	0	0	Severe myoclonic epilepsy in infancy	Jul-14
107610	F591E00	47	0	1	0	Severe sensorineural hearing loss	Jan-14
4376	PJX..00	25	0	0	0	"Sex chromosome abnormality, male phenotype, unspecified"	Feb-09
34667	PJyz.00	65	0	9	2	Sex chromosome anomaly NOS	Feb-09
55119	PJy1.00	17	0	0	0	Sex chromosome mosaicism	Feb-09
65983	PJy1z00	1	0	0	0	Sex chromosome mosaicism NOS	Feb-09
35014	B622.00	77	0	7	0	Sezary's disease	Feb-09
100532	B622z00	3	0	0	0	Sezary's disease NOS	Aug-10
12841	PJ3y000	42	0	0	0	Shprintzen syndrome	Feb-09
62041	PJ53500	21	0	2	0	Shwachman-Diamond syndrome	Feb-09

For peer review only - http://bmjopen.bmj.com/site/about/guidelines.xhtml

35839	F130500	90	0	17	0	Shy-Drager syndrome	Feb-09
2360	N002.00	12690	0	576	1	Sicca (Sjogren's) syndrome	Feb-09
104288	1X0..00	6	0	0	0	Sick Cell Thalas Scr Prog fam orig African or African-Carib	Jun-12
103055	1X1..00	23	0	0	0	Sick Cell Thalas Scr Prog fam orig South Asia (Asian)	Oct-11
107027	K0G..00	5	0	0	0	Sickle cell nephropathy	Sep-13
23519	D106.00	4532	0	489	166	Sickle-cell anaemia	Feb-09
57397	D106z00	75	0	2	0	Sickle-cell anaemia NOS	Feb-09
69964	D106000	14	0	1	0	Sickle-cell anaemia of unspecified type	Feb-09
31370	D106200	1244	0	26	0	Sickle-cell anaemia with crisis	Feb-09
31306	D106300	109	0	8	1	Sickle-cell anaemia with haemoglobin C disease	Feb-09
8119	D106400	55	0	1	1	Sickle-cell anaemia with haemoglobin D disease	Feb-09
93872	D106500	20	0	0	2	Sickle-cell anaemia with haemoglobin E disease	Feb-09
32937	D106100	19	0	1	0	Sickle-cell anaemia with no crisis	Feb-09
31075	D104211	360	0	125	100	Sickle-cell thalassaemia	Feb-09
3616	D105.00	13001	0	1063	741	Sickle-cell trait	Feb-09
99476	PKy5H00	8	0	0	0	Simpson-Golabi-Behmel syndrome	May-10
107846	P240000	1	0	0	0	Single congenital cerebral cyst	Mar-14
62163	P6y4100	26	0	0	0	Single coronary artery	Feb-09
18437	G566000	157	0	7	0	Sinoatrial block	Feb-09
426	G577.00	2593	0	109	1	Sinus arrhythmia	Feb-09
31717	P20..15	22	0	2	0	Sinus pericranii	Feb-09
43560	PK31.00	21	0	1	0	Situs inversus abdominalis	Feb-09
23513	PK3z.00	39	0	8	0	Situs inversus NOS	Feb-09
63861	PK32.00	2	0	0	0	Situs inversus thoracis	Feb-09
110797	PK34.00	1	0	0	0	Situs inversus with levocardia	May-16
72246	PK30.00	6	0	0	0	"Situs inversus, unspecified"	Feb-09

For peer review only - http://bmjopen.bmj.com/site/about/guidelines.xhtml

11632	PH12.11	588	0	19	0	Sjogren - Larsson syndrome	Feb-09
7603	Fy03.00	16408	0	2567	0	Sleep apnoea	Feb-09
23779	H5B..00	2863	0	276	0	Sleep apnoea	Feb-09
60083	PB1..00	9	0	0	0	Small intestine atresia and stenosis	Feb-09
70579	PB10z00	2	0	0	0	Small intestine atresia NOS	Feb-09
25042	PB1z.00	10	0	0	0	Small intestine atresia or stenosis NOS	Feb-09
36871	PJ33300	87	0	4	0	Smith-Magenis syndrome	Feb-09
36314	C1zy211	222	0	13	0	Sotos syndrome	Feb-09
25570	F23y200	221	0	2	0	Spastic cerebral palsy	Feb-09
104775	F230111	49	0	1	0	Spastic diplegic cerebral palsy	Aug-12
59254	ZS42100	32	0	1	0	Spastic dysarthria	Feb-09
105133	F2B1.00	21	0	0	0	Spastic hemiplegic cerebral palsy	Nov-12
9375	F241100	1317	0	48	1	Spastic paraplegia	Feb-09
104580	F2B0.00	101	0	1	0	Spastic quadriplegic cerebral palsy	Jul-12
25990	ZPA2100	992	0	8	0	Special educational needs	Feb-09
22896	ZS...00	1425	0	12	0	Speech and language disorder	Feb-09
43892	ZS5..00	47	0	1	0	Speech and language dyspraxias	Feb-09
28845	ZS6..00	94	0	13	0	Speech and phonology impairments	Feb-09
3417	E2F3.12	6785	0	1109	0	Speech development disorder	Feb-09
110439	1B96.00	40	0	5	0	Speech impairment	Jan-16
3567	E2F3.00	3146	0	44	0	Speech or language developmental disorder	Feb-09
1277	E2F3z00	3822	0	245	1	Speech or language developmental disorder NOS	Feb-09
32923	ZT23.00	548	0	25	0	Speech problem	Feb-09
33403	1B9Z.00	1043	0	8	0	Speech problem NOS	Feb-09
3336	PG17.00	6133	0	142	2	Spina bifida occulta	Feb-09
27436	PD1..14	43	0	1	0	Sponge kidney	Feb-09
4892	H33z000	3720	0	442	0	Status asthmaticus NOS	Feb-09
4093	F253.11	2882	0	577	0	Status epilepticus	Feb-09
22685	F26y200	250	0	1	0	Status migrainosus	Feb-09

For peer review only - http://bmjopen.bmj.com/site/about/guidelines.xhtml

93910	F24y012	1	0	0	0	Steele - Richardson Oszewski syndrome	Feb-09
49034	F24y011	14	0	0	0	Steele Richardson Olszewsk syn	Feb-09
7037	F24y200	94	0	7	0	Steele-Richardson-Olszewski syndrome	Feb-09
16103	C164.12	741	0	51	0	Stein - Leventhal syndrome	Feb-09
50109	PB2..12	5	0	0	0	Stenosis large intestine	Feb-09
16731	P713.11	18	0	0	0	Stenosis of aortic arch	Feb-09
46120	P230.12	31	0	0	0	Stenosis of aqueduct of Sylvius	Feb-09
51675	P74z200	15	0	0	0	Stenosis of inferior vena cava	Feb-09
35852	J50z200	104	0	0	0	Stenosis of intestine NOS	Feb-09
2670	P735.00	785	0	13	0	Stenosis of pulmonary artery	Feb-09
29179	J572.00	135	0	7	0	Stenosis of rectum and anus	Feb-09
53581	J572z00	19	0	2	0	Stenosis of rectum and anus NOS	Feb-09
41197	P74z300	25	0	0	0	Stenosis of superior vena cava	Feb-09
29172	H5y1200	287	0	9	0	Stenosis of trachea	Feb-09
98579	F4J7011	2	0	0	0	Stilling-Turck-Duane syndrome	Feb-10
3769	G56z000	1072	0	156	0	Stokes-Adams syndrome	Feb-09
4749	PK61.00	506	0	20	0	Sturge-Weber syndrome	Feb-09
16539	P6y0.00	411	0	14	0	Subaortic stenosis	Feb-09
73431	J106300	1	0	0	0	Subdiaphragmatic oesophageal diverticulum	Feb-09
105037	P741000	1	0	0	0	Subdiaphragmatic total anomalous pulmonary venous return	Oct-12
36042	C313200	41	0	0	0	Sucrose-isomaltose intolerance	Feb-09
4098	N337100	1745	0	141	0	Sudek's atrophy	Feb-09
111382	D417.12	2	0	0	0	Sulphaemoglobinaemia	Oct-16
36434	J421000	94	0	4	0	Superior mesenteric artery syndrome	Feb-09
8412	G8y2200	219	0	23	0	Superior vena cava syndrome	Feb-09
64216	ZS42113	7	0	0	0	Suprabulbar palsy type of dysarthria	Feb-09

For peer review only - http://bmjopen.bmj.com/site/about/guidelines.xhtml

103039	P741100	1	0	0	0	Supradiaphragmatic total anomalous pulmonary venous return	Oct-11
53964	P722400	43	0	0	0	Supra-valvular aortic stenosis	Feb-09
1536	G57y900	51876	0	4010	0	Supraventricular tachycardia NOS	Feb-09
43838	PF1z.00	265	0	1	0	Syndactyly NOS	Feb-09
41117	PF11.00	33	0	1	0	Syndactyly of fingers without bone fusion	Feb-09
63079	PF10.00	33	0	0	0	"Syndactyly of multiple digits, unspecified"	Feb-09
67014	PF14.00	15	0	1	0	Syndactyly of toes with bone fusion	Feb-09
108734	PF14.13	2	0	0	0	Syndactyly of toes with bone fusion	Oct-14
56836	PF13.00	77	0	3	0	Syndactyly of toes without bone fusion	Feb-09
47358	F160100	23	0	2	0	Syringobulbia	Feb-09
69740	F160.00	17	0	0	0	Syringomyelia and syringobulbia	Feb-09
96785	F160z00	4	0	0	0	Syringomyelia or syringobulbia NOS	Apr-09
43593	P11y.11	21	0	2	0	Syringomyelocele	Feb-09
7871	N000.00	19120	0	1358	47	Systemic lupus erythematosus	Feb-09
45726	ZRq9.00	11	0	1	0	Systemic lupus erythematosus disease activity index	Feb-09
42719	N000z00	347	0	4	0	Systemic lupus erythematosus NOS	Feb-09
29519	N000300	123	0	11	2	Systemic lupus erythematosus with organ or sys involv	Feb-09
28417	N001.12	1671	0	49	0	Systemic sclerosis	Feb-09
110174	N001200	1	0	0	0	Systemic sclerosis induced by drugs and chemicals	Oct-15
37640	G757.00	234	0	4	0	Takayasu's disease	Feb-09
68533	D313211	21	0	1	0	TAR syndrome	Feb-09
40025	P511300	12	0	0	0	Taussig-Bing syndrome	Feb-09
21169	F101300	266	0	135	130	Tay-Sach's disease	Feb-09
3175	F254000	13930	0	664	2	Temporal lobe epilepsy	Feb-09

For peer review only - http://bmjopen.bmj.com/site/about/guidelines.xhtml

36325	B470300	78	0	1	0	Teratoma of undescended testis	Feb-09
15118	B7B..11	312	0	24	0	"Teratoma, benign"	Feb-09
48765	PCy4.12	23	0	0	0	"Testicular agenesis, bilateral"	Feb-09
38998	PCy5.12	59	0	1	0	"Testicular agenesis, unilateral"	Feb-09
63528	C172000	14	0	1	0	Testicular hypofunc- defect adrenocortical hormone synthesis	Feb-09
9577	C172.12	6404	0	377	0	Testicular hypogonadism	Feb-09
4864	P52..00	2444	0	79	1	Tetralogy of Fallot	Feb-09
63046	P52z.00	42	0	0	0	Tetralogy of Fallot NOS	Feb-09
38967	P520.00	34	0	2	0	"Tetralogy of Fallot, unspecified"	Feb-09
1171	D104.00	6076	0	275	84	Thalassaemia	Feb-09
1174	D104100	2813	0	184	105	Thalassaemia minor NEC	Feb-09
4666	D104z00	191	0	0	0	Thalassaemia NOS	Feb-09
54429	D104200	42	0	4	3	Thalassaemia with haemoglobin S disease	Feb-09
56900	PG44200	5	0	0	0	Thanatophoric dwarfism	Feb-09
67262	PG44211	2	0	0	0	Thanatophoric dysplasia	Feb-09
23484	F392111	51	0	6	0	Thomsen's disease	Feb-09
57510	PH33312	7	0	0	0	Thomson's disease	Feb-09
62286	SJ21.00	7	0	0	0	Thoracic cord injury without spinal bone injury	Feb-09
102775	SJ21z00	1	0	0	0	"Thoracic cord injury without spinal bone injury, NOS"	Aug-11
51550	N129200	47	0	2	0	Thoracic disc disorder with myelopathy	Feb-09
23390	N121.00	575	0	15	0	Thoracic disc displacement without myelopathy	Feb-09
51691	N12B100	39	0	0	0	Thoracic disc prolapse with myelopathy	Feb-09
40333	N12C100	65	0	2	0	Thoracic disc prolapse with radiculopathy	Feb-09
103025	C14z.00	3	0	1	0	Thymus disease NOS	Sep-11

For peer review only - http://bmjopen.bmj.com/site/about/guidelines.xhtml

69113	C06y100	6	0	0	0	Thyroid atrophy	Feb-09
14704	C04..12	76375	0	3117	110	Thyroid deficiency	Feb-09
24681	66B8.00	3932	0	5	0	Thyroid dis.treatment changed	Feb-09
38292	66B9.00	986	0	6	0	Thyroid dis.treatment started	Feb-09
35957	C06z.00	1079	0	29	0	Thyroid disorder NOS	Feb-09
42603	AC22.00	2	0	0	0	Thyroid echinococcus granulosus	Feb-09
28530	66B4.00	798	0	9	0	Thyroid eye disease	Feb-09
23014	C04z.12	98	0	0	0	Thyroid insufficiency	Feb-09
1346	C05..00	8097	0	649	11	Thyroiditis	Feb-09
677	C02..00	100352	0	9527	54	Thyrotoxicosis	Feb-09
64856	C02y200	5	0	0	0	Thyrotoxicosis factitia	Feb-09
49508	C024.00	45	0	4	0	Thyrotoxicosis from ectopic thyroid nodule	Feb-09
56270	C024z00	4	0	1	0	Thyrotoxicosis from ectopic thyroid nodule NOS	Feb-09
64656	C024000	1	0	0	0	Thyrotoxicosis from ectopic thyroid nodule with no crisis	Feb-09
26699	C02zz00	2292	0	46	0	Thyrotoxicosis NOS	Feb-09
43136	C02y.00	49	0	0	0	Thyrotoxicosis of other specified origin	Feb-09
34220	C02yz00	38	0	0	0	Thyrotoxicosis of other specified origin NOS	Feb-09
106532	C02y100	2	0	0	0	Thyrotoxicosis of other specified origin with crisis	Aug-13
51273	C02y000	17	0	0	0	Thyrotoxicosis of other specified origin with no crisis	Feb-09
26701	C02z000	68	0	2	0	Thyrotoxicosis without mention of goitre or cause no crisis	Feb-09
15565	C02z.00	1188	0	76	0	Thyrotoxicosis without mention of goitre or other cause	Feb-09
3194	C02z100	253	0	21	0	"Thyrotoxicosis without mention of goitre, cause with crisis"	Feb-09

For peer review only - http://bmjopen.bmj.com/site/about/guidelines.xhtml

18688	F24y100	453	0	8	0	Todd's paralysis	Feb-09
8187	F251500	3156	0	85	0	Tonic-clonic epilepsy	Feb-09
22804	F251011	410	0	7	0	Tonic-clonic epilepsy	Feb-09
68975	PF27100	1	0	0	0	Total absence of ulna	Feb-09
51941	P741.00	61	0	0	0	Total anomalous pulmonary venous return - TAPVR	Feb-09
68784	P741z00	16	0	0	0	Total anomalous pulmonary venous return NOS	Feb-09
95444	790M000	20	0	0	0	Total cavopulmonary con extrac inf cav vein pulmon art con	Feb-09
91455	790M100	16	0	0	0	Total cavopulmonary connection with lateral atrial tunnel	Feb-09
39081	P334000	6	0	0	0	Total congenital cataract	Feb-09
49621	F475100	10	0	0	0	Total internal ophthalmoplegia	Feb-09
66061	PF26200	2	0	0	0	Total radial absence	Feb-09
23315	C020.00	853	0	49	0	Toxic diffuse goitre	Feb-09
49334	C020z00	105	0	1	0	Toxic diffuse goitre NOS	Feb-09
57011	C020100	13	0	0	0	Toxic diffuse goitre with crisis	Feb-09
26702	C020000	69	0	1	0	Toxic diffuse goitre with no crisis	Feb-09
55416	G52y700	8	0	1	0	Toxic myocarditis	Feb-09
63323	F382.00	4	0	0	0	Toxic myoneural disorder	Feb-09
32916	F394.00	19	0	0	0	Toxic myopathy	Feb-09
50893	K0C4.00	11	0	0	0	"Toxic nephropathy, not elsewhere classified"	Feb-09
45081	F37..11	38	0	3	0	Toxic neuropathy	Feb-09
49361	C023z00	57	0	2	0	Toxic nodular goitre NOS	Feb-09
100004	C023100	1	0	0	0	Toxic nodular goitre unspecified with crisis	Jun-10
68512	C023000	6	0	0	0	Toxic nodular goitre unspecified with no crisis	Feb-09
22373	F4H3400	359	0	25	0	Toxic optic neuropathy	Feb-09
53280	C021.00	78	0	3	0	Toxic uninodular goitre	Feb-09

For peer review only - http://bmjopen.bmj.com/site/about/guidelines.xhtml

For peer review only

61498	C021z00	14	0	2	0	Toxic uninodular goitre NOS	Feb-09
26869	C021000	10	0	1	0	Toxic uninodular goitre with no crisis	Feb-09
47068	C294.00	56	0	1	0	Trace element deficiency	Feb-09
68903	Q313400	3	0	0	0	Tracheobronchial haemorrhage origin in the perinatal period	Feb-09
4048	J10y200	941	0	32	0	Tracheo-oesophageal fistula	Feb-09
51167	H5y0400	76	0	2	0	Tracheo-oesophageal fistula following tracheostomy	Feb-09
62637	D011200	27	0	0	0	Transcobalamin II deficiency	Feb-09
93178	ZS78411	2	0	0	0	Transcortical motor aphasia	Feb-09
105929	ZS78611	1	0	1	0	Transcortical sensory aphasia	Apr-13
101822	ZS78600	1	0	1	0	Transcortical sensory dysphasia	Mar-11
65785	PK31.11	3	0	0	0	Transposition of abdominal viscera	Feb-09
59279	PB53100	9	0	0	0	Transposition of appendix	Feb-09
66200	PB53200	2	0	0	0	Transposition of caecum	Feb-09
45857	PB53300	15	0	1	0	Transposition of colon	Feb-09
48206	7927300	78	0	0	0	Transposition of coronary artery NEC	Feb-09
39857	7H40600	18	0	0	0	Transposition of iliopsoas muscle	Feb-09
67877	PB53.00	5	0	0	0	Transposition of intestine	Feb-09
71180	PB53000	1	0	0	0	"Transposition of intestine, unspecified"	Feb-09
71874	P74z700	3	0	1	0	Transposition of pulmonary veins	Feb-09
66199	PA77.00	9	0	0	0	Transposition of stomach	Feb-09
47596	PK30.11	12	0	0	0	Transposition of viscera unspecified	Feb-09
6494	F037.00	2471	0	125	0	Transverse myelitis	Feb-09
42239	G140112	19	0	0	0	Tricuspid incompetence - rheumatic	Feb-09
34869	G140412	107	0	3	0	"Tricuspid incompetence, cause unspecified"	Feb-09
1779	G542000	783	0	15	0	"Tricuspid incompetence, non-rheumatic"	Feb-09

For peer review only - http://bmjopen.bmj.com/site/about/guidelines.xhtml

42128	G140400	318	0	7	0	"Tricuspid insufficiency, cause unspecified"	Feb-09
97738	G542011	3	0	0	0	"Tricuspid insufficiency, non-rheumatic"	Oct-09
21980	G140111	231	0	8	0	Tricuspid regurgitation - rheumatic	Feb-09
9286	G140413	6093	0	42	0	"Tricuspid regurgitation, cause unspecified"	Feb-09
35372	G542012	1335	0	5	0	"Tricuspid regurgitation, non-rheumatic"	Feb-09
72306	G140500	3	0	0	0	"Tricuspid stenosis and insufficiency, cause unspecified"	Feb-09
49551	G140514	34	0	0	0	"Tricuspid stenosis and regurgitation, cause unspecified"	Feb-09
56029	G140300	26	0	0	0	"Tricuspid stenosis, cause unspecified"	Feb-09
35724	G542100	26	0	3	0	"Tricuspid stenosis, non-rheumatic"	Feb-09
16373	G140.00	457	0	28	0	Tricuspid valve disease NEC	Feb-09
43855	G542z00	108	0	0	0	Tricuspid valve disorders NOS	Feb-09
2817	G542.00	297	0	11	0	"Tricuspid valve disorders, non-rheumatic"	Feb-09
103577	F262E00	92	0	0	0	Trigeminal autonomic cephalalgia	Jan-12
38476	PG0B.00	127	0	3	0	Trigonocephaly	Feb-09
107520	PG04.12	1	0	0	0	Trigorhinophalangeal dysplasia	Dec-13
110299	PB6yw13	1	0	0	0	Trilobular liver	Dec-15
92988	PD3C.00	2	0	0	0	Triple kidney with triple pelvis	Feb-09
6377	PJy2.11	73	0	2	0	Triple X female	Feb-09
34913	PJ52300	35	0	0	0	Triploidy	Feb-09
54377	PJ52.00	12	0	0	0	Trisomies of autosomes NEC	Feb-09
70198	PJ50600	7	0	0	0	Trisomy 12	Feb-09
18415	PJ0..12	366	0	12	2	Trisomy 21	Feb-09
61627	PJ0z.11	30	0	0	0	Trisomy 21 NOS	Feb-09

For peer review only - http://bmjopen.bmj.com/site/about/guidelines.xhtml

42701	PJ00.00	10	0	0	0	"Trisomy 21, meiotic nondisjunction"	Feb-09
107919	PJ01.11	1	0	0	0	"Trisomy 21, mitotic nondisjunction"	Apr-14
32010	PJ01.00	29	0	0	0	"Trisomy 21, mosaicism"	Feb-09
61499	PJ02.00	11	0	2	0	"Trisomy 21, translocation"	Feb-09
100024	PJ50800	2	0	0	0	Trisomy 22	Jun-10
37591	PJ50100	3	0	0	0	Trisomy 7	Feb-09
107119	PJ52z00	1	0	0	0	Trisomy of autosomes NEC NOS	Oct-13
46264	PC70.00	43	0	3	0	True hermaphroditism	Feb-09
41371	P502.11	73	0	0	0	Truncus arteriosus	Feb-09
45187	P500.12	28	0	11	0	Truncus arteriosus	Feb-09
62310	C37y300	11	0	3	0	Trypsinogen deficiency	Feb-09
11146	C134300	625	0	41	4	TSH - thyroid-stimulating hormone deficiency	Feb-09
30721	PJ63100	26	0	1	1	"Turner's phenotype, karyotype 45X"	Feb-09
92599	PJ63200	10	0	0	0	"Turner's phenotype, karyotype 46X iso (Xq)"	Feb-09
98244	PJ63000	2	0	0	0	"Turner's phenotype, karyotype normal"	Dec-09
51868	PJ63400	38	0	1	0	"Turner's phenotype, mosaicism 45X/46XX or 45X/46XY"	Feb-09
65206	PJ63600	4	0	0	0	"Turner's phenotype, other variant karyotypes"	Feb-09
64945	PJ63612	3	0	0	0	"Turner's phenotype, partial X deletion karyotype"	Feb-09
97871	PJ63611	1	0	0	0	"Turner's phenotype, ring chromosome karyotype"	Oct-09
4943	PJ63.00	2501	0	161	0	Turner's syndrome	Feb-09
53168	PJ63z00	49	0	2	0	Turner's syndrome NOS	Feb-09

For peer review only - http://bmjopen.bmj.com/site/about/guidelines.xhtml

109385	PJ63300	4	0	0	0	"Turner's,karyotype 46X + abnorm. sex chromosome,not iso(Xq)"	Mar-15
40570	PJ63500	76	0	0	0	"Turner's,mosaic, 45X/other cell line with abn.sex chromosome"	Feb-09
1549	C10E.00	92258	0	2218	2	Type 1 diabetes mellitus	Feb-09
17858	C108.12	6354	0	175	0	Type 1 diabetes mellitus	Feb-09
35288	C10E800	340	0	30	0	Type 1 diabetes mellitus - poor control	Feb-09
45914	C108812	9	0	0	0	Type 1 diabetes mellitus - poor control	Feb-09
109628	C10P011	1	0	0	0	Type 1 diabetes mellitus in remission	May-15
40682	C10E900	82	0	1	0	Type 1 diabetes mellitus maturity onset	Feb-09
97446	C108912	2	0	0	0	Type 1 diabetes mellitus maturity onset	Aug-09
18642	C10EH00	11	0	0	0	Type 1 diabetes mellitus with arthropathy	Feb-09
49554	C10EF00	20	0	0	0	Type 1 diabetes mellitus with diabetic cataract	Feb-09
110400	C108F12	1	0	0	0	Type 1 diabetes mellitus with diabetic cataract	Dec-15
22871	C10EP00	63	0	4	0	Type 1 diabetes mellitus with exudative maculopathy	Feb-09
69993	C10E600	12	0	0	0	Type 1 diabetes mellitus with gangrene	Feb-09
55239	C10EQ00	304	0	5	0	Type 1 diabetes mellitus with gastroparesis	Feb-09
39070	C10EE00	85	0	1	0	Type 1 diabetes mellitus with hypoglycaemic coma	Feb-09
70766	C108E12	2	0	0	0	Type 1 diabetes mellitus with hypoglycaemic coma	Feb-09

For peer review only - http://bmjopen.bmj.com/site/about/guidelines.xhtml

10692	C10EM00	5880	0	106	0	Type 1 diabetes mellitus with ketoacidosis	Feb-09
40837	C10EN00	242	0	26	0	Type 1 diabetes mellitus with ketoacidotic coma	Feb-09
68105	C10EB00	11	0	0	0	Type 1 diabetes mellitus with mononeuropathy	Feb-09
47650	C10E300	37	0	0	0	Type 1 diabetes mellitus with multiple complications	Feb-09
10418	C10ED00	487	0	9	0	Type 1 diabetes mellitus with nephropathy	Feb-09
42831	C10E200	24	0	1	0	Type 1 diabetes mellitus with neurological complications	Feb-09
61829	C108212	6	0	0	0	Type 1 diabetes mellitus with neurological complications	Feb-09
18230	C108J12	3	0	1	0	Type 1 diabetes mellitus with neuropathic arthropathy	Feb-09
54008	C10EJ00	61	0	0	0	Type 1 diabetes mellitus with neuropathic arthropathy	Feb-09
47649	C10E100	21	0	2	0	Type 1 diabetes mellitus with ophthalmic complications	Feb-09
102740	C108112	1	0	0	0	Type 1 diabetes mellitus with ophthalmic complications	Aug-11
93468	C10EG00	5	0	0	0	Type 1 diabetes mellitus with peripheral angiopathy	Feb-09
30294	C10EL00	464	0	5	0	Type 1 diabetes mellitus with persistent microalbuminuria	Feb-09
30323	C10EK00	521	0	5	0	Type 1 diabetes mellitus with persistent proteinuria	Feb-09
46301	C10EC00	34	0	0	0	Type 1 diabetes mellitus with polyneuropathy	Feb-09
21983	C108012	5	0	0	0	Type 1 diabetes mellitus with renal complications	Feb-09

For peer review only

47582	C10E000	50	0	2	0	Type 1 diabetes mellitus with renal complications	Feb-09
18387	C10E700	536	0	12	0	Type 1 diabetes mellitus with retinopathy	Feb-09
41049	C108712	9	0	0	0	Type 1 diabetes mellitus with retinopathy	Feb-09
68390	C108512	15	0	0	0	Type 1 diabetes mellitus with ulcer	Feb-09
69676	C10EA00	53	0	1	0	Type 1 diabetes mellitus without complication	Feb-09
111106	C108A12	1	0	0	0	Type 1 diabetes mellitus without complication	Jul-16
104453	66At011	114	0	0	0	Type 1 diabetic dietary review	Jun-12
24599	Z271G11	150	0	3	0	Type 1 dip	Feb-09
758	C10F.00	906638	0	12665	21	Type 2 diabetes mellitus	Feb-09
17859	C109.12	46888	0	1121	2	Type 2 diabetes mellitus	Feb-09
25627	C10F700	1307	0	57	0	Type 2 diabetes mellitus - poor control	Feb-09
45913	C109712	45	0	1	0	Type 2 diabetes mellitus - poor control	Feb-09
110611	C10P111	12	0	0	0	Type 2 diabetes mellitus in remission	Mar-16
49869	C109G12	1	0	0	0	Type 2 diabetes mellitus with arthropathy	Feb-09
59253	C10FG00	50	0	2	0	Type 2 diabetes mellitus with arthropathy	Feb-09
44779	C109E12	9	0	1	0	Type 2 diabetes mellitus with diabetic cataract	Feb-09
44982	C10FE00	154	0	5	0	Type 2 diabetes mellitus with diabetic cataract	Feb-09
25591	C10FQ00	189	0	5	0	Type 2 diabetes mellitus with exudative maculopathy	Feb-09

For peer review only - http://bmjopen.bmj.com/site/about/guidelines.xhtml

For peer review only

12736	C10F500	54	0	4	0	Type 2 diabetes mellitus with gangrene	Feb-09
46150	C109512	7	0	0	0	Type 2 diabetes mellitus with gangrene	Feb-09
63690	C10FR00	177	0	5	0	Type 2 diabetes mellitus with gastroparesis	Feb-09
46917	C10FD00	106	0	2	0	Type 2 diabetes mellitus with hypoglycaemic coma	Feb-09
61071	C109D12	7	0	1	0	Type 2 diabetes mellitus with hypoglycaemic coma	Feb-09
32627	C10FN00	855	0	50	0	Type 2 diabetes mellitus with ketoacidosis	Feb-09
51756	C10FP00	30	0	0	0	Type 2 diabetes mellitus with ketoacidotic coma	Feb-09
62674	C10FA00	71	0	0	0	Type 2 diabetes mellitus with mononeuropathy	Feb-09
65267	C10F300	37	0	2	0	Type 2 diabetes mellitus with multiple complications	Feb-09
108005	C109312	56	0	0	0	Type 2 diabetes mellitus with multiple complications	May-14
12640	C10FC00	2067	0	64	0	Type 2 diabetes mellitus with nephropathy	Feb-09
24836	C109C12	10	0	1	0	Type 2 diabetes mellitus with nephropathy	Feb-09
34268	C10F200	176	0	3	0	Type 2 diabetes mellitus with neurological complications	Feb-09
45919	C109212	16	0	0	0	Type 2 diabetes mellitus with neurological complications	Feb-09
35385	C10FH00	214	0	7	0	Type 2 diabetes mellitus with neuropathic arthropathy	Feb-09
66965	C109H12	9	0	0	0	Type 2 diabetes mellitus with neuropathic arthropathy	Feb-09

For peer review only - http://bmjopen.bmj.com/site/about/guidelines.xhtml

47321	C10F100	100	0	1	0	Type 2 diabetes mellitus with ophthalmic complications	Feb-09
70316	C109112	39	0	0	0	Type 2 diabetes mellitus with ophthalmic complications	Feb-09
37806	C10FF00	83	0	2	0	Type 2 diabetes mellitus with peripheral angiopathy	Feb-09
60699	C109F12	4	0	0	0	Type 2 diabetes mellitus with peripheral angiopathy	Feb-09
18390	C10FM00	8573	0	54	0	Type 2 diabetes mellitus with persistent microalbuminuria	Feb-09
26054	C10FL00	4613	0	48	3	Type 2 diabetes mellitus with persistent proteinuria	Feb-09
18425	C10FB00	255	0	4	0	Type 2 diabetes mellitus with polyneuropathy	Feb-09
109865	C109B12	3	0	0	0	Type 2 diabetes mellitus with polyneuropathy	Jul-15
18777	C10F000	231	0	18	0	Type 2 diabetes mellitus with renal complications	Feb-09
18496	C10F600	1868	0	42	2	Type 2 diabetes mellitus with retinopathy	Feb-09
42762	C109612	23	0	1	0	Type 2 diabetes mellitus with retinopathy	Feb-09
49074	C10F400	116	0	4	0	Type 2 diabetes mellitus with ulcer	Feb-09
65704	C109412	14	0	2	0	Type 2 diabetes mellitus with ulcer	Feb-09
47954	C10F900	871	0	6	0	Type 2 diabetes mellitus without complication	Feb-09
105784	C109912	3	0	0	0	Type 2 diabetes mellitus without complication	Feb-13
102611	66At111	243	0	0	0	Type 2 diabetic dietary review	Jul-11
44403	Z271H11	26	0	0	0	Type 2 dip	Feb-09
37957	C10K.00	89	0	2	0	Type A insulin resistance	Feb-09

For peer review only - http://bmjopen.bmj.com/site/about/guidelines.xhtml

56885	C10K000	1	0	0	0	Type A insulin resistance without complication	Feb-09
24423	C108.13	793	0	17	0	Type I diabetes mellitus	Feb-09
46850	C108811	18	0	0	0	Type I diabetes mellitus - poor control	Feb-09
105337	C10E811	8	0	0	0	Type I diabetes mellitus - poor control	Dec-12
108360	C10P000	2	0	0	0	Type I diabetes mellitus in remission	Jul-14
63017	C108911	1	0	0	0	Type I diabetes mellitus maturity onset	Feb-09
96235	C10E911	12	0	0	0	Type I diabetes mellitus maturity onset	Apr-09
62352	C108H11	1	0	0	0	Type I diabetes mellitus with arthropathy	Feb-09
17545	C108F11	5	0	1	0	Type I diabetes mellitus with diabetic cataract	Feb-09
97894	C10EP11	1	0	0	0	Type I diabetes mellitus with exudative maculopathy	Oct-09
102112	C10E611	6	0	0	0	Type I diabetes mellitus with gangrene	Apr-11
108724	C10EQ11	1	0	0	0	Type I diabetes mellitus with gastroparesis	Oct-14
42729	C108E11	14	0	0	0	Type I diabetes mellitus with hypoglycaemic coma	Feb-09
62209	C10EM11	46	0	0	0	Type I diabetes mellitus with ketoacidosis	Feb-09
66145	C10EN11	1	0	0	0	Type I diabetes mellitus with ketoacidotic coma	Feb-09
99231	C108B11	1	0	0	0	Type I diabetes mellitus with mononeuropathy	Apr-10
91942	C10E311	5	0	0	0	Type I diabetes mellitus with multiple complications	Feb-09

For peer review only - http://bmjopen.bmj.com/site/about/guidelines.xhtml

108007	C108311	1	0	0	0	Type I diabetes mellitus with multiple complications	May-14
66872	C108D11	7	0	0	0	Type I diabetes mellitus with nephropathy	Feb-09
49146	C108211	1	0	0	0	Type I diabetes mellitus with neurological complications	Feb-09
60208	C108J11	3	0	0	0	Type I diabetes mellitus with neuropathic arthropathy	Feb-09
99311	C10E111	1	0	0	0	Type I diabetes mellitus with ophthalmic complications	May-10
102620	C10EL11	1	0	0	0	Type I diabetes mellitus with persistent microalbuminuria	Jul-11
91943	C10EC11	1	0	0	0	Type I diabetes mellitus with polyneuropathy	Feb-09
61344	C108011	6	0	0	0	Type I diabetes mellitus with renal complications	Feb-09
109837	C10E011	1	0	0	0	Type I diabetes mellitus with renal complications	Jul-15
38161	C108711	14	0	1	0	Type I diabetes mellitus with retinopathy	Feb-09
95343	C10E711	15	0	0	0	Type I diabetes mellitus with retinopathy	Feb-09
51957	C108511	10	0	1	0	Type I diabetes mellitus with ulcer	Feb-09
93878	C10E511	6	0	0	0	Type I diabetes mellitus with ulcer	Feb-09
62613	C10EA11	4	0	0	0	Type I diabetes mellitus without complication	Feb-09
95992	C108A11	1	0	0	0	Type I diabetes mellitus without complication	Feb-09
102704	66At000	7865	0	8	0	Type I diabetic dietary review	Aug-11
18219	C109.13	4924	0	125	0	Type II diabetes mellitus	Feb-09
22884	C10F.11	5124	0	13	0	Type II diabetes mellitus	Feb-09

For peer review only - http://bmjopen.bmj.com/site/about/guidelines.xhtml

24458	C109711	49	0	2	0	Type II diabetes mellitus - poor control	Feb-09
47315	C10F711	88	0	0	0	Type II diabetes mellitus - poor control	Feb-09
107824	C10P100	27	0	0	0	Type II diabetes mellitus in remission	Mar-14
18143	C109G11	1	0	0	0	Type II diabetes mellitus with arthropathy	Feb-09
103902	C10FG11	17	0	0	0	Type II diabetes mellitus with arthropathy	Mar-12
48192	C109E11	14	0	4	0	Type II diabetes mellitus with diabetic cataract	Feb-09
93727	C10FE11	52	0	1	0	Type II diabetes mellitus with diabetic cataract	Feb-09
111798	C10FQ11	1	0	0	0	Type II diabetes mellitus with exudative maculopathy	May-17
62107	C109511	7	0	1	0	Type II diabetes mellitus with gangrene	Feb-09
104323	C10F511	3	0	0	0	Type II diabetes mellitus with gangrene	Jun-12
56268	C109D11	2	0	0	0	Type II diabetes mellitus with hypoglycaemic coma	Feb-09
98723	C10FD11	22	0	0	0	Type II diabetes mellitus with hypoglycaemic coma	Mar-10
106528	C10FN11	2	0	0	0	Type II diabetes mellitus with ketoacidosis	Aug-13
106061	C10FP11	1	0	0	0	Type II diabetes mellitus with ketoacidotic coma	May-13
50813	C109A11	1	0	0	0	Type II diabetes mellitus with mononeuropathy	Feb-09
95351	C10FA11	11	0	0	0	Type II diabetes mellitus with mononeuropathy	Feb-09

For peer review only - http://bmjopen.bmj.com/site/about/guidelines.xhtml

43227	C10F311	9	0	0	0	Type II diabetes mellitus with multiple complications	Feb-09
64571	C109C11	5	0	1	0	Type II diabetes mellitus with nephropathy	Feb-09
102201	C10FC11	36	0	1	0	Type II diabetes mellitus with nephropathy	May-11
67905	C109211	5	0	0	0	Type II diabetes mellitus with neurological complications	Feb-09
98616	C10F211	2	0	0	0	Type II diabetes mellitus with neurological complications	Mar-10
47816	C109H11	7	0	3	0	Type II diabetes mellitus with neuropathic arthropathy	Feb-09
109197	C10FH11	4	0	0	0	Type II diabetes mellitus with neuropathic arthropathy	Jan-15
59725	C109111	2	0	0	0	Type II diabetes mellitus with ophthalmic complications	Feb-09
100964	C10F111	5	0	0	0	Type II diabetes mellitus with ophthalmic complications	Nov-10
54899	C109F11	4	0	0	0	Type II diabetes mellitus with peripheral angiopathy	Feb-09
104639	C10FF11	24	0	0	0	Type II diabetes mellitus with peripheral angiopathy	Aug-12
60796	C10FL11	60	0	0	0	Type II diabetes mellitus with persistent proteinuria	Feb-09
47409	C109B11	4	0	0	0	Type II diabetes mellitus with polyneuropathy	Feb-09
50527	C10FB11	17	0	0	0	Type II diabetes mellitus with polyneuropathy	Feb-09
50225	C109011	9	0	1	0	Type II diabetes mellitus with renal complications	Feb-09
57278	C10F011	5	0	0	0	Type II diabetes mellitus with renal complications	Feb-09

For peer review only - http://bmjopen.bmj.com/site/about/guidelines.xhtml

49655	C10F611	37	0	0	0	Type II diabetes mellitus with retinopathy	Feb-09
58604	C109611	21	0	5	0	Type II diabetes mellitus with retinopathy	Feb-09
55075	C109411	19	0	1	0	Type II diabetes mellitus with ulcer	Feb-09
91646	C10F411	5	0	0	0	Type II diabetes mellitus with ulcer	Feb-09
53392	C10F911	256	0	0	0	Type II diabetes mellitus without complication	Feb-09
109103	C109911	0	0	0	0	Type II diabetes mellitus without complication	Jan-15
101801	66At100	165941	0	35	0	Type II diabetic dietary review	Feb-11
111509	K08yB11	1	0	0	0	Type IV renal tubular acidosis	Dec-16
31001	C302611	47	0	0	0	Tyrosinaemia	Feb-09
55159	46F7.11	0	0	0	2	Tyrosine cryst.- urine	Feb-09
45295	C302300	5	0	0	0	Tyrosinosis	Feb-09
63807	C302400	11	0	0	0	Tyrosinuria	Feb-09
30433	J411.00	221	0	8	0	Ulcerative (chronic) enterocolitis	Feb-09
42822	J412.00	35	0	2	0	Ulcerative (chronic) ileocolitis	Feb-09
704	J410100	92252	0	4661	46	Ulcerative colitis	Feb-09
1784	J41..12	32854	0	1756	30	Ulcerative colitis and/or proctitis	Feb-09
48732	J410000	39	0	3	0	Ulcerative ileocolitis	Feb-09
104428	J08zF11	172	0	2	0	Ulcerative oral mucositis	Jun-12
104259	J413.00	408	0	4	0	Ulcerative pancolitis	Jun-12
8347	J410300	3226	0	103	0	Ulcerative proctitis	Feb-09
6650	J410.00	8183	0	464	4	Ulcerative proctocolitis	Feb-09
33456	J410z00	135	0	1	0	Ulcerative proctocolitis NOS	Feb-09
24858	J410200	197	0	1	0	Ulcerative rectosigmoiditis	Feb-09
105762	B62C.00	7	0	0	0	Unifocal Langerhans-cell histiocytosis	Feb-13
63930	P911.00	21	0	0	0	Unilateral complete cleft lip	Feb-09
73531	P901.00	3	0	0	0	Unilateral complete cleft palate	Feb-09

For peer review only - http://bmjopen.bmj.com/site/about/guidelines.xhtml

62987	P921.00	16	0	0	0	Unilateral complete cleft palate with cleft lip	Feb-09
102267	J06y700	4	0	0	0	Unilateral condylar mandibular hyperplasia	May-11
103529	J06y800	1	0	0	0	Unilateral condylar mandibular hypoplasia	Jan-12
23958	PD02100	362	0	4	0	Unilateral congenital absence of kidney	Feb-09
45970	PE30000	266	0	3	0	Unilateral congenital dislocation of hip	Feb-09
34019	PE31000	78	0	0	0	Unilateral congenital subluxation of hip	Feb-09
38772	PE34000	135	0	0	0	Unilateral dysplastic hip	Feb-09
68946	F255500	4	0	0	0	Unilateral epilepsy	Feb-09
54012	P912.00	54	0	0	0	Unilateral incomplete cleft lip	Feb-09
73558	P902.00	8	0	0	0	Unilateral incomplete cleft palate	Feb-09
54144	P922.00	5	0	0	0	Unilateral incomplete cleft palate with cleft lip	Feb-09
31961	PD00100	80	0	0	0	Unilateral renal agenesis	Feb-09
54656	PD04111	3	0	0	0	Unilateral renal dysgenesis	Feb-09
24120	PD04100	63	0	2	0	Unilateral renal dysplasia	Feb-09
30650	PD03100	46	0	1	0	Unilateral renal hypoplasia	Feb-09
43919	K090.00	309	0	3	0	Unilateral small kidney	Feb-09
105369	K090100	3	0	1	0	Unilateral small kidney with contralateral hypertrophy	Dec-12
31097	K16X.00	188	0	1	0	"Uninhibited neuropathic bladder, NEC"	Feb-09
62520	K03W.00	8	0	0	0	"Unsp nephrit synd, diff endocap prolif glomerulonephritis"	Feb-09
30301	K03X.00	38	0	1	0	"Unsp nephrit synd, diff mesang prolif glomerulonephritis"	Feb-09

For peer review only - http://bmjopen.bmj.com/site/about/guidelines.xhtml

73574	P2y..00	6	0	0	0	Unspec nervous system anomaly of brain/cord/nervous system	Feb-09
36125	K03U.00	35	0	1	0	"Unspecif nephr synd, diff concentric glomerulonephritis"	Feb-09
25280	PAz2.00	93	0	8	0	Unspecified anomalies of stomach	Feb-09
44752	PDz2.00	103	0	6	0	Unspecified anomaly of bladder	Feb-09
22492	PGz2.00	84	0	7	0	Unspecified anomaly of bones	Feb-09
28247	PGz4.00	53	0	2	0	Unspecified anomaly of connective tissue	Feb-09
26626	P6z0.00	89	0	6	0	Unspecified anomaly of heart valve	Feb-09
46880	PDz3.00	75	0	10	0	Unspecified anomaly of urethra	Feb-09
70721	E117500	6	0	0	0	"Unspecified bipolar affect disord, partial/unspec remission"	Feb-09
24230	E117600	282	0	0	0	"Unspecified bipolar affective disorder, in full remission"	Feb-09
63698	E117100	12	0	0	0	"Unspecified bipolar affective disorder, mild"	Feb-09
68647	E117200	14	0	0	0	"Unspecified bipolar affective disorder, moderate"	Feb-09
27986	E117z00	310	0	5	0	"Unspecified bipolar affective disorder, NOS"	Feb-09
73423	E117300	3	0	0	0	"Unspecified bipolar affective disorder, severe, no psychosis"	Feb-09
49763	E117000	89	0	0	0	"Unspecified bipolar affective disorder, unspecified"	Feb-09
68326	E117400	14	0	0	0	"Unspecified bipolar affective disorder,severe with psychosis"	Feb-09
71029	PJ5z.00	4	0	0	0	Unspecified conditions due to autosomal anomalies	Feb-09
64449	C108z00	3	0	0	0	Unspecified diabetes mellitus with multiple complications	Feb-09
62920	J34y.00	101	0	11	11	Unspecified diaphragmatic hernia	Feb-09

For peer review only - http://bmjopen.bmj.com/site/about/guidelines.xhtml

5182	K03z.00	3309	0	206	3	Unspecified glomerulonephritis NOS	Feb-09
66506	E101000	1	0	0	0	Unspecified hebephrenic schizophrenia	Feb-09
99620	F435000	1	0	0	0	Unspecified hereditary choroid dystrophy	Jun-10
42511	F427000	14	0	0	0	Unspecified hereditary retinal dystrophies	Feb-09
94560	F4J3000	13	0	0	0	Unspecified heterotropia	Feb-09
73638	AB4zz00	2	0	0	0	Unspecified histoplasmosis NOS	Feb-09
96000	A907.00	2	0	0	0	Unspecified late congenital syphilis	Feb-09
69370	P118000	2	0	0	0	Unspecified spina bifida without hydrocephalus - closed	Feb-09
95018	P110z00	2	0	0	0	Unspecified spina bifida without hydrocephalus NOS	Feb-09
66679	PG51.15	12	0	0	0	Van der Hoeve's syndrome	Feb-09
2155	G341000	990	0	27	0	Ventricular cardiac aneurysm	Feb-09
246	P54..00	27151	0	753	1	Ventricular septal defect	Feb-09
23692	P520.11	250	0	14	0	Ventricular septal defect in Fallot's tetralogy	Feb-09
34067	P54z.00	488	0	3	0	Ventricular septal defect NOS	Feb-09
42132	P540.00	220	0	0	0	"Ventricular septal defect, unspecified"	Feb-09
7794	G571.11	3193	0	84	0	Ventricular tachycardia	Feb-09
35762	7010000	119	0	10	0	Ventriculocisternostomy	Feb-09
26280	F28y300	450	0	0	0	Ventriculomegaly	Feb-09
2482	D011100	69	0	5	0	Vit B12 defic anaemia due to malabsorption with proteinuria	Feb-09
5007	C24..00	218	0	2	2	Vitamin A deficiency	Feb-09
31479	C241.00	24	0	1	0	Vitamin A deficiency + Bitot's spot and conjunctival xerosis	Feb-09
50073	C240.00	12	0	0	0	Vitamin A deficiency with conjunctival xerosis	Feb-09

For peer review only - http://bmjopen.bmj.com/site/about/guidelines.xhtml

61766	C242.00	2	0	0	0	Vitamin A deficiency with corneal xerosis	Feb-09
102237	C244.00	1	0	0	0	Vitamin A deficiency with keratomalacia	May-11
66943	C245.00	8	0	3	0	Vitamin A deficiency with night blindness	Feb-09
45889	C24y.00	1	0	0	0	Vitamin A deficiency with other manifestation	Feb-09
49270	C24y100	10	0	0	0	Vitamin A deficiency with xeroderma	Feb-09
49266	C247000	6	0	0	0	Vitamin A deficiency with xerophthalmia	Feb-09
60199	C246.00	18	0	2	0	Vitamin A deficiency with xerophthalmic corneal scars	Feb-09
42792	C26z.00	228	0	0	0	Vitamin B deficiency NOS	Feb-09
94706	42d6.00	69	0	2	906	Vitamin B profile	Feb-09
4667	C25..11	272	0	91	83	Vitamin B1 deficiency	Feb-09
5271	D011.11	4712	0	201	41	Vitamin B12 deficiency anaemia	Feb-09
32953	D011000	222	0	0	0	Vitamin B12 deficiency anaemia due to dietary causes	Feb-09
24059	C260.12	175	0	4	2	Vitamin B2 deficiency	Feb-09
43354	C261.11	48	0	1	1	Vitamin B6 deficiency	Feb-09
48023	C261.00	39	0	0	0	Vitamin B6 deficiency syndrome	Feb-09
45946	C26..00	343	0	4	2	Vitamin B-complex deficiency	Feb-09
37282	C27..12	134	0	2	2	Vitamin C deficiency	Feb-09
104812	D01y000	2	0	0	0	Vitamin C deficiency anaemia	Aug-12
106409	C2B..00	3201	0	7	2	Vitamin D insufficiency	Jun-13
53111	8B73.00	3033	0	14	2	Vitamin D supplements	Feb-09
105896	C28A.00	9	0	0	0	Vitamin D-dependent rickets	Mar-13
51679	C291000	32	0	0	0	Vitamin E deficiency	Feb-09
62257	D01y100	3	0	0	0	Vitamin E deficiency anaemia	Feb-09
100830	C291100	6	0	0	0	Vitamin P deficiency	Nov-10

For peer review only - http://bmjopen.bmj.com/site/about/guidelines.xhtml

33586	C353200	38	0	3	0	Vitamin-D-resistant rickets	Feb-09
29168	F427C00	384	0	3	0	Vitelliform dystrophy	Feb-09
975	M295100	45691	0	4650	4	Vitiligo	Feb-09
65925	C310211	6	0	0	0	Von Gierke's disease	Feb-09
28443	PK62.00	242	0	10	0	Von Hippel-Lindau syndrome	Feb-09
23137	42Qx.00	0	0	0	507	von Willebrand factor activity	Feb-09
34555	42jf.00	1	0	0	618	von Willebrand factor antigen level	Feb-09
34554	42jg.00	0	0	0	213	von Willebrand factor collagen binding assay	Feb-09
109412	42hB.00	0	0	0	2	von Willebrand Normandy screening test	Mar-15
17520	D304.00	4168	0	166	7	Von Willebrand's disease	Feb-09
108235	C333011	1	0	0	0	Waldenstrom macroglobulinaemia	Jun-14
57509	C330000	10	0	1	0	Waldenstrom's hypergammaglobulinaemic purpura	Feb-09
10411	C333000	887	0	29	1	Waldenstrom's macroglobulinaemia	Feb-09
4810	G754.00	3741	0	128	3	Wegener's granulomatosis	Feb-09
66490	C180.11	4	0	0	0	Wermer's syndrome	Feb-09
57803	C1zy400	2	0	1	0	Werner's syndrome	Feb-09
18636	E011200	415	0	18	0	Wernicke-Korsakov syndrome	Feb-09
51158	ZS78D13	7	0	0	0	Wernicke's aphasia	Feb-09
65074	ZS78D00	6	0	0	0	Wernicke's dysphasia	Feb-09
68256	PJ36.00	1	0	0	0	"Whole chromosome monosomy, meiotic nondisjunction"	Feb-09
95598	PJ37.00	2	0	0	0	"Whole chromosome monosomy, mosaicism"	Feb-09
67815	PJ37z00	1	0	0	0	"Whole chromosome monosomy, mosaicism NOS"	Feb-09
101982	PJ50z00	1	0	0	0	Whole chromosome trisomy syndrome NOS	Apr-11
37702	PJ50.00	21	0	2	0	Whole chromosome trisomy syndromes	Feb-09

For peer review only - http://bmjopen.bmj.com/site/about/guidelines.xhtml

107670	PJ50w00	1	0	0	0	"Whole chromosome trisomy, meitotic nondisjunction"	Feb-14
45512	PJ50x00	8	0	0	0	"Whole chromosome trisomy, mosaicism"	Feb-09
32054	PKy9111	72	0	1	0	Wiedemann - Beckwith syndrome	Feb-09
48476	Q317200	26	0	0	0	Wilson-Mikity syndrome	Feb-09
3870	C351011	404	0	28	0	Wilson's disease	Feb-09
31322	C391200	49	0	5	0	Wiskott - Aldrich syndrome	Feb-09
8230	G567400	7350	0	488	1	Wolff-Parkinson-White syndrome	Feb-09
109868	C327300	2	0	0	0	Wolman disease	Jul-15
70109	F151300	7	0	1	0	X-linked bulbo-spinal atrophy	Feb-09
107207	P235.00	1	0	0	0	X-linked hydrocephalus	Nov-13
107983	C353700	7	0	0	0	X-linked hypophosphataemic rickets	May-14
35335	PH15.00	48	0	3	0	X-linked ichthyosis	Feb-09
32782	PJy2.00	67	0	2	0	XXX syndrome	Feb-09
11948	PJy3.00	39	0	0	0	XXY syndrome	Feb-09
41152	PJ64000	21	0	1	0	"XY, female phenotype"	Feb-09
23355	C115.11	239	0	10	0	Zollinger - Ellison syndrome	Feb-09

For peer review only - http://bmjopen.bmj.com/site/about/guidelines.xhtml

Appendix 4 ICD10 codes to identify a children with a chronic, not life-limiting condition

ICD 10 code

A15.0

A15.1

A15.2

A15.3

A15.4

A15.5

A15.6

A15.7

A15.8

A15.9

A16.0

A16.1

A16.2

A16.3

A16.4

A16.5

A16.7

A16.8

For peer review only - http://bmjopen.bmj.com/site/about/guidelines.xhtml

A16.9

A18.0

A18.1

A18.2

A18.3

A18.4

A18.5

A18.6

A18.7

A18.8

A19.0

A19.1

A19.2

A19.8

A19.9

A50.0

A50.1

A50.2

A50.3

A50.4

For peer review only

A50.5

A50.6

A50.7

A50.9

A81.2

A81.8

A81.9

B18.0

B18.1

B18.2

B18.8

B18.9

B37.7

B37.1

B37.5

B37.6

B38.1

B39.1

B40.1

B44.0

For peer review only - http://bmjopen.bmj.com/site/about/guidelines.xhtml

B44.7

B45.0

B45.1

B45.2

B45.3

B45.7

B45.8

B45.9

B46.0

B46.1

B46.2

B46.3

B46.4

B46.5

B46.8

B46.9

B48.7

B50.0

B51.0

B52.0

For peer review only

B55.0

B55.1

B55.2

B55.9

B57.2

B57.3

B57.4

B57.5

B58.0

B59

B67.0

B67.1

B67.2

B67.3

B67.4

B67.5

B67.6

B67.7

B67.8

B67.9

For peer review only

For peer review only - http://bmjopen.bmj.com/site/about/guidelines.xhtml

B69.0

B69.1

B69.8

B69.9

B73

B74.0

B74.1

B74.2

B74.3

B74.4

B74.8

B74.9

B78.7

B90.0

B90.1

B90.2

B90.8

B90.9

B91

B92

For peer review only - http://bmjopen.bmj.com/site/about/guidelines.xhtml

B94.0

B94.1

B94.2

B94.8

B94.9

D00.0

D00.1

D00.2

D01.0

D01.1

D01.2

D01.3

D01.4

D01.5

D01.7

D01.9

D02.0

D02.1

D02.2

D02.3

For peer review only - http://bmjopen.bmj.com/site/about/guidelines.xhtml

D02.4

D05.0

D05.1

D05.7

D05.9

D06.0

D06.1

D06.7

D06.9

D07.0

D07.1

D07.2

D07.3

D07.4

D07.5

D07.6

D09.0

D09.1

D09.2

D09.3

For peer review only - http://bmjopen.bmj.com/site/about/guidelines.xhtml

D09.7

D09.9

D12.0

D12.1

D12.2

D12.3

D12.4

D12.5

D12.6

D12.7

D12.8

D12.9

D13.0

D13.1

D13.2

D13.3

D13.4

D13.5

D13.6

D13.7

For peer review only

D13.9

D14.1

D14.2

D14.3

D14.4

D15.0

D151

D15.2

D15.7

D15.9

D20.0

D20.1

D32.0

D32.1

D34

D35.0

D35.1

D35.2

D35.3

D35.4

For peer review only - http://bmjopen.bmj.com/site/about/guidelines.xhtml

D35.5

D35.6

D35.7

D35.8

D35.9

D37.0

D37.1

D37.2

D37.3

D37.4

D37.5

D37.6

D37.7

D37.9

D38.0

D38.1

D38.2

D38.3

D38.4

D38.5

For peer review only - http://bmjopen.bmj.com/site/about/guidelines.xhtml

D38.6

D39.0

D39.1

D39.2

D39.7

D39.9

D40.0

D40.1

D40.7

D40.9

D41.0

D41.1

D41.2

D41.3

D41.4

D41.7

D41.9

D42.0

D42.1

D42.9

For peer review only - http://bmjopen.bmj.com/site/about/guidelines.xhtml

For peer review only

D44.0

D44.1

D44.2

D44.3

D44.5

D44.7

D44.8

D44.9

D45

D46.0

D46.1

D46.2

D46.4

D46.5

D46.6

D46.7

D46.9

D47.0

D47.1

D47.2

For peer review only - http://bmjopen.bmj.com/site/about/guidelines.xhtml

D47.3

D47.4

D47.5

D47.7

D47.9

D55.0

D55.1

D55.2

D55.3

D55.8

D55.9

D56.0

D56.2

D56.4

D56.8

D56.9

D57.0

D57.1

D57.2

D57.8

For peer review only - http://bmjopen.bmj.com/site/about/guidelines.xhtml

D58.0

D58.1

D58.2

D58.8

D58.9

D63.0

D63.8

D66

D68.0

D68.1

D68.2

D68.4

D68.5

D68.6

D68.8

D68.9

D69.0

D69.1

D69.2

D69.3

For peer review only - http://bmjopen.bmj.com/site/about/guidelines.xhtml

D69.4

D69.5

D69.6

D69.8

D69.9

D71

D72.0

D72.1

D72.8

D72.9

D73.0

D73.1

D73.2

D73.3

D73.4

D73.5

D73.8

D73.9

D74.0

D74.8

For peer review only - http://bmjopen.bmj.com/site/about/guidelines.xhtml

D74.9

D75.0

D75.1

D75.8

D75.9

D76.1

D76.2

D76.3

D80.0

D80.1

D80.2

D80.3

D80.4

D80.8

D80.9

D82.0

D82.2

D82.3

D82.4

D82.5

For peer review only - http://bmjopen.bmj.com/site/about/guidelines.xhtml

D82.6

D82.7

D82.8

D82.9

D84.0

D84.1

D84.8

D84.9

E00.0

E00.1

E00.2

E00.9

E03.0

E03.1

E07.1

E10

E11

E12

E13

E14

For peer review only - http://bmjopen.bmj.com/site/about/guidelines.xhtml

E22.0

E23.0

E24.4

E25.0

E25.8

E25.9

E26.8

E29.1

E31.1

E31.8

E31.9

E34.0

E34.1

E34.2

E34.5

E35.0

E66.0

E66.1

E66.2

E66.8

For peer review only - http://bmjopen.bmj.com/site/about/guidelines.xhtml

E66.9

E70.0

E70.3

E70.8

E70.9

E78.0

E78.1

E78.2

E78.3

E78.4

E78.5

E78.6

E78.8

E78.9

E79.8

E79.9

E80.0

E80.1

E80.2

E80.3

For peer review only - http://bmjopen.bmj.com/site/about/guidelines.xhtml

E80.5

E80.7

E83.1

E83.2

E83.3

E83.4

E83.5

E83.8

E83.9

E85.0

E85.1

E85.2

E85.3

E85.4

E85.8

E85.9

E88.2

E88.3

E88.8

E88.9

For peer review only - http://bmjopen.bmj.com/site/about/guidelines.xhtml

F00.0

F00.1

F00.2

F00.9

F01.0

F01.1

F01.2

F01.3

F01.8

F01.9

F02.1

F02.2

F02.3

F02.4

F02.8

F03

F04

F05.0

F05.1

F05.8

For peer review only

F05.9

F06.0

F06.1

F06.2

F06.3

F06.4

F06.5

F06.6

F06.7

F06.8

F07.0

F07.1

F07.2

F07.8

F07.9

F09

F10

F11

F12

F13

For peer review only

F14

F15

F16

F17

F18

F19

F20.0

F20.1

F20.2

F20.3

F20.4

F20.5

F20.6

F20.8

F20.9

F21

F22.0

F22.8

F22.9

F23.0

For peer review only

For peer review only - http://bmjopen.bmj.com/site/about/guidelines.xhtml

F23.1

F23.2

F23.3

F23.8

F23.9

F24

F25.0

F25.1

F25.2

F25.8

F25.9

F28

F29

F30.0

F30.1

F30.2

F30.8

F30.9

F31.0

F31.1

For peer review only

For peer review only - http://bmjopen.bmj.com/site/about/guidelines.xhtml

F31.2

F31.3

F31.4

F31.5

F31.6

F31.7

F31.8

F31.9

F32.0

F32.1

F32.2

F32.3

F32.8

F32.9

F33.0

F33.1

F33.2

F33.3

F33.4

F33.8

For peer review only

F33.9

F34.0

F34.1

F34.8

F34.9

F38.0

F38.1

F38.8

F39

F40.0

F40.1

F40.2

F40.8

F40.9

F41.0

F41.1

F41.2

F41.3

F41.8

F41.9

For peer review only - http://bmjopen.bmj.com/site/about/guidelines.xhtml

F42.0

F42.1

F42.2

F42.8

F42.9

F43.0

F43.1

F43.2

F43.8

F43.9

F44.0

F44.1

F44.2

F44.3

F44.4

F44.5

F44.6

F44.7

F44.8

F44.9

For peer review only

For peer review only - http://bmjopen.bmj.com/site/about/guidelines.xhtml

F45.0

F45.1

F45.2

F45.3

F45.4

F45.8

F45.9

F48.1

F48.8

F48.9

F50.0

F50.1

F50.2

F50.3

F50.4

F50.5

F50.8

F50.9

F53.0

F53.1

For peer review only - http://bmjopen.bmj.com/site/about/guidelines.xhtml

F53.8

F53.9

F54

F70

F71

F72

F73

F78

F79

F80.0

F80.1

F80.2

F80.8

F80.9

F82

F83

F84.1

F84.3

F84.4

F84.5

For peer review only

For peer review only - http://bmjopen.bmj.com/site/about/guidelines.xhtml

F84.6

F84.8

F84.9

F88

F89

F90.0

F90.1

F90.8

F90.9

F91.0

F91.1

F91.2

F91.3

F91.8

F91.9

F92.0

F92.8

F92.9

F93.0

F93.1

For peer review only

F93.2

F93.3

F93.8

F93.9

F94.0

F94.1

F94.2

F94.8

F94.9

F95.0

F95.1

F95.2

F95.8

F95.9

F98.0

F98.1

F98.2

F98.3

F98.4

F98.5

For peer review only - http://bmjopen.bmj.com/site/about/guidelines.xhtml

F98.6

F98.8

F98.9

G00.0

G00.1

G00.2

G00.3

G00.8

G00.9

G01

G02.0

G02.1

G02.8

G03.0

G03.1

G03.2

G03.8

G03.9

G04.0

G04.1

For peer review only

For peer review only - http://bmjopen.bmj.com/site/about/guidelines.xhtml

G04.2

G04.8

G04.9

G05.0

G05.1

G05.2

G05.8

G06.0

G06.1

G06.2

G07

G08

G09

G11.0

G11.4

G11.8

G11.9

G13.0

G13.1

G13.2

For peer review only

G13.8

G14

G21.0

G21.1

G21.2

G21.3

G21.4

G21.8

G21.9

G22

G23.1

G23.2

G23.3

G23.9

G24.1

G24.2

G24.3

G24.4

G24.5

G24.8

For peer review only - http://bmjopen.bmj.com/site/about/guidelines.xhtml

G24.9

G25.0

G25.1

G25.2

G25.3

G25.4

G25.5

G25.6

G25.8

G25.9

G26

G30.0

G30.1

G30.8

G30.9

G31.0

G31.1

G31.2

G32.0

G32.8

For peer review only - http://bmjopen.bmj.com/site/about/guidelines.xhtml

G36.0

G36.1

G36.8

G36.9

G37.0

G37.1

G37.2

G37.3

G37.4

G37.5

G37.8

G37.9

G40.0

G40.1

G40.2

G40.3

G40.6

G40.7

G40.8

G40.9

For peer review only

For peer review only - http://bmjopen.bmj.com/site/about/guidelines.xhtml

G41.0

G41.1

G41.2

G41.8

G41.9

G43.0

G43.1

G43.2

G43.3

G43.8

G43.9

G44.0

G44.1

G44.2

G44.3

G44.4

G44.8

G45.0

G45.1

G45.2

For peer review only - http://bmjopen.bmj.com/site/about/guidelines.xhtml

G45.3

G45.4

G45.8

G45.9

G46.0

G46.1

G46.2

G46.3

G46.4

G46.5

G46.6

G46.7

G46.8

G47.0

G47.1

G47.2

G47.3

G47.4

G47.8

G47.9

For peer review only - http://bmjopen.bmj.com/site/about/guidelines.xhtml

G50.0
G50.1
G50.8
G50.9
G51.0
G51.1
G51.2
G51.3
G51.4
G51.8
G51.9
G52.0
G52.1
G52.2
G52.3
G52.7
G52.8
G52.9
G53.0
G53.1

For peer review only - http://bmjopen.bmj.com/site/about/guidelines.xhtml

G53.2

G53.3

G53.8

G54.0

G54.1

G54.2

G54.3

G54.4

G54.5

G54.6

G54.7

G54.8

G54.9

G55.0

G55.1

G55.2

G55.3

G55.8

G56.0

G56.1

For peer review only - http://bmjopen.bmj.com/site/about/guidelines.xhtml

G56.2

G56.3

G56.4

G56.8

G56.9

G57.0

G57.1

G57.2

G57.3

G57.4

G57.5

G57.6

G57.8

G57.9

G58.0

G58.7

G58.8

G58.9

G59.0

G59.8

For peer review only - http://bmjopen.bmj.com/site/about/guidelines.xhtml

G60.2

G60.3

G60.8

G60.9

G61.0

G61.1

G61.8

G61.9

G62.0

G62.1

G62.2

G62.8

G62.9

G63.1

G63.2

G63.3

G63.5

G63.6

G63.8

G64

For peer review only - http://bmjopen.bmj.com/site/about/guidelines.xhtml

G70.0

G70.1

G70.8

G71.8

G71.9

G72.0

G72.1

G72.2

G72.3

G72.4

G72.8

G72.9

G73.0

G73.1

G73.2

G73.3

G73.5

G73.6

G73.7

G80.1

For peer review only - http://bmjopen.bmj.com/site/about/guidelines.xhtml

G80.2

G80.3

G80.4

G80.9

G81.0

G81.1

G81.9

G82.0

G82.1

G82.2

G83.0

G83.1

G83.2

G83.3

G83.4

G83.5

G83.8

G83.9

G90.0

G90.1

290

For peer review only

G90.2

G90.4

G90.8

G90.9

G91.0

G91.1

G91.2

G91.3

G91.8

G91.9

G92

G93.0

G93.1

G93.2

G93.3

G93.8

G93.9

G94.1

G94.2

G94.8

For peer review only

G95.0

G95.1

G95.2

G95.8

G95.9

G96.0

G96.1

G96.8

G96.9

G99.0

G99.1

G99.2

G99.8

H05.1

H05.2

H05.3

H05.4

H05.5

H05.8

H05.9

For peer review only - http://bmjopen.bmj.com/site/about/guidelines.xhtml

H13.3

H17.0

H17.1

H17.8

H17.9

H18.0

H18.1

H18.2

H18.3

H18.4

H18.5

H18.6

H18.7

H18.8

H18.9

H19.3

H21.0

H21.2

H21.2

H21.3

For peer review only - http://bmjopen.bmj.com/site/about/guidelines.xhtml

H21.4

H21.5

H21.8

H21.9

H26.0

H26.1

H26.2

H26.3

H26.4

H26.8

H26.9

H27.0

H27.1

H27.8

H27.9

H28.0

H28.1

H28.2

H31.0

H31.1

For peer review only - http://bmjopen.bmj.com/site/about/guidelines.xhtml

H31.2

H31.3

H31.4

H31.8

H31.9

H32.8

H33.0

H33.1

H33.2

H33.3

H33.4

H33.5

H34.0

H34.1

H34.2

H34.8

H34.9

H35.0

H35.1

H35.2

For peer review only - http://bmjopen.bmj.com/site/about/guidelines.xhtml

H35.3

H35.4

H35.6

H35.7

H35.8

H35.9

H40.0

H40.1

H40.2

H40.3

H40.4

H40.5

H40.6

H40.8

H40.9

H42.0

H43.0

H43.1

H43.2

H43.3

For peer review only

For peer review only - http://bmjopen.bmj.com/site/about/guidelines.xhtml

H43.8

H43.9

H44.0

H44.1

H44.2

H44.3

H44.4

H44.5

H44.6

H44.7

H44.8

H44.9

H47.0

H47.1

H47.3

H47.4

H47.5

H47.6

H47.7

H54.0

For peer review only - http://bmjopen.bmj.com/site/about/guidelines.xhtml

H54.1

H54.2

H54.4

H60.2

H65.2

H65.3

H65.4

H66.1

H66.2

H66.3

H69.0

H69.8

H69.9

H70.1

H73.1

H74.0

H74.1

H74.2

H74.3

H75.0

298

For peer review only

H80.0

H80.1

H80.2

H80.8

H80.9

H81.0

H81.4

H83.0

H83.2

H90.0

H90.3

H90.5

H90.6

H91.0

H91.1

H91.2

H91.3

H91.8

H91.9

I43.1

299

For peer review only

I60.0

I60.1

I60.2

I60.3

I60.4

I60.5

I60.6

I60.7

I60.8

I60.9

I61.0

I61.1

I61.2

I61.4

I61.5

I61.6

I61.8

I61.9

I62.0

I62.1

For peer review only - http://bmjopen.bmj.com/site/about/guidelines.xhtml

I62.9

I63.0

I63.1

I63.2

I63.3

I63.4

I63.5

I63.6

I63.8

I63.9

I64

I65.0

I65.1

I65.2

I65.3

I65.8

I65.9

I66.0

I66.1

I66.2

For peer review only - http://bmjopen.bmj.com/site/about/guidelines.xhtml

I66.3

I66.4

I66.8

I66.9

I67.0

I67.1

I67.2

I67.3

I67.4

I67.5

I67.6

I67.7

I67.8

I67.9

I68.0

I68.2

I68.8

I69.0

I69.1

I69.2

For peer review only - http://bmjopen.bmj.com/site/about/guidelines.xhtml

I69.3

I69.4

I69.8

I72.0

I72.5

I72.9

I79.2

J41.0

J41.1

J41.8

J42

J43.0

J43.1

J43.2

J43.8

J43.9

J44.0

J44.1

J44.8

J44.9

For peer review only

J45.0

J45.1

J45.8

J45.9

J46

J47

J60

J61

J62.0

J62.8

J63.0

J63.1

J63.2

J63.3

J63.4

J63.5

J63.8

J64

J65

J66.0

For peer review only - http://bmjopen.bmj.com/site/about/guidelines.xhtml

J66.1

J66.2

J66.8

J67.0

J67.1

J67.2

J67.3

J67.4

J67.5

J67.6

J67.7

J67.8

J67.9

J68.0

J68.1

J68.2

J68.3

J68.4

J68.8

J68.9

For peer review only - http://bmjopen.bmj.com/site/about/guidelines.xhtml

J69.0

J69.1

J69.8

J70.0

J70.1

J70.2

J70.3

J70.4

J70.8

J70.9

J80

J81

J82

J84.0

J84.8

J84.9

J85.0

J85.1

J85.2

J85.3

For peer review only

For peer review only - http://bmjopen.bmj.com/site/about/guidelines.xhtml

J86.0

J86.9

J98.0

J98.1

J98.2

J98.3

J98.5

J98.6

J98.8

J98.9

J99.0

J99.1

K20

K21.0

K22.0

K22.1

K22.2

K22.3

K22.4

K22.5

For peer review only - http://bmjopen.bmj.com/site/about/guidelines.xhtml

K22.6

K22.7

K22.8

K22.9

K23.0

K23.1

K23.8

K25

K26

K27

K28

K29.0

K29.1

K29.2

K29.3

K29.4

K29.5

K29.6

K29.7

K29.8

For peer review only - http://bmjopen.bmj.com/site/about/guidelines.xhtml

K29.9

K31.0

K31.1

K31.2

K31.3

K31.4

K31.5

K31.6

K31.7

K31.8

K31.9

K50.0

K50.1

K50.8

K50.9

K51.0

K51.2

K51.3

K51.4

K51.5

For peer review only - http://bmjopen.bmj.com/site/about/guidelines.xhtml

K51.8

K51.9

K52.0

K52.1

K52.2

K52.3

K52.8

K52.9

K55.1

K55.2

K55.8

K57.0

K57.1

K57.2

K57.3

K57.4

K57.5

K57.8

K57.9

K59.2

310

For peer review only

K63.0

K63.1

K63.2

K63.3

K66.0

K66.1

K66.8

K66.9

K67.3

K70.0

K70.1

K70.2

K70.3

K70.4

K70.9

K73.0

K73.1

K73.2

K73.8

K73.9

For peer review only - http://bmjopen.bmj.com/site/about/guidelines.xhtml

K75.0

K75.1

K75.2

K75.3

K75.4

K75.8

K75.9

K76.0

K76.1

K76.2

K76.3

K76.4

K76.6

K76.7

K76.9

K80.0

K80.1

K80.2

K80.3

K80.4

For peer review only - http://bmjopen.bmj.com/site/about/guidelines.xhtml

K80.5
K80.8
K81.0
K81.1
K81.8
K81.9
K82.0
K82.1
K82.2
K82.3
K82.4
K82.8
K82.9
K83.0
K83.1
K83.2
K83.3
K83.4
K83.5
K83.8

For peer review only - http://bmjopen.bmj.com/site/about/guidelines.xhtml

K83.9

K85.0

K85.1

K85.2

K85.3

K85.8

K85.9

K86.0

K86.1

K86.2

K86.3

K86.9

K87.0

K90.0

K90.1

K90.2

K90.3

K90.4

K90.8

K90.0

314

For peer review only

K93.0

K93.1

L10.0

L10.1

L10.2

L10.3

L10.4

L10.5

L10.8

L10.9

L11.0

L11.8

L11.9

L12.0

L12.1

L12.2

L12.3

L12.8

L12.9

L13.0

For peer review only

For peer review only - http://bmjopen.bmj.com/site/about/guidelines.xhtml

L13.1

L13.8

L13.9

L14

L28.0

L28.1

L28.2

L40.0

L40.1

L40.2

L40.3

L40.4

L40.5

L40.8

L40.9

L41.0

L41.1

L41.3

L41.4

L41.5

For peer review only - http://bmjopen.bmj.com/site/about/guidelines.xhtml

L41.8

L41.9

L42

L43.0

L43.1

L43.2

L43.3

L43.8

L43.9

L44.0

L44.1

L44.2

L44.3

L44.4

L44.8

L44.9

L45

L57.0

L57.1

L57.2

For peer review only - http://bmjopen.bmj.com/site/about/guidelines.xhtml

L57.3

L57.4

L57.5

L57.8

L57.9

L58.1

L59.0

L59.8

L59.9

L62.0

L87.0

L87.1

L87.2

L87.8

L87.9

L88

L90.0

L90.1

L90.2

L90.3

For peer review only - http://bmjopen.bmj.com/site/about/guidelines.xhtml

L90.4

L90.5

L90.6

L90.8

L90.9

L92.0

L92.1

L92.2

L92.3

L92.8

L92.9

L93.0

L93.1

L93.2

L95.0

L95.1

L95.8

L95.9

L98.5

L99.0

For peer review only

For peer review only - http://bmjopen.bmj.com/site/about/guidelines.xhtml

M01.1

M03.6

M05.0

M05.1

M05.2

M05.3

M05.8

M05.9

M06.0

M06.1

M06.2

M06.3

M06.4

M06.8

M06.9

M07.0

M07.1

M07.2

M07.3

M07.4

For peer review only - http://bmjopen.bmj.com/site/about/guidelines.xhtml

M07.6

M08.0

M08.1

M08.2

M08.3

M08.4

M08.8

M08.9

M09.0

M09.1

M09.2

M09.8

M10.0

M10.1

M10.2

M10.3

M10.4

M10.9

M11.0

M11.1

For peer review only

For peer review only - http://bmjopen.bmj.com/site/about/guidelines.xhtml

M11.2
M11.8
M11.9
M12.0
M12.1
M12.2
M12.3
M12.4
M12.5
M12.8
M13.0
M13.1
M13.8
M13.9
M14.0
M14.2
M14.3
M14.4
M14.5
M14.6

For peer review only - http://bmjopen.bmj.com/site/about/guidelines.xhtml

M14.8

M30.0

M30.1

M30.2

M30.3

M30.8

M31.0

M31.1

M31.2

M31.4

M31.5

M31.6

M31.7

M31.8

M31.9

M32.0

M32.8

M32.9

M33.0

M33.1

For peer review only - http://bmjopen.bmj.com/site/about/guidelines.xhtml

M33.2

M33.9

M34.0

M34.1

M34.2

M34.8

M34.9

M35.0

M35.1

M35.2

M35.3

M35.4

M35.5

M35.6

M35.7

M35.8

M35.9

M36.0

M36.1

M36.2

For peer review only - http://bmjopen.bmj.com/site/about/guidelines.xhtml

M36.3

M36.4

M40.0

M40.1

M40.2

M40.3

M40.4

M40.5

M41.0

M41.1

M41.2

M41.3

M41.4

M41.5

M41.8

M41.9

M42.0

M42.1

M42.9

M43.0

For peer review only - http://bmjopen.bmj.com/site/about/guidelines.xhtml

M43.1

M43.2

M43.3

M43.4

M43.5

M43.6

M43.8

M43.9

M45

M46.0

M46.1

M46.2

M46.3

M46.4

M46.5

M46.8

M46.9

M47.0

M47.1

M47.2

For peer review only - http://bmjopen.bmj.com/site/about/guidelines.xhtml

M47.8

M47.9

M48.0

M48.1

M48.2

M48.3

M48.4

M48.5

M48.8

M48.9

M49.0

M49.5

M50.0

M50.1

M50.2

M50.3

M50.8

M50.9

M51.0

M51.1

For peer review only

M51.2

M51.3

M51.4

M51.8

M51.9

M53.0

M53.1

M53.2

M53.3

M53.8

M53.9

M54.0

M54.1

M54.2

M54.3

M54.4

M54.5

M54.6

M54.8

M54.9

For peer review only - http://bmjopen.bmj.com/site/about/guidelines.xhtml

M60.0

M60.1

M60.2

M60.8

M60.9

M61.0

M61.1

M61.2

M61.3

M61.4

M61.5

M61.9

M62.0

M62.1

M62.2

M62.3

M62.4

M62.5

M62.6

M62.8

For peer review only

M62.9

M63.8

M80.1

M80.2

M80.3

M80.4

M80.5

M80.8

M80.9

M81.1

M81.2

M81.3

M81.4

M81.5

M81.6

M81.8

M81.9

M82.0

M82.1

M82.8

For peer review only

For peer review only - http://bmjopen.bmj.com/site/about/guidelines.xhtml

M84.0

M84.1

M84.2

M84.8

M84.9

M85.0

M85.1

M85.2

M85.3

M85.4

M85.5

M85.6

M85.8

M85.9

M86.3

M86.4

M86.5

M86.6

M89.0

M89.1

For peer review only - http://bmjopen.bmj.com/site/about/guidelines.xhtml

M89.2

M89.3

M89.4

M89.6

M89.8

M89.9

M90.0

M90.4

M90.6

M90.7

M90.8

M91.0

M91.1

M91.2

M91.3

M91.8

M91.9

M92.0

M92.1

M92.2

For peer review only

For peer review only - http://bmjopen.bmj.com/site/about/guidelines.xhtml

M92.3

M92.4

M92.5

M92.6

M92.7

M92.8

M92.9

M93.0

M93.1

M93.2

M93.8

M93.9

M94.0

M94.1

M94.2

M94.3

M94.8

M94.9

N00

N01

For peer review only - http://bmjopen.bmj.com/site/about/guidelines.xhtml

N02

N03

N04

N05

N07

N08.1

N08.2

N08.3

N08.4

N08.5

N08.8

N11.0

N11.1

N11.8

N11.9

N12

N13.0

N13.1

N13.2

N13.3

For peer review only

For peer review only - http://bmjopen.bmj.com/site/about/guidelines.xhtml

N13.4

N13.5

N13.6

N13.7

N13.8

N13.9

N14.0

N14.1

N14.2

N14.3

N14.4

N15.0

N15.1

N15.8

N15.9

N16.0

N16.1

N16.2

N16.3

N16.4

For peer review only - http://bmjopen.bmj.com/site/about/guidelines.xhtml

N16.5

N16.8

N20.0

N20.1

N20.2

N20.9

N21.0

N21.1

N21.8

N21.9

N22.0

N22.8

N23

N25.0

N25.9

N26

N28.0

N28.1

N28.8

N28.9

For peer review only

For peer review only - http://bmjopen.bmj.com/site/about/guidelines.xhtml

N29.0

N29.8

N31.0

N31.1

N31.2

N31.8

N31.9

N32.0

N32.1

N32.2

N32.3

N32.4

N32.8

N32.9

N33.0

N33.8

N35.0

N35.1

N35.8

N35.9

For peer review only

For peer review only - http://bmjopen.bmj.com/site/about/guidelines.xhtml

N36.0

N36.1

N36.2

N36.3

N36.8

N36.9

N39.1

N39.3

N39.4

N40

N42.0

N42.1

N42.2

N42.3

N42.8

N42.9

N70.1

N70.9

N71.1

N71.9

For peer review only - http://bmjopen.bmj.com/site/about/guidelines.xhtml

N72

N73.0

N73.1

N73.2

N73.3

N73.4

N73.5

N73.6

N73.8

N73.9

N74.0

N74.1

N74.2

N74.3

N74.4

N74.8

N80.0

N80.1

N80.2

N80.3

For peer review only

For peer review only - http://bmjopen.bmj.com/site/about/guidelines.xhtml

N80.4

N80.5

N80.6

N80.8

N80.9

N81.0

N81.1

N81.2

N81.3

N81.4

N81.5

N81.6

N81.8

N81.9

N82.0

N82.1

N82.2

N82.3

N82.4

N82.5

For peer review only - http://bmjopen.bmj.com/site/about/guidelines.xhtml

N82.8

N82.9

N85.0

N85.1

N85.2

N85.3

N85.4

N85.5

N85.6

N85.7

N85.8

N85.9

N87.0

N87.1

N87.2

N87.9

N88.0

N88.1

N88.2

N88.3

For peer review only
For peer review only - http://bmjopen.bmj.com/site/about/guidelines.xhtml

N88.4

N88.8

N88.9

O35.4

P10.0

P10.2

P10.3

P10.4

P10.8

P10.9

P27.0

P27.1

P27.8

P27.9

P35.2

P35.9

P37.0

P37.1

P52.0

P52.1

For peer review only - http://bmjopen.bmj.com/site/about/guidelines.xhtml

P52.2

P52.3

P52.5

P52.6

P52.8

P52.9

P57.0

P57.8

P57.9

P75

P90

P91.1

P96.0

Q00.1

Q00.1

Q02

Q03.0

Q03.8

Q04.1

Q04.5

For peer review only - http://bmjopen.bmj.com/site/about/guidelines.xhtml

Q04.8

Q06.0

Q06.1

Q06.2

Q06.3

Q06.4

Q06.8

Q06.9

Q07.8

Q07.9

Q10.4

Q10.7

Q11.0

Q11.1

Q11.2

Q11.3

Q12.0

Q12.1

Q12.2

Q12.3

For peer review only

For peer review only - http://bmjopen.bmj.com/site/about/guidelines.xhtml

Q12.4

Q12.8

Q12.9

Q13.0

Q13.1

Q13.2

Q13.3

Q13.4

Q13.8

Q13.9

Q14.0

Q14.1

Q14.2

Q14.3

Q14.8

Q14.9

Q15.0

Q15.8

Q15.9

Q16.0

For peer review only - http://bmjopen.bmj.com/site/about/guidelines.xhtml

Q16.1

Q16.2

Q16.3

Q16.4

Q16.5

Q16.9

Q18.8

Q20.1

Q20.2

Q20.5

Q21.0

Q21.1

Q21.2

Q21.4

Q21.9

Q22.2

Q22.3

Q22.8

Q22.9

Q23.1

For peer review only

Q23.3

Q23.8

Q24.0

Q24.1

Q24.2

Q24.3

Q24.4

Q24.5

Q24.6

Q24.8

Q24.9

Q25.0

Q25.1

Q25.2

Q25.3

Q25.7

Q25.8

Q25.9

Q26.0

Q26.1

For peer review only - http://bmjopen.bmj.com/site/about/guidelines.xhtml

Q26.3

Q26.5

Q26.9

Q27.0

Q27.1

Q27.2

Q27.3

Q27.4

Q27.8

Q27.9

Q28.0

Q28.1

Q28.3

Q28.9

Q30.0

Q30.1

Q30.2

Q30.3

Q30.8

Q30.9

For peer review only - http://bmjopen.bmj.com/site/about/guidelines.xhtml

Q31.0

Q31.1

Q31.2

Q31.3

Q31.5

Q31.8

Q31.9

Q32.0

Q32.2

Q32.3

Q32.4

Q33.0

Q33.1

Q33.2

Q33.3

Q33.4

Q33.5

Q33.8

Q33.9

Q34.0

For peer review only - http://bmjopen.bmj.com/site/about/guidelines.xhtml

Q34.1

Q34.8

Q34.9

Q35.1

Q35.3

Q35.5

Q35.7

Q35.9

Q36.0

Q36.1

Q36.9

Q37.0

Q37.1

Q37.2

Q37.3

Q37.4

Q37.5

Q37.8

Q37.9

Q38.0

For peer review only

For peer review only - http://bmjopen.bmj.com/site/about/guidelines.xhtml

Q38.3

Q38.4

Q38.6

Q38.7

Q38.8

Q39.0

Q39.1

Q39.2

Q39.3

Q39.4

Q39.5

Q39.8

Q39.9

Q40.2

Q40.3

Q40.8

Q41.1

Q41.2

Q41.8

Q42.0

For peer review only

Q42.1

Q42.2

Q42.3

Q42.8

Q42.9

Q43.1

Q43.3

Q43.4

Q43.5

Q43.6

Q43.9

Q44.0

Q44.1

Q44.3

Q44.4

Q44.5

Q44.7

Q45.0

Q45.1

Q45.2

For peer review only

For peer review only - http://bmjopen.bmj.com/site/about/guidelines.xhtml

Q45.3

Q45.8

Q45.9

Q50.0

Q51.0

Q51.1

Q51.2

Q51.3

Q51.4

Q51.5

Q51.6

Q51.7

Q51.8

Q51.9

Q52.1

Q52.2

Q52.4

Q54.0

Q54.1

Q54.2

For peer review only - http://bmjopen.bmj.com/site/about/guidelines.xhtml

Q54.3

Q54.8

Q54.9

Q55.0

Q55.5

Q56.0

Q56.1

Q56.2

Q56.3

Q56.4

Q60.2

Q60.4

Q60.5

Q61.0

Q61.1

Q61.2

Q61.3

Q61.5

Q61.8

Q62.0

For peer review only

Q62.1

Q62.3

Q62.4

Q62.5

Q62.6

Q62.8

Q63.0

Q63.1

Q63.2

Q63.8

Q63.9

Q64.0

Q64.1

Q64.3

Q64.4

Q64.5

Q64.6

Q64.7

Q64.8

Q64.9

For peer review only

For peer review only - http://bmjopen.bmj.com/site/about/guidelines.xhtml

Q65.0

Q65.1

Q65.2

Q65.8

Q65.9

Q67.5

Q68.2

Q71.0

Q71.1

Q71.2

Q71.3

Q71.4

Q71.5

Q71.6

Q71.8

Q71.9

Q72.0

Q72.1

Q72.2

Q72.3

For peer review only

For peer review only - http://bmjopen.bmj.com/site/about/guidelines.xhtml

Q72.4

Q72.5

Q72.6

Q72.7

Q72.8

Q72.9

Q73.0

Q73.1

Q73.8

Q74.0

Q74.1

Q74.2

Q74.8

Q74.9

Q75.1

Q75.3

Q75.4

Q75.5

Q75.8

Q75.9

For peer review only - http://bmjopen.bmj.com/site/about/guidelines.xhtml

Q76.1

Q76.2

Q76.3

Q76.4

Q77.0

Q77.1

Q77.5

Q77.6

Q77.7

Q77.8

Q77.9

Q78.0

Q78.1

Q78.2

Q78.3

Q78.4

Q78.6

Q78.8

Q79.0

Q79.4

For peer review only

For peer review only - http://bmjopen.bmj.com/site/about/guidelines.xhtml

Q79.5

Q79.6

Q79.8

Q80.0

Q80.1

Q80.2

Q80.3

Q80.8

Q80.9

Q82.0

Q82.2

Q82.3

Q82.9

Q85.0

Q85.9

Q85.0

Q86.1

Q86.2

Q86.8

Q87.3

For peer review only - http://bmjopen.bmj.com/site/about/guidelines.xhtml

Q87.4

Q87.5

Q89.1

Q89.2

Q89.3

Q89.4

Q89.7

Q89.8

Q89.9

Q90.0

Q90.1

Q90.2

Q90.9

Q92.2

Q92.3

Q92.5

Q92.6

Q92.9

Q93.0

Q93.1

For peer review only

Q93.6

Q93.7

Q93.9

Q97.0

Q97.1

Q97.2

Q97.3

Q97.8

Q97.9

Q98.0

Q99.0

Q99.1

Q99.2

Q99.8

Q99.9

For peer review only

STROBE Statement—Checklist of items that should be included in reports of ***cohort studies***

	Item No	Recommendation	Page No
**Title and abstract**	1	(*a*) Indicate the study's design with a commonly used term in the title or the abstract<br>(*b*) Provide in the abstract an informative and balanced summary of what was done and what was found	1
**Introduction**			
Background/rationale	2	Explain the scientific background and rationale for the investigation being reported	3-4
Objectives	3	State specific objectives, including any prespecified hypotheses	4
**Methods**			
Study design	4	Present key elements of study design early in the paper	5 + abstract + Title
Setting	5	Describe the setting, locations, and relevant dates, including periods of recruitment, exposure, follow-up, and data collection	5
Participants	6	(*a*) Give the eligibility criteria, and the sources and methods of selection of participants. Describe methods of follow-up<br>(*b*) For matched studies, give matching criteria and number of exposed and unexposed	5-7 + Table 1
Variables	7	Clearly define all outcomes, exposures, predictors, potential confounders, and effect modifiers. Give diagnostic criteria, if applicable	8 -9
Data sources/ measurement	8*	For each variable of interest, give sources of data and details of methods of assessment (measurement). Describe comparability of assessment methods if there is more than one group	8 -9
Bias	9	Describe any efforts to address potential sources of bias	9-10
Study size	10	Explain how the study size was arrived at	5,10
Quantitative variables	11	Explain how quantitative variables were handled in the analyses. If applicable, describe which groupings were chosen and why	9-10
Statistical methods	12	(*a*) Describe all statistical methods, including those used to control for confounding<br>(*b*) Describe any methods used to examine subgroups and interactions<br>(*c*) Explain how missing data were addressed<br>(*d*) If applicable, explain how loss to follow-up was addressed<br>(*e*) Describe any sensitivity analyses	9-10
**Results**			
Participants	13*	(a) Report numbers of individuals at each stage of study—eg numbers potentially eligible, examined for eligibility, confirmed eligible, included in the study, completing follow-up, and analysed<br>(b) Give reasons for non-participation at each stage<br>(c) Consider use of a flow diagram	
Descriptive data	14*	(a) Give characteristics of study participants (eg demographic, clinical, social) and information on exposures and potential confounders<br>(b) Indicate number of participants with missing data for each variable of interest<br>(c) Summarise follow-up time (eg, average and total amount)	
Outcome data	15*	Report numbers of outcome events or summary measures over time	

For peer review only

For peer review only - http://bmjopen.bmj.com/site/about/guidelines.xhtml

For peer review only

For peer review only - http://bmjopen.bmj.com/site/about/guidelines.xhtml

Main results	16	(*a*) Give unadjusted estimates and, if applicable, confounder-adjusted estimates and their precision (eg, 95% confidence interval). Make clear which confounders were adjusted for and why they were included<br>(*b*) Report category boundaries when continuous variables were categorized<br>(*c*) If relevant, consider translating estimates of relative risk into absolute risk for a meaningful time period	
Other analyses	17	Report other analyses done—eg analyses of subgroups and interactions, and sensitivity analyses	
**Discussion**			
Key results	18	Summarise key results with reference to study objectives	
Limitations	19	Discuss limitations of the study, taking into account sources of potential bias or imprecision. Discuss both direction and magnitude of any potential bias	
Interpretation	20	Give a cautious overall interpretation of results considering objectives, limitations, multiplicity of analyses, results from similar studies, and other relevant evidence	
Generalisability	21	Discuss the generalisability (external validity) of the study results	
**Other information**			
Funding	22	Give the source of funding and the role of the funders for the present study and, if applicable, for the original study on which the present article is based	

*Give information separately for exposed and unexposed groups.

Note: An Explanation and Elaboration article discusses each checklist item and gives methodological background and published examples of transparent reporting. The STROBE checklist is best used in conjunction with this article (freely available on the Web sites of PLoS Medicine at http://www.plosmedicine.org/, Annals of Internal Medicine at http://www.annals.org/, and Epidemiology at http://www.epidem.com/). Information on the STROBE Initiative is available at http://www.strobe-statement.org.

For peer review only

For peer review only - http://bmjopen.bmj.com/site/about/guidelines.xhtml
